# Identification of long non-coding RNAs and microRNAs involved in anther development in the tropical *Camellia oleifera*

**DOI:** 10.1186/s12864-022-08836-7

**Published:** 2022-08-16

**Authors:** Lingshan Kong, Yanjing Zhuo, Jieru Xu, Xiangxu Meng, Yue Wang, Wenxiu Zhao, Hanggui Lai, Jinhui Chen, Jian Wang

**Affiliations:** 1 Sanya Nanfan Research Institute of Hainan University, Hainan Yazhou Bay Seed Laboratory, 572025 Sanya, P. R. China; 2 Key Laboratory of Genetics and Germplasm Innovation of Tropical Special Forest Trees and Ornamental Plants, Ministry of Education/Engineering Research Center of Rare and Precious Tree Species in Hainan Province, School of Forestry, Hainan University, 570228 Haikou, P. R. China; 3 School of Horticulture, Hainan University, 570228 Haikou, P. R. China; 4 School of Public Administration, Hainan University, 570228 Haikou, P. R. China; 5 School of Tropical Crops, Hainan University, 570228 Haikou, P. R. China

**Keywords:** Anther development, MicroRNAs, Long non-coding RNAs, Network, *Camellia oleifera*

## Abstract

**Background:**

Explored the molecular science of anther development is important for improving productivity and overall yield of crops. Although the role of regulatory RNAs, including long non-coding RNAs (lncRNAs) and microRNAs (miRNAs), in regulating anther development has been established, their identities and functions in *Camellia oleifera*, an important industrial crop, have yet not been clearly explored. Here, we report the identification and characterization of genes, lncRNAs and miRNAs during three stages of the tropical *C. oleifera* anther development by single-molecule real-time sequencing, RNA sequencing and small RNA sequencing, respectively.

**Results:**

These stages, *viz.* the pollen mother cells stage, tetrad stage and uninucleate pollen stage, were identified by analyzing paraffin sections of floral buds during rapid expansion periods. A total of 18,393 transcripts, 414 putative lncRNAs and 372 miRNAs were identified, of which 5,324 genes, 115 lncRNAs, and 44 miRNAs were differentially accumulated across three developmental stages. Of these, 44 and 92 genes were predicted be regulated by 37 and 30 differentially accumulated lncRNAs and miRNAs, respectively. Additionally, 42 differentially accumulated lncRNAs were predicted as targets of 27 miRNAs. Gene ontology enrichment indicated that potential target genes of lncRNAs were enriched in photosystem II, regulation of autophagy and carbohydrate phosphatase activity, which are essential for anther development. Functional annotation of genes targeted by miRNAs indicated that they are relevant to transcription and metabolic processes that play important roles in microspore development. An interaction network was built with 2 lncRNAs, 6 miRNAs and 10 mRNAs. Among these, miR396 and miR156 family were up-regulated, while their targets, genes (GROWTH REGULATING FACTORS and SQUAMOSA PROMOTER BINDING PROTEIN-LIKE genes) and lncRNAs, were down-regulated. Further, the trans-regulated targets of these lncRNAs, like wall-associated kinase2 and phosphomannose isomerase1, are involved in pollen wall formation during anther development.

**Conclusions:**

This study unravels lncRNAs, miRNAs and miRNA-lncRNA-mRNA networks involved in development of anthers of the tropical *C. oleifera* lays a theoretical foundation for further elucidation of regulatory roles of lncRNAs and miRNAs in anther development.

**Supplementary Information:**

The online version contains supplementary material available at 10.1186/s12864-022-08836-7.

## Background

Anther development is crucial for male fertility and sexual reproduction in plants [1]. Cells in an anther undergo meiosis to produce microspores, which further develop into mature pollen grains [2]. According to morphological features, anther development can be divided into 14 stages: during stages 1 to 4, cell division events occur within the developing anther primordia to establish characteristic structure of an anther. As pollen mother cells undergo meiosis at stages 5 and 6, they produce tetrads (stage 7); dissolution of the tetrad callose wall and release of microspores mark stage 8. These microspores undergo mitosis and develop into mature pollen grains during stages 9 to stage 12; this follows anther dehiscence, pollen release and stamen senescence at stages 13 to 14 [3]. Flowering is a complex reproductive process, which requires tight control of gene expression to form a regulatory network [4]. With the development of genomics and transcriptomics tools, genes which were involved in anther development have been widely studied. For instance, the genes involved in sporopollenin synthesis, such as TETRAKETIDE α-PYRONE REDUCTASE1 (*TKPR1*), ATP-Binding Cassette Transporter G26 (*ABCG26*) and POLYKETIDE SYNTHASE A (*PKSA*), play a significant role in pollen wall formation [5–7].

Following the advent of the high-throughput sequencing, a large number of non-coding RNAs (ncRNAs) were identified [8, 9]. Based on the product length, ncRNAs could be subdivided into two groups: long ncRNAs (lncRNAs, length > 200 nt) and microRNAs (miRNAs) that are about 21 nt [10]. Recent studies have revealed that plant lncRNAs may regulate gene expression in floral transition [11], flower development [12], flowering time regulation [13] and male sterility [14]. For example, Huang et al. [15] found 14 lncRNAs that were highly co-expressed with 10 pollen-associated target genes and might participate in pollen development in *Brassica rapa*. Further, many miRNA families are involved in flowering-related processes, such as the induction of floral competence, floral patterning, and the development of floral organs [4]. For example, miR156, miR156a and miR156b regulate SQUAMOSA PROMOTER BINDING PROTEIN-LIKE6 (*SPL6*), *SPL8*, *SPL12* and *SPL3* that are involved in cell proliferation in early anther development and regulation of flowering time [16–18]. In addition, miR396, miR396a and miR396b regulate GROWTH REGULATING FACTORS (*GRFs*) that are involved in development of the pistil and determination of floral organ specification [19–21].

*Camellia oleifera* is an evergreen shrub or a small tree, belonging to the family Theaceae, which is an important source of oil derived from woody plants. *C. oleifera* oil can be used for making a wide range of products, such as lubricants, detergent, rustproof oil, and biopesticides, medicines, cosmetics and functional foods that have high economic value [22]. Although, *C. oleifera* produces a large number of flowers, most of them are do not produce seeds in cultivation. Currently, most of the omics research is mainly focuses on the aspect of fruit development in *C. oleifera*, while the genes involved in flower bud development and their regulatory mechanisms remain to be elucidated.

Here, we have investigated the role of lncRNA and miRNA in the tropical *C. oleifera* anther development. We used PacBio single-molecule real-time (SMRT) sequencing, RNA sequencing (RNA-seq) and small RNA sequencing to identify and characterize mRNAs, lncRNAs and miRNAs differentially expressed in different developmental stages of anthers. Gene ontology (GO) analysis indicated that genes targeted by lncRNAs and miRNAs were involved in meiosis, pollen wall formation and microspore development. Additionally, we identified a network of interactions between ncRNAs and mRNAs that might participate in anther development. Overall, this study is helpful in not only understanding of roles of miRNAs and lncRNAs in the tropical *C. oleifera* anther development, but also provides a theoretical basis for further research on the anther development in *C. oleifera* and other industrial crops.

## Results

### Identification of key anther development stage in the tropical *C. oleifera*

**Fig. 1:**
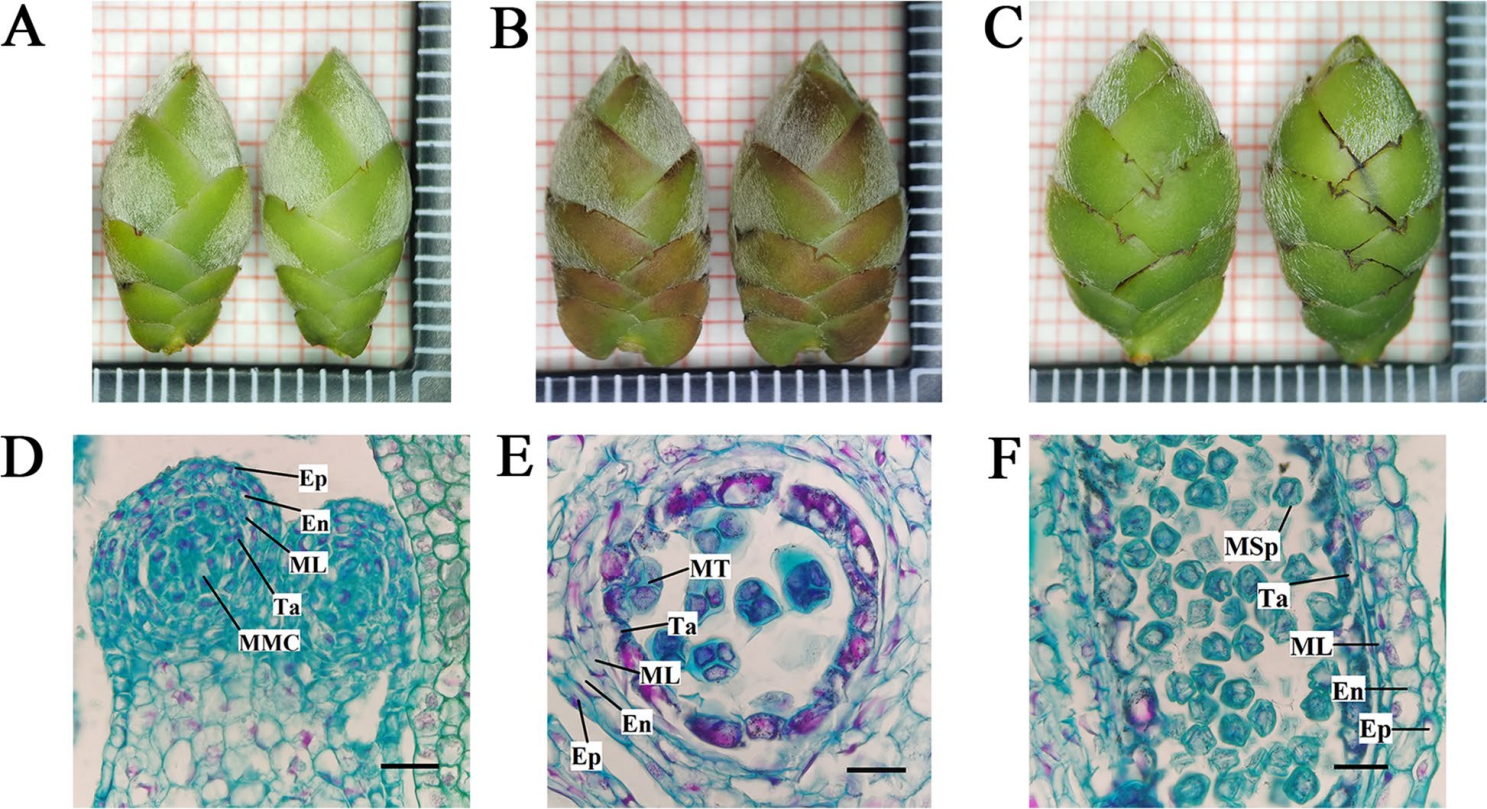
Development of the tropical *C. oleifera* anthers. External morphology of flower buds at pollen mother cells (**A**), tetrad (**B**) and uninucleate pollen (**C**) stages. **D** Anther in pollen mother cells stage. Thick callose walls appear outside the pollen mother cells, where all the anther cell types are present. **E** Anther in tetrad stage. Tetrad microspores have an irregular appearance. The cytoplasm of the tapetum is dense. **F** Anther in uninucleate pollen stage. Callose wall have degenerated, microspores are released, and their appearance is irregular, with a nucleus located centrally. The cells in the tapetum have begun to degenerate. En, endothecium; Ep, epidermis; ML, middle layer; MMC, microspore mother cell; MT, microspore tetrad; Ta, tapetum; MSp, microspores. The length of each grid in panels **A-C** is 1 mm, while bars in panels **D-F** are 50 μm

Phenotypic data was collected over 8 times of measurements. Striking contrast in floral bud appearance (length) was revealed in the 3rd round of measurement (Table 1; Fig. 1A-C). The analysis of paraffin section showed significant changes in the anthers during these measurements (Fig. 1). Specifically, microspore mother cells appeared at the 2nd measurement (Fig. 1D); at 3rd measurement, we found that meiosis had completed, tetrads were formed and pollen wall formation had begun (Fig. 1E). Subsequently, callose wall, surrounding the tetrads, had degenerated and individual microspores were released at the 4th measurement (Fig. 1F). These three stages corresponded to pollen mother cells stage (CoA1), tetrad stage (CoA2), and uninucleate pollen stage (CoA3) [3]. The floral buds from the 2nd measurement to the 4th measurement were chosen as samples for the follow up experiments.

**Table 1:** Growth measurements of floral buds of the tropical *C. oleifera*

Number of measurements	Length (mm)	Breadth (mm)	Thickness (mm)
1st	12.29^dx^	5.96^f^	5.05^e^
2nd	13.07^d^	6.24^ef^	5.24^de^
3rd	14.33^c^	6.87^de^	5.75^de^
4th	14.65^c^	7.23^d^	6.06^ cd^
5th	15.15^bc^	7.61^ cd^	6.65^bc^
6th	15.87^b^	8.18^bc^	7.23^b^
7th	16.99^a^	8.70^ab^	8.05^a^
8th	17.70^a^	9.09^a^	8.61^a^

^x^ Means with the same letter within the same column are not statistically significant difference (*p* < 0.05)

### Analysis of transcriptome data

To unravel the changes in genes expression during different stages of the tropical *C. oleifera* anther development, RNAs from three stages of anther, each with three biological replicates, were sequenced and analyzed by PacBio Sequel platform. A total of 391,875 polymerase reads (average read length of 72,247 bp) and 9,545,656 subreads (average read length of 2,895 bp) were produced with SMRT (Table 2). To derive a more accurate sequence information, a CCS was generated from reads that fully-passed at least twice through the insert, for a total of 346,466 CCSs (average read length of 3,141 bp) (Table 2). SMRTlink identified 289,298 full-length reads and 287,941 full-length non-chimeric (FLNC) reads (average read length of 2,980 bp; Table 2), respectively. The FLNC reads of the same transcript were clustered, and redundant reads were removed to obtain consensus reads using the ICE algorithm. Non-full-length non-chimeric reads were used to correct the consensus reads using arrow software, and 30,275 polished consensus sequences were ultimately obtained, with a mean length of 2,861 bp (Table 2). RNA-seq generated 63,625,261 (CoA1), 66,872,477 (CoA2), 67,554,470 (CoA3) raw reads for three stages, respectively. After trimming, 62,424,908 (CoA1), 65,373,048 (CoA2), 65,984,584 (CoA3) clean reads, respectively, were retained (Table 3). The raw reads of Illumina sequencing were then used to correct the SMRT data. After removing redundant sequences, a total of 18,393 transcripts, with a mean length of 2,812 bp, were retained.

**Table 2:** Summary of reads from SMRT sequencing

Item	Number	Mean length
Polymerase reads	391,875	72,247
Subreads	9,545,656	2,895
Circular consensus sequences (CCS)	346,466	3,141
Full-length non-chimeric reads (FLNC)	287,941	2,980
Polished consensus reads	30,275	2,861
Corrected polished consensus reads	30,275	2,861
Full-length transcripts (genes)	18,393	2,812

**Table 3:** Summary of Illumina sequencing

Library	Groups	Raw reads		Clean reads		Clean bases(G)	
		Total	Average	Total	Average	Total	Average
RNA-seq	CoA1	63,625,261	21,208,420.33	62,424,908	20,808,302.67	18.73	6.24
	CoA2	66,872,477	22,290,825.67	65,373,048	21,791,016	19.62	6.54
	CoA3	67,554,470	22,518,156.67	65,984,584	21,994,861.33	19.79	6.60
	Total	198,052,208	66,017,402.67	193,782,540	64,594,180	58.14	19.38
Small RNA-seq	CoA1	38,868,735	12,956,245	38,084,176	12,694,725.33	1.90	0.63
	CoA2	39,821,973	13,273,991	39,286,803	13,095,601	1.96	0.65
	CoA3	38,862,444	12,954,148	38,320,966	12,773,655.33	1.92	0.64
	Total	117,553,152	39,184,384	115,691,945	38,563,981.67	5.79	1.93

The sequencing of small RNAs unveiled the dynamic regulation of miRNAs on gene expression during anther development. After sequencing of nine small RNA libraries, a total of 117,553,152 high quality reads were obtained from the CoA1 (38,868,735), CoA2 (39,821,973) and CoA3 (38,862,444), respectively (Table 3). After removing contaminant and low quality reads, 38,084,176 (CoA1), 39,286,803 (CoA2) and 38,320,966 (CoA3) clean reads, respectively, were obtained in the size range of 18–30 nt.

### Identification and characterization of lncRNA and miRNA

**Fig. 2:**
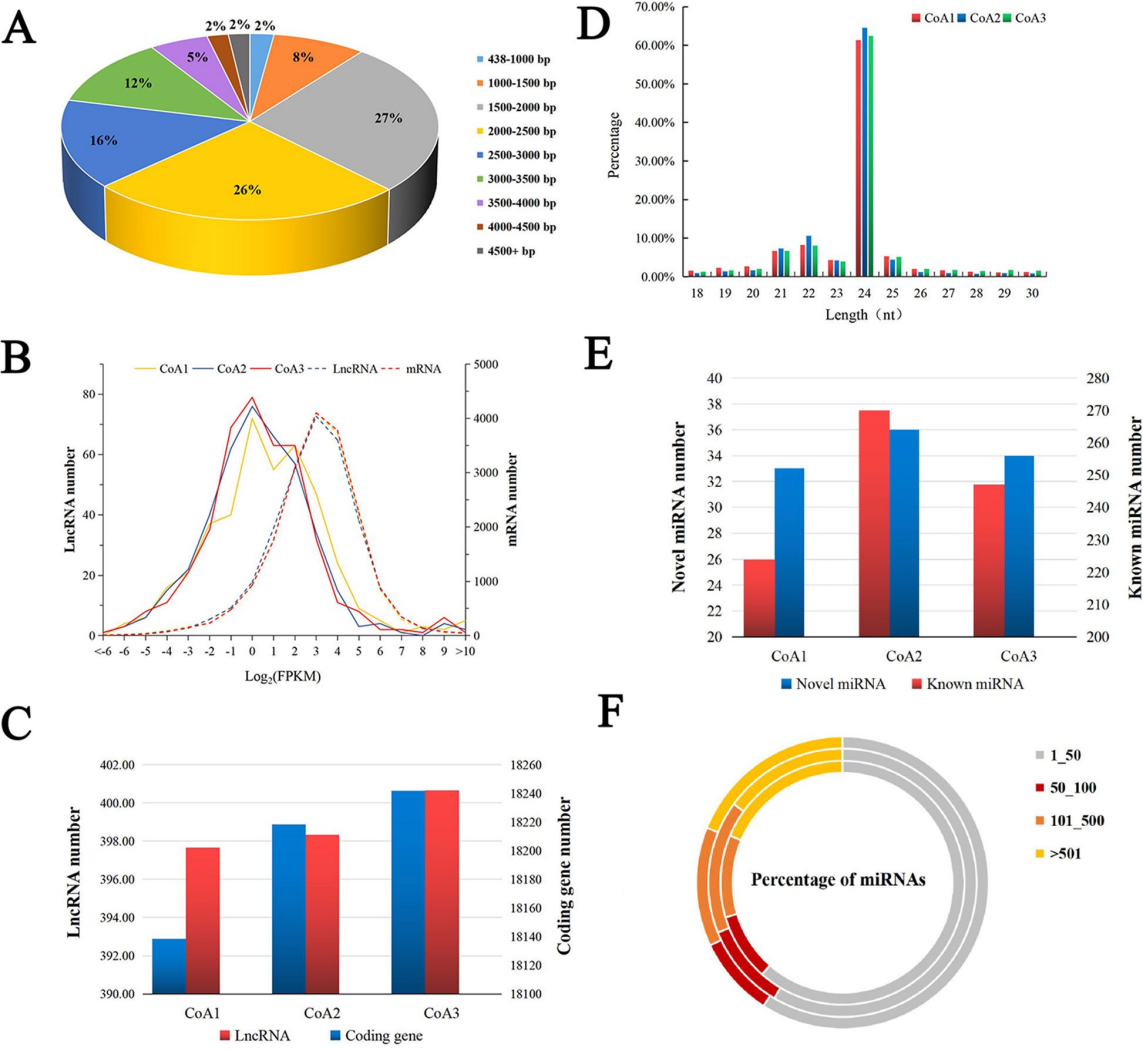
Characteristics of lncRNAs and miRNAs identified in the tropical *C. oleifera*. **A** Length distribution of lncRNAs. **B** Accumulation levels of lncRNAs. **C** The number of lncRNAs and coding genes expressed. **D** Length distribution of the clean sRNAs. **E** The number of known and novel miRNAs. CoA1, CoA2 and CoA3 represent floral buds at three developmental stages in pollen mother cells, tetrad and uninucleate pollen stages, respectively, of *C.oleifera*. **F** MiRNA accumulation levels. The inside, middle and outer rings represent CoA1, CoA2, and CoA3, respectively

During the tropical *C. oleifera* anther development, a total of 414 transcripts were annotated as lncRNAs (Supplementary Fig. S1 and Supplementary Table S1). These 414 putative lncRNA sequences ranged from 438 bp to 5,637 bp, with an average length of 2,367 bp (Fig. 2A and Supplementary Table S1). About half of the lncRNAs (53%) had lengths ranging from 1,500 bp to 2,500 bp, while only 10% were shorter than 1,500 bp (Fig. 2A). By combining the results of Minimap2 and FEELnc classifications, 97 lncRNAs were classified into four groups: 29 sense lncRNAs (29.90%), 61 lincRNAs (62.89%), 3 antisense lncRNAs (3.09%) and 4 sense intronic lncRNAs (4.12%) (Supplementary Table 1). Most of the lncRNAs were expressed at relatively low levels, with FPKM values (log_2_) ranging from -1 to 1 in all samples, as compared to expression of mRNAs (Fig. 2B). The number of lncRNAs slightly increased over time during development, a trend also found in the mRNA component of transcriptome (Fig. 2C).

Clean sRNAs reads ranged from 15 to 30 nucleotides (Fig. 2D). The majority of clean sRNA reads were 21–25 nt, and the most abundant sRNAs were 24 nt long, representing more than 60% of the total clean reads. The 21-nt sRNAs, which were mainly miRNAs, were the third most abundant size class. The abundance of 21- and 24-nt sRNAs were higher in the CoA2 library as compared with CoA1 and CoA3 libraries. A total of 335 known and 37 novel miRNAs were identified, which accounted for 224 (CoA1), 270 (CoA2) and 247 (CoA3) known miRNAs, and 33 (CoA1), 36 (CoA2) and 34 (CoA3) novel miRNAs, respectively (Supplementary Table S2 and Fig. 2E). The first base of known miRNAs was dominantly a ‘U’, whereas ‘A’ base ratio was largest in novel miRNAs (Supplementary Fig. S2 and S3). The expression dynamics of miRNAs in three stages of anthers were analyzed using TPM values (Supplementary Table S2). About 40% miRNAs exhibited high-levels of expression (≥ 50 TPM) [23]: about 9% of these showed TPM values between 50 and 100, about 13% miRNAs had TPM values between 101 and 500, and about 17% had TPM values > 501 (Fig. 2F). Top 30 miRNAs (based on TPM) belonged to ten families of miR166, miR159, miR160, miR396, miR168, miR403, miR408, miR167, miR319, and miR395.

### Analysis of differentially expressed mRNAs, lncRNAs, and miRNAs in anther development

**Fig. 3:**
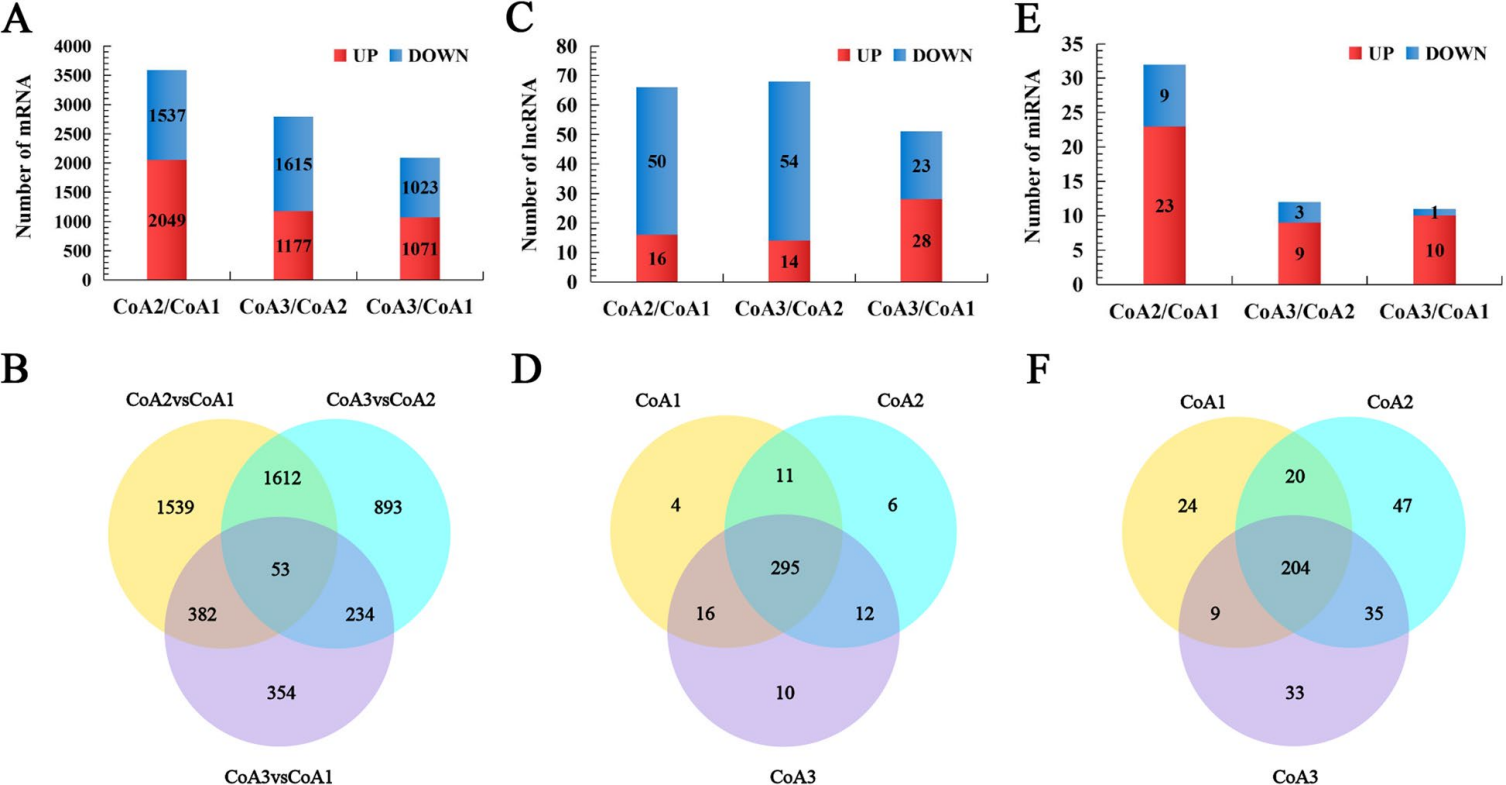
Analysis of mRNA, lncRNA and miRNA accumulations in three developmental stages of anther in the tropical *C. oleifera*. Column diagrams representing the numbers of differentially expressed genes (DEGs; **A**), differentially accumulated lncRNAs (**C**) and differentially accumulated miRNAs (**E**). **B** Venn diagram showing total numbers of DEGs identified in CoA2 vs. CoA1, CoA2 vs. CoA3, and CoA3 vs. CoA1, and overlap among these comparison groups. **D** Venn diagram showing the total number of lncRNAs identified in CoA1, CoA2 and CoA3 and overlap amongst these groups. **F** Venn diagram showing the total number of miRNAs identified in CoA1, CoA2 and CoA3 and the overlap amongst these groups

To investigate the gene expression patterns at three stages of the tropical *C. oleifera* anther development, the FPKM values were used to normalize the reads from RNA-seq. A total of 5,324 mRNAs were differentially expressed (Fig. 3A and Supplementary Table S3) between three developmental stages. The largest number of differentially expressed mRNAs (3,586) were found by comparing CoA2 vs. CoA1, with the development of floral bud, the number of differentially expressed mRNAs decreased gradually (Fig. 3A). Of 5,324 mRNAs, 1,593, 893 and 354 mRNAs were uniquely differentially expressed in CoA2 vs. CoA1, CoA3 vs. CoA2 and CoA3 vs. CoA1, respectively, and 53 mRNAs were differentially expressed in all control groups (Fig. 3B). GO enrichment analysis of significantly differentially expressed mRNAs revealed seven significant terms of biological processes among up-regulated mRNAs in CoA2 vs. CoA1 (Supplementary Table S4). The most significantly enriched processes were ‘microtubule-based movement’ and ‘carbohydrate metabolic process’. Seventeen terms were significant among down-regulated mRNAs in CoA2 vs. CoA1, where most enriched terms were ‘regulation of transcription, DNA-templated’ and ‘regulation of cellular metabolic process’ (Supplementary Table S4). After the exploration of functional catagories of differentially expressed mRNA, we found 14 genes related to pollen development, pollen wall formation, flowering time control, which may play important roles in floral bud development of the tropical *C. oleifera* (Supplementary Table S5).

A total of 115 lncRNAs were differentially accumulated in three developmental stages. With the development of anther, the number of differentially expressed lncRNAs gradually increased (Fig. 3C). The majority of lncRNAs (295) were detected in all the three developmental stages (Fig. 3D).

**Fig. 4:**
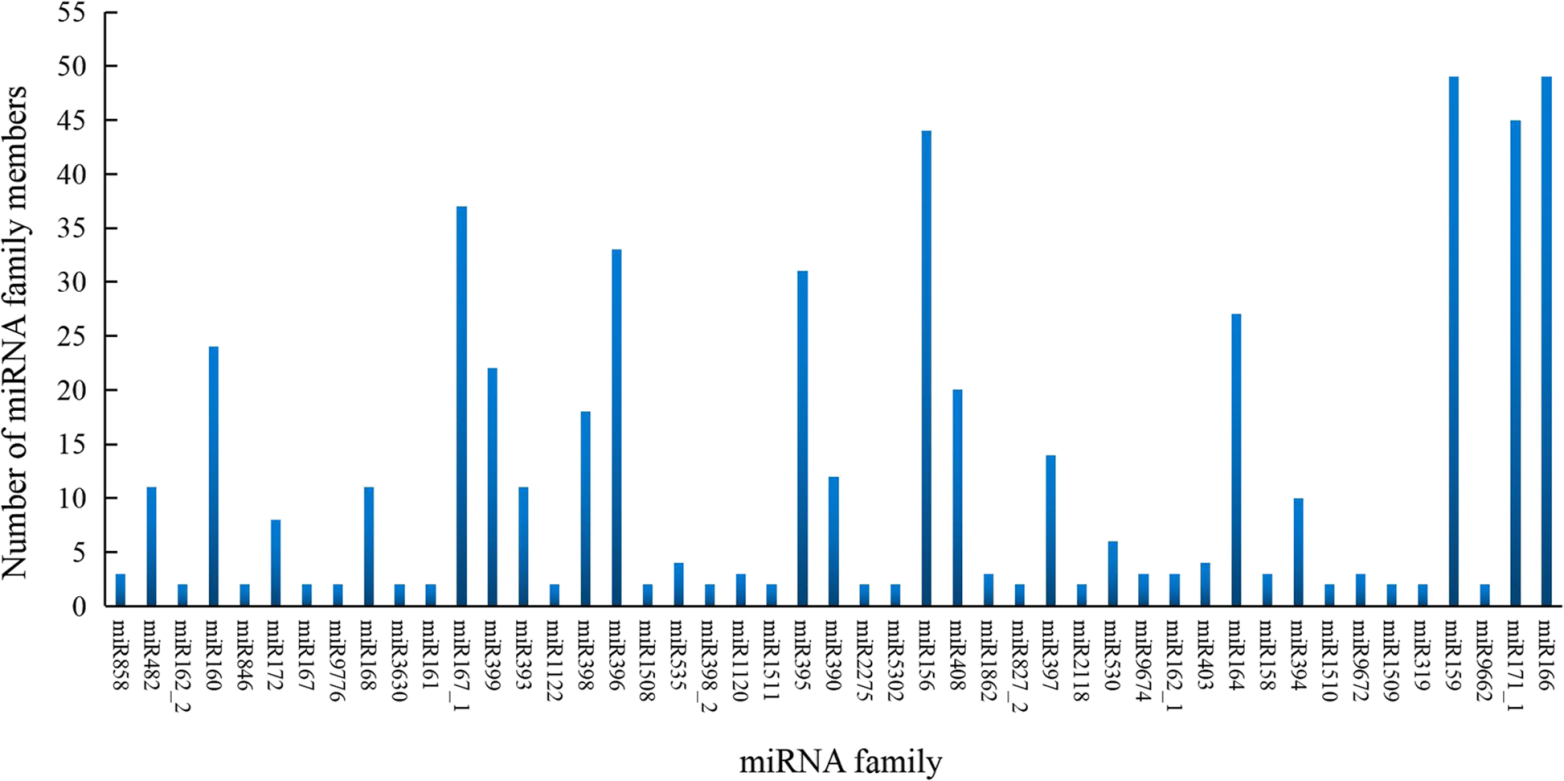
Number of miRNAs identified from known miRNA families

A total of 44 miRNAs were differentially accumulated amongst the three stages at a threshold of |log_2_FC|≥ 1 and q-value ≤ 0.05 (Fig. 3E), the largest number of differentially accumulated miRNAs (32) were found by comparing CoA2 vs. CoA1. However, we did not detect significant differences in accumulations of novel miRNAs across three developmental stages. Comparative analysis of 372 miRNAs (335 known and 37 novel miRNAs) showed that 204 miRNAs were common in three stages, while 24, 47 and 33 miRNAs were specific to CoA1, CoA2, and CoA3, respectively (Fig. 3F). These known miRNAs were assigned to 74 known families, of which 36.49% of the families contained a single member, 14.86% contained more than 20 members, and miR166 and miR159 families have 49 members (Fig. 4).

### Target genes identification and analysis of differentially accumulated lncRNA and miRNA in anther development

A total 65 genes were predicted as potential trans-regulated targets of 48 differentially accumulated lncRNAs (Supplementary Table S6). The correlation analysis between lncRNAs and their potential target genes showed that 88.7% had a positive, while 11.3% had a negative correlation. The GO enrichment analysis of the target genes of differentially expressed lncRNAs in CoA2 vs. CoA1 and CoA3 vs. CoA2 comparisons were enrichment in the same 26 GO terms, such as ‘alditol metabolic process’, ‘regulation of autophagy’ and ‘carbohydrate phosphatase activity’ (Supplementary Table S7). Additionally, the target genes of up-regulated lncRNAs were significant enriched in photosystem-related GO term such as ‘photosystem II reaction center’ and ‘photosynthetic membrane’ (Supplementary Table S7). The KEGG enrichment indicated that the target genes of up-regulated lncRNAs were significant enriched in 3 pathways, ‘ascorbate metabolism, ‘aldarate metabolism’ and ‘ribosome’; whereas target genes of down-regulated lncRNAs were significant enriched in 9 pathways that included ‘RNA polymerase’, ‘phagosome’, ‘starch and sucrose metabolism’ (Supplementary Table S8). Additionally, 44 target genes of 37 differentially accumulated lncRNAs were differentially expressed in the three developmental stages. Further, gene annotation indicated that 10 differentially accumulated lncRNAs that were highly co-expressed with 7 differentially accumulated target genes, such as AUXIN RESISTANT1 (*AXR1*), Phosphomannose isomerase1 (*PMI1*), wall associated kinase2 (*WAK2*) and Dicer-Like-3b (*DCL3b*). These were related to transcription, cell wall formation, and flowering time control, which indicated these lncRNAs may play important role in floral bud development in the tropical *C. oleifera* (Supplementary Table S9).

A total of 286 genes were predicted as putative targets of 44 differentially accumulated miRNAs, resulting in a total of 570 putative miRNA-target modules, as several miRNAs and targets displayed one-to-many relationships (Supplementary Table S10). Among these, 527 modules were predicted to function through miRNA-directed cleavage, while 43 modules might function through translational inhibition (Supplementary Table S10). Further, 54 (18.9%) target genes were mapped to transcription factors belonging to 15 families, amongst which, GRF, HD-ZIP, GRAS, ARF, SBP and TCP were significantly enriched. Additionally, 92 differentially accumulated genes were predicted as targets of 30 differentially accumulated miRNAs. Further, Pearson’s correlation analysis indicated that 14 differently accumulated miRNAs were negatively correlated with 25 differentially accumulated target genes, and GO enrichment analysis indicated that these genes were enriched in 75 terms, such as ‘regulation of transcription, DNA-templated’, ‘intracellular organelle’ and ‘organic cyclic compound binding’ (Supplementary Fig. S4A). KEGG enrichment indicated that 14 target genes were enriched in 4 pathways, which included ‘oxidative phosphorylation’ and ‘ubiquinone and other terpenoid-quinone biosynthesis’ (Supplementary Fig. S5A).

**Fig. 5:**
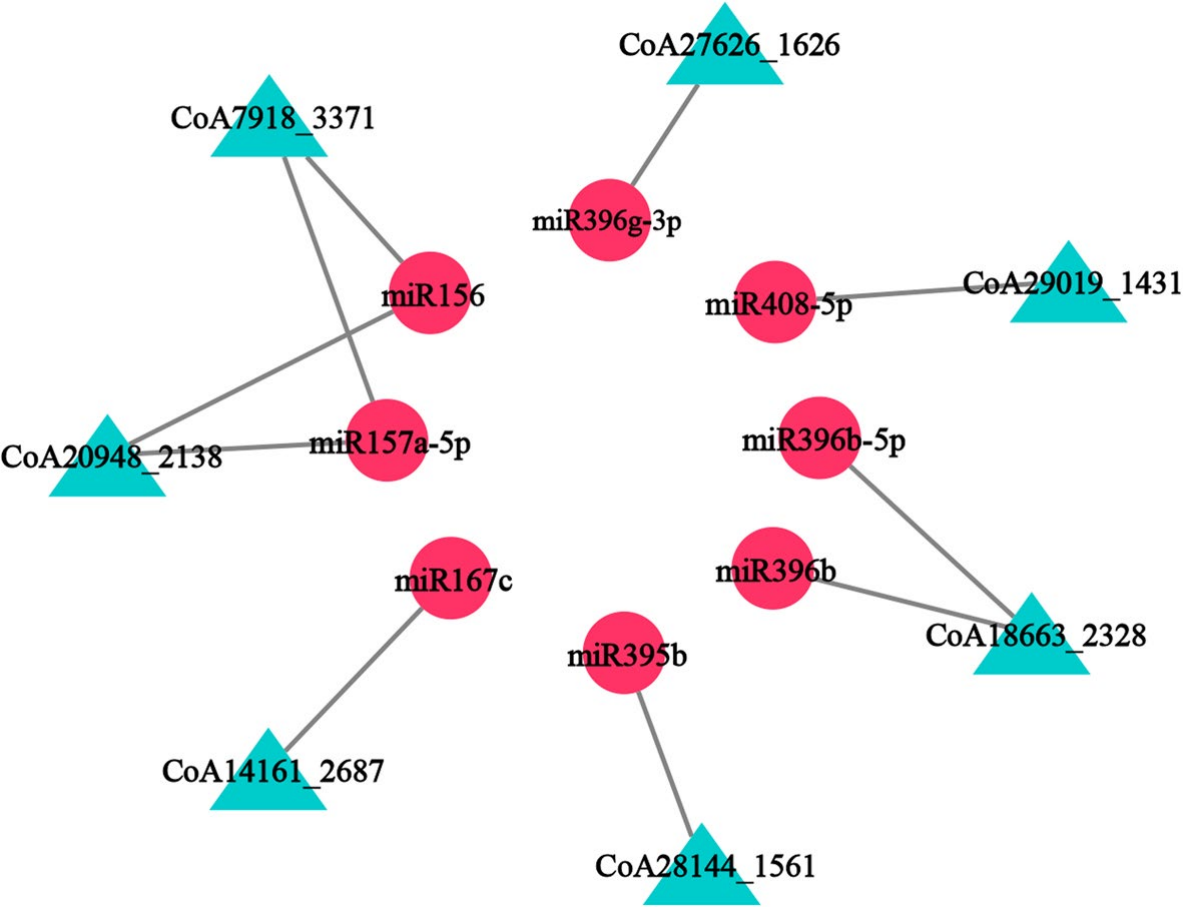
A interaction diagram of miRNAs regulating differently accumulated lncRNAs. Rose red rounds represent miRNA, whereas lncRNAs are represented by the blue triangles

Next, we examined whether the lncRNAs could be targets of miRNAs. Indeed, psRNATarget program predicted 42 differentially accumulated lncRNAs as targets of 27 differentially accumulated miRNAs (Supplementary Table S11). Moreover, 27 differentially accumulated genes were targeted by these 42 differentially accumulated lncRNAs. Further, Pearson’s correlation analysis indicated that 8 differently accumulated miRNAs were negatively correlated with 7 differentially accumulated lncRNAs, which target 11 differentially accumulated gene. These genes were enriched in 14 GO terms, such as ‘glutathione catabolic process’ and ‘omega peptidase activity’ (Supplementary Fig. S4B). KEGG enrichment indicated that the target genes were enriched in 2 pathways, ‘RNA polymerase’ and ‘Fructose and mannose metabolism’ (Supplementary Fig. S5B). Based on these results, we investigated miRNA-lncRNA relationships and modeled a interaction diagram of 7 miRNAs and 8 lncRNAs. It was evident that one lncRNA could potentially be targeted by 1–2 miRNAs and one miRNA could target 1–2 lncRNAs (Fig. 5).

### Constructing miRNA-lncRNA-mRNA gene regulatory networks in the developing anthers

**Fig. 6:**
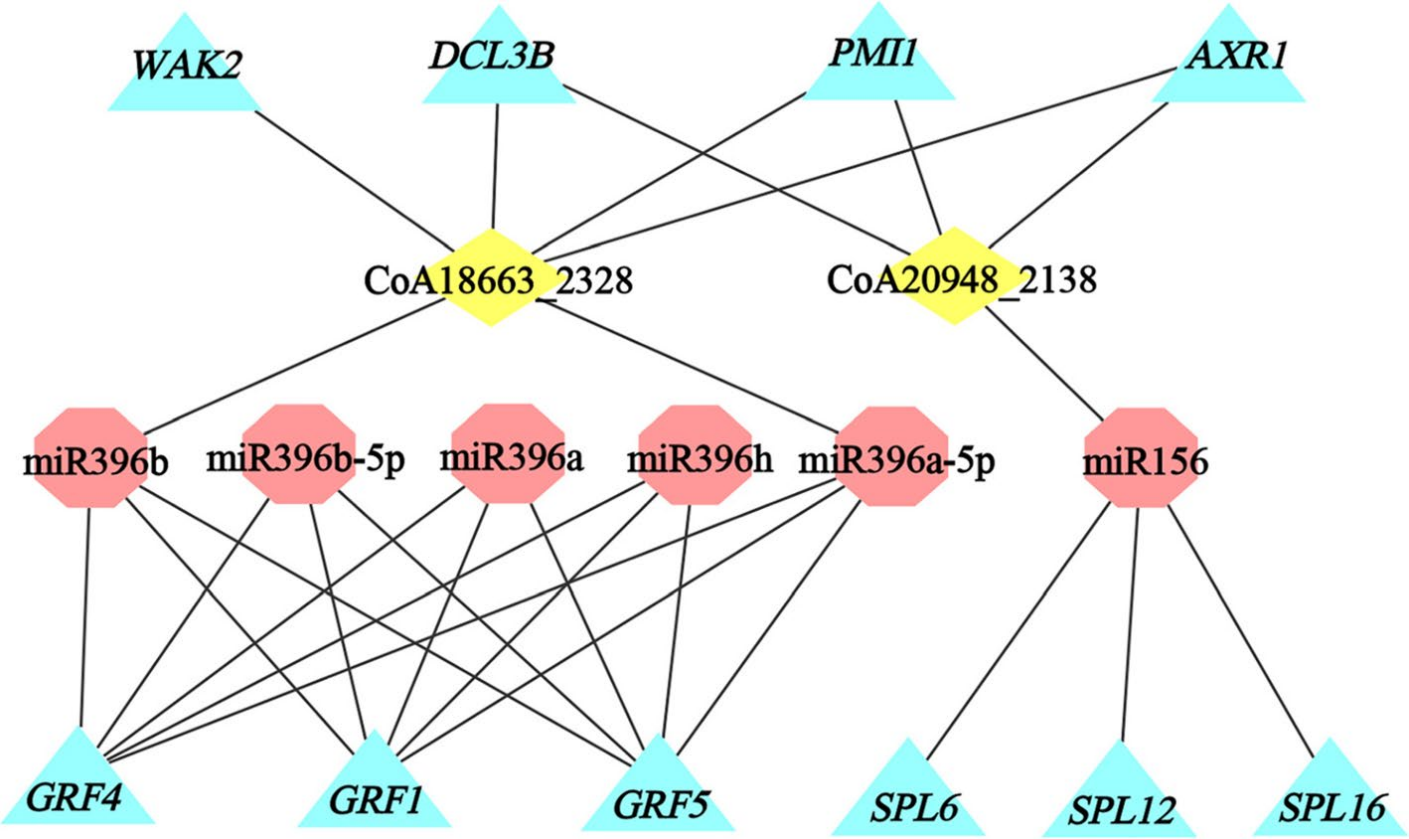
A key regulatory network of miRNAs, lncRNAs and mRNAs in the tropical *C. oleifera* anther development. Red octagons are miRNA, yellow rhombus are lncRNAs and blue triangles are mRNAs

We further explored the regulatory mechanisms conferred by miRNAs during anther development. Accordingly, we selected differently accumulated genes that were targeted by lncRNAs (CoA18663_2328 and CoA20948_2138) as well as were associated with pollen wall formation (*WAK2* and *PMI1*), meiosis and transcription (*AXR1* and *DCL3b*) to construct an interaction network with miRNAs and their target genes (Fig. 6). This network defined connections between 6 miRNAs and 6 genes involved in anther development. Among them, the member of miR396 family (miR396a, miR396b, miR396a-5p, miR396b-5p, miR396h) modulated the transcript levels of genes related to formation of pollen mother cells and meiosis through their targeted regulation of *GRF* family (*GRF1*, *GRF4* and *GRF5*; Fig. 6). Additionally, miR156 targeted *SPL* family, such as *SPL6*, *SPL12* and *SPL16*, and were involved in early anther development (Fig. 6). In this network, all miRNAs were up-regulated, whereas 2 lncRNAs and 10 mRNAs were down-regulated (Supplementary Table S12).

### Validation of gene expression by quantitative reverse transcription PCR (qRT-PCR)

**Fig. 7:**
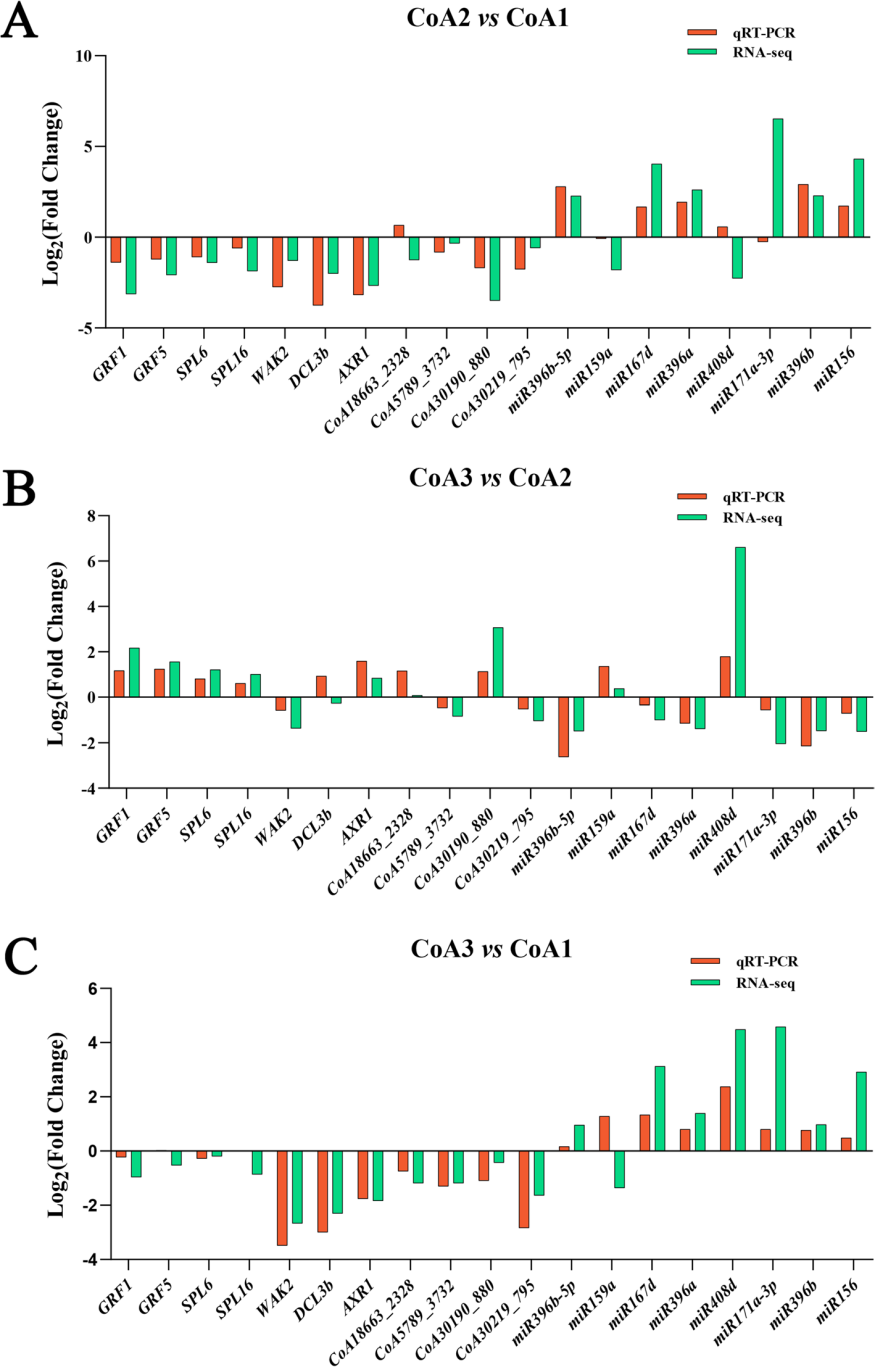
qRT-PCR of the expression levels of miRNAs, lncRNAs and genes in anther from three developmental stages. The *TubA* was used as internal controls for lncRNAs and mRNAs in A, B and C; the *5S* were used as internal controls for miRNAs in A, B and C

To verify the accuracy of the RNA-Seq and small RNA-seq results, the relative expression levels analysis of 4 lncRNAs, 7 genes and 8 miRNAs were investigated with qRT-PCR. According to the results obtained from qRT-PCR, the expression pattern of most selected miRNAs, lncRNAs and genes were largely similar with the expression levels calculated using our sequencing data (Fig. 7). Although the fold-change (FC) values detected by qRT-PCR did not exactly match the FC values which calculated by sequencing, but the expression profiles were basically consistent for all selected miRNAs, lncRNAs and genes tested, which indicated that the sequencing data were reliable.

## Discussion

### Transcriptome mRNA in the anther development of the tropical *C. oleifera*

Anther development is a multistage and complex process that determines yield and quality of most of the industrial crops. This process is controlled by a complex network of numerous genes [24, 25]. With a plethora of plant genomes now sequenced, -omics driven technologies are playing a significant role in the study of the regulatory mechanism of anther development [25, 26]. Still, the identification and functional exploration of *C. oleifera* molecular regulatory network during anther development had been very limited, as previous investigations were mainly focused on cytological studies and single regulatory genes [27–29]. In this study, the anther development in the tropical *C. oleifera* was morphologically and cytologically monitored on temporal scales, and important stages were selected for sequencing, to systematically identify the mRNAs, lncRNAs and miRNAs, and their regulatory network.

Several studies have reported direct correlation of floral bud size and other inflorescence characteristics to anther and microspore development, as it is shown that the development of anther and microspore advances as the size of floral buds increase [30–32]. For instance, the meiotic phase of pollen mother cell can be predicted accurately by using the length-breadth ratio of floral bud in the tropical *C. oleifera* [29]. Our morphological observations and analysis of paraffin sections, combined with previous studies [27, 28], also show that significant changes in floral bud lengths reflected the changes in anthers. Therefore, it suggests that the morphological characteristics of floral bud could effectively judge the developmental stages of the tropical *C. oleifera* anthers.

In order to explore the mechanism underlying anther development, data on transcriptome of the three development stages of anther were obtained by using SMRT sequencing and RNA-seq. By combining SMRT with Illumina sequencing, 30,275 complete transcripts were obtained, which demonstrated a more complete assembly of transcriptome than by Huddleston et al. [33]. Differences in expression of regulatory genes at various developmental stages of anthers was evident, where the largest number of DEGs (3,586) were found by comparing CoA2 vs. CoA1. These observations are supported by previous studies in *Arabidopsis* [34] and *Hordeum vulgare* [35] regarding large-scale transcriptional re-organization in pollen mother cells when they enter meiosis.

The pollen walls maintain pollen structure, provide protection from various environmental stresses and preserve pollen germination [36]. Previous studies have found that pollen walls were the most intricate cell walls in plants. They are composed of exine and intine layers that are formed in a complex series of biological events involving a large number of genes [37]. Callose synthesis has a vital function in building a properly sculpted exine, whose integrity is essential for pollen viability [38]. Recent research shows that callose synthase5 (*CalS5*) and AUXIN RESPONSE FACTOR17 (*ARF17*) are important to callose wall formation [38, 39]. In our study, *CalS5* and *ARF17* were significantly up-regulated at the tetrad stage, which indicated that they may regulated callose wall formation of the tropical *C. oleifera*. Additionally, we found that *A6*, which can regulate the degradation of callose wall and is conducive to the transformation from tetrad wall to pollen wall [40], was significantly up-regulated at uninucleate pollen stage, and might may play a similar role in the tropical *C. oleifera*. Further, sporopollenin is an important component of pollen walls and it is synthesized by a pathway of approximately eight genes in *Arabidopsis* [41]. Previous studies have shown that ABORTED MICROSPORES (*AMS*) plays a central role in coordinating sporopollen biosynthesis and the secretion of materials for pollen wall patterning [42]. *AMS* can directly regulate the expression of genes [43] involved in processes like fatty acid hydroxylation (*CYP703A2* and *CYP704B1*) [5, 44], phenolic synthesis (*PKSB* and *PKSA*, *TKPR1* and *TKPR2*) [6, 7], and lipid transport (*ABCG26*) [45]. In our study, *AMS*, *CYP703A2*, *CYP704B1*, *PKSB*, *PKSA*, *TKPR1*, *TKPR2*, and *ABCG26* were continuously up-regulated during anther development, indicating that these genes may play important roles in the establishment of the tropical *C. oleifera* pollen wall. The expression of *MYB80* [46] and *RPK2* [47], which regulates programmed cell death (PCD) of tapetum, is highest in the tetrad stage, which may indicate that more cells of tapetum started undergoing PCD in the tetrad stage; this might benefit the development of pollen wall in the tropical *C. oleifera*. Additionally, some genes related to the regulation of flowering time, such as *EBS* [48] and *JMJ30* [49], were also differentially expressed in the three stages of anther development, and hints towards their engagement in regulating the process of anther development in the tropical *C. oleifera*. The genes identified here would provide a reference for future studies on anther development in *C. oleifera*.

### LncRNAs are involved in the regulation of anther development in the tropical *C. oleifera*

LncRNAs are unravelling as key regulators of plant development [50], and their role in plant sexual reproduction has been confirmed. For example, lncRNAs related to sexual reproduction have been systematically identified in *Gossypium hirsutum* [24], *Hordeum vulgare* [35] and *Brassica rapa* [15], but their role in the development of male buds of *C. oleifera* had still remained unknown. Here, we have identified a total of 414 putative lncRNAs in three different development stages of *C. oleifera* anthers by RNA-seq.

Previous studies in *B. rapa* [15] and Populus [51] indicated that lncRNAs are shorter and accumulated at significantly lower levels than protein-coding transcripts. Our study showed that the average length of lncRNAs was 2,367 bp, whereas mRNAs were of an average length of 2,811 bp. On the other hand, the accumulation level of these lncRNAs was lower than those of mRNAs, which was similar to previous studies [15]. LncRNAs could regulate gene expression in trans-acting manner [51]; such as trans-acting lncRNAs have been discovered in large numbers recently [52]. Here, trans-acting functions of lncRNAs were explored with the help of co-expression analyses, where a total of 65 genes were predicted as potential trans-regulated targets of 48 differentially accumulated lncRNAs. Previous studies have shown that autophagy is required for post meiotic anther development, including PCD-mediated degradation of the tapetum in rice [53, 54]. We found that the target genes of lncRNAs were enriched in the GO terms of ‘autophagy’ and in the KEGG pathway of ‘phagosome’, which indicated that these lncRNA may regulate the expression of genes related to autophagy and participate in the context of anther development. Additionally, several target genes have been involved in anther development and regulation of flowering time, such as *VTC2*, *PMI1*, and *WAK2*. Previous studies have suggested that an increase in ascorbic acid content delays flowering [55], and that *VTC2* is necessary for ascorbate generation [56]. Additionally, *PMI1* [57], and *WAK2* are involved in cell wall biogenesis [58], and these genes might also be involved in the formation of pollen walls. This result further indicates that lncRNAs are likely to play a regulatory role in anther development in the tropical *C. oleifera*.

### MiRNAs regulated lncRNAs and mRNAs in anther development in the tropical *C. oleifera*

MiRNAs are reported to play essential roles in flowering by directing RNA cleavage or inhibiting translation of the target transcripts [59]. Here, we studied the regulatory roles of miRNAs during anther development in the tropical *C. oleifera* with the help of small RNA-seq analysis. We found that 73% of differentially expressed miRNAs were concentrated in CoA2 vs. CoA1, indicating a large amount of regulatory changes between pollen mother cells stage and tetrad stage. The GO analysis of target genes of differentially expressed miRNAs showed that 9 most significantly enriched pathways belonged to ‘RNA metabolism’ category, which is mainly involved in transcriptional regulation. These findings support the previous reports on transcription-related genes as key miRNA targets during anther development [60, 61].

The *SPL* family of transcription factors is functionally diverse, their members in different plants species are involved in many fundamental aspects of plant growth and development, including juvenile-to-adult and floral phase transitions [62], pollen development [17], grain yield [63] and leaf initiation rate [64]. MiR156 is one of the most conserved miRNA families in plants [65]. It regulates plant growth and development, including vegetative to reproductive phase transition and floral organ development by regulating *SPLs* [66, 67]. For example, Xing et al. [17] found that miR156 targeted *SPLs* were absolutely required for male fertility in *Arabidopsis* and they worked in early anther development. Here, we identified a strong negative co-expression relationship between *SPLs* (*SPL6*, *SPL12* and *SPL16*) and miR156 during anther development of the tropical *C. oleifera*. We found that *SPLs* had high transcript levels, while miR156 had a low transcriptional level at pollen mother cell stage. These have significantly opposite expression levels at tetrad stage, which indicates that miR156-*SPL* network may play an important role in cell proliferation and meiosis in early anther development of the tropical *C. oleifera*.

MiR396 is also evolutionarily conserved among plant species [68]. Previously, it was reported that the miR396-*GRF* network is an important module affecting plant growth. For example, Yu et al. [69] found that during the grain filling stage of wheat, miR396 is involved in the development of grains by regulating the expression of *GRFs* (*GRF1*, *GRF6*, and *GRF9*). Baucher et al. [19] and Liang et al. [20] found this network plays an important role in proper development of the pistils and determination of floral organ specification. Additionally, some studies have implicated the GRF-GRF-INTERACTING FACTOR (*GIF*) in the specification and formation of archesporial cells and competence of carpel margin meristems [70–72]. Here, we found that *GRFs* (*GRF1*, *GRF4*, and *GRF5*) were targeted by miR396 with a strong negative correlation. Specifically, miR396s had low levels of accumulation during pollen mother cell stage, which significantly increased in the tetrad stage, while the *GRFs* have opposite expression levels. Additionally, we also found *GIF1* followed the same expression-pattern like the *GRFs*, which indicates that miR396-*GRF*-*GIF1* network may play an important role in the formation of pollen mother cells and meiosis of the tropical *C. oleifera*.

Next, the co-expression relationship between miRNA and lncRNA were calculated by Pearson correlation coefficient. We found that two lncRNAs are negatively co-expressed with miR396- and miR156-members, respectively. Further, analysis of the target genes of lncRNAs showed that they were related to anther development. For example, *AXR1* is required for normal auxin response and is related to DNA repair [73, 74]. *AXR1* was highly expressed in pollen mother cell stage, indicating that it may participate in meiosis of pollen mother cell. Additionally, some of lncRNA-target genes were involved in cell wall biogenesis (*PMI1*, *WAK2*), and in small RNA biogenesis pathway (*DCL3b*) [75]. These results strongly implicate lncRNAs with roles in the tropical *C. oleifera* anther development. We have build a comprehensive network of RNA-mediated interactions, putting together the miRNA-lncRNA, miRNA-mRNA, and lncRNA-mRNA interactions, which consists of 6 miRNAs, 2 lncRNAs, and 10 mRNAs. This network shows that the mutual regulation of miRNA, lncRNA and mRNA may play a crucial role in the tropical *C. oleifera* anther development.

## Conclusions

Here, we performed a comprehensive characterization of miRNAs and lncRNA from pollen mother cell, tetrad and uninucleate pollen stages, which were resolved as critical during the tropical *C. oleifera* anther development, and predicted their potential roles. By simultaneously deploying multiple transcriptome-profiling technologies, a total of 5,324 genes, 115 lncRNAs, and 44 miRNAs were identified and their patterns of differential accumulation during three stages were analyzed. Moreover, miRNAs (like miR396a and miR156) and lncRNAs (CoA18663_2328 and CoA20948_2138), along with their target genes (such as *GRF4*, *SPL6*, *WAK2* and *PMI1*), which associated with early development of anthers, pollen wall formation and flowering time regulation, were used to build an interaction network to comprehensively understand their role in anther development. Overall, this study identified potential miRNA-lncRNA-mRNA networks involved in the tropical *C. oleifera* anther development and lays down a genomics foundation for further investigations.

## Methods

### Plant materials

*C.oleifera* has a long history of cultivation in Hainan [76, 77]. Through natural domestication and breeding, it has adapted to the local climate of Hainan and has excellent economic characteristics. The plant material was identified and collected by Jinhui Chen, Jian Wang and Hanggui Lai (Hainan University) from the mountain in Tunchang district of Hainan Province, and was planted in the tropical *C. oleifera* germplasm resource bank in Hainan University (Danzhou, Hainan, China; 109°29′25″ E, 19°30′40″ N) by grafting. The voucher specimens have been deposited in the herbarium of Key Laboratory of Genetics and Germplasm Innovation of Tropical Special Forest Trees and Ornamental Plants, Hainan University, China. Floral buds were collected from 4-years old clonal *C. oleifera* trees at the tropical *C. oleifera* germplasm resource bank. The preliminary experiments in this study found that the development time of flower of the tropical *C. oleifera* was long, which takes about 9 months, the morphological differentiation and development of floral organs may take place from May to December. Therefore, measurements and acquisitions were carried out an interval of every ten days, between 24 September to 4 December 2020. The three developmental stages of anthers were selected for this study on the basis of phenotypic and histological observations as described below. These three stages corresponded to pollen mother cells stage (CoA1), tetrad stage (CoA2), and uninucleate pollen stage (CoA3).

### Measurement and anatomical observation on floral buds plant materials

Five clonal *C. oleifera* trees with same growth status were selected, and then ten similarly sized floral buds in the same place in every tree were randomly selected; their length, breadth and thickness were measured by an electronic vernier calliper (IP54, Shangshen, Shanghai, China) at an interval of every ten days. Data was analyzed with the help of SPSS version 26 software (SPSS Statistics, IBM, New York, USA). Additionally, fifteen similarly sized floral buds were gently excised, six of these floral buds were used for paraffin section experiment and another nine floral buds were used for RNA isolation. The scales were removed with the help of tweezers, 1/3 of the floral bud tissue was longitudinally cut by a sharp blade until a part of the anther was exposed. Then, the remaining (2/3 of the floral bud) tissues were immersed in FAA stationary solution (70% ethanol:formaldehyde:acetic acid, 9:1:1) immediately, which was vacuum infiltrated (25 Pa) for 30 min to fully eliminate the air from the material. These were fixed overnight at 4℃, after which the samples were dehydrated in a graded ethanol series (70%, 85%, 90%, 95% and 100% twice), made transparent with the help of N-butanol, and embedded in paraffin overnight at 65℃ [78]. The embedded sample block was mounted on rotary microtome (KD-2258, Kedee, China) and sliced to a thickness of 10 µm [79]. The sections were double dyed with 1% Safranin and 1% Fast Green, and sealed with neutral resin on slides. Clear and recognizable sections were selected and analyzed using an optical microscope (CX23, Olympus, Tokyo, Japan). Stages of anther development were classified as described in a previous study [3].

### RNA isolation and sequencing

Based on the microscopic observations, samples from three important anther development stages (CoA1: pollen mother cells stage; CoA2: tetrad stage; CoA3: uninucleate pollen stage) were collected. One biological replicate comprised of three anthers from a floral bud. Three such biological replicates were used for each stage, which formed the nine samples (CoA11, CoA12, CoA13; CoA21, CoA22, CoA23; CoA31, CoA32, CoA33) for RNA extraction and sequencing. For each stage, anthers were dissected from floral buds with the help of tweezers and collected in a centrifuge tube, which were frozen in liquid nitrogen and stored at -80℃. Total RNA was extracted from anthers using EASYspin Plus Complex Plant RNA Kit (Aidlab Biotech, Beijing, China) from nine samples. Total RNA concentration was detected by NanoDrop 2000 (Thermo Scientific, USA), and quality was tested on an Agilent 2100 Bioanalyzer (Agilent Technologies, USA). Only RNA samples with absorption values of 260/280 ratio between 1.8 to 2.1 and a 260/230 ratio ≥ 1.8, and with RNA integrity number (RIN) ≥ 8.8 were selected for follow-up experiments.

For Illumina sequencing, polyadenylated mRNA was enriched by oligo (dT) magnetic beads, and then fragmentation buffer was added to break the mRNA into short pieces. Random hexamers were used to reverse transcribe mRNA into single-stranded cDNA; dNTPs, DNA polymerase I and buffer were added to synthesize double-stranded cDNA. After using AMPure XP beads to purify double-stranded cDNA, they were subjected to end repair, the addition of the poly-A tail, ligation of the sequencing linker, and fragment size selection. Finally, 9 cDNA libraries were subjected to PCR enrichment and sequenced on an Illumina HiSeq 2500 platform (Illumina, San Diego, CA, USA).

### PacBio SMRT sequencing library preparation

To prepare the SMRTbell library, equal amounts of 9 total RNA samples were combined to generate an RNA pool for SMRT sequencing. Oligo-dTs were used to enrich for mRNAs containing poly-A tails in this pool, and SMARTer PCR cDNA synthesis kit (Clontech, USA) was used to synthesize cDNA with mRNA as template. PCR was used to amplify the cDNAs. cDNA was subjected to injury repair, end-repair, ligated to SMRT dumbbell-type linkers, unligated linker sequences at both ends of cDNA were removed, primers were added, and DNA polymerase was used to form a complete/full-length transcriptome library, which was sequenced using manufacturer’s insturctions. The subread sequences were obtained by processing the raw sequence data with the help of SMRTlink v8.0 software. Circular consensus sequences (CCSs) were obtained following the correction between subreads. Full-length sequences containing 5’ primers, 3’ primers, and poly-A tails were clustered using the Iterative Isomer Clustering (ICE) algorithm. Finally, the consensus sequences were polished to obtain high-quality sequences for subsequent analysis.

### Illumina and PacBio data analysis

Illumina data was mapped to the PacBio data by using LoRDEC software [80] to obtain clean reads. Redundant sequences were removed with the help of CD-HIT [81], and full-length transcripts were annotated by BLAST v2.2.26 [82], KOBAS v2.0 [83] and HMMER v3.1 [84] searches against public databases, including Swiss-Prot [85], Pfam [86], GO [87] and the Kyoto Encyclopedia of Genes and Genomes (KEGG) [88]. Full-length transcripts from this study were used as reference sequences (ref) for each of the genes, clean reads obtained by Illumina sequencing were aligned to refs, and read counts of all genes were obtained. Further, these read counts were converted into fragments per kilobase of transcript per million mapped reads (FPKM) values. Differentially expressed genes (DEGs) were determined across three developmental stages (CoA2 vs. CoA1, CoA2 vs. CoA3, and CoA3 vs. CoA1) on the basis of the criteria of |log_2_foldchange|≥ 1 (log_2_FC) and q-value ≤ 0.05. All DEGs were mapped to individual terms in GO database, and the number of genes per term was calculated. GO enrichment analysis was then performed using GOseq software to identify significantly enriched terms in the DEGs. The enriched GO terms were checked using an *p*-value < 0.05 as the cut off for significant GO terms. KOBAS software [89] was used to perform KEGG (http://www.genome.jp/kegg/) pathway enrichment analysis.

### LncRNA identification and classification from SMRT sequences

Five steps were adapted to identify lncRNAs from SMRT sequences of *C. oleifera*: (1) PLEK [90] was used with a default parameter of -minlength 200 to evaluate the coding potential of transcripts that lacked genome sequences and annotations. (2) Transcripts with length < 200 bp were removed. (3) Coding-non-coding index [91] was used at the default parameters to distinguish between coded and non-coded sequences; (4) coding potential calculator 2 [92] was used for the support vector machine classifier to evaluate the coding potential of a transcript based on the biological sequence characteristics of each ORF in transcripts; the e-value was set to “1e-10”. (5) Finally, transcripts were searched against Pfam database [86] to eliminate transcripts encoding proteins and protein coding domains (cutoff E-value = 0.001). At last, transcripts without coding potentials were selected as candidate lncRNAs.

LncRNA classification was carried out according to their genomic location [93]. First, lncRNAs were classified by aligning the SMRT sequences of *C. oleifera* to the genome of *C. oleifera* [94] with the help of Minimap2 [95]. In the second analysis, FEELnc (isBest = 1) was used for this purpose [96]. Finally, the results of these two classifications were combined and lncRNAs were classified into four categories: sense lncRNAs (generic overlap with known exon), long intergenic non-coding RNAs (lincRNAs) (contained intergenic lncRNAs), antisense lncRNAs (overlapped with a known gene on the opposite strand), and sense intronic lncRNAs (falls entirely within an intron of a known gene) [97–99].

### Sequencing and dentification of miRNAs

Total RNAs from nine samples were used for the preparation of small RNA libraries with the help of NEBNext multiplex small RNA library prep kit for Illumina (NEB, USA) following the manufacturer’s protocol. Library quality was assessed on an Agilent Bioanalyzer 2100 system after using Qubit2.0 for preliminary quantification; the library was diluted to 1 ng/µl. The libraries were sequenced (50-bp single-end reads) on an Illumina SE50 platform. The sequences, with lengths ranging between 18–30 bp, from the clean reads were mapped to the SMRT sequences by Bowtie [100]. The miRBase20.0 database was used as a reference, and srna-tools-cli (http://srna-workbench.cmp.uea.ac.uk/) was used to identify potential miRNAs and to draw their secondary structures. miREvo (set the parameter to “-i -r -M -m -k -p10 -g 50000”) [101] and miRDeep2 [102] were used to identify potential Dicer cleavage sites, explore secondary structures and determine minimum free energy of small RNA tags so that novel miRNAs could be predicted in the samples. miRNA candidates were obtained after removing tags originating from protein-coding genes, repeat sequences, rRNAs, tRNAs, snRNAs, and snoRNAs by mapping them against the Rfam database. For all candidate miRNAs, custom scripts were used to calculate miRNA counts and to estimate base bias at the first position of identified miRNAs. The miRNA families were identified by comparing the sequences to miRbase (http://www.mirbase.org/ftp.shtml) and Rfam databases (http://rfam.sanger.ac.uk/search/). TPM (transcript per million) value was used to estimate the expression level of miRNAs [103]. The DEGseq2 was used for the differential expression analysis with |log_2_FC|≥ 1 and q-value ≤ 0.05 as the threshold.

### Predicting the potential target genes and functional annotation of lncRNAs and miRNAs

It is known that lncRNAs may act in ‘trans’ [51]. In order to predict the genes trans-regulated by differentially expressed lncRNAs, BLAST was used to search the full-length transcriptome sequences of our libraries at an e-value cutoff of ‘1e-5’ and identity = 80%. Then, Pearson correlation (|correlation|≥ 0.8, *P*-value ≤ 0.05) was used to select potential targets on the basis of the expression correlation coefficients between lncRNAs and mRNAs.

The miRNA targets were predicted with the help of psRNATarget with expectation ≤ 3 (https://www.zhaolab.org/psRNATarget/) [104, 105] and the probable target mRNAs were selected on the basis of a negative Pearson’s correlation coefficient between miRNA and mRNA expression levels (correlation ≤  − 0.8, *P*-value ≤ 0.05). The miRNA targets in lncRNAs were predicted with the help of psRNATarget with expectation ≤ 5, and a negative Pearson’s correlation coefficient between miRNA and lncRNA expression levels ≤  − 0.67 (at *P*-value ≤ 0.05).

All lncRNA- and miRNA- target genes were subjected to GO and KEGG pathway enrichment analyses. Pearson’s correlation coefficients were calculated in R (version 4.1.1) to construct the interaction network between miRNAs, ncRNAs and mRNAs, and the network was visualized with the help of Cytoscape (version 3.8.2).

### Validation of miRNA, lncRNA and gene expression by qRT-PCR

Total RNA of all nine anther samples were reverse transcribed into cDNA using GoScript™ Reverse Transcription System (Promega, USA). Four and seven differentially expressed lncRNAs and mRNAs, which from three developmental stages of anther, were chosen for qRT-PCR verification. The specific primers for the lncRNAs and mRNAs were designed using Primer Premier v5 (Supplementary Table S13). For lncRNAs and mRNAs, qRT-PCR was performed using TB Green Premix Ex Taq II (Tli RNase H Plus; Takara, Beijing, China) in a final qRT-PCR reaction mixture containing 5.0 µL 2 × TB Green Premix Ex Taq, 0.8 μL primers, 2.0 µL cDNA, and 6.0 µL ddH2O. The alpha-tubulin (*TubA*) genes served as internal controls for normalization. In addition, eight miRNAs which related to anther development were selected for qRT-PCR verification. The miRNA RT/qPCR Detection kit (Aidlab, Beijing, China) was used to reverse transcribe total RNA into cDNA and qRT-PCR analysis for miRNAs, following the manufacturer’s recommendations. The two-tailed qRT-PCR method was used to design primers for miRNAs [106] (Supplementary Table S13). The 5S ribosomal RNA (*5S*) served as internal controls for normalization. The expression levels of the lncRNAs, genes and miRNAs were calculated using the 2^–△△Ct^ method against the internal controls [107]. For each biological replicate, three technical replicates were performed to ensure reproducibility and reliability.

## Supplementary Information

**Additional file 1: Supplementary Fig. S1. **Predicted total number of lncRNAs. **Supplementary Fig. S2.** First base composition of the known miRNA (18-30 nt length) in nine samples. (A). CoA11. (B). CoA12. (C). CoA13. (D). CoA21. (E). CoA22. (F). CoA23. (G). CoA31. (H). CoA32. (I). CoA33.** Supplementary Fig. S3.** First base composition of novel miRNA in nine samples. (A). CoA11. (B). CoA12. (C). CoA13. (D). CoA21. (E). CoA22. (F). CoA23. (G). CoA31. (H). CoA32. (I). CoA33. **Supplementary Fig. S4.** GO analysis of the biological functions of target genes of lncRNAs and miRNAs. GO terms of 25 target genes of 14 differently accumulated miRNAs (A) and 11 target genes of 7 differentially accumulated lncRNAs, which were targeted by 8 differentially accumulated miRNAs (B).** Supplementary Fig. S5. **KEGG pathway enrichment analysis of target genes of lncRNAs and miRNAs. KEGG pathway enrichment analysis of 25 target genes of 14 differently accumulated miRNAs (A) and 11 target genes of 7 differentially accumulated lncRNAs which were targeted by 8 differentially accumulated miRNAs (B).

**Additional file 2: Supplementary Table S1.** LncRNAs identified in the tropical *C. oleifera.* **Supplementary Table S2.** MiRNAs identified in the tropical *C. oleifera.* **Supplementary Table S3.** The significantly differentially expressed mRNAs. **Supplementary Table S4.** Significantly enriched GO categories of differentially accumulating mRNAs. **Supplementary Table S5.** List of differentially expressed genes crucial for the tropical *C. oleifera* anther development. **Supplementary Table S6.** Potential trans-regulated target genes of differentially accumulated lncRNAs. **Supplementary Table S7.** Significant GO categories of differentially accumulating lncRNA-target genes. **Supplementary Table S8.** Significant KEGG categories of differentially accumulating lncRNA-target genes. **Supplementary Table S9.** Target genes of differentially expressed lncRNAs involved in floral bud development. **Supplementary Table S10.** Analysis of target genes for differentially accumulated miRNAs. **Supplementary Table S11.** The differently accumulated lncRNAs predicted as targets of differentially accumulated miRNAs. **Supplementary Table S12.** Differentially expressed genes and lncRNAs in miRNA-lncRNA-mRNA network. **Supplementary Table S13.** Oligonucleotide primers used in qRT-PCR assays in this study.

## Data Availability

The raw sequence data reported in this paper have been deposited in the Genome Sequence Archive [108] in National Genomics Data Center [109], China National Center for Bioinformation / Beijing Institute of Genomics, Chinese Academy of Sciences (GSA: CRA006274, CRA006275 and CRA006279) that are publicly accessible at https://ngdc.cncb.ac.cn/gsa. The datasets analysed during this study are included in this published article and its supplementary information files.

## Abbreviations

lncRNAs: Long non-coding RNAs
miRNAs: MicroRNAs
SMRT: Single-molecule real-time
RNA-seq: RNA sequencing
GO: Gene ontology
RIN: RNA integrity number
CCS: Circular consensus sequence
ICE: Iterative Isomer Clustering
ref: Reference sequences
KEGG: Kyoto Encyclopedia of Genes and Genomes
FPKM: Fragments per kilobase of transcript per million mapped reads
DEGs: Differentially expressed genes
FC: Fold change
lincRNAs: Long intergenic non-coding RNAs
TPM: Transcript per million
FLNC: Full-length nonchimeric
qRT-PCR: Quantitative Reverse-Transcription Polymerase Chain Reaction
vs.: Versus
