# Supplementary material for: Identification of long non-coding RNAs and microRNAs involved in anther development in the tropical Camellia oleifera
Source: BMC Genomics. 2022 Aug 16;23:596. doi: 10.1186/s12864-022-08836-7 (PMC9380326; doi:10.1186/s12864-022-08836-7)
Supplement: Supplementary file 1 — Additional file 1: Supplementary Fig. S1. Predicted total number of lncRNAs. Supplementary Fig. S2. First base composition of the known miRNA (18-30 nt length) in nine samples. (A). CoA11. (B). CoA12. (C). CoA13. (D). CoA21. (E). CoA22. (F). CoA23. (G). CoA31. (H). CoA32. (I). CoA33. Supplementary Fig. S3. First base composition of novel miRNA in nine samples. (A). CoA11. (B). CoA12. (C). CoA13. (D). CoA21. (E). CoA22. (F). CoA23. (G). CoA31. (H). CoA32. (I). CoA33. Supplementary Fig. S4. GO analysis of the biological functions of target genes of lncRNAs and miRNAs. GO terms of 25 target genes of 14 differently accumulated miRNAs (A) and 11 target genes of 7 differentially accumulated lncRNAs, which were targeted by 8 differentially accumulated miRNAs (B). Supplementary Fig. S5. KEGG pathway enrichment analysis of target genes of lncRNAs and miRNAs. KEGG pathway enrichment analysis of 25 target genes of 14 differently accumulated miRNAs (A) and 11 target genes of 7 differentially accumulated lncRNAs which were targeted by 8 differentially accumulated miRNAs (B). [file 12864_2022_8836_MOESM1_ESM.pdf]

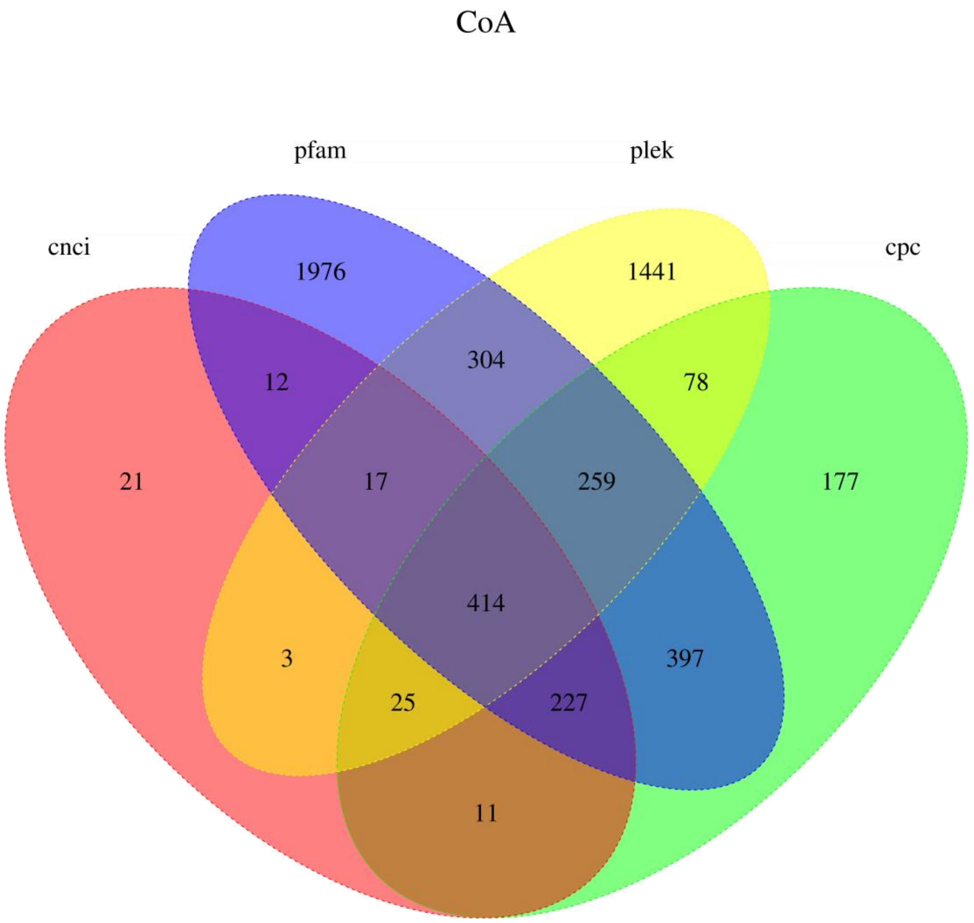

Figure S1. Predicted total number of lncRNAs.

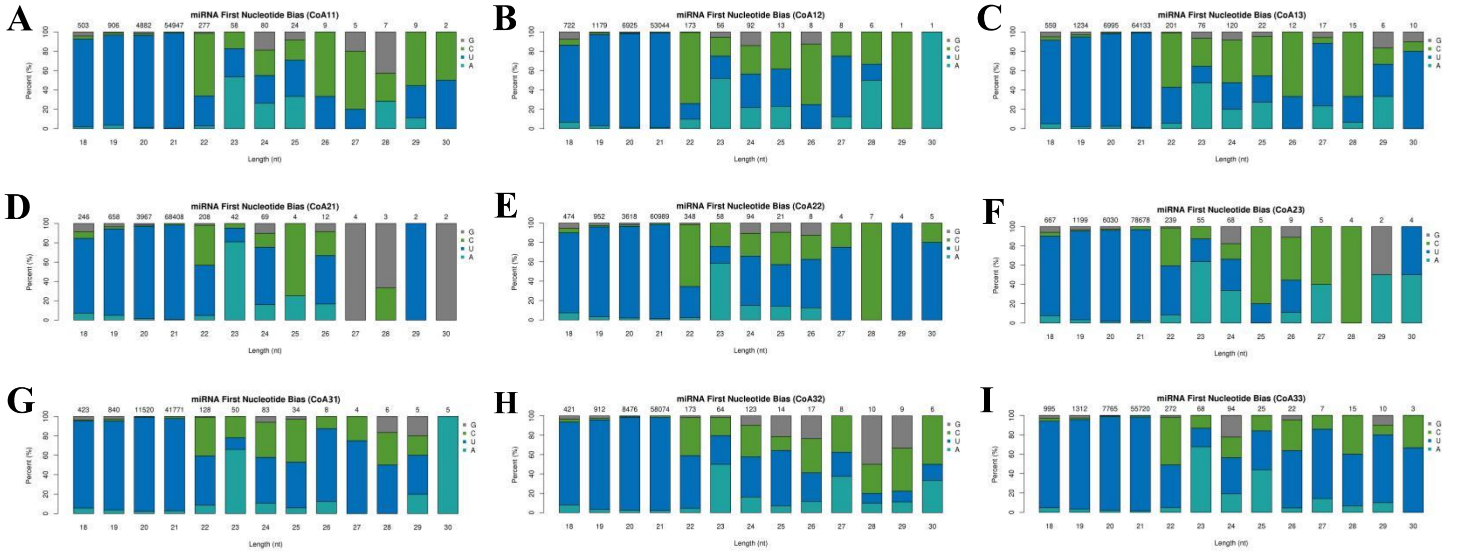

Figure S2. First base composition of the known miRNA (18-30 nt length) in nine samples. (**A**). CoA11. (**B**). CoA12. (**C**). CoA13. (**D**). CoA21. (**E**). CoA22. (**F**). CoA23. (**G**). CoA31. (**H**). CoA32. (**I**). CoA33.

.

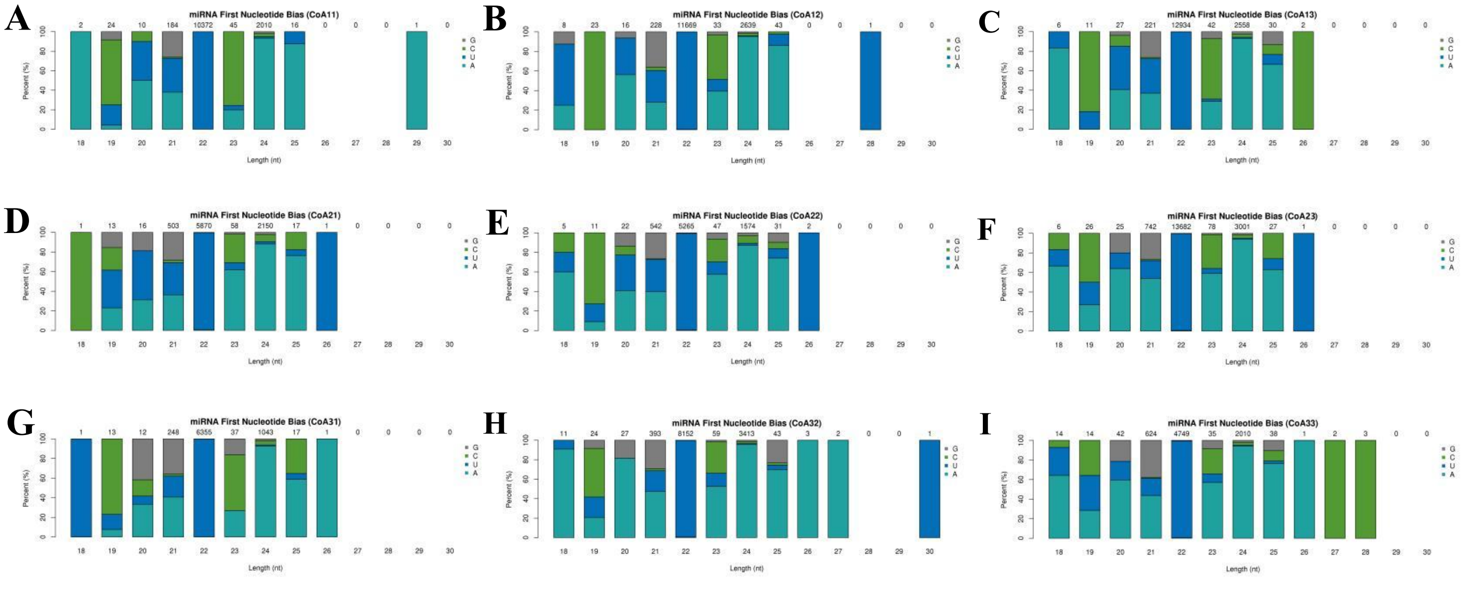

Figure S3. First base composition of novel miRNA in nine samples. (**A**). CoA11. (**B**). CoA12. (**C**). CoA13. (**D**). CoA21. (**E**). CoA22. (**F**). CoA23. (**G**). CoA31. (**H**). CoA32. (**I**). CoA33.

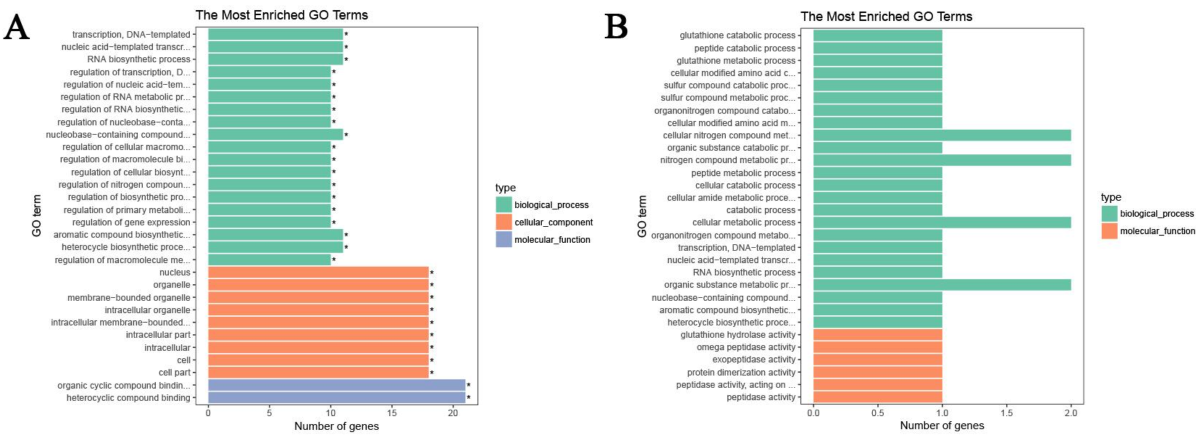

Figure S4. GO analysis of the biological functions of target genes of lncRNAs and miRNAs. GO terms of 25 target genes of 14 differently accumulated miRNAs (**A**) and 11 target genes of 7 differentially accumulated lncRNAs, which were targeted by 8 differentially accumulated miRNAs (**B**).

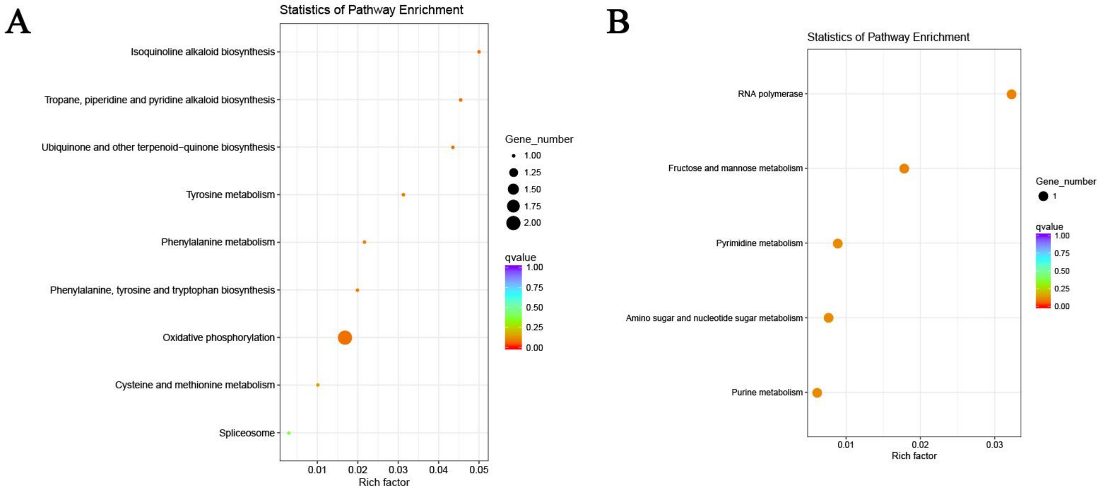

Figure S5. KEGG pathway enrichment analysis of target genes of lncRNAs and miRNAs. KEGG pathway enrichment analysis of 25 target genes of 14 differently accumulated miRNAs (**A**) and 11 target genes of 7 differentially accumulated lncRNAs which were targeted by 8 differentially accumulated miRNAs (**B**).
