# Supplementary material for: Identification of long non-coding RNAs and microRNAs involved in anther development in the tropical Camellia oleifera
Source: BMC Genomics. 2022 Aug 16;23:596. doi: 10.1186/s12864-022-08836-7 (PMC9380326; doi:10.1186/s12864-022-08836-7)
Supplement: Supplementary file 2 — Additional file 2: Supplementary Table S1. LncRNAs identified in the tropical C. oleifera. Supplementary Table S2. MiRNAs identified in the tropical C. oleifera. Supplementary Table S3. The significantly differentially expressed mRNAs. Supplementary Table S4. Significantly enriched GO categories of differentially accumulating mRNAs. Supplementary Table S5. List of differentially expressed genes crucial for the tropical C. oleifera anther development. Supplementary Table S6. Potential trans-regulated target genes of differentially accumulated lncRNAs. Supplementary Table S7. Significant GO categories of differentially accumulating lncRNA-target genes. Supplementary Table S8. Significant KEGG categories of differentially accumulating lncRNA-target genes. Supplementary Table S9. Target genes of differentially expressed lncRNAs involved in floral bud development. Supplementary Table S10. Analysis of target genes for differentially accumulated miRNAs. Supplementary Table S11. The differently accumulated lncRNAs predicted as targets of differentially accumulated miRNAs. Supplementary Table S12. Differentially expressed genes and lncRNAs in miRNA-lncRNA-mRNA network. Supplementary Table S13. Oligonucleotide primers used in qRT-PCR assays in this study. [file 12864_2022_8836_MOESM2_ESM.pdf]

Supplementary Table S1. LncRNAs identified in the tropical *Camellia oleifera*

Transcript ID	LncRNA ID	LncRNA length (bp)	Class_lncRNA	Expression （FPKM value）		
				CoA1	CoA2	CoA3
transcript_HQ_CoA_transcript14538/f2p0/2655	CoA14538_2655	2654	lincRNAs	3.096666667	1.226666667	1.51
transcript_HQ_CoA_transcript11831/f2p0/2913	CoA11831_2913	2913		0.07	1.16	0.706666667
transcript_HQ_CoA_transcript10938/f2p0/3014	CoA10938_3014	3014		5.466666667	5.503333333	7.1
transcript_HQ_CoA_transcript11848/f2p0/2922	CoA11848_2922	2922		4.37	1.13	1.756666667
transcript_HQ_CoA_transcript12772/f2p0/2803	CoA12772_2803	2815	lincRNAs	0.303333333	0.343333333	0.253333333
transcript_HQ_CoA_transcript11879/f2p0/2942	CoA11879_2942	2942		8.96	6.866666667	3.436666667
transcript_HQ_CoA_transcript10073/f2p0/3107	CoA10073_3107	3108		3.453333333	4.603333333	2.43
transcript_HQ_CoA_transcript10095/f3p0/3099	CoA10095_3099	3099		0.533333333	0.37	0.64
transcript_HQ_CoA_transcript11906/f4p0/2889	CoA11906_2889	2889		49.50666667	9.483333333	12.71666667
transcript_HQ_CoA_transcript12817/f2p0/2807	CoA12817_2807	2807	sense lncRNAs	14.92	7.013333333	57.63666667
transcript_HQ_CoA_transcript11006/f4p0/2994	CoA11006_2994	2994	lincRNAs	0.4	0.32	0.51
transcript_HQ_CoA_transcript10098/f2p0/3078	CoA10098_3078	3081		0.086666667	0.31	0.24
transcript_HQ_CoA_transcript11930/f2p0/2897	CoA11930_2897	2897		3.246666667	1.93	1.946666667
transcript_HQ_CoA_transcript13761/f2p0/2722	CoA13761_2722	2719	sense lncRNAs	0.456666667	0.323333333	0.266666667
transcript_HQ_CoA_transcript11954/f3p0/2888	CoA11954_2888	2888		42.43333333	2.463333333	7.88
transcript_HQ_CoA_transcript13763/f2p0/2719	CoA13763_2719	2720		8.79	12.01666667	7.566666667
transcript_HQ_CoA_transcript11055/f2p0/2489	CoA11055_2489	2495		1.703333333	0.813333333	0.733333333
transcript_HQ_CoA_transcript12874/f2p0/2812	CoA12874_2812	2815		5.696666667	1.556666667	1.34
transcript_HQ_CoA_transcript1106/f2p0/5637	CoA1106_5637	5637		5.203333333	2.266666667	3.623333333
transcript_HQ_CoA_transcript14683/f2p0/2634	CoA14683_2634	2634		15.63666667	4.04	6.066666667
transcript_HQ_CoA_transcript11969/f2p0/2826	CoA11969_2826	2821		15.07666667	7.186666667	4.61
transcript_HQ_CoA_transcript12891/f2p0/2838	CoA12891_2838	2838	sense lncRNAs	0.923333333	0.88	0.97
transcript_HQ_CoA_transcript13798/f2p0/2724	CoA13798_2724	2715		2.323333333	2.186666667	2.776666667
transcript_HQ_CoA_transcript11088/f2p0/3005	CoA11088_3005	3002	lincRNAs	0.066666667	0.13	0.256666667
transcript_HQ_CoA_transcript10198/f2p0/3111	CoA10198_3111	3108	lincRNAs	1.3	0.486666667	2.016666667
transcript_HQ_CoA_transcript12016/f2p0/2898	CoA12016_2898	2897		5.98	5.596666667	6.166666667
transcript_HQ_CoA_transcript14794/f2p0/2626	CoA14794_2626	2628	lincRNAs	1.343333333	0.956666667	0.606666667
transcript_HQ_CoA_transcript13009/f2p0/2807	CoA13009_2807	2808	lincRNAs	28.05666667	11.67666667	10.45
transcript_HQ_CoA_transcript11227/f5p0/2963	CoA11227_2963	2965	sense lncRNAs	2.166666667	3.076666667	2.443333333
transcript_HQ_CoA_transcript1214/f3p0/5522	CoA1214_5522	5521		11.79333333	7.21	10.15333333
transcript_HQ_CoA_transcript12184/f2p0/2866	CoA12184_2866	2866		22.52333333	17.91666667	8.066666667
transcript_HQ_CoA_transcript12197/f3p0/2873	CoA12197_2873	2871		3.936666667	1.32	5.453333333
transcript_HQ_CoA_transcript13996/f2p0/2715	CoA13996_2715	2717		1.316666667	0.95	1.476666667
transcript_HQ_CoA_transcript13999/f2p0/2689	CoA13999_2689	2689	lincRNAs	1.753333333	0.95	1.19
transcript_HQ_CoA_transcript13131/f2p0/2776	CoA13131_2776	2774		4.046666667	4.95	5.93
transcript_HQ_CoA_transcript11312/f2p0/2974	CoA11312_2974	2967		0.94	0.433333333	0.796666667

transcript_HQ_CoA_transcript11316/f2p0/3088	CoA11316_3088	3089		1.806666667	1.953333333	1.246666667
transcript_HQ_CoA_transcript14933/f2p0/2611	CoA14933_2611	2611		7.18	3.45	2.11
transcript_HQ_CoA_transcript10392/f2p0/3073	CoA10392_3073	3071	lincRNAs	1.516666667	1.61	1.323333333
transcript_HQ_CoA_transcript11324/f2p0/2989	CoA11324_2989	2992		0	0.35	0.92
transcript_HQ_CoA_transcript14035/f3p0/2725	CoA14035_2725	2726		6.636666667	5.186666667	6.356666667
transcript_HQ_CoA_transcript1227/f4p0/5444	CoA1227_5444	5445		12.52666667	4.873333333	3.5
transcript_HQ_CoA_transcript14994/f2p0/2619	CoA14994_2619	2620		10.94333333	4.523333333	1.423333333
transcript_HQ_CoA_transcript12307/f2p0/2907	CoA12307_2907	2906	lincRNAs	20.56666667	17.01333333	19.03
transcript_HQ_CoA_transcript10432/f2p0/3080	CoA10432_3080	3066	lincRNAs	1.553333333	3.37	2.266666667
transcript_HQ_CoA_transcript13257/f6p0/2765	CoA13257_2765	2765		52.18	59.05666667	41.75666667
transcript_HQ_CoA_transcript13292/f2p0/2761	CoA13292_2761	2761		0.423333333	1.126666667	1.253333333
transcript_HQ_CoA_transcript13314/f2p0/2762	CoA13314_2762	2763		5.836666667	5.366666667	4.16
transcript_HQ_CoA_transcript11465/f2p0/2974	CoA11465_2974	2976		5.726666667	3.8	1.886666667
transcript_HQ_CoA_transcript11478/f2p0/2967	CoA11478_2967	2968		0.88	0.57	4.72
transcript_HQ_CoA_transcript14161/f3p0/2687	CoA14161_2687	2687		20.15666667	10.65666667	9.746666667
transcript_HQ_CoA_transcript14211/f2p0/2677	CoA14211_2677	2676		2.226666667	2.393333333	1.953333333
transcript_HQ_CoA_transcript10554/f2p0/3057	CoA10554_3057	3056		0.983333333	0.393333333	0.45
transcript_HQ_CoA_transcript10566/f2p0/3056	CoA10566_3056	3056		0.456666667	0.38	0.573333333
transcript_HQ_CoA_transcript10567/f2p0/3040	CoA10567_3040	3040		19.22	2.963333333	11.95666667
transcript_HQ_CoA_transcript14251/f2p0/2673	CoA14251_2673	2673		2.783333333	2.05	2.083333333
transcript_HQ_CoA_transcript15206/f3p0/2586	CoA15206_2586	2586	lincRNAs	20.04	5.93	8.023333333
transcript_HQ_CoA_transcript11599/f15p0/2875	CoA11599_2875	2875		20.85	1.553333333	7.173333333
transcript_HQ_CoA_transcript14288/f2p0/2671	CoA14288_2671	2675	sense lncRNAs	3.643333333	0.303333333	2.493333333
transcript_HQ_CoA_transcript11614/f2p0/2938	CoA11614_2938	2939		5.65	0.53	3.603333333
transcript_HQ_CoA_transcript13474/f2p0/2746	CoA13474_2746	2750		0.276666667	0.186666667	0.133333333
transcript_HQ_CoA_transcript13480/f3p0/2771	CoA13480_2771	2772		2.033333333	1.696666667	1.536666667
transcript_HQ_CoA_transcript14341/f4p0/2639	CoA14341_2639	2640		4.273333333	4.96	5.706666667
transcript_HQ_CoA_transcript14354/f2p0/2670	CoA14354_2670	2672		15.14	2.486666667	3.5
transcript_HQ_CoA_transcript13507/f2p0/2715	CoA13507_2715	2725	lincRNAs	0.896666667	0.583333333	0.866666667
transcript_HQ_CoA_transcript15286/f3p0/2581	CoA15286_2581	2583	sense lncRNAs	1.893333333	1.7	1.516666667
transcript_HQ_CoA_transcript13547/f2p0/2734	CoA13547_2734	2736		1.68	0.97	1.023333333
transcript_HQ_CoA_transcript13552/f3p0/2735	CoA13552_2735	2749		0.636666667	0.55	0.223333333
transcript_HQ_CoA_transcript15334/f4p0/2562	CoA15334_2562	2562		17.1	12.78666667	20.67333333
transcript_HQ_CoA_transcript10727/f2p0/3016	CoA10727_3016	3017		12.30666667	3.346666667	1.026666667
transcript_HQ_CoA_transcript11759/f6p0/2902	CoA11759_2902	2902		7.266666667	0.906666667	3.453333333
transcript_HQ_CoA_transcript14458/f2p0/2663	CoA14458_2663	2663		1.98	1.396666667	1.43
transcript_HQ_CoA_transcript15442/f2p0/2595	CoA15442_2595	2597	sense lncRNAs	0.2	0.206666667	0.293333333
transcript_HQ_CoA_transcript15444/f3p0/2619	CoA15444_2619	2619		1.04	0.903333333	0.976666667
transcript_HQ_CoA_transcript10797/f2p0/3032	CoA10797_3032	3032	lincRNAs	17.09333333	3.45	11.07
transcript_HQ_CoA_transcript19020/f2p0/2278	CoA19020_2278	2278		14.66	14.05	16.44333333

transcript_HQ_CoA_transcript18138/f2p0/2358	CoA18138_2358	2357		2.636666667	5.303333333	6.376666667
transcript_HQ_CoA_transcript19027/f2p0/2275	CoA19027_2275	2262		0.126666667	0.123333333	0.253333333
transcript_HQ_CoA_transcript10823/f4p0/3027	CoA10823_3027	3030	sense lncRNAs	0.503333333	0.38	0.313333333
transcript_HQ_CoA_transcript15487/f2p0/2617	CoA15487_2617	2624		0.796666667	0.413333333	0.323333333
transcript_HQ_CoA_transcript17271/f2p0/2457	CoA17271_2457	2458		34.50666667	12.66666667	18.50666667
transcript_HQ_CoA_transcript17297/f2p0/2426	CoA17297_2426	2424		0.16	0.07	0.57
transcript_HQ_CoA_transcript10870/f2p0/3047	CoA10870_3047	3048		40.76666667	30.67666667	18.40666667
transcript_HQ_CoA_transcript19128/f2p0/2283	CoA19128_2283	2284		5.186666667	2.283333333	2.746666667
transcript_HQ_CoA_transcript19923/f2p0/2227	CoA19923_2227	2222	sense lncRNAs	0.176666667	0.203333333	0.06
transcript_HQ_CoA_transcript18250/f4p0/2323	CoA18250_2323	2328		1.37	0.83	0.713333333
transcript_HQ_CoA_transcript18265/f4p0/2327	CoA18265_2327	2328	lincRNAs	1.05	6.856666667	2.276666667
transcript_HQ_CoA_transcript17383/f2p0/2409	CoA17383_2409	2405	lincRNAs	2.683333333	1.08	1.113333333
transcript_HQ_CoA_transcript17384/f2p0/2415	CoA17384_2415	2417		2.18	0.956666667	0.923333333
transcript_HQ_CoA_transcript19992/f2p0/2210	CoA19992_2210	2205		1.163333333	1.043333333	1.643333333
transcript_HQ_CoA_transcript15608/f5p0/2531	CoA15608_2531	2532	lincRNAs	2.993333333	0.973333333	2.143333333
transcript_HQ_CoA_transcript16493/f2p0/2480	CoA16493_2480	2481		2.37	0.316666667	0.593333333
transcript_HQ_CoA_transcript16496/f2p0/2488	CoA16496_2488	2491		1.456666667	1.27	1.276666667
transcript_HQ_CoA_transcript19996/f2p0/2201	CoA19996_2201	2203		5.646666667	3.433333333	5.376666667
transcript_HQ_CoA_transcript18346/f5p0/2326	CoA18346_2326	2326		2.653333333	1.05	0.916666667
transcript_HQ_CoA_transcript17464/f2p0/2399	CoA17464_2399	2401		0.333333333	1.093333333	0.58
transcript_HQ_CoA_transcript18375/f2p0/2329	CoA18375_2329	2328		5.26	7.903333333	4.123333333
transcript_HQ_CoA_transcript18383/f3p0/2307	CoA18383_2307	2309		1.503333333	1.553333333	1.24
transcript_HQ_CoA_transcript20064/f2p0/2261	CoA20064_2261	2261		0.17	0.176666667	0.133333333
transcript_HQ_CoA_transcript20069/f2p0/2196	CoA20069_2196	2196		1.203333333	1.943333333	0.593333333
transcript_HQ_CoA_transcript17508/f2p0/2398	CoA17508_2398	2399		13.87666667	12.49333333	9.393333333
transcript_HQ_CoA_transcript15702/f2p0/2564	CoA15702_2564	2568		0.24	1.66	0.49
transcript_HQ_CoA_transcript16613/f2p0/2473	CoA16613_2473	2474		0.683333333	0.266666667	0.616666667
transcript_HQ_CoA_transcript20132/f3p0/2197	CoA20132_2197	2197		1.536666667	1.156666667	1.32
transcript_HQ_CoA_transcript15757/f2p0/2544	CoA15757_2544	2552		11.84666667	4.063333333	6.33
transcript_HQ_CoA_transcript18493/f7p0/2296	CoA18493_2296	2296		10.02	4.816666667	7.293333333
transcript_HQ_CoA_transcript18507/f4p0/2308	CoA18507_2308	2306		0.733333333	0.803333333	0.75
transcript_HQ_CoA_transcript19426/f2p0/2259	CoA19426_2259	2253	sense lncRNAs	0.87	0.636666667	0.81
transcript_HQ_CoA_transcript15806/f2p0/2531	CoA15806_2531	2531	lincRNAs	1.443333333	1.033333333	0.856666667
transcript_HQ_CoA_transcript16688/f2p0/2450	CoA16688_2450	2450		0.313333333	0.316666667	0.053333333
transcript_HQ_CoA_transcript18549/f2p0/2304	CoA18549_2304	2306	lincRNAs	10.23333333	6.22	7.91
transcript_HQ_CoA_transcript18552/f5p0/2315	CoA18552_2315	2318		10.42666667	8.366666667	8.236666667
transcript_HQ_CoA_transcript18553/f2p0/2318	CoA18553_2318	2319		0.893333333	0.526666667	0.07
transcript_HQ_CoA_transcript20253/f2p0/2169	CoA20253_2169	2170		0.146666667	0.176666667	0.163333333
transcript_HQ_CoA_transcript15867/f2p0/2528	CoA15867_2528	2528		0.623333333	0.95	1.586666667
transcript_HQ_CoA_transcript18611/f2p0/2339	CoA18611_2339	2317		1.706666667	0.7	0.6

transcript_HQ_CoA_transcript16791/f3p0/2458	CoA16791_2458	2457	lincRNAs	0.363333333	0.156666667	0.143333333
transcript_HQ_CoA_transcript19556/f2p0/2256	CoA19556_2256	2257		16.29333333	13.31666667	9.95
transcript_HQ_CoA_transcript17760/f4p0/2350	CoA17760_2350	2352		2.433333333	3.276666667	4.16
transcript_HQ_CoA_transcript18663/f2p0/2328	CoA18663_2328	2328	lincRNAs	12.71	4.703333333	5.603333333
transcript_HQ_CoA_transcript19604/f2p0/2235	CoA19604_2235	2235		17.17333333	11.34666667	17.70666667
transcript_HQ_CoA_transcript17816/f2p0/2376	CoA17816_2376	2378		0.116666667	0.133333333	0.176666667
transcript_HQ_CoA_transcript19633/f3p0/2229	CoA19633_2229	2229		4.93	5.006666667	2.03
transcript_HQ_CoA_transcript18760/f2p0/2295	CoA18760_2295	2295		1.343333333	0.736666667	0.846666667
transcript_HQ_CoA_transcript20435/f2p0/2171	CoA20435_2171	2171		24.95666667	13.43666667	12.68666667
transcript_HQ_CoA_transcript16061/f2p0/2516	CoA16061_2516	2516		1.77	0.803333333	0.533333333
transcript_HQ_CoA_transcript16961/f2p0/2435	CoA16961_2435	2438		0.183333333	0.243333333	0.34
transcript_HQ_CoA_transcript20469/f2p0/2171	CoA20469_2171	2172		1.546666667	1.54	2.126666667
transcript_HQ_CoA_transcript17904/f3p0/2347	CoA17904_2347	2347		9.376666667	7.11	4.506666667
transcript_HQ_CoA_transcript19729/f2p0/2224	CoA19729_2224	2221		1.656666667	2.716666667	1.366666667
transcript_HQ_CoA_transcript16998/f2p0/2389	CoA16998_2389	2392		17.1	7.096666667	10.34
transcript_HQ_CoA_transcript19759/f2p0/2226	CoA19759_2226	2228	lincRNAs	18.98333333	9.026666667	9.193333333
transcript_HQ_CoA_transcript17945/f2p0/2372	CoA17945_2372	2366		0.03	0.026666667	0.083333333
transcript_HQ_CoA_transcript19762/f3p0/2235	CoA19762_2235	2236		7.07	9.343333333	7.11
transcript_HQ_CoA_transcript20528/f2p0/2179	CoA20528_2179	2181		13.95666667	10.55333333	13.13666667
transcript_HQ_CoA_transcript18860/f2p0/2295	CoA18860_2295	2296		4.74	2.793333333	2.776666667
transcript_HQ_CoA_transcript20533/f3p0/2169	CoA20533_2169	2170	sense lncRNAs	5.936666667	2.55	5.186666667
transcript_HQ_CoA_transcript20539/f2p0/2168	CoA20539_2168	2178	sense lncRNAs	0.23	0.24	0.06
transcript_HQ_CoA_transcript20553/f5p0/2118	CoA20553_2118	2118		0.096666667	0.086666667	0.03
transcript_HQ_CoA_transcript18880/f2p0/2291	CoA18880_2291	2295	lincRNAs	0.306666667	0.713333333	0.386666667
transcript_HQ_CoA_transcript17976/f3p0/2312	CoA17976_2312	2312		0.106666667	4.306666667	0.013333333
transcript_HQ_CoA_transcript18893/f3p0/2291	CoA18893_2291	2294		1.42	0.81	1.186666667
transcript_HQ_CoA_transcript18895/f2p0/2271	CoA18895_2271	2272		1.576666667	0.743333333	1.94
transcript_HQ_CoA_transcript17073/f2p0/2398	CoA17073_2398	2405		1.636666667	1.263333333	0.896666667
transcript_HQ_CoA_transcript18916/f2p0/2286	CoA18916_2286	2289		0.443333333	0.453333333	0.43
transcript_HQ_CoA_transcript19853/f2p0/2218	CoA19853_2218	2225	lincRNAs	0.4	0.356666667	0.513333333
transcript_HQ_CoA_transcript16203/f2p0/2501	CoA16203_2501	2498		0.22	0.103333333	0.136666667
transcript_HQ_CoA_transcript18954/f2p0/2279	CoA18954_2279	2284		5.8	1.573333333	6.926666667
transcript_HQ_CoA_transcript20631/f3p0/2158	CoA20631_2158	2158		1.096666667	0.503333333	0.36
transcript_HQ_CoA_transcript18973/f2p0/2332	CoA18973_2332	2333	lincRNAs	0.8	1.003333333	1.066666667
transcript_HQ_CoA_transcript17113/f5p0/2425	CoA17113_2425	2424		0.1	0.026666667	0.15
transcript_HQ_CoA_transcript20685/f2p0/2168	CoA20685_2168	2182	lincRNAs	1.926666667	1.563333333	0.48
transcript_HQ_CoA_transcript20708/f2p0/2154	CoA20708_2154	2154		5.76	8.783333333	4.36
transcript_HQ_CoA_transcript21778/f2p0/2077	CoA21778_2077	2079		2.53	1.946666667	1.74
transcript_HQ_CoA_transcript24434/f2p0/1905	CoA24434_1905	1907		4.056666667	3.46	3.673333333
transcript_HQ_CoA_transcript20758/f2p0/2129	CoA20758_2129	2124	antisense lncRNAs	0.47	0.476666667	0.616666667

transcript_HQ_CoA_transcript23586/f2p0/1951	CoA23586_1951	1951		0.316666667	0.393333333	0.446666667
transcript_HQ_CoA_transcript22724/f2p0/2008	CoA22724_2008	2008		7.776666667	1.69	5.76
transcript_HQ_CoA_transcript20948/f2p0/2138	CoA20948_2138	2138	lincRNAs	11.13	3.136666667	4.63
transcript_HQ_CoA_transcript22770/f2p0/2008	CoA22770_2008	2008		5.41	2.9	6.016666667
transcript_HQ_CoA_transcript21855/f2p0/2077	CoA21855_2077	2077		5.856666667	4.853333333	5.246666667
transcript_HQ_CoA_transcript20820/f3p0/2148	CoA20820_2148	2146		1.296666667	0.843333333	0.913333333
transcript_HQ_CoA_transcript2452/f7p0/4646	CoA2452_4646	4646		1.683333333	1.003333333	1.15
transcript_HQ_CoA_transcript23672/f3p0/1944	CoA23672_1944	1943		2.603333333	1.71	1.683333333
transcript_HQ_CoA_transcript24545/f3p0/1873	CoA24545_1873	1873		64.15	22.67666667	23.85
transcript_HQ_CoA_transcript23689/f2p0/1928	CoA23689_1928	1931		1.426666667	0.98	1.26
transcript_HQ_CoA_transcript23694/f2p0/1933	CoA23694_1933	1935		0.51	1.37	1.333333333
transcript_HQ_CoA_transcript21021/f2p0/2108	CoA21021_2108	2114		4.333333333	1.663333333	1.52
transcript_HQ_CoA_transcript2457/f2p0/4687	CoA2457_4687	4686		0.873333333	0.226666667	0.356666667
transcript_HQ_CoA_transcript22854/f2p0/1992	CoA22854_1992	1989		3.056666667	1.4	2.233333333
transcript_HQ_CoA_transcript24576/f2p0/1864	CoA24576_1864	1867	lincRNAs	0.27	0.423333333	0.163333333
transcript_HQ_CoA_transcript25401/f2p0/1809	CoA25401_1809	1809		1.156666667	0.87	1.146666667
transcript_HQ_CoA_transcript23751/f2p0/1930	CoA23751_1930	1930		5.653333333	4.49	3.87
transcript_HQ_CoA_transcript21093/f2p0/2129	CoA21093_2129	2129		6.036666667	5.703333333	4.796666667
transcript_HQ_CoA_transcript21099/f2p0/2126	CoA21099_2126	2136		4.353333333	2.243333333	3.063333333
transcript_HQ_CoA_transcript21113/f2p0/2115	CoA21113_2115	2114		2.913333333	1.88	2.086666667
transcript_HQ_CoA_transcript23795/f2p0/1957	CoA23795_1957	1957	lincRNAs	0.41	0.44	0.3
transcript_HQ_CoA_transcript22019/f3p0/1997	CoA22019_1997	1992		2.113333333	1.753333333	2.173333333
transcript_HQ_CoA_transcript22020/f2p0/2069	CoA22020_2069	2069		118.4933333	96.72666667	83.54
transcript_HQ_CoA_transcript23805/f2p0/1929	CoA23805_1929	1929	lincRNAs	63.42	12.37333333	6.393333333
transcript_HQ_CoA_transcript21128/f3p0/2112	CoA21128_2112	2114	lincRNAs	2.273333333	1.74	1.556666667
transcript_HQ_CoA_transcript21129/f2p0/2127	CoA21129_2127	2125		1.506666667	0.856666667	1.086666667
transcript_HQ_CoA_transcript21136/f2p0/2143	CoA21136_2143	2142		0	0.286666667	0.226666667
transcript_HQ_CoA_transcript25500/f2p0/1830	CoA25500_1830	1830	lincRNAs	3.463333333	1.36	4.663333333
transcript_HQ_CoA_transcript21148/f2p0/2123	CoA21148_2123	2127	sense lncRNAs	2.47	2.55	1.816666667
transcript_HQ_CoA_transcript25518/f3p0/1798	CoA25518_1798	1798		8.953333333	3.963333333	4.12
transcript_HQ_CoA_transcript23838/f3p0/1928	CoA23838_1928	1927		0.65	0.796666667	1.14
transcript_HQ_CoA_transcript24688/f3p0/1859	CoA24688_1859	1852		0.02	0.07	0.036666667
transcript_HQ_CoA_transcript25525/f2p0/1835	CoA25525_1835	1842	sense lncRNAs	0.1	0.08	0
transcript_HQ_CoA_transcript24692/f2p0/1857	CoA24692_1857	1857		0.193333333	0.42	0.403333333
transcript_HQ_CoA_transcript21181/f2p0/2087	CoA21181_2087	2088		0.173333333	0.116666667	0.153333333
transcript_HQ_CoA_transcript23882/f3p0/1942	CoA23882_1942	1944		3.933333333	0.766666667	0.61
transcript_HQ_CoA_transcript23894/f2p0/1915	CoA23894_1915	1914		2.79	2.396666667	2
transcript_HQ_CoA_transcript23900/f2p0/1921	CoA23900_1921	1923		3.99	1.77	1.74
transcript_HQ_CoA_transcript23910/f2p0/1931	CoA23910_1931	1933		2.32	1.69	1.986666667
transcript_HQ_CoA_transcript25593/f3p0/1781	CoA25593_1781	1781		0.973333333	1.39	1.046666667

transcript_HQ_CoA_transcript22136/f3p0/2043	CoA22136_2043	2042		0.946666667	0.983333333	0.443333333
transcript_HQ_CoA_transcript23936/f3p0/1928	CoA23936_1928	1926		0.49	0.356666667	0.103333333
transcript_HQ_CoA_transcript24784/f3p0/1844	CoA24784_1844	1845		0.25	0.266666667	0.466666667
transcript_HQ_CoA_transcript24787/f2p0/1850	CoA24787_1850	1848	sense lncRNAs	0.106666667	0.206666667	0.106666667
transcript_HQ_CoA_transcript22190/f3p0/2042	CoA22190_2042	2042		6.47	3.666666667	3.086666667
transcript_HQ_CoA_transcript24863/f2p0/1847	CoA24863_1847	1847		2.57	1.266666667	1.49
transcript_HQ_CoA_transcript25733/f3p0/1794	CoA25733_1794	1794		0.223333333	1.136666667	2.35
transcript_HQ_CoA_transcript22252/f2p0/2061	CoA22252_2061	2061	lincRNAs	0	0	4.156666667
transcript_HQ_CoA_transcript24042/f2p0/1915	CoA24042_1915	1917		0.076666667	0.106666667	0.23
transcript_HQ_CoA_transcript25747/f5p0/1777	CoA25747_1777	1777		2.183333333	3.38	2.756666667
transcript_HQ_CoA_transcript21323/f4p0/2071	CoA21323_2071	2068	lincRNAs	0.05	0.033333333	0.096666667
transcript_HQ_CoA_transcript22275/f2p0/2040	CoA22275_2040	2047		0.59	0.333333333	0.846666667
transcript_HQ_CoA_transcript24075/f2p0/1907	CoA24075_1907	1907		1.313333333	1.483333333	0.966666667
transcript_HQ_CoA_transcript24903/f2p0/1841	CoA24903_1841	1841		0.06	0.203333333	0.293333333
transcript_HQ_CoA_transcript25775/f2p0/1792	CoA25775_1792	1792		1.823333333	1.076666667	1.22
transcript_HQ_CoA_transcript21341/f3p0/2102	CoA21341_2102	2102		2.603333333	2.423333333	3.023333333
transcript_HQ_CoA_transcript24089/f2p0/1896	CoA24089_1896	1896		1.793333333	0.563333333	1.206666667
transcript_HQ_CoA_transcript22298/f3p0/2026	CoA22298_2026	2027	sense lncRNAs	1.33	1	1.483333333
transcript_HQ_CoA_transcript22301/f4p0/2005	CoA22301_2005	2005		7.976666667	13.61	10.24
transcript_HQ_CoA_transcript24924/f4p0/1835	CoA24924_1835	1835		1.863333333	1.336666667	1.776666667
transcript_HQ_CoA_transcript23223/f3p0/1973	CoA23223_1973	1972	lincRNAs	0.073333333	0.096666667	0.053333333
transcript_HQ_CoA_transcript24939/f2p0/1836	CoA24939_1836	1836		0.3	0.253333333	0.573333333
transcript_HQ_CoA_transcript22322/f2p0/2024	CoA22322_2024	2025		3.5	1.116666667	1.713333333
transcript_HQ_CoA_transcript24941/f2p0/1865	CoA24941_1865	1868		0.29	0.016666667	0.073333333
transcript_HQ_CoA_transcript24147/f2p0/1910	CoA24147_1910	1908		5.92	5.063333333	3.453333333
transcript_HQ_CoA_transcript21403/f4p0/2099	CoA21403_2099	2099		4.31	2.483333333	2.71
transcript_HQ_CoA_transcript24164/f4p0/1895	CoA24164_1895	1894	lincRNAs	4.76	0.966666667	3.356666667
transcript_HQ_CoA_transcript24174/f2p0/1915	CoA24174_1915	1915		3.856666667	3.42	2.73
transcript_HQ_CoA_transcript22367/f2p0/2110	CoA22367_2110	2121	sense lncRNAs	0.13	0.243333333	0.336666667
transcript_HQ_CoA_transcript24978/f2p0/1832	CoA24978_1832	1834		3.923333333	4.056666667	4.556666667
transcript_HQ_CoA_transcript24979/f5p0/1834	CoA24979_1834	1834		13.40333333	4.586666667	9.94
transcript_HQ_CoA_transcript24980/f2p0/1856	CoA24980_1856	1857		0.276666667	0.886666667	1.446666667
transcript_HQ_CoA_transcript24184/f2p0/1870	CoA24184_1870	1867		1.146666667	1.13	2.05
transcript_HQ_CoA_transcript22382/f2p0/2058	CoA22382_2058	2057		93.54666667	37.19333333	43.53333333
transcript_HQ_CoA_transcript24211/f2p0/1882	CoA24211_1882	1885		2.716666667	1.9	1.17
transcript_HQ_CoA_transcript21460/f2p0/2094	CoA21460_2094	2100		4.763333333	4.283333333	5.003333333
transcript_HQ_CoA_transcript24228/f2p0/1893	CoA24228_1893	1893		5.483333333	4.506666667	3.15
transcript_HQ_CoA_transcript22436/f2p0/2032	CoA22436_2032	2039		0.296666667	0.013333333	0.193333333
transcript_HQ_CoA_transcript25939/f5p0/1761	CoA25939_1761	1761		132.63	74.94	92.03666667
transcript_HQ_CoA_transcript25964/f2p0/1774	CoA25964_1774	1776		0.27	0.15	0.23

transcript_HQ_CoA_transcript22474/f2p0/2028	CoA22474_2028	2027		0.103333333	0.26	0.22
transcript_HQ_CoA_transcript24271/f11p0/1880	CoA24271_1880	1880	lincRNAs	2.196666667	1.13	3.923333333
transcript_HQ_CoA_transcript22508/f3p0/2023	CoA22508_2023	2026		14.6	10.02666667	8.143333333
transcript_HQ_CoA_transcript26032/f3p0/1761	CoA26032_1761	1761		0.996666667	1.533333333	0.173333333
transcript_HQ_CoA_transcript21582/f3p0/2097	CoA21582_2097	2097		4.483333333	3.39	3.656666667
transcript_HQ_CoA_transcript21591/f2p0/2103	CoA21591_2103	2100	sense lncRNAs	1.303333333	0.776666667	0.603333333
transcript_HQ_CoA_transcript26062/f2p0/1793	CoA26062_1793	1789		9.196666667	6.456666667	5.14
transcript_HQ_CoA_transcript26075/f3p0/1754	CoA26075_1754	1753	sense lncRNAs	1.86	2.133333333	1.936666667
transcript_HQ_CoA_transcript23498/f2p0/1954	CoA23498_1954	1954	lincRNAs	2.81	1.436666667	1.79
transcript_HQ_CoA_transcript26089/f2p0/1751	CoA26089_1751	1762		0.1	0.106666667	0
transcript_HQ_CoA_transcript22599/f2p0/2023	CoA22599_2023	2023		0.363333333	0.033333333	0.086666667
transcript_HQ_CoA_transcript26236/f2p0/1755	CoA26236_1755	1755		1.96	0.896666667	1.283333333
transcript_HQ_CoA_transcript24401/f2p0/1882	CoA24401_1882	1882	lincRNAs	11.78666667	12.48666667	11.55666667
transcript_HQ_CoA_transcript24417/f3p0/1886	CoA24417_1886	1887		2.24	1.206666667	1.286666667
transcript_HQ_CoA_transcript27151/f2p0/1664	CoA27151_1664	1664	ıse intronic lncRN	1.746666667	0.56	0.96
transcript_HQ_CoA_transcript28069/f2p0/1570	CoA28069_1570	1571		4.393333333	5.803333333	4.05
transcript_HQ_CoA_transcript27195/f2p0/1660	CoA27195_1660	1660		3.66	2.203333333	1.026666667
transcript_HQ_CoA_transcript26174/f2p0/1741	CoA26174_1741	1741	lincRNAs	2.09	4.416666667	2.696666667
transcript_HQ_CoA_transcript25254/f2p0/1836	CoA25254_1836	1840		9.733333333	5.276666667	6.996666667
transcript_HQ_CoA_transcript27199/f3p0/1657	CoA27199_1657	1638		0.053333333	0.066666667	0.073333333
transcript_HQ_CoA_transcript27229/f2p0/1672	CoA27229_1672	1674	lincRNAs	0.44	0.576666667	0.76
transcript_HQ_CoA_transcript25293/f2p0/1800	CoA25293_1800	1786		0.656666667	0.78	0.616666667
transcript_HQ_CoA_transcript26217/f2p0/1744	CoA26217_1744	1744		7.906666667	10.58333333	7.606666667
transcript_HQ_CoA_transcript28144/f2p0/1561	CoA28144_1561	1561		8.803333333	5.936666667	2.71
transcript_HQ_CoA_transcript28959/f2p0/1452	CoA28959_1452	1482		0.026666667	0	0.023333333
transcript_HQ_CoA_transcript26367/f2p0/1730	CoA26367_1730	1730		9.78	5.836666667	5.993333333
transcript_HQ_CoA_transcript26227/f2p0/1768	CoA26227_1768	1767		0.37	0.053333333	0.243333333
transcript_HQ_CoA_transcript29843/f2p0/1208	CoA29843_1208	1208		1.34	0.446666667	0.556666667
transcript_HQ_CoA_transcript28163/f3p0/1573	CoA28163_1573	1582		0.166666667	0.986666667	0.58
transcript_HQ_CoA_transcript28983/f2p0/1440	CoA28983_1440	1440	lincRNAs	25.44	21.66	9.35
transcript_HQ_CoA_transcript25330/f2p0/1819	CoA25330_1819	1819		0.606666667	1.18	1.486666667
transcript_HQ_CoA_transcript29862/f2p0/1210	CoA29862_1210	1210		19.72	4.916666667	11.11666667
transcript_HQ_CoA_transcript27304/f2p0/1658	CoA27304_1658	1655		4.636666667	6.946666667	1.73
transcript_HQ_CoA_transcript29019/f3p0/1431	CoA29019_1431	1431		2.08	2.17	0.28
transcript_HQ_CoA_transcript29909/f2p0/1182	CoA29909_1182	1180		1.543333333	1.076666667	2.293333333
transcript_HQ_CoA_transcript28240/f2p0/1554	CoA28240_1554	1560		1.88	3.28	1.823333333
transcript_HQ_CoA_transcript29063/f2p0/1423	CoA29063_1423	1423	sense lncRNAs	3.223333333	1.07	2.06
transcript_HQ_CoA_transcript26450/f2p0/1721	CoA26450_1721	1718		0.296666667	0.653333333	0.296666667
transcript_HQ_CoA_transcript28273/f2p0/1542	CoA28273_1542	1541	lincRNAs	0.146666667	0.246666667	0.406666667
transcript_HQ_CoA_transcript29945/f2p0/1157	CoA29945_1157	1157	lincRNAs	11.31666667	9.15	7.84

transcript_HQ_CoA_transcript29103/f2p0/1418	CoA29103_1418	1408	sense lncRNAs	0.27	0.776666667	0.62
transcript_HQ_CoA_transcript2739/f3p0/4565	CoA2739_4565	4566		0.146666667	0.136666667	0.203333333
transcript_HQ_CoA_transcript2650/f2p0/4584	CoA2650_4584	4595	sense lncRNAs	7.16	4.52	4.483333333
transcript_HQ_CoA_transcript28303/f5p0/1542	CoA28303_1542	1542		5.05	3.336666667	5.42
transcript_HQ_CoA_transcript29990/f2p0/1121	CoA29990_1121	1127		0.036666667	0	0.1
transcript_HQ_CoA_transcript28312/f2p0/1538	CoA28312_1538	1538		1.31	0.91	1.266666667
transcript_HQ_CoA_transcript26519/f2p0/1736	CoA26519_1736	1738		43.42333333	17.73	14.82333333
transcript_HQ_CoA_transcript27419/f2p0/1663	CoA27419_1663	1664		2.33	0.543333333	1.936666667
transcript_HQ_CoA_transcript26541/f3p0/1716	CoA26541_1716	1716	lincRNAs	12.65666667	5.636666667	11.07
transcript_HQ_CoA_transcript27444/f2p0/1635	CoA27444_1635	1636	lincRNAs	44.56666667	23.48	34.32333333
transcript_HQ_CoA_transcript29174/f3p0/1417	CoA29174_1417	1417		12.26666667	4.893333333	7.39
transcript_HQ_CoA_transcript2835/f2p0/4517	CoA2835_4517	4509		3.226666667	2.96	1.74
transcript_HQ_CoA_transcript26566/f3p0/1718	CoA26566_1718	1718		1.643333333	0.7	0.576666667
transcript_HQ_CoA_transcript26580/f2p0/1704	CoA26580_1704	1706	sense lncRNAs	0.343333333	0.353333333	0.096666667
transcript_HQ_CoA_transcript30083/f2p0/1051	CoA30083_1051	1051	lincRNAs	12.96666667	12.65	144.52
transcript_HQ_CoA_transcript26596/f2p0/1717	CoA26596_1717	1716		0.133333333	0.303333333	0.3
transcript_HQ_CoA_transcript29266/f2p0/1380	CoA29266_1380	1380	lincRNAs	0.93	0.483333333	0.77
transcript_HQ_CoA_transcript28435/f2p0/1528	CoA28435_1528	1528		4.5	2.793333333	5.706666667
transcript_HQ_CoA_transcript26631/f2p0/1704	CoA26631_1704	1705		2.093333333	4.216666667	4.363333333
transcript_HQ_CoA_transcript29292/f2p0/1371	CoA29292_1371	1370		7.543333333	5.25	2
transcript_HQ_CoA_transcript28452/f2p0/1539	CoA28452_1539	1542		0.486666667	1.036666667	0.363333333
transcript_HQ_CoA_transcript26656/f2p0/1703	CoA26656_1703	1704	se intronic lncRN	0.466666667	0.17	0.026666667
transcript_HQ_CoA_transcript29313/f2p0/1357	CoA29313_1357	1355	sense lncRNAs	0.536666667	0.686666667	0.386666667
transcript_HQ_CoA_transcript27583/f2p0/1625	CoA27583_1625	1624		0.32	0.193333333	0.466666667
transcript_HQ_CoA_transcript30190/f2p0/880	CoA30190_880	880	lincRNAs	1057.24	85.52333333	778.5366667
transcript_HQ_CoA_transcript27612/f3p0/1606	CoA27612_1606	1606		0.466666667	8.313333333	0.926666667
transcript_HQ_CoA_transcript30207/f3p0/815	CoA30207_815	815		1423.356667	571.2766667	547.0766667
transcript_HQ_CoA_transcript27621/f2p0/1617	CoA27621_1617	1619		2.826666667	2.803333333	1.7
transcript_HQ_CoA_transcript27626/f2p0/1626	CoA27626_1626	1627		1.933333333	0.193333333	1.763333333
transcript_HQ_CoA_transcript30219/f3p0/795	CoA30219_795	795	lincRNAs	1609.066667	959.8366667	521.4
transcript_HQ_CoA_transcript29361/f2p0/1373	CoA29361_1373	1373		0	9.653333333	0.796666667
transcript_HQ_CoA_transcript30228/f6p0/772	CoA30228_772	772		1402.993333	1770.363333	1511.623333
transcript_HQ_CoA_transcript30231/f2p0/775	CoA30231_775	774		456.2333333	553.3366667	44.96
transcript_HQ_CoA_transcript28544/f4p0/1506	CoA28544_1506	1506		6.073333333	2.053333333	3.286666667
transcript_HQ_CoA_transcript27644/f2p0/1623	CoA27644_1623	1632		1.266666667	0.243333333	0.98
transcript_HQ_CoA_transcript30250/f2p0/733	CoA30250_733	733		408.0766667	18.89666667	558.1333333
transcript_HQ_CoA_transcript30275/f3p0/593	CoA30275_593	593		713.63	773.1433333	892.9533333
transcript_HQ_CoA_transcript29402/f2p0/1347	CoA29402_1347	1348		0.58	2.003333333	0.303333333
transcript_HQ_CoA_transcript30280/f2p0/533	CoA30280_533	533		126.48	69.91333333	302.46
transcript_HQ_CoA_transcript30282/f2p0/438	CoA30282_438	438		1135.563333	1530.53	876.28

transcript_HQ_CoA_transcript29405/f3p0/1340	CoA29405_1340	1340	sense lncRNAs	21.39333333	10.25	12.35333333
transcript_HQ_CoA_transcript28577/f2p0/1503	CoA28577_1503	1504		0.053333333	1.053333333	1.813333333
transcript_HQ_CoA_transcript28595/f4p0/1492	CoA28595_1492	1492	lincRNAs	263.2233333	176.1033333	195.0933333
transcript_HQ_CoA_transcript26792/f3p0/1692	CoA26792_1692	1692		1.903333333	3.576666667	4.87
transcript_HQ_CoA_transcript27719/f2p0/1620	CoA27719_1620	1619		3.343333333	0.953333333	1.32
transcript_HQ_CoA_transcript26818/f2p0/1693	CoA26818_1693	1693		1.756666667	1.063333333	1.076666667
transcript_HQ_CoA_transcript29554/f2p0/1315	CoA29554_1315	1315		8.61	5.043333333	8.44
transcript_HQ_CoA_transcript29555/f2p0/1301	CoA29555_1301	1306		7.62	3.316666667	4.753333333
transcript_HQ_CoA_transcript29558/f2p0/1302	CoA29558_1302	1303		1.703333333	0.523333333	0.786666667
transcript_HQ_CoA_transcript29573/f2p0/1296	CoA29573_1296	1296	lincRNAs	651.1033333	18.17	46.04333333
transcript_HQ_CoA_transcript26880/f3p0/1672	CoA26880_1672	1673		15.35666667	9.373333333	13.77666667
transcript_HQ_CoA_transcript29579/f2p0/1303	CoA29579_1303	1303		21.68333333	13.16666667	9.343333333
transcript_HQ_CoA_transcript27822/f2p0/1594	CoA27822_1594	1595		0.776666667	0.816666667	0.756666667
transcript_HQ_CoA_transcript27823/f2p0/1588	CoA27823_1588	1587	lincRNAs	0.396666667	0.38	0.43
transcript_HQ_CoA_transcript27869/f2p0/1590	CoA27869_1590	1591		10.01666667	6.153333333	5.09
transcript_HQ_CoA_transcript29648/f2p0/1282	CoA29648_1282	1282		1.836666667	1.393333333	0.956666667
transcript_HQ_CoA_transcript28757/f2p0/1473	CoA28757_1473	1477		1.08	0.796666667	0.513333333
transcript_HQ_CoA_transcript29656/f2p0/1276	CoA29656_1276	1276		5.49	2.86	4.45
transcript_HQ_CoA_transcript29664/f3p0/1273	CoA29664_1273	1273		8.336666667	5.72	4.81
transcript_HQ_CoA_transcript29697/f2p0/1263	CoA29697_1263	1263	antisense lncRNAs	7.306666667	3.223333333	4.083333333
transcript_HQ_CoA_transcript29727/f2p0/1231	CoA29727_1231	1231		11.90333333	11.86	4.926666667
transcript_HQ_CoA_transcript29734/f2p0/1249	CoA29734_1249	1249		1.213333333	0.866666667	0.733333333
transcript_HQ_CoA_transcript29741/f2p0/1246	CoA29741_1246	1246		0.966666667	0.676666667	0.73
transcript_HQ_CoA_transcript28841/f3p0/1462	CoA28841_1462	1461		0.856666667	1.04	0.523333333
transcript_HQ_CoA_transcript29765/f2p0/1241	CoA29765_1241	1243		30.4	23.84	37.88666667
transcript_HQ_CoA_transcript27037/f3p0/1660	CoA27037_1660	1660		115.01	35.83	63.22666667
transcript_HQ_CoA_transcript27952/f3p0/1591	CoA27952_1591	1591		1.366666667	1.77	0.916666667
transcript_HQ_CoA_transcript27964/f2p0/1577	CoA27964_1577	1579		0.123333333	0.04	0.043333333
transcript_HQ_CoA_transcript27073/f4p0/1614	CoA27073_1614	1614	lincRNAs	20.4	11.90333333	24.98666667
transcript_HQ_CoA_transcript29828/f2p0/1210	CoA29828_1210	1210		0.813333333	0.41	0.683333333
transcript_HQ_CoA_transcript28014/f3p0/1581	CoA28014_1581	1582	lincRNAs	2.846666667	2.41	1.603333333
transcript_HQ_CoA_transcript27104/f2p0/1658	CoA27104_1658	1659		0.046666667	0.08	0.043333333
transcript_HQ_CoA_transcript28034/f2p0/1589	CoA28034_1589	1590		0.143333333	0.223333333	0.173333333
transcript_HQ_CoA_transcript5308/f2p0/3826	CoA5308_3826	3827	sense lncRNAs	8.473333333	2.116666667	1.65
transcript_HQ_CoA_transcript7135/f2p0/3499	CoA7135_3499	3499		3.263333333	2.443333333	2.313333333
transcript_HQ_CoA_transcript6309/f4p0/3636	CoA6309_3636	3636		11.87333333	7.1	7.213333333
transcript_HQ_CoA_transcript4522/f2p0/3973	CoA4522_3973	3972		1.49	0.646666667	0.863333333
transcript_HQ_CoA_transcript3314/f2p0/4322	CoA3314_4322	4317		1.11	0.796666667	0.91
transcript_HQ_CoA_transcript3320/f2p0/4313	CoA3320_4313	4313		5.436666667	2.616666667	2.613333333
transcript_HQ_CoA_transcript7241/f2p0/3479	CoA7241_3479	3479		24.63666667	19.47	27.1

transcript_HQ_CoA_transcript6384/f3p0/3659	CoA6384_3659	3659	ıse intronic lncRN.	1.686666667	7.33	2.623333333
transcript_HQ_CoA_transcript6392/f2p0/3671	CoA6392_3671	3664		14.17666667	9.063333333	8.723333333
transcript_HQ_CoA_transcript3355/f5p0/4305	CoA3355_4305	4305		1.02	1.796666667	0.99
transcript_HQ_CoA_transcript7290/f2p0/3482	CoA7290_3482	3493	sense lncRNAs	0.846666667	0.45	0.82
transcript_HQ_CoA_transcript5527/f2p0/3796	CoA5527_3796	3796	lincRNAs	2.116666667	3.126666667	1.686666667
transcript_HQ_CoA_transcript4599/f2p0/4002	CoA4599_4002	3998		0.403333333	0.89	0.516666667
transcript_HQ_CoA_transcript6449/f2p0/3600	CoA6449_3600	3603		1.226666667	1.103333333	0.986666667
transcript_HQ_CoA_transcript4053/f3p0/4120	CoA4053_4120	4126		3.516666667	2.53	2.33
transcript_HQ_CoA_transcript3445/f2p0/4292	CoA3445_4292	4292		9.386666667	1.71	2.253333333
transcript_HQ_CoA_transcript5626/f2p0/3819	CoA5626_3819	3819		9.303333333	30.99333333	8.06
transcript_HQ_CoA_transcript5627/f3p0/3763	CoA5627_3763	3762		4.206666667	2.296666667	1.766666667
transcript_HQ_CoA_transcript5701/f2p0/3740	CoA5701_3740	3738		0.733333333	0.32	0.983333333
transcript_HQ_CoA_transcript4789/f2p0/3953	CoA4789_3953	3946		0.076666667	0.123333333	0.94
transcript_HQ_CoA_transcript4801/f2p0/3956	CoA4801_3956	3956		5.76	1.776666667	2.353333333
transcript_HQ_CoA_transcript7520/f2p0/3465	CoA7520_3465	3469	sense lncRNAs	3.366666667	2.256666667	1.743333333
transcript_HQ_CoA_transcript7543/f2p0/3438	CoA7543_3438	3437		1.14	2.833333333	0.44
transcript_HQ_CoA_transcript8111/f4p0/3339	CoA8111_3339	3339	lincRNAs	5.466666667	3.036666667	2.83
transcript_HQ_CoA_transcript5789/f2p0/3732	CoA5789_3732	3732	lincRNAs	7.443333333	5.28	3.296666667
transcript_HQ_CoA_transcript5797/f2p0/3752	CoA5797_3752	3753		0.933333333	1.416666667	0.74
transcript_HQ_CoA_transcript4844/f2p0/3906	CoA4844_3906	3909		0.566666667	0.43	1.543333333
transcript_HQ_CoA_transcript4296/f2p0/4040	CoA4296_4040	4041		0.53	1.343333333	0.376666667
transcript_HQ_CoA_transcript4303/f2p0/4047	CoA4303_4047	4044		0.126666667	0.036666667	0.353333333
transcript_HQ_CoA_transcript8232/f2p0/3336	CoA8232_3336	3336	ıse intronic lncRN.	6.046666667	3.943333333	2.78
transcript_HQ_CoA_transcript5871/f2p0/3738	CoA5871_3738	3735		0.686666667	0.776666667	1.576666667
transcript_HQ_CoA_transcript8255/f2p0/3338	CoA8255_3338	3337		7.66	2.71	6.623333333
transcript_HQ_CoA_transcript4364/f2p0/4045	CoA4364_4045	4045		3.486666667	3.293333333	8.066666667
transcript_HQ_CoA_transcript8910/f2p0/3321	CoA8910_3321	3321		12.56333333	8.486666667	2.933333333
transcript_HQ_CoA_transcript8918/f14p0/3172	CoA8918_3172	3176	lincRNAs	8.663333333	7.59	4.693333333
transcript_HQ_CoA_transcript8932/f2p0/3241	CoA8932_3241	3241		26.66	26.69666667	30.55666667
transcript_HQ_CoA_transcript5950/f4p0/3704	CoA5950_3704	3704	antisense lncRNAs	19.99333333	8.646666667	12.54666667
transcript_HQ_CoA_transcript5972/f2p0/3714	CoA5972_3714	3731		5.143333333	2.53	4.05
transcript_HQ_CoA_transcript8976/f4p0/3210	CoA8976_3210	3209		0.103333333	0.05	0.07
transcript_HQ_CoA_transcript7816/f2p0/3407	CoA7816_3407	3416		11.03666667	5.23	7.296666667
transcript_HQ_CoA_transcript8374/f2p0/3344	CoA8374_3344	3346		1.07	2.266666667	1.183333333
transcript_HQ_CoA_transcript6000/f2p0/3694	CoA6000_3694	3694	lincRNAs	4.763333333	3.65	4.236666667
transcript_HQ_CoA_transcript8390/f3p0/3314	CoA8390_3314	3314		6.996666667	3.523333333	3.366666667
transcript_HQ_CoA_transcript9012/f2p0/3232	CoA9012_3232	3245		36.79333333	9.14	7.97
transcript_HQ_CoA_transcript6943/f2p0/3538	CoA6943_3538	3539		0.95	1.12	0.746666667
transcript_HQ_CoA_transcript8448/f3p0/3312	CoA8448_3312	3311		1.06	1.21	0.52
transcript_HQ_CoA_transcript7918/f4p0/3371	CoA7918_3371	3371		15.40666667	6.71	15.76

transcript_HQ_CoA_transcript8485/f2p0/3300	CoA8485_3300	3300		1.086666667	0.686666667	0.9
transcript_HQ_CoA_transcript7972/f2p0/3365	CoA7972_3365	3366		0.26	0.123333333	0.13
transcript_HQ_CoA_transcript8536/f2p0/3336	CoA8536_3336	3339		0.03	0.11	0.053333333
transcript_HQ_CoA_transcript7994/f2p0/3361	CoA7994_3361	3363		6.876666667	3.466666667	2.146666667
transcript_HQ_CoA_transcript9150/f5p0/3190	CoA9150_3190	3190		4.553333333	21.54333333	2.33
transcript_HQ_CoA_transcript5209/f4p0/3834	CoA5209_3834	3834		1.85	1.576666667	1.766666667
transcript_HQ_CoA_transcript9194/f2p0/3203	CoA9194_3203	3203		0.973333333	1.36	1.953333333
transcript_HQ_CoA_transcript8601/f4p0/3277	CoA8601_3277	3278		4.38	4.163333333	2.31
transcript_HQ_CoA_transcript9271/f3p0/3185	CoA9271_3185	3185		27.53333333	3.016666667	3.546666667
transcript_HQ_CoA_transcript9312/f2p0/3253	CoA9312_3253	3258		0.383333333	0.41	1.02
transcript_HQ_CoA_transcript9344/f2p0/3235	CoA9344_3235	3235	sense lncRNAs	2.966666667	2.216666667	2.08
transcript_LQ_CoA_transcript23450/f2p0/2027	CoA23450_2027	2015		1.656666667	1.3	0.956666667
transcript_HQ_CoA_transcript8777/f2p0/3260	CoA8777_3260	3260		5.07	19.88666667	4.026666667
transcript_HQ_CoA_transcript8855/f2p0/3251	CoA8855_3251	3250		1.473333333	0.503333333	1.28
transcript_HQ_CoA_transcript8872/f2p0/3308	CoA8872_3308	3302		0.276666667	0.58	0.33
transcript_HQ_CoA_transcript9691/f2p0/3155	CoA9691_3155	3155		8.916666667	1.763333333	23.55666667
transcript_HQ_CoA_transcript9705/f3p0/3157	CoA9705_3157	3157		1.15	1.276666667	0.713333333
transcript_HQ_CoA_transcript9710/f2p0/3150	CoA9710_3150	3162	lincRNAs	0.79	0.346666667	0.573333333

Supplementary Table S2. MiRNAs identified in the tropical *C. oleifera*

MiRNA ID	Expression （TPM value）		
	CoA1	CoA2	CoA3
aau-miR160	5224.533187	4823.451137	6693.783296
aau-miR168	497.0633107	1161.857598	817.9457684
ahy-miR156a	15.49846953	515.3120597	132.3792952
ahy-miR398	0	0	9.877811472
aly-miR158b-3p	10.48738879	8.092806775	6.774285267
aly-miR161-5p.1	44.96580931	54.35352037	66.52894417
aly-miR164a-3p	0	0	1.997527061
aly-miR165a-3p	78.40085703	94.033054	77.9972829
aly-miR165a-5p	0	1.655911521	0
aly-miR167b-3p	1.862509545	0	0
aly-miR395i	0	0	1.592252735
aly-miR408-5p	1.945949312	4.967734564	0
ama-miR396-3p	0	3.311823043	0
aof-miR160b	58.60845363	40.24592816	0
aof-miR393a	0	1.655911521	8.23150956
aof-miR5139b	13.64942599	18.64824777	19.89065139
aqc-miR159	24.02559421	4.540655559	13.14329621
aqc-miR167	0	0	3.18450547
aqc-miR171f	24.28259785	34.77531479	24.58815581
ata-miR167f-3p	0	1.597364987	0
ata-miR393-5p	0	9.198404187	6.477109294
ata-miR395b-3p	28.45937825	75.63624563	35.89607248
ata-miR408-3p	0	1.655911521	138.5157212
ata-miR9672-5p	1.862509545	1.597364987	1.646301912
ata-miR9674b-3p	0	1.655911521	1.646301912
ata-miR9776-5p	0	1.655911521	0
ata-miR9863a-3p	17.47898132	75.94388588	106.470858
ata-miR9863a-5p	0	3.311823043	3.292603824
ath-miR156a-5p	31.28765639	20.26286602	12.52373966
ath-miR157a-5p	9.47942726	232.7739015	84.31051918
ath-miR158a-3p	23.31682926	25.69400367	23.18498069
ath-miR158b	0	0	1.646301912
ath-miR159a	54657.30912	19540.07493	24503.4575
ath-miR159b-3p	9064.467183	4519.723547	4174.071208
ath-miR159c	309.7719805	90.0932995	95.78403435
ath-miR160a-5p	6409.144908	7382.270895	9391.879936
ath-miR161.1	44.96580931	54.35352037	66.52894417
ath-miR162a-3p	169.7288117	427.2929852	135.70228
ath-miR164a	80.76390783	72.30220013	134.0829082
ath-miR164c-5p	12.4956814	8.344246176	20.48500333
ath-miR165a-3p	78.40085703	94.033054	77.9972829
ath-miR165a-5p	0	1.655911521	0
ath-miR166a-3p	120673.2409	131881.8637	108588.3715
ath-miR167a-5p	8.687222541	111.3847903	70.41244898
ath-miR167d	365.3792463	6948.658576	3654.516888
ath-miR168a-3p	495.5134637	1158.914307	797.7038919
ath-miR168a-5p	1340.659931	3225.231983	2326.774669
ath-miR170-3p	0	0	1.592252735
ath-miR170-5p	747.5184706	1604.019558	2231.314346
ath-miR171a-3p	0	114.29592	27.66283744
ath-miR171b-3p	24.28259785	34.77531479	24.58815581
ath-miR172c	0	0	1.997527061
ath-miR319a	829.3256539	237.5358144	460.9619755
ath-miR319c	1024.658272	386.9862095	681.3438217
ath-miR390a-5p	944.8584076	1153.939741	762.7693038
ath-miR3932b-5p	1.862509545	1.655911521	0

ath-miR393a-5p	0	9.198404187	6.477109294
ath-miR393b-3p	0	1.655911521	0
ath-miR394a	31.04496755	183.4929247	125.2843548
ath-miR395a	620.6615515	3437.015925	1147.312359
ath-miR396a-3p	0	2.574758101	1.592252735
ath-miR396a-5p	63.6456176	384.070594	148.0585067
ath-miR396b-3p	0	3.311823043	0
ath-miR396b-5p	1503.018741	9461.911358	3464.139823
ath-miR397a	0	0	1.646301912
ath-miR398a-3p	0	0	9.877811472
ath-miR398b-5p	3.495796265	0	0
ath-miR399a	4.649540858	18.78259411	10.17498744
ath-miR399b	179.3884872	212.5007302	294.083259
ath-miR403-3p	1288.329221	2397.805936	2857.175538
ath-miR408-3p	1006.177641	273.1503797	24604.80262
ath-miR408-5p	1.945949312	4.967734564	0
ath-miR5658	3.725019091	14.11373425	5.236081709
ath-miR773a	1.549846953	0	0
ath-miR8175	3.725019091	18.57859001	40.48375307
ath-miR845a	7.387694888	0	5.182032532
ath-miR846-3p	1.549846953	0	1.997527061
ath-miR846-5p	0	1.597364987	0
ath-miR858a	535.4296865	1362.218451	1344.882471
ath-miR858b	532.0173301	1360.989619	1344.531246
atr-miR390.1	937.4083694	1149.709072	762.7693038
atr-miR396a	0	1.655911521	0
atr-miR8577	1.945949312	5.518048674	0
bdi-miR159a-3p	27.2499747	5.518048674	12.52373966
bdi-miR162	137.4812223	299.9315546	96.0831248
bdi-miR166g-3p	0	1.287379051	0
bdi-miR167e-3p	0	3.311823043	4.884856559
bdi-miR7782-3p	0	1.287379051	0
bdi-miR845	1.549846953	0	3.18450547
bna-miR167d	365.3792463	6948.658576	3654.516888
bna-miR168b	0	1.287379051	0
bra-miR390-3p	123.7762347	216.4745522	30.3376102
bra-miR408-5p	1006.177641	273.1503797	24604.80262
cca-miR171	0	0	1.592252735
cca-miR390	0	1.655911521	0
cca-miR396a-3p	5.754408169	30.54464517	3.589779797
cca-miR396c	0	12.5749158	1.997527061
cln-miR162	23.87302947	90.91909302	21.5369533
cme-miR166i	476.5006478	239.0190768	277.6979572
cme-miR1863	0	3.194729973	0
cpa-miR166e	100938.6297	116981.9441	95643.13636
cpa-miR8155	13.64942599	15.76350374	19.89065139
cre-miR1171	17.99977027	10.29903241	0
csi-miR156d-5p	0	27.96988707	11.36196585
csi-miR159b-5p	28.20905895	66.12486511	40.29448645
csi-miR160a-5p	5224.533187	4823.451137	6697.778351
csi-miR160c-5p	492.1044051	255.4002067	335.8134051
csi-miR166i-3p	3.099693905	2.884744037	0
csi-miR166j-3p	15.19927294	15.45965984	17.05737107
csi-miR167d-3p	6.991592529	274.8051183	74.51560145
csi-miR169l-3p	0	1.597364987	1.646301912
csi-miR2275a-3p	0	4.230669623	0
csi-miR396a-3p	1.549846953	15.47077106	9.769713118
csi-miR399f-5p	1.862509545	1.597364987	0
eun-miR397a-5p	0	0	3.643828974
eun-miR397b-3p	0	0	1.646301912

eun-miR482c-3p	355.2074558	216.5002041	170.2457756
fve-miR159a-5p	0	0	1.646301912
fve-miR159b	17.77054744	4.230669623	14.11599239
fve-miR159c	1.862509545	0	0
fve-miR164a-3p	166.4968008	392.4263627	151.9237089
fve-miR2109	1.862509545	0	0
fve-miR396b-3p	0	1.655911521	0
fve-miR396e	0	1.655911521	0
ghr-miR827a	0	0	1.592252735
gma-miR1508a	0	1.597364987	0
gma-miR1508b	0	1.597364987	0
gma-miR1510a-3p	0	0	1.646301912
gma-miR1510b-3p	0	0	1.646301912
gma-miR1511	0	2.574758101	0
gma-miR159a-5p	9.646306793	0	1.646301912
gma-miR159e-5p	9.646306793	0	1.646301912
gma-miR160b	5224.533187	4823.451137	6693.783296
gma-miR164b	80.76390783	72.30220013	134.0829082
gma-miR166m	67.99012632	82.82014421	101.9910868
gma-miR166u	101871.7406	118119.0526	96132.55657
gma-miR167h	8.687222541	111.3847903	67.22794351
gma-miR167i	4.962203451	63.02383291	24.31618444
gma-miR168b	1339.110084	3225.231983	2326.774669
gma-miR171a	24.28259785	34.77531479	24.58815581
gma-miR172d	7.783797248	9.022764583	30.47091316
gma-miR2118a-3p	9.72974656	0	0
gma-miR319c	1568.394437	538.9657449	1102.82895
gma-miR319q	1568.394437	538.9657449	1102.82895
gma-miR390b-5p	0	2.943290572	0
gma-miR390e	944.8584076	1153.939741	762.7693038
gma-miR395d	0	0	1.592252735
gma-miR396a-3p	0	2.574758101	1.592252735
gma-miR396b-3p	0	3.253276508	0
gma-miR396h	63.6456176	384.070594	148.0585067
gma-miR408d	1007.727488	273.1503797	24610.44398
gma-miR4414b	64.77252751	12.77891989	82.78477199
gma-miR4995	1.549846953	3.311823043	0
gma-miR5368	39.03689115	28.89984487	28.58148445
gma-miR6300	1.549846953	4.909188029	8.123411206
gra-miR166c	8.457999715	1.287379051	0
gra-miR166d	0	1.597364987	0
gra-miR167a	0	0	1.592252735
gra-miR167c	8.687222541	111.3847903	67.22794351
gso-miR482a	0	0	1.646301912
han-miR3630-3p	3.891898624	4.599202093	0
hbr-miR156	9.47942726	232.7739015	84.31051918
hbr-miR396a	5.754408169	13.37052728	0
hbr-miR476	0	1.597364987	0
hbr-miR6173	1.862509545	0	0
hci-miR164a	5.441745576	1.287379051	3.589779797
htu-miR171a	49.78891404	172.6217226	90.63276087
hvu-miR5048a	0	0	3.238554647
hvu-miR5049c	0	3.862137152	0
lja-miR166-3p	120691.4212	131779.1167	108514.7621
lus-miR159b	9060.971386	4522.298305	4172.478956
lus-miR172j	7.783797248	9.022764583	30.47091316
lus-miR398f	0	3.862137152	21.29555198
mdm-miR1511	5.837847936	0	0
mdm-miR159a	3.412356498	1.287379051	1.997527061
mdm-miR319b-5p	7.783797248	0	0

mdm-miR395l	0	2.574758101	1.997527061
mdm-miR396a	1.862509545	28.41421933	33.08799677
mdm-miR535d	15.08805237	32.1955875	56.1919982
mes-miR159a-5p	1.862509545	0	0
mes-miR166i	50.44880171	57.92292485	86.61058766
mes-miR482c	0	0	1.592252735
mes-miR535c	3.891898624	0	1.592252735
mes-miR535d	17.03400168	32.1955875	53.00749273
mtr-miR166b	21258.58962	13009.64921	18643.1093
mtr-miR171c	0	0	3.995054123
mtr-miR171e-5p	38.68298174	63.67019047	13.76476724
mtr-miR319a-3p	667.8317466	205.1657602	425.7756332
mtr-miR393b-3p	0	10.97755081	0
mtr-miR395a	28.45937825	75.63624563	35.89607248
mtr-miR395g	28.45937825	74.65885251	37.48832522
mtr-miR4414a-5p	64.77252751	12.77891989	82.78477199
novel_1	12.43333811	19.33290822	13.25139456
novel_10	164.7877048	531.367768	414.5029744
novel_11	171.2567134	169.890557	196.2134384
novel_12	143.1799715	168.2173922	161.10271
novel_14	24.48403986	4.599202093	12.11846533
novel_15	13.62164518	6.436895254	1.592252735
novel_16	5.045643217	2.943290572	4.884856559
novel_17	8.937541841	6.74688119	12.36159212
novel_18	13.42020317	6.623646085	3.292603824
novel_19	7.304255122	4.172123088	6.828334444
novel_2	62017.89151	35374.0529	33297.1921
novel_20	0	7.483946131	3.292603824
novel_24	11.47425385	0	1.592252735
novel_25	12.43333811	13.79760628	41.26545715
novel_26	216.7796691	601.2969131	295.4772397
novel_27	8.374559949	30.12867739	38.43217683
novel_28	14.97005012	31.97133974	68.87424542
novel_29	0	1.597364987	8.474636356
novel_3	10052.54372	6366.626225	8415.468539
novel_30	57.66961706	171.3351495	102.8574101
novel_31	0	13.18377644	8.177460383
novel_33	316.6952969	244.4372856	246.5459694
novel_34	17.98630426	24.24209625	19.75734843
novel_35	1.945949312	185.5704626	0
novel_37	54.6744594	42.27651418	24.85648721
novel_38	105.498267	71.7667936	59.78177799
novel_39	60.42886757	134.9958034	103.0734178
novel_4	1.549846953	1.597364987	0
novel_40	12.97522352	58.41966162	4.884856559
novel_42	0	8.402792711	0
novel_43	37.689335	84.77989963	99.29456036
novel_44	79.35994128	131.8183267	44.90928613
novel_5	908.1637628	803.3121104	660.2737637
novel_6	23.46261232	50.50863648	29.39011862
novel_7	52.45709431	70.59153842	108.0648362
novel_8	192.5384154	450.7623818	235.9928267
novel_9	132.7626538	327.0103149	353.3466769
nta-miR397	0	0	1.646301912
osa-miR156k	0	4.172123088	6.423060117
osa-miR159a.1	9232.629092	4542.543918	4205.75603
osa-miR159c	1.862509545	0	0
osa-miR159d	1.862509545	0	0
osa-miR159e	1.862509545	0	0
osa-miR159f	283.6142559	79.29138829	116.5429235

osa-miR160e-5p	2386.06373	3568.968081	4167.323853
osa-miR160f-5p	0	3.194729973	0
osa-miR164d	3.495796265	2.884744037	18.48747627
osa-miR164e	3.495796265	1.287379051	1.592252735
osa-miR166b-5p	53.13129678	48.41453473	28.4733861
osa-miR166d-5p	64.64011312	51.3578253	32.06316589
osa-miR166e-3p	10.00784667	10.0001577	1.592252735
osa-miR166g-3p	61987.01298	56471.20859	78782.57973
osa-miR166h-5p	10.88349115	5.82803461	5.587306858
osa-miR166i-3p	1.945949312	4.230669623	1.997527061
osa-miR166k-3p	0	1.287379051	1.592252735
osa-miR166k-5p	0	1.287379051	0
osa-miR166m	90581.33789	86528.91935	52104.24464
osa-miR167d-5p	365.3792463	6954.428064	3654.516888
osa-miR168a-5p	0	1.597364987	1.646301912
osa-miR171b	49.78891404	172.6217226	90.63276087
osa-miR171c-5p	13.64942599	0	3.292603824
osa-miR171e-5p	0	5.149516203	0
osa-miR171i-3p	3.099693905	1.287379051	4.938905736
osa-miR1863a	0	3.194729973	0
osa-miR1874-3p	5.587528636	0	0
osa-miR2275a	0	1.287379051	0
osa-miR319a-3p.2-	5.754408169	1.655911521	0
osa-miR390-3p	0	0	1.646301912
osa-miR393a	0	9.198404187	6.477109294
osa-miR395b	50.46989818	76.2562175	230.5785873
osa-miR396a-3p	0	1.287379051	0
osa-miR396c-3p	1.862509545	4.79209496	0
osa-miR396e-5p	1.549846953	19.07532678	18.46054618
osa-miR396f-5p	1.549846953	19.07532678	18.46054618
osa-miR396g	0	1.655911521	0
osa-miR397b	0	0	3.643828974
osa-miR398b	3.412356498	93.28984702	656.2807102
osa-miR399a	1.862509545	1.597364987	0
osa-miR399j	0	1.655911521	4.884856559
osa-miR408-3p	1206.822197	288.7918211	26038.64827
osa-miR5072	4.962203451	1.287379051	0
osa-miR5083	54.91799702	127.4674124	75.51714221
osa-miR5144-5p	1.862509545	0	0
osa-miR5538	0	3.311823043	0
pab-miR159a	0	1.287379051	0
pab-miR171a	8.687222541	11.77316229	5.236081709
pab-miR3711	69.06721283	52.98537233	110.3597282
pab-miR396a-3p	0	3.311823043	0
pab-miR858a	1.945949312	3.311823043	1.646301912
pde-miR159	0	1.287379051	0
peu-miR2916	0	2.574758101	0
ppe-miR1511-3p	15.5953753	15.76350374	19.89065139
ppe-miR393a	0	9.198404187	6.477109294
ppe-miR396a	1.862509545	28.3556728	31.44169486
ppe-miR398b	0	3.862137152	21.29555198
ppe-miR482e	0	1.287379051	1.592252735
ppe-miR482f	15.13692965	14.11373425	16.32780168
ppt-miR160b	1.549846953	2.943290572	3.995054123
ppt-miR166j	191.0575961	212.3637604	209.8198871
ppt-miR166m	3.412356498	4.172123088	0
ppt-miR171a	0	0	1.646301912
ppt-miR171b	0	0	1.646301912
ppt-miR319a	704.5199018	211.5552201	565.2367425
ppt-miR390c-5p	1.945949312	4.172123088	3.238554647

ppt-miR408b	1004.315131	273.1503797	24576.40676
ppt-miR535a	11.11271398	1.287379051	11.82128936
ppt-miR894	3094.226892	1765.472977	2617.646468
pta-miR159a	11.95379598	4.482109024	11.9563178
pta-miR159c	3.412356498	1.287379051	1.997527061
pta-miR319	503.7661755	174.7856434	318.0262757
ptc-miR160g	0	0	3.589779797
ptc-miR160h	1.549846953	0	1.997527061
ptc-miR166n	22051.92682	13754.38122	18822.15937
ptc-miR166p	6.59549017	2.574758101	3.18450547
ptc-miR167f-5p	8.687222541	111.3847903	67.22794351
ptc-miR167h-5p	0	4.540655559	11.2538675
ptc-miR171c	44.12472731	73.85212981	37.11189546
ptc-miR319i	1.945949312	0	0
ptc-miR393a-3p	4.649540858	12.26492986	1.646301912
ptc-miR396e-3p	0	3.253276508	0
ptc-miR397b	0	0	3.643828974
ptc-miR530a	0	1.287379051	0
ptc-miR6476a	0	1.287379051	6.36901094
ptc-miR6478	16.53336308	7.483946131	13.52164045
pvu-miR2118	9.72974656	0	0
pvu-miR482-5p	3.725019091	1.287379051	1.646301912
rgl-miR5139	13.64942599	23.24744987	26.3137115
rgl-miR5141	168.9433887	147.4425149	163.9379048
sly-miR167b-5p	0	2.884744037	1.592252735
sly-miR168a-3p	1.549846953	0	0
sly-miR171b-5p	1.549846953	0	0
sly-miR171d	42.178778	70.90883924	37.11189546
sly-miR397-5p	0	0	1.646301912
sly-miR482a	1.549846953	4.540655559	3.238554647
smo-miR171b	0	2.574758101	1.997527061
sof-miR159c	3.412356498	1.287379051	1.997527061
sof-miR408e	0	0	8.366538002
ssp-miR1128	1.549846953	0	0
stu-miR167d-3p	1.549846953	26.0847586	0
stu-miR171b-3p	3.099693905	1.287379051	4.938905736
stu-miR171d-5p	1.549846953	0	0
stu-miR398a-3p	0	3.862137152	23.29307905
stu-miR408b-5p	175.0835277	97.14204579	2527.103535
stu-miR530	0	1.287379051	0
tae-miR1122c-3p	96.56157394	20.12851969	35.3826998
tae-miR395b	615.6993481	3350.691871	1102.077053
tae-miR7757-5p	0	1.287379051	0
tae-miR9653a-3p	0	1.287379051	1.997527061
tae-miR9662a-3p	1.549846953	8.279557607	9.285185009
tae-miR9662b-3p	1.549846953	8.279557607	9.285185009
tae-miR9668-5p	0	1.655911521	0
tae-miR9670-3p	0	1.655911521	1.646301912
tae-miR9672a-3p	3.412356498	1.597364987	1.646301912
tae-miR9672b	5.35830581	6.565099551	6.936432798
tae-miR9674b-5p	1.945949312	7.911025136	4.938905736
tae-miR9773	0	1.597364987	1.592252735
tae-miR9776	0	1.655911521	0
tcc-miR530a	6.824712996	1.287379051	3.18450547
vca-miR166a-5p	3.725019091	0	0
vca-miR396b-5p	0	1.655911521	0
vca-miR535-3p	11.11271398	1.287379051	11.82128936
vca-miR535-5p	0	0	1.592252735
vun-miR164	15.67881507	15.81708108	31.84696918
vvi-miR166a	21264.3929	13006.08594	18632.33996

vvi-miR167c	10.54973209	113.9595484	70.87177248
vvi-miR171f	44.12472731	72.19621829	38.75819738
vvi-miR3630-3p	332.8967931	338.5057687	362.9634369
vvi-miR396a	45.64584733	354.3054799	137.8294701
vvi-miR396b	1507.668282	9524.503143	3511.639263
vvi-miR399i	21.71132335	0	0
zma-miR162-3p	169.7288117	427.2929852	132.4096762
zma-miR164g-3p	1.945949312	0	0
zma-miR164h-5p	3.495796265	1.287379051	3.238554647
zma-miR166h-3p	120642.5575	131856.4039	108572.9605
zma-miR171a-3p	0	114.29592	27.66283744
zma-miR171b-3p	24.28259785	34.77531479	24.58815581
zma-miR396g-3p	63.6456176	384.070594	148.0585067
zma-miR396g-5p	0	1.287379051	0
zma-miR398a-3p	5.587528636	65.51899486	945.7274159
zma-miR408b-5p	0	0	1.997527061

Supplementary Table S3. The significantly differentially expressed mRNAs

Transcript ID	CoA1_FPKM	CoA2_FPKM	CoA3_FPKM	Fold_change-CoA2/CoA1	p-value	q-value	UP/DOWN regulate	Fold_change-CoA3/CoA2	p-value	q-value	UP/DOWN regulate	Fold_change-CoA3/CoA1	p-value	q-value	UP/DOWN regulate
transcript_HQ_CoA_transcript10015/f3p0/3106	1.68	4.503333333	2.7	1.6015	0.00041267	0.0023989	UP	-0.91442	0.038856	0.11249		0.67449	0.084943	0.22908	
transcript_HQ_CoA_transcript10033/f2p0/3127	0.043333333	3.71	0.01	6.5915	3.2197E-12	9.6497E-11	UP	-8.6147	3.8537E-11	2.0356E-09	DOWN	-2.0393	0.56641	0.74902	
transcript_HQ_CoA_transcript10048/f3p0/3110	10.09333333	4.016666667	3.683333333	-1.1921	0.0047759	0.019106	DOWN	-0.26647	0.32767	0.51874		-1.4693	0.00022488	0.0027372	DOWN
transcript_HQ_CoA_transcript10056/f2p0/3109	9.503333333	0.716666667	13.12666667	-3.5552	3.7449E-08	5.8268E-07	DOWN	4.0174	6.9557E-14	6.6058E-12	UP	0.44972	0.36928	0.5864	
transcript_HQ_CoA_transcript10071/f4p0/3078	32.57666667	6.04	9.88	-2.2637	2.6056E-11	6.9948E-10	DOWN	0.55352	0.1326	0.27911		-1.7231	3.8681E-18	1.3921E-15	DOWN
transcript_HQ_CoA_transcript10074/f5p0/3098	0.38	34.57	3.33	6.6523	2.6876E-67	1.585E-64	UP	-3.5147	4.4506E-11	2.285E-09	DOWN	3.1234	0.00001206	0.00024733	UP
transcript_HQ_CoA_transcript10091/f2p0/3119	22.42	8.086666667	6.406666667	-1.3254	0.0015064	0.0072914	DOWN	-0.47292	0.26866	0.45522		-1.8093	6.7149E-12	8.0556E-10	DOWN
transcript_HQ_CoA_transcript10100/f2p0/3137	1.44	10.76	2.04	3.0671	5.3292E-19	3.093E-17	UP	-2.5544	9.1574E-24	3.9163E-21	DOWN	0.50216	0.2672	0.47914	
transcript_HQ_CoA_transcript10103/f4p0/3099	0.326666667	63.31333333	3.396666667	7.7872	3.2399E-67	1.7949E-64	UP	-4.3372	6.597E-09	2.1363E-07	DOWN	3.4338	0.00028932	0.0033547	UP
transcript_HQ_CoA_transcript10116/f6p0/3040	5.74	11.53666667	10.6	1.1778	0.006158	0.023567	UP	-0.27486	0.27801	0.4659		0.88833	0.046649	0.15279	
transcript_HQ_CoA_transcript10120/f2p0/3101	2.08	4.796666667	2.466666667	1.3484	0.002437	0.010923	UP	-1.0902	0.0022394	0.012158	DOWN	0.24674	0.56673	0.74918	
transcript_HQ_CoA_transcript10129/f5p0/3104	11.54666667	5.013333333	6.22	-1.0544	0.00084578	0.0044433	DOWN	0.15677	0.59808	0.75618		-0.90852	0.00052555	0.0053681	
transcript_HQ_CoA_transcript10132/f3p0/3108	3.55	8.723333333	4.323333333	1.4748	0.00021923	0.0013925	UP	-1.1666	0.054148	0.14533		0.29224	0.60587	0.77715	
transcript_HQ_CoA_transcript10136/f8p0/3062	45.97333333	87.80666667	40.53666667	1.0976	2.645E-07	3.4664E-06	UP	-1.2599	7.0443E-07	1.4157E-05	DOWN	-0.17664	0.50414	0.70422	
transcript_HQ_CoA_transcript10139/f2p0/3119	4.79	9.113333333	6.593333333	1.0839	0.00020308	0.0013036	UP	-0.60471	0.025275	0.08211		0.46599	0.097685	0.25286	
transcript_HQ_CoA_transcript10142/f2p0/3146	19.80333333	60.97333333	27.71666667	1.775	6.2167E-36	1.0824E-33	UP	-1.2747	0.00055054	0.003934	DOWN	0.48697	0.18068	0.37582	
transcript_HQ_CoA_transcript10145/f2p0/3104	2.693333333	23.14333333	5.283333333	3.2707	3.5456E-20	2.2275E-18	UP	-2.2849	7.2319E-15	8.1321E-13	DOWN	0.9729	0.0071007	0.039245	
transcript_HQ_CoA_transcript10146/f4p0/3095	0.72	40.03	15.33333333	6.0043	1.1201E-19	6.7278E-18	UP	-1.5354	0.35186	0.54384		4.4533	0.0039101	0.025434	UP
transcript_HQ_CoA_transcript10154/f2p0/3160	9.833333333	4.2	6.046666667	-1.0735	4.5212E-06	4.5971E-05	DOWN	0.38171	0.16729	0.32872		-0.70345	0.0047949	0.029583	
transcript_HQ_CoA_transcript10159/f2p0/3095	0.02	3.453333333	0	7.4269	2.3836E-08	3.877E-07	UP	-9.4105	7.2986E-11	3.6254E-09	DOWN	-1.9839	0.6233	0.78907	
transcript_HQ_CoA_transcript10169/f2p0/3095	2.043333333	0.613333333	1.546666667	-1.5918	0.0011373	0.0057319	DOWN	1.1927	0.0040396	0.01984	UP	-0.41117	0.32598	0.54285	
transcript_HQ_CoA_transcript10183/f2p0/3120	12.49333333	5.163333333	4.636666667	-1.1374	0.0085988	0.031123	DOWN	-0.28105	0.5714	0.73609		-1.432	3.6427E-05	0.00061909	DOWN
transcript_HQ_CoA_transcript10204/f4p0/3071	7.576666667	1.44	3.616666667	-2.2472	4.3881E-12	1.2877E-10	DOWN	1.1828	0.0020431	0.011293	UP	-1.0755	0.00081748	0.0076322	DOWN
transcript_HQ_CoA_transcript10213/f2p0/3087	2.19	4.953333333	1.89	1.3366	7.0195E-06	0.0000687	UP	-1.5384	2.5878E-07	5.8198E-06	DOWN	-0.21297	0.55984	0.74471	
transcript_HQ_CoA_transcript10227/f2p0/3091	203.0566667	65.69666667	48.64333333	-1.4853	0.00658	0.024896	DOWN	-0.56654	0.41731	0.607		-2.0589	7.2319E-05	0.0010898	DOWN
transcript_HQ_CoA_transcript10258/f2p0/3097	6.843333333	32.52333333	13.31666667	2.4027	4.6788E-07	5.8268E-06	UP	-1.4418	0.00026586	0.00216	DOWN	0.95362	0.051768	0.16425	
transcript_HQ_CoA_transcript10262/f6p0/3038	21.31	42.29333333	32.41	1.1459	6.6669E-11	1.6513E-09	UP	-0.53196	0.0012106	0.0074311		0.60064	0.00041125	0.0044066	
transcript_HQ_CoA_transcript10263/f2p0/3132	293.3966667	80.08666667	55.94	-1.7386	0.0039752	0.016472	DOWN	-0.6482	0.3681	0.56018		-2.3926	4.0463E-06	0.00010091	DOWN
transcript_HQ_CoA_transcript10288/f5p0/3054	12.18666667	5.51	8.66	-1.0127	0.0062712	0.02391	DOWN	0.52244	0.12185	0.263		-0.50077	0.043857	0.14629	
transcript_HQ_CoA_transcript10293/f2p0/3094	9.156666667	19.70333333	10.62	1.2478	0.0019701	0.0091669	UP	-1.0404	0.0046442	0.022113	DOWN	0.19656	0.43852	0.64942	
transcript_HQ_CoA_transcript10316/f2p0/3079	13.86	5.533333333	14.25	-1.1801	1.0438E-05	9.7117E-05	DOWN	1.2173	8.1399E-07	0.00001606	UP	0.026091	0.91435	0.96218	
transcript_HQ_CoA_transcript10327/f2p0/3066	5.946666667	1.9	4.05	-1.4827	0.00039651	0.0023204	DOWN	0.94334	0.0097317	0.039185		-0.55261	0.20615	0.40863	
transcript_HQ_CoA_transcript10329/f3p0/3077	14.39666667	42.51666667	12.65333333	1.7346	4.4431E-06	4.5278E-05	UP	-1.9003	3.3755E-08	9.3742E-07	DOWN	-0.18063	0.50745	0.70706	
transcript_HQ_CoA_transcript10331/f3p0/3035	0.943333333	1.876666667	1.386666667	1.143	0.0044306	0.017943	UP	-0.57811	0.10061	0.22965		0.55412	0.22318	0.4298	
transcript_HQ_CoA_transcript10368/f2p0/3063	0.963333333	6.913333333	1.596666667	2.9684	6.3558E-07	7.7226E-06	UP	-2.268	3.8957E-05	0.00043699	DOWN	0.69055	0.40577	0.62009	
transcript_HQ_CoA_transcript10401/f2p0/3078	0.126666667	33.92666667	1.736666667	8.1475	6.333E-47	1.654E-44	UP	-4.3776	0.016149	0.058335		3.7554	0.014781	0.066479	
transcript_HQ_CoA_transcript10402/f5p0/3080	19.28333333	35.95	43.39333333	1.0746	0.00025363	0.0015741	UP	0.10871	0.67401	0.80993		1.1699	1.1382E-08	6.428E-07	UP
transcript_HQ_CoA_transcript10420/f2p0/3064	2	4.736666667	2.593333333	1.3925	0.0016002	0.0076843	UP	-1.0118	0.012248	0.047083	DOWN	0.36786	0.27524	0.48856	
transcript_HQ_CoA_transcript10421/f8p0/3062	1.083333333	26.08333333	0.42	4.7401	5.4721E-16	2.5073E-14	UP	-6.096	6.147E-15	6.9548E-13	DOWN	-1.3707	0.12861	0.3016	
transcript_HQ_CoA_transcript10431/f5p0/3081	51.35	21.47666667	26.48666667	-1.1176	0.003215	0.013759	DOWN	0.16565	0.68999	0.82093		-0.96368	0.00074071	0.007059	
transcript_HQ_CoA_transcript10432/f2p0/3080	1.553333333	3.37	2.266666667	1.2881	0.0018881	0.008835	UP	-0.73604	0.033699	0.10161		0.53849	0.16777	0.35815	
transcript_HQ_CoA_transcript10437/f2p0/3082	2.723333333	5.766666667	3.396666667	1.2316	0.0010147	0.0052143	UP	-0.90674	0.0043684	0.021103		0.31258	0.41649	0.62963	
transcript_HQ_CoA_transcript10443/f2p0/3081	10.03666667	20.06333333	3.14	1.1269	0.011951	0.04067	UP	-2.8143	1.6218E-11	9.1184E-10	DOWN	-1.6978	0.00073507	0.0070126	DOWN
transcript_HQ_CoA_transcript10447/f5p0/2977	13.33666667	31.00333333	30.76666667	1.374	0.00048544	0.0027556	UP	-0.14585	0.79582	0.88743		1.2144	0.0098448	0.049698	UP
transcript_HQ_CoA_transcript10449/f2p0/3057	1.206666667	4.77	1.05	2.1696	1.9905E-05	0.00017101	UP	-2.3344	6.8579E-08	1.7623E-06	DOWN	-0.1765	0.78946	0.89846	
transcript_HQ_CoA_transcript10450/f2p0/3095	26.43666667	11.74666667	20.57333333	-1.0278	2.4081E-05	0.00020213	DOWN	0.66188	0.00064312	0.0044752		-0.37646	0.083441	0.22626	
transcript_HQ_CoA_transcript10484/f3p0/3065	8.133333333	16.72666667	12.44	1.2191	0.0011159	0.0056387	UP	-0.60586	0.11955	0.25925		0.59905	0.019521	0.081788	
transcript_HQ_CoA_transcript10487/f4p0/3016	0	64.33	5.676666667	13.455	1.9877E-10	4.6E-09	UP	-3.5759	0.097204	0.22387		9.8651	0.011575	0.056	
transcript_HQ_CoA_transcript10494/f2p0/3067	7.373333333	13.93	7.666666667	1.0827	0.0028924	0.01262	UP	-1.0028	0.0012667	0.0077058	DOWN	0.065509	0.83377	0.9229	
transcript_HQ_CoA_transcript10503/f27p0/3022	9.026666667	126.5533333	14.88333333	3.9593	4.0764E-69	2.5698E-66	UP	-3.2443	1.2292E-38	1.7331E-35	DOWN	0.70227	0.024797	0.096737	
transcript_HQ_CoA_transcript10542/f3p0/3053	2.003333333	0.253333333	1.833333333	-2.8139	0.0018366	0.0086383	DOWN	2.6827	6.7461E-06	0.00009698	UP	-0.14848	0.82721	0.91976	
transcript_HQ_CoA_transcript10544/f3p0/3069	0.446666667	32.90666667	2.893333333	6.3395	5.163E-86	6.2927E-83	UP	-3.6313	0.012292	0.047194	DOWN	2.6929	0.026403	0.10162	
transcript_HQ_CoA_transcript10548/f2p0/3057	5.02	1.24	3.266666667	-1.842	5.5888E-05	0.00042484	DOWN	1.2372	0.0006935	0.0047593	UP	-0.61734	0.10619	0.2669	
transcript_HQ_CoA_transcript10550/f9p0/2994	4.17	14.25666667	40.6	1.9053	0.0010275	0.0052705	UP	1.3943	0.012016	0.046406	UP	3.2886	3.4431E-30	7.8998E-27	UP
transcript_HQ_CoA_transcript10562/f2p0/3058	9.373333333	20.65	10.88333333	1.2947	1.2184E-05	0.00011149	UP	-1.0718	0.00001697	0.00021188	DOWN	0.21022	0.36002	0.57702	
transcript_HQ_CoA_transcript10567/f2p0/3040	19.22	2.963333333	11.95666667	-2.5902	0.0034624	0.014687	DOWN	1.8816	1.8027E-06	3.1498E-05	UP	-0.71334	NA	NA	
transcript_HQ_CoA_transcript10590/f3p0/3032	2.916666667	7.326666667	2.733333333	1.4944	0.00037334	0.0022032	UP	-1.5636	1.4748E-08	4.424E-07	DOWN	-0.084419	0.84289	0.92775	
transcript_HQ_CoA_transcript10616/f2p0/3052	3.963333333	1.55	4.536666667	-1.2081	0.0017164	0.0081621	DOWN	1.4087	4.2057E-05	0.00046634	UP	0.18829	0.51661	0.71324	
transcript_HQ_CoA_transcript10637/f3p0/3042	7.853333333	26.7	8.27	1.9352	1.3584E-07	1.8862E-06	UP	-1.8499	2.8867E-07	6.4212E-06	DOWN	0.071892	0.71009	0.84784	
transcript_HQ_CoA_transcript10649/f2p0/3059	7.646666667	20.54666667	12.79	1.5931	5.3883E-07	6.6142E-06	UP	-0.84734	0.0016822	0.0096777		0.73416	0.0056615	0.033489	
transcript_HQ_CoA_transcript10654/f2p0/3061	7.276666667	2.703333333	7.66	-1.2835	6.2198E-05	0.00046584	DOWN	1.3444	0.00025262	0.002072	UP	0.050282	0.89552	0.95359	
transcript_HQ_CoA_transcript10660/f14p0/3004	14.57666667	55.69666667	30.72666667	2.1428	0.00097163	0.0050222	UP	-1.0354	0.052069	0.14097		1.0918	0.024467	0.095849	
transcript_HQ_CoA_transcript1067/f4p0/5673	0.553333333	2.163333333	1.996666667	2.1152	0.0070716	0.026395	UP	-0.24642	0.71352	0.83589		1.8563	0.0028716	0.020095	UP
transcript_HQ_CoA_transcript10682/f2p0/3032	2.15	5.366666667	2.173333333	1.4726	2.0323E-05	0.00017427	UP	-1.4274	0.0005056	0.0036883	DOWN	0.033733	0.94369	0.97485	
transcript_HQ_CoA_transcript10697/f2p0/3038	11.87666667	0.54	34.75	-4.2987	3.286E-10	7.3712E-09	DOWN	5.8749	8.0791E-23	3.0221E-20	UP	1.5611	0.00015102	0.00199	UP
transcript_HQ_CoA_transcript10729/f3p0/3044	16.74666667	4.523333333	9.796666667	-1.7334	2.8633E-09	5.4584E-08	DOWN	0.96354	0.00001099	0.00014736		-0.78139	0.0061962	0.035776	
transcript_HQ_CoA_transcript10746/f5p0/3028	1.256666667	20.69333333	2.406666667	4.2072	2.8199E-23	2.1661E-21	UP	-3.243	2.5366E-07	5.7116E-06	DOWN	0.94901	0.17674	0.37039	
transcript_HQ_CoA_transcript10755/f2p0/3039	8.6	15.74666667	18.17333333	1.0497	0.002758	0.01212	UP	0.058935	0.90221	0.94795		1.0942	0.0080751	0.043132	UP
transcript_HQ_CoA_transcript10757/f13p0/2990	15.22333333	37.48333333	16.01333333	1.4624	3.9066E-06	4.0192E-05	UP	-1.3835	5.6383E-06	8.3051E-05	DOWN	0.066221	0.68811	0.83391	
transcript_HQ_CoA_transcript1076/f3p0/5638	9.79	21.39333333	11.61666667	1.2773	8.4735E-06	8.1277E-05	UP	-1.019	2.7254E-05	0.0003194	DOWN	0.24558	0.24727	0.45684	
transcript_HQ_CoA_transcript10777/f3p0/3018	3.17	39.89666667	19.97666667	3.7768	2.7741E-13	9.4796E-12	UP	-1.1028	0.17125	0.33427		2.6597	0.00017546	0.0022412	UP
transcript_HQ_CoA_transcript10789/f2p0/3025	3.203333333	6.463333333	5.896666667	1.169	2.4124E-05	0.0002024	UP	-0.29578	0.29365	0.48294		0.85997	0.0048211	0.029705	
transcript_HQ_CoA_transcript10790/f2p0/3039	2.933333333	6.136666667	4.88	1.2239	0.00057655	0.0031883	UP	-0.49391	0.053536	0.14401		0.7181	0.029306	0.10993	
transcript_HQ_CoA_transcript10797/f2p0/3032	17.09333333	3.45	11.07	-2.1477	1.2467E-12	3.9295E-11	DOWN	1.5207	1.9742E-07	4.5631E-06	UP	-0.63929	0.024176	0.095002	
transcript_HQ_CoA_transcript10798/f3p0/2978	2.783333333	22.29	11.38	3.1943	1.2127E-07	1.7029E-06	UP	-1.1258	0.0068636	0.029914	DOWN	2.0512	0.00022425	0.0027313	UP
transcript_HQ_CoA_transcript1080/f3p0/5639	24.33	10.86666667	16.81333333	-1.0067	6.3573E-07	7.7226E-06	DOWN	0.47249	0.10936	0.24354		-0.54739	0.059318	0.18011	
transcript_HQ_CoA_transcript10817/f2p0/3019	18.56	5	9.583333333	-1.7416	8.6343E-06	8.2645E-05	DOWN	0.78882	0.0082549	0.034536		-0.96181	0.0054703	0.0326	

transcript_HQ_CoA_transcript10834/f4p0/3022	22.96666667	60.47	24.87666667	1.5542	3.1784E-08	5.0397E-07	UP	-1.4303	1.1401E-05	0.00015164	DOWN	0.11079	0.7349	0.86497	
transcript_HQ_CoA_transcript10835/f2p0/3015	9.406666667	2.47	5.473333333	-1.7699	3.5531E-09	6.6761E-08	DOWN	1.0068	0.00056141	0.0039931	UP	-0.77519	0.014149	0.064428	
transcript_HQ_CoA_transcript10861/f3p0/3005	5.293333333	1.713333333	2.086666667	-1.4435	0.0026618	0.01176	DOWN	0.10414	0.82979	0.90723		-1.3527	7.3366E-05	0.0011019	DOWN
transcript_HQ_CoA_transcript10868/f3p0/3087	7.073333333	15.17333333	10.39	1.2701	0.00001382	0.00012434	UP	-0.69467	0.0018004	0.010242		0.56116	0.086069	0.23103	
transcript_HQ_CoA_transcript10869/f6p0/2975	12.38333333	3.576666667	14.53	-1.6223	0.0014176	0.0069329	DOWN	1.8465	1.1752E-05	0.00015597	UP	0.21384	0.51662	0.71324	
transcript_HQ_CoA_transcript10881/f2p0/3023	7.103333333	2.986666667	7.473333333	-1.0914	6.967E-06	6.8222E-05	DOWN	1.1612	0.00019944	0.0017186	UP	0.057749	0.84582	0.92953	
transcript_HQ_CoA_transcript10884/f5p0/2916	50.27	14.23666667	21.98333333	-1.6862	0.00005793	0.00043782	DOWN	0.48217	0.0052039	0.02418		-1.2118	0.0018216	0.014231	DOWN
transcript_HQ_CoA_transcript1089/f2p0/5675	2.026666667	0.48	2.29	-1.9324	7.0876E-08	1.0416E-06	DOWN	2.1204	1.19E-08	3.6783E-07	UP	0.17505	0.57348	0.75413	
transcript_HQ_CoA_transcript10913/f2p0/3019	10.55	28.5	9.093333333	1.5748	0.0001095	0.00076115	UP	-1.7846	9.2854E-06	0.00012739	DOWN	-0.22086	0.58816	0.76489	
transcript_HQ_CoA_transcript10917/f3p0/3018	3.95	12.91333333	6.14	1.8683	1.1615E-05	0.00010676	UP	-1.2357	0.0010651	0.0067286	DOWN	0.62171	0.070334	0.20137	
transcript_HQ_CoA_transcript10920/f5p0/3004	8.94	26.92666667	8.366666667	1.7337	9.8305E-12	2.7886E-10	UP	-1.8282	9.8519E-12	5.7877E-10	DOWN	-0.10695	0.64849	0.80632	
transcript_HQ_CoA_transcript10925/f3p0/3033	3.86	9.593333333	3.803333333	1.4778	0.00022527	0.0014246	UP	-1.4836	1.1249E-05	0.00014995	DOWN	-0.018754	0.96149	0.98318	
transcript_HQ_CoA_transcript10926/f2p0/3024	10	2.713333333	4.183333333	-1.7095	2.5524E-06	2.7368E-05	DOWN	0.45813	0.20618	0.38049		-1.2656	3.1209E-05	0.00054975	DOWN
transcript_HQ_CoA_transcript10927/f2p0/2966	24.62	10.27	13.04	-1.1031	9.9798E-08	1.4187E-06	DOWN	0.1882	0.29333	0.48268		-0.92658	1.2613E-06	3.8331E-05	
transcript_HQ_CoA_transcript10939/f2p0/3027	10.88666667	3.86	8.103333333	-1.3497	0.0008828	0.0046152	DOWN	0.92326	1.6844E-05	0.00021076		-0.43709	0.24124	0.4501	
transcript_HQ_CoA_transcript10952/f2p0/3069	15.20333333	28.11	19.65333333	1.0215	0.0083251	0.030295	UP	-0.64141	0.086444	0.20526		0.36818	0.047693	0.15513	
transcript_HQ_CoA_transcript10961/f2p0/3013	8.116666667	2.493333333	3.646666667	-1.5568	0.0028274	0.012384	DOWN	0.40722	0.23767	0.41915		-1.16	0.01698	0.073508	
transcript_HQ_CoA_transcript10991/f2p0/3024	11.56	27.71	39.06333333	1.43	3.7569E-06	0.00003887	UP	0.35909	0.3112	0.5026		1.7748	1.2109E-05	0.00024807	UP
transcript_HQ_CoA_transcript10997/f2p0/3001	33.01333333	11.22666667	19.33	-1.4125	2.0501E-05	0.00017547	DOWN	0.6391	0.065519	0.16772		-0.78204	0.039405	0.13588	
transcript_HQ_CoA_transcript1100/f3p0/5601	8.206666667	14.79666667	19.67	1.0078	1.9808E-06	2.1895E-05	UP	0.26169	0.18717	0.3558		1.2569	1.5926E-08	8.5896E-07	UP
transcript_HQ_CoA_transcript11004/f2p0/2999	12.75333333	3.696666667	8.55	-1.6476	1.5469E-05	0.00013668	DOWN	1.0914	0.012621	0.0482	UP	-0.56762	0.072314	0.2048	
transcript_HQ_CoA_transcript11009/f2p0/2996	20.08666667	7.233333333	14.12333333	-1.3221	0.00023172	0.0014583	DOWN	0.81689	0.00056998	0.004043		-0.51502	0.14926	0.33213	
transcript_HQ_CoA_transcript11012/f3p0/2938	9.766666667	4.276666667	7.066666667	-1.0382	0.00032191	0.0019385	DOWN	0.57241	0.033474	0.10111		-0.47757	0.017337	0.074716	
transcript_HQ_CoA_transcript11019/f3p0/3001	7.83	27.11333333	10.97333333	1.9597	8.2534E-12	2.3576E-10	UP	-1.465	1.9908E-07	4.5955E-06	DOWN	0.48243	0.038302	0.13298	
transcript_HQ_CoA_transcript11022/f7p0/2998	54.75666667	133.7466667	66.00333333	1.4552	3.7243E-12	1.1071E-10	UP	-1.1818	1.2116E-08	3.735E-07	DOWN	0.26067	0.052478	0.16573	
transcript_HQ_CoA_transcript1105/f2p0/5589	3.613333333	0.48	5.406666667	-2.7344	9.0393E-06	8.6075E-05	DOWN	3.316	3.8497E-06	6.0464E-05	UP	0.56664	0.29429	0.50988	
transcript_HQ_CoA_transcript11051/f3p0/2994	28.57	335.8	210.77	3.7021	1.4798E-11	4.0929E-10	UP	-0.81318	0.029815	0.092906		2.8748	8.3961E-08	3.7496E-06	UP
transcript_HQ_CoA_transcript11059/f3p0/3007	4.766666667	17.02666667	5.943333333	2.0085	1.7279E-08	2.8587E-07	UP	-1.6839	9.3071E-07	1.7995E-05	DOWN	0.31083	0.20143	0.40292	
transcript_HQ_CoA_transcript11063/f3p0/3006	1.446666667	5.953333333	16.04666667	2.2625	0.01245	0.042055	UP	1.2326	0.13906	0.28915		3.4812	1.5151E-16	4.4142E-14	UP
transcript_HQ_CoA_transcript11068/f2p0/3023	11.67333333	2.273333333	12.19666667	-2.1529	0.0079331	0.029076	DOWN	2.2242	0.0062951	0.02806	UP	0.060847	0.85468	0.9339	
transcript_HQ_CoA_transcript1107/f7p0/5609	4.433333333	1.48	6.536666667	-1.44	3.9419E-05	0.0003121	DOWN	2.0032	4.9422E-10	2.0847E-08	UP	0.55254	0.002519	0.01816	
transcript_HQ_CoA_transcript11073/f2p0/2990	0.63	1.523333333	1.536666667	1.4124	0.013017	0.043513	UP	-0.12202	0.77824	0.8756		1.2808	0.015598	0.069344	
transcript_HQ_CoA_transcript1111/f2p0/5609	1.82	8.916666667	7.53	2.4585	0.0017173	0.0081631	UP	-0.38968	0.4468	0.63471		2.0519	0.013296	0.061815	
transcript_HQ_CoA_transcript11111/f2p0/3025	16.37333333	6.506666667	11.50666667	-1.1786	0.0018584	0.0087273	DOWN	0.66892	0.0043722	0.021111		-0.52272	0.15582	0.3415	
transcript_HQ_CoA_transcript11116/f2p0/2999	8.25	2.52	5.17	-1.5366	0.00138	0.0067766	DOWN	0.86271	0.05119	0.13906		-0.68512	0.020158	0.083523	
transcript_HQ_CoA_transcript11123/f2p0/3006	8.43	3.776666667	6.396666667	-1.0158	0.0023531	0.010625	DOWN	0.62076	0.072134	0.17998		-0.4069	0.19727	0.39703	
transcript_HQ_CoA_transcript11125/f2p0/2992	3.476666667	7.743333333	3.653333333	1.3157	0.00075417	0.0040174	UP	-1.2471	0.0088463	0.036445	DOWN	0.054971	0.91016	0.96019	
transcript_HQ_CoA_transcript11142/f3p0/2987	8.886666667	46.63	21.13333333	2.5336	0.00082466	0.0043485	UP	-1.2565	0.32893	0.51984		1.2682	0.3978	0.61345	
transcript_HQ_CoA_transcript11149/f2p0/3007	4.53	10.76333333	7.183333333	1.3779	0.0047808	0.019117	UP	-0.72381	0.10067	0.22971		0.64429	0.14734	0.32945	
transcript_HQ_CoA_transcript11154/f2p0/2985	32.55666667	13.61333333	17.07	-1.096	0.0063107	0.024021	DOWN	0.15576	0.62443	0.7749		-0.95138	0.0025204	0.018163	
transcript_HQ_CoA_transcript11164/f2p0/2980	29.04333333	6.036666667	9.523333333	-2.1047	1.8544E-10	4.3352E-09	DOWN	0.52147	0.028847	0.090722		-1.5968	3.0505E-07	1.1427E-05	DOWN
transcript_HQ_CoA_transcript11213/f4p0/2978	12.68333333	5.356666667	6.3	-1.0803	0.013176	0.043909	DOWN	0.08552	0.66639	0.80516		-1.0078	0.014739	0.066356	
transcript_HQ_CoA_transcript11232/f3p0/2957	9.1	2.576666667	1.7	-1.6897	0.011162	0.038526	DOWN	-0.74822	0.040488	0.11615		-2.4503	0.00013383	0.0018027	DOWN
transcript_HQ_CoA_transcript11236/f2p0/2963	0.02	149.1033333	23.17666667	12.841	1.1288E-28	1.385E-26	UP	-2.839	0.17444	0.33898		9.9722	0.0034667	0.023197	UP
transcript_HQ_CoA_transcript11239/f2p0/3023	0.286666667	2.69	7.223333333	3.447	0.0090692	0.032523	UP	1.2224	0.13807	0.28771		4.6596	3.4312E-09	2.1717E-07	UP
transcript_HQ_CoA_transcript11242/f2p0/2980	13.17333333	29.91666667	15.11666667	1.3759	0.0086081	0.031145	UP	-1.1655	0.026638	0.085523		0.19594	0.36948	0.58661	
transcript_HQ_CoA_transcript1125/f11p0/5569	15.75333333	5.42	16.66666667	-1.3892	1.6411E-11	4.5118E-10	DOWN	1.4641	3.6461E-06	5.7463E-05	UP	0.063128	0.83603	0.92431	
transcript_HQ_CoA_transcript11252/f3p0/2976	43.68	15.24666667	29.25	-1.3612	4.7365E-10	1.0346E-08	DOWN	0.78406	6.3661E-06	9.1877E-05		-0.58868	0.0013972	0.011673	
transcript_HQ_CoA_transcript11261/f3p0/2984	8.423333333	17.09333333	18.32333333	1.1721	0.00047863	0.00272	UP	-0.032187	0.94863	0.97283		1.1251	0.022063	0.089004	
transcript_HQ_CoA_transcript11264/f2p0/2969	0.94	10.59666667	2.196666667	3.6413	5.5679E-11	1.3982E-09	UP	-2.3949	1.7794E-05	0.00022037	DOWN	1.2384	0.12932	0.30266	
transcript_HQ_CoA_transcript11287/f2p0/2960	0.856666667	4.373333333	5.37	2.5192	0.00066609	0.0036092	UP	0.145	0.79847	0.88897		2.6484	0.00017792	0.0022678	UP
transcript_HQ_CoA_transcript11295/f2p0/2962	0.93	9.736666667	8.953333333	3.5574	0.00046641	0.0026597	UP	-0.26888	0.41666	0.60634		3.2703	0.00064786	0.0063554	UP
transcript_HQ_CoA_transcript11299/f3p0/2958	14.96333333	38.83333333	18.16	1.5345	6.2817E-08	9.3367E-07	UP	-1.2545	1.6742E-06	2.9562E-05	DOWN	0.26782	0.23748	0.44587	
transcript_HQ_CoA_transcript11315/f8p0/2961	50.14666667	141.1366667	43.52666667	1.6408	1.2359E-10	2.9769E-09	UP	-1.8372	1.9142E-13	1.6628E-11	DOWN	-0.20683	0.50231	0.70284	
transcript_HQ_CoA_transcript11318/f3p0/2966	10.53666667	4.663333333	12.31	-1.0175	0.0002674	0.0016484	DOWN	1.2528	2.6461E-07	5.9292E-06	UP	0.22272	0.258	0.46859	
transcript_HQ_CoA_transcript11324/f2p0/2989	0	0.35	0.92	5.986	0.0018002	0.0084975	UP	1.2589	0.34451	0.53659		7.2314	0.00053125	0.0054203	UP
transcript_HQ_CoA_transcript11328/f3p0/2962	6.996666667	0.996666667	13.17333333	-2.6901	1.1359E-05	0.00010467	DOWN	3.5933	8.4318E-15	9.3665E-13	UP	0.89379	0.043426	0.14521	
transcript_HQ_CoA_transcript11334/f2p0/2957	36.33	16	17.98666667	-1.0305	1.5862E-08	2.6386E-07	DOWN	0.025545	0.89618	0.9449		-1.0181	4.2924E-09	2.6982E-07	DOWN
transcript_HQ_CoA_transcript11341/f2p0/2955	3.89	0.273333333	4.303333333	-3.6351	4.5614E-05	0.00035516	DOWN	3.759	1.0292E-06	1.9508E-05	UP	0.1128	0.81563	0.91418	
transcript_HQ_CoA_transcript11352/f2p0/2969	18.19333333	7.896666667	9.563333333	-1.0456	1.1188E-06	1.3011E-05	DOWN	0.11667	0.72969	0.84645		-0.93953	0.0038768	0.025261	
transcript_HQ_CoA_transcript11360/f2p0/3024	32.54333333	12.39333333	17.20333333	-1.2547	0.00068706	0.0037009	DOWN	0.33453	0.25108	0.43471		-0.92909	0.0034911	0.023319	
transcript_HQ_CoA_transcript11366/f2p0/2966	0.673333333	4.693333333	5.68	2.9579	0.0018589	0.0087273	UP	0.13028	0.8385	0.91156		3.0744	0.00078488	0.0073728	UP
transcript_HQ_CoA_transcript11373/f4p0/2925	8.216666667	16.09	12.84	1.1303	3.5086E-09	6.6127E-08	UP	-0.47794	0.0029855	0.015384		0.63947	0.00029481	0.0034011	
transcript_HQ_CoA_transcript11374/f2p0/2992	0.07	0.763333333	0	3.6335	0.00022874	0.0014434	UP	-7.2019	1.2982E-06	2.3677E-05	DOWN	-3.5801	0.25816	0.46879	
transcript_HQ_CoA_transcript11393/f2p0/2960	4.273333333	1.06	3.046666667	-1.853	0.00033377	0.0020007	DOWN	1.379	0.0073695	0.031663	UP	-0.48937	0.20334	0.4055	
transcript_HQ_CoA_transcript11397/f4p0/2946	14.73333333	0.763333333	51.36666667	-4.0683	5.1587E-05	0.00039527	DOWN	5.8816	8.5622E-05	0.00084786	UP	1.8011	3.0761E-07	1.1499E-05	UP
transcript_HQ_CoA_transcript11408/f5p0/2954	2.273333333	22.76666667	2.256666667	3.5068	2.8525E-08	4.5585E-07	UP	-3.5129	1.3293E-10	6.3284E-09	DOWN	-0.020086	0.97419	0.98939	
transcript_HQ_CoA_transcript11415/f3p0/2960	4.416666667	9.213333333	11.97	1.2303	0.0005005	0.0028255	UP	0.21828	0.40395	0.59441		1.4348	9.1504E-07	2.9159E-05	UP
transcript_HQ_CoA_transcript11419/f2p0/2946	0.436666667	17.42333333	0.03	5.1183	6.5092E-23	4.8771E-21	UP	-9.2889	1.6142E-20	4.1125E-18	DOWN	-3.8012	0.015689	0.069641	
transcript_HQ_CoA_transcript11439/f2p0/2949	7.356666667	1.406666667	8.733333333	-2.2565	0.00089884	0.0046897	DOWN	2.4888	5.7453E-21	1.5717E-18	UP	0.22768	0.69933	0.8415	
transcript_HQ_CoA_transcript11440/f2p0/2963	62.94	24.27	36.57333333	-1.2268	7.0157E-05	0.00051677	DOWN	0.43145	0.02301	0.076444		-0.8065	0.0061573	0.03563	
transcript_HQ_CoA_transcript11448/f2p0/2975	6.283333333	12.12333333	6.57	1.1116	0.00024752	0.001541	UP	-1.0323	0.0073497	0.0316	DOWN	0.064676	0.85969	0.93686	
transcript_HQ_CoA_transcript11462/f2p0/2954	6.573333333	33.54666667	8.396666667	2.471	1.4773E-05	0.00013168	UP	-2.1046	0.00066695	0.004613	DOWN	0.35571	0.46259	0.67063	
transcript_HQ_CoA_transcript11463/f3p0/3032	5.476666667	10.25666667	3.936666667	1.0543	0.00035738	0.0021206	UP	-1.5284	1.1595E-06	2.1488E-05	DOWN	-0.48698	0.12488	0.2965	
transcript_HQ_CoA_transcript11466/f2p0/2974	11.98	22.46666667	11.4	1.0726	0.0071292	0.026572	UP	-1.1275	0.001876	0.010544	DOWN	-0.066913	0.84797	0.93061	
transcript_HQ_CoA_transcript11467/f2p0/2976	6.64	1.97	6.63	-1.5788	0.00015795	0.0010451	DOWN	1.5809	0.0000542	0.0005759	UP	-0.0093251	0.97513	0.98969	
transcript_HQ_CoA_transcript11470/f2p0/2936	10.41333333	1.11	3.453333333	-3.0073	0.0024167	0.010856	DOWN	1.4529	0.060567	0.15823		-1.5693	0.042269	0.14259	
transcript_HQ_CoA_transcript11471/f2p0/2966	18.32666667	39.92333333	24.97666667	1.2832	2.5726E-06	2.7568E-05	UP	-0.82556	0.00042948	0.003217		0.44481	0.03765	0.13118	
transcript_HQ_CoA_transcript11492/f2p0/2972	2.633333333	0.53	0.716666667	-2.1537	0.0016472	0.0078831	DOWN	0.29531	0.63989	0.7852		-1.8737	0.0031719	0.021641	DOWN

transcript_HQ_CoA_transcript11513/f3p0/2952	1.303333333	5.566666667	4.01	2.2015	0.0049147	0.019578	UP	-0.59249	0.42218	0.61147		1.5978	6.5196E-06	0.00014902	UP
transcript_HQ_CoA_transcript11523/f2p0/2938	0.35	2.916666667	2.33	3.1784	0.00022886	0.0014434	UP	-0.45172	0.51496	0.68929		2.7191	8.8869E-06	0.00019213	UP
transcript_HQ_CoA_transcript11534/f10p0/2845	202.4466667	29.75333333	65.62	-2.6288	4.3777E-10	9.6194E-09	DOWN	0.98266	0.0064844	0.028639		-1.654	0.00019336	0.0024293	DOWN
transcript_HQ_CoA_transcript11537/f2p0/2934	1.043333333	18.71	5.073333333	4.3476	2.4726E-23	1.9154E-21	UP	-2.0314	0.002937	0.015181	DOWN	2.3004	0.0015292	0.012393	UP
transcript_HQ_CoA_transcript1154/f3p0/5558	3.52	1.313333333	2.33	-1.2535	6.6139E-06	6.5043E-05	DOWN	0.65895	0.019679	0.067826		-0.60711	0.0091404	0.047074	
transcript_HQ_CoA_transcript11566/f3p0/3011	1.986666667	0.27	3.57	-2.6644	1.7966E-06	2.0028E-05	DOWN	3.5227	4.9941E-14	4.7925E-12	UP	0.84318	0.011965	0.057266	
transcript_HQ_CoA_transcript11567/f2p0/2971	0.413333333	19.63333333	2.21	5.7293	8.4355E-29	1.042E-26	UP	-3.3044	1.9375E-12	1.3907E-10	DOWN	2.4128	2.4765E-05	0.00044962	UP
transcript_HQ_CoA_transcript11570/f3p0/2954	13.87666667	32.2	23.25	1.369	0.0021483	0.0098361	UP	-0.62943	0.11656	0.25486		0.72893	0.011721	0.056388	
transcript_HQ_CoA_transcript11596/f2p0/2948	4	7.28	7.84	1.0226	0.014631	0.047613	UP	-0.051981	0.92153	0.95861		0.95946	0.044602	0.14799	
transcript_HQ_CoA_transcript11599/f15p0/2875	20.85	1.553333333	7.173333333	-3.6009	2.1203E-21	1.4628E-19	DOWN	2.0646	3.2838E-06	5.2566E-05	UP	-1.5466	5.2219E-08	2.4576E-06	DOWN
transcript_HQ_CoA_transcript11604/f3p0/2952	35.35	6.906666667	8.386666667	-2.1958	2.0965E-11	5.7037E-10	DOWN	0.12455	0.64298	0.78745		-2.0826	6.1551E-14	1.1528E-11	DOWN
transcript_HQ_CoA_transcript11609/f3p0/2930	107.04	38.64666667	111.2633333	-1.3401	0.00011082	0.00076946	DOWN	1.3856	0.00077219	0.0051825	UP	0.036755	0.9189	0.96458	
transcript_HQ_CoA_transcript11612/f2p0/2926	28.74	8.466666667	12.56	-1.584	0.0075876	0.028029	DOWN	0.4264	0.40147	0.592		-1.1745	0.052416	0.16565	
transcript_HQ_CoA_transcript11614/f2p0/2938	5.65	0.53	3.603333333	-3.2221	8.9418E-05	0.00063982	DOWN	2.5661	0.0021003	0.011553	UP	-0.66582	0.075155	0.21003	
transcript_HQ_CoA_transcript1163/f2p0/5581	7.843333333	2.32	4.763333333	-1.6195	8.7441E-05	0.00062714	DOWN	0.89379	0.00078175	0.005239		-0.73533	0.046703	0.15291	
transcript_HQ_CoA_transcript11634/f11p0/2864	69.61666667	13.21	83.78333333	-2.2164	1.325E-08	2.2306E-07	DOWN	2.4975	4.6837E-09	1.5781E-07	UP	0.2694	0.32675	0.54365	
transcript_HQ_CoA_transcript11640/f2p0/2923	4.063333333	9.203333333	4.91	1.3508	8.9313E-05	0.00063932	UP	-1.078	0.012215	0.046977	DOWN	0.25987	0.5794	0.75828	
transcript_HQ_CoA_transcript11645/f10p0/2898	5.6	2.33	3.69	-1.0978	0.0067012	0.025286	DOWN	0.5043	0.19852	0.37036		-0.60519	0.041275	0.14026	
transcript_HQ_CoA_transcript11657/f5p0/2928	30.00333333	83.21333333	41.29	1.6243	3.5696E-27	3.7505E-25	UP	-1.1526	7.8705E-13	6.2721E-11	DOWN	0.45946	0.0041153	0.026428	
transcript_HQ_CoA_transcript11672/f2p0/2942	8.026666667	15.22	12.38333333	1.0572	0.010518	0.036702	UP	-0.43196	0.24806	0.4312		0.61526	0.07344	0.20653	
transcript_HQ_CoA_transcript11674/f2p0/2927	0.27	6.686666667	0.62	4.7839	5.1492E-16	2.3653E-14	UP	-3.5624	6.6339E-16	8.444E-14	DOWN	1.2031	0.1616	0.34973	
transcript_HQ_CoA_transcript11677/f2p0/2958	4.43	8.276666667	6.64	1.0581	0.00047104	0.0026819	UP	-0.46998	0.023825	0.078527		0.57702	0.052101	0.16491	
transcript_HQ_CoA_transcript11684/f12p0/2902	17.71	6.02	19.49666667	-1.4392	0.0086975	0.0314	DOWN	1.5667	0.0068532	0.029886	UP	0.12104	0.79697	0.90282	
transcript_HQ_CoA_transcript11700/f7p0/2875	0.126666667	376.2633333	44.49333333	11.665	1.5804E-62	6.7194E-60	UP	-3.2418	0.13023	0.27555		8.4008	0.0023594	0.017295	UP
transcript_HQ_CoA_transcript11705/f3p0/2877	8.493333333	19.19333333	10.93333333	1.3403	0.00028067	0.0017213	UP	-0.97751	0.00060259	0.0042362		0.35135	0.23541	0.44418	
transcript_HQ_CoA_transcript11710/f3p0/2901	2.05	9.576666667	5.13	2.3586	1.4033E-06	1.6044E-05	UP	-1.033	0.022706	0.075725		1.3131	0.0014549	0.011986	UP
transcript_HQ_CoA_transcript11730/f5p0/2871	34.39666667	12.35333333	20.02666667	-1.3163	6.7887E-09	1.2085E-07	DOWN	0.54384	0.0044954	0.021576		-0.78629	0.00085186	0.0078849	
transcript_HQ_CoA_transcript11738/f2p0/2913	0.393333333	5.73	6.18	4.0055	2.9503E-11	7.7833E-10	UP	-0.05331	0.9217	0.95868		3.9398	1.264E-11	1.3977E-09	UP
transcript_HQ_CoA_transcript11759/f6p0/2902	7.266666667	0.906666667	3.453333333	-2.8131	6.3669E-13	2.0823E-11	DOWN	1.7519	0.00021557	0.0018284	UP	-1.073	0.0011143	0.0097537	DOWN
transcript_HQ_CoA_transcript11774/f3p0/2914	15.79333333	5.8	16.64	-1.2961	0.0032806	0.01401	DOWN	1.3588	7.2243E-09	2.319E-07	UP	0.051897	0.89929	0.95544	
transcript_HQ_CoA_transcript11785/f2p0/2931	4.983333333	1.543333333	4.61	-1.5372	0.0014842	0.0071974	DOWN	1.4217	6.3521E-06	9.1747E-05	UP	-0.12697	0.74719	0.87268	
transcript_HQ_CoA_transcript11786/f6p0/2907	16.59666667	30.22333333	19.76333333	1.0282	1.2515E-08	2.1244E-07	UP	-0.7607	1.0018E-05	0.00013636		0.2548	0.20028	0.40137	
transcript_HQ_CoA_transcript11794/f3p0/2987	7.826666667	0.713333333	1.643333333	-3.2522	0.0021367	0.0097926	DOWN	1.0087	0.44521	0.63318		-2.2601	0.0025798	0.018475	DOWN
transcript_HQ_CoA_transcript11812/f2p0/2892	26.93	10.29	15.69666667	-1.2162	0.00013645	0.00092391	DOWN	0.44742	0.065448	0.16758		-0.78275	0.011354	0.055293	
transcript_HQ_CoA_transcript11816/f2p0/2905	3.543333333	1.07	1.11	-1.5866	8.0722E-05	0.00058515	DOWN	-0.080246	0.85783	0.92245		-1.6786	5.3724E-06	0.00012757	DOWN
transcript_HQ_CoA_transcript11829/f2p0/2912	6.78	1.986666667	6.96	-1.6003	1.3531E-05	0.00012198	DOWN	1.6574	9.3684E-08	2.3236E-06	UP	0.04397	0.90413	0.95728	
transcript_HQ_CoA_transcript11831/f2p0/2913	0.07	1.16	0.706666667	4.1787	6.1329E-06	0.00006077	UP	-0.843	0.29635	0.48567		3.3206	0.0048466	0.029822	UP
transcript_HQ_CoA_transcript1184/f2p0/5572	5.356666667	1.596666667	2.456666667	-1.5773	8.7007E-05	0.00062501	DOWN	0.4657	0.23216	0.41257		-1.1249	0.0018536	0.014386	DOWN
transcript_HQ_CoA_transcript11842/f2p0/2913	0.936666667	0	0.096666667	-7.1912	6.4452E-05	0.00048055	DOWN	3.8672	0.32592	0.51686		-3.354	0.039843	0.13685	
transcript_HQ_CoA_transcript11848/f2p0/2922	4.37	1.13	1.756666667	-1.8169	1.6558E-05	0.00014526	DOWN	0.49249	0.21836	0.39553		-1.3355	0.00046337	0.004874	DOWN
transcript_HQ_CoA_transcript1185/f2p0/5525	1.576666667	0.24	0.156666667	-2.6042	4.7463E-07	5.8988E-06	DOWN	-0.74133	0.56461	0.73059		-3.3704	0.00018362	0.0023308	DOWN
transcript_HQ_CoA_transcript11865/f3p0/2904	13.27666667	3.483333333	4.073333333	-1.8022	0.00010962	0.00076168	DOWN	0.096054	0.87226	0.93071		-1.7121	0.0009844	0.0088659	DOWN
transcript_HQ_CoA_transcript11866/f3p0/2925	42.26333333	14.34333333	40.83	-1.3938	0.004905	0.019554	DOWN	1.3393	0.010769	0.04254	UP	-0.065888	0.81584	0.91421	
transcript_HQ_CoA_transcript11887/f2p0/2903	42.33333333	18.46333333	28.21666667	-1.045	3.0839E-07	3.9816E-06	DOWN	0.46303	0.015496	0.056448		-0.59301	0.0020886	0.01582	
transcript_HQ_CoA_transcript11893/f2p0/2925	4.453333333	8.196666667	5.086666667	1.0443	0.0017986	0.0084921	UP	-0.84787	0.010523	0.041713		0.18357	0.5564	0.7422	
transcript_HQ_CoA_transcript11898/f2p0/2893	1.613333333	5.153333333	1.09	1.8187	0.00062163	0.0034006	UP	-2.3851	5.8026E-05	0.00060949	DOWN	-0.57583	0.46458	0.67214	
transcript_HQ_CoA_transcript11902/f2p0/2895	5.903333333	1.313333333	1.873333333	-2.02	0.00041283	0.002399	DOWN	0.35624	0.47458	0.65808		-1.6766	0.0041635	0.026618	DOWN
transcript_HQ_CoA_transcript11903/f3p0/2892	20.66	36.91	36.76333333	1.0015	0.00049275	0.0027881	UP	-0.16699	0.47891	0.66164		0.8226	5.1414E-05	0.00081919	
transcript_HQ_CoA_transcript11906/f4p0/2889	49.50666667	9.483333333	12.71666667	-2.2564	4.3688E-05	0.00034191	DOWN	0.31249	0.59428	0.75357		-1.954	5.0315E-09	3.0887E-07	DOWN
transcript_HQ_CoA_transcript11918/f2p0/2916	33.42666667	11.21	57.45333333	-1.4212	2.3875E-07	3.163E-06	DOWN	2.2041	1.6398E-10	7.609E-09	UP	0.77077	0.021955	0.088761	
transcript_HQ_CoA_transcript11953/f2p0/2890	2.346666667	6.756666667	4.31	1.6976	1.5511E-07	2.1273E-06	UP	-0.80254	0.02694	0.08622		0.88036	0.037016	0.12949	
transcript_HQ_CoA_transcript11954/f3p0/2888	42.43333333	2.463333333	7.88	-3.947	3.6401E-71	2.6619E-68	DOWN	1.5344	3.3547E-07	7.3462E-06	UP	-2.4242	6.7974E-21	3.3721E-18	DOWN
transcript_HQ_CoA_transcript11957/f2p0/2894	0.793333333	2.336666667	1.383333333	1.7256	9.5371E-05	0.00067554	UP	-0.90942	0.0089893	0.036926		0.80383	0.091476	0.24117	
transcript_HQ_CoA_transcript11966/f3p0/2891	30.13	9.196666667	4.113333333	-1.5855	0.0050132	0.019898	DOWN	-1.2918	0.03651	0.10743		-2.8853	1.0017E-24	8.3572E-22	DOWN
transcript_HQ_CoA_transcript11971/f6p0/2802	11.07333333	34.56666667	6.083333333	1.811	7.3734E-07	8.8684E-06	UP	-2.6547	3.4057E-72	3.1212E-68	DOWN	-0.85728	0.02623	0.10108	
transcript_HQ_CoA_transcript11972/f3p0/2934	3.403333333	9.28	16.60666667	1.5984	0.00015754	0.0010438	UP	0.69814	0.094558	0.21914		2.2847	2.9039E-10	2.2975E-08	UP
transcript_HQ_CoA_transcript11973/f3p0/2856	9.123333333	3.593333333	6.92	-1.1774	0.00043949	0.0025275	DOWN	0.79097	0.030575	0.094423		-0.39926	0.32364	0.54074	
transcript_HQ_CoA_transcript11983/f2p0/2868	24.31333333	6.966666667	34.95666667	-1.6701	2.6685E-05	0.00022145	DOWN	2.1777	6.0125E-15	6.8449E-13	UP	0.49859	0.19519	0.39436	
transcript_HQ_CoA_transcript11984/f2p0/2883	2.213333333	0.57	2.13	-1.8039	0.003083	0.013303	DOWN	1.7347	0.00075433	0.0050812	UP	-0.079538	0.88549	0.94903	
transcript_HQ_CoA_transcript12000/f3p0/2888	1.433333333	4.623333333	8.103333333	1.8878	0.00731	0.027135	UP	0.60859	0.35749	0.54945		2.4843	2.8353E-12	3.8549E-10	UP
transcript_HQ_CoA_transcript12010/f4p0/2826	19.52	7.946666667	13.74	-1.1318	0.001733	0.0082229	DOWN	0.62812	0.051596	0.14002		-0.51441	0.040683	0.13885	
transcript_HQ_CoA_transcript12015/f2p0/2884	12.28333333	3.186666667	1.75	-1.7685	0.0029058	0.012658	DOWN	-1.0537	0.082182	0.19776		-2.8311	1.8456E-10	1.5328E-08	DOWN
transcript_HQ_CoA_transcript12020/f3p0/2875	16.21	6.46	10.78666667	-1.1769	1.9128E-07	2.5828E-06	DOWN	0.58449	0.041605	0.11868		-0.60388	0.032953	0.11927	
transcript_HQ_CoA_transcript12047/f2p0/2890	20.99	6.963333333	5.153333333	-1.4527	0.00011984	0.00082394	DOWN	-0.56355	0.15587	0.3135		-2.0271	6.0285E-11	5.6732E-09	DOWN
transcript_HQ_CoA_transcript12060/f2p0/2895	0.866666667	4.083333333	2.413333333	2.4161	1.5696E-05	0.00013856	UP	-0.92486	0.1689	0.33098		1.4758	0.022938	0.09133	
transcript_HQ_CoA_transcript12066/f2p0/2887	2.78	45.89666667	16.18	4.2316	2.9128E-16	1.3689E-14	UP	-1.6634	0.00020949	0.0017867	DOWN	2.553	2.1533E-13	3.5931E-11	UP
transcript_HQ_CoA_transcript12068/f3p0/2889	5.27	22.64333333	5.94	2.2632	3.8474E-06	3.9695E-05	UP	-2.0969	1.749E-07	4.1047E-06	DOWN	0.15555	0.68141	0.82961	
transcript_HQ_CoA_transcript12077/f3p0/2870	3.866666667	0	0.206666667	-9.2165	1.7443E-06	1.9492E-05	DOWN	5.0671	0.035404	0.10527		-4.1625	0.0021005	0.015886	DOWN
transcript_HQ_CoA_transcript12079/f3p0/2883	32.21666667	63.19	57.98	1.1282	0.0057321	0.022165	UP	-0.28562	0.53176	0.70344		0.8323	0.030464	0.11294	
transcript_HQ_CoA_transcript12081/f2p0/2879	45.87666667	16.84	31.59666667	-1.2778	0.0025066	0.011171	DOWN	0.73741	0.058959	0.15522		-0.55224	0.020813	0.085513	
transcript_HQ_CoA_transcript12096/f2p0/2883	2.3	5.963333333	5.51	1.5478	3.3245E-05	0.00026976	UP	-0.26165	0.52511	0.6978		1.2708	0.0053603	0.032133	UP
transcript_HQ_CoA_transcript12107/f2p0/2953	2.333333333	9.603333333	5.133333333	2.2005	2.1872E-11	5.9416E-10	UP	-1.0605	8.5624E-05	0.00084786	DOWN	1.1277	0.00020145	0.0024984	UP
transcript_HQ_CoA_transcript12116/f2p0/2891	3.646666667	8.316666667	3.073333333	1.3483	0.0072181	0.02686	UP	-1.5759	0.008631	0.035775	DOWN	-0.24305	0.61953	0.78651	
transcript_HQ_CoA_transcript12129/f13p0/2775	83.87666667	29.30333333	71.72	-1.3798	0.0013006	0.0064439	DOWN	1.1531	0.00045375	0.0033663	UP	-0.23537	0.54897	0.73764	
transcript_HQ_CoA_transcript12143/f5p0/2865	10.07333333	2.816666667	8.083333333	-1.6752	2.3079E-05	0.0001948	DOWN	1.3576	3.4104E-06	5.4262E-05	UP	-0.33054	0.31785	0.5348	
transcript_HQ_CoA_transcript12150/f2p0/2869	8.296666667	18.82333333	13.06	1.3449	2.5241E-07	3.3199E-06	UP	-0.67709	0.00039608	0.0030074		0.65368	0.012022	0.057422	
transcript_HQ_CoA_transcript12162/f2p0/2903	12.26666667	5.013333333	6.28	-1.1495	0.0018339	0.0086299	DOWN	0.17577	0.62423	0.77481		-0.98381	0.0025289	0.018203	
transcript_HQ_CoA_transcript12166/f2p0/2886	4.146666667	0.036666667	0.636666667	-6.3955	0.00021987	0.0013953	DOWN	3.7819	0.012041	0.046481	UP	-2.6308	0.07082	0.20213	
transcript_HQ_CoA_transcript12175/f6p0/2870	18.03666667	34.74666667	26.74333333	1.1089	8.2537E-10	1.7404E-08	UP	-0.53116	0.00037138	0.0028507		0.56488	0.00040492	0.0043438	

transcript_HQ_CoA_transcript12195/f3p0/2869	10.56333333	18.94	2.873333333	1.0119	1.7119E-05	0.00014939	UP	-2.8715	2.4725E-13	2.0981E-11	DOWN	-1.8744	6.9625E-06	0.00015739	DOWN
transcript_HQ_CoA_transcript12210/f3p0/2898	32	8.71	14.84666667	-1.7309	9.5903E-07	1.1326E-05	DOWN	0.61658	0.0044055	0.021216		-1.1233	0.00054862	0.0055574	DOWN
transcript_HQ_CoA_transcript12212/f2p0/2862	38.72333333	13.26333333	41.52333333	-1.3646	0.00062091	0.0033976	DOWN	1.48	3.3803E-05	0.00038845	UP	0.10244	0.71599	0.85185	
transcript_HQ_CoA_transcript12213/f7p0/2852	28.29333333	59.17666667	37.67666667	1.2177	4.6211E-09	8.4737E-08	UP	-0.80323	4.0021E-05	0.00044701		0.40215	0.0083806	0.044356	
transcript_HQ_CoA_transcript12223/f2p0/2876	6.72	15.59666667	3.553333333	1.3834	3.2342E-05	0.00026397	UP	-2.2763	4.6135E-06	7.0291E-05	DOWN	-0.90358	0.050764	0.16168	
transcript_HQ_CoA_transcript12224/f2p0/2873	2.89	12.17	9.72	2.2281	5.0374E-05	0.00038662	UP	-0.48252	0.18243	0.34987		1.7356	0.00074649	0.0070994	UP
transcript_HQ_CoA_transcript12245/f4p0/2804	21.52	63.11333333	27.94	1.7198	9.8444E-09	1.7043E-07	UP	-1.3356	1.2699E-05	0.00016602	DOWN	0.37118	0.083571	0.22648	
transcript_HQ_CoA_transcript12254/f2p0/2858	6.92	12.61	7.103333333	1.0314	0.00060389	0.0033134	UP	-0.98238	0.0081525	0.034246		0.035501	0.91572	0.96288	
transcript_HQ_CoA_transcript12258/f2p0/2869	5.516666667	0.956666667	4.99	-2.3935	1.2644E-05	0.00011483	DOWN	2.253	0.00024609	0.0020254	UP	-0.15006	0.71993	0.85529	
transcript_HQ_CoA_transcript12262/f2p0/2859	7.39	1.756666667	3.976666667	-1.9419	1.1951E-06	1.3845E-05	DOWN	1.05	0.0087866	0.036265	UP	-0.90182	0.001369	0.01149	
transcript_HQ_CoA_transcript12265/f3p0/2859	18.92	5.44	8.456666667	-1.6416	2.4725E-12	7.5086E-11	DOWN	0.4968	0.2068	0.38125		-1.1579	0.00065574	0.0064056	DOWN
transcript_HQ_CoA_transcript1227/f4p0/5444	12.52666667	4.873333333	3.5	-1.2105	0.0067442	0.025407	DOWN	-0.60882	0.08699	0.20627		-1.8302	0.00015874	0.0020693	DOWN
transcript_HQ_CoA_transcript12272/f3p0/2882	16.93	5.143333333	6.513333333	-1.5734	3.6783E-11	9.5522E-10	DOWN	0.19845	0.40681	0.59724		-1.3869	1.2198E-12	1.763E-10	DOWN
transcript_HQ_CoA_transcript12285/f2p0/2891	6.186666667	15.97333333	7.533333333	1.5355	0.00011405	0.0007889	UP	-1.2436	0.00070549	0.0048268	DOWN	0.27898	0.44268	0.65335	
transcript_HQ_CoA_transcript12293/f2p0/2855	195.6	65.00333333	87.29333333	-1.4557	0.00024628	0.0015347	DOWN	0.27974	0.33023	0.52134		-1.1836	0.0045536	0.028624	DOWN
transcript_HQ_CoA_transcript12302/f2p0/2854	1.853333333	8.533333333	5.216666667	2.3753	7.6477E-06	7.4172E-05	UP	-0.87521	0.0541	0.14523		1.4872	0.0053974	0.032323	UP
transcript_HQ_CoA_transcript12306/f2p0/2859	33.45	123.05	83.71666667	2.0484	1.2151E-19	7.2594E-18	UP	-0.71989	0.16235	0.32246		1.3116	0.0065298	0.037108	UP
transcript_HQ_CoA_transcript12315/f3p0/2902	1.016666667	5.576666667	11.07333333	2.6155	2.6184E-05	0.00021797	UP	0.85829	0.11349	0.24973		3.4614	4.4028E-15	1.023E-12	UP
transcript_HQ_CoA_transcript12326/f2p0/2837	12.69	4.983333333	7.506666667	-1.1856	0.00034478	0.0020552	DOWN	0.4286	0.16524	0.32631		-0.76784	0.0033022	0.02235	
transcript_HQ_CoA_transcript12339/f2p0/2853	2.9	7.67	3.256666667	1.5495	4.3506E-05	0.00034078	UP	-1.3614	0.0054398	0.025027	DOWN	0.17629	0.72838	0.86104	
transcript_HQ_CoA_transcript12341/f2p0/2878	0	29.69	5.576666667	12.266	3.4651E-18	1.8854E-16	UP	-2.5121	0.20226	0.37561		9.7387	0.012686	0.059677	
transcript_HQ_CoA_transcript12361/f2p0/2849	103.3566667	44.68	90.62	-1.0497	2.1752E-09	4.2395E-08	DOWN	0.87489	9.6498E-06	0.00013189		-0.18729	0.27467	0.48795	
transcript_HQ_CoA_transcript12366/f2p0/2870	0	48.86	2.173333333	13.027	6.3859E-25	5.6674E-23	UP	-4.642	0.0058958	0.026689	DOWN	8.3687	0.032245	0.11738	
transcript_HQ_CoA_transcript12380/f3p0/2849	10.99	25.00333333	19.45666667	1.3288	1.1522E-08	1.9669E-07	UP	-0.50121	0.011761	0.045632		0.81638	0.00054489	0.0055257	
transcript_HQ_CoA_transcript12385/f2p0/2851	4.036666667	8.876666667	5.84	1.2858	0.0066605	0.025174	UP	-0.73268	0.11933	0.25896		0.53801	0.17726	0.37103	
transcript_HQ_CoA_transcript12392/f2p0/2868	1.34	15.92666667	1.39	3.687	0.00080089	0.0042379	UP	-3.6254	0.0010209	0.0064924	DOWN	0.049421	0.92082	0.96509	
transcript_HQ_CoA_transcript12407/f7p0/2833	5.566666667	15.12	5.956666667	1.5971	8.6288E-16	3.8759E-14	UP	-1.4833	2.0463E-09	7.4564E-08	DOWN	0.10205	0.72923	0.86148	
transcript_HQ_CoA_transcript12409/f2p0/2847	11.27666667	25.47333333	10.26	1.3374	0.00029256	0.0017829	UP	-1.4715	4.9489E-05	0.00053801	DOWN	-0.14694	0.62121	0.7876	
transcript_HQ_CoA_transcript1241/f2p0/5509	7.736666667	3.04	4.183333333	-1.1922	1.6956E-05	0.00014825	DOWN	0.3097	0.22201	0.39989		-0.89351	0.00013386	0.0018027	
transcript_HQ_CoA_transcript12415/f3p0/2856	13.33333333	4.6	10.85	-1.3716	1.5385E-07	2.1117E-06	DOWN	1.0899	1.6516E-09	6.1404E-08	UP	-0.29525	0.21019	0.41383	
transcript_HQ_CoA_transcript12430/f10p0/2793	6.666666667	92.20666667	34.21666667	3.9663	3.4299E-20	2.1623E-18	UP	-1.5881	2.9671E-06	0.0000486	DOWN	2.364	7.9081E-10	5.7372E-08	UP
transcript_HQ_CoA_transcript12431/f2p0/2849	13.38333333	24.91	12.01	1.0545	2.6934E-06	2.8711E-05	UP	-1.1997	5.1999E-05	0.00055703	DOWN	-0.1582	0.61389	0.78215	
transcript_HQ_CoA_transcript12433/f2p0/2856	3.11	5.753333333	3.58	1.0566	0.0019747	0.0091813	UP	-0.83626	0.00098256	0.0062948		0.2063	0.55654	0.74234	
transcript_HQ_CoA_transcript12438/f6p0/2668	10.79333333	19.21666667	13.96	1.0006	0.0037744	0.015783	UP	-0.62092	0.065739	0.1681		0.36675	0.11056	0.27455	
transcript_HQ_CoA_transcript12459/f2p0/2951	13.08333333	23.25666667	10.19	1.0044	0.0021556	0.0098622	UP	-1.3603	2.3142E-05	0.00027651	DOWN	-0.36888	0.041307	0.14026	
transcript_HQ_CoA_transcript12460/f2p0/2844	2.03	15.20666667	13.15333333	3.0957	4.5539E-10	9.9706E-09	UP	-0.37859	0.28967	0.47923		2.7006	5.6775E-10	4.3062E-08	UP
transcript_HQ_CoA_transcript12461/f3p0/2950	11.25	28.34	15.23333333	1.4901	1.3555E-08	2.2756E-07	UP	-1.0433	9.2478E-06	0.00012697	DOWN	0.43395	0.018561	0.078826	
transcript_HQ_CoA_transcript12467/f6p0/2814	23.47333333	46.28	19.33666667	1.1483	0.00025881	0.0016031	UP	-1.4133	5.9021E-08	1.5543E-06	DOWN	-0.27839	0.27674	0.49048	
transcript_HQ_CoA_transcript12477/f2p0/2835	5.503333333	2.056666667	5	-1.25	0.0014243	0.0069549	DOWN	1.1278	0.0026395	0.01391	UP	-0.13438	0.6527	0.80984	
transcript_HQ_CoA_transcript12480/f2p0/2830	13.71	5.026666667	4.416666667	-1.2959	5.4648E-10	1.1795E-08	DOWN	-0.33633	0.40718	0.59762		-1.6458	5.7096E-06	0.00013384	DOWN
transcript_HQ_CoA_transcript12488/f2p0/2839	8.77	0	6.806666667	-10.368	5.0263E-12	1.4703E-10	DOWN	9.997	2.4212E-14	2.4792E-12	UP	-0.3758	0.61258	0.78111	
transcript_HQ_CoA_transcript12511/f3p0/2834	9.363333333	3.36	5.956666667	-1.305	0.001899	0.008877	DOWN	0.65934	0.090277	0.21184		-0.65923	0.012834	0.060155	
transcript_HQ_CoA_transcript12520/f5p0/2832	80.19333333	32.4	28.48333333	-1.1542	0.0013843	0.0067893	DOWN	-0.33856	0.20494	0.379		-1.5043	0.00002429	0.0004423	DOWN
transcript_HQ_CoA_transcript12522/f6p0/2828	18.77333333	43.37	28.02333333	1.3717	7.609E-12	2.1804E-10	UP	-0.77863	3.2643E-06	0.0000523		0.58004	0.0021081	0.01593	
transcript_HQ_CoA_transcript1254/f4p0/5475	11.09333333	3.876666667	7.283333333	-1.4037	0.011509	0.039519	DOWN	0.78513	0.079391	0.19322		-0.62672	0.10973	0.27309	
transcript_HQ_CoA_transcript12553/f3p0/2839	4.03	0.663333333	0.883333333	-2.4072	0.0022239	0.010131	DOWN	0.24115	0.70038	0.82805		-2.1793	0.0047153	0.029294	DOWN
transcript_HQ_CoA_transcript12558/f2p0/2861	1.57	20.93	7.383333333	3.935	1.9726E-12	6.0713E-11	UP	-1.6644	9.9693E-05	0.00096477	DOWN	2.2552	6.6588E-05	0.0010194	UP
transcript_HQ_CoA_transcript12562/f3p0/2836	4.093333333	10.06	7.916666667	1.4661	7.5922E-09	1.3398E-07	UP	-0.49768	0.07967	0.19349		0.95612	0.0052319	0.0316	
transcript_HQ_CoA_transcript12565/f4p0/2800	2.623333333	10.96	3.04	2.2417	2.5294E-08	4.0922E-07	UP	-2.0219	1.7791E-07	4.1541E-06	DOWN	0.20603	0.55519	0.74117	
transcript_HQ_CoA_transcript12573/f2p0/2814	26.61666667	53	28.61666667	1.1545	4.7251E-07	5.8804E-06	UP	-1.0441	6.3338E-08	1.6608E-06	DOWN	0.098015	0.58404	0.76204	
transcript_HQ_CoA_transcript12574/f2p0/2779	7.076666667	1.916666667	4.233333333	-1.7152	2.122E-07	2.8359E-06	DOWN	0.97972	0.003868	0.019125		-0.74872	0.0026055	0.018608	
transcript_HQ_CoA_transcript12607/f2p0/2827	5.6	11.15	11.29333333	1.1803	0.0061777	0.023623	UP	-0.15287	0.63824	0.78424		1.012	0.0082376	0.043788	UP
transcript_HQ_CoA_transcript12642/f2p0/2825	5.41	33.9	8.476666667	2.7968	7.4557E-12	2.1465E-10	UP	-2.1607	1.4144E-15	1.7399E-13	DOWN	0.62516	0.14759	0.32981	
transcript_HQ_CoA_transcript12653/f3p0/2835	9.666666667	3.356666667	6.693333333	-1.4049	0.0065404	0.024776	DOWN	0.86301	0.061714	0.16047		-0.55036	0.20319	0.40534	
transcript_HQ_CoA_transcript12656/f2p0/2815	16.40333333	7.05	4.89	-1.0609	1.2797E-08	2.1643E-07	DOWN	-0.68049	0.011088	0.043528		-1.7543	1.8692E-12	2.5992E-10	DOWN
transcript_HQ_CoA_transcript12660/f3p0/2825	2.163333333	14.84333333	9.22	2.9307	1.2253E-09	2.4946E-08	UP	-0.832	0.059245	0.15575		2.0847	6.8286E-15	1.5285E-12	UP
transcript_HQ_CoA_transcript12668/f2p0/2832	1.993333333	17.95666667	3.446666667	3.2976	6.8748E-09	1.2226E-07	UP	-2.5175	1.3704E-06	0.00002487	DOWN	0.77035	0.18374	0.38	
transcript_HQ_CoA_transcript1267/f2p0/5481	4.693333333	2.13	1.69	-1.0006	0.00060479	0.0033154	DOWN	-0.47605	0.10621	0.23845		-1.4887	4.474E-08	2.1441E-06	DOWN
transcript_HQ_CoA_transcript12676/f2p0/2818	61.02666667	13.13333333	27.88333333	-2.0387	1.3241E-07	1.8479E-06	DOWN	0.93701	0.0012073	0.0074135		-1.1156	0.0064593	0.036866	DOWN
transcript_HQ_CoA_transcript12682/f2p0/2810	1.756666667	77.16666667	15.81666667	5.5976	8.0953E-34	1.3333E-31	UP	-2.4011	0.00011091	0.0010483	DOWN	3.1844	5.2766E-06	0.00012578	UP
transcript_HQ_CoA_transcript12694/f9p0/2799	17.85	33.52	19.47333333	1.0697	1.0556E-06	1.2347E-05	UP	-0.93519	7.7808E-05	0.00077931		0.12153	0.56175	0.74574	
transcript_HQ_CoA_transcript12695/f2p0/2822	19.28	38.93333333	13.96	1.1701	6.532E-08	9.6851E-07	UP	-1.6212	4.2056E-14	4.1222E-12	DOWN	-0.46412	0.077483	0.21483	
transcript_HQ_CoA_transcript12698/f2p0/2843	50.88666667	22.04	25.32333333	-1.0588	0.0032249	0.013798	DOWN	0.055902	0.89128	0.94163		-1.0143	0.00028431	0.0033092	DOWN
transcript_HQ_CoA_transcript12714/f3p0/2737	5.93	38.68	8.06	2.8887	7.1936E-09	1.2756E-07	UP	-2.4235	2.5763E-17	4.0708E-15	DOWN	0.44858	0.3606	0.57756	
transcript_HQ_CoA_transcript12717/f9p0/2767	10.09333333	41.56333333	14.45333333	2.2101	8.12E-16	3.6564E-14	UP	-1.6901	1.2509E-09	4.7665E-08	DOWN	0.50767	0.046483	0.15236	
transcript_HQ_CoA_transcript12732/f2p0/2816	11.67333333	25.66666667	16.17	1.2938	2.027E-10	4.685E-09	UP	-0.79922	0.19977	0.37207		0.47994	0.42778	0.6401	
transcript_HQ_CoA_transcript12751/f2p0/2818	39.86333333	137.9566667	72.39	1.9599	2.6237E-11	7.0229E-10	UP	-1.0765	0.0019657	0.010948	DOWN	0.87113	0.00097695	0.0088031	
transcript_HQ_CoA_transcript12762/f2p0/2833	7.27	23.9	5.883333333	1.863	0.00015648	0.0010384	UP	-2.1704	7.0399E-08	1.7971E-06	DOWN	-0.31826	0.42457	0.63688	
transcript_HQ_CoA_transcript12767/f2p0/2815	18.15	3.373333333	5.513333333	-2.2716	9.6143E-06	9.0743E-05	DOWN	0.57208	0.27326	0.46068		-1.7129	8.5216E-10	6.158E-08	DOWN
transcript_HQ_CoA_transcript12778/f5p0/2788	17.01	77.36	24.14333333	2.3488	7.67E-14	2.7822E-12	UP	-1.8353	4.0818E-08	1.1147E-06	DOWN	0.49988	0.026757	0.10253	
transcript_HQ_CoA_transcript1278/f2p0/5470	3.203333333	8.67	3.59	1.5971	5.3972E-10	1.1663E-08	UP	-1.4072	0.014535	0.053819		0.17422	0.7652	0.88323	
transcript_HQ_CoA_transcript12781/f3p0/2823	0.56	68.39333333	0.616666667	7.0835	5.9223E-09	1.0636E-07	UP	-6.9028	1.9553E-17	3.1437E-15	DOWN	0.16865	0.88955	0.9508	
transcript_HQ_CoA_transcript12785/f3p0/2818	0.583333333	25.34	2.136666667	5.667	1.0116E-17	5.3451E-16	UP	-3.7291	0.0047194	0.022398	DOWN	1.9195	0.075199	0.21009	
transcript_HQ_CoA_transcript12787/f3p0/2758	3.246666667	26.77333333	6.943333333	3.2084	3.5049E-15	1.5006E-13	UP	-2.0997	9.375E-07	1.8069E-05	DOWN	1.0959	0.006391	0.036635	UP
transcript_HQ_CoA_transcript12798/f2p0/2813	6.44	0.676666667	5.523333333	-3.0664	0.00018207	0.0011846	DOWN	2.8761	0.0038337	0.019002	UP	-0.20767	0.7524	0.87537	
transcript_HQ_CoA_transcript12802/f2p0/2871	32.83333333	10.58	7.456666667	-1.4606	9.6832E-07	1.1416E-05	DOWN	-0.66528	0.017994	0.063293		-2.1397	2.7586E-23	1.9046E-20	DOWN
transcript_HQ_CoA_transcript12811/f7p0/2788	155.1933333	56.78666667	150.1466667	-1.2993	1.7561E-08	2.9027E-07	DOWN	1.2549	4.0869E-08	1.1147E-06	UP	-0.056121	0.81063	0.91118	
transcript_HQ_CoA_transcript12830/f2p0/2879	0.63	3.516666667	1.593333333	2.5472	0.013724	0.045378	UP	-1.2265	0.22428	0.40299		1.3084	0.065561	0.19236	
transcript_HQ_CoA_transcript12833/f2p0/2819	2.28	9.703333333	6.99	2.2669	2.8941E-06	3.0637E-05	UP	-0.6322	0.091639	0.21413		1.6184	0.00055961	0.0056406	UP

transcript_HQ_CoA_transcript12842/f2p0/2817	23.15666667	9.963333333	17.35	-1.0636	1.0288E-07	1.458E-06	DOWN	0.65123	2.4147E-05	0.00028739		-0.42426	0.016546	0.072136	
transcript_HQ_CoA_transcript12848/f5p0/2785	0.65	17.87	2.936666667	4.9699	3.4378E-27	3.6541E-25	UP	-2.7671	3.809E-12	2.4411E-10	DOWN	2.1871	0.00031791	0.003602	UP
transcript_HQ_CoA_transcript12853/f2p0/2799	4.106666667	0.283333333	6.67	-3.7058	2.6389E-07	3.4608E-06	DOWN	4.4526	5.5556E-08	1.4736E-06	UP	0.72916	0.35276	0.57032	
transcript_HQ_CoA_transcript12854/f2p0/2873	7.896666667	0.89	7.776666667	-2.9774	3.9207E-09	7.3159E-08	DOWN	2.9529	2.4504E-12	1.6957E-10	UP	-0.03702	0.90209	0.95665	
transcript_HQ_CoA_transcript12857/f3p0/2795	11	3.65	12.24333333	-1.4412	9.9665E-08	1.4187E-06	DOWN	1.5904	1.3564E-07	3.2669E-06	UP	0.13752	0.65571	0.81179	
transcript_HQ_CoA_transcript12862/f3p0/2803	59.70333333	25.77	34.38666667	-1.0608	3.6204E-11	9.4151E-10	DOWN	0.26552	0.096037	0.22178		-0.80701	7.4634E-07	2.4463E-05	
transcript_HQ_CoA_transcript12863/f2p0/2804	1.956666667	8.786666667	9.816666667	2.3675	0.00010548	0.00073718	UP	-0.015519	0.97142	0.98502		2.3358	5.9709E-06	0.00013926	UP
transcript_HQ_CoA_transcript12868/f7p0/2819	13.61666667	53.14333333	9.7	2.0897	1.5734E-05	0.00013883	UP	-2.5941	2.2091E-22	7.7865E-20	DOWN	-0.51278	0.25679	0.46732	
transcript_HQ_CoA_transcript12874/f2p0/2812	5.696666667	1.556666667	1.34	-1.7423	0.00020151	0.0012944	DOWN	-0.34349	0.5248	0.69752		-2.0965	1.3887E-11	1.5082E-09	DOWN
transcript_HQ_CoA_transcript1288/f4p0/5436	2.35	5.796666667	4.99	1.4962	0.015274	0.049215	UP	-0.36764	0.551	0.71891		1.1121	0.056031	0.17327	
transcript_HQ_CoA_transcript129/f2p0/7639	0.1	1.066666667	0.11	3.5591	3.2802E-08	5.1876E-07	UP	-3.4203	1.0356E-15	1.309E-13	DOWN	0.1321	0.89491	0.95352	
transcript_HQ_CoA_transcript12934/f2p0/2802	4.453333333	46.73666667	11.22666667	3.5367	5.5735E-36	9.7976E-34	UP	-2.2074	1.0017E-17	1.716E-15	DOWN	1.3175	0.00030856	0.0035243	UP
transcript_HQ_CoA_transcript12936/f2p0/2791	0.966666667	2.166666667	1.636666667	1.3243	0.0057651	0.022273	UP	-0.54423	0.24658	0.42968		0.76785	0.19539	0.39462	
transcript_HQ_CoA_transcript12940/f8p0/2737	41.47333333	11.42333333	22.42666667	-1.7156	9.5048E-17	4.6837E-15	DOWN	0.82777	0.0020789	0.011456		-0.89824	0.00043452	0.0046208	
transcript_HQ_CoA_transcript12972/f2p0/2795	3.236666667	30.23333333	12.73	3.3884	7.7498E-14	2.8056E-12	UP	-1.4056	0.0010511	0.0066548	DOWN	1.9697	1.699E-17	5.2855E-15	UP
transcript_HQ_CoA_transcript12973/f2p0/2788	5.18	1.4	3.246666667	-1.7033	0.001001	0.0051579	DOWN	1.0479	0.049451	0.1353		-0.66802	0.028404	0.10734	
transcript_HQ_CoA_transcript12980/f5p0/2761	20.40666667	9.006666667	17.25333333	-1.0221	1.7056E-07	2.3201E-06	DOWN	0.78496	5.5348E-07	1.1515E-05		-0.24894	0.14705	0.32904	
transcript_HQ_CoA_transcript12982/f3p0/2805	13.71333333	5.463333333	9.94	-1.1738	9.1855E-06	8.7236E-05	DOWN	0.71029	0.011616	0.045176		-0.47506	0.11821	0.28624	
transcript_HQ_CoA_transcript13000/f4p0/2788	11.99333333	38.35	38.12333333	1.8516	1.1402E-09	2.3422E-08	UP	-0.1592	0.50505	0.68182		1.6787	2.1584E-10	1.7686E-08	UP
transcript_HQ_CoA_transcript13008/f2p0/2784	2.746666667	9.853333333	5.426666667	2.0314	8.1341E-06	7.8391E-05	UP	-1.0194	0.0010653	0.0067286	DOWN	0.99707	0.017505	0.075318	
transcript_HQ_CoA_transcript13009/f2p0/2807	28.05666667	11.67666667	10.45	-1.1182	3.4115E-06	3.5579E-05	DOWN	-0.29897	0.26506	0.45112		-1.4284	3.3198E-11	3.2938E-09	DOWN
transcript_HQ_CoA_transcript1301/f4p0/5401	6.326666667	11.91	6.876666667	1.0765	4.4299E-05	0.0003458	UP	-0.93594	0.0006081	0.0042639		0.12712	0.6929	0.83689	
transcript_HQ_CoA_transcript13012/f2p0/2744	2.45	10.11	5.456666667	2.1884	1.7601E-13	6.1762E-12	UP	-1.0297	1.3469E-05	0.00017484	DOWN	1.1481	0.00065908	0.0064348	UP
transcript_HQ_CoA_transcript13017/f2p0/2786	5.143333333	10.78666667	2.7	1.227	3.4067E-08	5.3784E-07	UP	-2.1454	1.0811E-24	5.0807E-22	DOWN	-0.93169	0.0015826	0.012746	
transcript_HQ_CoA_transcript13021/f4p0/2786	7.173333333	25.84333333	8.46	2.0175	5.8407E-06	5.8223E-05	UP	-1.7717	4.1725E-05	0.00046351	DOWN	0.23093	0.47328	0.679	
transcript_HQ_CoA_transcript13039/f2p0/2781	2.2	13.52333333	14.42333333	2.7703	1.5958E-08	2.6498E-07	UP	-0.056499	0.88968	0.9408		2.7026	1.0758E-16	3.1848E-14	UP
transcript_HQ_CoA_transcript13040/f2p0/2762	5.996666667	20.75	3.426666667	1.9455	0.0014627	0.007108	UP	-2.713	3.8819E-09	1.3324E-07	DOWN	-0.77994	0.24843	0.45834	
transcript_HQ_CoA_transcript13042/f2p0/2780	7.45	3.233333333	6.09	-1.0367	0.0023303	0.010543	DOWN	0.76304	0.040249	0.11555		-0.2889	0.44359	0.65429	
transcript_HQ_CoA_transcript13055/f2p0/2790	2.383333333	0.84	1.69	-1.3659	0.0063383	0.024116	DOWN	0.87142	0.059508	0.15624		-0.50591	0.22896	0.43632	
transcript_HQ_CoA_transcript13059/f7p0/2772	19.77333333	39.48666667	34.9	1.1518	1.8495E-08	3.0407E-07	UP	-0.31851	0.14479	0.29716		0.81976	0.0001395	0.0018649	
transcript_HQ_CoA_transcript13064/f2p0/2782	2.846666667	0.803333333	1.396666667	-1.6669	9.9459E-05	0.00070015	DOWN	0.65598	0.17943	0.34568		-1.023	0.012074	0.057638	
transcript_HQ_CoA_transcript13067/f2p0/2841	7.556666667	17.24	14.78	1.3534	1.7396E-07	2.3628E-06	UP	-0.36852	0.05922	0.15571		0.97252	0.00051295	0.0052712	
transcript_HQ_CoA_transcript13069/f4p0/2777	15.87333333	2.703333333	5.516666667	-2.396	7.2614E-06	7.0802E-05	DOWN	0.88717	0.067176	0.17078		-1.5175	0.0076503	0.041447	DOWN
transcript_HQ_CoA_transcript13085/f2p0/2794	28.96666667	8.896666667	10.18666667	-1.5436	6.8921E-06	6.7561E-05	DOWN	0.037504	0.88568	0.93884		-1.5166	1.0547E-06	3.3021E-05	DOWN
transcript_HQ_CoA_transcript13088/f5p0/2701	94.13666667	30.47333333	35.42	-1.4752	6.4337E-06	6.3544E-05	DOWN	0.05792	0.79188	0.88524		-1.4274	1.1976E-06	3.6883E-05	DOWN
transcript_HQ_CoA_transcript13116/f2p0/2769	4.216666667	34.42	10.29	3.1916	4.7666E-18	2.5706E-16	UP	-1.8969	2.0605E-06	3.5296E-05	DOWN	1.2815	0.00027952	0.003272	UP
transcript_HQ_CoA_transcript13118/f2p0/2810	2.886666667	5.953333333	3.9	1.2117	0.00054394	0.0030309	UP	-0.7616	0.0071769	0.030944		0.4359	0.25532	0.46584	
transcript_HQ_CoA_transcript13121/f6p0/2748	43.46	91.29333333	182.65	1.2288	3.8122E-05	0.00030329	UP	0.8837	0.18426	0.35214		2.099	0.017768	0.076168	
transcript_HQ_CoA_transcript13123/f2p0/2770	1.183333333	10.05666667	3.426666667	3.24	6.0167E-08	8.9575E-07	UP	-1.6884	0.001833	0.010366	DOWN	1.5385	0.0014175	0.011794	UP
transcript_HQ_CoA_transcript13133/f2p0/2791	2.146666667	16.75	3.71	3.1253	4.2211E-09	7.8187E-08	UP	-2.3133	0.00024897	0.0020472	DOWN	0.79789	0.15171	0.33519	
transcript_HQ_CoA_transcript13152/f5p0/2772	82.33666667	34.53333333	68.43	-1.0925	8.9957E-05	0.00064317	DOWN	0.82298	0.0011248	0.0070269		-0.28087	0.14818	0.3308	
transcript_HQ_CoA_transcript13157/f4p0/2777	0.626666667	20.84333333	7.62	5.2302	0.00050307	0.0028369	UP	-1.6043	0.00039502	0.0030005	DOWN	3.6051	0.00024296	0.0029109	UP
transcript_HQ_CoA_transcript13160/f4p0/2765	5.88	12.13	6.25	1.2244	0.0014178	0.0069329	UP	-1.121	0.006451	0.028526	DOWN	0.087717	0.81567	0.91418	
transcript_HQ_CoA_transcript13176/f2p0/2783	1.66	11.01333333	3.2	2.8955	3.2094E-08	5.0844E-07	UP	-1.9366	0.00072023	0.0049086	DOWN	0.94484	0.11049	0.27454	
transcript_HQ_CoA_transcript13202/f2p0/2768	5.643333333	0	5.593333333	-9.6347	0.013671	0.045213	DOWN	9.6725	2.8439E-14	2.8641E-12	UP	0.020957	0.99065	0.99628	
transcript_HQ_CoA_transcript13203/f3p0/2767	6.73	2.55	7.49	-1.2353	0.0077805	0.02862	DOWN	1.3977	0.0015244	0.0089267	UP	0.1515	0.66557	0.81776	
transcript_HQ_CoA_transcript1321/f2p0/5471	2.543333333	1.076666667	2.093333333	-1.0787	0.0019141	0.008936	DOWN	0.80958	0.039648	0.11417		-0.28235	0.47174	0.67789	
transcript_HQ_CoA_transcript13210/f2p0/2768	10.19	21.90666667	39.96666667	1.2679	0.00015631	0.0010376	UP	0.74802	0.25809	0.44264		2.0018	0.0013476	0.011341	UP
transcript_HQ_CoA_transcript13224/f3p0/2766	25.49	11.10333333	16.10333333	-1.0324	6.1552E-05	0.00046176	DOWN	0.38094	0.030992	0.095471		-0.66566	0.005429	0.032448	
transcript_HQ_CoA_transcript13233/f2p0/2780	9.16	17.81333333	11.21333333	1.1155	0.0011503	0.0057836	UP	-0.82357	0.0061301	0.027512		0.27968	0.24202	0.45089	
transcript_HQ_CoA_transcript13237/f2p0/2765	0	3.46	0.416666667	9.1465	2.3924E-11	6.4893E-10	UP	-3.2261	0.0020423	0.011292	DOWN	5.9056	0.026483	0.10178	
transcript_HQ_CoA_transcript13238/f2p0/2765	6.68	2.323333333	6.736666667	-1.3715	3.2585E-05	0.00026571	DOWN	1.3796	3.4884E-06	5.5358E-05	UP	-0.0040689	0.98984	0.99608	
transcript_HQ_CoA_transcript13242/f3p0/2771	15.51666667	5.8	20.19333333	-1.2706	4.386E-07	5.4959E-06	DOWN	1.6558	5.2365E-08	1.3991E-06	UP	0.3743	0.20681	0.40909	
transcript_HQ_CoA_transcript13249/f2p0/2754	0.39	1.836666667	0.443333333	2.4247	0.00034309	0.0020465	UP	-2.2602	0.0010408	0.0065981	DOWN	0.15451	0.85595	0.93445	
transcript_HQ_CoA_transcript13269/f2p0/2780	1.48	10.94	4.07	3.0614	3.5525E-08	5.5701E-07	UP	-1.5987	0.00056115	0.0039928	DOWN	1.4519	0.0005336	0.0054413	UP
transcript_HQ_CoA_transcript13277/f2p0/2886	16.85	32.74	15.49333333	1.0976	0.0014917	0.0072319	UP	-1.2174	0.0045621	0.021816	DOWN	-0.13221	0.76208	0.88186	
transcript_HQ_CoA_transcript13283/f2p0/2767	2.07	0.286666667	1.293333333	-2.6947	0.00018129	0.0011824	DOWN	2.0118	0.010279	0.040904	UP	-0.69184	0.15657	0.3427	
transcript_HQ_CoA_transcript13293/f2p0/2769	8.13	24.48	7.123333333	1.7666	5.7962E-05	0.00043787	UP	-1.95	6.2391E-06	9.0616E-05	DOWN	-0.19654	0.46199	0.67013	
transcript_HQ_CoA_transcript13308/f2p0/2777	41.61333333	14.68333333	15.82666667	-1.3492	2.0013E-12	6.1494E-11	DOWN	-0.041438	0.75938	0.86489		-1.402	1.8563E-14	3.7857E-12	DOWN
transcript_HQ_CoA_transcript13310/f3p0/2762	56.97333333	18.72666667	23.17666667	-1.4312	1.5821E-06	1.7877E-05	DOWN	0.14443	0.61957	0.77179		-1.3002	8.4516E-16	2.1849E-13	DOWN
transcript_HQ_CoA_transcript13311/f2p0/2709	14.86666667	6.513333333	7.993333333	-1.0403	0.00091805	0.0047817	DOWN	0.16104	0.54063	0.71085		-0.89199	0.00060736	0.0060247	
transcript_HQ_CoA_transcript13341/f8p0/2752	12.69666667	30.77333333	16.88333333	1.4341	0.0037544	0.015721	UP	-1.0243	0.018316	0.064153		0.39845	0.25345	0.4639	
transcript_HQ_CoA_transcript13343/f6p0/2741	31.19333333	13.99333333	25.31333333	-1.0086	1.6307E-05	0.00014333	DOWN	0.71778	0.00072163	0.0049134		-0.30266	0.16635	0.35611	
transcript_HQ_CoA_transcript13347/f2p0/2595	2.996666667	6.263333333	5.673333333	1.236	0.0011496	0.0057819	UP	-0.3006	0.4174	0.607		0.92222	0.0069841	0.038835	
transcript_HQ_CoA_transcript13351/f4p0/2737	0	13.44333333	0.726666667	11.096	4.7607E-18	2.5706E-16	UP	-4.3503	3.8731E-05	0.00043499	DOWN	6.7343	0.0059349	0.034804	UP
transcript_HQ_CoA_transcript13370/f2p0/2765	12.32	2.886666667	3.443333333	-1.9505	8.9484E-07	1.0623E-05	DOWN	0.14256	0.7825	0.87866		-1.8227	0.00001606	0.00031427	DOWN
transcript_HQ_CoA_transcript13372/f3p0/2761	10.61333333	3.713333333	11.7	-1.3613	1.9562E-05	0.00016838	DOWN	1.5044	0.00020815	0.0017804	UP	0.13129	0.7074	0.84625	
transcript_HQ_CoA_transcript13380/f2p0/2742	5.7	2.1	3.91	-1.2738	0.01289	0.043159	DOWN	0.7491	0.003832	0.018998		-0.53929	0.25255	0.46296	
transcript_HQ_CoA_transcript13382/f7p0/2742	11.29333333	36.30666667	20.35333333	1.855	2.5214E-11	6.7989E-10	UP	-0.99687	0.00027446	0.0022171		0.84413	0.0047808	0.029566	
transcript_HQ_CoA_transcript13384/f5p0/2737	8.503333333	21.92	11.41333333	1.5287	2.5839E-11	6.9469E-10	UP	-1.0877	3.5499E-08	9.7844E-07	DOWN	0.42835	0.099136	0.25546	
transcript_HQ_CoA_transcript13388/f2p0/2764	3.723333333	14.42	3.976666667	2.0942	1.4205E-12	4.4469E-11	UP	-1.9932	5.2149E-13	4.2671E-11	DOWN	0.092018	0.80504	0.90785	
transcript_HQ_CoA_transcript13403/f2p0/2764	8.086666667	3.083333333	11.66333333	-1.2572	0.0074245	0.027486	DOWN	1.8013	0.001885	0.010582	UP	0.53407	0.3512	0.56881	
transcript_HQ_CoA_transcript13408/f2p0/2769	15.07	4.356666667	11.55	-1.63	4.4814E-07	5.6001E-06	DOWN	1.2485	3.296E-07	7.2351E-06	UP	-0.39279	0.17769	0.37156	
transcript_HQ_CoA_transcript1341/f3p0/5394	11.5	4.526666667	5.44	-1.2126	0.00953	0.033844	DOWN	0.1329	0.75482	0.86179		-1.0911	0.00049006	0.0050877	DOWN
transcript_HQ_CoA_transcript13422/f2p0/2778	9.936666667	21.95333333	15.48333333	1.3166	0.00036985	0.0021861	UP	-0.6529	0.0083976	0.035014		0.64932	0.070488	0.20159	
transcript_HQ_CoA_transcript13424/f2p0/2752	13.37666667	4.556666667	4.336666667	-1.4027	0.00053315	0.0029817	DOWN	-0.21247	0.47285	0.65638		-1.627	0.00001583	0.00031044	DOWN
transcript_HQ_CoA_transcript13425/f2p0/2740	5.346666667	13.67666667	7.11	1.5237	1.0291E-05	0.00009614	UP	-1.0975	0.0010981	0.0068881	DOWN	0.41438	0.19034	0.38811	
transcript_HQ_CoA_transcript13432/f3p0/2744	1.363333333	20.47	11.14333333	4.1083	4.1377E-10	9.1249E-09	UP	-1.0368	0.0064706	0.028592	DOWN	3.0532	5.945E-07	2.0096E-05	UP
transcript_HQ_CoA_transcript13438/f6p0/2737	20.64333333	7.913333333	12.75666667	-1.2147	0.0001512	0.0010088	DOWN	0.54681	0.013827	0.051721		-0.68167	0.03241	0.11787	

transcript_HQ_CoA_transcript13439/f9p0/2763	53.05333333	94.86666667	46.51666667	1.0003	3.9507E-05	0.00031254	UP	-1.1799	1.1562E-06	0.00002145	DOWN	-0.19259	0.3519	0.56939	
transcript_HQ_CoA_transcript13444/f2p0/2758	50.99	20.54666667	45.07	-1.1561	3.6515E-10	8.171E-09	DOWN	0.98874	1.6335E-05	0.00020549		-0.17821	0.43053	0.64246	
transcript_HQ_CoA_transcript13453/f2p0/2753	5.313333333	10.47333333	5	1.1174	0.0023508	0.010622	UP	-1.2061	0.00048337	0.0035567	DOWN	-0.10102	0.72038	0.85558	
transcript_HQ_CoA_transcript13458/f2p0/2738	4.48	0.413333333	7.73	-3.3079	3.8059E-05	0.00030291	DOWN	4.0904	1.4491E-13	1.2957E-11	UP	0.77268	0.236	0.44474	
transcript_HQ_CoA_transcript13475/f2p0/2737	11.05666667	25.04	21.42333333	1.3258	0.0041105	0.016895	UP	-0.3373	0.58832	0.74923		0.97296	0.063994	0.18909	
transcript_HQ_CoA_transcript13477/f2p0/2807	20.35	5.366666667	4.046666667	-1.7632	1.8048E-14	7.1109E-13	DOWN	-0.56661	0.036967	0.10839		-2.3417	2.4852E-27	3.8014E-24	DOWN
transcript_HQ_CoA_transcript13479/f4p0/2719	1.713333333	12.54666667	1.246666667	3.0081	0.0033823	0.014394	UP	-3.4284	0.00012188	0.0011305	DOWN	-0.43632	0.6421	0.80117	
transcript_HQ_CoA_transcript13486/f6p0/2696	11.08333333	27.35	17.2	1.4714	1.5402E-06	1.7435E-05	UP	-0.82202	0.0019885	0.011054		0.63551	0.0034696	0.023208	
transcript_HQ_CoA_transcript13497/f3p0/2739	4.84	11.20333333	6.913333333	1.3835	0.00075447	0.0040178	UP	-0.84662	0.03911	0.11303		0.52225	0.28262	0.4967	
transcript_HQ_CoA_transcript13510/f2p0/2782	3.1	18.39	5.79	2.7658	2.8783E-10	6.5206E-09	UP	-1.8411	3.5807E-07	7.7762E-06	DOWN	0.90939	0.010538	0.052188	
transcript_HQ_CoA_transcript13512/f2p0/2740	9.716666667	1.753333333	3.976666667	-2.3338	7.1139E-05	0.00052336	DOWN	1.0355	0.067495	0.17132		-1.3057	0.022667	0.090647	
transcript_HQ_CoA_transcript13530/f3p0/2756	23.15666667	49.88666667	40.93666667	1.2507	2.4643E-05	0.00020619	UP	-0.42882	0.081958	0.1974		0.81081	0.0011586	0.010043	
transcript_HQ_CoA_transcript13534/f2p0/2782	12.45	3.923333333	12.26333333	-1.5137	0.011136	0.038462	DOWN	1.4704	0.00034434	0.0026641	UP	-0.051693	0.91474	0.96235	
transcript_HQ_CoA_transcript13538/f2p0/2721	5.696666667	2.12	6.33	-1.2851	0.002074	0.0095555	DOWN	1.4265	0.00039093	0.0029744	UP	0.13201	0.73909	0.86717	
transcript_HQ_CoA_transcript13543/f2p0/2739	0.163333333	3.686666667	0.4	4.6774	7.3466E-09	1.3009E-07	UP	-3.3768	0.00048327	0.0035567	DOWN	1.2907	0.30398	0.52049	
transcript_HQ_CoA_transcript13559/f3p0/2764	8.406666667	3.16	6.35	-1.271	5.2592E-05	0.00040212	DOWN	0.86034	0.0017116	0.0098158		-0.42197	0.10828	0.27045	
transcript_HQ_CoA_transcript13580/f5p0/2730	13.87666667	36.45	13.38666667	1.5589	5.3468E-06	0.00005365	UP	-1.6052	1.9959E-06	3.4382E-05	DOWN	-0.059561	0.75432	0.87653	
transcript_HQ_CoA_transcript13591/f2p0/2737	25.46	6.293333333	9.916666667	-1.8681	5.5512E-14	2.0586E-12	DOWN	0.5062	0.030293	0.093776		-1.3729	2.3548E-09	1.5382E-07	DOWN
transcript_HQ_CoA_transcript13595/f2p0/2733	2.68	1	1.956666667	-1.2802	0.0044439	0.017974	DOWN	0.83424	0.052028	0.14088		-0.45689	0.16986	0.36069	
transcript_HQ_CoA_transcript13612/f31p0/2638	12.39	36.92333333	25.25	1.745	1.7669E-11	4.843E-10	UP	-0.69992	0.004243	0.020667		1.0309	0.00015652	0.0020477	UP
transcript_HQ_CoA_transcript13630/f3p0/2729	4.403333333	9.876666667	4.553333333	1.331	9.1362E-06	8.6858E-05	UP	-1.2727	8.6557E-06	0.00011983	DOWN	0.045622	0.87265	0.9432	
transcript_HQ_CoA_transcript13633/f7p0/2644	75.73333333	29.42333333	39.32666667	-1.231	0.004608	0.018536	DOWN	0.28383	0.541	0.71098		-0.95859	0.0016869	0.013416	
transcript_HQ_CoA_transcript13644/f2p0/2738	22.04666667	9.45	15.53	-1.0504	0.00051841	0.0029144	DOWN	0.54433	0.4364	0.62525		-0.52427	0.40163	0.617	
transcript_HQ_CoA_transcript13665/f2p0/2733	1.606666667	13.06333333	5.973333333	3.2227	3.8097E-08	5.9125E-07	UP	-1.2708	0.078991	0.19255		1.932	0.024984	0.097342	
transcript_HQ_CoA_transcript13678/f3p0/2756	5.686666667	1.136666667	3.323333333	-2.2024	0.00009978	0.00070215	DOWN	1.4219	0.0048358	0.022832	UP	-0.79118	0.041141	0.13991	
transcript_HQ_CoA_transcript13683/f4p0/2718	0.7	2.33	1.393333333	1.8806	0.0041717	0.0171	UP	-0.89739	0.036368	0.10719		0.97324	0.16994	0.36077	
transcript_HQ_CoA_transcript13687/f2p0/2732	0	18.59666667	0.816666667	11.536	3.196E-20	2.0218E-18	UP	-4.6126	0.0030528	0.015687	DOWN	6.91	0.0045415	0.028564	UP
transcript_HQ_CoA_transcript13702/f18p0/2653	83.37	32.25	78.87666667	-1.2011	8.2654E-05	0.00059632	DOWN	1.1287	0.00013697	0.0012465	UP	-0.084958	0.55359	0.74014	
transcript_HQ_CoA_transcript13707/f6p0/2714	34.65666667	62.85666667	43.83333333	1.0162	1.0529E-11	2.9752E-10	UP	-0.67185	2.1615E-08	6.2292E-07		0.3323	0.012993	0.060747	
transcript_HQ_CoA_transcript13711/f2p0/2722	0.956666667	8.193333333	2.113333333	3.2769	1.6822E-10	3.958E-09	UP	-2.112	0.00012225	0.0011334	DOWN	1.1492	0.046312	0.15199	
transcript_HQ_CoA_transcript13724/f2p0/2737	6.203333333	2.093333333	2.433333333	-1.4134	0.0001662	0.0010935	DOWN	0.070105	0.82863	0.9065		-1.3566	0.00024747	0.0029553	DOWN
transcript_HQ_CoA_transcript13725/f2p0/2760	6.92	17.97666667	7.516666667	1.5347	3.2611E-05	0.0002658	UP	-1.4158	4.7295E-06	7.1702E-05	DOWN	0.10756	0.71212	0.84959	
transcript_HQ_CoA_transcript13735/f3p0/2719	15.39	5.983333333	7.816666667	-1.2275	0.014781	0.047988	DOWN	0.24333	0.35691	0.54885		-0.99324	0.040556	0.13862	
transcript_HQ_CoA_transcript13745/f2p0/2734	3.88	15.06	11.67333333	2.1107	3.5103E-08	5.5182E-07	UP	-0.51487	0.011103	0.043578		1.5839	1.0464E-05	0.00021926	UP
transcript_HQ_CoA_transcript13754/f2p0/2722	35.63333333	12.00666667	21.54666667	-1.4424	0.00077968	0.00414	DOWN	0.70937	0.09331	0.2171		-0.74252	0.0042078	0.026836	
transcript_HQ_CoA_transcript13768/f2p0/2732	21.34	5.333333333	16.99	-1.8625	1.6968E-10	3.9822E-09	DOWN	1.5281	1.0374E-06	1.9604E-05	UP	-0.34425	0.21685	0.42203	
transcript_HQ_CoA_transcript1377/f2p0/5366	1.573333333	3.62	3.18	1.3717	0.0041236	0.016945	UP	-0.34268	0.46991	0.6536		1.0158	0.047033	0.15361	
transcript_HQ_CoA_transcript13770/f2p0/2720	11.1	2.086666667	16.77	-2.2673	9.3192E-10	1.9471E-08	DOWN	2.8455	1.5661E-12	1.1622E-10	UP	0.56827	0.168	0.35836	
transcript_HQ_CoA_transcript13793/f5p0/2722	112.6833333	44.91333333	75.75	-1.182	3.2332E-06	3.3951E-05	DOWN	0.60889	0.000181	0.0015828		-0.58453	0.0075307	0.040992	
transcript_HQ_CoA_transcript13794/f2p0/2718	11.02	2.163333333	2.583333333	-2.2073	2.0517E-12	6.2934E-11	DOWN	0.12707	0.71644	0.83769		-2.0938	7.3986E-12	8.7267E-10	DOWN
transcript_HQ_CoA_transcript13805/f2p0/2750	17.9	2.993333333	3.426666667	-2.4333	1.3543E-18	7.618E-17	DOWN	0.042678	0.90849	0.9511		-2.4	3.0225E-14	5.9654E-12	DOWN
transcript_HQ_CoA_transcript13808/f7p0/2708	35.95666667	67.67333333	42.94	1.0822	0.00022764	0.0014381	UP	-0.81642	0.0035507	0.01784		0.2527	0.092237	0.2428	
transcript_HQ_CoA_transcript13828/f2p0/2703	0.996666667	0.276666667	0.976666667	-1.6936	0.011874	0.040471	DOWN	1.6814	0.014903	0.054851		-0.023675	0.96776	0.98625	
transcript_HQ_CoA_transcript13837/f10p0/2702	17.26333333	42.01333333	23.02	1.4472	9.2916E-07	1.1002E-05	UP	-1.0126	3.7613E-07	8.1394E-06	DOWN	0.42138	0.15644	0.3425	
transcript_HQ_CoA_transcript13848/f2p0/2711	0.31	18.89333333	3.333333333	6.1242	5.8176E-15	2.4338E-13	UP	-2.6391	0.00022099	0.0018601	DOWN	3.4673	0.0002088	0.0025773	UP
transcript_HQ_CoA_transcript13855/f2p0/2712	1.503333333	11.75666667	3.506666667	3.1087	1.7907E-11	4.8935E-10	UP	-1.8898	9.2433E-06	0.00012697	DOWN	1.2055	0.00066776	0.0065023	UP
transcript_HQ_CoA_transcript13885/f2p0/2666	7.506666667	17.54666667	9.796666667	1.3895	0.000113	0.00078285	UP	-0.99988	0.0026408	0.013913		0.37786	0.19673	0.39638	
transcript_HQ_CoA_transcript13891/f2p0/2717	0.993333333	29.26333333	3.91	5.0033	1.0987E-17	5.772E-16	UP	-3.0237	6.1199E-07	1.2533E-05	DOWN	1.9663	0.002256	0.016765	UP
transcript_HQ_CoA_transcript13908/f2p0/2710	3.246666667	10.61333333	1.663333333	1.8613	0.0013845	0.0067893	UP	-2.8271	9.0734E-07	1.7599E-05	DOWN	-0.97681	0.017913	0.076697	
transcript_HQ_CoA_transcript13910/f2p0/2725	1.433333333	35.94	4.593333333	4.8095	2.3428E-21	1.5982E-19	UP	-3.1224	4.4251E-12	2.8065E-10	DOWN	1.6758	0.00020135	0.0024984	UP
transcript_HQ_CoA_transcript13914/f2p0/2709	9.073333333	16.28	10.07333333	1.014	0.0023583	0.010643	UP	-0.84831	0.00114	0.0071071		0.15169	0.58598	0.76314	
transcript_HQ_CoA_transcript13917/f2p0/2713	6.056666667	1.826666667	4.186666667	-1.5902	3.7951E-05	0.00030245	DOWN	1.0659	0.0049452	0.023229	UP	-0.53482	0.089491	0.23764	
transcript_HQ_CoA_transcript13930/f3p0/2717	37.15	5.206666667	8.683333333	-2.664	1.6232E-10	3.829E-09	DOWN	0.56029	0.20885	0.38414		-2.1141	1.5536E-15	3.8535E-13	DOWN
transcript_HQ_CoA_transcript13937/f3p0/2690	2.12	0.08	3.376666667	-4.5178	0.00079009	0.0041916	DOWN	5.2675	0.00021775	0.0018375	UP	0.72701	0.60413	0.77601	
transcript_HQ_CoA_transcript13940/f2p0/2712	0.916666667	2.996666667	1.64	1.8431	0.014451	0.047186	UP	-1.001	0.086479	0.20532		0.83007	0.19085	0.38893	
transcript_HQ_CoA_transcript13949/f2p0/2726	3.683333333	9.33	9.87	1.4684	0.0132	0.043956	UP	-0.0096942	0.99054	0.99501		1.4481	0.07258	0.2053	
transcript_HQ_CoA_transcript13953/f2p0/2706	18.31	49.70666667	2.523333333	1.6105	3.6444E-05	0.00029211	UP	-4.4578	3.2061E-74	5.8764E-70	DOWN	-2.864	6.2885E-12	7.695E-10	DOWN
transcript_HQ_CoA_transcript13991/f2p0/2694	11.35666667	3.366666667	5.35	-1.6072	4.7696E-06	4.8282E-05	DOWN	0.5125	0.042248	0.12		-1.1067	0.00071887	0.0068943	DOWN
transcript_HQ_CoA_transcript13997/f3p0/2693	1.623333333	5.243333333	3.816666667	1.8247	3.9826E-05	0.00031465	UP	-0.5898	0.14869	0.30288		1.2226	0.00099132	0.0089183	UP
transcript_HQ_CoA_transcript13998/f3p0/2692	18.22666667	533.3833333	40.18	5.0327	3.8002E-24	3.1295E-22	UP	-3.8539	7.2046E-24	3.2208E-21	DOWN	1.162	0.016253	0.071182	
transcript_HQ_CoA_transcript140/f3p0/7642	8.01	2.606666667	3.67	-1.4844	3.3021E-05	0.00026834	DOWN	0.35713	0.22665	0.40589		-1.137	0.00014635	0.0019395	DOWN
transcript_HQ_CoA_transcript14003/f11p0/2687	27.32666667	7.463333333	35.79666667	-1.6968	0.00019444	0.0012543	DOWN	2.1174	1.2125E-08	3.735E-07	UP	0.40425	0.24316	0.45229	
transcript_HQ_CoA_transcript14007/f2p0/2704	2.963333333	0.796666667	3.013333333	-1.7248	0.0010694	0.0054354	DOWN	1.7496	0.0011697	0.0072407	UP	0.012444	0.97457	0.9894	
transcript_HQ_CoA_transcript14008/f4p0/2684	0.06	350.9433333	22.67	12.58	4.6951E-71	3.3014E-68	UP	-4.0967	0.0047617	0.022535	DOWN	8.4639	7.1882E-06	0.00016149	UP
transcript_HQ_CoA_transcript14029/f2p0/2692	23	4.153333333	33.35	-2.3129	0.00020974	0.001341	DOWN	2.8404	2.4929E-13	2.1056E-11	UP	0.51963	0.31165	0.5284	
transcript_HQ_CoA_transcript14043/f2p0/2702	20.27333333	56.64333333	23.46	1.6399	6.5803E-07	7.9722E-06	UP	-1.4233	1.0619E-05	0.0001432	DOWN	0.20379	0.19689	0.3966	
transcript_HQ_CoA_transcript14057/f11p0/2679	21.75333333	42.65333333	20.53666667	1.1264	1.0075E-07	0.00000143	UP	-1.1942	1.8537E-06	3.2174E-05	DOWN	-0.080843	0.76171	0.88159	
transcript_HQ_CoA_transcript14066/f2p0/2693	17.65	33.30333333	21.06	1.0755	0.00049609	0.0028044	UP	-0.80852	0.02529	0.08213		0.25488	0.45153	0.66164	
transcript_HQ_CoA_transcript14068/f5p0/2686	48.59	12.52	27	-1.8058	0.00070676	0.0037898	DOWN	0.99952	0.075648	0.18657		-0.81808	0.1138	0.2793	
transcript_HQ_CoA_transcript14075/f2p0/2695	2.86	0.743333333	1.576666667	-1.7921	6.9269E-05	0.00051167	DOWN	0.92162	0.053777	0.14447		-0.88184	0.042727	0.14343	
transcript_HQ_CoA_transcript14077/f4p0/2666	16.49666667	7.293333333	13.71666667	-1.0125	0.00040565	0.0023679	DOWN	0.74973	0.011512	0.044835		-0.27478	0.33989	0.55799	
transcript_HQ_CoA_transcript14087/f2p0/2694	51.31333333	19.51	36.89666667	-1.2497	2.6738E-10	6.0875E-09	DOWN	0.76966	0.0010556	0.0066787		-0.49123	0.034456	0.12312	
transcript_HQ_CoA_transcript14090/f4p0/2614	37.79666667	15.21	28.66333333	-1.1605	2.7695E-05	0.00022921	DOWN	0.76085	1.9782E-05	0.0002414		-0.41	0.097427	0.25237	
transcript_HQ_CoA_transcript14101/f3p0/2723	0.62	1.35	0.673333333	1.2906	0.014648	0.047641	UP	-1.1713	0.017444	0.061809		0.10431	0.8686	0.94146	
transcript_HQ_CoA_transcript14103/f37p0/2517	84.70666667	154.2266667	106.44	1.0234	2.1485E-08	3.4976E-07	UP	-0.68518	0.00012833	0.0011808		0.32587	0.029253	0.1098	
transcript_HQ_CoA_transcript14105/f5p0/2689	113.51	8.25	29.53666667	-3.6553	4.8412E-15	2.0393E-13	DOWN	1.7062	0.00012415	0.0011487	UP	-1.9566	2.1141E-07	8.3449E-06	DOWN
transcript_HQ_CoA_transcript14120/f2p0/2687	11.95	3.053333333	15.93333333	-1.801	4.1517E-06	4.2451E-05	DOWN	2.2188	8.8104E-18	1.5235E-15	UP	0.40507	0.22164	0.42797	
transcript_HQ_CoA_transcript14124/f7p0/2662	13.11333333	3.926666667	15.83333333	-1.5856	0.00007794	0.00056701	DOWN	1.8588	4.5943E-06	7.0057E-05	UP	0.26253	0.33806	0.55602	
transcript_HQ_CoA_transcript14128/f20p0/2637	0.013333333	29.84333333	0.836666667	11.201	7.2188E-19	4.1242E-17	UP	-5.2874	0.0004314	0.0032275	DOWN	5.8991	0.0068812	0.038461	UP

transcript_HQ_CoA_transcript14129/f3p0/2684	8.42	0.873333333	0.176666667	-3.0683	2.2024E-05	0.0001871	DOWN	-2.501	0.016469	0.059245		-5.5859	2.5314E-20	1.1063E-17	DOWN
transcript_HQ_CoA_transcript14135/f3p0/2688	9.963333333	4.053333333	5.396666667	-1.1356	0.0014958	0.0072483	DOWN	0.25595	0.46419	0.64903		-0.89224	6.3429E-05	0.00098248	
transcript_HQ_CoA_transcript14137/f3p0/2692	18.55333333	82.21	30.30666667	2.3141	4.4629E-20	2.7942E-18	UP	-1.6032	4.3183E-11	2.2359E-09	DOWN	0.69812	0.00011764	0.0016296	
transcript_HQ_CoA_transcript14138/f2p0/2702	4.236666667	10.22666667	3.516666667	1.4397	0.0022081	0.010069	UP	-1.6889	0.00079642	0.0053217	DOWN	-0.26259	0.54993	0.73826	
transcript_HQ_CoA_transcript14160/f2p0/2685	19.25666667	6.463333333	16.81666667	-1.4053	1.7075E-06	1.9128E-05	DOWN	1.217	0.00025112	0.002064	UP	-0.1997	0.42226	0.63438	
transcript_HQ_CoA_transcript14167/f2p0/2729	7.546666667	2.093333333	4.793333333	-1.7236	0.01175	0.040144	DOWN	1.0525	0.0047789	0.022587	UP	-0.67713	0.29234	0.50741	
transcript_HQ_CoA_transcript14174/f2p0/2685	2.35	7.906666667	4.076666667	1.9395	0.0052667	0.02072	UP	-1.1437	0.096817	0.22324		0.78153	0.05582	0.17298	
transcript_HQ_CoA_transcript14181/f2p0/2688	42.53666667	16.83333333	26.02666667	-1.184	2.7375E-14	1.0536E-12	DOWN	0.4797	0.00021552	0.0018284		-0.71592	4.5898E-07	1.6232E-05	
transcript_HQ_CoA_transcript14203/f9p0/2610	17.89	72.26666667	24.16	2.1828	1.2196E-19	7.2631E-18	UP	-1.728	1.374E-08	4.1696E-07	DOWN	0.44149	0.1307	0.3046	
transcript_HQ_CoA_transcript14204/f2p0/2670	4.06	8.903333333	3.656666667	1.2774	0.0055294	0.021545	UP	-1.4319	0.0010715	0.0067653	DOWN	-0.16544	0.59732	0.77068	
transcript_HQ_CoA_transcript14205/f2p0/2701	21.26666667	4.04	8.343333333	-2.2389	1.9246E-10	4.4595E-09	DOWN	0.88446	0.0080217	0.033815		-1.3646	0.00004109	0.00067946	DOWN
transcript_HQ_CoA_transcript14214/f2p0/2678	3.84	0.906666667	1.613333333	-1.902	8.2176E-05	0.00059358	DOWN	0.65359	0.26212	0.44746		-1.2585	0.0036206	0.023974	DOWN
transcript_HQ_CoA_transcript14216/f9p0/2646	57.24333333	105.6233333	75.49666667	1.043	1.2354E-12	3.9007E-11	UP	-0.63292	5.5881E-05	0.00059308		0.39786	0.0061776	0.035725	
transcript_HQ_CoA_transcript14221/f4p0/2671	38.48	6.19	6.393333333	-2.5237	0.00047486	0.002702	DOWN	-0.089342	0.8992	0.94643		-2.619	2.8354E-07	1.0709E-05	DOWN
transcript_HQ_CoA_transcript14224/f9p0/2557	29.38	11.09	21.25333333	-1.2585	1.0792E-05	0.00010005	DOWN	0.7871	0.010015	0.040059		-0.48078	0.11833	0.28643	
transcript_HQ_CoA_transcript14227/f3p0/2691	2.886666667	7.45	9.443333333	1.5374	0.00026227	0.0016217	UP	0.17368	0.58523	0.74729		1.6986	1.2399E-07	5.2199E-06	UP
transcript_HQ_CoA_transcript14233/f5p0/2607	88.81	176.7366667	77.03333333	1.1579	0.00066662	0.003611	UP	-1.3518	0.00012458	0.0011515	DOWN	-0.20717	0.2335	0.44186	
transcript_HQ_CoA_transcript14240/f9p0/2635	36.08333333	81.53333333	48.01	1.3351	3.7653E-17	1.9228E-15	UP	-0.91267	3.1545E-10	1.4034E-08		0.41044	0.015767	0.069888	
transcript_HQ_CoA_transcript14248/f2p0/2681	15.57666667	2.496666667	2.216666667	-2.541	0.0037308	0.015646	DOWN	-0.28795	0.76043	0.86531		-2.8346	1.1785E-09	8.1019E-08	DOWN
transcript_HQ_CoA_transcript14257/f16p0/2611	2.84	62.03	23.18333333	4.5932	1.5012E-61	6.099E-59	UP	-1.5246	0.063852	0.1647		3.0552	0.00013387	0.0018027	UP
transcript_HQ_CoA_transcript14258/f3p0/2671	16.04	31.68333333	28.62333333	1.1268	0.00059666	0.0032819	UP	-0.29319	0.21577	0.39246		0.82277	0.011791	0.056625	
transcript_HQ_CoA_transcript14268/f2p0/2669	8.643333333	17.23666667	14.98333333	1.144	5.1229E-07	6.3239E-06	UP	-0.32698	0.39908	0.59008		0.80517	0.035122	0.12485	
transcript_HQ_CoA_transcript14275/f2p0/2674	7.133333333	12.95333333	17.09666667	1.0068	0.00089651	0.0046804	UP	0.25417	0.20767	0.38245		1.2488	1.0305E-05	0.00021667	UP
transcript_HQ_CoA_transcript14288/f2p0/2671	3.643333333	0.303333333	2.493333333	-3.4168	7.6495E-11	1.8771E-09	DOWN	2.8921	7.5961E-06	0.00010702	UP	-0.53518	0.31237	0.52917	
transcript_HQ_CoA_transcript14294/f2p0/2710	16.69333333	6.97	5.393333333	-1.0966	0.011616	0.039791	DOWN	-0.5358	0.10249	0.23267		-1.6456	8.3679E-05	0.0012268	DOWN
transcript_HQ_CoA_transcript14300/f2p0/2654	20.15	68.00666667	27.09	1.9041	5.5844E-18	3.0028E-16	UP	-1.4807	5.6334E-14	5.3778E-12	DOWN	0.41096	0.10363	0.26302	
transcript_HQ_CoA_transcript14307/f2p0/2673	4.733333333	10.15333333	10.11666667	1.27	0.0011084	0.0056066	UP	-0.14799	0.45823	0.64433		1.1076	0.0044069	0.027849	UP
transcript_HQ_CoA_transcript14325/f25p0/2557	20.36333333	61.95666667	19.85666667	1.7796	2.0162E-06	2.2258E-05	UP	-1.7951	2.1974E-05	0.00026498	DOWN	-0.030093	0.92115	0.96509	
transcript_HQ_CoA_transcript14334/f2p0/2666	0.656666667	18.30666667	22.09	4.9895	1.1167E-14	4.5469E-13	UP	0.16641	0.89655	0.94517		5.1393	0.00070395	0.0067822	UP
transcript_HQ_CoA_transcript14338/f3p0/2686	64.54666667	15.00666667	54.78666667	-1.9555	2.4058E-14	9.3581E-13	DOWN	1.709	8.1984E-09	2.6043E-07	UP	-0.25705	0.41541	0.62873	
transcript_HQ_CoA_transcript14344/f6p0/2652	4.86	11.05666667	24	1.3613	0.011279	0.038832	UP	0.96674	0.0012589	0.0076632		2.3129	8.6322E-06	0.00018795	UP
transcript_HQ_CoA_transcript14352/f2p0/2679	83.70666667	23.38333333	31.84333333	-1.7185	0.00025885	0.0016031	DOWN	0.31477	0.36882	0.56076		-1.4116	0.00030898	0.0035269	DOWN
transcript_HQ_CoA_transcript14354/f2p0/2670	15.14	2.486666667	3.5	-2.4422	6.5923E-09	1.1769E-07	DOWN	0.34653	0.44501	0.63304		-2.1076	4.8897E-07	1.7193E-05	DOWN
transcript_HQ_CoA_transcript14357/f2p0/2698	11.78333333	3.093333333	11.62	-1.7859	3.4286E-08	5.4037E-07	DOWN	1.7647	1.2236E-05	0.00016158	UP	-0.031732	0.93364	0.97082	
transcript_HQ_CoA_transcript14364/f4p0/2682	18.21333333	6.403333333	16.83666667	-1.3665	1.5806E-06	0.00001787	DOWN	1.2485	7.5143E-06	0.00010611	UP	-0.12909	0.6255	0.79087	
transcript_HQ_CoA_transcript14372/f2p0/2653	4.03	0.716666667	1.743333333	-2.2953	0.0082315	0.030025	DOWN	1.1045	0.11677	0.25515		-1.2043	0.063126	0.18727	
transcript_HQ_CoA_transcript14377/f2p0/2658	5.84	2.196666667	3.83	-1.2469	0.001333	0.0065881	DOWN	0.64558	0.094393	0.21889		-0.61346	0.080183	0.21993	
transcript_HQ_CoA_transcript1439/f2p0/5334	0.803333333	2.606666667	4.57	1.8348	0.007081	0.026425	UP	0.67478	0.28001	0.46827		2.4973	2.014E-09	1.325E-07	UP
transcript_HQ_CoA_transcript14417/f2p0/2745	9.793333333	17.73	13.89	1.0173	5.0655E-07	6.2615E-06	UP	-0.50649	0.0020237	0.011219		0.49812	0.0097571	0.049401	
transcript_HQ_CoA_transcript14422/f2p0/2675	13.19666667	32.31	20.49666667	1.4506	1.0566E-08	1.8155E-07	UP	-0.80728	0.00066797	0.0046183		0.63095	0.029381	0.11013	
transcript_HQ_CoA_transcript14423/f2p0/2668	1.26	3.653333333	1.26	1.7028	0.00030546	0.0018473	UP	-1.6933	0.00024524	0.0020193	DOWN	-0.0026745	0.99548	0.99771	
transcript_HQ_CoA_transcript14424/f2p0/2689	21.19	7.633333333	14.34	-1.3227	3.348E-06	3.4996E-05	DOWN	0.75864	0.001168	0.0072333		-0.57433	0.049534	0.15925	
transcript_HQ_CoA_transcript14428/f3p0/2651	11.47333333	73.82666667	71.83	2.8504	6.5999E-46	1.6529E-43	UP	-0.14809	0.854	0.92028		2.6881	0.0095814	0.048771	UP
transcript_HQ_CoA_transcript14430/f2p0/2668	6.223333333	13.43	8.48	1.2768	3.1127E-07	4.0046E-06	UP	-0.8192	0.0002177	0.0018375		0.44375	0.070627	0.2018	
transcript_HQ_CoA_transcript14434/f2p0/2664	1.476666667	0.116666667	2.183333333	-3.5646	0.011334	0.03897	DOWN	4.0972	1.6592E-06	2.9412E-05	UP	0.54179	0.56811	0.75016	
transcript_HQ_CoA_transcript14442/f2p0/2650	16.08	5.683333333	10.98	-1.3442	1.0323E-10	2.5097E-09	DOWN	0.79303	0.00036499	0.0028098		-0.56317	0.0073028	0.040037	
transcript_HQ_CoA_transcript14445/f2p0/2659	11.84333333	24.07333333	23.09	1.1784	6.5563E-07	7.9537E-06	UP	-0.19639	0.56368	0.7301		0.97	0.0069282	0.038617	
transcript_HQ_CoA_transcript14451/f3p0/2670	3.46	62.33333333	20.26666667	4.3593	4.015E-17	2.0333E-15	UP	-1.7563	0.00046902	0.0034664	DOWN	2.5861	6.9516E-05	0.0010554	UP
transcript_HQ_CoA_transcript14456/f4p0/2643	6.04	11.77333333	5.17	1.118	0.0060112	0.02307	UP	-1.3357	0.00052664	0.0037988	DOWN	-0.23013	0.37741	0.59386	
transcript_HQ_CoA_transcript1446/f3p0/5326	4.93	1.723333333	2.666666667	-1.3823	0.0034278	0.01456	DOWN	0.50332	0.32585	0.51684		-0.88869	0.013405	0.062107	
transcript_HQ_CoA_transcript14460/f2p0/2650	1.576666667	8.056666667	2.476666667	2.4835	0.00012152	0.00083364	UP	-1.8105	0.0049213	0.023164	DOWN	0.6589	0.18305	0.37896	
transcript_HQ_CoA_transcript14478/f5p0/2652	17.76666667	5.216666667	13.63666667	-1.5742	0.0025005	0.011147	DOWN	1.2072	0.027224	0.08687		-0.38186	0.14058	0.31964	
transcript_HQ_CoA_transcript14481/f5p0/2649	12.37333333	3.786666667	10.75	-1.5087	0.014348	0.046968	DOWN	1.3175	0.016223	0.058509		-0.20621	0.5996	0.77218	
transcript_HQ_CoA_transcript14490/f7p0/2646	19.26333333	70.59666667	11.13	2.02	3.6984E-05	0.00029577	UP	-2.8255	9.862E-17	1.3905E-14	DOWN	-0.81483	0.05207	0.1649	
transcript_HQ_CoA_transcript14496/f2p0/2645	12.16	3.66	4.39	-1.6157	0.0040518	0.016721	DOWN	0.14645	0.78476	0.88046		-1.4791	0.00014604	0.0019368	DOWN
transcript_HQ_CoA_transcript14501/f5p0/2655	7.433333333	0	2.743333333	-10.022	1.2612E-11	3.515E-10	DOWN	8.5659	1.4888E-11	8.3964E-10	UP	-1.4634	0.029565	0.11059	
transcript_HQ_CoA_transcript14506/f2p0/2652	39.8	72.66333333	47.64333333	1.0367	0.00028485	0.0017422	UP	-0.7599	0.0011616	0.0072002		0.26289	0.2276	0.43476	
transcript_HQ_CoA_transcript14509/f3p0/2675	0	27.07	2.93	12.015	1.1305E-17	5.9218E-16	UP	-3.3022	0.076111	0.18731		8.6984	0.026021	0.10049	
transcript_HQ_CoA_transcript14538/f2p0/2655	3.096666667	1.226666667	1.51	-1.1798	0.0052464	0.020663	DOWN	0.14703	0.70272	0.82931		-1.0442	0.0072497	0.039817	DOWN
transcript_HQ_CoA_transcript1454/f3p0/5309	38.83666667	9.326666667	22.06	-1.9005	2.1835E-29	2.8513E-27	DOWN	1.0845	3.6025E-05	0.0004091	UP	-0.82839	0.0014319	0.011864	
transcript_HQ_CoA_transcript14544/f2p0/2687	3.543333333	9.4	4.026666667	1.5846	0.0056442	0.021889	UP	-1.3889	0.0015821	0.0092059	DOWN	0.18517	0.71588	0.85185	
transcript_HQ_CoA_transcript14574/f9p0/2623	7.083333333	42.19666667	7.89	2.7463	1.5743E-09	3.1421E-08	UP	-2.597	3.1687E-10	1.4063E-08	DOWN	0.13822	0.72773	0.86061	
transcript_HQ_CoA_transcript1458/f2p0/5295	8.273333333	0	9.546666667	-11.233	4.7491E-20	2.9632E-18	DOWN	11.435	1.7638E-21	5.388E-19	UP	0.19054	0.39474	0.61025	
transcript_HQ_CoA_transcript14586/f2p0/2679	7.166666667	23.75666667	14.23666667	1.9076	3.9563E-09	7.373E-08	UP	-0.88263	0.01687	0.060368		1.0099	0.012927	0.060532	
transcript_HQ_CoA_transcript14591/f6p0/2574	49.94666667	17.33	26.23333333	-1.3851	0.00053587	0.002996	DOWN	0.44132	0.1337	0.28067		-0.95467	0.0072577	0.039837	
transcript_HQ_CoA_transcript14597/f4p0/2608	0	25.70333333	2.676666667	11.937	5.6087E-23	4.2197E-21	UP	-3.3853	0.04815	0.13259		8.5369	0.028926	0.10887	
transcript_HQ_CoA_transcript14606/f2p0/2651	7.623333333	70.31333333	4.78	3.3845	1.0427E-16	5.1244E-15	UP	-4.0345	1.5176E-15	1.8545E-13	DOWN	-0.66586	0.18766	0.38482	
transcript_HQ_CoA_transcript14607/f2p0/2639	3.31	6.4	7.47	1.1242	0.0028172	0.012348	UP	0.054392	0.88677	0.93973		1.1663	0.0023387	0.017177	UP
transcript_HQ_CoA_transcript14608/f2p0/2669	21.08	8.146666667	15.92333333	-1.2269	0.0014289	0.0069662	DOWN	0.8214	0.0067199	0.02948		-0.41445	0.26269	0.47441	
transcript_HQ_CoA_transcript14638/f2p0/2638	5.61	2.323333333	5.393333333	-1.1231	0.0071696	0.026701	DOWN	1.0592	0.0018306	0.010356	UP	-0.076517	0.84898	0.93111	
transcript_HQ_CoA_transcript14643/f2p0/2663	4.663333333	20.03666667	5.933333333	2.2795	2.4483E-07	3.2341E-06	UP	-1.9204	0.00000581	8.5126E-05	DOWN	0.34476	0.23457	0.44313	
transcript_HQ_CoA_transcript14663/f2p0/2698	5.173333333	12.62333333	8.67	1.445	2.083E-10	4.8022E-09	UP	-0.69179	0.0006098	0.0042726		0.74049	0.0060236	0.035155	
transcript_HQ_CoA_transcript14675/f4p0/2638	16.29	6.353333333	11.40333333	-1.1941	2.4537E-08	3.9803E-07	DOWN	0.6908	0.00020874	0.0017817		-0.51598	0.0090726	0.046896	
transcript_HQ_CoA_transcript14678/f2p0/2629	2.366666667	5.603333333	3.716666667	1.4136	0.00011252	0.00078008	UP	-0.74993	0.078359	0.19135		0.64865	0.16747	0.35781	
transcript_HQ_CoA_transcript14679/f3p0/2634	5.923333333	12.31333333	4.693333333	1.2096	0.0032119	0.013748	UP	-1.5394	0.00005876	0.00061508	DOWN	-0.34192	0.26287	0.47461	
transcript_HQ_CoA_transcript14682/f5p0/2607	1.34	2.96	2.14	1.2963	0.0022317	0.010159	UP	-0.6199	0.065104	0.16698		0.66504	0.10975	0.27311	
transcript_HQ_CoA_transcript14683/f2p0/2634	15.63666667	4.04	6.066666667	-1.8094	0.00034493	0.0020554	DOWN	0.46821	0.28621	0.47514		-1.3541	0.00050236	0.0051832	DOWN
transcript_HQ_CoA_transcript14696/f3p0/2653	8.056666667	2.053333333	4.4	-1.8207	1.1454E-09	2.3502E-08	DOWN	0.94893	0.0075208	0.032178		-0.88172	0.0039303	0.025527	
transcript_HQ_CoA_transcript14701/f3p0/2634	0.59	1.666666667	1.193333333	1.6544	0.013602	0.045025	UP	-0.64327	0.28639	0.47535		0.99732	0.20747	0.40991	

transcript_HQ_CoA_transcript14703/f2p0/2621	1.166666667	2.846666667	0.913333333	1.4593	0.013936	0.045931	UP	-1.8058	0.0011916	0.0073561	DOWN	-0.36093	0.49654	0.69818	
transcript_HQ_CoA_transcript14713/f4p0/2627	12.30333333	0.89	5.75	-3.6349	1.2265E-16	5.9793E-15	DOWN	2.5321	2.6312E-09	9.2922E-08	UP	-1.1126	0.0026235	0.018683	DOWN
transcript_HQ_CoA_transcript14720/f9p0/2550	11.73	33.32	11.23	1.666	2.7877E-06	2.9665E-05	UP	-1.7298	6.9035E-07	0.00001389	DOWN	-0.076689	0.77297	0.88821	
transcript_HQ_CoA_transcript14728/f11p0/2567	0.823333333	7.353333333	22.58333333	3.3489	3.4444E-05	0.00027814	UP	1.4722	0.3386	0.53021		4.8084	0.0011307	0.0098596	UP
transcript_HQ_CoA_transcript14729/f3p0/2646	37.55333333	12.05333333	21.42333333	-1.4489	0.00070688	0.0037898	DOWN	0.65	0.11817	0.25731		-0.81316	0.001158	0.010043	
transcript_HQ_CoA_transcript14733/f4p0/2626	11.3	1.006666667	4.986666667	-3.347	3.2379E-14	1.2384E-12	DOWN	2.1638	1.5658E-05	0.00019793	UP	-1.1924	0.00014464	0.0019225	DOWN
transcript_HQ_CoA_transcript14737/f2p0/2630	27.40333333	6.733333333	22.46	-1.8797	6.2832E-05	0.00047	DOWN	1.6134	0.00022104	0.0018601	UP	-0.27954	0.31684	0.53354	
transcript_HQ_CoA_transcript14755/f2p0/2643	4.086666667	8.56	5.71	1.2123	0.01294	0.043289	UP	-0.72421	0.098183	0.2256		0.47782	0.23768	0.44616	
transcript_HQ_CoA_transcript14756/f3p0/2627	20.44666667	39.7	23.29333333	1.1125	1.2578E-15	5.5142E-14	UP	-0.90665	0.0063765	0.028326		0.19267	0.55916	0.74437	
transcript_HQ_CoA_transcript14758/f7p0/2600	1.426666667	41.62	36.24666667	5.0475	1.1957E-33	1.9346E-31	UP	-0.3049	0.80259	0.8908		4.7262	0.00026177	0.0031019	UP
transcript_HQ_CoA_transcript14765/f2p0/2798	6.173333333	27.41333333	10.28333333	2.3103	2.6903E-20	1.7257E-18	UP	-1.5567	2.6965E-06	4.4527E-05	DOWN	0.74075	0.027971	0.10603	
transcript_HQ_CoA_transcript14768/f2p0/2644	70.13	14.71333333	14.41333333	-2.0983	0.00032267	0.0019424	DOWN	-0.19676	0.63746	0.78364		-2.3034	4.7959E-05	0.00077353	DOWN
transcript_HQ_CoA_transcript14785/f2p0/2691	4.636666667	82.55666667	14.32333333	4.2985	3.9246E-17	1.9931E-15	UP	-2.6436	5.0807E-05	0.00054843	DOWN	1.6392	0.0024027	0.017535	UP
transcript_HQ_CoA_transcript14787/f3p0/2633	1.66	14.99666667	3.436666667	3.3508	8.1735E-18	4.3565E-16	UP	-2.2742	9.5488E-13	7.3848E-11	DOWN	1.0625	0.0069605	0.038738	UP
transcript_HQ_CoA_transcript14790/f2p0/2635	17.18666667	1.52	6.326666667	-3.3524	1.5848E-19	9.3164E-18	DOWN	1.906	3.4626E-10	1.522E-08	UP	-1.4576	2.5504E-06	6.9663E-05	DOWN
transcript_HQ_CoA_transcript14791/f5p0/2638	36.81	14.70333333	18.92	-1.1789	0.0075568	0.02793	DOWN	0.209	0.4824	0.66436		-0.98123	0.029229	0.10976	
transcript_HQ_CoA_transcript14798/f3p0/2656	9.04	3.14	3.493333333	-1.3549	1.0682E-05	9.9186E-05	DOWN	-0.0019483	0.99551	0.99769		-1.3707	2.5219E-07	9.7453E-06	DOWN
transcript_HQ_CoA_transcript14803/f4p0/2629	1.726666667	11.38333333	2.506666667	2.8819	3.7143E-15	1.5828E-13	UP	-2.3048	0.00018007	0.0015777	DOWN	0.56196	0.41761	0.63057	
transcript_HQ_CoA_transcript14806/f5p0/2625	6.67	0.58	9.656666667	-3.3498	1.2028E-14	4.8757E-13	DOWN	3.8855	3.9945E-19	8.1351E-17	UP	0.52342	0.10468	0.26487	
transcript_HQ_CoA_transcript14812/f2p0/2686	5.256666667	2.056666667	3.58	-1.1981	0.00571	0.022098	DOWN	0.65008	0.033407	0.10096		-0.55806	0.1659	0.35532	
transcript_HQ_CoA_transcript14813/f2p0/2625	21.60333333	6.463333333	2.183333333	-1.605	0.0099816	0.035167	DOWN	-1.7058	0.00012661	0.0011673	DOWN	-3.3178	6.7867E-09	4.0125E-07	DOWN
transcript_HQ_CoA_transcript14818/f2p0/2621	7.53	56.82333333	17.38333333	3.0765	1.0151E-32	1.5727E-30	UP	-1.8463	9.963E-12	5.8343E-10	DOWN	1.2165	0.0001993	0.0024835	UP
transcript_HQ_CoA_transcript1482/f2p0/5292	2.93	1.136666667	1.476666667	-1.2009	0.0036121	0.015209	DOWN	0.23746	0.63258	0.78027		-0.97895	0.05734	0.17579	
transcript_HQ_CoA_transcript14826/f11p0/2579	9.43	18.27333333	26.87	1.0985	0.0050166	0.019908	UP	0.40885	0.05643	0.15014		1.4971	7.1328E-05	0.0010784	UP
transcript_HQ_CoA_transcript14845/f2p0/2629	1.99	5.96	3.453333333	1.749	0.00014131	0.00095192	UP	-0.93465	0.036306	0.10711		0.79837	0.09935	0.25583	
transcript_HQ_CoA_transcript14866/f3p0/2622	9.603333333	19.54333333	10.26666667	1.1752	4.5997E-05	0.00035738	UP	-1.0763	1.2321E-08	3.7827E-07	DOWN	0.08791	0.75775	0.87936	
transcript_HQ_CoA_transcript1487/f3p0/5271	16.95666667	3.703333333	9.11	-2.0653	5.8822E-07	7.1692E-06	DOWN	1.1691	0.0053954	0.024866	UP	-0.90641	2.5833E-07	9.9407E-06	
transcript_HQ_CoA_transcript14872/f2p0/2671	4.89	1.5	2.59	-1.5581	2.5727E-05	0.00021438	DOWN	0.64736	0.091521	0.21392		-0.92396	0.0019048	0.014696	
transcript_HQ_CoA_transcript14887/f6p0/2507	8.753333333	62.47	12.32333333	2.9993	2.7674E-13	9.4746E-12	UP	-2.4949	1.1093E-08	3.4579E-07	DOWN	0.4895	0.090222	0.23907	
transcript_HQ_CoA_transcript14895/f3p0/2622	5.27	39.49	5.716666667	3.0773	7.8739E-13	2.5614E-11	UP	-2.9464	1.7135E-12	1.2536E-10	DOWN	0.11636	0.72753	0.8605	
transcript_HQ_CoA_transcript14899/f2p0/2617	2.746666667	9.523333333	3.926666667	1.9242	0.0011475	0.0057742	UP	-1.4046	0.010626	0.042067	DOWN	0.50715	0.16139	0.34942	
transcript_HQ_CoA_transcript14900/f2p0/2614	13.92333333	28.47	10.09666667	1.1717	0.00003074	0.00025213	UP	-1.6349	4.5782E-10	1.9515E-08	DOWN	-0.4751	0.062202	0.18565	
transcript_HQ_CoA_transcript14906/f2p0/2609	7.133333333	2.336666667	3.076666667	-1.4902	0.014846	0.048142	DOWN	0.27058	0.67462	0.81041		-1.2279	1.0537E-05	0.00022027	DOWN
transcript_HQ_CoA_transcript14911/f2p0/2625	8.3	16.47	10.69	1.1301	1.3913E-05	0.00012505	UP	-0.77564	0.026089	0.083995		0.34257	0.29631	0.51178	
transcript_HQ_CoA_transcript14918/f2p0/2597	7.943333333	27.61333333	8.746666667	1.9649	1.0564E-06	1.2349E-05	UP	-1.8127	2.2864E-05	0.00027391	DOWN	0.1381	0.63226	0.79536	
transcript_HQ_CoA_transcript14919/f2p0/2637	4.38	17.83333333	11.11333333	2.1905	0.00022244	0.0014091	UP	-0.84504	0.05674	0.15079		1.3344	0.0076884	0.04157	UP
transcript_HQ_CoA_transcript14930/f2p0/2607	0.173333333	6.073333333	8.62	5.3352	1.6262E-08	2.6953E-07	UP	0.38021	0.81531	0.89941		5.699	0.0047728	0.029536	UP
transcript_HQ_CoA_transcript14944/f2p0/2612	2.563333333	18.66666667	17.48333333	3.0283	2.0624E-16	9.768E-15	UP	-0.20427	0.80707	0.89323		2.8087	0.00090503	0.0082563	UP
transcript_HQ_CoA_transcript14947/f3p0/2707	19.30333333	6.99	10.84333333	-1.3135	2.8496E-11	7.5721E-10	DOWN	0.477	0.061633	0.16033		-0.84918	0.00029858	0.0034317	
transcript_HQ_CoA_transcript14968/f3p0/2621	3.083333333	11.16	1.966666667	1.9979	1.2396E-07	1.7389E-06	UP	-2.63	7.8329E-12	4.7072E-10	DOWN	-0.64119	0.18215	0.3778	
transcript_HQ_CoA_transcript14969/f2p0/2618	5.12	15.12333333	8.326666667	1.7034	0.00014364	0.0009658	UP	-0.99988	0.020008	0.068598		0.69351	0.010473	0.052025	
transcript_HQ_CoA_transcript14974/f2p0/2635	2.756666667	18.07	7.413333333	2.9028	1.8909E-09	3.7171E-08	UP	-1.4495	2.3106E-05	0.00027627	DOWN	1.437	0.0013472	0.011341	UP
transcript_HQ_CoA_transcript14980/f3p0/2629	7.136666667	19.52333333	7.096666667	1.6139	4.1572E-05	0.00032747	UP	-1.6192	2.7955E-09	9.8159E-08	DOWN	-0.019477	0.95962	0.98285	
transcript_HQ_CoA_transcript14991/f2p0/2606	17.39333333	46.14333333	19.52333333	1.5732	4.8133E-10	1.0501E-08	UP	-1.4004	6.5106E-09	2.1121E-07	DOWN	0.15988	0.31549	0.53234	
transcript_HQ_CoA_transcript14993/f3p0/2633	10.24333333	4.483333333	8.216666667	-1.0602	0.01003	0.035316	DOWN	0.73754	0.11155	0.24696		-0.33503	0.41972	0.63242	
transcript_HQ_CoA_transcript14994/f2p0/2619	10.94333333	4.523333333	1.423333333	-1.132	0.0002887	0.0017623	DOWN	-1.8116	6.259E-06	9.0833E-05	DOWN	-2.9527	9.452E-14	1.6844E-11	DOWN
transcript_HQ_CoA_transcript14999/f6p0/2591	15.25333333	30.00666667	23.47333333	1.1425	1.9663E-07	2.649E-06	UP	-0.51192	0.012977	0.049318		0.61749	8.5116E-05	0.0012429	
transcript_HQ_CoA_transcript15010/f12p0/2586	6.74	12.13	20.12333333	1.0022	0.0072319	0.0269	UP	0.58631	0.10218	0.23218		1.5763	3.7148E-05	0.00062785	UP
transcript_HQ_CoA_transcript15013/f2p0/2637	18.04333333	38.52333333	29.91	1.257	1.2591E-07	1.7638E-06	UP	-0.5101	0.010982	0.043166		0.73328	0.0013302	0.011242	
transcript_HQ_CoA_transcript15037/f2p0/2626	1.61	4.666666667	4.703333333	1.6886	0.0003289	0.0019728	UP	-0.14321	0.77383	0.87294		1.5358	0.001472	0.012089	UP
transcript_HQ_CoA_transcript1504/f5p0/5289	2.25	7.74	5.756666667	1.9332	0.000487	0.0027607	UP	-0.57697	0.27048	0.45722		1.3438	0.00002824	0.00050324	UP
transcript_HQ_CoA_transcript15044/f6p0/2537	8.993333333	25.20333333	9.47	1.6575	3.6125E-05	0.00028992	UP	-1.5602	0.00018364	0.0016036	DOWN	0.083104	0.771	0.88659	
transcript_HQ_CoA_transcript15057/f2p0/2594	3.81	65.28666667	9.773333333	4.2586	1.1224E-19	6.7278E-18	UP	-2.8975	3.6805E-12	2.3678E-10	DOWN	1.3497	0.0030742	0.021197	UP
transcript_HQ_CoA_transcript15060/f3p0/2619	21.53666667	6.886666667	20.68333333	-1.4991	1.8058E-09	3.5652E-08	DOWN	1.4428	1.618E-09	6.0399E-08	UP	-0.067131	0.75908	0.87979	
transcript_HQ_CoA_transcript15070/f2p0/2621	19.95	4.94	9.996666667	-1.8674	5.5052E-05	0.00041901	DOWN	0.8681	0.0058214	0.026444		-1.0075	0.025838	0.099948	
transcript_HQ_CoA_transcript15091/f2p0/2605	4.063333333	15.83	4.726666667	2.0974	0.012747	0.042808	UP	-1.8667	0.022594	0.075417		0.21751	0.5686	0.75051	
transcript_HQ_CoA_transcript15092/f2p0/2629	4.093333333	10.45	4.156666667	1.5117	0.00085369	0.0044784	UP	-1.47	5.1274E-09	1.7123E-07	DOWN	0.028445	0.9516	0.97896	
transcript_HQ_CoA_transcript15094/f8p0/2568	11.78333333	4.61	5.416666667	-1.2114	0.00019839	0.0012767	DOWN	0.081034	0.77645	0.87473		-1.1419	0.00045857	0.004829	DOWN
transcript_HQ_CoA_transcript1510/f2p0/5251	13.68333333	5.05	11.42	-1.288	1.6937E-05	0.00014823	DOWN	1.0237	2.2812E-11	1.2519E-09	UP	-0.27497	0.30713	0.52392	
transcript_HQ_CoA_transcript15102/f2p0/2622	31.82333333	13.75333333	28.85333333	-1.0454	9.5351E-05	0.00067554	DOWN	0.90351	0.0029829	0.015375		-0.15364	0.49032	0.69235	
transcript_HQ_CoA_transcript15106/f2p0/2626	4.763333333	1.82	5.553333333	-1.2454	0.00058346	0.0032177	DOWN	1.4762	4.7486E-06	7.1931E-05	UP	0.21848	0.37471	0.59153	
transcript_HQ_CoA_transcript15122/f7p0/2583	57.80666667	18.23666667	31.41	-1.518	0.00017048	0.0011191	DOWN	0.61967	0.023618	0.077972		-0.90896	0.026637	0.10214	
transcript_HQ_CoA_transcript15131/f2p0/2593	1.25	8.316666667	3.686666667	2.9762	0.0025553	0.01135	UP	-1.3578	0.17337	0.3373		1.5987	0.018928	0.07996	
transcript_HQ_CoA_transcript15141/f3p0/2593	16.92333333	32.03666667	21.71333333	1.0724	0.0037555	0.015722	UP	-0.69814	0.010583	0.041903		0.36065	0.22844	0.43568	
transcript_HQ_CoA_transcript15143/f3p0/2602	2.556666667	14.97	2.016666667	2.7229	0.0033754	0.014371	UP	-3.0237	0.00013385	0.0012243	DOWN	-0.31458	0.68755	0.83367	
transcript_HQ_CoA_transcript15144/f2p0/2617	6.62	12.85	8.156666667	1.1239	0.00018021	0.0011762	UP	-0.81914	0.0053526	0.024737		0.29155	0.37415	0.59087	
transcript_HQ_CoA_transcript15151/f7p0/2589	12.84333333	23.23	16.80333333	1.0192	3.5811E-05	0.0002879	UP	-0.61772	0.0061814	0.027695		0.38787	0.078026	0.21582	
transcript_HQ_CoA_transcript15181/f2p0/2592	18.22333333	5.49	8.433333333	-1.576	1.5393E-14	6.1714E-13	DOWN	0.47327	0.018909	0.065789		-1.1152	6.1103E-09	3.6414E-07	DOWN
transcript_HQ_CoA_transcript15183/f2p0/2613	241.4433333	80.4	178.92	-1.4457	7.8044E-06	7.5572E-05	DOWN	1.0345	0.0051115	0.023863	UP	-0.42163	0.28244	0.49648	
transcript_HQ_CoA_transcript15191/f2p0/2620	10.12	2.513333333	3.293333333	-1.8577	2.4729E-08	4.0079E-07	DOWN	0.23518	0.42319	0.6125		-1.6358	1.1812E-07	5.0186E-06	DOWN
transcript_HQ_CoA_transcript15201/f2p0/2593	11.62333333	71.34666667	13.58666667	2.7759	9.7811E-31	1.3445E-28	UP	-2.5462	6.523E-23	2.5438E-20	DOWN	0.21712	0.38049	0.59665	
transcript_HQ_CoA_transcript15203/f2p0/2578	13.67	29.65333333	13.36	1.2785	0.00045493	0.0026037	UP	-1.3113	0.00030047	0.0023834	DOWN	-0.045961	0.84526	0.92919	
transcript_HQ_CoA_transcript15206/f3p0/2586	20.04	5.93	8.023333333	-1.6116	1.4569E-11	4.0357E-10	DOWN	0.29067	0.38956	0.58044		-1.3319	3.7543E-06	9.4916E-05	DOWN
transcript_HQ_CoA_transcript15252/f3p0/2598	47.24333333	17.78666667	30.46333333	-1.2594	0.00018364	0.0011939	DOWN	0.62645	0.10012	0.22872		-0.64548	0.097771	0.25297	
transcript_HQ_CoA_transcript15263/f2p0/2614	3.526666667	1.5	3.43	-1.064	0.010668	0.037106	DOWN	1.0293	0.0083442	0.034838	UP	-0.046445	0.87323	0.94358	
transcript_HQ_CoA_transcript15267/f3p0/2601	13.20666667	3.096666667	8.33	-1.9049	0.0053948	0.021115	DOWN	1.2389	0.072301	0.18024		-0.67525	0.084869	0.22898	
transcript_HQ_CoA_transcript15275/f7p0/2555	44.23666667	14.70666667	39.91	-1.4372	1.2357E-05	0.00011273	DOWN	1.2981	1.8724E-10	8.6448E-09	UP	-0.15043	0.63146	0.79517	
transcript_HQ_CoA_transcript15278/f3p0/2626	120.44	25.91	19.63666667	-2.0919	0.0004843	0.0027505	DOWN	-0.54294	0.2067	0.38114		-2.642	3.434E-07	1.2606E-05	DOWN
transcript_HQ_CoA_transcript15281/f2p0/2586	1.65	8.52	2.343333333	2.5408	0.000279	0.0017128	UP	-1.9911	0.0057486	0.026165	DOWN	0.53419	0.3818	0.59779	

transcript_HQ_CoA_transcript15292/f6p0/2572	59.18	17.59666667	50.97333333	-1.5724	3.9612E-06	4.0684E-05	DOWN	1.3856	0.0042538	0.020714	UP	-0.20116	0.61463	0.78241	
transcript_HQ_CoA_transcript15302/f2p0/2613	9.813333333	18.20333333	13.62666667	1.0677	0.0088402	0.031814	UP	-0.58458	0.04703	0.13025		0.46766	0.24544	0.45479	
transcript_HQ_CoA_transcript15326/f3p0/2572	9.796666667	2.886666667	11.96666667	-1.6133	0.0030527	0.013185	DOWN	1.8879	1.8655E-07	4.3338E-06	UP	0.26662	0.54215	0.73255	
transcript_HQ_CoA_transcript15335/f2p0/2581	3.716666667	1.186666667	1.586666667	-1.5218	0.014153	0.046471	DOWN	0.28853	0.66457	0.80375		-1.241	0.0010827	0.0095495	DOWN
transcript_HQ_CoA_transcript15337/f3p0/2594	5.32	18.44	13.25666667	1.9474	5.4929E-13	1.8094E-11	UP	-0.62263	0.0016524	0.0095363		1.3125	3.5413E-07	1.2948E-05	UP
transcript_HQ_CoA_transcript15343/f2p0/2580	5.096666667	12.75666667	3.756666667	1.508	0.00058254	0.0032136	UP	-1.9179	0.00023146	0.0019244	DOWN	-0.42652	0.46589	0.67348	
transcript_HQ_CoA_transcript15358/f2p0/2587	12.51	25.09333333	26.84333333	1.1572	6.574E-10	1.4057E-08	UP	-0.04423	0.82698	0.90574		1.1001	2.7584E-07	1.0519E-05	UP
transcript_HQ_CoA_transcript15369/f2p0/2579	23.96	8.27	25.57333333	-1.3976	0.0018145	0.0085499	DOWN	1.4743	2.6593E-07	5.9516E-06	UP	0.067695	0.87931	0.94601	
transcript_HQ_CoA_transcript15378/f3p0/2593	32.53	12.21	29.31666667	-1.2539	1.0141E-06	0.0000119	DOWN	1.1146	0.00011026	0.0010449	UP	-0.15127	0.56562	0.74828	
transcript_HQ_CoA_transcript15399/f6p0/2524	76.64666667	28.57	34.45333333	-1.2648	0.012974	0.043379	DOWN	0.1332	0.5376	0.70793		-1.145	0.018875	0.079791	
transcript_HQ_CoA_transcript15404/f2p0/2570	0	16.97666667	1.236666667	11.354	1.3736E-15	5.9933E-14	UP	-3.9484	0.023014	0.076444		7.3897	0.0013536	0.011376	UP
transcript_HQ_CoA_transcript15413/f10p0/2548	25.8	169.9266667	42.67	2.8962	9.8686E-16	4.3897E-14	UP	-2.1476	3.5563E-08	9.7873E-07	DOWN	0.73358	0.020429	0.084417	
transcript_HQ_CoA_transcript1542/f2p0/5239	14.45333333	3.246666667	8.193333333	-2.0221	1.304E-06	1.5022E-05	DOWN	1.1971	0.0026479	0.013938	UP	-0.83503	0.0017814	0.014009	
transcript_HQ_CoA_transcript1543/f2p0/5234	4.083333333	0.136666667	7.146666667	-4.7705	9.8366E-13	3.1494E-11	DOWN	5.5562	4.7177E-36	5.0865E-33	UP	0.77703	0.12762	0.30047	
transcript_HQ_CoA_transcript15458/f3p0/2583	15.58666667	32.14333333	22.64	1.2034	2.4907E-06	2.6848E-05	UP	-0.65805	4.7562E-05	0.00051891		0.5334	0.027415	0.10425	
transcript_HQ_CoA_transcript15461/f2p0/2561	13.89	6.113333333	14.55333333	-1.0252	9.3489E-06	8.8695E-05	DOWN	1.091	8.3336E-05	0.00082969	UP	0.053472	0.84702	0.93007	
transcript_HQ_CoA_transcript15471/f3p0/2564	3.726666667	0.943333333	1.473333333	-1.8131	0.0091055	0.032596	DOWN	0.49639	0.38873	0.57998		-1.3275	0.049455	0.15913	
transcript_HQ_CoA_transcript15475/f2p0/2553	14.81333333	6.066666667	11.72	-1.1306	0.00021866	0.0013902	DOWN	0.7909	0.00051698	0.003749		-0.35077	0.19953	0.40022	
transcript_HQ_CoA_transcript15482/f2p0/2560	27.52	11.21333333	18.12666667	-1.1303	2.0686E-06	2.2741E-05	DOWN	0.53894	0.0012139	0.0074362		-0.60442	0.0090339	0.046785	
transcript_HQ_CoA_transcript15504/f2p0/2562	0.116666667	19.38666667	11.8	7.6733	9.7257E-20	5.9071E-18	UP	-0.85059	0.62542	0.77542		6.8035	0.0017834	0.014013	UP
transcript_HQ_CoA_transcript15514/f3p0/2557	5.16	1.956666667	2.816666667	-1.2714	0.0068056	0.02559	DOWN	0.39845	0.40889	0.59903		-0.885	0.0032322	0.021981	
transcript_HQ_CoA_transcript15518/f2p0/2543	7.88	37.89333333	11.91333333	2.432	3.2127E-09	6.0865E-08	UP	-1.8295	9.7867E-06	0.00013367	DOWN	0.58892	0.01853	0.078748	
transcript_HQ_CoA_transcript15520/f6p0/2536	9.33	33.60333333	16.60333333	2.0113	3.9758E-10	8.8105E-09	UP	-1.175	2.3057E-06	3.9059E-05	DOWN	0.82503	0.0021703	0.01629	
transcript_HQ_CoA_transcript1553/f3p0/5243	3.936666667	8.61	6.663333333	1.2826	0.00091286	0.0047574	UP	-0.51061	0.17097	0.33386		0.75827	0.0013623	0.011439	
transcript_HQ_CoA_transcript15530/f6p0/2505	4.693333333	18.25	9.976666667	2.0953	0.00018911	0.0012256	UP	-0.99937	0.052756	0.1423		1.0857	0.10861	0.27112	
transcript_HQ_CoA_transcript15533/f7p0/2545	24.97	69.00666667	21.01333333	1.628	1.1246E-05	0.00010379	UP	-1.8743	5.2576E-08	1.4027E-06	DOWN	-0.25824	0.22035	0.42636	
transcript_HQ_CoA_transcript15537/f9p0/2527	11.64333333	31.92333333	12.61666667	1.6242	0.00001984	0.00017053	UP	-1.4984	2.9166E-05	0.00033977	DOWN	0.1122	0.66996	0.8209	
transcript_HQ_CoA_transcript1555/f3p0/5273	9.896666667	4.156666667	6.48	-1.1029	2.2749E-05	0.00019246	DOWN	0.49396	0.15495	0.31223		-0.61903	0.033063	0.11956	
transcript_HQ_CoA_transcript15550/f2p0/2554	1.15	6.096666667	3.256666667	2.4984	0.0031462	0.013515	UP	-0.9892	0.23297	0.41357		1.4976	0.0030218	0.020883	UP
transcript_HQ_CoA_transcript15554/f4p0/2539	27.29333333	9.34	19.56	-1.4232	0.0023831	0.01073	DOWN	0.93349	0.036288	0.10709		-0.49947	0.10347	0.26298	
transcript_HQ_CoA_transcript1556/f3p0/5211	6.196666667	1.88	4.89	-1.5698	0.00039047	0.0022902	DOWN	1.211	0.0012207	0.0074703	UP	-0.3694	0.40517	0.61952	
transcript_HQ_CoA_transcript15577/f2p0/2556	8.71	0.843333333	2.063333333	-3.1525	3.6493E-05	0.00029223	DOWN	1.0999	0.085396	0.20345		-2.0678	0.00041492	0.0044407	DOWN
transcript_HQ_CoA_transcript15579/f5p0/2543	4.85	34.09	7.36	2.968	2.4728E-09	4.7723E-08	UP	-2.369	1.4564E-05	0.00018615	DOWN	0.58565	0.21493	0.42004	
transcript_HQ_CoA_transcript15583/f4p0/2546	9.406666667	2.78	11.66666667	-1.6117	4.2621E-07	5.3443E-06	DOWN	1.9237	1.266E-14	1.3812E-12	UP	0.3002	0.18132	0.37678	
transcript_HQ_CoA_transcript15596/f3p0/2561	8.09	2.766666667	13.55	-1.3844	0.0095155	0.033801	DOWN	2.1157	0.00038591	0.002946	UP	0.7227	0.13216	0.30705	
transcript_HQ_CoA_transcript15597/f16p0/2471	43.53666667	98.77666667	53.72666667	1.3317	0.0012899	0.0063977	UP	-1.0208	0.012188	0.046897	DOWN	0.30076	0.52202	0.71723	
transcript_HQ_CoA_transcript15605/f2p0/2539	1.54	7.68	7.246666667	2.4671	2.1469E-05	0.00018316	UP	-0.21769	0.58513	0.74727		2.2376	4.3953E-05	0.00071903	UP
transcript_HQ_CoA_transcript15607/f4p0/2548	18.50666667	86.82666667	23.09333333	2.3956	4.33E-13	1.4446E-11	UP	-2.0698	6.1376E-11	3.1076E-09	DOWN	0.31327	0.089235	0.2371	
transcript_HQ_CoA_transcript15608/f5p0/2531	2.993333333	0.973333333	2.143333333	-1.467	0.0021604	0.0098793	DOWN	0.98917	0.022505	0.075175		-0.48897	0.25736	0.4678	
transcript_HQ_CoA_transcript15616/f4p0/2549	18.51333333	7.58	13.89	-1.1322	6.548E-09	1.1702E-07	DOWN	0.73	0.00010303	0.00099081		-0.41423	0.034336	0.1229	
transcript_HQ_CoA_transcript15618/f2p0/2544	31.98333333	11.55333333	12.58	-1.3191	3.8142E-09	7.13E-08	DOWN	-0.031163	0.88919	0.94061		-1.3619	8.7108E-08	3.8527E-06	DOWN
transcript_HQ_CoA_transcript15628/f3p0/2559	4.293333333	0.92	5.903333333	-2.0552	0.00044915	0.0025741	DOWN	2.5164	0.00033827	0.0026283	UP	0.44924	0.40344	0.61818	
transcript_HQ_CoA_transcript1564/f2p0/5261	31.59666667	8.843333333	12.83666667	-1.7113	0.00011654	0.0008043	DOWN	0.39839	0.18406	0.35201		-1.3208	0.0010566	0.0093828	DOWN
transcript_HQ_CoA_transcript15642/f2p0/2548	3.753333333	0.283333333	9.84	-3.6193	7.1721E-07	8.649E-06	DOWN	5.0029	2.4725E-13	2.0981E-11	UP	1.3733	0.00049842	0.0051541	UP
transcript_HQ_CoA_transcript15650/f3p0/2546	69.89333333	143.43	64.03333333	1.2047	0.00013076	0.00088901	UP	-1.3223	6.8352E-06	9.7953E-05	DOWN	-0.13112	0.46423	0.6718	
transcript_HQ_CoA_transcript15651/f2p0/2535	12.00666667	41.07666667	16.09666667	1.9436	5.4836E-10	1.1822E-08	UP	-1.507	4.0222E-07	8.6441E-06	DOWN	0.42241	0.059227	0.17993	
transcript_HQ_CoA_transcript15654/f2p0/2545	1.62	3.856666667	4.193333333	1.4191	0.00022888	0.0014434	UP	-0.015702	0.97681	0.9878		1.3898	0.015819	0.070052	
transcript_HQ_CoA_transcript1567/f2p0/5217	1.363333333	3.01	1.03	1.2938	0.00062575	0.003418	UP	-1.6881	3.0145E-06	4.9225E-05	DOWN	-0.40709	0.33507	0.55272	
transcript_HQ_CoA_transcript15682/f2p0/2549	2.833333333	10.14	2.91	2.0387	0.0021971	0.010024	UP	-1.9803	0.00052094	0.0037711	DOWN	0.042815	0.93384	0.97087	
transcript_HQ_CoA_transcript15702/f2p0/2564	0.24	1.66	0.49	2.9013	0.0096563	0.034199	UP	-1.893	0.026706	0.08565		0.99392	0.38209	0.59804	
transcript_HQ_CoA_transcript15705/f6p0/2537	23.44333333	10.06333333	27.79333333	-1.0532	0.00035781	0.0021225	DOWN	1.3065	7.9368E-06	0.00011079	UP	0.2408	0.3922	0.60803	
transcript_HQ_CoA_transcript15707/f2p0/2536	5.363333333	11.18	7.426666667	1.2253	0.0024307	0.010902	UP	-0.74005	0.040738	0.11672		0.47145	0.085003	0.22914	
transcript_HQ_CoA_transcript15711/f6p0/2512	0.93	48.92	3.473333333	5.8972	7.0295E-30	9.3805E-28	UP	-3.9622	3.1363E-11	1.6711E-09	DOWN	1.9193	0.0052788	0.031802	UP
transcript_HQ_CoA_transcript15728/f3p0/2544	1.486666667	4.496666667	20.87666667	1.7084	0.0058191	0.022454	UP	2.0912	1.0032E-06	0.00001908	UP	3.793	3.6236E-15	8.5271E-13	UP
transcript_HQ_CoA_transcript15734/f2p0/2392	63.91333333	217.81	185.5566667	1.9391	3.2675E-15	1.4023E-13	UP	-0.39766	0.3453	0.53754		1.5253	4.5312E-05	0.00073798	UP
transcript_HQ_CoA_transcript15738/f6p0/2538	2.516666667	37.88666667	28.24333333	4.0726	8.9024E-11	2.1788E-09	UP	-0.54916	0.32854	0.51939		3.5055	9.1876E-12	1.0596E-09	UP
transcript_HQ_CoA_transcript15741/f2p0/2544	43.55666667	10.30333333	75.67	-1.9258	1.0998E-09	2.2683E-08	DOWN	2.7409	2.7779E-16	3.7165E-14	UP	0.8045	0.014675	0.066131	
transcript_HQ_CoA_transcript15761/f5p0/2477	15.12	34.07	0	1.3136	0.012346	0.041755	UP	-12.368	2.3988E-23	9.9928E-21	DOWN	-11.066	4.2384E-17	1.2966E-14	DOWN
transcript_HQ_CoA_transcript15764/f2p0/2545	2.7	26.87666667	7.016666667	3.4439	0.00026727	0.0016484	UP	-2.0603	0.013299	0.050228		1.3691	0.03057	0.11326	
transcript_HQ_CoA_transcript15787/f5p0/2524	101.4	36.44	74.98	-1.3401	2.1775E-05	0.00018541	DOWN	0.90605	0.00185	0.01043		-0.44227	0.19116	0.3892	
transcript_HQ_CoA_transcript15803/f2p0/2532	13.48666667	3.856666667	2.973333333	-1.6336	0.0011589	0.0058172	DOWN	-0.54116	0.31551	0.50643		-2.1874	1.589E-09	1.0638E-07	DOWN
transcript_HQ_CoA_transcript15809/f2p0/2527	1.16	6.506666667	4.853333333	2.6405	0.00014388	0.00096705	UP	-0.54949	0.21827	0.39545		2.077	0.0034603	0.02318	UP
transcript_HQ_CoA_transcript1581/f2p0/5227	1.893333333	6.683333333	1.123333333	1.9679	0.00075258	0.004011	UP	-2.7215	5.6985E-07	0.00001178	DOWN	-0.76783	0.080916	0.22101	
transcript_HQ_CoA_transcript15817/f2p0/2541	2.366666667	0.963333333	1.42	-1.142	0.012168	0.041288	DOWN	0.42551	0.42284	0.61218		-0.73058	0.11027	0.27421	
transcript_HQ_CoA_transcript15825/f9p0/2522	10.37333333	42.55333333	20.71333333	2.1792	6.845E-11	1.6888E-09	UP	-1.1704	0.00085787	0.0056378	DOWN	0.99724	0.00079946	0.0074867	
transcript_HQ_CoA_transcript15829/f3p0/2530	13.51	5.703333333	9.666666667	-1.0794	4.3105E-06	0.000044	DOWN	0.59414	0.060142	0.15732		-0.49825	0.081605	0.22242	
transcript_HQ_CoA_transcript1584/f3p0/5248	33.78666667	3.686666667	4.03	-3.0806	2.2537E-07	3.0009E-06	DOWN	-0.0078482	0.98678	0.99339		-3.0967	9.4124E-12	1.0731E-09	DOWN
transcript_HQ_CoA_transcript15849/f3p0/2532	7.986666667	26.07	9.733333333	1.8721	4.037E-07	5.0864E-06	UP	-1.5795	2.7724E-05	0.00032444	DOWN	0.27928	0.3923	0.6081	
transcript_HQ_CoA_transcript15854/f2p0/2506	0.04	20.78333333	10.33333333	9.1539	1.8361E-17	9.5364E-16	UP	-1.1416	0.58091	0.74345		8.0036	0.00229	0.016922	UP
transcript_HQ_CoA_transcript15864/f2p0/2580	10.18666667	21.23333333	19.72	1.1997	8.4622E-06	0.00008121	UP	-0.24148	0.33037	0.52143		0.94821	0.00019944	0.0024836	
transcript_HQ_CoA_transcript15883/f8p0/2513	33.06	12.11666667	20.74666667	-1.3014	1.1719E-08	1.9967E-07	DOWN	0.63189	0.0085708	0.03559		-0.68057	0.0012113	0.010423	
transcript_HQ_CoA_transcript15891/f7p0/2515	10.15666667	30.22333333	27.24333333	1.7258	6.1745E-20	3.8136E-18	UP	-0.26007	0.67941	0.81355		1.4526	0.015826	0.070064	
transcript_HQ_CoA_transcript15905/f7p0/2515	64.33333333	27.3	36.38333333	-1.0622	0.00014271	0.00096025	DOWN	0.24961	0.35789	0.5499		-0.82654	2.8717E-06	7.6613E-05	
transcript_HQ_CoA_transcript15908/f2p0/2556	5.783333333	10.89666667	5.56	1.0728	0.0020008	0.0092697	UP	-1.1134	1.5035E-05	0.00019097	DOWN	-0.054367	0.88351	0.94791	
transcript_HQ_CoA_transcript15909/f2p0/2550	27.64	9.7	24.05	-1.367	0.0017302	0.0082203	DOWN	1.1563	5.1289E-07	1.0829E-05	UP	-0.21933	0.59324	0.76832	
transcript_HQ_CoA_transcript1591/f2p0/5211	12.91	4.263333333	4.04	-1.4571	1.1644E-08	1.9858E-07	DOWN	-0.21806	0.49815	0.67662		-1.6879	5.7357E-09	3.4631E-07	DOWN
transcript_HQ_CoA_transcript1592/f11p0/5158	3.683333333	12.39333333	2.316666667	1.8977	1.1143E-05	0.00010305	UP	-2.5615	2.3692E-10	1.0722E-08	DOWN	-0.67561	0.040592	0.13869	
transcript_HQ_CoA_transcript15929/f5p0/2526	4.013333333	9.453333333	9.223333333	1.3673	0.0075578	0.02793	UP	-0.15607	0.73693	0.85162		1.2003	0.012692	0.059689	
transcript_HQ_CoA_transcript15930/f2p0/2531	2.62	0.753333333	1.726666667	-1.6292	0.002849	0.012463	DOWN	1.046	0.039422	0.11372		-0.59755	0.14522	0.32655	

transcript_HQ_CoA_transcript15938/f2p0/2527	32.60333333	12.18333333	25.07666667	-1.2783	8.7289E-07	1.0389E-05	DOWN	0.90087	0.00093346	0.0060414		-0.38736	0.1213	0.29081	
transcript_HQ_CoA_transcript15971/f2p0/2529	2.313333333	5.496666667	1.413333333	1.3965	0.00084194	0.0044282	UP	-2.1098	1.8208E-06	3.1694E-05	DOWN	-0.72606	0.15035	0.33364	
transcript_HQ_CoA_transcript15979/f2p0/2515	0.203333333	45.06666667	1.833333333	7.9002	2.2914E-28	2.756E-26	UP	-4.7428	3.9058E-11	2.0572E-09	DOWN	3.1415	0.0021849	0.016364	UP
transcript_HQ_CoA_transcript15986/f2p0/2518	22.91333333	42.74333333	35.82666667	1.0571	5.1809E-08	7.8668E-07	UP	-0.40776	0.039811	0.11457		0.63648	0.00071675	0.0068843	
transcript_HQ_CoA_transcript15997/f2p0/2527	4.51	15.86666667	8.29	1.9808	1.3235E-06	1.5209E-05	UP	-1.094	0.0011467	0.0071282	DOWN	0.87377	0.0083323	0.044164	
transcript_HQ_CoA_transcript16008/f3p0/2480	12.29333333	5.506666667	6.836666667	-1.0067	0.0040041	0.016566	DOWN	0.18003	0.54719	0.71598		-0.83995	0.0040587	0.026158	
transcript_HQ_CoA_transcript1602/f2p0/5226	5.873333333	1.413333333	2.916666667	-1.8928	0.0056795	0.022003	DOWN	0.86174	0.18753	0.35623		-1.04	0.035979	0.12712	
transcript_HQ_CoA_transcript16033/f14p0/2509	28.40333333	7.253333333	13.74333333	-1.8208	0.00036077	0.0021387	DOWN	0.77896	0.015867	0.057521		-1.0524	0.032023	0.11672	
transcript_HQ_CoA_transcript16035/f2p0/2519	38.40666667	12.76333333	22.39	-1.469	0.0012523	0.006235	DOWN	0.68053	0.13055	0.27602		-0.7972	0.010022	0.050345	
transcript_HQ_CoA_transcript16045/f2p0/2516	13.64333333	4.62	8.04	-1.4246	0.00013751	0.00093073	DOWN	0.678	0.099269	0.22727		-0.75771	0.0086089	0.045238	
transcript_HQ_CoA_transcript16048/f3p0/2533	7.656666667	15.28333333	10.58	1.1603	3.7707E-06	3.8969E-05	UP	-0.67931	0.00092594	0.0060036		0.46881	0.077445	0.21478	
transcript_HQ_CoA_transcript1605/f2p0/5193	16.89	4.546666667	15.73333333	-1.7406	1.1859E-24	1.0227E-22	DOWN	1.6319	1.2325E-06	2.2635E-05	UP	-0.1204	0.70455	0.84446	
transcript_HQ_CoA_transcript16053/f2p0/2547	12.37	4.606666667	5.506666667	-1.292	0.0010969	0.0055613	DOWN	0.12076	0.77776	0.87538		-1.1813	2.7781E-06	7.4878E-05	DOWN
transcript_HQ_CoA_transcript16082/f5p0/2494	12.63	25.91	16.62666667	1.2095	0.00034116	0.0020369	UP	-0.7863	0.00039261	0.0029847		0.40948	0.21194	0.41607	
transcript_HQ_CoA_transcript16085/f2p0/2277	12.46	2.573333333	5.666666667	-2.1339	4.1019E-11	1.0547E-09	DOWN	1.0015	0.0013645	0.0081866	UP	-1.1427	0.00002642	0.0004745	DOWN
transcript_HQ_CoA_transcript1609/f2p0/5223	17.28	2.926666667	6.68	-2.3835	1.2943E-07	1.8104E-06	DOWN	1.0189	0.02986	0.092989		-1.3784	2.539E-10	2.0263E-08	DOWN
transcript_HQ_CoA_transcript161/f2p0/7436	6.56	1.706666667	4.643333333	-1.8085	0.0041699	0.017098	DOWN	1.2849	0.00018745	0.0016314	UP	-0.53251	0.35642	0.57341	
transcript_HQ_CoA_transcript16107/f2p0/2511	0.486666667	1.916666667	1.47	2.1178	0.0035959	0.015165	UP	-0.50928	0.41569	0.60551		1.594	0.0094637	0.048292	UP
transcript_HQ_CoA_transcript16111/f20p0/2488	104.1933333	31.89333333	70.51666667	-1.5835	0.0078446	0.028798	DOWN	0.99579	0.0000211	0.00025612		-0.5939	0.29266	0.50783	
transcript_HQ_CoA_transcript1612/f2p0/5172	8.023333333	2.766666667	6.243333333	-1.3824	0.0024273	0.010895	DOWN	1.0171	0.00072569	0.0049373	UP	-0.37446	0.31648	0.53324	
transcript_HQ_CoA_transcript16120/f2p0/2509	7.486666667	2.83	3.076666667	-1.2692	0.0031701	0.013598	DOWN	-0.012721	0.9753	0.98698		-1.2915	0.00043859	0.004656	DOWN
transcript_HQ_CoA_transcript16122/f4p0/2508	33.49333333	11.54	23.96	-1.3724	0.00010717	0.00074785	DOWN	0.89562	0.0098953	0.039713		-0.48983	0.040264	0.13786	
transcript_HQ_CoA_transcript1613/f3p0/5139	14.19	3.213333333	4.426666667	-1.9631	4.8912E-05	0.00037683	DOWN	0.28965	0.62443	0.7749		-1.6891	6.1778E-06	0.00014299	DOWN
transcript_HQ_CoA_transcript16133/f4p0/2463	29.65	12.33666667	15.45	-1.1075	0.00016107	0.0010634	DOWN	0.17526	0.41541	0.6053		-0.94389	0.0014123	0.011761	
transcript_HQ_CoA_transcript16134/f2p0/2514	6.98	1.443333333	3.273333333	-2.1201	6.8544E-06	6.7263E-05	DOWN	1.0569	0.011401	0.044481	UP	-1.0754	0.024184	0.095014	
transcript_HQ_CoA_transcript16136/f2p0/2530	8.05	0.77	8.366666667	-3.2612	1.8996E-10	4.4184E-09	DOWN	3.3067	1.6799E-07	3.9526E-06	UP	0.036558	0.9405	0.9736	
transcript_HQ_CoA_transcript16138/f2p0/2522	2.103333333	5.413333333	3.753333333	1.5545	0.0057081	0.022095	UP	-0.69096	0.24741	0.43053		0.84841	0.077507	0.21487	
transcript_HQ_CoA_transcript16139/f4p0/2437	0	68.90666667	0.096666667	13.273	7.9399E-25	6.9123E-23	UP	-9.745	2.4459E-36	2.8019E-33	DOWN	3.5008	0.37491	0.59169	
transcript_HQ_CoA_transcript16149/f9p0/2425	20.25333333	59.70333333	60.27	1.6909	1.5081E-05	0.00013395	UP	-0.0912	0.88214	0.93624		1.5886	0.0067808	0.038062	UP
transcript_HQ_CoA_transcript16160/f3p0/2509	108.21	39.30666667	31.15	-1.3206	0.0031231	0.013447	DOWN	-0.48066	0.0019947	0.011079		-1.8107	1.8444E-05	0.00035227	DOWN
transcript_HQ_CoA_transcript16165/f2p0/2518	11.09666667	1.076666667	1.7	-3.1983	2.6634E-13	9.1356E-12	DOWN	0.5171	0.26945	0.45602		-2.6954	1.0318E-11	1.1619E-09	DOWN
transcript_HQ_CoA_transcript1618/f4p0/5174	2.84	7.816666667	9.013333333	1.5956	5.5249E-05	0.00042033	UP	0.061237	0.88869	0.94027		1.6468	1.256E-06	3.8231E-05	UP
transcript_HQ_CoA_transcript16182/f10p0/2493	16	37.75	19.13	1.3959	0.00011953	0.00082243	UP	-1.1334	0.0012186	0.0074603	DOWN	0.25012	0.32746	0.54443	
transcript_HQ_CoA_transcript16208/f3p0/2507	12.27333333	28.45333333	13.43666667	1.371	2.3015E-06	0.00002503	UP	-1.2356	5.5955E-06	0.00008251	DOWN	0.12377	0.55031	0.73853	
transcript_HQ_CoA_transcript16228/f2p0/2519	18.64333333	57.82666667	23.45666667	1.7913	2.1681E-20	1.4006E-18	UP	-1.4449	9.6412E-05	0.00093648	DOWN	0.33323	0.36116	0.57821	
transcript_HQ_CoA_transcript16257/f3p0/2483	19.40666667	7.203333333	11.27666667	-1.2873	0.00058095	0.0032081	DOWN	0.50272	0.081912	0.19731		-0.79324	0.03453	0.12324	
transcript_HQ_CoA_transcript16266/f2p0/2498	5.27	16.23	9.76	1.7936	0.00080921	0.0042806	UP	-0.91513	0.079355	0.19319		0.86874	0.056666	0.17449	
transcript_HQ_CoA_transcript16272/f2p0/2512	7.253333333	2.983333333	2.346666667	-1.1413	0.00064257	0.003498	DOWN	-0.49096	0.16962	0.33191		-1.6445	4.1795E-08	2.0135E-06	DOWN
transcript_HQ_CoA_transcript16275/f3p0/2494	14.18666667	0.086666667	17.05333333	-7.4252	5.2266E-12	1.524E-10	DOWN	7.6661	1.1745E-28	8.6109E-26	UP	0.24528	0.74945	0.87385	
transcript_HQ_CoA_transcript16288/f2p0/2000	1.58	0.186666667	0.763333333	-2.9481	0.00034843	0.0020736	DOWN	1.9413	0.085199	0.2031		-1.0211	0.23969	0.44814	
transcript_HQ_CoA_transcript16304/f2p0/2502	0.436666667	1.856666667	0.03	2.1867	0.015095	0.048801	UP	-6.3307	4.8793E-06	7.3365E-05	DOWN	-4.1606	0.017024	0.073644	
transcript_HQ_CoA_transcript16307/f2p0/1996	10.28666667	27.03	13.58	1.5818	0.0015802	0.0076063	UP	-1.1751	0.016704	0.059899		0.39262	0.062932	0.18691	
transcript_HQ_CoA_transcript16312/f2p0/2493	1208.7	372.1933333	145.5566667	-1.5572	0.0039745	0.016472	DOWN	-1.482	0.0098259	0.039452	DOWN	-3.0487	2.0318E-16	5.5663E-14	DOWN
transcript_HQ_CoA_transcript16317/f2p0/2495	6.323333333	64.04666667	7.71	3.4745	0.0062308	0.023781	UP	-3.2109	2.0592E-08	5.9437E-07	DOWN	0.25538	0.79411	0.90115	
transcript_HQ_CoA_transcript16325/f12p0/2437	59.79666667	18.96666667	53.31666667	-1.504	2.3473E-06	2.5483E-05	DOWN	1.3373	1.2159E-05	0.00016079	UP	-0.17977	0.59714	0.77065	
transcript_HQ_CoA_transcript1633/f2p0/5162	2.246666667	0.07	0.03	-4.8959	3.3808E-08	5.342E-07	DOWN	-1.4366	0.51845	0.69205		-6.3089	4.6196E-15	1.0599E-12	DOWN
transcript_HQ_CoA_transcript16331/f2p0/2516	6.58	17.22	7.386666667	1.5137	0.0055378	0.021564	UP	-1.3497	0.0074718	0.032027	DOWN	0.155	0.6567	0.81256	
transcript_HQ_CoA_transcript16332/f2p0/2484	4.533333333	20.49666667	26.59666667	2.3469	0.00075022	0.004001	UP	0.24158	0.55699	0.72446		2.5723	3.5037E-05	0.00060328	UP
transcript_HQ_CoA_transcript16334/f2p0/2487	2.04	6.283333333	3.423333333	1.8049	3.5644E-05	0.00028669	UP	-1.0353	0.039901	0.11476		0.75439	0.12107	0.29047	
transcript_HQ_CoA_transcript16343/f2p0/2525	76.78666667	25.1	39.94	-1.4583	7.0853E-18	3.7875E-16	DOWN	0.51713	0.011174	0.043792		-0.95246	0.00001298	0.0002621	
transcript_HQ_CoA_transcript16345/f2p0/2493	25.94	11.45333333	15.79	-1.0427	0.008767	0.031613	DOWN	0.33012	0.36362	0.55563		-0.72456	0.011126	0.054502	
transcript_HQ_CoA_transcript16350/f2p0/2517	4.066666667	11.25333333	5.546666667	1.6516	0.00014726	0.00098544	UP	-1.1839	0.0018003	0.010242	DOWN	0.45411	0.19006	0.38761	
transcript_HQ_CoA_transcript1636/f2p0/5178	1.246666667	0.28	1.363333333	-2.014	0.00028199	0.0017288	DOWN	2.1301	0.00066123	0.0045799	UP	0.10671	0.84017	0.9267	
transcript_HQ_CoA_transcript16383/f2p0/2494	42.07666667	17.67	42.67333333	-1.0961	2.1934E-05	0.00018642	DOWN	1.1066	2.9611E-05	0.00034438	UP	-0.00086654	0.99738	0.99858	
transcript_HQ_CoA_transcript16406/f2p0/2475	6.42	13.17333333	14.63333333	1.2065	0.0074614	0.027599	UP	-0.019588	0.96248	0.97983		1.1747	4.7005E-07	1.6592E-05	UP
transcript_HQ_CoA_transcript16413/f2p0/2502	12.60333333	22.78	14.45	1.0131	0.015193	0.049013	UP	-0.81519	0.024017	0.079019		0.18673	0.53705	0.729	
transcript_HQ_CoA_transcript1642/f3p0/5164	1.47	3.593333333	4.38	1.4517	1.2234E-06	1.4147E-05	UP	0.1376	0.67658	0.81209		1.5757	3.1491E-06	8.2339E-05	UP
transcript_HQ_CoA_transcript16420/f2p0/2484	18.93	7.73	20.16	-1.1515	2.8711E-05	0.00023698	DOWN	1.2381	8.7018E-06	0.00012028	UP	0.076543	0.78198	0.89388	
transcript_HQ_CoA_transcript16427/f2p0/2486	44.89666667	6.06	22.67666667	-2.7488	7.5038E-25	6.5954E-23	DOWN	1.7675	4.1552E-06	6.4054E-05	UP	-0.99114	0.0011781	0.010171	
transcript_HQ_CoA_transcript16432/f4p0/2449	4.466666667	20.84333333	13.34	2.3624	8.7519E-09	1.528E-07	UP	-0.78623	0.042501	0.12057		1.5652	0.0001111	0.0015483	UP
transcript_HQ_CoA_transcript16435/f3p0/2501	14.59666667	28.22333333	19.68666667	1.0945	6.9429E-05	0.00051261	UP	-0.65812	0.011905	0.046046		0.42506	0.14861	0.33136	
transcript_HQ_CoA_transcript16437/f2p0/2471	15.25666667	4.523333333	2.543333333	-1.5714	0.013069	0.043633	DOWN	-0.98393	0.075984	0.18712		-2.5699	1.9982E-05	0.00037577	DOWN
transcript_HQ_CoA_transcript1645/f2p0/5154	9.59	3.043333333	4.61	-1.4979	1.9793E-08	3.2424E-07	DOWN	0.45039	0.044268	0.12441		-1.0609	4.2789E-06	0.00010542	DOWN
transcript_HQ_CoA_transcript16452/f2p0/2463	0.243333333	38.65666667	74.34	7.3929	4.9205E-31	6.9733E-29	UP	0.83879	0.61738	0.77021		8.2183	0.00013934	0.0018641	UP
transcript_HQ_CoA_transcript16459/f2p0/2471	8.606666667	37.78666667	18.20333333	2.295	1.123E-32	1.7253E-30	UP	-1.1957	5.9206E-06	8.6676E-05	DOWN	1.0867	8.7938E-05	0.001276	UP
transcript_HQ_CoA_transcript16461/f2p0/2481	34.49	14.58333333	26.83	-1.0993	0.00066115	0.0035867	DOWN	0.72827	4.2458E-06	6.5396E-05		-0.38119	0.20004	0.40098	
transcript_HQ_CoA_transcript1649/f3p0/5158	1.13	3.496666667	11.10333333	1.78	0.0025882	0.011487	UP	1.522	3.7894E-09	1.3031E-07	UP	3.2888	3.5445E-11	3.4978E-09	UP
transcript_HQ_CoA_transcript16493/f2p0/2480	2.37	0.316666667	0.593333333	-2.7948	0.0028574	0.012494	DOWN	0.78531	0.3759	0.56804		-2.0195	0.0064946	0.036987	DOWN
transcript_HQ_CoA_transcript16495/f2p0/2469	1.896666667	3.896666667	2.4	1.2034	0.00054834	0.0030498	UP	-0.84872	0.0046961	0.022306		0.34373	0.39628	0.61188	
transcript_HQ_CoA_transcript16507/f3p0/2465	6.763333333	2.663333333	5	-1.1655	0.005887	0.022677	DOWN	0.73237	0.073573	0.18243		-0.44421	0.1013	0.25943	
transcript_HQ_CoA_transcript16512/f2p0/2472	20.10666667	6.15	18.49333333	-1.5612	8.9281E-07	0.00001062	DOWN	1.4298	5.8228E-07	1.1992E-05	UP	-0.14124	0.66303	0.81609	
transcript_HQ_CoA_transcript16528/f4p0/2469	13.3	51.78	18.06	2.1228	2.4191E-06	2.6158E-05	UP	-1.6675	0.00041249	0.0031126	DOWN	0.44237	0.16997	0.3608	
transcript_HQ_CoA_transcript16539/f2p0/2493	8.583333333	3.34	3.766666667	-1.1913	0.00067421	0.0036413	DOWN	0.0082386	0.98024	0.98977		-1.1963	4.0526E-05	0.00067378	DOWN
transcript_HQ_CoA_transcript1654/f2p0/5170	12.69	3.24	9.556666667	-1.8152	1.0999E-13	3.912E-12	DOWN	1.4097	4.2136E-10	1.8087E-08	UP	-0.41685	0.0085048	0.044858	
transcript_HQ_CoA_transcript16552/f2p0/2495	40.71	8.363333333	26.28666667	-2.1494	1.9999E-07	2.6864E-06	DOWN	1.4966	0.001193	0.0073615	UP	-0.66221	0.1859	0.3828	
transcript_HQ_CoA_transcript1656/f2p0/5254	8.083333333	1.946666667	3.536666667	-1.9128	9.3959E-08	1.3473E-06	DOWN	0.72755	0.048268	0.13286		-1.1976	1.1469E-10	1.0219E-08	DOWN
transcript_HQ_CoA_transcript16560/f2p0/2478	2.89	46.65	5.33	4.1808	2.9163E-64	1.3329E-61	UP	-3.2686	7.8906E-10	3.1578E-08	DOWN	0.89836	0.12594	0.29801	
transcript_HQ_CoA_transcript16561/f2p0/2499	12.84666667	24.51666667	27.50666667	1.0777	2.4151E-05	0.00020254	UP	0.026284	0.91163	0.95264		1.092	5.3955E-12	6.7832E-10	UP
transcript_HQ_CoA_transcript1657/f2p0/5196	5.46	0.176666667	2.39	-4.8056	8.1108E-06	7.8207E-05	DOWN	3.6447	0.00010424	0.00099667	UP	-1.1764	0.086494	0.23175	

transcript_HQ_CoA_transcript16577/f2p0/2472	0.756666667	3.153333333	0.656666667	2.2612	0.0057869	0.022348	UP	-2.4403	0.0006902	0.004747	DOWN	-0.1978	0.79885	0.90393	
transcript_HQ_CoA_transcript16583/f2p0/2458	3.25	27.91	8.213333333	3.2662	3.2182E-11	8.4049E-10	UP	-1.911	0.00066893	0.0046232	DOWN	1.3405	0.0020245	0.015419	UP
transcript_HQ_CoA_transcript16592/f3p0/2478	18.76333333	86.97333333	35.19	2.3653	2.8536E-10	6.4806E-09	UP	-1.4316	0.00037779	0.0028888	DOWN	0.91945	0.010581	0.052313	
transcript_HQ_CoA_transcript16601/f3p0/2460	20.56333333	5.393333333	19.46666667	-1.7768	2.9848E-06	3.1561E-05	DOWN	1.6989	1.654E-06	2.9347E-05	UP	-0.088094	0.75375	0.87616	
transcript_HQ_CoA_transcript16606/f2p0/2469	2.49	9.92	4.17	2.1605	2.5266E-06	2.7187E-05	UP	-1.4007	7.7262E-08	1.9452E-06	DOWN	0.74808	0.13061	0.3045	
transcript_HQ_CoA_transcript16619/f2p0/2516	2.18	13.67	2.363333333	2.8547	0.0010199	0.005235	UP	-2.7425	0.0013489	0.0081126	DOWN	0.10043	0.82588	0.91897	
transcript_HQ_CoA_transcript16620/f3p0/2447	0.206666667	2.886666667	0.953333333	3.9359	1.5921E-06	1.7978E-05	UP	-1.7436	0.050965	0.13853		2.1789	0.075165	0.21003	
transcript_HQ_CoA_transcript16634/f3p0/2468	5.876666667	62.6	11.30666667	3.5592	2.8259E-10	6.4258E-09	UP	-2.5993	5.9135E-05	0.0006176	DOWN	0.94432	0.049827	0.15978	
transcript_HQ_CoA_transcript16639/f2p0/2471	2.62	16.77333333	3.75	2.842	9.2449E-11	2.2565E-09	UP	-2.3175	7.6812E-15	8.5847E-13	DOWN	0.51293	0.32199	0.53895	
transcript_HQ_CoA_transcript16645/f2p0/2497	0.643333333	6.243333333	1.72	3.4967	0.0011073	0.0056048	UP	-2.0283	0.082066	0.19758		1.4527	0.095974	0.24998	
transcript_HQ_CoA_transcript1665/f2p0/5262	11.34	4.02	7.033333333	-1.3225	0.00088364	0.0046183	DOWN	0.64636	0.18387	0.35183		-0.69012	0.05428	0.16974	
transcript_HQ_CoA_transcript16661/f2p0/2493	1.78	61.75	6.56	5.2899	4.959E-31	6.9738E-29	UP	-3.3999	5.6112E-15	6.4685E-13	DOWN	1.8756	7.9634E-07	2.5825E-05	UP
transcript_HQ_CoA_transcript16667/f2p0/2495	18.13333333	7.246666667	9.016666667	-1.1749	1.4276E-06	1.6284E-05	DOWN	0.16756	0.44676	0.63471		-1.0189	3.8441E-06	9.6841E-05	DOWN
transcript_HQ_CoA_transcript16669/f4p0/2462	21.71	6.366666667	11.78666667	-1.6444	0.00073559	0.0039287	DOWN	0.73594	0.054295	0.14558		-0.91683	0.068357	0.19775	
transcript_HQ_CoA_transcript16670/f4p0/2432	12.06666667	28.62666667	16.58	1.3987	8.1906E-06	7.8769E-05	UP	-0.94136	0.0017756	0.010132		0.44492	0.22434	0.4314	
transcript_HQ_CoA_transcript16674/f6p0/2415	57.94	21.57666667	26.45	-1.2664	4.4155E-12	1.2937E-10	DOWN	0.13904	0.51296	0.68758		-1.1406	3.369E-08	1.6713E-06	DOWN
transcript_HQ_CoA_transcript1668/f4p0/5152	6.24	1.106666667	2.186666667	-2.3166	1.2363E-05	0.00011273	DOWN	0.81852	0.24599	0.42884		-1.5074	0.0020071	0.015318	DOWN
transcript_HQ_CoA_transcript16681/f2p0/2460	8.05	58.6	11.34	3.0707	2.8267E-08	4.5291E-07	UP	-2.543	1.6898E-09	6.257E-08	DOWN	0.51029	0.26433	0.47617	
transcript_HQ_CoA_transcript16686/f4p0/2449	0.986666667	95.18333333	5.19	6.7601	3.3714E-51	9.9412E-49	UP	-4.3483	1.8895E-10	8.7018E-09	DOWN	2.396	0.001492	0.012182	UP
transcript_HQ_CoA_transcript16701/f2p0/2472	10.19666667	22.49333333	15.96666667	1.3076	1.1319E-06	1.3155E-05	UP	-0.64533	0.0037273	0.018559		0.64995	0.0087798	0.045847	
transcript_HQ_CoA_transcript16707/f7p0/2479	18.55	8.11	14.72333333	-1.0478	3.9182E-05	0.0003109	DOWN	0.7113	0.0016719	0.0096397		-0.34847	0.17744	0.37124	
transcript_HQ_CoA_transcript16714/f2p0/2457	0.853333333	5.2	15	2.7417	4.9696E-08	7.5776E-07	UP	1.3553	0.00021626	0.0018317	UP	4.0844	4.2076E-16	1.1033E-13	UP
transcript_HQ_CoA_transcript16720/f4p0/2393	9.333333333	21.17	24.92666667	1.3131	0.0019879	0.009229	UP	0.13846	0.86409	0.92619		1.4414	0.076142	0.21214	
transcript_HQ_CoA_transcript16725/f2p0/1978	9.74	3.953333333	5.316666667	-1.1524	0.00012141	0.00083317	DOWN	0.28924	0.35848	0.55037		-0.87473	0.0050836	0.030905	
transcript_HQ_CoA_transcript1673/f2p0/5133	15.17	4.306666667	9.95	-1.6673	7.972E-17	3.9712E-15	DOWN	1.0615	4.8116E-08	1.2893E-06	UP	-0.61802	9.2341E-05	0.0013325	
transcript_HQ_CoA_transcript16734/f3p0/2457	59.39	25.91	39.58666667	-1.0483	3.0132E-09	5.7263E-08	DOWN	0.46261	0.0065068	0.02873		-0.5974	0.00077215	0.0072868	
transcript_HQ_CoA_transcript16739/f3p0/2476	2.923333333	29.37	5.33	3.4845	4.0184E-21	2.731E-19	UP	-2.5972	1.4579E-07	3.4795E-06	DOWN	0.87511	0.081207	0.22154	
transcript_HQ_CoA_transcript16744/f8p0/2419	10.06	22.7	16.77	1.3403	2.2074E-05	0.00018744	UP	-0.58624	0.0097104	0.039126		0.7394	0.0066399	0.03757	
transcript_HQ_CoA_transcript1675/f6p0/5052	3.576666667	7.353333333	9.4	1.192	2.186E-06	2.3859E-05	UP	0.20657	0.35045	0.54255		1.3865	8.613E-08	3.8186E-06	UP
transcript_HQ_CoA_transcript16770/f2p0/2437	32.01	8.75	41.19333333	-1.7249	8.9437E-07	1.0623E-05	DOWN	2.0777	2.3354E-13	2.0003E-11	UP	0.3426	0.32365	0.54074	
transcript_HQ_CoA_transcript16780/f2p0/2460	9.19	25.84	12.41	1.6686	1.6193E-07	2.2093E-06	UP	-1.2252	3.6196E-05	0.00041074	DOWN	0.42971	0.038656	0.13395	
transcript_HQ_CoA_transcript16788/f9p0/2439	20.92666667	67.95	18.43	1.8644	2.5606E-09	4.9277E-08	UP	-2.0367	3.6627E-12	2.3678E-10	DOWN	-0.186	0.38057	0.59669	
transcript_HQ_CoA_transcript16793/f2p0/2466	12.28333333	5.15	13.76	-1.0767	0.00057183	0.003166	DOWN	1.2506	2.1675E-06	0.00003699	UP	0.15976	0.55256	0.73962	
transcript_HQ_CoA_transcript168/f2p0/7403	2.28	0.823333333	1.32	-1.3282	0.0018839	0.0088223	DOWN	0.52847	0.096986	0.22349		-0.81031	0.035598	0.12612	
transcript_HQ_CoA_transcript16800/f6p0/2387	16.83666667	39.07666667	31.97	1.3885	1.5013E-06	1.7037E-05	UP	-0.44464	0.019484	0.067344		0.93009	0.00043717	0.0046437	
transcript_HQ_CoA_transcript16803/f5p0/2452	96.85666667	34.05666667	82.77	-1.3537	4.0027E-28	4.6609E-26	DOWN	1.1276	7.6666E-10	3.0749E-08	UP	-0.23829	0.18305	0.37896	
transcript_HQ_CoA_transcript16810/f2p0/2460	15.25666667	6.233333333	11.74	-1.1305	0.001732	0.0082222	DOWN	0.76179	0.001335	0.0080466		-0.38235	0.25743	0.46783	
transcript_HQ_CoA_transcript16818/f6p0/2444	6.97	25.17	43.57666667	1.9971	6.4222E-11	1.5996E-09	UP	0.64909	0.0051281	0.023917		2.6372	1.5898E-16	4.5595E-14	UP
transcript_HQ_CoA_transcript16821/f2p0/2452	0.88	3.696666667	0.92	2.2501	0.00060206	0.0033064	UP	-2.1613	0.0026017	0.013759	DOWN	0.074514	0.92794	0.96814	
transcript_HQ_CoA_transcript16830/f2p0/2462	12.76	31.60666667	18.27333333	1.4834	8.1875E-06	7.8769E-05	UP	-0.93844	0.0088991	0.036622		0.52957	0.19514	0.39434	
transcript_HQ_CoA_transcript16840/f5p0/2380	26.7	55.42666667	36.41	1.2085	0.00024674	0.001537	UP	-0.752	0.017843	0.062929		0.44451	0.013622	0.062761	
transcript_HQ_CoA_transcript16844/f2p0/2461	5.183333333	0.523333333	0.266666667	-3.21	0.00014605	0.00097986	DOWN	-0.99386	0.51749	0.69148		-4.2251	1.2342E-07	5.2079E-06	DOWN
transcript_HQ_CoA_transcript16857/f2p0/2454	0.886666667	48.54333333	4.333333333	5.9159	1.4026E-89	1.9725E-86	UP	-3.6095	1.453E-06	2.6265E-05	DOWN	2.291	0.009114	0.047004	UP
transcript_HQ_CoA_transcript16863/f3p0/2365	2.876666667	9.7	2.73	1.9233	7.6343E-06	7.4121E-05	UP	-1.9945	0.00056092	0.0039927	DOWN	-0.085225	0.88222	0.94732	
transcript_HQ_CoA_transcript16870/f2p0/2440	10.05	4.51	6.366666667	-1.005	6.5655E-05	0.00048872	DOWN	0.34794	0.11117	0.24632		-0.66914	0.0032477	0.022046	
transcript_HQ_CoA_transcript16873/f2p0/2445	53.21333333	125.7333333	56.35	1.4066	3.0337E-05	0.00024938	UP	-1.3139	0.00029124	0.002326	DOWN	0.07969	0.7499	0.87397	
transcript_HQ_CoA_transcript16881/f3p0/2440	1.973333333	6.246666667	6.676666667	1.8637	0.0051478	0.020344	UP	-0.071569	0.91008	0.95183		1.7734	0.0027678	0.01954	UP
transcript_HQ_CoA_transcript16895/f2p0/2455	29.24333333	63.75333333	29.90333333	1.2877	5.7595E-14	2.1315E-12	UP	-1.2471	1.8523E-09	6.8039E-08	DOWN	0.026666	0.90114	0.95632	
transcript_HQ_CoA_transcript16897/f2p0/2451	21.43666667	9.416666667	10.64	-1.0329	1.2139E-05	0.00011118	DOWN	0.02606	0.90472	0.94888		-1.0188	2.7162E-05	0.00048593	DOWN
transcript_HQ_CoA_transcript16898/f2p0/2447	16.17666667	56.38666667	21.31	1.9646	2.633E-08	4.2486E-07	UP	-1.561	7.1368E-06	0.00010156	DOWN	0.39085	0.11905	0.28726	
transcript_HQ_CoA_transcript16902/f3p0/2457	6.383333333	1.203333333	8.396666667	-2.2359	0.00018893	0.0012248	DOWN	2.6303	2.9506E-12	1.9883E-10	UP	0.38469	0.39465	0.61021	
transcript_HQ_CoA_transcript16905/f5p0/2387	17.35666667	31.16333333	16.36333333	1.0069	8.5539E-05	0.0006152	UP	-1.0813	1.7913E-05	0.0002217	DOWN	-0.087406	0.6745	0.82441	
transcript_HQ_CoA_transcript16908/f2p0/2423	0.57	36.33	3.716666667	6.1849	3.0804E-24	2.5598E-22	UP	-3.4378	3.0771E-05	0.00035741	DOWN	2.7291	0.0029116	0.020297	UP
transcript_HQ_CoA_transcript16911/f3p0/2447	25.65333333	51.68333333	29.22333333	1.1464	0.011574	0.039676	UP	-0.95173	0.034075	0.10249		0.1841	0.40709	0.62133	
transcript_HQ_CoA_transcript1692/f2p0/5161	1.3	5.393333333	1.163333333	2.2017	0.00058431	0.0032215	UP	-2.3583	0.00033179	0.0025845	DOWN	-0.16936	0.66906	0.82029	
transcript_HQ_CoA_transcript16924/f6p0/2422	56.03333333	16.28333333	24.13666667	-1.6302	4.3914E-15	1.8541E-13	DOWN	0.41844	0.0062722	0.027978		-1.2227	1.0178E-10	9.1134E-09	DOWN
transcript_HQ_CoA_transcript1693/f2p0/5134	0.546666667	12.98333333	2.556666667	4.7489	5.2768E-41	1.0962E-38	UP	-2.4967	2.9143E-05	0.00033977	DOWN	2.2367	0.00084967	0.0078766	UP
transcript_HQ_CoA_transcript16936/f5p0/2396	24.39333333	83.35666667	24.53333333	1.9361	1.8432E-06	0.00002051	UP	-1.9154	3.2601E-06	5.2278E-05	DOWN	0.0067061	0.98057	0.99239	
transcript_HQ_CoA_transcript16937/f2p0/2443	7.656666667	19.59	6.323333333	1.5192	4.5451E-05	0.00035419	UP	-1.7981	7.4576E-07	0.00001489	DOWN	-0.29024	0.25386	0.46442	
transcript_HQ_CoA_transcript16942/f2p0/2445	6.93	17.14333333	8.806666667	1.4687	6.4227E-05	0.00047926	UP	-1.1148	0.0020269	0.011227	DOWN	0.34007	0.13804	0.31605	
transcript_HQ_CoA_transcript16946/f3p0/2432	26.35	10.68	7.083333333	-1.1623	0.0023453	0.010602	DOWN	-0.71305	0.22523	0.4041		-1.8888	8.4823E-05	0.0012406	DOWN
transcript_HQ_CoA_transcript1695/f2p0/5118	15.28333333	33.15666667	29.08333333	1.2697	3.3022E-09	6.2432E-08	UP	-0.331	0.16683	0.32819		0.92527	1.7861E-05	0.00034364	
transcript_HQ_CoA_transcript16983/f2p0/2444	5.643333333	45.72333333	9.096666667	3.1611	6.5978E-14	2.4156E-12	UP	-2.4528	7.9679E-07	1.5797E-05	DOWN	0.69454	0.085808	0.23064	
transcript_HQ_CoA_transcript16996/f6p0/2398	1.473333333	11.72666667	3.836666667	3.15	2.7385E-20	1.7506E-18	UP	-1.7634	1.6975E-15	2.0605E-13	DOWN	1.3764	0.00061622	0.0061007	UP
transcript_HQ_CoA_transcript16998/f2p0/2389	17.1	7.096666667	10.34	-1.1063	3.7542E-07	4.7663E-06	DOWN	0.38559	0.10221	0.2322		-0.73243	0.0003914	0.0042434	
transcript_HQ_CoA_transcript17000/f2p0/2402	3.04	10.68333333	24.86	1.9899	0.0025195	0.011221	UP	1.0679	0.0025509	0.013556	UP	3.0432	1.8153E-06	5.2063E-05	UP
transcript_HQ_CoA_transcript17010/f4p0/2401	3.806666667	13.96666667	11.98666667	2.0332	0.00018655	0.0012116	UP	-0.36837	0.41779	0.60742		1.6522	0.00018876	0.0023829	UP
transcript_HQ_CoA_transcript17022/f2p0/2438	15.17666667	6.553333333	10.38	-1.0627	7.8692E-05	0.00057135	DOWN	0.52556	0.050343	0.13713		-0.54843	0.0275	0.10455	
transcript_HQ_CoA_transcript17023/f2p0/2440	9.6	0.526666667	15.89333333	-4.0592	2.6512E-06	2.8345E-05	DOWN	4.7466	3.2737E-19	6.742E-17	UP	0.68457	0.35433	0.57171	
transcript_HQ_CoA_transcript17027/f5p0/2416	21.82	42.81	24.24	1.1361	9.5355E-05	0.00067554	UP	-0.97375	0.0033126	0.016819		0.14928	0.57829	0.75756	
transcript_HQ_CoA_transcript17043/f2p0/2423	12.64666667	4.663333333	0.743333333	-1.2846	0.00034985	0.0020813	DOWN	-2.773	0.0085917	0.035644	DOWN	-4.0768	5.7872E-06	0.00013549	DOWN
transcript_HQ_CoA_transcript1705/f2p0/5129	3.916666667	0.333333333	5.596666667	-3.4375	0.0043282	0.017596	DOWN	3.9036	1.5413E-12	1.1484E-10	UP	0.4672	0.73424	0.86463	
transcript_HQ_CoA_transcript17086/f3p0/2458	4.563333333	12.4	4.583333333	1.6087	0.00151	0.007307	UP	-1.5832	0.00045704	0.0033874	DOWN	0.011549	0.97811	0.99123	
transcript_HQ_CoA_transcript17100/f8p0/2366	15.31333333	29.54666667	22.53666667	1.123	0.0040121	0.016591	UP	-0.54752	0.03308	0.10024		0.56073	0.16426	0.35297	
transcript_HQ_CoA_transcript17102/f2p0/2428	41.02	11.42	38.51333333	-1.6986	1.9566E-11	5.3308E-10	DOWN	1.6036	1.8586E-05	0.00022909	UP	-0.10638	0.74994	0.87397	
transcript_HQ_CoA_transcript17104/f2p0/2430	0.09	1.566666667	1.33	4.3418	0.0016341	0.0078289	UP	-0.38248	0.49624	0.67524		3.9352	0.0020365	0.015491	UP
transcript_HQ_CoA_transcript17105/f6p0/2427	41.25666667	9.21	21.63333333	-2.0163	1.7863E-13	6.256E-12	DOWN	1.076	5.7008E-07	0.00001178	UP	-0.95124	0.00055274	0.0055899	
transcript_HQ_CoA_transcript1711/f8p0/5021	13.44333333	5.866666667	7.33	-1.0395	0.0010071	0.0051822	DOWN	0.17308	0.61994	0.77199		-0.87839	0.0078141	0.042057	
transcript_HQ_CoA_transcript17110/f2p0/2441	16.22666667	1.013333333	10.19	-3.8359	1.4971E-12	4.6548E-11	DOWN	3.1941	4.8524E-14	4.7058E-12	UP	-0.65985	0.10579	0.26663	

transcript_HQ_CoA_transcript17127/f2p0/2459	2.71	14.55333333	3.78	2.5841	5.0518E-08	7.6964E-07	UP	-2.1007	3.0357E-06	4.9415E-05	DOWN	0.47064	0.17759	0.37147	
transcript_HQ_CoA_transcript17145/f2p0/2404	14.41	4.696666667	8.296666667	-1.4453	3.5581E-06	3.6939E-05	DOWN	0.65948	0.015969	0.0578		-0.79947	0.0011756	0.010159	
transcript_HQ_CoA_transcript17168/f2p0/2434	16.24	34.35333333	30.65333333	1.2394	0.003979	0.016475	UP	-0.28689	0.3885	0.57977		0.93887	0.025645	0.099371	
transcript_HQ_CoA_transcript1718/f2p0/5101	6.886666667	2.156666667	1.73	-1.533	0.00013902	0.00093926	DOWN	-0.45931	0.18461	0.35236		-2.0007	4.0526E-06	0.00010093	DOWN
transcript_HQ_CoA_transcript17180/f2p0/2426	6.473333333	14.11333333	5.84	1.2794	0.0065251	0.024732	UP	-1.422	0.0030671	0.015743	DOWN	-0.15307	0.62192	0.78804	
transcript_HQ_CoA_transcript17199/f3p0/2419	16.03333333	0.14	0.766666667	-6.687	3.7689E-07	4.7816E-06	DOWN	2.2389	0.074572	0.19268		-4.4498	0.0054646	0.032583	DOWN
transcript_HQ_CoA_transcript17202/f2p0/2419	6.836666667	0.726666667	7.113333333	-3.1235	2.6285E-07	3.4497E-06	DOWN	3.1782	4.1103E-08	1.1194E-06	UP	0.044574	0.90496	0.95765	
transcript_HQ_CoA_transcript17229/f3p0/2420	7.863333333	22.61333333	27.71333333	1.6857	2.6912E-09	5.1628E-08	UP	0.13742	0.53304	0.70435		1.8111	2.3151E-16	6.249E-14	UP
transcript_HQ_CoA_transcript17240/f3p0/2411	5.71	0.423333333	1.006666667	-3.5857	9.5676E-05	0.00067665	DOWN	1.0862	0.29592	0.4851		-2.5156	8.2947E-07	2.6821E-05	DOWN
transcript_HQ_CoA_transcript17244/f4p0/2389	11.68333333	3.613333333	5.55	-1.5439	0.00014208	0.00095639	DOWN	0.45765	0.10569	0.23775		-1.0967	0.0050849	0.030905	DOWN
transcript_HQ_CoA_transcript1725/f4p0/5114	12.70333333	4.783333333	8.393333333	-1.2535	2.0355E-06	2.2444E-05	DOWN	0.65252	0.012659	0.048301		-0.61465	0.039799	0.13675	
transcript_HQ_CoA_transcript17250/f3p0/2428	3.043333333	1.143333333	2.17	-1.2371	0.012461	0.042055	DOWN	0.76494	0.12019	0.26013		-0.48479	0.2189	0.42473	
transcript_HQ_CoA_transcript17252/f4p0/2383	3.64	8.966666667	2.903333333	1.4444	0.0054056	0.021133	UP	-1.784	0.00032078	0.0025137	DOWN	-0.35109	0.37257	0.58948	
transcript_HQ_CoA_transcript17253/f2p0/2423	5.94	0.813333333	4.786666667	-2.6894	5.3837E-08	8.1389E-07	DOWN	2.3763	7.4725E-06	0.0001056	UP	-0.32504	0.33746	0.55532	
transcript_HQ_CoA_transcript17255/f2p0/2439	27.86666667	9.133333333	17.29	-1.4433	2.8393E-08	4.5414E-07	DOWN	0.77894	0.0011752	0.0072647		-0.67724	0.015563	0.069269	
transcript_HQ_CoA_transcript17258/f5p0/2414	21.42666667	51	25.89	1.4238	5.9621E-08	8.8979E-07	UP	-1.1283	3.5449E-08	9.7844E-07	DOWN	0.28105	0.31768	0.53456	
transcript_HQ_CoA_transcript17266/f8p0/2365	16.47666667	39.15333333	21	1.418	1.6516E-06	1.8559E-05	UP	-1.0592	0.00014146	0.0012785	DOWN	0.34579	0.078385	0.21652	
transcript_HQ_CoA_transcript17268/f2p0/2418	3.403333333	9.536666667	10.49666667	1.6281	0.00012554	0.00085802	UP	0.0027956	0.99398	0.99687		1.6198	1.1144E-09	7.837E-08	UP
transcript_HQ_CoA_transcript17271/f2p0/2457	34.50666667	12.66666667	18.50666667	-1.3135	0.00060163	0.0033059	DOWN	0.40991	0.30045	0.49077		-0.91356	9.3036E-05	0.0013415	
transcript_HQ_CoA_transcript17274/f2p0/2414	3.82	1.546666667	3.693333333	-1.1579	0.0050917	0.020157	DOWN	1.1119	0.0067577	0.029601	UP	-0.056315	0.86879	0.94152	
transcript_HQ_CoA_transcript17280/f2p0/2434	21.78333333	7.956666667	17.23333333	-1.281	0.00015492	0.0010293	DOWN	0.95219	0.0024409	0.013047		-0.34103	0.20813	0.4109	
transcript_HQ_CoA_transcript17282/f3p0/2424	2.52	61.12	10.39	4.7764	1.4093E-31	2.0947E-29	UP	-2.7174	2.7931E-12	1.8961E-10	DOWN	2.0445	2.4618E-11	2.5244E-09	UP
transcript_HQ_CoA_transcript17298/f4p0/2420	2.683333333	21.55	4.3	3.1787	1.6719E-10	3.9388E-09	UP	-2.467	4.4021E-06	0.00006735	DOWN	0.69577	0.15751	0.34417	
transcript_HQ_CoA_transcript1730/f2p0/5100	4.246666667	7.756666667	4.836666667	1.0221	3.1235E-06	3.2856E-05	UP	-0.82826	9.7665E-05	0.00094664		0.18186	0.40327	0.61817	
transcript_HQ_CoA_transcript17308/f2p0/2414	9.893333333	3.09	7.043333333	-1.5379	0.00055344	0.0030763	DOWN	1.0336	0.0018754	0.010544	UP	-0.51522	0.23966	0.44814	
transcript_HQ_CoA_transcript17310/f2p0/2438	2.333333333	9.68	5.833333333	2.2265	9.9939E-05	0.00070286	UP	-0.90256	0.079095	0.19268		1.311	0.0058924	0.034632	UP
transcript_HQ_CoA_transcript17322/f2p0/2412	3.106666667	14.57	9.586666667	2.4157	6.0001E-06	5.9584E-05	UP	-0.77232	0.12666	0.27053		1.6291	1.7538E-05	0.00033826	UP
transcript_HQ_CoA_transcript17342/f7p0/2404	0.203333333	39.58	161.26	7.7561	1.2821E-22	9.4136E-21	UP	1.916	0.32543	0.51645		9.6556	3.2397E-06	8.4467E-05	UP
transcript_HQ_CoA_transcript17344/f46p0/2307	0	167.7366667	9.42	14.457	5.0488E-31	7.0459E-29	UP	-4.2998	0.022897	0.076125		10.141	0.0094473	0.048222	UP
transcript_HQ_CoA_transcript17357/f7p0/2381	12.41	25.73666667	15.52333333	1.207	8.0108E-06	7.7448E-05	UP	-0.87571	0.0019058	0.010681		0.31865	0.19632	0.39587	
transcript_HQ_CoA_transcript17362/f2p0/2417	0.293333333	62.51333333	1.296666667	7.9067	5.5372E-29	6.8864E-27	UP	-5.7409	0.00039394	0.0029936	DOWN	2.1476	0.13617	0.31333	
transcript_HQ_CoA_transcript17368/f13p0/2314	7.313333333	18.05666667	16.93333333	1.47	5.9085E-06	5.8802E-05	UP	-0.2475	0.3829	0.5755		1.2093	0.00034091	0.0038026	UP
transcript_HQ_CoA_transcript17386/f2p0/2403	10.86666667	37.58666667	16.83666667	1.9509	9.3521E-10	1.9518E-08	UP	-1.3021	0.0012117	0.0074351	DOWN	0.63466	0.066165	0.19354	
transcript_HQ_CoA_transcript17389/f10p0/2365	0.196666667	25.77	3.12	7.2334	8.2059E-26	7.5768E-24	UP	-3.1991	9.3807E-06	0.0001285	DOWN	4.0166	2.3291E-05	0.00042793	UP
transcript_HQ_CoA_transcript1741/f2p0/5090	3.55	0.263333333	0.36	-3.5465	0.0001403	0.00094601	DOWN	0.2766	0.64445	0.78821		-3.2868	0.00014956	0.0019749	DOWN
transcript_HQ_CoA_transcript17415/f2p0/2500	23.08333333	44.02	21.51666667	1.1064	0.00018945	0.0012269	UP	-1.1932	3.0923E-05	0.00035895	DOWN	-0.099721	0.63326	0.79597	
transcript_HQ_CoA_transcript17434/f3p0/2418	13.28666667	24.25333333	12.46666667	1.0259	9.7492E-07	1.1484E-05	UP	-1.1101	1.3115E-06	2.3848E-05	DOWN	-0.096831	0.69218	0.83646	
transcript_HQ_CoA_transcript17445/f4p0/2402	2.183333333	13.35	5.223333333	2.7724	1.2441E-11	3.4726E-10	UP	-1.5036	6.3217E-05	0.00065316	DOWN	1.2575	0.0017276	0.013662	UP
transcript_HQ_CoA_transcript17451/f3p0/2406	14.54333333	6.293333333	16.08666667	-1.0626	0.00097682	0.0050475	DOWN	1.2056	0.00050143	0.003666	UP	0.13251	0.7047	0.84453	
transcript_HQ_CoA_transcript17456/f2p0/2388	13.44333333	0.466666667	8.123333333	-4.7288	2.4084E-11	6.5229E-10	DOWN	3.9857	2.7064E-21	7.874E-19	UP	-0.74575	0.18819	0.38545	
transcript_HQ_CoA_transcript17469/f3p0/2409	9.35	49.28666667	15.81333333	2.5651	1.3711E-15	5.9933E-14	UP	-1.7912	1.1199E-05	0.0001495	DOWN	0.75978	0.032007	0.1167	
transcript_HQ_CoA_transcript17481/f2p0/2405	0.013333333	48.14333333	39.20666667	11.76	5.497E-21	3.6947E-19	UP	-0.4321	0.83698	0.91088		11.312	0.00086106	0.0079501	UP
transcript_HQ_CoA_transcript17497/f2p0/2464	5.33	10.62333333	12.60666667	1.1565	0.00029646	0.0018012	UP	0.084568	0.77029	0.87069		1.2292	2.232E-08	1.1606E-06	UP
transcript_HQ_CoA_transcript1750/f9p0/5079	7.81	3.196666667	9.123333333	-1.1374	0.00049339	0.0027903	DOWN	1.35	0.00061712	0.0043123	UP	0.20316	0.62463	0.79021	
transcript_HQ_CoA_transcript1752/f4p0/5067	15.73666667	2.633333333	10.43666667	-2.4083	2.1732E-18	1.2039E-16	DOWN	1.8227	6.7817E-12	4.1573E-10	UP	-0.59852	0.0036888	0.024321	
transcript_HQ_CoA_transcript17520/f3p0/2366	54.09	21.68	40.15333333	-1.1664	2.866E-13	9.7572E-12	DOWN	0.73726	6.3932E-05	0.00065906		-0.44078	0.014176	0.064502	
transcript_HQ_CoA_transcript1756/f2p0/5091	3.146666667	0.8	5.15	-1.8376	0.00034694	0.002066	DOWN	2.544	6.2219E-10	2.5343E-08	UP	0.6953	0.04832	0.15648	
transcript_HQ_CoA_transcript17568/f3p0/2427	4.253333333	9.63	6.07	1.3532	0.0042952	0.017481	UP	-0.80441	0.026881	0.086107		0.53355	0.24762	0.45738	
transcript_HQ_CoA_transcript17579/f4p0/2419	46.97666667	14.98333333	25.64666667	-1.5214	2.4076E-05	0.00020213	DOWN	0.64176	0.034632	0.10369		-0.88919	0.00083597	0.0077732	
transcript_HQ_CoA_transcript17605/f3p0/2404	15.30333333	30.24	16.99666667	1.1422	6.0976E-06	6.0454E-05	UP	-0.98468	5.9368E-05	0.00061933		0.1448	0.50885	0.7077	
transcript_HQ_CoA_transcript17610/f2p0/2392	4.466666667	1.6	4.086666667	-1.3108	0.00090714	0.0047316	DOWN	1.1841	0.00095765	0.0061675	UP	-0.13943	0.63628	0.7978	
transcript_HQ_CoA_transcript17628/f3p0/2366	33.01333333	9.256666667	12.46666667	-1.6468	9.6364E-06	9.0857E-05	DOWN	0.25855	0.46811	0.65204		-1.4027	1.9323E-10	1.5977E-08	DOWN
transcript_HQ_CoA_transcript17632/f2p0/2383	4.216666667	29.05666667	5.92	2.9453	1.1297E-11	3.1824E-10	UP	-2.4498	1.7168E-08	5.0671E-07	DOWN	0.48364	0.2538	0.46436	
transcript_HQ_CoA_transcript17644/f2p0/2398	4.203333333	8.963333333	4.713333333	1.2559	3.8991E-07	4.9297E-06	UP	-1.0812	3.8621E-06	6.0534E-05	DOWN	0.16243	0.56741	0.74964	
transcript_HQ_CoA_transcript17654/f15p0/2372	0.043333333	55.02	18.09333333	10.456	1.4184E-25	1.2901E-23	UP	-1.766	0.37836	0.57049		8.6601	0.010542	0.052188	
transcript_HQ_CoA_transcript1767/f2p0/5069	16.32333333	30.21666667	29.68333333	1.0418	3.7653E-06	3.8935E-05	UP	-0.16984	0.41197	0.60197		0.8595	3.9699E-07	1.4288E-05	
transcript_HQ_CoA_transcript17695/f3p0/2376	17.01666667	7.616666667	9.976666667	-1.0018	0.00044861	0.0025718	DOWN	0.24073	0.21936	0.39656		-0.7738	0.0047509	0.02943	
transcript_HQ_CoA_transcript17704/f7p0/2377	30.62333333	4.336666667	12.52333333	-2.6488	6.0455E-05	0.00045427	DOWN	1.3535	0.02697	0.086271		-1.3042	0.0016984	0.013472	DOWN
transcript_HQ_CoA_transcript17712/f2p0/2385	6.206666667	1.75	3.643333333	-1.6556	4.8529E-07	6.0109E-06	DOWN	0.88573	0.0086053	0.035685		-0.78156	0.0051592	0.031263	
transcript_HQ_CoA_transcript17723/f3p0/2373	17.23	30.96	25.31666667	1.0182	7.4164E-05	0.00054311	UP	-0.45414	0.04947	0.13533		0.54971	0.01738	0.074838	
transcript_HQ_CoA_transcript17724/f3p0/2374	0.76	36.40333333	14.04666667	5.7458	1.51E-55	4.9297E-53	UP	-1.502	0.058611	0.15446		4.2269	5.1046E-07	1.7779E-05	UP
transcript_HQ_CoA_transcript17741/f2p0/2352	1.736666667	27.24	2.786666667	4.1476	1.0696E-16	5.2422E-15	UP	-3.4475	1.5051E-07	3.5734E-06	DOWN	0.68346	0.30771	0.52432	
transcript_HQ_CoA_transcript17750/f4p0/2374	0	38.90333333	2.986666667	12.355	4.7927E-22	3.3961E-20	UP	-3.809	0.02883	0.090698		8.5319	0.02902	0.10917	
transcript_HQ_CoA_transcript17754/f3p0/2374	7.95	1.823333333	5.58	-1.967	1.8697E-06	2.0754E-05	DOWN	1.4634	0.0013638	0.0081866	UP	-0.51374	0.11108	0.27528	
transcript_HQ_CoA_transcript17778/f2p0/2369	7.65	2.226666667	5.223333333	-1.627	0.0091049	0.032596	DOWN	1.0545	0.016248	0.058579		-0.58041	0.27387	0.48683	
transcript_HQ_CoA_transcript17788/f9p0/2298	20.66333333	53.6	26.43	1.5479	4.7393E-05	0.00036654	UP	-1.1768	0.00075678	0.0050959	DOWN	0.35719	0.19462	0.39359	
transcript_HQ_CoA_transcript17789/f4p0/2312	21.74333333	40.34	25.01666667	1.0526	0.00022969	0.0014475	UP	-0.84508	0.00068393	0.0047109		0.1957	0.40466	0.61931	
transcript_HQ_CoA_transcript1779/f2p0/5094	1.286666667	4.586666667	5.233333333	1.9773	0.0024928	0.011121	UP	0.044693	0.93988	0.96841		2.0096	0.00055563	0.0056098	UP
transcript_HQ_CoA_transcript17790/f2p0/2370	5.506666667	13.60333333	8.103333333	1.4776	5.0078E-05	0.00038452	UP	-0.90206	0.0077476	0.03291		0.56103	0.045143	0.14941	
transcript_HQ_CoA_transcript17791/f2p0/2380	8.29	1.67	7.216666667	-2.1533	1.3063E-06	1.5039E-05	DOWN	1.9493	7.8533E-08	1.9745E-06	UP	-0.21338	0.47556	0.68088	
transcript_HQ_CoA_transcript17793/f2p0/2369	1.456666667	3.826666667	7.9	1.5449	0.00044594	0.0025581	UP	0.90765	0.0097572	0.039254		2.4411	2.5875E-11	2.6386E-09	UP
transcript_HQ_CoA_transcript17794/f3p0/2480	9.4	3.993333333	9.443333333	-1.0758	0.013379	0.044414	DOWN	1.0735	0.0043625	0.021093	UP	-0.012483	0.97075	0.98811	
transcript_HQ_CoA_transcript17800/f4p0/2312	2.556666667	11.47666667	5.956666667	2.3328	1.2362E-05	0.00011273	UP	-1.1027	0.018355	0.06424		1.2181	0.032827	0.11899	
transcript_HQ_CoA_transcript17803/f3p0/2377	4.693333333	11.36333333	6.986666667	1.4252	0.00014657	0.0009812	UP	-0.85427	0.0072948	0.031379		0.55898	0.062424	0.186	
transcript_HQ_CoA_transcript17810/f2p0/2391	9.81	33.40666667	14.17666667	1.9255	3.4519E-08	5.4357E-07	UP	-1.391	1.8883E-08	5.5202E-07	DOWN	0.52409	0.10436	0.26443	
transcript_HQ_CoA_transcript17813/f3p0/2347	0.886666667	5.333333333	6.553333333	2.7708	0.0024608	0.01101	UP	0.20151	0.8442	0.91526		2.9524	0.0093021	0.047746	UP
transcript_HQ_CoA_transcript17815/f3p0/2321	14.49	5.74	12.16	-1.1836	0.0022649	0.010288	DOWN	0.94133	0.0036401	0.018175		-0.25461	0.46704	0.67432	
transcript_HQ_CoA_transcript17830/f2p0/2368	9.316666667	17.09333333	11.11	1.0409	0.0039086	0.016249	UP	-0.78472	0.0041992	0.020492		0.2434	0.42239	0.6344	
transcript_HQ_CoA_transcript1784/f2p0/5050	4.52	1.313333333	1.903333333	-1.6378	0.0026522	0.011726	DOWN	0.39827	0.46741	0.65158		-1.2528	0.011502	0.055763	

transcript_HQ_CoA_transcript17852/f4p0/2358	39.08	16.15333333	37.11333333	-1.1108	0.00012912	0.00087852	DOWN	1.0332	0.0046045	0.021978	UP	-0.088166	0.77086	0.88659		
transcript_HQ_CoA_transcript17860/f7p0/2359	1.493333333	42.96	19.67	4.9796	1.2024E-14	4.8757E-13	UP	-1.2544	0.13439	0.28187		3.7143	9.6862E-05	0.0013836	UP	
transcript_HQ_CoA_transcript17867/f2p0/2364	3.373333333	9.65	5.706666667	1.6681	2.9959E-09	5.6993E-08	UP	-0.89454	0.0020365	0.01127		0.76139	0.019012	0.08024		
transcript_HQ_CoA_transcript17868/f2p0/2352	2.816666667	121.9666667	6.26	5.5865	5.8798E-53	1.7622E-50	UP	-4.4222	5.736E-13	4.6315E-11	DOWN	1.1517	0.11672	0.28418		
transcript_HQ_CoA_transcript17880/f2p0/2355	6.443333333	0.303333333	5.563333333	-4.1869	0.00027661	0.0016987	DOWN	3.9573	0.00054174	0.0038909	UP	-0.23893	0.62131	0.7876		
transcript_HQ_CoA_transcript17886/f3p0/2375	0.6	51.97333333	3.033333333	6.5775	2.0566E-72	1.5666E-69	UP	-4.2162	1.338E-08	4.0807E-07	DOWN	2.3478	0.014046	0.064067		
transcript_HQ_CoA_transcript17895/f8p0/2325	56.51666667	12.99	45.61	-1.9476	2.1158E-07	2.8296E-06	DOWN	1.6296	6.2621E-05	0.0006481	UP	-0.32986	0.22524	0.43229		
transcript_HQ_CoA_transcript17896/f2p0/2361	3.183333333	6.793333333	3.636666667	1.2673	0.010092	0.035508	UP	-1.0659	0.032746	0.099437		0.19041	0.64629	0.80447		
transcript_HQ_CoA_transcript17899/f2p0/2367	2.11	6.62	8.476666667	1.8146	0.0068118	0.025603	UP	0.20845	0.80363	0.89142		2.0109	0.019652	0.082113		
transcript_HQ_CoA_transcript179/f2p0/7391	68.75333333	10.07333333	11.73333333	-2.6426	0.015248	0.049148	DOWN	0.11218	0.8331	0.90888		-2.5424	0.0072196	0.039722	DOWN	
transcript_HQ_CoA_transcript17908/f2p0/2348	5.466666667	1.733333333	3.896666667	-1.5333	0.0012829	0.0063671	DOWN	1.0376	0.07036	0.17685		-0.50444	0.29566	0.51133		
transcript_HQ_CoA_transcript17911/f2p0/2378	2.52	12.03666667	2.173333333	2.4138	0.00066273	0.0035942	UP	-2.6171	3.4435E-05	0.00039448	DOWN	-0.2156	0.75601	0.8781		
transcript_HQ_CoA_transcript17926/f2p0/2369	10.65	30.89	12.24666667	1.694	0.00036556	0.0021649	UP	-1.4892	0.00034361	0.002661	DOWN	0.19222	0.54939	0.7379		
transcript_HQ_CoA_transcript17932/f14p0/2338	0	42.95	2.606666667	12.513	9.3503E-22	6.5747E-20	UP	-4.2063	1.9048E-09	6.9687E-08	DOWN	8.2916	6.5895E-08	3.0238E-06	UP	
transcript_HQ_CoA_transcript17933/f3p0/2380	11.78	30.73666667	13.55	1.5399	7.0648E-09	1.254E-07	UP	-1.3301	2.1886E-06	3.7247E-05	DOWN	0.19809	0.51289	0.71055		
transcript_HQ_CoA_transcript17954/f4p0/2319	6.446666667	25.46666667	7.506666667	2.1599	0.00010076	0.00070768	UP	-1.9102	7.387E-08	1.8701E-06	DOWN	0.2338	0.7077	0.84638		
transcript_HQ_CoA_transcript17955/f6p0/2337	27.96666667	8.166666667	17.61	-1.6379	1.0933E-09	2.2611E-08	DOWN	0.97095	0.00082636	0.005472		-0.67711	0.0040315	0.026019		
transcript_HQ_CoA_transcript17958/f4p0/2322	4.286666667	17.17666667	4.633333333	2.1826	0.00037904	0.0022311	UP	-2.0431	9.539E-13	7.3848E-11	DOWN	0.12009	0.85844	0.93606		
transcript_HQ_CoA_transcript1796/f3p0/5086	3.186666667	1.036666667	1.953333333	-1.4446	0.0023524	0.010625	DOWN	0.75828	0.29266	0.48222		-0.70328	0.28337	0.49756		
transcript_HQ_CoA_transcript17962/f2p0/2373	80.99666667	10.12666667	15.67666667	-2.8817	0.00043339	0.0024983	DOWN	0.50649	0.35198	0.54392		-2.3816	0.023104	0.09173		
transcript_HQ_CoA_transcript17969/f6p0/2331	19.86333333	7.196666667	5.436666667	-1.2757	0.0049024	0.019548	DOWN	-0.57657	0.22254	0.40063		-1.8681	5.3203E-11	5.0598E-09	DOWN	
transcript_HQ_CoA_transcript17976/f3p0/2312	0.106666667	4.306666667	0.013333333	5.4942	2.0368E-13	7.0525E-12	UP	-8.3269	7.2247E-11	3.6181E-09	DOWN	-2.8452	0.25018	0.46049		
transcript_HQ_CoA_transcript1798/f2p0/5056	2.916666667	7.12	7.123333333	1.4316	8.2099E-09	1.4418E-07	UP	-0.14856	0.50881	0.68468		1.2715	2.6729E-08	1.3704E-06	UP	
transcript_HQ_CoA_transcript17982/f2p0/2378	29.39666667	7.813333333	26.06333333	-1.7603	2.334E-14	9.0983E-13	DOWN	1.5801	1.3031E-10	6.2201E-09	UP	-0.19096	0.4655	0.67315		
transcript_HQ_CoA_transcript17983/f2p0/2355	51.79333333	13.55333333	13.64333333	-1.7643	0.00072583	0.0038823	DOWN	-0.1408	0.79317	0.88604		-1.9174	0.00021011	0.00259	DOWN	
transcript_HQ_CoA_transcript17990/f2p0/2372	37.60666667	16.75	31.08666667	-1.0155	0.00042005	0.0024333	DOWN	0.74647	0.00032651	0.0025509		-0.27973	0.28429	0.49852		
transcript_HQ_CoA_transcript17991/f5p0/2328	6.336666667	20.34	8.39	1.8527	6.7109E-07	8.1197E-06	UP	-1.4368	4.835E-06	7.2938E-05	DOWN	0.40422	0.25119	0.46162		
transcript_HQ_CoA_transcript18004/f2p0/2395	3.533333333	7.686666667	3.106666667	1.2784	1.2226E-06	1.4146E-05	UP	-1.4537	1.5957E-07	3.7643E-06	DOWN	-0.18873	0.59359	0.76847		
transcript_HQ_CoA_transcript18045/f2p0/2390	9.856666667	1.646666667	7.12	-2.4663	0.00024202	0.0015127	DOWN	1.9791	0.0049017	0.02309	UP	-0.49496	0.28805	0.50268		
transcript_HQ_CoA_transcript18058/f2p0/2355	10.43333333	30.9	15.36666667	1.73	3.8649E-06	3.9835E-05	UP	-1.1549	0.0048723	0.022969	DOWN	0.56118	0.050519	0.16116		
transcript_HQ_CoA_transcript18074/f5p0/2348	38.59333333	9.57	24.34333333	-1.8773	4.9632E-06	5.0071E-05	DOWN	1.1899	0.00014236	0.0012853	UP	-0.69797	0.096016	0.25		
transcript_HQ_CoA_transcript18076/f4p0/2252	81.36	12.72333333	22.6	-2.5387	0.0018959	0.0088668	DOWN	0.6961	0.40754	0.59788		-1.8517	9.694E-07	3.0784E-05	DOWN	
transcript_HQ_CoA_transcript18081/f2p0/2334	2.463333333	10.78666667	6.713333333	2.2875	0.0014368	0.0069956	UP	-0.83425	0.16362	0.32417		1.4372	0.0036235	0.023984	UP	
transcript_HQ_CoA_transcript18083/f7p0/2344	20.90666667	0.11	1.703333333	-7.4562	5.2959E-14	1.9679E-12	DOWN	3.8064	0.00012937	0.0011898	UP	-3.6539	3.7519E-05	0.00063296	DOWN	
transcript_HQ_CoA_transcript18087/f2p0/2345	3.02	0.346666667	1.853333333	-2.968	4.1022E-05	0.00032354	DOWN	2.2725	0.0019116	0.010705	UP	-0.7067	0.14607	0.32756		
transcript_HQ_CoA_transcript18090/f4p0/2346	2.786666667	5.59	4.016666667	1.1731	0.0024777	0.011068	UP	-0.61325	0.11311	0.2493		0.5471	0.24512	0.45456		
transcript_HQ_CoA_transcript18097/f3p0/2369	4.476666667	9.606666667	6.046666667	1.2686	9.1598E-05	0.00065312	UP	-0.82837	0.0097159	0.039139		0.42635	0.15074	0.33404		
transcript_HQ_CoA_transcript18102/f4p0/2325	41.29333333	17.65333333	26.89666667	-1.0598	1.4104E-06	1.6116E-05	DOWN	0.4488	0.022259	0.074545		-0.62492	0.0045749	0.028699		
transcript_HQ_CoA_transcript18147/f3p0/2346	9.773333333	3.863333333	7.49	-1.1748	3.0235E-06	3.1896E-05	DOWN	0.79637	0.00056814	0.0040331		-0.39007	0.073661	0.20705		
transcript_HQ_CoA_transcript18148/f3p0/2342	0.99	2.34	1.346666667	1.3987	0.015394	0.049479	UP	-0.95628	0.067301	0.171		0.42889	0.52952	0.72289		
transcript_HQ_CoA_transcript18154/f2p0/2369	0	1.226666667	11.09666667	7.4152	1.0197E-06	1.1957E-05	UP	3.015	0.11501	0.25237		10.413	0.0076929	0.04157	UP	
transcript_HQ_CoA_transcript18159/f2p0/2359	9.97	23.65666667	15.37666667	1.3947	0.0010662	0.005423	UP	-0.75551	0.066652	0.16977		0.62703	0.026009	0.10048		
transcript_HQ_CoA_transcript18165/f2p0/2342	44.28333333	85.28666667	58.96666667	1.1009	1.5044E-11	4.1545E-10	UP	-0.67104	0.0012042	0.0074016		0.417	0.053532	0.16813		
transcript_HQ_CoA_transcript18169/f3p0/2348	17.23666667	4.626666667	8.543333333	-1.7037	0.0012642	0.0062888	DOWN	0.72062	0.098748	0.22639		-0.99908	0.026473	0.10177		
transcript_HQ_CoA_transcript18182/f2p0/2353	8.386666667	0.973333333	12.89	-2.9338	5.1665E-07	6.3735E-06	DOWN	3.5429	2.5025E-07	5.6559E-06	UP	0.59802	0.25923	0.47003		
transcript_HQ_CoA_transcript18187/f14p0/2298	106.23	46.01	79.55333333	-1.0542	7.6735E-12	2.1954E-10	DOWN	0.64128	1.0553E-06	1.9901E-05		-0.42458	0.0012372	0.010596		
transcript_HQ_CoA_transcript18192/f3p0/2352	24.61333333	8.846666667	21.45666667	-1.3154	9.4567E-07	0.00001119	DOWN	1.1218	0.00022266	0.0018704	UP	-0.20607	0.51364	0.71111		
transcript_HQ_CoA_transcript18199/f4p0/2345	5.91	13.22666667	12.48666667	1.3451	0.0010452	0.005343	UP	-0.24643	0.46412	0.64899		1.0852	0.00056759	0.0056961	UP	
transcript_HQ_CoA_transcript18207/f2p0/2345	10.28	0.163333333	1.14	-5.7659	0.0096533	0.034195	DOWN	2.5409	0.13832	0.28797		-3.2275	0.00062032	0.0061347	DOWN	
transcript_HQ_CoA_transcript18229/f6p0/2311	4.813333333	32.06666667	30.94666667	2.9048	2.2595E-06	2.4617E-05	UP	-0.19798	0.50705	0.68311		2.6924	7.4635E-06	0.00016598	UP	
transcript_HQ_CoA_transcript18231/f2p0/2337	4.54	10.3	5.736666667	1.3175	0.00040787	0.0023778	UP	-0.97588	0.010515	0.0417		0.3299	0.40389	0.61856		
transcript_HQ_CoA_transcript18232/f2p0/2335	25.85	4.556666667	7.146666667	-2.3439	1.4293E-21	9.8976E-20	DOWN	0.48996	0.072326	0.18024		-1.8653	4.0532E-22	2.3999E-19	DOWN	
transcript_HQ_CoA_transcript18236/f2p0/2353	8.87	3.6	7.276666667	-1.1552	0.00023899	0.0014968	DOWN	0.85527	0.0095388	0.038587		-0.31132	0.34035	0.55831		
transcript_HQ_CoA_transcript18237/f2p0/2359	3.686666667	7.74	4.43	1.2302	0.00026633	0.0016433	UP	-0.95141	0.036098	0.10671		0.26658	0.57918	0.75821		
transcript_HQ_CoA_transcript18244/f7p0/2340	12.59333333	25.69	14.90333333	1.1922	0.00041343	0.0024018	UP	-0.94642	0.0032661	0.016625		0.2336	0.20545	0.40795		
transcript_HQ_CoA_transcript18258/f2p0/2330	42.24	12.28333333	10.8	-1.6408	0.0044741	0.018068	DOWN	-0.32539	0.55891	0.72608		-1.9782	1.4875E-10	1.2818E-08	DOWN	
transcript_HQ_CoA_transcript18265/f4p0/2327	1.05	6.856666667	2.276666667	2.8688	3.6013E-08	5.6285E-07	UP	-1.7254	0.099834	0.22834		1.1258	0.33525	0.55292		
transcript_HQ_CoA_transcript18277/f52p0/2192	103.52	389.6833333	118.1266667	2.0706	3.7164E-10	8.2958E-09	UP	-1.8758	1.0997E-12	8.3291E-11	DOWN	0.18422	0.49053	0.69249		
transcript_HQ_CoA_transcript18304/f3p0/2348	1.21	63.75	4.463333333	5.8584	9.6864E-16	4.3192E-14	UP	-3.9744	0.025537	0.082727		1.8726	0.19898	0.39963		
transcript_HQ_CoA_transcript18309/f3p0/2336	69.20666667	153.1666667	86.99	1.3017	5.2997E-21	3.5752E-19	UP	-0.96294	5.268E-12	3.3181E-10		0.32711	0.031937	0.11654		
transcript_HQ_CoA_transcript18325/f3p0/2344	17.33333333	5.203333333	16.32	-1.5701	1.7599E-05	0.00015321	DOWN	1.4833	0.0007291	0.0049568	UP	-0.098682	0.79627	0.90254		
transcript_HQ_CoA_transcript18331/f3p0/2404	41.57333333	13.33333333	13.70666667	-1.5085	0.0019389	0.0090381	DOWN	-0.10896	0.71885	0.83917		-1.6265	0.00020124	0.0024984	DOWN	
transcript_HQ_CoA_transcript18334/f5p0/2330	8.81	3.826666667	6.47	-1.049	0.00012898	0.00087822	DOWN	0.60639	0.015396	0.056192		-0.45447	0.082278	0.2237		
transcript_HQ_CoA_transcript18344/f2p0/2336	6.293333333	0.536666667	3.533333333	-3.3897	0.00058953	0.0032463	DOWN	2.5959	0.0021132	0.011586	UP	-0.81221	0.41303	0.62686		
transcript_HQ_CoA_transcript18351/f2p0/2323	2.843333333	11.69333333	10.84666667	2.1997	5.2131E-17	2.6255E-15	UP	-0.26225	0.59302	0.75286		1.9214	0.00020244	0.0025089	UP	
transcript_HQ_CoA_transcript18353/f3p0/2356	6.013333333	12.09	7.416666667	1.175	0.00054044	0.003016	UP	-0.87028	0.0075322	0.032197		0.29312	0.268	0.48015		
transcript_HQ_CoA_transcript18354/f2p0/2325	6.863333333	16.81333333	9.106666667	1.453	4.9582E-06	5.0053E-05	UP	-1.0418	0.0019329	0.010801	DOWN	0.39805	0.2086	0.41171		
transcript_HQ_CoA_transcript18359/f2p0/2350	1.356666667	5.336666667	3.01	2.1758	0.0022841	0.010357	UP	-1.0112	0.094492	0.21904		1.1495	0.024241	0.095194		
transcript_HQ_CoA_transcript18363/f3p0/2338	6.276666667	15.11	7.673333333	1.449	0.0012679	0.0063005	UP	-1.1358	0.017426	0.06177		0.29792	0.40251	0.61763		
transcript_HQ_CoA_transcript18369/f2p0/2332	4.05	1.29	1.646666667	-1.5121	9.0255E-05	0.00064505	DOWN	0.21494	0.59555	0.75421		-1.3098	0.00043695	0.0046437	DOWN	
transcript_HQ_CoA_transcript18378/f2p0/2326	16.43	53.7	31.77333333	1.8784	1.0981E-12	3.4913E-11	UP	-0.91327	0.00044686	0.0033254		0.95129	1.5149E-05	0.00029964		
transcript_HQ_CoA_transcript18380/f2p0/2333	38.42	11.48	18.75666667	-1.6054	1.3322E-05	0.00012045	DOWN	0.5715	0.030207	0.093635		-1.043	0.002995	0.020721	DOWN	
transcript_HQ_CoA_transcript18392/f2p0/2326	0.073333333	245.3	8.2	11.818	2.5684E-64	1.204E-61	UP	-5.0486	0.0057319	0.026108	DOWN	6.7488	0.0058689	0.034516	UP	
transcript_HQ_CoA_transcript18415/f12p0/2272	22.01333333	55.58666667	30.61	1.5075	3.1108E-06	3.2742E-05	UP	-1.0162	0.00030511	0.0024126	DOWN	0.47712	0.034124	0.12238		
transcript_HQ_CoA_transcript18417/f2p0/2334	9.14	18.33333333	16.43666667	1.167	1.6138E-05	0.00014198	UP	-0.30794	0.15112	0.30644		0.84562	0.00011291	0.0015701		
transcript_HQ_CoA_transcript18418/f3p0/2329	10.33	23.95333333	12.82	1.3824	0.00055385	0.0030767	UP	-1.0703	0.0032284	0.016465	DOWN	0.29987	0.22215	0.42859		
transcript_HQ_CoA_transcript18426/f2p0/2368	2.4	88.66	10.50333333	5.3417	1.2882E-26	1.2662E-24	UP	-3.2015	5.1275E-05	0.00055219	DOWN	2.1285	0.019791	0.082597		
transcript_HQ_CoA_transcript18448/f4p0/2324	7.656666667	19.99333333	6.103333333	1.5387	0.0099722	0.035155	UP	-1.8603	0.0015921	0.0092524	DOWN	-0.33638	0.32696	0.54379		
transcript_HQ_CoA_transcript18457/f2p0/2326	45.30666667	15.64	37.97666667	-1.3993	0.00030193	0.0018284	DOWN	1.1373	0.00013637	0.0012441	UP	-0.26985	0.48811	0.69065		

transcript_HQ_CoA_transcript18460/f2p0/2315	3.52	10.21	15.93666667	1.6793	4.7627E-06	4.8266E-05	UP	0.50408	0.14944	0.30382		2.1719	5.1494E-10	3.9382E-08	UP
transcript_HQ_CoA_transcript18465/f3p0/2322	13.27666667	4.246666667	6.056666667	-1.536	0.013752	0.045446	DOWN	0.38427	0.53185	0.70344		-1.1602	0.0070411	0.039022	DOWN
transcript_HQ_CoA_transcript18475/f2p0/2347	113.5133333	212.5133333	164.5766667	1.0657	1.776E-06	1.9822E-05	UP	-0.51876	0.0018482	0.010426		0.53344	0.0094902	0.0484	
transcript_HQ_CoA_transcript18486/f2p0/2319	8.61	1.65	5.793333333	-2.2352	4.588E-09	8.4214E-08	DOWN	1.6696	1.4725E-08	4.424E-07	UP	-0.57738	0.071689	0.20385	
transcript_HQ_CoA_transcript1850/f2p0/5017	4.33	8.053333333	9.63	1.0456	6.4536E-06	6.3706E-05	UP	0.11241	0.65922	0.79976		1.1456	6.5065E-05	0.0010019	UP
transcript_HQ_CoA_transcript18508/f4p0/2320	6.083333333	23.59333333	9.37	2.1266	3.8968E-11	1.0088E-09	UP	-1.496	9.9567E-07	0.00001903	DOWN	0.61634	0.020644	0.085076	
transcript_HQ_CoA_transcript18517/f2p0/2317	10.89666667	3.926666667	8.253333333	-1.309	4.9523E-05	0.00038089	DOWN	0.91183	0.0015563	0.0090845		-0.40887	0.097296	0.25224	
transcript_HQ_CoA_transcript18527/f2p0/2309	8.053333333	1.99	5.12	-1.8529	9.5644E-07	1.1303E-05	DOWN	1.1965	0.0013883	0.0082993	UP	-0.66827	0.025912	0.10017	
transcript_HQ_CoA_transcript18557/f2p0/2312	4.103333333	8.51	8.913333333	1.2076	0.0052335	0.020625	UP	-0.080495	0.73774	0.85194		1.1148	0.0057	0.033684	UP
transcript_HQ_CoA_transcript18573/f2p0/2333	8.933333333	45.76666667	23.95666667	2.5297	5.2887E-19	3.0792E-17	UP	-1.0777	0.00075058	0.0050653	DOWN	1.4385	0.00002518	0.00045625	UP
transcript_HQ_CoA_transcript18574/f5p0/2276	6.903333333	0.473333333	15.66333333	-3.7866	0.00020179	0.0012958	DOWN	4.9539	5.6626E-09	1.8667E-07	UP	1.1602	0.022757	0.090787	
transcript_HQ_CoA_transcript18621/f3p0/2319	24.69	7.003333333	29.11333333	-1.6688	8.4428E-13	2.7271E-11	DOWN	1.9182	4.6674E-11	2.3896E-09	UP	0.23831	0.34099	0.55889	
transcript_HQ_CoA_transcript18626/f2p0/2310	44.48333333	87.4	58.23333333	1.1336	0.00022431	0.001419	UP	-0.74248	0.010636	0.042097		0.3792	0.01321	0.061484	
transcript_HQ_CoA_transcript1865/f2p0/4970	7.173333333	2.593333333	4.066666667	-1.3072	0.0078722	0.028888	DOWN	0.50472	0.21587	0.39249		-0.81676	0.094754	0.2474	
transcript_HQ_CoA_transcript18654/f7p0/2292	11.98333333	4.483333333	10.80666667	-1.2959	0.010318	0.036184	DOWN	1.1465	0.033377	0.10092		-0.15857	0.63393	0.79654	
transcript_HQ_CoA_transcript18663/f2p0/2328	12.71	4.703333333	5.603333333	-1.2614	0.0011987	0.0059991	DOWN	0.089637	0.80129	0.89057		-1.1845	0.0021852	0.016364	DOWN
transcript_HQ_CoA_transcript18667/f2p0/2300	17.72	5.2	15.05666667	-1.6315	1.5212E-05	0.00013483	DOWN	1.3892	0.0004246	0.0031883	UP	-0.25346	0.48866	0.69098	
transcript_HQ_CoA_transcript18669/f3p0/2308	22.91	5.196666667	8.34	-1.9906	2.1368E-06	0.00002342	DOWN	0.52505	0.084341	0.2015		-1.4775	0.00045547	0.0048074	DOWN
transcript_HQ_CoA_transcript18678/f3p0/2310	3.403333333	16.17	6.276666667	2.3611	0.00017984	0.0011742	UP	-1.4798	0.0061834	0.027697	DOWN	0.87318	0.050942	0.16206	
transcript_HQ_CoA_transcript18686/f2p0/2306	10.70333333	21.11	11.03	1.1559	0.0064035	0.024333	UP	-1.0945	0.0039797	0.019588	DOWN	0.048611	0.89418	0.953	
transcript_HQ_CoA_transcript18691/f2p0/2257	6.44	1.906666667	2.983333333	-1.6123	0.0017663	0.0083592	DOWN	0.49044	0.13167	0.27767		-1.1306	0.016209	0.071037	
transcript_HQ_CoA_transcript18702/f2p0/2291	0.316666667	231.36	17.04	9.7262	8.0787E-17	4.0134E-15	UP	-3.9075	0.044201	0.12424		5.7958	0.032463	0.11798	
transcript_HQ_CoA_transcript18707/f2p0/2317	2.103333333	12.13	4.943333333	2.7153	0.00028215	0.0017289	UP	-1.4563	0.022185	0.074324		1.242	0.02148	0.087519	
transcript_HQ_CoA_transcript18713/f2p0/2306	23.67	8.203333333	13.17666667	-1.3802	1.2267E-10	2.9586E-09	DOWN	0.53401	0.0049292	0.023172		-0.85765	1.2179E-05	0.00024917	
transcript_HQ_CoA_transcript18718/f2p0/2312	27.61	9.25	22.74333333	-1.4224	1.7245E-09	3.4231E-08	DOWN	1.151	3.632E-08	9.9656E-07	UP	-0.28391	0.25138	0.46187	
transcript_HQ_CoA_transcript1872/f2p0/5043	3.77	7.25	3.013333333	1.0862	0.00021747	0.0013839	UP	-1.4104	9.3245E-14	8.6317E-12	DOWN	-0.33317	0.28482	0.49898	
transcript_HQ_CoA_transcript18754/f2p0/2315	15.32	4.226666667	27.12333333	-1.6923	2.0458E-05	0.00017518	DOWN	2.5243	1.2496E-15	1.5587E-13	UP	0.81879	0.03146	0.11548	
transcript_HQ_CoA_transcript18762/f15p0/2290	38.79666667	108.24	37.46333333	1.6503	8.0113E-08	1.1652E-06	UP	-1.6868	3.5378E-08	9.7803E-07	DOWN	-0.050289	0.85171	0.93241	
transcript_HQ_CoA_transcript1877/f2p0/5010	5.436666667	1.64	3.233333333	-1.5818	0.00020961	0.0013409	DOWN	0.82375	0.02392	0.078799		-0.76701	0.084199	0.22774	
transcript_HQ_CoA_transcript18784/f2p0/2291	16.04666667	31.05666667	21.54666667	1.1341	0.0007233	0.0038721	UP	-0.69297	0.019673	0.067819		0.4272	0.053239	0.16759	
transcript_HQ_CoA_transcript18793/f4p0/2271	0	75.43	6.83	13.281	3.0982E-26	2.9969E-24	UP	-3.6046	0.052265	0.14136		9.6609	0.013416	0.062107	
transcript_HQ_CoA_transcript18799/f2p0/2297	8.193333333	17.67	12.12	1.3016	0.0089879	0.032295	UP	-0.71814	0.12288	0.26464		0.56937	0.048214	0.15619	
transcript_HQ_CoA_transcript18815/f3p0/2301	16.46	6.313333333	14.33333333	-1.2207	0.0016652	0.0079573	DOWN	1.0284	0.013183	0.049886	UP	-0.20539	0.49682	0.69836	
transcript_HQ_CoA_transcript18821/f3p0/2307	4.536666667	40.01333333	6.04	3.2908	1.7354E-09	3.4411E-08	UP	-2.8597	9.0811E-08	2.2615E-06	DOWN	0.41836	0.14162	0.32113	
transcript_HQ_CoA_transcript18822/f2p0/2265	14.23333333	26.26	13.85	1.0555	0.00024757	0.001541	UP	-1.0756	0.00016472	0.0014585	DOWN	-0.034634	0.8958	0.95363	
transcript_HQ_CoA_transcript18824/f2p0/2321	2.21	31.02	3.323333333	3.9628	1.6235E-11	4.4701E-10	UP	-3.3718	4.8941E-10	2.0717E-08	DOWN	0.5807	0.18953	0.38714	
transcript_HQ_CoA_transcript18835/f4p0/2292	1.89	8.523333333	6.333333333	2.3496	1.1962E-09	2.4423E-08	UP	-0.59989	0.19036	0.35981		1.7385	0.00043387	0.0046167	UP
transcript_HQ_CoA_transcript18836/f3p0/2288	5.376666667	22.37	6.27	2.221	3.7871E-07	4.8014E-06	UP	-2.0068	8.7525E-07	1.7048E-05	DOWN	0.20179	0.70405	0.8443	
transcript_HQ_CoA_transcript18837/f2p0/2283	1.29	11.03666667	2.116666667	3.2702	4.1631E-13	1.3914E-11	UP	-2.5374	2.0383E-08	5.9055E-07	DOWN	0.72013	0.14789	0.33028	
transcript_HQ_CoA_transcript18839/f2p0/2352	1.88	139.2666667	4.966666667	6.3864	3.2359E-37	6.0989E-35	UP	-4.9504	6.9556E-06	9.9446E-05	DOWN	1.4194	0.11202	0.27651	
transcript_HQ_CoA_transcript18846/f2p0/2292	4.626666667	15.03666667	9.686666667	1.8616	6.4594E-11	1.6067E-09	UP	-0.79747	0.018325	0.064171		1.0523	0.0046288	0.028908	UP
transcript_HQ_CoA_transcript18861/f2p0/2280	0.046666667	65.78333333	15.05333333	10.635	2.0093E-32	3.0359E-30	UP	-2.2584	0.085006	0.20269		8.3683	3.0817E-06	8.1155E-05	UP
transcript_HQ_CoA_transcript18864/f2p0/2296	2.583333333	7.94	13.30666667	1.7779	5.8936E-05	0.0004445	UP	0.58178	0.067763	0.17184		2.3483	3.2326E-09	2.0602E-07	UP
transcript_HQ_CoA_transcript18874/f4p0/2291	21.61666667	6.343333333	10.05333333	-1.6378	0.0073798	0.027339	DOWN	0.55683	0.44488	0.63304		-1.09	0.037542	0.13091	
transcript_HQ_CoA_transcript18879/f22p0/2172	14.00666667	33.43666667	12.69333333	1.4178	7.5712E-05	0.00055326	UP	-1.5561	1.0379E-06	1.9604E-05	DOWN	-0.14954	0.61829	0.78532	
transcript_HQ_CoA_transcript18888/f10p0/2167	14.18	125.3133333	23.81666667	3.3122	3.9116E-27	4.0411E-25	UP	-2.5505	1.4053E-14	1.5241E-12	DOWN	0.74774	0.0036247	0.023984	
transcript_HQ_CoA_transcript18894/f2p0/2315	6.966666667	1.783333333	7.803333333	-1.8364	0.00020979	0.001341	DOWN	1.9992	7.2189E-06	0.00010257	UP	0.1558	0.74541	0.87158	
transcript_HQ_CoA_transcript18899/f2p0/2293	19.71333333	3.353333333	28.05666667	-2.4039	7.9105E-15	3.2646E-13	DOWN	2.9122	3.1286E-12	2.0853E-10	UP	0.49662	0.16108	0.34899	
transcript_HQ_CoA_transcript18906/f3p0/2266	32.40333333	13.25	20.35333333	-1.1334	1.4102E-05	0.00012625	DOWN	0.46661	0.018667	0.065085		-0.6784	0.0021713	0.01629	
transcript_HQ_CoA_transcript18907/f3p0/2292	18.16666667	43.11	19.30333333	1.4276	0.00017324	0.0011352	UP	-1.3159	3.9121E-06	6.1181E-05	DOWN	0.095088	0.814	0.91332	
transcript_HQ_CoA_transcript18912/f2p0/2297	9.776666667	23.54	9.923333333	1.4309	3.8037E-05	0.00030287	UP	-1.3961	4.9833E-05	0.00054079	DOWN	0.020741	0.95142	0.97889	
transcript_HQ_CoA_transcript18915/f13p0/2238	83.66	235.97	105.2166667	1.6392	2.8574E-06	3.0283E-05	UP	-1.2873	0.0053565	0.024749	DOWN	0.33844	0.34232	0.56046	
transcript_HQ_CoA_transcript18918/f2p0/2316	23.39	42.00666667	27.51	1.0121	0.00019976	0.001285	UP	-0.77618	0.0055959	0.025597		0.22384	0.35586	0.57311	
transcript_HQ_CoA_transcript18919/f7p0/2265	19.07666667	46.46	26.68	1.4662	0.00015038	0.0010041	UP	-0.9612	0.035669	0.10579		0.49032	0.16708	0.35717	
transcript_HQ_CoA_transcript1892/f4p0/4971	6.803333333	1.683333333	3.056666667	-1.8602	2.8676E-05	0.00023679	DOWN	0.70186	0.025078	0.081571		-1.1687	0.0023207	0.017082	DOWN
transcript_HQ_CoA_transcript18921/f2p0/2291	5.056666667	1.613333333	3.27	-1.5004	0.00040123	0.0023443	DOWN	0.86913	0.024076	0.079183		-0.64321	0.080764	0.22078	
transcript_HQ_CoA_transcript1893/f2p0/4970	25.80333333	9.01	23.68	-1.3885	0.0028451	0.012452	DOWN	1.2607	0.0081968	0.034369	UP	-0.13936	0.469	0.67602	
transcript_HQ_CoA_transcript18935/f8p0/2200	20.84333333	6.666666667	15.28333333	-1.4941	4.7954E-07	5.9518E-06	DOWN	1.0603	0.0011305	0.0070576	UP	-0.44416	0.16678	0.35674	
transcript_HQ_CoA_transcript18955/f2p0/2281	14.31	55.73666667	19.19666667	2.1246	4.4748E-07	5.5995E-06	UP	-1.6838	0.00010108	0.00097559	DOWN	0.42686	0.067045	0.19518	
transcript_HQ_CoA_transcript18971/f2p0/2304	5.22	1.543333333	2.56	-1.5875	0.00029342	0.0017875	DOWN	0.55169	0.26885	0.45533		-1.0482	0.011899	0.057025	
transcript_HQ_CoA_transcript18972/f13p0/2266	96.02666667	225.2233333	73.07666667	1.3911	3.0887E-13	1.0496E-11	UP	-1.7737	1.7858E-17	2.8966E-15	DOWN	-0.39435	0.036967	0.12947	
transcript_HQ_CoA_transcript18974/f2p0/2289	1.733333333	66.40666667	10.63	5.4394	9.4049E-22	6.5878E-20	UP	-2.7961	1.462E-07	3.4847E-06	DOWN	2.6272	3.0007E-11	3.0252E-09	UP
transcript_HQ_CoA_transcript18977/f3p0/2273	0	61.62	1.906666667	12.958	5.6496E-26	5.2697E-24	UP	-5.1569	0.0010172	0.0064758	DOWN	7.7852	0.0464	0.15214	
transcript_HQ_CoA_transcript18979/f4p0/2265	3.723333333	1.6	16.66666667	-1.0628	0.014578	0.0475	DOWN	3.2342	4.2589E-16	5.4973E-14	UP	2.16	3.7317E-08	1.8265E-06	UP
transcript_HQ_CoA_transcript18990/f2p0/2285	1.603333333	32.98666667	2.613333333	4.5347	1.134E-27	1.2718E-25	UP	-3.8054	1.4126E-11	8.0408E-10	DOWN	0.7153	0.26811	0.48025	
transcript_HQ_CoA_transcript18998/f3p0/2276	10.29333333	21.81666667	16.83	1.2343	0.010766	0.037413	UP	-0.50026	0.22791	0.40718		0.71961	0.041038	0.13972	
transcript_HQ_CoA_transcript19000/f3p0/2295	3.973333333	33.37666667	5.456666667	3.2462	1.1235E-18	6.3592E-17	UP	-2.7809	1.5587E-17	2.5739E-15	DOWN	0.45072	0.22157	0.42787	
transcript_HQ_CoA_transcript19008/f13p0/2224	78.85333333	33.74333333	56.88666667	-1.071	2.338E-12	7.1477E-11	DOWN	0.60074	1.7568E-05	0.00021787		-0.48231	0.0023446	0.017207	
transcript_HQ_CoA_transcript19013/f6p0/2276	5.71	11.07	7.62	1.1191	0.0083227	0.030292	UP	-0.68656	0.02468	0.080576		0.41575	0.34832	0.56625	
transcript_HQ_CoA_transcript19017/f2p0/2306	17.78666667	39.43666667	22.92333333	1.3098	3.3653E-05	0.0002726	UP	-0.93569	0.0013947	0.0083322		0.36129	0.12594	0.29801	
transcript_HQ_CoA_transcript19034/f3p0/2270	52.19333333	93.95666667	94.78333333	1.0085	6.7834E-08	1.0025E-06	UP	-0.13532	0.38729	0.57905		0.86038	1.4043E-06	4.2116E-05	
transcript_HQ_CoA_transcript19038/f2p0/2273	4.63	16.47333333	9.78	1.9977	9.1057E-12	2.593E-10	UP	-0.91273	0.00049758	0.0036393		1.0722	3.4681E-05	0.00059885	UP
transcript_HQ_CoA_transcript19048/f2p0/2270	8.923333333	2.186666667	4.843333333	-1.8689	4.4947E-06	4.5779E-05	DOWN	0.97737	0.022124	0.074187		-0.90353	0.011377	0.055363	
transcript_HQ_CoA_transcript19054/f2p0/2272	10.37666667	30.16	13.54	1.7041	1.8673E-08	3.0672E-07	UP	-1.3148	4.754E-06	7.1953E-05	DOWN	0.37657	0.042309	0.14267	
transcript_HQ_CoA_transcript19058/f4p0/2265	15.82	29.52	13.84333333	1.0574	0.0029419	0.012772	UP	-1.254	4.0694E-05	0.00045369	DOWN	-0.20849	0.47717	0.68206	
transcript_HQ_CoA_transcript19071/f3p0/2293	19.09333333	7.646666667	13.55	-1.1546	2.1625E-07	2.8857E-06	DOWN	0.6631	0.006075	0.027331		-0.50363	0.023011	0.091501	
transcript_HQ_CoA_transcript19074/f2p0/2281	11.40666667	2.643333333	1.156666667	-1.8974	0.0087763	0.031617	DOWN	-1.3931	0.088763	0.20931		-3.3017	2.8017E-23	1.9046E-20	DOWN
transcript_HQ_CoA_transcript19104/f10p0/2248	2.923333333	36.14	24.29666667	3.8144	4.2809E-09	7.9214E-08	UP	-0.73327	0.12075	0.26109		3.0652	1.5499E-07	6.3536E-06	UP
transcript_HQ_CoA_transcript19106/f2p0/2277	38.95	1.883333333	115.1333333	-4.1873	8.7751E-08	1.2642E-06	DOWN	5.7355	9.3288E-28	5.6996E-25	UP	1.5402	0.0074081	0.040481	UP

transcript_HQ_CoA_transcript19109/f6p0/2262	17.59333333	37.17333333	19.78333333	1.2283	0.0050252	0.019933	UP	-1.0536	0.011337	0.044267	DOWN	0.16175	0.73811	0.86671	
transcript_HQ_CoA_transcript19133/f3p0/2288	10.50333333	18.93666667	14.16	1.0158	0.0077592	0.028554	UP	-0.56699	0.19881	0.3708		0.43523	0.20997	0.41366	
transcript_HQ_CoA_transcript19134/f2p0/2267	14.63333333	4.216666667	8.336666667	-1.6532	1.3357E-05	0.00012071	DOWN	0.84036	0.015891	0.057562		-0.82401	0.0060732	0.035352	
transcript_HQ_CoA_transcript19139/f4p0/2243	3.826666667	54.56666667	8.626666667	4.0002	4.9277E-78	4.5044E-75	UP	-2.8053	1.6071E-17	2.6301E-15	DOWN	1.1811	0.0037159	0.024473	UP
transcript_HQ_CoA_transcript1914/f8p0/4943	5.2	1.776666667	3.343333333	-1.3747	0.0013971	0.0068475	DOWN	0.76255	0.029404	0.091876		-0.62454	0.099513	0.25607	
transcript_HQ_CoA_transcript19141/f2p0/2279	1.4	14.17333333	9.46	3.5513	1.0087E-05	9.4381E-05	UP	-0.72055	0.45548	0.6423		2.8129	0.0071084	0.039276	UP
transcript_HQ_CoA_transcript19155/f2p0/2264	139.1766667	8.19	47.32666667	-3.9516	2.2597E-09	4.3949E-08	DOWN	2.3754	4.3405E-09	1.4788E-07	UP	-1.5822	0.079271	0.21815	
transcript_HQ_CoA_transcript19157/f3p0/2267	0.933333333	6.12	0.74	2.9085	0.00005406	0.00041266	UP	-3.2493	2.2423E-05	0.0002695	DOWN	-0.35009	0.62878	0.7932	
transcript_HQ_CoA_transcript19163/f3p0/2294	13.09333333	5.096666667	5.776666667	-1.2116	0.00018349	0.0011934	DOWN	0.038866	0.88512	0.93842		-1.1844	3.5177E-05	0.00060486	DOWN
transcript_HQ_CoA_transcript19165/f2p0/2262	15.44	4.31	6.32	-1.7183	0.0042479	0.017343	DOWN	0.42479	0.5015	0.67876		-1.3018	4.1371E-05	0.00068349	DOWN
transcript_HQ_CoA_transcript19169/f3p0/2267	9.573333333	1.186666667	9.456666667	-2.9158	0.0014264	0.0069596	DOWN	2.899	0.0012687	0.0077152	UP	-0.026681	0.91848	0.96435	
transcript_HQ_CoA_transcript19172/f2p0/2267	7.37	17.73333333	7.823333333	1.4109	0.0036826	0.015463	UP	-1.3367	1.786E-06	3.1296E-05	DOWN	0.064141	0.89952	0.95554	
transcript_HQ_CoA_transcript19174/f2p0/2266	51.36	11.56	13.30333333	-2.0147	1.3691E-07	1.8991E-06	DOWN	0.061332	0.78063	0.87721		-1.9616	3.324E-08	1.6579E-06	DOWN
transcript_HQ_CoA_transcript19191/f2p0/2277	5.076666667	14.81333333	11.00333333	1.7252	0.00012306	0.00084229	UP	-0.60064	0.12902	0.27392		1.1122	6.3698E-05	0.00098581	UP
transcript_HQ_CoA_transcript19197/f2p0/2274	1.36	5.5	3.153333333	2.1946	2.4248E-08	3.9369E-07	UP	-0.94962	0.029613	0.092389		1.2326	0.017161	0.074046	
transcript_HQ_CoA_transcript19198/f3p0/2310	27.58666667	10.93	13.12666667	-1.1901	7.9911E-08	1.1639E-06	DOWN	0.12134	0.56301	0.7296		-1.0797	6.7649E-07	2.2535E-05	DOWN
transcript_HQ_CoA_transcript19201/f2p0/2250	1.526666667	84.27	8	5.9546	4.5458E-38	8.7481E-36	UP	-3.5486	4.725E-08	1.268E-06	DOWN	2.3921	0.0003332	0.0037406	UP
transcript_HQ_CoA_transcript19206/f2p0/2268	10.31333333	3.743333333	6.116666667	-1.2934	8.5483E-06	8.1893E-05	DOWN	0.53597	0.19411	0.36499		-0.7696	0.029729	0.11107	
transcript_HQ_CoA_transcript19214/f4p0/2259	0.576666667	6755.223333	366.35	13.677	1.171E-155	3.568E-152	UP	-4.3612	0.014647	0.054061		9.2982	6.2827E-07	2.1082E-05	UP
transcript_HQ_CoA_transcript1924/f3p0/4965	2.293333333	9.766666667	1.56	2.269	2.8181E-06	2.9902E-05	UP	-2.8261	2.0243E-10	9.1838E-09	DOWN	-0.57027	0.12257	0.2929	
transcript_HQ_CoA_transcript19261/f5p0/2217	11.63333333	28.44666667	16.34666667	1.4524	1.1846E-06	1.3733E-05	UP	-0.94481	0.00019336	0.0016749		0.49398	0.043377	0.1451	
transcript_HQ_CoA_transcript19266/f2p0/2264	19.14666667	7.67	17.88333333	-1.1615	3.6236E-07	4.6197E-06	DOWN	1.0664	1.1615E-08	3.6023E-07	UP	-0.10724	0.56121	0.74552	
transcript_HQ_CoA_transcript19271/f3p0/2254	10.97	34.80666667	7.723333333	1.8302	0.0004025	0.002351	UP	-2.3315	5.0378E-06	7.5255E-05	DOWN	-0.51395	0.17209	0.36399	
transcript_HQ_CoA_transcript19272/f2p0/2206	2.873333333	21.52666667	17.66333333	3.0778	8.729E-16	3.9114E-14	UP	-0.42084	0.57846	0.74144		2.6428	0.00027702	0.0032493	UP
transcript_HQ_CoA_transcript19276/f3p0/2276	87.51	29.70333333	18.32	-1.4325	0.0016745	0.0079964	DOWN	-0.81681	0.082666	0.19853		-2.2622	6.8048E-10	5.0161E-08	DOWN
transcript_HQ_CoA_transcript19281/f2p0/2279	5.123333333	24.23666667	7.62	2.4114	5.4713E-08	8.2598E-07	UP	-1.8345	1.3613E-05	0.00017646	DOWN	0.56516	0.031894	0.11648	
transcript_HQ_CoA_transcript1929/f3p0/4953	12.01666667	24.16	12.67333333	1.1674	7.2951E-07	0.00000878	UP	-1.0812	3.0682E-06	4.9811E-05	DOWN	0.074091	0.73785	0.8666	
transcript_HQ_CoA_transcript19296/f3p0/2251	7.56	165.1966667	30.74666667	4.6311	9.3802E-46	2.3174E-43	UP	-2.5781	4.0759E-13	3.3652E-11	DOWN	2.0385	9.3124E-10	6.6252E-08	UP
transcript_HQ_CoA_transcript193/f2p0/7287	2.18	4.51	2.496666667	1.2117	0.013792	0.045537	UP	-0.99833	3.4398E-07	7.5146E-06	DOWN	0.19999	0.68384	0.83114	
transcript_HQ_CoA_transcript19308/f8p0/2200	36.86333333	8.906666667	31.64333333	-1.8918	1.4777E-19	8.7426E-18	DOWN	1.6612	3.5617E-05	0.00040498	UP	-0.24469	0.50364	0.70401	
transcript_HQ_CoA_transcript19310/f2p0/2261	0.016666667	17.56333333	3.063333333	10.192	3.3976E-14	1.2968E-12	UP	-2.642	0.13986	0.29024		7.5348	0.00018547	0.0023526	UP
transcript_HQ_CoA_transcript19315/f2p0/2264	6.466666667	14.29	8.856666667	1.3226	0.0028804	0.01258	UP	-0.84058	0.071834	0.17958		0.46586	0.40039	0.61593	
transcript_HQ_CoA_transcript19330/f3p0/2250	6.233333333	12.52	12.55333333	1.1871	0.014	0.046083	UP	-0.16424	0.75748	0.86374		1.0085	0.0091569	0.047146	UP
transcript_HQ_CoA_transcript19332/f3p0/2251	11.55	2.616666667	12.44333333	-1.9899	1.4319E-08	2.3928E-07	DOWN	2.1005	1.7017E-06	2.9962E-05	UP	0.098811	0.80728	0.90928	
transcript_HQ_CoA_transcript19342/f2p0/2252	2.183333333	12.92666667	2.916666667	2.7252	1.4033E-07	1.9406E-06	UP	-2.2954	4.2392E-05	0.00046892	DOWN	0.41437	0.39897	0.61472	
transcript_HQ_CoA_transcript19345/f2p0/2254	11.02333333	261.3733333	43.51333333	4.7365	1.8721E-56	6.338E-54	UP	-2.7357	7.7084E-19	1.503E-16	DOWN	1.9886	2.1349E-11	2.2265E-09	UP
transcript_HQ_CoA_transcript19353/f8p0/2210	38.54666667	16.78	18.85333333	-1.0313	0.0010532	0.0053739	DOWN	0.013813	0.96273	0.97995		-1.0308	6.6013E-05	0.0010123	DOWN
transcript_HQ_CoA_transcript19357/f5p0/2245	3.726666667	17.18666667	7.116666667	2.3751	1.3009E-08	2.1961E-07	UP	-1.4216	0.00034403	0.0026629	DOWN	0.93904	0.0046767	0.029112	
transcript_HQ_CoA_transcript19359/f2p0/2272	5.373333333	12.44666667	4.906666667	1.3684	0.00023792	0.0014916	UP	-1.503	0.00007339	0.00074277	DOWN	-0.14609	0.62267	0.78849	
transcript_HQ_CoA_transcript19372/f4p0/2256	285.1333333	92.78	98.99	-1.4735	7.4729E-08	1.0956E-06	DOWN	-0.032219	0.91347	0.95369		-1.5177	1.154E-09	8.015E-08	DOWN
transcript_HQ_CoA_transcript19375/f2p0/2300	18.79666667	6.493333333	9.486666667	-1.3816	1.3546E-10	3.2372E-09	DOWN	0.39785	0.059332	0.15589		-0.99557	1.6802E-08	8.965E-07	
transcript_HQ_CoA_transcript19380/f4p0/2250	1.73	5.43	5.146666667	1.8177	4.1303E-06	4.2256E-05	UP	-0.23479	0.61554	0.76917		1.5726	0.0015145	0.012305	UP
transcript_HQ_CoA_transcript19388/f2p0/2250	0.843333333	30.43	4.253333333	5.3673	9.8219E-20	5.9458E-18	UP	-2.9892	1.3305E-05	0.00017284	DOWN	2.3606	0.0042761	0.027187	UP
transcript_HQ_CoA_transcript19393/f2p0/2248	1.94	6.033333333	4.776666667	1.8009	5.4275E-07	6.6505E-06	UP	-0.49766	0.21654	0.39332		1.2882	0.0081035	0.043226	UP
transcript_HQ_CoA_transcript19416/f2p0/2252	23.47666667	9.853333333	25.01333333	-1.0901	0.00053052	0.0029706	DOWN	1.1782	0.0038242	0.018965	UP	0.076888	0.84045	0.92685	
transcript_HQ_CoA_transcript19429/f3p0/2255	29.56666667	7.9	23.97	-1.7546	7.2836E-06	0.00007098	DOWN	1.4495	3.9351E-11	2.0666E-09	UP	-0.31512	0.38536	0.60087	
transcript_HQ_CoA_transcript19431/f3p0/2296	8.38	0.116666667	2.193333333	-5.9619	7.7916E-07	9.3285E-06	DOWN	4.0076	0.0015205	0.0089095	UP	-1.9587	0.029423	0.11019	
transcript_HQ_CoA_transcript19447/f3p0/2248	14.4	31.6	18.95666667	1.3012	9.0366E-08	1.2998E-06	UP	-0.88905	0.0020255	0.011223		0.3989	0.11387	0.27936	
transcript_HQ_CoA_transcript19457/f6p0/2194	21.71	102.7466667	22.46666667	2.4149	2.9377E-12	8.8335E-11	UP	-2.3603	3.3926E-12	2.2271E-10	DOWN	0.041849	0.78514	0.89579	
transcript_HQ_CoA_transcript19459/f25p0/2153	712.42	138.6966667	159.6166667	-2.2441	0.00051931	0.0029185	DOWN	0.066806	0.85063	0.91875		-2.1826	0.0085195	0.04491	DOWN
transcript_HQ_CoA_transcript19460/f4p0/2236	6.573333333	12.16666667	7.023333333	1.0436	0.00010814	0.000753	UP	-0.93646	2.5457E-05	0.00030046		0.096455	0.7588	0.87961	
transcript_HQ_CoA_transcript19468/f2p0/2300	6.586666667	23.93666667	8.15	2.0301	8.9705E-05	0.00064162	UP	-1.7019	0.0023915	0.012839	DOWN	0.31329	0.40496	0.61934	
transcript_HQ_CoA_transcript19470/f2p0/2262	35.31333333	15.65666667	31.82	-1.0011	0.00041957	0.0024313	DOWN	0.85596	0.0040512	0.019891		-0.15755	0.54499	0.73466	
transcript_HQ_CoA_transcript19488/f2p0/2240	1.566666667	26.82333333	3.356666667	4.2467	3.6646E-06	3.8023E-05	UP	-3.109	6.0372E-07	1.2391E-05	DOWN	1.1222	0.22715	0.43452	
transcript_HQ_CoA_transcript19492/f2p0/2242	0.78	7.453333333	1.876666667	3.4139	3.8983E-06	4.0128E-05	UP	-2.152	1.7907E-09	6.6039E-08	DOWN	1.2464	0.13852	0.31676	
transcript_HQ_CoA_transcript19508/f3p0/2224	2.2	0.51	1.32	-1.9237	0.00074057	0.0039507	DOWN	1.1995	0.038746	0.11229		-0.73859	0.16925	0.36002	
transcript_HQ_CoA_transcript19511/f4p0/2227	35.85	10.78333333	35.48333333	-1.5917	9.0582E-05	0.00064688	DOWN	1.566	5.2575E-07	1.1038E-05	UP	-0.03618	0.92626	0.96725	
transcript_HQ_CoA_transcript19513/f2p0/2242	1.996666667	17.04	13.46333333	3.235	4.1424E-09	7.6884E-08	UP	-0.47931	0.14499	0.29744		2.7457	2.6031E-07	9.9959E-06	UP
transcript_HQ_CoA_transcript19520/f2p0/2258	32.50666667	13.21	24.78	-1.1208	0.0021073	0.0096845	DOWN	0.73657	0.039878	0.11473		-0.39698	0.0057046	0.033696	
transcript_HQ_CoA_transcript19525/f2p0/2239	1.716666667	7.64	4.636666667	2.2973	0.00025433	0.0015772	UP	-0.85756	0.063787	0.16455		1.4251	0.0054326	0.032459	UP
transcript_HQ_CoA_transcript19529/f7p0/2206	2.906666667	5.823333333	4.776666667	1.1699	0.0035084	0.014847	UP	-0.45833	0.19801	0.37001		0.69945	0.047559	0.15478	
transcript_HQ_CoA_transcript19534/f3p0/2261	1.413333333	46.36333333	15.63	5.2084	3.4484E-26	3.3007E-24	UP	-1.6898	0.019691	0.067831		3.5019	2.7838E-05	0.00049657	UP
transcript_HQ_CoA_transcript19554/f2p0/2228	23.45333333	7.92	13.73	-1.4216	1.9781E-07	2.661E-06	DOWN	0.64866	0.0012052	0.0074054		-0.78435	0.0012424	0.010616	
transcript_HQ_CoA_transcript19560/f2p0/2234	21.91666667	5.376666667	20.57	-1.8791	2.9396E-11	7.7662E-10	DOWN	1.7904	3.2351E-07	7.1099E-06	UP	-0.10027	0.74661	0.87239	
transcript_HQ_CoA_transcript19574/f5p0/2185	5.566666667	18.02666667	6.54	1.8384	1.3612E-05	0.00012253	UP	-1.5931	8.2044E-07	0.00001617	DOWN	0.23501	0.60619	0.77743	
transcript_HQ_CoA_transcript19580/f2p0/2250	0.746666667	10.44	0.82	3.9242	7.5717E-05	0.00055326	UP	-3.8119	9.2985E-05	0.00090752	DOWN	0.10397	0.95019	0.97848	
transcript_HQ_CoA_transcript19615/f3p0/2238	13.98	4.863333333	8.37	-1.3848	4.7441E-05	0.00036673	DOWN	0.64212	0.016984	0.060681		-0.75347	0.0078957	0.04245	
transcript_HQ_CoA_transcript19653/f2p0/2223	2.616666667	7.966666667	2.5	1.7521	0.00065538	0.0035596	UP	-1.8177	0.00009204	0.00089925	DOWN	-0.076612	0.8745	0.94409	
transcript_HQ_CoA_transcript19655/f2p0/2230	5.88	67.20333333	14.35333333	3.6797	2.7391E-42	5.9616E-40	UP	-2.3628	4.5995E-05	0.00050271	DOWN	1.303	0.02632	0.10141	
transcript_HQ_CoA_transcript19656/f5p0/2223	0.47	5.593333333	4.42	3.6834	5.2633E-06	5.2842E-05	UP	-0.45711	0.46976	0.6535		3.2137	3.8181E-05	0.00064118	UP
transcript_HQ_CoA_transcript19666/f4p0/2207	102.54	17.85666667	42.84666667	-2.3719	1.3385E-38	2.6032E-36	DOWN	1.0978	0.0061534	0.027589	UP	-1.2876	0.00039813	0.0042911	DOWN
transcript_HQ_CoA_transcript19667/f7p0/2229	0	82.06666667	13.61666667	13.371	7.1903E-27	7.1832E-25	UP	-2.7385	0.062584	0.16232		10.617	0.00007558	0.001126	UP
transcript_HQ_CoA_transcript19668/f2p0/2240	20.91333333	4.673333333	8.94	-2.0173	2.1441E-08	3.4967E-07	DOWN	0.78555	0.0011162	0.0069848		-1.2423	0.00026332	0.0031182	DOWN
transcript_HQ_CoA_transcript19677/f2p0/2231	16.30333333	6.103333333	7.683333333	-1.2593	5.063E-06	5.0942E-05	DOWN	0.17568	0.56288	0.72948		-1.0946	2.4436E-06	6.7045E-05	DOWN
transcript_HQ_CoA_transcript19681/f3p0/2239	2.706666667	93.08	17.51	5.276	1.0166E-29	1.3468E-27	UP	-2.5307	0.023971	0.078938		2.7272	0.0018432	0.014345	UP
transcript_HQ_CoA_transcript19682/f2p0/2009	3.14	8.7	9.58	1.6475	0.0012906	0.0063995	UP	-0.033687	0.96113	0.9793		1.5982	0.018164	0.077517	
transcript_HQ_CoA_transcript19684/f24p0/2221	170.0133333	22.92333333	112.7566667	-2.7449	1.8447E-15	8.0108E-14	DOWN	2.1369	4.2594E-07	9.1203E-06	UP	-0.62002	0.16764	0.35801	
transcript_HQ_CoA_transcript19719/f3p0/2221	0.81	9.35	2.45	3.683	1.3398E-05	0.00012096	UP	-2.085	0.00032823	0.0025622	DOWN	1.5891	0.064819	0.19091	
transcript_HQ_CoA_transcript1974/f2p0/4934	3.66	7.01	4.863333333	1.1031	0.00031527	0.0019022	UP	-0.676	0.15111	0.30644		0.41351	0.35611	0.57327	

transcript_HQ_CoA_transcript19740/f2p0/2225	3.66	20.27666667	4.31	2.6275	1.2041E-07	1.692E-06	UP	-2.395	8.0178E-07	1.5853E-05	DOWN	0.22017	0.52235	0.71725	
transcript_HQ_CoA_transcript19741/f3p0/2232	32.76333333	13.27	12.14	-1.1537	5.647E-08	8.4901E-07	DOWN	-0.25912	0.37285	0.56506		-1.4243	1.2911E-08	7.2032E-07	DOWN
transcript_HQ_CoA_transcript19746/f2p0/2224	27.09333333	1.723333333	9.706666667	-3.7969	4.2755E-12	1.2567E-10	DOWN	2.3088	0.00034304	0.0026595	UP	-1.4964	0.00089364	0.008185	DOWN
transcript_HQ_CoA_transcript19766/f2p0/2236	20.72333333	38.94	30.47333333	1.0753	0.0002908	0.0017739	UP	-0.50973	0.032956	0.099959		0.55172	0.055085	0.17134	
transcript_HQ_CoA_transcript19767/f2p0/2259	1.13	4.066666667	0.623333333	1.9819	0.00039251	0.0022992	UP	-2.863	1.1678E-12	8.8082E-11	DOWN	-0.89816	0.22881	0.43616	
transcript_HQ_CoA_transcript19772/f2p0/2229	11.13	23.39	11.30666667	1.2396	7.2504E-05	0.00053212	UP	-1.1989	2.5726E-06	4.2867E-05	DOWN	0.027406	0.92653	0.96732	
transcript_HQ_CoA_transcript19785/f2p0/2248	5.213333333	1.5	2.58	-1.6533	0.0018118	0.0085393	DOWN	0.63239	0.28236	0.47113		-1.0285	0.024507	0.095954	
transcript_HQ_CoA_transcript19789/f2p0/2235	4.92	21.00666667	6.59	2.2945	0.0010546	0.0053795	UP	-1.8565	0.0040305	0.019811	DOWN	0.42285	0.35989	0.57692	
transcript_HQ_CoA_transcript19792/f2p0/2214	6.053333333	2.223333333	3.71	-1.3019	0.0022252	0.010135	DOWN	0.58751	0.17229	0.33577		-0.72478	0.036601	0.12858	
transcript_HQ_CoA_transcript19799/f3p0/2230	3.23	10.35333333	1.88	1.8401	1.4109E-07	1.9497E-06	UP	-2.6233	1.6297E-13	1.4293E-11	DOWN	-0.79614	0.045663	0.15064	
transcript_HQ_CoA_transcript19808/f2p0/2217	28.23	8.363333333	32.7	-1.6128	0.0016357	0.0078343	DOWN	1.8309	0.00027266	0.0022064	UP	0.20554	0.45097	0.66108	
transcript_HQ_CoA_transcript19810/f2p0/2212	5.463333333	11.85666667	4.636666667	1.2806	0.0026892	0.011864	UP	-1.5039	0.0010412	0.0065981	DOWN	-0.23719	0.5095	0.70825	
transcript_HQ_CoA_transcript19820/f2p0/2215	12.90333333	2.553333333	18.76666667	-2.2044	4.8535E-11	1.2375E-09	DOWN	2.737	2.3417E-14	2.4249E-12	UP	0.52159	0.084103	0.22758	
transcript_HQ_CoA_transcript19825/f47p0/2167	8.676666667	373.8	75.73333333	5.5819	1.905E-120	3.869E-117	UP	-2.4186	0.022736	0.075783		3.1497	0.0031761	0.021656	UP
transcript_HQ_CoA_transcript19835/f2p0/2221	15.20333333	2.61	5.446666667	-2.425	0.0010609	0.0054022	DOWN	0.92522	0.0064424	0.028519		-1.5062	0.027162	0.10363	
transcript_HQ_CoA_transcript19843/f2p0/2241	3.646666667	12.42	5.283333333	1.8962	0.0040348	0.01667	UP	-1.3726	0.019703	0.067855		0.51272	0.23935	0.44787	
transcript_HQ_CoA_transcript1985/f5p0/4920	21.55666667	44.96666667	28.32	1.2206	8.1086E-05	0.00058663	UP	-0.8276	0.0016199	0.0093875		0.38108	0.24656	0.45585	
transcript_HQ_CoA_transcript19855/f3p0/2219	32.94333333	63.31333333	51.93333333	1.103	1.6096E-06	1.8164E-05	UP	-0.43553	0.076331	0.18764		0.65515	0.0027289	0.019308	
transcript_HQ_CoA_transcript19857/f2p0/2220	15.01666667	5.056666667	22.57	-1.3879	0.012253	0.041507	DOWN	1.9912	0.00042757	0.0032066	UP	0.59263	0.085804	0.23064	
transcript_HQ_CoA_transcript19863/f7p0/2207	59.6	19.39	45.74333333	-1.4652	8.9223E-15	3.6656E-13	DOWN	1.0859	7.6595E-06	0.0001075	UP	-0.39025	0.09731	0.25224	
transcript_HQ_CoA_transcript19877/f2p0/2205	79.25333333	258.75	110.3333333	1.8593	4.4078E-08	6.7888E-07	UP	-1.3597	0.010267	0.040873	DOWN	0.48844	0.36101	0.57802	
transcript_HQ_CoA_transcript19892/f3p0/2202	18.85666667	5.266666667	11.23666667	-1.7123	0.014439	0.047171	DOWN	0.98606	0.2032	0.37685		-0.7405	0.067353	0.19571	
transcript_HQ_CoA_transcript19903/f4p0/2217	21.65333333	2.473333333	13.73	-2.9998	3.6021E-08	5.6285E-07	DOWN	2.3967	0.0032307	0.016471	UP	-0.61614	0.32916	0.54611	
transcript_HQ_CoA_transcript19926/f4p0/2196	15.69	6.233333333	7.773333333	-1.1816	1.6164E-07	2.2069E-06	DOWN	0.17206	0.47426	0.65774		-1.0211	8.5536E-07	2.7544E-05	DOWN
transcript_HQ_CoA_transcript19929/f5p0/2198	10.77333333	58.07	19.06	2.5829	1.3629E-09	2.7471E-08	UP	-1.7561	1.4315E-05	0.00018374	DOWN	0.81513	0.026024	0.10049	
transcript_HQ_CoA_transcript19935/f7p0/2138	42.67666667	15.81666667	33.80666667	-1.2788	5.6988E-13	1.8705E-11	DOWN	0.94493	1.8061E-05	0.00022322		-0.3466	0.097055	0.25185	
transcript_HQ_CoA_transcript19942/f7p0/2198	9.156666667	21.17666667	15.11333333	1.3643	6.0277E-05	0.00045312	UP	-0.63892	0.064629	0.16619		0.71339	0.057065	0.17518	
transcript_HQ_CoA_transcript19943/f2p0/2217	11.42666667	20.98666667	6.966666667	1.0457	0.0051624	0.020396	UP	-1.7409	1.5606E-21	4.8482E-19	DOWN	-0.70843	0.072634	0.20539	
transcript_HQ_CoA_transcript19947/f4p0/2204	3.17	29.83	2.656666667	3.3851	7.7133E-11	1.8903E-09	UP	-3.6273	7.2696E-11	3.6208E-09	DOWN	-0.25428	0.70897	0.84715	
transcript_HQ_CoA_transcript1995/f2p0/4943	4.093333333	9.9	7.16	1.428	7.0455E-05	0.00051875	UP	-0.61483	0.078903	0.19242		0.8015	0.00017823	0.0022702	
transcript_HQ_CoA_transcript19956/f2p0/2205	15.46666667	84.01666667	27.62666667	2.5889	4.8897E-36	8.6789E-34	UP	-1.7412	2.8004E-10	1.255E-08	DOWN	0.83555	0.0051656	0.031292	
transcript_HQ_CoA_transcript19959/f2p0/2199	14.98666667	33.37333333	15.09333333	1.3164	0.00018866	0.0012235	UP	-1.2978	0.00043646	0.0032574	DOWN	0.0046736	0.9879	0.99527	
transcript_HQ_CoA_transcript19970/f3p0/2229	12.01	4.803333333	7.523333333	-1.1612	2.6089E-06	2.7925E-05	DOWN	0.50696	0.10722	0.24008		-0.66566	0.032426	0.1179	
transcript_HQ_CoA_transcript19973/f2p0/2200	5.93	13.73666667	8.473333333	1.3656	2.3582E-06	2.5586E-05	UP	-0.84025	0.0076272	0.032519		0.51246	0.12205	0.29207	
transcript_HQ_CoA_transcript2001/f2p0/5000	21.33333333	9	12.89	-1.0934	9.3356E-08	1.3397E-06	DOWN	0.37713	0.039399	0.11371		-0.72868	8.3112E-06	0.0001827	
transcript_HQ_CoA_transcript20015/f3p0/2209	29.35666667	54.29	46.18666667	1.0417	0.0014272	0.0069596	UP	-0.37144	0.13194	0.27797		0.65682	0.018466	0.078549	
transcript_HQ_CoA_transcript2002/f2p0/4996	7.11	2.61	7.75	-1.3066	0.00015762	0.0010438	DOWN	1.4281	0.00007936	0.00079226	UP	0.1105	0.68336	0.83077	
transcript_HQ_CoA_transcript20027/f2p0/2201	11.97	39.25	19.17333333	1.875	3.0495E-16	1.4222E-14	UP	-1.1876	4.2552E-05	0.00047041	DOWN	0.67299	0.015317	0.068438	
transcript_HQ_CoA_transcript20037/f4p0/2198	22.23	52.11666667	41.94666667	1.3836	4.0353E-12	1.1899E-10	UP	-0.45423	0.018462	0.064528		0.91725	7.4156E-06	0.00016559	
transcript_HQ_CoA_transcript20050/f3p0/2147	9.66	22.86333333	15.36666667	1.412	8.1507E-06	0.00007851	UP	-0.72215	0.081838	0.19719		0.67498	0.07192	0.20427	
transcript_HQ_CoA_transcript20052/f5p0/2200	16.8	30.18333333	18.1	1.0231	0.0017111	0.0081483	UP	-0.90087	0.00037739	0.002887		0.10723	0.72383	0.85804	
transcript_HQ_CoA_transcript20055/f2p0/2195	8.196666667	28.17666667	20.97	1.9329	1.4261E-22	1.0428E-20	UP	-0.57085	0.24155	0.42383		1.3465	0.0051367	0.031138	UP
transcript_HQ_CoA_transcript20070/f3p0/2193	12.57	1.01	17.94	-3.464	1.2235E-09	2.4937E-08	DOWN	3.978	5.1613E-10	2.157E-08	UP	0.50118	0.39309	0.60888	
transcript_HQ_CoA_transcript20086/f2p0/2205	1.03	4.166666667	1.296666667	2.2126	0.015404	0.049502	UP	-1.8426	0.04511	0.12604		0.35393	0.77069	0.88659	
transcript_HQ_CoA_transcript20087/f7p0/2165	17.09	44.46	18.63	1.5257	2.3743E-10	5.4395E-09	UP	-1.3938	1.6846E-05	0.00021076	DOWN	0.11924	0.71843	0.85383	
transcript_HQ_CoA_transcript20090/f2p0/2193	4.636666667	28.45333333	10.94333333	2.83	0.00017827	0.0011652	UP	-1.5026	0.041235	0.11785		1.3079	0.15387	0.33823	
transcript_HQ_CoA_transcript20092/f2p0/2221	22.22666667	7.666666667	19.68333333	-1.3981	0.00005207	0.00039834	DOWN	1.2245	0.00014935	0.0013406	UP	-0.1834	0.51518	0.71243	
transcript_HQ_CoA_transcript20099/f3p0/2186	103.1466667	196.6433333	111.47	1.0868	2.2039E-15	9.5253E-14	UP	-0.96323	7.0824E-07	1.4218E-05		0.11083	0.5568	0.74247	
transcript_HQ_CoA_transcript201/f2p0/7284	3.933333333	1.703333333	4.97	-1.0396	0.00079577	0.0042157	DOWN	1.3784	8.2679E-06	0.00011498	UP	0.3268	0.22912	0.43644	
transcript_HQ_CoA_transcript20136/f3p0/2163	5.53	10.80666667	6.833333333	1.1552	0.013746	0.045436	UP	-0.8341	0.058572	0.15442		0.30772	0.26382	0.47577	
transcript_HQ_CoA_transcript20138/f3p0/2193	6.156666667	17.50333333	12.05666667	1.6643	1.9742E-09	3.8684E-08	UP	-0.68781	0.014114	0.052623		0.96425	0.00054364	0.005518	
transcript_HQ_CoA_transcript20144/f2p0/2191	9.676666667	26.82	13.30333333	1.6447	9.2411E-07	1.0949E-05	UP	-1.1717	0.00017489	0.001536	DOWN	0.45862	0.069409	0.19966	
transcript_HQ_CoA_transcript20159/f2p0/2211	3.086666667	0.506666667	1.72	-2.3969	0.0092995	0.033186	DOWN	1.5386	0.10441	0.23573		-0.86863	0.037999	0.13207	
transcript_HQ_CoA_transcript20162/f3p0/2195	26.41333333	55.33666667	24.44666667	1.2169	5.8887E-08	8.7955E-07	UP	-1.3214	6.3915E-12	3.9712E-10	DOWN	-0.11593	0.65493	0.81137	
transcript_HQ_CoA_transcript20170/f2p0/2224	8.493333333	1.9	2.356666667	-1.9679	0.0070434	0.026338	DOWN	0.1472	0.7506	0.85926		-1.8356	0.0047444	0.02942	DOWN
transcript_HQ_CoA_transcript20185/f2p0/2215	12.86333333	3.116666667	5.6	-1.8856	5.2705E-11	1.3364E-09	DOWN	0.68945	0.0058861	0.026671		-1.2081	1.3605E-06	4.1005E-05	DOWN
transcript_HQ_CoA_transcript20190/f2p0/2194	18.9	34.84666667	25.32	1.0367	0.0002102	0.0013428	UP	-0.60592	0.038591	0.11201		0.41878	0.15039	0.33367	
transcript_HQ_CoA_transcript20193/f7p0/2127	5.343333333	21.60666667	9.83	2.1889	5.083E-08	7.7375E-07	UP	-1.2777	6.1417E-05	0.0006378	DOWN	0.89655	0.046855	0.15328	
transcript_HQ_CoA_transcript20202/f5p0/2192	20.15333333	5.133333333	11.06333333	-1.7982	0.0021036	0.0096774	DOWN	0.95682	0.017935	0.063131		-0.8535	0.12859	0.3016	
transcript_HQ_CoA_transcript20240/f7p0/2153	18.44	54.01666667	28.26	1.7238	2.6215E-11	7.0229E-10	UP	-1.1019	1.9683E-05	0.00024051	DOWN	0.60835	0.0058666	0.034513	
transcript_HQ_CoA_transcript20242/f2p0/2191	1.166666667	17.52333333	1.54	4.0603	5.5583E-09	1.0041E-07	UP	-3.6373	4.9287E-14	4.7547E-12	DOWN	0.40965	0.60256	0.77476	
transcript_HQ_CoA_transcript20246/f3p0/2182	10.55	30.62666667	10.4	1.6917	4.5132E-09	8.3008E-08	UP	-1.699	3.0115E-09	1.0534E-07	DOWN	-0.020094	0.93134	0.97008	
transcript_HQ_CoA_transcript20250/f2p0/2203	2.82	11.6	5.473333333	2.2358	0.00015675	0.0010398	UP	-1.2345	2.7483E-05	0.00032188	DOWN	0.98392	0.092039	0.24252	
transcript_HQ_CoA_transcript20252/f3p0/2181	19.07	7.546666667	10.58333333	-1.1715	0.0031665	0.013586	DOWN	0.33129	0.36766	0.55973		-0.85371	0.012504	0.059056	
transcript_HQ_CoA_transcript20271/f2p0/2193	13.29333333	5.896666667	6.68	-1.0238	0.00013805	0.00093369	DOWN	0.027194	0.93848	0.96756		-1.0096	0.0033379	0.0225	DOWN
transcript_HQ_CoA_transcript20276/f2p0/2220	6.733333333	2.646666667	5.293333333	-1.1848	0.011953	0.04067	DOWN	0.82609	0.043771	0.12324		-0.36935	0.34325	0.56122	
transcript_HQ_CoA_transcript20278/f3p0/2200	68.28	26.42	49.36333333	-1.2205	1.4987E-10	3.5538E-09	DOWN	0.75475	0.00096289	0.0061982		-0.47715	0.016449	0.071835	
transcript_HQ_CoA_transcript20280/f2p0/2159	19.26333333	47.87	21.1	1.4727	3.9708E-25	3.5585E-23	UP	-1.3278	3.3531E-13	2.7936E-11	DOWN	0.13245	0.51746	0.71403	
transcript_HQ_CoA_transcript20283/f22p0/2129	11.65	31.71333333	17.89	1.6027	6.3504E-06	0.00006279	UP	-0.99847	0.0018346	0.010372		0.59195	0.097734	0.25291	
transcript_HQ_CoA_transcript20289/f2p0/2180	0.943333333	16.64	4.13	4.2606	1.7839E-11	4.8823E-10	UP	-2.111	0.0013836	0.0082795	DOWN	2.1371	0.00013258	0.0017907	UP
transcript_HQ_CoA_transcript20303/f3p0/2180	7.94	20.35333333	21.36333333	1.5128	0.00036538	0.0021646	UP	-0.064927	0.83655	0.91068		1.4338	2.5829E-05	0.00046616	UP
transcript_HQ_CoA_transcript20309/f2p0/2182	31.64	10.98666667	12.59333333	-1.3834	7.1861E-06	7.0142E-05	DOWN	0.054151	0.76665	0.8689		-1.3398	2.8978E-06	7.7197E-05	DOWN
transcript_HQ_CoA_transcript20326/f4p0/2178	2.663333333	22.76	2.646666667	3.2592	7.1464E-19	4.0956E-17	UP	-3.2546	7.8515E-17	1.1513E-14	DOWN	-0.0094296	0.9833	0.99354	
transcript_HQ_CoA_transcript20341/f4p0/2163	10.35333333	82.42	11.28333333	3.1713	6.4645E-15	2.6921E-13	UP	-3.0236	4.057E-14	4.0195E-12	DOWN	0.13213	0.70743	0.84625	
transcript_HQ_CoA_transcript20346/f2p0/2186	24.7	61.54	39.17	1.4874	7.3508E-09	1.3009E-07	UP	-0.80739	0.00052279	0.0037794		0.66697	0.0010797	0.0095324	
transcript_HQ_CoA_transcript20350/f5p0/2168	70.13666667	27.48333333	54.89	-1.1758	0.0038735	0.016142	DOWN	0.82677	0.035662	0.10579		-0.36096	0.043933	0.14643	
transcript_HQ_CoA_transcript20360/f5p0/2191	248.4666667	58.42	75.68666667	-1.9707	0.001823	0.0085809	DOWN	0.24119	0.48187	0.66408		-1.7361	0.0026165	0.018666	DOWN
transcript_HQ_CoA_transcript20372/f4p0/2185	29.79666667	12.62666667	27.39333333	-1.0865	1.3403E-07	1.8677E-06	DOWN	0.96522	0.0001239	0.001147		-0.13316	0.58109	0.75961	
transcript_HQ_CoA_transcript20374/f5p0/2151	14.02666667	2.95	7.27	-2.1333	0.00023878	0.0014963	DOWN	1.1964	0.05165	0.14011		-0.94772	0.023293	0.092362	

transcript_HQ_CoA_transcript20376/f2p0/2175	16.33	44.71	18.56333333	1.6144	4.5905E-14	1.7233E-12	UP	-1.4151	1.0213E-06	1.9379E-05	DOWN	0.18642	0.51	0.70849	
transcript_HQ_CoA_transcript20398/f3p0/2175	11.2	23.46666667	18.06333333	1.2216	4.0441E-11	1.0413E-09	UP	-0.51509	0.072972	0.18124		0.69408	0.018427	0.078422	
transcript_HQ_CoA_transcript20419/f2p0/2167	77.69333333	19.01	23.66666667	-1.8766	2.3991E-07	3.1737E-06	DOWN	0.16584	0.56017	0.72716		-1.7212	1.8332E-07	7.3951E-06	DOWN
transcript_HQ_CoA_transcript2042/f6p0/4781	1.906666667	11.48666667	4.66	2.7535	6.5896E-37	1.2169E-34	UP	-1.4425	4.1518E-05	0.00046176	DOWN	1.2972	0.0012041	0.010381	UP
transcript_HQ_CoA_transcript20424/f2p0/2178	8.636666667	2.8	8.096666667	-1.488	0.0037463	0.015701	DOWN	1.4005	0.0012341	0.0075452	UP	-0.098571	0.82436	0.91832	
transcript_HQ_CoA_transcript20427/f2p0/2174	27.01666667	67.53666667	52.57333333	1.4777	1.6242E-20	1.0567E-18	UP	-0.50594	0.00042342	0.0031833		0.96002	1.492E-07	6.168E-06	
transcript_HQ_CoA_transcript20434/f3p0/2199	9.97	27.52666667	7.1	1.6134	0.0019743	0.0091813	UP	-2.0944	8.6264E-05	0.00085237	DOWN	-0.49212	0.090967	0.24059	
transcript_HQ_CoA_transcript20438/f13p0/2155	101.7733333	35.38	55.96666667	-1.3894	1.9598E-05	0.00016858	DOWN	0.52039	0.019383	0.067082		-0.8783	0.0036671	0.024212	
transcript_HQ_CoA_transcript20449/f2p0/2194	0	269.85	29.52333333	15.062	8.4578E-33	1.333E-30	UP	-3.3358	0.011767	0.045638	DOWN	11.71	1.2878E-06	0.00003907	UP
transcript_HQ_CoA_transcript2045/f4p0/4871	4.296666667	12.91666667	6.88	1.7494	1.5615E-16	7.5127E-15	UP	-1.0629	6.7023E-06	9.6426E-05	DOWN	0.6748	0.005494	0.032677	
transcript_HQ_CoA_transcript20468/f2p0/2179	6.643333333	13.63333333	11.00666667	1.1767	0.0083492	0.030352	UP	-0.44786	0.21724	0.39428		0.71734	0.086499	0.23175	
transcript_HQ_CoA_transcript2047/f2p0/4943	4.076666667	1.806666667	3.43	-1.017	9.4062E-05	0.00066834	DOWN	0.76613	0.01522	0.055703		-0.26374	0.38016	0.59633	
transcript_HQ_CoA_transcript20472/f8p0/2109	15.13	147.4466667	38.36333333	3.4371	2.8879E-26	2.8083E-24	UP	-2.0817	9.3845E-14	8.6436E-12	DOWN	1.3432	5.4096E-05	0.00085672	UP
transcript_HQ_CoA_transcript2051/f2p0/4882	4.113333333	0.78	2.72	-2.1702	0.014054	0.046212	DOWN	1.6346	0.010769	0.04254	UP	-0.55425	0.45235	0.66234	
transcript_HQ_CoA_transcript20518/f2p0/2163	25.88	9.256666667	16.00666667	-1.3567	0.0068104	0.025603	DOWN	0.67802	0.29246	0.48214		-0.69087	0.24604	0.4553	
transcript_HQ_CoA_transcript20519/f2p0/2169	30.79	58.78333333	31.95	1.0913	0.00002344	0.0001973	UP	-1.034	1.286E-06	2.3525E-05	DOWN	0.046205	0.8291	0.92053	
transcript_HQ_CoA_transcript20542/f2p0/2208	0.746666667	8.14	2.99	3.6389	4.6364E-06	4.7064E-05	UP	-1.5591	0.079391	0.19322		2.0648	0.086868	0.23256	
transcript_HQ_CoA_transcript20544/f2p0/2161	0.05	88.57666667	9.946666667	10.938	4.0359E-26	3.8429E-24	UP	-3.2397	0.025803	0.083381		7.6811	1.002E-07	4.3175E-06	UP
transcript_HQ_CoA_transcript20546/f2p0/2163	3.243333333	66.66666667	6.003333333	4.5275	1.027E-23	8.1633E-22	UP	-3.6247	1.5934E-10	7.4124E-09	DOWN	0.88843	0.094191	0.24653	
transcript_HQ_CoA_transcript20558/f2p0/2148	2.116666667	8.643333333	4.29	2.201	7.9718E-05	0.00057834	UP	-1.1742	0.0067981	0.029715	DOWN	1.013	0.046335	0.15201	
transcript_HQ_CoA_transcript20585/f2p0/2161	0.246666667	8.056666667	0.353333333	5.1595	0.00011865	0.00081704	UP	-4.5821	2.2733E-05	0.00027269	DOWN	0.57858	0.74348	0.87015	
transcript_HQ_CoA_transcript20589/f2p0/2169	24.76	8.65	14.22666667	-1.3677	3.722E-08	5.7961E-07	DOWN	0.57639	0.034741	0.10386		-0.8032	0.00063964	0.0062885	
transcript_HQ_CoA_transcript20590/f2p0/2175	22.67	80.10333333	38.00333333	1.9827	1.7634E-26	1.724E-24	UP	-1.2268	8.3125E-14	7.7735E-12	DOWN	0.74388	4.2519E-05	0.00069868	
transcript_HQ_CoA_transcript20591/f2p0/2160	70.42	15.41	11.50333333	-2.0051	3.3763E-07	4.3316E-06	DOWN	-0.61751	0.28483	0.47374		-2.638	1.5391E-08	8.3579E-07	DOWN
transcript_HQ_CoA_transcript20599/f2p0/2163	6.706666667	15.88333333	5.713333333	1.4009	0.00052401	0.0029404	UP	-1.6312	3.2048E-06	5.1482E-05	DOWN	-0.24447	0.56711	0.74947	
transcript_HQ_CoA_transcript20607/f3p0/2156	2.313333333	9.77	4.873333333	2.2443	5.5453E-08	8.3577E-07	UP	-1.1499	4.1233E-08	1.1213E-06	DOWN	1.0841	0.01651	0.072048	
transcript_HQ_CoA_transcript20621/f2p0/2160	16.59	4.406666667	2.04	-1.7866	0.011068	0.038281	DOWN	-1.2334	0.11213	0.24781		-3.0278	3.6143E-09	2.2797E-07	DOWN
transcript_HQ_CoA_transcript20624/f3p0/2153	50.06666667	16.13666667	46.51666667	-1.4817	3.2598E-09	6.1694E-08	DOWN	1.3708	4.1747E-11	2.1738E-09	UP	-0.12146	0.61663	0.78414	
transcript_HQ_CoA_transcript20627/f4p0/2075	35.15333333	14.49666667	32.33666667	-1.1178	5.0851E-06	5.1137E-05	DOWN	1.0002	8.3663E-05	0.00083216	UP	-0.12868	0.5169	0.71342	
transcript_HQ_CoA_transcript20644/f2p0/2154	4.683333333	1.83	4.486666667	-1.1782	0.0084369	0.03061	DOWN	1.1355	0.0043972	0.021198	UP	-0.053854	0.88364	0.94794	
transcript_HQ_CoA_transcript20646/f2p0/2182	15.87	43.36333333	17.57666667	1.5992	5.0395E-06	5.0734E-05	UP	-1.4487	1.3588E-05	0.00017626	DOWN	0.13895	0.47092	0.67731	
transcript_HQ_CoA_transcript20648/f2p0/2154	2.6	0.246666667	1.176666667	-3.2668	3.6002E-05	0.00028919	DOWN	2.1254	0.0074876	0.032064	UP	-1.1525	0.018825	0.079617	
transcript_HQ_CoA_transcript20665/f2p0/2135	6.22	29.26	4.323333333	2.3545	0.0010948	0.0055518	UP	-2.8829	0.00008615	0.00085177	DOWN	-0.53719	0.093595	0.24545	
transcript_HQ_CoA_transcript20668/f3p0/2190	4.26	10.14666667	4.536666667	1.435	0.00032598	0.0019602	UP	-1.3201	6.9624E-05	0.00070897	DOWN	0.10108	0.7781	0.89134	
transcript_HQ_CoA_transcript20701/f2p0/2212	5.836666667	23.40333333	8.903333333	2.1613	3.7128E-14	1.4053E-12	UP	-1.5556	2.5367E-10	1.1396E-08	DOWN	0.59209	0.072892	0.20567	
transcript_HQ_CoA_transcript20712/f22p0/2083	11.59666667	32.33333333	9.49	1.6248	0.0073984	0.027397	UP	-1.9156	0.00021018	0.001791	DOWN	-0.30118	0.48837	0.69081	
transcript_HQ_CoA_transcript20725/f5p0/2117	14.82	31.08666667	24.95666667	1.227	5.8441E-07	7.1323E-06	UP	-0.46368	0.06487	0.1666		0.75034	0.01129	0.055121	
transcript_HQ_CoA_transcript20728/f2p0/2144	6.296666667	17.24	13.07333333	1.6296	3.8863E-05	0.00030878	UP	-0.56744	0.10499	0.23671		1.0493	2.8835E-05	0.00051286	UP
transcript_HQ_CoA_transcript20733/f2p0/2158	23.17333333	7.633333333	10.56666667	-1.4396	9.3151E-10	1.9471E-08	DOWN	0.30793	0.2147	0.39132		-1.1431	5.9245E-08	2.7461E-06	DOWN
transcript_HQ_CoA_transcript20735/f2p0/2166	12.95	46.52666667	17.16	2.0129	5.9521E-12	1.7272E-10	UP	-1.5857	4.1472E-05	0.00046154	DOWN	0.41268	0.24185	0.45073	
transcript_HQ_CoA_transcript20739/f2p0/2185	26.27	79.76	28.43666667	1.7705	1.7211E-07	2.3394E-06	UP	-1.642	4.9027E-06	7.3596E-05	DOWN	0.11453	0.62226	0.78824	
transcript_HQ_CoA_transcript20743/f16p0/1985	0.416666667	213.6	9.806666667	9.1759	1.6772E-85	1.9164E-82	UP	-4.5961	0.00020747	0.0017762	DOWN	4.5645	1.2031E-05	0.00024702	UP
transcript_HQ_CoA_transcript20746/f4p0/2148	37.39666667	11.51333333	24.7	-1.5577	1.2678E-10	3.0498E-09	DOWN	0.96416	1.6906E-05	0.00021122		-0.60409	0.0044076	0.027849	
transcript_HQ_CoA_transcript20748/f3p0/2159	10.57666667	4.656666667	10.22	-1.0269	0.004373	0.017746	DOWN	0.96992	0.0019317	0.010798		-0.067172	0.8178	0.9154	
transcript_HQ_CoA_transcript20761/f5p0/2153	46.85	107.5333333	73.62333333	1.3474	4.7005E-11	1.2053E-09	UP	-0.68887	0.0043267	0.020952		0.64568	0.0017985	0.014101	
transcript_HQ_CoA_transcript20771/f2p0/2159	7.553333333	20.31	17.09	1.6	1.2436E-05	0.00011329	UP	-0.40487	0.13443	0.28192		1.1805	0.0028025	0.019704	UP
transcript_HQ_CoA_transcript20772/f6p0/2116	5.853333333	43.58	9.193333333	3.0595	3.5465E-17	1.8162E-15	UP	-2.3966	1.9617E-10	8.9664E-09	DOWN	0.65007	0.047968	0.15569	
transcript_HQ_CoA_transcript20785/f4p0/2065	22.04666667	6.116666667	13.37	-1.6585	0.0011196	0.0056559	DOWN	0.93259	0.09735	0.22406		-0.73796	0.016818	0.072993	
transcript_HQ_CoA_transcript20793/f3p0/2129	10.45666667	81.99	15.64333333	3.147	8.5051E-14	3.0669E-12	UP	-2.5542	4.6191E-09	1.5621E-07	DOWN	0.57853	0.05362	0.16838	
transcript_HQ_CoA_transcript20801/f2p0/2116	4.006666667	0.573333333	4.346666667	-2.6974	0.00012879	0.00087728	DOWN	2.8084	4.8053E-06	0.00007261	UP	0.10194	0.87759	0.94519	
transcript_HQ_CoA_transcript20815/f5p0/2139	8.796666667	18.23333333	14.86	1.2051	0.0021051	0.0096819	UP	-0.42595	0.48411	0.66567		0.76733	0.22314	0.42976	
transcript_HQ_CoA_transcript20826/f2p0/2134	0.386666667	13.50666667	4.266666667	5.2778	2.3183E-23	1.8035E-21	UP	-1.7871	0.00042596	0.0031971	DOWN	3.4768	6.2693E-08	2.8913E-06	UP
transcript_HQ_CoA_transcript20836/f3p0/2100	21.56	52.01666667	39.79666667	1.4145	5.6312E-09	1.0153E-07	UP	-0.52766	0.0014869	0.0087741		0.87631	0.00034487	0.0038411	
transcript_HQ_CoA_transcript2084/f2p0/4899	11.86333333	3.383333333	6.39	-1.6629	1.942E-06	2.1479E-05	DOWN	0.77618	0.020412	0.069606		-0.89761	0.00050684	0.0052177	
transcript_HQ_CoA_transcript20853/f2p0/2212	12.79333333	4.21	13.57333333	-1.4527	0.00035205	0.0020924	DOWN	1.5318	1.6501E-09	6.1404E-08	UP	0.070297	0.83432	0.92318	
transcript_HQ_CoA_transcript20857/f2p0/2152	2.143333333	25.95333333	4.356666667	3.7786	1.3285E-10	3.1831E-09	UP	-2.7269	2.5147E-06	4.2132E-05	DOWN	1.0346	0.042512	0.1431	
transcript_HQ_CoA_transcript20859/f2p0/2145	8.076666667	3.546666667	7.433333333	-1.0294	0.0020812	0.0095842	DOWN	0.91413	0.00018835	0.0016383		-0.12722	0.6617	0.81508	
transcript_HQ_CoA_transcript2086/f2p0/4906	0.566666667	4.28	2.266666667	3.07	9.5265E-12	2.7086E-10	UP	-1.0534	0.0046485	0.022125	DOWN	2.0033	3.9292E-08	1.9181E-06	UP
transcript_HQ_CoA_transcript20871/f4p0/2119	114.6566667	42.38333333	72.74	-1.2834	9.6495E-06	9.0934E-05	DOWN	0.62601	0.035976	0.10639		-0.66982	0.040537	0.13858	
transcript_HQ_CoA_transcript2089/f3p0/4867	0.09	17.97666667	3.066666667	7.6986	5.0075E-05	0.00038452	UP	-2.6647	0.00021735	0.0018375	DOWN	5.0289	0.021038	0.08618	
transcript_HQ_CoA_transcript20894/f2p0/2136	3.5	11.21	1.343333333	1.8596	0.002362	0.010654	UP	-3.1986	3.7505E-06	5.8956E-05	DOWN	-1.3528	0.14351	0.32376	
transcript_HQ_CoA_transcript20895/f5p0/2148	15.90333333	5.98	14.08333333	-1.2527	3.5256E-05	0.00028395	DOWN	1.0854	5.6064E-05	0.00059467	UP	-0.17771	0.5262	0.7203	
transcript_HQ_CoA_transcript20903/f2p0/2189	7.906666667	26.49	10.31	1.9386	2.5294E-05	0.00021116	UP	-1.5291	0.0010069	0.0064239	DOWN	0.39412	0.22306	0.42975	
transcript_HQ_CoA_transcript20905/f6p0/2128	86.31666667	25.39333333	86.64666667	-1.5927	3.317E-09	6.2646E-08	DOWN	1.6121	7.6392E-09	2.4436E-07	UP	0.0047015	0.98509	0.9943	
transcript_HQ_CoA_transcript2091/f3p0/4857	0.22	3.07	1.09	4.0403	0.0070508	0.02635	UP	-1.6572	0.43157	0.6206		2.3622	0.016052	0.070604	
transcript_HQ_CoA_transcript20916/f3p0/2137	5	53.85333333	9.526666667	3.6094	1.1027E-16	5.3902E-15	UP	-2.6483	2.1361E-28	1.5059E-25	DOWN	0.94678	0.041357	0.14037	
transcript_HQ_CoA_transcript20919/f2p0/2146	0.726666667	61.64333333	5.88	6.5601	3.761E-43	8.5949E-41	UP	-3.5241	7.0948E-13	5.6787E-11	DOWN	3.0247	2.1219E-06	5.9828E-05	UP
transcript_HQ_CoA_transcript20920/f6p0/2159	34.08666667	9.966666667	23.18	-1.6142	2.6267E-08	4.2422E-07	DOWN	1.0687	0.00054644	0.0039136	UP	-0.55715	0.10913	0.27197	
transcript_HQ_CoA_transcript20937/f2p0/2136	0	11.51666667	0.376666667	10.441	3.7051E-15	1.5826E-13	UP	-5.0416	3.2004E-07	7.0504E-06	DOWN	5.399	0.064644	0.19058	
transcript_HQ_CoA_transcript20939/f12p0/2112	21.08	55.03666667	36.36	1.5453	3.4504E-07	4.4174E-06	UP	-0.75186	0.045014	0.12579		0.77735	0.05701	0.1751	
transcript_HQ_CoA_transcript20942/f2p0/2132	5.97	2.656666667	5.556666667	-1.0143	0.013946	0.045956	DOWN	0.93585	0.029921	0.09303		-0.090161	0.82714	0.91976	
transcript_HQ_CoA_transcript20943/f2p0/2163	176.7833333	43.87	46.28333333	-1.8705	1.4326E-05	0.00012794	DOWN	-0.066889	0.79501	0.88708		-1.9457	4.5967E-06	0.0001119	DOWN
transcript_HQ_CoA_transcript20947/f2p0/2120	13.10333333	5.49	9.536666667	-1.0984	4.4214E-05	0.0003454	DOWN	0.64074	0.0050228	0.023546		-0.47018	0.067916	0.19672	
transcript_HQ_CoA_transcript20948/f2p0/2138	11.13	3.136666667	4.63	-1.6786	2.9937E-08	4.7592E-07	DOWN	0.42771	0.241	0.42327		-1.2617	3.9854E-05	0.00066543	DOWN
transcript_HQ_CoA_transcript20956/f2p0/2133	67.80333333	6.28	3.36	-3.2801	3.0623E-59	1.1197E-56	DOWN	-1.0497	0.0018251	0.010331	DOWN	-4.3448	2.9934E-70	5.4944E-66	DOWN
transcript_HQ_CoA_transcript20974/f2p0/2131	11.97666667	4.97	28.81666667	-1.1375	0.013317	0.044267	DOWN	2.4188	3.6193E-08	9.9457E-07	UP	1.2697	0.00002224	0.00041026	UP
transcript_HQ_CoA_transcript20980/f2p0/2156	2.753333333	10.31666667	3.993333333	2.052	3.7239E-09	6.9733E-08	UP	-1.5026	0.00040748	0.0030812	DOWN	0.53644	0.22929	0.43668	
transcript_HQ_CoA_transcript20982/f2p0/2129	7.91	22.69666667	10.00333333	1.6853	9.8382E-12	2.7886E-10	UP	-1.3337	0.00003386	0.00038886	DOWN	0.33796	0.3135	0.5304	
transcript_HQ_CoA_transcript20985/f3p0/2147	3.73	22.72	5.183333333	2.7902	5.3808E-13	1.7789E-11	UP	-2.2939	0.0030449	0.015655	DOWN	0.47827	0.54872	0.73748	

transcript_HQ_CoA_transcript20986/f2p0/2136	24.13	46.81333333	22.85333333	1.1334	0.00066955	0.0036234	UP	-1.1918	0.0018071	0.010265	DOWN	-0.072333	0.8037	0.90691	
transcript_HQ_CoA_transcript20992/f3p0/2141	10.22666667	4.333333333	14.52333333	-1.107	0.0084203	0.030562	DOWN	1.6051	1.3664E-07	3.2825E-06	UP	0.48835	0.16379	0.35258	
transcript_HQ_CoA_transcript20996/f2p0/2139	25.99333333	7.226666667	13.77333333	-1.6977	1.0412E-09	2.1607E-08	DOWN	0.78487	0.0012381	0.0075595		-0.92468	1.0629E-05	0.00022171	
transcript_HQ_CoA_transcript2100/f3p0/4865	5.24	19.02	3.883333333	2.0227	3.5072E-11	9.1467E-10	UP	-2.453	8.0844E-18	1.4248E-15	DOWN	-0.44226	0.069172	0.19931	
transcript_HQ_CoA_transcript21021/f2p0/2108	4.333333333	1.663333333	1.52	-1.2316	0.00062766	0.0034264	DOWN	-0.28479	0.50609	0.68267		-1.5303	4.3627E-05	0.00071465	DOWN
transcript_HQ_CoA_transcript21031/f6p0/2050	7.006666667	15.38333333	9.65	1.2903	0.00011633	0.00080317	UP	-0.82129	0.0072866	0.031351		0.45845	0.22784	0.43508	
transcript_HQ_CoA_transcript21041/f3p0/2166	1.693333333	12.53	14.76	3.0251	0.0086796	0.031347	UP	0.070137	0.82701	0.90574		3.0848	0.054689	0.17047	
transcript_HQ_CoA_transcript21043/f6p0/2121	5.563333333	17.32	170.57	1.7757	0.00069062	0.003719	UP	3.1977	0.017013	0.060749		4.963	0.00047174	0.0049365	UP
transcript_HQ_CoA_transcript21050/f3p0/2129	21.34333333	8.363333333	13.53666667	-1.1922	0.00012366	0.00084612	DOWN	0.54234	0.023508	0.077718		-0.66362	0.037209	0.13004	
transcript_HQ_CoA_transcript21051/f2p0/2136	16.23666667	85.10333333	34.79	2.5558	8.0703E-13	2.616E-11	UP	-1.4439	0.00032722	0.0025555	DOWN	1.0981	0.00010913	0.0015296	UP
transcript_HQ_CoA_transcript21054/f6p0/2043	25.99333333	9.256666667	18.73666667	-1.3414	2.481E-12	7.5219E-11	DOWN	0.872	4.8314E-06	7.2938E-05		-0.48114	0.0085088	0.044866	
transcript_HQ_CoA_transcript21058/f6p0/2076	12.97666667	78.77666667	23.43	2.788	2.022E-13	7.0144E-12	UP	-1.9028	6.7097E-08	1.737E-06	DOWN	0.87003	0.017586	0.075579	
transcript_HQ_CoA_transcript21067/f2p0/2123	3.263333333	23.38	2.916666667	3.0154	1.7768E-15	7.7342E-14	UP	-3.1882	9.0406E-07	1.7554E-05	DOWN	-0.19023	0.79396	0.90113	
transcript_HQ_CoA_transcript2108/f2p0/4930	6.663333333	2.896666667	5.593333333	-1.0355	6.0683E-05	0.00045561	DOWN	0.79752	0.00011676	0.0010891		-0.25159	0.29103	0.50606	
transcript_HQ_CoA_transcript21085/f2p0/2136	1.016666667	14.65333333	2.176666667	4.0079	9.1833E-39	1.8053E-36	UP	-2.8796	1.6375E-08	4.8723E-07	DOWN	1.1178	0.106	0.2667	
transcript_HQ_CoA_transcript21102/f2p0/2125	6.303333333	14.02	13.06333333	1.3287	1.8775E-05	0.00016206	UP	-0.25896	0.48157	0.66386		1.0547	0.0025429	0.018275	UP
transcript_HQ_CoA_transcript21103/f3p0/2132	32.50333333	9.783333333	31.48333333	-1.5888	1.1094E-06	1.2927E-05	DOWN	1.5408	2.4261E-10	1.0953E-08	UP	-0.057427	0.81662	0.91475	
transcript_HQ_CoA_transcript21105/f3p0/2131	6.833333333	15.95	11.12	1.3792	8.153E-08	1.182E-06	UP	-0.66846	0.00012539	0.0011578		0.69821	0.010295	0.051421	
transcript_HQ_CoA_transcript21108/f3p0/2136	31.47666667	75.3	26.86	1.4236	2.1876E-05	0.00018602	UP	-1.6362	5.9339E-06	8.6802E-05	DOWN	-0.22643	0.3585	0.57561	
transcript_HQ_CoA_transcript2114/f3p0/4836	10.22666667	2.593333333	6.79	-1.8033	0.00018154	0.0011832	DOWN	1.2193	0.010863	0.042836	UP	-0.59581	0.061513	0.18432	
transcript_HQ_CoA_transcript21144/f2p0/2126	1.24	0.103333333	0.106666667	-3.3965	0.0006796	0.0036661	DOWN	-0.14584	0.9371	0.96685		-3.5608	0.00066534	0.0064821	DOWN
transcript_HQ_CoA_transcript21147/f2p0/2139	2.093333333	13.23333333	4.086666667	2.8264	2.0929E-08	3.4194E-07	UP	-1.8465	0.00025872	0.0021104	DOWN	0.96773	0.078911	0.21732	
transcript_HQ_CoA_transcript21160/f4p0/2142	3.94	33.48666667	6.673333333	3.2719	2.4388E-14	9.4661E-13	UP	-2.4967	7.186E-08	1.8268E-06	DOWN	0.7592	0.071091	0.20265	
transcript_HQ_CoA_transcript21161/f2p0/2144	1.016666667	6.233333333	1.416666667	2.7723	0.0002824	0.0017296	UP	-2.2804	0.0023448	0.012633	DOWN	0.4813	0.57659	0.75624	
transcript_HQ_CoA_transcript21164/f2p0/2119	18.55666667	3.923333333	15.13666667	-2.0957	7.5532E-10	1.5982E-08	DOWN	1.797	3.6455E-15	4.3108E-13	UP	-0.30892	0.31861	0.53571	
transcript_HQ_CoA_transcript2117/f2p0/4866	9.273333333	2.33	4.256666667	-1.8396	7.2904E-10	1.548E-08	DOWN	0.71765	0.011608	0.045154		-1.1333	7.4308E-07	2.4399E-05	DOWN
transcript_HQ_CoA_transcript21176/f5p0/2046	0	59.32	6.513333333	12.793	5.3784E-12	1.5657E-10	UP	-3.3602	0.08907	0.2099		9.4157	0.015965	0.070425	
transcript_HQ_CoA_transcript21185/f2p0/2127	11.01666667	33.53333333	12.98333333	1.7697	5.7319E-05	0.00043374	UP	-1.5213	0.00046754	0.0034591	DOWN	0.23446	0.29662	0.51217	
transcript_HQ_CoA_transcript21187/f3p0/2121	0.743333333	69.61	9.593333333	6.714	7.082E-27	7.1139E-25	UP	-3.0429	0.012756	0.048609	DOWN	3.6542	9.4362E-05	0.0013519	UP
transcript_HQ_CoA_transcript21198/f2p0/2139	3.736666667	11.03333333	10.55666667	1.7182	0.00063937	0.003484	UP	-0.19892	0.64754	0.79052		1.5071	0.0022616	0.016786	UP
transcript_HQ_CoA_transcript21205/f3p0/2124	7.56	13.48666667	7.323333333	1.0067	0.0093113	0.033222	UP	-1.0483	0.00066665	0.0046127	DOWN	-0.054927	0.87541	0.9443	
transcript_HQ_CoA_transcript21213/f5p0/2088	20.47333333	7.983333333	12.89666667	-1.1987	1.5413E-09	3.0796E-08	DOWN	0.54302	0.017558	0.062078		-0.66711	0.0032415	0.022021	
transcript_HQ_CoA_transcript21227/f3p0/2130	15.30666667	33.27333333	14	1.2899	0.0011005	0.0055764	UP	-1.396	0.00013498	0.0012323	DOWN	-0.12089	0.7168	0.85244	
transcript_HQ_CoA_transcript21239/f2p0/2121	34.02666667	9.75	25.4	-1.6645	7.4816E-08	1.096E-06	DOWN	1.2308	0.00090941	0.0059191	UP	-0.44397	0.23598	0.44474	
transcript_HQ_CoA_transcript21240/f2p0/2119	7.826666667	2.163333333	5.706666667	-1.6924	5.8138E-06	5.7985E-05	DOWN	1.2537	0.00058997	0.0041687	UP	-0.45141	0.2307	0.43854	
transcript_HQ_CoA_transcript21245/f9p0/2109	6.26	27.01666667	8.273333333	2.2856	1.0075E-09	2.0978E-08	UP	-1.8583	1.7688E-08	5.204E-07	DOWN	0.41314	0.3323	0.5494	
transcript_HQ_CoA_transcript21247/f2p0/2141	2.496666667	4.633333333	1.35	1.0353	0.014628	0.04761	UP	-1.9103	3.1096E-06	5.0306E-05	DOWN	-0.88765	0.064337	0.18986	
transcript_HQ_CoA_transcript21249/f2p0/2124	6.07	17.59333333	8.753333333	1.7186	1.5464E-05	0.00013668	UP	-1.164	4.2726E-05	0.00047205	DOWN	0.53919	0.20318	0.40534	
transcript_HQ_CoA_transcript2125/f2p0/4899	6.416666667	2	4.62	-1.5356	5.7182E-06	5.7063E-05	DOWN	1.0647	2.2948E-07	5.2446E-06	UP	-0.48204	0.1135	0.27901	
transcript_HQ_CoA_transcript21259/f4p0/2092	13.39333333	62.51666667	16.75666667	2.378	1.7955E-14	7.0896E-13	UP	-2.0487	1.5497E-16	2.1357E-14	DOWN	0.31761	0.34952	0.56744	
transcript_HQ_CoA_transcript21274/f2p0/2121	6.266666667	23.76	17.74666667	2.0859	7.5833E-10	1.6028E-08	UP	-0.54335	0.4317	0.62075		1.5291	0.021624	0.08789	
transcript_HQ_CoA_transcript21278/f3p0/2102	3.226666667	8.91	5.066666667	1.6314	0.00029256	0.0017829	UP	-0.96116	0.0082326	0.034494		0.65735	0.14919	0.33212	
transcript_HQ_CoA_transcript21295/f2p0/2125	1.026666667	8.1	1.346666667	3.136	1.7123E-06	1.9169E-05	UP	-2.7274	2.8556E-07	6.3674E-06	DOWN	0.39965	0.567	0.74941	
transcript_HQ_CoA_transcript21301/f2p0/1978	5.926666667	2.35	5.763333333	-1.176	0.00047031	0.0026786	DOWN	1.1443	0.00086525	0.0056802	UP	-0.042793	0.89711	0.95441	
transcript_HQ_CoA_transcript21319/f6p0/2090	4.59	45.12	7.06	3.4415	3.1158E-05	0.00025532	UP	-2.825	0.00073445	0.0049766	DOWN	0.60627	0.55137	0.73895	
transcript_HQ_CoA_transcript2132/f2p0/4847	2.863333333	7.833333333	4.336666667	1.6229	1.4652E-05	0.00013073	UP	-1.0074	0.012799	0.048732	DOWN	0.60237	0.10741	0.26908	
transcript_HQ_CoA_transcript21321/f7p0/2106	6.33	89.96666667	15.33666667	3.9962	2.4492E-14	9.4865E-13	UP	-2.689	5.6767E-05	0.00060039	DOWN	1.291	0.021762	0.088254	
transcript_HQ_CoA_transcript21326/f2p0/2117	3.643333333	9.94	6.023333333	1.6028	0.0021195	0.0097336	UP	-0.87932	0.032252	0.09836		0.71259	0.083658	0.22664	
transcript_HQ_CoA_transcript21335/f2p0/2111	46.5	99.12333333	72.49333333	1.2571	1.9646E-07	2.6488E-06	UP	-0.58363	0.10453	0.23592		0.65962	0.077992	0.21576	
transcript_HQ_CoA_transcript21344/f2p0/2109	12.89	5.576666667	7.553333333	-1.0556	0.0030061	0.013014	DOWN	0.28617	0.27671	0.46424		-0.77901	0.015682	0.069629	
transcript_HQ_CoA_transcript21346/f3p0/2113	1.453333333	4.45	27.44333333	1.754	0.0039083	0.016249	UP	2.5224	0.012082	0.04663	UP	4.2654	0.0014401	0.011907	UP
transcript_HQ_CoA_transcript21347/f2p0/2108	4.123333333	9.006666667	34.60666667	1.2742	0.012428	0.042002	UP	1.8085	0.029038	0.091151		3.0673	0.00010901	0.0015296	UP
transcript_HQ_CoA_transcript21352/f2p0/2112	0.77	4.063333333	5.873333333	2.5433	0.0017451	0.0082698	UP	0.40499	0.51951	0.69287		2.9379	8.9143E-08	3.9144E-06	UP
transcript_HQ_CoA_transcript21357/f2p0/2104	11.31666667	20.50666667	18.24	1.0261	0.014844	0.048142	UP	-0.3148	0.16599	0.32733		0.69481	0.08595	0.23092	
transcript_HQ_CoA_transcript21359/f2p0/2110	8.663333333	3.013333333	15.83666667	-1.3721	0.00011405	0.0007889	DOWN	2.2365	1.5704E-08	4.688E-07	UP	0.8528	0.012496	0.05904	
transcript_HQ_CoA_transcript21361/f2p0/2116	22.48333333	56.86666667	32.49	1.4992	0.0018716	0.0087758	UP	-0.95778	0.00005101	0.00054998		0.53034	0.25726	0.46766	
transcript_HQ_CoA_transcript21366/f2p0/2104	6.58	1.803333333	6.573333333	-1.7264	3.9568E-05	0.00031288	DOWN	1.7256	0.00034364	0.002661	UP	-0.012112	0.97485	0.98955	
transcript_HQ_CoA_transcript21374/f11p0/2088	19.00333333	38.23666667	33.67333333	1.1739	6.6024E-06	0.000065	UP	-0.3385	0.080958	0.19571		0.82251	0.00017598	0.0022463	
transcript_HQ_CoA_transcript21385/f2p0/1975	9.69	0	1.856666667	-9.9159	3.7737E-10	8.3929E-09	DOWN	7.5644	0.0018158	0.010298	UP	-2.3564	0.20085	0.40218	
transcript_HQ_CoA_transcript214/f4p0/7191	0.903333333	1.66	1.46	1.0265	0.00020826	0.0013331	UP	-0.33234	0.14402	0.29617		0.6823	0.018287	0.077932	
transcript_HQ_CoA_transcript2140/f2p0/4896	3.35	6.796666667	4.993333333	1.1724	0.0037182	0.015598	UP	-0.59219	0.033945	0.10218		0.56923	0.10954	0.27268	
transcript_HQ_CoA_transcript21407/f11p0/2086	37.34333333	6.14	31.72	-2.472	2.4207E-05	0.00020289	DOWN	2.2548	0.00061656	0.00431	UP	-0.22695	0.59141	0.7671	
transcript_HQ_CoA_transcript21410/f2p0/2112	8.07	2.346666667	3.7	-1.6119	0.00028374	0.0017363	DOWN	0.5044	0.18054	0.34724		-1.1221	0.0060029	0.035068	DOWN
transcript_HQ_CoA_transcript21416/f5p0/2105	45.03	14.01333333	24.97	-1.5173	9.5593E-05	0.00067659	DOWN	0.66387	0.081726	0.19707		-0.86505	0.0005595	0.0056406	
transcript_HQ_CoA_transcript21429/f2p0/2108	0.016666667	36.10333333	7.056666667	11.121	2.5306E-20	1.6291E-18	UP	-2.4806	0.17236	0.33586		8.6247	0.0053048	0.031893	UP
transcript_HQ_CoA_transcript21446/f4p0/2099	3.563333333	16.02666667	6.306666667	2.3285	5.8092E-09	1.0453E-07	UP	-1.4934	2.0221E-10	9.1838E-09	DOWN	0.82557	0.062495	0.186	
transcript_HQ_CoA_transcript21450/f6p0/2083	51.54	9.683333333	22	-2.2541	1.0828E-05	0.00010034	DOWN	1.0232	0.05033	0.13713		-1.2441	0.025764	0.099788	
transcript_HQ_CoA_transcript21471/f4p0/2099	17.49333333	42.30333333	17.64333333	1.4285	2.315E-09	4.4929E-08	UP	-1.3988	1.2611E-05	0.0001651	DOWN	0.01843	0.95847	0.98223	
transcript_HQ_CoA_transcript21476/f2p0/2095	21.55	8.923333333	17.1	-1.1117	0.0000925	0.00065852	DOWN	0.77538	0.075673	0.1866		-0.34823	0.36895	0.586	
transcript_HQ_CoA_transcript21486/f2p0/2106	7.21	19.63333333	16.74333333	1.6066	4.7071E-06	4.7755E-05	UP	-0.38619	0.035093	0.10459		1.2075	0.00030399	0.0034765	UP
transcript_HQ_CoA_transcript21492/f2p0/2133	28.12	11.26666667	11.41666667	-1.1703	4.8262E-07	5.9859E-06	DOWN	-0.13849	0.65129	0.79325		-1.3197	1.5866E-05	0.00031081	DOWN
transcript_HQ_CoA_transcript21496/f3p0/2113	19.52	36.05333333	36.02333333	1.0523	0.0018558	0.0087172	UP	-0.1634	0.6523	0.79396		0.87363	0.0053336	0.032014	
transcript_HQ_CoA_transcript21497/f2p0/2124	22.50666667	49.81	42.10666667	1.3083	4.8381E-09	8.8449E-08	UP	-0.39055	0.24157	0.42383		0.90436	0.0049013	0.030068	
transcript_HQ_CoA_transcript215/f7p0/7159	2.196666667	12.41666667	3.14	2.6368	3.2335E-05	0.00026397	UP	-2.0985	0.0024871	0.013256	DOWN	0.52371	0.2422	0.45097	
transcript_HQ_CoA_transcript21509/f4p0/2050	9.923333333	2.12	7.976666667	-2.0783	0.00029431	0.0017911	DOWN	1.7631	0.00085992	0.0056493	UP	-0.32901	0.56084	0.74543	
transcript_HQ_CoA_transcript21517/f2p0/2101	6.886666667	21.28	9.923333333	1.7644	0.0016521	0.0079013	UP	-1.2321	0.071979	0.17985		0.52184	0.5148	0.71222	
transcript_HQ_CoA_transcript21519/f3p0/2098	8.19	18.29	16.25666667	1.3189	0.00004725	0.00036587	UP	-0.32079	0.26864	0.45522		0.98586	0.00028297	0.0032957	
transcript_HQ_CoA_transcript21542/f2p0/2091	4.433333333	24.55	25.87666667	2.6363	2.5609E-12	7.7513E-11	UP	-0.04411	0.9582	0.97828		2.5787	0.0014895	0.012172	UP
transcript_HQ_CoA_transcript21546/f3p0/2102	6.943333333	23.81666667	11.32333333	1.9357	3.8902E-07	4.9218E-06	UP	-1.222	0.0011747	0.0072644	DOWN	0.70126	0.019334	0.081227	

transcript_HQ_CoA_transcript21549/f2p0/2106	2.93	24.83	3.276666667	3.2432	2.4107E-09	4.6589E-08	UP	-3.0821	1.4188E-08	4.2841E-07	DOWN	0.14861	0.77669	0.89071	
transcript_HQ_CoA_transcript21551/f2p0/2097	0.71	42.52	5.86	6.0984	1.292E-16	6.2652E-15	UP	-2.9991	0.00052204	0.0037761	DOWN	3.0792	0.0014708	0.012085	UP
transcript_HQ_CoA_transcript21558/f2p0/2108	1.25	13.92666667	2.676666667	3.6308	1.1124E-33	1.8158E-31	UP	-2.523	1.6349E-12	1.2083E-10	DOWN	1.0971	0.039359	0.13577	
transcript_HQ_CoA_transcript21564/f6p0/2053	19.31666667	5.803333333	16.48666667	-1.5604	0.001873	0.0087791	DOWN	1.3499	0.00034447	0.0026641	UP	-0.22315	0.58271	0.76099	
transcript_HQ_CoA_transcript21568/f3p0/2096	68.13333333	27.45333333	49.07	-1.154	6.1471E-15	2.5658E-13	DOWN	0.69028	4.1593E-09	1.4197E-07		-0.47629	0.00064817	0.0063554	
transcript_HQ_CoA_transcript21574/f2p0/2107	32.21666667	0	16.77	-11.763	1.2902E-16	6.2652E-15	DOWN	10.799	1.0747E-17	1.8017E-15	UP	-0.96877	0.13623	0.31336	
transcript_HQ_CoA_transcript21583/f2p0/2098	19.20333333	68.72666667	23.05666667	2.0021	1.4783E-20	9.6523E-19	UP	-1.7224	3.0277E-06	4.9372E-05	DOWN	0.26627	0.45903	0.66773	
transcript_HQ_CoA_transcript21589/f2p0/2095	0.256666667	13.98666667	53.51333333	5.9369	6.5654E-21	4.3966E-19	UP	1.837	0.2256	0.4046		7.7621	8.8556E-05	0.0012829	UP
transcript_HQ_CoA_transcript21593/f2p0/2109	0	12.06	2.973333333	10.512	1.8415E-16	8.7672E-15	UP	-2.1656	0.073383	0.18203		8.3309	0.033033	0.11952	
transcript_HQ_CoA_transcript21601/f3p0/2087	1.113333333	16.54	2.543333333	4.0572	8.405E-09	1.4718E-07	UP	-2.8509	1.9453E-05	0.00023802	DOWN	1.1923	0.07961	0.21878	
transcript_HQ_CoA_transcript21610/f8p0/2060	0.773333333	180.0366667	7.103333333	8.0623	1.129E-48	3.1755E-46	UP	-4.8191	5.9259E-15	6.7885E-13	DOWN	3.2275	0.00016301	0.0021175	UP
transcript_HQ_CoA_transcript21618/f2p0/2097	1.903333333	12.97	5.18	2.939	1.2258E-10	2.9586E-09	UP	-1.4902	0.00056028	0.0039896	DOWN	1.4382	0.00032955	0.003711	UP
transcript_HQ_CoA_transcript21627/f3p0/2086	2.243333333	13.98	11.42	2.8014	1.1487E-06	1.3342E-05	UP	-0.44036	0.38141	0.57383		2.348	6.0824E-10	4.5383E-08	UP
transcript_HQ_CoA_transcript21637/f2p0/2085	10.56333333	19.35666667	17.4	1.0403	0.00019324	0.0012479	UP	-0.32012	0.42688	0.61623		0.70844	0.064372	0.18993	
transcript_HQ_CoA_transcript21644/f4p0/2063	0.116666667	45.69	0.843333333	8.6679	7.7056E-24	6.2334E-22	UP	-5.8705	9.6146E-12	5.7031E-10	DOWN	2.7851	0.033498	0.12075	
transcript_HQ_CoA_transcript21672/f2p0/2087	0.21	36.24	1.256666667	7.624	3.9125E-27	4.0411E-25	UP	-4.9843	0.0015032	0.0088338	DOWN	2.6204	0.14296	0.32307	
transcript_HQ_CoA_transcript2170/f2p0/4840	0.096666667	8.323333333	1.136666667	6.6185	6.3085E-19	3.6498E-17	UP	-3.0121	0.00059586	0.0042028	DOWN	3.5957	0.0049414	0.030253	UP
transcript_HQ_CoA_transcript21703/f2p0/2091	34.02333333	76.00666667	34.00666667	1.31	3.6277E-09	6.8091E-08	UP	-1.2937	1.0625E-05	0.0001432	DOWN	0.005515	0.98463	0.99417	
transcript_HQ_CoA_transcript21706/f2p0/2095	5.043333333	13.92333333	7.866666667	1.624	0.00056141	0.003113	UP	-0.97216	0.0033223	0.016859		0.63941	0.12073	0.28998	
transcript_HQ_CoA_transcript21711/f2p0/2085	5.423333333	10.98666667	8.816666667	1.1856	8.2543E-05	0.00059575	UP	-0.47192	0.040155	0.11538		0.69829	0.034527	0.12324	
transcript_HQ_CoA_transcript21719/f2p0/2070	7.95	28.37	12.22333333	1.9693	5.5732E-07	6.8199E-06	UP	-1.343	0.00025863	0.0021104	DOWN	0.61431	0.012769	0.059959	
transcript_HQ_CoA_transcript21744/f2p0/2077	2.66	14.78333333	6.006666667	2.6422	1.552E-18	8.6767E-17	UP	-1.4548	3.301E-10	1.4579E-08	DOWN	1.1728	0.0012396	0.010603	UP
transcript_HQ_CoA_transcript2175/f2p0/4811	13.79333333	4.213333333	4.38	-1.5488	3.528E-06	3.6668E-05	DOWN	-0.082089	0.75483	0.86179		-1.6438	8.3466E-08	3.7366E-06	DOWN
transcript_HQ_CoA_transcript21750/f2p0/2084	1.16	48.75	9.223333333	5.5175	1.2694E-12	3.9807E-11	UP	-2.4924	0.10145	0.23107		3.0106	0.0036778	0.024274	UP
transcript_HQ_CoA_transcript21771/f6p0/2027	35.47333333	70.12	47.00333333	1.1476	0.00029932	0.001815	UP	-0.72934	0.018526	0.064691		0.40525	0.0080629	0.043097	
transcript_HQ_CoA_transcript2178/f2p0/4827	0	5.553333333	0.546666667	10.683	2.4622E-17	1.2752E-15	UP	-3.4702	0.0012546	0.0076446	DOWN	7.1983	0.0018667	0.014469	UP
transcript_HQ_CoA_transcript21784/f2p0/2096	7.906666667	2.96	11.33666667	-1.2692	1.7708E-05	0.00015394	DOWN	1.7745	6.1267E-05	0.00063696	UP	0.49453	0.24166	0.45059	
transcript_HQ_CoA_transcript21789/f2p0/2077	1.113333333	11.2	1.866666667	3.4641	0.00014877	0.00099381	UP	-2.6702	0.0095874	0.038715	DOWN	0.78006	0.34048	0.5584	
transcript_HQ_CoA_transcript21805/f2p0/2080	0.9	2.75	1.963333333	1.7925	0.0089932	0.032301	UP	-0.66539	0.26351	0.44938		1.1115	0.041484	0.14073	
transcript_HQ_CoA_transcript21806/f5p0/2078	60.18666667	142.5166667	56.43333333	1.4107	1.3643E-08	2.2861E-07	UP	-1.4901	1.2757E-08	3.9036E-07	DOWN	-0.092459	0.61159	0.78046	
transcript_HQ_CoA_transcript21836/f4p0/2062	6.453333333	54.36666667	10.28666667	3.2455	1.5869E-09	3.1604E-08	UP	-2.5564	9.8203E-06	0.00013393	DOWN	0.67393	0.11054	0.27455	
transcript_HQ_CoA_transcript21844/f2p0/2092	5.023333333	10.36666667	10.35666667	1.2254	0.012631	0.042511	UP	-0.17851	0.69298	0.82296		1.0342	7.1245E-05	0.0010781	UP
transcript_HQ_CoA_transcript21853/f2p0/2066	13.26333333	25.29666667	37.55666667	1.1108	0.0095827	0.033986	UP	0.39442	0.4475	0.63512		1.4896	0.00022395	0.0027295	UP
transcript_HQ_CoA_transcript21857/f3p0/2073	7.22	1.003333333	1.316666667	-2.6795	7.7502E-05	0.00056425	DOWN	0.22759	0.71938	0.83927		-2.464	3.9879E-05	0.00066543	DOWN
transcript_HQ_CoA_transcript21869/f2p0/2073	1.306666667	5.366666667	0.843333333	2.2112	0.0043124	0.017535	UP	-2.7839	9.4935E-05	0.00092311	DOWN	-0.59119	0.49144	0.69324	
transcript_HQ_CoA_transcript21871/f4p0/2076	19.91333333	44.52	30.05666667	1.3101	4.1439E-08	6.3985E-07	UP	-0.7118	0.0069935	0.030332		0.58629	0.054062	0.16942	
transcript_HQ_CoA_transcript21878/f2p0/2071	0.683333333	3.7	0.733333333	2.5933	9.8347E-05	0.00069313	UP	-2.4663	5.7847E-05	0.000609	DOWN	0.11532	0.86946	0.94182	
transcript_HQ_CoA_transcript2189/f4p0/4771	2.86	5.316666667	5.283333333	1.057	0.0008894	0.004647	UP	-0.15732	0.69444	0.8238		0.88347	0.046219	0.15179	
transcript_HQ_CoA_transcript21914/f2p0/2084	1.086666667	5.483333333	2.543333333	2.5274	0.00015126	0.0010089	UP	-1.2879	0.073572	0.18243		1.2253	0.15214	0.33593	
transcript_HQ_CoA_transcript21915/f2p0/2071	0.033333333	2.513333333	6.226666667	6.3586	4.4182E-05	0.00034534	UP	1.1799	0.38739	0.57912		7.5292	0.0014223	0.011824	UP
transcript_HQ_CoA_transcript21934/f2p0/2066	8.693333333	2.873333333	10.47333333	-1.429	0.0028898	0.012612	DOWN	1.6956	0.00026781	0.0021729	UP	0.25746	0.5428	0.73299	
transcript_HQ_CoA_transcript21936/f21p0/2018	29.77666667	77.75	38.78	1.5265	1.6986E-05	0.00014844	UP	-1.134	0.00097332	0.0062465	DOWN	0.37915	0.069035	0.19902	
transcript_HQ_CoA_transcript21937/f3p0/2063	25.31	6.44	10.13	-1.8536	0.00014811	0.00099039	DOWN	0.51884	0.25145	0.43529		-1.3422	0.0022062	0.016485	DOWN
transcript_HQ_CoA_transcript21940/f5p0/2033	18.43666667	34.30666667	17.94666667	1.0551	1.0233E-05	9.5648E-05	UP	-1.0885	1.1321E-05	0.0001507	DOWN	-0.046249	0.86156	0.93775	
transcript_HQ_CoA_transcript21944/f2p0/2066	7.85	28.38666667	8.53	2.011	7.7003E-06	7.4604E-05	UP	-1.8814	1.9301E-05	0.00023632	DOWN	0.11716	0.70033	0.84213	
transcript_HQ_CoA_transcript21945/f4p0/2023	107.5	14.77333333	49.62666667	-2.7314	9.7714E-15	4.0054E-13	DOWN	1.6047	4.1443E-14	4.0839E-12	UP	-1.1366	0.00029195	0.0033766	DOWN
transcript_HQ_CoA_transcript21953/f3p0/2094	9.45	18.9	12.55333333	1.1569	1.346E-10	3.2209E-09	UP	-0.73087	0.014772	0.054414		0.41326	0.19549	0.39475	
transcript_HQ_CoA_transcript21959/f2p0/2080	64.81666667	22.45	58.47333333	-1.3813	1.9967E-13	6.9403E-12	DOWN	1.225	0.00048897	0.0035907	UP	-0.16816	0.60378	0.7758	
transcript_HQ_CoA_transcript21962/f15p0/2057	13.34333333	48.55333333	28.22666667	2.0486	7.1479E-06	6.9806E-05	UP	-0.95348	0.045472	0.12681		1.0819	0.029527	0.11047	
transcript_HQ_CoA_transcript21964/f2p0/2094	38.07666667	9.693333333	11.87666667	-1.8313	3.7595E-08	5.8444E-07	DOWN	0.16788	0.82849	0.90643		-1.6774	0.013946	0.063754	
transcript_HQ_CoA_transcript21980/f3p0/2098	53.45333333	311.7566667	80.19666667	2.7087	7.2546E-58	2.5506E-55	UP	-2.1111	1.4613E-14	1.5755E-12	DOWN	0.58298	0.030964	0.11426	
transcript_HQ_CoA_transcript21986/f2p0/2064	32.36666667	84.56	15.97	1.5322	0.0019936	0.0092506	UP	-2.5375	9.143E-20	2.1213E-17	DOWN	-1.0165	0.036229	0.12771	
transcript_HQ_CoA_transcript21993/f2p0/2062	0.723333333	2.666666667	0.95	2.0522	0.00081812	0.0043191	UP	-1.6425	0.013723	0.051508		0.39922	0.60641	0.77761	
transcript_HQ_CoA_transcript22007/f2p0/2017	1.68	93.87	6.603333333	5.9691	3.957E-113	7.234E-110	UP	-3.9772	5.2606E-17	7.9034E-15	DOWN	1.9772	0.00064077	0.0062928	UP
transcript_HQ_CoA_transcript22013/f3p0/2072	4.423333333	9.603333333	4.553333333	1.2855	0.0002095	0.0013406	UP	-1.2341	0.00010171	0.00098114	DOWN	0.038106	0.91	0.96019	
transcript_HQ_CoA_transcript22018/f2p0/2074	0.223333333	30.13666667	1.996666667	7.3481	1.0931E-23	8.6136E-22	UP	-4.0567	1.2191E-09	4.6845E-08	DOWN	3.2755	0.0013086	0.011094	UP
transcript_HQ_CoA_transcript22023/f5p0/2049	8.683333333	3.436666667	8.573333333	-1.1925	0.00071829	0.0038487	DOWN	1.1651	0.00017835	0.0015656	UP	-0.037819	0.9129	0.96151	
transcript_HQ_CoA_transcript22033/f4p0/2015	45.63666667	7.4	27.05	-2.4718	8.4838E-25	7.3508E-23	DOWN	1.7159	1.5593E-06	0.00002783	UP	-0.76756	0.024083	0.094739	
transcript_HQ_CoA_transcript22049/f2p0/2089	14.55	30.82333333	12.88333333	1.2371	1.3494E-07	1.8774E-06	UP	-1.4111	1.195E-06	2.2057E-05	DOWN	-0.18614	0.50462	0.7047	
transcript_HQ_CoA_transcript22056/f4p0/2041	6.783333333	2.563333333	3.45	-1.2735	0.014279	0.046784	DOWN	0.2815	0.40253	0.59318		-1.0026	0.04022	0.13776	
transcript_HQ_CoA_transcript22060/f4p0/2012	117.72	46.63333333	56.44	-1.1809	8.0966E-09	1.4233E-07	DOWN	0.12133	0.49439	0.67373		-1.0709	1.6229E-08	8.7103E-07	DOWN
transcript_HQ_CoA_transcript22063/f4p0/2016	9.803333333	18.01	12.70666667	1.0265	0.012208	0.041395	UP	-0.65056	0.00082052	0.005447		0.36313	0.36266	0.57974	
transcript_HQ_CoA_transcript22069/f2p0/2067	19.28	6.896666667	6.62	-1.3194	4.5215E-07	5.6425E-06	DOWN	-0.22474	0.46812	0.65204		-1.5556	1.2648E-09	8.6626E-08	DOWN
transcript_HQ_CoA_transcript22076/f2p0/2040	1.036666667	2.89	4.25	1.6461	0.00027213	0.0016745	UP	0.40139	0.23814	0.41974		2.0346	4.611E-06	0.00011195	UP
transcript_HQ_CoA_transcript22081/f9p0/1996	608.1766667	234.87	388.23	-1.213	1.9585E-09	3.8417E-08	DOWN	0.57117	0.0032497	0.01655		-0.65528	0.00094348	0.0085308	
transcript_HQ_CoA_transcript22086/f2p0/2052	4.733333333	28.79333333	8.543333333	2.7511	3.9168E-11	1.0114E-09	UP	-1.8711	0.00056491	0.0040148	DOWN	0.86441	0.12482	0.29647	
transcript_HQ_CoA_transcript22087/f2p0/2077	16.11666667	4.166666667	6.966666667	-1.813	2.3826E-07	3.1588E-06	DOWN	0.61175	0.098285	0.22575		-1.2126	6.7986E-09	4.0125E-07	DOWN
transcript_HQ_CoA_transcript22089/f2p0/2063	8.186666667	14.82666667	10.24333333	1.0262	0.0048117	0.019224	UP	-0.70037	0.033873	0.10203		0.31346	0.181	0.37638	
transcript_HQ_CoA_transcript2209/f4p0/4798	0.113333333	15.36	1.516666667	7.212	1.2597E-43	2.9526E-41	UP	-3.4597	0.0002315	0.0019244	DOWN	3.7401	0.0010715	0.0094778	UP
transcript_HQ_CoA_transcript22091/f5p0/2019	57.9	13.67	56.9	-1.9368	2.6662E-08	4.2983E-07	DOWN	1.9373	3.9729E-06	6.1868E-05	UP	-0.010884	0.98053	0.99239	
transcript_HQ_CoA_transcript22098/f3p0/2051	4.573333333	1.18	4.03	-1.7861	0.00083928	0.0044175	DOWN	1.5976	0.00045279	0.0033627	UP	-0.19903	0.5977	0.77089	
transcript_HQ_CoA_transcript22100/f3p0/2158	43.01333333	6.736666667	10.21333333	-2.5365	8.3358E-05	0.00060069	DOWN	0.4313	0.58304	0.7453		-2.1138	0.00023618	0.0028427	DOWN
transcript_HQ_CoA_transcript22101/f2p0/2043	2.05	19.08333333	9.97	3.37	7.5074E-14	2.7286E-12	UP	-1.0425	0.14328	0.29494		2.3143	0.0030741	0.021197	UP
transcript_HQ_CoA_transcript22113/f2p0/2054	19.25333333	8.26	16.78333333	-1.0435	0.00047523	0.0027032	DOWN	0.84717	0.017133	0.061072		-0.20962	0.49034	0.69235	
transcript_HQ_CoA_transcript22114/f3p0/2071	14.74333333	27.19	32.76333333	1.0279	0.0098649	0.03485	UP	0.12256	0.59842	0.75649		1.1379	0.0025748	0.018447	UP
transcript_HQ_CoA_transcript22118/f60p0/2000	84.20666667	334.73	149.6266667	2.1481	1.2211E-15	5.3741E-14	UP	-1.3113	6.9883E-09	2.2511E-07	DOWN	0.82636	0.0030328	0.020951	
transcript_HQ_CoA_transcript2212/f2p0/4804	25.03666667	4.413333333	10.32666667	-2.3561	8.9263E-20	5.4579E-18	DOWN	1.0761	3.4838E-12	2.2724E-10	UP	-1.292	5.2623E-08	2.4703E-06	DOWN
transcript_HQ_CoA_transcript22122/f4p0/1967	8.056666667	40.88666667	12.18333333	2.5264	1.3156E-06	1.5127E-05	UP	-1.9047	7.3377E-05	0.00074277	DOWN	0.60871	0.185	0.38174	
transcript_HQ_CoA_transcript22123/f2p0/2047	23.13666667	92.31333333	44.33	2.1766	4.7781E-09	8.7441E-08	UP	-1.2182	0.0005468	0.0039136	DOWN	0.94405	3.7078E-05	0.00062726	

transcript_HQ_CoA_transcript22128/f3p0/2080	78.42333333	29.78333333	65.77333333	-1.2396	1.8014E-10	4.2169E-09	DOWN	0.99737	1.1723E-07	2.8498E-06		-0.25474	0.2239	0.43075	
transcript_HQ_CoA_transcript22134/f2p0/2067	13.91666667	4.96	9.126666667	-1.332	0.00095706	0.0049567	DOWN	0.73781	0.010796	0.042619		-0.60882	0.088618	0.23587	
transcript_HQ_CoA_transcript22139/f2p0/2032	10.99	21.12666667	10.54	1.1086	0.00083942	0.0044175	UP	-1.1544	0.0018432	0.010411	DOWN	-0.059361	0.84171	0.92725	
transcript_HQ_CoA_transcript22146/f5p0/2080	14.54666667	50.52	26.05	1.9389	1.798E-13	6.2851E-12	UP	-1.0935	8.6915E-07	0.00001696	DOWN	0.83445	0.004483	0.028277	
transcript_HQ_CoA_transcript22157/f5p0/2047	4.25	25.98333333	10.66333333	2.7663	3.3955E-13	1.1411E-11	UP	-1.4	0.088372	0.20868		1.3533	0.10806	0.27014	
transcript_HQ_CoA_transcript22165/f4p0/2020	5.33	26.22666667	11.95	2.4294	1.924E-10	4.4595E-09	UP	-1.2626	0.0051878	0.024128	DOWN	1.1542	0.0068996	0.038524	UP
transcript_HQ_CoA_transcript22171/f3p0/1992	63.43666667	127.68	59.87333333	1.178	0.00054753	0.0030463	UP	-1.2573	0.00057925	0.0041008	DOWN	-0.093264	0.66924	0.82031	
transcript_HQ_CoA_transcript22177/f4p0/2033	6.606666667	13.24333333	10.64666667	1.1379	0.011289	0.03884	UP	-0.42726	0.40107	0.59174		0.69993	0.096929	0.25169	
transcript_HQ_CoA_transcript22179/f3p0/2069	51.75	18.34333333	25.51	-1.335	1.6088E-13	5.6891E-12	DOWN	0.31542	0.20663	0.38105		-1.0321	1.7491E-05	0.00033826	DOWN
transcript_HQ_CoA_transcript22182/f2p0/2061	0.856666667	5.52	2.413333333	2.7918	0.0052151	0.020566	UP	-1.2696	0.19696	0.36868		1.5094	0.0096979	0.049204	UP
transcript_HQ_CoA_transcript22185/f2p0/2041	20.02333333	37.74333333	26.56666667	1.0774	0.00067099	0.003625	UP	-0.66588	0.012637	0.048234		0.39975	0.10354	0.26302	
transcript_HQ_CoA_transcript22208/f3p0/2047	0.47	5.17	5.286666667	3.6603	0.00010436	0.00073097	UP	-0.10092	0.77076	0.87091		3.5401	5.4911E-05	0.00086441	UP
transcript_HQ_CoA_transcript22211/f2p0/2059	0	4.763333333	35.21	9.1236	1.8129E-07	2.4551E-06	UP	2.7452	0.23131	0.41156		11.852	0.0024159	0.017603	UP
transcript_HQ_CoA_transcript22213/f3p0/2060	6.263333333	2.29	3.396666667	-1.3031	0.00031485	0.001901	DOWN	0.42828	0.24568	0.4285		-0.88808	0.002995	0.020721	
transcript_HQ_CoA_transcript22217/f2p0/2061	12.99	27.38	22.53666667	1.244	8.1487E-07	9.7305E-06	UP	-0.43328	0.0020183	0.011193		0.79683	0.00125	0.010677	
transcript_HQ_CoA_transcript22225/f3p0/2055	10.62666667	22.46333333	17.29666667	1.2531	1.5283E-05	0.0001353	UP	-0.53482	0.054172	0.14536		0.70375	0.0074516	0.04067	
transcript_HQ_CoA_transcript22229/f8p0/2036	354.7033333	120.68	153.07	-1.4014	1.0923E-06	1.2735E-05	DOWN	0.18306	0.42721	0.61662		-1.2288	1.0853E-06	3.3822E-05	DOWN
transcript_HQ_CoA_transcript22250/f9p0/2027	28.84333333	117.4333333	43.06666667	2.2006	1.3135E-09	2.6592E-08	UP	-1.6083	0.00000253	4.2348E-05	DOWN	0.57898	0.039688	0.13647	
transcript_HQ_CoA_transcript22251/f2p0/2037	24.41333333	45.10666667	24.75666667	1.0552	1.1985E-05	0.00010983	UP	-1.0245	3.8575E-06	6.0534E-05	DOWN	0.017386	0.91607	0.96294	
transcript_HQ_CoA_transcript22266/f4p0/2020	9.006666667	24.61333333	13.83333333	1.6103	5.8258E-05	0.00043975	UP	-0.97699	2.8945E-05	0.0003377		0.61936	0.12232	0.2925	
transcript_HQ_CoA_transcript22268/f2p0/2042	0.256666667	148.7366667	13.52	9.3334	2.4457E-39	4.968E-37	UP	-3.609	0.0023747	0.012768	DOWN	5.7115	1.2131E-07	5.1304E-06	UP
transcript_HQ_CoA_transcript22273/f2p0/2045	2.94	0.746666667	1.303333333	-1.8436	0.0032101	0.013744	DOWN	0.68941	0.39262	0.58299		-1.1643	0.049431	0.15911	
transcript_HQ_CoA_transcript22284/f4p0/1969	13.09333333	69.92	77.39333333	2.5826	3.0042E-10	6.7639E-09	UP	0.0039347	0.98881	0.99418		2.5725	5.2922E-14	1.0225E-11	UP
transcript_HQ_CoA_transcript22286/f3p0/2059	21.96666667	44.18333333	36.1	1.1707	3.102E-07	3.9965E-06	UP	-0.43814	0.023162	0.07677		0.7203	0.0025546	0.018345	
transcript_HQ_CoA_transcript22287/f2p0/2045	7.686666667	3.08	3.403333333	-1.1506	0.01367	0.045213	DOWN	-0.019567	0.96023	0.97902		-1.1864	0.015576	0.069309	
transcript_HQ_CoA_transcript22296/f2p0/2053	13.51666667	28.36666667	21.76	1.2247	4.8309E-07	5.9877E-06	UP	-0.53274	0.010655	0.042135		0.68082	0.0037112	0.02445	
transcript_HQ_CoA_transcript22297/f4p0/2020	13.55333333	6.143333333	12.99333333	-1.0045	0.0086439	0.031249	DOWN	0.95513	0.0088808	0.036571		-0.061254	0.80143	0.90586	
transcript_HQ_CoA_transcript22300/f3p0/2031	35.76666667	11.28333333	36.12333333	-1.5178	6.9822E-12	2.0166E-10	DOWN	1.5436	0.00019807	0.0017085	UP	0.012703	0.97361	0.98913	
transcript_HQ_CoA_transcript22304/f2p0/2050	25.38333333	9.806666667	15.34	-1.2229	3.6646E-07	4.6622E-06	DOWN	0.49553	0.0070249	0.030447		-0.73826	0.00062744	0.0061918	
transcript_HQ_CoA_transcript22306/f2p0/2047	0	17.47666667	1.053333333	10.996	3.3085E-17	1.699E-15	UP	-4.2211	4.2217E-05	0.00046727	DOWN	6.7587	0.0049208	0.030168	UP
transcript_HQ_CoA_transcript22310/f6p0/1960	22.10666667	40.45666667	53.18333333	1.0401	1.4829E-05	0.00013198	UP	0.23195	0.36435	0.55624		1.2585	0.00000148	4.3894E-05	UP
transcript_HQ_CoA_transcript22312/f2p0/2041	3.85	0.236666667	0.636666667	-3.8814	0.00079141	0.0041962	DOWN	1.3112	0.25442	0.43841		-2.585	0.0014527	0.011982	DOWN
transcript_HQ_CoA_transcript22322/f2p0/2024	3.5	1.116666667	1.713333333	-1.5228	0.01424	0.04669	DOWN	0.48411	0.41822	0.60779		-1.0475	0.063221	0.18738	
transcript_HQ_CoA_transcript22324/f3p0/2054	0.156666667	11.50333333	0.88	6.3675	2.6778E-24	2.2355E-22	UP	-3.8611	1.5049E-05	0.00019102	DOWN	2.4964	0.06415	0.1894	
transcript_HQ_CoA_transcript22329/f2p0/2042	3.01	1.246666667	1.386666667	-1.1169	0.013466	0.04468	DOWN	0.0041548	0.9932	0.99652		-1.1239	0.019281	0.081103	
transcript_HQ_CoA_transcript2233/f2p0/4792	7.413333333	14.25	11.66666667	1.116	0.011538	0.039589	UP	-0.438	0.0019946	0.011079		0.6618	0.11926	0.28738	
transcript_HQ_CoA_transcript22337/f2p0/2077	9.453333333	59.61333333	14.69333333	2.8146	5.1592E-14	1.921E-12	UP	-2.1555	1.1825E-05	0.00015683	DOWN	0.64568	0.15287	0.33687	
transcript_HQ_CoA_transcript22347/f2p0/2052	0.016666667	53.92333333	5.046666667	11.638	7.1845E-21	4.7937E-19	UP	-3.5241	0.047765	0.13183		8.0988	0.0074582	0.040682	UP
transcript_HQ_CoA_transcript22358/f20p0/1944	49.49666667	274.4266667	61.52333333	2.6333	4.5018E-61	1.7892E-58	UP	-2.3008	3.7392E-23	1.523E-20	DOWN	0.32071	0.16862	0.35927	
transcript_HQ_CoA_transcript22375/f2p0/2046	5.966666667	15.93333333	7.866666667	1.5741	1.4623E-05	0.00013053	UP	-1.1806	0.00032276	0.0025271	DOWN	0.38008	0.27681	0.49048	
transcript_HQ_CoA_transcript22380/f2p0/2045	0	107.7566667	7.456666667	13.585	3.8747E-28	4.5409E-26	UP	-3.9581	0.031049	0.095581		9.612	0.013894	0.06355	
transcript_HQ_CoA_transcript22382/f2p0/2058	93.54666667	37.19333333	43.53333333	-1.1945	0.00015763	0.0010438	DOWN	0.093075	0.79509	0.88708		-1.1121	1.0915E-06	3.3957E-05	DOWN
transcript_HQ_CoA_transcript22387/f2p0/2021	43.19	80.73333333	37.53666667	1.0541	0.00024552	0.0015324	UP	-1.2516	8.4468E-06	0.00011729	DOWN	-0.20927	0.40185	0.61713	
transcript_HQ_CoA_transcript22393/f2p0/2046	12.35333333	29.87	15.2	1.4391	8.7382E-06	8.3552E-05	UP	-1.1272	0.0003752	0.0028739	DOWN	0.2987	0.18958	0.3872	
transcript_HQ_CoA_transcript22394/f2p0/2026	2.49	0.503333333	1.333333333	-2.2117	0.014913	0.048307	DOWN	1.2795	0.17921	0.3454		-0.93869	0.059853	0.18103	
transcript_HQ_CoA_transcript22397/f3p0/2019	4.71	11.36333333	6.266666667	1.433	0.00030147	0.0018262	UP	-0.97602	0.043295	0.12222		0.44332	0.41682	0.62985	
transcript_HQ_CoA_transcript22400/f5p0/1940	5.216666667	15.14666667	5.61	1.6666	0.0040873	0.01683	UP	-1.5641	0.0084662	0.035244	DOWN	0.092614	0.785	0.89577	
transcript_HQ_CoA_transcript22408/f5p0/2014	7.14	34.93	51.20333333	2.4637	0.00007717	0.0005623	UP	0.40752	0.17808	0.34363		2.8565	1.4841E-06	4.3937E-05	UP
transcript_HQ_CoA_transcript22435/f2p0/2029	6.826666667	94.14666667	8.593333333	3.9484	9.9888E-16	4.4324E-14	UP	-3.6244	1.4323E-16	1.9889E-14	DOWN	0.31193	0.54539	0.73494	
transcript_HQ_CoA_transcript22443/f2p0/2050	6.63	2.373333333	7.93	-1.3144	0.00016621	0.0010935	DOWN	1.567	6.2197E-05	0.00064443	UP	0.23947	0.47014	0.67688	
transcript_HQ_CoA_transcript22445/f3p0/2018	8.463333333	21.86	12.15666667	1.5262	2.0387E-06	2.2467E-05	UP	-0.99718	2.1598E-05	0.00026112		0.51668	0.097448	0.25238	
transcript_HQ_CoA_transcript22447/f2p0/2035	4.443333333	8.47	5.423333333	1.0779	0.001454	0.0070717	UP	-0.78523	0.0057866	0.026325		0.28139	0.46567	0.67328	
transcript_HQ_CoA_transcript2245/f2p0/4785	0.666666667	3.21	2.78	2.4157	8.0813E-05	0.00058535	UP	-0.33111	0.51279	0.68746		2.0712	2.7543E-06	7.4457E-05	UP
transcript_HQ_CoA_transcript22454/f2p0/2034	7.316666667	1.25	1.82	-2.4288	0.0000646	0.00048146	DOWN	0.40975	0.44511	0.63312		-2.0261	0.00033587	0.0037576	DOWN
transcript_HQ_CoA_transcript22457/f3p0/2023	17.02666667	32.75	25.08	1.1038	4.9603E-11	1.2613E-09	UP	-0.53469	0.00080008	0.0053404		0.55707	0.0029412	0.020434	
transcript_HQ_CoA_transcript22459/f2p0/2036	158.0766667	61.84	161.6933333	-1.1858	5.1019E-07	6.3022E-06	DOWN	1.2287	1.4111E-08	4.2681E-07	UP	0.02995	0.83216	0.92228	
transcript_HQ_CoA_transcript2246/f2p0/4788	13.56	4.593333333	5.773333333	-1.4008	1.3128E-05	0.00011894	DOWN	0.17943	0.60387	0.76082		-1.2322	6.5798E-05	0.0010106	DOWN
transcript_HQ_CoA_transcript22462/f4p0/1990	18.60666667	5.973333333	20.70666667	-1.4894	1.3961E-07	1.9321E-06	DOWN	1.6519	1.5079E-08	4.5086E-07	UP	0.15106	0.58826	0.76497	
transcript_HQ_CoA_transcript22471/f2p0/2071	2.533333333	15.45	6.83	2.7532	7.9986E-13	2.5973E-11	UP	-1.3228	1.4005E-05	0.00018027	DOWN	1.4186	5.4658E-05	0.00086189	UP
transcript_HQ_CoA_transcript22472/f14p0/2000	60.29666667	109.6733333	78.19	1.0213	0.0047065	0.018865	UP	-0.64041	0.064399	0.16583		0.36978	0.29534	0.51098	
transcript_HQ_CoA_transcript22477/f6p0/2008	40.24333333	17.51	21.43	-1.0508	7.0299E-07	8.4888E-06	DOWN	0.14281	0.49574	0.67485		-0.91949	0.00009209	0.0013309	
transcript_HQ_CoA_transcript22480/f2p0/2036	20.73333333	8.29	22.05333333	-1.1764	1.3444E-07	1.872E-06	DOWN	1.2606	0.00043497	0.0032512	UP	0.073831	0.82711	0.91976	
transcript_HQ_CoA_transcript22482/f3p0/2030	6.343333333	0.09	6.403333333	-5.8888	4.194E-07	5.2734E-06	DOWN	5.8864	1.5216E-13	1.3539E-11	UP	-0.020932	0.97843	0.99138	
transcript_HQ_CoA_transcript22494/f5p0/2039	20.60333333	43.23	30.15333333	1.2457	0.00012654	0.0008635	UP	-0.67151	0.0014612	0.0086616		0.56011	0.065465	0.1922	
transcript_HQ_CoA_transcript22495/f2p0/2037	0.036666667	54	15.66	10.772	7.7726E-20	4.7684E-18	UP	-1.9364	0.25216	0.43594		8.8147	5.0145E-05	0.00080385	UP
transcript_HQ_CoA_transcript22497/f10p0/1976	23.42	72.67666667	40.67333333	1.7941	7.4853E-09	1.3235E-07	UP	-0.9912	0.0008615	0.0056577		0.79013	1.9422E-07	7.7329E-06	
transcript_HQ_CoA_transcript22499/f3p0/2013	10.88333333	4.193333333	7.88	-1.2225	0.010813	0.037534	DOWN	0.77139	0.027878	0.088512		-0.46424	0.25462	0.46517	
transcript_HQ_CoA_transcript2251/f2p0/4816	4.423333333	1.38	4.29	-1.4985	0.00070534	0.003786	DOWN	1.4612	0.0010785	0.0068004	UP	-0.05089	0.85495	0.93405	
transcript_HQ_CoA_transcript22524/f3p0/2039	5.983333333	17.73	3.783333333	1.7314	2.3791E-06	2.5767E-05	UP	-2.3926	1.0612E-11	6.1552E-10	DOWN	-0.67365	0.019868	0.082748	
transcript_HQ_CoA_transcript22543/f2p0/2021	7.59	59.34333333	13.71	3.132	3.1989E-13	1.083E-11	UP	-2.2573	5.1735E-05	0.00055454	DOWN	0.8607	0.066747	0.19465	
transcript_HQ_CoA_transcript22552/f3p0/2021	42.29	14.6	29.89666667	-1.4031	0.00053837	0.0030063	DOWN	0.89405	0.048083	0.13249		-0.51886	0.21501	0.42012	
transcript_HQ_CoA_transcript22556/f2p0/2029	7.27	2.743333333	4.01	-1.2507	4.1972E-05	0.00033032	DOWN	0.39287	0.24473	0.42749		-0.87059	0.0061422	0.035585	
transcript_HQ_CoA_transcript22563/f9p0/1992	58.62666667	24.24	28.95	-1.1225	1.5104E-09	3.0211E-08	DOWN	0.10807	0.65998	0.80011		-1.0266	6.7699E-06	0.00015379	DOWN
transcript_HQ_CoA_transcript22570/f3p0/2015	8.13	25.73	12.44	1.8256	9.2369E-10	1.9366E-08	UP	-1.2068	2.2555E-06	3.8279E-05	DOWN	0.60693	0.020116	0.083422	
transcript_HQ_CoA_transcript2258/f2p0/4841	6.82	2.836666667	6.106666667	-1.1082	0.0035877	0.015137	DOWN	0.9412	0.015354	0.056095		-0.17687	0.55986	0.74471	
transcript_HQ_CoA_transcript22582/f2p0/2012	0.016666667	96.96333333	7.37	12.475	2.0687E-22	1.4831E-20	UP	-3.8566	0.037923	0.11063		8.6019	0.0052072	0.031476	UP
transcript_HQ_CoA_transcript22584/f4p0/1964	36.93333333	14.88	23.79333333	-1.1591	8.0254E-09	1.4121E-07	DOWN	0.52927	0.0030879	0.015832		-0.64116	0.0022489	0.016732	
transcript_HQ_CoA_transcript22597/f2p0/2009	13.14666667	3.993333333	10.39333333	-1.5382	0.00035243	0.002094	DOWN	1.1975	0.0064252	0.028468	UP	-0.35206	0.16771	0.3581	
transcript_HQ_CoA_transcript22602/f7p0/2038	25.26	46.21333333	27.89	1.0253	0.0018594	0.0087273	UP	-0.88431	1.6873E-05	0.00021096		0.13078	0.65801	0.81283	

transcript_HQ_CoA_transcript22603/f2p0/2042	21.51	63.84	16.48	1.7319	5.8505E-06	5.8256E-05	UP	-2.105	1.6777E-06	2.9596E-05	DOWN	-0.3866	0.24567	0.45501	
transcript_HQ_CoA_transcript22619/f2p0/2017	2.276666667	5.08	4.026666667	1.3288	0.001653	0.0079026	UP	-0.50775	0.25096	0.43461		0.80646	0.062126	0.18558	
transcript_HQ_CoA_transcript22621/f2p0/2019	7.333333333	36.83	5.2	2.4796	1.3934E-08	2.3306E-07	UP	-2.9712	5.1198E-13	4.2081E-11	DOWN	-0.50298	0.094654	0.24731	
transcript_HQ_CoA_transcript2263/f2p0/4775	12.80333333	3.566666667	6.196666667	-1.6817	4.911E-10	1.0701E-08	DOWN	0.64456	0.018501	0.064616		-1.0498	4.53E-10	3.4936E-08	DOWN
transcript_HQ_CoA_transcript22650/f11p0/1936	26.07	6.776666667	22.44333333	-1.803	1.304E-10	3.1286E-09	DOWN	1.5831	2.5978E-05	0.00030581	UP	-0.23007	0.46341	0.67119	
transcript_HQ_CoA_transcript22654/f2p0/2035	1.696666667	7.416666667	4.62	2.3042	0.00010735	0.00074875	UP	-0.80661	0.12743	0.27158		1.4824	0.017234	0.074309	
transcript_HQ_CoA_transcript22658/f3p0/2019	0.833333333	4.856666667	6.283333333	2.6874	4.9472E-05	0.00038066	UP	0.26084	0.79551	0.88723		2.9354	0.007954	0.042639	UP
transcript_HQ_CoA_transcript2267/f3p0/4717	7.343333333	15.89333333	7.35	1.2529	9.3295E-05	0.00066392	UP	-1.2572	0.00028406	0.0022795	DOWN	-0.015784	0.94943	0.97794	
transcript_HQ_CoA_transcript22690/f3p0/2020	0.036666667	40.52666667	4.853333333	10.326	1.3824E-19	8.2054E-18	UP	-3.1918	0.032241	0.098343		7.1211	1.9827E-05	0.00037363	UP
transcript_HQ_CoA_transcript22691/f2p0/2011	10.17	21.89333333	7.43	1.2929	0.0011978	0.0059961	UP	-1.7255	2.1586E-06	3.6873E-05	DOWN	-0.44621	0.16092	0.34889	
transcript_HQ_CoA_transcript22692/f15p0/1994	37.04	15.29666667	32.16	-1.1188	8.6364E-09	1.5109E-07	DOWN	0.92487	3.7187E-06	5.8557E-05		-0.20588	0.34746	0.56534	
transcript_HQ_CoA_transcript22698/f6p0/1932	28.17666667	50.67666667	18.33666667	1.0133	1.2192E-06	1.4116E-05	UP	-1.6193	1.9011E-13	1.6593E-11	DOWN	-0.6196	0.0024934	0.018032	
transcript_HQ_CoA_transcript22699/f2p0/2017	1.24	2.94	3.153333333	1.3741	0.0080207	0.029356	UP	-0.020092	0.96841	0.98344		1.3419	0.010364	0.051659	
transcript_HQ_CoA_transcript22724/f2p0/2008	7.776666667	1.69	5.76	-2.0603	0.00028377	0.0017363	DOWN	1.618	8.2314E-05	0.00082041	UP	-0.44993	0.33657	0.55434	
transcript_HQ_CoA_transcript2273/f2p0/4780	10.11333333	2.736666667	4.68	-1.7358	1.6004E-12	4.959E-11	DOWN	0.61753	0.020052	0.068711		-1.1297	2.4398E-06	0.00006704	DOWN
transcript_HQ_CoA_transcript22731/f3p0/1961	9.146666667	25.13666667	13.80666667	1.617	5.1058E-10	1.1099E-08	UP	-1.005	0.00053994	0.0038795	DOWN	0.59847	0.049164	0.15861	
transcript_HQ_CoA_transcript22735/f20p0/1993	31.98333333	12.50666667	41.70333333	-1.1989	1.4806E-05	0.00013185	DOWN	1.5906	2.2005E-09	7.9238E-08	UP	0.37869	0.17225	0.36418	
transcript_HQ_CoA_transcript22739/f2p0/2026	16.65	6.353333333	13.9	-1.2487	0.0084482	0.030639	DOWN	0.98305	4.7245E-06	7.1684E-05		-0.27716	0.51952	0.71553	
transcript_HQ_CoA_transcript22757/f2p0/2001	10.15	20.83	10.54333333	1.2034	0.00028559	0.0017456	UP	-1.1347	0.00047845	0.0035254	DOWN	0.054564	0.82322	0.91783	
transcript_HQ_CoA_transcript22766/f4p0/1966	54.63	17.60333333	23.61333333	-1.4799	3.0555E-08	4.8533E-07	DOWN	0.26857	0.31595	0.50688		-1.222	2.4426E-08	1.2594E-06	DOWN
transcript_HQ_CoA_transcript2277/f2p0/4771	1.943333333	10.18	2.546666667	2.5543	9.9075E-18	5.2501E-16	UP	-2.1532	9.7624E-12	5.7536E-10	DOWN	0.38815	0.21348	0.41824	
transcript_HQ_CoA_transcript22772/f4p0/1965	0.66	9.156666667	0.776666667	3.9286	7.0029E-12	2.0194E-10	UP	-3.7118	1.3964E-11	7.9973E-10	DOWN	0.20765	0.78655	0.89644	
transcript_HQ_CoA_transcript22774/f2p0/2028	1.526666667	5.586666667	5.283333333	2.0118	0.00032801	0.0019687	UP	-0.20371	0.82173	0.90287		1.7944	0.056783	0.1747	
transcript_HQ_CoA_transcript22775/f7p0/1951	38.34333333	88.22	73.66333333	1.3655	1.1503E-12	3.6383E-11	UP	-0.40423	0.20106	0.3738		0.94752	0.0014083	0.011745	
transcript_HQ_CoA_transcript22787/f4p0/2018	10.43666667	36.62	14.65666667	1.9769	5.9187E-05	0.00044621	UP	-1.4715	0.0051108	0.023863	DOWN	0.49205	0.31656	0.53331	
transcript_HQ_CoA_transcript22807/f2p0/2008	5.383333333	10.12	5.533333333	1.0735	0.00019109	0.0012358	UP	-1.0235	0.00084929	0.0055932	DOWN	0.037205	0.90626	0.95837	
transcript_HQ_CoA_transcript22817/f3p0/2021	5.316666667	31.29666667	16.95	2.722	3.7121E-22	2.6407E-20	UP	-1.0149	0.027355	0.087213		1.6935	0.00023877	0.0028683	UP
transcript_HQ_CoA_transcript22823/f9p0/1990	2.763333333	25.06333333	31.01333333	3.3506	2.0067E-22	1.45E-20	UP	0.18409	0.83654	0.91068		3.5203	3.2971E-05	0.00057582	UP
transcript_HQ_CoA_transcript22828/f2p0/1999	39.49333333	17.08333333	21.34666667	-1.0617	0.0014229	0.0069497	DOWN	0.17668	0.48628	0.66762		-0.8955	0.0035126	0.02342	
transcript_HQ_CoA_transcript22835/f4p0/2006	43.69	10.14333333	17.31333333	-1.9422	1.7117E-13	6.0412E-12	DOWN	0.61748	0.12455	0.26739		-1.3342	0.0003576	0.0039612	DOWN
transcript_HQ_CoA_transcript22840/f5p0/1975	13.85333333	25.65	18.91333333	1.0459	3.3795E-05	0.00027362	UP	-0.59288	0.00062622	0.0043692		0.44031	0.068474	0.19798	
transcript_HQ_CoA_transcript22844/f3p0/1991	14.47	26.58333333	17.84	1.0464	0.00068051	0.0036699	UP	-0.73569	0.0045619	0.021816		0.29723	0.21956	0.42532	
transcript_HQ_CoA_transcript22866/f2p0/1997	50.54	22.13333333	21.95666667	-1.0434	7.6496E-05	0.00055806	DOWN	-0.1632	0.3205	0.51166		-1.2174	6.1254E-07	2.0668E-05	DOWN
transcript_HQ_CoA_transcript2287/f3p0/4851	21.46333333	7.986666667	9.98	-1.2935	0.00065848	0.0035743	DOWN	0.19019	0.61256	0.76674		-1.1146	2.4719E-10	1.9813E-08	DOWN
transcript_HQ_CoA_transcript22881/f2p0/2012	7.4	3.203333333	4.74	-1.068	0.0062529	0.023856	DOWN	0.43706	0.2548	0.43889		-0.64192	0.011567	0.055977	
transcript_HQ_CoA_transcript22891/f2p0/2009	3.67	16.27333333	3.736666667	2.3091	3.0667E-07	3.965E-06	UP	-2.289	1.0239E-10	4.9914E-09	DOWN	0.0083577	0.9846	0.99417	
transcript_HQ_CoA_transcript22899/f3p0/2008	600.1566667	200.1133333	309.5066667	-1.4164	0.0037912	0.015844	DOWN	0.46783	0.21071	0.38662		-0.95809	0.022756	0.090787	
transcript_HQ_CoA_transcript22913/f6p0/1937	44.54666667	11.82333333	33.27	-1.7223	0.00079478	0.0042116	DOWN	1.3088	0.011177	0.043792	UP	-0.42644	0.0067473	0.037931	
transcript_HQ_CoA_transcript22919/f15p0/1971	31.39666667	77.80333333	42.07333333	1.4642	8.3797E-08	1.212E-06	UP	-1.0274	0.00011886	0.0011059	DOWN	0.42377	0.14123	0.32061	
transcript_HQ_CoA_transcript2293/f2p0/4762	9.386666667	43.05	2.863333333	2.3337	5.4634E-12	1.5879E-10	UP	-4.0445	1.1477E-46	3.0052E-43	DOWN	-1.7194	3.4254E-06	8.7933E-05	DOWN
transcript_HQ_CoA_transcript22933/f2p0/2001	0.126666667	1.41	1.013333333	3.7281	0.013824	0.045635	UP	-0.69333	0.61597	0.76923		3.0143	0.010323	0.051531	
transcript_HQ_CoA_transcript2294/f2p0/4798	8.203333333	2.326666667	4.673333333	-1.6903	9.6353E-06	9.0857E-05	DOWN	0.86295	0.0087588	0.03619		-0.83701	0.014202	0.064603	
transcript_HQ_CoA_transcript22948/f2p0/1986	3.256666667	9.826666667	5.903333333	1.7666	1.5088E-05	0.00013395	UP	-0.8913	0.0015623	0.0091138		0.86015	0.036667	0.12878	
transcript_HQ_CoA_transcript22963/f4p0/1984	0.236666667	41.24666667	2.246666667	7.6089	1.439E-41	3.059E-39	UP	-4.3161	0.0013642	0.0081866	DOWN	3.2733	0.023321	0.092432	
transcript_HQ_CoA_transcript22966/f2p0/1985	29.27666667	12.89666667	15.50666667	-1.0309	0.0019819	0.0092123	DOWN	0.11941	0.59519	0.75394		-0.92302	0.0047353	0.029393	
transcript_HQ_CoA_transcript22973/f2p0/1990	3.533333333	27.14	9.163333333	3.1188	9.4935E-09	1.6498E-07	UP	-1.7167	1.5128E-05	0.00019162	DOWN	1.3871	0.0050114	0.030552	UP
transcript_HQ_CoA_transcript22988/f3p0/1992	46.64	10.74333333	23.85333333	-1.9668	2.1142E-14	8.2591E-13	DOWN	1.01	7.9296E-06	0.00011078	UP	-0.96775	0.00013751	0.0018451	
transcript_HQ_CoA_transcript22992/f2p0/1991	22.18	6.13	15.11333333	-1.6877	1.5951E-08	2.6498E-07	DOWN	1.1471	0.00010659	0.0010159	UP	-0.55252	0.0059075	0.034698	
transcript_HQ_CoA_transcript22993/f2p0/1985	29.63666667	127.8333333	42.91	2.2822	1.008E-19	6.0821E-18	UP	-1.7345	1.1494E-13	1.043E-11	DOWN	0.53352	0.0076551	0.041461	
transcript_HQ_CoA_transcript23012/f3p0/1998	33.21666667	109.5	50.96666667	1.892	3.1099E-11	8.1572E-10	UP	-1.2617	6.8598E-06	9.8153E-05	DOWN	0.61584	0.0025704	0.018422	
transcript_HQ_CoA_transcript23017/f2p0/1998	21.48666667	39.87666667	21.43333333	1.057	0.0031027	0.013375	UP	-1.0416	0.0053641	0.024772	DOWN	0.0019951	0.99328	0.99661	
transcript_HQ_CoA_transcript23022/f4p0/1978	11.28	2.296666667	2.106666667	-2.1758	0.00012079	0.00082956	DOWN	-0.23034	0.73045	0.8469		-2.4192	6.0473E-12	7.4999E-10	DOWN
transcript_HQ_CoA_transcript23026/f2p0/2002	49.25333333	15.49333333	33.7	-1.4923	5.7259E-05	0.00043346	DOWN	0.95287	0.015368	0.056134		-0.55378	0.018206	0.077624	
transcript_HQ_CoA_transcript23027/f2p0/1988	16.34666667	7.163333333	8.756666667	-1.0604	0.014083	0.046292	DOWN	0.15862	0.70969	0.8339		-0.91157	0.00029641	0.0034131	
transcript_HQ_CoA_transcript23028/f3p0/1985	3.833333333	70.91	19.36333333	4.388	1.5461E-23	1.2131E-21	UP	-2.0386	5.4427E-06	8.0451E-05	DOWN	2.3363	6.1274E-12	7.5483E-10	UP
transcript_HQ_CoA_transcript23031/f2p0/2002	0	96.39	6.456666667	13.411	5.2445E-29	6.5671E-27	UP	-4.0159	0.027861	0.088471		9.3802	0.016368	0.071601	
transcript_HQ_CoA_transcript23033/f3p0/1910	16.7	2.51	7.736666667	-2.5777	2.3216E-16	1.0939E-14	DOWN	1.4571	0.0090744	0.037188	UP	-1.1351	0.021913	0.088653	
transcript_HQ_CoA_transcript23046/f2p0/1989	7.66	17.51	14.10333333	1.3562	0.00022961	0.0014475	UP	-0.45956	0.27071	0.45749		0.88225	0.019554	0.081868	
transcript_HQ_CoA_transcript23054/f2p0/1985	36.96666667	136.3066667	38.31	2.0491	4.0134E-08	6.2181E-07	UP	-1.9917	4.642E-08	1.2494E-06	DOWN	0.044875	0.84082	0.92692	
transcript_HQ_CoA_transcript23059/f35p0/1900	81.69333333	165.7833333	212.98	1.1874	0.0015336	0.0074111	UP	0.20686	0.59574	0.75436		1.3764	0.0022468	0.016724	UP
transcript_HQ_CoA_transcript23096/f2p0/1995	3.106666667	76.64666667	32.03	4.7829	3.1907E-28	3.8126E-26	UP	-1.3567	0.3151	0.5063		3.4119	0.016383	0.071633	
transcript_HQ_CoA_transcript23113/f2p0/1977	0.213333333	21.26333333	1.176666667	6.7274	5.9736E-14	2.1974E-12	UP	-4.2946	0.0079563	0.033609	DOWN	2.4175	0.13586	0.3129	
transcript_HQ_CoA_transcript23120/f2p0/2024	5.643333333	49.33666667	15.10333333	3.2979	1.7417E-14	6.9072E-13	UP	-1.8599	5.0164E-05	0.00054341	DOWN	1.4236	6.6664E-05	0.0010197	UP
transcript_HQ_CoA_transcript23129/f2p0/1983	22.23666667	8.213333333	10.58666667	-1.3002	0.0018524	0.0087035	DOWN	0.23983	0.59346	0.75304		-1.07	0.001433	0.011864	DOWN
transcript_HQ_CoA_transcript23132/f3p0/1975	92.77	22.55333333	114.3666667	-1.9066	3.5903E-08	5.6197E-07	DOWN	2.2092	5.3254E-10	2.2084E-08	UP	0.29206	0.25558	0.46603	
transcript_HQ_CoA_transcript23151/f2p0/1981	41.54666667	8.923333333	24.77	-2.0529	2.1114E-10	4.8615E-09	DOWN	1.2998	0.00050607	0.0036883	UP	-0.76584	0.027605	0.10484	
transcript_HQ_CoA_transcript23153/f2p0/1966	10.46333333	2.263333333	24.18666667	-2.0885	0.002216	0.010098	DOWN	3.2917	5.0315E-19	1.0024E-16	UP	1.1956	0.041056	0.13976	
transcript_HQ_CoA_transcript23155/f2p0/1991	16.2	37.26	15.91333333	1.3711	0.0001013	0.00071065	UP	-1.3955	0.00010456	0.00099919	DOWN	-0.037806	0.87674	0.94492	
transcript_HQ_CoA_transcript23175/f4p0/1963	1850.87	618.88	1234.686667	-1.4326	1.3253E-14	5.3531E-13	DOWN	0.84838	0.00046431	0.0034372		-0.59587	0.0068517	0.038312	
transcript_HQ_CoA_transcript23183/f2p0/1944	1.186666667	5.17	1.816666667	2.3255	0.0037493	0.015707	UP	-1.6066	0.095577	0.22097		0.70136	0.5624	0.74593	
transcript_HQ_CoA_transcript23191/f2p0/1989	28.56666667	81.50333333	28.67666667	1.6651	8.6806E-08	1.2516E-06	UP	-1.641	5.8634E-05	0.00061412	DOWN	0.0085298	0.98203	0.99291	
transcript_HQ_CoA_transcript23197/f3p0/2002	130.8033333	44.42333333	56.96666667	-1.3827	6.9903E-05	0.0005151	DOWN	0.18882	0.59482	0.75376		-1.2058	9.6922E-15	2.1434E-12	DOWN
transcript_HQ_CoA_transcript23202/f21p0/1964	58.89	23.87333333	44.29333333	-1.1227	0.0046675	0.018754	DOWN	0.72055	0.082073	0.19758		-0.4142	0.064016	0.18912	
transcript_HQ_CoA_transcript23205/f4p0/1975	450.6	158.9866667	385.4833333	-1.3399	6.021E-14	2.2104E-12	DOWN	1.1139	0.00019721	0.0017051	UP	-0.23868	0.39385	0.60957	
transcript_HQ_CoA_transcript23219/f2p0/1981	0	13.13	1.046666667	10.518	1.0021E-14	4.0893E-13	UP	-3.779	8.1579E-06	0.00011354	DOWN	6.727	0.00035096	0.0038995	UP
transcript_HQ_CoA_transcript23239/f17p0/1929	57.80666667	133.41	87.30666667	1.362	5.2046E-07	6.4118E-06	UP	-0.76027	0.0012135	0.0074362		0.5893	0.0022088	0.016494	
transcript_HQ_CoA_transcript23240/f2p0/2001	7.09	1.986666667	6.31	-1.6799	2.7776E-06	2.9575E-05	DOWN	1.5311	7.2966E-08	1.8524E-06	UP	-0.16194	0.59496	0.76927	
transcript_HQ_CoA_transcript23255/f2p0/1969	0.603333333	5.173333333	1.513333333	3.2911	0.00038402	0.0022582	UP	-1.9256	0.0011166	0.0069848	DOWN	1.3499	0.1491	0.33209	
transcript_HQ_CoA_transcript23267/f2p0/1988	14.45333333	6.136666667	6.403333333	-1.0678	0.0041868	0.017151	DOWN	-0.083951	0.80097	0.89051		-1.1671	0.00054119	0.0055094	DOWN

transcript_HQ_CoA_transcript23283/f3p0/1913	38.77	9.073333333	16.58333333	-1.9873	0.008772	0.031613	DOWN	0.7645	0.32109	0.51212		-1.2303	0.0015183	0.012326	DOWN
transcript_HQ_CoA_transcript23285/f5p0/1929	12.02	27.64	13.57333333	1.3577	0.001033	0.0052928	UP	-1.1743	0.003918	0.019346	DOWN	0.17053	0.54336	0.73331	
transcript_HQ_CoA_transcript23301/f3p0/2044	11.86	3.103333333	7.933333333	-1.7844	0.00050234	0.0028345	DOWN	1.2005	0.0017093	0.0098078	UP	-0.59498	0.19908	0.3997	
transcript_HQ_CoA_transcript23311/f2p0/1970	9.32	20.17666667	9.623333333	1.2936	0.0055693	0.021659	UP	-1.2202	0.0095555	0.038637	DOWN	0.057011	0.90366	0.95713	
transcript_HQ_CoA_transcript23318/f2p0/1981	106.3166667	43.30333333	90.29666667	-1.1523	1.4237E-06	1.6258E-05	DOWN	0.91553	0.0021285	0.011656		-0.24845	0.29754	0.51318	
transcript_HQ_CoA_transcript23331/f2p0/1972	24.50666667	2.58	9.256666667	-3.1135	4.7941E-08	7.3283E-07	DOWN	1.7185	0.00080371	0.0053588	UP	-1.4064	0.00028167	0.0032892	DOWN
transcript_HQ_CoA_transcript23340/f2p0/1984	30.95	65.28333333	44.66	1.2352	6.7849E-05	0.0005024	UP	-0.6949	0.032753	0.099437		0.52731	0.021178	0.086632	
transcript_HQ_CoA_transcript23347/f2p0/1966	20.42333333	5.366666667	7.753333333	-1.7722	1.0817E-07	1.5271E-06	DOWN	0.37919	0.32454	0.51542		-1.4037	4.9001E-07	1.7197E-05	DOWN
transcript_HQ_CoA_transcript23358/f2p0/1972	0.51	91.74	6.84	7.6475	1.2967E-64	6.2385E-62	UP	-3.8802	1.9255E-07	4.4562E-06	DOWN	3.7514	2.6413E-05	0.0004745	UP
transcript_HQ_CoA_transcript23360/f2p0/1984	4.95	1.27	3.026666667	-1.8141	0.0066639	0.025176	DOWN	1.0859	0.028938	0.090947		-0.73799	0.18467	0.38136	
transcript_HQ_CoA_transcript23361/f2p0/1948	3.67	1.056666667	6.713333333	-1.6404	0.00065038	0.0035367	DOWN	2.5039	2.8798E-10	1.2874E-08	UP	0.85235	0.033945	0.12198	
transcript_HQ_CoA_transcript23379/f2p0/1978	7.923333333	1.163333333	0.986666667	-2.5867	1.6538E-08	2.7386E-07	DOWN	-0.43562	0.73009	0.84666		-3.0281	0.0010872	0.0095756	DOWN
transcript_HQ_CoA_transcript23382/f4p0/1951	76.25333333	30.81333333	29.57333333	-1.1598	8.5173E-10	1.7898E-08	DOWN	-0.2056	0.20355	0.37736		-1.3765	1.417E-13	2.4248E-11	DOWN
transcript_HQ_CoA_transcript23395/f2p0/1964	8.486666667	32.09333333	14.56666667	2.088	9.3514E-09	1.6267E-07	UP	-1.3074	5.4125E-05	0.00057544	DOWN	0.76871	0.0021443	0.016131	
transcript_HQ_CoA_transcript23400/f2p0/1964	19.59	7.52	11.82333333	-1.2184	3.9301E-05	0.00031158	DOWN	0.50156	0.030717	0.09472		-0.73181	0.013705	0.062966	
transcript_HQ_CoA_transcript23401/f9p0/1939	43.37666667	85.72333333	61.50333333	1.1474	4.4704E-08	6.8737E-07	UP	-0.6321	0.0027934	0.014537		0.50248	0.0070144	0.038944	
transcript_HQ_CoA_transcript23408/f2p0/1961	23.64	8.71	19.14666667	-1.2916	0.0035333	0.014942	DOWN	0.97717	8.6158E-05	0.00085177		-0.3233	0.40135	0.61687	
transcript_HQ_CoA_transcript23410/f2p0/1960	14.87	6.65	9.906666667	-1.0148	0.0055239	0.021532	DOWN	0.43589	0.23472	0.41559		-0.59081	0.0061091	0.035485	
transcript_HQ_CoA_transcript23418/f4p0/1933	17.62	4.626666667	9.943333333	-1.7755	1.6788E-06	1.8853E-05	DOWN	0.95674	0.00025472	0.0020849		-0.83189	0.018947	0.080021	
transcript_HQ_CoA_transcript23423/f4p0/1957	27.02666667	5.716666667	6.053333333	-2.0861	1.1288E-08	1.9322E-07	DOWN	-0.047245	0.91877	0.95693		-2.1454	8.152E-09	4.7501E-07	DOWN
transcript_HQ_CoA_transcript23425/f5p0/1960	2.3	9.58	6.426666667	2.2526	0.00016592	0.0010923	UP	-0.73984	0.37015	0.56214		1.4938	0.059224	0.17993	
transcript_HQ_CoA_transcript23452/f10p0/1934	3.126666667	79.37666667	4.62	4.8197	8.7616E-23	6.5114E-21	UP	-4.2436	1.2473E-09	4.763E-08	DOWN	0.56334	0.47739	0.68223	
transcript_HQ_CoA_transcript23470/f4p0/1954	17.40666667	7.29	11.22333333	-1.1039	1.0411E-05	9.6914E-05	DOWN	0.4692	0.075441	0.18615		-0.64584	0.022453	0.090024	
transcript_HQ_CoA_transcript23482/f2p0/1962	1.063333333	0.163333333	0.436666667	-2.5178	0.010175	0.035761	DOWN	1.2271	0.31528	0.50631		-1.3062	0.13606	0.31314	
transcript_HQ_CoA_transcript23484/f3p0/1962	16.07666667	2.18	4.48	-2.6719	4.9609E-05	0.0003814	DOWN	0.8462	0.18495	0.35287		-1.8392	0.00011903	0.0016414	DOWN
transcript_HQ_CoA_transcript23491/f3p0/1955	24.49666667	10.92666667	12.48666667	-1.0057	0.00034389	0.0020506	DOWN	0.038586	0.87933	0.93443		-0.97813	3.5573E-05	0.00060837	
transcript_HQ_CoA_transcript23500/f2p0/1956	17.40333333	5.856666667	9.903333333	-1.3995	0.0090211	0.032389	DOWN	0.61573	0.01031	0.041019		-0.79683	0.11816	0.28615	
transcript_HQ_CoA_transcript23501/f7p0/1929	67.97666667	29.42	71.98333333	-1.0514	8.7514E-09	1.528E-07	DOWN	1.1399	2.3632E-14	2.4334E-12	UP	0.07684	0.64678	0.80477	
transcript_HQ_CoA_transcript23525/f2p0/1955	5.3	20.87333333	8.213333333	2.1737	0.00023111	0.001456	UP	-1.5205	0.0077187	0.032833	DOWN	0.63548	0.3151	0.53192	
transcript_HQ_CoA_transcript23526/f2p0/1973	1.643333333	6.763333333	4.1	2.1966	2.8024E-09	5.348E-08	UP	-0.87173	0.00059223	0.004183		1.3138	0.0018681	0.014474	UP
transcript_HQ_CoA_transcript23532/f2p0/1967	7.683333333	13.85333333	7.256666667	1.0235	0.00023556	0.0014804	UP	-1.078	0.0024497	0.013083	DOWN	-0.068156	0.85216	0.93254	
transcript_HQ_CoA_transcript23538/f32p0/1888	62.92666667	122.53	83.78333333	1.1179	5.2297E-07	6.4383E-06	UP	-0.70013	1.2439E-05	0.0001639		0.40633	0.041981	0.14188	
transcript_HQ_CoA_transcript23544/f13p0/1902	35.35333333	87.34333333	53.46333333	1.4583	2.2092E-07	2.9438E-06	UP	-0.85352	0.00040144	0.0030418		0.59252	0.035772	0.12651	
transcript_HQ_CoA_transcript23545/f2p0/1953	5.22	33.32	8.766666667	2.838	3.6706E-08	5.7306E-07	UP	-2.0733	5.0696E-05	0.00054775	DOWN	0.75077	0.0094034	0.048091	
transcript_HQ_CoA_transcript23551/f2p0/1953	26.42333333	51.62666667	34.15	1.117	1.9257E-09	3.7816E-08	UP	-0.74301	5.3774E-07	1.1251E-05		0.36258	0.046144	0.15171	
transcript_HQ_CoA_transcript23559/f3p0/1892	10.89666667	24.80666667	9.32	1.3469	9.3844E-05	0.00066731	UP	-1.5647	3.1332E-06	5.0554E-05	DOWN	-0.22917	0.47482	0.68014	
transcript_HQ_CoA_transcript2356/f3p0/4729	4.33	8.516666667	6.513333333	1.1386	0.00033628	0.0020124	UP	-0.53709	0.0047703	0.022557		0.588	0.057389	0.1758	
transcript_HQ_CoA_transcript23592/f3p0/1914	2.82	14.52333333	4.616666667	2.5323	1.5225E-10	3.6008E-09	UP	-1.8112	1.5362E-09	5.7909E-08	DOWN	0.7068	0.14368	0.32394	
transcript_HQ_CoA_transcript23605/f3p0/1943	23.21666667	9.733333333	19.37	-1.0915	2.2339E-05	0.00018942	DOWN	0.83256	0.0010911	0.0068585		-0.27046	0.20249	0.4043	
transcript_HQ_CoA_transcript23610/f2p0/1956	26.08	3.326666667	5.103333333	-2.8101	6.2203E-26	5.7726E-24	DOWN	0.45469	0.13522	0.2832		-2.3674	1.6576E-23	1.217E-20	DOWN
transcript_HQ_CoA_transcript23618/f2p0/1959	17.02666667	5.046666667	13.95	-1.5873	9.5962E-06	9.0619E-05	DOWN	1.3111	1.7703E-05	0.0002194	UP	-0.28806	0.40564	0.62008	
transcript_HQ_CoA_transcript23621/f3p0/1945	3.6	56.51666667	5.773333333	4.1347	1.9293E-13	6.7313E-12	UP	-3.4371	2.2986E-12	1.6204E-10	DOWN	0.68422	0.21914	0.42511	
transcript_HQ_CoA_transcript23629/f3p0/1907	76.15	15.77333333	54.04333333	-2.1133	1.7412E-06	1.9469E-05	DOWN	1.6098	1.5065E-05	0.00019109	UP	-0.5128	0.13521	0.31173	
transcript_HQ_CoA_transcript2363/f2p0/4757	5.123333333	9.79	8.83	1.0683	0.0087686	0.031613	UP	-0.29112	0.26094	0.44606		0.76774	0.03717	0.12993	
transcript_HQ_CoA_transcript23641/f3p0/1943	8.653333333	17.27666667	8.413333333	1.1539	2.6616E-05	0.00022098	UP	-1.1942	7.2937E-07	1.4595E-05	DOWN	-0.053558	0.85863	0.93621	
transcript_HQ_CoA_transcript23652/f2p0/1964	17.58666667	6.34	13.48666667	-1.3144	5.3208E-09	9.6407E-08	DOWN	0.93564	2.4781E-05	0.00029399		-0.39032	0.047208	0.15405	
transcript_HQ_CoA_transcript23654/f3p0/1967	6.46	17.67666667	14.16333333	1.6132	4.5527E-05	0.00035464	UP	-0.48663	0.21137	0.38738		1.1136	0.00041942	0.0044811	UP
transcript_HQ_CoA_transcript2366/f2p0/4736	8.626666667	26.95	14.08333333	1.8159	0.001467	0.0071235	UP	-1.0714	0.00022409	0.0018798	DOWN	0.72831	0.20462	0.40708	
transcript_HQ_CoA_transcript23662/f3p0/1944	15.4	5.446666667	9.706666667	-1.3337	0.00023764	0.0014909	DOWN	0.67327	0.11035	0.24503		-0.67523	0.13337	0.30899	
transcript_HQ_CoA_transcript23663/f2p0/1966	27.11333333	11.02666667	24.80333333	-1.1464	1.4822E-09	2.9712E-08	DOWN	1.0253	2.3432E-09	8.4049E-08	UP	-0.13227	0.46787	0.67508	
transcript_HQ_CoA_transcript23698/f2p0/1948	6.646666667	0.863333333	1.683333333	-2.7722	1.1544E-05	0.00010616	DOWN	0.79803	0.22906	0.40873		-1.9865	0.0001085	0.0015249	DOWN
transcript_HQ_CoA_transcript23714/f2p0/1938	12.35666667	22.98	31.04	1.0579	1.8416E-05	0.00015964	UP	0.3047	0.381	0.57358		1.3499	0.00021493	0.0026389	UP
transcript_HQ_CoA_transcript23715/f2p0/1939	0.113333333	33.31333333	0.68	8.3714	2.7574E-27	2.9654E-25	UP	-5.7337	0.00004577	0.00050055	DOWN	2.6258	0.2657	0.47781	
transcript_HQ_CoA_transcript23716/f3p0/1959	6.85	48.14	17.07333333	2.9896	3.6078E-14	1.3713E-12	UP	-1.6444	0.0075324	0.032197	DOWN	1.3306	0.020158	0.083523	
transcript_HQ_CoA_transcript23717/f2p0/1939	18.10333333	6.223333333	10.77	-1.4166	0.0083507	0.030352	DOWN	0.65485	0.051893	0.14064		-0.77029	0.10794	0.2699	
transcript_HQ_CoA_transcript2373/f2p0/4701	7.41	3.21	4.346666667	-1.0749	0.0027061	0.011933	DOWN	0.30364	0.40662	0.59719		-0.78094	0.0021361	0.016076	
transcript_HQ_CoA_transcript23735/f3p0/1970	0.23	3.406666667	0	4.1718	0.007307	0.02713	UP	-8.7297	3.3393E-08	9.2877E-07	DOWN	-4.5805	0.24368	0.45285	
transcript_HQ_CoA_transcript23739/f2p0/1935	20.43333333	4.193333333	13.60666667	-2.1579	0.00011683	0.00080584	DOWN	1.5641	5.802E-07	1.1962E-05	UP	-0.60234	0.2062	0.40863	
transcript_HQ_CoA_transcript23771/f2p0/1945	9.62	21.25333333	25.38	1.3038	9.4973E-06	0.00008987	UP	0.14196	0.79989	0.88985		1.4315	0.011075	0.054309	
transcript_HQ_CoA_transcript23774/f4p0/1901	34.35	71.3	58.86	1.212	0.00008187	0.00059183	UP	-0.4283	0.13718	0.28625		0.77201	8.9321E-05	0.001293	
transcript_HQ_CoA_transcript23778/f2p0/1942	0.126666667	45.17	5.016666667	8.6095	1.5964E-29	2.0996E-27	UP	-3.3122	0.025281	0.082114		5.2773	0.00031609	0.0035903	UP
transcript_HQ_CoA_transcript23779/f8p0/1930	0	21.12333333	34.79333333	11.146	9.1557E-08	1.3149E-06	UP	0.61985	0.72709	0.84453		11.751	2.8964E-05	0.00051465	UP
transcript_HQ_CoA_transcript23793/f2p0/1928	32.21333333	13.76333333	23.00333333	-1.0687	0.00057984	0.0032036	DOWN	0.58409	0.040021	0.11505		-0.49584	0.016924	0.073332	
transcript_HQ_CoA_transcript23797/f3p0/1956	23.16	48.97666667	48.27333333	1.2186	0.00012373	0.00084627	UP	-0.13658	0.72537	0.8434		1.0709	0.0041357	0.026515	UP
transcript_HQ_CoA_transcript23799/f3p0/1929	5.4	22.05	14.05666667	2.1886	7.6595E-13	2.4961E-11	UP	-0.79761	0.015052	0.055247		1.3777	7.0206E-06	0.00015811	UP
transcript_HQ_CoA_transcript23800/f4p0/1865	43.10666667	81.13	35.76666667	1.0979	0.0074256	0.027486	UP	-1.3526	0.0003099	0.002442	DOWN	-0.26895	0.29164	0.50649	
transcript_HQ_CoA_transcript23805/f2p0/1929	63.42	12.37333333	6.393333333	-2.2393	0.0003034	0.0018354	DOWN	-1.0699	0.01418	0.052741		-3.3156	6.3576E-09	3.7765E-07	DOWN
transcript_HQ_CoA_transcript2381/f2p0/4808	14.04666667	3.676666667	6.723333333	-1.79	5.1879E-10	1.1238E-08	DOWN	0.7246	6.2159E-05	0.00064441		-1.077	3.3312E-05	0.00058012	DOWN
transcript_HQ_CoA_transcript23813/f2p0/1932	8.51	30.21333333	12.59333333	2.0058	2.8726E-08	4.5866E-07	UP	-1.4189	4.0796E-06	6.3154E-05	DOWN	0.5733	0.091103	0.24071	
transcript_HQ_CoA_transcript23816/f5p0/1911	16.35666667	51.19666667	45.91333333	1.8072	3.5408E-08	5.5577E-07	UP	-0.3138	0.38004	0.57242		1.48	9.8867E-07	3.1288E-05	UP
transcript_HQ_CoA_transcript2385/f13p0/4605	30.49333333	7.126666667	13.70333333	-1.9009	0.0011449	0.0057647	DOWN	0.75967	0.15765	0.31584		-1.1554	0.00049164	0.0050983	DOWN
transcript_HQ_CoA_transcript23854/f2p0/1919	8.26	1.813333333	1.79	-2.0084	8.0916E-05	0.00058563	DOWN	-0.18757	0.79508	0.88708		-2.2125	1.9885E-05	0.00037434	DOWN
transcript_HQ_CoA_transcript23859/f4p0/1888	1.586666667	4.546666667	4.12	1.6858	0.0023646	0.010658	UP	-0.27715	0.73385	0.84922		1.3904	0.12538	0.29725	
transcript_HQ_CoA_transcript2386/f5p0/4686	20.84666667	42.72333333	57.37333333	1.1832	4.2135E-05	0.00033132	UP	0.27778	0.49048	0.67059		1.4498	7.5492E-05	0.0011256	UP
transcript_HQ_CoA_transcript23861/f2p0/1929	6.003333333	12.11333333	8.42	1.1546	0.00044276	0.0025415	UP	-0.65963	0.031933	0.097647		0.48349	0.070014	0.20086	
transcript_HQ_CoA_transcript23873/f2p0/1923	6.046666667	0.49	6.076666667	-3.4722	3.0741E-07	3.9718E-06	DOWN	3.4721	3.3429E-06	5.3419E-05	UP	-0.0075192	0.98709	0.99502	
transcript_HQ_CoA_transcript2388/f2p0/4705	4.15	17.01666667	7.59	2.2025	1.5107E-07	2.0797E-06	UP	-1.3204	0.0094545	0.038339	DOWN	0.86855	0.07891	0.21732	
transcript_HQ_CoA_transcript23882/f3p0/1942	3.933333333	0.766666667	0.61	-2.2537	0.0068853	0.025842	DOWN	-0.46765	0.54784	0.71647		-2.7271	0.0005542	0.0055984	DOWN
transcript_HQ_CoA_transcript23887/f2p0/1980	5.396666667	15.29666667	8.88	1.6507	0.00016463	0.001085	UP	-0.92877	0.019834	0.06818		0.71051	0.0095732	0.048742	

transcript_HQ_CoA_transcript23912/f4p0/1915	34.12333333	100.99	49.14333333	1.7268	4.7714E-31	6.8149E-29	UP	-1.1821	0.00038896	0.0029644	DOWN	0.53074	0.10471	0.26487	
transcript_HQ_CoA_transcript23919/f6p0/1881	14.30333333	31.01333333	14.84	1.2802	1.6706E-05	0.00014635	UP	-1.2194	1.6467E-05	0.00020687	DOWN	0.048115	0.8483	0.9308	
transcript_HQ_CoA_transcript2394/f7p0/4703	1.246666667	4.013333333	1.986666667	1.7967	0.0048663	0.019412	UP	-1.1187	0.080211	0.19424		0.665	0.077922	0.2156	
transcript_HQ_CoA_transcript23941/f2p0/1921	2.006666667	9.296666667	12.86666667	2.37	0.00014845	0.0009923	UP	0.31894	0.48201	0.66414		2.6763	1.4793E-09	9.9825E-08	UP
transcript_HQ_CoA_transcript23959/f3p0/1929	14.11666667	33.02666667	14.78333333	1.3885	0.00016012	0.0010583	UP	-1.3204	5.2213E-05	0.000559	DOWN	0.05575	0.83348	0.9229	
transcript_HQ_CoA_transcript2396/f3p0/4722	9.64	3.206666667	3.32	-1.4381	0.00001505	0.00013376	DOWN	-0.089015	0.82327	0.90374		-1.5382	4.9465E-07	1.7294E-05	DOWN
transcript_HQ_CoA_transcript23960/f2p0/1916	8.45	21.05	19.33	1.4624	4.726E-06	4.7921E-05	UP	-0.24951	0.6565	0.79741		1.2001	0.039781	0.13671	
transcript_HQ_CoA_transcript23988/f3p0/1934	3.936666667	12.14	6.34	1.7532	0.00017744	0.0011615	UP	-1.0734	0.028409	0.089713		0.66886	0.27824	0.49187	
transcript_HQ_CoA_transcript24001/f2p0/1922	128.3	362.1233333	376.6333333	1.6708	0.00001143	0.00010522	UP	-0.091269	0.74626	0.8566		1.5653	3.1411E-05	0.00055278	UP
transcript_HQ_CoA_transcript24003/f2p0/1910	12.36666667	4.713333333	15.81	-1.2341	0.00048763	0.0027621	DOWN	1.5921	1.0021E-05	0.00013636	UP	0.34556	0.22115	0.4275	
transcript_HQ_CoA_transcript24011/f3p0/1928	10.54	19.2	11.82	1.0322	0.0010811	0.0054903	UP	-0.85457	0.002131	0.011663		0.1651	0.58901	0.76556	
transcript_HQ_CoA_transcript2402/f3p0/4700	5.606666667	2.426666667	7.666666667	-1.0541	0.00045659	0.002611	DOWN	1.5122	2.5847E-13	2.1633E-11	UP	0.44438	0.070414	0.2015	
transcript_HQ_CoA_transcript24021/f2p0/1913	1.106666667	9.336666667	3.353333333	3.2466	7.9959E-08	1.1639E-06	UP	-1.6519	0.0097717	0.039295	DOWN	1.5795	0.041371	0.14039	
transcript_HQ_CoA_transcript24032/f2p0/1810	14.89666667	38.03333333	13.82	1.5222	0.00012263	0.00083997	UP	-1.6227	3.9455E-05	0.00044176	DOWN	-0.11436	0.63547	0.79757	
transcript_HQ_CoA_transcript24039/f3p0/1910	50.92666667	19.92	16.62	-1.1832	5.6117E-05	0.00042641	DOWN	-0.41609	0.11891	0.25837		-1.6129	7.1346E-10	5.2174E-08	DOWN
transcript_HQ_CoA_transcript2406/f2p0/4768	0.573333333	5.286666667	1.696666667	3.3673	1.6254E-14	6.4882E-13	UP	-1.7788	0.00015148	0.0013564	DOWN	1.5764	0.0054656	0.032583	UP
transcript_HQ_CoA_transcript24062/f2p0/1917	14.12666667	43.41666667	13.69666667	1.7816	1.3248E-05	0.00011984	UP	-1.8225	2.0294E-06	3.4829E-05	DOWN	-0.05356	0.86113	0.93743	
transcript_HQ_CoA_transcript2407/f2p0/4713	8.206666667	2.066666667	1.983333333	-1.8384	0.011378	0.039115	DOWN	-0.22289	0.61172	0.76616		-2.0675	0.0019991	0.015279	DOWN
transcript_HQ_CoA_transcript24071/f5p0/1848	20.01666667	47.24	41.9	1.3757	0.00042655	0.0024655	UP	-0.2964	0.36318	0.5552		1.0698	0.0061379	0.035573	UP
transcript_HQ_CoA_transcript24078/f2p0/1916	7.643333333	15.45	7.546666667	1.1772	0.00049198	0.0027846	UP	-1.1956	7.7904E-05	0.00077985	DOWN	-0.030885	0.92858	0.96845	
transcript_HQ_CoA_transcript24098/f2p0/1902	1.163333333	10.96	17.44666667	3.3441	0.0027638	0.012137	UP	0.5417	0.43864	0.62712		3.8792	1.2283E-05	0.00025023	UP
transcript_HQ_CoA_transcript24109/f6p0/1888	11.99666667	53.87666667	18.04666667	2.3396	3.5416E-08	5.5577E-07	UP	-1.7446	7.0014E-06	0.00010002	DOWN	0.5823	0.029495	0.11039	
transcript_HQ_CoA_transcript24119/f3p0/1907	1.936666667	4.983333333	1.123333333	1.4989	0.0023083	0.010459	UP	-2.2951	6.5741E-08	1.7092E-06	DOWN	-0.80319	0.17735	0.37113	
transcript_HQ_CoA_transcript24124/f2p0/1883	15.53333333	5.37	6.263333333	-1.3691	0.00034144	0.0020379	DOWN	0.057121	0.90171	0.94785		-1.3249	0.0024846	0.017988	DOWN
transcript_HQ_CoA_transcript24127/f3p0/1930	4.53	9.423333333	2.41	1.2103	0.010663	0.037096	UP	-2.116	0.00029026	0.0023192	DOWN	-0.92033	0.06882	0.19864	
transcript_HQ_CoA_transcript24133/f2p0/1908	3.623333333	18.51333333	7.946666667	2.5438	2.3411E-07	3.1104E-06	UP	-1.3819	0.011689	0.04541	DOWN	1.1477	0.010556	0.052221	
transcript_HQ_CoA_transcript24134/f4p0/1899	48.68666667	20.70333333	31.23	-1.0795	0.00062068	0.0033974	DOWN	0.43324	0.029934	0.093056		-0.65728	0.019309	0.081197	
transcript_HQ_CoA_transcript24140/f4p0/1911	6.91	2.08	5.61	-1.5645	1.7025E-05	0.00014871	DOWN	1.2776	0.00017866	0.0015675	UP	-0.29982	0.35594	0.57314	
transcript_HQ_CoA_transcript24143/f2p0/1896	4.78	17.05333333	12.19	2.0153	1.8128E-06	2.0184E-05	UP	-0.64989	0.14195	0.29312		1.3512	0.00012169	0.0016706	UP
transcript_HQ_CoA_transcript24160/f10p0/1889	0.25	324.7266667	17.67333333	10.51	4.3974E-77	3.6543E-74	UP	-4.3262	0.0020877	0.011498	DOWN	6.1685	5.744E-08	2.6759E-06	UP
transcript_HQ_CoA_transcript24164/f4p0/1895	4.76	0.966666667	3.356666667	-2.1168	0.0098463	0.034798	DOWN	1.6324	0.00011661	0.0010888	UP	-0.50086	0.48598	0.68925	
transcript_HQ_CoA_transcript2417/f3p0/4714	0.036666667	1.353333333	0.033333333	5.143	7.5093E-05	0.00054914	UP	-5.3117	0.00050571	0.0036883	DOWN	-0.18332	0.95042	0.97852	
transcript_HQ_CoA_transcript24191/f2p0/1902	26.51666667	6.306666667	9.816666667	-1.9107	2.8759E-20	1.8319E-18	DOWN	0.48924	0.03193	0.097647		-1.4346	1.4026E-13	2.4248E-11	DOWN
transcript_HQ_CoA_transcript24192/f2p0/1900	76.80333333	26.20666667	50.38666667	-1.3927	5.4543E-13	1.7999E-11	DOWN	0.7834	0.00011307	0.0010655		-0.62149	0.0004313	0.0045949	
transcript_HQ_CoA_transcript24203/f3p0/1903	6.586666667	34.44333333	7.043333333	2.5435	6.1264E-10	1.3161E-08	UP	-2.4434	8.4121E-10	3.3592E-08	DOWN	0.087702	0.80076	0.90538	
transcript_HQ_CoA_transcript24206/f3p0/1911	5.94	24.86333333	5.353333333	2.2383	4.1102E-08	6.3573E-07	UP	-2.3831	2.103E-09	7.6177E-08	DOWN	-0.15826	0.65291	0.80984	
transcript_HQ_CoA_transcript24210/f2p0/1893	52.28666667	23.43333333	30.37666667	-1.0147	0.00010822	0.00075312	DOWN	0.23811	0.35448	0.54647		-0.78692	0.00074428	0.0070857	
transcript_HQ_CoA_transcript24224/f15p0/1820	64.99	119.8933333	103.9733333	1.0487	2.3205E-06	2.5222E-05	UP	-0.35902	0.093794	0.21795		0.67716	3.6272E-06	0.00009247	
transcript_HQ_CoA_transcript24226/f7p0/1896	8.833333333	29.62666667	12.50333333	1.8815	3.3984E-07	4.3569E-06	UP	-1.3733	0.00047855	0.0035254	DOWN	0.49567	0.14119	0.32061	
transcript_HQ_CoA_transcript24233/f4p0/1882	124.27	53.78666667	93.1	-1.0649	8.275E-07	9.8556E-06	DOWN	0.65251	0.0010414	0.0065981		-0.42347	0.006484	0.036961	
transcript_HQ_CoA_transcript24239/f2p0/1890	11.55333333	60	13.39666667	2.5412	1.1394E-08	1.9467E-07	UP	-2.3226	3.3207E-09	1.1528E-07	DOWN	0.20708	0.5217	0.71708	
transcript_HQ_CoA_transcript24250/f3p0/1889	7.846666667	16.24	9.333333333	1.2259	0.0017314	0.0082222	UP	-0.96644	0.0082423	0.0345		0.24643	0.30071	0.51667	
transcript_HQ_CoA_transcript2426/f3p0/4761	8.826666667	3.006666667	2.08	-1.3938	6.1702E-05	0.00046269	DOWN	-0.66929	0.087086	0.20644		-2.0752	1.899E-07	7.5814E-06	DOWN
transcript_HQ_CoA_transcript24267/f3p0/1914	45.66333333	20.08333333	28.43	-1.0277	1.1213E-05	0.00010364	DOWN	0.35668	0.082789	0.19859		-0.68395	0.0037894	0.024841	
transcript_HQ_CoA_transcript24269/f2p0/1903	64.04333333	21.25666667	46.44666667	-1.4533	1.4709E-05	0.00013117	DOWN	0.98116	2.9403E-06	4.8257E-05		-0.48119	0.11368	0.27917	
transcript_HQ_CoA_transcript24303/f8p0/1873	8.02	35.13	9.673333333	2.3032	4.7486E-08	7.2709E-07	UP	-2.015	8.396E-06	0.00011667	DOWN	0.2731	0.41369	0.62754	
transcript_HQ_CoA_transcript24309/f13p0/1833	0.33	158.36	197.2166667	9.105	1.0588E-59	3.9505E-57	UP	0.22372	0.90132	0.9477		9.316	4.0617E-07	1.4561E-05	UP
transcript_HQ_CoA_transcript24314/f3p0/1888	30.19666667	11.82333333	25.63666667	-1.1945	3.5311E-05	0.00028426	DOWN	0.9644	0.0013005	0.0078774		-0.24227	0.45972	0.66816	
transcript_HQ_CoA_transcript24319/f3p0/1890	23.9	9.196666667	17.37	-1.2296	4.1168E-06	4.2142E-05	DOWN	0.77393	0.0040699	0.019972		-0.46577	0.064677	0.19061	
transcript_HQ_CoA_transcript24322/f2p0/1894	42.71333333	13.81666667	50.68666667	-1.4977	0.00014643	0.00098116	DOWN	1.7313	1.0178E-09	3.9692E-08	UP	0.22429	0.52022	0.71611	
transcript_HQ_CoA_transcript24323/f2p0/1914	6.673333333	0.19	6.36	-5.0125	1.3549E-09	2.737E-08	DOWN	4.9274	2.3067E-19	5.0332E-17	UP	-0.091903	0.87157	0.94284	
transcript_HQ_CoA_transcript2433/f9p0/4664	0.076666667	39.39333333	1.896666667	9.1627	2.8957E-44	6.8753E-42	UP	-4.5195	1.7539E-08	5.1683E-07	DOWN	4.6297	0.00007337	0.0011019	UP
transcript_HQ_CoA_transcript24331/f2p0/1896	9.593333333	48.75	14.20333333	2.5086	6.4473E-10	1.3802E-08	UP	-1.9299	6.8409E-06	9.7959E-05	DOWN	0.56426	0.067493	0.19599	
transcript_HQ_CoA_transcript24333/f4p0/1865	63.95666667	16.53333333	62.68	-1.8028	6.2437E-18	3.3474E-16	DOWN	1.7638	6.0614E-06	8.8244E-05	UP	-0.05064	0.89037	0.9511	
transcript_HQ_CoA_transcript24340/f2p0/1908	17.51333333	5.11	8.406666667	-1.6375	0.00043563	0.00251	DOWN	0.56627	0.12737	0.27149		-1.0808	0.014432	0.065374	
transcript_HQ_CoA_transcript24344/f4p0/1883	14.38333333	4.476666667	8.32	-1.5385	2.7325E-08	4.3936E-07	DOWN	0.75181	0.0017472	0.0099919		-0.79852	0.00064274	0.0063088	
transcript_HQ_CoA_transcript24359/f2p0/1707	17.35	51.7	24.85333333	1.7485	3.7258E-09	6.9733E-08	UP	-1.2149	2.6333E-05	0.00030939	DOWN	0.51964	0.020547	0.084772	
transcript_HQ_CoA_transcript24361/f3p0/1890	7.663333333	13.96333333	9.123333333	1.0209	9.6187E-05	0.00067974	UP	-0.76652	0.00073121	0.0049666		0.24199	0.3139	0.53069	
transcript_HQ_CoA_transcript24363/f2p0/1885	35.83	10.66	12.08666667	-1.6074	1.8363E-09	3.6177E-08	DOWN	0.043346	0.87422	0.93169		-1.5761	2.8403E-19	1.1448E-16	DOWN
transcript_HQ_CoA_transcript24414/f3p0/1881	9.946666667	40.48666667	15.52666667	2.1979	1.0695E-06	1.2494E-05	UP	-1.5363	0.00093403	0.006043	DOWN	0.64666	0.048907	0.1579	
transcript_HQ_CoA_transcript24416/f2p0/1888	2.866666667	0.553333333	0.473333333	-2.2387	0.0012155	0.0060699	DOWN	-0.37772	0.66823	0.80595		-2.6231	1.4739E-05	0.00029279	DOWN
transcript_HQ_CoA_transcript2442/f2p0/4679	14.57	4.923333333	7.493333333	-1.4207	2.5117E-07	3.3082E-06	DOWN	0.46724	0.076555	0.18797		-0.9655	3.0767E-06	8.1138E-05	
transcript_HQ_CoA_transcript24425/f2p0/1891	5.373333333	16.56333333	5.976666667	1.7883	0.00033085	0.0019838	UP	-1.6145	0.00023421	0.0019442	DOWN	0.15918	0.67172	0.82218	
transcript_HQ_CoA_transcript24439/f3p0/1879	11.77	31.34	15.91	1.5593	3.0425E-07	3.9366E-06	UP	-1.1102	0.0031765	0.016222	DOWN	0.43665	0.21329	0.41799	
transcript_HQ_CoA_transcript24446/f2p0/1931	6.613333333	1.16	4.216666667	-2.4175	0.00067765	0.0036588	DOWN	1.7657	0.0075678	0.032341	UP	-0.65876	0.1416	0.32113	
transcript_HQ_CoA_transcript24447/f2p0/1878	18.15	94.34666667	25.34333333	2.5393	4.4223E-13	1.47E-11	UP	-2.0493	7.6468E-08	1.9279E-06	DOWN	0.47637	0.10542	0.26601	
transcript_HQ_CoA_transcript2445/f2p0/4687	2.876666667	0.466666667	2.726666667	-2.5083	0.00024811	0.0015434	DOWN	2.4054	1.4443E-05	0.000185	UP	-0.11032	0.83445	0.92323	
transcript_HQ_CoA_transcript24455/f6p0/1874	26.81666667	84.95333333	27.27333333	1.814	0.0002148	0.0013688	UP	-1.7921	0.00002158	0.00026108	DOWN	0.010923	0.97919	0.99173	
transcript_HQ_CoA_transcript24461/f2p0/1892	6.99	18.26	8.713333333	1.536	0.00012206	0.00083673	UP	-1.2072	3.462E-07	7.5541E-06	DOWN	0.31857	0.45615	0.66542	
transcript_HQ_CoA_transcript24465/f2p0/1888	31.92666667	71.85	76.50333333	1.3413	0.0097585	0.034521	UP	-0.067915	0.73414	0.84939		1.2574	0.012351	0.05861	
transcript_HQ_CoA_transcript24472/f2p0/1872	4.796666667	21.60333333	5.706666667	2.3222	1.4645E-06	1.6672E-05	UP	-2.0713	1.2148E-06	2.2378E-05	DOWN	0.23805	0.61778	0.78505	
transcript_HQ_CoA_transcript24485/f3p0/1903	7.95	2.346666667	5.7	-1.6117	2.5318E-06	2.7227E-05	DOWN	1.1304	0.00023219	0.0019292	UP	-0.49385	0.10121	0.25925	
transcript_HQ_CoA_transcript24488/f3p0/1879	78.49	144.2966667	100.8833333	1.0287	5.6633E-06	5.6577E-05	UP	-0.66036	6.2815E-09	2.0414E-07		0.35692	0.1001	0.25725	
transcript_HQ_CoA_transcript24492/f2p0/1903	9.416666667	24.68	21.99	1.574	6.7185E-05	0.00049788	UP	-0.32829	0.21275	0.38889		1.23	0.00024936	0.002974	UP
transcript_HQ_CoA_transcript24499/f3p0/1876	92.61666667	32.16	15.15666667	-1.369	0.0013602	0.0067011	DOWN	-1.2507	0.01112	0.043609	DOWN	-2.6278	3.0826E-09	1.9715E-07	DOWN
transcript_HQ_CoA_transcript24504/f2p0/1889	3.253333333	8.416666667	2.11	1.5552	0.0095849	0.033986	UP	-2.1579	2.7834E-09	9.8108E-08	DOWN	-0.61629	0.3378	0.55569	
transcript_HQ_CoA_transcript24505/f2p0/1893	2.21	6.313333333	38.98333333	1.687	0.0012208	0.0060932	UP	2.529	0.056221	0.14977		4.1999	0.0021157	0.015974	UP
transcript_HQ_CoA_transcript24506/f3p0/1952	5.356666667	17.16	9	1.8671	0.00001411	0.00012626	UP	-1.1044	0.0041701	0.020355	DOWN	0.74881	0.010171	0.050913	
transcript_HQ_CoA_transcript24512/f2p0/1879	7.796666667	3.263333333	5.523333333	-1.0751	0.013604	0.045025	DOWN	0.58496	0.1712	0.33422		-0.50258	0.051998	0.16479	

transcript_HQ_CoA_transcript24522/f2p0/1885	4.063333333	1.546666667	1.87	-1.2398	0.012088	0.041038	DOWN	0.1401	0.79422	0.88677		-1.1133	0.014042	0.064067	
transcript_HQ_CoA_transcript24528/f2p0/1878	1.04	55.45666667	24.67	5.8933	2.6108E-34	4.3392E-32	UP	-1.3166	0.00017023	0.0015001	DOWN	4.5624	1.4883E-17	4.7925E-15	UP
transcript_HQ_CoA_transcript24531/f4p0/1857	39.32	16.6	34.71666667	-1.0874	2.5205E-11	6.7989E-10	DOWN	0.91466	5.6969E-06	8.3602E-05		-0.18484	0.35685	0.57386	
transcript_HQ_CoA_transcript24543/f2p0/1890	1.98	7.236666667	2.063333333	2.0563	8.1079E-06	7.8207E-05	UP	-1.9742	7.6969E-07	1.5334E-05	DOWN	0.068259	0.88684	0.9495	
transcript_HQ_CoA_transcript24545/f3p0/1873	64.15	22.67666667	23.85	-1.3577	2.8067E-07	0.00000366	DOWN	-0.064933	0.79942	0.8896		-1.4351	8.5268E-14	1.5344E-11	DOWN
transcript_HQ_CoA_transcript24556/f4p0/1860	22.52	48.98666667	38.51666667	1.2852	6.6234E-12	1.916E-10	UP	-0.4988	0.00029273	0.0023359		0.77334	0.00004244	0.00069825	
transcript_HQ_CoA_transcript24577/f2p0/1866	13.84	27.17	16.41666667	1.1454	0.00055198	0.0030691	UP	-0.89191	0.0041115	0.020133		0.24026	0.3107	0.5275	
transcript_HQ_CoA_transcript24590/f2p0/1866	3.74	18.02	1.003333333	2.4393	5.3753E-07	6.6043E-06	UP	-4.3107	3.6964E-28	2.4197E-25	DOWN	-1.8932	0.0035492	0.023638	DOWN
transcript_HQ_CoA_transcript24624/f2p0/1872	31.22	60.41333333	31.01333333	1.1088	0.0011112	0.0056168	UP	-1.1057	0.010274	0.040893	DOWN	-0.014049	0.97488	0.98955	
transcript_HQ_CoA_transcript24631/f3p0/1862	63.52666667	22.18333333	23.81666667	-1.3592	2.0258E-15	8.7762E-14	DOWN	-0.044195	0.76118	0.86565		-1.4158	5.3269E-17	1.6029E-14	DOWN
transcript_HQ_CoA_transcript24646/f2p0/1881	2.953333333	47.11666667	13.74666667	4.1843	1.3967E-10	3.3293E-09	UP	-1.9213	1.3731E-08	4.1696E-07	DOWN	2.2445	0.00036438	0.0040271	UP
transcript_HQ_CoA_transcript24651/f3p0/1852	61.92333333	175.3533333	75.77333333	1.673	2.2936E-10	5.2612E-09	UP	-1.3678	6.3271E-07	1.2885E-05	DOWN	0.29154	0.18711	0.38417	
transcript_HQ_CoA_transcript24658/f3p0/1855	37.77	72.71	52.78	1.1052	2.014E-07	2.7019E-06	UP	-0.61156	0.001606	0.0093213		0.48104	0.0014954	0.012199	
transcript_HQ_CoA_transcript24659/f3p0/1951	7.166666667	20.07	13.48	1.644	0.0010485	0.0053543	UP	-0.71029	0.26207	0.44746		0.91937	0.066507	0.19414	
transcript_HQ_CoA_transcript24664/f3p0/1956	11.36333333	57.16333333	14.93666667	2.4963	3.0785E-11	8.098E-10	UP	-2.0973	6.4481E-08	1.6884E-06	DOWN	0.38564	0.25856	0.46919	
transcript_HQ_CoA_transcript24670/f3p0/1862	4.37	9.216666667	13.42333333	1.2405	0.00012269	0.00084008	UP	0.38657	0.23387	0.41444		1.6141	2.1563E-06	6.0611E-05	UP
transcript_HQ_CoA_transcript24677/f6p0/1857	24.98333333	9.64	21.51333333	-1.2465	0.0022386	0.010186	DOWN	1.0204	0.0093935	0.038125	UP	-0.23461	0.54681	0.73599	
transcript_HQ_CoA_transcript24678/f2p0/1875	4.78	11.61666667	10.45666667	1.4702	0.0054037	0.021133	UP	-0.30777	0.34717	0.53928		1.1477	0.022358	0.089778	
transcript_HQ_CoA_transcript24687/f2p0/1859	4.136666667	21.69	17.45666667	2.6255	0.0024384	0.010926	UP	-0.49406	0.54184	0.71167		2.1096	0.008664	0.045409	UP
transcript_HQ_CoA_transcript24694/f3p0/1861	0.07	787.82	296.7733333	13.871	2.432E-53	7.4103E-51	UP	-1.5286	0.50459	0.68155		12.328	0.001018	0.0090798	UP
transcript_HQ_CoA_transcript24695/f2p0/1868	0	73.7	5.583333333	12.909	7.2389E-24	5.8819E-22	UP	-3.8703	0.029909	0.09303		9.0224	0.020947	0.085956	
transcript_HQ_CoA_transcript24698/f9p0/1847	21.62666667	45.48333333	35.63333333	1.2349	5.5971E-11	1.4036E-09	UP	-0.48392	0.23018	0.41013		0.73687	0.069347	0.19954	
transcript_HQ_CoA_transcript24702/f2p0/1868	1.653333333	20.36666667	6.15	3.7825	6.4103E-19	3.6854E-17	UP	-1.8631	0.012623	0.0482	DOWN	1.9041	0.018974	0.080098	
transcript_HQ_CoA_transcript24716/f2p0/1786	17.71	7.853333333	11.05333333	-1.0074	0.0029276	0.012728	DOWN	0.34287	0.31219	0.50372		-0.67667	0.085932	0.2309	
transcript_HQ_CoA_transcript24728/f2p0/1878	0.37	44.60666667	8.22	7.0024	2.3816E-36	4.3109E-34	UP	-2.5529	0.1664	0.32776		4.4376	0.024103	0.094777	
transcript_HQ_CoA_transcript2474/f2p0/4665	1.313333333	3.246666667	1.896666667	1.4641	0.00026853	0.0016541	UP	-0.93205	3.5109E-05	0.00040025		0.52047	0.21467	0.41983	
transcript_HQ_CoA_transcript24745/f2p0/1870	15.87666667	34.61666667	27.80666667	1.2795	1.0309E-09	2.1418E-08	UP	-0.46297	0.026006	0.083779		0.80488	0.00094928	0.0085706	
transcript_HQ_CoA_transcript2475/f2p0/4721	8.57	16.55333333	17.37666667	1.1005	0.0054049	0.021133	UP	-0.065425	0.85378	0.92014		1.0213	1.2309E-05	0.00025049	UP
transcript_HQ_CoA_transcript24755/f4p0/1783	46.04333333	87.43666667	53.06333333	1.1089	0.0080886	0.029563	UP	-0.892	0.025795	0.083381		0.20345	0.30342	0.52006	
transcript_HQ_CoA_transcript24766/f2p0/1866	7.99	47.56333333	17.48666667	2.7374	1.7703E-31	2.5891E-29	UP	-1.6001	9.1796E-11	4.5229E-09	DOWN	1.1235	6.8663E-05	0.001045	UP
transcript_HQ_CoA_transcript24773/f3p0/1820	10.72666667	55.87666667	68.41666667	2.5541	2.3761E-06	0.00002575	UP	0.14287	0.74108	0.85391		2.6825	1.8171E-09	1.1997E-07	UP
transcript_HQ_CoA_transcript24778/f2p0/1861	5.963333333	1.073333333	5.086666667	-2.3095	2.1409E-05	0.00018273	DOWN	2.081	0.00013445	0.0012291	UP	-0.23872	0.60841	0.77905	
transcript_HQ_CoA_transcript24779/f2p0/1857	24.24666667	45.21666667	52.39666667	1.0408	0.0021059	0.0096833	UP	0.072032	0.75537	0.86219		1.1018	6.5348E-05	0.0010054	UP
transcript_HQ_CoA_transcript24780/f4p0/1827	11.65	34.37333333	13.81333333	1.7423	2.4706E-07	3.2589E-06	UP	-1.4769	1.1097E-05	0.00014836	DOWN	0.25071	0.37622	0.5929	
transcript_HQ_CoA_transcript24781/f4p0/1793	12.37	26.18666667	13.84	1.2393	0.0047827	0.01912	UP	-1.0698	0.0060214	0.027176	DOWN	0.15736	0.60861	0.77913	
transcript_HQ_CoA_transcript24786/f3p0/1862	28.88333333	10.92333333	20.78	-1.2581	9.9756E-08	1.4187E-06	DOWN	0.78006	0.0033437	0.016946		-0.48838	0.077677	0.21518	
transcript_HQ_CoA_transcript24788/f2p0/1850	19.26666667	8.416666667	19.58	-1.042	0.00018208	0.0011846	DOWN	1.062	0.0013576	0.0081615	UP	0.0081077	0.97983	0.99209	
transcript_HQ_CoA_transcript24791/f7p0/1845	11.17666667	31.24	28.86666667	1.6374	0.00038146	0.0022446	UP	-0.27174	0.59364	0.75315		1.3521	0.020015	0.083174	
transcript_HQ_CoA_transcript24796/f2p0/1901	13.94666667	0.78	10.11	-4.0055	5.0939E-10	1.1087E-08	DOWN	3.5754	1.0694E-06	2.0062E-05	UP	-0.4486	0.513	0.7106	
transcript_HQ_CoA_transcript24803/f5p0/1814	5.49	56.25333333	14.68666667	3.544	1.5485E-19	9.1323E-18	UP	-2.1089	9.4763E-11	4.6396E-09	DOWN	1.4201	2.3634E-05	0.00043294	UP
transcript_HQ_CoA_transcript24813/f2p0/1866	12.08666667	1.013333333	0.983333333	-3.3909	3.0496E-05	0.00025035	DOWN	-0.22783	0.81703	0.90013		-3.6365	1.474E-09	9.9825E-08	DOWN
transcript_HQ_CoA_transcript24829/f9p0/1809	6.75	53.84	74.34666667	3.1769	2.5377E-07	3.3353E-06	UP	0.31038	0.41734	0.607		3.471	4.027E-10	3.1188E-08	UP
transcript_HQ_CoA_transcript24869/f2p0/1855	28.23333333	11.77666667	18.14	-1.1307	0.0041254	0.016948	DOWN	0.49305	0.15316	0.30924		-0.64533	0.10516	0.26566	
transcript_HQ_CoA_transcript2487/f2p0/4670	1.033333333	0.273333333	0.32	-1.8062	0.0036048	0.015192	DOWN	0.11599	0.85836	0.9227		-1.6987	0.0066736	0.037711	DOWN
transcript_HQ_CoA_transcript24880/f4p0/1838	1.386666667	31.65666667	6.736666667	4.701	4.9484E-24	4.0568E-22	UP	-2.3655	0.00026906	0.0021821	DOWN	2.3194	0.0034973	0.023346	UP
transcript_HQ_CoA_transcript24891/f2p0/1847	4.143333333	32.65333333	7.45	3.1732	1.3942E-06	0.00001596	UP	-2.2678	7.871E-07	1.5647E-05	DOWN	0.8878	0.25739	0.46781	
transcript_HQ_CoA_transcript24892/f2p0/1858	1.263333333	3.716666667	1.786666667	1.734	0.0057811	0.02233	UP	-1.2077	0.04833	0.13299		0.51048	0.39322	0.60897	
transcript_HQ_CoA_transcript24893/f3p0/1844	43.49	7.273333333	14.68333333	-2.3392	0.0065878	0.02492	DOWN	0.81072	0.30216	0.49248		-1.5452	0.0076001	0.041244	DOWN
transcript_HQ_CoA_transcript24921/f2p0/1854	18.44	4.406666667	9.363333333	-1.8953	1.6905E-06	1.8961E-05	DOWN	0.9232	0.011524	0.04486		-0.98378	0.0014679	0.012075	
transcript_HQ_CoA_transcript24929/f11p0/1826	76.37	13.06	37.30333333	-2.3739	1.0088E-07	1.4308E-06	DOWN	1.3372	0.0038873	0.01921	UP	-1.0473	0.014617	0.065953	
transcript_HQ_CoA_transcript24933/f2p0/1888	19.89	48.16666667	20.46666667	1.4338	2.183E-06	2.3841E-05	UP	-1.3852	1.3902E-06	2.5204E-05	DOWN	0.035881	0.84655	0.92989	
transcript_HQ_CoA_transcript24935/f3p0/1861	24.8	8.103333333	9.666666667	-1.4709	1.2633E-05	0.00011479	DOWN	0.10773	0.73639	0.85119		-1.3731	4.0893E-05	0.00067742	DOWN
transcript_HQ_CoA_transcript24950/f2p0/1858	12.62666667	5.283333333	10.91333333	-1.1013	6.9156E-05	0.00051109	DOWN	0.88469	0.02196	0.073788		-0.22685	0.53425	0.72692	
transcript_HQ_CoA_transcript24957/f2p0/1873	6.99	2.903333333	3.77	-1.1344	0.011811	0.040277	DOWN	0.25402	0.55009	0.71814		-0.8899	0.018673	0.079163	
transcript_HQ_CoA_transcript24977/f3p0/1830	5.876666667	48.56	22.69666667	3.243	7.4828E-11	1.8387E-09	UP	-1.2746	0.0049237	0.023165	DOWN	1.9517	1.5296E-07	6.3093E-06	UP
transcript_HQ_CoA_transcript24984/f2p0/1922	8.456666667	23.90666667	19.45	1.6465	2.7759E-05	0.00022963	UP	-0.44046	0.21572	0.39246		1.1939	1.8753E-07	7.5233E-06	UP
transcript_HQ_CoA_transcript24997/f2p0/1836	0.956666667	6.33	2.143333333	2.9238	3.2527E-05	0.00026536	UP	-1.7513	0.015227	0.055719		1.1606	0.10601	0.2667	
transcript_HQ_CoA_transcript25/f3p0/8779	3.536666667	8.016666667	2.523333333	1.3061	0.012606	0.042433	UP	-1.784	0.00091549	0.005944	DOWN	-0.48893	0.16187	0.35017	
transcript_HQ_CoA_transcript2500/f7p0/4656	3.383333333	6.913333333	7.19	1.1674	0.013274	0.044154	UP	-0.096089	0.76061	0.86531		1.0616	0.029032	0.1092	
transcript_HQ_CoA_transcript25000/f3p0/1848	107.6533333	36.99666667	72.02	-1.3767	6.1374E-11	1.5349E-09	DOWN	0.80981	5.9977E-09	1.9666E-07		-0.58057	0.0046112	0.028817	
transcript_HQ_CoA_transcript25008/f2p0/1834	36.68666667	69.60666667	54.08666667	1.1001	7.1282E-05	0.0005242	UP	-0.5251	0.029496	0.092115		0.56169	0.0038026	0.02491	
transcript_HQ_CoA_transcript25023/f3p0/1840	12.55333333	43.12666667	19.29666667	1.9089	7.2001E-05	0.00052907	UP	-1.2843	0.0074549	0.031969	DOWN	0.61273	0.042803	0.14355	
transcript_HQ_CoA_transcript25024/f3p0/1859	13.26333333	25.57666667	20.68666667	1.1097	3.3234E-05	0.00026976	UP	-0.4598	0.022533	0.075256		0.63761	0.012711	0.059745	
transcript_HQ_CoA_transcript25033/f8p0/1771	1.336666667	8.83	87.31333333	2.8953	2.9821E-10	6.7224E-09	UP	3.1997	0.030115	0.093446		6.0784	6.0972E-05	0.00094601	UP
transcript_HQ_CoA_transcript25036/f2p0/1829	15.76666667	4.736666667	13.98666667	-1.5776	0.0025892	0.011488	DOWN	1.4042	0.0059367	0.026841	UP	-0.18566	0.75418	0.87647	
transcript_HQ_CoA_transcript25039/f2p0/1821	25.34	6.053333333	33.58333333	-1.93	4.5257E-08	6.9471E-07	DOWN	2.3279	1.6984E-06	2.9932E-05	UP	0.38683	0.32329	0.54038	
transcript_HQ_CoA_transcript25052/f2p0/1827	30.94333333	72.01	47.32666667	1.3701	1.9098E-10	4.4364E-09	UP	-0.74948	0.00067506	0.0046604		0.60922	0.019843	0.08271	
transcript_HQ_CoA_transcript25062/f3p0/1842	1.813333333	196.6	9.953333333	6.9613	5.5272E-39	1.1104E-36	UP	-4.4648	5.8269E-05	0.00061099	DOWN	2.4802	0.0027968	0.019704	UP
transcript_HQ_CoA_transcript25069/f2p0/1831	9.41	30.08	12.77333333	1.8417	5.3066E-05	0.00040542	UP	-1.3963	0.0012409	0.0075717	DOWN	0.43286	0.10164	0.26006	
transcript_HQ_CoA_transcript25095/f14p0/1805	141.42	29.12	179.38	-2.1349	1.1005E-09	2.2683E-08	DOWN	2.4751	1.1169E-10	5.4157E-09	UP	0.32838	0.37924	0.59562	
transcript_HQ_CoA_transcript25102/f5p0/1826	14.99666667	34.46333333	14.79333333	1.3543	1.0638E-08	1.8245E-07	UP	-1.3624	2.1606E-12	1.535E-10	DOWN	-0.021439	0.92486	0.96645	
transcript_HQ_CoA_transcript25131/f2p0/1828	5.053333333	9.29	6.013333333	1.0524	0.010847	0.037641	UP	-0.79468	0.020771	0.070594		0.24351	0.5056	0.70573	
transcript_HQ_CoA_transcript25137/f3p0/1821	6.256666667	28.34	21.01333333	2.347	8.0381E-08	1.1681E-06	UP	-0.58785	0.25412	0.43811		1.7453	0.00035137	0.0039016	UP
transcript_HQ_CoA_transcript2514/f2p0/4669	12.59666667	4.833333333	6.71	-1.2295	3.0261E-07	3.9199E-06	DOWN	0.32848	0.14562	0.29831		-0.91351	3.9546E-06	9.8891E-05	
transcript_HQ_CoA_transcript25140/f2p0/1832	3.036666667	72.83333333	23.42333333	4.7542	0.0024722	0.011051	UP	-1.7436	0.38449	0.57698		2.9937	0.30641	0.52323	
transcript_HQ_CoA_transcript25141/f2p0/1836	237.8133333	21.21666667	46.45	-3.3543	0.0040565	0.016729	DOWN	1.014	0.25869	0.4433		-2.3514	1.1036E-07	4.7218E-06	DOWN
transcript_HQ_CoA_transcript25142/f2p0/1832	9.306666667	2.56	5.2	-1.7148	6.8649E-08	1.0121E-06	DOWN	0.89137	0.020479	0.069797		-0.83465	0.010147	0.050873	
transcript_HQ_CoA_transcript25144/f2p0/1839	1.873333333	166.41	13.96	6.6452	7.8913E-57	2.7221E-54	UP	-3.714	1.0183E-08	3.2015E-07	DOWN	2.9152	1.0739E-05	0.00022323	UP
transcript_HQ_CoA_transcript25145/f3p0/1827	6.073333333	179.3	14.4	5.0606	2.2894E-54	7.2164E-52	UP	-3.7918	1.6451E-10	7.6142E-09	DOWN	1.2528	0.03737	0.13044	

transcript_HQ_CoA_transcript25150/f8p0/1795	116.1566667	298.9566667	333.1533333	1.5346	1.808E-06	2.0142E-05	UP	-0.0025481	0.99517	0.99746		1.5151	0.0002679	0.0031663	UP
transcript_HQ_CoA_transcript25172/f3p0/1824	29.94	7.083333333	16.24666667	-1.9346	2.0798E-06	0.00002285	DOWN	1.0509	0.0015273	0.0089411	UP	-0.8918	0.01484	0.066714	
transcript_HQ_CoA_transcript25173/f2p0/1826	3.503333333	7.513333333	5.71	1.2459	0.0022528	0.01024	UP	-0.54828	0.031517	0.096713		0.68615	0.10628	0.26706	
transcript_HQ_CoA_transcript25179/f2p0/1827	0.793333333	5.603333333	1.14	2.9722	4.2369E-10	9.3324E-09	UP	-2.4442	5.0993E-07	0.00001078	DOWN	0.51445	0.45889	0.66759	
transcript_HQ_CoA_transcript25187/f2p0/1827	53.66	114.72	75.93333333	1.261	0.00027207	0.0016745	UP	-0.74473	0.043632	0.123		0.50502	0.056752	0.17466	
transcript_HQ_CoA_transcript25188/f2p0/1833	31.68	61.50666667	33.35666667	1.1252	2.8576E-05	0.00023607	UP	-1.0381	4.2212E-05	0.00046727	DOWN	0.074053	0.7568	0.87874	
transcript_HQ_CoA_transcript25189/f10p0/1809	33.35333333	62.93333333	42.25	1.0883	0.00000763	7.4119E-05	UP	-0.72388	0.0038598	0.019095		0.35015	0.2044	0.4067	
transcript_HQ_CoA_transcript25190/f2p0/1826	7.713333333	87.54333333	26.16666667	3.6752	2.1787E-21	1.4974E-19	UP	-1.9051	3.7674E-07	8.143E-06	DOWN	1.7563	2.0454E-15	5.0058E-13	UP
transcript_HQ_CoA_transcript25193/f9p0/1806	0.093333333	126.15	1.916666667	10.391	2.4626E-10	5.6207E-09	UP	-6.1284	1.8193E-06	3.1694E-05	DOWN	4.2546	0.012122	0.057809	
transcript_HQ_CoA_transcript25196/f2p0/1820	31.10333333	13.34333333	21.07666667	-1.0759	0.0062618	0.02388	DOWN	0.51204	0.0073267	0.031509		-0.57276	0.11052	0.27454	
transcript_HQ_CoA_transcript25198/f77p0/1762	939.51	398.5666667	421.3066667	-1.0931	0.0023014	0.010432	DOWN	-0.065307	0.6434	0.7876		-1.1681	0.00064008	0.0062894	DOWN
transcript_HQ_CoA_transcript25208/f2p0/1825	8.903333333	2	3.25	-1.9884	3.1413E-07	4.0386E-06	DOWN	0.54356	0.17729	0.3426		-1.4574	2.3572E-06	6.5061E-05	DOWN
transcript_HQ_CoA_transcript25223/f2p0/1842	9.063333333	78.85666667	13.53666667	3.2903	7.5932E-12	2.1793E-10	UP	-2.6948	1.1432E-06	2.1251E-05	DOWN	0.58038	0.14605	0.32756	
transcript_HQ_CoA_transcript25246/f2p0/1820	68.66	15.49666667	58.89	-2.009	9.7321E-13	3.1214E-11	DOWN	1.7814	4.5336E-07	9.6512E-06	UP	-0.23777	0.4512	0.66132	
transcript_HQ_CoA_transcript25247/f2p0/1826	166.9966667	328.73	285.7033333	1.1358	6.788E-09	1.2085E-07	UP	-0.35058	0.075613	0.18653		0.77256	0.00087663	0.0080614	
transcript_HQ_CoA_transcript25268/f3p0/1834	1.626666667	14.48333333	1.303333333	3.3117	4.8442E-09	8.8473E-08	UP	-3.6322	7.4107E-12	4.4829E-10	DOWN	-0.33622	0.55926	0.74445	
transcript_HQ_CoA_transcript25273/f2p0/1830	7.65	16.39333333	28.58333333	1.2641	6.7891E-08	1.0026E-06	UP	0.65191	0.0085076	0.035383		1.9041	1.3638E-11	1.4901E-09	UP
transcript_HQ_CoA_transcript25276/f2p0/1811	5.933333333	12.42	9.84	1.2329	4.1575E-05	0.00032747	UP	-0.4861	0.070325	0.17679		0.73471	0.01605	0.070604	
transcript_HQ_CoA_transcript25292/f2p0/1816	55.01333333	20.38666667	25.78	-1.2963	0.00017772	0.0011627	DOWN	0.19829	0.48827	0.66887		-1.1084	0.00005688	0.00089157	DOWN
transcript_HQ_CoA_transcript25294/f2p0/1845	13.19	34.37	10.55666667	1.5182	0.0040684	0.016765	UP	-1.8734	0.0016882	0.0097028	DOWN	-0.36269	0.61158	0.78046	
transcript_HQ_CoA_transcript25298/f2p0/1818	17.61666667	32.9	28.19	1.0918	0.014707	0.047781	UP	-0.38837	0.18585	0.35407		0.68642	0.091176	0.24081	
transcript_HQ_CoA_transcript25311/f2p0/1823	0.303333333	2.436666667	9.416666667	3.1715	0.0032003	0.013712	UP	1.8572	0.088485	0.20889		5.0128	7.4166E-05	0.0011086	UP
transcript_HQ_CoA_transcript25314/f4p0/1818	8.94	17.87666667	9.9	1.1363	0.0015717	0.0075733	UP	-0.98787	0.0014401	0.008559		0.13678	0.62068	0.78743	
transcript_HQ_CoA_transcript25317/f3p0/1813	2.976666667	45.95666667	8.29	4.1132	1.4538E-22	1.0589E-20	UP	-2.6266	5.5682E-09	1.8422E-07	DOWN	1.4729	0.00036464	0.0040271	UP
transcript_HQ_CoA_transcript25326/f7p0/1787	18.88	2.533333333	16.22333333	-2.7358	1.077E-12	3.4367E-11	DOWN	2.5176	6.9806E-08	1.7895E-06	UP	-0.23236	0.61394	0.78215	
transcript_HQ_CoA_transcript2533/f2p0/4639	2.083333333	0.443333333	1.306666667	-2.1126	0.0010414	0.0053301	DOWN	1.4502	0.078855	0.19233		-0.67196	0.38305	0.59878	
transcript_HQ_CoA_transcript25333/f3p0/1823	87.92333333	37.82333333	69.82	-1.0461	5.6766E-05	0.00043044	DOWN	0.72067	0.0050811	0.02377		-0.33858	0.065125	0.19143	
transcript_HQ_CoA_transcript25337/f10p0/1784	28.70666667	8.266666667	11.04333333	-1.6539	0.0001694	0.0011132	DOWN	0.30174	0.54334	0.71304		-1.3664	3.8664E-06	9.7066E-05	DOWN
transcript_HQ_CoA_transcript25343/f2p0/1831	56.05333333	21.98	74.24	-1.1776	5.6558E-06	5.6533E-05	DOWN	1.5924	7.9739E-07	1.5797E-05	UP	0.40233	0.19147	0.38978	
transcript_HQ_CoA_transcript25352/f2p0/1817	17.41666667	32.40333333	20.50666667	1.0488	9.6797E-07	1.1416E-05	UP	-0.80979	4.4993E-05	0.00049363		0.2273	0.352	0.5695	
transcript_HQ_CoA_transcript25370/f2p0/1858	4.066666667	0.71	2.22	-2.3202	3.8654E-06	3.9835E-05	DOWN	1.4667	0.0023372	0.012599	UP	-0.86754	0.038784	0.13432	
transcript_HQ_CoA_transcript25375/f2p0/1795	49.1	8.62	39.96	-2.3804	0.00026239	0.0016217	DOWN	2.0773	0.00031968	0.0025062	UP	-0.30903	0.65949	0.81373	
transcript_HQ_CoA_transcript25394/f2p0/1801	22.60666667	58.61	23.59666667	1.5314	5.717E-07	6.9912E-06	UP	-1.4694	1.2173E-06	2.2402E-05	DOWN	0.049242	0.82991	0.92119	
transcript_HQ_CoA_transcript25402/f2p0/1806	6.58	12.40333333	6.743333333	1.0625	0.010314	0.036183	UP	-1.0271	0.0070987	0.030692	DOWN	0.022974	0.94274	0.97468	
transcript_HQ_CoA_transcript25409/f4p0/1788	33.09333333	9.393333333	53.58666667	-1.6481	2.9273E-05	0.00024118	DOWN	2.3428	3.4148E-09	1.1832E-07	UP	0.68401	0.010165	0.050913	
transcript_HQ_CoA_transcript25419/f2p0/1810	25.96666667	54.11333333	34.25333333	1.2115	2.8417E-07	3.6977E-06	UP	-0.80433	0.00013706	0.0012467		0.3955	0.075705	0.21118	
transcript_HQ_CoA_transcript25422/f2p0/1833	15.68666667	5.23	9.42	-1.4415	4.2602E-07	5.3443E-06	DOWN	0.70595	0.01431	0.053183		-0.74626	0.0028749	0.020095	
transcript_HQ_CoA_transcript25430/f4p0/1768	17.12666667	3.866666667	13.4	-2.0056	1.1687E-06	1.3566E-05	DOWN	1.6485	1.4588E-06	2.6343E-05	UP	-0.36484	0.36179	0.57886	
transcript_HQ_CoA_transcript25434/f3p0/1837	6.016666667	30.76666667	40.23666667	2.5569	5.4769E-05	0.00041738	UP	0.22187	0.45012	0.63757		2.7598	3.695E-06	9.3546E-05	UP
transcript_HQ_CoA_transcript25441/f4p0/1744	0	62.08666667	2.26	12.55	1.7551E-24	1.4855E-22	UP	-4.9367	1.463E-10	6.8935E-09	DOWN	7.6007	1.8503E-05	0.00035305	UP
transcript_HQ_CoA_transcript25444/f2p0/1805	47.08	0	0	-12.002	9.7505E-06	9.1555E-05	DOWN	NA	NA	NA	NA	-12.165	7.4479E-06	0.00016598	DOWN
transcript_HQ_CoA_transcript25456/f4p0/1796	10.07666667	48.44333333	21.71	2.4124	1.0231E-23	8.1633E-22	UP	-1.3021	1.8382E-09	6.7655E-08	DOWN	1.0987	0.00005195	0.00082561	UP
transcript_HQ_CoA_transcript25469/f2p0/1802	5.353333333	32.42333333	63.71666667	2.7327	2.0036E-05	0.00017197	UP	0.86093	0.16942	0.33166		3.5822	9.15E-18	3.0536E-15	UP
transcript_HQ_CoA_transcript25470/f7p0/1789	16.05333333	43.29	26.06666667	1.5755	3.9322E-07	4.9632E-06	UP	-0.86129	0.0023217	0.01253		0.70258	0.028732	0.10828	
transcript_HQ_CoA_transcript25477/f2p0/1803	24.44666667	59.1	40.33666667	1.4401	2.6753E-06	2.8569E-05	UP	-0.69905	0.00033077	0.0025799		0.72704	0.016212	0.071037	
transcript_HQ_CoA_transcript25491/f3p0/1799	0	378.9666667	159.9233333	15.215	2.172E-35	3.711E-33	UP	-1.3722	0.53962	0.70992		27.586	1.6538E-12	2.3351E-10	UP
transcript_HQ_CoA_transcript25500/f2p0/1830	3.463333333	1.36	4.663333333	-1.1863	0.0090993	0.032586	DOWN	1.6215	1.3692E-05	0.00017711	UP	0.42198	0.19918	0.39973	
transcript_HQ_CoA_transcript25503/f3p0/1785	9.376666667	67.47333333	9.626666667	3.0108	7.9195E-23	5.9095E-21	UP	-2.9685	1.2427E-19	2.7442E-17	DOWN	0.028677	0.9195	0.96458	
transcript_HQ_CoA_transcript25508/f3p0/1841	16.21	80.62333333	35.71	2.4855	1.1535E-11	3.2445E-10	UP	-1.3357	7.3671E-05	0.00074521	DOWN	1.1374	3.4878E-06	8.9355E-05	UP
transcript_HQ_CoA_transcript25511/f3p0/1769	5.266666667	18.94666667	20.20666667	2.0092	6.5419E-09	1.1702E-07	UP	-0.044902	0.91814	0.95666		1.9492	7.8672E-05	0.0011655	UP
transcript_HQ_CoA_transcript25513/f3p0/1793	47.03	97.67	79.33333333	1.2188	3.3915E-05	0.00027447	UP	-0.42634	0.29255	0.48216		0.77737	0.064605	0.19049	
transcript_HQ_CoA_transcript25516/f2p0/1796	22.42333333	92.28666667	47.25333333	2.1867	4.0329E-08	6.243E-07	UP	-1.0928	0.0007513	0.0050683	DOWN	1.0824	0.0037645	0.024722	UP
transcript_HQ_CoA_transcript25519/f2p0/1803	13.73666667	1.26	2.623333333	-3.3081	2.3372E-09	4.5312E-08	DOWN	0.91335	0.14408	0.29619		-2.4068	4.7353E-06	0.00011404	DOWN
transcript_HQ_CoA_transcript25520/f2p0/1813	9.31	49.97333333	14.16333333	2.5947	1.8789E-12	5.8023E-11	UP	-1.9768	5.519E-07	1.1495E-05	DOWN	0.60384	0.046314	0.15199	
transcript_HQ_CoA_transcript25521/f2p0/1791	65.40333333	8.68	42.91333333	-2.7547	1.8325E-09	3.614E-08	DOWN	2.1498	1.0782E-12	8.2E-11	UP	-0.61628	0.098649	0.25463	
transcript_HQ_CoA_transcript25526/f5p0/1782	85.52	25.5	18.44	-1.6024	2.576E-14	9.9355E-13	DOWN	-0.60494	0.022857	0.076049		-2.2203	6.0174E-26	7.3633E-23	DOWN
transcript_HQ_CoA_transcript25529/f2p0/1796	7.466666667	1.96	4.053333333	-1.7521	0.0026108	0.011574	DOWN	0.88975	0.068418	0.17304		-0.87772	0.11904	0.28726	
transcript_HQ_CoA_transcript25531/f2p0/1791	133.5333333	60.09	62.53666667	-1.0186	0.015476	0.049665	DOWN	-0.076949	0.75246	0.86016		-1.1037	0.0090073	0.046729	DOWN
transcript_HQ_CoA_transcript25532/f2p0/1800	0	45.48666667	3.016666667	12.196	1.3728E-10	3.2764E-09	UP	-4.0929	0.0093782	0.038078	DOWN	8.0864	3.2556E-05	0.00057074	UP
transcript_HQ_CoA_transcript25537/f3p0/1802	8.49	36.83666667	13.21	2.2761	4.803E-11	1.2264E-09	UP	-1.6255	1.6545E-05	0.00020771	DOWN	0.63656	0.037728	0.13133	
transcript_HQ_CoA_transcript25545/f9p0/1770	21.37	9.58	12.13666667	-1.0099	0.00015229	0.0010154	DOWN	0.19327	0.43579	0.62471		-0.82905	0.0024431	0.017725	
transcript_HQ_CoA_transcript25553/f2p0/1809	16.65	32.50333333	16.47	1.1151	0.0033775	0.014377	UP	-1.1197	1.6397E-05	0.00020613	DOWN	-0.016716	0.96293	0.98367	
transcript_HQ_CoA_transcript25575/f3p0/1777	1.51	72.47	5.116666667	5.75	1.0301E-20	6.8234E-19	UP	-3.9665	4.8492E-10	2.0574E-08	DOWN	1.771	0.041776	0.14134	
transcript_HQ_CoA_transcript25578/f2p0/1792	3.423333333	113.35	70.25333333	5.2441	9.4331E-05	0.00066973	UP	-0.832	0.57651	0.73968		4.394	0.017954	0.076852	
transcript_HQ_CoA_transcript25579/f5p0/1796	27.28333333	99.90666667	44.06666667	2.039	3.2683E-26	3.1448E-24	UP	-1.3252	0.0008969	0.0058617	DOWN	0.69903	0.071791	0.20401	
transcript_HQ_CoA_transcript25582/f10p0/1763	703.0566667	281.7	468.9433333	-1.1788	1.5584E-05	0.00013764	DOWN	0.59731	0.0069235	0.030114		-0.59074	0.035242	0.12519	
transcript_HQ_CoA_transcript25587/f2p0/1825	0.343333333	38.63666667	78.32	7.0127	0.00037095	0.0021912	UP	0.885	0.51781	0.69166		7.8716	0.0022064	0.016485	UP
transcript_HQ_CoA_transcript25594/f2p0/1793	35.32333333	111.7666667	77.34666667	1.8495	8.0913E-06	7.8102E-05	UP	-0.70529	0.072678	0.18092		1.1303	5.6422E-05	0.00088591	UP
transcript_HQ_CoA_transcript25598/f3p0/1743	13.34	38.84333333	5.683333333	1.7118	1.798E-11	4.9061E-10	UP	-2.9141	5.0736E-10	2.1329E-08	DOWN	-1.2168	0.014403	0.065274	
transcript_HQ_CoA_transcript25599/f2p0/1786	4.04	20.27	7.553333333	2.4951	4.1099E-10	9.0746E-09	UP	-1.567	0.00054334	0.0038994	DOWN	0.91328	0.042842	0.14365	
transcript_HQ_CoA_transcript25605/f2p0/1795	50.86666667	94.55	50.91666667	1.046	0.0011628	0.0058354	UP	-1.044	9.0211E-06	0.00012413	DOWN	-0.0086607	0.97522	0.9897	
transcript_HQ_CoA_transcript25621/f5p0/1778	7.48	33.36	11.26666667	2.3303	2.1385E-05	0.00018261	UP	-1.7147	0.0028653	0.014857	DOWN	0.60028	0.11994	0.28876	
transcript_HQ_CoA_transcript25625/f2p0/1792	11.72	2.646666667	4.226666667	-1.9914	1.2062E-08	2.0532E-07	DOWN	0.54663	0.14231	0.29363		-1.4573	7.6866E-05	0.0011433	DOWN
transcript_HQ_CoA_transcript25632/f2p0/1783	21.01	90.15	31.38333333	2.2608	1.0012E-06	1.1771E-05	UP	-1.6725	0.00036584	0.0028127	DOWN	0.57528	0.030105	0.11215	
transcript_HQ_CoA_transcript25640/f5p0/1770	531.41	165.9966667	395.63	-1.5299	8.6163E-05	0.00061919	DOWN	1.1035	4.3364E-05	0.00047823	UP	-0.43569	0.20051	0.40165	
transcript_HQ_CoA_transcript25650/f2p0/1788	12.5	2.95	5.55	-1.9787	0.0043875	0.017781	DOWN	0.77694	0.23248	0.41297		-1.2102	0.040731	0.13891	
transcript_HQ_CoA_transcript25652/f2p0/1789	48.62666667	14.41	35.07333333	-1.614	7.5795E-07	9.0865E-06	DOWN	1.1459	0.00012064	0.0011208	UP	-0.47999	0.024847	0.096851	
transcript_HQ_CoA_transcript25662/f2p0/1780	14.90333333	2.9	3.073333333	-2.2034	7.1667E-10	1.5253E-08	DOWN	-0.063663	0.87424	0.93169		-2.2799	8.9937E-10	6.4484E-08	DOWN
transcript_HQ_CoA_transcript25664/f2p0/1784	27.65	87.22666667	40.2	1.8465	2.0493E-06	2.2542E-05	UP	-1.2684	0.00046766	0.0034591	DOWN	0.56227	0.15712	0.34353	

transcript_HQ_CoA_transcript25669/f2p0/1787	18.95	36.69	21.40333333	1.1047	0.0038978	0.016218	UP	-0.93829	8.8347E-05	0.00086967		0.15486	0.69198	0.83633	
transcript_HQ_CoA_transcript2567/f9p0/4573	16.59333333	0.086666667	2.773333333	-7.366	1.589E-16	7.6245E-15	DOWN	4.7706	4.1526E-10	1.7942E-08	UP	-2.6043	3.0409E-06	8.0426E-05	DOWN
transcript_HQ_CoA_transcript25685/f2p0/1788	22.23333333	43.10333333	24.92666667	1.1254	1.9036E-05	0.00016408	UP	-0.94156	0.000228	0.0019021		0.17076	0.48898	0.69119	
transcript_HQ_CoA_transcript25693/f4p0/1769	8.1	3.133333333	7.296666667	-1.21	0.001818	0.0085641	DOWN	1.0619	0.0050683	0.023716	UP	-0.15961	0.57932	0.75828	
transcript_HQ_CoA_transcript25705/f3p0/1795	10.65	19.93666667	19.63333333	1.0548	0.00000282	2.9904E-05	UP	-0.15623	0.67707	0.81215		0.88522	0.01846	0.078543	
transcript_HQ_CoA_transcript25706/f2p0/1769	104.4966667	9.003333333	63.92666667	-3.3884	9.0476E-20	5.5136E-18	DOWN	2.6872	3.7852E-09	1.3031E-07	UP	-0.70967	0.14157	0.32113	
transcript_HQ_CoA_transcript25711/f2p0/1789	12.60333333	5.496666667	4.63	-1.0283	0.0032776	0.014	DOWN	-0.41283	0.23917	0.4211		-1.4554	2.1932E-06	6.1273E-05	DOWN
transcript_HQ_CoA_transcript25722/f2p0/1784	4.92	2	5.473333333	-1.1346	0.0051416	0.020328	DOWN	1.2924	0.00015631	0.0013948	UP	0.14618	0.63284	0.79556	
transcript_HQ_CoA_transcript25724/f2p0/1780	2.066666667	22.38	4.06	3.5937	6.4105E-19	3.6854E-17	UP	-2.6099	7.4203E-23	2.8335E-20	DOWN	0.97258	0.042213	0.14246	
transcript_HQ_CoA_transcript25733/f3p0/1794	0.223333333	1.136666667	2.35	2.4644	0.01052	0.036702	UP	0.92974	0.16337	0.32384		3.3851	6.3851E-06	0.0001465	UP
transcript_HQ_CoA_transcript25734/f2p0/1787	5.986666667	17.01333333	4.69	1.6737	0.00084338	0.0044345	UP	-1.9997	5.7459E-05	0.00060596	DOWN	-0.33942	0.28317	0.49737	
transcript_HQ_CoA_transcript25742/f3p0/1780	67.16333333	21.78333333	54.00333333	-1.4795	4.5634E-05	0.00035516	DOWN	1.1617	0.00054316	0.0038994	UP	-0.32956	0.39405	0.60964	
transcript_HQ_CoA_transcript25743/f2p0/1783	94.42666667	32.35	40.80666667	-1.3838	1.0821E-06	1.2625E-05	DOWN	0.17526	0.39722	0.58781		-1.221	6.8165E-07	2.2666E-05	DOWN
transcript_HQ_CoA_transcript25756/f2p0/1703	28.28666667	10.97666667	20.88333333	-1.2193	1.3126E-07	1.8347E-06	DOWN	0.78454	0.0011172	0.0069865		-0.44555	0.03894	0.13476	
transcript_HQ_CoA_transcript25759/f8p0/1701	1.01	101.89	5.61	6.8476	8.0629E-60	3.071E-57	UP	-4.3397	1.8687E-15	2.2534E-13	DOWN	2.4907	0.0011588	0.010043	UP
transcript_HQ_CoA_transcript2577/f2p0/4633	9.63	1.91	7.02	-2.1939	4.7967E-06	4.8503E-05	DOWN	1.7272	7.004E-08	1.7923E-06	UP	-0.47455	0.30759	0.52422	
transcript_HQ_CoA_transcript25789/f2p0/1786	13.88666667	27.28	41.98333333	1.1515	0.0045395	0.018293	UP	0.48839	0.22445	0.40314		1.6247	0.00027834	0.0032625	UP
transcript_HQ_CoA_transcript25794/f5p0/1755	0.146666667	495.43	55.05	11.885	5.886E-89	7.6863E-86	UP	-3.2926	0.019211	0.066546		8.5832	2.9777E-06	7.9097E-05	UP
transcript_HQ_CoA_transcript25798/f4p0/1726	14.39	109.6933333	14.71	3.0849	2.7197E-23	2.098E-21	UP	-3.0514	3.6909E-16	4.8322E-14	DOWN	0.020877	0.96179	0.98321	
transcript_HQ_CoA_transcript25804/f4p0/1772	0.473333333	144.9066667	4.773333333	8.4158	8.4712E-62	3.5198E-59	UP	-5.0449	9.2721E-10	3.647E-08	DOWN	3.3587	0.009119	0.047004	UP
transcript_HQ_CoA_transcript25817/f3p0/1780	59.78	169.32	75.96	1.6764	6.8002E-11	1.68E-09	UP	-1.3095	4.2271E-07	9.0723E-06	DOWN	0.35371	0.11719	0.28484	
transcript_HQ_CoA_transcript25819/f2p0/1770	2.033333333	17.22	4.076666667	3.2716	2.4746E-09	4.7723E-08	UP	-2.2488	3.5637E-06	5.6456E-05	DOWN	1.0077	0.048274	0.15636	
transcript_HQ_CoA_transcript25826/f2p0/1792	114.2666667	45.57333333	90.65	-1.1604	5.3849E-09	9.7375E-08	DOWN	0.83393	8.7217E-06	0.00012029		-0.33894	0.059095	0.17961	
transcript_HQ_CoA_transcript25844/f2p0/1762	12.22	25.42333333	15.61333333	1.2118	0.010802	0.037502	UP	-0.83727	0.015434	0.056274		0.35983	0.37531	0.59207	
transcript_HQ_CoA_transcript25845/f3p0/1778	40.67	9.97	29.2	-1.8407	0.0011342	0.0057199	DOWN	1.402	0.00061068	0.0042771	UP	-0.45866	0.44005	0.65094	
transcript_HQ_CoA_transcript25851/f2p0/1765	2.763333333	6.81	3.623333333	1.4712	0.0065041	0.024675	UP	-1.0587	0.04596	0.12788		0.39652	0.42724	0.63954	
transcript_HQ_CoA_transcript25855/f2p0/1773	6.07	36.5	9.286666667	2.7495	2.8903E-11	7.6579E-10	UP	-2.1251	4.1049E-06	6.3493E-05	DOWN	0.6105	0.12306	0.29385	
transcript_HQ_CoA_transcript25866/f2p0/1793	17.47333333	5.573333333	12.67333333	-1.5092	8.4673E-05	0.00060969	DOWN	1.0321	0.0091705	0.037422	UP	-0.48752	0.25648	0.46684	
transcript_HQ_CoA_transcript25871/f10p0/1693	31.36333333	71.28666667	68.5	1.364	0.00029809	0.0018087	UP	-0.23009	0.6707	0.80764		1.1167	0.016031	0.070565	
transcript_HQ_CoA_transcript2588/f2p0/4627	5.356666667	27.57333333	15.80333333	2.5317	2.1157E-23	1.653E-21	UP	-0.96075	7.9221E-08	1.9864E-06		1.5572	5.6402E-09	3.421E-07	UP
transcript_HQ_CoA_transcript25885/f6p0/1742	127.0266667	368.4266667	231.59	1.7061	1.4673E-09	2.9446E-08	UP	-0.82154	0.0030405	0.015637		0.86821	0.00997	0.050178	
transcript_HQ_CoA_transcript25886/f3p0/1774	2.993333333	110.2833333	9.703333333	5.3618	3.8296E-77	3.3339E-74	UP	-3.6422	1.925E-09	7.0284E-08	DOWN	1.7056	0.015012	0.067336	
transcript_HQ_CoA_transcript25887/f2p0/1779	23.41666667	8.203333333	18.15	-1.3559	0.0063697	0.024215	DOWN	0.98226	0.00070602	0.0048286		-0.3833	0.36368	0.58062	
transcript_HQ_CoA_transcript25905/f6p0/1720	121.5066667	34.56333333	96.34666667	-1.6631	2.1211E-16	1.002E-14	DOWN	1.3213	3.7626E-05	0.00042531	UP	-0.35418	0.25883	0.4695	
transcript_HQ_CoA_transcript25906/f2p0/1771	1.366666667	86.72333333	5.603333333	6.1636	7.9297E-31	1.0983E-28	UP	-4.1016	1.0024E-08	3.1568E-07	DOWN	2.0468	0.0099427	0.050068	
transcript_HQ_CoA_transcript25910/f2p0/1769	6.476666667	0.696666667	17.61333333	-3.0972	1.3355E-07	1.8623E-06	DOWN	4.5443	3.8448E-18	7.1183E-16	UP	1.4371	8.4605E-05	0.0012384	UP
transcript_HQ_CoA_transcript25913/f2p0/1776	31.50333333	11.58333333	18.52333333	-1.3097	0.0025116	0.011191	DOWN	0.52948	0.071147	0.17825		-0.79053	0.043652	0.14581	
transcript_HQ_CoA_transcript25918/f2p0/1797	21.15333333	80.56333333	33.24666667	2.0982	5.3562E-11	1.3563E-09	UP	-1.4318	3.8641E-06	6.0534E-05	DOWN	0.65232	0.0029159	0.020311	
transcript_HQ_CoA_transcript25926/f2p0/1778	0.02	15.38	1.07	9.6205	1.5996E-13	4.959E-11	UP	-4.0178	0.000204	0.0017538	DOWN	5.5865	0.011305	0.05517	
transcript_HQ_CoA_transcript25931/f2p0/1755	47.24333333	7.7	12.25666667	-2.4461	2.5396E-06	2.7279E-05	DOWN	0.51727	0.24472	0.42749		-1.9418	0.00060486	0.0060077	DOWN
transcript_HQ_CoA_transcript25959/f2p0/1769	19.73666667	144.7366667	21.76666667	3.0368	1.9481E-27	2.1097E-25	UP	-2.8973	8.3192E-28	5.258E-25	DOWN	0.12664	0.63652	0.79792	
transcript_HQ_CoA_transcript25979/f2p0/1760	4.886666667	12.46666667	2.7	1.5216	0.0032539	0.013913	UP	-2.3575	5.2365E-06	7.7779E-05	DOWN	-0.84645	0.12108	0.29047	
transcript_HQ_CoA_transcript2599/f4p0/4604	1.136666667	3.013333333	2.37	1.5642	0.00001743	0.00015188	UP	-0.49352	0.064282	0.16567		1.0575	0.0022849	0.016911	UP
transcript_HQ_CoA_transcript25993/f2p0/1729	21.25	99.19666667	26.21333333	2.3874	1.66E-14	6.6118E-13	UP	-2.0795	1.1006E-09	4.265E-08	DOWN	0.29459	0.28974	0.50482	
transcript_HQ_CoA_transcript25994/f2p0/1761	23.61	9.19	10.07333333	-1.2137	0.0012323	0.0061464	DOWN	-0.015945	0.96494	0.981		-1.2392	0.0011396	0.0099177	DOWN
transcript_HQ_CoA_transcript26018/f8p0/1735	7.52	47.06333333	35.22333333	2.8337	7.6456E-06	7.4172E-05	UP	-0.5303	0.50888	0.68471		2.2839	0.063494	0.18797	
transcript_HQ_CoA_transcript26028/f3p0/1739	65.17	28.15333333	45.78666667	-1.0505	3.3998E-05	0.00027502	DOWN	0.55264	0.00003482	0.00039789		-0.51159	0.03605	0.12727	
transcript_HQ_CoA_transcript26031/f7p0/1724	34.41666667	75.98333333	52.19	1.3051	7.4406E-10	1.5781E-08	UP	-0.6831	0.033639	0.10148		0.60873	0.043155	0.14452	
transcript_HQ_CoA_transcript26035/f2p0/1819	0.13	22.89	5.193333333	7.7686	4.9958E-17	2.523E-15	UP	-2.2947	0.012113	0.046681	DOWN	5.46	5.5641E-06	0.00013127	UP
transcript_HQ_CoA_transcript26036/f3p0/1751	13.81333333	39.68333333	21.05666667	1.6778	1.9295E-07	2.6034E-06	UP	-1.0658	0.0010292	0.0065433	DOWN	0.60105	0.014779	0.066479	
transcript_HQ_CoA_transcript26043/f2p0/1776	17.49333333	35.07666667	25.64	1.1704	0.0007678	0.0040805	UP	-0.61252	0.078614	0.19187		0.54527	0.061212	0.18392	
transcript_HQ_CoA_transcript26053/f4p0/1741	0.283333333	1.76	37.00333333	2.8109	0.0041657	0.017091	UP	4.3131	0.0051143	0.023871	UP	7.1062	1.0624E-05	0.00022171	UP
transcript_HQ_CoA_transcript26057/f16p0/1733	103.14	223.04	113.5133333	1.2806	1.2437E-06	1.4355E-05	UP	-1.1154	0.00014775	0.0013315	DOWN	0.15198	0.58404	0.76204	
transcript_HQ_CoA_transcript26061/f2p0/1715	0.086666667	2.32	1.016666667	4.8664	0.00014196	0.0009559	UP	-1.2848	0.34936	0.54151		3.5709	0.056165	0.17346	
transcript_HQ_CoA_transcript26067/f3p0/1754	31.06	59.87333333	28.23	1.1136	0.0021269	0.0097625	UP	-1.2455	0.00050388	0.0036795	DOWN	-0.14532	0.42628	0.63831	
transcript_HQ_CoA_transcript26074/f3p0/1745	0.146666667	123.7966667	6.206666667	9.8832	4.777E-54	1.4802E-51	UP	-4.4769	0.0016925	0.0097247	DOWN	5.3876	4.3089E-05	0.00070741	UP
transcript_HQ_CoA_transcript26076/f3p0/1755	4.72	11.45333333	5.736666667	1.423	0.015094	0.048801	UP	-1.1316	0.043442	0.12259		0.27709	0.474	0.67945	
transcript_HQ_CoA_transcript26086/f2p0/1765	0.123333333	8.676666667	1.796666667	6.2657	4.0376E-11	1.0411E-09	UP	-2.4025	0.00010419	0.00099667	DOWN	3.8522	0.00019843	0.0024777	UP
transcript_HQ_CoA_transcript26096/f4p0/1747	0.166666667	149.8666667	3.92	9.9537	4.6663E-67	2.4374E-64	UP	-5.3945	3.054E-06	4.9625E-05	DOWN	4.5415	0.00022218	0.0027133	UP
transcript_HQ_CoA_transcript26098/f2p0/1759	35.67	15.01333333	40.19666667	-1.0732	0.0050127	0.019898	DOWN	1.2465	0.00053954	0.0038781	UP	0.1614	0.37547	0.59208	
transcript_HQ_CoA_transcript26102/f8p0/1752	0.183333333	555.6233333	74.53	11.674	2.771E-63	1.2062E-60	UP	-3.0089	0.16366	0.32419		8.6459	0.0018704	0.01448	UP
transcript_HQ_CoA_transcript26105/f3p0/1769	13.19666667	26.67	14.21666667	1.1873	0.0001505	0.0010045	UP	-1.0648	0.00053122	0.0038288	DOWN	0.11004	0.68969	0.83497	
transcript_HQ_CoA_transcript26116/f3p0/1753	5.28	39.85	18.84666667	3.0805	4.7248E-13	1.5677E-11	UP	-1.236	0.003024	0.015565	DOWN	1.8329	3.0162E-11	3.0252E-09	UP
transcript_HQ_CoA_transcript26119/f2p0/1733	7.173333333	42.66	9.536666667	2.7211	1.482E-07	2.0433E-06	UP	-2.2985	0.00024421	0.0020126	DOWN	0.41014	0.48498	0.68822	
transcript_HQ_CoA_transcript26127/f3p0/1787	10.22666667	22.95333333	20.26	1.3228	9.275E-10	1.9423E-08	UP	-0.3278	0.085726	0.20393		0.9817	6.5847E-05	0.0010106	
transcript_HQ_CoA_transcript26130/f3p0/1760	12.11333333	54.66333333	24.98	2.3554	4.5137E-08	6.9344E-07	UP	-1.2824	2.1465E-11	1.1886E-09	DOWN	1.0571	0.013829	0.063361	
transcript_HQ_CoA_transcript26139/f3p0/1748	28.88666667	65.53333333	34.86	1.3532	3.4068E-05	0.00027546	UP	-1.0704	0.00040929	0.0030922	DOWN	0.26924	0.15438	0.33891	
transcript_HQ_CoA_transcript26148/f2p0/1769	12.47666667	1.79	11.03	-2.6911	7.4179E-05	0.00054311	DOWN	2.4996	0.00055392	0.0039567	UP	-0.19573	0.77649	0.89056	
transcript_HQ_CoA_transcript26157/f2p0/1733	5.876666667	28.74333333	24.78333333	2.4256	3.7266E-06	3.8601E-05	UP	-0.32188	0.62846	0.77736		2.0924	0.0030127	0.020828	UP
transcript_HQ_CoA_transcript26160/f2p0/1731	2.206666667	0.406666667	1.52	-2.2493	0.0047528	0.019031	DOWN	1.6786	0.11206	0.24772		-0.58309	0.52275	0.71735	
transcript_HQ_CoA_transcript26166/f4p0/1737	18.67333333	5.443333333	59.01	-1.6154	0.00039069	0.0022908	DOWN	3.2714	2.6317E-19	5.6398E-17	UP	1.6463	4.3006E-07	1.5328E-05	UP
transcript_HQ_CoA_transcript26167/f3p0/1756	0.15	27.39333333	1.96	7.7219	4.6964E-29	5.9214E-27	UP	-3.9667	1.386E-11	7.9638E-10	DOWN	3.7416	0.00077584	0.0073103	UP
transcript_HQ_CoA_transcript26172/f2p0/1765	4.683333333	14.74333333	2.46	1.8431	5.6439E-05	0.0004285	UP	-2.7604	7.3004E-12	4.4308E-10	DOWN	-0.93251	0.032173	0.11717	
transcript_HQ_CoA_transcript26174/f2p0/1741	2.09	4.416666667	2.696666667	1.231	0.011141	0.038475	UP	-0.8555	0.06585	0.16819		0.36362	0.43111	0.64307	
transcript_HQ_CoA_transcript26175/f2p0/1741	10.49	38.82	38.99666667	2.0223	0.0014485	0.0070469	UP	-0.088601	0.8847	0.93803		1.9237	0.0023231	0.017086	UP
transcript_HQ_CoA_transcript26190/f3p0/1742	40.22666667	219.15	61.05666667	2.5947	5.1214E-30	6.9355E-28	UP	-1.9913	8.2833E-39	1.2652E-35	DOWN	0.59228	0.0096987	0.049204	
transcript_HQ_CoA_transcript26192/f2p0/1752	27.50666667	51.13666667	38.52333333	1.0314	0.004991	0.019831	UP	-0.5425	0.088044	0.20809		0.47684	0.10422	0.26427	
transcript_HQ_CoA_transcript26196/f3p0/1727	3.583333333	7.46	25.77666667	1.2056	0.00029665	0.0018017	UP	1.6865	0.033698	0.10161		2.8793	0.00016085	0.0020924	UP
transcript_HQ_CoA_transcript26207/f2p0/1745	16.17666667	3.196666667	5.193333333	-2.2127	4.9594E-08	7.5683E-07	DOWN	0.5814	0.18488	0.35282		-1.6432	6.058E-11	5.6732E-09	DOWN

transcript_HQ_CoA_transcript26223/f2p0/1742	60.21	23.89666667	48.25	-1.1783	1.2377E-11	3.4599E-10	DOWN	0.85582	0.00022782	0.0019021		-0.33465	0.13127	0.30551	
transcript_HQ_CoA_transcript26231/f2p0/1734	4.923333333	26.81333333	6.5	2.6098	4.767E-11	1.2189E-09	UP	-2.1833	1.1053E-06	2.0673E-05	DOWN	0.41185	0.35409	0.57152	
transcript_HQ_CoA_transcript26240/f3p0/1747	10.10666667	29.15	29.43666667	1.7045	1.7663E-05	0.00015369	UP	-0.15367	0.62826	0.77722		1.5389	2.0735E-07	8.2025E-06	UP
transcript_HQ_CoA_transcript26246/f2p0/1753	94.73666667	20.55666667	39.46	-2.067	5.2824E-08	8.001E-07	DOWN	0.80084	0.00051371	0.0037281		-1.2743	0.00066371	0.0064731	DOWN
transcript_HQ_CoA_transcript26250/f2p0/1743	8.626666667	3.49	4.373333333	-1.1533	0.00023358	0.001469	DOWN	0.17952	0.60181	0.759		-0.986	0.0014919	0.012182	
transcript_HQ_CoA_transcript2627/f2p0/4624	16.59	7.4	13.49	-1.0158	2.7481E-05	0.00022754	DOWN	0.71696	6.8648E-08	1.7623E-06		-0.30976	0.17051	0.36148	
transcript_HQ_CoA_transcript26283/f8p0/1652	95.12666667	187.68	194.5433333	1.1458	0.00007249	0.00053212	UP	-0.10958	0.80946	0.89518		1.0192	0.018585	0.078873	
transcript_HQ_CoA_transcript26286/f3p0/1749	570.9633333	1087.16	747.73	1.0928	0.0089339	0.032113	UP	-0.66949	0.014603	0.053973		0.40768	0.26878	0.48112	
transcript_HQ_CoA_transcript26299/f2p0/1753	6.126666667	1.156666667	5.836666667	-2.2359	0.00020126	0.0012933	DOWN	2.1742	0.00097899	0.0062741	UP	-0.071328	0.89437	0.9531	
transcript_HQ_CoA_transcript26302/f2p0/1740	11.20666667	26.76	11.11333333	1.4054	1.5978E-07	2.1832E-06	UP	-1.4066	2.1764E-06	3.7074E-05	DOWN	-0.013848	0.96779	0.98625	
transcript_HQ_CoA_transcript26304/f2p0/1743	11.32666667	31.63333333	12.70666667	1.6441	0.00015284	0.0010187	UP	-1.469	0.0004973	0.0036387	DOWN	0.16159	0.5943	0.76901	
transcript_HQ_CoA_transcript2631/f2p0/4608	3.126666667	9.046666667	4.546666667	1.7008	0.00083075	0.0043769	UP	-1.1211	0.0027829	0.014495	DOWN	0.56413	0.30982	0.52653	
transcript_HQ_CoA_transcript26318/f3p0/1763	32.67666667	74.44	57.01666667	1.3455	1.0556E-05	9.8163E-05	UP	-0.53082	0.10203	0.23205		0.80412	0.00068386	0.0066379	
transcript_HQ_CoA_transcript26325/f7p0/1712	86.07	25.85666667	60.48333333	-1.5735	1.0538E-08	1.8124E-07	DOWN	1.0645	0.00094211	0.0060845	UP	-0.52151	0.080524	0.22063	
transcript_HQ_CoA_transcript26333/f2p0/1746	17.41333333	60.52	21.19666667	1.9754	4.6145E-07	5.7506E-06	UP	-1.6829	3.5113E-05	0.00040025	DOWN	0.27787	0.27678	0.49048	
transcript_HQ_CoA_transcript26345/f4p0/1711	30.87333333	94.93666667	50.50666667	1.792	1.1157E-07	1.5726E-06	UP	-1.0725	0.00095545	0.0061577	DOWN	0.70654	0.00015281	0.0020092	
transcript_HQ_CoA_transcript26366/f2p0/1786	207.1833333	90.78	135.9733333	-1.0345	2.4331E-12	7.4014E-11	DOWN	0.43287	0.00001255	0.0001649		-0.61411	2.0813E-05	0.00038783	
transcript_HQ_CoA_transcript26370/f2p0/1731	15.02666667	75.20666667	17.13666667	2.4946	8.2946E-13	2.6839E-11	UP	-2.2911	6.6142E-12	4.0697E-10	DOWN	0.18958	0.48133	0.68545	
transcript_HQ_CoA_transcript26371/f3p0/1735	2.836666667	8.436666667	10.2	1.7638	0.00081341	0.0042974	UP	0.12614	0.83613	0.91049		1.8735	0.002244	0.016716	UP
transcript_HQ_CoA_transcript26374/f11p0/1700	24.53333333	292.5266667	73.23333333	3.7252	3.1971E-47	8.6138E-45	UP	-2.1198	8.3674E-05	0.00083216	DOWN	1.5924	0.0017354	0.013718	UP
transcript_HQ_CoA_transcript26381/f2p0/1733	9.166666667	2.936666667	6.256666667	-1.5079	0.00021955	0.0013937	DOWN	0.95404	0.065902	0.16828		-0.56444	0.15967	0.34725	
transcript_HQ_CoA_transcript26394/f4p0/1719	10.55666667	19.58666667	14.63	1.0376	0.00054256	0.0030258	UP	-0.556	0.033169	0.10041		0.46925	0.039247	0.13546	
transcript_HQ_CoA_transcript26402/f2p0/1685	4.28	22.11666667	8.9	2.5666	6.8634E-06	6.7316E-05	UP	-1.4923	0.00096468	0.0062031	DOWN	1.0583	0.019646	0.082104	
transcript_HQ_CoA_transcript26407/f27p0/1697	190.7866667	72.22666667	95.13666667	-1.2671	0.00024562	0.0015324	DOWN	0.26219	0.5198	0.69316		-1.0128	0.0047849	0.029572	DOWN
transcript_HQ_CoA_transcript26408/f2p0/1730	2.853333333	12.89	13.73333333	2.3134	0.0041878	0.017151	UP	-0.026842	0.96942	0.98411		2.2745	2.7683E-06	7.4724E-05	UP
transcript_HQ_CoA_transcript26409/f2p0/1726	4.363333333	12.57	9.003333333	1.6971	8.3919E-05	0.0006045	UP	-0.64944	0.059788	0.15666		1.0343	0.0025561	0.018349	UP
transcript_HQ_CoA_transcript26410/f2p0/1754	10.3	24.48	15.30333333	1.4163	0.00014623	0.0009807	UP	-0.83345	0.00071402	0.004873		0.56764	0.14427	0.32489	
transcript_HQ_CoA_transcript26418/f2p0/1724	0	43.64666667	2.313333333	12.074	3.4294E-07	4.3936E-06	UP	-4.4193	NA	NA	NA	7.6393	0.050666	0.16148	
transcript_HQ_CoA_transcript26430/f2p0/1736	3.16	9.52	5.01	1.7438	3.2425E-06	0.00003401	UP	-1.0811	0.00059431	0.0041961	DOWN	0.64981	0.15944	0.34687	
transcript_HQ_CoA_transcript2646/f4p0/4603	9.14	17.34	1.77	1.069	0.00053141	0.0029738	UP	-3.4154	2.1077E-09	7.6197E-08	DOWN	-2.3603	3.0346E-05	0.0005362	DOWN
transcript_HQ_CoA_transcript26464/f4p0/1727	60.17	0.04	0.083333333	-10.332	0.00007822	0.00056837	DOWN	0.91134	0.8159	0.89969		-9.4356	0.00045433	0.0047982	DOWN
transcript_HQ_CoA_transcript26474/f2p0/1728	184.9433333	64.76666667	139.62	-1.3691	4.2362E-07	5.3228E-06	DOWN	0.97179	0.00011314	0.0010657		-0.40776	0.05583	0.17298	
transcript_HQ_CoA_transcript26475/f2p0/1716	4.173333333	10.04666667	8.713333333	1.4168	9.8436E-06	9.2199E-05	UP	-0.3525	0.19555	0.36697		1.051	0.00085782	0.0079241	UP
transcript_HQ_CoA_transcript26483/f2p0/1714	12.17	32.14333333	16.36	1.5715	1.2474E-05	0.00011357	UP	-1.1349	0.0010994	0.0068936	DOWN	0.42324	0.040714	0.13889	
transcript_HQ_CoA_transcript26485/f5p0/1700	38.20333333	10.22333333	20.17333333	-1.7421	3.9145E-14	1.4756E-12	DOWN	0.83089	0.00013277	0.0012161		-0.92356	3.344E-06	8.6327E-05	
transcript_HQ_CoA_transcript26489/f2p0/1720	9.156666667	1.416666667	13.45666667	-2.5165	0.00002228	0.00018901	DOWN	3.0903	6.7268E-21	1.8132E-18	UP	0.55822	0.24887	0.45896	
transcript_HQ_CoA_transcript2649/f2p0/4684	1.503333333	4.99	1.506666667	1.8827	0.011041	0.038222	UP	-1.8659	0.01183	0.045832	DOWN	0.0037734	0.99101	0.99628	
transcript_HQ_CoA_transcript26503/f2p0/1739	16.52666667	93.98	25.92333333	2.676	2.9718E-16	1.3931E-14	UP	-2.0114	3.6121E-07	7.8352E-06	DOWN	0.65101	0.054168	0.16958	
transcript_HQ_CoA_transcript26506/f2p0/1714	5.476666667	13.28	16.06333333	1.4257	0.000062	0.00046454	UP	0.12962	0.75207	0.86005		1.5442	3.7941E-05	0.00063832	UP
transcript_HQ_CoA_transcript26517/f2p0/1729	10.22	2.39	9.2	-1.9544	0.0020049	0.0092847	DOWN	1.7859	1.8746E-05	0.00023076	UP	-0.179	0.76807	0.88472	
transcript_HQ_CoA_transcript26523/f2p0/1629	10.87333333	22.88	24.89666667	1.243	2.7958E-06	2.9717E-05	UP	-0.028713	0.90588	0.94936		1.2012	3.6826E-06	9.3362E-05	UP
transcript_HQ_CoA_transcript26527/f2p0/1716	0.64	0.02	0.19	-4.7944	0.011786	0.040219	DOWN	2.994	0.28518	0.47412		-1.7967	0.20499	0.40746	
transcript_HQ_CoA_transcript26529/f2p0/1724	141.1833333	45.74666667	128.9633333	-1.4886	5.352E-06	5.3673E-05	DOWN	1.3585	0.00027279	0.0022065	UP	-0.14023	0.57911	0.75818	
transcript_HQ_CoA_transcript26532/f4p0/1713	32.82666667	9.783333333	25.05666667	-1.6012	0.01225	0.041504	DOWN	1.2436	0.024319	0.079825		-0.37362	0.37176	0.5889	
transcript_HQ_CoA_transcript26548/f2p0/1716	26.76333333	61.74333333	27.18666667	1.3542	4.0883E-10	9.0449E-09	UP	-1.3161	1.4315E-05	0.00018374	DOWN	0.026292	0.93164	0.97012	
transcript_HQ_CoA_transcript2655/f2p0/4592	9.64	3.473333333	4.036666667	-1.3141	1.2996E-08	2.1959E-07	DOWN	0.069234	0.82657	0.90552		-1.2595	2.6253E-06	7.1388E-05	DOWN
transcript_HQ_CoA_transcript26551/f2p0/1729	10.50666667	23.94333333	23.26333333	1.3473	1.2041E-07	1.692E-06	UP	-0.19236	0.48312	0.6651		1.1436	1.5329E-05	0.00030189	UP
transcript_HQ_CoA_transcript26560/f2p0/1713	22.09666667	1.023333333	8.293333333	-4.2625	3.365E-06	3.5154E-05	DOWN	2.8838	0.0011178	0.0069881	UP	-1.3976	0.1454	0.32666	
transcript_HQ_CoA_transcript2658/f3p0/4601	2.35	4.966666667	4.403333333	1.226	0.0014566	0.0070824	UP	-0.31109	0.35332	0.5453		0.90227	0.00077523	0.0073084	
transcript_HQ_CoA_transcript26588/f3p0/1715	66.32333333	18.13666667	34.94333333	-1.733	2.7894E-06	2.9666E-05	DOWN	0.82239	0.032661	0.099238		-0.91969	0.02376	0.093789	
transcript_HQ_CoA_transcript26592/f9p0/1617	63.89	205.7366667	79.39	1.83	3.6446E-05	0.00029211	UP	-1.4752	0.05999	0.15702		0.34382	0.72334	0.85774	
transcript_HQ_CoA_transcript26606/f2p0/1706	5.386666667	1.056666667	8.276666667	-2.1806	3.1484E-05	0.00025776	DOWN	2.8184	2.3693E-11	1.2955E-09	UP	0.62569	0.16631	0.35608	
transcript_HQ_CoA_transcript26617/f3p0/1717	143.0566667	526.0966667	178.4433333	2.0423	5.4981E-11	1.3883E-09	UP	-1.7106	9.1411E-13	7.1297E-11	DOWN	0.31821	0.3319	0.54914	
transcript_HQ_CoA_transcript2662/f3p0/4496	3.16	1.11	2.456666667	-1.3468	0.0036493	0.015348	DOWN	1.0021	0.031227	0.096044		-0.35537	0.48062	0.68492	
transcript_HQ_CoA_transcript26620/f3p0/1739	3.193333333	7.483333333	1.596666667	1.3908	0.00010479	0.00073319	UP	-2.3794	1.2076E-10	5.8245E-09	DOWN	-1.0048	0.042536	0.1431	
transcript_HQ_CoA_transcript26632/f3p0/1692	60.76	25.63	60.31666667	-1.0857	4.3553E-05	0.000341	DOWN	1.0871	2.6159E-06	4.3387E-05	UP	-0.011087	0.96008	0.98285	
transcript_HQ_CoA_transcript26658/f2p0/1707	11.98	2.756666667	7.826666667	-1.9596	1.8653E-06	2.0718E-05	DOWN	1.34	0.002365	0.012731	UP	-0.63206	0.11695	0.28448	
transcript_HQ_CoA_transcript26662/f3p0/1736	6.3	0.906666667	8.976666667	-2.6642	0.0029169	0.012689	DOWN	3.1831	3.016E-06	4.9225E-05	UP	0.50671	0.44534	0.65588	
transcript_HQ_CoA_transcript26667/f5p0/1692	9.043333333	24.78666667	13.83333333	1.6092	2.5465E-06	2.7321E-05	UP	-0.99352	0.0036308	0.018133		0.6038	0.14861	0.33136	
transcript_HQ_CoA_transcript26675/f2p0/1709	27.85	52.05333333	52.25333333	1.0728	0.00026591	0.0016412	UP	-0.1403	0.68951	0.82071		0.91899	0.0069031	0.038524	
transcript_HQ_CoA_transcript26685/f2p0/1712	21.62333333	41.17666667	12.87666667	1.0945	0.010479	0.036594	UP	-1.8353	1.1191E-05	0.0001495	DOWN	-0.75284	0.00046522	0.0048879	
transcript_HQ_CoA_transcript2669/f2p0/4595	10.01333333	3.8	5.42	-1.2474	2.1449E-06	2.3481E-05	DOWN	0.37062	0.11999	0.25988		-0.88957	0.00001632	0.00031868	
transcript_HQ_CoA_transcript26690/f5p0/1697	57.57333333	19.63666667	50.97333333	-1.3964	1.1722E-05	0.00010758	DOWN	1.2207	9.6955E-07	0.00001855	UP	-0.18741	0.53437	0.72692	
transcript_HQ_CoA_transcript26691/f3p0/1691	0	17.71333333	2.706666667	10.702	8.1447E-15	3.3536E-13	UP	-2.8613	0.093847	0.21796		7.8255	0.045277	0.14969	
transcript_HQ_CoA_transcript26692/f2p0/1704	13.79333333	28.15	19.29666667	1.1995	0.00016471	0.0010851	UP	-0.70713	0.018236	0.063934		0.4795	0.01236	0.058621	
transcript_HQ_CoA_transcript26693/f2p0/1720	11.7	1.9	9.36	-2.4466	9.9416E-07	1.1696E-05	DOWN	2.1189	5.3752E-06	7.9646E-05	UP	-0.33925	0.29143	0.50647	
transcript_HQ_CoA_transcript26699/f15p0/1667	52.41333333	104.86	64.56666667	1.155	2.7582E-09	5.2802E-08	UP	-0.84727	1.6256E-06	2.8929E-05		0.29563	0.0681	0.19716	
transcript_HQ_CoA_transcript26701/f4p0/1698	20.28333333	7.856666667	12.32333333	-1.222	0.00070491	0.0037848	DOWN	0.4996	0.12697	0.27105		-0.73195	0.020232	0.083754	
transcript_HQ_CoA_transcript26706/f2p0/1703	45.87333333	15.97666667	7.133333333	-1.3725	0.0092081	0.032918	DOWN	-1.2885	0.00077039	0.0051723	DOWN	-2.6744	4.0306E-08	1.9572E-06	DOWN
transcript_HQ_CoA_transcript26711/f6p0/1636	30.41333333	8.37	15.57333333	-1.6856	0.0035369	0.014951	DOWN	0.72483	0.15224	0.30779		-0.97256	0.010255	0.051249	
transcript_HQ_CoA_transcript26714/f8p0/1694	50.92	7.506666667	42.63333333	-2.5392	0.0089038	0.032024	DOWN	2.3474	3.2224E-12	2.14E-10	UP	-0.21633	0.86075	0.9373	
transcript_HQ_CoA_transcript26721/f2p0/1697	1.483333333	28.04333333	7.58	4.3749	2.067E-24	1.7334E-22	UP	-2.0218	7.7795E-06	0.00010885	DOWN	2.3397	4.4812E-06	0.00010923	UP
transcript_HQ_CoA_transcript26722/f2p0/1707	8.426666667	168.53	17.39	4.4907	1.6343E-27	1.7891E-25	UP	-3.4283	3.2119E-45	7.3588E-42	DOWN	1.049	0.017819	0.076329	
transcript_HQ_CoA_transcript26734/f2p0/1739	35.72666667	70.23333333	49.34666667	1.1368	3.0925E-07	3.9899E-06	UP	-0.65377	0.036632	0.1077		0.47042	0.11632	0.28358	
transcript_HQ_CoA_transcript26738/f4p0/1679	4.163333333	37.41666667	20.17666667	3.3517	7.8453E-07	9.3866E-06	UP	-1.0549	0.041059	0.11745		2.2812	3.5713E-05	0.00060922	UP
transcript_HQ_CoA_transcript26739/f2p0/1704	3.223333333	8.643333333	3.303333333	1.5978	0.0046703	0.018757	UP	-1.5402	0.0012442	0.0075888	DOWN	0.043967	0.92837	0.96836	
transcript_HQ_CoA_transcript26742/f2p0/1712	7.673333333	1.73	3.9	-2.0109	0.0014485	0.0070469	DOWN	1.0329	0.060069	0.15715		-0.98849	0.035479	0.12579	
transcript_HQ_CoA_transcript26745/f2p0/1686	90.44	35.77	63.11	-1.1742	0.00018143	0.0011829	DOWN	0.66427	0.041446	0.11835		-0.52128	0.0034049	0.022876	
transcript_HQ_CoA_transcript26750/f3p0/1647	67.19333333	173.1833333	95.44	1.5132	4.0075E-12	1.1836E-10	UP	-0.99581	2.3468E-05	0.00027985		0.50616	0.031709	0.1161	

transcript_HQ_CoA_transcript26753/f3p0/1735	20.11333333	6.866666667	15.25	-1.3937	6.6465E-05	0.00049355	DOWN	1.0104	0.022826	0.075973		-0.39307	0.33288	0.55006	
transcript_HQ_CoA_transcript26754/f2p0/1698	5.223333333	22.11	12.86666667	2.2498	4.2162E-08	6.4992E-07	UP	-0.93295	0.0024298	0.013003		1.3025	0.0037861	0.02483	UP
transcript_HQ_CoA_transcript26780/f6p0/1682	21.14666667	68.06333333	38.13333333	1.8452	1.5389E-31	2.2689E-29	UP	-0.97705	0.003353	0.016986		0.85457	0.011257	0.055024	
transcript_HQ_CoA_transcript26789/f3p0/1708	156.6633333	45.30333333	134.59	-1.6224	5.6655E-10	1.22E-08	DOWN	1.4065	0.00000763	0.00010723	UP	-0.22842	0.41743	0.63041	
transcript_HQ_CoA_transcript26792/f3p0/1692	1.903333333	3.576666667	4.87	1.074	0.014877	0.048214	UP	0.29789	0.41398	0.60399		1.3597	0.0018475	0.014358	UP
transcript_HQ_CoA_transcript26814/f2p0/1688	16.69333333	41.37333333	20.94333333	1.4762	3.4117E-05	0.00027574	UP	-1.1391	0.00070903	0.0048474	DOWN	0.32411	0.12355	0.29462	
transcript_HQ_CoA_transcript26829/f2p0/1693	0.043333333	234.64	13.53666667	12.548	2.9935E-27	3.2004E-25	UP	-4.2483	0.0026543	0.013954	DOWN	8.2927	0.00012463	0.0017033	UP
transcript_HQ_CoA_transcript26832/f2p0/1737	83.10666667	7.006666667	33.5	-3.3995	1.1763E-17	6.1443E-16	DOWN	2.0888	1.0714E-09	4.1606E-08	UP	-1.3223	6.5709E-07	2.1969E-05	DOWN
transcript_HQ_CoA_transcript26838/f2p0/1524	27.43	66.23333333	46.72333333	1.4268	1.2078E-17	6.2907E-16	UP	-0.63634	0.053667	0.14425		0.77781	0.021022	0.086149	
transcript_HQ_CoA_transcript26841/f2p0/1696	4.003333333	13.95333333	6.916666667	1.9645	1.8587E-07	2.5152E-06	UP	-1.1485	0.019936	0.068416		0.8014	0.11899	0.28726	
transcript_HQ_CoA_transcript26842/f2p0/1689	40.83333333	81.81333333	45.75666667	1.155	9.0123E-07	1.0685E-05	UP	-0.97719	7.4723E-06	0.0001056		0.16503	0.32981	0.54669	
transcript_HQ_CoA_transcript26847/f2p0/1683	0.176666667	47.38	100.98	8.2353	1.5897E-24	1.3517E-22	UP	0.96318	0.60988	0.7651		9.1848	2.1714E-06	6.0757E-05	UP
transcript_HQ_CoA_transcript26854/f7p0/1663	0	107.8966667	24.88333333	13.248	4.1567E-28	4.8096E-26	UP	-2.2345	0.059587	0.15636		10.998	1.4803E-06	4.3894E-05	UP
transcript_HQ_CoA_transcript26865/f2p0/1676	18.54333333	6.793333333	16.33	-1.28	0.00070176	0.0037734	DOWN	1.0979	0.0018403	0.010398	UP	-0.19388	0.47233	0.67837	
transcript_HQ_CoA_transcript26869/f3p0/1704	39.93	181.4933333	60.54333333	2.3534	1.7904E-16	8.5685E-15	UP	-1.7238	0.00006188	0.00064187	DOWN	0.6151	0.12377	0.29488	
transcript_HQ_CoA_transcript26892/f3p0/1676	57.15333333	15.39333333	36.86333333	-1.7391	4.9202E-11	1.2528E-09	DOWN	1.11	1.164E-14	1.2776E-12	UP	-0.64069	0.0081697	0.043496	
transcript_HQ_CoA_transcript26894/f2p0/1682	21.27333333	91.11333333	30.53	2.2702	1.1922E-15	5.2772E-14	UP	-1.7513	4.3355E-08	1.1755E-06	DOWN	0.50586	0.04258	0.14321	
transcript_HQ_CoA_transcript26905/f6p0/1681	0	117.6666667	0.176666667	13.37	6.8147E-16	3.0992E-14	UP	-9.5558	2.8824E-07	6.4195E-06	DOWN	3.7845	0.3366	0.55434	
transcript_HQ_CoA_transcript26915/f8p0/1642	9.39	58.49666667	15.52	2.7984	3.0336E-12	9.1066E-11	UP	-2.07	6.8343E-08	1.7594E-06	DOWN	0.71586	0.019983	0.083098	
transcript_HQ_CoA_transcript26922/f2p0/1672	4.24	39.16333333	13.52	3.3854	8.9796E-17	4.4369E-15	UP	-1.6885	0.0011555	0.0071724	DOWN	1.6831	0.00047882	0.0050043	UP
transcript_HQ_CoA_transcript2693/f2p0/4594	12.00666667	3.156666667	5.94	-1.7847	0.0011514	0.0057879	DOWN	0.79491	0.16659	0.32795		-1.0044	0.0077538	0.041822	DOWN
transcript_HQ_CoA_transcript26937/f2p0/1676	5.423333333	35.96	10.47333333	2.8502	1.4257E-05	0.00012745	UP	-1.8985	0.0044018	0.021209	DOWN	0.93889	0.010355	0.051659	
transcript_HQ_CoA_transcript26945/f3p0/1676	13.50666667	28.20333333	11.89666667	1.2333	0.0012122	0.0060569	UP	-1.3965	5.4642E-08	1.4521E-06	DOWN	-0.17791	0.61299	0.7814	
transcript_HQ_CoA_transcript2695/f2p0/4568	17.63666667	3.93	12.11	-2.016	1.1725E-13	4.1623E-12	DOWN	1.4734	6.768E-06	9.7219E-05	UP	-0.55483	0.030137	0.11215	
transcript_HQ_CoA_transcript26950/f2p0/1677	5.543333333	12.49	13.84	1.3228	6.4808E-08	9.617E-07	UP	0.009908	0.97127	0.98502		1.3203	1.8525E-05	0.00035309	UP
transcript_HQ_CoA_transcript26964/f2p0/1684	38.09666667	6.093333333	21.51	-2.5021	1.5153E-10	3.5883E-09	DOWN	1.6627	1.4698E-07	3.4986E-06	UP	-0.8491	0.034796	0.12399	
transcript_HQ_CoA_transcript26968/f3p0/1674	8.1	34.32333333	7.266666667	2.2586	1.9216E-12	5.9244E-11	UP	-2.392	2.751E-18	5.1451E-16	DOWN	-0.14811	0.69689	0.83988	
transcript_HQ_CoA_transcript26972/f2p0/1695	25.45333333	7.83	17.33333333	-1.5583	2.0641E-07	2.7665E-06	DOWN	0.99749	0.0062505	0.027929		-0.57088	0.083188	0.2257	
transcript_HQ_CoA_transcript26979/f3p0/1714	8.27	18.01666667	7.94	1.2971	0.0010898	0.0055311	UP	-1.342	0.0026203	0.013837	DOWN	-0.059648	0.86617	0.9406	
transcript_HQ_CoA_transcript26989/f3p0/1669	13.33666667	42.69	37.93	1.8522	0.00057908	0.0032006	UP	-0.31698	0.45226	0.63956		1.5172	0.0053334	0.032014	UP
transcript_HQ_CoA_transcript26994/f4p0/1672	5.08	70.26666667	18.13333333	3.9386	2.9602E-14	1.1345E-12	UP	-2.0943	0.00039781	0.0030193	DOWN	1.8322	0.0016754	0.013336	UP
transcript_HQ_CoA_transcript26998/f2p0/1697	1.003333333	421.6366667	128.94	8.8747	3.64E-246	3.327E-242	UP	-1.8187	0.27435	0.46147		7.0409	2.9515E-05	0.00052394	UP
transcript_HQ_CoA_transcript27000/f2p0/1676	6.263333333	19.57	7.286666667	1.7883	1.1073E-10	2.6812E-09	UP	-1.5692	6.5486E-08	1.7074E-06	DOWN	0.20739	0.57395	0.75441	
transcript_HQ_CoA_transcript27013/f2p0/1670	4.886666667	33.61333333	9.05	2.959	7.4612E-15	3.0861E-13	UP	-2.0573	1.1264E-07	2.7491E-06	DOWN	0.88645	0.011957	0.057244	
transcript_HQ_CoA_transcript27015/f2p0/1675	13.77666667	32.28333333	27.06666667	1.3794	2.0987E-05	0.00017938	UP	-0.39465	0.172	0.33542		0.97169	3.8865E-05	0.00065029	
transcript_HQ_CoA_transcript27026/f3p0/1664	1.586666667	5.916666667	3.803333333	2.089	0.00066925	0.0036231	UP	-0.81049	0.22051	0.39798		1.2625	0.046157	0.15172	
transcript_HQ_CoA_transcript27035/f2p0/1678	3.88	7.486666667	5.146666667	1.0839	0.009516	0.033801	UP	-0.68605	0.039093	0.113		0.38698	0.33662	0.55434	
transcript_HQ_CoA_transcript27037/f3p0/1660	115.01	35.83	63.22666667	-1.5085	2.6928E-05	0.00022326	DOWN	0.65173	0.056225	0.14977		-0.86798	0.00013861	0.0018571	
transcript_HQ_CoA_transcript27038/f2p0/1673	1.183333333	78.23333333	7.316666667	6.2041	7.8799E-28	9.0037E-26	UP	-3.5782	7.859E-07	0.00001564	DOWN	2.6128	0.0027664	0.019537	UP
transcript_HQ_CoA_transcript2705/f2p0/4569	1.426666667	0.436666667	2.89	-1.5381	0.0057985	0.022388	DOWN	2.6059	0.009072	0.037188	UP	1.0544	0.25816	0.46879	
transcript_HQ_CoA_transcript27053/f2p0/1696	3.876666667	56.07333333	9.196666667	4.0198	9.0737E-12	2.5879E-10	UP	-2.7381	0.00013458	0.0012296	DOWN	1.2659	0.063134	0.18727	
transcript_HQ_CoA_transcript27060/f2p0/1690	3.593333333	14.31	3.99	2.1618	1.5867E-05	0.0001398	UP	-2.0014	0.00001734	0.00021562	DOWN	0.14764	0.70536	0.84502	
transcript_HQ_CoA_transcript27062/f20p0/1644	91.95666667	197.45	241.19	1.2675	2.374E-07	3.1496E-06	UP	0.1284	0.76784	0.86965		1.3793	0.00057782	0.0057755	UP
transcript_HQ_CoA_transcript27063/f2p0/1668	66.27666667	29.23333333	42.04333333	-1.0249	8.3397E-10	1.7545E-08	DOWN	0.37577	0.024375	0.079966		-0.66226	0.00014477	0.0019227	
transcript_HQ_CoA_transcript27075/f3p0/1678	86.49666667	15.34333333	23.04333333	-2.3763	0.00086207	0.0045185	DOWN	0.46298	0.5243	0.69708		-1.9213	3.5634E-06	9.0967E-05	DOWN
transcript_HQ_CoA_transcript27080/f3p0/1640	63.55666667	22.03	36.42333333	-1.3747	1.2403E-07	1.7389E-06	DOWN	0.56944	0.03346	0.1011		-0.81826	0.0038789	0.025265	
transcript_HQ_CoA_transcript27102/f2p0/1667	1.353333333	8.973333333	0.7	2.8428	0.010346	0.036242	UP	-3.7941	0.00055829	0.0039786	DOWN	-0.95898	0.18069	0.37582	
transcript_HQ_CoA_transcript2712/f3p0/4573	6.7	1.84	5.36	-1.7227	0.00002271	0.00019221	DOWN	1.4	2.8669E-05	0.00033469	UP	-0.33276	0.3487	0.56671	
transcript_HQ_CoA_transcript27120/f2p0/1680	4.023333333	28.90333333	7.223333333	3.0221	3.0131E-16	1.4088E-14	UP	-2.153	0.00010422	0.00099667	DOWN	0.85363	0.14115	0.3206	
transcript_HQ_CoA_transcript27128/f3p0/1663	32.46	64.39666667	32.42333333	1.1536	0.00017665	0.0011567	UP	-1.149	2.3835E-06	4.0228E-05	DOWN	-0.0083104	0.97808	0.99123	
transcript_HQ_CoA_transcript27138/f2p0/1654	45.86666667	9.183333333	21.89333333	-2.177	3.0865E-09	5.8595E-08	DOWN	1.1084	0.0086988	0.036003	UP	-1.0809	0.0027368	0.019351	DOWN
transcript_HQ_CoA_transcript27142/f2p0/1667	26.66	67.54	34	1.5049	1.8667E-10	4.3586E-09	UP	-1.1411	1.2513E-06	2.2913E-05	DOWN	0.35019	0.061515	0.18432	
transcript_HQ_CoA_transcript27144/f2p0/1672	11.86333333	0.746666667	11.53	-3.8409	1.7814E-10	4.1754E-09	DOWN	3.8115	7.231E-20	1.6992E-17	UP	-0.036959	0.93156	0.97012	
transcript_HQ_CoA_transcript27153/f4p0/1626	175.0666667	333.16	255.1866667	1.0845	3.9014E-11	1.0088E-09	UP	-0.53445	1.2294E-06	2.2601E-05		0.53821	0.00037438	0.0041148	
transcript_HQ_CoA_transcript27155/f4p0/1625	18.00333333	32.69	21.11	1.0138	0.0069741	0.026122	UP	-0.75848	0.10705	0.23978		0.24168	0.59154	0.7671	
transcript_HQ_CoA_transcript27162/f4p0/1661	15.34	4.333333333	14.62666667	-1.6813	0.010619	0.037005	DOWN	1.6238	0.00092381	0.0059938	UP	-0.069685	0.9048	0.9576	
transcript_HQ_CoA_transcript27164/f2p0/1660	0.986666667	2.826666667	2.063333333	1.6807	0.011723	0.040087	UP	-0.57222	0.35608	0.54823		1.0984	0.070616	0.2018	
transcript_HQ_CoA_transcript27178/f4p0/1646	10.35666667	0.103333333	38.09	-6.2489	0.00022737	0.0014368	DOWN	8.1281	1.084E-33	1.0457E-30	UP	1.8937	0.28594	0.50011	
transcript_HQ_CoA_transcript27183/f5p0/1652	0	73.39	18.43	12.7	6.8974E-25	6.0917E-23	UP	-2.1328	0.26741	0.45386		10.551	0.0069247	0.03861	UP
transcript_HQ_CoA_transcript27190/f3p0/1655	3.213333333	15.47666667	2.023333333	2.4577	6.036E-06	5.9876E-05	UP	-3.112	6.2482E-09	2.0348E-07	DOWN	-0.66559	0.17711	0.3708	
transcript_HQ_CoA_transcript27192/f2p0/1642	32.30333333	67.93666667	36.18666667	1.2389	5.7125E-09	1.0289E-07	UP	-1.0521	0.0015289	0.0089472	DOWN	0.17206	0.61253	0.78111	
transcript_HQ_CoA_transcript27194/f2p0/1708	335.37	106.5433333	214.1666667	-1.482	5.2087E-08	7.896E-07	DOWN	0.84297	0.003287	0.016717		-0.65149	0.00082295	0.0076676	
transcript_HQ_CoA_transcript27204/f3p0/1636	20.34666667	38.94666667	42.23	1.0879	0.00031715	0.0019123	UP	-0.014225	0.95397	0.97609		1.0607	1.2896E-05	0.0002607	UP
transcript_HQ_CoA_transcript27205/f2p0/1653	52.69333333	16.69333333	45.95	-1.5256	9.1075E-05	0.00064964	DOWN	1.3391	0.00069355	0.0047593	UP	-0.19808	0.40182	0.61713	
transcript_HQ_CoA_transcript27209/f2p0/1657	1.546666667	78.01666667	6.18	5.8226	3.322E-41	6.9807E-39	UP	-3.807	5.8175E-10	2.3854E-08	DOWN	2.0006	0.0033146	0.0224	UP
transcript_HQ_CoA_transcript27212/f2p0/1655	4.556666667	32.14	7.306666667	2.966	3.0891E-11	8.1143E-10	UP	-2.2839	3.287E-10	1.4552E-08	DOWN	0.66925	0.10359	0.26302	
transcript_HQ_CoA_transcript27214/f2p0/1657	8.396666667	25.64	19.09	1.7807	0.0014987	0.0072602	UP	-0.5986	0.17982	0.34613		1.1723	0.0039724	0.025737	UP
transcript_HQ_CoA_transcript27226/f2p0/1676	42.74333333	85.2	52.78666667	1.1503	3.0073E-07	3.8992E-06	UP	-0.83098	0.0014663	0.0086835		0.3071	0.29099	0.50603	
transcript_HQ_CoA_transcript27228/f6p0/1633	5.75	51.20666667	14.31333333	3.3571	1.4853E-09	2.9742E-08	UP	-2.0151	3.1533E-05	0.00036557	DOWN	1.3254	0.0007568	0.0071751	UP
transcript_HQ_CoA_transcript27237/f2p0/1658	0.25	45.92333333	3.886666667	7.6983	1.6602E-42	3.6568E-40	UP	-3.6562	0.042055	0.1196		4.0328	0.0099146	0.049954	UP
transcript_HQ_CoA_transcript27252/f3p0/1650	12.35	64.86666667	11.95	2.5702	2.8083E-13	9.5787E-12	UP	-2.5695	0.17831	0.34394		-0.014218	0.99399	0.99674	
transcript_HQ_CoA_transcript27257/f4p0/1625	64.46666667	140.8033333	85.8	1.295	1.2952E-09	2.6251E-08	UP	-0.86702	9.5073E-06	0.00013004		0.4147	0.013485	0.062311	
transcript_HQ_CoA_transcript27258/f2p0/1630	2.523333333	56.96	7.11	4.653	6.7513E-82	6.8571E-79	UP	-3.1532	2.2426E-12	1.587E-10	DOWN	1.4839	0.0089393	0.046469	UP
transcript_HQ_CoA_transcript27277/f3p0/1653	5.686666667	1.56	18.85333333	-1.7379	0.01506	0.048721	DOWN	3.4771	8.7045E-12	5.2139E-10	UP	1.7335	0.0037229	0.024503	UP
transcript_HQ_CoA_transcript27301/f4p0/1631	73.55333333	26.89666667	43.85	-1.2694	0.00033413	0.0020015	DOWN	0.53389	0.12846	0.27308		-0.74949	4.7407E-06	0.00011404	
transcript_HQ_CoA_transcript27302/f2p0/1652	8.23	17.61666667	14.54666667	1.2772	9.4851E-05	0.0006729	UP	-0.42842	0.16912	0.33128		0.83495	0.006979	0.038818	
transcript_HQ_CoA_transcript27303/f4p0/1600	22.06666667	45.37	17.08	1.2179	0.00019119	0.001236	UP	-1.5779	4.5699E-07	9.7172E-06	DOWN	-0.37236	0.1567	0.34288	
transcript_HQ_CoA_transcript27309/f2p0/1676	8.206666667	62.09333333	81.38666667	3.0727	2.251E-10	5.1764E-09	UP	0.26759	0.81569	0.89961		3.3276	0.0066684	0.037707	UP
transcript_HQ_CoA_transcript27317/f2p0/1693	43	111.6433333	71.31666667	1.5391	5.5819E-08	8.4059E-07	UP	-0.78188	0.0025864	0.013689		0.74441	0.01076	0.053049	

transcript_HQ_CoA_transcript27323/f2p0/1643	7.753333333	29.05333333	9.016666667	2.0632	7.2563E-16	3.2918E-14	UP	-1.8383	1.7198E-11	9.64E-10	DOWN	0.21261	0.54114	0.73206	
transcript_HQ_CoA_transcript27325/f2p0/1668	2.083333333	4.496666667	5.12	1.2484	0.01208	0.04102	UP	0.040111	0.90387	0.94879		1.2783	0.0045854	0.028738	UP
transcript_HQ_CoA_transcript27326/f3p0/1629	147.8233333	41.76333333	96.32666667	-1.6672	1.4305E-10	3.4009E-09	DOWN	1.0484	1.7022E-05	0.00021212	UP	-0.62909	0.01048	0.052042	
transcript_HQ_CoA_transcript27336/f2p0/1630	40.69	15.11	24.72333333	-1.2691	6.5179E-05	0.00048557	DOWN	0.54772	0.015476	0.056405		-0.73297	0.011644	0.056199	
transcript_HQ_CoA_transcript27342/f10p0/1616	135.2	19.45333333	79.94666667	-2.617	2.5942E-07	3.4071E-06	DOWN	1.869	0.00013768	0.0012505	UP	-0.76158	0.034245	0.12269	
transcript_HQ_CoA_transcript27352/f2p0/1637	48.08666667	9.7	34.21	-2.1519	3.5716E-11	9.3015E-10	DOWN	1.6642	2.2005E-07	5.048E-06	UP	-0.50124	0.1637	0.3525	
transcript_HQ_CoA_transcript2736/f3p0/4530	11.05	2.916666667	6.403333333	-1.7786	5.0911E-05	0.00039058	DOWN	0.97316	0.0089115	0.036664		-0.8152	0.066198	0.19355	
transcript_HQ_CoA_transcript27367/f3p0/1642	20.45333333	7.15	16.32	-1.3665	0.00017888	0.0011684	DOWN	1.0377	0.0042294	0.020612	UP	-0.34027	0.29047	0.50568	
transcript_HQ_CoA_transcript27374/f2p0/1658	0.383333333	195.58	19.95666667	9.1724	5.4863E-60	2.134E-57	UP	-3.4376	0.0023222	0.01253	DOWN	5.718	8.74E-10	6.2911E-08	UP
transcript_HQ_CoA_transcript27381/f2p0/1645	0	209.8933333	12.01333333	14.19	9.388E-32	1.4068E-29	UP	-4.2466	0.001515	0.0088828	DOWN	9.9284	8.0622E-05	0.0011877	UP
transcript_HQ_CoA_transcript27389/f3p0/1620	19.26	6.32	13.89	-1.4582	4.1844E-05	0.00032945	DOWN	0.99781	0.0078411	0.033218		-0.47491	0.11273	0.27766	
transcript_HQ_CoA_transcript27394/f2p0/1655	0.043333333	29.32333333	1.003333333	9.4744	3.546E-12	1.0575E-10	UP	-4.9892	0.0024967	0.013295	DOWN	4.4643	0.044585	0.14798	
transcript_HQ_CoA_transcript27395/f2p0/1632	24.42333333	49.14666667	37.52333333	1.1732	3.8787E-10	8.6056E-09	UP	-0.53485	0.033526	0.10122		0.62521	0.014263	0.064735	
transcript_HQ_CoA_transcript27419/f2p0/1663	2.33	0.543333333	1.936666667	-1.9121	0.010176	0.035761	DOWN	1.6435	0.03688	0.10821		-0.282	0.65745	0.81268	
transcript_HQ_CoA_transcript27423/f15p0/1619	996.53	41.31666667	26.30333333	-4.4765	0.00013287	0.00090199	DOWN	-0.78024	0.35803	0.55007		-5.2588	2.367E-06	6.5138E-05	DOWN
transcript_HQ_CoA_transcript27437/f3p0/1640	16.28666667	38.13666667	15.88333333	1.3983	0.00010068	0.00070737	UP	-1.4218	0.00004424	0.00048672	DOWN	-0.037983	0.88409	0.9482	
transcript_HQ_CoA_transcript27439/f7p0/1632	57.33	205.59	152.03	1.9955	1.1667E-20	7.7002E-19	UP	-0.56991	0.14792	0.30168		1.411	0.00023798	0.0028606	UP
transcript_HQ_CoA_transcript2745/f2p0/4601	10.25	3.043333333	5.13	-1.602	3.4702E-05	0.00027973	DOWN	0.61305	0.061096	0.15916		-1.0019	0.00037661	0.0041319	DOWN
transcript_HQ_CoA_transcript27457/f2p0/1628	26.47333333	10.21333333	26.03333333	-1.2205	0.00051268	0.0028875	DOWN	1.1952	0.00007847	0.00078423	UP	-0.035234	0.90475	0.9576	
transcript_HQ_CoA_transcript27459/f2p0/1464	17.94333333	6.37	21.61666667	-1.3609	0.0018913	0.0088478	DOWN	1.6435	0.00012256	0.0011357	UP	0.27042	0.26961	0.48209	
transcript_HQ_CoA_transcript2746/f2p0/4548	4.213333333	8.026666667	4.636666667	1.0933	0.00057614	0.003187	UP	-0.94328	0.0026804	0.014065		0.13698	0.55268	0.73965	
transcript_HQ_CoA_transcript27467/f2p0/1651	3.576666667	18.77333333	6.36	2.5193	0.0002463	0.0015347	UP	-1.6905	0.00062354	0.0043539	DOWN	0.81928	0.17212	0.364	
transcript_HQ_CoA_transcript27468/f2p0/1620	32.63333333	71.44	56.65666667	1.303	0.00005452	0.00041568	UP	-0.49971	0.089651	0.21085		0.79115	2.7699E-05	0.00049456	
transcript_HQ_CoA_transcript27471/f2p0/1641	32.75666667	5.613333333	25.94	-2.3772	1.1787E-09	2.4105E-08	DOWN	2.0421	1.7884E-06	3.1307E-05	UP	-0.3499	0.44213	0.65293	
transcript_HQ_CoA_transcript27480/f2p0/1644	17.65333333	35.31	28.38333333	1.1568	2.2357E-05	0.00018949	UP	-0.45674	0.03227	0.098399		0.6879	0.0098339	0.04967	
transcript_HQ_CoA_transcript27485/f2p0/1616	7.786666667	35.00666667	11.41333333	2.3464	5.7532E-08	8.6347E-07	UP	-1.7955	5.1009E-05	0.00054998	DOWN	0.53718	0.072608	0.20535	
transcript_HQ_CoA_transcript27513/f2p0/1630	34.39666667	168.24	50.38666667	2.4404	3.4899E-27	3.688E-25	UP	-1.8719	2.414E-09	8.6084E-08	DOWN	0.55639	0.059775	0.1809	
transcript_HQ_CoA_transcript27518/f2p0/1657	1.223333333	30.82666667	1.613333333	4.7925	4.117E-06	4.2142E-05	UP	-4.4159	4.2193E-22	1.4061E-19	DOWN	0.37093	0.76612	0.88369	
transcript_HQ_CoA_transcript27558/f2p0/1628	19.69	43.33	21	1.3111	2.1164E-06	0.00002321	UP	-1.1959	0.00011407	0.0010716	DOWN	0.10105	0.73814	0.86671	
transcript_HQ_CoA_transcript27563/f2p0/1640	12.45333333	53.34666667	20.71333333	2.2636	1.2624E-24	1.0835E-22	UP	-1.5071	1.779E-07	4.1541E-06	DOWN	0.74242	0.030245	0.1124	
transcript_HQ_CoA_transcript27569/f2p0/1627	3.51	115.41	10.71666667	5.2135	1.4237E-24	1.2163E-22	UP	-3.5837	2.5796E-08	7.3417E-07	DOWN	1.6157	0.014277	0.064783	
transcript_HQ_CoA_transcript27575/f2p0/1641	23.45333333	55.90333333	7.7	1.399	0.00019063	0.0012332	UP	-2.9853	1.6327E-05	0.00020549	DOWN	-1.6028	0.013035	0.060869	
transcript_HQ_CoA_transcript27578/f7p0/1615	34.96333333	112.7433333	87.41333333	1.8626	3.5632E-10	7.9831E-09	UP	-0.53344	0.23	0.40988		1.3123	0.0012285	0.010542	UP
transcript_HQ_CoA_transcript2760/f2p0/4577	11.78666667	4.676666667	9.29	-1.1646	1.1528E-05	0.00010606	DOWN	0.82362	0.0023213	0.01253		-0.35473	0.12608	0.29819	
transcript_HQ_CoA_transcript27604/f8p0/1565	135.7	52.93	84.22666667	-1.2005	1.3872E-07	1.9213E-06	DOWN	0.51839	0.02837	0.089654		-0.69488	0.011334	0.05525	
transcript_HQ_CoA_transcript27606/f3p0/1615	48.16	10.99333333	8.756666667	-1.9732	0.00023622	0.0014825	DOWN	-0.46401	0.15167	0.30714		-2.4524	5.5165E-07	1.8997E-05	DOWN
transcript_HQ_CoA_transcript27612/f3p0/1606	0.466666667	8.313333333	0.926666667	4.3253	3.7872E-14	1.4305E-12	UP	-3.3297	0.0010899	0.0068534	DOWN	0.98186	0.49537	0.69727	
transcript_HQ_CoA_transcript27617/f3p0/1608	10.94666667	21.78666667	19.84333333	1.1521	0.01171	0.040061	UP	-0.27201	0.37171	0.56404		0.86498	0.038798	0.13434	
transcript_HQ_CoA_transcript27622/f3p0/1618	7.073333333	1.8	7.91	-1.7804	0.0035654	0.015061	DOWN	1.9538	0.0014606	0.0086612	UP	0.16161	0.64202	0.80117	
transcript_HQ_CoA_transcript27626/f2p0/1626	1.933333333	0.193333333	1.763333333	-3.186	0.00023309	0.0014664	DOWN	3.0626	0.00011046	0.0010463	UP	-0.13818	0.78476	0.89562	
transcript_HQ_CoA_transcript27629/f9p0/1602	1.62	166.84	17.59333333	6.8645	4.1556E-29	5.2759E-27	UP	-3.3728	0.0060559	0.027272	DOWN	3.4714	0.0017629	0.013899	UP
transcript_HQ_CoA_transcript27643/f5p0/1591	10.96333333	39.32666667	42.78666667	2.0057	2.4319E-10	5.5575E-09	UP	-0.029884	0.94154	0.96914		1.9595	1.8639E-05	0.00035453	UP
transcript_HQ_CoA_transcript27650/f2p0/1607	19.64666667	48.44	30.67333333	1.4663	6.5113E-06	6.4241E-05	UP	-0.82727	0.019864	0.068271		0.62794	0.036714	0.12885	
transcript_HQ_CoA_transcript27652/f4p0/1611	59.61333333	21.99666667	43.20333333	-1.2637	0.00037034	0.0021883	DOWN	0.80979	0.0044953	0.021576		-0.46711	0.11103	0.27526	
transcript_HQ_CoA_transcript27665/f2p0/1610	1.926666667	5.73	3.673333333	1.7185	0.00019254	0.0012438	UP	-0.75958	0.18849	0.35731		0.94708	0.14612	0.32763	
transcript_HQ_CoA_transcript27667/f3p0/1636	186.5	32.14666667	100.85	-2.3769	2.914E-08	4.6446E-07	DOWN	1.4787	0.00014845	0.0013364	UP	-0.90766	0.017083	0.073806	
transcript_HQ_CoA_transcript27670/f3p0/1639	9.65	4.356666667	11.56333333	-1.0003	0.00059671	0.0032819	DOWN	1.2605	0.00015704	0.0014007	UP	0.24896	0.42969	0.64168	
transcript_HQ_CoA_transcript27677/f2p0/1632	7.81	25.78	9.853333333	1.9053	2.7283E-07	3.5679E-06	UP	-1.5446	0.0036175	0.018081	DOWN	0.34396	0.53493	0.7274	
transcript_HQ_CoA_transcript27685/f2p0/1608	12.28	2.586666667	8.59	-2.0821	5.2168E-09	9.4805E-08	DOWN	1.5655	0.0010342	0.0065679	UP	-0.52898	0.19338	0.39216	
transcript_HQ_CoA_transcript27704/f2p0/1639	12.16333333	2.636666667	7.206666667	-2.0552	0.00001812	0.00015744	DOWN	1.3061	0.00054682	0.0039136	UP	-0.75901	0.098513	0.25435	
transcript_HQ_CoA_transcript27709/f2p0/1606	15.46666667	2.21	5.26	-2.6135	5.8091E-08	8.698E-07	DOWN	1.0672	0.049731	0.13588		-1.5579	6.996E-06	0.00015775	DOWN
transcript_HQ_CoA_transcript27719/f2p0/1620	3.343333333	0.953333333	1.32	-1.6705	0.0053898	0.021103	DOWN	0.33254	0.54457	0.71408		-1.3495	0.013447	0.062204	
transcript_HQ_CoA_transcript27722/f2p0/1604	13.02	144.4766667	79.99	3.6535	1.7527E-13	6.1621E-12	UP	-1.0123	0.0074403	0.03193	DOWN	2.6256	2.7198E-10	2.1611E-08	UP
transcript_HQ_CoA_transcript27725/f2p0/1639	8.456666667	15.93	13.80666667	1.0665	4.0473E-06	4.1546E-05	UP	-0.35113	0.064958	0.16673		0.70256	0.0019742	0.015142	
transcript_HQ_CoA_transcript27731/f3p0/1612	63.85	19.02666667	52.12333333	-1.6185	0.002478	0.011068	DOWN	1.3342	0.004088	0.020045	UP	-0.2944	0.43217	0.64402	
transcript_HQ_CoA_transcript27738/f2p0/1620	0.043333333	177.2233333	13.39	12.089	4.9965E-27	5.1318E-25	UP	-3.8712	0.041975	0.11939		8.2142	0.010534	0.052188	
transcript_HQ_CoA_transcript27744/f6p0/1603	2.846666667	22.46666667	13.49666667	3.1095	9.9314E-06	9.2968E-05	UP	-0.85506	0.11664	0.25498		2.2409	0.003077	0.021206	UP
transcript_HQ_CoA_transcript27747/f5p0/1607	34.82666667	68.74	37.46666667	1.1453	0.0003262	0.0019604	UP	-1.0298	1.1369E-06	2.1177E-05	DOWN	0.10339	0.72459	0.85849	
transcript_HQ_CoA_transcript27763/f2p0/1616	9.483333333	44.93333333	23.49	2.4108	9.6405E-08	1.3791E-06	UP	-1.0806	1.0648E-12	8.1316E-11	DOWN	1.3152	0.0034517	0.023131	UP
transcript_HQ_CoA_transcript27768/f2p0/1640	9.776666667	39.82	23.44666667	2.1898	6.0946E-12	1.7658E-10	UP	-0.91267	4.5568E-05	0.00049893		1.2654	0.00013227	0.0017878	UP
transcript_HQ_CoA_transcript27770/f8p0/1602	38.23666667	70.38666667	59.04333333	1.0534	9.6682E-05	0.00068245	UP	-0.41321	0.055657	0.14864		0.62664	0.0025603	0.018372	
transcript_HQ_CoA_transcript27776/f2p0/1610	15.32	3.486666667	13.23666667	-1.9395	0.00069514	0.0037403	DOWN	1.7458	0.0060776	0.027337	UP	-0.21056	0.69139	0.83592	
transcript_HQ_CoA_transcript27777/f2p0/1641	203.81	43.15333333	130.64	-2.0961	2.6495E-12	8.0064E-11	DOWN	1.4397	1.4372E-05	0.00018434	UP	-0.66598	0.067079	0.19519	
transcript_HQ_CoA_transcript27782/f2p0/1629	3.066666667	144.0266667	31.02333333	5.7427	3.9423E-29	5.0401E-27	UP	-2.3733	5.2697E-13	4.2928E-11	DOWN	3.3525	3.5026E-13	5.6895E-11	UP
transcript_HQ_CoA_transcript27788/f3p0/1589	31.60333333	76.35666667	27.67	1.4309	0.0010292	0.0052764	UP	-1.6132	0.00013754	0.0012504	DOWN	-0.19516	0.51545	0.71259	
transcript_HQ_CoA_transcript27794/f2p0/1603	10.39	35.02	14.05333333	1.9108	3.8141E-07	4.8322E-06	UP	-1.4684	3.4078E-06	5.4262E-05	DOWN	0.43106	0.29215	0.50713	
transcript_HQ_CoA_transcript2780/f2p0/4534	6.543333333	27.44666667	10.50333333	2.2331	1.2008E-15	5.3028E-14	UP	-1.538	8.7148E-06	0.00012028	DOWN	0.68231	0.030377	0.11269	
transcript_HQ_CoA_transcript27802/f4p0/1602	0.05	29.99	45.67333333	9.4933	1.129E-18	6.3704E-17	UP	0.50024	0.74976	0.85884		9.9806	3.3478E-05	0.00058245	UP
transcript_HQ_CoA_transcript27818/f3p0/1579	168.1666667	57.63666667	107.1866667	-1.3759	6.1717E-10	1.3243E-08	DOWN	0.73413	0.00083188	0.005499		-0.65449	9.2053E-06	0.00019624	
transcript_HQ_CoA_transcript27821/f4p0/1594	0	82.30333333	2.896666667	12.807	1.07E-23	8.4683E-22	UP	-4.9658	0.0028566	0.01482	DOWN	7.8257	0.045272	0.14969	
transcript_HQ_CoA_transcript27829/f4p0/1591	8.153333333	36.93	15.09333333	2.3422	1.6958E-10	3.9822E-09	UP	-1.4415	0.00027624	0.0022256	DOWN	0.8885	0.017768	0.076168	
transcript_HQ_CoA_transcript27832/f2p0/1591	19.34666667	1.996666667	9.976666667	-3.1313	8.6098E-22	6.0774E-20	DOWN	2.1753	1.6216E-06	2.8887E-05	UP	-0.96349	0.0052906	0.03185	
transcript_HQ_CoA_transcript27843/f2p0/1601	6.226666667	14.1	4.853333333	1.3383	5.3906E-07	6.6142E-06	UP	-1.6879	4.1866E-10	1.8013E-08	DOWN	-0.36184	0.27775	0.49171	
transcript_HQ_CoA_transcript27850/f2p0/1604	51.68666667	12.71	28.65666667	-1.8679	4.7277E-11	1.2105E-09	DOWN	1.0145	3.6416E-06	5.7441E-05	UP	-0.86412	0.00032661	0.0036846	
transcript_HQ_CoA_transcript27856/f3p0/1603	10.03	20.09333333	17.48333333	1.1774	0.0078028	0.028679	UP	-0.35001	0.082075	0.19758		0.81234	0.063094	0.18724	
transcript_HQ_CoA_transcript27858/f3p0/1621	57.50333333	25.33	96.38	-1.0174	0.001051	0.0053656	DOWN	1.7595	1.3188E-05	0.00017168	UP	0.7301	0.020851	0.085619	
transcript_HQ_CoA_transcript27870/f2p0/1601	2.76	37.72666667	14.9	3.9287	4.9142E-46	1.2478E-43	UP	-1.474	2.2599E-05	0.00027144	DOWN	2.4426	1.1615E-09	8.015E-08	UP
transcript_HQ_CoA_transcript27872/f3p0/1604	56.20666667	110.6533333	77.44666667	1.1366	2.0732E-07	2.7753E-06	UP	-0.66311	3.1515E-07	6.9545E-06		0.46041	0.029409	0.11017	
transcript_HQ_CoA_transcript27877/f2p0/1602	23.96333333	50.23666667	51.37666667	1.2108	9.5277E-05	0.00067554	UP	-0.10434	0.7062	0.83171		1.0942	0.00004871	0.0007829	UP

transcript_HQ_CoA_transcript27879/f3p0/1596	29.35	9.213333333	42.18	-1.5131	1.8506E-05	0.00016012	DOWN	2.0454	2.3599E-08	6.7586E-07	UP	0.51932	0.19157	0.38978	
transcript_HQ_CoA_transcript27883/f2p0/1591	34.15	9.493333333	15.34	-1.6734	0.00056726	0.0031426	DOWN	0.55541	0.10994	0.24438		-1.1329	0.013879	0.063498	
transcript_HQ_CoA_transcript27916/f2p0/1619	0	37.56333333	1.323333333	11.732	1.3551E-16	6.5538E-15	UP	-5.0134	0.0018594	0.010477	DOWN	6.7005	0.0055965	0.033158	UP
transcript_HQ_CoA_transcript27926/f2p0/1609	16.99333333	4.413333333	5.583333333	-1.8001	1.0403E-08	1.7925E-07	DOWN	0.18596	0.63673	0.78301		-1.624	3.1094E-06	8.1532E-05	DOWN
transcript_HQ_CoA_transcript27927/f2p0/1587	29.95666667	5.3	32.69	-2.3447	5.9323E-06	5.8975E-05	DOWN	2.4811	8.8323E-10	3.5041E-08	UP	0.12382	0.79691	0.90282	
transcript_HQ_CoA_transcript27928/f2p0/1595	105.3833333	42.71	59.45333333	-1.153	8.2164E-08	1.1893E-06	DOWN	0.33429	0.11917	0.25876		-0.8292	0.00085015	0.007877	
transcript_HQ_CoA_transcript27932/f2p0/1611	4.333333333	8.243333333	8.64	1.0773	0.00046168	0.0026335	UP	-0.085286	0.76737	0.86941		0.98101	0.0056171	0.033259	
transcript_HQ_CoA_transcript27938/f2p0/1590	2.41	144.3333333	2.843333333	6.044	2.1211E-13	7.3306E-12	UP	-5.7545	6.4108E-07	1.3012E-05	DOWN	0.27315	0.7659	0.88355	
transcript_HQ_CoA_transcript27946/f10p0/1568	48.36666667	109.74	51.88666667	1.3472	1.6215E-07	2.2106E-06	UP	-1.2394	2.8556E-06	4.7068E-05	DOWN	0.094411	0.60575	0.77715	
transcript_HQ_CoA_transcript27949/f3p0/1583	14.12666667	26.75666667	29.11666667	1.0974	0.0092514	0.033028	UP	-0.050715	0.89839	0.94587		1.035	1.1955E-06	0.00003688	UP
transcript_HQ_CoA_transcript27979/f8p0/1563	24.45666667	9.13	16.80333333	-1.2426	0.0087323	0.031506	DOWN	0.71655	0.14811	0.30204		-0.54155	0.13922	0.31763	
transcript_HQ_CoA_transcript27980/f4p0/1573	0	42.83333333	9.176666667	11.816	1.8088E-22	1.3122E-20	UP	-2.3544	0.014745	0.054367		9.4464	8.0423E-05	0.0011857	UP
transcript_HQ_CoA_transcript27984/f3p0/1585	0	133.0466667	10.27333333	13.492	4.2021E-26	3.9805E-24	UP	-3.8358	0.0085293	0.03545	DOWN	9.6407	0.00027068	0.003193	UP
transcript_HQ_CoA_transcript27991/f2p0/1616	21.44333333	38.49666667	23.88333333	1.0046	3.9391E-05	0.00031203	UP	-0.83654	1.2116E-05	0.00016034		0.15436	0.50652	0.7062	
transcript_HQ_CoA_transcript28016/f2p0/1563	19.42	63.07666667	26.4	1.863	1.8789E-18	1.0473E-16	UP	-1.3901	0.001017	0.0064758	DOWN	0.45977	0.27684	0.49049	
transcript_HQ_CoA_transcript28017/f3p0/1559	0.97	704.6966667	28.08333333	9.6514	1.321E-109	2.195E-106	UP	-4.7735	2.7737E-05	0.00032444	DOWN	4.8603	3.9521E-07	1.4252E-05	UP
transcript_HQ_CoA_transcript28024/f2p0/1583	38.29	70.02	40.3	1.0324	7.2056E-11	1.773E-09	UP	-0.95008	5.9271E-11	3.0094E-09		0.069453	0.63753	0.79876	
transcript_HQ_CoA_transcript28031/f2p0/1607	44.16	2.163333333	4.2	-4.1724	5.0992E-16	2.3482E-14	DOWN	0.77686	0.19793	0.36992		-3.4049	1.074E-22	6.7974E-20	DOWN
transcript_HQ_CoA_transcript28035/f2p0/1571	30.87666667	75.84666667	54.85	1.4663	9.735E-08	1.3893E-06	UP	-0.6275	0.0082959	0.034684		0.82498	0.00056186	0.005651	
transcript_HQ_CoA_transcript28040/f2p0/1578	26.51	147.7833333	60.76	2.6468	2.733E-25	2.4735E-23	UP	-1.4326	3.3922E-06	5.4113E-05	DOWN	1.2015	1.8348E-05	0.00035155	UP
transcript_HQ_CoA_transcript28049/f2p0/1602	10.85333333	32.56333333	14.77666667	1.7418	1.1802E-11	3.3143E-10	UP	-1.2844	5.1216E-06	7.6196E-05	DOWN	0.44566	0.1272	0.29978	
transcript_HQ_CoA_transcript28051/f3p0/1541	6.186666667	19.63333333	15.02	1.8575	0.00041791	0.0024239	UP	-0.53228	0.23517	0.41611		1.3092	0.025499	0.098886	
transcript_HQ_CoA_transcript28053/f3p0/1571	40.37	16.95333333	33.38	-1.0985	5.254E-09	9.5386E-08	DOWN	0.82194	0.0047071	0.022345		-0.28868	0.3118	0.52844	
transcript_HQ_CoA_transcript28056/f2p0/1578	17.59333333	2.966666667	11.22	-2.414	4.2065E-15	1.7843E-13	DOWN	1.7581	5.4714E-05	0.00058102	UP	-0.66911	0.063038	0.18714	
transcript_HQ_CoA_transcript28058/f6p0/1563	28.69	68.67666667	43.55666667	1.4145	1.3853E-06	1.5868E-05	UP	-0.81231	0.00087496	0.0057378		0.59039	0.0074807	0.040757	
transcript_HQ_CoA_transcript28059/f4p0/1491	13.06666667	164.52	26.91	3.8003	9.1341E-14	3.2808E-12	UP	-2.7603	3.7435E-17	5.7179E-15	DOWN	1.0311	0.054304	0.16975	
transcript_HQ_CoA_transcript28065/f2p0/1573	0.37	5.183333333	3.82	4.0664	9.5738E-05	0.00067683	UP	-0.6259	0.52549	0.6982		3.4232	0.00017719	0.0022601	UP
transcript_HQ_CoA_transcript2807/f2p0/4558	5.31	11.43	14.03666667	1.2826	0.00079729	0.0042225	UP	0.17074	0.75719	0.86358		1.4367	0.015212	0.068037	
transcript_HQ_CoA_transcript28073/f2p0/1582	1.076666667	38.2	0.703333333	5.2899	0.0063052	0.024005	UP	-5.8704	7.011E-08	1.7923E-06	DOWN	-0.59736	0.64865	0.80633	
transcript_HQ_CoA_transcript28099/f4p0/1541	18.63333333	36.71666667	19.69	1.1431	0.00034751	0.0020687	UP	-1.0606	7.7488E-05	0.00077739	DOWN	0.069929	0.77854	0.89158	
transcript_HQ_CoA_transcript28111/f2p0/1562	42.17333333	17.14666667	34.86	-1.1489	1.4789E-05	0.00013176	DOWN	0.87103	0.0027127	0.014214		-0.28985	0.35036	0.56774	
transcript_HQ_CoA_transcript28112/f2p0/1594	18.85666667	52.05666667	24.14	1.629	3.1928E-06	3.3546E-05	UP	-1.2629	1.7402E-05	0.0002161	DOWN	0.35216	0.18665	0.38348	
transcript_HQ_CoA_transcript28117/f10p0/1566	6.453333333	367.75	95.82666667	5.9869	2.9172E-46	7.5117E-44	UP	-2.0489	0.0045465	0.021761	DOWN	3.9222	3.0995E-08	1.563E-06	UP
transcript_HQ_CoA_transcript28123/f2p0/1570	24.23	3.883333333	13.69	-2.4718	4.4231E-10	9.7075E-09	DOWN	1.6566	4.5176E-06	6.8944E-05	UP	-0.82673	0.030842	0.11393	
transcript_HQ_CoA_transcript28125/f4p0/1562	5.923333333	13.8	6.38	1.3953	0.0036028	0.015191	UP	-1.2814	0.00038098	0.0029108	DOWN	0.10167	0.80969	0.91063	
transcript_HQ_CoA_transcript28131/f2p0/1583	10.23666667	66.1	16.7	2.8375	2.7943E-31	4.0545E-29	UP	-2.1267	5.9291E-20	1.4299E-17	DOWN	0.69865	0.0018689	0.014474	
transcript_HQ_CoA_transcript28140/f3p0/1561	24.10333333	46.92666667	57.86	1.1265	1.5774E-05	0.00013912	UP	0.14714	0.63288	0.78054		1.2579	0.00013522	0.0018183	UP
transcript_HQ_CoA_transcript28146/f2p0/1554	59.9	20.28666667	54.81666667	-1.4086	7.0472E-06	6.8897E-05	DOWN	1.294	1.0942E-05	0.00014682	UP	-0.12731	0.68224	0.83	
transcript_HQ_CoA_transcript28160/f2p0/1592	58.91666667	25.73333333	33.49666667	-1.0197	0.0038922	0.016198	DOWN	0.21928	0.58623	0.74772		-0.81509	0.0075544	0.041085	
transcript_HQ_CoA_transcript28164/f2p0/1565	28.03	1.576666667	20.33666667	-3.9189	0.0025604	0.011367	DOWN	3.4961	6.1324E-05	0.0006372	UP	-0.45133	0.80562	0.90808	
transcript_HQ_CoA_transcript28172/f6p0/1537	87.26666667	36.30333333	46.16	-1.1078	9.9007E-11	2.4102E-09	DOWN	0.19934	0.28575	0.47462		-0.92062	1.4574E-06	4.3426E-05	
transcript_HQ_CoA_transcript28174/f2p0/1561	3.283333333	0.526666667	2.756666667	-2.4711	0.0002411	0.0015079	DOWN	2.2505	0.00043648	0.0032574	UP	-0.23276	0.69651	0.83965	
transcript_HQ_CoA_transcript28179/f3p0/1557	76.55	32.74666667	89.86	-1.0789	5.1753E-05	0.00039637	DOWN	1.3147	3.9768E-06	6.1876E-05	UP	0.22708	0.4793	0.68394	
transcript_HQ_CoA_transcript28185/f2p0/1559	58.47666667	12.16666667	38.66666667	-2.0949	9.5104E-06	8.9948E-05	DOWN	1.5075	0.0025828	0.013676	UP	-0.59686	0.072051	0.20436	
transcript_HQ_CoA_transcript2819/f3p0/4556	8.896666667	2.82	3.703333333	-1.4782	0.00098998	0.0051069	DOWN	0.20909	0.71603	0.8374		-1.2803	0.0017811	0.014009	DOWN
transcript_HQ_CoA_transcript28191/f4p0/1555	20.97666667	71.1	29.59	1.9224	1.4293E-07	1.9721E-06	UP	-1.4249	4.3909E-05	0.00048366	DOWN	0.48585	0.013255	0.06164	
transcript_HQ_CoA_transcript28204/f2p0/1561	33.06333333	8.623333333	61.4	-1.7781	8.9588E-13	2.8886E-11	DOWN	2.6997	1.2357E-09	4.7283E-08	UP	0.90906	0.029853	0.11139	
transcript_HQ_CoA_transcript28208/f15p0/1512	357.71	133.7866667	148.2533333	-1.284	0.0035815	0.015125	DOWN	0.0040097	0.9844	0.9922		-1.2882	0.0016412	0.013109	DOWN
transcript_HQ_CoA_transcript28212/f4p0/1553	233.6133333	4.153333333	17.63	-5.5676	0.00042291	0.0024475	DOWN	1.9038	0.0033715	0.017071	UP	-3.6822	0.015	0.067322	
transcript_HQ_CoA_transcript28214/f2p0/1558	17.85	35.97666667	22.28333333	1.1726	1.1646E-05	0.000107	UP	-0.84634	0.00031271	0.00246		0.31404	0.12656	0.29891	
transcript_HQ_CoA_transcript28221/f3p0/1554	41.64666667	13.21	27.86333333	-1.5085	0.0015537	0.0074966	DOWN	0.93598	0.0079089	0.033455		-0.58546	0.19232	0.39079	
transcript_HQ_CoA_transcript28235/f2p0/1556	13.52	87.74333333	38.00333333	2.8633	7.8555E-25	6.8715E-23	UP	-1.3338	0.016272	0.058642		1.5153	0.0067097	0.037848	UP
transcript_HQ_CoA_transcript28236/f2p0/1534	3.866666667	19.53666667	11.45333333	2.5372	0.0037494	0.015707	UP	-0.87917	0.32603	0.51693		1.6359	0.14536	0.3266	
transcript_HQ_CoA_transcript28253/f2p0/1547	6.396666667	34.12333333	13.23333333	2.5831	6.781E-09	1.2085E-07	UP	-1.5242	0.0011153	0.0069816	DOWN	1.0464	0.022044	0.088969	
transcript_HQ_CoA_transcript28256/f4p0/1527	7.666666667	151.4666667	36.35333333	4.4654	6.3286E-45	1.5427E-42	UP	-2.1994	0.0016144	0.009364	DOWN	2.2512	0.00090009	0.0082276	UP
transcript_HQ_CoA_transcript28258/f4p0/1507	95.9	357.0633333	635.54	2.0803	1.8715E-10	4.3642E-09	UP	0.67305	0.26222	0.44759		2.7363	3.1684E-07	1.1796E-05	UP
transcript_HQ_CoA_transcript28261/f11p0/1524	2.74	329.5533333	18.57666667	7.1025	3.6344E-42	7.8168E-40	UP	-4.3027	1.597E-09	5.9737E-08	DOWN	2.7824	0.00050821	0.0052289	UP
transcript_HQ_CoA_transcript28262/f2p0/1551	20.90666667	8.01	11.67666667	-1.2357	0.0048056	0.019204	DOWN	0.38477	0.21303	0.3892		-0.864	0.050696	0.16155	
transcript_HQ_CoA_transcript28266/f2p0/1551	9.893333333	3.793333333	7.153333333	-1.2256	0.003325	0.01418	DOWN	0.74988	0.12923	0.27421		-0.48609	0.33893	0.55684	
transcript_HQ_CoA_transcript28270/f2p0/1560	2.12	15.84666667	5.033333333	3.0608	4.0915E-10	9.0449E-09	UP	-1.7848	3.898E-06	6.1013E-05	DOWN	1.266	0.04324	0.14467	
transcript_HQ_CoA_transcript28272/f8p0/1540	175.9	61.13	112.9133333	-1.3757	3.9338E-07	4.9632E-06	DOWN	0.72853	0.00097886	0.0062741		-0.6588	0.010482	0.052042	
transcript_HQ_CoA_transcript28281/f2p0/1542	81.20666667	29.42666667	13.67666667	-1.3132	1.6023E-11	4.4182E-10	DOWN	-1.2533	0.00029741	0.002364	DOWN	-2.5802	1.8322E-16	5.0955E-14	DOWN
transcript_HQ_CoA_transcript28296/f9p0/1517	352.53	146.9933333	233.9533333	-1.1054	1.0376E-05	9.6681E-05	DOWN	0.517	0.0012886	0.007821		-0.60029	0.007688	0.04157	
transcript_HQ_CoA_transcript28298/f3p0/1571	78.07	344.78	99.39666667	2.3092	5.5159E-11	1.389E-09	UP	-1.9401	1.1171E-06	2.0829E-05	DOWN	0.35676	0.38793	0.60372	
transcript_HQ_CoA_transcript28313/f4p0/1506	5.036666667	99.63	39.35	4.5037	4.1356E-23	3.1635E-21	UP	-1.4894	0.09069	0.21262		2.996	0.00045185	0.004783	UP
transcript_HQ_CoA_transcript28318/f3p0/1606	8.47	19.42666667	19.40666667	1.3497	1.0431E-08	1.7957E-07	UP	-0.12935	0.75407	0.8614		1.2065	0.0042098	0.02684	UP
transcript_HQ_CoA_transcript28320/f3p0/1543	0	113.3	12.53333333	13.205	6.161E-25	5.4944E-23	UP	-3.3221	0.0086486	0.03584	DOWN	9.8666	2.2177E-05	0.00040951	UP
transcript_HQ_CoA_transcript28328/f2p0/1545	12.72666667	2.443333333	5.773333333	-2.2358	2.7074E-08	4.357E-07	DOWN	1.0965	0.0091246	0.037319	UP	-1.1523	4.3646E-05	0.00071465	DOWN
transcript_HQ_CoA_transcript28361/f3p0/1534	20.08333333	6.56	11.21333333	-1.467	3.3649E-05	0.0002726	DOWN	0.6486	0.092257	0.2153		-0.83247	0.023774	0.093825	
transcript_HQ_CoA_transcript28377/f10p0/1492	125.7033333	342.75	143.4466667	1.6052	4.0189E-13	1.3481E-11	UP	-1.405	1.485E-07	3.5302E-06	DOWN	0.18781	0.48058	0.68492	
transcript_HQ_CoA_transcript28379/f2p0/1542	5.673333333	43.18	17.33333333	3.0841	4.7263E-14	1.7706E-12	UP	-1.4529	6.1012E-05	0.00063467	DOWN	1.6165	1.0424E-06	3.2707E-05	UP
transcript_HQ_CoA_transcript2838/f3p0/4494	14.41	48.33666667	13.20666667	1.9023	1.0777E-07	1.5238E-06	UP	-2.0226	2.3995E-09	8.573E-08	DOWN	-0.13168	0.72066	0.85566	
transcript_HQ_CoA_transcript28380/f32p0/1470	301.6066667	101.53	197.4566667	-1.3931	5.4123E-06	5.4248E-05	DOWN	0.78758	0.010928	0.043029		-0.61836	8.7275E-06	0.00018961	
transcript_HQ_CoA_transcript28384/f3p0/1564	8.746666667	27.41333333	7.61	1.824	1.9844E-07	2.6676E-06	UP	-1.9919	2.5586E-05	0.00030178	DOWN	-0.18428	0.74025	0.868	
transcript_HQ_CoA_transcript28388/f2p0/1529	12.97666667	31.44	20.51666667	1.4487	3.2712E-07	4.1997E-06	UP	-0.75766	0.18758	0.35629		0.67557	0.2245	0.43157	
transcript_HQ_CoA_transcript28392/f2p0/1527	11.77666667	29.13333333	29.77333333	1.4471	4.2526E-06	4.3458E-05	UP	-0.099682	0.68584	0.81783		1.3361	2.4345E-07	9.4674E-06	UP
transcript_HQ_CoA_transcript28393/f3p0/1539	66.98	23.79	68.74	-1.382	0.012459	0.042055	DOWN	1.4126	0.0097383	0.039204	UP	0.021856	0.9394	0.97308	
transcript_HQ_CoA_transcript28396/f2p0/1531	21.6	56.39666667	32.01666667	1.5452	1.2559E-08	2.1299E-07	UP	-0.96515	0.00018941	0.0016453		0.56759	0.045552	0.15042	
transcript_HQ_CoA_transcript28405/f2p0/1545	26.28333333	61.22666667	39.83666667	1.3802	3.1923E-05	0.00026089	UP	-0.78102	0.0062104	0.027784		0.5871	0.012148	0.057901	

transcript_HQ_CoA_transcript28410/f3p0/1518	12.68	73.07333333	13.94666667	2.696	3.4006E-16	1.5819E-14	UP	-2.5483	2.1863E-14	2.2898E-12	DOWN	0.13509	0.64247	0.80145	
transcript_HQ_CoA_transcript28419/f2p0/1532	49.91333333	148.2533333	80.56	1.7331	2.0737E-07	2.7753E-06	UP	-1.0452	0.00033929	0.002634	DOWN	0.67773	0.013678	0.062924	
transcript_HQ_CoA_transcript28437/f3p0/1530	32.88333333	12.55666667	24.29333333	-1.2354	2.9081E-05	0.00023981	DOWN	0.81127	7.4821E-05	0.00075536		-0.43697	0.0868	0.23245	
transcript_HQ_CoA_transcript28444/f2p0/1545	18.97	34.09	18.54	1.0267	0.013935	0.045931	UP	-1.0499	0.0099931	0.040001	DOWN	-0.036552	0.85802	0.93571	
transcript_HQ_CoA_transcript28447/f6p0/1449	64.07333333	141.3	72.31	1.3115	0.00033778	0.0020194	UP	-1.1361	0.0014978	0.0088188	DOWN	0.16321	0.64322	0.802	
transcript_HQ_CoA_transcript28458/f2p0/1527	2655.67	999.9733333	1930.51	-1.2715	6.3594E-06	6.2845E-05	DOWN	0.82024	0.005098	0.023825		-0.46069	0.10178	0.26017	
transcript_HQ_CoA_transcript2846/f2p0/4517	0.983333333	0.096666667	0.39	-3.162	1.4039E-05	0.00012588	DOWN	1.8262	0.046329	0.12868		-1.3461	0.046998	0.15358	
transcript_HQ_CoA_transcript28466/f2p0/1526	20.28333333	8.753333333	27.82666667	-1.0675	0.0029819	0.012921	DOWN	1.5319	0.00023848	0.0019752	UP	0.45286	0.20913	0.41247	
transcript_HQ_CoA_transcript28467/f5p0/1508	312.6666667	71.80333333	214.44	-1.9707	2.2474E-15	9.6904E-14	DOWN	1.43	3.2588E-06	5.2278E-05	UP	-0.55254	0.065898	0.19307	
transcript_HQ_CoA_transcript28477/f11p0/1511	212.5233333	52.10333333	209.08	-1.8674	1.2925E-09	2.6225E-08	DOWN	1.8524	2.9035E-14	2.9081E-12	UP	-0.025493	0.9262	0.96725	
transcript_HQ_CoA_transcript28481/f5p0/1493	145.1	798.8766667	563.3566667	2.6323	5.9039E-27	6.0299E-25	UP	-0.65906	0.10714	0.23993		1.9576	1.1184E-07	4.774E-06	UP
transcript_HQ_CoA_transcript28485/f2p0/1519	10.67666667	31.39333333	18.61	1.7345	3.5621E-07	4.5476E-06	UP	-0.91521	0.0010374	0.0065818		0.80591	0.0035722	0.023748	
transcript_HQ_CoA_transcript28497/f2p0/1541	14.28	49.16	26.77666667	1.9461	2.5326E-13	8.7031E-12	UP	-1.0215	0.0014046	0.0083832	DOWN	0.91148	0.0038565	0.025183	
transcript_HQ_CoA_transcript28502/f2p0/1518	2.023333333	518.2866667	315.6766667	8.1502	9.9396E-84	1.0689E-80	UP	-0.84759	0.48211	0.66416		7.2863	1.0167E-09	7.2049E-08	UP
transcript_HQ_CoA_transcript28522/f8p0/1499	204.1666667	42.64666667	198.3566667	-2.0583	0.0016831	0.0080285	DOWN	2.0787	8.9807E-08	2.2396E-06	UP	0.0036102	0.99686	0.99832	
transcript_HQ_CoA_transcript28536/f2p0/1508	32.78666667	14.42666667	17.76666667	-1.0441	0.00037817	0.0022274	DOWN	0.16013	0.62412	0.77481		-0.89481	0.00115	0.0099799	
transcript_HQ_CoA_transcript28540/f2p0/1513	25.89333333	56.10666667	40.88666667	1.2716	1.0771E-12	3.4367E-11	UP	-0.60875	6.3435E-05	0.00065505		0.65055	0.00056974	0.0057103	
transcript_HQ_CoA_transcript28544/f4p0/1506	6.073333333	2.053333333	3.286666667	-1.4099	0.00043346	0.0024983	DOWN	0.53018	0.1679	0.32953		-0.89103	0.016015	0.070537	
transcript_HQ_CoA_transcript28547/f2p0/1514	39.37666667	16.23	19.81333333	-1.1157	8.1902E-06	7.8769E-05	DOWN	0.1381	0.48648	0.66767		-0.99135	5.4101E-06	0.00012797	
transcript_HQ_CoA_transcript28558/f6p0/1492	18.19666667	98.62	59.55666667	2.6076	2.9341E-11	7.7628E-10	UP	-0.86661	0.0079616	0.033624		1.7252	0.00009958	0.0014136	UP
transcript_HQ_CoA_transcript28561/f3p0/1509	10.72	2.563333333	8.866666667	-1.8818	0.00013485	0.00091413	DOWN	1.6074	0.0013012	0.007879	UP	-0.28722	0.42933	0.64131	
transcript_HQ_CoA_transcript28562/f2p0/1510	50.95333333	105.7466667	77.03666667	1.2134	3.9032E-17	1.9877E-15	UP	-0.60874	1.4575E-05	0.00018617		0.59252	5.7623E-05	0.00090168	
transcript_HQ_CoA_transcript28568/f2p0/1507	64.75666667	17.06	48.41333333	-1.778	1.0963E-09	2.2647E-08	DOWN	1.3509	1.4553E-07	3.4777E-06	UP	-0.43747	0.16039	0.34808	
transcript_HQ_CoA_transcript28573/f2p0/1500	2.216666667	0.526666667	1.81	-1.8966	0.0050107	0.019897	DOWN	1.6243	0.027959	0.088633		-0.28391	0.63751	0.79876	
transcript_HQ_CoA_transcript28577/f2p0/1503	0.053333333	1.053333333	1.813333333	4.5089	0.0024394	0.010928	UP	0.64566	0.44432	0.63269		5.1415	0.00032977	0.0037112	UP
transcript_HQ_CoA_transcript28583/f2p0/1506	58.65333333	18.47666667	40.50333333	-1.5023	1.0842E-12	3.4532E-11	DOWN	0.97192	0.00011343	0.0010668		-0.54219	0.013593	0.062671	
transcript_HQ_CoA_transcript28584/f2p0/1505	47.97666667	10.92333333	44.74	-1.9519	2.0836E-06	2.2878E-05	DOWN	1.861	4.0356E-06	6.2632E-05	UP	-0.10553	0.78989	0.89873	
transcript_HQ_CoA_transcript28586/f2p0/1514	41.42666667	4.136666667	18.10666667	-3.1824	1.2506E-20	8.2242E-19	DOWN	1.9766	4.28E-07	9.1539E-06	UP	-1.2163	0.0010589	0.0093949	DOWN
transcript_HQ_CoA_transcript28596/f2p0/1500	11.65333333	23.44666667	9.083333333	1.15	0.0020381	0.0094005	UP	-1.5086	5.7158E-05	0.00060348	DOWN	-0.37033	0.18625	0.38313	
transcript_HQ_CoA_transcript28597/f2p0/1501	30.63333333	360.4866667	41.59333333	3.7285	1.1707E-42	2.6101E-40	UP	-3.2835	3.965E-22	1.3712E-19	DOWN	0.4304	0.14248	0.32236	
transcript_HQ_CoA_transcript28598/f2p0/1514	0.976666667	111.51	16.02	7.0454	7.6199E-16	3.4397E-14	UP	-2.9643	1.1127E-07	2.7193E-06	DOWN	4.0602	3.6584E-07	1.3297E-05	UP
transcript_HQ_CoA_transcript28599/f5p0/1487	28.31666667	56.9	35.31	1.1651	0.00045046	0.0025808	UP	-0.84304	0.00045551	0.0033775		0.31	0.26053	0.47164	
transcript_HQ_CoA_transcript2861/f4p0/4509	55.26333333	16.20666667	17.44	-1.6268	8.3967E-06	8.0667E-05	DOWN	-0.046888	0.78046	0.87712		-1.683	1.3294E-06	0.0000402	DOWN
transcript_HQ_CoA_transcript28615/f4p0/1493	0	239.1633333	36.97333333	14.234	3.6389E-27	3.8015E-25	UP	-2.8414	0.16733	0.32875		11.376	0.0035944	0.023837	UP
transcript_HQ_CoA_transcript28618/f5p0/1459	10.29666667	158.2533333	46.19666667	4.1119	5.7735E-24	4.7121E-22	UP	-1.8987	0.0016448	0.009507	DOWN	2.1982	0.00027159	0.0032017	UP
transcript_HQ_CoA_transcript28620/f2p0/1501	16.24333333	7.016666667	9.506666667	-1.0638	0.0056255	0.02184	DOWN	0.31219	0.40948	0.59975		-0.7661	0.015937	0.070371	
transcript_HQ_CoA_transcript28631/f2p0/1497	9.21	54.95333333	11.88666667	2.7507	2.8237E-11	7.5253E-10	UP	-2.3769	2.3749E-11	1.2955E-09	DOWN	0.36103	0.33124	0.54826	
transcript_HQ_CoA_transcript28641/f3p0/1563	0.673333333	226.6366667	5.266666667	8.5597	2.3968E-93	3.6516E-90	UP	-5.5857	4.1228E-22	1.3994E-19	DOWN	2.9579	0.00012431	0.0017003	UP
transcript_HQ_CoA_transcript28645/f2p0/1498	27.35666667	59.54666667	27.89333333	1.2839	0.00036405	0.0021574	UP	-1.2493	0.00028453	0.0022824	DOWN	0.022443	0.93247	0.97032	
transcript_HQ_CoA_transcript28650/f3p0/1501	0.05	144.3966667	7.686666667	11.638	5.2998E-26	4.9687E-24	UP	-4.3645	0.014169	0.052731		7.2439	0.019217	0.080918	
transcript_HQ_CoA_transcript28654/f7p0/1490	0.1	5.923333333	61.49666667	6.0356	3.4842E-08	5.4818E-07	UP	3.2501	0.13987	0.29024		9.2678	0.00095232	0.0085938	UP
transcript_HQ_CoA_transcript28673/f2p0/1488	39.33	100.0266667	127.2	1.5007	8.759E-09	1.528E-07	UP	0.2245	0.66676	0.80529		1.7132	0.00085115	0.0078823	UP
transcript_HQ_CoA_transcript28702/f2p0/1487	268.8	76.82	85.92666667	-1.6263	0.00023582	0.0014815	DOWN	-0.018246	0.96802	0.98331		-1.6582	2.4339E-10	1.9681E-08	DOWN
transcript_HQ_CoA_transcript28706/f2p0/1487	16.53666667	7.253333333	13.89333333	-1.035	0.0049682	0.019763	DOWN	0.7807	0.00029814	0.0023687		-0.26524	0.42231	0.63438	
transcript_HQ_CoA_transcript28712/f2p0/1483	2.376666667	32.84666667	4.073333333	3.9666	1.2617E-12	3.9632E-11	UP	-3.1683	1.8127E-08	5.316E-07	DOWN	0.78188	0.14224	0.32202	
transcript_HQ_CoA_transcript28718/f2p0/1488	18.41666667	72.23666667	22.70333333	2.1437	2.8813E-09	5.487E-08	UP	-1.8324	1.2234E-08	3.7624E-07	DOWN	0.2987	0.24096	0.44988	
transcript_HQ_CoA_transcript28721/f2p0/1515	36.03	133.8866667	65.21333333	2.0562	1.4734E-28	1.7839E-26	UP	-1.1772	2.4116E-06	4.0665E-05	DOWN	0.86584	0.0013042	0.011062	
transcript_HQ_CoA_transcript28726/f2p0/1480	13.15666667	47.06333333	17.11666667	2.007	0.011213	0.038656	UP	-1.5853	0.013111	0.049694	DOWN	0.40696	0.51592	0.71277	
transcript_HQ_CoA_transcript28729/f4p0/1429	68.90666667	25.70666667	70.45	-1.2645	1.1651E-09	2.3853E-08	DOWN	1.3004	6.4987E-08	1.6992E-06	UP	0.022215	0.92529	0.96673	
transcript_HQ_CoA_transcript28731/f9p0/1455	2.476666667	67.61666667	7.573333333	4.9202	1.2218E-22	9.0068E-21	UP	-3.3081	4.2792E-11	2.2219E-09	DOWN	1.601	0.013677	0.062924	
transcript_HQ_CoA_transcript28733/f4p0/1478	66.48333333	22.44666667	35.14333333	-1.4204	3.9645E-09	7.3807E-08	DOWN	0.50127	0.032601	0.099146		-0.93072	1.553E-06	4.5535E-05	
transcript_HQ_CoA_transcript28736/f3p0/1518	11.55666667	65.09	21.71	2.6577	3.3523E-12	1.0031E-10	UP	-1.7393	1.5263E-06	2.7374E-05	DOWN	0.90574	3.6464E-05	0.00061914	
transcript_HQ_CoA_transcript28737/f177p0/1428	1977.55	582.6366667	1712.856667	-1.6107	4.5487E-07	5.6726E-06	DOWN	1.4054	1.4004E-07	3.3598E-06	UP	-0.21499	0.47954	0.68407	
transcript_HQ_CoA_transcript28738/f3p0/1494	27.17666667	57.92	41.80333333	1.2416	1.2279E-08	2.0862E-07	UP	-0.61005	0.0021216	0.011625		0.61918	0.00012653	0.0017267	
transcript_HQ_CoA_transcript28749/f3p0/1450	37.31333333	68.60666667	76.42333333	1.0432	2.0432E-06	2.2496E-05	UP	0.0073835	0.96314	0.98009		1.0376	9.4032E-06	0.0002	UP
transcript_HQ_CoA_transcript28756/f2p0/1491	0	6.843333333	235.3866667	9.055	3.6591E-10	8.1779E-09	UP	5.0289	0.011866	0.045932	UP	14.07	3.8169E-05	0.00064118	UP
transcript_HQ_CoA_transcript2876/f4p0/4471	1.253333333	3.636666667	1.473333333	1.6871	0.00026463	0.0016345	UP	-1.4538	0.00084657	0.0055796	DOWN	0.22042	0.54244	0.7328	
transcript_HQ_CoA_transcript28767/f3p0/1463	5.326666667	1.81	3.403333333	-1.4171	0.0028783	0.012574	DOWN	0.77098	0.063732	0.16443		-0.65652	0.12678	0.29917	
transcript_HQ_CoA_transcript2877/f2p0/4560	0.886666667	3.316666667	4.69	2.0173	0.0024703	0.011047	UP	0.36365	0.57056	0.73522		2.3724	6.0479E-06	0.0001407	UP
transcript_HQ_CoA_transcript28770/f3p0/1475	52.53666667	161.67	94.53	1.7843	8.9503E-09	1.5599E-07	UP	-0.92458	0.00041073	0.0031006		0.84705	0.00042827	0.0045676	
transcript_HQ_CoA_transcript28771/f11p0/1408	99.92333333	255.0066667	255.1833333	1.5006	2.4908E-08	4.0334E-07	UP	-0.12289	0.69161	0.82184		1.3658	5.1502E-05	0.00081987	UP
transcript_HQ_CoA_transcript28786/f5p0/1468	28.86333333	53.78	30.99666667	1.0654	0.0016748	0.0079964	UP	-0.94475	0.00026667	0.0021647		0.10593	0.70262	0.84318	
transcript_HQ_CoA_transcript28796/f15p0/1453	73.43	24.04	56.18333333	-1.4671	3.1823E-06	3.3455E-05	DOWN	1.0792	1.1602E-08	3.6023E-07	UP	-0.39764	0.18714	0.38418	
transcript_HQ_CoA_transcript28811/f2p0/1466	10.39666667	176.88	40.97666667	4.2544	2.653E-17	1.3701E-15	UP	-2.2599	1.7987E-06	3.1458E-05	DOWN	1.9807	1.432E-14	3.0564E-12	UP
transcript_HQ_CoA_transcript28822/f3p0/1512	135.1266667	43.95	97.07666667	-1.4807	7.9473E-07	9.5025E-06	DOWN	1.0056	0.0027406	0.01432	UP	-0.48637	0.069858	0.20051	
transcript_HQ_CoA_transcript28832/f2p0/1479	38.41666667	91.77666667	61.96666667	1.4172	1.2446E-09	2.5309E-08	UP	-0.70656	0.010727	0.042401		0.69796	0.015959	0.070425	
transcript_HQ_CoA_transcript28835/f2p0/1461	46.44666667	12.74666667	33.49333333	-1.7045	1.7293E-06	1.9348E-05	DOWN	1.2336	3.4657E-05	0.00039646	UP	-0.48252	0.1822	0.37784	
transcript_HQ_CoA_transcript28836/f2p0/1477	27.78666667	105.28	46.48333333	2.0854	4.3695E-15	1.8491E-13	UP	-1.3294	2.3358E-07	5.3317E-06	DOWN	0.74195	0.00087823	0.0080697	
transcript_HQ_CoA_transcript28842/f2p0/1469	0	8.57	35.35333333	9.3797	2.4035E-12	7.3235E-11	UP	1.9208	0.34041	0.53218		11.285	0.0038702	0.025252	UP
transcript_HQ_CoA_transcript2885/f2p0/4521	4.006666667	12	6.333333333	1.7473	1.5944E-07	2.1801E-06	UP	-1.0873	0.00096673	0.0062107	DOWN	0.64897	0.047336	0.15435	
transcript_HQ_CoA_transcript28853/f44p0/1405	1563.083333	627.3966667	1409.136667	-1.1767	0.0015514	0.0074876	DOWN	1.033	5.6102E-05	0.00059473	UP	-0.15265	0.67809	0.82689	
transcript_HQ_CoA_transcript28861/f2p0/1463	12.76	42.27666667	21.22	1.8946	9.2377E-05	0.0006579	UP	-1.1569	0.00022587	0.0018895	DOWN	0.7217	0.17348	0.36597	
transcript_HQ_CoA_transcript28863/f5p0/1461	47.68666667	117.4333333	110.6333333	1.443	4.2478E-07	5.3337E-06	UP	-0.21317	0.40145	0.592		1.219	9.0296E-06	0.00019362	UP
transcript_HQ_CoA_transcript28874/f2p0/1490	14.99	4.736666667	13.73666667	-1.5223	0.0029114	0.012676	DOWN	1.3843	0.0011464	0.0071282	UP	-0.14843	0.7798	0.89267	
transcript_HQ_CoA_transcript28876/f2p0/1006	9.353333333	52.06333333	16.69	2.6465	1.303E-08	2.1975E-07	UP	-1.7929	0.000181	0.0015828	DOWN	0.83911	0.016396	0.071664	
transcript_HQ_CoA_transcript28880/f7p0/1428	72.63	198.69	79.39666667	1.6192	2.8675E-07	3.7259E-06	UP	-1.4803	1.6708E-05	0.00020955	DOWN	0.1257	0.59828	0.77132	
transcript_HQ_CoA_transcript28881/f2p0/1455	1167.053333	521.3333333	736.1433333	-1.0058	0.00019833	0.0012767	DOWN	0.33707	0.13342	0.28025		-0.67967	0.0086603	0.045404	
transcript_HQ_CoA_transcript28882/f2p0/1458	1.843333333	131.88	14.1	6.3922	0.0092225	0.032949	UP	-3.3746	4.7786E-07	1.0137E-05	DOWN	2.9913	0.25164	0.46201	
transcript_HQ_CoA_transcript28884/f3p0/1468	27.61333333	134.1933333	63.45333333	2.4524	1.5639E-14	6.2564E-13	UP	-1.224	0.00018977	0.0016477	DOWN	1.2149	0.00088957	0.0081559	UP

transcript_HQ_CoA_transcript28886/f4p0/1447	18.78	71.58666667	35.64333333	2.0919	1.1272E-26	1.1139E-24	UP	-1.1582	2.975E-11	1.5897E-09	DOWN	0.9206	0.00011478	0.0015948	
transcript_HQ_CoA_transcript28890/f2p0/1428	47.59	3.47	22.66333333	-3.6013	9.7875E-14	3.5017E-12	DOWN	2.5328	3.7804E-08	1.0357E-06	UP	-1.0833	0.034426	0.12305	
transcript_HQ_CoA_transcript28893/f4p0/1455	0.36	51.76666667	236.42	7.3083	9.5087E-07	1.1244E-05	UP	2.0523	0.31661	0.50766		9.3473	1.3458E-06	4.0629E-05	UP
transcript_HQ_CoA_transcript28900/f2p0/1332	65.07333333	151.46	104.3533333	1.3979	9.5464E-06	9.0242E-05	UP	-0.68946	0.056905	0.15105		0.69307	0.047276	0.1542	
transcript_HQ_CoA_transcript28906/f2p0/1457	25.94666667	69.67666667	45.79333333	1.579	2.0455E-08	3.3479E-07	UP	-0.75285	0.0011627	0.0072045		0.81413	0.00024545	0.002937	
transcript_HQ_CoA_transcript2892/f3p0/4496	3.93	1.306666667	4.413333333	-1.4476	0.00075984	0.004043	DOWN	1.6171	4.8303E-05	0.00052636	UP	0.1569	0.59327	0.76832	
transcript_HQ_CoA_transcript28924/f2p0/1476	21.39666667	57.78666667	20.38333333	1.5973	3.2716E-05	0.00026631	UP	-1.6564	3.9016E-05	0.00043738	DOWN	-0.071431	0.83452	0.92324	
transcript_HQ_CoA_transcript28926/f2p0/1449	8.866666667	16.66	4.5	1.0779	0.00070586	0.0037877	UP	-2.0282	9.0776E-05	0.00089032	DOWN	-0.96076	0.086092	0.23103	
transcript_HQ_CoA_transcript28944/f7p0/1442	3.513333333	338.3066667	36.47666667	6.7399	2.3912E-56	7.9484E-54	UP	-3.3482	1.8279E-05	0.00022577	DOWN	3.3746	6.6249E-06	0.00015106	UP
transcript_HQ_CoA_transcript28945/f2p0/1475	10.54333333	26.01	22.95	1.4532	1.1368E-12	3.6018E-11	UP	-0.32365	0.16128	0.32118		1.1167	1.5073E-05	0.00029845	UP
transcript_HQ_CoA_transcript28948/f5p0/1405	112.74	46.83333333	67.88333333	-1.1368	0.0057128	0.022104	DOWN	0.40915	0.34649	0.53857		-0.73934	0.0032259	0.021946	
transcript_HQ_CoA_transcript28958/f2p0/1450	11.80333333	22.10666667	15.02666667	1.0772	0.00013881	0.00093817	UP	-0.72116	0.0063787	0.02833		0.34304	0.16203	0.35029	
transcript_HQ_CoA_transcript28960/f2p0/1443	18.31	34.03333333	43.40666667	1.0559	0.00024447	0.0015269	UP	0.19371	0.5944	0.75358		1.238	0.0019855	0.015191	UP
transcript_HQ_CoA_transcript28969/f3p0/1438	0.053333333	257.4833333	25.57666667	12.411	9.4833E-28	1.0769E-25	UP	-3.4801	0.077935	0.19056		8.9	0.0068014	0.038131	UP
transcript_HQ_CoA_transcript28972/f3p0/1447	102.7433333	9.053333333	57.66333333	-3.2823	8.4245E-06	8.0891E-05	DOWN	2.4977	0.00015951	0.0014199	UP	-0.799	0.21868	0.42445	
transcript_HQ_CoA_transcript28973/f2p0/1439	3.7	19.24666667	7.386666667	2.5573	0.00028524	0.0017441	UP	-1.5327	0.0415	0.11846		1.0133	0.21033	0.41393	
transcript_HQ_CoA_transcript28979/f2p0/1445	36.22333333	84.48666667	38.81666667	1.3858	0.00010868	0.00075604	UP	-1.2795	0.00020827	0.0017804	DOWN	0.093631	0.61689	0.78441	
transcript_HQ_CoA_transcript28994/f5p0/1436	0.21	5559.383333	265.3266667	14.831	1.46E-35	2.5182E-33	UP	-4.545	0.04275	0.12105		10.271	0.0005373	0.0054759	UP
transcript_HQ_CoA_transcript28995/f2p0/1435	20.08666667	49.86	29.05	1.4815	2.9975E-06	3.1658E-05	UP	-0.93492	0.0014162	0.0084359		0.53272	0.086498	0.23175	
transcript_HQ_CoA_transcript28996/f3p0/1497	12.33333333	23.67666667	17.98	1.0874	0.0031901	0.013674	UP	-0.53506	0.080429	0.19461		0.54098	0.059841	0.18103	
transcript_HQ_CoA_transcript29003/f3p0/1418	7.526666667	18.19333333	39.18666667	1.4665	0.0056886	0.022034	UP	0.92409	0.051443	0.13965		2.3776	5.9188E-11	5.6E-09	UP
transcript_HQ_CoA_transcript29006/f2p0/1442	90.94666667	39.08333333	92.24	-1.0614	0.00022339	0.0014143	DOWN	1.0812	0.0029305	0.015156	UP	0.0083264	0.98108	0.99259	
transcript_HQ_CoA_transcript29011/f21p0/1362	676.63	271.58	398.7366667	-1.1893	0.00058222	0.0032128	DOWN	0.43187	0.25686	0.44102		-0.7665	0.0025392	0.018263	
transcript_HQ_CoA_transcript2902/f2p0/4480	1.956666667	4.793333333	2.393333333	1.4295	0.00029571	0.0017972	UP	-1.144	5.2331E-05	0.00055994	DOWN	0.27537	0.47141	0.67774	
transcript_HQ_CoA_transcript29020/f2p0/1430	1.71	28.57	8.06	4.2028	3.0275E-07	3.9199E-06	UP	-1.9563	0.012852	0.048902	DOWN	2.2395	0.00037566	0.0041264	UP
transcript_HQ_CoA_transcript29022/f6p0/1435	91.57	38.47333333	55.69666667	-1.09	2.5744E-11	6.9315E-10	DOWN	0.38336	0.092902	0.21639		-0.71776	0.00094547	0.0085446	
transcript_HQ_CoA_transcript29023/f2p0/1421	164.7033333	60.88333333	95.4	-1.2977	0.00075276	0.004011	DOWN	0.50338	0.042939	0.12144		-0.80379	0.034932	0.12433	
transcript_HQ_CoA_transcript29024/f13p0/1254	69.25333333	170.2433333	104.8333333	1.4676	3.0488E-06	3.2145E-05	UP	-0.8615	0.0024359	0.013025		0.59407	0.0029104	0.020297	
transcript_HQ_CoA_transcript29028/f2p0/1437	105.2166667	29.46666667	48.01666667	-1.6595	0.0072907	0.027086	DOWN	0.55489	0.24431	0.42712		-1.1208	0.016407	0.071687	
transcript_HQ_CoA_transcript29033/f2p0/1456	64.18666667	116.9033333	88.30666667	1.0222	8.9439E-06	8.5296E-05	UP	-0.55503	2.1729E-05	0.00026254		0.45512	0.03226	0.11742	
transcript_HQ_CoA_transcript29036/f2p0/1428	63.97333333	25.64666667	29.23666667	-1.157	1.5182E-06	1.7218E-05	DOWN	0.031785	0.89042	0.94108		-1.1372	1.003E-11	1.1364E-09	DOWN
transcript_HQ_CoA_transcript29041/f3p0/1426	12.67333333	34.37666667	26.45666667	1.5995	6.2259E-10	1.3344E-08	UP	-0.52616	0.018413	0.06442		1.0604	8.0135E-08	3.6051E-06	UP
transcript_HQ_CoA_transcript29048/f5p0/1423	188.87	29.88	105.6466667	-2.5049	2.9599E-10	6.6807E-09	DOWN	1.6831	2.6818E-06	4.4323E-05	UP	-0.83546	0.034531	0.12324	
transcript_HQ_CoA_transcript29051/f8p0/1401	0.516666667	174.6566667	248.4066667	8.5413	1.0369E-69	7.0212E-67	UP	0.4121	0.8055	0.89234		8.9375	4.0136E-07	1.4417E-05	UP
transcript_HQ_CoA_transcript2907/f5p0/4442	12.08666667	40.9	16.9	1.9092	9.8045E-06	9.1921E-05	UP	-1.4393	1.0329E-05	0.00014003	DOWN	0.45974	0.19727	0.39703	
transcript_HQ_CoA_transcript29070/f3p0/1419	26.69333333	108.6	37.96666667	2.1815	4.4201E-09	8.1378E-08	UP	-1.6665	1.2011E-06	2.2148E-05	DOWN	0.50253	0.06813	0.19718	
transcript_HQ_CoA_transcript29076/f2p0/1414	9.316666667	3.17	4.716666667	-1.3755	0.014982	0.048505	DOWN	0.39527	0.4564	0.64291		-0.99287	0.013661	0.062889	
transcript_HQ_CoA_transcript29077/f2p0/1418	0.243333333	247.71	21.48333333	10.171	3.2039E-47	8.6138E-45	UP	-3.6873	0.0077871	0.033047	DOWN	6.4648	5.5046E-08	2.5775E-06	UP
transcript_HQ_CoA_transcript29084/f25p0/1388	384.5533333	840.2966667	1004.67	1.3043	0.00042893	0.0024769	UP	0.11612	0.61925	0.77176		1.4043	0.00025891	0.003074	UP
transcript_HQ_CoA_transcript29091/f2p0/1445	131.7666667	43.54666667	80.27333333	-1.441	3.4076E-19	1.9967E-17	DOWN	0.72674	0.00006815	0.00069705		-0.72634	0.00016549	0.0021316	
transcript_HQ_CoA_transcript29097/f2p0/1418	18.84	94.87666667	30.92666667	2.5023	8.4767E-27	8.4224E-25	UP	-1.7562	3.0405E-06	4.9449E-05	DOWN	0.73262	0.054492	0.17001	
transcript_HQ_CoA_transcript29109/f2p0/1398	38.22666667	6.203333333	15.79666667	-2.4558	3.4532E-06	3.5993E-05	DOWN	1.2011	0.0000588	0.00061515	UP	-1.2679	0.0063467	0.036439	DOWN
transcript_HQ_CoA_transcript29112/f3p0/1426	31.5	10.44	33.09	-1.4387	4.7441E-07	5.8988E-06	DOWN	1.5241	1.9035E-08	5.5467E-07	UP	0.071724	0.80377	0.90694	
transcript_HQ_CoA_transcript29113/f2p0/1422	2.806666667	28.59333333	7.293333333	3.5301	4.1719E-11	1.0712E-09	UP	-2.1308	1.4796E-05	0.00018846	DOWN	1.3837	0.0088215	0.046013	UP
transcript_HQ_CoA_transcript29117/f2p0/1406	258.9166667	68.76	259.21	-1.6905	0.007315	0.027148	DOWN	1.7452	0.003855	0.019076	UP	0.039867	0.94385	0.97485	
transcript_HQ_CoA_transcript29118/f2p0/1418	98.34666667	20.82666667	54.06333333	-2.0394	8.8381E-05	0.00063314	DOWN	1.2003	0.016567	0.059554		-0.85434	0.0021898	0.016392	
transcript_HQ_CoA_transcript2912/f2p0/4526	9.89	3.196666667	6.61	-1.4404	0.0070396	0.026334	DOWN	0.87091	0.083924	0.20068		-0.58379	0.036091	0.12736	
transcript_HQ_CoA_transcript29128/f2p0/1406	4.783333333	25.98333333	5.673333333	2.6039	6.3275E-07	7.6965E-06	UP	-2.3544	3.3009E-06	5.2794E-05	DOWN	0.23821	0.52346	0.71772	
transcript_HQ_CoA_transcript29129/f2p0/1409	57.84333333	22.02333333	30.94333333	-1.2437	5.1161E-10	1.1108E-08	DOWN	0.34433	0.070745	0.17746		-0.91045	1.1389E-05	0.00023514	
transcript_HQ_CoA_transcript29164/f4p0/1394	6.42	64.1	18.82	3.4765	1.0268E-47	2.8441E-45	UP	-1.8898	1.4966E-06	2.6893E-05	DOWN	1.575	0.00045301	0.0047925	UP
transcript_HQ_CoA_transcript29174/f3p0/1417	12.26666667	4.893333333	7.39	-1.1508	0.0010149	0.0052143	DOWN	0.42762	0.21661	0.39341		-0.73551	0.0053604	0.032133	
transcript_HQ_CoA_transcript29178/f2p0/1438	14.98666667	42.20666667	30.05666667	1.654	3.786E-06	3.9083E-05	UP	-0.64318	0.047786	0.13187		0.99993	0.0017539	0.01384	
transcript_HQ_CoA_transcript29198/f2p0/1395	20.08	320.8033333	49.75333333	4.1492	6.3831E-79	6.1419E-76	UP	-2.8222	7.7893E-11	3.8587E-09	DOWN	1.3138	0.0027994	0.019704	UP
transcript_HQ_CoA_transcript292/f3p0/6925	1.203333333	4.473333333	1.613333333	2.0927	0.0027586	0.01212	UP	-1.6551	0.0023313	0.012575	DOWN	0.42266	0.50793	0.70728	
transcript_HQ_CoA_transcript29203/f2p0/1398	4.05	47.38333333	39.16666667	3.7721	1.9089E-06	2.1151E-05	UP	-0.42035	0.65192	0.7937		3.3289	0.020328	0.084019	
transcript_HQ_CoA_transcript29209/f2p0/1399	41.41	76.55	77.99666667	1.0457	2.8779E-06	3.0483E-05	UP	-0.11491	0.4782	0.66106		0.91845	8.2074E-05	0.0012071	
transcript_HQ_CoA_transcript29211/f2p0/1388	30.61	10.14666667	19.04333333	-1.4256	1.5146E-08	2.5241E-07	DOWN	0.7528	0.0065945	0.02902		-0.68479	0.005171	0.031314	
transcript_HQ_CoA_transcript29213/f6p0/1369	59.43333333	138.46	88.26666667	1.3935	0.0041797	0.017129	UP	-0.81855	0.068898	0.17387		0.56427	0.2019	0.40343	
transcript_HQ_CoA_transcript29215/f2p0/1402	18.81333333	196.2033333	66.03333333	3.5395	6.7E-123	1.531E-119	UP	-1.6981	0.0016261	0.0094137	DOWN	1.8271	0.00054384	0.005518	UP
transcript_HQ_CoA_transcript29217/f2p0/1391	3.36	424.25	12.43	7.1645	2.7088E-29	3.5122E-27	UP	-5.234	1.2802E-05	0.00016712	DOWN	1.9132	0.077768	0.2154	
transcript_HQ_CoA_transcript29220/f3p0/1399	79.84666667	24.61333333	65.67	-1.5224	4.0819E-07	5.1359E-06	DOWN	1.2545	9.1181E-05	0.00089324	UP	-0.28054	0.34985	0.56757	
transcript_HQ_CoA_transcript29222/f3p0/1396	346.59	138.29	266.7066667	-1.1704	1.0879E-05	0.00010076	DOWN	0.81176	0.0052043	0.02418		-0.36893	0.27262	0.48529	
transcript_HQ_CoA_transcript29223/f3p0/1396	235.9933333	77.95333333	181.48	-1.4362	2.0947E-09	4.0913E-08	DOWN	1.0527	0.0015594	0.0090996	UP	-0.39604	0.17709	0.3708	
transcript_HQ_CoA_transcript29224/f2p0/1419	50.09	113.6733333	65.07	1.3455	4.1722E-09	7.7359E-08	UP	-0.95983	1.9225E-06	3.3242E-05		0.37283	0.025097	0.09766	
transcript_HQ_CoA_transcript29239/f3p0/1393	97.94	189.58	122.95	1.1247	2.1494E-05	0.00018328	UP	-0.77675	0.00024253	0.0020015		0.33372	0.12959	0.30293	
transcript_HQ_CoA_transcript29241/f2p0/1373	84.33	27.04	49.22333333	-1.5085	1.3946E-05	0.00012529	DOWN	0.72871	0.022348	0.074762		-0.78973	0.0019793	0.015161	
transcript_HQ_CoA_transcript29242/f2p0/1411	40.64	7.043333333	31.39333333	-2.3777	1.224E-27	1.3645E-25	DOWN	2.0011	1.2531E-08	3.8408E-07	UP	-0.3878	0.22912	0.43644	
transcript_HQ_CoA_transcript29243/f2p0/1401	23.88666667	49.32666667	30.81333333	1.2058	3.6784E-06	3.8144E-05	UP	-0.83167	0.00091184	0.0059283		0.36076	0.11827	0.28634	
transcript_HQ_CoA_transcript29252/f4p0/1364	22.93333333	151.7633333	30.80333333	2.8906	5.0747E-47	1.3446E-44	UP	-2.459	1.4208E-29	1.0851E-26	DOWN	0.41832	0.12374	0.29485	
transcript_HQ_CoA_transcript29262/f4p0/1358	3.816666667	82.22333333	65.83	4.6052	9.7256E-43	2.1951E-40	UP	-0.46655	0.4346	0.62388		4.1215	7.3859E-11	6.8816E-09	UP
transcript_HQ_CoA_transcript29267/f4p0/1383	16.53	33.16333333	24.30666667	1.1674	8.3864E-08	1.212E-06	UP	-0.59854	0.00012824	0.0011806		0.55571	0.011677	0.056289	
transcript_HQ_CoA_transcript29276/f2p0/1413	96.07333333	29.09666667	55.56666667	-1.5707	1.2489E-12	3.93E-11	DOWN	0.79356	0.00054784	0.0039178		-0.78928	0.00040411	0.0043402	
transcript_HQ_CoA_transcript29285/f5p0/1335	2.626666667	517.6633333	149.1033333	7.7813	2.7688E-65	1.3681E-62	UP	-1.9116	0.21261	0.3888		5.856	0.00023456	0.0028306	UP
transcript_HQ_CoA_transcript29286/f2p0/1377	26.94333333	49.00333333	33.34	1.0335	1.7684E-05	0.00015381	UP	-0.71115	0.0041255	0.020191		0.30824	0.20469	0.40718	
transcript_HQ_CoA_transcript29288/f2p0/1430	77.56666667	31.19	60.74666667	-1.15	7.759E-08	1.1339E-06	DOWN	0.80099	0.00090565	0.0059063		-0.36071	0.08056	0.22064	
transcript_HQ_CoA_transcript29295/f4p0/1319	23.23333333	59.24333333	31.91666667	1.5113	1.2775E-06	1.4735E-05	UP	-1.0434	0.0004762	0.0035124	DOWN	0.45518	0.0090383	0.046785	
transcript_HQ_CoA_transcript293/f2p0/6912	4.596666667	1.956666667	3.866666667	-1.0879	0.013355	0.04436	DOWN	0.85788	0.046779	0.1297		-0.24411	0.37222	0.58923	
transcript_HQ_CoA_transcript29318/f5p0/1378	423.5733333	156.3366667	330.78	-1.2691	6.3721E-05	0.00047587	DOWN	0.91361	0.00382	0.018949		-0.36713	0.05596	0.17318	
transcript_HQ_CoA_transcript29327/f2p0/1367	51.24333333	11.51333333	19.72666667	-1.9804	9.7774E-11	2.3833E-09	DOWN	0.61544	0.021554	0.072741		-1.379	1.7947E-08	9.5483E-07	DOWN

transcript_HQ_CoA_transcript29328/f3p0/1366	29.60666667	151.5366667	31.48	2.5163	4.5494E-09	8.359E-08	UP	-2.4228	2.9023E-09	1.0172E-07	DOWN	0.080962	0.76386	0.88297	
transcript_HQ_CoA_transcript29336/f2p0/1335	30.71666667	12.15666667	28.02666667	-1.1646	0.00004566	0.00035522	DOWN	1.0426	6.5846E-05	0.00067575	UP	-0.13471	0.48215	0.686	
transcript_HQ_CoA_transcript2934/f2p0/4477	8.596666667	1.776666667	6.473333333	-2.1156	5.5539E-13	1.8262E-11	DOWN	1.7114	2.3123E-09	8.3104E-08	UP	-0.41788	0.17531	0.36846	
transcript_HQ_CoA_transcript29340/f2p0/1384	34.81666667	12.15666667	39.79	-1.3515	2.2593E-06	2.4617E-05	DOWN	1.5486	5.5562E-06	8.1997E-05	UP	0.1847	0.54111	0.73206	
transcript_HQ_CoA_transcript29364/f2p0/1356	0.143333333	57.57	5.07	8.7997	1.3188E-05	0.00011941	UP	-3.6142	0.048464	0.13324		5.1675	0.00027336	0.0032184	UP
transcript_HQ_CoA_transcript29365/f4p0/1339	0.15	380.3166667	17.96666667	11.499	5.0807E-64	2.2655E-61	UP	-4.5391	0.013836	0.051743		6.9412	0.0063398	0.036414	UP
transcript_HQ_CoA_transcript29366/f2p0/1357	115.0066667	41.79	121.06	-1.3048	3.4004E-12	1.0158E-10	DOWN	1.3846	2.4966E-07	5.6494E-06	UP	0.068466	0.79993	0.9047	
transcript_HQ_CoA_transcript29371/f4p0/1370	3.706666667	153.5366667	21.37333333	5.5369	1.3615E-65	6.9144E-63	UP	-2.9921	2.2522E-06	3.8258E-05	DOWN	2.5289	0.00038304	0.0041825	UP
transcript_HQ_CoA_transcript29376/f2p0/1346	6.656666667	2.57	3.036666667	-1.2153	0.0034941	0.014797	DOWN	0.10145	0.8217	0.90287		-1.1256	0.0052561	0.031696	DOWN
transcript_HQ_CoA_transcript29381/f2p0/1349	4.41	71.65666667	18.54	4.203	2.2849E-21	1.5645E-19	UP	-2.0899	0.0085094	0.035383	DOWN	2.0991	0.0066073	0.037443	UP
transcript_HQ_CoA_transcript29383/f2p0/1362	106.27	250.6133333	160.9166667	1.4039	1.3576E-08	2.277E-07	UP	-0.78984	0.0048503	0.022895		0.60083	0.003327	0.022459	
transcript_HQ_CoA_transcript29391/f2p0/1361	21.12666667	1.446666667	9.326666667	-3.7184	1.8409E-05	0.00015964	DOWN	2.5404	0.00048716	0.0035802	UP	-1.1874	0.062183	0.18562	
transcript_HQ_CoA_transcript29395/f2p0/1352	13.23333333	113.8766667	23.55666667	3.2729	3.6572E-14	1.3872E-12	UP	-2.4291	5.7637E-06	8.4514E-05	DOWN	0.82936	0.069628	0.20012	
transcript_HQ_CoA_transcript29402/f2p0/1347	0.58	2.003333333	0.303333333	1.9425	0.0095872	0.033987	UP	-2.8669	0.0040349	0.019822	DOWN	-0.94643	0.49368	0.69554	
transcript_HQ_CoA_transcript29406/f5p0/1324	99.82333333	216.92	190.4633333	1.2849	2.9514E-08	4.7001E-07	UP	-0.3453	0.11804	0.2572		0.927	5.8446E-09	3.5173E-07	
transcript_HQ_CoA_transcript29407/f3p0/1362	8.373333333	22.33666667	18.48666667	1.5753	1.0713E-08	1.8356E-07	UP	-0.39727	0.51249	0.68725		1.1647	0.057269	0.17566	
transcript_HQ_CoA_transcript29412/f2p0/1344	31.83333333	84.25	45.92333333	1.5929	0.00030119	0.0018251	UP	-1.0494	0.018728	0.065236		0.52932	0.049437	0.15911	
transcript_HQ_CoA_transcript29414/f2p0/1346	40.42	109.07	27.73	1.5902	3.4849E-09	6.5748E-08	UP	-2.1327	4.4189E-22	1.4463E-19	DOWN	-0.5532	0.033289	0.12016	
transcript_HQ_CoA_transcript29418/f13p0/1327	14.90333333	1207.99	240.28	6.5089	2.713E-152	7.084E-149	UP	-2.4697	0.016426	0.059103		4.0219	9.9815E-05	0.0014158	UP
transcript_HQ_CoA_transcript29420/f3p0/1338	31.11333333	77.22333333	45.68	1.4752	3.6389E-12	1.0835E-10	UP	-0.91271	5.967E-06	8.7077E-05		0.54973	0.0019291	0.014865	
transcript_HQ_CoA_transcript29423/f2p0/1346	0.713333333	156.0866667	11.31666667	7.9703	1.9245E-74	1.5297E-71	UP	-3.9138	2.2238E-06	0.00003781	DOWN	4.0421	7.1557E-05	0.0010801	UP
transcript_HQ_CoA_transcript29424/f3p0/1346	22.66666667	40.88333333	29.50333333	1.0157	2.6895E-06	2.8687E-05	UP	-0.62507	0.011788	0.045697		0.3771	0.11751	0.28537	
transcript_HQ_CoA_transcript29434/f3p0/1356	540.66	239.7033333	510.5833333	-1.0296	0.0096862	0.034279	DOWN	0.94543	0.00016858	0.0014884		-0.092293	0.80738	0.90932	
transcript_HQ_CoA_transcript2944/f2p0/4471	10.99	0.973333333	4.18	-3.3454	1.5762E-09	3.1425E-08	DOWN	1.9305	0.00011096	0.0010483	UP	-1.4218	0.01038	0.051698	
transcript_HQ_CoA_transcript29440/f2p0/1332	7.516666667	2.676666667	4.95	-1.3305	0.0014226	0.0069497	DOWN	0.73709	0.10107	0.23041		-0.60412	0.16105	0.34899	
transcript_HQ_CoA_transcript29447/f2p0/1320	63.85666667	10.74666667	27.28666667	-2.3827	0.00011685	0.00080584	DOWN	1.1869	0.00077425	0.0051926	UP	-1.2088	0.032693	0.11862	
transcript_HQ_CoA_transcript29455/f2p0/1344	61.39333333	26.24	37.06666667	-1.0753	0.00018422	0.0011973	DOWN	0.34859	0.024748	0.080756		-0.73775	0.0064415	0.036799	
transcript_HQ_CoA_transcript29457/f3p0/1348	38.39666667	69.47666667	34.18333333	1.0176	0.0068053	0.02559	UP	-1.1747	0.0025831	0.013676	DOWN	-0.17131	0.44129	0.65231	
transcript_HQ_CoA_transcript29462/f8p0/1315	51.50333333	177.8166667	106.4666667	1.9712	1.8279E-08	3.0078E-07	UP	-0.90046	0.086004	0.20446		1.053	0.036471	0.12831	
transcript_HQ_CoA_transcript29474/f4p0/1323	37.22	11.07	54.85333333	-1.584	1.366E-09	2.7504E-08	DOWN	2.1469	2.5139E-10	1.1321E-08	UP	0.55208	0.081922	0.22298	
transcript_HQ_CoA_transcript29479/f2p0/1327	13.68333333	137.8533333	36.65	3.5011	7.092E-14	2.588E-12	UP	-2.0296	9.4271E-07	1.8131E-05	DOWN	1.4552	0.010104	0.050699	
transcript_HQ_CoA_transcript29483/f2p0/1337	223.5033333	5.516666667	5.013333333	-5.2275	0.0015609	0.0075293	DOWN	-0.28647	0.83464	0.90947		-5.5138	0.00015725	0.0020528	DOWN
transcript_HQ_CoA_transcript29491/f2p0/1326	18.93666667	8.283333333	20.43	-1.0438	0.014102	0.046334	DOWN	1.1698	0.0016359	0.0094619	UP	0.11145	0.74855	0.87315	
transcript_HQ_CoA_transcript29492/f3p0/1326	18.63333333	40.71333333	25.14666667	1.282	1.0301E-07	1.4588E-06	UP	-0.83791	0.00065842	0.0045644		0.43297	0.14019	0.31914	
transcript_HQ_CoA_transcript29495/f2p0/1319	6.543333333	14.99	13.38	1.3747	0.00084651	0.0044458	UP	-0.31832	0.39248	0.5829		1.0428	0.0091169	0.047004	UP
transcript_HQ_CoA_transcript29503/f2p0/1338	9.133333333	564.05	182.9633333	6.0974	1.6272E-40	3.3425E-38	UP	-1.753	0.10046	0.22935		4.3321	0.00018345	0.0023303	UP
transcript_HQ_CoA_transcript29507/f3p0/1320	207.03	63.10666667	197.23	-1.5613	3.2395E-24	2.6799E-22	DOWN	1.4923	1.4174E-07	3.396E-06	UP	-0.080486	0.77099	0.88659	
transcript_HQ_CoA_transcript29515/f2p0/1318	108.2866667	26.20333333	123.2166667	-1.8896	2.9917E-06	3.1615E-05	DOWN	2.1131	6.8096E-08	1.7579E-06	UP	0.21112	0.62202	0.78805	
transcript_HQ_CoA_transcript29519/f2p0/1358	0.39	13.42666667	3.75	5.2337	3.7438E-06	3.8757E-05	UP	-2.039	0.0033825	0.017112	DOWN	3.1854	0.0035676	0.023734	UP
transcript_HQ_CoA_transcript29525/f2p0/1321	33.35666667	61.68666667	49.52333333	1.0377	2.391E-07	3.1652E-06	UP	-0.45772	0.0040095	0.019723		0.56753	0.0013266	0.011216	
transcript_HQ_CoA_transcript29530/f5p0/1306	1.576666667	82.25333333	62.89	5.84	5.1872E-28	5.9643E-26	UP	-0.51204	0.67703	0.81215		5.3138	1.7544E-05	0.00033826	UP
transcript_HQ_CoA_transcript29552/f4p0/1304	10.03333333	211.5666667	107.6733333	4.5342	2.0794E-08	3.4004E-07	UP	-1.076	0.46617	0.65071		3.4461	0.0060807	0.035376	UP
transcript_HQ_CoA_transcript29553/f2p0/1309	0.293333333	20.5	14.38	6.252	5.0106E-26	4.7219E-24	UP	-0.63397	0.46494	0.64969		5.6075	3.2419E-07	1.1997E-05	UP
transcript_HQ_CoA_transcript29559/f2p0/1309	20.89666667	6.56	11.75333333	-1.5253	6.6254E-05	0.00049218	DOWN	0.70756	0.10387	0.23499		-0.82823	0.069715	0.20023	
transcript_HQ_CoA_transcript2956/f2p0/4465	1.243333333	3.333333333	1.643333333	1.5694	0.00019527	0.0012588	UP	-1.1721	0.0031622	0.016171	DOWN	0.38632	0.27818	0.49187	
transcript_HQ_CoA_transcript29561/f2p0/1299	3.083333333	146.49	8.186666667	5.7589	1.2E-21	8.3735E-20	UP	-4.3137	2.1016E-09	7.6177E-08	DOWN	1.429	0.11142	0.27552	
transcript_HQ_CoA_transcript29567/f2p0/1309	341.8566667	33.73333333	180.76	-3.1637	5.8522E-09	1.052E-07	DOWN	2.2428	1.5162E-05	0.00019192	UP	-0.93281	0.0021499	0.016159	
transcript_HQ_CoA_transcript29569/f2p0/1295	17.57	128.06	40.31	3.0344	3.174E-11	8.31E-10	UP	-1.8088	1.3781E-05	0.00017801	DOWN	1.2113	0.0010038	0.0089872	UP
transcript_HQ_CoA_transcript2957/f5p0/4482	7.773333333	2.136666667	3.273333333	-1.7387	0.0002886	0.0017622	DOWN	0.48343	0.16122	0.32114		-1.2642	0.0019768	0.01515	DOWN
transcript_HQ_CoA_transcript29573/f2p0/1296	651.1033333	18.17	46.04333333	-5.0447	4.8148E-06	4.8659E-05	DOWN	1.2327	0.1692	0.33137		-3.8209	8.7418E-38	4.0114E-34	DOWN
transcript_HQ_CoA_transcript29585/f2p0/1300	0.513333333	49.74666667	1.336666667	6.6815	2.1101E-14	8.2591E-13	UP	-5.3109	5.1204E-11	2.607E-09	DOWN	1.362	0.10263	0.26143	
transcript_HQ_CoA_transcript29588/f7p0/1295	373.1866667	1237.966667	411.8133333	1.8931	3.1228E-10	7.0137E-09	UP	-1.7229	3.9647E-15	4.632E-13	DOWN	0.1562	0.61061	0.77991	
transcript_HQ_CoA_transcript29593/f2p0/1292	79	21.31333333	59	-1.7286	5.3817E-11	1.3608E-09	DOWN	1.3136	1.2571E-05	0.0001649	UP	-0.42648	0.18142	0.37683	
transcript_HQ_CoA_transcript29595/f2p0/1294	22.89666667	93.49666667	36.56333333	2.1921	9.0926E-13	2.9266E-11	UP	-1.5012	2.871E-06	4.7281E-05	DOWN	0.67799	0.0033986	0.022842	
transcript_HQ_CoA_transcript29597/f2p0/1291	53.58	20.12666667	32.56333333	-1.2727	0.00012241	0.00083883	DOWN	0.57839	0.20012	0.37254		-0.70461	0.080561	0.22064	
transcript_HQ_CoA_transcript29599/f2p0/1298	0.936666667	70.30333333	5.463333333	6.4336	1.6927E-49	4.8352E-47	UP	-3.8455	5.6904E-07	0.00001178	DOWN	2.5685	0.014802	0.066558	
transcript_HQ_CoA_transcript29603/f9p0/1216	5.713333333	898.3133333	157.7566667	7.4767	1.427E-69	9.317E-67	UP	-2.6485	0.022956	0.07628		4.8085	0.00011563	0.0016042	UP
transcript_HQ_CoA_transcript29604/f2p0/1292	0	17.36333333	1.92	10.192	7.9809E-14	2.8835E-12	UP	-3.3323	0.0033376	0.016927	DOWN	6.8459	0.0042842	0.02721	UP
transcript_HQ_CoA_transcript29607/f2p0/1300	70.14333333	30.02	49.85333333	-1.0708	2.6387E-06	2.8228E-05	DOWN	0.57973	0.00030475	0.0024118		-0.50306	0.020094	0.083368	
transcript_HQ_CoA_transcript29609/f3p0/1292	13.78666667	46.38666667	33.42333333	1.9034	3.3439E-13	1.1279E-11	UP	-0.61225	0.0094961	0.038448		1.28	4.6295E-05	0.00075035	UP
transcript_HQ_CoA_transcript29610/f4p0/1259	2.14	88.79333333	177.18	5.5411	8.9223E-33	1.3942E-30	UP	0.88935	0.28912	0.47867		6.4138	1.5349E-07	6.3167E-06	UP
transcript_HQ_CoA_transcript29625/f3p0/1282	123.01	26.73	70.84333333	-2.0329	6.5341E-07	7.932E-06	DOWN	1.2543	0.0010037	0.0064078	UP	-0.79022	0.066201	0.19355	
transcript_HQ_CoA_transcript29632/f2p0/1288	26.02	67.22666667	48.95333333	1.5211	1.7827E-08	2.9441E-07	UP	-0.60458	0.00001633	0.00020549		0.90534	0.00076814	0.0072676	
transcript_HQ_CoA_transcript29652/f2p0/1299	48.68	202.1333333	59.66333333	2.228	1.217E-11	3.4071E-10	UP	-1.9187	1.9205E-11	1.0699E-09	DOWN	0.29524	0.20392	0.40618	
transcript_HQ_CoA_transcript29661/f2p0/1286	4.353333333	251.5233333	22.32666667	6.0212	2.8517E-31	4.1051E-29	UP	-3.6406	4.8734E-06	7.3337E-05	DOWN	2.3657	0.00162	0.012968	UP
transcript_HQ_CoA_transcript29665/f3p0/1268	32.05333333	61.58333333	36.84333333	1.0943	0.00060179	0.0033059	UP	-0.8808	0.0042827	0.020805		0.20168	0.4832	0.68686	
transcript_HQ_CoA_transcript29671/f2p0/1263	49.64666667	15.90333333	40.39333333	-1.4934	3.7409E-10	8.3387E-09	DOWN	1.2051	0.00003339	0.00038467	UP	-0.29914	0.22485	0.43202	
transcript_HQ_CoA_transcript2968/f2p0/4464	6.626666667	2.163333333	2.186666667	-1.479	0.00016367	0.0010798	DOWN	-0.10889	0.8065	0.89287		-1.5976	6.1914E-07	2.0814E-05	DOWN
transcript_HQ_CoA_transcript29680/f2p0/1269	1.71	52.64333333	4.753333333	5.133	3.5917E-25	3.2347E-23	UP	-3.627	2.1958E-17	3.4997E-15	DOWN	1.4891	0.055633	0.17255	
transcript_HQ_CoA_transcript29682/f5p0/1256	187.13	46.37	69.44333333	-1.8548	1.8184E-16	8.6801E-15	DOWN	0.44199	0.30192	0.49228		-1.4216	0.00016424	0.0021274	DOWN
transcript_HQ_CoA_transcript29685/f4p0/1251	357.0733333	55.46666667	90.66666667	-2.5396	1.3534E-08	2.2741E-07	DOWN	0.55291	0.089228	0.21014		-1.9944	3.4925E-06	8.9355E-05	DOWN
transcript_HQ_CoA_transcript29689/f3p0/1237	1.813333333	72.97	2.77	5.5046	1.3621E-44	3.2765E-42	UP	-4.869	0.0012381	0.0075595	DOWN	0.61861	0.62886	0.79321	
transcript_HQ_CoA_transcript29697/f2p0/1263	7.306666667	3.223333333	4.083333333	-1.0367	0.0133	0.044224	DOWN	0.21032	0.65208	0.79383		-0.83695	0.030265	0.11243	
transcript_HQ_CoA_transcript29714/f2p0/1249	72.72666667	31.47333333	46.01	-1.052	1.8736E-05	0.0001618	DOWN	0.39973	0.078974	0.19254		-0.66365	0.007165	0.039506	
transcript_HQ_CoA_transcript29720/f3p0/1254	31.65666667	302.66	56.36666667	3.4711	1.3588E-07	1.8862E-06	UP	-2.5997	1.4586E-08	4.39E-07	DOWN	0.84897	0.21785	0.4234	
transcript_HQ_CoA_transcript29723/f3p0/1277	38.12333333	82.85	47.3	1.2674	0.0039089	0.016249	UP	-0.95685	0.020308	0.069368		0.2994	0.1909	0.38893	
transcript_HQ_CoA_transcript29725/f2p0/1258	23.47	86.12333333	34.17	2.0328	1.3966E-09	2.8088E-08	UP	-1.485	1.0347E-06	1.9591E-05	DOWN	0.53644	0.016069	0.070628	
transcript_HQ_CoA_transcript2973/f5p0/4391	6.466666667	71.9	18.06	3.6515	2.0353E-22	1.4649E-20	UP	-2.1526	2.5711E-11	1.3901E-09	DOWN	1.4846	9.9779E-09	5.7232E-07	UP
transcript_HQ_CoA_transcript29739/f2p0/1266	39.1	9.95	20.60666667	-1.8148	0.0011053	0.005598	DOWN	0.91752	0.07161	0.17911		-0.90582	0.1427	0.32266	

transcript_HQ_CoA_transcript29740/f2p0/1253	77.93	25.93333333	48.12	-1.4346	9.6423E-19	5.4746E-17	DOWN	0.74091	0.0004271	0.0032044		-0.70676	0.0002488	0.0029693	
transcript_HQ_CoA_transcript2975/f2p0/4451	7.39	1.3	1.646666667	-2.3866	6.2695E-05	0.00046918	DOWN	0.20222	0.7779	0.87549		-2.1913	1.1898E-07	5.0436E-06	DOWN
transcript_HQ_CoA_transcript29750/f2p0/1227	0.106666667	22.47333333	27.69666667	7.9988	4.272E-16	1.9722E-14	UP	0.18268	0.86733	0.9282		8.1772	0.00021111	0.0025988	UP
transcript_HQ_CoA_transcript29753/f2p0/1249	0.446666667	1214.316667	88.59	11.566	5.828E-163	2.131E-159	UP	-3.9084	0.010873	0.042862	DOWN	7.6353	3.871E-06	9.7066E-05	UP
transcript_HQ_CoA_transcript29756/f3p0/1229	40.51	85.97	35.96	1.2507	0.00029834	0.0018096	UP	-1.4198	1.2487E-06	2.2888E-05	DOWN	-0.18137	0.51891	0.71512	
transcript_HQ_CoA_transcript29762/f3p0/1235	156.52	30.66	78.69333333	-2.1865	1.779E-14	7.0398E-13	DOWN	1.1927	0.00071297	0.0048719	UP	-1.0042	0.0012424	0.010616	DOWN
transcript_HQ_CoA_transcript29763/f2p0/1240	131.2533333	56.02	96.23666667	-1.0793	1.003E-09	2.0909E-08	DOWN	0.64145	1.4383E-05	0.00018436		-0.44949	0.0049537	0.030319	
transcript_HQ_CoA_transcript29775/f2p0/1228	95.68	39.25333333	109.77	-1.1384	5.3868E-08	8.1389E-07	DOWN	1.3444	4.3442E-12	2.7647E-10	UP	0.19373	0.1932	0.39197	
transcript_HQ_CoA_transcript29777/f3p0/1230	1164.706667	424.8466667	866.5233333	-1.3177	1.7541E-07	2.3807E-06	DOWN	0.89499	0.00036791	0.0028274		-0.43221	0.071948	0.20427	
transcript_HQ_CoA_transcript29784/f2p0/1242	11.21	79.15333333	35.50666667	2.9702	9.1589E-19	5.2163E-17	UP	-1.3028	0.0029729	0.015345	DOWN	1.6549	0.00088849	0.00815	UP
transcript_HQ_CoA_transcript29786/f4p0/1237	14.16666667	281.1166667	18.52666667	4.485	1.1681E-28	1.4237E-26	UP	-4.0838	0.0025755	0.013644	DOWN	0.38542	0.70192	0.84284	
transcript_HQ_CoA_transcript29793/f3p0/1229	82.14	26.78666667	27.97	-1.4348	9.5417E-05	0.0006756	DOWN	-0.10233	0.74555	0.85626		-1.5516	1.112E-08	6.2999E-07	DOWN
transcript_HQ_CoA_transcript29796/f3p0/1226	50.54333333	223.36	50.9	2.3192	2.8852E-11	7.6557E-10	UP	-2.2774	4.9949E-56	2.2888E-52	DOWN	0.027695	0.93862	0.97286	
transcript_HQ_CoA_transcript29802/f3p0/1223	551.0033333	235.17	582.24	-1.0777	4.8656E-08	7.4313E-07	DOWN	1.1635	3.2055E-08	8.9426E-07	UP	0.074813	0.71577	0.85185	
transcript_HQ_CoA_transcript29803/f4p0/1210	19.18	85.68333333	50.97	2.3539	0.00033748	0.0020182	UP	-0.87448	0.16423	0.32479		1.4601	0.070744	0.20194	
transcript_HQ_CoA_transcript29808/f2p0/1218	49.92666667	6.19	35.51333333	-2.8794	1.7553E-09	3.4762E-08	DOWN	2.3851	4.7036E-24	2.1553E-21	UP	-0.50292	0.24195	0.45086	
transcript_HQ_CoA_transcript29814/f3p0/1227	119.47	29.13	54.06	-1.8865	3.9728E-07	5.0091E-06	DOWN	0.73535	0.0021694	0.011838		-1.1609	0.00034689	0.0038566	DOWN
transcript_HQ_CoA_transcript29819/f2p0/1222	15.08	42.36666667	20.60666667	1.6604	1.5236E-06	1.7258E-05	UP	-1.2018	0.00035715	0.0027563	DOWN	0.44501	0.081938	0.22298	
transcript_HQ_CoA_transcript2983/f4p0/4442	12.78666667	5.59	14.82333333	-1.0126	0.0099573	0.035116	DOWN	1.2314	0.0014873	0.0087741	UP	0.20543	0.34876	0.56674	
transcript_HQ_CoA_transcript29830/f4p0/1200	26.35	386.5833333	63.44	4.041	2.3944E-55	7.6798E-53	UP	-2.7412	8.7689E-12	5.2353E-10	DOWN	1.285	0.0027974	0.019704	UP
transcript_HQ_CoA_transcript29836/f2p0/1226	0.923333333	411.0966667	23.71666667	8.9685	3.373E-167	1.542E-163	UP	-4.2558	0.00055432	0.003958	DOWN	4.6929	6.0623E-06	0.00014085	UP
transcript_HQ_CoA_transcript29844/f2p0/1184	0.676666667	59.17	295.14	6.6011	0.00016649	0.0010948	UP	2.2032	0.34559	0.53772		8.79	3.6331E-06	9.2491E-05	UP
transcript_HQ_CoA_transcript2985/f2p0/4456	8.253333333	26.72	10.01	1.8633	1.4114E-09	2.8354E-08	UP	-1.5799	1.7786E-08	5.2243E-07	DOWN	0.27025	0.25631	0.46684	
transcript_HQ_CoA_transcript29853/f4p0/1202	1.216666667	102.88	7.183333333	6.5806	7.5827E-51	2.2004E-48	UP	-3.9939	5.4509E-16	6.9867E-14	DOWN	2.571	0.00015076	0.0019879	UP
transcript_HQ_CoA_transcript29857/f2p0/1192	46.08666667	167.9666667	91.36333333	2.0437	1.3547E-09	2.737E-08	UP	-1.0401	0.00018039	0.0015797	DOWN	0.98795	0.00284	0.019926	
transcript_HQ_CoA_transcript29862/f2p0/1210	19.72	4.916666667	11.11666667	-1.8709	0.0042343	0.017299	DOWN	1.0885	0.17048	0.33306		-0.79559	0.14302	0.32314	
transcript_HQ_CoA_transcript29865/f2p0/1227	52.03	95.51666667	79.99333333	1.0389	4.259E-06	4.3499E-05	UP	-0.40902	0.017407	0.061737		0.61683	0.0013437	0.011324	
transcript_HQ_CoA_transcript29866/f3p0/1201	78.80666667	30.33333333	35.55	-1.2058	4.8452E-05	0.0003736	DOWN	0.064236	0.80131	0.89057		-1.1549	2.2784E-06	6.3269E-05	DOWN
transcript_HQ_CoA_transcript29867/f2p0/1196	164.86	56.27666667	171.9266667	-1.3786	3.2521E-06	3.4091E-05	DOWN	1.4564	7.1141E-08	1.811E-06	UP	0.064357	0.84914	0.93111	
transcript_HQ_CoA_transcript29871/f4p0/1159	38.71	184.47	246.6133333	2.431	4.5911E-05	0.00035686	UP	0.27502	0.50448	0.68151		2.69	5.6268E-07	1.9305E-05	UP
transcript_HQ_CoA_transcript29874/f2p0/1192	60.83666667	21.41333333	48.37333333	-1.3361	0.00006794	0.00050279	DOWN	1.0148	0.0032315	0.016471	UP	-0.33507	0.089615	0.23784	
transcript_HQ_CoA_transcript29879/f2p0/1182	54.48666667	22.71333333	48.43	-1.0781	0.0094371	0.033605	DOWN	0.9572	0.067967	0.17212		-0.13355	0.80663	0.90887	
transcript_HQ_CoA_transcript29884/f3p0/1199	5.336666667	259.7666667	21.65666667	5.7722	4.3624E-37	8.1381E-35	UP	-3.7386	2.412E-12	1.681E-10	DOWN	2.0197	0.0011365	0.0098958	UP
transcript_HQ_CoA_transcript29913/f2p0/1178	1.65	50.66	12.75	5.1002	4.5129E-21	3.0558E-19	UP	-2.1267	0.0036238	0.018108	DOWN	2.9592	0.00036311	0.0040175	UP
transcript_HQ_CoA_transcript29916/f5p0/1178	0.79	1288.346667	96.96333333	10.824	4.2261E-67	2.2724E-64	UP	-3.8928	0.0013393	0.0080673	DOWN	6.9132	8.802E-08	3.8744E-06	UP
transcript_HQ_CoA_transcript29919/f2p0/1177	86.47666667	16.66666667	59.43	-2.2254	3.4316E-18	1.8727E-16	DOWN	1.686	8.643E-10	3.4364E-08	UP	-0.54903	0.056618	0.17438	
transcript_HQ_CoA_transcript29922/f2p0/1171	35.05666667	64.59	33.79333333	1.0583	0.011134	0.038462	UP	-1.0884	4.4741E-05	0.00049164	DOWN	-0.043955	0.91234	0.96114	
transcript_HQ_CoA_transcript29927/f5p0/1167	11.07333333	98.15666667	76.02	3.3445	1.0702E-06	1.2494E-05	UP	-0.52758	0.035608	0.10569		2.7971	2.3619E-05	0.00043294	UP
transcript_HQ_CoA_transcript29932/f2p0/1165	0.28	69.22333333	10.79	8.1284	1.9161E-32	2.9192E-30	UP	-2.8303	0.054395	0.1458		5.2797	6.0447E-05	0.00093867	UP
transcript_HQ_CoA_transcript29941/f2p0/1198	14.99333333	35.26333333	26.06333333	1.4208	0.0024298	0.010901	UP	-0.60311	0.21928	0.39656		0.80395	0.023896	0.094204	
transcript_HQ_CoA_transcript2995/f3p0/4423	1.3	4.22	2.096666667	1.8571	0.0023934	0.010769	UP	-1.1634	0.063192	0.16362		0.68285	0.13991	0.3187	
transcript_HQ_CoA_transcript29950/f2p0/1154	30.02	63.21333333	28.72666667	1.2319	0.011326	0.038952	UP	-1.2691	0.00025956	0.0021163	DOWN	-0.051273	0.91469	0.96235	
transcript_HQ_CoA_transcript29963/f2p0/1175	0.813333333	102.3	9.366666667	7.122	1.6055E-27	1.7682E-25	UP	-3.5986	8.5227E-07	1.6707E-05	DOWN	3.5112	0.00031662	0.0035931	UP
transcript_HQ_CoA_transcript29964/f2p0/1148	83	233.4266667	144.08	1.6535	1.5018E-16	7.2442E-15	UP	-0.84708	7.6347E-06	0.00010723		0.79438	3.9261E-07	1.4186E-05	
transcript_HQ_CoA_transcript29965/f5p0/1137	15.80333333	950.3666667	78.71333333	6.073	1.964E-288	3.59E-284	UP	-3.7357	1.043E-16	1.4594E-14	DOWN	2.3222	4.3538E-07	1.5487E-05	UP
transcript_HQ_CoA_transcript29966/f4p0/1127	67.69	134.8066667	119.6866667	1.1485	1.9491E-14	7.663E-13	UP	-0.3169	0.0031595	0.016163		0.81906	2.7381E-08	1.3999E-06	
transcript_HQ_CoA_transcript29972/f2p0/1142	27.25	102.07	43.64333333	2.0812	1.0921E-09	2.261E-08	UP	-1.3776	1.1386E-07	2.7751E-06	DOWN	0.68821	0.03449	0.12321	
transcript_HQ_CoA_transcript29975/f3p0/1155	40.22666667	15.85666667	92.22666667	-1.1648	0.0055367	0.021564	DOWN	2.4017	2.6002E-05	0.0003059	UP	1.2227	0.0079426	0.04259	UP
transcript_HQ_CoA_transcript2998/f4p0/4439	0.786666667	4.42	1.096666667	2.6789	9.0094E-07	1.0685E-05	UP	-2.1615	4.261E-06	6.5575E-05	DOWN	0.50238	0.28878	0.50357	
transcript_HQ_CoA_transcript29987/f5p0/1108	208.3433333	82.35333333	238.8533333	-1.2	9.0276E-06	8.6049E-05	DOWN	1.401	0.00024292	0.0020038	UP	0.19042	0.55053	0.73858	
transcript_HQ_CoA_transcript29993/f2p0/1126	122.7266667	318.95	175.9866667	1.5322	2.7468E-14	1.055E-12	UP	-1.0013	0.00051965	0.0037632	DOWN	0.51739	0.070381	0.20145	
transcript_HQ_CoA_transcript3000/f2p0/4468	6.966666667	2.753333333	3.716666667	-1.1885	4.0855E-06	4.1891E-05	DOWN	0.2823	0.31765	0.50864		-0.91874	0.00022255	0.002716	
transcript_HQ_CoA_transcript30000/f2p0/1148	0.07	56.44	120.3166667	9.8004	7.5029E-16	3.3953E-14	UP	0.97749	0.66247	0.80208		10.751	0.0026122	0.018649	UP
transcript_HQ_CoA_transcript30016/f2p0/1117	289.4666667	41.98	83.8	-2.6468	2.8372E-08	4.5414E-07	DOWN	0.86757	0.015114	0.055405		-1.7876	0.0002002	0.0024913	DOWN
transcript_HQ_CoA_transcript30031/f3p0/1075	0.076666667	380.5333333	276.0033333	12.41	7.4698E-33	1.1875E-30	UP	-0.56485	0.76511	0.86798		11.815	0.00002482	0.00045017	UP
transcript_HQ_CoA_transcript30037/f2p0/1090	16.50333333	237.84	72.51333333	4.0183	1.8665E-58	6.6909E-56	UP	-1.8501	0.00031841	0.0024984	DOWN	2.1537	2.1511E-05	0.00039938	UP
transcript_HQ_CoA_transcript30044/f3p0/1090	276.51	60.8	157.9633333	-2.0218	6.6066E-14	2.4156E-12	DOWN	1.2373	0.00088325	0.005786	UP	-0.79591	0.042612	0.14322	
transcript_HQ_CoA_transcript30050/f2p0/1073	324.0933333	114.9533333	202.41	-1.3395	1.3866E-13	4.9128E-12	DOWN	0.66923	0.00042041	0.0031636		-0.68296	0.00076448	0.0072368	
transcript_HQ_CoA_transcript30062/f6p0/1084	1041.38	212.08	426.6266667	-2.1533	5.3288E-15	2.2344E-13	DOWN	0.86003	0.0049244	0.023165		-1.3027	5.6186E-05	0.00088295	DOWN
transcript_HQ_CoA_transcript30063/f2p0/1077	622.1666667	227.0633333	244.2266667	-1.318	0.00001405	0.00012591	DOWN	-0.025715	0.93181	0.96444		-1.3532	6.7445E-08	3.0871E-06	DOWN
transcript_HQ_CoA_transcript30066/f8p0/1057	1619.046667	627.2333333	780.66	-1.2367	0.00070309	0.0037772	DOWN	0.18117	0.58584	0.74755		-1.0632	0.0034662	0.023197	DOWN
transcript_HQ_CoA_transcript3007/f2p0/4438	14.54333333	32.21666667	13.69333333	1.3054	0.00050263	0.0028352	UP	-1.3863	0.00010578	0.0010093	DOWN	-0.092737	0.75104	0.87449	
transcript_HQ_CoA_transcript30071/f3p0/1089	635.5033333	263.0933333	472.1733333	-1.1324	1.0339E-05	9.6443E-05	DOWN	0.70601	0.0067996	0.029715		-0.43656	0.061185	0.18386	
transcript_HQ_CoA_transcript30076/f2p0/1069	485.8033333	161.7633333	275.0566667	-1.4379	1.765E-09	3.4883E-08	DOWN	0.62464	0.023029	0.076473		-0.82264	0.0072848	0.03995	
transcript_HQ_CoA_transcript30079/f11p0/1037	982.0766667	380.8	645.62	-1.2307	8.8293E-06	8.4335E-05	DOWN	0.61794	0.059749	0.15663		-0.62328	0.035473	0.12579	
transcript_HQ_CoA_transcript30085/f2p0/1051	754.2533333	341.9333333	428.3233333	-1.003	0.010472	0.036577	DOWN	0.1845	0.55759	0.72488		-0.82594	0.047615	0.1549	
transcript_HQ_CoA_transcript30092/f2p0/1052	35.02333333	12.21666667	15.13333333	-1.3554	2.9405E-05	0.00024194	DOWN	0.17714	0.66089	0.80086		-1.1903	0.0026165	0.018666	DOWN
transcript_HQ_CoA_transcript30093/f2p0/1040	82.63	182.94	104.45	1.284	0.0019951	0.0092552	UP	-0.95072	3.6523E-10	1.5901E-08		0.32385	0.41639	0.62963	
transcript_HQ_CoA_transcript30098/f2p0/1029	41.63	146.9066667	66.89	1.9939	3.7447E-10	8.3387E-09	UP	-1.2952	1.4524E-05	0.00018577	DOWN	0.68499	0.00019166	0.0024145	
transcript_HQ_CoA_transcript30104/f2p0/1025	56.17	7.59	88.59	-2.6932	0.0012536	0.0062397	DOWN	3.3362	1.0475E-07	2.5771E-06	UP	0.63407	0.29239	0.50744	
transcript_HQ_CoA_transcript30115/f2p0/1015	414.0733333	135.18	179.6966667	-1.4636	0.00050397	0.0028411	DOWN	0.25094	0.31237	0.50396		-1.2213	0.0015993	0.012824	DOWN
transcript_HQ_CoA_transcript30128/f3p0/1005	257.8433333	54.48333333	138.4966667	-2.0644	5.1924E-09	9.4455E-08	DOWN	1.1876	0.000238	0.0019721	UP	-0.8908	0.024018	0.094544	
transcript_HQ_CoA_transcript30135/f2p0/985	44.03666667	155.47	86.67	1.988	2.1233E-18	1.1799E-16	UP	-0.98815	2.5429E-06	4.2488E-05		0.98659	5.2557E-05	0.00083378	
transcript_HQ_CoA_transcript30137/f2p0/1010	180.6433333	66.16666667	161.33	-1.3189	0.0012577	0.0062583	DOWN	1.1468	0.00030202	0.0023923	UP	-0.18366	0.62092	0.78746	
transcript_HQ_CoA_transcript30143/f2p0/984	89.94666667	39.12333333	46.05	-1.0561	0.00044165	0.0025383	DOWN	0.089558	0.71922	0.83927		-0.97712	0.0017385	0.013736	
transcript_HQ_CoA_transcript30148/f2p0/963	180.1666667	689.6266667	242.6	2.1168	1.4625E-12	4.5603E-11	UP	-1.6634	4.4391E-06	6.7803E-05	DOWN	0.43969	0.10185	0.26031	
transcript_HQ_CoA_transcript30151/f2p0/945	277.1966667	108.8466667	190.5933333	-1.1849	6.6483E-10	1.4193E-08	DOWN	0.64986	0.0093439	0.037958		-0.54847	0.037491	0.1308	
transcript_HQ_CoA_transcript30160/f3p0/938	31.67666667	88.70333333	36.61333333	1.6579	6.9474E-07	8.3948E-06	UP	-1.4316	7.2126E-05	0.00073119	DOWN	0.21216	0.42533	0.63745	
transcript_HQ_CoA_transcript30168/f3p0/826	3663.873333	300.1766667	149.1	-3.4753	7.7979E-08	1.1387E-06	DOWN	-1.1437	0.12875	0.2735		-4.6225	3.84E-07	1.3902E-05	DOWN

transcript_HQ_CoA_transcript30169/f6p0/899	0	1.636666667	2061.756667	6.0387	0.0018516	0.0087018	UP	10.226	6.4627E-05	0.00066436	UP	29.724	2.7783E-14	5.5431E-12	UP
transcript_HQ_CoA_transcript30186/f6p0/863	4148.89	1017.323333	2121.836667	-1.8753	5.8386E-14	2.1564E-12	DOWN	0.91042	0.00043062	0.0032242		-0.97553	0.00038598	0.0042045	
transcript_HQ_CoA_transcript30190/f2p0/880	1057.24	85.52333333	778.5366667	-3.4899	2.8373E-07	3.6945E-06	DOWN	3.0717	3.0749E-06	4.9875E-05	UP	-0.43133	0.15991	0.34751	
transcript_HQ_CoA_transcript30198/f4p0/821	956.2866667	48.82	79.27	-4.161	5.7621E-08	8.6347E-07	DOWN	0.56289	0.51178	0.68656		-3.6028	1.5148E-09	1.0184E-07	DOWN
transcript_HQ_CoA_transcript30201/f11p0/821	9624.403333	454.6766667	440.1766667	-4.2734	3.1773E-11	8.31E-10	DOWN	-0.18265	0.78302	0.87893		-4.4601	1.6685E-07	6.8208E-06	DOWN
transcript_HQ_CoA_transcript30208/f2p0/821	4.933333333	1939.303333	153.85	8.7916	2.028E-216	1.236E-212	UP	-3.7868	2.7173E-07	6.0739E-06	DOWN	4.9883	2.3504E-11	2.4374E-09	UP
transcript_HQ_CoA_transcript30210/f2p0/814	3893.14	195.5333333	251.21	-4.1937	1.8149E-08	2.9893E-07	DOWN	0.23165	0.7707	0.87091		-3.9668	8.0443E-12	9.4047E-10	DOWN
transcript_HQ_CoA_transcript30211/f7p0/792	7485.013333	1173.62	1621.28	-2.5356	8.2364E-09	1.4451E-07	DOWN	0.33051	0.51111	0.68612		-2.2109	8.2618E-06	0.00018183	DOWN
transcript_HQ_CoA_transcript30215/f2p0/801	101.7566667	252.3	153.8166667	1.4663	3.4919E-14	1.33E-12	UP	-0.84673	0.0076868	0.032743		0.60793	0.059945	0.18118	
transcript_HQ_CoA_transcript30216/f2p0/802	94.42	233.2766667	211.3433333	1.47	9.5876E-16	4.2856E-14	UP	-0.27021	0.55328	0.72091		1.1861	0.0065315	0.037108	UP
transcript_HQ_CoA_transcript30224/f3p0/779	2257.433333	139.9133333	109.89	-3.828	0.00029696	0.0018031	DOWN	-0.53227	0.29477	0.484		-4.3705	2.3417E-06	0.00006483	DOWN
transcript_HQ_CoA_transcript30227/f2p0/780	2299.56	410.3666667	145.7633333	-2.3494	3.4644E-05	0.00027938	DOWN	-1.6333	0.00021915	0.001846	DOWN	-3.9904	1.6252E-16	4.5892E-14	DOWN
transcript_HQ_CoA_transcript30230/f3p0/748	0.263333333	1937.176667	103.34	13.001	2.8934E-67	1.653E-64	UP	-4.3627	0.027946	0.088619		8.6038	0.013395	0.062107	
transcript_HQ_CoA_transcript30234/f2p0/774	2899.713333	1258.063333	3926.443333	-1.0701	0.00012204	0.00083673	DOWN	1.5112	0.00011268	0.001063	UP	0.43041	0.21127	0.41523	
transcript_HQ_CoA_transcript30237/f2p0/757	2378.966667	958.95	2497.343333	-1.1603	1.3204E-08	2.2248E-07	DOWN	1.2333	3.1873E-05	0.00036904	UP	0.061915	0.83552	0.92396	
transcript_HQ_CoA_transcript30244/f2p0/736	392.58	958.0633333	521.59	1.4458	3.2593E-28	3.8693E-26	UP	-1.0108	1.663E-06	2.9422E-05	DOWN	0.42243	0.052316	0.16545	
transcript_HQ_CoA_transcript30248/f2p0/728	2270.686667	858.4833333	764.8833333	-1.2602	2.4144E-07	3.1917E-06	DOWN	-0.29725	0.19577	0.36727		-1.568	7.0428E-14	1.2799E-11	DOWN
transcript_HQ_CoA_transcript30249/f5p0/748	2110.046667	856.65	570.2866667	-1.1268	4.9765E-06	5.0155E-05	DOWN	-0.72938	0.19202	0.36212		-1.8719	0.00029991	0.0034378	DOWN
transcript_HQ_CoA_transcript30250/f2p0/733	408.0766667	18.89666667	558.1333333	-4.3192	7.5294E-09	1.33E-07	DOWN	4.7705	2.3474E-18	4.4819E-16	UP	0.44423	0.44668	0.65705	
transcript_HQ_CoA_transcript30254/f3p0/735	1513.336667	387.8466667	1621.863333	-1.8191	3.8104E-16	1.7636E-14	DOWN	1.9174	7.9727E-08	1.9963E-06	UP	0.08792	0.79628	0.90254	
transcript_HQ_CoA_transcript30257/f2p0/722	411.9266667	817.6466667	608.3966667	1.156	3.2678E-05	0.00026611	UP	-0.57725	0.00049232	0.0036081		0.5651	0.022937	0.09133	
transcript_HQ_CoA_transcript30265/f2p0/655	0.383333333	12757.74	946.2533333	15.078	1.506E-27	1.6687E-25	UP	-3.8972	0.081686	0.19703		11.164	0.0031416	0.0215	UP
transcript_HQ_CoA_transcript30267/f2p0/620	896.9966667	3635.983333	590.7166667	2.1933	3.2538E-18	1.781E-16	UP	-2.7774	2.5587E-42	4.6899E-39	DOWN	-0.59843	0.0067287	0.037873	
transcript_HQ_CoA_transcript30273/f3p0/589	4.896666667	39930.32333	2122.593333	13.174	5.0453E-23	3.8115E-21	UP	-4.3964	0.058595	0.15445		8.7665	0.0061872	0.035758	UP
transcript_HQ_CoA_transcript30277/f3p0/563	2.823333333	52741.39333	2708.583333	14.37	6.9553E-27	7.0252E-25	UP	-4.4443	0.0191	0.066283		9.9151	8.9014E-06	0.00019222	UP
transcript_HQ_CoA_transcript30279/f3p0/555	2488.2	927.1866667	2593.58	-1.2883	3.1323E-09	5.9404E-08	DOWN	1.3552	1.2547E-05	0.0001649	UP	0.05704	0.85493	0.93405	
transcript_HQ_CoA_transcript304/f5p0/6894	3.91	10.41	7.406666667	1.575	0.011543	0.0396	UP	-0.63114	0.019412	0.067156		0.92849	0.11264	0.27752	
transcript_HQ_CoA_transcript3042/f4p0/4425	10.04666667	2.57	6.03	-1.844	0.00039545	0.002315	DOWN	1.0972	0.026914	0.086153		-0.75741	0.012227	0.058157	
transcript_HQ_CoA_transcript3048/f8p0/4383	3.123333333	79.75666667	28.69666667	4.8348	1.352E-34	2.2677E-32	UP	-1.5961	0.13741	0.28659		3.2234	0.0048764	0.029965	UP
transcript_HQ_CoA_transcript3050/f2p0/4406	12.43666667	2.15	5.676666667	-2.3747	3.0005E-06	3.1671E-05	DOWN	1.2261	0.0046969	0.022306	UP	-1.1568	0.0092989	0.047743	DOWN
transcript_HQ_CoA_transcript3070/f2p0/4409	6.15	1.883333333	4.093333333	-1.5659	2.6899E-07	3.5202E-06	DOWN	0.97716	0.0009527	0.0061421		-0.59961	0.019141	0.080661	
transcript_HQ_CoA_transcript3089/f3p0/4430	7.743333333	14.1	8.57	1.0114	2.1801E-05	0.00018555	UP	-0.86555	8.2323E-08	2.0585E-06		0.13476	0.58612	0.76322	
transcript_HQ_CoA_transcript309/f2p0/6955	0.53	1.47	0.75	1.6372	0.00036665	0.0021701	UP	-1.1202	0.013787	0.051652		0.50668	0.35912	0.5763	
transcript_HQ_CoA_transcript3097/f2p0/4403	4.853333333	10.07666667	8.713333333	1.2155	0.00092687	0.004818	UP	-0.38028	0.23702	0.41833		0.82409	0.0077251	0.041679	
transcript_HQ_CoA_transcript3101/f2p0/4403	3.383333333	0.016666667	2.31	-7.7829	1.6507E-05	0.00014488	DOWN	7.2487	5.0459E-11	2.5762E-09	UP	-0.58873	NA	NA	
transcript_HQ_CoA_transcript3124/f4p0/4384	1.08	3.156666667	3.086666667	1.7053	2.8918E-08	4.6133E-07	UP	-0.19151	0.5318	0.70344		1.5007	4.6317E-05	0.00075035	UP
transcript_HQ_CoA_transcript3132/f2p0/4418	4.98	10.05333333	6.146666667	1.1728	0.0039243	0.016305	UP	-0.86639	0.015532	0.056552		0.29524	0.37382	0.59069	
transcript_HQ_CoA_transcript3135/f2p0/4400	2.43	5.636666667	3.476666667	1.3785	0.0028974	0.01263	UP	-0.8649	0.023426	0.077475		0.50291	0.14338	0.32362	
transcript_HQ_CoA_transcript3154/f3p0/4400	3.846666667	7.773333333	2.51	1.1461	0.01181	0.040277	UP	-1.7631	4.9262E-05	0.00053617	DOWN	-0.62794	0.029762	0.11115	
transcript_HQ_CoA_transcript3157/f2p0/4355	13.42	4.11	3.506666667	-1.5842	0.014321	0.046886	DOWN	-0.37208	0.23697	0.41832		-1.9642	0.001829	0.014274	DOWN
transcript_HQ_CoA_transcript3161/f2p0/4379	4.646666667	1.98	6.09	-1.0798	9.6741E-05	0.0006826	DOWN	1.4692	1.0457E-11	6.0848E-10	UP	0.37671	0.106	0.2667	
transcript_HQ_CoA_transcript3169/f3p0/4401	2.446666667	13.74	2.476666667	2.6494	2.1521E-13	7.4237E-12	UP	-2.6382	5.4265E-17	8.0863E-15	DOWN	6.7999E-06	0.99998	1	
transcript_HQ_CoA_transcript3178/f2p0/4373	6.783333333	2.103333333	4.04	-1.5474	2.9735E-08	4.7312E-07	DOWN	0.79242	0.018068	0.063478		-0.76588	0.006513	0.037049	
transcript_HQ_CoA_transcript3183/f2p0/4396	7.996666667	2.183333333	8.09	-1.6863	0.0077049	0.028394	DOWN	1.6984	0.00557	0.02551	UP	0.00061696	0.99797	0.99883	
transcript_HQ_CoA_transcript3185/f9p0/4270	0.47	7.383333333	7.52	4.1191	3.9416E-36	7.0647E-34	UP	-0.074851	0.94069	0.96859		4.0315	5.4283E-05	0.00085745	UP
transcript_HQ_CoA_transcript3193/f2p0/4372	2.676666667	0.476666667	0.436666667	-2.3303	3.6023E-07	4.5958E-06	DOWN	-0.26451	0.6417	0.78653		-2.6087	1.7059E-11	1.8311E-09	DOWN
transcript_HQ_CoA_transcript3205/f2p0/4354	6.116666667	2.316666667	1.84	-1.2516	3.7692E-05	0.00030065	DOWN	-0.48023	0.087199	0.2066		-1.7421	5.1341E-08	2.435E-06	DOWN
transcript_HQ_CoA_transcript3208/f3p0/4336	9.67	4.073333333	4.783333333	-1.0942	2.9004E-06	3.0686E-05	DOWN	0.08432	0.65327	0.79487		-1.0219	5.5369E-06	0.0001308	DOWN
transcript_HQ_CoA_transcript3209/f2p0/4348	0.183333333	0.62	0.02	1.9222	0.0058358	0.022513	UP	-5.0742	5.6458E-06	8.3051E-05	DOWN	-3.1784	0.029846	0.11139	
transcript_HQ_CoA_transcript3216/f3p0/4324	9.78	0.766666667	0.126666667	-3.5504	1.0691E-09	2.216E-08	DOWN	-2.6644	0.029148	0.091325		-6.2482	8.2133E-20	3.4263E-17	DOWN
transcript_HQ_CoA_transcript3227/f3p0/4394	1.923333333	4.1	1.776666667	1.2469	0.0074426	0.027538	UP	-1.3495	0.013398	0.050549		-0.11374	0.84912	0.93111	
transcript_HQ_CoA_transcript3229/f6p0/4345	17.46333333	3.76	3.503333333	-2.0092	0.0027188	0.011983	DOWN	-0.27478	0.72489	0.84322		-2.3015	2.8001E-07	1.0619E-05	DOWN
transcript_HQ_CoA_transcript3230/f2p0/4357	2.733333333	5.57	7.37	1.2056	0.0026489	0.011717	UP	0.25006	0.41246	0.60234		1.4405	0.00014311	0.0019049	UP
transcript_HQ_CoA_transcript3233/f3p0/4368	9.03	17	8.71	1.0587	0.0025183	0.011218	UP	-1.0949	0.00024459	0.0020148	DOWN	-0.048299	0.86768	0.94117	
transcript_HQ_CoA_transcript3234/f2p0/4359	1.42	13.62666667	3.53	3.4078	1.6237E-09	3.2301E-08	UP	-2.093	9.802E-06	0.00013378	DOWN	1.3065	0.0069082	0.038541	UP
transcript_HQ_CoA_transcript3240/f2p0/4346	9.926666667	3.853333333	7.29	-1.1988	0.00013548	0.000918	DOWN	0.76435	0.0016502	0.0095297		-0.44812	0.10609	0.26672	
transcript_HQ_CoA_transcript3263/f2p0/4358	8.363333333	20	8.423333333	1.4088	0.0024119	0.010837	UP	-1.3979	0.0019279	0.010784	DOWN	-0.0011083	0.99762	0.9986	
transcript_HQ_CoA_transcript3267/f2p0/4351	3.27	1.273333333	1.656666667	-1.21	0.0027484	0.012084	DOWN	0.24061	0.57945	0.74219		-0.98352	0.011084	0.05434	
transcript_HQ_CoA_transcript3279/f5p0/4321	13.61333333	28.93333333	17.25666667	1.2631	0.00004764	0.00036811	UP	-0.91089	0.0018213	0.010323		0.33896	0.058639	0.17847	
transcript_HQ_CoA_transcript3289/f2p0/4409	13.39666667	4.536666667	5.08	-1.4002	6.9248E-06	6.7846E-05	DOWN	0.00056471	0.99864	0.99937		-1.4101	7.9212E-07	2.5799E-05	DOWN
transcript_HQ_CoA_transcript3316/f2p0/4328	43.06333333	9.393333333	15.26333333	-2.0282	0.00051574	0.002902	DOWN	0.54245	0.30883	0.5		-1.4983	1.3645E-05	0.00027433	DOWN
transcript_HQ_CoA_transcript3330/f5p0/4338	1.356666667	0.27	0.703333333	-2.1477	0.0060072	0.023067	DOWN	1.2014	0.14054	0.29101		-0.95613	0.031863	0.11641	
transcript_HQ_CoA_transcript3333/f2p0/4422	27.16333333	9.926666667	25.44666667	-1.3057	8.9869E-11	2.1965E-09	DOWN	1.2041	8.4024E-05	0.00083518	UP	-0.11241	0.70204	0.84289	
transcript_HQ_CoA_transcript336/f2p0/6833	10.84	0.01	0.023333333	-10.104	3.9786E-08	6.1694E-07	DOWN	1.4289	0.46209	0.64726		-8.6918	5.5971E-05	0.00088033	DOWN
transcript_HQ_CoA_transcript3367/f3p0/4261	0.51	3.99	4.193333333	3.1335	2.9363E-15	1.2631E-13	UP	-0.056888	0.89382	0.94317		3.0651	6.4897E-10	4.8031E-08	UP
transcript_HQ_CoA_transcript3372/f2p0/4362	4.113333333	8.523333333	2.746666667	1.2073	0.00012907	0.0008785	UP	-1.7794	4.119E-10	1.7848E-08	DOWN	-0.58572	0.092358	0.24294	
transcript_HQ_CoA_transcript3377/f5p0/4323	19.73	35.19333333	23.79666667	1.0006	8.0317E-06	7.7609E-05	UP	-0.71971	0.0023327	0.012579		0.26795	0.12426	0.29557	
transcript_HQ_CoA_transcript3380/f4p0/4302	7.74	36.20666667	62.11333333	2.4112	1.5916E-07	2.178E-06	UP	0.66737	0.62248	0.77389		3.0621	0.020926	0.085891	
transcript_HQ_CoA_transcript3386/f2p0/4330	11.19666667	2.196666667	6.463333333	-2.1901	2.3791E-05	0.00019997	DOWN	1.3867	0.00023952	0.0019794	UP	-0.81368	0.041302	0.14026	
transcript_HQ_CoA_transcript3388/f2p0/4302	5.843333333	1.133333333	3.13	-2.2287	0.00057549	0.0031853	DOWN	1.3359	0.013153	0.049799	UP	-0.90218	0.15533	0.34067	
transcript_HQ_CoA_transcript3396/f2p0/4285	6.373333333	1.886666667	4.223333333	-1.579	0.0047589	0.01905	DOWN	0.97263	0.090925	0.21298		-0.61652	0.1133	0.27864	
transcript_HQ_CoA_transcript3401/f2p0/4360	5.486666667	0.983333333	3.503333333	-2.347	9.2265E-05	0.00065736	DOWN	1.6851	7.4499E-08	1.8834E-06	UP	-0.66871	0.2261	0.43326	
transcript_HQ_CoA_transcript3404/f3p0/4331	3.823333333	20.73666667	4.07	2.5827	5.9384E-09	1.0654E-07	UP	-2.4778	7.0286E-09	2.2601E-07	DOWN	0.090829	0.79589	0.90232	
transcript_HQ_CoA_transcript3413/f2p0/4329	4.993333333	10.27333333	5.5	1.2098	0.00036911	0.0021838	UP	-1.064	0.0015893	0.0092397	DOWN	0.13403	0.53427	0.72692	
transcript_HQ_CoA_transcript3425/f2p0/4290	0.686666667	8.59	5.31	3.8072	0.0001455	0.00097648	UP	-0.80693	0.34138	0.53338		2.9823	2.6581E-06	7.2068E-05	UP
transcript_HQ_CoA_transcript3426/f2p0/4278	19.29666667	39.64	46.63	1.1993	2.4815E-06	2.6797E-05	UP	0.089056	0.50119	0.67866		1.2748	7.584E-08	3.4226E-06	UP
transcript_HQ_CoA_transcript3437/f2p0/4315	3.35	0.82	1.19	-1.9017	0.00072487	0.0038783	DOWN	0.42317	0.45946	0.64523		-1.4885	0.0088526	0.046096	DOWN
transcript_HQ_CoA_transcript3445/f2p0/4292	9.386666667	1.71	2.253333333	-2.3091	1.7529E-05	0.00015267	DOWN	0.2345	0.62244	0.77389		-2.0856	4.1959E-06	0.00010389	DOWN
transcript_HQ_CoA_transcript3483/f2p0/4271	2.15	11.50666667	6.26	2.5506	1.197E-09	2.4423E-08	UP	-1.0074	0.0022702	0.0123	DOWN	1.5332	6.4569E-05	0.00099594	UP

transcript_HQ_CoA_transcript3484/f2p0/4267	1.206666667	2.44	1.43	1.1613	0.0013784	0.006774	UP	-0.92689	0.0029784	0.015361		0.2222	0.56193	0.74583	
transcript_HQ_CoA_transcript3492/f2p0/4247	10.20666667	21.05333333	7.396666667	1.2055	0.0079163	0.029027	UP	-1.6455	1.2393E-06	2.2739E-05	DOWN	-0.45449	0.26467	0.47665	
transcript_HQ_CoA_transcript3497/f2p0/4254	19.24666667	38.1	31.54333333	1.1344	0.0049337	0.019642	UP	-0.41677	0.25821	0.44275		0.70524	0.019997	0.083138	
transcript_HQ_CoA_transcript3512/f2p0/4255	9.42	32.17333333	7.623333333	1.9368	1.5356E-07	2.1092E-06	UP	-2.2168	9.3332E-07	1.8026E-05	DOWN	-0.29451	0.4111	0.62519	
transcript_HQ_CoA_transcript3513/f3p0/4233	2.683333333	9.413333333	5.836666667	1.9722	9.851E-08	1.4037E-06	UP	-0.842	0.043661	0.12304		1.1176	0.008121	0.043306	UP
transcript_HQ_CoA_transcript3529/f11p0/4220	4.193333333	7.826666667	5.256666667	1.0687	0.011785	0.040219	UP	-0.72766	0.0070466	0.030513		0.32477	0.46149	0.66967	
transcript_HQ_CoA_transcript3535/f8p0/4233	97.78333333	38.92666667	58.23666667	-1.1899	5.7251E-05	0.00043346	DOWN	0.43795	0.065446	0.16758		-0.76258	0.0013184	0.011162	
transcript_HQ_CoA_transcript3538/f2p0/4245	2.566666667	0.23	3.01	-3.3507	0.00016296	0.0010755	DOWN	3.556	9.188E-07	1.7783E-05	UP	0.19874	0.8042	0.90726	
transcript_HQ_CoA_transcript3569/f3p0/4256	4.783333333	0.616666667	7.86	-2.7985	5.5157E-11	1.389E-09	DOWN	3.5443	5.9213E-05	0.00061807	UP	0.72994	0.37298	0.5898	
transcript_HQ_CoA_transcript3573/f3p0/4229	26.36333333	11.76666667	21.99	-1.0164	2.2756E-07	3.0279E-06	DOWN	0.75455	0.00054849	0.0039209		-0.27341	0.18006	0.37514	
transcript_HQ_CoA_transcript3575/f3p0/4245	4.016666667	8.43	5.83	1.2303	0.00069519	0.0037403	UP	-0.69504	0.038334	0.1115		0.52402	0.042398	0.1429	
transcript_HQ_CoA_transcript3577/f4p0/4244	5.116666667	1.44	4.843333333	-1.6826	0.00047799	0.0027172	DOWN	1.604	0.0001005	0.00097105	UP	-0.093048	0.84448	0.92856	
transcript_HQ_CoA_transcript3581/f2p0/4240	1.4	4.553333333	2.303333333	1.8607	5.4391E-06	5.4486E-05	UP	-1.1405	0.0058511	0.026553	DOWN	0.70614	0.059317	0.18011	
transcript_HQ_CoA_transcript3594/f4p0/4221	3.873333333	9.783333333	3.786666667	1.4963	0.00010744	0.00074914	UP	-1.5166	7.3288E-05	0.00074256	DOWN	-0.032147	0.90684	0.95854	
transcript_HQ_CoA_transcript360/f2p0/6798	3.91	7.96	2.15	1.1676	0.011474	0.039417	UP	-2.0102	8.1337E-06	0.00011337	DOWN	-0.85569	0.0040258	0.026001	
transcript_HQ_CoA_transcript3602/f3p0/4274	1.646666667	18.78333333	3.5	3.6479	0.013934	0.045931	UP	-2.5391	0.048099	0.13249		1.0952	0.12893	0.30213	
transcript_HQ_CoA_transcript3603/f4p0/4220	9.02	17.76333333	8.546666667	1.1445	0.0032698	0.013974	UP	-1.2179	0.0025746	0.013644	DOWN	-0.087277	0.7101	0.84784	
transcript_HQ_CoA_transcript3606/f2p0/4231	4.276666667	0.993333333	4.38	-1.9438	2.1985E-07	2.9316E-06	DOWN	1.9905	3.9601E-14	3.9448E-12	UP	0.031332	0.91973	0.96458	
transcript_HQ_CoA_transcript3613/f3p0/4232	32.62	81.27333333	48.31666667	1.4873	6.2707E-11	1.5661E-09	UP	-0.90147	1.8536E-06	3.2174E-05		0.5715	0.014494	0.065544	
transcript_HQ_CoA_transcript3618/f2p0/4228	2.883333333	7.293333333	5.736666667	1.5038	8.0454E-06	0.0000777	UP	-0.50986	0.09714	0.22376		0.98128	0.00040259	0.0043264	
transcript_HQ_CoA_transcript3620/f2p0/4250	4.046666667	1.726666667	4.013333333	-1.0697	0.0012114	0.0060544	DOWN	1.0612	0.00020869	0.0017817	UP	-0.020074	0.94284	0.97468	
transcript_HQ_CoA_transcript3622/f2p0/4245	14.97	3.6	14.76	-1.8925	8.3084E-10	1.7499E-08	DOWN	1.8837	2.4125E-33	2.2109E-30	UP	-0.02232	0.93673	0.9721	
transcript_HQ_CoA_transcript3624/f2p0/4219	8.376666667	19.83666667	13.77666667	1.3933	1.0656E-05	9.8988E-05	UP	-0.66353	0.061487	0.15997		0.7179	0.085488	0.23004	
transcript_HQ_CoA_transcript3625/f2p0/4233	2.003333333	5.16	4.383333333	1.506	0.0017982	0.0084921	UP	-0.35792	0.62868	0.7775		1.1342	0.14949	0.33243	
transcript_HQ_CoA_transcript3631/f2p0/4220	0.113333333	22.45333333	0.99	7.8191	1.3894E-18	7.7917E-17	UP	-4.6002	0.0005957	0.0042028	DOWN	3.201	0.027092	0.10351	
transcript_HQ_CoA_transcript3649/f2p0/4224	3.056666667	8.493333333	3.626666667	1.6316	4.3216E-06	4.4089E-05	UP	-1.3656	0.00044572	0.0033183	DOWN	0.25097	0.56311	0.7466	
transcript_HQ_CoA_transcript3711/f2p0/4242	2.816666667	7.756666667	3.573333333	1.5948	0.00042609	0.0024635	UP	-1.2708	0.0067697	0.029628	DOWN	0.31545	0.57534	0.75525	
transcript_HQ_CoA_transcript3737/f7p0/4146	5.003333333	1.12	6.533333333	-1.9848	4.9263E-09	8.9793E-08	DOWN	2.3749	7.9894E-14	7.5483E-12	UP	0.37615	0.1334	0.309	
transcript_HQ_CoA_transcript3742/f2p0/4202	10.86333333	4.723333333	7.086666667	-1.0256	0.0049961	0.019848	DOWN	0.42213	0.24088	0.4231		-0.6166	0.0068864	0.038466	
transcript_HQ_CoA_transcript3747/f2p0/4177	1.81	4.696666667	3.03	1.5139	0.00067024	0.0036239	UP	-0.75888	0.091234	0.21346		0.74504	0.12578	0.29781	
transcript_HQ_CoA_transcript3748/f155p0/4098	0.926666667	7.31	4.33	3.0988	0.00010472	0.00073319	UP	-0.85072	0.31988	0.51099		2.2353	4.9647E-05	0.00079656	UP
transcript_HQ_CoA_transcript3754/f4p0/4144	2.89	11.77	25.77333333	2.1792	0.0016833	0.0080285	UP	1.0108	0.40997	0.60013		3.1705	0.0053585	0.032133	UP
transcript_HQ_CoA_transcript3759/f2p0/4187	8.003333333	2.706666667	2.99	-1.4272	0.002	0.0092683	DOWN	-0.005007	0.98644	0.99324		-1.4402	0.0015841	0.012753	DOWN
transcript_HQ_CoA_transcript3767/f4p0/4177	29.55333333	263.0833333	176.3033333	3.2933	2.6655E-09	5.1189E-08	UP	-0.71093	0.16824	0.33005		2.5669	2.0707E-05	0.00038665	UP
transcript_HQ_CoA_transcript3769/f2p0/4186	6.776666667	1.53	4.813333333	-1.9753	5.4488E-07	6.6721E-06	DOWN	1.4797	0.0015697	0.009151	UP	-0.50915	0.24804	0.45788	
transcript_HQ_CoA_transcript3782/f5p0/4099	11.09333333	21.57	10.65666667	1.1163	9.7028E-14	3.4782E-12	UP	-1.1604	7.4611E-08	1.8837E-06	DOWN	-0.056707	0.81045	0.91118	
transcript_HQ_CoA_transcript3804/f3p0/4172	0	0.51	0.396666667	6.9879	0.0061188	0.023432	UP	-0.51088	0.69955	0.8275		6.4665	9.4313E-05	0.0013519	UP
transcript_HQ_CoA_transcript3807/f4p0/4136	0.59	12.47	3.286666667	4.5395	1.4993E-14	6.0241E-13	UP	-2.0399	0.017483	0.061906		2.4826	0.013408	0.062107	
transcript_HQ_CoA_transcript3808/f4p0/4191	20.51	49.38666667	17.74	1.4278	5.8295E-08	8.7213E-07	UP	-1.6381	9.0482E-12	5.3845E-10	DOWN	-0.22273	0.25192	0.46236	
transcript_HQ_CoA_transcript3821/f2p0/4199	3.996666667	1.386666667	3.256666667	-1.3849	0.0042305	0.017289	DOWN	1.0867	0.00015031	0.0013479	UP	-0.31	0.46687	0.67417	
transcript_HQ_CoA_transcript3826/f6p0/4179	17.27333333	6.203333333	7.63	-1.3576	0.0095422	0.033874	DOWN	0.16718	0.70061	0.82824		-1.1988	0.0013725	0.011503	DOWN
transcript_HQ_CoA_transcript3827/f3p0/4260	17.94666667	1.38	9.886666667	-3.4735	1.5216E-05	0.00013483	DOWN	2.6435	0.00015554	0.0013887	UP	-0.84598	0.10557	0.26616	
transcript_HQ_CoA_transcript3851/f6p0/4162	0.053333333	36	1.75	9.5005	1.9122E-43	4.4251E-41	UP	-4.5004	0.0002999	0.0023806	DOWN	4.9797	0.00013493	0.0018157	UP
transcript_HQ_CoA_transcript3859/f2p0/4162	3.043333333	0.786666667	0.653333333	-1.7888	2.7868E-07	3.6366E-06	DOWN	-0.43576	0.36575	0.55791		-2.2391	1.4047E-08	7.7194E-07	DOWN
transcript_HQ_CoA_transcript3862/f2p0/4173	7.626666667	2.22	3.5	-1.6129	3.9857E-09	7.4127E-08	DOWN	0.51108	0.23018	0.41013		-1.1166	0.0026223	0.018683	DOWN
transcript_HQ_CoA_transcript3868/f5p0/4162	17.95333333	37.91333333	31.22	1.2406	9.6658E-09	1.6766E-07	UP	-0.4306	0.015875	0.05754		0.79685	1.7393E-05	0.00033675	
transcript_HQ_CoA_transcript3872/f2p0/4156	3.183333333	0.213333333	1.03	-3.8532	0.00063986	0.0034857	DOWN	2.2409	0.23108	0.41122		-1.626	0.17681	0.37039	
transcript_HQ_CoA_transcript3875/f2p0/4173	10.85	4.173333333	14.17666667	-1.22	0.0031347	0.013481	DOWN	1.611	5.7962E-05	0.00060949	UP	0.37801	0.060821	0.18325	
transcript_HQ_CoA_transcript3881/f5p0/4116	9.433333333	4.16	12.85333333	-1.0396	0.0010923	0.0055423	DOWN	1.487	4.3488E-08	1.1761E-06	UP	0.43638	0.074269	0.20816	
transcript_HQ_CoA_transcript3886/f3p0/4152	23.10333333	9.213333333	13.99	-1.1892	0.00085683	0.0044936	DOWN	0.46327	0.22791	0.40718		-0.73654	0.00085411	0.0078978	
transcript_HQ_CoA_transcript3896/f2p0/4154	12.96	3.396666667	20.37333333	-1.7735	3.0379E-10	6.8313E-09	DOWN	2.4311	3.5574E-17	5.5258E-15	UP	0.64511	0.021425	0.08737	
transcript_HQ_CoA_transcript390/f2p0/6672	5.013333333	1.586666667	11.36	-1.4937	1.5628E-08	2.6021E-07	DOWN	2.7183	1.0187E-05	0.00013825	UP	1.2101	0.031292	0.1151	
transcript_HQ_CoA_transcript3908/f3p0/4146	6.883333333	19.99666667	7.956666667	1.6881	6.9163E-05	0.00051109	UP	-1.4665	0.0010565	0.0066799	DOWN	0.20751	0.44077	0.65165	
transcript_HQ_CoA_transcript3913/f3p0/4040	11.83666667	4.556666667	15.97	-1.2364	0.00054121	0.0030193	DOWN	1.6637	3.1473E-08	8.7937E-07	UP	0.41728	0.21361	0.4183	
transcript_HQ_CoA_transcript3914/f5p0/4126	15.52666667	7.01	10.68333333	-1.004	0.00010094	0.00070871	DOWN	0.46306	0.076567	0.18797		-0.55207	0.0084065	0.044429	
transcript_HQ_CoA_transcript3916/f2p0/4110	2.036666667	5.123333333	1.84	1.4805	0.0053907	0.021103	UP	-1.6319	0.0014924	0.008795	DOWN	-0.16328	0.65706	0.81256	
transcript_HQ_CoA_transcript3919/f2p0/4206	6.116666667	2.436666667	2.256666667	-1.1842	0.0026632	0.011763	DOWN	-0.25383	0.48185	0.66408		-1.4489	9.3959E-05	0.0013514	DOWN
transcript_HQ_CoA_transcript3943/f2p0/4152	4.08	8.81	9.753333333	1.2686	3.4562E-05	0.00027897	UP	-0.0056522	0.97409	0.98651		1.2516	9.5783E-06	0.00020325	UP
transcript_HQ_CoA_transcript3955/f2p0/4145	19.48	7.563333333	10.28	-1.2187	7.7658E-09	1.3691E-07	DOWN	0.30148	0.20916	0.38456		-0.93004	1.8042E-06	5.1825E-05	
transcript_HQ_CoA_transcript3970/f2p0/4129	5.066666667	1.976666667	1.393333333	-1.2231	0.00037442	0.0022081	DOWN	-0.64514	0.037943	0.11067		-1.8788	5.335E-09	3.2641E-07	DOWN
transcript_HQ_CoA_transcript3975/f2p0/4132	9.456666667	17.16666667	14.34	1.0164	0.002016	0.0093237	UP	-0.39898	0.10328	0.23399		0.60314	0.025211	0.097997	
transcript_HQ_CoA_transcript3979/f3p0/4119	3.223333333	30.59666667	8.646666667	3.4259	5.2925E-15	2.2243E-13	UP	-1.9628	0.00066355	0.004593	DOWN	1.4473	0.014929	0.067065	
transcript_HQ_CoA_transcript3986/f2p0/4117	1.986666667	6.536666667	3.61	1.8599	0.00004205	0.00033079	UP	-0.97838	0.047301	0.13081		0.87006	0.08135	0.22187	
transcript_HQ_CoA_transcript3987/f2p0/4107	6.01	1.746666667	2.3	-1.6561	0.00026004	0.0016094	DOWN	0.26259	0.48204	0.66414		-1.4024	0.00015389	0.0020191	DOWN
transcript_HQ_CoA_transcript4020/f9p0/4095	10.99666667	31.12	11.39333333	1.6645	4.3153E-10	9.4936E-09	UP	-1.6008	2.5344E-07	5.7116E-06	DOWN	0.050852	0.85403	0.93374	
transcript_HQ_CoA_transcript404/f3p0/6608	1.766666667	5.226666667	5.466666667	1.7101	0.0011532	0.0057935	UP	-0.076252	0.85789	0.92246		1.6196	0.00024441	0.0029264	UP
transcript_HQ_CoA_transcript4051/f2p0/4136	2.66	6.576666667	3.683333333	1.4546	9.6937E-06	9.1162E-05	UP	-0.98474	0.0027526	0.014367		0.45742	0.1327	0.3079	
transcript_HQ_CoA_transcript4056/f6p0/4072	16.46333333	6.773333333	22.21666667	-1.1113	0.006769	0.02549	DOWN	1.5431	5.3528E-05	0.00057023	UP	0.42072	0.086221	0.23134	
transcript_HQ_CoA_transcript4062/f3p0/4102	18.19	7.626666667	12.80666667	-1.1032	1.8526E-05	0.00016022	DOWN	0.58826	0.015205	0.055673		-0.52598	0.056172	0.17346	
transcript_HQ_CoA_transcript4066/f4p0/4090	11.09666667	23.6	12.73666667	1.2294	2.2826E-06	2.4855E-05	UP	-1.0312	7.3848E-10	2.9749E-08	DOWN	0.18761	0.44491	0.65536	
transcript_HQ_CoA_transcript4106/f3p0/4109	4.58	1.773333333	1.95	-1.2346	0.010904	0.037805	DOWN	0.032581	0.96262	0.97989		-1.2146	0.036208	0.12769	
transcript_HQ_CoA_transcript4113/f2p0/4135	1.556666667	4.293333333	4.01	1.6254	3.5466E-05	0.00028538	UP	-0.25231	0.21633	0.39298		1.3602	0.00031732	0.0035976	UP
transcript_HQ_CoA_transcript4136/f2p0/4129	3.93	1.406666667	3.436666667	-1.3533	0.0080692	0.029504	DOWN	1.1396	0.0014341	0.0085318	UP	-0.22351	0.65634	0.81231	
transcript_HQ_CoA_transcript4142/f2p0/4091	8.14	2.983333333	5.666666667	-1.3062	0.00022153	0.0014038	DOWN	0.78625	0.0098711	0.039625		-0.53161	0.03111	0.11469	
transcript_HQ_CoA_transcript4149/f2p0/4075	5.73	2.493333333	3.586666667	-1.0525	0.0061753	0.023618	DOWN	0.39021	0.24436	0.42712		-0.67406	0.032772	0.11883	
transcript_HQ_CoA_transcript4151/f2p0/4090	1.56	4.786666667	4.846666667	1.7577	1.3543E-05	0.00012203	UP	-0.10671	0.80665	0.89297		1.6405	0.00037881	0.0041511	UP
transcript_HQ_CoA_transcript4158/f3p0/4092	1.046666667	4.876666667	0.99	2.3729	0.00080022	0.0042356	UP	-2.4155	0.00014853	0.0013365	DOWN	-0.054813	0.93517	0.97162	
transcript_HQ_CoA_transcript4161/f2p0/4179	3.55	0.53	4.236666667	-2.5649	0.00086826	0.004547	DOWN	2.841	7.3352E-10	2.9614E-08	UP	0.25845	0.705	0.84484	
transcript_HQ_CoA_transcript4181/f2p0/4076	25.71666667	4.416666667	3.176666667	-2.3985	4.6809E-10	1.0236E-08	DOWN	-0.60711	0.34369	0.53591		-3.0194	1.2576E-07	5.2822E-06	DOWN

transcript_HQ_CoA_transcript4186/f3p0/4066	2.033333333	5.383333333	2.99	1.5681	0.0011484	0.0057772	UP	-1.005	0.068157	0.17248		0.55046	0.3391	0.55703	
transcript_HQ_CoA_transcript4193/f2p0/4047	1.193333333	6.446666667	4.8	2.5595	0.00016087	0.0010625	UP	-0.56004	0.3108	0.50222		1.9912	2.0002E-05	0.00037577	UP
transcript_HQ_CoA_transcript4222/f2p0/4060	4.46	1.866666667	5.046666667	-1.1082	0.0010307	0.0052827	DOWN	1.2939	3.6734E-05	0.00041588	UP	0.17562	0.55984	0.74471	
transcript_HQ_CoA_transcript4225/f2p0/4059	19.61333333	7.783333333	13.46666667	-1.1775	0.0027866	0.012226	DOWN	0.65227	0.0084996	0.035358		-0.53942	0.11046	0.2745	
transcript_HQ_CoA_transcript4231/f3p0/4055	9.02	0.466666667	5.133333333	-4.1243	1.1067E-27	1.2489E-25	DOWN	3.2954	1.5159E-10	7.0698E-09	UP	-0.83923	0.061509	0.18432	
transcript_HQ_CoA_transcript4239/f3p0/4054	12.32666667	3.843333333	3.756666667	-1.5291	6.9992E-08	1.0303E-06	DOWN	-0.17605	0.65614	0.79724		-1.7159	5.1716E-07	1.7944E-05	DOWN
transcript_HQ_CoA_transcript4256/f5p0/4028	12.19	21.72333333	19.63333333	1.0025	3.9276E-05	0.00031151	UP	-0.30189	0.24579	0.42865		0.68708	0.001225	0.010521	
transcript_HQ_CoA_transcript4260/f2p0/4063	0.803333333	8.673333333	0.6	3.5653	6.4021E-05	0.00047793	UP	-4.0095	3.6817E-12	2.3678E-10	DOWN	-0.45349	0.64093	0.80045	
transcript_HQ_CoA_transcript4279/f3p0/4049	2.513333333	9.75	1.483333333	2.0966	1.7181E-05	0.00014979	UP	-2.8599	1.5386E-09	5.7909E-08	DOWN	-0.7742	0.023032	0.091526	
transcript_HQ_CoA_transcript4280/f2p0/4089	23.87333333	3.173333333	12.04333333	-2.7693	6.4754E-11	1.6085E-09	DOWN	1.7667	4.9475E-10	2.0847E-08	UP	-1.0127	0.016804	0.072955	
transcript_HQ_CoA_transcript4284/f2p0/4051	3.366666667	1.34	1.913333333	-1.1789	0.012453	0.042055	DOWN	0.35634	0.3585	0.55037		-0.83243	0.029882	0.11146	
transcript_HQ_CoA_transcript4287/f2p0/4069	1.01	12.45666667	6.066666667	3.7937	1.1055E-12	3.5089E-11	UP	-1.2055	0.0090125	0.036997	DOWN	2.5755	3.1892E-07	0.00001185	UP
transcript_HQ_CoA_transcript430/f3p0/6539	0.536666667	6.65	5.886666667	3.7538	6.2796E-11	1.5662E-09	UP	-0.31776	0.45011	0.63757		3.4265	2.3208E-12	3.2029E-10	UP
transcript_HQ_CoA_transcript4304/f3p0/4056	0.453333333	2.126666667	0.713333333	2.3492	0.0042157	0.017242	UP	-1.6927	0.057672	0.15258		0.64652	0.48867	0.69098	
transcript_HQ_CoA_transcript4309/f4p0/4033	1.64	0.166666667	0.053333333	-3.2405	0.0098411	0.034786	DOWN	-1.6983	0.38364	0.57609		-4.9641	1.8761E-05	0.00035635	DOWN
transcript_HQ_CoA_transcript431/f2p0/6485	18.49	70.14666667	22.8	2.0777	1.312E-21	9.1201E-20	UP	-1.7679	4.9752E-23	1.9824E-20	DOWN	0.29722	0.22348	0.43025	
transcript_HQ_CoA_transcript4310/f2p0/4031	3.106666667	1.326666667	1.593333333	-1.0933	0.013161	0.043874	DOWN	0.12061	0.79512	0.88708		-0.98179	0.014093	0.064206	
transcript_HQ_CoA_transcript4324/f2p0/4034	16.96	7.123333333	5.163333333	-1.097	1.4419E-06	1.6435E-05	DOWN	-0.61544	0.022697	0.075707		-1.725	3.1551E-11	3.1474E-09	DOWN
transcript_HQ_CoA_transcript4327/f3p0/4050	11.74666667	4.643333333	5.946666667	-1.2008	0.00070671	0.0037898	DOWN	0.21453	0.48766	0.66823		-0.99632	0.00022675	0.0027555	
transcript_HQ_CoA_transcript4334/f2p0/4119	6.443333333	15.55666667	9.84	1.4251	1.2489E-09	2.537E-08	UP	-0.81358	3.9779E-07	8.5677E-06		0.60061	0.0065321	0.037108	
transcript_HQ_CoA_transcript4336/f2p0/4061	11.04333333	3.033333333	7.24	-1.7047	0.00097817	0.0050531	DOWN	1.0845	0.0071084	0.030722	UP	-0.6304	0.099086	0.2554	
transcript_HQ_CoA_transcript4352/f4p0/4028	3.62	7.693333333	8.513333333	1.2416	0.0091215	0.032647	UP	-0.014875	0.96552	0.98147		1.2154	0.0028686	0.020089	UP
transcript_HQ_CoA_transcript4369/f2p0/4044	5.8	12.21333333	10.50666667	1.2268	2.2997E-05	0.00019428	UP	-0.36638	0.24176	0.42402		0.8479	0.011403	0.055416	
transcript_HQ_CoA_transcript4376/f11p0/3999	0.39	3.696666667	1.81	3.3455	0.001383	0.0067856	UP	-1.1109	0.28557	0.47446		2.221	0.0038343	0.025082	UP
transcript_HQ_CoA_transcript4386/f2p0/4024	0.526666667	2.433333333	1.293333333	2.3456	1.3974E-05	0.00012548	UP	-1.0405	0.014087	0.052531		1.2915	0.011313	0.055183	
transcript_HQ_CoA_transcript4396/f2p0/4030	1.77	0.396666667	1.363333333	-1.9709	0.0049804	0.019798	DOWN	1.5797	0.0047695	0.022557	UP	-0.39967	0.47275	0.67866	
transcript_HQ_CoA_transcript4404/f2p0/4046	1.076666667	5.43	14.59666667	2.488	2.1938E-18	1.2117E-16	UP	1.2727	9.1813E-05	0.00089799	UP	3.7496	6.9344E-25	6.3641E-22	UP
transcript_HQ_CoA_transcript4410/f5p0/3957	5.08	18.12	22.48333333	1.9914	6.7784E-06	6.6554E-05	UP	0.16505	0.62046	0.77238		2.144	2.2593E-07	8.8421E-06	UP
transcript_HQ_CoA_transcript4414/f3p0/4049	19.42333333	6.48	12.01666667	-1.429	6.6751E-11	1.6513E-09	DOWN	0.75064	0.00021344	0.0018163		-0.69156	0.00032169	0.003638	
transcript_HQ_CoA_transcript4421/f2p0/4038	11.00666667	2.793333333	3.103333333	-1.8575	0.0024329	0.010907	DOWN	0.032832	0.96185	0.97976		-1.8323	4.7947E-08	2.2859E-06	DOWN
transcript_HQ_CoA_transcript4434/f3p0/4047	18.04333333	73.92	23.12666667	2.1986	5.185E-12	1.5143E-10	UP	-1.8256	2.3857E-07	5.4319E-06	DOWN	0.35989	0.18193	0.37757	
transcript_HQ_CoA_transcript4437/f2p0/4037	6.66	2.556666667	1.14	-1.2097	0.00032762	0.001967	DOWN	-1.3081	0.01476	0.054401		-2.5336	1.1402E-06	3.5411E-05	DOWN
transcript_HQ_CoA_transcript4452/f4p0/3983	0.8	2.086666667	2.853333333	1.5336	0.0071359	0.026592	UP	0.31095	0.68071	0.8143		1.8327	0.016514	0.072048	
transcript_HQ_CoA_transcript4459/f2p0/4013	4.21	1.87	1.976666667	-1.0099	0.01106	0.038267	DOWN	-0.059688	0.87244	0.93084		-1.0844	0.0049153	0.030144	DOWN
transcript_HQ_CoA_transcript448/f2p0/6457	1.743333333	7.86	1.52	2.2988	0.00001034	9.6443E-05	UP	-2.5033	2.2794E-14	2.3738E-12	DOWN	-0.21217	0.69444	0.83796	
transcript_HQ_CoA_transcript4488/f2p0/4020	20.41666667	5.16	10.93	-1.8071	0.0012325	0.0061464	DOWN	0.89965	0.10678	0.23935		-0.91842	0.00022873	0.0027748	
transcript_HQ_CoA_transcript4499/f2p0/4040	1.836666667	3.386666667	1.17	1.0358	0.0075277	0.02783	UP	-1.6778	5.8349E-07	1.2001E-05	DOWN	-0.65327	0.10359	0.26302	
transcript_HQ_CoA_transcript4506/f2p0/3998	6.11	12.64666667	7.256666667	1.2077	2.2911E-10	5.2612E-09	UP	-0.95263	6.438E-07	1.3053E-05		0.24203	0.23359	0.44193	
transcript_HQ_CoA_transcript4510/f3p0/4001	9.83	31.12666667	9.553333333	1.8442	0.0026047	0.011552	UP	-1.8552	9.3188E-14	8.6317E-12	DOWN	-0.026078	0.96577	0.98521	
transcript_HQ_CoA_transcript4515/f4p0/4002	24.94666667	7.856666667	8.963333333	-1.5329	2.6408E-05	0.00021955	DOWN	0.050536	0.82876	0.9065		-1.492	3.1465E-06	8.2339E-05	DOWN
transcript_HQ_CoA_transcript4529/f2p0/3991	1.173333333	0.086666667	1.54	-3.6692	0.00018972	0.0012278	DOWN	4.0482	4.9601E-06	7.4336E-05	UP	0.37243	0.4789	0.68358	
transcript_HQ_CoA_transcript4539/f3p0/4016	1.183333333	2.58	1.93	1.263	0.01481	0.048059	UP	-0.54966	0.23843	0.42013		0.69936	0.10178	0.26017	
transcript_HQ_CoA_transcript457/f2p0/6481	7.01	16.70333333	10.96333333	1.4025	0.0018881	0.008835	UP	-0.76441	0.0019417	0.010831		0.62831	0.12457	0.29597	
transcript_HQ_CoA_transcript4574/f2p0/3984	4.64	1.863333333	3.053333333	-1.1634	0.0017066	0.0081294	DOWN	0.56636	0.16304	0.32334		-0.6096	0.074467	0.20858	
transcript_HQ_CoA_transcript4581/f3p0/3951	3.036666667	8.793333333	6.116666667	1.7023	2.8621E-07	3.7215E-06	UP	-0.67605	0.0032515	0.016554		1.0113	0.0060144	0.035112	UP
transcript_HQ_CoA_transcript4587/f2p0/4009	1.053333333	0.343333333	3.27	-1.4699	0.0078506	0.028814	DOWN	3.0916	1.1777E-07	2.8592E-06	UP	1.611	0.0018544	0.014386	UP
transcript_HQ_CoA_transcript4592/f4p0/3982	13.62666667	3.743333333	5.25	-1.7256	7.5563E-08	1.106E-06	DOWN	0.35748	0.35217	0.54412		-1.3775	4.0046E-05	0.00066762	DOWN
transcript_HQ_CoA_transcript4596/f3p0/4066	1.53	5.81	2.296666667	2.1026	2.9372E-05	0.00024177	UP	-1.4738	0.052615	0.14205		0.61189	0.45137	0.66148	
transcript_HQ_CoA_transcript4597/f2p0/3976	5.56	18.61333333	5.926666667	1.9014	5.8388E-07	7.1306E-06	UP	-1.7982	1.757E-06	3.0876E-05	DOWN	0.090877	0.76125	0.88139	
transcript_HQ_CoA_transcript4603/f2p0/4060	4.963333333	2.1	4.476666667	-1.0873	0.003612	0.015209	DOWN	0.93026	0.0058654	0.026591		-0.16809	0.6649	0.81738	
transcript_HQ_CoA_transcript4620/f4p0/3957	0.213333333	3.873333333	1.25	4.3191	6.7455E-15	2.7964E-13	UP	-1.799	8.1303E-06	0.00011337	DOWN	2.5085	2.0118E-05	0.00037757	UP
transcript_HQ_CoA_transcript4623/f3p0/3989	6.63	2.08	9.716666667	-1.5306	2.1875E-05	0.00018602	DOWN	2.0785	9.8191E-19	1.8945E-16	UP	0.53838	0.084786	0.22883	
transcript_HQ_CoA_transcript4653/f2p0/3988	6.023333333	2.22	1.87	-1.29	0.00052278	0.0029345	DOWN	-0.40212	0.14902	0.30331		-1.7043	2.8394E-06	7.5973E-05	DOWN
transcript_HQ_CoA_transcript4654/f2p0/3976	2.196666667	5.356666667	2.92	1.4266	0.01402	0.04614	UP	-0.99474	0.1459	0.29872		0.41691	0.42747	0.63978	
transcript_HQ_CoA_transcript4656/f4p0/3908	0.463333333	6.356666667	0.52	3.9042	3.9065E-05	0.00031011	UP	-3.7692	6.5691E-10	2.6638E-08	DOWN	0.12853	0.91047	0.96028	
transcript_HQ_CoA_transcript466/f3p0/6437	3.606666667	1.04	4.153333333	-1.6307	0.0076135	0.028113	DOWN	1.8502	7.5508E-09	2.4196E-07	UP	0.20405	0.70412	0.84432	
transcript_HQ_CoA_transcript4666/f2p0/4003	9.823333333	4.343333333	6.03	-1.034	0.010125	0.035612	DOWN	0.32898	0.21758	0.39474		-0.71381	0.070286	0.20131	
transcript_HQ_CoA_transcript4672/f2p0/3956	2.486666667	5.146666667	1.936666667	1.2243	0.0042896	0.017462	UP	-1.5818	0.00011421	0.0010724	DOWN	-0.37032	0.27525	0.48856	
transcript_HQ_CoA_transcript469/f2p0/6417	14.67	48.70666667	18.42666667	1.8905	3.4525E-16	1.602E-14	UP	-1.559	8.3263E-18	1.4535E-15	DOWN	0.31935	0.10552	0.26615	
transcript_HQ_CoA_transcript4708/f2p0/3988	52.85666667	5.603333333	12.59666667	-3.1103	5.4248E-07	6.6505E-06	DOWN	1.0307	0.016761	0.060074		-2.0852	0.00039522	0.004269	DOWN
transcript_HQ_CoA_transcript4723/f3p0/3963	6.313333333	28.47	26.34	2.354	9.9696E-09	1.7244E-07	UP	-0.27971	0.39581	0.58606		2.0601	2.3902E-10	1.9412E-08	UP
transcript_HQ_CoA_transcript4725/f2p0/3956	0.593333333	1.356666667	0.203333333	1.3295	0.0082965	0.030233	UP	-2.8763	2.4854E-06	4.1717E-05	DOWN	-1.5526	0.041318	0.14026	
transcript_HQ_CoA_transcript4730/f4p0/3883	25.17333333	72.76333333	28.48333333	1.6883	6.5164E-27	6.6185E-25	UP	-1.5032	4.9541E-32	4.324E-29	DOWN	0.17287	0.28718	0.50152	
transcript_HQ_CoA_transcript4740/f5p0/3927	24.10333333	7.236666667	13.39	-1.5664	2.2965E-05	0.0001941	DOWN	0.72176	0.014997	0.055107		-0.85693	0.0020892	0.01582	
transcript_HQ_CoA_transcript4747/f2p0/3963	0.696666667	0.04	1.73	-4.0905	0.0053718	0.021043	DOWN	5.3958	1.0172E-07	2.5094E-06	UP	1.3149	0.028612	0.10797	
transcript_HQ_CoA_transcript4751/f2p0/3946	52.23666667	18.15333333	17.62	-1.3876	0.0054389	0.021233	DOWN	-0.17327	0.73743	0.85179		-1.5719	1.5443E-06	4.5353E-05	DOWN
transcript_HQ_CoA_transcript4755/f4p0/3911	3.656666667	26.76	7.353333333	3.0379	8.8316E-14	3.1783E-12	UP	-2.003	4.3516E-25	2.2059E-22	DOWN	1.0212	0.015697	0.069644	
transcript_HQ_CoA_transcript4784/f2p0/3921	99.74	44	81.80333333	-1.035	8.7122E-05	0.00062559	DOWN	0.75164	0.015953	0.057763		-0.29469	0.27167	0.48427	
transcript_HQ_CoA_transcript4790/f2p0/3950	1.37	0.353333333	0.986666667	-1.8177	0.0037984	0.015869	DOWN	1.3424	0.010781	0.042578	UP	-0.48604	0.33087	0.54803	
transcript_HQ_CoA_transcript4792/f2p0/3941	12.87666667	1.523333333	5.356666667	-2.9449	1.2962E-11	3.6068E-10	DOWN	1.6887	0.00082201	0.005449	UP	-1.2644	0.00738	0.040376	DOWN
transcript_HQ_CoA_transcript4801/f2p0/3956	5.76	1.776666667	2.353333333	-1.551	0.00098052	0.0050624	DOWN	0.26653	0.55566	0.72319		-1.2966	2.9876E-05	0.0005288	DOWN
transcript_HQ_CoA_transcript4816/f2p0/3926	10.32333333	0.6	4.663333333	-3.976	2.4256E-10	5.55E-09	DOWN	2.8103	1.3719E-11	7.9076E-10	UP	-1.1711	0.047912	0.15562	
transcript_HQ_CoA_transcript4819/f3p0/3927	11.26	4.92	7.06	-1.0561	0.00087197	0.0045651	DOWN	0.38506	0.20474	0.3789		-0.6828	0.0011102	0.0097224	
transcript_HQ_CoA_transcript4826/f3p0/3935	3.873333333	7.743333333	4.22	1.1436	6.7957E-05	0.00050279	UP	-1.0217	0.0002548	0.0020849	DOWN	0.10982	0.73082	0.86243	
transcript_HQ_CoA_transcript4827/f2p0/3918	15.33	4.56	9.58	-1.6084	1.0021E-08	1.73E-07	DOWN	0.92355	0.0020179	0.011193		-0.69501	0.015989	0.070478	
transcript_HQ_CoA_transcript4828/f2p0/3953	2.206666667	6.03	4.353333333	1.5965	1.3754E-07	1.9064E-06	UP	-0.61977	0.044489	0.12478		0.96593	0.0071346	0.039385	
transcript_HQ_CoA_transcript4833/f2p0/3950	15.98666667	4.956666667	8.42	-1.5352	4.2905E-09	7.9311E-08	DOWN	0.62179	0.0087798	0.036261		-0.92687	8.7289E-06	0.00018961	
transcript_HQ_CoA_transcript4834/f2p0/3940	5.296666667	2.033333333	2.496666667	-1.2342	0.0024297	0.010901	DOWN	0.15528	0.70852	0.83289		-1.0912	0.0003911	0.0042427	DOWN
transcript_HQ_CoA_transcript4839/f7p0/3888	2.453333333	27.01333333	9.786666667	3.6365	6.0142E-17	3.0124E-15	UP	-1.6219	2.2347E-05	0.00026877	DOWN	2.0004	7.4168E-12	8.7267E-10	UP

transcript_HQ_CoA_transcript4845/f2p0/3936	30.72666667	10.7	20.16	-1.363	8.0789E-08	1.1731E-06	DOWN	0.7615	0.00010726	0.0010207		-0.61376	0.0095334	0.048574	
transcript_HQ_CoA_transcript4854/f15p0/3854	42.78333333	18.61666667	33.20666667	-1.0414	1.0019E-08	1.73E-07	DOWN	0.67317	0.0043627	0.021093		-0.38073	0.066481	0.19409	
transcript_HQ_CoA_transcript486/f2p0/6409	30.22333333	12.9	18.54666667	-1.073	0.00037889	0.0022309	DOWN	0.37389	0.096964	0.22347		-0.71177	0.01629	0.071309	
transcript_HQ_CoA_transcript4861/f2p0/3974	4.143333333	9.82	6.26	1.4102	0.00019356	0.0012495	UP	-0.79848	0.00016125	0.0014334		0.59721	0.11916	0.28734	
transcript_HQ_CoA_transcript4867/f3p0/3930	8.31	0.836666667	11.37333333	-3.1686	5.1723E-19	3.0211E-17	DOWN	3.6212	4.0704E-49	1.2434E-45	UP	0.44101	0.11438	0.28016	
transcript_HQ_CoA_transcript4868/f2p0/3947	8.5	3.04	3.993333333	-1.3161	0.00032509	0.0019557	DOWN	0.23909	0.44894	0.63644		-1.0906	0.0050158	0.030561	DOWN
transcript_HQ_CoA_transcript4885/f3p0/3910	11.40333333	0.88	36.42666667	-3.5432	0.00013301	0.00090261	DOWN	5.2126	5.4305E-10	2.2469E-08	UP	1.6592	0.0010198	0.0090904	UP
transcript_HQ_CoA_transcript4886/f8p0/3896	4.47	10.42666667	25.1	1.3819	0.0054194	0.021161	UP	1.1231	0.00022831	0.0019032	UP	2.4928	9.5863E-08	4.1659E-06	UP
transcript_HQ_CoA_transcript4888/f2p0/3931	2.73	7.246666667	14.99333333	1.5771	0.0024883	0.011106	UP	0.90115	0.020662	0.070316		2.4642	5.4923E-07	1.8949E-05	UP
transcript_HQ_CoA_transcript4890/f2p0/3922	1.433333333	20.18666667	1.663333333	3.9684	1.2706E-08	2.1528E-07	UP	-3.7503	1.2974E-10	6.2091E-09	DOWN	0.20391	0.74645	0.8723	
transcript_HQ_CoA_transcript4891/f2p0/3960	6.956666667	2.18	16.22333333	-1.4957	7.8139E-05	0.00056821	DOWN	2.7287	1.0683E-22	3.9163E-20	UP	1.2186	0.00018693	0.002368	UP
transcript_HQ_CoA_transcript4900/f2p0/3858	259.31	19.68	34.06666667	-3.5839	5.0032E-20	3.1112E-18	DOWN	0.66157	0.081352	0.1964		-2.9325	1.0167E-17	3.3324E-15	DOWN
transcript_HQ_CoA_transcript4904/f5p0/3926	2.486666667	10.3	11.6	2.2211	0.0028373	0.012424	UP	-0.014808	0.97673	0.9878		2.1955	0.00017226	0.0022096	UP
transcript_HQ_CoA_transcript4911/f2p0/3919	21.59666667	8.53	10.66333333	-1.2005	9.0472E-05	0.00064635	DOWN	0.17352	0.56727	0.73279		-1.0373	0.00023289	0.0028178	DOWN
transcript_HQ_CoA_transcript4920/f3p0/3903	1.833333333	4.806666667	2.966666667	1.5521	1.3129E-05	0.00011894	UP	-0.85443	0.0060428	0.027233		0.68455	0.037286	0.13027	
transcript_HQ_CoA_transcript4940/f2p0/3924	15.16	51.43333333	24.56666667	1.9311	1.8029E-12	5.5772E-11	UP	-1.2269	1.1504E-06	2.1363E-05	DOWN	0.6908	0.00018051	0.0022962	
transcript_HQ_CoA_transcript4941/f5p0/3929	1.176666667	3.486666667	1.893333333	1.7275	2.1717E-05	0.00018501	UP	-1.0313	0.0071651	0.030901	DOWN	0.68251	0.058089	0.17723	
transcript_HQ_CoA_transcript495/f2p0/6364	19.31666667	8.093333333	16.84333333	-1.1109	0.015139	0.048916	DOWN	0.90514	0.0014103	0.0084148		-0.21699	0.60746	0.7784	
transcript_HQ_CoA_transcript4959/f3p0/3915	3.286666667	9.036666667	4.113333333	1.6278	7.8282E-08	1.1422E-06	UP	-1.2994	0.00001739	0.0002161	DOWN	0.31599	0.29022	0.50536	
transcript_HQ_CoA_transcript4967/f2p0/3928	0.296666667	2.616666667	2.63	3.2914	0.00097065	0.0050199	UP	-0.1216	0.85337	0.91987		3.1598	0.00019795	0.0024751	UP
transcript_HQ_CoA_transcript4968/f2p0/3902	5.023333333	14.94333333	5.37	1.7209	4.7674E-06	4.8282E-05	UP	-1.6038	0.00017164	0.0015118	DOWN	0.10422	0.78163	0.89382	
transcript_HQ_CoA_transcript4972/f2p0/3897	4.673333333	12.86333333	7.333333333	1.6327	3.732E-07	4.7414E-06	UP	-0.95901	0.01961	0.067652		0.66005	0.083756	0.22678	
transcript_HQ_CoA_transcript4978/f2p0/3893	2.543333333	6.64	3.893333333	1.5484	9.6668E-08	1.3818E-06	UP	-0.91018	0.011736	0.045565		0.6248	0.13797	0.31602	
transcript_HQ_CoA_transcript4981/f2p0/3928	0.28	1.35	1.893333333	2.4157	0.0088167	0.031736	UP	0.3505	0.71185	0.83526		2.7542	0.0123	0.058392	
transcript_HQ_CoA_transcript5005/f2p0/3888	5.613333333	43.38	7.916666667	3.1146	8.9462E-24	7.1735E-22	UP	-2.6097	1.3573E-15	1.6809E-13	DOWN	0.49127	0.059608	0.18054	
transcript_HQ_CoA_transcript5008/f2p0/3937	5.593333333	27.66666667	8.526666667	2.4452	1.3973E-06	1.5985E-05	UP	-1.8193	0.00080332	0.0053581	DOWN	0.61301	0.15319	0.33742	
transcript_HQ_CoA_transcript5014/f2p0/3896	3.47	7.003333333	6.703333333	1.1708	2.6194E-05	0.00021797	UP	-0.18911	0.63621	0.78278		0.96828	0.020588	0.084919	
transcript_HQ_CoA_transcript5016/f2p0/3933	3.106666667	1.056666667	7.41	-1.4162	0.0052529	0.020684	DOWN	2.6577	1.1135E-06	2.0804E-05	UP	1.2314	0.014551	0.065736	
transcript_HQ_CoA_transcript5018/f2p0/3884	2.266666667	0.69	1.23	-1.5368	0.0013102	0.0064842	DOWN	0.66153	0.27167	0.45872		-0.88931	0.12685	0.29921	
transcript_HQ_CoA_transcript5022/f2p0/3942	7.61	3.386666667	7.84	-1.0013	0.0034617	0.014687	DOWN	1.0585	0.00018842	0.0016383	UP	0.045234	0.86943	0.94182	
transcript_HQ_CoA_transcript5024/f2p0/3878	0.216666667	0.976666667	0.38	2.3412	0.0035433	0.014974	UP	-1.5153	0.035045	0.10453		0.81081	0.31988	0.53694	
transcript_HQ_CoA_transcript5029/f2p0/3882	0.303333333	5.21	2.833333333	4.2572	3.3775E-13	1.1372E-11	UP	-1.0013	0.078268	0.19117		3.2429	4.7239E-06	0.00011394	UP
transcript_HQ_CoA_transcript503/f3p0/6379	11.28666667	4.543333333	8.133333333	-1.1744	0.0050038	0.019874	DOWN	0.71023	0.079333	0.19319		-0.47715	0.021548	0.087698	
transcript_HQ_CoA_transcript5033/f2p0/3892	0.15	3.163333333	1.72	4.4984	2.9524E-07	3.8308E-06	UP	-1.0005	0.31591	0.50685		3.4838	0.0040776	0.026239	UP
transcript_HQ_CoA_transcript504/f2p0/6364	2.016666667	3.726666667	3.986666667	1.0355	0.00018175	0.0011842	UP	-0.042448	0.88874	0.94027		0.97962	0.00058748	0.0058605	
transcript_HQ_CoA_transcript5040/f2p0/3941	4.81	1.2	8.123333333	-1.8036	0.0058012	0.022394	DOWN	2.5755	4.9096E-05	0.00053468	UP	0.75657	0.011403	0.055416	
transcript_HQ_CoA_transcript5042/f2p0/3872	2.42	7.373333333	2.243333333	1.7671	0.0017751	0.0083987	UP	-1.8566	7.8529E-05	0.00078439	DOWN	-0.10409	0.82886	0.92043	
transcript_HQ_CoA_transcript5048/f2p0/3893	0.726666667	8.01	0.836666667	3.6133	0.0014019	0.0068693	UP	-3.3804	5.1357E-05	0.00055224	DOWN	0.21669	0.83356	0.9229	
transcript_HQ_CoA_transcript506/f2p0/6371	6.66	19.66	14.40333333	1.6983	0.0037347	0.015657	UP	-0.5732	0.32877	0.51967		1.1137	1.3247E-06	4.0122E-05	UP
transcript_HQ_CoA_transcript5071/f3p0/3866	4.263333333	9.576666667	6.08	1.3231	0.0070452	0.02634	UP	-0.80694	0.042217	0.11993		0.50353	0.32008	0.53707	
transcript_HQ_CoA_transcript5078/f2p0/3948	10.32333333	3.686666667	6.926666667	-1.3218	0.0039322	0.016324	DOWN	0.7542	0.00095738	0.0061675		-0.58196	0.184	0.38033	
transcript_HQ_CoA_transcript5082/f2p0/3874	6.61	2.59	2.903333333	-1.2186	0.0010388	0.0053195	DOWN	0.020701	0.96465	0.98081		-1.2079	0.001059	0.0093949	DOWN
transcript_HQ_CoA_transcript5088/f4p0/3869	6.45	11.61333333	4.473333333	1.0086	0.0027347	0.012038	UP	-1.5384	2.7767E-07	6.1991E-06	DOWN	-0.54195	0.039689	0.13647	
transcript_HQ_CoA_transcript5090/f2p0/3901	2.33	0.3	0.62	-2.7529	3.3327E-05	0.00027032	DOWN	0.87826	0.12795	0.27232		-1.8912	0.0012637	0.010778	DOWN
transcript_HQ_CoA_transcript5102/f2p0/3867	2.216666667	5.313333333	3.99	1.4159	5.37E-07	6.6022E-06	UP	-0.56032	0.011838	0.045847		0.8433	0.0047242	0.029334	
transcript_HQ_CoA_transcript5114/f6p0/3855	46.66	18.74666667	48.85666667	-1.1727	2.5141E-07	3.3091E-06	DOWN	1.233	6.475E-07	1.3114E-05	UP	0.049756	0.83214	0.92228	
transcript_HQ_CoA_transcript5133/f4p0/3869	8.003333333	3.403333333	9.143333333	-1.0862	0.0076124	0.028113	DOWN	1.2682	0.0031738	0.016218	UP	0.17124	0.67147	0.82204	
transcript_HQ_CoA_transcript5149/f2p0/3869	15.57666667	2.083333333	2.98	-2.7671	3.0474E-11	8.0276E-10	DOWN	0.37179	0.16809	0.32982		-2.4038	9.2851E-10	6.6252E-08	DOWN
transcript_HQ_CoA_transcript5162/f2p0/3859	3.673333333	0.83	2.736666667	-1.948	0.0019834	0.0092149	DOWN	1.5337	0.0060484	0.027245	UP	-0.4273	0.3439	0.56174	
transcript_HQ_CoA_transcript5173/f2p0/3858	6.766666667	21.39333333	8.616666667	1.821	7.1964E-10	1.5298E-08	UP	-1.4587	0.44999	0.63754		0.34693	0.85715	0.93513	
transcript_HQ_CoA_transcript5176/f2p0/3855	8.043333333	2.84	5.803333333	-1.346	4.6227E-08	7.084E-07	DOWN	0.87358	2.2953E-05	0.00027461		-0.48468	0.030161	0.11218	
transcript_HQ_CoA_transcript5183/f3p0/3840	9.626666667	3.586666667	4.483333333	-1.2683	0.0014131	0.0069187	DOWN	0.17626	0.46726	0.65158		-1.1051	0.0030827	0.021232	DOWN
transcript_HQ_CoA_transcript5185/f3p0/3854	4.42	1.236666667	3.01	-1.6802	0.00065631	0.0035636	DOWN	1.1447	0.0065276	0.028782	UP	-0.54862	0.19236	0.39082	
transcript_HQ_CoA_transcript5190/f5p0/3836	10.43666667	95.49333333	10.15666667	3.3951	2.0353E-06	2.2444E-05	UP	-3.3642	2.9881E-16	3.9688E-14	DOWN	0.01119	0.98843	0.99541	
transcript_HQ_CoA_transcript520/f4p0/6321	3.636666667	9.623333333	4.876666667	1.5801	0.012527	0.042217	UP	-1.1084	0.056177	0.14975		0.45339	0.55209	0.7392	
transcript_HQ_CoA_transcript5203/f2p0/3860	7.083333333	22.06666667	8.406666667	1.8201	0.00041956	0.0024313	UP	-1.5359	0.0021907	0.011922	DOWN	0.26702	0.67699	0.82633	
transcript_HQ_CoA_transcript5205/f2p0/3864	4.196666667	0.956666667	1.443333333	-1.9654	2.5796E-05	0.00021486	DOWN	0.41091	0.60101	0.75857		-1.5649	0.013878	0.063498	
transcript_HQ_CoA_transcript5219/f12p0/3820	0.946666667	10.91666667	10.16666667	3.7164	7.5875E-12	2.1793E-10	UP	-0.22842	0.8054	0.89234		3.4694	0.00039058	0.0042395	UP
transcript_HQ_CoA_transcript522/f2p0/6395	1.856666667	13.24333333	2.15	2.9828	8.5583E-17	4.2402E-15	UP	-2.7802	3.1843E-17	4.9884E-15	DOWN	0.19067	0.58111	0.75961	
transcript_HQ_CoA_transcript5260/f2p0/3866	2.773333333	0.93	6.543333333	-1.4392	0.0056726	0.021981	DOWN	2.6599	4.1602E-10	1.7942E-08	UP	1.2105	0.017145	0.073993	
transcript_HQ_CoA_transcript5268/f2p0/3830	58.03666667	16.73333333	52.28666667	-1.6522	1.5149E-07	2.084E-06	DOWN	1.4988	7.0994E-07	1.4237E-05	UP	-0.16321	0.62233	0.78827	
transcript_HQ_CoA_transcript527/f2p0/6327	0.93	0.176666667	0.29	-2.2506	0.0021284	0.0097671	DOWN	0.58669	0.35728	0.54928		-1.6737	0.016148	0.070856	
transcript_HQ_CoA_transcript5289/f2p0/3923	2.276666667	4.503333333	4.07	1.1347	0.0031252	0.013452	UP	-0.28388	0.38555	0.57764		0.83815	0.0034747	0.023234	
transcript_HQ_CoA_transcript529/f4p0/6313	12.95333333	41.24	23.23	1.8318	1.3531E-11	3.7594E-10	UP	-0.97855	3.9256E-06	6.1288E-05		0.84034	0.0022897	0.016922	
transcript_HQ_CoA_transcript5302/f3p0/3832	10	1.06	4.753333333	-3.1029	0.00063478	0.00346	DOWN	2.0374	0.028582	0.090106		-1.0767	0.005827	0.034346	DOWN
transcript_HQ_CoA_transcript5313/f2p0/3821	3.42	6.726666667	2.89	1.1436	0.001283	0.0063671	UP	-1.3772	7.6852E-05	0.00077227	DOWN	-0.24656	0.37832	0.59499	
transcript_HQ_CoA_transcript5316/f2p0/3849	38.57666667	11.20666667	24.02666667	-1.6467	1.9375E-06	2.1441E-05	DOWN	0.96004	0.0027853	0.014499		-0.69678	0.0064924	0.036986	
transcript_HQ_CoA_transcript5359/f3p0/3804	23.83333333	1.213333333	1.473333333	-4.141	2.194E-13	7.5539E-12	DOWN	0.12363	0.8493	0.91807		-4.0294	4.5111E-40	2.7601E-36	DOWN
transcript_HQ_CoA_transcript5365/f4p0/3803	6.983333333	12.61	8.8	1.0216	0.0048442	0.019328	UP	-0.67451	0.0042767	0.020787		0.33425	0.31244	0.52919	
transcript_HQ_CoA_transcript5367/f3p0/3696	11.98333333	2.613333333	8.2	-2.0586	1.2207E-10	2.9521E-09	DOWN	1.5046	1.9424E-12	1.3907E-10	UP	-0.56476	0.048624	0.15721	
transcript_HQ_CoA_transcript5369/f2p0/3823	426.28	42.14333333	61.91	-3.2	2.926E-18	1.6113E-16	DOWN	0.41417	0.19621	0.36784		-2.7953	2.8573E-25	3.085E-22	DOWN
transcript_HQ_CoA_transcript5378/f2p0/3781	3.66	0.496666667	9.706666667	-2.7566	0.0023458	0.010602	DOWN	4.206	0.00001303	0.00016976	UP	1.432	0.073434	0.20653	
transcript_HQ_CoA_transcript5380/f2p0/3865	15.09	40.26666667	17.97	1.573	1.4372E-08	2.3995E-07	UP	-1.3084	0.00004977	0.00054042	DOWN	0.25264	0.3659	0.58294	
transcript_HQ_CoA_transcript5388/f2p0/3808	9.976666667	4.21	7.866666667	-1.0999	0.00062265	0.0034051	DOWN	0.7468	0.0063014	0.028081		-0.36296	0.27963	0.49347	
transcript_HQ_CoA_transcript5390/f2p0/3843	23.83666667	6.063333333	5.143333333	-1.8445	0.0000425	0.00033362	DOWN	-0.37587	0.20855	0.38371		-2.2296	1.5958E-08	8.5896E-07	DOWN
transcript_HQ_CoA_transcript5392/f3p0/3812	28.37333333	10.91333333	38.01333333	-1.2039	0.00015782	0.0010447	DOWN	1.627	2.5659E-06	4.2795E-05	UP	0.41101	0.06267	0.18635	
transcript_HQ_CoA_transcript5414/f3p0/3913	6.233333333	1.483333333	8.236666667	-1.8947	4.9952E-05	0.00038387	DOWN	2.3055	1.8347E-11	1.0253E-09	UP	0.39718	0.37049	0.58764	
transcript_HQ_CoA_transcript5436/f2p0/3823	0.13	1.576666667	4.46	3.7365	3.4976E-07	4.4684E-06	UP	1.3623	0.0011584	0.0071826	UP	5.0858	3.4504E-20	1.4728E-17	UP
transcript_HQ_CoA_transcript5438/f3p0/3807	0.693333333	6.16	0.703333333	3.2836	0.0077871	0.02863	UP	-3.3075	0.21574	0.39246		-0.046973	0.98996	0.99614	

transcript_HQ_CoA_transcript5441/f6p0/3768	13.59666667	24.88666667	18.77333333	1.0193	0.0086663	0.031312	UP	-0.54216	0.13557	0.28372		0.46424	0.076344	0.21241	
transcript_HQ_CoA_transcript5453/f3p0/3802	0.843333333	3.043333333	5.746666667	2.0369	0.015562	0.049818	UP	0.77982	0.0088178	0.036344		2.8004	0.00016446	0.0021274	UP
transcript_HQ_CoA_transcript5468/f5p0/3747	14.08333333	6.276666667	13.08	-1.0184	6.5949E-06	6.4962E-05	DOWN	0.92028	4.7031E-06	7.1537E-05		-0.10958	0.5975	0.77083	
transcript_HQ_CoA_transcript5469/f3p0/3795	26.8	4.653333333	7.77	-2.3448	0.00075126	0.0040054	DOWN	0.57927	0.44018	0.62867		-1.7807	1.1461E-08	6.4531E-07	DOWN
transcript_HQ_CoA_transcript5470/f2p0/3790	7.403333333	18.62333333	12.50333333	1.4857	6.2851E-16	2.8726E-14	UP	-0.7283	0.00022926	0.0019093		0.74468	0.00024106	0.0028919	
transcript_HQ_CoA_transcript5474/f4p0/3787	56.27	19.16333333	51.86	-1.4071	0.0010096	0.005192	DOWN	1.2971	0.00059956	0.0042202	UP	-0.12306	0.69668	0.83979	
transcript_HQ_CoA_transcript5490/f2p0/3793	0.016666667	1.203333333	0.523333333	6.2405	3.9223E-07	4.9556E-06	UP	-1.2942	0.21306	0.3892		4.9436	0.0070261	0.038985	UP
transcript_HQ_CoA_transcript5500/f2p0/3790	0.71	12.68	14.43	4.3492	7.6039E-21	5.0551E-19	UP	0.014079	0.98836	0.9939		4.3444	0.00039563	0.004269	UP
transcript_HQ_CoA_transcript5506/f2p0/3786	1.41	5.37	3.093333333	2.0705	0.0001714	0.0011247	UP	-0.94077	0.021716	0.073099		1.12	0.051056	0.16233	
transcript_HQ_CoA_transcript5508/f2p0/3774	21.48333333	43.86333333	36.81333333	1.1774	0.0015792	0.0076038	UP	-0.4027	0.1565	0.31426		0.76374	0.050022	0.16012	
transcript_HQ_CoA_transcript5524/f2p0/3795	16.41333333	6.25	10.03	-1.2373	8.2168E-07	9.7965E-06	DOWN	0.53733	0.053623	0.14417		-0.71162	0.00092151	0.0083776	
transcript_HQ_CoA_transcript5551/f2p0/3772	3.053333333	6.26	4.43	1.1941	0.0017166	0.0081621	UP	-0.66214	0.025036	0.081477		0.521	0.094904	0.24765	
transcript_HQ_CoA_transcript5561/f3p0/3771	3.43	20.32	3.67	2.722	5.3794E-09	9.7372E-08	UP	-2.6192	1.7023E-09	6.2905E-08	DOWN	0.089694	0.78322	0.8944	
transcript_HQ_CoA_transcript5568/f2p0/3826	0	9.866666667	0	11.139	0.0043586	0.017704	UP	-11.268	0.0039269	0.019384	DOWN	NA	NA	NA	NA
transcript_HQ_CoA_transcript5572/f2p0/3786	1.923333333	7.243333333	1.416666667	2.0972	0.00013879	0.00093817	UP	-2.5408	0.00000489	7.3466E-05	DOWN	-0.45774	0.38021	0.59633	
transcript_HQ_CoA_transcript5573/f3p0/3761	0.38	1.54	1.356666667	2.1401	0.0093532	0.033352	UP	-0.3012	0.62155	0.77331		1.8259	0.0045784	0.028711	UP
transcript_HQ_CoA_transcript5574/f2p0/3771	1.196666667	3.653333333	2.936666667	1.7679	2.3579E-07	3.1305E-06	UP	-0.47554	0.093773	0.21792		1.2803	0.00085298	0.0078913	UP
transcript_HQ_CoA_transcript5583/f2p0/3764	37.28333333	14.2	32.58333333	-1.2235	0.012226	0.041446	DOWN	1.0166	0.019666	0.067807		-0.21711	0.54154	0.73225	
transcript_HQ_CoA_transcript5598/f2p0/3735	6.563333333	2.366666667	3.19	-1.318	1.6735E-05	0.00014653	DOWN	0.27878	0.31938	0.5105		-1.0508	0.00044966	0.0047626	DOWN
transcript_HQ_CoA_transcript5605/f2p0/3778	1.233333333	3.246666667	10.05666667	1.5499	4.5033E-06	4.5815E-05	UP	1.4815	6.0058E-12	3.7698E-10	UP	3.0193	1.1518E-27	1.9219E-24	UP
transcript_HQ_CoA_transcript5612/f10p0/3696	12.77333333	34.61	20.54333333	1.5879	0.0002734	0.0016807	UP	-0.89334	0.00028926	0.0023142		0.68226	0.094034	0.24626	
transcript_HQ_CoA_transcript5615/f2p0/3750	10.15	3.536666667	4.556666667	-1.3779	4.7325E-05	0.0003663	DOWN	0.21345	0.38452	0.57698		-1.1762	0.0001689	0.002171	DOWN
transcript_HQ_CoA_transcript5616/f3p0/3763	4.13	12.02333333	8.12	1.7095	2.4215E-05	0.00020289	UP	-0.72762	0.060645	0.15834		0.96953	0.0067716	0.038039	
transcript_HQ_CoA_transcript5626/f2p0/3819	9.303333333	30.99333333	8.06	1.8719	0.010602	0.036956	UP	-2.0628	0.0037379	0.018592	DOWN	-0.20513	0.57758	0.75698	
transcript_HQ_CoA_transcript5633/f2p0/3757	3.673333333	1.463333333	3.636666667	-1.169	0.012266	0.041536	DOWN	1.159	0.0059731	0.026979	UP	-0.022449	0.95105	0.97871	
transcript_HQ_CoA_transcript5637/f2p0/3765	3.863333333	28.32666667	6.106666667	3.0544	7.998E-09	1.4087E-07	UP	-2.359	5.3293E-45	1.0853E-41	DOWN	0.68041	0.2169	0.42203	
transcript_HQ_CoA_transcript5654/f2p0/3823	2.373333333	5.743333333	4.566666667	1.4314	9.7645E-06	9.1597E-05	UP	-0.49251	0.11638	0.25464		0.92805	0.0050658	0.03083	
transcript_HQ_CoA_transcript5661/f3p0/3757	20.35666667	8.603333333	19.38666667	-1.0868	0.0038589	0.0161	DOWN	1.0096	2.2958E-06	3.8926E-05	UP	-0.087825	0.78696	0.89669	
transcript_HQ_CoA_transcript5665/f2p0/3801	16.20333333	5.373333333	8.06	-1.4524	1.079E-07	1.5245E-06	DOWN	0.44767	0.11698	0.25556		-1.015	6.1882E-06	0.00014305	DOWN
transcript_HQ_CoA_transcript5672/f2p0/3748	16.75333333	5.436666667	9.176666667	-1.4638	9.5728E-08	1.3705E-06	DOWN	0.59716	0.019523	0.067442		-0.87744	2.7449E-05	0.00049058	
transcript_HQ_CoA_transcript5703/f2p0/3751	8.666666667	3.636666667	15.34666667	-1.0975	0.0010525	0.0053718	DOWN	1.9147	0.00012762	0.0011754	UP	0.80529	0.056895	0.1749	
transcript_HQ_CoA_transcript5714/f2p0/3749	4.926666667	11.99	7.25	1.4174	0.00024039	0.0015051	UP	-0.85888	0.021931	0.073715		0.54828	0.21035	0.41393	
transcript_HQ_CoA_transcript5755/f3p0/3737	4.113333333	14.88	6.096666667	1.997	6.6228E-16	3.0194E-14	UP	-1.4373	6.6167E-12	4.0697E-10	DOWN	0.54761	0.056148	0.17346	
transcript_HQ_CoA_transcript5761/f2p0/3739	2.436666667	0.513333333	0.42	-2.075	0.00036936	0.0021839	DOWN	-0.46162	0.38002	0.57242		-2.5517	1.2615E-05	0.00025585	DOWN
transcript_HQ_CoA_transcript5763/f2p0/3733	20.56666667	52.36	24.67666667	1.5066	1.0374E-07	1.468E-06	UP	-1.2389	1.894E-07	4.3888E-06	DOWN	0.25568	0.24798	0.45788	
transcript_HQ_CoA_transcript5764/f3p0/3657	14.04333333	44.47333333	37.43	1.8322	1.8705E-09	3.681E-08	UP	-0.40888	0.16415	0.32473		1.4109	5.8338E-22	3.2448E-19	UP
transcript_HQ_CoA_transcript5765/f3p0/3724	2.406666667	5.663333333	0.65	1.3807	0.00032606	0.0019602	UP	-3.2637	6.8198E-11	3.4341E-09	DOWN	-1.8958	0.00013735	0.0018443	DOWN
transcript_HQ_CoA_transcript5767/f5p0/3635	10.58	3.213333333	4.12	-1.5745	0.00041489	0.0024079	DOWN	0.2141	0.52392	0.69672		-1.3679	0.0024167	0.017603	DOWN
transcript_HQ_CoA_transcript5780/f3p0/3741	5.42	44.49666667	12.16333333	3.2169	1.0438E-10	2.5343E-09	UP	-2.0537	9.045E-06	0.00012437	DOWN	1.151	4.2227E-05	0.00069575	UP
transcript_HQ_CoA_transcript5783/f2p0/3770	3.02	36.96666667	7.49	3.7958	4.9033E-13	1.6239E-11	UP	-2.4539	1.4862E-10	6.9847E-09	DOWN	1.3255	0.0035719	0.023748	UP
transcript_HQ_CoA_transcript5806/f2p0/3739	6.78	12.4	6.61	1.0548	0.013789	0.045537	UP	-1.069	0.011807	0.045761	DOWN	-0.028856	0.93595	0.97186	
transcript_HQ_CoA_transcript5821/f2p0/3748	13.05333333	44.03666667	28.41666667	1.9252	5.1232E-10	1.1111E-08	UP	-0.78636	0.013086	0.049624		1.1247	2.8514E-07	1.0747E-05	UP
transcript_HQ_CoA_transcript5822/f2p0/3713	11.30666667	4.64	5.883333333	-1.1366	5.8257E-05	0.00043975	DOWN	0.19784	0.43557	0.62449		-0.95047	7.9209E-05	0.0011715	
transcript_HQ_CoA_transcript5831/f2p0/3831	22.31	5.81	14.9	-1.8044	1.1239E-09	2.3113E-08	DOWN	1.2151	1.6715E-05	0.00020955	UP	-0.60067	0.0093376	0.047848	
transcript_HQ_CoA_transcript5843/f2p0/3714	3.806666667	11.84	8.79	1.7833	0.00025003	0.0015537	UP	-0.58666	0.0092921	0.037789		1.1871	0.0089449	0.046484	UP
transcript_HQ_CoA_transcript5850/f2p0/3716	4.793333333	29.67	11.22333333	2.7691	1.3564E-07	1.8858E-06	UP	-1.5495	0.00031018	0.0024432	DOWN	1.2099	0.0013358	0.011282	UP
transcript_HQ_CoA_transcript5856/f3p0/3695	1.603333333	4.636666667	4	1.7193	0.00012636	0.00086262	UP	-0.38265	0.46092	0.64629		1.3202	0.0069937	0.038876	UP
transcript_HQ_CoA_transcript5879/f2p0/3779	3.146666667	0.3	0.573333333	-3.2876	5.2624E-09	9.5444E-08	DOWN	0.82766	0.1777	0.34321		-2.4689	2.7627E-08	1.4086E-06	DOWN
transcript_HQ_CoA_transcript5884/f10p0/3621	24.5	58.10666667	25.85	1.4095	5.8231E-07	7.1161E-06	UP	-1.3172	5.6423E-06	8.3051E-05	DOWN	0.079707	0.68996	0.8351	
transcript_HQ_CoA_transcript589/f2p0/6233	0.443333333	5.366666667	4.113333333	3.7256	1.4879E-10	3.5327E-09	UP	-0.5059	0.39052	0.58118		3.2059	1.2493E-06	3.8092E-05	UP
transcript_HQ_CoA_transcript5908/f2p0/3705	0.183333333	18.53	1.51	6.8184	1.3271E-14	5.3531E-13	UP	-3.7253	0.00026465	0.002153	DOWN	3.074	0.0091938	0.047296	UP
transcript_HQ_CoA_transcript591/f2p0/6218	1.063333333	2.033333333	2.213333333	1.106	0.012792	0.042863	UP	-0.036264	0.92733	0.96203		1.0575	0.0019536	0.015016	UP
transcript_HQ_CoA_transcript5914/f7p0/3635	13.09	24.62666667	21.37333333	1.063	5.6181E-05	0.00042671	UP	-0.35129	0.15983	0.31918		0.70033	0.017707	0.075993	
transcript_HQ_CoA_transcript5917/f2p0/3752	7.846666667	17.1	10.56333333	1.2884	2.5929E-08	4.1914E-07	UP	-0.84666	2.4545E-07	5.5728E-06		0.42849	0.086339	0.23149	
transcript_HQ_CoA_transcript5919/f6p0/3706	3.863333333	33.26333333	8.586666667	3.2731	1.8118E-24	1.5264E-22	UP	-2.1177	9.3404E-11	4.5898E-09	DOWN	1.1416	3.3206E-06	8.5845E-05	UP
transcript_HQ_CoA_transcript5945/f5p0/3692	21.53	6.763333333	17.46	-1.5001	3.8339E-05	0.00030475	DOWN	1.2069	0.0011309	0.0070576	UP	-0.30474	0.14829	0.33086	
transcript_HQ_CoA_transcript5947/f3p0/3715	2.29	7.076666667	2.013333333	1.7855	7.3413E-05	0.00053814	UP	-1.9705	3.0167E-12	2.0254E-10	DOWN	-0.19678	0.66854	0.81991	
transcript_HQ_CoA_transcript595/f2p0/6236	0.07	0.946666667	0.663333333	3.9493	9.7427E-06	9.1529E-05	UP	-0.62612	0.49268	0.6721		3.3109	0.0043316	0.027464	UP
transcript_HQ_CoA_transcript5951/f3p0/3659	4.623333333	1.863333333	2.613333333	-1.159	0.00059401	0.003269	DOWN	0.3392	0.28887	0.47842		-0.83193	0.0055816	0.033091	
transcript_HQ_CoA_transcript5955/f2p0/3706	4.28	15.11333333	3.986666667	1.9819	3.2998E-06	3.4551E-05	UP	-2.0677	9.1808E-05	0.00089799	DOWN	-0.10091	0.81668	0.91476	
transcript_HQ_CoA_transcript5958/f3p0/3666	0.2	11.63333333	0.7	6.0043	1.0005E-14	4.0893E-13	UP	-4.1906	9.0089E-10	3.5511E-08	DOWN	1.8027	0.092277	0.2428	
transcript_HQ_CoA_transcript5962/f2p0/3717	3.85	1.073333333	2.04	-1.679	0.0099616	0.035124	DOWN	0.77654	0.12454	0.26738		-0.91699	0.15168	0.33519	
transcript_HQ_CoA_transcript5967/f2p0/3704	8.673333333	22.57333333	8.343333333	1.5795	0.0058934	0.022692	UP	-1.6139	0.0026322	0.013887	DOWN	-0.049058	0.88405	0.9482	
transcript_HQ_CoA_transcript5975/f2p0/3718	4.933333333	11.50666667	8.956666667	1.4024	0.0011886	0.0059601	UP	-0.53779	0.19762	0.36961		0.85114	3.2634E-05	0.0005713	
transcript_HQ_CoA_transcript5995/f2p0/3694	5	10.61333333	12.01666667	1.2292	0.0041051	0.016884	UP	0.03776	0.93173	0.96441		1.2552	0.00014015	0.0018722	UP
transcript_HQ_CoA_transcript6006/f3p0/3661	6.706666667	16.94333333	5.783333333	1.5308	0.007783	0.028624	UP	-1.7335	0.0014813	0.008747	DOWN	-0.21744	0.50863	0.7077	
transcript_HQ_CoA_transcript6009/f2p0/3716	5.4	1.63	3.453333333	-1.5906	1.0371E-05	9.6681E-05	DOWN	0.93546	0.022142	0.074221		-0.66552	0.05737	0.1758	
transcript_HQ_CoA_transcript602/f3p0/6210	0.31	2.766666667	0.42	3.358	0.00050059	0.0028255	UP	-2.8443	0.00029666	0.002362	DOWN	0.49592	0.58299	0.76124	
transcript_HQ_CoA_transcript6023/f3p0/3694	6.153333333	2.29	3.22	-1.2492	0.0039492	0.016383	DOWN	0.32727	0.47795	0.66091		-0.93445	0.00047044	0.0049287	
transcript_HQ_CoA_transcript6039/f2p0/3693	4.033333333	30.43333333	5.923333333	3.0922	3.2978E-17	1.6983E-15	UP	-2.5248	4.7658E-12	3.0122E-10	DOWN	0.55226	0.057416	0.17585	
transcript_HQ_CoA_transcript6093/f2p0/3681	9.36	1.73	7.873333333	-2.2712	1.031E-09	2.1418E-08	DOWN	2.0232	2.0462E-08	5.9157E-07	UP	-0.25898	0.24716	0.45668	
transcript_HQ_CoA_transcript6098/f2p0/3675	0.113333333	10.62	0.736666667	6.6571	2.0232E-14	7.9375E-13	UP	-4.0285	1.0797E-07	2.6493E-06	DOWN	2.6174	0.047778	0.15532	
transcript_HQ_CoA_transcript610/f2p0/6221	5.803333333	11.32	9.49	1.125	1.4611E-06	1.6643E-05	UP	-0.41332	0.064707	0.16637		0.69986	0.00038533	0.0042024	
transcript_HQ_CoA_transcript6103/f10p0/3651	20.58333333	7.08	13.72333333	-1.3821	1.0994E-05	0.00010171	DOWN	0.80128	0.0068836	0.029969		-0.59178	0.0085397	0.044961	
transcript_HQ_CoA_transcript6106/f35p0/3518	42.78	10.15666667	43.20666667	-1.9235	4.8449E-14	1.8114E-12	DOWN	1.9564	6.5702E-08	1.7092E-06	UP	0.022143	0.94069	0.97369	
transcript_HQ_CoA_transcript6109/f3p0/3686	5.656666667	2.443333333	4.25	-1.0638	2.9354E-05	0.00024174	DOWN	0.65378	0.035415	0.10527		-0.42036	0.16421	0.35294	
transcript_HQ_CoA_transcript6122/f4p0/3668	7.093333333	17.57	6.643333333	1.4938	0.0004577	0.0026149	UP	-1.5795	0.00036312	0.0027988	DOWN	-0.10281	0.81091	0.91118	
transcript_HQ_CoA_transcript6146/f3p0/3669	3.303333333	21.6	16.42666667	2.887	1.6951E-07	2.3075E-06	UP	-0.54862	0.17578	0.34061		2.3226	1.7987E-05	0.00034535	UP
transcript_HQ_CoA_transcript6149/f3p0/3665	12.22333333	3.73	5.893333333	-1.5215	0.00054566	0.0030396	DOWN	0.48399	0.26062	0.44574		-1.0511	0.00023519	0.0028363	DOWN

transcript_HQ_CoA_transcript6154/f2p0/3657	11.05	25.56333333	13.57666667	1.3614	0.00003938	0.00031203	UP	-1.0637	0.00018072	0.0015819	DOWN	0.28579	0.238	0.4466	
transcript_HQ_CoA_transcript616/f3p0/6193	1.503333333	0.616666667	0.816666667	-1.1357	0.0045427	0.018298	DOWN	0.25848	0.53113	0.703		-0.88844	0.010313	0.051497	
transcript_HQ_CoA_transcript6161/f4p0/3617	4.913333333	12.25666667	21.38	1.4814	0.00038955	0.0022855	UP	0.65329	0.045861	0.12773		2.1216	1.5179E-06	0.00004472	UP
transcript_HQ_CoA_transcript617/f2p0/6186	5.356666667	1.916666667	3.083333333	-1.3278	2.0807E-10	4.8022E-09	DOWN	0.53885	0.059773	0.15665		-0.80017	0.0031317	0.021449	
transcript_HQ_CoA_transcript6192/f9p0/3644	23.36	47.96666667	20.17	1.1787	1.3588E-05	0.00012237	UP	-1.38	5.2967E-06	7.8547E-05	DOWN	-0.21206	0.49422	0.69614	
transcript_HQ_CoA_transcript6195/f4p0/3623	8.07	1.91	3.95	-1.9006	0.00099495	0.0051296	DOWN	0.86671	0.1222	0.26343		-1.0453	0.00057195	0.0057241	DOWN
transcript_HQ_CoA_transcript6200/f2p0/3666	15.62	5.856666667	12.26666667	-1.2634	2.7727E-07	3.6207E-06	DOWN	0.91978	0.00011428	0.0010725		-0.35488	0.13943	0.31784	
transcript_HQ_CoA_transcript6203/f2p0/3660	5.903333333	2.476666667	5.366666667	-1.0996	0.00023611	0.0014824	DOWN	0.95928	0.0081981	0.034369		-0.15156	0.61909	0.78604	
transcript_HQ_CoA_transcript6206/f5p0/3630	6.14	2.76	4.363333333	-1.0162	0.0035923	0.015153	DOWN	0.52064	0.057779	0.1528		-0.50692	0.085435	0.22997	
transcript_HQ_CoA_transcript621/f2p0/6173	2.77	7.503333333	5.49	1.5995	9.1771E-06	8.7201E-05	UP	-0.59472	0.055094	0.1474		0.9909	0.00016609	0.0021378	
transcript_HQ_CoA_transcript6210/f3p0/3643	3.953333333	22.41666667	5.56	2.6602	2.4505E-11	6.6271E-10	UP	-2.1574	9.3566E-09	2.9535E-07	DOWN	0.49061	0.059437	0.18026	
transcript_HQ_CoA_transcript6218/f2p0/3670	4.076666667	1.01	3.526666667	-1.8587	0.00012602	0.00086095	DOWN	1.6435	0.0016102	0.0093429	UP	-0.22542	0.54651	0.73585	
transcript_HQ_CoA_transcript6235/f4p0/3650	5.993333333	27.12333333	8.136666667	2.3415	2.9195E-10	6.5977E-09	UP	-1.8897	5.4683E-07	1.1415E-05	DOWN	0.43786	0.052057	0.16488	
transcript_HQ_CoA_transcript6260/f2p0/3632	6.206666667	83.18333333	21.38333333	3.9256	3.5103E-28	4.1404E-26	UP	-2.1033	0.00033149	0.0025833	DOWN	1.8066	0.0009257	0.0084053	UP
transcript_HQ_CoA_transcript6266/f3p0/3645	1.496666667	5.11	3.43	1.9359	7.5375E-07	9.0539E-06	UP	-0.73704	0.2915	0.48122		1.1808	0.10822	0.2704	
transcript_HQ_CoA_transcript6268/f2p0/3680	7.653333333	2.063333333	4.2	-1.7464	1.8485E-05	0.00016009	DOWN	0.88976	0.005059	0.02368		-0.86907	0.0094331	0.048199	
transcript_HQ_CoA_transcript6281/f2p0/3674	5.05	13.64	4.913333333	1.5794	0.00011555	0.00079856	UP	-1.628	3.3387E-12	2.2012E-10	DOWN	-0.05838	0.88335	0.94791	
transcript_HQ_CoA_transcript6284/f4p0/3624	5.9	30.19333333	12.34333333	2.5185	1.3645E-11	3.7854E-10	UP	-1.4446	5.9528E-05	0.00062029	DOWN	1.0608	1.3511E-08	7.4925E-07	UP
transcript_HQ_CoA_transcript6288/f2p0/3773	4.786666667	2.156666667	3.443333333	-1.0046	0.0026258	0.011626	DOWN	0.5266	0.13018	0.27552		-0.48858	0.1294	0.30266	
transcript_HQ_CoA_transcript6303/f2p0/3653	6.7	13.80666667	8.206666667	1.2044	1.3004E-06	1.4989E-05	UP	-0.90171	1.0449E-05	0.00014131		0.28874	0.29459	0.51026	
transcript_HQ_CoA_transcript6305/f2p0/3639	5.653333333	17.36	6.076666667	1.757	6.912E-09	1.228E-07	UP	-1.6496	8.8713E-10	3.5119E-08	DOWN	0.095847	0.71154	0.84896	
transcript_HQ_CoA_transcript6306/f2p0/3645	1.306666667	3.853333333	4.11	1.6869	0.0012695	0.0063068	UP	-0.046152	0.90595	0.94936		1.6297	0.0019799	0.015161	UP
transcript_HQ_CoA_transcript6315/f4p0/3562	7.62	2.726666667	6.676666667	-1.3261	0.00022396	0.0014173	DOWN	1.145	0.00064606	0.0044923	UP	-0.19256	0.57965	0.75856	
transcript_HQ_CoA_transcript6327/f8p0/3594	2.646666667	8.88	4.68	1.9153	1.4016E-05	0.00012573	UP	-1.0932	0.0043842	0.021147	DOWN	0.81016	0.017243	0.074328	
transcript_HQ_CoA_transcript6332/f2p0/3654	34.27	81.42	50.07666667	1.4122	4.0974E-13	1.3719E-11	UP	-0.85349	0.00018675	0.0016269		0.54577	0.0039078	0.025434	
transcript_HQ_CoA_transcript635/f2p0/6174	4.093333333	12.38333333	7.206666667	1.7609	2.0277E-16	9.6285E-15	UP	-0.92623	0.021668	0.07302		0.82042	0.04109	0.13982	
transcript_HQ_CoA_transcript6357/f9p0/3616	9.783333333	4.386666667	9.776666667	-1.0146	0.00052466	0.0029432	DOWN	1.0179	0.00042833	0.003211	UP	-0.0078909	0.97219	0.9885	
transcript_HQ_CoA_transcript6365/f2p0/3661	8.07	3.156666667	7.52	-1.1881	0.00048617	0.0027582	DOWN	1.0841	0.0013849	0.0082844	UP	-0.11598	0.62035	0.78718	
transcript_HQ_CoA_transcript64/f3p0/8145	10.58666667	4.45	7.9	-1.0967	5.9887E-06	5.9503E-05	DOWN	0.68119	0.0012378	0.0075595		-0.42752	0.017104	0.073869	
transcript_HQ_CoA_transcript640/f2p0/6167	0.013333333	2.313333333	2.076666667	7.9048	3.0907E-06	3.2548E-05	UP	-0.28348	0.80944	0.89518		7.6142	1.2851E-11	1.4124E-09	UP
transcript_HQ_CoA_transcript6400/f4p0/3592	1.936666667	8.536666667	5.66	2.2478	0.0090464	0.032454	UP	-0.71719	0.40378	0.59424		1.5235	0.00048766	0.0050685	UP
transcript_HQ_CoA_transcript6403/f2p0/3636	8.106666667	1.833333333	5.236666667	-1.9943	2.6598E-07	3.4833E-06	DOWN	1.3652	0.00013168	0.0012086	UP	-0.63949	0.031734	0.11612	
transcript_HQ_CoA_transcript6405/f2p0/3625	6.463333333	1.81	2.57	-1.6663	0.0068033	0.02559	DOWN	0.33165	0.53353	0.70469		-1.3457	0.0031561	0.021567	DOWN
transcript_HQ_CoA_transcript6415/f2p0/3612	1.633333333	0.026666667	1.003333333	-5.8106	0.00017853	0.0011665	DOWN	5.1786	6.2345E-05	0.0006456	UP	-0.65454	0.56462	0.74774	
transcript_HQ_CoA_transcript642/f5p0/6148	6.96	20.06666667	10.40333333	1.6741	1.5534E-10	3.6693E-09	UP	-1.0954	5.4563E-07	1.1404E-05	DOWN	0.56784	0.011882	0.056972	
transcript_HQ_CoA_transcript6423/f2p0/3630	31.46333333	5.823333333	21.83333333	-2.309	9.9958E-05	0.00070286	DOWN	1.7634	4.5779E-07	9.7228E-06	UP	-0.55261	0.32511	0.54204	
transcript_HQ_CoA_transcript6425/f4p0/3647	4.726666667	11.97333333	14.69666667	1.508	5.11E-08	7.7657E-07	UP	0.13885	0.49554	0.67475		1.6334	1.4259E-09	9.6938E-08	UP
transcript_HQ_CoA_transcript6429/f2p0/3612	7.023333333	1.256666667	7.583333333	-2.2969	0.0012126	0.0060573	DOWN	2.4261	1.197E-07	2.9021E-06	UP	0.11441	0.86767	0.94117	
transcript_HQ_CoA_transcript6446/f3p0/3613	5	9.263333333	3.36	1.0543	0.0077636	0.028564	UP	-1.6154	0.00012468	0.0011518	DOWN	-0.57328	0.11768	0.28555	
transcript_HQ_CoA_transcript6448/f2p0/3612	17.28333333	38.26333333	12.82	1.3104	6.6144E-05	0.00049156	UP	-1.7366	4.5108E-08	1.2177E-06	DOWN	-0.4387	0.037765	0.13143	
transcript_HQ_CoA_transcript6453/f2p0/3628	2.256666667	8.68	8.31	2.0861	0.00010663	0.00074459	UP	-0.20288	0.71833	0.83889		1.8733	3.0682E-06	0.00008103	UP
transcript_HQ_CoA_transcript6468/f5p0/3612	14.29666667	3.613333333	21.20666667	-1.8365	0.0004719	0.002686	DOWN	2.3933	7.4113E-25	3.5748E-22	UP	0.54658	0.25208	0.46245	
transcript_HQ_CoA_transcript6474/f7p0/3594	24.85	70.81666667	66.36	1.68	6.7003E-07	8.1123E-06	UP	-0.2388	0.19768	0.36964		1.4263	4.0073E-06	0.00010007	UP
transcript_HQ_CoA_transcript6475/f2p0/3611	1.946666667	10.52666667	1.23	2.6112	1.1891E-11	3.3342E-10	UP	-3.2532	4.0454E-10	1.7571E-08	DOWN	-0.6557	0.33271	0.54992	
transcript_HQ_CoA_transcript6483/f2p0/3628	19.1	7.75	15.4	-1.1251	0.0031985	0.013707	DOWN	0.81729	0.031771	0.09725		-0.32086	0.043726	0.14598	
transcript_HQ_CoA_transcript6484/f2p0/3609	11.00666667	20.33333333	11.85	1.0533	8.22E-07	9.7965E-06	UP	-0.93295	4.7995E-10	2.0411E-08		0.10673	0.60482	0.77655	
transcript_HQ_CoA_transcript6492/f5p0/3609	21.73333333	76.28	24.75666667	1.9695	7.002E-11	1.7252E-09	UP	-1.7696	1.0626E-06	1.9976E-05	DOWN	0.18659	0.50381	0.70412	
transcript_HQ_CoA_transcript6521/f3p0/3592	1.646666667	3.456666667	4.923333333	1.2117	0.0086909	0.031382	UP	0.37964	0.3624	0.55456		1.5803	4.1192E-06	0.00010231	UP
transcript_HQ_CoA_transcript6537/f3p0/3625	9.693333333	20.27	13.79	1.2071	2.9014E-07	3.7673E-06	UP	-0.69638	0.0019056	0.010681		0.49876	0.022045	0.088969	
transcript_HQ_CoA_transcript6543/f2p0/3587	6.32	2.373333333	0.986666667	-1.2591	8.0768E-07	9.651E-06	DOWN	-1.4171	0.0060658	0.02731	DOWN	-2.6868	3.3996E-09	2.1592E-07	DOWN
transcript_HQ_CoA_transcript6545/f3p0/3701	4.406666667	15.23	6.33	1.9532	6.5366E-15	2.716E-13	UP	-1.4074	0.00082789	0.0054781	DOWN	0.53262	0.20557	0.40803	
transcript_HQ_CoA_transcript6547/f3p0/3591	40.93333333	15.3	23.5	-1.2707	1.0959E-10	2.6572E-09	DOWN	0.47534	0.0055132	0.025298		-0.80722	2.8741E-05	0.00051167	
transcript_HQ_CoA_transcript6549/f2p0/3606	23.05	5.493333333	8.156666667	-1.9164	1.7078E-11	4.6881E-10	DOWN	0.41787	0.040971	0.11726		-1.5086	1.2603E-08	7.0526E-07	DOWN
transcript_HQ_CoA_transcript6554/f3p0/3666	3.3	10.89666667	7.453333333	1.9303	0.0067829	0.025531	UP	-0.70628	0.011943	0.046132		1.2056	0.080477	0.22057	
transcript_HQ_CoA_transcript6557/f7p0/3568	27.76666667	54.46666667	49.78	1.1204	6.7046E-06	0.0000659	UP	-0.25306	0.49242	0.67195		0.85456	0.0073241	0.040129	
transcript_HQ_CoA_transcript6559/f2p0/3598	1.383333333	17.85333333	14.73	3.8381	4.4362E-08	6.8268E-07	UP	-0.40789	0.50305	0.68018		3.4166	9.2027E-11	8.3622E-09	UP
transcript_HQ_CoA_transcript6577/f2p0/3610	1.886666667	4.92	1.676666667	1.5434	0.0019669	0.009155	UP	-1.7133	0.00126	0.0076678	DOWN	-0.18426	0.69127	0.83589	
transcript_HQ_CoA_transcript6578/f5p0/3592	43.55333333	12.60666667	21.79	-1.6483	1.2764E-08	2.1606E-07	DOWN	0.64925	0.00054756	0.0039174		-1.0093	9.4262E-05	0.0013519	DOWN
transcript_HQ_CoA_transcript6582/f2p0/3604	6.203333333	2.18	4.053333333	-1.3589	0.00011283	0.00078194	DOWN	0.76487	0.03406	0.10246		-0.60569	0.084475	0.22822	
transcript_HQ_CoA_transcript6589/f2p0/3594	3.253333333	15.74	2.1	2.4303	6.2925E-05	0.00047051	UP	-3.0633	5.8406E-07	1.2001E-05	DOWN	-0.64433	0.31314	0.52989	
transcript_HQ_CoA_transcript6591/f2p0/3683	3.836666667	15.84	4.066666667	2.2037	2.1471E-08	3.4976E-07	UP	-2.1193	9.3242E-08	2.3158E-06	DOWN	0.070807	0.8142	0.91348	
transcript_HQ_CoA_transcript660/f3p0/6145	5.16	1.783333333	9.856666667	-1.3869	1.8619E-07	2.5177E-06	DOWN	2.3203	3.6664E-28	2.4197E-25	UP	0.92139	4.4632E-06	0.00010908	
transcript_HQ_CoA_transcript6601/f3p0/3599	8.83	3.486666667	2.366666667	-1.1901	0.00083984	0.0044184	DOWN	-0.70593	0.0068115	0.02974		-1.9067	1.1763E-07	5.0094E-06	DOWN
transcript_HQ_CoA_transcript6629/f2p0/3599	26.82333333	6.916666667	28.19333333	-1.8113	0.011736	0.040105	DOWN	1.8919	0.00010402	0.00099667	UP	0.068284	0.91394	0.9621	
transcript_HQ_CoA_transcript6631/f3p0/3616	13.15666667	5.336666667	6.013333333	-1.1363	0.0004855	0.0027556	DOWN	0.023206	0.94505	0.97055		-1.1268	8.6055E-06	0.00018759	DOWN
transcript_HQ_CoA_transcript6637/f2p0/3587	3.586666667	10.87333333	3.27	1.7678	6.8636E-08	1.0121E-06	UP	-1.8753	1.9788E-06	3.4148E-05	DOWN	-0.122	0.79925	0.90413	
transcript_HQ_CoA_transcript6638/f2p0/3577	41.57	7.446666667	9.56	-2.3456	8.5512E-06	8.1893E-05	DOWN	0.20781	0.65421	0.79563		-2.1439	0.0001992	0.0024835	DOWN
transcript_HQ_CoA_transcript664/f2p0/6121	2.01	7.973333333	4.113333333	2.1403	1.0612E-08	1.8216E-07	UP	-1.1006	4.2926E-05	0.00047397	DOWN	1.0272	0.01098	0.053905	
transcript_HQ_CoA_transcript6648/f2p0/3617	53.42	22.37333333	46.03666667	-1.0976	1.8938E-07	2.5589E-06	DOWN	0.88997	6.5099E-06	9.3805E-05		-0.22009	0.097061	0.25185	
transcript_HQ_CoA_transcript6662/f3p0/3576	3.15	10.43666667	4.31	1.8863	1.3917E-08	2.33E-07	UP	-1.4235	9.5372E-07	1.8285E-05	DOWN	0.4495	0.12812	0.30096	
transcript_HQ_CoA_transcript6669/f2p0/3567	7.783333333	19.73	21.03333333	1.4996	1.6988E-14	6.7516E-13	UP	-0.063243	0.8243	0.90426		1.4247	1.5922E-06	4.6537E-05	UP
transcript_HQ_CoA_transcript6684/f2p0/3554	0	0.83	0.46	7.4631	0.0026463	0.011712	UP	-1.0494	0.77863	0.87588		6.3931	0.063375	0.18774	
transcript_HQ_CoA_transcript6695/f5p0/3572	12.64333333	5.376666667	10.73	-1.0807	0.0043709	0.017744	DOWN	0.83659	0.046499	0.12907		-0.253	0.49099	0.69281	
transcript_HQ_CoA_transcript6719/f2p0/3565	2.21	19.33333333	5.386666667	3.2837	1.3594E-33	2.1801E-31	UP	-1.9735	3.0106E-07	6.6805E-06	DOWN	1.2978	0.0042206	0.026899	UP
transcript_HQ_CoA_transcript6720/f3p0/3609	5.433333333	10.92666667	4.433333333	1.1722	0.0038611	0.016102	UP	-1.4602	1.8568E-09	6.8068E-08	DOWN	-0.30324	0.48705	0.69022	
transcript_HQ_CoA_transcript6733/f2p0/3564	15.67	6.306666667	17.15	-1.172	0.014231	0.046666	DOWN	1.294	3.5877E-10	1.5694E-08	UP	0.11058	0.79908	0.90406	
transcript_HQ_CoA_transcript6741/f2p0/3570	6.89	2.633333333	4.136666667	-1.2364	2.6841E-06	2.8646E-05	DOWN	0.50752	0.056216	0.14977		-0.74071	0.00082213	0.0076656	
transcript_HQ_CoA_transcript6763/f2p0/3566	4.77	29.22666667	6.26	2.7782	2.7746E-12	8.3704E-11	UP	-2.3747	1.9944E-08	5.8024E-07	DOWN	0.38964	0.27782	0.49172	
transcript_HQ_CoA_transcript678/f2p0/6197	3.526666667	0.17	0.21	-4.1849	6.7561E-20	4.1587E-18	DOWN	0.18365	0.79716	0.88811		-4.0181	5.1784E-29	9.505E-26	DOWN

transcript_HQ_CoA_transcript6783/f6p0/3535	42.66333333	11.50666667	24.66333333	-1.7358	5.9347E-17	2.9807E-15	DOWN	0.94475	5.4224E-06	8.0215E-05		-0.80177	0.00034384	0.0038319	
transcript_HQ_CoA_transcript6786/f3p0/3551	3.55	16.15333333	3.043333333	2.3427	2.2894E-05	0.0001936	UP	-2.5524	1.7628E-06	3.0949E-05	DOWN	-0.2244	0.5097	0.70838	
transcript_HQ_CoA_transcript679/f3p0/6105	4.983333333	18.67666667	16.81333333	2.044	1.6336E-09	3.2463E-08	UP	-0.29108	0.19592	0.36748		1.7416	1.3805E-08	7.6096E-07	UP
transcript_HQ_CoA_transcript6791/f2p0/3577	2.093333333	0.786666667	0.53	-1.2921	0.010621	0.037005	DOWN	-0.69008	0.32193	0.51301		-1.9915	0.0012731	0.010843	DOWN
transcript_HQ_CoA_transcript6802/f2p0/3557	7.493333333	0.526666667	2.813333333	-3.6448	3.3767E-22	2.4114E-20	DOWN	2.231	1.5917E-05	0.00020092	UP	-1.4228	0.00037383	0.0041118	DOWN
transcript_HQ_CoA_transcript6807/f3p0/3547	14.82	4.76	11.77666667	-1.497	7.5585E-07	9.0672E-06	DOWN	1.1658	0.00010352	0.00099338	UP	-0.3414	0.097584	0.25267	
transcript_HQ_CoA_transcript681/f2p0/6107	3.586666667	10.36	7.633333333	1.7001	1.0603E-11	2.9913E-10	UP	-0.59837	0.005123	0.023903		1.0883	3.5433E-08	1.7483E-06	UP
transcript_HQ_CoA_transcript6811/f3p0/3559	7.793333333	14.46	9.243333333	1.0534	0.00071905	0.0038517	UP	-0.78883	0.085531	0.20365		0.25147	0.55271	0.73965	
transcript_HQ_CoA_transcript6815/f2p0/3556	0.963333333	22.03	3.35	4.6921	6.1E-30	8.2E-28	UP	-2.8722	1.1483E-13	1.043E-11	DOWN	1.8057	0.00012086	0.0016654	UP
transcript_HQ_CoA_transcript6835/f3p0/3549	25.02333333	10.46333333	14.08333333	-1.1087	1.6187E-08	2.6855E-07	DOWN	0.28059	0.13314	0.27983		-0.83964	3.2123E-07	1.1912E-05	
transcript_HQ_CoA_transcript6842/f2p0/3543	1.893333333	10.27	0.606666667	2.5923	9.7913E-09	1.6967E-07	UP	-4.2077	2.057E-14	2.1766E-12	DOWN	-1.6254	0.0040217	0.025992	DOWN
transcript_HQ_CoA_transcript6852/f4p0/3529	9.546666667	21.87	10.11333333	1.3494	0.00056567	0.0031347	UP	-1.2669	9.2919E-08	2.3109E-06	DOWN	0.072643	0.84284	0.92775	
transcript_HQ_CoA_transcript6869/f2p0/3534	3.356666667	8.216666667	1.933333333	1.435	0.00029559	0.0017972	UP	-2.2311	1.6015E-08	4.7731E-07	DOWN	-0.80886	0.012533	0.059168	
transcript_HQ_CoA_transcript6872/f2p0/3521	3.886666667	1.216666667	1.796666667	-1.5207	0.0003769	0.0022206	DOWN	0.43238	0.42243	0.61169		-1.0983	0.034249	0.12269	
transcript_HQ_CoA_transcript6888/f2p0/3531	0.93	11.28	1.793333333	3.8114	3.2908E-13	1.1121E-11	UP	-2.8361	3.1996E-11	1.6999E-09	DOWN	0.95861	0.067917	0.19672	
transcript_HQ_CoA_transcript6889/f2p0/3538	1.796666667	4.693333333	3.293333333	1.528	0.00003603	0.00028928	UP	-0.65151	0.037625	0.11002		0.86493	0.011469	0.055666	
transcript_HQ_CoA_transcript6910/f22p0/3514	9.596666667	55.36333333	13.8	2.6896	9.2061E-26	8.4576E-24	UP	-2.1562	3.9931E-06	6.2078E-05	DOWN	0.51873	0.24089	0.44988	
transcript_HQ_CoA_transcript6911/f2p0/3554	6.513333333	0.876666667	1.156666667	-2.7237	2.8161E-07	3.6695E-06	DOWN	0.22827	0.67373	0.80981		-2.5051	2.8077E-09	1.8019E-07	DOWN
transcript_HQ_CoA_transcript6920/f3p0/3547	17.02	7.296666667	14.72333333	-1.0719	5.9912E-08	8.9267E-07	DOWN	0.86436	0.0007275	0.0049478		-0.21876	0.35979	0.57687	
transcript_HQ_CoA_transcript6921/f2p0/3537	8.096666667	3.116666667	6.706666667	-1.236	0.00264	0.011686	DOWN	0.95662	0.00032195	0.0025218		-0.2875	0.44372	0.65432	
transcript_HQ_CoA_transcript6928/f2p0/3537	26.93	0.983333333	0.463333333	-4.5309	6.1902E-07	7.5346E-06	DOWN	-1.2649	0.19954	0.37173		-5.8133	2.1885E-15	5.2855E-13	DOWN
transcript_HQ_CoA_transcript6929/f3p0/3539	5.89	25.19666667	8.29	2.2604	4.4804E-07	5.6001E-06	UP	-1.7524	4.0742E-06	6.3124E-05	DOWN	0.49255	0.28518	0.49923	
transcript_HQ_CoA_transcript693/f2p0/6099	10.93333333	3.646666667	5.63	-1.4303	9.9278E-12	2.8096E-10	DOWN	0.48415	0.022086	0.074103		-0.95941	2.1547E-07	8.469E-06	
transcript_HQ_CoA_transcript6930/f2p0/3537	14.63666667	6.113333333	6.45	-1.1116	0.0011415	0.0057491	DOWN	-0.067051	0.85253	0.91967		-1.1914	5.1242E-07	1.7813E-05	DOWN
transcript_HQ_CoA_transcript6942/f3p0/3592	14.63333333	2.466666667	17.13	-2.4421	2.2956E-09	4.4599E-08	DOWN	2.665	2.6E-09	9.1999E-08	UP	0.2134	0.48919	0.69139	
transcript_HQ_CoA_transcript6946/f2p0/3545	4.446666667	1.52	5.926666667	-1.3724	0.0020655	0.0095189	DOWN	1.7957	5.1592E-06	7.6694E-05	UP	0.41083	0.13815	0.31625	
transcript_HQ_CoA_transcript6957/f2p0/3540	7.996666667	3.483333333	6.156666667	-1.0256	0.0021337	0.0097861	DOWN	0.65726	0.071463	0.17884		-0.38245	0.14645	0.32801	
transcript_HQ_CoA_transcript6969/f6p0/3498	48.18	20.32666667	31.48333333	-1.0982	5.2042E-08	7.8956E-07	DOWN	0.48781	0.013782	0.051649		-0.62109	0.00056051	0.0056466	
transcript_HQ_CoA_transcript6992/f2p0/3492	9.936666667	3.48	1.7	-1.3917	0.010652	0.037078	DOWN	-1.1729	0.0063055	0.028093	DOWN	-2.5731	2.3487E-08	1.2144E-06	DOWN
transcript_HQ_CoA_transcript6994/f3p0/3531	7.04	23.18666667	11.77666667	1.8808	1.8528E-06	2.0592E-05	UP	-1.1473	0.00027729	0.0022311	DOWN	0.72207	0.020595	0.08493	
transcript_HQ_CoA_transcript7011/f2p0/3533	2.116666667	4.79	6.223333333	1.3398	0.00025045	0.0015558	UP	0.21956	0.55971	0.72691		1.5468	3.7401E-05	0.00063155	UP
transcript_HQ_CoA_transcript7014/f2p0/3530	4.906666667	11.48666667	6.353333333	1.3721	1.4956E-06	1.6983E-05	UP	-0.98752	0.0084052	0.035029		0.37313	0.35482	0.57205	
transcript_HQ_CoA_transcript7016/f2p0/3506	5.373333333	1.926666667	4.22	-1.3306	0.00024564	0.0015324	DOWN	0.98796	0.00055782	0.0039768		-0.35269	0.25881	0.4695	
transcript_HQ_CoA_transcript7032/f11p0/3436	25.79666667	7.933333333	19.49	-1.5321	0.00039899	0.0023335	DOWN	1.1206	0.0067575	0.029601	UP	-0.42229	0.10095	0.25884	
transcript_HQ_CoA_transcript7034/f2p0/3527	0.066666667	0.94	0.65	3.9555	0.00024246	0.0015149	UP	-0.63194	0.49207	0.67182		3.3099	0.011905	0.057037	
transcript_HQ_CoA_transcript706/f2p0/6064	0.82	15.66	1.38	4.4182	4.9817E-35	8.4329E-33	UP	-3.6576	7.7798E-18	1.3844E-15	DOWN	0.74424	0.15323	0.33747	
transcript_HQ_CoA_transcript7061/f2p0/3514	14.66666667	4.123333333	11.65333333	-1.6856	2.3999E-08	0.00000039	DOWN	1.352	8.2831E-07	0.00001629	UP	-0.34467	0.26243	0.47415	
transcript_HQ_CoA_transcript7071/f2p0/3516	32.91333333	0.833333333	4.43	-5.1692	8.197E-24	6.6016E-22	DOWN	2.2914	0.00065778	0.0045616	UP	-2.8851	5.7621E-08	2.6775E-06	DOWN
transcript_HQ_CoA_transcript7072/f2p0/3513	6.37	2.593333333	10.73	-1.1357	0.013398	0.044462	DOWN	1.8807	2.5885E-08	7.3558E-07	UP	0.73447	0.068952	0.19887	
transcript_HQ_CoA_transcript7075/f2p0/3519	7.233333333	2.433333333	4.79	-1.431	0.00028712	0.0017538	DOWN	0.85179	0.039482	0.11378		-0.59185	0.033563	0.12091	
transcript_HQ_CoA_transcript7077/f2p0/3519	1.566666667	3.07	2.51	1.1522	0.013058	0.043619	UP	-0.46264	0.27374	0.46107		0.67522	0.048401	0.15663	
transcript_HQ_CoA_transcript7113/f2p0/3518	32.12333333	3.256666667	14.10333333	-3.1514	6.1665E-13	2.0203E-11	DOWN	1.9495	2.4629E-09	8.7656E-08	UP	-1.2106	0.0023432	0.017203	DOWN
transcript_HQ_CoA_transcript7120/f2p0/3525	3.386666667	0.686666667	2.39	-2.1567	0.0095473	0.033881	DOWN	1.6784	0.044311	0.12447		-0.49402	0.21949	0.42532	
transcript_HQ_CoA_transcript7132/f4p0/3498	5.133333333	21.96333333	6.053333333	2.2443	2.1542E-06	2.3568E-05	UP	-2.0067	2.0019E-05	0.00024397	DOWN	0.22535	0.64695	0.80489	
transcript_HQ_CoA_transcript7133/f5p0/3512	7.72	16.1	14.56666667	1.2246	1.5691E-06	1.7751E-05	UP	-0.30314	0.31299	0.50448		0.90945	0.002192	0.016402	
transcript_HQ_CoA_transcript7137/f5p0/3490	40.73	12.88	35.90333333	-1.5146	1.7569E-09	3.4762E-08	DOWN	1.3301	4.9442E-05	0.00053781	UP	-0.19669	0.45869	0.66746	
transcript_HQ_CoA_transcript7163/f2p0/3482	48.8	17.00333333	28.98666667	-1.3887	0.0036474	0.015343	DOWN	0.65669	0.21933	0.39656		-0.74209	0.015089	0.067586	
transcript_HQ_CoA_transcript7166/f3p0/3497	13.88	2.573333333	8.173333333	-2.2892	6.5298E-13	2.1317E-11	DOWN	1.5234	0.0003256	0.0025461	UP	-0.77781	0.020458	0.084496	
transcript_HQ_CoA_transcript7175/f5p0/3475	68.59333333	20.50333333	21.33333333	-1.5711	0.00058101	0.0032081	DOWN	-0.1064	0.73237	0.84831		-1.6923	0.00023171	0.0028055	DOWN
transcript_HQ_CoA_transcript7176/f2p0/2996	2.563333333	0.433333333	3.883333333	-2.3763	1.6286E-06	1.8333E-05	DOWN	2.9703	1.513E-10	7.0698E-09	UP	0.58277	0.11294	0.27801	
transcript_HQ_CoA_transcript7178/f3p0/3508	2.953333333	11.98	5.093333333	2.1818	1.4854E-06	1.6878E-05	UP	-1.3915	0.0010404	0.0065981	DOWN	0.77716	0.017599	0.075599	
transcript_HQ_CoA_transcript7209/f3p0/3514	2.483333333	15.48333333	3.286666667	2.7671	1.1153E-06	1.2979E-05	UP	-2.3556	4.5705E-05	0.00050013	DOWN	0.39946	0.45933	0.66795	
transcript_HQ_CoA_transcript7217/f2p0/3590	2.8	8.966666667	5.796666667	1.8369	2.7487E-11	7.3361E-10	UP	-0.76922	0.015293	0.055908		1.0547	0.0013387	0.011292	UP
transcript_HQ_CoA_transcript7220/f2p0/3459	4.29	7.803333333	4.253333333	1.019	0.0013693	0.0067404	UP	-1.0351	0.00051195	0.0037207	DOWN	-0.027415	0.91615	0.96294	
transcript_HQ_CoA_transcript7226/f7p0/3402	6.796666667	16.22666667	8.646666667	1.3953	5.9718E-07	7.2735E-06	UP	-1.0448	0.0029067	0.015041	DOWN	0.33723	0.27978	0.4936	
transcript_HQ_CoA_transcript7229/f3p0/3486	3.573333333	12.54	6.476666667	1.9622	2.7519E-08	4.4131E-07	UP	-1.1041	0.0013224	0.0079891	DOWN	0.84648	0.030638	0.11332	
transcript_HQ_CoA_transcript7236/f2p0/3543	2.05	0.466666667	1.38	-2.0123	0.0033042	0.014107	DOWN	1.4385	0.042099	0.11969		-0.58379	0.14962	0.3326	
transcript_HQ_CoA_transcript7238/f16p0/3466	32.72	75.07666667	33.53666667	1.3623	8.4043E-09	1.4718E-07	UP	-1.3205	1.3701E-12	1.025E-10	DOWN	0.028925	0.89513	0.95352	
transcript_HQ_CoA_transcript7239/f3p0/3568	0.156666667	3.34	14.38	4.6036	0.00092662	0.004818	UP	1.9052	NA	NA	NA	6.4882	8.8487E-34	2.707E-30	UP
transcript_HQ_CoA_transcript7248/f5p0/3505	15.87333333	4.353333333	28.12666667	-1.7388	0.00020805	0.0013323	DOWN	2.5743	6.1093E-09	1.996E-07	UP	0.82695	0.034994	0.1245	
transcript_HQ_CoA_transcript726/f2p0/6044	5.403333333	10.82333333	6.83	1.169	0.00033575	0.00201	UP	-0.82129	0.0069234	0.030114		0.33493	0.2423	0.45105	
transcript_HQ_CoA_transcript7262/f2p0/3486	4.013333333	0.546666667	3.596666667	-2.7523	1.6185E-06	1.8231E-05	DOWN	2.5852	1.8941E-05	0.00023284	UP	-0.17617	0.60807	0.77889	
transcript_HQ_CoA_transcript7275/f2p0/3483	1.833333333	9.323333333	2.353333333	2.4773	4.7654E-07	5.9185E-06	UP	-2.1045	1.8627E-05	0.00022944	DOWN	0.36096	0.34521	0.56308	
transcript_HQ_CoA_transcript7315/f3p0/3470	0.973333333	3.043333333	2.79	1.8362	0.01121	0.038653	UP	-0.32182	0.64667	0.78992		1.5025	0.0058321	0.034365	UP
transcript_HQ_CoA_transcript7316/f2p0/3487	1.886666667	22.83333333	3.5	3.7477	5.0404E-69	3.0716E-66	UP	-2.8468	6.3219E-12	3.9413E-10	DOWN	0.88764	0.070954	0.20239	
transcript_HQ_CoA_transcript7332/f2p0/3494	14.13666667	2.82	9.676666667	-2.2089	0.0019409	0.0090434	DOWN	1.6624	0.022341	0.074762		-0.55776	0.0021717	0.01629	
transcript_HQ_CoA_transcript7335/f2p0/3477	3.013333333	5.76	5.88	1.0876	0.00073156	0.0039095	UP	-0.11304	0.64877	0.79132		0.96244	0.0018925	0.014614	
transcript_HQ_CoA_transcript7337/f9p0/3462	9.613333333	21.61666667	11.89	1.327	4.4183E-06	4.5051E-05	UP	-1.0063	0.00020595	0.0017673	DOWN	0.30697	0.25684	0.46734	
transcript_HQ_CoA_transcript7350/f3p0/3457	20.16333333	7.863333333	10.91666667	-1.1827	0.0022094	0.010073	DOWN	0.30609	0.44966	0.63712		-0.89079	1.0092E-05	0.00021317	
transcript_HQ_CoA_transcript7369/f3p0/3464	14.34333333	5.11	5.536666667	-1.3308	9.5852E-09	1.6642E-07	DOWN	-0.037728	0.89822	0.94587		-1.3803	2.0921E-06	5.9079E-05	DOWN
transcript_HQ_CoA_transcript7384/f2p0/3461	10.67666667	1.99	18.47333333	-2.2566	1.4109E-10	3.3585E-09	DOWN	3.0434	2.4234E-16	3.2661E-14	UP	0.7743	0.0063735	0.03657	
transcript_HQ_CoA_transcript7386/f4p0/3401	37.67333333	15.01333333	29.72	-1.1687	5.6256E-06	5.6262E-05	DOWN	0.829	0.0014393	0.0085567		-0.35126	0.023844	0.094037	
transcript_HQ_CoA_transcript7389/f2p0/3455	38.54	9.303333333	24.73	-1.892	0.00034002	0.0020308	DOWN	1.256	6.7984E-11	3.4327E-09	UP	-0.64829	0.18684	0.38369	
transcript_HQ_CoA_transcript7399/f4p0/3449	3.953333333	0.446666667	0.616666667	-3.0191	0.0013718	0.0067488	DOWN	0.29458	0.82707	0.90574		-2.7375	0.028625	0.108	
transcript_HQ_CoA_transcript7414/f3p0/3456	5.906666667	28.33	8.176666667	2.4336	2.5295E-09	4.8729E-08	UP	-1.9454	1.5082E-05	0.00019117	DOWN	0.47343	0.13881	0.3171	
transcript_HQ_CoA_transcript7426/f4p0/3422	7.313333333	2.293333333	0.353333333	-1.5224	5.5652E-05	0.00042323	DOWN	-2.8148	1.2081E-09	4.6518E-08	DOWN	-4.3469	2.8691E-19	1.1448E-16	DOWN
transcript_HQ_CoA_transcript7427/f5p0/3464	53.04333333	15.76	31.09333333	-1.6037	9.5616E-06	9.0339E-05	DOWN	0.83207	0.00014094	0.001275		-0.78386	0.022267	0.089473	
transcript_HQ_CoA_transcript7452/f2p0/3453	4.26	11.74	4.956666667	1.6262	1.6465E-05	0.00014458	UP	-1.3998	1.9485E-05	0.00023825	DOWN	0.21372	0.4578	0.66662	
transcript_HQ_CoA_transcript7455/f2p0/3463	14.42333333	27.09	10.17	1.0711	0.0031118	0.013405	UP	-1.5511	6.0203E-10	2.4576E-08	DOWN	-0.49389	0.13689	0.31435	

transcript_HQ_CoA_transcript7465/f3p0/3460	6.143333333	2.23	3.383333333	-1.3029	6.7778E-08	1.0025E-06	DOWN	0.44577	0.1363	0.28494		-0.86935	0.0017099	0.013546	
transcript_HQ_CoA_transcript7471/f2p0/3470	14.14333333	2.933333333	2.96	-2.112	3.1506E-13	1.0686E-11	DOWN	-0.1108	0.82984	0.90723		-2.2365	1.0629E-06	3.3181E-05	DOWN
transcript_HQ_CoA_transcript7475/f2p0/3423	10.24666667	4.42	13.50333333	-1.0547	0.00038934	0.0022851	DOWN	1.4612	4.3352E-11	2.2383E-09	UP	0.39249	0.16214	0.3505	
transcript_HQ_CoA_transcript7480/f2p0/3493	3.933333333	1.493333333	2.653333333	-1.2442	0.0080634	0.029489	DOWN	0.66738	0.10738	0.2404		-0.58891	0.19733	0.39707	
transcript_HQ_CoA_transcript7481/f4p0/3426	8.493333333	3.12	8.65	-1.2826	2.6707E-08	4.3018E-07	DOWN	1.3168	5.2287E-10	2.1732E-08	UP	0.021478	0.90896	0.95985	
transcript_HQ_CoA_transcript7486/f2p0/3475	1.193333333	4.4	1.4	2.0448	0.0084348	0.030608	UP	-1.7981	9.0197E-05	0.00088645	DOWN	0.2314	0.79325	0.90087	
transcript_HQ_CoA_transcript7491/f2p0/3440	6.233333333	18.51666667	9.43	1.7414	5.0025E-07	6.1878E-06	UP	-1.1409	0.00029377	0.0023431	DOWN	0.58592	0.050301	0.16078	
transcript_HQ_CoA_transcript7499/f4p0/3412	5.146666667	0.506666667	17.85	-3.1843	4.0787E-07	5.1354E-06	DOWN	4.9684	1.1451E-14	1.2644E-12	UP	1.7712	1.4592E-05	0.00029075	UP
transcript_HQ_CoA_transcript7501/f10p0/3438	3.813333333	17.52	5.783333333	2.3598	5.3002E-08	8.0214E-07	UP	-1.7565	0.00082149	0.0054476	DOWN	0.5896	0.22188	0.42815	
transcript_HQ_CoA_transcript7524/f3p0/3435	1.72	28.29666667	5.836666667	4.1901	3.2137E-29	4.1375E-27	UP	-2.4113	8.2493E-07	1.6241E-05	DOWN	1.7649	0.00085509	0.0079029	UP
transcript_HQ_CoA_transcript7527/f2p0/3483	19.12666667	5.573333333	9.4	-1.6306	3.6618E-07	4.6619E-06	DOWN	0.603	0.014164	0.052731		-1.0369	0.00036753	0.0040517	DOWN
transcript_HQ_CoA_transcript7532/f2p0/3436	10.37666667	3.003333333	8.52	-1.6356	2.6368E-10	6.0107E-09	DOWN	1.3547	7.5604E-06	0.0001066	UP	-0.29226	0.29291	0.50816	
transcript_HQ_CoA_transcript7543/f2p0/3438	1.14	2.833333333	0.44	1.4826	0.0090789	0.032538	UP	-2.8571	9.6209E-07	1.8427E-05	DOWN	-1.3867	0.0104	0.051774	
transcript_HQ_CoA_transcript7551/f4p0/3440	6.686666667	17.26333333	11.16666667	1.5119	3.8029E-05	0.00030287	UP	-0.76546	0.0022799	0.012345		0.73638	0.050539	0.16116	
transcript_HQ_CoA_transcript7567/f2p0/3434	2.453333333	8.296666667	10	1.8881	0.0014693	0.0071329	UP	0.13401	0.8274	0.90591		2.011	3.3866E-07	1.2482E-05	UP
transcript_HQ_CoA_transcript7568/f2p0/3396	1.416666667	7.06	3.636666667	2.4803	4.2713E-12	1.2567E-10	UP	-1.1004	0.0084147	0.035053	DOWN	1.366	0.0041671	0.026632	UP
transcript_HQ_CoA_transcript7569/f2p0/3496	5.586666667	2.176666667	3.116666667	-1.1896	0.002866	0.012529	DOWN	0.35037	0.38928	0.58031		-0.85164	0.0010022	0.0089774	
transcript_HQ_CoA_transcript7572/f2p0/3447	5.063333333	0.546666667	2.55	-3.031	4.9436E-05	0.00038054	DOWN	2.0551	0.008091	0.034037	UP	-0.98477	0.10166	0.26007	
transcript_HQ_CoA_transcript7583/f2p0/3427	17.33	5.993333333	5.883333333	-1.3803	0.0059488	0.022867	DOWN	-0.14397	0.81892	0.90112		-1.535	0.0041319	0.026499	DOWN
transcript_HQ_CoA_transcript7585/f2p0/3422	8.38	2.79	6.836666667	-1.4123	0.00040588	0.0023684	DOWN	1.1566	0.01734	0.061607		-0.26728	0.60699	0.77797	
transcript_HQ_CoA_transcript7588/f2p0/3452	9.173333333	20.59666667	11.3	1.3381	1.0241E-06	1.1994E-05	UP	-1.0218	1.3685E-05	0.00017711	DOWN	0.30179	0.23709	0.44547	
transcript_HQ_CoA_transcript7598/f6p0/3433	53.83666667	5.7	6.586666667	-3.1173	5.2387E-10	1.1334E-08	DOWN	0.075971	0.79912	0.88937		-3.0486	8.1678E-11	7.5337E-09	DOWN
transcript_HQ_CoA_transcript7607/f3p0/3436	0.753333333	3.16	2.11	2.2242	0.0018674	0.0087606	UP	-0.7176	0.3042	0.49478		1.495	0.032882	0.11909	
transcript_HQ_CoA_transcript7608/f2p0/3427	11.49333333	30.55666667	13.22666667	1.5808	1.0954E-05	0.0001014	UP	-1.3628	3.6385E-05	0.00041243	DOWN	0.20389	0.35699	0.57403	
transcript_HQ_CoA_transcript7609/f2p0/3438	1.933333333	10.52333333	6.216666667	2.617	3.7396E-18	2.0287E-16	UP	-0.90658	0.00011577	0.0010821		1.698	1.6298E-06	4.7335E-05	UP
transcript_HQ_CoA_transcript761/f2p0/5979	10.92	21.44666667	18.26666667	1.1285	0.0061972	0.023683	UP	-0.39125	0.15744	0.31555		0.72731	0.033396	0.1205	
transcript_HQ_CoA_transcript7612/f2p0/3454	6.323333333	2.636666667	5.126666667	-1.1193	0.0036896	0.015489	DOWN	0.80919	0.00065951	0.0045702		-0.31915	0.35807	0.57517	
transcript_HQ_CoA_transcript7635/f2p0/3442	1.07	3.05	1.64	1.6897	0.00043842	0.0025229	UP	-1.0608	0.0032265	0.016464	DOWN	0.61338	0.22499	0.4322	
transcript_HQ_CoA_transcript7637/f2p0/3437	32.83666667	11.89	19.22666667	-1.2794	0.0011941	0.0059826	DOWN	0.52272	0.11731	0.25599		-0.77159	0.0093954	0.048064	
transcript_HQ_CoA_transcript7639/f3p0/3405	1.783333333	4.08	1.05	1.3357	0.0040388	0.016679	UP	-2.0812	2.6417E-06	4.3739E-05	DOWN	-0.75815	0.1139	0.2794	
transcript_HQ_CoA_transcript7646/f2p0/3432	4.28	15.80666667	4.86	2.0476	2.8337E-11	7.5408E-10	UP	-1.8483	2.5409E-13	2.1364E-11	DOWN	0.18648	0.56233	0.74593	
transcript_HQ_CoA_transcript7653/f3p0/3427	19.00666667	7.396666667	12.15	-1.2239	7.8315E-05	0.00056884	DOWN	0.57211	0.066712	0.16987		-0.66203	0.0064476	0.036811	
transcript_HQ_CoA_transcript7665/f2p0/3428	2.22	7.47	2.613333333	1.9226	2.7475E-06	2.9272E-05	UP	-1.684	1.4967E-05	0.00019024	DOWN	0.22499	0.53565	0.72791	
transcript_HQ_CoA_transcript7678/f2p0/3421	21.14	6.956666667	16.93666667	-1.4385	0.00034505	0.0020555	DOWN	1.1174	0.001651	0.009531	UP	-0.33218	0.25647	0.46684	
transcript_HQ_CoA_transcript7686/f2p0/3410	2.126666667	0.543333333	0.723333333	-1.8142	0.0049061	0.019554	DOWN	0.3003	0.66548	0.80442		-1.5263	0.015004	0.067322	
transcript_HQ_CoA_transcript769/f10p0/5942	6.85	13.01	14.03	1.088	0.0090274	0.032399	UP	-0.050828	0.89821	0.94587		1.0254	0.0021177	0.015983	UP
transcript_HQ_CoA_transcript7691/f4p0/3399	2.23	7.273333333	8.56	1.9123	0.0053675	0.021035	UP	0.045674	0.94305	0.96971		1.9428	2.4732E-12	3.3878E-10	UP
transcript_HQ_CoA_transcript7696/f2p0/3407	5.866666667	2.453333333	3.48	-1.115	0.001426	0.0069596	DOWN	0.36024	0.26879	0.45533		-0.76619	0.030736	0.1136	
transcript_HQ_CoA_transcript7697/f3p0/3418	12.38666667	27.60666667	24.18	1.3533	0.015077	0.048769	UP	-0.36826	0.47731	0.66028		0.97003	0.0021128	0.015959	
transcript_HQ_CoA_transcript7704/f3p0/3406	13.95333333	3.503333333	12.21666667	-1.8321	1.4642E-12	4.5603E-11	DOWN	1.6556	9.509E-17	1.3511E-14	UP	-0.19022	0.37638	0.59294	
transcript_HQ_CoA_transcript7706/f3p0/3409	2.933333333	0.156666667	6.643333333	-4.0354	0.00002516	0.00021013	DOWN	5.2225	4.0752E-19	8.2081E-17	UP	1.1828	0.053867	0.16895	
transcript_HQ_CoA_transcript7708/f3p0/3412	15.88333333	2.49	17.25666667	-2.5096	2.508E-06	2.7019E-05	DOWN	2.6585	8.5554E-10	3.4089E-08	UP	0.13758	0.79614	0.90249	
transcript_HQ_CoA_transcript7724/f2p0/3409	4.213333333	19.05333333	6.086666667	2.3474	1.0523E-17	5.5443E-16	UP	-1.7982	9.6933E-12	5.7312E-10	DOWN	0.53617	0.04003	0.13732	
transcript_HQ_CoA_transcript7728/f2p0/3406	9.043333333	2.686666667	5.396666667	-1.595	6.5538E-05	0.00048805	DOWN	0.84464	0.00060345	0.0042381		-0.76215	0.03536	0.12549	
transcript_HQ_CoA_transcript7735/f2p0/3408	5.976666667	15.17666667	6.696666667	1.507	0.00043964	0.0025275	UP	-1.3063	0.00030157	0.0023897	DOWN	0.18676	0.67654	0.82609	
transcript_HQ_CoA_transcript7737/f2p0/3419	6.433333333	2.593333333	8.616666667	-1.1544	0.0014115	0.0069129	DOWN	1.5729	1.4269E-08	4.3015E-07	UP	0.40772	0.11316	0.27838	
transcript_HQ_CoA_transcript7740/f3p0/3395	0.78	0.206666667	0.316666667	-1.6864	0.01468	0.047727	DOWN	0.34707	0.6789	0.81331		-1.3479	0.045565	0.15042	
transcript_HQ_CoA_transcript7741/f3p0/3422	6.646666667	17.66	4.23	1.5692	0.0001268	0.00086497	UP	-2.2239	3.5987E-10	1.5705E-08	DOWN	-0.66595	0.049866	0.15985	
transcript_HQ_CoA_transcript7748/f2p0/3389	0.216666667	1.006666667	3.93	2.3131	0.0077413	0.028505	UP	1.8428	0.013288	0.050207		4.1486	3.3227E-08	1.6579E-06	UP
transcript_HQ_CoA_transcript7749/f3p0/3443	18.26666667	6.23	13.07333333	-1.4074	3.5801E-08	5.6085E-07	DOWN	0.92371	4.8695E-06	7.3337E-05		-0.49451	0.030955	0.11425	
transcript_HQ_CoA_transcript7754/f2p0/3449	1.113333333	5.536666667	3.826666667	2.4895	1.3662E-06	1.5669E-05	UP	-0.68245	0.29239	0.48214		1.7914	0.011473	0.055666	
transcript_HQ_CoA_transcript7759/f5p0/3405	5.363333333	14.75	8.243333333	1.6089	0.00012694	0.00086564	UP	-0.98432	0.033632	0.10147		0.61226	0.18913	0.38661	
transcript_HQ_CoA_transcript7766/f2p0/3419	6.826666667	19.93666667	6.48	1.723	2.5699E-05	0.00021424	UP	-1.7908	1.7061E-05	0.00021244	DOWN	-0.079927	0.76792	0.88471	
transcript_HQ_CoA_transcript7772/f2p0/3400	2.846666667	0.856666667	2.306666667	-1.5826	0.00017775	0.0011627	DOWN	1.2797	0.00016192	0.0014386	UP	-0.31339	0.39884	0.61457	
transcript_HQ_CoA_transcript7785/f2p0/3403	6.533333333	2.76	5.56	-1.099	3.7197E-05	0.00029696	DOWN	0.86827	0.0096095	0.038778		-0.24321	0.45332	0.66323	
transcript_HQ_CoA_transcript7791/f2p0/3393	10.12333333	3.69	7.443333333	-1.2832	7.0733E-05	0.00052059	DOWN	0.85904	0.00018886	0.0016413		-0.43668	0.14227	0.32203	
transcript_HQ_CoA_transcript7802/f2p0/3407	6.563333333	2.576666667	6.48	-1.1762	0.00019808	0.0012755	DOWN	1.1628	0.0014957	0.0088093	UP	-0.025556	0.93821	0.97271	
transcript_HQ_CoA_transcript7819/f2p0/3394	7.34	14.38666667	13.44	1.1348	0.0023873	0.010744	UP	-0.25187	0.37046	0.56242		0.87033	0.0075102	0.040893	
transcript_HQ_CoA_transcript7825/f2p0/3402	11.19	3.213333333	8.376666667	-1.6734	0.0026195	0.011604	DOWN	1.2398	0.021002	0.071195		-0.44312	0.4228	0.63471	
transcript_HQ_CoA_transcript7826/f2p0/3397	2.246666667	0.206666667	0.216666667	-3.2882	6.0411E-09	1.0828E-07	DOWN	-0.10913	0.90408	0.94879		-3.4163	5.2136E-08	2.4576E-06	DOWN
transcript_HQ_CoA_transcript7832/f2p0/3381	16.84	44.71666667	18.41	1.5747	2.2437E-06	2.4475E-05	UP	-1.4337	3.5773E-05	0.0004065	DOWN	0.12692	0.60976	0.77964	
transcript_HQ_CoA_transcript7846/f2p0/3406	6.61	13.76666667	8.096666667	1.2149	0.00089814	0.0046874	UP	-0.91136	0.0037768	0.018755		0.28938	0.35147	0.56904	
transcript_HQ_CoA_transcript7856/f2p0/3415	60.98333333	152.3733333	95.55666667	1.5182	0.0015352	0.0074173	UP	-0.85518	0.056769	0.15084		0.64788	0.019027	0.080285	
transcript_HQ_CoA_transcript7859/f2p0/3379	3.533333333	8.316666667	4.186666667	1.4002	0.00053189	0.0029755	UP	-1.1397	0.00332	0.016852	DOWN	0.24776	0.549	0.73764	
transcript_HQ_CoA_transcript7865/f12p0/3335	7.586666667	14.13	9.316666667	1.0357	0.013583	0.044977	UP	-0.73137	0.062176	0.1614		0.29165	0.29763	0.51326	
transcript_HQ_CoA_transcript7866/f2p0/3395	8.08	1.816666667	3.73	-1.9837	1.9968E-13	6.9403E-12	DOWN	0.88713	0.017131	0.061072		-1.1119	0.00057574	0.005759	DOWN
transcript_HQ_CoA_transcript7871/f3p0/3348	9.286666667	3.986666667	5.636666667	-1.0653	3.3372E-06	3.4903E-05	DOWN	0.34362	0.18114	0.34794		-0.73304	0.0035104	0.023413	
transcript_HQ_CoA_transcript7876/f3p0/3407	6.983333333	28.09333333	9.176666667	2.1453	1.7813E-06	1.9869E-05	UP	-1.7641	4.048E-16	5.2622E-14	DOWN	0.37229	0.41359	0.62748	
transcript_HQ_CoA_transcript7880/f5p0/3377	4.893333333	47.5	20.74	3.456	1.7386E-13	6.1242E-12	UP	-1.3532	0.00058839	0.0041591	DOWN	2.0885	3.6648E-08	1.7986E-06	UP
transcript_HQ_CoA_transcript7882/f3p0/3398	4.926666667	12.63	7.763333333	1.5077	0.00040945	0.0023832	UP	-0.83432	0.01737	0.061647		0.66021	0.047437	0.15457	
transcript_HQ_CoA_transcript7901/f3p0/3333	32.28333333	7.546666667	31.15	-1.9442	4.4312E-10	9.7136E-09	DOWN	1.8791	3.5617E-05	0.00040498	UP	-0.077251	0.84256	0.92751	
transcript_HQ_CoA_transcript7904/f3p0/3365	10.67333333	29.52333333	13.02666667	1.6287	2.6586E-09	5.1108E-08	UP	-1.3323	0.00000569	8.3568E-05	DOWN	0.28322	0.28383	0.49787	
transcript_HQ_CoA_transcript7912/f3p0/3386	11.99	2.403333333	5.653333333	-2.1395	0.0073701	0.027316	DOWN	1.0817	0.0052957	0.024499	UP	-1.0715	0.15848	0.34548	
transcript_HQ_CoA_transcript7915/f3p0/3388	7.24	2.81	6.6	-1.2474	0.015428	0.049563	DOWN	1.1076	0.074804	0.18486		-0.14813	0.73738	0.86622	
transcript_HQ_CoA_transcript7916/f9p0/3356	8.936666667	3.023333333	11.01333333	-1.405	3.8549E-10	8.5632E-09	DOWN	1.712	4.8565E-15	5.6338E-13	UP	0.29443	0.17583	0.36933	
transcript_HQ_CoA_transcript7918/f4p0/3371	15.40666667	6.71	15.76	-1.0606	0.0045397	0.018293	DOWN	1.1057	0.0054164	0.024938	UP	0.035557	0.91783	0.96405	
transcript_HQ_CoA_transcript7924/f2p0/3403	1.73	13.59	6.1	3.1389	2.1248E-12	6.5068E-11	UP	-1.2795	0.011239	0.043961	DOWN	1.8458	0.00093504	0.0084712	UP
transcript_HQ_CoA_transcript7927/f2p0/3377	36.36	8.423333333	12.84666667	-1.9891	0.00010717	0.00074785	DOWN	0.46588	0.23644	0.41788		-1.5313	0.00060676	0.0060233	DOWN
transcript_HQ_CoA_transcript793/f2p0/5946	5.66	1.43	10	-1.8351	0.014389	0.04705	DOWN	2.6416	3.1279E-10	1.3949E-08	UP	0.79709	0.19228	0.39075	

transcript_HQ_CoA_transcript7940/f2p0/3391	5.543333333	12.67	11.11666667	1.3436	4.1556E-08	6.4113E-07	UP	-0.31904	0.36082	0.55291		1.0128	0.0033357	0.022493	UP
transcript_HQ_CoA_transcript7946/f2p0/3373	13.24666667	32.09333333	17.82666667	1.4434	5.9837E-05	0.00045018	UP	-1.0198	0.009241	0.037656	DOWN	0.40997	0.11194	0.27638	
transcript_HQ_CoA_transcript7962/f2p0/3366	3.936666667	16.09333333	1.883333333	2.1976	1.3373E-08	2.2492E-07	UP	-3.2538	6.9803E-13	5.6115E-11	DOWN	-1.0706	0.0068469	0.038306	DOWN
transcript_HQ_CoA_transcript7965/f2p0/3369	4.55	0.043333333	0.076666667	-6.4818	4.8896E-09	8.9214E-08	DOWN	0.63569	0.78076	0.87724		-5.8958	7.7118E-09	4.5223E-07	DOWN
transcript_HQ_CoA_transcript7980/f2p0/3415	7.13	21.85333333	6.64	1.7901	4.5058E-07	5.6267E-06	UP	-1.8738	5.7467E-07	1.1862E-05	DOWN	-0.09807	0.75104	0.87449	
transcript_HQ_CoA_transcript7985/f2p0/3367	6.75	2.556666667	6.033333333	-1.2741	0.012347	0.041755	DOWN	1.1053	0.024236	0.079639		-0.17812	0.59583	0.77018	
transcript_HQ_CoA_transcript7986/f2p0/3372	11.48333333	3.956666667	4.033333333	-1.3871	0.00014025	0.00094601	DOWN	-0.1313	0.63517	0.78224		-1.5293	8.1078E-06	0.0001793	DOWN
transcript_HQ_CoA_transcript7998/f2p0/3357	2.983333333	12.63	3.346666667	2.2458	9.1187E-09	1.5877E-07	UP	-2.0727	2.4258E-08	6.9257E-07	DOWN	0.15911	0.58584	0.76312	
transcript_HQ_CoA_transcript8003/f8p0/3326	14.17333333	43.63666667	20.27333333	1.7923	3.5289E-09	6.6442E-08	UP	-1.2685	0.00022403	0.0018798	DOWN	0.50845	0.088563	0.23576	
transcript_HQ_CoA_transcript8004/f3p0/3366	6.516666667	30.05333333	5.566666667	2.3734	7.0625E-07	8.5226E-06	UP	-2.5734	3.7415E-09	1.2915E-07	DOWN	-0.21519	0.59638	0.77034	
transcript_HQ_CoA_transcript8012/f2p0/3361	7.24	0.386666667	0.13	-4.0745	0.0006242	0.0034122	DOWN	-1.7293	0.25541	0.43944		-5.8138	0.0014226	0.011824	DOWN
transcript_HQ_CoA_transcript8035/f2p0/3370	0.786666667	8.956666667	0.586666667	3.6727	0.00054648	0.0030431	UP	-4.098	0.096271	0.22226		-0.42838	0.90864	0.95967	
transcript_HQ_CoA_transcript8045/f2p0/3351	25.00666667	5.87	18.56333333	-1.9384	3.7043E-08	5.7771E-07	DOWN	1.5103	0.0000866	0.00085476	UP	-0.44051	0.16971	0.36053	
transcript_HQ_CoA_transcript8046/f2p0/3365	29.40333333	11.80666667	25.67333333	-1.1743	1.5396E-05	0.00013617	DOWN	0.97167	5.6182E-05	0.00059489		-0.21342	0.41087	0.62508	
transcript_HQ_CoA_transcript8049/f2p0/3366	17.07666667	3.36	14.01333333	-2.2249	0.0028944	0.012626	DOWN	1.9178	5.8476E-10	2.3924E-08	UP	-0.31218	0.6564	0.81231	
transcript_HQ_CoA_transcript8052/f5p0/3361	8.44	34.53666667	8.533333333	2.1956	3.8312E-12	1.1352E-10	UP	-2.1671	7.1028E-12	4.3396E-10	DOWN	0.015534	0.95674	0.9815	
transcript_HQ_CoA_transcript8057/f3p0/3357	3.67	34.51666667	5.903333333	3.3945	4.9293E-14	1.8391E-12	UP	-2.6996	5.206E-09	1.7349E-07	DOWN	0.68177	0.040662	0.1388	
transcript_HQ_CoA_transcript8064/f4p0/3312	17.78666667	5.863333333	3.136666667	-1.4509	0.0030284	0.013098	DOWN	-1.0424	0.019011	0.066082		-2.5069	1.0891E-12	1.5866E-10	DOWN
transcript_HQ_CoA_transcript807/f2p0/5930	0.643333333	1.28	0.916666667	1.158	0.0015159	0.0073335	UP	-0.63631	0.02782	0.088374		0.5092	0.20305	0.40516	
transcript_HQ_CoA_transcript8082/f2p0/3358	4.726666667	0.603333333	1.496666667	-2.8185	1.7983E-08	2.9645E-07	DOWN	1.1838	0.10093	0.23015		-1.6473	0.0026214	0.018683	DOWN
transcript_HQ_CoA_transcript809/f2p0/5916	2.503333333	0.516666667	0.576666667	-2.1292	0.00010786	0.00075152	DOWN	0.015154	0.98053	0.98987		-2.1276	0.00046622	0.0048955	DOWN
transcript_HQ_CoA_transcript8092/f2p0/3364	3.343333333	13.52333333	8.603333333	2.1671	3.9721E-12	1.175E-10	UP	-0.79514	0.0081011	0.034069		1.3574	3.9604E-05	0.00066206	UP
transcript_HQ_CoA_transcript8095/f14p0/3319	105.7066667	33.50333333	84.58333333	-1.4916	0.00002302	0.00019439	DOWN	1.1724	0.0017544	0.010027	UP	-0.33243	0.37638	0.59294	
transcript_HQ_CoA_transcript8099/f5p0/3369	16.60666667	68.49333333	17.42333333	2.2047	7.4812E-10	1.5848E-08	UP	-2.1383	1.1598E-09	4.4847E-08	DOWN	0.055004	0.80588	0.90826	
transcript_HQ_CoA_transcript8102/f2p0/3349	24.29666667	8.83	25.25	-1.3032	0.00063361	0.0034547	DOWN	1.3678	1.8929E-08	5.5246E-07	UP	0.051376	0.88804	0.94992	
transcript_HQ_CoA_transcript8107/f2p0/3355	3.806666667	9.036666667	4.02	1.4003	0.011145	0.038481	UP	-1.3185	0.006318	0.028128	DOWN	0.071171	0.86458	0.93949	
transcript_HQ_CoA_transcript8127/f2p0/3346	29.19	5.95	20.00333333	-2.1785	4.6559E-05	0.00036128	DOWN	1.6219	0.00012433	0.0011498	UP	-0.56553	0.13598	0.31304	
transcript_HQ_CoA_transcript8128/f2p0/3359	1.456666667	4.633333333	2.106666667	1.8251	0.0022483	0.010222	UP	-1.2755	0.053142	0.14314		0.53432	0.3043	0.52079	
transcript_HQ_CoA_transcript8131/f3p0/3445	0.79	14.70333333	2.103333333	4.3917	3.9331E-15	1.6722E-13	UP	-2.9645	2.0496E-07	4.7196E-06	DOWN	1.4114	0.015162	0.067844	
transcript_HQ_CoA_transcript8139/f4p0/3336	10.38	24.7	21.23333333	1.4002	0.00042514	0.0024596	UP	-0.348	0.30477	0.49553		1.038	0.0002062	0.0025487	UP
transcript_HQ_CoA_transcript8150/f2p0/3348	9.706666667	0.763333333	10.84	-3.5062	4.6029E-23	3.5063E-21	DOWN	3.6623	3.0027E-30	2.3929E-27	UP	0.14378	0.55793	0.74351	
transcript_HQ_CoA_transcript8151/f9p0/3310	25.93	7.53	7.51	-1.6441	1.8896E-10	4.4007E-09	DOWN	-0.14602	0.56103	0.7278		-1.8008	5.5601E-18	1.9626E-15	DOWN
transcript_HQ_CoA_transcript8182/f5p0/3287	6.73	55.98666667	8.916666667	3.224	1.0736E-30	1.4647E-28	UP	-2.808	5.8946E-22	1.8955E-19	DOWN	0.40265	0.085354	0.22985	
transcript_HQ_CoA_transcript8213/f4p0/3332	11.55333333	22.55333333	25.77333333	1.124	1.4212E-05	0.00012711	UP	0.052182	0.86712	0.92807		1.1639	0.00069173	0.006679	UP
transcript_HQ_CoA_transcript8222/f2p0/3379	1.213333333	8.596666667	0.816666667	2.9911	1.2228E-15	5.3741E-14	UP	-3.5431	4.5193E-21	1.2551E-18	DOWN	-0.5668	0.21992	0.42571	
transcript_HQ_CoA_transcript8224/f2p0/3291	3.103333333	0.836666667	2.266666667	-1.7623	0.0019963	0.0092584	DOWN	1.3015	0.0037503	0.018638	UP	-0.46699	0.33424	0.55181	
transcript_HQ_CoA_transcript8255/f2p0/3338	7.66	2.71	6.623333333	-1.34	1.6096E-05	0.00014168	DOWN	1.1485	0.00085474	0.0056213	UP	-0.2029	0.55797	0.74351	
transcript_HQ_CoA_transcript8259/f2p0/3350	16.97	5.893333333	0.086666667	-1.3844	0.013192	0.043943	DOWN	-6.1647	1.5778E-07	3.7315E-06	DOWN	-7.538	3.5643E-05	0.00060858	DOWN
transcript_HQ_CoA_transcript8261/f9p0/3274	64.16333333	23.61333333	29.05333333	-1.2979	4.7905E-08	7.3283E-07	DOWN	0.16588	0.5476	0.71629		-1.1436	6.6404E-05	0.0010174	DOWN
transcript_HQ_CoA_transcript8263/f2p0/3365	9.803333333	4.22	6.156666667	-1.0812	0.00386	0.016101	DOWN	0.4082	0.3008	0.49108		-0.68278	0.0054215	0.032414	
transcript_HQ_CoA_transcript8264/f5p0/3342	14.74333333	40.14	16.19333333	1.6106	2.5101E-06	2.7026E-05	UP	-1.4651	7.4115E-06	0.00010498	DOWN	0.13219	0.49434	0.69626	
transcript_HQ_CoA_transcript8274/f2p0/3328	6.056666667	11.00666667	8.883333333	1.0175	0.0016081	0.0077122	UP	-0.44855	0.15887	0.31752		0.55444	0.072884	0.20567	
transcript_HQ_CoA_transcript8280/f2p0/3329	4.293333333	0.86	2.226666667	-2.0974	0.012692	0.042653	DOWN	1.1773	0.1804	0.34707		-0.93841	0.030918	0.11414	
transcript_HQ_CoA_transcript8295/f4p0/3335	13.44666667	5.77	5.036666667	-1.066	3.8867E-07	4.9208E-06	DOWN	-0.34599	0.084682	0.20207		-1.424	5.9153E-14	1.1193E-11	DOWN
transcript_HQ_CoA_transcript8302/f2p0/3318	6.2	1.593333333	7.343333333	-1.8242	3.7067E-08	5.7771E-07	DOWN	2.0835	3.7165E-07	8.052E-06	UP	0.24714	0.53811	0.73001	
transcript_HQ_CoA_transcript8305/f2p0/3353	1.283333333	15.28666667	1.876666667	3.7228	9.7033E-13	3.1177E-11	UP	-3.1593	2.5645E-06	4.2795E-05	DOWN	0.54653	0.47953	0.68407	
transcript_HQ_CoA_transcript8323/f2p0/3315	14.95	1.82	8.306666667	-2.8663	8.5184E-05	0.00061288	DOWN	2.054	3.288E-11	1.7418E-09	UP	-0.8284	0.21583	0.42099	
transcript_HQ_CoA_transcript8324/f2p0/3323	4.52	1.83	3.563333333	-1.1705	0.0024755	0.011063	DOWN	0.82589	0.023118	0.076666		-0.35482	0.2195	0.42532	
transcript_HQ_CoA_transcript8348/f5p0/3345	10.44	20.83666667	9.616666667	1.1476	0.0010068	0.005182	UP	-1.2705	1.1294E-05	0.00015045	DOWN	-0.13377	0.62573	0.79107	
transcript_HQ_CoA_transcript8349/f3p0/3333	31.78666667	8.243333333	10.36	-1.8139	5.0976E-08	7.7533E-07	DOWN	0.19676	0.52878	0.7009		-1.6281	2.5372E-14	5.1175E-12	DOWN
transcript_HQ_CoA_transcript8352/f7p0/3294	31.20333333	9.51	17.70333333	-1.5448	6.5923E-08	9.7666E-07	DOWN	0.73203	0.011289	0.044108		-0.82437	4.6849E-05	0.00075763	
transcript_HQ_CoA_transcript8353/f8p0/3288	31.88	11.45333333	32.26666667	-1.3068	3.6464E-07	4.6456E-06	DOWN	1.3276	1.8066E-06	3.1507E-05	UP	0.0081099	0.96891	0.98677	
transcript_HQ_CoA_transcript836/f2p0/5893	6.876666667	2.713333333	4.496666667	-1.1846	2.9196E-10	6.5977E-09	DOWN	0.58121	0.0018451	0.010416		-0.61551	0.00065301	0.0063891	
transcript_HQ_CoA_transcript8383/f3p0/3302	12.20666667	4.313333333	3.576666667	-1.3536	0.00045503	0.0026037	DOWN	-0.4246	0.22605	0.40504		-1.7895	3.5794E-05	0.00061002	DOWN
transcript_HQ_CoA_transcript839/f4p0/5888	2.473333333	6.096666667	6.76	1.4779	0.00010136	0.00071083	UP	-0.011889	0.97422	0.98651		1.4522	2.063E-08	1.0819E-06	UP
transcript_HQ_CoA_transcript8391/f3p0/3339	3.656666667	6.836666667	5.096666667	1.0644	0.00004112	0.00032417	UP	-0.56855	0.0052561	0.024371		0.48309	0.085256	0.22962	
transcript_HQ_CoA_transcript8399/f2p0/3322	16.22333333	29.11333333	15.73666667	1.0108	0.00060136	0.0033055	UP	-1.0523	0.00011729	0.0010935	DOWN	-0.053931	0.77098	0.88659	
transcript_HQ_CoA_transcript8403/f4p0/3327	15.26	1.156666667	2.13	-3.6043	1.2901E-10	3.0993E-09	DOWN	0.74098	0.15824	0.31666		-2.8708	2.3387E-08	1.2126E-06	DOWN
transcript_HQ_CoA_transcript8404/f3p0/3308	52.65	16.76333333	10.92	-1.5299	0.011931	0.04061	DOWN	-0.75817	0.030904	0.095233		-2.2963	2.4594E-05	0.0004474	DOWN
transcript_HQ_CoA_transcript8409/f5p0/3296	2.57	5.673333333	3.153333333	1.3042	0.00081271	0.0042954	UP	-1.0003	0.0024642	0.013149	DOWN	0.28964	0.42514	0.63738	
transcript_HQ_CoA_transcript8413/f2p0/3334	4.47	18.26333333	10.96333333	2.2006	3.7646E-12	1.1173E-10	UP	-0.87751	0.072976	0.18124		1.3089	0.0076935	0.04157	UP
transcript_HQ_CoA_transcript843/f3p0/5886	3.45	0.41	1.386666667	-2.8841	0.00022663	0.0014326	DOWN	1.5808	0.050991	0.13859		-1.3141	0.0094372	0.048199	DOWN
transcript_HQ_CoA_transcript8443/f2p0/3301	1.27	2.723333333	1.65	1.2788	0.014028	0.04616	UP	-0.88554	0.045522	0.1269		0.37903	0.38093	0.59683	
transcript_HQ_CoA_transcript8479/f5p0/3294	4.426666667	13.57666667	6.88	1.7689	2.0144E-07	2.7019E-06	UP	-1.1299	4.4253E-11	2.2784E-09	DOWN	0.6289	0.071413	0.20325	
transcript_HQ_CoA_transcript8489/f2p0/3303	1.13	2.996666667	1.37	1.5572	0.00095854	0.0049629	UP	-1.288	0.017369	0.061647		0.25459	0.64023	0.80006	
transcript_HQ_CoA_transcript8493/f2p0/3327	5.596666667	13.51666667	6.413333333	1.429	0.005279	0.020755	UP	-1.2259	0.014425	0.053523		0.18958	0.41772	0.63064	
transcript_HQ_CoA_transcript8496/f4p0/3299	4.55	11.55	3.83	1.486	0.00048051	0.0027299	UP	-1.7459	1.6701E-06	2.9519E-05	DOWN	-0.27007	0.52374	0.71788	
transcript_HQ_CoA_transcript8499/f2p0/3315	4.36	17.82666667	8.993333333	2.2033	5.1131E-11	1.2983E-09	UP	-1.1422	0.0030307	0.015595	DOWN	1.0461	0.0065896	0.037354	UP
transcript_HQ_CoA_transcript8514/f3p0/3343	59.41333333	15.21	20.69333333	-1.8434	0.0029315	0.012739	DOWN	0.30193	0.30658	0.49747		-1.5475	0.0083165	0.044111	DOWN
transcript_HQ_CoA_transcript8517/f2p0/3319	24.60333333	6.926666667	16.86	-1.6757	1.5327E-07	2.1068E-06	DOWN	1.1271	1.5383E-09	5.7909E-08	UP	-0.55894	0.052563	0.16591	
transcript_HQ_CoA_transcript8518/f2p0/3283	1.016666667	7.196666667	5.453333333	2.9358	0.0016803	0.0080187	UP	-0.52605	0.54992	0.71802		2.4025	6.9892E-06	0.00015775	UP
transcript_HQ_CoA_transcript8535/f3p0/3299	11.83	3.133333333	7.656666667	-1.7688	0.0025551	0.01135	DOWN	1.1451	0.025851	0.083498		-0.63627	0.087032	0.23283	
transcript_HQ_CoA_transcript8537/f3p0/3349	2.143333333	11.81666667	5.786666667	2.6338	1.6794E-20	1.0887E-18	UP	-1.1833	4.3264E-06	6.6358E-05	DOWN	1.4373	2.5582E-06	0.00006977	UP
transcript_HQ_CoA_transcript8541/f2p0/3309	5.756666667	17.59333333	5.27	1.7895	5.8689E-08	8.7731E-07	UP	-1.9031	1.1881E-09	4.5845E-08	DOWN	-0.12794	0.64869	0.80633	
transcript_HQ_CoA_transcript8553/f2p0/3359	4.813333333	14.78	9.463333333	1.7814	4.9655E-06	5.0071E-05	UP	-0.80144	0.030024	0.093244		0.96712	8.3979E-06	0.00018394	
transcript_HQ_CoA_transcript8554/f2p0/3281	4.896666667	1.006666667	2.293333333	-2.1118	0.0020388	0.0094005	DOWN	1.0535	0.043219	0.12202		-1.0708	0.10103	0.25895	
transcript_HQ_CoA_transcript856/f3p0/5914	8.413333333	1.573333333	5.563333333	-2.2826	1.5863E-05	0.0001398	DOWN	1.6684	1.2346E-07	2.9853E-06	UP	-0.62188	0.22352	0.43028	
transcript_HQ_CoA_transcript8568/f3p0/3311	16.8	32.47	16.46	1.1144	0.00097924	0.0050572	UP	-1.1356	0.00022522	0.0018858	DOWN	-0.034209	0.86856	0.94146	
transcript_HQ_CoA_transcript8571/f2p0/3310	2.6	0.743333333	1.4	-1.6703	0.010532	0.036737	DOWN	0.80222	0.31263	0.50417		-0.88295	0.11109	0.27528	

transcript_HQ_CoA_transcript8575/f2p0/3299	8.173333333	2.823333333	1.623333333	-1.369	0.00013132	0.00089215	DOWN	-0.95958	0.010944	0.043055		-2.3393	1.0026E-12	1.4722E-10	DOWN
transcript_HQ_CoA_transcript8576/f4p0/3286	13.39666667	2.606666667	7.84	-2.1927	5.7593E-08	8.6347E-07	DOWN	1.4242	0.00026509	0.0021547	UP	-0.78134	0.00035991	0.0039845	
transcript_HQ_CoA_transcript8583/f2p0/3298	12.44666667	2.69	7.453333333	-2.0463	2.4557E-14	9.4916E-13	DOWN	1.3031	0.00014166	0.0012797	UP	-0.75443	0.012461	0.058932	
transcript_HQ_CoA_transcript8587/f3p0/3305	0.773333333	4.393333333	1.25	2.6714	9.2848E-06	8.8133E-05	UP	-1.9604	0.00098385	0.0062986	DOWN	0.69927	0.18117	0.37668	
transcript_HQ_CoA_transcript859/f3p0/5867	3.433333333	9.813333333	6.503333333	1.6467	5.5581E-06	5.5617E-05	UP	-0.74294	0.091232	0.21346		0.89232	0.019332	0.081227	
transcript_HQ_CoA_transcript8606/f6p0/3250	30.18	12.23666667	18.15	-1.1521	2.6443E-05	0.00021974	DOWN	0.4251	0.024812	0.080937		-0.73933	0.0024897	0.018013	
transcript_HQ_CoA_transcript8615/f2p0/3277	40.06	8.816666667	1.826666667	-2.0071	0.00004666	0.00036166	DOWN	-2.4276	1.8545E-12	1.3382E-10	DOWN	-4.4534	5.8451E-18	2.0243E-15	DOWN
transcript_HQ_CoA_transcript8619/f2p0/3280	1.036666667	2.57	5.203333333	1.4232	0.0014179	0.0069329	UP	0.92008	0.25314	0.437		2.3308	0.0035797	0.023772	UP
transcript_HQ_CoA_transcript8625/f2p0/3325	0.566666667	1.383333333	1.416666667	1.4443	0.012652	0.042566	UP	-0.11556	0.74909	0.85833		1.3177	0.026521	0.10182	
transcript_HQ_CoA_transcript8630/f17p0/3228	80.23666667	29.03	37.47333333	-1.3101	8.9372E-07	1.0623E-05	DOWN	0.21094	0.31357	0.50522		-1.11	8.4841E-08	3.7797E-06	DOWN
transcript_HQ_CoA_transcript8661/f2p0/3310	5.693333333	0.94	3.446666667	-2.4765	4.2877E-05	0.00033642	DOWN	1.7511	0.011281	0.044087	UP	-0.73531	0.084956	0.22908	
transcript_HQ_CoA_transcript8663/f2p0/3277	4.326666667	14.41333333	4.96	1.8923	0.0011466	0.0057716	UP	-1.6735	0.0017855	0.010176	DOWN	0.20177	0.66925	0.82031	
transcript_HQ_CoA_transcript8664/f12p0/3263	34.59333333	5.883333333	26.32	-2.3937	9.7274E-08	1.3893E-06	DOWN	1.9999	1.1494E-07	2.7978E-06	UP	-0.40692	0.3937	0.60941	
transcript_HQ_CoA_transcript8669/f2p0/3307	2.233333333	11.38	5.68	2.5582	3.1838E-05	0.00026043	UP	-1.1618	0.14214	0.29336		1.378	0.061515	0.18432	
transcript_HQ_CoA_transcript8678/f3p0/3222	4.656666667	19.34666667	5.05	2.211	9.0217E-18	4.7946E-16	UP	-2.0778	7.7518E-09	2.4753E-07	DOWN	0.12029	0.74799	0.87307	
transcript_HQ_CoA_transcript8681/f2p0/3272	19.42666667	8.483333333	14.17666667	-1.0378	2.7835E-05	0.00023016	DOWN	0.58853	0.00041569	0.0031329		-0.46123	0.039738	0.13659	
transcript_HQ_CoA_transcript8682/f3p0/3271	3.553333333	14.10333333	4.266666667	2.1255	5.7385E-05	0.00043405	UP	-1.8633	1.2492E-05	0.00016449	DOWN	0.25141	0.66054	0.81429	
transcript_HQ_CoA_transcript8693/f2p0/3285	25.53666667	6.71	14.02333333	-1.7856	1.1233E-09	2.3113E-08	DOWN	0.91792	0.00014679	0.0013241		-0.87817	0.00048562	0.0050502	
transcript_HQ_CoA_transcript8698/f2p0/3290	4.403333333	1.523333333	1.906666667	-1.3791	1.3458E-05	0.00012138	DOWN	0.19407	0.65039	0.79265		-1.1976	0.0014323	0.011864	DOWN
transcript_HQ_CoA_transcript8704/f3p0/3261	3.28	11	6.023333333	1.9078	1.7742E-06	1.9814E-05	UP	-1.0159	0.0015391	0.0089984	DOWN	0.87824	0.0067273	0.037873	
transcript_HQ_CoA_transcript8706/f2p0/3278	6.803333333	0.14	0.573333333	-5.4311	6.5462E-11	1.6239E-09	DOWN	1.8756	0.015389	0.056179		-3.5705	7.3449E-07	2.4204E-05	DOWN
transcript_HQ_CoA_transcript8708/f2p0/3304	9.456666667	3.15	10.88333333	-1.397	0.011215	0.038657	DOWN	1.6165	0.00015053	0.0013492	UP	0.20566	0.62504	0.7905	
transcript_HQ_CoA_transcript8724/f2p0/3269	7.25	17.31666667	9.01	1.412	4.3821E-09	8.0792E-08	UP	-1.0869	2.8277E-08	7.9858E-07	DOWN	0.31152	0.18963	0.38726	
transcript_HQ_CoA_transcript8732/f8p0/3233	5.086666667	14.38666667	18.72666667	1.6675	0.00037612	0.0022167	UP	0.23318	0.49517	0.67444		1.8856	3.3047E-06	8.5553E-05	UP
transcript_HQ_CoA_transcript8741/f2p0/3287	1.683333333	29.09333333	4.38	4.2823	2.532E-37	4.8218E-35	UP	-2.8968	3.3341E-21	9.5486E-19	DOWN	1.3711	0.00003265	0.0005713	UP
transcript_HQ_CoA_transcript8743/f4p0/3255	4.313333333	1.8	2.596666667	-1.0979	0.0057295	0.022159	DOWN	0.35961	0.24965	0.43294		-0.74935	0.028654	0.10802	
transcript_HQ_CoA_transcript8776/f3p0/3257	3.156666667	9.543333333	7.316666667	1.7674	0.00067079	0.003625	UP	-0.51435	0.34738	0.53945		1.2368	0.048707	0.1574	
transcript_HQ_CoA_transcript8777/f2p0/3260	5.07	19.88666667	4.026666667	2.1441	1.3293E-14	5.3531E-13	UP	-2.4436	5.6604E-10	2.3314E-08	DOWN	-0.31412	0.5002	0.70085	
transcript_HQ_CoA_transcript8778/f8p0/3208	3.466666667	19.39	4.213333333	2.6057	0.00014878	0.00099381	UP	-2.3039	0.00136	0.0081727	DOWN	0.29032	0.59624	0.77034	
transcript_HQ_CoA_transcript8783/f2p0/3261	26.41	7.356666667	11	-1.7036	0.0002078	0.0013315	DOWN	0.42676	0.2621	0.44746		-1.2855	0.0059153	0.034733	DOWN
transcript_HQ_CoA_transcript8784/f3p0/3212	0.516666667	2.576666667	1.916666667	2.4506	0.0014329	0.0069818	UP	-0.5651	0.39705	0.58761		1.8755	0.00025943	0.0030782	UP
transcript_HQ_CoA_transcript8790/f3p0/3258	11.44666667	31.11333333	14.33	1.5983	1.1736E-07	1.6529E-06	UP	-1.2732	4.8542E-09	1.6325E-07	DOWN	0.31377	0.2501	0.46039	
transcript_HQ_CoA_transcript8815/f26p0/3198	31.01333333	62.15	33.5	1.1627	5.9091E-14	2.178E-12	UP	-1.0324	9.3516E-05	0.00091125	DOWN	0.11668	0.67007	0.82095	
transcript_HQ_CoA_transcript8816/f19p0/3222	66.23666667	18.29666667	35.57666667	-1.6901	4.9059E-07	6.0724E-06	DOWN	0.78944	0.015188	0.055642		-0.91278	6.9446E-06	0.00015737	
transcript_HQ_CoA_transcript882/f2p0/5920	16.28333333	5.303333333	9.386666667	-1.4712	1.3886E-05	0.00012487	DOWN	0.66905	0.0004769	0.0035161		-0.81171	0.010359	0.051659	
transcript_HQ_CoA_transcript8821/f4p0/3241	4.643333333	15.28	8.043333333	1.9246	0.0021563	0.0098627	UP	-1.1195	0.0747	0.18467		0.78809	0.035609	0.12613	
transcript_HQ_CoA_transcript8826/f10p0/3239	20.33	8.69	8.31	-1.0716	8.0723E-10	1.7041E-08	DOWN	-0.21372	0.18847	0.35731		-1.2972	1.6402E-14	3.4605E-12	DOWN
transcript_HQ_CoA_transcript8828/f3p0/3291	0.116666667	18.43333333	1.09	7.3928	3.6689E-05	0.00029367	UP	-4.2194	4.6914E-08	1.2608E-06	DOWN	3.1645	0.061257	0.18398	
transcript_HQ_CoA_transcript8829/f2p0/3246	3.16	5.663333333	3.203333333	1.0016	9.6541E-05	0.00068172	UP	-0.97727	9.8847E-05	0.0009576		0.011424	0.96984	0.98741	
transcript_HQ_CoA_transcript8838/f3p0/3250	5.466666667	11.92	6.93	1.2786	8.1999E-06	7.8818E-05	UP	-0.92862	0.00029559	0.0023556		0.33756	0.19053	0.3884	
transcript_HQ_CoA_transcript884/f5p0/5863	4.723333333	1.236666667	0.97	-1.7709	0.00094694	0.0049098	DOWN	-0.50619	0.13053	0.27601		-2.2942	1.8389E-05	0.00035176	DOWN
transcript_HQ_CoA_transcript8845/f2p0/3258	0.453333333	13.34	0.74	5.047	6.2805E-08	9.3367E-07	UP	-4.3125	2.6808E-14	2.7298E-12	DOWN	0.73264	0.59034	0.76659	
transcript_HQ_CoA_transcript8867/f2p0/3253	1.81	0.576666667	0.886666667	-1.5062	0.0052554	0.020689	DOWN	0.48662	0.71322	0.83574		-1.0342	0.37542	0.59208	
transcript_HQ_CoA_transcript8878/f2p0/3289	26.35666667	11.33333333	9.536666667	-1.0712	0.00084755	0.00445	DOWN	-0.38783	0.13105	0.27666		-1.4713	2.9154E-08	1.4782E-06	DOWN
transcript_HQ_CoA_transcript8884/f2p0/3246	6.973333333	2.676666667	6.01	-1.2259	0.00026744	0.0016484	DOWN	1.0177	0.00032553	0.0025461	UP	-0.22167	0.45647	0.66554	
transcript_HQ_CoA_transcript8890/f2p0/3329	9.893333333	20.57	11.78	1.2055	1.2172E-08	2.07E-07	UP	-0.95481	1.3273E-09	5.0368E-08		0.2388	0.27784	0.49172	
transcript_HQ_CoA_transcript8905/f3p0/3280	10.77666667	3.306666667	15.48333333	-1.5325	0.013476	0.044706	DOWN	2.0861	3.7124E-17	5.7179E-15	UP	0.5399	0.34639	0.5642	
transcript_HQ_CoA_transcript8912/f2p0/3245	0.63	3.543333333	0.813333333	2.7007	1.0568E-05	9.8222E-05	UP	-2.3003	1.8341E-06	3.1895E-05	DOWN	0.37798	0.64448	0.80298	
transcript_HQ_CoA_transcript8913/f2p0/3250	2.253333333	10.34	4.933333333	2.3738	1.1366E-08	1.9438E-07	UP	-1.2224	0.056164	0.14973		1.1355	0.075262	0.2102	
transcript_HQ_CoA_transcript8939/f2p0/3245	1.89	4.653333333	3.546666667	1.4724	0.00010048	0.00070622	UP	-0.55801	0.15039	0.30532		0.90174	0.037672	0.13123	
transcript_HQ_CoA_transcript8940/f2p0/3279	6.54	14.57	7.726666667	1.313	4.7397E-05	0.00036654	UP	-1.0724	0.00059641	0.0042028	DOWN	0.2285	0.48772	0.69059	
transcript_HQ_CoA_transcript8945/f3p0/3257	18.97	8.116666667	28.84333333	-1.0896	0.0041338	0.016972	DOWN	1.6796	3.2187E-05	0.00037245	UP	0.58153	0.13853	0.31676	
transcript_HQ_CoA_transcript895/f2p0/5870	12.43333333	5.526666667	8.083333333	-1.0147	4.4342E-07	5.5525E-06	DOWN	0.39482	0.0076223	0.032506		-0.63152	0.00048351	0.0050396	
transcript_HQ_CoA_transcript8957/f7p0/3188	31.81666667	6.15	8.74	-2.2183	8.8379E-08	1.2722E-06	DOWN	0.35411	0.29024	0.47982		-1.8733	6.1619E-07	2.0753E-05	DOWN
transcript_HQ_CoA_transcript8982/f2p0/3111	7.413333333	1.44	3.62	-2.2126	1.7042E-05	0.00014879	DOWN	1.1612	0.069212	0.17447		-1.0581	0.066011	0.19327	
transcript_HQ_CoA_transcript8989/f2p0/3287	9.043333333	1.636666667	4.32	-2.3425	0.00055401	0.0030767	DOWN	1.2756	0.035759	0.10596		-1.0747	0.02618	0.10093	
transcript_HQ_CoA_transcript8995/f2p0/3264	11.23666667	3.953333333	5.96	-1.3667	1.9166E-05	0.00016513	DOWN	0.44938	0.061661	0.16035		-0.92739	0.0013743	0.011508	
transcript_HQ_CoA_transcript9012/f2p0/3232	36.79333333	9.14	7.97	-1.8538	4.718E-23	3.579E-21	DOWN	-0.34026	0.13195	0.27797		-2.206	6.6967E-24	5.3442E-21	DOWN
transcript_HQ_CoA_transcript9028/f2p0/3240	2.576666667	9.496666667	4.816666667	2.042	0.00013062	0.00088838	UP	-1.1479	0.0012136	0.0074362	DOWN	0.884	0.070667	0.20184	
transcript_HQ_CoA_transcript9037/f4p0/3212	3.11	10.97666667	2.55	1.9756	5.639E-08	8.485E-07	UP	-2.2633	1.4578E-11	8.2471E-10	DOWN	-0.30013	0.39779	0.61345	
transcript_HQ_CoA_transcript9038/f2p0/3234	164.9333333	71.62333333	142.99	-1.0513	1.3597E-09	2.7437E-08	DOWN	0.84369	0.00075192	0.0050706		-0.2194	0.34619	0.56407	
transcript_HQ_CoA_transcript9040/f2p0/3235	8.716666667	2.16	8.516666667	-1.8262	0.0010153	0.0052143	DOWN	1.7983	0.0018248	0.010331	UP	-0.039597	0.89623	0.95376	
transcript_HQ_CoA_transcript9041/f2p0/3231	12.87666667	42.5	22.21	1.8781	7.0856E-37	1.2954E-34	UP	-1.075	3.1806E-05	0.0003685	DOWN	0.79011	0.0028741	0.020095	
transcript_HQ_CoA_transcript9050/f2p0/3249	1.853333333	13.34333333	2.116666667	2.9912	0.010713	0.037249	UP	-2.7478	0.016348	0.058857		0.22793	0.7744	0.88922	
transcript_HQ_CoA_transcript9061/f5p0/3153	5.17	26.19666667	16.52666667	2.5071	8.7967E-10	1.8464E-08	UP	-0.82142	0.012376	0.047457		1.6729	8.7569E-08	3.8638E-06	UP
transcript_HQ_CoA_transcript9071/f5p0/3217	39.59666667	77.11666667	44.90333333	1.1395	0.00031075	0.0018774	UP	-0.94184	0.00098495	0.0063035		0.18256	0.52606	0.7202	
transcript_HQ_CoA_transcript9074/f2p0/3229	7.783333333	1.746666667	3.906666667	-2.039	0.0010408	0.0053284	DOWN	1.0398	0.074989	0.18524		-1.0081	0.0064329	0.036784	DOWN
transcript_HQ_CoA_transcript908/f2p0/5852	0.193333333	1.503333333	0.723333333	3.0721	0.014362	0.046996	UP	-1.1593	0.17285	0.3365		1.8991	0.080291	0.22016	
transcript_HQ_CoA_transcript9084/f3p0/3245	1.073333333	2.406666667	3.79	1.3436	0.011796	0.040242	UP	0.49019	0.28748	0.47668		1.8214	2.0807E-05	0.00038783	UP
transcript_HQ_CoA_transcript9085/f11p0/3199	11.72	21.31666667	34.72666667	1.0216	5.2544E-06	5.2781E-05	UP	0.57407	0.1762	0.34117		1.5818	0.00010863	0.0015256	UP
transcript_HQ_CoA_transcript9093/f3p0/3220	10.42333333	1.406666667	6.21	-2.7506	2.5373E-06	0.00002727	DOWN	1.9938	1.7069E-12	1.2536E-10	UP	-0.76558	0.13936	0.31778	
transcript_HQ_CoA_transcript9096/f3p0/3260	2.323333333	0.516666667	1.32	-2.071	0.0037706	0.015778	DOWN	1.2341	0.04188	0.11916		-0.8431	0.13054	0.30442	
transcript_HQ_CoA_transcript9102/f5p0/3228	21.32666667	0.893333333	26.98666667	-4.415	6.0221E-39	1.1967E-36	DOWN	4.7621	1.9941E-49	7.31E-46	UP	0.33428	0.18862	0.38592	
transcript_HQ_CoA_transcript9103/f3p0/3235	14.74666667	30.12	16.46666667	1.1937	0.00020493	0.0013141	UP	-1.0232	0.00050618	0.0036883	DOWN	0.15788	0.46385	0.6715	
transcript_HQ_CoA_transcript9106/f12p0/3199	9.416666667	22.06333333	15.26	1.3737	1.4975E-07	2.0631E-06	UP	-0.6671	0.0066994	0.029411		0.69449	0.0005132	0.0052712	
transcript_HQ_CoA_transcript9121/f3p0/3246	1.8	8.36	10.25	2.362	9.4601E-06	8.9611E-05	UP	0.13944	0.7022	0.82886		2.49	7.2868E-06	0.00016331	UP
transcript_HQ_CoA_transcript9129/f2p0/3227	34.64666667	8.43	15.22666667	-1.8911	5.943E-10	1.2782E-08	DOWN	0.69767	0.0004053	0.0030672		-1.2038	5.0724E-05	0.00081031	DOWN
transcript_HQ_CoA_transcript9134/f2p0/3224	3.976666667	8.583333333	6.503333333	1.2598	2.1803E-06	2.3825E-05	UP	-0.54467	0.007486	0.032064		0.70356	0.010877	0.053494	
transcript_HQ_CoA_transcript9147/f7p0/3184	7.38	50.95	11.27333333	2.9576	3.7266E-09	6.9733E-08	UP	-2.3318	5.0094E-06	7.4903E-05	DOWN	0.61104	0.021592	0.087781	

transcript_HQ_CoA_transcript9150/f5p0/3190	4.553333333	21.54333333	2.33	2.3822	0.01405	0.046207	UP	-3.3156	0.0003489	0.002696	DOWN	-0.94739	0.087447	0.23349	
transcript_HQ_CoA_transcript9159/f2p0/3256	4.853333333	12.46666667	1.943333333	1.5008	0.00000585	5.8256E-05	UP	-2.8091	2.5385E-20	6.2877E-18	DOWN	-1.319	9.6328E-05	0.0013781	DOWN
transcript_HQ_CoA_transcript9174/f2p0/3215	7.31	2.603333333	3.456666667	-1.3128	0.004501	0.018164	DOWN	0.25314	0.32594	0.51686		-1.0729	0.013762	0.063137	
transcript_HQ_CoA_transcript9180/f2p0/3214	33.36666667	11.67333333	13.83	-1.3748	0.0015952	0.0076664	DOWN	0.09148	0.71207	0.83535		-1.2931	0.00081137	0.0075789	DOWN
transcript_HQ_CoA_transcript9206/f2p0/3203	13.06	25.98666667	16.57333333	1.1776	0.013194	0.043943	UP	-0.8228	0.087793	0.20761		0.34078	0.1222	0.29229	
transcript_HQ_CoA_transcript9230/f4p0/3223	19.96666667	8.706666667	18.19	-1.0472	0.00065534	0.0035596	DOWN	0.9026	0.00009347	0.00091125		-0.1552	0.5963	0.77034	
transcript_HQ_CoA_transcript9239/f8p0/3191	30.45666667	9.186666667	18.72	-1.5926	2.4824E-05	0.00020751	DOWN	0.89201	0.047525	0.13133		-0.7134	0.029481	0.11037	
transcript_HQ_CoA_transcript9246/f4p0/3192	45.93	14.13	30.66333333	-1.5514	3.9217E-09	7.3159E-08	DOWN	0.96781	2.5972E-12	1.7896E-10		-0.59492	0.015617	0.069387	
transcript_HQ_CoA_transcript9249/f12p0/3181	39.69	106.7166667	51.9	1.5849	2.3623E-12	7.2101E-11	UP	-1.1901	7.9809E-07	1.5797E-05	DOWN	0.38237	0.05336	0.16788	
transcript_HQ_CoA_transcript9258/f3p0/3206	12.80333333	3.65	4.63	-1.6829	0.00041946	0.0024313	DOWN	0.21035	0.5934	0.75304		-1.4803	0.0012051	0.010385	DOWN
transcript_HQ_CoA_transcript9261/f7p0/3162	90.46666667	23.85666667	66.37333333	-1.7707	3.0429E-20	1.9316E-18	DOWN	1.3238	1.4919E-10	6.9935E-09	UP	-0.45782	0.051138	0.16253	
transcript_HQ_CoA_transcript9266/f3p0/3218	16.77666667	34.95	21.60333333	1.2223	0.00003304	0.00026834	UP	-0.85224	0.0012875	0.0078165		0.35703	0.18381	0.38009	
transcript_HQ_CoA_transcript9271/f3p0/3185	27.53333333	3.016666667	3.546666667	-3.0135	1.1244E-05	0.00010379	DOWN	0.049246	0.9414	0.96905		-2.9728	1.298E-10	1.14E-08	DOWN
transcript_HQ_CoA_transcript9272/f2p0/3227	6.526666667	17.75	8.81	1.6082	1.3714E-06	1.5719E-05	UP	-1.1569	0.0017925	0.010206	DOWN	0.43789	0.29827	0.51402	
transcript_HQ_CoA_transcript9283/f3p0/3229	36.90666667	13.77333333	23.74	-1.2581	7.3585E-14	2.6798E-12	DOWN	0.63288	0.00049083	0.0035999		-0.63876	0.000371	0.0040851	
transcript_HQ_CoA_transcript9289/f2p0/3200	0	18.61333333	1.46	11.795	1.0441E-22	7.7282E-21	UP	-3.8141	0.0154	0.056196		7.9638	0.041594	0.14099	
transcript_HQ_CoA_transcript9292/f2p0/3208	2.233333333	0.683333333	0.923333333	-1.5423	0.00086453	0.0045287	DOWN	0.27132	0.58844	0.74928		-1.2827	0.0030851	0.021241	DOWN
transcript_HQ_CoA_transcript931/f2p0/5817	5.47	18.02	7.443333333	1.8639	0.0027049	0.01193	UP	-1.4029	0.010644	0.042112	DOWN	0.44756	0.27179	0.4844	
transcript_HQ_CoA_transcript9315/f2p0/3218	8.263333333	3.496666667	2.613333333	-1.0822	0.00017824	0.0011652	DOWN	-0.55593	0.10241	0.23254		-1.6503	5.6587E-07	1.9357E-05	DOWN
transcript_HQ_CoA_transcript9316/f2p0/3198	12.35666667	4.65	7.67	-1.2787	0.00087254	0.0045668	DOWN	0.58114	0.014809	0.054537		-0.70695	0.035024	0.12459	
transcript_HQ_CoA_transcript9326/f4p0/3196	6.84	14.91	5.703333333	1.2714	0.0043058	0.017513	UP	-1.5243	2.4884E-06	0.00004173	DOWN	-0.26674	0.49805	0.69911	
transcript_HQ_CoA_transcript9335/f2p0/3208	1.52	5.636666667	2.29	2.0445	1.0321E-08	1.7801E-07	UP	-1.447	7.0983E-06	0.00010125	DOWN	0.58653	0.16659	0.35652	
transcript_HQ_CoA_transcript9337/f2p0/3176	13.38666667	5.55	7.886666667	-1.1178	3.0651E-08	4.8643E-07	DOWN	0.361	0.071639	0.17915		-0.76939	7.4621E-05	0.0011145	
transcript_HQ_CoA_transcript9341/f2p0/3205	5.016666667	12.91	6.816666667	1.4987	0.0017524	0.0082997	UP	-1.0653	2.1996E-09	7.9238E-08	DOWN	0.42301	0.37104	0.58823	
transcript_HQ_CoA_transcript9352/f2p0/3201	8.203333333	17.57666667	6.076666667	1.2669	0.0016462	0.0078804	UP	-1.6891	2.4958E-05	0.00029533	DOWN	-0.43647	0.055933	0.17313	
transcript_HQ_CoA_transcript9353/f5p0/3020	118.4733333	22.85333333	57.78	-2.2076	2.1413E-06	2.3455E-05	DOWN	1.2014	0.00076606	0.0051508	UP	-1.0197	0.031397	0.11535	
transcript_HQ_CoA_transcript9360/f5p0/3205	2.83	24.27	4.01	3.2642	6.8984E-17	3.4458E-15	UP	-2.7663	8.3206E-13	6.5454E-11	DOWN	0.48527	0.10223	0.26082	
transcript_HQ_CoA_transcript9374/f2p0/3264	6.086666667	2.626666667	3.073333333	-1.0553	4.9295E-05	0.00037961	DOWN	0.079923	0.80439	0.89176		-0.9873	0.0015124	0.0123	
transcript_HQ_CoA_transcript9375/f2p0/3195	8.656666667	0.996666667	3.88	-2.9224	0.00061788	0.0033841	DOWN	1.7991	0.0027175	0.014235	UP	-1.1406	0.17909	0.37363	
transcript_HQ_CoA_transcript9381/f5p0/3183	8.946666667	22.63	15.68333333	1.4938	2.3962E-09	4.6356E-08	UP	-0.66834	0.081009	0.19576		0.81259	0.028479	0.10756	
transcript_HQ_CoA_transcript9382/f3p0/3196	2.73	12.58666667	1.54	2.3608	2.7979E-06	2.9721E-05	UP	-3.1678	2.069E-09	7.5245E-08	DOWN	-0.81762	0.038616	0.13389	
transcript_HQ_CoA_transcript9383/f3p0/3191	0.403333333	34.49666667	3.906666667	6.6099	1.2783E-25	1.1685E-23	UP	-3.3192	0.00021517	0.0018267	DOWN	3.2736	0.00033593	0.0037576	UP
transcript_HQ_CoA_transcript9384/f2p0/3205	0.836666667	14.85666667	5.856666667	4.3377	1.0401E-13	3.7066E-12	UP	-1.501	0.0010322	0.0065602	DOWN	2.8217	1.1233E-09	7.8396E-08	UP
transcript_HQ_CoA_transcript9395/f3p0/3220	26.85	7.54	25.94	-1.6718	1.0334E-13	3.6901E-12	DOWN	1.6233	2.2776E-13	1.9691E-11	UP	-0.06065	0.68977	0.83497	
transcript_HQ_CoA_transcript9399/f2p0/3198	0.826666667	5.346666667	0.196666667	2.8365	2.2565E-05	0.00019116	UP	-4.9365	0.021972	0.073815		-2.1158	0.19159	0.38978	
transcript_HQ_CoA_transcript9407/f4p0/2949	12.30333333	4.56	5.176666667	-1.2894	0.00013633	0.00092342	DOWN	0.036727	0.86609	0.92742		-1.2633	0.00010622	0.0014975	DOWN
transcript_HQ_CoA_transcript9426/f2p0/3194	1.396666667	16.40666667	2.503333333	3.7203	1.4582E-12	4.557E-11	UP	-2.8683	5.894E-08	1.5543E-06	DOWN	0.83878	0.070686	0.20184	
transcript_HQ_CoA_transcript943/f2p0/5833	0.59	4.926666667	4.433333333	3.1719	0.0005391	0.0030094	UP	-0.28513	0.75195	0.86005		2.8792	1.8288E-10	1.5258E-08	UP
transcript_HQ_CoA_transcript9439/f4p0/3172	0.693333333	4.893333333	0.593333333	2.9997	1.2575E-05	0.00011432	UP	-3.2401	1.2961E-05	0.00016909	DOWN	-0.25564	0.74965	0.87392	
transcript_HQ_CoA_transcript9448/f3p0/3148	9.203333333	2.673333333	7.39	-1.6368	4.0841E-06	4.1891E-05	DOWN	1.3249	2.4998E-05	0.00029561	UP	-0.32317	0.19463	0.39359	
transcript_HQ_CoA_transcript9449/f2p0/3190	26.50666667	66.84333333	8.03	1.4723	7.2192E-05	0.00053026	UP	-3.1897	2.6462E-19	5.6398E-17	DOWN	-1.728	1.4673E-08	7.9918E-07	DOWN
transcript_HQ_CoA_transcript9459/f2p0/3185	3.62	1.2	2.273333333	-1.4165	0.001998	0.0092617	DOWN	0.74386	0.07295	0.18123		-0.68335	0.041879	0.14162	
transcript_HQ_CoA_transcript9469/f2p0/3181	9.496666667	4.073333333	7.016666667	-1.0749	0.0020186	0.0093332	DOWN	0.63609	0.028205	0.089241		-0.45077	0.10356	0.26302	
transcript_HQ_CoA_transcript9475/f2p0/3167	15.30333333	27.38	18.25333333	1.0152	0.0062108	0.02372	UP	-0.75944	0.034487	0.10335		0.24341	0.15629	0.34228	
transcript_HQ_CoA_transcript9478/f2p0/3183	8.776666667	1.83	1.016666667	-2.1209	1.2401E-06	1.4322E-05	DOWN	-1.0143	0.15581	0.31344		-3.1507	5.8056E-07	0.00001977	DOWN
transcript_HQ_CoA_transcript9518/f2p0/3169	8.196666667	19.03	14.79666667	1.3825	6.8914E-07	8.3325E-06	UP	-0.51405	0.044936	0.12563		0.85488	0.0046942	0.029197	
transcript_HQ_CoA_transcript9526/f2p0/3145	6.156666667	2.43	4.586666667	-1.1882	1.9375E-05	0.00016685	DOWN	0.75812	0.0091101	0.037292		-0.44129	0.12187	0.29173	
transcript_HQ_CoA_transcript9530/f2p0/3153	5.276666667	15.78666667	11.52666667	1.7258	0.00093561	0.0048572	UP	-0.58588	0.20959	0.38516		1.126	0.0024603	0.017842	UP
transcript_HQ_CoA_transcript9538/f2p0/3185	2.993333333	38.29666667	10.71666667	3.8548	1.2915E-20	8.4631E-19	UP	-2.0017	5.3204E-08	1.4174E-06	DOWN	1.8391	2.3813E-09	1.5499E-07	UP
transcript_HQ_CoA_transcript9572/f2p0/3178	1.396666667	0.14	5.616666667	-3.113	0.0064882	0.02462	DOWN	5.1263	2.4349E-11	1.3243E-09	UP	2.0001	0.0014788	0.012129	UP
transcript_HQ_CoA_transcript9575/f7p0/3121	276.64	70.10666667	85.56333333	-1.8534	0.00099437	0.0051281	DOWN	0.14645	0.72673	0.84426		-1.7135	0.00071377	0.0068593	DOWN
transcript_HQ_CoA_transcript959/f2p0/5796	8.97	1.05	4.753333333	-2.9544	3.5432E-09	6.6643E-08	DOWN	2.0417	0.00020469	0.0017589	UP	-0.92333	0.0030519	0.021067	
transcript_HQ_CoA_transcript9606/f2p0/3168	1.556666667	4.59	1.836666667	1.7251	0.0033504	0.014271	UP	-1.4728	0.0074458	0.031946	DOWN	0.2391	0.56765	0.7498	
transcript_HQ_CoA_transcript963/f2p0/5767	22.00333333	6.516666667	15.03333333	-1.6145	0.0011257	0.0056837	DOWN	1.0527	1.754E-07	4.111E-06	UP	-0.56964	0.21669	0.42182	
transcript_HQ_CoA_transcript9649/f2p0/3155	22.64	5.373333333	10.08	-1.8936	0.0060092	0.02307	DOWN	0.72143	0.28161	0.47039		-1.1824	0.00038881	0.004225	DOWN
transcript_HQ_CoA_transcript9653/f2p0/3186	7.416666667	18.01666667	7.516666667	1.4294	0.00005717	0.00043315	UP	-1.4042	5.3542E-05	0.00057023	DOWN	0.01296	0.96206	0.98325	
transcript_HQ_CoA_transcript9667/f7p0/3091	43.46666667	10.76666667	16.47333333	-1.8815	9.7162E-06	9.1328E-05	DOWN	0.46616	0.18813	0.35693		-1.4235	0.00049614	0.0051334	DOWN
transcript_HQ_CoA_transcript9668/f2p0/3160	7.516666667	21.85333333	17.94333333	1.6767	1.5207E-06	1.7236E-05	UP	-0.42278	0.11044	0.24515		1.2434	7.7347E-05	0.0011496	UP
transcript_HQ_CoA_transcript9673/f2p0/3145	12.07	4.04	8.826666667	-1.4193	4.828E-06	4.8765E-05	DOWN	0.96414	0.0006698	0.0046258		-0.46694	0.04607	0.15152	
transcript_HQ_CoA_transcript9689/f2p0/3203	10.67	3.396666667	7.483333333	-1.4846	3.7041E-06	0.00003839	DOWN	0.97582	0.0013148	0.0079536		-0.5201	0.01866	0.079163	
transcript_HQ_CoA_transcript9691/f2p0/3155	8.916666667	1.763333333	23.55666667	-2.1382	0.00050416	0.0028413	DOWN	3.5714	2.5359E-26	1.3671E-23	UP	1.4169	0.0078021	0.042033	UP
transcript_HQ_CoA_transcript9699/f2p0/3158	5.6	1.876666667	3.27	-1.4197	0.012503	0.042161	DOWN	0.63453	0.20637	0.38077		-0.79461	0.14163	0.32113	
transcript_HQ_CoA_transcript9701/f3p0/3152	1.3	16.51666667	2.75	3.8353	2.8187E-12	8.4895E-11	UP	-2.7307	3.0293E-07	6.7058E-06	DOWN	1.0894	0.0098872	0.04983	UP
transcript_HQ_CoA_transcript9704/f2p0/3179	3.83	7.856666667	6.763333333	1.199	0.00070228	0.0037747	UP	-0.38131	0.16671	0.32807		0.80599	0.0055796	0.03309	
transcript_HQ_CoA_transcript9713/f2p0/3147	3.28	19.46333333	1.913333333	2.7133	2.2117E-05	0.00018772	UP	-3.4561	9.9973E-07	1.9068E-05	DOWN	-0.75943	0.19254	0.39111	
transcript_HQ_CoA_transcript9716/f2p0/3157	2.923333333	0.193333333	3.563333333	-3.7043	0.00053796	0.0030049	DOWN	3.9973	7.9635E-05	0.00079457	UP	0.27954	0.43695	0.64825	
transcript_HQ_CoA_transcript9722/f2p0/3154	22.54666667	1.406666667	1.633333333	-3.8135	2.8361E-05	0.0002344	DOWN	0.08428	0.90321	0.94837		-3.743	0.0021234	0.016	DOWN
transcript_HQ_CoA_transcript9729/f2p0/3143	16.81666667	34.01666667	20.61333333	1.1875	0.00068229	0.0036774	UP	-0.88635	0.0051238	0.023903		0.28771	0.18874	0.38604	
transcript_HQ_CoA_transcript9736/f2p0/3155	11.9	4.613333333	10.50666667	-1.1993	0.0082131	0.029964	DOWN	1.0158	0.0057982	0.026364	UP	-0.19498	0.55885	0.74418	
transcript_HQ_CoA_transcript9738/f3p0/3140	4.783333333	2.043333333	3.28	-1.0647	0.0019036	0.0088938	DOWN	0.53335	0.058466	0.15419		-0.54351	0.10136	0.25951	
transcript_HQ_CoA_transcript9740/f2p0/3143	14.68333333	5.16	5.946666667	-1.3801	0.00057128	0.0031639	DOWN	0.066237	0.83225	0.90859		-1.3237	5.1952E-05	0.00082561	DOWN
transcript_HQ_CoA_transcript9753/f2p0/3229	0.3	9.696666667	1.12	5.2728	3.1424E-18	1.7252E-16	UP	-3.2887	7.6406E-12	4.6067E-10	DOWN	1.9692	0.010128	0.050795	
transcript_HQ_CoA_transcript9758/f2p0/3164	1.923333333	3.473333333	2.836666667	1.012	0.012004	0.040814	UP	-0.42976	0.23319	0.41368		0.56857	0.11973	0.2883	
transcript_HQ_CoA_transcript9761/f3p0/3139	47.67333333	15.08	13.28	-1.4789	0.00022838	0.0014417	DOWN	-0.35486	0.34322	0.53558		-1.8482	6.6603E-13	1.002E-10	DOWN
transcript_HQ_CoA_transcript9762/f2p0/3162	5.013333333	13.04333333	9.856666667	1.5523	2.1341E-07	2.8499E-06	UP	-0.57388	0.093473	0.21736		0.96636	0.0014636	0.012047	
transcript_HQ_CoA_transcript9764/f4p0/3110	7.566666667	15.85333333	9.91	1.2364	0.00082441	0.0043485	UP	-0.83344	0.0011455	0.0071282		0.38792	0.23658	0.44514	
transcript_HQ_CoA_transcript977/f3p0/5742	6.063333333	1.643333333	3.596666667	-1.7147	0.00052803	0.0029575	DOWN	0.95508	0.025331	0.082218		-0.77096	0.025204	0.097992	
transcript_HQ_CoA_transcript9774/f2p0/3138	16.95	2.12	32.24333333	-2.8149	2.0768E-09	4.0607E-08	DOWN	3.7633	3.9676E-15	4.632E-13	UP	0.93563	0.015874	0.070191	
transcript_HQ_CoA_transcript9777/f2p0/3168	13.68	30.76666667	37.48666667	1.3431	0.00012023	0.00082635	UP	0.13038	0.76306	0.86692		1.4597	0.00003147	0.00055329	UP

transcript_HQ_CoA_transcript9780/f2p0/3158	14.13	3.54	6.746666667	-1.8544	2.3464E-09	4.5442E-08	DOWN	0.78391	0.00022927	0.0019093		-1.0816	0.00012611	0.0017223	DOWN
transcript_HQ_CoA_transcript9783/f3p0/2998	29.51	4.673333333	18.38666667	-2.4789	3.8211E-11	9.9087E-10	DOWN	1.8114	9.3269E-10	3.6606E-08	UP	-0.6812	0.01581	0.070042	
transcript_HQ_CoA_transcript9790/f2p0/3145	0.116666667	4.323333333	0.143333333	5.3144	1.316E-07	1.838E-06	UP	-5.0833	0.00029418	0.0023453	DOWN	0.19818	0.9402	0.97355	
transcript_HQ_CoA_transcript9796/f2p0/3143	28.61666667	8.913333333	12.71333333	-1.5427	2.1874E-09	4.2588E-08	DOWN	0.36739	0.15506	0.31232		-1.1859	3.5604E-07	1.2992E-05	DOWN
transcript_HQ_CoA_transcript9799/f2p0/3138	0.206666667	26.67	5.323333333	7.187	1.9502E-27	2.1097E-25	UP	-2.4656	0.0054776	0.025156	DOWN	4.7036	8.3931E-05	0.0012295	UP
transcript_HQ_CoA_transcript980/f2p0/5774	11.53666667	4.196666667	6.466666667	-1.3205	6.4337E-05	0.00047989	DOWN	0.48417	0.068766	0.17371		-0.84724	0.00076856	0.0072679	
transcript_HQ_CoA_transcript9857/f3p0/3123	0.88	13.88666667	1.586666667	4.1518	1.7673E-07	2.3969E-06	UP	-3.2757	1.0813E-17	1.8017E-15	DOWN	0.85926	0.34618	0.56407	
transcript_HQ_CoA_transcript9859/f3p0/3143	0.526666667	5.643333333	0.923333333	3.6658	0.00032703	0.0019641	UP	-2.7989	0.013673	0.051398		0.84654	0.3895	0.60521	
transcript_HQ_CoA_transcript9865/f2p0/3149	1.496666667	4.426666667	1.14	1.6848	0.011015	0.038141	UP	-2.093	0.0013412	0.0080756	DOWN	-0.41975	0.35176	0.56921	
transcript_HQ_CoA_transcript9873/f2p0/3135	3.1	7.986666667	4.716666667	1.5346	0.00024568	0.0015324	UP	-0.92401	0.022604	0.075437		0.59774	0.078309	0.21635	
transcript_HQ_CoA_transcript9875/f4p0/3126	10.32333333	4.48	8.576666667	-1.052	3.6835E-07	4.683E-06	DOWN	0.78712	0.00026461	0.002153		-0.27732	0.16914	0.35988	
transcript_HQ_CoA_transcript9879/f4p0/3127	27.54666667	12.34666667	20.08333333	-1.0155	0.0027884	0.012231	DOWN	0.55564	0.00042377	0.0031847		-0.46919	0.13787	0.3159	
transcript_HQ_CoA_transcript9898/f2p0/3125	11.19666667	2.803333333	6.053333333	-1.8594	5.6312E-09	1.0153E-07	DOWN	0.97057	0.00020513	0.0017619		-0.89897	0.0027131	0.01922	
transcript_HQ_CoA_transcript9899/f2p0/3115	13.07666667	4.456666667	4.3	-1.4182	0.0025895	0.011488	DOWN	-0.1845	0.71748	0.83848		-1.6122	1.1114E-09	7.837E-08	DOWN
transcript_HQ_CoA_transcript990/f3p0/5751	13.82	67.19666667	14.33333333	2.4677	9.6847E-07	1.1416E-05	UP	-2.374	4.272E-41	7.1184E-38	DOWN	0.077526	0.8773	0.94515	
transcript_HQ_CoA_transcript9904/f2p0/3140	7.08	15.24	7.003333333	1.276	0.0018111	0.0085379	UP	-1.291	0.00081308	0.0054055	DOWN	-0.027615	0.91038	0.96028	
transcript_HQ_CoA_transcript9916/f3p0/3116	27.23	57.31	33.66666667	1.2415	0.00001534	0.00013574	UP	-0.92552	0.00099684	0.0063707		0.30306	0.059533	0.18047	
transcript_HQ_CoA_transcript9926/f2p0/3129	6.226666667	11.55333333	5.51	1.0601	0.0065784	0.024895	UP	-1.2261	0.003704	0.018458	DOWN	-0.17966	0.62979	0.79395	
transcript_HQ_CoA_transcript9932/f3p0/3124	19.58333333	54.43	21.01666667	1.6378	2.4019E-05	0.0002018	UP	-1.5347	3.4908E-05	0.0003984	DOWN	0.089455	0.74419	0.87059	
transcript_HQ_CoA_transcript9937/f2p0/3134	9.666666667	4.34	4.28	-1.0099	0.0076622	0.028259	DOWN	-0.15495	0.64505	0.78869		-1.1778	6.3296E-05	0.00098126	DOWN
transcript_HQ_CoA_transcript9939/f2p0/3116	15.70333333	5.206666667	3.773333333	-1.4259	1.7858E-07	2.4201E-06	DOWN	-0.61034	0.14181	0.29298		-2.0476	7.5983E-07	0.00002486	DOWN
transcript_HQ_CoA_transcript994/f4p0/5750	7.49	3.353333333	5.38	-1.0005	0.0011317	0.0057093	DOWN	0.52471	0.10941	0.24354		-0.49012	0.16477	0.35373	
transcript_HQ_CoA_transcript9940/f3p0/3143	5.876666667	12.02	20.22666667	1.1718	0.0012669	0.0062978	UP	0.60623	0.11555	0.25321		1.7673	1.7521E-06	5.0566E-05	UP
transcript_HQ_CoA_transcript9967/f4p0/3131	0.856666667	22.61333333	2.01	4.8897	5.3883E-20	3.3393E-18	UP	-3.6435	7.2092E-12	4.3899E-10	DOWN	1.2312	0.036015	0.1272	
transcript_HQ_CoA_transcript9973/f2p0/3116	3.456666667	9.893333333	2.383333333	1.6745	1.8902E-05	0.000163	UP	-2.2002	3.4022E-12	2.2271E-10	DOWN	-0.53848	0.24806	0.45788	
transcript_HQ_CoA_transcript9978/f3p0/3128	14.92	3.48	14.61666667	-1.9654	1.634E-06	1.8383E-05	DOWN	1.9476	0.00032626	0.0025501	UP	-0.03076	0.94435	0.97497	
transcript_HQ_CoA_transcript9984/f2p0/3114	4.933333333	9.94	6.156666667	1.1611	6.0111E-05	0.00045205	UP	-0.84012	0.01252	0.047937		0.31115	0.35149	0.56904	
transcript_HQ_CoA_transcript9989/f2p0/3116	6.48	12.53666667	17.41333333	1.1093	0.005975	0.022948	UP	0.30942	0.27321	0.46068		1.407	0.0002188	0.0026792	UP
transcript_HQ_CoA_transcript9998/f2p0/3154	8.856666667	3.866666667	5.35	-1.055	0.005912	0.02274	DOWN	0.32802	0.30759	0.49864		-0.73812	0.014162	0.064457	
transcript_LQ_CoA_transcript11569/f2p0/3023	6	2.606666667	3.336666667	-1.0527	0.0042376	0.017308	DOWN	0.20785	0.50005	0.67796		-0.85576	0.010226	0.051144	
transcript_LQ_CoA_transcript25563/f2p0/1761	0.996666667	32.85666667	13.59666667	5.186	4.3982E-13	1.4646E-11	UP	-1.3966	0.13182	0.27787		3.779	0.00062452	0.0061696	UP
transcript_LQ_CoA_transcript26637/f2p0/1773	16.02	0.926666667	23.48	-3.9329	1.2984E-05	0.0001178	DOWN	4.4645	3.3964E-10	1.4964E-08	UP	0.52536	0.39511	0.61061	
transcript_LQ_CoA_transcript29548/f3p0/1274	35.64	110.7233333	56.64333333	1.8069	4.1658E-14	1.5671E-12	UP	-1.1198	1.2296E-09	4.7151E-08	DOWN	0.67381	0.0023211	0.017082	
transcript_HQ_CoA_transcript10030/f3p0/3151	5.433333333	3.263333333	2.4	-0.6083	0.39647	0.57121		-0.55648	0.43342	0.62268		-1.1761	0.0051738	0.031317	DOWN
transcript_HQ_CoA_transcript10053/f2p0/3121	1.176666667	0.606666667	5.143333333	-0.81039	0.14658	0.28318		2.9606	6.5665E-20	1.5631E-17	UP	2.134	1.0714E-08	6.1072E-07	UP
transcript_HQ_CoA_transcript10082/f2p0/3106	7.426666667	7.25	36.65333333	0.13817	0.77754	0.86858		2.2008	1.191E-06	2.2006E-05	UP	2.3224	1.9168E-05	0.00036308	UP
transcript_HQ_CoA_transcript1012/f3p0/5746	0.313333333	5.116666667	11.98333333	4.1338	0.033429	0.091211		1.11	0.55876	0.72604		5.2299	8.7572E-47	8.0369E-43	UP
transcript_HQ_CoA_transcript10130/f6p0/3090	8.7	13.04	17.52666667	0.75792	0.034483	0.093521		0.2551	0.48234	0.66433		1.002	8.8484E-05	0.0012829	UP
transcript_HQ_CoA_transcript10140/f2p0/3127	15.17	2.953333333	2.516666667	-2.1545	0.35956	0.53487		-0.41405	0.86595	0.92732		-2.5836	0.00063047	0.006215	DOWN
transcript_HQ_CoA_transcript10162/f2p0/3127	33.87	26.02666667	15.09333333	-0.23169	0.38343	0.55895		-0.93325	1.2927E-07	3.1176E-06		-1.1755	4.6602E-06	0.00011255	DOWN
transcript_HQ_CoA_transcript10228/f2p0/3091	28.99666667	12.34	8.143333333	-1.0815	0.10083	0.21368		-0.76426	0.070765	0.17748		-1.8536	0.0010202	0.0090904	DOWN
transcript_HQ_CoA_transcript10254/f2p0/3082	3.486666667	1.733333333	0.803333333	-0.80014	0.40184	0.57647		-1.3226	0.19417	0.36505		-2.1395	4.2732E-07	0.00001526	DOWN
transcript_HQ_CoA_transcript10283/f2p0/3076	3.65	4.836666667	7.96	0.6076	0.37811	0.55354		0.53395	0.40789	0.59815		1.1274	0.00010564	0.0014915	UP
transcript_HQ_CoA_transcript10289/f3p0/3077	36.95666667	19.20333333	2.716666667	-0.79287	0.2891	0.45935		-2.9502	4.0228E-07	8.6441E-06	DOWN	-3.7582	1.5574E-10	1.3295E-08	DOWN
transcript_HQ_CoA_transcript10291/f2p0/3074	1.38	1.953333333	2.856666667	0.65366	0.12284	0.24799		0.38838	0.28251	0.47134		1.0293	0.0037491	0.024647	UP
transcript_HQ_CoA_transcript10310/f2p0/3083	0.066666667	0	1.133333333	-3.4102	0.38811	0.56367		7.5252	1.2848E-07	3.1027E-06	UP	4.1009	0.001406	0.01173	UP
transcript_HQ_CoA_transcript10315/f2p0/3079	1.586666667	0.716666667	3.953333333	-0.9507	0.44964	0.62205		2.2532	0.050282	0.13704		1.2928	0.002047	0.015552	UP
transcript_HQ_CoA_transcript10345/f2p0/3088	3.326666667	4.68	9.396666667	0.64897	0.081687	0.18289		0.86015	0.0065735	0.028949		1.4963	2.6985E-05	0.00048322	UP
transcript_HQ_CoA_transcript10371/f3p0/3102	11.49333333	10.39	5.773333333	0.00036164	0.99901	0.99965		-0.99249	0.007821	0.033152		-1.0055	0.0047929	0.029583	DOWN
transcript_HQ_CoA_transcript10388/f4p0/3071	5.85	4.206666667	1.933333333	-0.3429	0.5712	0.72696		-1.2451	0.063703	0.16443		-1.6023	0.0001078	0.0015185	DOWN
transcript_HQ_CoA_transcript10397/f3p0/3074	7.67	4.9	2.46	-0.50987	0.21281	0.37054		-1.1127	0.081801	0.19715		-1.637	0.0020238	0.015419	DOWN
transcript_HQ_CoA_transcript10518/f2p0/3066	34.57666667	18.08	13.73	-0.81505	0.13866	0.27194		-0.53121	0.17681	0.34202		-1.3544	0.0019037	0.014694	DOWN
transcript_HQ_CoA_transcript10571/f2p0/3053	6.773333333	2.76	0.566666667	-1.0975	0.15693	0.2981		-2.4299	7.1289E-05	0.0007239	DOWN	-3.5412	9.0699E-06	0.00019403	DOWN
transcript_HQ_CoA_transcript1058/f2p0/5690	2.876666667	3.633333333	0.57	0.50941	0.47416	0.64556		-2.8249	2.2235E-07	5.0944E-06	DOWN	-2.3304	0.0046722	0.02911	DOWN
transcript_HQ_CoA_transcript1059/f2p0/5712	13.65333333	6.22	3.493333333	-0.98953	0.00010816	0.000753		-0.95253	0.079961	0.19389		-1.9561	7.8633E-05	0.0011655	DOWN
transcript_HQ_CoA_transcript10613/f2p0/3050	1.433333333	2.586666667	4.523333333	0.98701	0.12745	0.2551		0.69252	0.24033	0.42237		1.6647	0.00033133	0.0037264	UP
transcript_HQ_CoA_transcript10647/f2p0/3048	10.47	10.55	26.31666667	0.17244	0.70002	0.81958		1.1477	0.00021264	0.0018111	UP	1.3097	0.00013131	0.0017775	UP
transcript_HQ_CoA_transcript1065/f2p0/5652	7.986666667	7.256666667	3.226666667	-0.0064625	0.98858	0.99473		-1.2962	0.0024127	0.012919	DOWN	-1.3136	0.003602	0.02386	DOWN
transcript_HQ_CoA_transcript10681/f2p0/3038	9.583333333	4.533333333	4.27	-0.92887	0.00016949	0.0011134		-0.23108	0.34516	0.53736		-1.1721	9.3236E-08	4.0746E-06	DOWN
transcript_HQ_CoA_transcript10696/f2p0/3050	1.95	2.38	5.383333333	0.45181	0.32997	0.50374		1.0119	0.0038817	0.019188	UP	1.4533	3.6064E-05	0.00061405	UP
transcript_HQ_CoA_transcript10705/f2p0/2985	10.36333333	5.36	4.393333333	-0.80646	0.043721	0.11296		-0.43876	0.094488	0.21904		-1.2579	0.0014317	0.011864	DOWN
transcript_HQ_CoA_transcript10726/f2p0/3029	15.30333333	9.896666667	5.013333333	-0.48088	0.12238	0.24734		-1.125	1.0388E-07	2.5591E-06	DOWN	-1.617	4.4173E-08	2.1225E-06	DOWN
transcript_HQ_CoA_transcript10727/f2p0/3016	12.30666667	3.346666667	1.026666667	-1.7461	0.073205	0.16877		-1.7992	0.0099647	0.039941	DOWN	-3.5585	3.7846E-05	0.00063731	DOWN
transcript_HQ_CoA_transcript10731/f4p0/3036	26.80333333	13.85666667	9.306666667	-0.78733	0.027448	0.078029		-0.73633	0.04843	0.13322		-1.5364	1.9571E-07	7.7753E-06	DOWN
transcript_HQ_CoA_transcript10769/f2p0/3034	40.71	10.03	9.08	-1.8239	0.037806	0.10043		-0.31984	0.72998	0.84666		-2.1564	0.00016465	0.0021274	DOWN
transcript_HQ_CoA_transcript10800/f8p0/2968	4.56	4.026666667	10.92666667	-0.0090105	0.9782	0.98924		1.296	0.00011483	0.001076	UP	1.2738	0.00034611	0.0038503	UP
transcript_HQ_CoA_transcript10803/f3p0/3042	35.91666667	27.37666667	2.856666667	-0.25271	0.45755	0.62975		-3.41	3.355E-27	1.8634E-24	DOWN	-3.6736	1.4394E-32	3.7743E-29	DOWN
transcript_HQ_CoA_transcript10814/f2p0/3018	6.98	2.766666667	1.46	-1.1901	0.075456	0.17263		-1.0531	0.070681	0.17742		-2.2586	6.8461E-05	0.0010435	DOWN
transcript_HQ_CoA_transcript10880/f2p0/3012	19.74	10.76333333	8.356666667	-0.71996	7.1856E-05	0.00052821		-0.50989	0.014634	0.054035		-1.2422	5.8646E-10	4.3937E-08	DOWN
transcript_HQ_CoA_transcript10918/f5p0/2997	21.76	14.96666667	9.69	-0.36815	0.2659	0.43353		-0.77371	0.049535	0.13547		-1.1537	0.0034164	0.022928	DOWN
transcript_HQ_CoA_transcript10932/f2p0/3019	1.92	2.416666667	7.583333333	0.49191	0.15898	0.30078		1.5017	0.00063392	0.0044212	UP	1.9789	8.4359E-06	0.00018455	UP
transcript_HQ_CoA_transcript10949/f2p0/3010	1.2	0.966666667	4.136666667	-0.13457	0.86882	0.9265		1.9478	0.00051707	0.003749	UP	1.7991	0.0029876	0.020685	UP
transcript_HQ_CoA_transcript10966/f2p0/2991	7.513333333	5.633333333	2.873333333	-0.26843	0.33779	0.51283		-1.1182	0.022567	0.075344		-1.4006	0.0033664	0.022667	DOWN
transcript_HQ_CoA_transcript11011/f2p0/3009	0.066666667	0.3	1.003333333	2.2334	0.31488	0.48731		1.6592	0.20281	0.37633		3.887	0.00026534	0.0031401	UP
transcript_HQ_CoA_transcript11021/f2p0/3014	8.853333333	4.366666667	3.25	-0.86645	0.065144	0.15417		-0.5662	0.12205	0.2633		-1.4463	0.0010135	0.0090524	DOWN
transcript_HQ_CoA_transcript11041/f2p0/3075	20.37666667	10.81	10.13	-0.76274	0.0020198	0.0093365		-0.23417	0.31619	0.50713		-1.0098	1.5744E-08	8.5246E-07	DOWN
transcript_HQ_CoA_transcript11070/f2p0/3011	3.83	1.67	1.043333333	-1.0514	0.017985	0.055735		-0.83051	0.10893	0.24274		-1.8953	1.6782E-05	0.00032563	DOWN
transcript_HQ_CoA_transcript11074/f2p0/3049	0.156666667	0.026666667	168.0933333	-2.2203	0.42592	0.59981		12.37	0.0012004	0.007388	UP	10.127	0.00089159	0.0081703	UP
transcript_HQ_CoA_transcript11076/f2p0/2986	0.943333333	2.826666667	5.43	1.7235	0.059129	0.14278		0.82084	0.23911	0.4211		2.5316	4.0154E-05	0.00066821	UP

transcript_HQ_CoA_transcript11092/f2p0/2997	5.156666667	8.14	12.28666667	0.79473	0.11238	0.23205		0.4643	0.305	0.49574		1.2485	2.8048E-06	7.5355E-05	UP
transcript_HQ_CoA_transcript11100/f7p0/2965	0.126666667	0.3	31.45333333	1.3823	0.32601	0.49938		6.6304	0.0042019	0.020494	UP	8	0.0062383	0.035951	UP
transcript_HQ_CoA_transcript11192/f4p0/2982	28.71333333	15.78	4.71	-0.75954	0.328	0.5016		-1.8586	0.0064839	0.028639	DOWN	-2.6228	1.6727E-05	0.00032489	DOWN
transcript_HQ_CoA_transcript11196/f2p0/2985	0	0	4.916666667	NA	NA	NA	NA	9.6067	1.0736E-13	9.8394E-12	UP	9.6132	1.0234E-13	1.8061E-11	UP
transcript_HQ_CoA_transcript11225/f2p0/2962	10.09	9.94	1.156666667	0.12847	0.87489	0.93036		-3.2293	5.4665E-08	1.4521E-06	DOWN	-3.1158	9.1591E-06	0.00019571	DOWN
transcript_HQ_CoA_transcript11259/f2p0/3000	1.113333333	2.35	5.026666667	1.2053	0.11623	0.23788		0.93994	0.094644	0.21923		2.1377	0.00062855	0.0061994	UP
transcript_HQ_CoA_transcript11260/f2p0/3005	11.26	6.95	5.57	-0.53671	0.15947	0.30146		-0.46291	0.08761	0.20729		-1.0145	0.0031803	0.021677	DOWN
transcript_HQ_CoA_transcript11290/f2p0/2985	1.846666667	3.736666667	0.073333333	1.1907	0.060431	0.14537		-6.027	5.1779E-10	2.157E-08	DOWN	-4.8412	3.6528E-06	9.2767E-05	DOWN
transcript_HQ_CoA_transcript11311/f8p0/2909	40.5	18.46	16.18666667	-0.98455	1.4279E-06	1.6284E-05		-0.32957	0.18827	0.35709		-1.327	4.8708E-10	3.7407E-08	DOWN
transcript_HQ_CoA_transcript11332/f2p0/2984	6.433333333	4.973333333	2.686666667	-0.23227	0.60352	0.75279		-1.0328	0.029753	0.09276		-1.2757	0.00070365	0.0067822	DOWN
transcript_HQ_CoA_transcript11465/f2p0/2974	5.726666667	3.8	1.886666667	-0.44157	0.44419	0.61749		-1.1207	0.02992	0.09303		-1.5758	0.0015124	0.0123	DOWN
transcript_HQ_CoA_transcript11478/f2p0/2967	0.88	0.57	4.72	-0.42026	0.64373	0.7815		2.851	0.00004489	0.00049298	UP	2.4227	2.8652E-08	1.4568E-06	UP
transcript_HQ_CoA_transcript11481/f2p0/2945	16.08	6.933333333	6.126666667	-1.0979	0.05475	0.13423		-0.30339	0.60033	0.75781		-1.4091	0.00046833	0.0049093	DOWN
transcript_HQ_CoA_transcript11497/f2p0/2951	2.686666667	3.533333333	6.3	0.5291	0.21759	0.37609		0.70442	0.17698	0.34215		1.2194	0.0077198	0.041663	UP
transcript_HQ_CoA_transcript11511/f2p0/2948	30.07666667	33.44333333	7.373333333	0.36786	0.61425	0.75989		-2.3781	0.0003311	0.0025813	DOWN	-2.0266	4.481E-06	0.00010923	DOWN
transcript_HQ_CoA_transcript11520/f2p0/2876	0	0.02	11.29	1.8448	0.648	0.7847		8.9571	0.00020995	0.0017898	UP	10.793	0.00015185	0.001998	UP
transcript_HQ_CoA_transcript11548/f2p0/2952	3.03	3.293333333	7.26	0.29674	0.52206	0.68665		0.97324	0.013626	0.05124		1.2569	2.6574E-06	7.2068E-05	UP
transcript_HQ_CoA_transcript11563/f12p0/2930	0	0	75.25333333	NA	NA	NA	NA	27.325	2.6665E-12	1.8305E-10	UP	27.005	4.7725E-12	6.0413E-10	UP
transcript_HQ_CoA_transcript11576/f2p0/2939	0	0.133333333	0.683333333	4.544	0.2212	0.38054		2.2435	0.25602	0.44007		6.7715	0.0055643	0.033031	UP
transcript_HQ_CoA_transcript11587/f3p0/2950	15.08666667	9.613333333	7.283333333	-0.48627	0.10567	0.22144		-0.56048	0.046611	0.12931		-1.0587	1.8281E-07	7.3907E-06	DOWN
transcript_HQ_CoA_transcript11590/f2p0/2952	1.9	0.46	0.146666667	-1.9136	0.047531	0.1203		-1.7601	0.12946	0.27456		-3.6752	0.0029306	0.020391	DOWN
transcript_HQ_CoA_transcript11592/f2p0/2935	10.82333333	15.49666667	25.54666667	0.72322	0.18741	0.33853		0.54913	0.2439	0.4266		1.2576	0.0043024	0.027288	UP
transcript_HQ_CoA_transcript11593/f2p0/2751	143.85	70.98	56.38666667	-0.89486	0.070451	0.16401		-0.44923	0.31252	0.50407		-1.3539	4.3425E-05	0.00071231	DOWN
transcript_HQ_CoA_transcript11597/f2p0/2955	11.46	7.59	3.743333333	-0.46205	0.23619	0.39863		-1.1456	0.011849	0.045876	DOWN	-1.6212	9.7326E-07	3.0854E-05	DOWN
transcript_HQ_CoA_transcript11693/f2p0/2919	0.636666667	0.65	2.126666667	0.15319	0.83768	0.90753		1.5913	0.0027247	0.014264	UP	1.7369	0.00035485	0.0039342	UP
transcript_HQ_CoA_transcript1174/f2p0/5653	1.72	0.663333333	6.343333333	-1.1761	0.19113	0.34319		3.0699	0.0001274	0.001174	UP	1.8806	1.7903E-10	1.5005E-08	UP
transcript_HQ_CoA_transcript11772/f2p0/2915	12.18666667	8.273333333	4.426666667	-0.40788	0.13585	0.26771		-1.0461	3.5342E-07	7.6843E-06	DOWN	-1.466	6.2028E-08	2.8678E-06	DOWN
transcript_HQ_CoA_transcript11790/f2p0/2916	1.246666667	0.89	3.926666667	-0.31792	0.58728	0.74035		2.0026	6.2106E-07	1.2691E-05	UP	1.6712	4.8246E-05	0.0007768	UP
transcript_HQ_CoA_transcript11846/f2p0/2908	0.453333333	1.076666667	2.036666667	1.4605	0.1159	0.23741		0.75	0.20292	0.37646		2.191	0.0075991	0.041244	UP
transcript_HQ_CoA_transcript11879/f2p0/2942	8.96	6.866666667	3.436666667	-0.26187	0.60978	0.75682		-1.1256	0.013464	0.050758		-1.3966	0.00014655	0.0019407	DOWN
transcript_HQ_CoA_transcript11883/f3p0/2937	17.19666667	8.166666667	7.803333333	-0.93731	0.010081	0.035478		-0.19754	0.59703	0.75521		-1.145	2.9907E-06	7.9328E-05	DOWN
transcript_HQ_CoA_transcript11885/f4p0/2892	9.896666667	4.916666667	2.74	-0.86296	0.013656	0.04518		-0.99182	0.011069	0.043461		-1.8657	1.4863E-05	0.00029461	DOWN
transcript_HQ_CoA_transcript11896/f2p0/2913	7.5	5.3	3.55	-0.38025	0.53831	0.70066		-0.70441	0.27953	0.46768		-1.0921	0.0036886	0.024321	DOWN
transcript_HQ_CoA_transcript11897/f3p0/2909	6.733333333	2.906666667	1.566666667	-0.99926	NA	NA		-1.1132	NA	NA	NA	-2.1244	2.5455E-06	6.9631E-05	DOWN
transcript_HQ_CoA_transcript11969/f2p0/2826	15.07666667	7.186666667	4.61	-0.92601	0.01108	0.038315		-0.77797	0.035449	0.10534		-1.7139	1.212E-06	3.7201E-05	DOWN
transcript_HQ_CoA_transcript11974/f17p0/2869	0.313333333	0.35	456.3266667	0.36417	0.69399	0.81514		10.223	1.0065E-06	1.9118E-05	UP	10.573	2.7984E-07	1.0619E-05	UP
transcript_HQ_CoA_transcript120/f7p0/7631	8.323333333	10.47666667	1.026666667	0.50193	0.3576	0.53283		-3.4981	2.2037E-16	2.9919E-14	DOWN	-3.0143	9.9346E-07	3.1386E-05	DOWN
transcript_HQ_CoA_transcript12039/f7p0/2854	70.3	43.72	28.96666667	-0.55042	0.18134	0.3309		-0.74219	0.042121	0.11973		-1.3022	4.0643E-05	0.0006745	DOWN
transcript_HQ_CoA_transcript12054/f3p0/2887	3.95	3.993333333	0.886666667	0.15454	0.85183	0.91645		-2.2772	0.0058608	0.026577	DOWN	-2.1333	0.0043916	0.027777	DOWN
transcript_HQ_CoA_transcript12084/f2p0/2879	2.033333333	2.15	7.616666667	0.29116	0.74571	0.8483		1.6402	0.048617	0.13355		1.9151	0.00016425	0.0021274	UP
transcript_HQ_CoA_transcript12087/f3p0/2893	4.843333333	4.953333333	10.83	0.21943	0.70891	0.82503		0.93934	0.087557	0.20725		1.1468	1.3725E-06	4.1231E-05	UP
transcript_HQ_CoA_transcript12108/f2p0/2890	7.613333333	7.016666667	17.21	0.040612	0.92148	0.95708		1.1431	2.3287E-08	6.6795E-07	UP	1.1732	0.001395	0.01166	UP
transcript_HQ_CoA_transcript12133/f2p0/2881	5.56	8.043333333	21.22	0.66152	0.18363	0.33387		1.2726	0.0042087	0.020522	UP	1.9237	5.1783E-13	7.9872E-11	UP
transcript_HQ_CoA_transcript12146/f5p0/2820	5.116666667	5.106666667	1.266666667	0.13864	0.87102	0.92802		-2.1052	0.020494	0.069833		-1.9798	0.0070328	0.038999	DOWN
transcript_HQ_CoA_transcript12147/f2p0/2904	6.73	8.68	14.14333333	0.52579	0.11896	0.24202		0.54979	0.12881	0.27358		1.0646	0.0030884	0.02125	UP
transcript_HQ_CoA_transcript12196/f3p0/2859	18.41666667	26.59333333	6.94	0.68664	0.13511	0.26657		-2.0736	2.9354E-06	4.8254E-05	DOWN	-1.3998	0.00056545	0.0056777	DOWN
transcript_HQ_CoA_transcript12243/f2p0/2868	52.13	18.34	20.89	-1.3797	0.10808	0.22498		0.068174	0.93659	0.96667		-1.3238	1.9661E-05	0.00037128	DOWN
transcript_HQ_CoA_transcript1229/f2p0/5510	13.10666667	6.033333333	3.313333333	-0.97463	0.0002418	0.0015118		-1.0021	7.4526E-05	0.00075344	DOWN	-1.9881	6.6619E-18	2.2644E-15	DOWN
transcript_HQ_CoA_transcript12328/f2p0/2845	3.506666667	5.036666667	8.01	0.65756	0.15463	0.29472		0.533	0.078753	0.19213		1.1809	0.0023544	0.017265	UP
transcript_HQ_CoA_transcript12360/f2p0/2881	2.593333333	6.71	6.12	1.5189	0.094367	0.20321		-0.25376	0.76809	0.86973		1.2505	0.0012135	0.010438	UP
transcript_HQ_CoA_transcript12371/f3p0/2846	4.23	2.236666667	20.16	-0.67845	NA	NA		2.9692	0.0019024	0.01067	UP	2.2755	0.0018399	0.014335	UP
transcript_HQ_CoA_transcript12386/f4p0/2831	14.19333333	8.29	5.08	-0.63108	0.28941	0.4596		-0.87803	0.025782	0.083372		-1.519	0.005956	0.034894	DOWN
transcript_HQ_CoA_transcript1239/f2p0/5582	0.223333333	0.423333333	1.24	1.0978	0.35761	0.53283		1.3549	0.14908	0.30334		2.4437	0.00080925	0.007563	UP
transcript_HQ_CoA_transcript12436/f2p0/2849	1.146666667	4.89	5.89	2.2827	0.018778	0.057717		0.12103	0.87578	0.93237		2.3862	0.0010704	0.009473	UP
transcript_HQ_CoA_transcript1246/f2p0/5500	1.603333333	1.536666667	3.953333333	0.084499	0.89458	0.94215		1.2113	0.02926	0.091582		1.2856	2.6344E-05	0.00047407	UP
transcript_HQ_CoA_transcript12495/f10p0/2815	11.5	8.023333333	4.37	-0.36059	0.35861	0.53405		-1.0238	0.0052815	0.024458	DOWN	-1.3964	0.00059125	0.0058885	DOWN
transcript_HQ_CoA_transcript12532/f2p0/2843	10.37666667	6.486666667	2.996666667	-0.55583	0.27766	0.44633		-1.2393	0.015558	0.056615		-1.8042	3.3442E-08	1.6635E-06	DOWN
transcript_HQ_CoA_transcript12546/f2p0/2782	1.676666667	3.013333333	5.606666667	1.0483	0.10662	0.22284		0.70815	0.19327	0.36387		1.7421	2.2099E-05	0.00040848	UP
transcript_HQ_CoA_transcript12595/f3p0/2852	4.966666667	2.066666667	0.893333333	-1.1309	0.095987	0.20582		-1.3308	0.07575	0.18672		-2.4783	4.6081E-09	2.8769E-07	DOWN
transcript_HQ_CoA_transcript126/f2p0/7716	0.16	0.73	7.506666667	2.4486	NA	NA	NA	3.1353	0.25374	0.43764		5.565	5.9986E-06	0.00013973	UP
transcript_HQ_CoA_transcript12614/f4p0/2795	2.253333333	1.89	0.683333333	-0.11789	0.86082	0.92194		-1.6278	0.023267	0.077049		-1.7593	0.00029998	0.0034378	DOWN
transcript_HQ_CoA_transcript12634/f2p0/2820	8.41	4.21	3.106666667	-0.84948	0.078863	0.17824		-0.57141	0.32844	0.51928		-1.4365	0.0046772	0.029112	DOWN
transcript_HQ_CoA_transcript12674/f2p0/2836	10.72	18.05333333	38.26	0.9211	0.0047609	0.01905		0.92581	3.4451E-08	9.553E-07		1.8327	2.9412E-10	2.3071E-08	UP
transcript_HQ_CoA_transcript12677/f2p0/2818	5.203333333	3.1	2.22	-0.59502	0.058649	0.14186		-0.62293	0.03948	0.11378		-1.2314	6.4093E-05	0.00099103	DOWN
transcript_HQ_CoA_transcript1271/f3p0/5466	2.83	2.96	1.096666667	0.20724	0.73531	0.842		-1.5559	0.0074341	0.031922	DOWN	-1.3638	0.00086987	0.0080113	DOWN
transcript_HQ_CoA_transcript12711/f2p0/2808	5.22	5.066666667	20.42666667	0.16309	0.82617	0.89969		1.8091	0.010351	0.041129	UP	1.9597	7.1491E-08	3.2561E-06	UP
transcript_HQ_CoA_transcript12716/f2p0/2825	1.25	3.34	10.53333333	1.5596	0.020766	0.062512		1.5245	0.002176	0.011867	UP	3.0696	1.1514E-15	2.9351E-13	UP
transcript_HQ_CoA_transcript1274/f2p0/5464	6.49	4.606666667	15.34333333	-0.3393	0.41508	0.58977		1.5717	1.9966E-10	9.1033E-09	UP	1.2224	0.00076052	0.0072029	UP
transcript_HQ_CoA_transcript12743/f3p0/2866	9.346666667	5.076666667	4.303333333	-0.71593	0.078561	0.1778		-0.40494	0.29425	0.48356		-1.1316	0.0041164	0.026428	DOWN
transcript_HQ_CoA_transcript12744/f2p0/2821	4.433333333	6.743333333	14.33	0.74061	0.061913	0.14812		0.9403	0.0051668	0.024042		1.6691	4.1996E-06	0.00010389	UP
transcript_HQ_CoA_transcript12755/f2p0/2866	5.086666667	9.466666667	10.40333333	1.034	0.040575	0.10629		0.0050542	0.99087	0.99514		1.0258	0.0031514	0.021549	UP
transcript_HQ_CoA_transcript12817/f2p0/2807	14.92	7.013333333	57.63666667	-0.91307	0.012376	0.041837		2.8602	2.6878E-12	1.8382E-10	UP	1.9355	8.8436E-11	8.1162E-09	UP
transcript_HQ_CoA_transcript12822/f2p0/2809	6.743333333	9.826666667	16.66	0.70509	0.0031457	0.013515		0.62924	0.076881	0.18854		1.3214	0.00027675	0.0032493	UP
transcript_HQ_CoA_transcript12831/f3p0/2754	2.796666667	5.15	9.453333333	1.0076	0.11141	0.23043		0.72951	0.0066327	0.02914		1.7274	0.0024252	0.017626	UP
transcript_HQ_CoA_transcript1287/f2p0/5460	1.616666667	1.943333333	0.696666667	0.41486	0.36866	0.54437		-1.6247	0.00025619	0.0020944	DOWN	-1.2219	0.00099891	0.0089526	DOWN
transcript_HQ_CoA_transcript12885/f2p0/2823	34.51	32.32333333	11.34666667	0.048486	0.93182	0.96221		-1.6533	0.0030555	0.015687	DOWN	-1.6166	0.00094106	0.0085132	DOWN
transcript_HQ_CoA_transcript12899/f2p0/2796	49.89333333	25.74666667	17.43666667	-0.81113	0.059601	0.14366		-0.71266	0.0011716	0.0072497		-1.533	0.00017247	0.0022106	DOWN
transcript_HQ_CoA_transcript12935/f2p0/2795	4.11	3.656666667	1.99	-0.0012464	0.99739	0.9987		-1.0236	0.016214	0.058501		-1.0367	0.0091751	0.047226	DOWN
transcript_HQ_CoA_transcript12981/f2p0/2788	1.1	2.433333333	4.833333333	1.2575	0.19096	0.34297		0.86532	0.14782	0.30155		2.1137	0.0060746	0.035352	UP

transcript_HQ_CoA_transcript12984/f2p0/2786	19.98333333	4.146666667	0.473333333	-2.0365	NA	NA	NA	-3.3173	0.043662	0.12304		-5.3859	2.4338E-21	1.2764E-18	DOWN
transcript_HQ_CoA_transcript1302/f2p0/5445	7.043333333	6.336666667	18.31333333	0.00023885	0.99936	0.99968		1.3899	1.2864E-08	3.9297E-07	UP	1.3794	3.0278E-06	8.0194E-05	UP
transcript_HQ_CoA_transcript13074/f2p0/2788	13.31666667	8.496666667	2.89	-0.51675	0.3708	0.54617		-1.6967	0.004693	0.022302	DOWN	-2.2218	1.2031E-06	3.6989E-05	DOWN
transcript_HQ_CoA_transcript13154/f2p0/2801	66.67333333	36.42333333	33.17333333	-0.72522	0.017179	0.053797		-0.27724	0.31425	0.50568		-1.0146	1.4538E-06	4.3388E-05	DOWN
transcript_HQ_CoA_transcript13156/f2p0/2770	4.973333333	6.76	14.07333333	0.58722	0.075201	0.17233		0.90552	0.01351	0.050887		1.4822	7.4648E-06	0.00016598	UP
transcript_HQ_CoA_transcript13204/f2p0/2759	3.64	3.556666667	1.166666667	0.13404	0.80102	0.8839		-1.7436	2.5806E-06	4.2961E-05	DOWN	-1.6211	0.0061135	0.035488	DOWN
transcript_HQ_CoA_transcript13298/f2p0/2756	6.456666667	7.256666667	0.153333333	0.34225	0.46376	0.63542		-5.7252	3.5275E-10	1.5468E-08	DOWN	-5.387	3.6733E-11	3.6055E-09	DOWN
transcript_HQ_CoA_transcript13320/f2p0/2755	0.276666667	0.483333333	12.50666667	0.96074	0.27511	0.44361		4.6057	0.00021626	0.0018317	UP	5.5534	0.00047903	0.0050043	UP
transcript_HQ_CoA_transcript13332/f2p0/2753	39.8	19.53	19.33333333	-0.88149	0.00044266	0.0025415		-0.1629	0.2136	0.38998		-1.055	9.6041E-06	0.00020356	DOWN
transcript_HQ_CoA_transcript13355/f2p0/2767	11.18	6.066666667	5.02	-0.72238	0.0012369	0.0061661		-0.42768	0.059577	0.15635		-1.1619	5.2505E-07	1.8149E-05	DOWN
transcript_HQ_CoA_transcript13385/f5p0/2740	9.473333333	14.53666667	22.42666667	0.75277	0.054661	0.13408		0.50096	0.19663	0.36835		1.2432	1.3759E-08	7.6066E-07	UP
transcript_HQ_CoA_transcript1342/f3p0/5412	2.986666667	4.013333333	1.346666667	0.56572	0.24334	0.40746		-1.7014	0.0023175	0.012519	DOWN	-1.1507	0.0096931	0.049203	DOWN
transcript_HQ_CoA_transcript1344/f2p0/5388	1.36	1.246666667	6.806666667	0.037486	0.91941	0.95579		2.2931	1.2501E-15	1.5587E-13	UP	2.3203	1.1411E-20	5.2363E-18	UP
transcript_HQ_CoA_transcript13454/f7p0/2727	16.67333333	12.72	4.513333333	-0.26205	0.54445	0.70598		-1.6278	7.5265E-05	0.0007584	DOWN	-1.8998	1.8984E-11	2.0026E-09	DOWN
transcript_HQ_CoA_transcript13520/f2p0/2732	6.53	13.91	0.18	1.2147	0.58259	0.73637		-6.3703	0.017485	0.061906		-5.1604	3.3603E-05	0.00058394	DOWN
transcript_HQ_CoA_transcript13558/f7p0/2725	48.05	28.01	13.79666667	-0.69244	0.6752	0.8035		-1.1243	0.47516	0.65859		-1.8249	0.0008446	0.0078415	DOWN
transcript_HQ_CoA_transcript13561/f3p0/2744	31.85	16.28333333	5.93	-0.81324	0.051673	0.12867		-1.6129	1.3165E-06	2.3915E-05	DOWN	-2.4368	1.7706E-11	1.8785E-09	DOWN
transcript_HQ_CoA_transcript13598/f3p0/2733	1.536666667	2.77	5.126666667	1.0376	0.045834	0.11697		0.71058	0.10255	0.23277		1.7335	0.00013912	0.0018626	UP
transcript_HQ_CoA_transcript13645/f46p0/2624	0.013333333	0.05	128.6666667	1.9344	0.59052	0.7428		11.274	0.00013796	0.0012519	UP	13.301	0.0003244	0.003662	UP
transcript_HQ_CoA_transcript13671/f3p0/2752	6.56	7.81	18.06	0.42929	0.26372	0.43116		1.0396	0.0039487	0.019477	UP	1.4555	0.00004811	0.0007753	UP
transcript_HQ_CoA_transcript137/f2p0/7585	0.533333333	0.69	12.28666667	0.62168	0.71512	0.82882		3.9474	NA	NA	NA	4.55	0.0028049	0.019711	UP
transcript_HQ_CoA_transcript13700/f7p0/2714	4.84	11.27666667	13.86	1.378	0.023359	0.06889		0.16327	0.71933	0.83927		1.5263	0.00081955	0.0076476	UP
transcript_HQ_CoA_transcript13744/f10p0/2656	10.75666667	10.39	23.07	0.1255	0.70683	0.82407		0.9801	0.0011104	0.0069555		1.0908	0.00012258	0.0016791	UP
transcript_HQ_CoA_transcript13762/f2p0/2717	11.13666667	3.793333333	2.216666667	-1.4178	0.02448	0.071482		-0.90069	0.07956	0.19339		-2.3305	1.0535E-05	0.00022027	DOWN
transcript_HQ_CoA_transcript13767/f3p0/2717	31.66333333	28.03	15.13	0.00010983	0.99972	0.99972		-1.0486	0.00022299	0.0018723	DOWN	-1.062	4.3772E-06	0.00010712	DOWN
transcript_HQ_CoA_transcript13802/f3p0/2759	18.13	6.606666667	5.34	-1.3278	0.038148	0.1012		-0.44534	0.48155	0.66386		-1.7802	5.1351E-05	0.00081919	DOWN
transcript_HQ_CoA_transcript13831/f3p0/2724	24.11	32.72	10.39666667	0.59884	0.0010563	0.0053852		-1.7934	5.9481E-05	0.00062015	DOWN	-1.2087	0.0045859	0.028738	DOWN
transcript_HQ_CoA_transcript13862/f3p0/2713	0.896666667	0.076666667	7.863333333	-3.3023	0.043707	0.11294		6.4579	5.7644E-10	2.369E-08	UP	3.1467	1.502E-06	4.4323E-05	UP
transcript_HQ_CoA_transcript13874/f7p0/2711	13.70333333	12.05666667	6.286666667	-0.017254	0.96284	0.98035		-1.0834	0.00010483	0.0010013	DOWN	-1.1124	0.0067129	0.037848	DOWN
transcript_HQ_CoA_transcript1388/f2p0/5373	1.31	1.076666667	0.526666667	-0.14532	0.81022	0.88958		-1.1643	0.041501	0.11846		-1.3206	0.0058214	0.034324	DOWN
transcript_HQ_CoA_transcript13911/f11p0/2568	42.56666667	26.06	20.35333333	-0.55815	0.12065	0.24468		-0.49769	0.13028	0.2756		-1.0687	1.4211E-05	0.00028444	DOWN
transcript_HQ_CoA_transcript13921/f2p0/2661	4.253333333	2.903333333	0.79	-0.40073	0.37805	0.55354		-2.0091	8.8633E-06	0.00012205	DOWN	-2.4236	3.2023E-08	1.606E-06	DOWN
transcript_HQ_CoA_transcript13936/f2p0/2706	9.33	3.44	1.083333333	-1.2838	0.16366	0.30731		-1.7747	0.031932	0.097647		-3.0748	0.0016931	0.013453	DOWN
transcript_HQ_CoA_transcript13971/f2p0/2691	0	0	18.71333333	NA	NA	NA	NA	11.408	0.0035021	0.017639	UP	11.413	0.0034867	0.023306	UP
transcript_HQ_CoA_transcript13990/f3p0/2710	10.54333333	9.3	3.413333333	-0.037498	0.90287	0.94683		-1.5756	4.3958E-05	0.00048391	DOWN	-1.6268	1.6846E-07	6.8562E-06	DOWN
transcript_HQ_CoA_transcript14045/f3p0/2704	4.533333333	6.52	10.73	0.68783	0.023208	0.068532		0.58496	0.1108	0.24571		1.259	0.0012688	0.010812	UP
transcript_HQ_CoA_transcript14067/f3p0/2688	59.02666667	43.90333333	29.05333333	-0.26288	0.26436	0.43188		-0.75118	0.0024553	0.013109		-1.0268	5.1658E-08	2.4438E-06	DOWN
transcript_HQ_CoA_transcript14069/f2p0/2694	0.95	0.89	4.943333333	0.0062898	0.99507	0.99743		2.3383	4.7404E-05	0.00051749	UP	2.3417	0.0018105	0.014189	UP
transcript_HQ_CoA_transcript14088/f11p0/2634	2.156666667	5.403333333	4.536666667	1.4723	0.02209	0.065777		-0.40814	0.48689	0.66799		1.0518	0.0059404	0.034824	UP
transcript_HQ_CoA_transcript14091/f3p0/2699	12.28	8.826666667	1.723333333	-0.33128	0.27925	0.44805		-2.5086	2.9229E-08	8.1918E-07	DOWN	-2.8523	1.616E-09	1.0747E-07	DOWN
transcript_HQ_CoA_transcript14097/f2p0/2720	2.716666667	3.266666667	6.836666667	0.44119	0.43953	0.61306		0.90224	0.069189	0.17444		1.3313	0.00046666	0.0048974	UP
transcript_HQ_CoA_transcript14098/f2p0/2720	6.596666667	3.716666667	2.87	-0.6332	0.26879	0.43684		-0.55899	0.32022	0.51141		-1.2074	0.001007	0.0090072	DOWN
transcript_HQ_CoA_transcript14146/f2p0/2680	5.823333333	5.56	12.89	0.079939	0.81168	0.89049		1.0739	0.00027621	0.0022256	UP	1.142	1.0056E-07	4.3227E-06	UP
transcript_HQ_CoA_transcript14161/f3p0/2687	20.15666667	10.65666667	9.746666667	-0.73038	0.081693	0.18289		-0.30155	0.42888	0.61834		-1.0463	0.00015304	0.0020108	DOWN
transcript_HQ_CoA_transcript14184/f2p0/2669	24.04	11.82666667	7.746666667	-0.85812	0.011686	0.039995		-0.7643	0.0043091	0.020897		-1.6347	2.0572E-06	5.8261E-05	DOWN
transcript_HQ_CoA_transcript14188/f2p0/2678	1.416666667	1.746666667	4.163333333	0.44055	0.50016	0.66759		1.1189	0.034469	0.10333		1.5466	0.00012143	0.0016687	UP
transcript_HQ_CoA_transcript14220/f2p0/2677	13.07	8.843333333	0.483333333	-0.39871	0.70677	0.82405		-4.3098	5.5095E-06	8.1373E-05	DOWN	-4.7247	8.7267E-09	5.0213E-07	DOWN
transcript_HQ_CoA_transcript14234/f4p0/2651	19.33	41.49333333	0.02	1.301	0.40243	0.5769		-10.932	1.783E-25	9.3373E-23	DOWN	-9.6825	0.00005804	0.00090742	DOWN
transcript_HQ_CoA_transcript14236/f2p0/2665	7.006666667	3.046666667	3.206666667	-1.0297	0.026726	0.07662		-0.10048	0.82097	0.9024		-1.142	0.0003347	0.0037529	DOWN
transcript_HQ_CoA_transcript14239/f2p0/2677	1.696666667	1.03	0.693333333	-0.57509	0.1922	0.34433		-0.68609	0.13932	0.28949		-1.2723	0.0080743	0.043132	DOWN
transcript_HQ_CoA_transcript14242/f2p0/2687	13.97666667	9.276666667	5.08	-0.45603	0.39761	0.5722		-1.0212	0.016168	0.05837		-1.4875	0.00074788	0.0071052	DOWN
transcript_HQ_CoA_transcript14316/f2p0/2727	1.29	1.093333333	10.94666667	-0.054455	0.92254	0.95752		3.1519	4.264E-18	7.8156E-16	UP	3.0815	6.909E-27	9.0582E-24	UP
transcript_HQ_CoA_transcript1432/f2p0/5350	6.76	6.75	3.276666667	0.14251	0.62035	0.76481		-1.1873	5.0668E-05	0.00054775	DOWN	-1.0546	0.00060756	0.0060247	DOWN
transcript_HQ_CoA_transcript14321/f2p0/2681	4.02	2.376666667	1.053333333	-0.62649	0.46781	0.63925		-1.2929	0.14059	0.29103		-1.9302	0.00014529	0.0019283	DOWN
transcript_HQ_CoA_transcript14329/f4p0/2666	3.273333333	4.86	0.8	0.69142	0.44788	0.62073		-2.7016	0.0030083	0.015488	DOWN	-2.0202	0.0010443	0.0092782	DOWN
transcript_HQ_CoA_transcript14330/f4p0/2661	9.46	16.19666667	22.66666667	0.94969	0.0039277	0.016312		0.33262	0.10156	0.23121		1.2683	2.1054E-05	0.00039154	UP
transcript_HQ_CoA_transcript14367/f2p0/2648	7.15	7.8	2.413333333	0.25532	0.56998	0.726		-1.8243	0.0002173	0.0018375	DOWN	-1.5776	5.3192E-06	0.00012663	DOWN
transcript_HQ_CoA_transcript14371/f3p0/2669	8.26	3.663333333	1.763333333	-0.99079	0.025638	0.074071		-1.2093	0.017916	0.063103		-2.213	8.2998E-07	2.6821E-05	DOWN
transcript_HQ_CoA_transcript1442/f3p0/5359	4.41	6.643333333	10.43333333	0.75137	0.0013631	0.0067132		0.49072	0.039519	0.11386		1.229	1.8194E-07	7.3719E-06	UP
transcript_HQ_CoA_transcript14441/f3p0/2637	37.49666667	24.29	18.70666667	-0.46255	0.016019	0.050969		-0.52634	0.016142	0.058323		-1.0018	8.8396E-07	2.8316E-05	DOWN
transcript_HQ_CoA_transcript14450/f3p0/2632	8.293333333	6.743333333	19.47	-0.13416	0.75909	0.85697		1.3782	1.1001E-06	2.0596E-05	UP	1.2294	0.0023053	0.017014	UP
transcript_HQ_CoA_transcript14487/f6p0/2625	15.96666667	15.57333333	2.813333333	0.10883	0.88161	0.93414		-2.5894	3.9606E-05	0.00044318	DOWN	-2.4955	1.6019E-06	4.6746E-05	DOWN
transcript_HQ_CoA_transcript1452/f3p0/5303	15.62666667	7.213333333	7.12	-0.96436	4.1282E-08	6.3797E-07		-0.16389	0.33978	0.53147		-1.1401	6.4381E-14	1.1937E-11	DOWN
transcript_HQ_CoA_transcript14559/f5p0/2637	0	0	81.23666667	NA	NA	NA	NA	27.272	2.9381E-12	1.9872E-10	UP	27.276	2.9191E-12	3.9398E-10	UP
transcript_HQ_CoA_transcript14625/f3p0/2628	3.47	2.04	0.673333333	-0.57977	0.36323	0.53862		-1.7592	0.026986	0.086292		-2.3591	3.8779E-05	0.00065004	DOWN
transcript_HQ_CoA_transcript14627/f2p0/2663	1.503333333	0.623333333	8.073333333	-1.1346	0.11808	0.2407		3.6253	0.00010698	0.0010191	UP	2.4764	0.0024015	0.017534	UP
transcript_HQ_CoA_transcript14673/f3p0/2643	12.6	11.04666667	6.033333333	-0.04126	0.91368	0.9525		-1.0065	0.00086613	0.005684	DOWN	-1.0609	0.0010152	0.0090635	DOWN
transcript_HQ_CoA_transcript1471/f3p0/5309	3.14	1.59	14.79333333	-0.82059	0.045891	0.11704		3.0622	3.3374E-15	3.9722E-13	UP	2.2286	8.9595E-11	8.1816E-09	UP
transcript_HQ_CoA_transcript14717/f2p0/2621	18.65666667	12.46666667	42.60666667	-0.42141	0.10163	0.2151		1.6253	1.6155E-20	5.1123E-18	UP	1.1922	1.8007E-06	5.1805E-05	UP
transcript_HQ_CoA_transcript1473/f3p0/5327	9.176666667	6.17	3.88	-0.4059	0.28034	0.44919		-0.82421	2.9161E-06	4.7979E-05		-1.2445	0.0007261	0.0069379	DOWN
transcript_HQ_CoA_transcript14743/f2p0/2630	36.28666667	14.10666667	5.253333333	-1.2383	0.079491	0.17919		-1.5577	0.065589	0.16783		-2.8052	1.1907E-06	3.6856E-05	DOWN
transcript_HQ_CoA_transcript14748/f2p0/2627	2.203333333	1.136666667	0.76	-0.83157	0.31737	0.49004		-0.69691	0.43085	0.62021		-1.5431	0.0010737	0.0094842	DOWN
transcript_HQ_CoA_transcript14751/f3p0/2632	91.97	63.98333333	45.57666667	-0.36986	0.26026	0.42719		-0.64498	0.019954	0.068451		-1.0242	0.0038414	0.02511	DOWN
transcript_HQ_CoA_transcript14774/f2p0/2604	4.403333333	3.693333333	12.92333333	-0.13319	0.84804	0.91418		1.6896	0.0099492	0.039895	UP	1.5482	3.2742E-05	0.00057237	UP
transcript_HQ_CoA_transcript14799/f4p0/2623	0.973333333	0.356666667	6.876666667	-1.3082	0.22041	0.37954		4.1876	0.00068136	0.0046968	UP	2.8651	0.0054834	0.032646	UP
transcript_HQ_CoA_transcript14821/f2p0/2639	15.13666667	8.106666667	4.093333333	-0.72563	0.04518	0.11573		-1.163	0.0045156	0.021657	DOWN	-1.9024	2.9444E-11	2.9859E-09	DOWN
transcript_HQ_CoA_transcript14827/f2p0/2630	9.353333333	3.963333333	3.9	-1.0212	0.19102	0.34304		-0.22287	0.77537	0.87408		-1.2624	0.0097948	0.049541	DOWN
transcript_HQ_CoA_transcript14843/f2p0/2619	39.88	16.21333333	6.236666667	-1.1107	0.21468	0.37289		-1.5069	0.023671	0.078133		-2.6352	0.00046089	0.0048507	DOWN
transcript_HQ_CoA_transcript14905/f2p0/2655	1.43	2.193333333	3.956666667	0.79381	0.06046	0.14542		0.68943	0.05671	0.15075		1.4718	0.00019143	0.0024132	UP

transcript_HQ_CoA_transcript14933/f2p0/2611	7.18	3.45	2.11	-0.91275	0.030439	0.084548		-0.82858	0.10508	0.23678		-1.7542	3.2592E-06	8.4853E-05	DOWN
transcript_HQ_CoA_transcript14945/f2p0/2619	0.16	0.14	4.203333333	-0.11412	0.95862	0.97804		4.8244	1.5752E-09	5.9165E-08	UP	4.7031	2.8195E-07	1.0671E-05	UP
transcript_HQ_CoA_transcript14983/f3p0/2633	0	0.036666667	178.96	2.4733	0.53575	0.69818		12.174	5.3882E-05	0.00057318	UP	28.336	4.0752E-13	6.5043E-11	UP
transcript_HQ_CoA_transcript15005/f9p0/2598	986.4366667	146.44	105.9266667	-2.6288	0.017746	0.055177		-0.59733	0.62042	0.77237		-3.23	0.0027438	0.019393	DOWN
transcript_HQ_CoA_transcript15056/f3p0/2586	17.28666667	11.59333333	3.64	-0.4147	0.10352	0.21791		-1.8207	1.6719E-09	6.2034E-08	DOWN	-2.2466	1.1797E-11	1.3124E-09	DOWN
transcript_HQ_CoA_transcript1508/f2p0/5276	3.44	1.456666667	1.273333333	-1.0681	0.019877	0.060375		-0.35621	0.35004	0.54224		-1.4394	0.0011238	0.0098264	DOWN
transcript_HQ_CoA_transcript15087/f3p0/2599	28.73333333	16.31	10.11666667	-0.67579	0.070363	0.16389		-0.82075	0.026356	0.084691		-1.5093	6.7135E-14	1.2323E-11	DOWN
transcript_HQ_CoA_transcript15111/f2p0/2634	5.133333333	2.363333333	2.293333333	-0.99951	0.35992	0.53523		-0.135	0.90485	0.94896		-1.1491	0.0037594	0.024697	DOWN
transcript_HQ_CoA_transcript15113/f3p0/2621	76.22666667	40.25666667	34.04	-0.76353	0.00076814	0.0040811		-0.39759	0.027301	0.087071		-1.1732	8.8685E-07	2.8359E-05	DOWN
transcript_HQ_CoA_transcript15132/f5p0/2585	5.656666667	2.496666667	2.023333333	-1.0374	0.08654	0.1908		-0.44126	0.50355	0.68065		-1.4916	0.0018112	0.014189	DOWN
transcript_HQ_CoA_transcript15140/f2p0/2607	5.41	3.583333333	1.86	-0.46451	0.43977	0.61331		-1.0756	0.066354	0.16915		-1.5495	0.00078068	0.0073446	DOWN
transcript_HQ_CoA_transcript15170/f4p0/2564	23.83333333	23.63333333	93.44666667	0.13773	0.56622	0.72304		1.8719	0.0021662	0.011824	UP	1.9968	0.00052483	0.0053668	UP
transcript_HQ_CoA_transcript15209/f2p0/2596	1.913333333	12.32333333	0.03	2.7718	0.3585	0.53393		-9.1855	0.0046082	0.021979	DOWN	-6.4045	0.0027967	0.019704	DOWN
transcript_HQ_CoA_transcript15214/f6p0/2575	23.09	33.06	4.666666667	0.66172	0.44969	0.62206		-2.9589	0.00068547	0.0047198	DOWN	-2.3092	1.4462E-07	5.9921E-06	DOWN
transcript_HQ_CoA_transcript15248/f2p0/2579	6.156666667	10.72	23.06333333	0.94903	0.015498	0.049674		0.96796	0.0043673	0.021103		1.9039	2.7212E-09	1.7526E-07	UP
transcript_HQ_CoA_transcript15260/f2p0/2598	8.586666667	8.53	2.14	0.10344	0.89584	0.94298		-2.0966	0.01583	0.057431		-2.0071	3.4823E-05	0.00060017	DOWN
transcript_HQ_CoA_transcript15261/f3p0/2579	2.94	4.94	7.34	0.92466	0.045136	0.11565		0.41008	0.23999	0.42199		1.321	7.3494E-05	0.0011021	UP
transcript_HQ_CoA_transcript15355/f2p0/2584	11.34333333	11.98	2.196666667	0.23857	0.73871	0.8437		-2.583	8.7387E-08	2.1822E-06	DOWN	-2.3605	0.00023418	0.0028278	DOWN
transcript_HQ_CoA_transcript15448/f5p0/2553	8.573333333	11.73	21.05666667	0.6322	0.11905	0.24216		0.67719	0.01846	0.064528		1.2946	0.00068756	0.006663	UP
transcript_HQ_CoA_transcript15450/f2p0/2570	3.85	8.38	24.11666667	1.2879	0.036131	0.096901		1.3787	2.5356E-05	0.00029945	UP	2.6537	2.3183E-06	6.4279E-05	UP
transcript_HQ_CoA_transcript15525/f2p0/2583	7.976666667	12.67	3.73	0.86386	0.13814	0.27111		-1.948	0.00089616	0.0058601	DOWN	-1.1004	0.00086232	0.0079577	DOWN
transcript_HQ_CoA_transcript15548/f2p0/2550	22.21333333	13.90333333	2.106666667	-0.55421	0.67789	0.80516		-2.8118	0.013402	0.050553		-3.384	6.4678E-12	7.8495E-10	DOWN
transcript_HQ_CoA_transcript15558/f2p0/2574	6.04	3.33	1.733333333	-0.73729	0.19126	0.34321		-1.0798	0.038599	0.11201		-1.8269	1.6376E-05	0.00031942	DOWN
transcript_HQ_CoA_transcript15559/f2p0/2598	14.93666667	16.48666667	30.67666667	0.29295	0.48629	0.65545		0.73921	0.0011468	0.0071282		1.0225	0.0090922	0.046976	UP
transcript_HQ_CoA_transcript15623/f3p0/2621	9.983333333	10.45333333	2.08	0.22238	0.5059	0.67231		-2.4906	1.9532E-10	8.95E-09	DOWN	-2.2785	8.4841E-09	4.928E-07	DOWN
transcript_HQ_CoA_transcript15640/f2p0/2549	9.46	6.426666667	4.186666667	-0.42619	0.32088	0.49417		-0.75818	0.039557	0.11395		-1.1939	0.00071899	0.0068943	DOWN
transcript_HQ_CoA_transcript15641/f2p0/2555	7.666666667	7.393333333	57.98666667	0.13278	0.81227	0.89071		2.7827	1.0642E-07	2.6146E-06	UP	2.9041	2.84E-27	4.0099E-24	UP
transcript_HQ_CoA_transcript15643/f2p0/2553	6.94	3.093333333	17.74	-0.97276	0.12966	0.25817		2.3465	0.00016547	0.001464	UP	1.3612	3.4953E-06	8.9355E-05	UP
transcript_HQ_CoA_transcript15696/f2p0/2530	5.84	3.576666667	2.606666667	-0.55983	0.10706	0.22348		-0.57669	0.14184	0.29298		-1.1499	0.0022387	0.016683	DOWN
transcript_HQ_CoA_transcript15698/f3p0/2573	0.953333333	1.32	6.51	0.6333	0.47788	0.64858		2.1634	0.0078261	0.033166	UP	2.7832	0.0041284	0.026486	UP
transcript_HQ_CoA_transcript15745/f3p0/2537	8.446666667	12.53333333	21.83	0.71228	0.13583	0.26771		0.66337	0.14931	0.30366		1.3635	0.0019391	0.014923	UP
transcript_HQ_CoA_transcript1576/f2p0/5240	2.223333333	1.086666667	0.213333333	-0.90751	0.27095	0.43906		-2.4564	0.0029614	0.015303	DOWN	-3.3786	2.5553E-07	9.8535E-06	DOWN
transcript_HQ_CoA_transcript15844/f2p0/2597	5.986666667	3.59	1.976666667	-0.58911	0.052341	0.12991		-1.008	0.0029791	0.015361	DOWN	-1.6095	6.6623E-06	0.00015172	DOWN
transcript_HQ_CoA_transcript15878/f8p0/2515	0.77	1.803333333	8.923333333	1.3947	0.071083	0.16511		2.1794	0.012292	0.047194	UP	3.5596	7.0636E-06	0.00015889	UP
transcript_HQ_CoA_transcript15893/f2p0/2531	2.81	6.253333333	19.38333333	1.3213	0.025017	0.072633		1.4545	0.0069263	0.030119	UP	2.768	2.6053E-09	1.6838E-07	UP
transcript_HQ_CoA_transcript15933/f2p0/2557	4.426666667	7.346666667	9.956666667	0.87816	0.0016653	0.0079573		0.28727	0.34957	0.5417		1.1542	0.00074621	0.0070994	UP
transcript_HQ_CoA_transcript15965/f4p0/2528	60.62666667	40.11	29.60333333	-0.42904	0.079395	0.17911		-0.60183	0.015623	0.056805		-1.0428	4.1788E-11	4.0583E-09	DOWN
transcript_HQ_CoA_transcript15993/f2p0/2508	2.02	5.25	5.046666667	1.5025	0.019324	0.059036		-0.18499	0.76502	0.86798		1.3085	0.00049259	0.0051024	UP
transcript_HQ_CoA_transcript16061/f2p0/2516	1.77	0.803333333	0.533333333	-0.99997	0.18999	0.3418		-0.73737	0.33352	0.52518		-1.7501	0.0045058	0.028411	DOWN
transcript_HQ_CoA_transcript16145/f2p0/2499	60.67666667	31.93666667	26.11666667	-0.79011	0.06261	0.14945		-0.43796	0.080087	0.19414		-1.2371	0.0014036	0.011721	DOWN
transcript_HQ_CoA_transcript16173/f2p0/2499	3.906666667	2.173333333	1.92	-0.69174	0.078629	0.17789		-0.33906	0.31218	0.50372		-1.0409	0.0067895	0.038087	DOWN
transcript_HQ_CoA_transcript16176/f3p0/2502	10.96666667	13.81	38.98	0.48745	0.053924	0.13284		1.3768	0.020142	0.068928		1.851	0.0011249	0.0098264	UP
transcript_HQ_CoA_transcript16204/f2p0/2497	21.99	8.62	7.116666667	-1.2276	0.022919	0.067799		-0.37095	0.5923	0.75234		-1.6112	0.0032057	0.021833	DOWN
transcript_HQ_CoA_transcript16207/f2p0/2512	17.63666667	8.09	2.486666667	-0.9862	0.3475	0.52276		-1.8301	0.083661	0.20016		-2.8285	2.1348E-07	8.4087E-06	DOWN
transcript_HQ_CoA_transcript16276/f4p0/2411	8.833333333	11.73333333	18.76333333	0.58168	0.10512	0.22062		0.52833	0.0061274	0.027512		1.0958	0.0010822	0.0095495	UP
transcript_HQ_CoA_transcript163/f2p0/7437	1.83	2.85	4.303333333	0.78116	0.019769	0.060104		0.45893	0.11944	0.25908		1.2282	1.849E-07	7.4426E-06	UP
transcript_HQ_CoA_transcript16315/f3p0/2498	13.64	15.57333333	2.06	0.32161	0.66216	0.79495		-3.0373	8.4072E-05	0.00083521	DOWN	-2.7244	3.7783E-23	2.4768E-20	DOWN
transcript_HQ_CoA_transcript16346/f2p0/2500	11.43333333	7.413333333	3.56	-0.47248	0.069306	0.16177		-1.2037	0.0011813	0.0072999	DOWN	-1.6871	8.5779E-06	0.00018721	DOWN
transcript_HQ_CoA_transcript16360/f2p0/2506	24.98333333	11.75	9.426666667	-0.94944	0.0027322	0.01203		-0.4618	0.10633	0.23856		-1.421	1.5912E-06	4.6537E-05	DOWN
transcript_HQ_CoA_transcript16365/f2p0/2493	0.213333333	0.176666667	5.85	-0.18588	0.88327	0.93517		4.9203	4.9662E-05	0.00053956	UP	4.7207	9.7566E-05	0.0013875	UP
transcript_HQ_CoA_transcript16374/f2p0/2489	11.44	7.463333333	4.396666667	-0.40233	0.57872	0.7332		-0.94586	0.14792	0.30168		-1.365	0.0046649	0.029075	DOWN
transcript_HQ_CoA_transcript16421/f2p0/2474	0.06	0.34	2.596666667	2.7786	0.18349	0.33369		2.7418	0.012571	0.048062	UP	5.5167	8.55E-09	4.9506E-07	UP
transcript_HQ_CoA_transcript16448/f2p0/2504	46.56333333	21.89	19.93333333	-0.9454	2.6807E-05	0.00022237		-0.27723	0.16977	0.33216		-1.2335	5.2451E-11	5.0143E-09	DOWN
transcript_HQ_CoA_transcript16472/f5p0/2471	3.49	1.203333333	1.253333333	-1.4476	0.22325	0.38301		-0.039562	0.97498	0.98688		-1.4959	0.00038901	0.004225	DOWN
transcript_HQ_CoA_transcript16500/f2p0/2380	104.8833333	39.05	21.34	-1.3166	0.38887	0.56418		-0.98472	0.52187	0.69491		-2.3067	2.7929E-06	7.5168E-05	DOWN
transcript_HQ_CoA_transcript16527/f2p0/2470	8.4	1.793333333	1.226666667	-2.0046	NA	NA	NA	-0.74639	0.66632	0.80512		-2.7713	1.2752E-05	0.00025835	DOWN
transcript_HQ_CoA_transcript16555/f2p0/2466	1.2	1.003333333	8.59	-0.094403	0.87543	0.93073		2.9473	1.2266E-13	1.1075E-11	UP	2.8384	5.3982E-10	4.1114E-08	UP
transcript_HQ_CoA_transcript16571/f3p0/2473	12.85666667	10.82666667	6.08	-0.0885	0.74883	0.85026		-0.97613	0.0017278	0.0098997		-1.0793	0.00083161	0.0077365	DOWN
transcript_HQ_CoA_transcript16575/f2p0/2440	64.93666667	74.78666667	31.53666667	0.34085	0.38552	0.56092		-1.3871	2.5402E-06	4.2481E-05	DOWN	-1.0552	0.0058435	0.03441	DOWN
transcript_HQ_CoA_transcript1658/f4p0/5166	20.25333333	6.323333333	7.916666667	-1.4523	NA	NA	NA	0.11898	NA	NA		-1.3493	0.00025235	0.0030057	DOWN
transcript_HQ_CoA_transcript16582/f5p0/2428	5.983333333	10.19666667	18.98666667	0.94301	0.0090733	0.032525		0.7556	0.0064432	0.028519		1.6849	8.847E-06	0.00019149	UP
transcript_HQ_CoA_transcript16622/f2p0/2499	4.77	3.62	10.90666667	-0.24562	0.49316	0.6613		1.4476	1.0999E-05	0.00014737	UP	1.1916	7.4694E-06	0.00016598	UP
transcript_HQ_CoA_transcript1666/f3p0/5150	5.093333333	7.683333333	16.02333333	0.73102	0.062082	0.14844		0.93617	0.01377	0.051624		1.6549	5.5703E-07	1.9146E-05	UP
transcript_HQ_CoA_transcript1667/f2p0/5153	5.326666667	5.73	2.66	0.28661	0.51815	0.68347		-1.2794	0.00082018	0.0054468	DOWN	-1.0069	0.0026456	0.018822	DOWN
transcript_HQ_CoA_transcript16704/f2p0/2461	0.726666667	2.906666667	0	2.1413	0.23788	0.40094		-8.7872	0.024542	0.080316		-6.6597	0.0066131	0.037464	DOWN
transcript_HQ_CoA_transcript16747/f3p0/2386	7.43	11.50333333	22.79333333	0.794	0.00027442	0.0016864		0.84562	0.087137	0.20652		1.6248	0.00070666	0.0067981	UP
transcript_HQ_CoA_transcript16750/f5p0/2448	19.39	17.85	52.19333333	0.018602	0.95199	0.97394		1.4156	2.5939E-06	4.3143E-05	UP	1.4234	1.6924E-10	1.425E-08	UP
transcript_HQ_CoA_transcript16757/f6p0/2422	17.14333333	27.4	37.8	0.82941	3.1523E-05	0.00025797		0.31223	0.48387	0.66553		1.1263	0.0083933	0.044384	UP
transcript_HQ_CoA_transcript16809/f7p0/2398	1.55	7.03	217.3466667	2.3272	NA	NA	NA	4.8444	0.02705	0.086441		7.1627	0.00013592	0.0018264	UP
transcript_HQ_CoA_transcript16833/f2p0/2443	3.773333333	1.38	1.176666667	-1.322	0.016699	0.052681		-0.37673	0.4716	0.6552		-1.7084	0.00092593	0.0084053	DOWN
transcript_HQ_CoA_transcript16888/f2p0/2390	0	0	10.92	NA	NA	NA	NA	10.442	0.0075273	0.03219	UP	10.448	0.0074951	0.040823	UP
transcript_HQ_CoA_transcript16951/f4p0/2455	22.84333333	14.84333333	6.37	-0.46831	0.27413	0.44257		-1.383	3.0818E-06	4.9944E-05	DOWN	-1.8618	5.9348E-07	2.0096E-05	DOWN
transcript_HQ_CoA_transcript16960/f2p0/2458	6.743333333	4.55	15.97666667	-0.4079	0.31651	0.48913		1.6614	3.1624E-06	5.0935E-05	UP	1.243	8.7901E-06	0.00019049	UP
transcript_HQ_CoA_transcript16971/f3p0/2497	16.70333333	27.00333333	34.82333333	0.87596	0.018662	0.057436		0.19745	0.56124	0.72797		1.06	4.6028E-06	0.0001119	UP
transcript_HQ_CoA_transcript16982/f2p0/2442	8.406666667	5.44	18.07333333	-0.44871	0.46399	0.63565		1.5469	0.0079542	0.033608	UP	1.089	0.0042807	0.027207	UP
transcript_HQ_CoA_transcript16990/f2p0/2440	10.12	4.73	4.726666667	-0.95377	0.0085281	0.030892		-0.1425	0.7087	0.83302		-1.1094	4.2432E-06	0.00010482	DOWN
transcript_HQ_CoA_transcript16995/f2p0/2430	77.99333333	47.69333333	30.87	-0.57106	0.16597	0.3101		-0.77373	0.001804	0.010253		-1.3536	0.00039426	0.0042643	DOWN
transcript_HQ_CoA_transcript170/f3p0/7431	25.21	50.52333333	59.26666667	1.1484	0.085191	0.1886		0.099111	0.87805	0.93387		1.2355	0.00031193	0.0035517	UP
transcript_HQ_CoA_transcript17020/f11p0/2380	1.936666667	0.46	40.76	-1.9556	0.028117	0.079535		6.3881	2.0181E-05	0.00024578	UP	4.4245	0.0058627	0.034501	UP

transcript_HQ_CoA_transcript17028/f2p0/2430	8.086666667	5.953333333	3.133333333	-0.31288	0.68406	0.80856		-1.0553	0.18567	0.35384		-1.3772	3.3487E-07	1.2367E-05	DOWN
transcript_HQ_CoA_transcript17088/f6p0/2422	2.313333333	1.696666667	23.33666667	-0.33169	0.62976	0.77179		3.6978	0.00014773	0.0013315	UP	3.3559	0.0081639	0.043487	UP
transcript_HQ_CoA_transcript17089/f14p0/2337	17.63333333	22.99666667	35.67333333	0.53864	0.0015015	0.0072695		0.4879	0.0077955	0.033067		1.0133	0.00000019	7.5814E-06	UP
transcript_HQ_CoA_transcript17094/f3p0/2432	11.08	6.793333333	4.713333333	-0.56127	0.07026	0.16376		-0.67417	0.03473	0.10384		-1.2481	0.00015323	0.0020119	DOWN
transcript_HQ_CoA_transcript17108/f4p0/2409	22.38	21.92333333	5.106666667	0.0836	0.89616	0.94308		-2.2183	0.0005967	0.0042033	DOWN	-2.1462	1.2252E-08	6.8773E-07	DOWN
transcript_HQ_CoA_transcript17137/f2p0/2423	14.32	0.833333333	2.37	-3.8413	0.056587	0.13794		1.262	0.36898	0.56092		-2.596	0.0019452	0.014958	DOWN
transcript_HQ_CoA_transcript17140/f2p0/2437	2.616666667	1.183333333	7.486666667	-0.97676	0.1264	0.25352		2.5147	2.0275E-12	1.446E-10	UP	1.5189	0.00282	0.019794	UP
transcript_HQ_CoA_transcript17147/f2p0/2420	1.353333333	1.266666667	6.123333333	0.05718	0.94356	0.96888		2.1085	6.1467E-05	0.00063796	UP	2.1564	0.00028475	0.0033122	UP
transcript_HQ_CoA_transcript17188/f2p0/2428	3.95	3.86	8.046666667	0.11023	0.80333	0.88568		0.9178	0.013173	0.049867		1.0189	0.0025925	0.018544	UP
transcript_HQ_CoA_transcript17217/f2p0/2415	2.263333333	1.363333333	0.543333333	-0.62194	0.56544	0.72256		-1.4382	0.21235	0.38845		-2.074	0.00015055	0.0019866	DOWN
transcript_HQ_CoA_transcript17220/f2p0/2470	13.74	7.33	6.786666667	-0.75911	0.0056201	0.021824		-0.24793	0.35229	0.54416		-1.019	1.9758E-05	0.00037272	DOWN
transcript_HQ_CoA_transcript17288/f4p0/2411	7.986666667	8.936666667	30.90666667	0.33155	0.45737	0.6296		1.6415	1.4732E-06	2.6525E-05	UP	1.9594	1.2556E-05	0.00025522	UP
transcript_HQ_CoA_transcript17291/f2p0/2422	4.66	3.133333333	2.05	-0.43046	0.33944	0.51434		-0.74248	0.07431	0.18388		-1.1846	0.0045425	0.028564	DOWN
transcript_HQ_CoA_transcript17295/f3p0/2410	0.546666667	0.693333333	0	0.57148	0.70111	0.82006		-6.7675	0.009137	0.037349	DOWN	-6.2122	0.000994	0.0089261	DOWN
transcript_HQ_CoA_transcript17302/f2p0/2437	3.29	7.236666667	17.80666667	1.241	0.25715	0.42349		1.1948	0.25227	0.436		2.4279	5.6788E-14	1.0858E-11	UP
transcript_HQ_CoA_transcript17430/f2p0/2405	11.43333333	11.99	35.83333333	0.23366	0.57352	0.72854		1.4354	2.4869E-05	0.00029465	UP	1.6562	4.4975E-05	0.00073314	UP
transcript_HQ_CoA_transcript17458/f4p0/2393	11.11666667	9.356666667	26.99	-0.1008	0.64899	0.78544		1.3778	6.2286E-06	9.0606E-05	UP	1.2655	1.2263E-05	0.00025009	UP
transcript_HQ_CoA_transcript17503/f2p0/2399	9.773333333	9.76	2.31	0.16903	0.74047	0.84454		-2.2242	5.3895E-18	9.7807E-16	DOWN	-2.069	9.7379E-05	0.0013875	DOWN
transcript_HQ_CoA_transcript17578/f2p0/2384	0	26.66	1.3	11.911	NA	NA	NA	-4.5493	NA	NA	NA	7.3443	0.0015134	0.012302	UP
transcript_HQ_CoA_transcript1762/f2p0/5121	1.663333333	2.95	4.38	0.98001	0.011303	0.038879		0.42219	0.14202	0.29317		1.3891	0.00010593	0.0014945	UP
transcript_HQ_CoA_transcript17620/f2p0/2401	7.066666667	6.713333333	2.816666667	0.079183	0.86416	0.92369		-1.3971	1.9278E-05	0.00023619	DOWN	-1.332	0.0022452	0.016718	DOWN
transcript_HQ_CoA_transcript17652/f2p0/2380	5.676666667	7.96	13.87	0.6458	0.22117	0.38054		0.66365	0.062165	0.16139		1.2938	0.002394	0.017493	UP
transcript_HQ_CoA_transcript17658/f3p0/2384	51.26333333	78.40666667	10.24333333	0.74242	0.61026	0.75721		-3.0349	0.027714	0.088113		-2.3063	4.0134E-05	0.00066821	DOWN
transcript_HQ_CoA_transcript17722/f2p0/2377	2.276666667	3.986666667	14.56	0.97787	0.011724	0.040087		1.7078	5.1734E-10	2.157E-08	UP	2.674	7.6128E-21	3.6772E-18	UP
transcript_HQ_CoA_transcript17797/f4p0/2363	14.74666667	10.03333333	7.03	-0.37218	0.43409	0.60803		-0.69423	0.21515	0.3918		-1.0816	0.0045095	0.028423	DOWN
transcript_HQ_CoA_transcript17808/f2p0/2390	41.23666667	19.16333333	19.95	-0.95859	1.4995E-05	0.0001334		-0.087853	0.68049	0.81419		-1.0592	5.6631E-07	1.9357E-05	DOWN
transcript_HQ_CoA_transcript17818/f2p0/2377	1.486666667	1.17	14.61333333	-0.20478	0.79373	0.87937		3.5614	0.00026117	0.0021276	UP	3.3457	0.00026138	0.0030992	UP
transcript_HQ_CoA_transcript17822/f3p0/2375	6.483333333	8.6	13.95	0.53147	0.41437	0.58907		0.55967	0.38489	0.57721		1.0822	0.0095188	0.048519	UP
transcript_HQ_CoA_transcript17851/f2p0/2370	6.233333333	8.97	12.98666667	0.70799	0.21012	0.36756		0.35325	0.50828	0.68427		1.0498	0.0011448	0.009945	UP
transcript_HQ_CoA_transcript17881/f2p0/2420	10.58666667	10.25	25.23	0.1176	0.72341	0.83424		1.1484	0.00077902	0.0052226	UP	1.2529	0.001338	0.011292	UP
transcript_HQ_CoA_transcript17884/f3p0/2388	6.59	7.066666667	1.133333333	0.25513	0.5883	0.74129		-2.7622	1.9144E-05	0.00023487	DOWN	-2.5235	4.3346E-06	0.00010651	DOWN
transcript_HQ_CoA_transcript17904/f3p0/2347	9.376666667	7.11	4.506666667	-0.26637	0.50471	0.67135		-0.7897	0.036222	0.10696		-1.0663	0.00048154	0.0050277	DOWN
transcript_HQ_CoA_transcript17987/f2p0/2360	0.223333333	0.446666667	79.67	1.1899	0.22723	0.38791		7.3506	1.0722E-05	0.00014429	UP	8.5231	4.1084E-07	0.0000147	UP
transcript_HQ_CoA_transcript17994/f2p0/2373	12.7	7.343333333	1.156666667	-0.65399	0.07211	0.1669		-2.7897	0.0012133	0.0074362	DOWN	-3.4577	2.0485E-05	0.00038344	DOWN
transcript_HQ_CoA_transcript17999/f4p0/2355	39.55666667	22.30666667	17.19	-0.69221	0.17569	0.32355		-0.49731	0.33494	0.52676		-1.2026	2.339E-10	1.9081E-08	DOWN
transcript_HQ_CoA_transcript18005/f9p0/2335	28.89	29.88	2.423333333	0.20722	0.79562	0.88042		-3.7536	1.7042E-20	4.2789E-18	DOWN	-3.5634	2.1686E-06	6.0757E-05	DOWN
transcript_HQ_CoA_transcript1802/f2p0/5043	5.62	1.97	0.866666667	-1.2994	0.12212	0.24711		-1.3604	0.072317	0.18024		-2.6734	0.00016487	0.0021278	DOWN
transcript_HQ_CoA_transcript18043/f2p0/2346	37.99666667	22.84	12.67666667	-0.58956	0.15281	0.29179		-0.97681	0.013956	0.05214		-1.5802	3.1443E-07	0.00001173	DOWN
transcript_HQ_CoA_transcript18056/f2p0/2350	120.3366667	33.30333333	17.33333333	-1.7341	0.1204	0.24433		-1.0626	0.11743	0.25615		-2.8048	0.0025061	0.018093	DOWN
transcript_HQ_CoA_transcript18095/f3p0/2361	6.5	2.766666667	3.096666667	-1.0877	0.020755	0.062502		0.02583	0.95686	0.97779		-1.0731	0.0010225	0.0091017	DOWN
transcript_HQ_CoA_transcript18136/f3p0/2350	36.52	19.79333333	15.98666667	-0.7489	0.1325	0.26252		-0.44244	0.39463	0.58512		-1.2006	0.00071929	0.0068943	DOWN
transcript_HQ_CoA_transcript18138/f2p0/2358	2.636666667	5.303333333	6.376666667	1.1806	0.015634	0.050002		0.092716	0.82565	0.90499		1.2598	0.0026803	0.019032	UP
transcript_HQ_CoA_transcript1816/f2p0/5076	6.143333333	3.32	2.546666667	-0.72815	0.018711	0.057549		-0.53985	0.071982	0.17985		-1.2799	2.4193E-07	9.4281E-06	DOWN
transcript_HQ_CoA_transcript18274/f3p0/2323	5.43	4.076666667	0.703333333	-0.2693	0.82062	0.89589		-2.6458	0.0041624	0.020323	DOWN	-2.9306	0.0029441	0.020438	DOWN
transcript_HQ_CoA_transcript18285/f2p0/2359	16.83	14.55333333	7.99	-0.016567	0.97656	0.98824		-1.0412	0.055423	0.14817		-1.0738	0.0089785	0.046606	DOWN
transcript_HQ_CoA_transcript18346/f5p0/2326	2.653333333	1.05	0.916666667	-1.1657	0.028427	0.080244		-0.33916	0.52769	0.70016		-1.5191	0.0066395	0.03757	DOWN
transcript_HQ_CoA_transcript18365/f6p0/2320	46.60666667	28.58666667	22.39666667	-0.57969	0.21706	0.37557		-0.4891	0.31971	0.51084		-1.0766	0.0067867	0.038083	DOWN
transcript_HQ_CoA_transcript18385/f3p0/2348	5.893333333	1.75	12.45333333	-1.6321	0.035773	0.096176		2.7048	0.00015778	0.0014059	UP	1.0635	0.0083134	0.044111	UP
transcript_HQ_CoA_transcript18391/f2p0/2329	4.133333333	5.64	0.98	0.60753	0.37843	0.55383		-2.6359	0.00015409	0.0013771	DOWN	-2.0455	0.0024327	0.017656	DOWN
transcript_HQ_CoA_transcript18420/f2p0/2324	7.053333333	3.15	3.15	-1.0285	0.021252	0.063713		-0.13534	0.72919	0.84613		-1.1752	0.0060443	0.035231	DOWN
transcript_HQ_CoA_transcript18484/f5p0/2292	7.74	10.00666667	2.933333333	0.50641	0.38061	0.5559		-1.8881	0.0031157	0.015965	DOWN	-1.3968	0.0013362	0.011282	DOWN
transcript_HQ_CoA_transcript18559/f2p0/2336	10.35	13.53666667	24.09333333	0.54269	0.025155	0.072963		0.68248	0.0038611	0.019096		1.214	2.0262E-06	5.7572E-05	UP
transcript_HQ_CoA_transcript18591/f2p0/2330	5.863333333	8.906666667	13.38666667	0.76267	0.026091	0.075107		0.43694	0.042781	0.12112		1.1886	5.9362E-05	0.00092531	UP
transcript_HQ_CoA_transcript18595/f2p0/2310	0.806666667	31.75	6.54	5.5093	NA	NA	NA	-2.4902	NA	NA	NA	3.0022	0.0004572	0.0048215	UP
transcript_HQ_CoA_transcript18611/f2p0/2339	1.706666667	0.7	0.6	-1.116	0.060255	0.145		-0.38929	0.55696	0.72446		-1.5155	0.0057057	0.033696	DOWN
transcript_HQ_CoA_transcript18714/f3p0/2306	16.30666667	13.93666667	3.556666667	-0.081094	0.7706	0.8642		-2.1157	1.1926E-20	3.1681E-18	DOWN	-2.2095	1.6952E-12	2.3752E-10	DOWN
transcript_HQ_CoA_transcript18769/f6p0/2269	16.18666667	23.57	43.30666667	0.70801	0.00058156	0.0032102		0.72252	1.8578E-06	3.2215E-05		1.4171	3.3472E-16	8.9041E-14	UP
transcript_HQ_CoA_transcript18773/f4p0/2296	25.37333333	10.06	11.05	-1.1454	0.025314	0.073295		-0.046579	0.93042	0.96371		-1.2036	7.5623E-08	3.4226E-06	DOWN
transcript_HQ_CoA_transcript18840/f7p0/2288	1.476666667	1.38	11.32333333	0.013794	0.99016	0.99532		2.9679	0.017943	0.063136		2.9699	0.0012394	0.010603	UP
transcript_HQ_CoA_transcript18886/f4p0/2284	3.273333333	4.116666667	7.07	0.51336	0.25287	0.41871		0.61197	0.1265	0.27032		1.1126	0.00047655	0.0049841	UP
transcript_HQ_CoA_transcript18890/f4p0/2270	115.9533333	87.06666667	50.46666667	-0.27856	0.46508	0.63676		-0.9184	0.0015076	0.0088568		-1.2048	0.0033761	0.022716	DOWN
transcript_HQ_CoA_transcript18901/f2p0/2303	2.14	7.173333333	4.773333333	1.8698	0.0213	0.063847		-0.7162	0.36425	0.55618		1.1428	0.0041124	0.02642	UP
transcript_HQ_CoA_transcript18984/f14p0/2273	44.33666667	29.83	102.8033333	-0.41956	0.091423	0.19883		1.6469	1.0604E-09	4.1265E-08	UP	1.2157	2.8081E-06	7.5355E-05	UP
transcript_HQ_CoA_transcript19003/f7p0/2270	2.706666667	3.023333333	48.57333333	0.34948	0.66843	0.7994		3.914	2.5789E-05	0.00030398	UP	4.2439	0.0018197	0.014231	UP
transcript_HQ_CoA_transcript19057/f5p0/2276	3.253333333	5.546666667	7.24	0.93151	0.016992	0.053358		0.23189	0.39084	0.58146		1.1499	0.0042287	0.026941	UP
transcript_HQ_CoA_transcript19069/f3p0/2273	0.483333333	0.09	29.10333333	-2.3159	0.25385	0.41987		8.2932	1.4261E-05	0.0001833	UP	5.9553	0.003784	0.02483	UP
transcript_HQ_CoA_transcript19136/f3p0/2271	0.073333333	0.183333333	15.02666667	1.3981	0.43219	0.60606		6.258	5.1215E-06	7.6196E-05	UP	7.6404	0.00011042	0.0015436	UP
transcript_HQ_CoA_transcript19147/f2p0/2270	35.88666667	16.36333333	16.31333333	-0.98531	0.01682	0.052962		-0.14456	0.72537	0.8434		-1.1439	2.0494E-05	0.00038344	DOWN
transcript_HQ_CoA_transcript19171/f2p0/2275	12.61666667	17.27	27.07666667	0.60787	0.012457	0.042055		0.49673	0.0074987	0.03209		1.0916	1.0285E-05	0.0002165	UP
transcript_HQ_CoA_transcript19223/f4p0/2262	15.11	5.326666667	1.6	-1.3629	0.12318	0.24854		-1.854	0.0086276	0.035769	DOWN	-3.2304	0.00001042	0.00021857	DOWN
transcript_HQ_CoA_transcript19230/f3p0/2265	13.36333333	10.73666667	6.656666667	-0.18692	0.7314	0.83965		-0.83105	0.16257	0.32279		-1.0266	0.0077068	0.041618	DOWN
transcript_HQ_CoA_transcript19270/f2p0/2253	26.62666667	17.13333333	12.78	-0.47713	0.0070408	0.026334		-0.57499	0.00068977	0.0047458		-1.0654	4.0536E-08	1.9632E-06	DOWN
transcript_HQ_CoA_transcript1928/f3p0/4969	6.25	3.786666667	3.13	-0.59429	0.16292	0.30623		-0.40328	0.32595	0.51686		-1.0076	4.5953E-05	0.00074709	DOWN
transcript_HQ_CoA_transcript19320/f2p0/2292	6.156666667	1.95	2.84	-1.4549	0.055815	0.13629		0.34734	0.65958	0.79983		-1.1189	0.00069144	0.006679	DOWN
transcript_HQ_CoA_transcript19334/f4p0/2246	9.13	10.43666667	25.29666667	0.32752	0.3445	0.51965		1.1515	0.0018159	0.010298	UP	1.4697	1.9332E-06	5.5185E-05	UP
transcript_HQ_CoA_transcript19338/f3p0/2272	15.31	7.786666667	4.746666667	-0.84108	0.099982	0.21231		-0.8383	0.11404	0.25074		-1.6908	5.8136E-15	1.3174E-12	DOWN
transcript_HQ_CoA_transcript19346/f2p0/2265	4.31	8.82	1.666666667	1.1662	0.1547	0.29482		-2.5109	0.0016953	0.0097379	DOWN	-1.3588	0.0067467	0.037931	DOWN
transcript_HQ_CoA_transcript19356/f2p0/2255	14.06666667	24.34333333	28.21666667	0.96619	0.0014959	0.0072483		0.061998	0.79343	0.8862		1.0141	0.00030092	0.0034457	UP
transcript_HQ_CoA_transcript19403/f2p0/2228	0.203333333	1.263333333	2.066666667	2.7639	0.017034	0.053471		0.58604	0.54935	0.71767		3.335	0.0011023	0.0096716	UP

transcript_HQ_CoA_transcript19422/f2p0/2235	2.38	3.02	0.06	0.49809	0.56839	0.72484		-5.8935	7.6338E-06	0.00010723	DOWN	-5.3995	1.5672E-05	0.00030832	DOWN
transcript_HQ_CoA_transcript19424/f2p0/2268	4.803333333	8.533333333	14.64666667	0.98521	0.00013948	0.000942		0.62433	0.025897	0.083584		1.5962	9.905E-08	4.2778E-06	UP
transcript_HQ_CoA_transcript19466/f3p0/2255	0.063333333	0.016666667	8.656666667	-1.8929	0.6033	0.75269		9.0514	0.0061497	0.02758	UP	7.1486	0.0073989	0.040443	UP
transcript_HQ_CoA_transcript19484/f2p0/2248	523.27	291.6033333	238.4033333	-0.70915	0.077313	0.17567		-0.4286	0.19017	0.35966		-1.146	0.0017788	0.014001	DOWN
transcript_HQ_CoA_transcript1951/f2p0/4956	85.57	55.08333333	40.86	-0.47862	0.014527	0.047374		-0.58222	5.8018E-05	0.00060949		-1.0737	4.6349E-08	2.2154E-06	DOWN
transcript_HQ_CoA_transcript1952/f2p0/4960	7.673333333	11.54666667	2.39	0.75973	0.090735	0.19755		-2.4177	9.1877E-24	3.9163E-21	DOWN	-1.6694	0.00051085	0.005253	DOWN
transcript_HQ_CoA_transcript19633/f3p0/2229	4.93	5.006666667	2.03	0.16023	0.81207	0.89054		-1.4263	0.023847	0.078585		-1.2793	0.0055225	0.032826	DOWN
transcript_HQ_CoA_transcript19663/f2p0/2220	2.143333333	3.643333333	5.446666667	0.93752	0.085161	0.18856		0.41982	0.36669	0.55888		1.3442	0.00037842	0.0041493	UP
transcript_HQ_CoA_transcript19739/f2p0/2205	2.843333333	5.766666667	7.276666667	1.2149	0.07413	0.1706		0.14856	0.80322	0.8912		1.3522	0.00025459	0.0030285	UP
transcript_HQ_CoA_transcript19759/f2p0/2226	18.98333333	9.026666667	9.193333333	-0.93467	0.0059686	0.022929		-0.10671	0.77144	0.87109		-1.0521	0.00010917	0.0015296	DOWN
transcript_HQ_CoA_transcript19794/f3p0/2295	9.173333333	15.05333333	29.91	0.88217	0.019999	0.060682		0.86065	0.0063721	0.028321		1.7286	1.8774E-05	0.00035635	UP
transcript_HQ_CoA_transcript19859/f3p0/2226	4.613333333	4.333333333	1.56	0.041542	0.94268	0.96832		-1.596	0.0081803	0.034326	DOWN	-1.5639	0.0009308	0.008437	DOWN
transcript_HQ_CoA_transcript1988/f2p0/4925	19.22333333	10.82	7.9	-0.72199	0.41026	0.58505		-0.57939	0.52254	0.69539		-1.3082	0.0040726	0.02622	DOWN
transcript_HQ_CoA_transcript19900/f4p0/2196	32.97	38.75333333	141.5866667	0.39393	0.22636	0.38691		1.757	0.0057948	0.026356	UP	2.1367	0.00059023	0.0058814	UP
transcript_HQ_CoA_transcript19940/f2p0/2209	0.156666667	2.776666667	104.7766667	4.2681	NA	NA	NA	5.123	0.13932	0.28949		9.3821	0.0014845	0.012144	UP
transcript_HQ_CoA_transcript19941/f7p0/2200	50.44333333	3.023333333	21.83333333	-3.9377	0.015919	0.050712		2.7241	0.10637	0.23859		-1.2256	0.0028567	0.020013	DOWN
transcript_HQ_CoA_transcript20002/f2p0/2209	31.18666667	14.02333333	12.49	-1.0237	0.029407	0.082306		-0.30129	0.47665	0.65989		-1.3349	2.3731E-05	0.00043429	DOWN
transcript_HQ_CoA_transcript20014/f2p0/2222	3.63	7.106666667	16.57666667	1.1064	0.22882	0.38982		1.1133	0.10545	0.23735		2.2046	0.00051786	0.00531	UP
transcript_HQ_CoA_transcript20022/f2p0/2218	6.17	9.613333333	2.703333333	0.78239	0.034338	0.093224		-1.9713	8.6712E-06	0.00011995	DOWN	-1.1978	0.0032981	0.02233	DOWN
transcript_HQ_CoA_transcript2003/f2p0/4914	59.36	41.19666667	26.96666667	-0.36469	0.043439	0.11239		-0.76762	4.4867E-07	9.5623E-06		-1.145	3.0559E-12	4.0645E-10	DOWN
transcript_HQ_CoA_transcript20057/f2p0/2198	0.583333333	0.526666667	47.20333333	-0.015938	0.98915	0.99497		6.4265	0.0012508	0.0076245	UP	6.3961	0.00045766	0.0048222	UP
transcript_HQ_CoA_transcript20067/f2p0/2223	0.023333333	0.113333333	12.56666667	2.8258	0.47347	0.64496		6.7647	0.016497	0.059312		9.5729	0.00029298	0.0033843	UP
transcript_HQ_CoA_transcript20127/f2p0/2194	1.283333333	0.986666667	0.086666667	-0.25693	0.86871	0.92648		-3.6103	0.023244	0.077		-3.8783	0.0024249	0.017626	DOWN
transcript_HQ_CoA_transcript20152/f3p0/2115	4.166666667	6.233333333	23.61	0.75317	0.23833	0.40143		1.7674	0.017722	0.062538		2.5076	5.4151E-05	0.00085685	UP
transcript_HQ_CoA_transcript20234/f2p0/2099	42.49	27.48	20.84333333	-0.5075	0.42865	0.60267		-0.52673	0.41519	0.60508		-1.0442	0.0001959	0.0024579	DOWN
transcript_HQ_CoA_transcript20247/f2p0/2189	6.036666667	2.24	1.543333333	-1.2903	0.075741	0.17304		-0.68427	0.13095	0.27655		-1.9861	0.0052662	0.031744	DOWN
transcript_HQ_CoA_transcript20272/f7p0/2170	0.033333333	0.043333333	42.83	0.62715	0.86481	0.92398		9.8037	0.0005859	0.0041447	UP	10.418	0.0037864	0.02483	UP
transcript_HQ_CoA_transcript20291/f2p0/2154	8.633333333	4.513333333	3.913333333	-0.75654	0.072438	0.16751		-0.3565	0.33534	0.52703		-1.1262	0.0044446	0.028054	DOWN
transcript_HQ_CoA_transcript20300/f3p0/2202	91.70333333	137.1066667	252.1533333	0.76745	0.10346	0.21783		0.72178	2.7769E-05	0.0003246		1.473	0.00084911	0.0078754	UP
transcript_HQ_CoA_transcript20329/f2p0/2175	1.313333333	0.746666667	0.26	-0.6716	0.24808	0.41314		-1.7068	0.016636	0.05974		-2.3889	0.0006994	0.0067459	DOWN
transcript_HQ_CoA_transcript20354/f5p0/2171	5.886666667	13.96333333	0.11	1.3933	0.16552	0.3094		-7.1235	6.0537E-09	1.9814E-07	DOWN	-5.7598	1.3113E-08	7.2937E-07	DOWN
transcript_HQ_CoA_transcript2036/f2p0/4901	2.436666667	2.583333333	5.466666667	0.27429	0.61974	0.76445		0.89275	0.1044	0.23573		1.1546	0.0004943	0.0051172	UP
transcript_HQ_CoA_transcript20367/f2p0/2185	0.166666667	0.423333333	1.85	1.5827	0.33724	0.51224		1.9394	0.0066313	0.02914	UP	3.507	0.0017092	0.013546	UP
transcript_HQ_CoA_transcript20413/f2p0/2183	22.88	15.60333333	10.34666667	-0.41606	0.27354	0.44204		-0.73053	0.059103	0.15553		-1.1565	9.4622E-07	0.0000301	DOWN
transcript_HQ_CoA_transcript20417/f2p0/2215	8.29	24.19666667	24.74666667	1.6653	0.043926	0.11336		-0.083131	0.92138	0.95851		1.5736	2.3633E-06	6.5134E-05	UP
transcript_HQ_CoA_transcript20418/f2p0/2192	23.75333333	13.55666667	9.256666667	-0.68637	0.24439	0.40863		-0.6729	0.27357	0.46096		-1.3679	5.3414E-06	0.000127	DOWN
transcript_HQ_CoA_transcript20426/f19p0/2147	87.75333333	72.3	43.25333333	-0.11965	0.73683	0.84239		-0.90197	0.0033103	0.016812		-1.0332	0.00016421	0.0021274	DOWN
transcript_HQ_CoA_transcript20498/f2p0/2172	18.36333333	12.80333333	6.746666667	-0.37481	0.29158	0.46177		-1.079	0.0023931	0.012844	DOWN	-1.4646	0.00011098	0.0015479	DOWN
transcript_HQ_CoA_transcript20516/f2p0/2169	22.85	22.83	49.35666667	0.15527	0.68366	0.80846		0.97024	4.1897E-05	0.00046513		1.1113	0.00077065	0.0072801	UP
transcript_HQ_CoA_transcript20557/f2p0/2170	7.05	12.13	14.99666667	0.94547	0.0099802	0.035167		0.14658	0.554	0.72147		1.0805	0.0010367	0.009219	UP
transcript_HQ_CoA_transcript20569/f31p0/2137	167.1733333	70.64	50.31333333	-1.1024	0.022855	0.067656		-0.63585	0.0055869	0.025569		-1.7461	0.00018618	0.00236	DOWN
transcript_HQ_CoA_transcript20586/f3p0/2147	10.32333333	3.943333333	2.06	-1.2563	0.077241	0.17553		-1.0574	0.13979	0.29021		-2.3246	6.9097E-07	2.2934E-05	DOWN
transcript_HQ_CoA_transcript20602/f2p0/2160	24.62333333	19.12333333	4.43	-0.22918	0.70762	0.82416		-2.2349	0.00084456	0.0055703	DOWN	-2.4735	3.9111E-12	5.1277E-10	DOWN
transcript_HQ_CoA_transcript2062/f3p0/4888	0.553333333	0.056666667	0.006666667	-3.0887	0.10309	0.21735		-3.3277	0.39138	0.58189		-6.4274	0.00039585	0.004269	DOWN
transcript_HQ_CoA_transcript20675/f2p0/2205	7.513333333	4.276666667	3.463333333	-0.70026	0.27498	0.44354		-0.42357	0.49682	0.67565		-1.1351	0.0083458	0.044197	DOWN
transcript_HQ_CoA_transcript20680/f2p0/2171	8.84	9.79	21.05	0.32926	0.42079	0.59519		0.93909	0.0052315	0.024282		1.2546	3.2709E-06	0.00008504	UP
transcript_HQ_CoA_transcript20685/f2p0/2168	1.926666667	1.563333333	0.48	-0.16932	0.74949	0.85055		-1.8289	0.0052444	0.024323	DOWN	-2.0074	0.0025318	0.018217	DOWN
transcript_HQ_CoA_transcript20687/f2p0/2172	6.013333333	4.603333333	2.216666667	-0.23992	0.6151	0.76056		-1.1847	0.0067844	0.029671	DOWN	-1.4365	0.0011069	0.0096976	DOWN
transcript_HQ_CoA_transcript20715/f2p0/2075	2.82	3.5	6.81	0.4489	0.40241	0.5769		0.81743	0.14274	0.29413		1.2582	0.0090955	0.046976	UP
transcript_HQ_CoA_transcript2083/f2p0/4858	6.54	6.686666667	3.133333333	0.17552	0.60965	0.75671		-1.2311	0.00029147	0.0023268	DOWN	-1.0676	8.7144E-07	2.8013E-05	DOWN
transcript_HQ_CoA_transcript20850/f2p0/2156	14.8	9.233333333	3.666666667	-0.54034	0.28004	0.44878		-1.4389	0.01735	0.061628		-1.992	0.00002559	0.00046277	DOWN
transcript_HQ_CoA_transcript20866/f2p0/2140	15.77333333	8.823333333	6.606666667	-0.69981	0.038158	0.10121		-0.55715	0.028608	0.090158		-1.2675	2.1912E-05	0.00040586	DOWN
transcript_HQ_CoA_transcript2088/f2p0/4853	3.123333333	1.97	1.17	-0.49716	0.15669	0.29783		-0.91201	0.037692	0.11018		-1.4243	0.00012353	0.0016908	DOWN
transcript_HQ_CoA_transcript2095/f5p0/4827	30.16333333	15.88333333	10.35666667	-0.79253	0.065767	0.15524		-0.74487	0.085812	0.20411		-1.5489	3.1014E-19	1.2112E-16	DOWN
transcript_HQ_CoA_transcript21010/f2p0/2136	1.406666667	0.956666667	0.08	-0.37357	0.57426	0.72933		-3.5791	0.00022212	0.0018667	DOWN	-3.9674	7.6854E-06	0.00017037	DOWN
transcript_HQ_CoA_transcript21039/f2p0/2147	0.826666667	1.846666667	2.72	1.3476	0.044546	0.11454		0.39509	0.38995	0.58074		1.7321	0.0012933	0.010989	UP
transcript_HQ_CoA_transcript21083/f4p0/2120	35.54	9.02	8.826666667	-1.8448	0.17447	0.32183		-0.1362	0.90595	0.94936		-1.9906	0.0031728	0.021641	DOWN
transcript_HQ_CoA_transcript21086/f2p0/2139	9.966666667	12.77333333	23.67333333	0.52329	0.2048	0.36014		0.73758	0.00060202	0.0042342		1.2474	0.0013237	0.011202	UP
transcript_HQ_CoA_transcript21100/f2p0/2128	0.556666667	0.263333333	13.38	-0.91199	0.37609	0.55133		5.5409	6.4859E-37	8.4914E-34	UP	4.5972	9.6616E-25	8.3572E-22	UP
transcript_HQ_CoA_transcript21117/f2p0/2157	26.35333333	22.07666667	11.09333333	-0.11181	0.80686	0.88761		-1.1372	0.0035641	0.017884	DOWN	-1.258	0.0015018	0.012246	DOWN
transcript_HQ_CoA_transcript21121/f2p0/2130	8.953333333	12.46666667	3.52	0.61205	0.33416	0.50866		-1.9657	0.00041375	0.0031209	DOWN	-1.3625	0.0033288	0.022464	DOWN
transcript_HQ_CoA_transcript21131/f8p0/2080	18.73333333	24.75333333	40.32333333	0.55879	0.048058	0.12138		0.56372	0.021691	0.073055		1.1091	6.7927E-05	0.0010364	UP
transcript_HQ_CoA_transcript21175/f2p0/2130	10.80666667	9.366666667	1.02	0.015891	0.99312	0.99669		-3.3902	NA	NA	NA	-3.3928	1.6597E-09	1.0998E-07	DOWN
transcript_HQ_CoA_transcript2120/f2p0/4858	1.38	1.523333333	3.176666667	0.28261	0.4577	0.62983		0.93183	0.015681	0.057005		1.2008	0.00056363	0.0056625	UP
transcript_HQ_CoA_transcript21209/f2p0/2121	4.57	1.786666667	1.466666667	-1.2143	0.058638	0.14186		-0.37629	0.54939	0.71769		-1.6034	0.0090166	0.046738	DOWN
transcript_HQ_CoA_transcript21218/f2p0/2105	7.376666667	26.26666667	0.73	1.9783	0.069049	0.16128		-5.2704	8.14E-09	2.5902E-07	DOWN	-3.3066	0.00093901	0.0084988	DOWN
transcript_HQ_CoA_transcript21234/f2p0/2122	2.1	2.443333333	5.596666667	0.38114	0.35443	0.53021		1.0561	0.034181	0.10269		1.4239	0.0047086	0.029267	UP
transcript_HQ_CoA_transcript21251/f2p0/2114	8.483333333	4.836666667	2.17	-0.65311	0.077055	0.1752		-1.3096	8.8348E-05	0.00086967	DOWN	-1.9754	2.1301E-08	1.1108E-06	DOWN
transcript_HQ_CoA_transcript21256/f4p0/2092	18.30666667	19.32333333	42.11333333	0.23719	0.44351	0.61696		0.9696	0.00012617	0.0011639		1.1943	6.1649E-06	0.00014288	UP
transcript_HQ_CoA_transcript21287/f5p0/2119	12.39333333	10.41666667	6.176666667	-0.1261	0.8012	0.88403		-0.87273	0.061959	0.16095		-1.0079	0.007941	0.04259	DOWN
transcript_HQ_CoA_transcript21305/f10p0/2071	1.92	1.07	34.96666667	-0.64689	0.36968	0.54522		4.9044	0.0011057	0.006931	UP	4.2409	0.0046611	0.029061	UP
transcript_HQ_CoA_transcript21313/f2p0/2120	8.563333333	9.11	19.86666667	0.23606	0.58001	0.73428		0.98211	2.0029E-06	3.4471E-05		1.2076	0.0023066	0.017016	UP
transcript_HQ_CoA_transcript21324/f3p0/2128	1.953333333	2.016666667	8.443333333	0.27739	0.88635	0.93699		1.8395	NA	NA	NA	2.1025	2.0147E-06	5.7333E-05	UP
transcript_HQ_CoA_transcript2134/f2p0/4844	4.55	7.023333333	1.15	0.75586	0.12894	0.25716		-2.7518	4.3409E-20	1.0609E-17	DOWN	-2.0025	0.0001188	0.0016408	DOWN
transcript_HQ_CoA_transcript2141/f3p0/4832	11	13.96333333	35.43	0.50648	0.06053	0.14557		1.1833	0.0021247	0.011639	UP	1.6777	3.2319E-05	0.00056712	UP
transcript_HQ_CoA_transcript21451/f2p0/2101	15.62666667	14.34	6.51	0.051328	0.89077	0.9396		-1.3031	0.00010757	0.0010232	DOWN	-1.2674	0.0008053	0.0075338	DOWN
transcript_HQ_CoA_transcript21461/f6p0/2015	12.46	7.06	5.476666667	-0.66549	0.0043748	0.017746		-0.5089	0.034703	0.1038		-1.1861	1.0884E-07	4.6678E-06	DOWN
transcript_HQ_CoA_transcript21484/f2p0/2092	7.226666667	4.466666667	3.31	-0.54345	0.068803	0.16081		-0.57166	0.08179	0.19715		-1.1267	0.00045428	0.0047982	DOWN
transcript_HQ_CoA_transcript21485/f2p0/2104	3.726666667	5.413333333	13.22666667	0.72244	0.36172	0.5372		1.0939	0.093199	0.21689		1.8068	8.6222E-05	0.001256	UP

transcript_HQ_CoA_transcript21540/f2p0/2094	1.656666667	1.236666667	0.4	-0.28376	0.74357	0.84681		-1.7528	0.032458	0.098793		-2.0492	0.0083898	0.044384	DOWN
transcript_HQ_CoA_transcript2157/f3p0/4846	11.77333333	13.27666667	5.683333333	0.33468	0.39816	0.57276		-1.3889	0.00007468	0.00075458	DOWN	-1.0661	0.00031197	0.0035517	DOWN
transcript_HQ_CoA_transcript21571/f2p0/2098	5.056666667	6.353333333	205.43	0.47322	0.44321	0.61668		4.9323	0.00052292	0.0037794	UP	5.3921	0.00032802	0.003696	UP
transcript_HQ_CoA_transcript21640/f5p0/2011	38.05	73.47333333	5.903333333	1.0775	0.3557	0.53128		-3.7591	6.229E-07	1.2714E-05	DOWN	-2.6924	1.5264E-05	0.00030158	DOWN
transcript_HQ_CoA_transcript21643/f3p0/2096	18.84666667	27.33	43.44333333	0.69707	0.03542	0.095487		0.5114	0.014645	0.054061		1.1961	3.5586E-05	0.00060837	UP
transcript_HQ_CoA_transcript21679/f5p0/2079	200.1433333	44.12333333	16.68666667	-2.0619	0.054273	0.13345		-1.5151	0.015744	0.057177		-3.586	0.00012857	0.0017494	DOWN
transcript_HQ_CoA_transcript21693/f2p0/2116	1.733333333	1.026666667	8.61	-0.56771	0.43161	0.60558		2.8804	2.8664E-08	8.0704E-07	UP	2.302	1.4499E-10	1.2553E-08	UP
transcript_HQ_CoA_transcript21729/f2p0/2090	2.816666667	2.693333333	7.896666667	0.098113	0.86857	0.92648		1.3834	0.00076603	0.0051508	UP	1.4733	0.0016957	0.013462	UP
transcript_HQ_CoA_transcript21780/f2p0/2074	15.53	25.75333333	32.96	0.88573	3.3346E-05	0.00027034		0.20548	0.15242	0.30806		1.0792	4.9385E-07	1.7294E-05	UP
transcript_HQ_CoA_transcript21788/f2p0/2082	12.66	3.39	2.93	-1.6905	0.082156	0.18371		-0.40735	0.6893	0.82056		-2.1109	1.3315E-07	5.5293E-06	DOWN
transcript_HQ_CoA_transcript2185/f2p0/4857	3.583333333	7.563333333	8.42	1.2727	0.029863	0.083239		-0.017996	0.96988	0.9844		1.2402	0.0048499	0.029822	UP
transcript_HQ_CoA_transcript21870/f2p0/2071	6.353333333	3.336666667	61.70333333	-0.76295	0.15571	0.29635		4.1277	0.00073723	0.0049899	UP	3.3481	0.0095448	0.048611	UP
transcript_HQ_CoA_transcript21897/f5p0/2037	22.76666667	28.25	50.30666667	0.46157	0.034434	0.093429		0.69133	0.00047422	0.0035006		1.1412	6.8351E-10	5.0183E-08	UP
transcript_HQ_CoA_transcript21903/f5p0/2060	489.72	236.3333333	186.8066667	-0.93652	0.067057	0.15774		-0.47535	0.14698	0.30042		-1.4191	0.0017734	0.013971	DOWN
transcript_HQ_CoA_transcript22052/f2p0/2065	9.763333333	12.72666667	35.71666667	0.54484	0.21726	0.37584		1.3441	0.00013917	0.0012615	UP	1.8757	1.4774E-05	0.00029317	UP
transcript_HQ_CoA_transcript22054/f2p0/2052	5.356666667	3.106666667	11.62	-0.64469	0.090446	0.19715		1.7629	4.6654E-09	1.5748E-07	UP	1.1052	8.1306E-06	0.00017959	UP
transcript_HQ_CoA_transcript22115/f3p0/2050	40.05333333	59.68333333	85.22666667	0.73377	0.018351	0.056682		0.36325	0.34701	0.53925		1.0806	0.0046887	0.029173	UP
transcript_HQ_CoA_transcript22127/f3p0/2091	39.60666667	43.04666667	82.20333333	0.26192	0.31636	0.48902		0.78576	0.015466	0.05638		1.0386	0.0023671	0.017332	UP
transcript_HQ_CoA_transcript22132/f2p0/2055	4.28	2.573333333	12.18333333	-0.62601	0.39086	0.56595		2.1158	0.0001124	0.0010609	UP	1.4826	0.0046108	0.028817	UP
transcript_HQ_CoA_transcript22190/f3p0/2042	6.47	3.666666667	3.086666667	-0.68252	0.14127	0.27564		-0.36616	0.45729	0.64365		-1.0609	0.0090489	0.046813	DOWN
transcript_HQ_CoA_transcript22197/f2p0/2043	0.36	3.676666667	3.35	3.4432	0.016902	0.053131		-0.2358	0.75798	0.86411		3.2005	0.0081707	0.043496	UP
transcript_HQ_CoA_transcript22227/f2p0/2045	0.143333333	0.83	1.476666667	2.7699	0.040968	0.10717		0.64952	0.35221	0.54412		3.3975	0.00090582	0.0082595	UP
transcript_HQ_CoA_transcript22231/f61p0/1911	7.116666667	8.186666667	904.16	0.35997	0.53099	0.6941		6.6911	0.00010362	0.00099389	UP	7.0379	2.9834E-05	0.00052857	UP
transcript_HQ_CoA_transcript22240/f3p0/2018	6.47	10.25333333	14.05	0.82962	0.051689	0.12868		0.30958	0.19626	0.36789		1.1233	0.0048458	0.029822	UP
transcript_HQ_CoA_transcript22283/f2p0/2050	0.566666667	0.623333333	14.78333333	0.36824	0.72334	0.83424		4.3636	9.4924E-11	4.6396E-09	UP	4.7184	1.4027E-20	6.2794E-18	UP
transcript_HQ_CoA_transcript22339/f3p0/2045	5.28	4.856666667	2.493333333	0.023721	0.95301	0.97443		-1.1157	0.008196	0.034369	DOWN	-1.1042	0.0070308	0.038999	DOWN
transcript_HQ_CoA_transcript22390/f2p0/2033	13.24333333	3.603333333	3.27	-1.6444	0.43671	0.61044		-0.35085	0.84068	0.91317		-2.014	0.0051068	0.031007	DOWN
transcript_HQ_CoA_transcript22449/f4p0/2033	2.5	5.32	5.22	1.2932	0.094199	0.20289		-0.22238	0.75621	0.86288		1.0576	0.0037986	0.024892	UP
transcript_HQ_CoA_transcript22458/f2p0/1852	91.72666667	17.25666667	5.276666667	-2.3103	0.080363	0.18072		-1.7974	0.10136	0.23092		-4.1179	0.00079484	0.0074549	DOWN
transcript_HQ_CoA_transcript2249/f2p0/4781	4.806666667	2.476666667	0.713333333	-0.81433	0.018179	0.056191		-1.9355	1.9804E-06	3.4148E-05	DOWN	-2.7592	4.7477E-22	2.7232E-19	DOWN
transcript_HQ_CoA_transcript22492/f2p0/2020	1.476666667	2.793333333	4.086666667	1.0369	0.070585	0.16422		0.41654	0.37371	0.56581		1.4433	0.00055813	0.0056319	UP
transcript_HQ_CoA_transcript22502/f2p0/2031	14.75666667	9.626666667	3.923333333	-0.48446	0.24412	0.40822		-1.4303	0.0016607	0.0095808	DOWN	-1.9233	4.2373E-11	4.0935E-09	DOWN
transcript_HQ_CoA_transcript22525/f2p0/2048	16.23	17.62	4.533333333	0.28117	0.48808	0.65702		-2.1028	0.0028889	0.014954	DOWN	-1.8351	0.0066923	0.037785	DOWN
transcript_HQ_CoA_transcript22572/f2p0/2011	0	10.76666667	102.8133333	24.205	NA	NA	NA	3.1428	0.37341	0.56554		27.161	3.5938E-12	4.7456E-10	UP
transcript_HQ_CoA_transcript22608/f2p0/2016	27.22	14.83666667	12.63666667	-0.70874	0.0095377	0.033864		-0.39265	0.16403	0.32461		-1.113	4.8727E-09	3.0114E-07	DOWN
transcript_HQ_CoA_transcript22616/f2p0/2014	10.77	15.69666667	1.926666667	0.69446	0.090429	0.19714		-3.173	2.0328E-16	2.7805E-14	DOWN	-2.4918	5.709E-10	4.3123E-08	DOWN
transcript_HQ_CoA_transcript22642/f3p0/1948	15.89666667	26.69	36.89666667	0.90387	0.001028	0.0052717		0.31677	0.10157	0.23121		1.2081	1.2768E-07	5.3507E-06	UP
transcript_HQ_CoA_transcript22737/f2p0/2000	14.89	8.123333333	7.49	-0.72608	0.014571	0.047485		-0.26921	0.29512	0.48438		-1.0076	0.00057037	0.0057115	DOWN
transcript_HQ_CoA_transcript22745/f2p0/1992	45.77	65.87333333	117.22	0.70525	0.13127	0.26065		0.66109	0.12289	0.26464		1.3552	8.7399E-06	0.00018962	UP
transcript_HQ_CoA_transcript22781/f4p0/1996	28.12333333	14.18333333	2.17	-0.84654	0.27565	0.44419		-2.8252	0.0014743	0.0087166	DOWN	-3.6898	1.1936E-06	0.00003688	DOWN
transcript_HQ_CoA_transcript22815/f2p0/2009	15.47666667	6.236666667	3.393333333	-1.1428	0.015675	0.050124		-1.0402	0.037949	0.11067		-2.193	7.5893E-08	3.4226E-06	DOWN
transcript_HQ_CoA_transcript22827/f7p0/1964	59.21333333	35.73666667	1.72	-0.59806	0.81176	0.89049		-4.5195	0.027033	0.086412		-5.1317	0.0011344	0.0098826	DOWN
transcript_HQ_CoA_transcript22846/f3p0/1992	49.25666667	90.29333333	107.2233333	0.99527	0.10493	0.22034		0.12656	0.83717	0.91089		1.1117	1.5339E-06	0.00004512	UP
transcript_HQ_CoA_transcript22874/f2p0/2000	1.15	1.576666667	10.92	0.59181	0.36283	0.53825		2.689	0.00020716	0.0017743	UP	3.2696	4.3047E-06	0.00010591	UP
transcript_HQ_CoA_transcript22878/f3p0/2005	9.876666667	7.226666667	4.003333333	-0.31397	0.41278	0.58766		-0.97694	0.029954	0.093088		-1.3045	0.00018846	0.0023818	DOWN
transcript_HQ_CoA_transcript22989/f3p0/2005	20.47333333	9.533333333	8.733333333	-0.95027	0.00017585	0.0011519		-0.26883	0.32683	0.5178		-1.2315	5.9255E-09	3.5544E-07	DOWN
transcript_HQ_CoA_transcript23037/f4p0/1892	1.47	2.31	328.7733333	0.81441	0.22533	0.38561		7.0696	0.0001376	0.0012504	UP	7.872	1.4482E-05	0.00028894	UP
transcript_HQ_CoA_transcript23081/f3p0/1996	23.64333333	13.50666667	11.05333333	-0.65119	0.021181	0.063562		-0.44029	0.041619	0.11868		-1.1028	5.8893E-05	0.0009192	DOWN
transcript_HQ_CoA_transcript23117/f2p0/2032	16.85333333	16.85666667	37.54666667	0.15902	0.39482	0.56977		0.99825	0.00001217	0.00016083		1.1452	3.426E-07	1.2602E-05	UP
transcript_HQ_CoA_transcript2312/f2p0/4744	2.716666667	1.763333333	1.046666667	-0.45124	0.26744	0.43541		-0.90675	0.039437	0.11374		-1.3726	0.0018173	0.014225	DOWN
transcript_HQ_CoA_transcript23126/f2p0/1980	29.49333333	15.69	14.01333333	-0.77096	0.034744	0.094033		-0.29907	0.4229	0.6122		-1.0821	1.5938E-09	1.0638E-07	DOWN
transcript_HQ_CoA_transcript2313/f2p0/4744	7.12	11.60333333	3.15	0.8625	0.025643	0.074071		-2.018	1.3642E-10	6.4612E-09	DOWN	-1.1703	0.00062175	0.0061455	DOWN
transcript_HQ_CoA_transcript23136/f2p0/1988	19.95666667	11.74666667	6.03	-0.59868	0.13587	0.26771		-1.1089	0.00017983	0.0015764	DOWN	-1.7225	0.00000206	5.8261E-05	DOWN
transcript_HQ_CoA_transcript23141/f2p0/1984	3.323333333	2.433333333	7.143333333	-0.2964	0.59138	0.74363		1.425	0.0013092	0.007925	UP	1.1162	0.0042736	0.02718	UP
transcript_HQ_CoA_transcript23143/f3p0/1983	43.10333333	20.65666667	16.30666667	-0.91567	0.00032079	0.0019324		-0.48438	0.071406	0.17879		-1.4112	4.7606E-14	9.2959E-12	DOWN
transcript_HQ_CoA_transcript23178/f2p0/1977	42.23	37.21	85.9	-0.0091201	0.97849	0.98942		1.04	0.0014152	0.0084327	UP	1.0176	0.00014793	0.0019562	UP
transcript_HQ_CoA_transcript23179/f2p0/1980	0.1	0.03	4.563333333	-1.4898	0.55037	0.71038		6.9211	5.0302E-08	1.346E-06	UP	5.3666	1.1613E-09	8.015E-08	UP
transcript_HQ_CoA_transcript2322/f2p0/4781	7.026666667	6.11	3.456666667	-0.056021	0.86423	0.92369		-0.96343	0.0012143	0.0074363		-1.0327	0.00028884	0.0033513	DOWN
transcript_HQ_CoA_transcript2328/f4p0/4727	9.253333333	5.963333333	4.363333333	-0.49012	0.13996	0.27382		-0.5864	0.038406	0.11168		-1.0883	6.6972E-05	0.0010235	DOWN
transcript_HQ_CoA_transcript23300/f2p0/2006	8.673333333	8.52	30.48666667	0.17327	0.75736	0.85565		1.6549	0.00051265	0.0037243	UP	1.8142	4.7394E-09	2.9489E-07	UP
transcript_HQ_CoA_transcript23310/f3p0/1998	0.003333333	0	1.526666667	NA	NA	NA	NA	7.2038	0.0043417	0.021019	UP	7.2053	0.0022877	0.016918	UP
transcript_HQ_CoA_transcript23316/f2p0/1985	5.876666667	5.813333333	2.81	0.12489	0.76189	0.85886		-1.1856	0.0017836	0.010168	DOWN	-1.0714	0.0038328	0.02508	DOWN
transcript_HQ_CoA_transcript2344/f2p0/4721	0.013333333	0.233333333	0.43	4.1957	0.045217	0.11581		0.74167	0.55136	0.71917		4.9311	0.00031208	0.0035517	UP
transcript_HQ_CoA_transcript23483/f3p0/1956	80.89333333	124.5066667	179.9566667	0.78756	0.00029718	0.0018038		0.37382	0.27495	0.4623		1.1458	0.00031117	0.0035498	UP
transcript_HQ_CoA_transcript23507/f2p0/1957	16.29666667	27.46	45.47666667	0.88394	0.049238	0.12375		0.60332	0.19668	0.36835		1.4774	2.9682E-08	1.5009E-06	UP
transcript_HQ_CoA_transcript23575/f2p0/1953	5.28	1.62	19.33333333	-1.5828	0.079487	0.17919		3.4583	2.1564E-05	0.00026108	UP	1.863	8.2415E-05	0.0012107	UP
transcript_HQ_CoA_transcript23597/f2p0/1952	0.966666667	1.693333333	3.01	0.96346	0.20735	0.36363		0.65654	0.22678	0.40601		1.6095	0.0038298	0.02507	UP
transcript_HQ_CoA_transcript23628/f2p0/1943	0.746666667	0.626666667	41.58666667	-0.022007	0.99018	0.99532		5.8842	0.0065229	0.028767	UP	5.8483	3.1939E-05	0.000561	UP
transcript_HQ_CoA_transcript23639/f3p0/1952	2.1	4.456666667	0.16	1.3276	NA	NA	NA	-5.1777	NA	NA	NA	-3.8526	0.00028871	0.0033513	DOWN
transcript_HQ_CoA_transcript23640/f5p0/1877	14.63	12.58666667	4.81	-0.098335	0.90549	0.94801		-1.4899	0.080289	0.19435		-1.6017	0.00011268	0.0015693	DOWN
transcript_HQ_CoA_transcript23683/f2p0/1942	23.18	9.813333333	58.67	-1.0769	0.085615	0.18929		2.3978	5.9941E-08	1.5763E-06	UP	1.3125	0.008224	0.043741	UP
transcript_HQ_CoA_transcript23742/f4p0/1916	0	0.016666667	58.14	0.98103	0.81	0.88943		11.528	0.0012479	0.0076091	UP	26.286	1.7176E-11	1.833E-09	UP
transcript_HQ_CoA_transcript23760/f2p0/1938	22.92	27.65333333	11.03333333	0.40264	0.43583	0.60963		-1.4506	0.0044942	0.021576	DOWN	-1.0599	2.3109E-05	0.00042502	DOWN
transcript_HQ_CoA_transcript238/f2p0/7085	1.93	1.926666667	0.26	0.146	0.86386	0.92367		-2.9971	0.00006314	0.00065273	DOWN	-2.8679	0.00004617	0.0007493	DOWN
transcript_HQ_CoA_transcript23852/f2p0/1939	4.326666667	9.243333333	0.103333333	1.2842	0.3036	0.475		-6.6643	3.2359E-19	6.7398E-17	DOWN	-5.4323	0.0014298	0.011864	DOWN
transcript_HQ_CoA_transcript23864/f2p0/1928	24.09	14.72	10.47666667	-0.53682	0.073789	0.16999		-0.65594	0.054248	0.14551		-1.2057	0.00038819	0.0042211	DOWN
transcript_HQ_CoA_transcript23900/f2p0/1921	3.99	1.77	1.74	-1.0346	0.030234	0.084054		-0.1611	0.73953	0.85294		-1.2067	0.0018476	0.014358	DOWN
transcript_HQ_CoA_transcript23920/f2p0/1926	9.87	10.43666667	37.06333333	0.26567	0.69287	0.81455		1.658	0.011767	0.045638	UP	1.9123	5.9712E-06	0.00013926	UP
transcript_HQ_CoA_transcript2393/f3p0/4693	6.416666667	10.46333333	16.29333333	0.87373	0.0002187	0.0013902		0.47746	0.015539	0.056557		1.3378	5.4437E-13	8.3266E-11	UP

transcript_HQ_CoA_transcript23935/f2p0/1917	19.99333333	10.01666667	5.623333333	-0.86513	0.086821	0.19131		-0.97032	0.023311	0.077165		-1.8447	7.3187E-06	0.00016362	DOWN
transcript_HQ_CoA_transcript23948/f3p0/1931	47.01666667	25.32666667	21.68666667	-0.74676	0.00047732	0.0027143		-0.36181	0.098948	0.22676		-1.12	2.9344E-10	2.3071E-08	DOWN
transcript_HQ_CoA_transcript24020/f3p0/1919	11.08333333	9.963333333	24.67333333	-0.015452	0.96713	0.98293		1.1934	0.011239	0.043961	UP	1.1667	0.0059616	0.034904	UP
transcript_HQ_CoA_transcript24094/f2p0/1910	15.13333333	9.64	6.913333333	-0.49878	0.018671	0.057436		-0.62295	0.0035176	0.017708		-1.1337	1.6743E-07	6.8292E-06	DOWN
transcript_HQ_CoA_transcript24186/f3p0/1954	0	0	42.92666667	NA	NA	NA	NA	12.079	0.00199	0.01106	UP	12.084	0.001981	0.015163	UP
transcript_HQ_CoA_transcript24200/f2p0/1921	18.65666667	16.64	6.296666667	0.03638	0.95537	0.97571		-1.5712	0.0042989	0.020862	DOWN	-1.5521	0.0086597	0.045404	DOWN
transcript_HQ_CoA_transcript24244/f2p0/1884	4.596666667	2.52	1.736666667	-0.73613	0.20304	0.35819		-0.67253	0.12851	0.27312		-1.4176	0.0093873	0.048036	DOWN
transcript_HQ_CoA_transcript24258/f2p0/1897	12.23	10.58666667	24.66333333	-0.041874	0.91659	0.95423		1.0709	0.0077336	0.032873	UP	1.0175	0.0019374	0.014921	UP
transcript_HQ_CoA_transcript24282/f2p0/1891	8.583333333	4.45	1.433333333	-0.8003	0.19859	0.35235		-1.7881	0.011183	0.043798	DOWN	-2.6012	9.4423E-06	0.0002006	DOWN
transcript_HQ_CoA_transcript24345/f3p0/1890	4.403333333	5.186666667	9.373333333	0.39757	0.17178	0.31824		0.69974	0.0064143	0.028446		1.0849	0.00016168	0.0021018	UP
transcript_HQ_CoA_transcript24427/f2p0/1911	0	0.04	20.51	2.4282	0.54348	0.70513		8.5693	0.013815	0.051718		10.983	0.0049388	0.030247	UP
transcript_HQ_CoA_transcript24428/f2p0/1902	0.813333333	3.33	10	2.1631	0.036661	0.098058		1.4816	0.098831	0.22652		3.6353	1.6713E-17	5.2855E-15	UP
transcript_HQ_CoA_transcript24435/f3p0/1876	9.973333333	7.57	4.433333333	-0.26787	0.52914	0.69244		-0.90341	0.026989	0.086292		-1.1819	0.00005841	0.00091243	DOWN
transcript_HQ_CoA_transcript2444/f2p0/4704	2.146666667	2.026666667	0.9	0.11115	0.85492	0.91833		-1.3621	0.042439	0.12043		-1.2677	0.0030363	0.020968	DOWN
transcript_HQ_CoA_transcript24451/f2p0/1878	7.963333333	7.253333333	2.443333333	0.027713	0.95144	0.97354		-1.7449	1.3881E-05	0.00017893	DOWN	-1.729	5.9385E-05	0.00092531	DOWN
transcript_HQ_CoA_transcript245/f2p0/7047	1.836666667	2.563333333	0.676666667	0.60648	0.30057	0.47164		-2.0488	0.00081251	0.0054037	DOWN	-1.4553	0.00080906	0.007563	DOWN
transcript_HQ_CoA_transcript24503/f2p0/1883	5.953333333	7.803333333	14.02666667	0.55912	0.11003	0.22805		0.68919	0.038982	0.11277		1.235	1.1697E-05	0.00024095	UP
transcript_HQ_CoA_transcript24552/f4p0/1858	5.653333333	2.706666667	2	-0.93772	0.11428	0.23495		-0.55833	0.40045	0.59125		-1.5037	0.004641	0.028965	DOWN
transcript_HQ_CoA_transcript24574/f2p0/1880	6.94	5.193333333	15.16	-0.24673	0.56429	0.7217		1.3685	0.0051457	0.023968	UP	1.1116	0.0072557	0.039837	UP
transcript_HQ_CoA_transcript24618/f4p0/1854	22.53666667	12.49	9.666666667	-0.69301	0.1803	0.32987		-0.50072	0.22717	0.40639		-1.2092	0.0063759	0.036572	DOWN
transcript_HQ_CoA_transcript24641/f2p0/1900	2.23	4.81	7.07	1.2728	0.051949	0.12911		0.42217	0.45644	0.64291		1.6777	0.0032864	0.022269	UP
transcript_HQ_CoA_transcript24822/f3p0/1856	7.34	6.38	17.26	-0.058197	0.87975	0.93287		1.2904	7.6091E-06	0.00010712	UP	1.2219	0.0013709	0.011495	UP
transcript_HQ_CoA_transcript24857/f2p0/1847	5.253333333	5.713333333	1.38	0.24863	0.73866	0.8437		-2.1849	0.0017794	0.010151	DOWN	-1.947	0.0017217	0.013633	DOWN
transcript_HQ_CoA_transcript24861/f2p0/1850	4.153333333	5.17	22.07	0.45431	0.29036	0.46059		2.0004	0.0078419	0.033218	UP	2.4411	0.00056174	0.005651	UP
transcript_HQ_CoA_transcript24936/f2p0/1844	12.44666667	16.57333333	32.69666667	0.55027	0.48121	0.65147		0.86617	0.24039	0.42244		1.4033	3.0781E-05	0.00054326	UP
transcript_HQ_CoA_transcript24974/f2p0/1835	2.666666667	2.246666667	0.97	-0.12415	0.817	0.89354		-1.3502	0.028423	0.089713		-1.4859	0.0078155	0.042057	DOWN
transcript_HQ_CoA_transcript24980/f2p0/1856	0.276666667	0.886666667	1.446666667	1.8707	0.056716	0.13818		0.53831	0.45397	0.64117		2.3988	0.00553	0.032859	UP
transcript_HQ_CoA_transcript24985/f2p0/1843	12.94	16.07666667	2.203333333	0.44801	0.54167	0.70359		-2.9845	0.0001488	0.0013369	DOWN	-2.5454	5.6907E-06	0.00013378	DOWN
transcript_HQ_CoA_transcript25042/f2p0/1846	0	24.64333333	300.7666667	11.301	NA	NA	NA	3.4943	0.1367	0.28548		14.78	9.1846E-06	0.00019603	UP
transcript_HQ_CoA_transcript25056/f2p0/1825	0	0	207.8233333	NA	NA	NA	NA	27.982	7.9345E-13	6.2957E-11	UP	28.004	7.6061E-13	1.129E-10	UP
transcript_HQ_CoA_transcript25059/f4p0/1839	4.56	5.88	11.43666667	0.53724	0.14863	0.28609		0.81871	0.12365	0.26579		1.3423	0.0069446	0.038667	UP
transcript_HQ_CoA_transcript2510/f2p0/4662	0.836666667	0.806666667	2.06	0.053133	0.95538	0.97571		1.2355	0.14915	0.30345		1.2805	0.002432	0.017656	UP
transcript_HQ_CoA_transcript25127/f3p0/1802	13.13	8.186666667	35.58666667	-0.53875	0.18089	0.33055		1.9657	2.4791E-07	5.6168E-06	UP	1.4187	0.0016702	0.0133	UP
transcript_HQ_CoA_transcript25133/f13p0/1735	0.21	0.083333333	186.4966667	-1.2553	0.49403	0.66211		11.085	0.00020831	0.0017804	UP	9.829	4.5631E-05	0.00074252	UP
transcript_HQ_CoA_transcript25147/f4p0/1800	117.6466667	66.93	56.91	-0.64212	0.04936	0.12399		-0.38569	0.15848	0.31698		-1.042	0.00079867	0.0074832	DOWN
transcript_HQ_CoA_transcript25161/f2p0/1822	15.55333333	7.146666667	4.61	-0.97837	0.0079875	0.029251		-0.77765	0.014861	0.054707		-1.7693	1.0269E-06	3.2332E-05	DOWN
transcript_HQ_CoA_transcript25165/f2p0/1825	17.44333333	9.9	46.35333333	-0.65518	0.0020636	0.0095126		2.1064	0.00020836	0.0017804	UP	1.4375	0.0072844	0.03995	UP
transcript_HQ_CoA_transcript25195/f2p0/1816	60.58	28.88	25.86	-0.87636	0.043768	0.11303		-0.33297	0.45628	0.64291		-1.2252	8.9977E-06	0.00019339	DOWN
transcript_HQ_CoA_transcript25204/f3p0/1833	3.143333333	1.93	11.95333333	-0.56556	0.28279	0.45209		2.5005	5.1449E-10	2.157E-08	UP	1.9224	1.6451E-08	8.8036E-07	UP
transcript_HQ_CoA_transcript25261/f2p0/1822	34.35666667	37.86333333	100.4633333	0.32118	0.59124	0.74355		1.2469	0.069608	0.17528		1.5544	0.0064446	0.036805	UP
transcript_HQ_CoA_transcript2529/f4p0/4673	3.336666667	4.86	8.596666667	0.69061	0.0083758	0.030424		0.67372	1.6058E-05	0.00020257		1.3526	6.0396E-09	3.611E-07	UP
transcript_HQ_CoA_transcript2531/f3p0/4660	1	0.683333333	3.406666667	-0.36747	0.63073	0.77232		2.1356	1.2569E-05	0.0001649	UP	1.752	0.0087282	0.04563	UP
transcript_HQ_CoA_transcript25322/f2p0/1811	2.333333333	4.786666667	5.156666667	1.2117	0.020393	0.061653		-0.05712	0.90108	0.94756		1.1411	0.0062017	0.035796	UP
transcript_HQ_CoA_transcript25342/f3p0/1813	12.54666667	7.256666667	5.426666667	-0.6473	0.16874	0.31393		-0.54854	0.248	0.4312		-1.2064	0.0019994	0.015279	DOWN
transcript_HQ_CoA_transcript25347/f2p0/1826	116.9233333	57.41333333	31.02333333	-0.88388	0.049813	0.12484		-1.034	0.0022249	0.012087	DOWN	-1.9262	3.2341E-06	8.4442E-05	DOWN
transcript_HQ_CoA_transcript25396/f2p0/1800	9.826666667	10.85	23.13	0.2847	0.44384	0.61724		0.95058	0.00044546	0.0033177		1.2249	0.00041202	0.0044123	UP
transcript_HQ_CoA_transcript25421/f2p0/1805	38.38333333	24.47333333	15.30666667	-0.51652	0.31865	0.49144		-0.80856	0.087262	0.20665		-1.3339	0.000966	0.0087087	DOWN
transcript_HQ_CoA_transcript25425/f2p0/1812	39.91666667	34.13333333	11.43	-0.10104	0.87308	0.92952		-1.7023	0.010126	0.040461	DOWN	-1.8122	4.1507E-12	5.3652E-10	DOWN
transcript_HQ_CoA_transcript25449/f3p0/1806	11.76666667	9.786666667	5.143333333	-0.14779	0.83364	0.90438		-1.0516	0.13826	0.28789		-1.2088	4.2204E-05	0.00069575	DOWN
transcript_HQ_CoA_transcript25452/f3p0/1827	30.52333333	19.14666667	14.7	-0.5168	0.00425	0.017347		-0.52946	0.0025234	0.013422		-1.0585	1.9496E-10	1.6047E-08	DOWN
transcript_HQ_CoA_transcript25484/f3p0/1804	50.44333333	43.44333333	24.45333333	-0.081725	0.81912	0.89489		-0.96027	0.004607	0.021979		-1.0528	1.6439E-06	4.7593E-05	DOWN
transcript_HQ_CoA_transcript25492/f3p0/1812	29.99333333	13.91	14.12	-0.95852	0.030809	0.085431		-0.11785	0.60799	0.76345		-1.0888	0.0081266	0.043324	DOWN
transcript_HQ_CoA_transcript25506/f2p0/1803	0.02	0	74.08333333	-0.94252	0.81733	0.89358		12.724	9.9925E-05	0.00096651	UP	11.768	1.6151E-05	0.00031571	UP
transcript_HQ_CoA_transcript25518/f3p0/1798	8.953333333	3.963333333	4.12	-1.0145	0.016377	0.051872		-0.089688	0.7539	0.86128		-1.119	0.0053022	0.031888	DOWN
transcript_HQ_CoA_transcript2552/f2p0/4628	5.506666667	2.696666667	2.276666667	-0.88403	0.0069562	0.02606		-0.39032	0.21304	0.3892		-1.2851	2.4058E-05	0.0004386	DOWN
transcript_HQ_CoA_transcript2555/f2p0/4630	5.143333333	3.136666667	11.17	-0.55268	0.10804	0.22491		1.6823	1.0254E-12	7.8968E-11	UP	1.1176	0.00043977	0.0046632	UP
transcript_HQ_CoA_transcript25558/f3p0/1787	87.02666667	87.96	41.48	0.15186	0.82089	0.89591		-1.193	0.091536	0.21392		-1.0569	0.0080532	0.043061	DOWN
transcript_HQ_CoA_transcript25629/f2p0/1784	16.39333333	19.76666667	37.96666667	0.44104	0.39721	0.57184		0.76397	0.072102	0.17997		1.1942	0.00043922	0.00466	UP
transcript_HQ_CoA_transcript25687/f18p0/1775	157.2633333	75.92333333	64.99	-0.90376	0.00045521	0.0026039		-0.36185	0.23911	0.4211		-1.2758	2.958E-06	7.8687E-05	DOWN
transcript_HQ_CoA_transcript25695/f3p0/1783	7.96	5.406666667	19.77666667	-0.41369	0.3559	0.53136		1.7385	5.8963E-05	0.00061615	UP	1.3136	0.0013147	0.011135	UP
transcript_HQ_CoA_transcript25730/f2p0/1823	5.82	6.943333333	17.44666667	0.41371	0.15416	0.29403		1.1714	9.1963E-05	0.00089898	UP	1.5719	2.0411E-06	5.7904E-05	UP
transcript_HQ_CoA_transcript25735/f3p0/1806	81.56	46.70666667	23.90333333	-0.66837	0.21591	0.37426		-1.1085	0.0037342	0.018579	DOWN	-1.7851	7.7497E-05	0.0011507	DOWN
transcript_HQ_CoA_transcript2583/f2p0/4616	0.946666667	0.27	4.393333333	-1.6474	0.078788	0.17818		3.8555	5.134E-07	1.0829E-05	UP	2.197	1.1223E-09	7.8396E-08	UP
transcript_HQ_CoA_transcript25838/f2p0/1774	11.47	8.906666667	3.676666667	-0.2176	0.65709	0.79138		-1.4285	0.0021827	0.011893	DOWN	-1.6592	9.7352E-06	0.00020586	DOWN
transcript_HQ_CoA_transcript25870/f5p0/1771	0.786666667	0.366666667	58.69666667	-0.90333	0.40944	0.58407		7.1846	0.00013378	0.0012242	UP	6.2678	0.00024061	0.0028884	UP
transcript_HQ_CoA_transcript25880/f2p0/1780	0	0	217.4733333	NA	NA	NA	NA	27.309	2.7451E-12	1.8705E-10	UP	28.003	7.6272E-13	1.129E-10	UP
transcript_HQ_CoA_transcript25966/f2p0/1789	16.7	8.406666667	3.1	-0.82891	0.078287	0.17742		-1.594	1.2931E-06	2.3607E-05	DOWN	-2.436	1.2956E-07	5.4046E-06	DOWN
transcript_HQ_CoA_transcript26003/f3p0/1779	0.756666667	0.393333333	0.06	-0.71771	0.56057	0.7186		-2.9476	0.093454	0.21736		-3.6958	0.0085425	0.044961	DOWN
transcript_HQ_CoA_transcript26045/f2p0/1756	38.77	33.41666667	15.86666667	-0.063634	0.76154	0.85873		-1.2301	0.00037617	0.0028789	DOWN	-1.3042	0.000155	0.0020293	DOWN
transcript_HQ_CoA_transcript26052/f2p0/1753	6.756666667	12.54666667	15.62	1.0848	0.045573	0.11653		0.14463	0.80085	0.89044		1.2139	0.0065079	0.037049	UP
transcript_HQ_CoA_transcript26065/f2p0/1784	48.38333333	20.34666667	9.883333333	-1.1265	0.035303	0.09525		-1.1564	0.041032	0.11741		-2.2931	3.1648E-18	1.1855E-15	DOWN
transcript_HQ_CoA_transcript2609/f2p0/4624	4.05	2.336666667	0.93	-0.64106	0.2529	0.41871		-1.4597	0.0015031	0.0088338	DOWN	-2.1159	1.5318E-05	0.00030189	DOWN
transcript_HQ_CoA_transcript26090/f2p0/1754	4.82	6.68	10.38333333	0.61435	0.18383	0.33413		0.49306	0.037911	0.11063		1.0991	0.0098515	0.049718	UP
transcript_HQ_CoA_transcript26100/f2p0/1753	0	0	71.66333333	NA	NA	NA	NA	12.631	0.0012246	0.0074894	UP	12.636	0.001219	0.010475	UP
transcript_HQ_CoA_transcript2611/f2p0/4619	3.79	2.703333333	1.67	-0.31964	0.37787	0.55341		-0.85449	0.0058535	0.026557		-1.1867	0.00068562	0.0066514	DOWN
transcript_HQ_CoA_transcript26140/f2p0/1767	66.68	71.83666667	29.27333333	0.23572	0.7053	0.82268		-1.43	0.017949	0.063146		-1.2025	0.001938	0.014921	DOWN
transcript_HQ_CoA_transcript26178/f2p0/1741	2.973333333	0.893333333	10.70666667	-1.6435	0.17262	0.31943		3.4894	0.00090558	0.0059063	UP	1.8382	1.3462E-05	0.00027153	UP
transcript_HQ_CoA_transcript2622/f2p0/4599	7.226666667	2.916666667	0.63	-1.1842	0.11823	0.24095		-2.3184	0.0052373	0.024296	DOWN	-3.5104	6.6205E-36	2.4304E-32	DOWN
transcript_HQ_CoA_transcript26259/f2p0/1739	14.15	7.766666667	50.94666667	-0.69286	0.1114	0.23043		2.6117	0.00043839	0.0032704	UP	1.9024	0.0094148	0.048136	UP

transcript_HQ_CoA_transcript26308/f14p0/1725	269.9466667	146.4233333	122.86	-0.71803	0.032796	0.089955		-0.40739	0.0044679	0.021483		-1.1398	0.00050166	0.0051788	DOWN
transcript_HQ_CoA_transcript26313/f2p0/1740	2.65	1.896666667	1.056666667	-0.31389	0.67855	0.80548		-1.0043	0.20481	0.37893		-1.3338	0.0055739	0.033067	DOWN
transcript_HQ_CoA_transcript26377/f3p0/1783	23.56	28.02666667	117.29	0.38599	0.7845	0.87346		1.9546	0.15297	0.30893		2.3274	2.8685E-06	7.6613E-05	UP
transcript_HQ_CoA_transcript26382/f2p0/1719	55.08	27.30333333	20.43333333	-0.86161	9.6928E-06	9.1162E-05		-0.56374	0.0060303	0.027204		-1.438	1.3333E-13	2.3308E-11	DOWN
transcript_HQ_CoA_transcript26384/f3p0/1764	54.28333333	38.19333333	26.44666667	-0.352	0.26487	0.43235		-0.67593	0.00076945	0.0051679		-1.0404	0.00054597	0.0055336	DOWN
transcript_HQ_CoA_transcript26385/f2p0/1726	81.54	127.9933333	190.7433333	0.8132	0.0018868	0.0088334		0.41443	0.23455	0.41541		1.2125	7.3781E-05	0.0011046	UP
transcript_HQ_CoA_transcript26428/f3p0/1717	90.94	54.08333333	45.04666667	-0.60427	0.052612	0.13042		-0.40366	0.098435	0.22598		-1.0191	6.8715E-05	0.001045	DOWN
transcript_HQ_CoA_transcript26433/f2p0/1763	14.03333333	12.63666667	1.81	-0.0068634	0.98377	0.99225		-2.9282	0.00043511	0.0032512	DOWN	-2.9505	0.00011089	0.0015478	DOWN
transcript_HQ_CoA_transcript26519/f2p0/1736	43.42333333	17.73	14.82333333	-1.1718	0.072789	0.16812		-0.38416	0.54254	0.71233		-1.5653	9.0665E-06	0.00019403	DOWN
transcript_HQ_CoA_transcript26524/f3p0/1676	0.49	100.1433333	25.77333333	7.9164	NA	NA	NA	-2.1297	0.19182	0.36196		5.7664	0.0016903	0.013436	UP
transcript_HQ_CoA_transcript26609/f20p0/1633	122.87	180.5333333	554.9566667	0.72905	0.11647	0.23826		1.4731	1.2714E-05	0.00016609	UP	2.1876	6.2822E-06	0.00014468	UP
transcript_HQ_CoA_transcript26627/f2p0/1736	20.71	33.61666667	56.39333333	0.82994	0.073232	0.16881		0.61143	0.21304	0.3892		1.428	0.0028906	0.020182	UP
transcript_HQ_CoA_transcript26697/f2p0/1707	13.59333333	11.3	34.12666667	-0.10567	0.80371	0.886		1.4477	1.4829E-05	0.00018875	UP	1.3292	0.0018926	0.014614	UP
transcript_HQ_CoA_transcript26752/f2p0/1702	23.12333333	27.10666667	47.05	0.3875	0.061672	0.14767		0.64633	0.00046406	0.0034366		1.0222	3.8015E-06	9.5978E-05	UP
transcript_HQ_CoA_transcript26929/f2p0/1691	0.623333333	0.32	22.41	-0.75544	0.62054	0.76499		5.9845	2.205E-22	7.7865E-20	UP	5.2029	1.1892E-10	1.0545E-08	UP
transcript_HQ_CoA_transcript2701/f2p0/4582	5.503333333	5.68	12.49333333	0.23472	0.6707	0.80093		0.94845	0.088624	0.20914		1.1701	0.00008033	0.0011857	UP
transcript_HQ_CoA_transcript27016/f2p0/1666	26.63	42.55	80.78	0.859	0.046436	0.11819		0.75372	0.10693	0.23962		1.5952	9.9917E-05	0.0014162	UP
transcript_HQ_CoA_transcript27039/f2p0/1673	14.26333333	23.86666667	30.39	0.90552	0.00021339	0.0013603		0.19784	0.30009	0.49048		1.0906	7.2964E-07	2.4087E-05	UP
transcript_HQ_CoA_transcript27091/f3p0/1647	0.93	3.176666667	2.756666667	2.0049	NA	NA	NA	-0.42255	NA	NA		1.5663	0.0031022	0.021294	UP
transcript_HQ_CoA_transcript27112/f3p0/1657	0.406666667	0.04	792.4366667	-3.1887	0.23308	0.39529		14.209	0.000239	0.0019768	UP	10.997	0.00048469	0.0050462	UP
transcript_HQ_CoA_transcript27149/f15p0/1583	178.8933333	82.63333333	68.07666667	-0.95935	0.033374	0.091152		-0.40486	0.35634	0.54853		-1.3811	0.0035625	0.023709	DOWN
transcript_HQ_CoA_transcript27195/f2p0/1660	3.66	2.203333333	1.026666667	-0.58877	0.31495	0.48737		-1.2267	0.056498	0.15025		-1.8267	0.0011639	0.010072	DOWN
transcript_HQ_CoA_transcript27274/f7p0/1629	52.07	92.47666667	142.76	0.98175	0.0073635	0.027306		0.48676	0.098542	0.22607		1.4538	7.0988E-07	2.3477E-05	UP
transcript_HQ_CoA_transcript27305/f3p0/1667	2.77	2.213333333	0.7	-0.14918	0.79603	0.88073		-1.835	0.0064677	0.028586	DOWN	-1.9963	0.0010424	0.0092652	DOWN
transcript_HQ_CoA_transcript27308/f2p0/1661	3.033333333	6.66	16.42666667	1.2548	0.11989	0.24351		1.1778	0.075402	0.18608		2.4206	2.073E-08	1.084E-06	UP
transcript_HQ_CoA_transcript27334/f3p0/1703	14.92	23.05333333	44.5	0.78956	0.0071122	0.02652		0.81801	0.036573	0.10755		1.5938	3.4037E-05	0.00058917	UP
transcript_HQ_CoA_transcript27337/f2p0/1656	8.33	4.173333333	3.093333333	-0.84537	0.031998	0.088113		-0.56375	0.17271	0.33629		-1.4203	0.00057802	0.0057755	DOWN
transcript_HQ_CoA_transcript27344/f2p0/1648	4.693333333	14.65	0.083333333	1.7759	0.36711	0.54268		-7.6078	3.153E-07	6.9545E-06	DOWN	-5.8448	3.5594E-05	0.00060837	DOWN
transcript_HQ_CoA_transcript27369/f5p0/1647	42.78	37.77333333	18.59333333	-0.024132	0.93808	0.96527		-1.1658	2.0684E-05	0.00025157	DOWN	-1.2033	1.1015E-05	0.0002282	DOWN
transcript_HQ_CoA_transcript27387/f2p0/1639	5.073333333	8.196666667	1.826666667	0.83617	0.097134	0.2076		-2.297	5.2285E-09	1.7361E-07	DOWN	-1.4731	0.0058365	0.03438	DOWN
transcript_HQ_CoA_transcript27393/f2p0/1631	6.24	7.793333333	26.57333333	0.48485	0.16617	0.3104		1.6705	0.031377	0.096349		2.1409	0.0045574	0.028638	UP
transcript_HQ_CoA_transcript274/f2p0/6978	1.216666667	1.15	0.153333333	0.032809	0.97967	0.99013		-3.0337	0.091678	0.21418		-3.021	0.0015918	0.012785	DOWN
transcript_HQ_CoA_transcript27407/f14p0/1622	686.9666667	474.7566667	244.3	-0.37649	0.35479	0.53041		-1.1063	0.0099072	0.039753	DOWN	-1.4914	0.00089461	0.0081898	DOWN
transcript_HQ_CoA_transcript27414/f5p0/1625	185.7333333	298.1566667	611.3733333	0.83865	0.017593	0.054803		0.89677	0.011413	0.044518		1.7226	3.8541E-06	9.6906E-05	UP
transcript_HQ_CoA_transcript27421/f2p0/1635	49.13333333	27.39	20.14	-0.69861	0.014	0.046083		-0.58869	0.00020633	0.001768		-1.2968	3.2781E-06	8.5106E-05	DOWN
transcript_HQ_CoA_transcript27462/f3p0/1631	8.543333333	8.636666667	64.38333333	0.23144	0.75486	0.85414		2.6996	3.2572E-05	0.00037595	UP	2.9158	1.6727E-13	2.8168E-11	UP
transcript_HQ_CoA_transcript27498/f4p0/1631	33.59666667	55.92333333	82.85666667	0.85946	0.091268	0.19852		0.43059	0.32233	0.51333		1.2823	0.0031303	0.021447	UP
transcript_HQ_CoA_transcript27539/f2p0/1637	0.643333333	0.363333333	66.28666667	-0.66266	0.56303	0.72077		7.4076	5.1401E-05	0.00055224	UP	6.7357	0.00014142	0.0018873	UP
transcript_HQ_CoA_transcript27597/f2p0/1627	36.78666667	23.88	12.51	-0.46514	0.22528	0.3856		-1.099	0.00014865	0.0013369	DOWN	-1.5745	2.4478E-06	6.7058E-05	DOWN
transcript_HQ_CoA_transcript27624/f2p0/1616	20.44666667	12.53333333	6.496666667	-0.55232	0.38883	0.56418		-1.0988	0.03669	0.10777		-1.6602	0.0048724	0.029951	DOWN
transcript_HQ_CoA_transcript27649/f3p0/1613	0	0	75.78333333	NA	NA	NA	NA	26.402	1.4006E-11	7.9973E-10	UP	26.399	1.4079E-11	1.5201E-09	UP
transcript_HQ_CoA_transcript27723/f4p0/1601	24.11333333	17.52333333	208.1233333	-0.23135	0.89715	0.94344		3.338	0.046769	0.12969		3.096	0.00087354	0.008037	UP
transcript_HQ_CoA_transcript27749/f2p0/1609	1.113333333	1.053333333	8.723333333	0.091354	0.90476	0.94768		2.9259	0.0037327	0.018576	UP	3.0036	0.0015882	0.012769	UP
transcript_HQ_CoA_transcript2782/f5p0/4490	27.82	22.12333333	1.146666667	-0.17972	0.92685	0.95941		-4.3861	0.0023055	0.012476	DOWN	-4.5829	0.0024257	0.017626	DOWN
transcript_HQ_CoA_transcript27824/f2p0/1605	1.78	0.713333333	0	-1.1654	0.40602	0.5806		-6.0845	0.027266	0.086974		-7.2572	2.3402E-05	0.00042955	DOWN
transcript_HQ_CoA_transcript27846/f2p0/1599	4.003333333	4.41	10.70666667	0.30898	0.58034	0.73454		1.1037	0.055478	0.14827		1.3999	0.0048977	0.030066	UP
transcript_HQ_CoA_transcript2787/f2p0/4557	1.696666667	1.143333333	0.17	-0.43784	0.54411	0.70578		-2.8913	0.0014314	0.0085212	DOWN	-3.352	1.8324E-08	9.7207E-07	DOWN
transcript_HQ_CoA_transcript27890/f3p0/1586	0.07	0.06	466.18	-0.0018362	0.99963	0.99968		12.762	0.00083357	0.0055058	UP	12.747	0.00084134	0.0078152	UP
transcript_HQ_CoA_transcript27903/f2p0/1591	49.10333333	82.92333333	102.8066667	0.90527	1.6588E-05	0.00014545		0.17581	0.38939	0.58034		1.069	1.2251E-06	3.7478E-05	UP
transcript_HQ_CoA_transcript2793/f3p0/4532	11.57333333	5.31	4.736666667	-0.99573	0.021161	0.063524		-0.29759	0.51989	0.69323		-1.3025	1.7786E-06	5.1249E-05	DOWN
transcript_HQ_CoA_transcript27940/f14p0/1553	125.6866667	76.66666667	36.02333333	-0.58672	0.60687	0.75516		-1.2132	0.17347	0.33746		-1.8102	1.3204E-12	1.8787E-10	DOWN
transcript_HQ_CoA_transcript27978/f5p0/1579	34.59666667	21.21666667	117.7266667	-0.57003	0.38905	0.56425		2.3445	0.00038775	0.0029576	UP	1.7625	9.5707E-08	4.1659E-06	UP
transcript_HQ_CoA_transcript27988/f2p0/1588	32.37	40.19666667	1.7	0.43915	0.40648	0.58094		-4.7159	1.5633E-68	9.5513E-65	DOWN	-4.2885	1.4173E-14	3.0564E-12	DOWN
transcript_HQ_CoA_transcript28068/f2p0/1570	71.72666667	47.77333333	19.51666667	-0.45584	0.44778	0.62073		-1.4263	0.0071944	0.031013	DOWN	-1.8917	1.4093E-06	4.2198E-05	DOWN
transcript_HQ_CoA_transcript28092/f2p0/1607	5.003333333	4.713333333	1.82	0.091376	0.887	0.93757		-1.5639	0.014177	0.052741		-1.4857	0.0022067	0.016485	DOWN
transcript_HQ_CoA_transcript28144/f2p0/1561	8.803333333	5.936666667	2.71	-0.44068	0.64016	0.77898		-1.2398	0.19456	0.36557		-1.692	0.00060001	0.0059627	DOWN
transcript_HQ_CoA_transcript2820/f2p0/4522	4.976666667	2.866666667	1.153333333	-0.65626	0.18325	0.33341		-1.443	0.0070088	0.030384	DOWN	-2.1103	7.6264E-10	5.5549E-08	DOWN
transcript_HQ_CoA_transcript28202/f4p0/1543	56.79666667	25.81666667	28.59666667	-1.0062	0.020609	0.062183		0.013783	0.97786	0.98843		-1.0027	0.00087841	0.0080697	DOWN
transcript_HQ_CoA_transcript28314/f2p0/1546	1.513333333	3.04	11.79	1.225	0.21316	0.37097		1.7972	0.025644	0.082999		3.0042	1.2012E-05	0.0002469	UP
transcript_HQ_CoA_transcript2834/f2p0/4553	3.43	3.263333333	8.653333333	0.10869	0.81105	0.89019		1.2458	0.00090714	0.0059108	UP	1.3404	2.4063E-05	0.0004386	UP
transcript_HQ_CoA_transcript28345/f2p0/1537	8.66	9.706666667	36.62333333	0.29767	0.50049	0.66788		1.8295	0.024402	0.080027		2.1146	0.0040698	0.026211	UP
transcript_HQ_CoA_transcript28366/f13p0/1438	0	36.76666667	507.7066667	11.454	NA	NA	NA	3.677	0.23377	0.41431		28.544	2.7478E-13	4.5032E-11	UP
transcript_HQ_CoA_transcript28369/f2p0/1530	66.01333333	35.80333333	11.11666667	-0.70383	0.43761	0.61123		-1.8069	0.014563	0.053915		-2.5277	0.00043133	0.0045949	DOWN
transcript_HQ_CoA_transcript28376/f2p0/1532	0.076666667	0.256666667	33.73333333	1.9846	0.23508	0.39747		6.8302	2.4652E-27	1.412E-24	UP	8.8228	7.9236E-21	3.7292E-18	UP
transcript_HQ_CoA_transcript28426/f5p0/1531	43.08666667	68.43	106.0433333	0.82744	3.4605E-05	0.00027919		0.48151	0.028454	0.089782		1.2969	4.9263E-08	2.3426E-06	UP
transcript_HQ_CoA_transcript28469/f4p0/1512	46.68666667	72.65666667	130.01	0.78703	0.0048768	0.01945		0.69079	0.039461	0.11378		1.4667	2.6865E-06	0.00007273	UP
transcript_HQ_CoA_transcript28486/f3p0/1516	5.563333333	1.71	38.19333333	-1.5264	0.19664	0.35008		4.3869	0.004726	0.022424	UP	2.8433	0.00067551	0.0065707	UP
transcript_HQ_CoA_transcript28492/f2p0/1520	42.07333333	31.50333333	115.0766667	-0.24867	0.53495	0.69752		1.7386	0.00017908	0.0015705	UP	1.4786	0.0047938	0.029583	UP
transcript_HQ_CoA_transcript28534/f6p0/1463	104.4666667	102.8233333	244.21	0.16026	0.68964	0.81237		1.1018	4.0112E-06	6.2305E-05	UP	1.2472	0.0012937	0.010989	UP
transcript_HQ_CoA_transcript28543/f2p0/1509	16.84666667	15.47666667	5.636666667	0.037657	0.93009	0.96111		-1.6062	2.6615E-06	4.4028E-05	DOWN	-1.5846	0.00026764	0.0031653	DOWN
transcript_HQ_CoA_transcript28552/f2p0/1502	4.25	1.19	1.053333333	-1.5891	0.36949	0.54511		-0.37885	0.84863	0.91782		-1.9951	0.0013516	0.011365	DOWN
transcript_HQ_CoA_transcript2863/f2p0/4528	1.74	3.543333333	5.536666667	1.1529	0.04238	0.11004		0.50911	0.34559	0.53772		1.6531	5.6994E-06	0.00013378	UP
transcript_HQ_CoA_transcript28634/f2p0/1496	3.31	1.543333333	13.14666667	-0.94786	0.10857	0.22569		2.9365	8.172E-11	4.0373E-09	UP	1.9762	2.0476E-05	0.00038344	UP
transcript_HQ_CoA_transcript28659/f2p0/1492	2.423333333	3.02	11.79666667	0.5248	0.46334	0.63513		1.8221	0.045956	0.12788		2.3306	0.0068468	0.038306	UP
transcript_HQ_CoA_transcript28688/f6p0/1444	72.34666667	49.96666667	19.63666667	-0.42803	0.52257	0.68701		-1.4705	0.037357	0.10938		-1.9046	5.4518E-05	0.00086042	DOWN
transcript_HQ_CoA_transcript28699/f4p0/1479	17.6	9.276666667	124.0033333	-0.75478	0.025001	0.072597		3.6181	3.0328E-06	4.9411E-05	UP	2.8496	0.003319	0.022422	UP
transcript_HQ_CoA_transcript28740/f5p0/1475	7.393333333	9.89	14.91333333	0.60275	0.14754	0.28458		0.4244	0.33622	0.52803		1.0116	0.006713	0.037848	UP
transcript_HQ_CoA_transcript28748/f3p0/1483	15.81333333	16.24666667	7.73	0.18015	0.67953	0.80607		-1.2005	0.0047714	0.022557	DOWN	-1.0332	0.000121	0.0016661	DOWN
transcript_HQ_CoA_transcript28754/f2p0/1475	44.17	34.89	21.62666667	-0.18826	0.49814	0.66571		-0.83934	0.0012127	0.0074362		-1.0404	3.5246E-05	0.00060519	DOWN

transcript_HQ_CoA_transcript28976/f10p0/1414	0	0	427.5166667	NA	NA	NA	NA	27.39	2.3695E-12	1.6576E-10	UP	28.548	2.7242E-13	4.5032E-11	UP
transcript_HQ_CoA_transcript28983/f2p0/1440	25.44	21.66	9.35	-0.087451	0.79307	0.87923		-1.355	7.6784E-07	1.5314E-05	DOWN	-1.4525	0.00000499	0.00011973	DOWN
transcript_HQ_CoA_transcript29002/f2p0/1431	1.576666667	4.59	19.01	1.628	0.16681	0.3113		1.9626	0.04701	0.13024		3.5861	9.5834E-11	8.6227E-09	UP
transcript_HQ_CoA_transcript29019/f3p0/1431	2.08	2.17	0.28	0.17707	0.81886	0.89472		-3.0633	0.0054425	0.025033	DOWN	-2.902	0.0036308	0.023999	DOWN
transcript_HQ_CoA_transcript29042/f2p0/1414	153.02	133.25	74	-0.040738	0.84911	0.9147		-0.9996	3.0574E-09	1.0654E-07		-1.0523	3.4652E-07	1.2695E-05	DOWN
transcript_HQ_CoA_transcript29067/f2p0/1364	23.36666667	9.826666667	11.74333333	-1.1234	0.04772	0.12065		0.12453	0.80053	0.8903		-1.0088	0.0086778	0.045431	DOWN
transcript_HQ_CoA_transcript29073/f2p0/1419	9.156666667	4.793333333	3.816666667	-0.76206	0.098378	0.20989		-0.48629	0.32838	0.51922		-1.2589	0.0047523	0.02943	DOWN
transcript_HQ_CoA_transcript29078/f3p0/1431	0	0	80.53	NA	NA	NA	NA	12.443	0.0014484	0.0086001	UP	12.448	0.0014421	0.011913	UP
transcript_HQ_CoA_transcript2924/f8p0/4404	8.833333333	7.163333333	2.88	-0.12108	0.78098	0.87109		-1.4625	0.00016379	0.0014512	DOWN	-1.5983	0.00054203	0.0055119	DOWN
transcript_HQ_CoA_transcript2927/f2p0/4538	2.996666667	1.803333333	1.386666667	-0.58558	0.1286	0.25669		-0.50712	0.24923	0.4325		-1.1067	0.002566	0.018405	DOWN
transcript_HQ_CoA_transcript29292/f2p0/1371	7.543333333	5.25	2	-0.39497	0.52703	0.69093		-1.5396	0.007915	0.033467	DOWN	-1.9457	0.000129	0.0017519	DOWN
transcript_HQ_CoA_transcript29306/f2p0/1390	63.69	41.35	26.66	-0.45844	0.21916	0.37792		-0.79814	0.01523	0.055719		-1.2674	8.957E-07	2.8592E-05	DOWN
transcript_HQ_CoA_transcript29382/f4p0/1347	1.816666667	0.933333333	242.2733333	-0.78786	0.37406	0.54933		7.9314	1.0038E-05	0.00013649	UP	7.1248	9.4406E-05	0.0013519	UP
transcript_HQ_CoA_transcript29387/f2p0/1355	2.093333333	1.05	53.17333333	-0.84753	0.14958	0.28729		5.5785	0.00011433	0.0010725	UP	4.7183	0.0010075	0.0090075	UP
transcript_HQ_CoA_transcript29398/f2p0/1351	1.946666667	0.936666667	0	-0.93343	0.24661	0.41163		-6.1381	0.00063798	0.0044462	DOWN	-7.0819	5.6537E-06	0.00013321	DOWN
transcript_HQ_CoA_transcript29438/f2p0/1362	17.18666667	26.84333333	3.403333333	0.81642	0.012037	0.040903		-3.1379	4.6565E-06	7.0888E-05	DOWN	-2.3392	0.00062516	0.0061726	DOWN
transcript_HQ_CoA_transcript29454/f2p0/1329	24.82333333	14.98666667	8.89	-0.57669	0.32899	0.50276		-0.89483	0.092522	0.21578		-1.4814	0.0082592	0.043879	DOWN
transcript_HQ_CoA_transcript29459/f6p0/1330	108.4466667	73.28	28.03333333	-0.4268	0.49041	0.6589		-1.4971	0.011928	0.046105	DOWN	-1.9348	0.00016313	0.0021176	DOWN
transcript_HQ_CoA_transcript29463/f2p0/1325	57.05	51.91	156.27	0.034907	0.9223	0.95752		1.4748	0.020125	0.068906		1.4957	0.009883	0.049822	UP
transcript_HQ_CoA_transcript29509/f2p0/1320	15.90666667	11.32666667	213.6833333	-0.32792	0.35636	0.5317		4.1325	0.00072044	0.0049086	UP	3.792	0.001996	0.015265	UP
transcript_HQ_CoA_transcript29571/f2p0/1294	91.78333333	146.6033333	226.6333333	0.83643	0.0010468	0.0053473		0.49237	0.0067513	0.029597		1.3152	3.6431E-07	1.3268E-05	UP
transcript_HQ_CoA_transcript2958/f3p0/4515	3.526666667	1.996666667	1.75	-0.63052	0.21219	0.36992		-0.36557	0.45303	0.64024		-1.0112	0.0015242	0.012357	DOWN
transcript_HQ_CoA_transcript29581/f5p0/1229	39.98666667	67.29	80.1	0.91135	6.6963E-05	0.00049664		0.10537	0.50452	0.68151		1.0048	1.0812E-05	0.00022449	UP
transcript_HQ_CoA_transcript2961/f2p0/4460	0.936666667	0.33	2.926666667	-1.3127	0.04115	0.10756		2.9563	2.271E-11	1.2519E-09	UP	1.6314	0.0012111	0.010423	UP
transcript_HQ_CoA_transcript2962/f2p0/4522	4.136666667	5.073333333	8.753333333	0.45498	0.34081	0.51588		0.62132	0.056836	0.15096		1.0655	0.0041934	0.026781	UP
transcript_HQ_CoA_transcript29620/f4p0/1261	20.67	18.96666667	48.54666667	0.079251	0.9071	0.94883		1.163	0.076237	0.18757		1.2313	0.00059206	0.0058933	UP
transcript_HQ_CoA_transcript29681/f2p0/1267	67.14666667	52.53666667	33.55	-0.20741	0.469	0.64026		-0.79183	0.0033887	0.017129		-1.0116	1.9065E-05	0.0003615	DOWN
transcript_HQ_CoA_transcript29688/f2p0/1258	0	0.89	183.6966667	5.8413	0.13572	0.26754		7.5642	0.042786	0.12112		13.389	0.00060986	0.0060443	UP
transcript_HQ_CoA_transcript29693/f2p0/1261	0	0	90.69333333	NA	NA	NA	NA	12.395	0.0015112	0.0088662	UP	12.4	0.0015038	0.012257	UP
transcript_HQ_CoA_transcript29727/f2p0/1231	11.90333333	11.86	4.926666667	0.12719	0.74689	0.84908		-1.4138	0.0011278	0.0070428	DOWN	-1.2972	0.002554	0.018345	DOWN
transcript_HQ_CoA_transcript29768/f3p0/1231	17.43333333	46.02666667	46.85666667	1.5107	0.059236	0.14297		-0.093992	0.90218	0.94795		1.4087	0.0004247	0.0045348	UP
transcript_HQ_CoA_transcript29821/f2p0/1215	150.21	87.30666667	39.20666667	-0.6454	0.16864	0.3138		-1.2891	0.012645	0.048257	DOWN	-1.943	4.0842E-08	1.9728E-06	DOWN
transcript_HQ_CoA_transcript29822/f2p0/1217	13.89	21.81666667	80.8	0.81525	0.0019067	0.0089039		1.7772	0.006783	0.029671	UP	2.5788	4.1013E-05	0.00067881	UP
transcript_HQ_CoA_transcript29837/f2p0/1210	22.15333333	24.57666667	49.62666667	0.32315	0.38877	0.56417		0.85453	0.0003246	0.0025404		1.1649	0.00013088	0.0017729	UP
transcript_HQ_CoA_transcript29878/f2p0/1195	134.5566667	121.59	47.74	-0.003704	0.99509	0.99743		-1.4786	0.018998	0.066049		-1.4921	6.9299E-06	0.00015723	DOWN
transcript_HQ_CoA_transcript29881/f4p0/1176	0.136666667	0.086666667	188.7333333	-0.45578	0.90662	0.9486		10.913	0.0033032	0.016783	UP	10.449	0.0056526	0.033447	UP
transcript_HQ_CoA_transcript29994/f3p0/1127	147.4166667	249.97	366.1966667	0.87451	0.13157	0.26095		0.44063	0.43748	0.62606		1.3044	0.0032131	0.021876	UP
transcript_HQ_CoA_transcript30017/f3p0/1106	862.0633333	394.5933333	392.52	-0.98889	0.014853	0.048145		-0.14413	0.49443	0.67374		-1.1436	0.001461	0.012031	DOWN
transcript_HQ_CoA_transcript30042/f2p0/1095	0.076666667	0.14	426.8133333	0.94901	0.79334	0.87931		11.568	0.0023171	0.012519	UP	12.504	3.3967E-05	0.00058874	UP
transcript_HQ_CoA_transcript3005/f2p0/4461	1.793333333	1.05	7.306666667	-0.54234	0.65792	0.79195		2.5824	0.02375	0.078349		2.0266	5.7661E-10	4.3376E-08	UP
transcript_HQ_CoA_transcript30067/f2p0/1068	13.54333333	14.37666667	62.1	0.31609	0.69187	0.814		1.9133	0.0058875	0.026671	UP	2.2084	0.00022683	0.0027555	UP
transcript_HQ_CoA_transcript30082/f2p0/1049	12.41666667	20.84666667	24.9	0.9016	0.0067333	0.025381		0.11236	0.68831	0.8198		1.002	9.1195E-05	0.0013191	UP
transcript_HQ_CoA_transcript30083/f2p0/1051	12.96666667	12.65	144.52	0.1587	0.79855	0.88207		3.3298	2.7221E-11	1.4589E-09	UP	3.4754	1.5649E-22	9.5749E-20	UP
transcript_HQ_CoA_transcript30114/f4p0/999	85.93666667	130.0633333	181.3833333	0.7636	0.00026146	0.0016176		0.32301	0.15034	0.30527		1.0728	5.7189E-08	2.671E-06	UP
transcript_HQ_CoA_transcript3012/f2p0/4433	8.71	5.696666667	2.983333333	-0.4576	0.2946	0.46507		-1.074	0.00030567	0.0024149	DOWN	-1.5464	9.7589E-05	0.0013875	DOWN
transcript_HQ_CoA_transcript30142/f3p0/972	0	0.146666667	586.3633333	2.8424	0.4745	0.64583		11.772	0.001928	0.010784	UP	28.316	4.229E-13	6.6917E-11	UP
transcript_HQ_CoA_transcript30152/f2p0/956	37.76666667	55.49333333	13.51	0.70687	0.14288	0.27804		-2.1803	6.0474E-07	1.2398E-05	DOWN	-1.4834	0.0011253	0.0098264	DOWN
transcript_HQ_CoA_transcript3016/f3p0/4484	6.4	3.21	3.133333333	-0.87424	0.13038	0.25933		-0.15591	0.79314	0.88604		-1.0383	0.00082708	0.0077022	DOWN
transcript_HQ_CoA_transcript30163/f2p0/930	96.29333333	124.6733333	206.58	0.54985	0.091919	0.19963		0.55882	0.078183	0.19102		1.0967	7.4425E-11	6.8993E-09	UP
transcript_HQ_CoA_transcript30176/f2p0/903	292.3733333	428.56	806.1366667	0.70685	0.019284	0.058956		0.77102	6.4804E-06	9.3453E-05		1.4671	8.8134E-07	2.8281E-05	UP
transcript_HQ_CoA_transcript30178/f2p0/901	0	0.386666667	192.2933333	3.9808	0.31204	0.48419		8.8632	0.018333	0.064175		26.636	9.2366E-12	1.0596E-09	UP
transcript_HQ_CoA_transcript30181/f2p0/896	421.5433333	609.4333333	839.88	0.68421	0.022368	0.066471		0.33197	0.12466	0.26759		1.0027	4.4175E-05	0.00072202	UP
transcript_HQ_CoA_transcript30182/f3p0/891	0	0.976666667	2338.09	5.3287	0.093521	0.20193		11.103	0.00059638	0.0042028	UP	29.938	1.8135E-14	3.7827E-12	UP
transcript_HQ_CoA_transcript30184/f2p0/898	20.63666667	10.78333333	8.51	-0.77377	0.028899	0.081269		-0.49462	0.19682	0.36849		-1.2817	9.6405E-05	0.0013781	DOWN
transcript_HQ_CoA_transcript30197/f3p0/846	3.406666667	2.043333333	880.0733333	-0.57513	0.40412	0.57842		8.6686	5.3484E-07	1.1204E-05	UP	8.0786	3.4183E-06	8.7876E-05	UP
transcript_HQ_CoA_transcript3021/f4p0/4418	4.246666667	2.15	1.44	-0.8509	0.038904	0.10272		-0.7051	0.091167	0.21336		-1.5675	4.1575E-05	0.00068625	DOWN
transcript_HQ_CoA_transcript30219/f3p0/795	1609.066667	959.8366667	521.4	-0.58835	0.22932	0.3904		-1.0371	2.4166E-06	4.0711E-05	DOWN	-1.6356	0.00029047	0.0033638	DOWN
transcript_HQ_CoA_transcript30226/f2p0/778	238.9466667	241.57	103.0433333	0.18637	0.47626	0.64761		-1.3829	0.0013491	0.0081126	DOWN	-1.2113	0.0025001	0.018067	DOWN
transcript_HQ_CoA_transcript30231/f2p0/775	456.2333333	553.3366667	44.96	0.50363	0.40277	0.57716		-3.83	2.4722E-11	1.3406E-09	DOWN	-3.3425	3.9928E-21	2.0358E-18	DOWN
transcript_HQ_CoA_transcript30235/f2p0/765	535.6166667	139.84	29.58666667	-1.8288	0.15006	0.28792		-2.36	0.0083879	0.034989	DOWN	-4.1929	8.9625E-06	0.00019302	DOWN
transcript_HQ_CoA_transcript30280/f2p0/533	126.48	69.91333333	302.46	-0.58675	0.48489	0.65457		1.8671	0.016794	0.060153		1.2619	0.0054902	0.032676	UP
transcript_HQ_CoA_transcript3049/f2p0/4426	21.26666667	13.47333333	10.08	-0.49802	0.045559	0.11652		-0.55895	0.013728	0.051508		-1.0708	0.00007142	0.0010789	DOWN
transcript_HQ_CoA_transcript3059/f2p0/4421	5.99	7.716666667	15.13	0.50756	0.086987	0.19158		0.82932	0.015088	0.05533		1.3261	8.3609E-06	0.00018335	UP
transcript_HQ_CoA_transcript3069/f5p0/4414	0.976666667	2.09	2.07	1.2304	0.35563	0.53128		-0.14166	0.91259	0.95326		1.0783	0.0020687	0.015697	UP
transcript_HQ_CoA_transcript3144/f2p0/4433	0.253333333	1.353333333	2.17	2.5581	0.046632	0.11859		0.54323	0.50112	0.67863		3.0811	0.0015175	0.012324	UP
transcript_HQ_CoA_transcript3164/f2p0/4398	3.133333333	3.376666667	1.453333333	0.23738	0.67487	0.80341		-1.331	0.031725	0.097159		-1.1081	0.0063141	0.036319	DOWN
transcript_HQ_CoA_transcript3166/f2p0/4365	3.576666667	3.32	17.28333333	0.019398	0.97613	0.98822		2.238	1.8438E-14	1.9648E-12	UP	2.2487	0.00003765	0.00063458	UP
transcript_HQ_CoA_transcript317/f3p0/6853	4.616666667	5	1.936666667	0.2569	0.49148	0.65985		-1.4967	0.00007103	0.00072168	DOWN	-1.2508	5.0203E-05	0.00080408	DOWN
transcript_HQ_CoA_transcript3180/f2p0/4421	1.143333333	1.21	0.193333333	0.22969	0.78968	0.87704		-2.7703	0.0008519	0.0056066	DOWN	-2.5552	0.0010899	0.0095898	DOWN
transcript_HQ_CoA_transcript3191/f9p0/4309	88.96666667	40.51	42.8	-0.99372	0.00069711	0.0037495		-0.062016	0.82447	0.9043		-1.0675	1.4983E-10	1.2851E-08	DOWN
transcript_HQ_CoA_transcript3258/f3p0/4341	8.966666667	11.27333333	3.84	0.48721	0.29505	0.4656		-1.6831	1.5916E-05	0.00020092	DOWN	-1.2104	0.0018172	0.014225	DOWN
transcript_HQ_CoA_transcript327/f2p0/6876	8.546666667	9.156666667	3.86	0.25743	0.33157	0.50548		-1.3942	1.7989E-12	1.3032E-10	DOWN	-1.1489	0.00001219	0.00024917	DOWN
transcript_HQ_CoA_transcript3288/f4p0/4330	3.006666667	2.743333333	7.92	0.0083432	0.98436	0.9924		1.3761	8.4937E-06	0.00011785	UP	1.3753	0.00053933	0.0054936	UP
transcript_HQ_CoA_transcript332/f2p0/6860	0.23	6.64	1.053333333	5.0519	NA	NA	NA	-2.8648	NA	NA	NA	2.1778	0.0039491	0.025613	UP
transcript_HQ_CoA_transcript3322/f5p0/4298	0.79	0.453333333	12.89666667	-0.66203	0.31236	0.48448		4.6757	6.8004E-17	1.0052E-14	UP	4.0033	4.3638E-13	6.8459E-11	UP
transcript_HQ_CoA_transcript3443/f3p0/4268	5.813333333	4.516666667	2.223333333	-0.21968	0.72008	0.8323		-1.1468	0.035297	0.10504		-1.3802	0.0011322	0.0098681	DOWN
transcript_HQ_CoA_transcript3444/f2p0/3970	9.533333333	3.92	4.666666667	-1.1461	0.075374	0.17253		0.11796	0.86863	0.92888		-1.038	0.0096456	0.049043	DOWN
transcript_HQ_CoA_transcript3446/f2p0/4274	1122.57	1479.036667	2536.563333	0.53772	0.42333	0.59754		0.63826	0.29849	0.48836		1.1658	0.0015658	0.012628	UP
transcript_HQ_CoA_transcript3486/f2p0/4272	7.983333333	3.94	3.923333333	-0.87557	0.0030217	0.013072		-0.14358	0.61645	0.76962		-1.0299	0.00001196	0.00024611	DOWN

transcript_HQ_CoA_transcript3489/f2p0/4305	35.65	25.09	4.326666667	-0.35085	0.77404	0.86631		-2.6569	1.3047E-06	2.3747E-05	DOWN	-3.0236	0.0062225	0.035882	DOWN
transcript_HQ_CoA_transcript3539/f2p0/4247	3.78	4.75	9.43	0.47786	0.044574	0.11458		0.83871	0.011704	0.045459		1.305	6.0281E-05	0.00093688	UP
transcript_HQ_CoA_transcript3553/f3p0/4264	4.296666667	2.82	1.696666667	-0.45927	0.18642	0.33744		-0.87454	0.0025681	0.013624		-1.3464	3.3659E-05	0.00058394	DOWN
transcript_HQ_CoA_transcript3574/f2p0/4248	4.653333333	5.626666667	9.76	0.44453	0.31373	0.48603		0.61952	0.12309	0.26487		1.0524	0.0080417	0.043021	UP
transcript_HQ_CoA_transcript358/f3p0/6796	4.496666667	2.656666667	9.383333333	-0.60981	0.034526	0.093582		1.6732	4.937E-07	1.0449E-05	UP	1.0518	0.00039239	0.0042491	UP
transcript_HQ_CoA_transcript3584/f2p0/4255	11.05666667	5.573333333	2.166666667	-0.84596	0.048983	0.12328		-1.5199	0.00078787	0.0052723	DOWN	-2.3744	5.9067E-07	2.0048E-05	DOWN
transcript_HQ_CoA_transcript3600/f2p0/4290	0.456666667	0.093333333	3.45	-2.1333	0.076007	0.17348		5.0839	2.4517E-12	1.6957E-10	UP	2.9366	3.7064E-10	2.8827E-08	UP
transcript_HQ_CoA_transcript3617/f2p0/4253	2.816666667	2.436666667	0.78	-0.044302	0.89273	0.94123		-1.7975	3.025E-07	6.7045E-06	DOWN	-1.8537	8.5076E-08	3.781E-06	DOWN
transcript_HQ_CoA_transcript3648/f2p0/4168	3.31	2.593333333	12.57666667	-0.20701	0.69048	0.8131		2.1289	9.442E-20	2.1633E-17	UP	1.9121	2.6733E-05	0.00047965	UP
transcript_HQ_CoA_transcript3656/f3p0/4220	20.67333333	10.85333333	4.183333333	-0.80594	0.1914	0.34329		-1.5002	0.01717	0.061168		-2.3159	7.9542E-24	6.0833E-21	DOWN
transcript_HQ_CoA_transcript3673/f2p0/4210	2.16	3.223333333	0.936666667	0.7154	0.23884	0.40213		-1.9126	0.0024784	0.013213	DOWN	-1.2069	0.0012885	0.010959	DOWN
transcript_HQ_CoA_transcript3683/f2p0/4196	1.516666667	0.926666667	6.133333333	-0.5462	0.33367	0.5081		2.5715	1.148E-10	5.5517E-09	UP	2.0111	7.4033E-05	0.0011075	UP
transcript_HQ_CoA_transcript3694/f2p0/4206	3.52	1.756666667	1.4	-0.84029	0.0094859	0.033748		-0.47151	0.24954	0.43279		-1.3234	0.0010879	0.0095773	DOWN
transcript_HQ_CoA_transcript37/f2p0/8561	2.45	2.686666667	5.8	0.33317	0.63377	0.77453		0.9346	0.17455	0.33911		1.2531	0.00027456	0.0032284	UP
transcript_HQ_CoA_transcript3732/f2p0/4200	6.54	5.593333333	0.913333333	-0.076919	0.85191	0.91648		-2.755	2.5629E-09	9.086E-08	DOWN	-2.8429	2.4495E-15	5.839E-13	DOWN
transcript_HQ_CoA_transcript3770/f3p0/4206	6.09	5.453333333	0.343333333	-0.031027	0.97076	0.98508		-4.0982	3.2713E-05	0.00037711	DOWN	-4.1536	3.7072E-18	1.3609E-15	DOWN
transcript_HQ_CoA_transcript3786/f2p0/4259	4.703333333	1.593333333	1.906666667	-1.4198	0.036401	0.097522		0.13499	0.85308	0.91987		-1.3007	0.00023585	0.0028406	DOWN
transcript_HQ_CoA_transcript3797/f2p0/4173	2.753333333	2.813333333	7.7	0.19416	0.71146	0.82675		1.3219	3.7832E-05	0.00042725	UP	1.5017	0.0011444	0.009945	UP
transcript_HQ_CoA_transcript3799/f2p0/4181	5.396666667	4.626666667	1.28	-0.054488	0.90846	0.94949		-2.001	4.3351E-12	2.7647E-10	DOWN	-2.067	3.3627E-05	0.00058394	DOWN
transcript_HQ_CoA_transcript3823/f2p0/4162	2.55	0.953333333	0.933333333	-1.2566	0.036185	0.097027		-0.19234	0.74528	0.85612		-1.4619	0.0023164	0.017068	DOWN
transcript_HQ_CoA_transcript3897/f2p0/4157	4.806666667	2.963333333	2.326666667	-0.54811	0.11054	0.22896		-0.4873	0.16682	0.32819		-1.0478	7.5122E-05	0.001121	DOWN
transcript_HQ_CoA_transcript3906/f3p0/4157	5.933333333	8.966666667	1.243333333	0.77603	0.10486	0.22022		-3.0326	5.6382E-10	2.3276E-08	DOWN	-2.2709	4.9382E-13	7.6814E-11	DOWN
transcript_HQ_CoA_transcript3928/f2p0/4203	1.756666667	1.176666667	0.193333333	-0.37392	NA	NA		-2.8316	NA	NA	NA	-3.2308	1.4175E-08	7.7491E-07	DOWN
transcript_HQ_CoA_transcript3956/f3p0/4125	5.293333333	10.75333333	1.816666667	1.195	0.038409	0.1018		-2.7102	4.4529E-25	2.2059E-22	DOWN	-1.5299	0.0088329	0.046033	DOWN
transcript_HQ_CoA_transcript3958/f4p0/4134	22.83666667	12.58	8.516666667	-0.68714	0.013186	0.043935		-0.72524	0.0081027	0.034069		-1.4251	3.7461E-13	6.0316E-11	DOWN
transcript_HQ_CoA_transcript3962/f2p0/4126	7.016666667	5.11	3.283333333	-0.2984	0.43403	0.60799		-0.78583	0.01194	0.046131		-1.0991	0.0093339	0.047843	DOWN
transcript_HQ_CoA_transcript3992/f2p0/4162	4.693333333	6.476666667	9.773333333	0.63446	0.16423	0.30782		0.42719	0.25038	0.43385		1.0507	0.00056995	0.0057103	UP
transcript_HQ_CoA_transcript4023/f2p0/4121	23.54	20.47333333	11.00666667	-0.039501	0.89417	0.94194		-1.0511	0.00033956	0.002635	DOWN	-1.103	7.9524E-07	2.5825E-05	DOWN
transcript_HQ_CoA_transcript4031/f2p0/4154	1.633333333	1.246666667	4.216666667	-0.24566	0.62134	0.76561		1.6162	2.6412E-05	0.00030993	UP	1.3599	0.00070633	0.0067981	UP
transcript_HQ_CoA_transcript4037/f2p0/4152	3.136666667	5.243333333	0.766666667	0.90666	0.076975	0.17507		-2.9048	1.1272E-19	2.5507E-17	DOWN	-2.0125	0.00025689	0.0030539	DOWN
transcript_HQ_CoA_transcript4041/f2p0/4116	4.356666667	4.066666667	1.553333333	0.038004	0.9461	0.96992		-1.5115	0.0067008	0.029411	DOWN	-1.4844	0.00023547	0.0028378	DOWN
transcript_HQ_CoA_transcript41/f2p0/8430	13.69	9.253333333	4.51	-0.41823	0.27239	0.44092		-1.1623	0.00096343	0.0061982	DOWN	-1.5944	1.7246E-06	4.9851E-05	DOWN
transcript_HQ_CoA_transcript4115/f2p0/4079	13.34333333	7.503333333	3.303333333	-0.70439	0.39573	0.57061		-1.2856	0.23814	0.41974		-2.006	0.0075763	0.041179	DOWN
transcript_HQ_CoA_transcript4117/f2p0/4146	16.57333333	10.52333333	7.363333333	-0.53358	0.31966	0.49267		-0.64553	0.12535	0.26865		-1.1881	0.0029831	0.020662	DOWN
transcript_HQ_CoA_transcript4121/f2p0/4099	2.453333333	1.463333333	1.18	-0.57357	0.13459	0.26577		-0.47455	0.18228	0.34966		-1.0626	0.0056127	0.033243	DOWN
transcript_HQ_CoA_transcript4146/f3p0/4065	3.65	5.476666667	10.16	0.75237	0.010897	0.037789		0.73789	0.0050285	0.02356		1.4771	5.6473E-09	3.421E-07	UP
transcript_HQ_CoA_transcript4148/f2p0/4088	4.896666667	4.803333333	1.373333333	0.092717	0.90605	0.94827		-1.9092	0.019087	0.066263		-1.8309	3.6394E-08	1.7909E-06	DOWN
transcript_HQ_CoA_transcript416/f2p0/6586	19.49666667	27.55	8.943333333	0.65486	0.0045741	0.018415		-1.7684	6.2401E-12	3.9036E-10	DOWN	-1.1262	2.039E-08	1.0724E-06	DOWN
transcript_HQ_CoA_transcript4190/f5p0/4051	4.71	6.126666667	11.34	0.51152	0.21412	0.37215		0.75399	0.021191	0.071664		1.2539	3.3165E-05	0.00057865	UP
transcript_HQ_CoA_transcript4198/f3p0/4080	3.33	3.376666667	1.03	0.15288	0.81543	0.89225		-1.8424	0.018699	0.065147		-1.7055	0.0023035	0.017007	DOWN
transcript_HQ_CoA_transcript4234/f2p0/4045	6.326666667	6.996666667	2.973333333	0.2715	0.65058	0.78653		-1.362	0.013714	0.051486		-1.1001	0.0039478	0.025613	DOWN
transcript_HQ_CoA_transcript4294/f2p0/4067	0.623333333	2.123333333	2.93	1.9925	0.022424	0.066604		0.26264	0.71716	0.83822		2.2349	0.00070427	0.0067822	UP
transcript_HQ_CoA_transcript4297/f2p0/4053	3.376666667	3.26	0.28	0.094386	0.81323	0.89108		-3.6642	1.4704E-09	5.5682E-08	DOWN	-3.579	5.6484E-12	7.0528E-10	DOWN
transcript_HQ_CoA_transcript4311/f2p0/4046	3.836666667	4.44	1.01	0.31805	0.7076	0.82416		-2.2586	0.00031879	0.0025003	DOWN	-1.9494	0.0074221	0.040535	DOWN
transcript_HQ_CoA_transcript4328/f2p0/4073	1.806666667	6.753333333	0.256666667	1.9834	0.068257	0.15974		-4.8312	3.1493E-06	5.0768E-05	DOWN	-2.8507	0.00090502	0.0082563	DOWN
transcript_HQ_CoA_transcript4359/f2p0/4023	10.61666667	3.96	4.84	-1.2975	0.023368	0.068907		0.15414	0.80275	0.89092		-1.1518	0.00059511	0.0059205	DOWN
transcript_HQ_CoA_transcript4362/f3p0/4036	10.9	5.2	4.773333333	-0.90465	0.0010667	0.005423		-0.2671	0.30194	0.49228		-1.1844	0.00014148	0.0018873	DOWN
transcript_HQ_CoA_transcript4364/f2p0/4045	3.486666667	3.293333333	8.066666667	0.07835	0.86221	0.92283		1.1537	7.8405E-06	0.00010962	UP	1.2178	0.0023269	0.017098	UP
transcript_HQ_CoA_transcript4365/f2p0/4127	52.88333333	19.77666667	13.48	-1.2197	0.31741	0.49006		-0.74223	0.53298	0.70435		-1.9733	4.2454E-05	0.00069825	DOWN
transcript_HQ_CoA_transcript4397/f2p0/4000	6.176666667	2.416666667	1.49	-1.1808	0.038008	0.10088		-0.87311	0.12044	0.2606		-2.0639	2.9743E-07	1.1187E-05	DOWN
transcript_HQ_CoA_transcript4428/f3p0/4085	11.56666667	10.23666667	2.05	-0.030509	0.97271	0.98617		-2.4221	0.0017284	0.0099002	DOWN	-2.4676	0.00016496	0.0021278	DOWN
transcript_HQ_CoA_transcript4436/f2p0/4054	3.1	2.746666667	0.783333333	-0.066852	0.93563	0.96404		-1.9501	0.016693	0.059875		-2.0237	9.7014E-05	0.0013836	DOWN
transcript_HQ_CoA_transcript4480/f5p0/3890	1.076666667	1.85	3.533333333	0.97047	0.15045	0.28841		0.7548	0.16218	0.32226		1.7113	2.5821E-05	0.00046616	UP
transcript_HQ_CoA_transcript4487/f2p0/4025	3.886666667	4.26	8.82	0.28623	0.56016	0.71822		0.89843	0.00099954	0.0063835		1.1753	0.0070869	0.039192	UP
transcript_HQ_CoA_transcript4495/f2p0/4042	18.33	18.1	42.54	0.13989	0.51502	0.68086		1.0763	1.2391E-12	9.3082E-11	UP	1.2039	1.5862E-10	1.3417E-08	UP
transcript_HQ_CoA_transcript4503/f2p0/3994	1.103333333	0.606666667	0.026666667	-0.66755	0.63861	0.77782		-5.0149	0.0067361	0.029537	DOWN	-5.6866	0.0003864	0.0042066	DOWN
transcript_HQ_CoA_transcript4520/f3p0/4005	18.64	41.05333333	1.633333333	1.2866	0.25674	0.42305		-4.777	1.6149E-13	1.4231E-11	DOWN	-3.5011	3.6541E-06	9.2767E-05	DOWN
transcript_HQ_CoA_transcript4545/f2p0/4006	32.64333333	25.14	12.98	-0.20881	0.35143	0.52738		-1.1089	5.6225E-09	1.8568E-07	DOWN	-1.3314	4.4094E-12	5.6205E-10	DOWN
transcript_HQ_CoA_transcript4578/f2p0/3987	5.943333333	5	2.243333333	-0.047313	0.93366	0.96289		-1.3375	0.012829	0.048835	DOWN	-1.3995	0.0016461	0.013135	DOWN
transcript_HQ_CoA_transcript4630/f2p0/3917	4.433333333	6.303333333	24.41	0.73519	NA	NA		1.7336	0.19074	0.36037		2.4543	6.1097E-10	4.5402E-08	UP
transcript_HQ_CoA_transcript4663/f2p0/4005	9.07	7.81	3.906666667	-0.041059	0.92303	0.9576		-1.1734	0.0055383	0.025378	DOWN	-1.2308	0.0050512	0.030751	DOWN
transcript_HQ_CoA_transcript4676/f3p0/3957	1.893333333	1.563333333	5.26	-0.12403	0.77437	0.86646		1.6035	2.4337E-07	5.5344E-06	UP	1.469	7.2562E-05	0.0010926	UP
transcript_HQ_CoA_transcript4693/f2p0/3975	10.42	9.006666667	4.463333333	-0.068781	0.83568	0.90601		-1.1506	0.00010215	0.00098443	DOWN	-1.2311	5.2194E-07	1.8076E-05	DOWN
transcript_HQ_CoA_transcript4694/f2p0/3975	19.87	13.37	10.03666667	-0.41634	0.083405	0.18564		-0.57361	0.017603	0.062204		-1.0013	5.7409E-05	0.00089909	DOWN
transcript_HQ_CoA_transcript4716/f3p0/3995	21.09333333	10.6	3.54	-0.8912	0.29145	0.46164		-1.7111	0.0021551	0.01177	DOWN	-2.6085	0.0002087	0.0025773	DOWN
transcript_HQ_CoA_transcript4789/f2p0/3953	0.076666667	0.123333333	0.94	0.85648	0.52148	0.6864		2.7917	0.0035678	0.017897	UP	3.6347	0.00092198	0.0083777	UP
transcript_HQ_CoA_transcript4840/f2p0/4025	0.41	0.993333333	1.996666667	1.4456	0.024508	0.071529		0.85605	0.0069207	0.030114		2.2886	3.6408E-05	0.00061909	UP
transcript_HQ_CoA_transcript4853/f5p0/3929	21.80333333	11.94	10.68	-0.72237	0.006353	0.024157		-0.30367	0.18587	0.35407		-1.0372	0.00011852	0.0016381	DOWN
transcript_HQ_CoA_transcript4858/f3p0/3947	2.956666667	4.416666667	0	0.6954	0.36355	0.53883		-10.14	1.7237E-12	1.2537E-10	DOWN	-9.4549	8.6559E-12	1.0056E-09	DOWN
transcript_HQ_CoA_transcript4882/f3p0/3916	0.876666667	0.903333333	0.293333333	0.19541	0.69967	0.8194		-1.7614	0.00048036	0.0035374	DOWN	-1.5785	0.0069962	0.038879	DOWN
transcript_HQ_CoA_transcript4895/f2p0/3916	13.59333333	13.8	3.903333333	0.18751	0.63853	0.77777		-1.9822	1.1392E-06	2.1199E-05	DOWN	-1.8083	4.9483E-09	3.0478E-07	DOWN
transcript_HQ_CoA_transcript4915/f2p0/3908	5.27	3.39	1.546666667	-0.44541	0.50398	0.67093		-1.2909	0.00037328	0.0028629	DOWN	-1.7518	0.0061848	0.035755	DOWN
transcript_HQ_CoA_transcript492/f3p0/6398	5.62	2.48	18.99333333	-0.98389	0.076872	0.17493		2.7475	7.8789E-08	1.9782E-06	UP	1.7502	1.3936E-15	3.5039E-13	UP
transcript_HQ_CoA_transcript4931/f2p0/3909	1.723333333	1.546666667	3.663333333	-0.0016353	0.99672	0.99814		1.1097	0.00028706	0.0022986	UP	1.0949	0.0013007	0.011042	UP
transcript_HQ_CoA_transcript4960/f2p0/3881	1.53	2.363333333	0.71	0.78578	0.010727	0.037291		-1.89	4.3817E-09	1.49E-07	DOWN	-1.1167	0.0054441	0.032507	DOWN
transcript_HQ_CoA_transcript4980/f3p0/3860	7.046666667	13.82	2.64	1.1021	0.15375	0.29341		-2.491	0.0027377	0.014312	DOWN	-1.4016	0.00074868	0.0071092	DOWN
transcript_HQ_CoA_transcript4986/f2p0/3899	6.09	4.966666667	0.7	-0.1013	0.84706	0.91357		-3.0123	6.1698E-07	1.2621E-05	DOWN	-3.1314	6.5002E-12	7.8495E-10	DOWN
transcript_HQ_CoA_transcript4997/f2p0/3883	5.853333333	3.71	2.64	-0.50419	0.022091	0.065777		-0.63799	0.062481	0.16207		-1.153	0.00069315	0.0066892	DOWN
transcript_HQ_CoA_transcript501/f5p0/6313	0.436666667	0.063333333	2.336666667	-2.6268	0.038652	0.10222		5.0611	1.0399E-08	3.2638E-07	UP	2.4113	2.4665E-10	1.9813E-08	UP

transcript_HQ_CoA_transcript5068/f2p0/3890	1.296666667	1.066666667	6.183333333	-0.093387	0.91504	0.95323		2.341	0.00094068	0.0060778	UP	2.2363	6.9818E-07	2.3132E-05	UP
transcript_HQ_CoA_transcript5079/f2p0/3894	4.016666667	4.186666667	9.23	0.22356	0.60664	0.75499		0.98875	0.00023585	0.0019552		1.2006	0.0014351	0.011872	UP
transcript_HQ_CoA_transcript5119/f4p0/3856	0	0.796666667	3.7	21.71	NA	NA	NA	2.0051	0.43683	0.62547		9.5986	1.8342E-14	3.7828E-12	UP
transcript_HQ_CoA_transcript5122/f3p0/3871	8.58	5.4	2.56	-0.50897	0.044585	0.11458		-1.2215	3.1245E-06	5.0457E-05	DOWN	-1.743	2.557E-09	1.6585E-07	DOWN
transcript_HQ_CoA_transcript5161/f2p0/3863	6.39	3.363333333	3.05	-0.76181	0.026005	0.074952		-0.30195	0.39567	0.5859		-1.0769	0.0001284	0.0017483	DOWN
transcript_HQ_CoA_transcript5164/f2p0/3867	6.686666667	6.28	1.74	0.05519	0.90092	0.94534		-1.997	1.1091E-05	0.00014836	DOWN	-1.956	3.7764E-07	1.3699E-05	DOWN
transcript_HQ_CoA_transcript5168/f3p0/3849	1.453333333	1.33	6.83	0.02476	0.96839	0.98362		2.2164	1.4502E-07	3.4701E-06	UP	2.2291	2.2057E-05	0.00040812	UP
transcript_HQ_CoA_transcript5195/f2p0/3866	6.216666667	4.113333333	2.506666667	-0.38623	0.61096	0.75767		-0.91879	0.24136	0.42361		-1.3194	6.1472E-06	0.00014264	DOWN
transcript_HQ_CoA_transcript5201/f2p0/3838	11.42666667	5.816666667	4.846666667	-0.78626	0.10296	0.21713		-0.4328	0.31315	0.50463		-1.2333	0.00020124	0.0024984	DOWN
transcript_HQ_CoA_transcript521/f2p0/6387	5.63	6.15	19.36666667	0.32878	0.741	0.84484		1.4588	0.25311	0.437		1.7751	8.2612E-06	0.00018183	UP
transcript_HQ_CoA_transcript5236/f4p0/3787	33.69666667	23.17666667	10.51666667	-0.38111	0.52188	0.68663		-1.2741	0.00013132	0.0012059	DOWN	-1.6707	0.0033084	0.022366	DOWN
transcript_HQ_CoA_transcript5246/f2p0/3833	4.13	6.413333333	9.44	0.79709	0.008395	0.030482		0.39385	0.17246	0.33592		1.178	7.9273E-07	2.5799E-05	UP
transcript_HQ_CoA_transcript5258/f2p0/3841	8.416666667	9.133333333	2.983333333	0.27149	0.23345	0.39576		-1.7635	3.051E-12	2.0409E-10	DOWN	-1.5042	2.5215E-07	9.7453E-06	DOWN
transcript_HQ_CoA_transcript5299/f2p0/3825	3.37	1.743333333	1.66	-0.81186	0.031655	0.087367		-0.21439	0.4987	0.67713		-1.0354	0.0038568	0.025183	DOWN
transcript_HQ_CoA_transcript5363/f2p0/3841	4.456666667	8.016666667	21.84333333	1.0209	0.13422	0.2652		1.2972	0.0001199	0.0011144	UP	2.3035	0.00033594	0.0037576	UP
transcript_HQ_CoA_transcript5372/f2p0/3828	1.63	0.853333333	7.16	-0.79418	0.20576	0.36146		2.928	2.7188E-11	1.4589E-09	UP	2.1218	1.2759E-09	8.7061E-08	UP
transcript_HQ_CoA_transcript5401/f2p0/3810	8.54	5.15	3.24	-0.54011	0.28664	0.4566		-0.84764	0.13891	0.28893		-1.4043	0.00010975	0.0015366	DOWN
transcript_HQ_CoA_transcript5433/f7p0/3763	1.346666667	2.343333333	8.823333333	1.0059	NA	NA	NA	1.7034	0.069354	0.17471		2.6977	6.1899E-13	9.3897E-11	UP
transcript_HQ_CoA_transcript5462/f2p0/3814	3.373333333	4.54	8.163333333	0.56594	0.093327	0.20166		0.71687	0.022651	0.075566		1.2726	0.00028239	0.003291	UP
transcript_HQ_CoA_transcript5464/f2p0/3827	5.606666667	3.85	2.796666667	-0.35091	0.42424	0.59821		-0.64397	0.18992	0.35933		-1.0105	0.0019694	0.015119	DOWN
transcript_HQ_CoA_transcript5476/f2p0/3812	1.94	2.39	9.363333333	0.51752	0.72856	0.83781		1.7506	0.33496	0.52676		2.2563	4.3998E-07	0.00001562	UP
transcript_HQ_CoA_transcript5501/f2p0/3780	1.296666667	0.906666667	4.68	-0.34417	0.48223	0.65253		2.2035	2.3084E-08	6.6317E-07	UP	1.8475	1.7534E-05	0.00033826	UP
transcript_HQ_CoA_transcript5533/f2p0/3831	2.83	1.243333333	0.686666667	-1.0347	0.13563	0.26743		-0.99564	0.098054	0.22538		-2.045	0.0015434	0.01248	DOWN
transcript_HQ_CoA_transcript5565/f2p0/3775	6.43	2.84	13.46666667	-1.0052	0.035275	0.095243		2.0699	7.5535E-06	0.00010658	UP	1.0533	6.2778E-06	0.00014468	UP
transcript_HQ_CoA_transcript558/f2p0/6265	1.806666667	0.966666667	0.473333333	-0.74867	0.1895	0.34111		-1.1626	0.0079812	0.033699	DOWN	-1.9223	0.00072409	0.0069331	DOWN
transcript_HQ_CoA_transcript5601/f2p0/3773	3.923333333	5.316666667	16.34666667	0.60924	0.17252	0.31932		1.4895	1.7569E-05	0.00021787	UP	2.0849	7.275E-06	0.00016324	UP
transcript_HQ_CoA_transcript562/f3p0/6309	30.44	11.94333333	11.05333333	-1.1665	0.039566	0.10415		-0.28363	0.61984	0.77197		-1.4625	4.628E-06	0.00011222	DOWN
transcript_HQ_CoA_transcript5700/f5p0/3747	1.42	14.21333333	7.443333333	3.4519	0.12406	0.24973		-1.0493	0.60469	0.76092		2.3916	0.0069351	0.038633	UP
transcript_HQ_CoA_transcript5704/f2p0/3761	5.643333333	5.52	2.6	0.1388	0.64086	0.77943		-1.2443	8.7929E-07	1.7109E-05	DOWN	-1.1193	6.4829E-05	0.00099911	DOWN
transcript_HQ_CoA_transcript5720/f2p0/3739	4.95	2.543333333	1.38	-0.81566	0.11665	0.23852		-1.0108	0.07679	0.18842		-1.837	1.6134E-06	4.6932E-05	DOWN
transcript_HQ_CoA_transcript5726/f2p0/3775	0.533333333	0.123333333	1.72	-2.0767	0.14845	0.28587		3.7267	0.0010947	0.0068712	UP	1.6449	0.00031212	0.0035517	UP
transcript_HQ_CoA_transcript5749/f10p0/3698	10.10333333	14.43	2.27	0.64303	0.23914	0.4025		-2.7939	7.9169E-07	1.5721E-05	DOWN	-2.1627	1.2539E-21	6.7692E-19	DOWN
transcript_HQ_CoA_transcript5789/f2p0/3732	7.443333333	5.28	3.296666667	-0.34306	0.21519	0.3735		-0.83675	0.0056187	0.025689		-1.1909	0.00048546	0.0050502	DOWN
transcript_HQ_CoA_transcript5799/f2p0/3738	9.076666667	8.483333333	2.916666667	0.02602	0.96046	0.97888		-1.6653	0.0008719	0.0057198	DOWN	-1.6501	1.4629E-05	0.00029091	DOWN
transcript_HQ_CoA_transcript5825/f2p0/3791	5.27	2.046666667	0.516666667	-1.2137	0.21667	0.37516		-2.1404	0.019685	0.067831		-3.3683	1.3631E-06	4.1017E-05	DOWN
transcript_HQ_CoA_transcript5839/f2p0/3734	27.47	23.31	6.806666667	-0.10602	0.78845	0.87633		-1.9086	3.4159E-07	7.4714E-06	DOWN	-2.0252	3.8462E-06	9.6841E-05	DOWN
transcript_HQ_CoA_transcript5889/f2p0/3731	3.31	2.11	10.72333333	-0.51847	0.30123	0.47236		2.1985	9.0274E-07	1.7546E-05	UP	1.6704	1.3573E-05	0.00027346	UP
transcript_HQ_CoA_transcript5913/f2p0/3713	7.796666667	5.733333333	3.26	-0.29986	0.28933	0.4596		-0.96143	0.0010967	0.006882		-1.2734	4.3437E-06	0.00010659	DOWN
transcript_HQ_CoA_transcript5938/f2p0/3741	3.593333333	2.83	0.26	-0.19283	0.85554	0.91855		-3.5872	3.2409E-05	0.00037454	DOWN	-3.7966	7.7546E-05	0.0011507	DOWN
transcript_HQ_CoA_transcript5970/f2p0/3710	10.97333333	16.27	53.48666667	0.76741	0.25104	0.41671		1.533	0.0010822	0.0068143	UP	2.2851	2.1718E-05	0.00040266	UP
transcript_HQ_CoA_transcript5989/f5p0/3674	3.656666667	2.336666667	1.236666667	-0.48617	0.15096	0.28916		-1.0689	0.014524	0.053802		-1.5695	8.1407E-05	0.0011983	DOWN
transcript_HQ_CoA_transcript5999/f2p0/3694	4.746666667	4.963333333	1.76	0.20379	0.7704	0.86418		-1.5985	0.032596	0.099146		-1.4096	0.0035267	0.023496	DOWN
transcript_HQ_CoA_transcript606/f2p0/6291	8.996666667	10.60666667	3.393333333	0.40182	0.14534	0.28145		-1.7943	8.5996E-32	7.1646E-29	DOWN	-1.4052	6.6221E-07	0.0000221	DOWN
transcript_HQ_CoA_transcript6082/f3p0/3685	3.75	2.733333333	13.52666667	-0.29818	0.52754	0.69136		2.1545	1.9856E-05	0.00024214	UP	1.8421	0.00041666	0.004455	UP
transcript_HQ_CoA_transcript6105/f2p0/3688	6.17	8.276666667	12.81	0.57025	0.2166	0.37513		0.49453	0.234	0.41455		1.0531	0.0069325	0.03863	UP
transcript_HQ_CoA_transcript6116/f2p0/3667	0.503333333	0.196666667	5.376666667	-1.1984	0.48352	0.65354		4.6494	1.0692E-06	2.0062E-05	UP	3.4422	7.0161E-05	0.0010643	UP
transcript_HQ_CoA_transcript6164/f2p0/3684	5.82	4.353333333	2.673333333	-0.26789	0.39361	0.56882		-0.85318	0.00051159	0.0037195		-1.1315	0.00015947	0.0020774	DOWN
transcript_HQ_CoA_transcript6165/f2p0/3675	5.946666667	2.966666667	1.083333333	-0.87562	0.16913	0.3144		-1.5565	0.068907	0.17387		-2.4484	3.4057E-05	0.00058917	DOWN
transcript_HQ_CoA_transcript6215/f2p0/3674	12.24333333	8.07	5.86	-0.43404	0.27254	0.44109		-0.63768	0.14639	0.29965		-1.0814	0.0025284	0.018203	DOWN
transcript_HQ_CoA_transcript6220/f2p0/3658	25.66666667	15.41333333	10.24333333	-0.60373	0.14146	0.27591		-0.71949	0.015757	0.057201		-1.3328	0.00021071	0.0025957	DOWN
transcript_HQ_CoA_transcript6254/f3p0/3651	1.9	1.633333333	11.17	-0.072636	0.86736	0.92556		2.6192	1.3934E-13	1.2519E-11	UP	2.5356	1.4268E-13	2.4248E-11	UP
transcript_HQ_CoA_transcript6273/f2p0/3672	5.763333333	10.96666667	12.9	1.0541	0.054127	0.13318		0.11466	0.81559	0.89958		1.1585	0.0087281	0.04563	UP
transcript_HQ_CoA_transcript6275/f2p0/3657	13.32666667	9.773333333	5.143333333	-0.30971	0.437	0.61076		-1.0621	0.0063258	0.028143	DOWN	-1.3828	6.3724E-08	2.9315E-06	DOWN
transcript_HQ_CoA_transcript6310/f2p0/3620	2.656666667	5.223333333	9.233333333	1.1243	0.033702	0.091838		0.67972	0.15624	0.31405		1.7901	1.6367E-06	4.7458E-05	UP
transcript_HQ_CoA_transcript6331/f3p0/3636	0.793333333	1.126666667	2.646666667	0.67843	0.44614	0.61924		1.048	0.16699	0.32844		1.7177	0.00019754	0.0024716	UP
transcript_HQ_CoA_transcript6338/f2p0/3657	7.356666667	8.18	1.8	0.28596	0.62993	0.77179		-2.3226	0.00006836	0.00069803	DOWN	-2.0472	8.0955E-08	3.6331E-06	DOWN
transcript_HQ_CoA_transcript6342/f5p0/3629	2.823333333	3.936666667	0.196666667	0.63519	0.46264	0.63445		-4.478	1.0413E-17	1.7672E-15	DOWN	-3.8517	4.6036E-05	0.00074779	DOWN
transcript_HQ_CoA_transcript6350/f2p0/3661	5.976666667	5.573333333	0.433333333	0.041327	0.89395	0.94186		-3.8406	1.5137E-20	3.9634E-18	DOWN	-3.8131	3.6403E-25	3.5168E-22	DOWN
transcript_HQ_CoA_transcript6370/f2p0/3621	3.586666667	4.42	17.78333333	0.45738	0.13746	0.27021		1.8503	8.4231E-09	2.6711E-07	UP	2.2975	9.2584E-11	8.3713E-09	UP
transcript_HQ_CoA_transcript6371/f3p0/3621	4.2	2.95	9	-0.31545	0.63363	0.77453		1.4152	0.026669	0.085586		1.0881	2.0516E-05	0.00038346	UP
transcript_HQ_CoA_transcript6373/f6p0/3614	4.606666667	5.773333333	9.69	0.47563	0.23122	0.39285		0.60657	0.061223	0.1594		1.0695	0.0028562	0.020013	UP
transcript_HQ_CoA_transcript6419/f7p0/3589	14.73	4.31	31.94333333	-1.5469	0.45724	0.62952		2.6609	0.2031	0.37671		1.1015	0.0026239	0.018683	UP
transcript_HQ_CoA_transcript6439/f4p0/3581	11.22	7.066666667	5.45	-0.51415	0.020197	0.061183		-0.51775	0.095831	0.22147		-1.0452	0.0004682	0.0049093	DOWN
transcript_HQ_CoA_transcript6481/f2p0/3609	28.57666667	21.02666667	6.32	-0.28854	0.64639	0.78359		-1.8694	1.4778E-05	0.00018836	DOWN	-2.174	4.8413E-05	0.00077881	DOWN
transcript_HQ_CoA_transcript6487/f2p0/3622	2.636666667	2.413333333	10.84	0.035736	0.94191	0.96769		2.0535	0.0092137	0.037562	UP	2.0731	0.0091276	0.047035	UP
transcript_HQ_CoA_transcript6497/f3p0/3579	3.033333333	1.436666667	0.53	-0.9114	0.32771	0.50127		-1.5902	0.0011566	0.0071765	DOWN	-2.5188	0.005355	0.032132	DOWN
transcript_HQ_CoA_transcript6510/f2p0/3605	2.363333333	0.796666667	8.46	-1.3503	0.159	0.30079		3.2283	0.00011668	0.0010889	UP	1.8603	0.00020336	0.0025187	UP
transcript_HQ_CoA_transcript6534/f3p0/3610	27.13	33.44666667	10.23666667	0.46599	0.351	0.52685		-1.839	4.3769E-06	6.7021E-05	DOWN	-1.388	0.0018436	0.014345	DOWN
transcript_HQ_CoA_transcript6560/f2p0/3618	5.676666667	5.73	11.63333333	0.19147	0.65108	0.78687		0.84834	0.034114	0.10257		1.0279	1.7895E-05	0.00034394	UP
transcript_HQ_CoA_transcript6633/f2p0/3584	13.49333333	5.873333333	6.236666667	-1.0731	0.051257	0.1279		-0.047919	0.92867	0.9626		-1.1303	0.00012904	0.0017519	DOWN
transcript_HQ_CoA_transcript6680/f13p0/3559	0.153333333	0.393333333	1.16	1.5132	0.16985	0.31547		1.4187	0.0056302	0.025728	UP	2.9303	0.00078695	0.0073885	UP
transcript_HQ_CoA_transcript6706/f2p0/3567	13.99666667	10.96	1.923333333	-0.20097	0.81672	0.89334		-2.634	0.00038964	0.0029658	DOWN	-2.8519	5.4062E-06	0.00012797	DOWN
transcript_HQ_CoA_transcript6714/f2p0/3520	4.293333333	2.166666667	1.796666667	-0.8374	0.0035876	0.015137		-0.40702	0.25873	0.44332		-1.2566	0.00023046	0.0027926	DOWN
transcript_HQ_CoA_transcript6717/f2p0/3563	2.74	2.106666667	6.643333333	-0.22372	0.50965	0.67575		1.499	1.8116E-07	4.2192E-06	UP	1.2632	8.2095E-06	0.00018111	UP
transcript_HQ_CoA_transcript6742/f2p0/3598	13.84333333	11.11	2.17	-0.17489	0.60737	0.75533		-2.4901	3.1259E-07	6.9114E-06	DOWN	-2.6798	4.0867E-12	5.32E-10	DOWN
transcript_HQ_CoA_transcript6751/f2p0/3567	2.183333333	5.663333333	5.296666667	1.5013	0.093949	0.20245		-0.20953	0.80721	0.8933		1.2791	0.00040128	0.0043149	UP
transcript_HQ_CoA_transcript6788/f2p0/3542	6.333333333	2.516666667	13.83666667	-1.1514	0.016073	0.05108		2.2779	2.3176E-06	3.9223E-05	UP	1.1142	0.00015184	0.001998	UP
transcript_HQ_CoA_transcript6789/f2p0/3541	9.74	5.936666667	4.71	-0.57187	0.17816	0.32689		-0.46727	0.27286	0.46035		-1.0511	3.7067E-05	0.00062726	DOWN
transcript_HQ_CoA_transcript6795/f2p0/3555	2.36	0.706666667	6.363333333	-1.5223	0.39813	0.57276		2.9481	NA	NA	NA	1.4132	0.0015581	0.012582	UP

transcript_HQ_CoA_transcript6830/f3p0/3622	14.55333333	13.87666667	7.013333333	0.089693	0.70717	0.82415	-1.1396	5.2495E-06	7.7909E-05	DOWN	-1.0624	1.9465E-08	1.0296E-06	DOWN
transcript_HQ_CoA_transcript6877/f2p0/3545	7.293333333	3.183333333	2.433333333	-1.0692	0.05075	0.12678	-0.52257	0.33837	0.53003		-1.6008	0.00016699	0.002148	DOWN
transcript_HQ_CoA_transcript6915/f2p0/3561	2.233333333	0.986666667	5.93	-1.0346	0.12435	0.2502	2.4592	3.951E-06	6.1633E-05	UP	1.4101	0.0020098	0.015333	UP
transcript_HQ_CoA_transcript7003/f2p0/3534	7.106666667	3.59	2.596666667	-0.83081	0.15796	0.29955	-0.59171	0.24808	0.4312		-1.4384	0.0029429	0.020438	DOWN
transcript_HQ_CoA_transcript7027/f2p0/3520	12.45333333	8.306666667	5.396666667	-0.44675	0.15861	0.30028	-0.74734	0.067189	0.17078		-1.2037	0.00045733	0.0048215	DOWN
transcript_HQ_CoA_transcript7052/f2p0/3539	5.703333333	4.103333333	2.766666667	-0.32873	0.47587	0.6472	-0.70365	0.10358	0.2345		-1.0451	0.0013257	0.011213	DOWN
transcript_HQ_CoA_transcript7067/f3p0/3538	1.336666667	3.41	5.18	1.5048	0.075262	0.17238	0.47562	0.44338	0.63182		1.9644	0.0013866	0.011595	UP
transcript_HQ_CoA_transcript7082/f4p0/3495	10.40333333	16.16	3.423333333	0.81776	0.11209	0.23156	-2.4164	1.3019E-06	2.3721E-05	DOWN	-1.6121	7.3579E-09	4.3287E-07	DOWN
transcript_HQ_CoA_transcript7086/f3p0/3545	10.37	8.14	3.786666667	-0.20405	0.49973	0.66721	-1.2515	9.6132E-08	2.3811E-06	DOWN	-1.4657	6.6782E-06	0.00015189	DOWN
transcript_HQ_CoA_transcript7144/f2p0/3481	4.186666667	5.71	0.15	0.561	0.56374	0.72144	-5.3813	7.0718E-06	0.00010095	DOWN	-4.8247	2.9797E-12	3.9922E-10	DOWN
transcript_HQ_CoA_transcript7151/f3p0/3480	13.65	8.72	6.29	-0.47876	0.11313	0.23331	-0.6207	0.0010047	0.006412		-1.1138	0.00029921	0.0034347	DOWN
transcript_HQ_CoA_transcript7152/f5p0/3528	2.73	2.833333333	5.89	0.17776	0.79481	0.88001	0.92732	0.14582	0.29863		1.0967	0.0060391	0.035212	UP
transcript_HQ_CoA_transcript7154/f3p0/3486	4.63	6.783333333	9.976666667	0.70904	0.015084	0.048784	0.39772	0.061169	0.15928		1.0956	2.2373E-06	6.2221E-05	UP
transcript_HQ_CoA_transcript7158/f2p0/3490	5.416666667	2.96	2.01	-0.66935	NA	NA	-0.77361	0.46409	0.64899		-1.4533	0.0015895	0.012774	DOWN
transcript_HQ_CoA_transcript7164/f2p0/3507	5.603333333	2.513333333	1.716666667	-1.0192	0.066529	0.15666	-0.70215	0.01437	0.053357		-1.7321	0.0015201	0.012335	DOWN
transcript_HQ_CoA_transcript7206/f4p0/3488	1.13	2.133333333	3.856666667	1.1064	0.081125	0.18205	0.66319	0.25487	0.43897		1.7584	4.8441E-06	0.00011638	UP
transcript_HQ_CoA_transcript7227/f2p0/3498	6.063333333	3.856666667	1.893333333	-0.48913	0.089536	0.1958	-1.1744	1.7959E-05	0.00022211	DOWN	-1.6777	5.9089E-07	2.0048E-05	DOWN
transcript_HQ_CoA_transcript7270/f2p0/3477	4.823333333	3.67	2.053333333	-0.25441	0.42835	0.60244	-0.96442	0.0024498	0.013083		-1.2309	1.2601E-05	0.00025585	DOWN
transcript_HQ_CoA_transcript7280/f3p0/3510	4.253333333	0.886666667	0.233333333	-2.13	0.061188	0.14675	-2.0213	0.16422	0.32479		-4.1684	0.0010301	0.0091648	DOWN
transcript_HQ_CoA_transcript7284/f3p0/3473	1.7	2.116666667	3.723333333	0.47876	0.18597	0.33695	0.66441	0.094217	0.21865		1.1327	0.0045141	0.028424	UP
transcript_HQ_CoA_transcript7367/f4p0/3456	14.48	6.06	2.913333333	-1.1418	0.10686	0.22317	-1.1666	0.11683	0.25526		-2.3189	9.7219E-19	3.7176E-16	DOWN
transcript_HQ_CoA_transcript7378/f2p0/3465	5.523333333	2.783333333	1.98	-0.84387	0.038611	0.10216	-0.64495	0.014372	0.053357		-1.4997	0.00016451	0.0021274	DOWN
transcript_HQ_CoA_transcript7380/f3p0/3459	5.766666667	11.89666667	25.25666667	1.2123	0.028892	0.081262	0.93731	0.012526	0.047944		2.1358	7.5667E-05	0.0011264	UP
transcript_HQ_CoA_transcript7382/f2p0/3458	2.606666667	2.813333333	5.233333333	0.29595	0.56991	0.726	0.72585	0.064848	0.16658		1.0073	0.0065809	0.037327	UP
transcript_HQ_CoA_transcript7407/f2p0/3450	2.626666667	3.443333333	6.88	0.54021	0.26007	0.42703	0.85899	0.0017302	0.0099072		1.3867	0.00054354	0.005518	UP
transcript_HQ_CoA_transcript742/f2p0/6026	2.75	1.08	0.86	-1.1789	0.033226	0.090851	-0.46875	0.20758	0.38238		-1.6598	0.0035994	0.023851	DOWN
transcript_HQ_CoA_transcript7577/f3p0/3440	6.866666667	6.29	13.83666667	0.010008	0.98444	0.9924	1.0143	0.028135	0.089111		1.01	0.0041065	0.026401	UP
transcript_HQ_CoA_transcript759/f2p0/5983	12.84666667	8.696666667	4.293333333	-0.42577	0.17844	0.32731	-1.1428	0.0059437	0.026866	DOWN	-1.5815	1.2152E-06	3.7238E-05	DOWN
transcript_HQ_CoA_transcript7600/f2p0/3458	4.713333333	1.826666667	1.973333333	-1.2466	0.15453	0.29459	-0.018131	0.98495	0.99242		-1.2736	0.0052906	0.03185	DOWN
transcript_HQ_CoA_transcript7621/f3p0/3430	2.683333333	2.06	0.046666667	-0.2333	0.7694	0.86406	-5.6243	1.4165E-06	2.5654E-05	DOWN	-5.8886	3.9219E-11	3.8291E-09	DOWN
transcript_HQ_CoA_transcript7622/f3p0/3447	6.87	10.61333333	16.34	0.7566	0.071278	0.16545	0.48261	0.080621	0.19503		1.2299	0.00056261	0.0056553	UP
transcript_HQ_CoA_transcript7645/f5p0/3402	16.15	8.033333333	5.603333333	-0.86034	0.0029901	0.012951	-0.66156	0.033106	0.10028		-1.5359	3.164E-08	1.5911E-06	DOWN
transcript_HQ_CoA_transcript7651/f2p0/3431	0.946666667	0.326666667	0.19	-1.3741	0.077556	0.17609	-0.90222	0.31243	0.50396		-2.2937	0.0051299	0.031117	DOWN
transcript_HQ_CoA_transcript7664/f2p0/3438	6.913333333	16.10333333	16.58333333	1.4139	0.0385	0.10198	-0.14675	0.82202	0.90291		1.2542	1.9297E-05	0.00036515	UP
transcript_HQ_CoA_transcript7681/f2p0/3505	5.286666667	2.973333333	2.266666667	-0.67837	0.12649	0.25368	-0.52433	0.20733	0.382		-1.2165	0.00068946	0.006671	DOWN
transcript_HQ_CoA_transcript7756/f4p0/3376	29.1	16.58666667	14.08333333	-0.64873	0.054493	0.13379	-0.38376	0.0056106	0.025658		-1.045	0.0013501	0.011357	DOWN
transcript_HQ_CoA_transcript7775/f3p0/3465	0.933333333	0.583333333	4.6	-0.50449	0.71167	0.82682	2.8417	0.0096144	0.038781	UP	2.3218	0.00055332	0.0055926	UP
transcript_HQ_CoA_transcript7798/f7p0/3353	16.37333333	20.70666667	1.856666667	0.4956	0.23794	0.401	-3.6201	3.6565E-34	3.7233E-31	DOWN	-3.1399	1.0812E-14	2.3626E-12	DOWN
transcript_HQ_CoA_transcript7807/f2p0/3416	10.69	5.023333333	3.8	-0.95568	0.19224	0.34436	-0.52421	0.48724	0.66805		-1.4912	6.3494E-06	0.00014586	DOWN
transcript_HQ_CoA_transcript7855/f3p0/3415	12.16666667	8.123333333	3.396666667	-0.44031	0.19482	0.34766	-1.3925	0.0000382	0.0004306	DOWN	-1.8413	7.3096E-06	0.00016362	DOWN
transcript_HQ_CoA_transcript7878/f2p0/3390	3.24	3.176666667	1.386666667	0.09974	0.86952	0.927	-1.3187	0.036515	0.10743		-1.232	0.00030241	0.0034606	DOWN
transcript_HQ_CoA_transcript7950/f3p0/3363	4.963333333	4.956666667	2.38	0.175	0.70413	0.8221	-1.2286	0.011489	0.044768	DOWN	-1.0681	0.0011249	0.0098264	DOWN
transcript_HQ_CoA_transcript7970/f11p0/3342	71.97666667	69.12666667	26.41333333	0.069809	0.91659	0.95423	-1.5252	0.007435	0.031922	DOWN	-1.4637	0.00099332	0.0089261	DOWN
transcript_HQ_CoA_transcript7994/f2p0/3361	6.876666667	3.466666667	2.146666667	-0.83793	0.10325	0.21755	-0.8043	0.12197	0.26317		-1.6553	0.0018197	0.014231	DOWN
transcript_HQ_CoA_transcript8032/f3p0/3377	18.03333333	8.366666667	8.836666667	-0.95256	2.0965E-06	2.3006E-05	-0.076803	0.65776	0.79864		-1.041	1.9651E-08	1.0365E-06	DOWN
transcript_HQ_CoA_transcript8042/f2p0/3347	0.453333333	0.433333333	2.31	0.13566	0.91494	0.95323	2.206	0.03597	0.10639		2.3317	1.6429E-05	0.00032012	UP
transcript_HQ_CoA_transcript8106/f2p0/3349	10.03666667	7.57	1.796666667	-0.27117	0.63745	0.77689	-2.1887	0.00067674	0.0046702	DOWN	-2.4725	3.4723E-10	2.7121E-08	DOWN
transcript_HQ_CoA_transcript8118/f2p0/3347	2.406666667	6.203333333	11.73	1.4744	0.065846	0.15535	0.77746	0.0609	0.15889		2.246	0.0018501	0.014371	UP
transcript_HQ_CoA_transcript8135/f3p0/3368	6.85	4.476666667	1.37	-0.45959	0.20362	0.35864	-1.8251	0.0015849	0.0092189	DOWN	-2.2987	5.9686E-05	0.00092842	DOWN
transcript_HQ_CoA_transcript8137/f2p0/3351	8.7	3.866666667	4.263333333	-0.99436	0.004075	0.016787	-0.029298	0.9391	0.96799		-1.035	7.2242E-05	0.0010896	DOWN
transcript_HQ_CoA_transcript8180/f3p0/3295	19.99666667	23.97333333	53.93	0.42195	0.23203	0.39388	1.0111	0.00054608	0.0039129	UP	1.4215	1.7958E-07	7.2923E-06	UP
transcript_HQ_CoA_transcript819/f2p0/5883	2.523333333	2.17	0.433333333	0.014215	0.99041	0.99542	-2.5148	NA	NA	NA	-2.5212	0.0058143	0.034294	DOWN
transcript_HQ_CoA_transcript8191/f2p0/3334	6.323333333	5.816666667	1.386666667	0.028514	0.92234	0.95752	-2.2229	5.7019E-05	0.00060236	DOWN	-2.2053	3.4803E-05	0.00060017	DOWN
transcript_HQ_CoA_transcript8207/f3p0/3341	4.47	6.953333333	0.193333333	0.77442	0.5331	0.69593	-5.2636	5.7412E-08	1.5185E-06	DOWN	-4.5078	0.00014784	0.0019562	DOWN
transcript_HQ_CoA_transcript8215/f2p0/3344	4.766666667	2.86	1.063333333	-0.59738	0.42887	0.60285	-1.5247	0.079933	0.19385		-2.1402	0.0023092	0.017029	DOWN
transcript_HQ_CoA_transcript8228/f2p0/3404	3.043333333	3.84	1.373333333	0.48173	0.13091	0.26006	-1.6241	1.6533E-07	3.8951E-06	DOWN	-1.1533	0.0009118	0.0083017	DOWN
transcript_HQ_CoA_transcript8248/f2p0/3401	29.72333333	31.56333333	12.80333333	0.23394	0.70722	0.82415	-1.4339	0.0067028	0.029412	DOWN	-1.2145	0.0043965	0.027798	DOWN
transcript_HQ_CoA_transcript8277/f2p0/3324	4.79	5.57	10.96	0.36622	0.31497	0.48737	0.82713	0.035101	0.1046		1.1822	0.0052565	0.031696	UP
transcript_HQ_CoA_transcript8279/f2p0/3360	14.50666667	23.87333333	6.556666667	0.86261	0.10054	0.2132	-1.986	3.3575E-05	0.00038607	DOWN	-1.1376	0.0011754	0.010159	DOWN
transcript_HQ_CoA_transcript830/f2p0/5893	20.91	7.743333333	8.476666667	-1.2448	0.02547	0.073655	-0.031316	0.94385	0.97001		-1.2919	0.0059937	0.035059	DOWN
transcript_HQ_CoA_transcript834/f3p0/5975	23.66333333	20.79666667	6.726666667	-0.031616	0.90887	0.94976	-1.7778	8.2083E-17	1.194E-14	DOWN	-1.8204	1.3065E-12	1.8735E-10	DOWN
transcript_HQ_CoA_transcript8361/f2p0/3331	5.9	1.793333333	0.506666667	-1.5646	0.034237	0.093034	-1.9422	0.014015	0.052317		-3.5192	1.1624E-11	1.3009E-09	DOWN
transcript_HQ_CoA_transcript8379/f2p0/3340	442.1466667	776.96	940.0933333	0.98809	0.18619	0.33708	0.11278	0.8712	0.93012		1.0887	0.0087077	0.045562	UP
transcript_HQ_CoA_transcript8402/f2p0/3313	48.93333333	30.89	5.473333333	-0.5162	0.12896	0.25716	-2.6236	5.2358E-07	1.1005E-05	DOWN	-3.154	4.2451E-12	5.4489E-10	DOWN
transcript_HQ_CoA_transcript8421/f5p0/3293	8.783333333	13.99666667	19.05333333	0.81802	0.020555	0.062042	0.3118	0.35124	0.54338		1.1182	0.00072322	0.0069283	UP
transcript_HQ_CoA_transcript8429/f2p0/3321	16.64666667	11.55	7.906666667	-0.38896	0.21849	0.37712	-0.69189	0.0080345	0.033846		-1.0907	0.00029153	0.0033739	DOWN
transcript_HQ_CoA_transcript8436/f10p0/3240	16.31	8.21	41.45333333	-0.82197	0.07697	0.17507	2.1762	7.2156E-06	0.00010257	UP	1.3403	0.0034977	0.023346	UP
transcript_HQ_CoA_transcript8437/f2p0/3318	31.99	20.95333333	14.59666667	-0.47036	0.16422	0.30782	-0.65808	0.034322	0.10303		-1.1401	7.3764E-07	2.4264E-05	DOWN
transcript_HQ_CoA_transcript8473/f2p0/3304	7.003333333	9.776666667	17.82666667	0.66734	0.098784	0.21056	0.72386	0.21211	0.38826		1.3761	0.006544	0.037165	UP
transcript_HQ_CoA_transcript8500/f2p0/3311	0.51	0.643333333	1.633333333	0.48831	0.41718	0.59161	1.191	0.0066095	0.029071	UP	1.6703	0.00040627	0.0043558	UP
transcript_HQ_CoA_transcript8530/f2p0/3083	3.516666667	5.366666667	11.43333333	0.77148	0.040044	0.1052	0.93899	0.0021528	0.011761		1.6966	1.2251E-10	1.0811E-08	UP
transcript_HQ_CoA_transcript8533/f2p0/3298	4.763333333	4.283333333	0.176666667	-0.010251	0.99356	0.99683	-4.6921	3.4673E-05	0.00039646	DOWN	-4.7165	8.9123E-06	0.00019223	DOWN
transcript_HQ_CoA_transcript8550/f2p0/3292	12.34666667	8.393333333	5.026666667	-0.40373	0.44468	0.61794	-0.88115	0.041699	0.11881		-1.2988	0.0016085	0.012892	DOWN
transcript_HQ_CoA_transcript8558/f6p0/3271	5.663333333	2.98	16.96333333	-0.83673	0.52538	0.68952	2.3886	0.17959	0.34581		1.5458	0.0097478	0.049384	UP
transcript_HQ_CoA_transcript8564/f2p0/3287	2.663333333	1.196666667	6.396666667	-1.004	0.021683	0.064773	2.276	6.5282E-08	1.7045E-06	UP	1.2591	0.0022159	0.01654	UP
transcript_HQ_CoA_transcript8593/f2p0/3309	2.496666667	1.72	1.19	-0.40905	0.34637	0.52175	-0.67038	0.10387	0.23499		-1.0911	0.0068335	0.038276	DOWN
transcript_HQ_CoA_transcript8605/f2p0/3290	6.17	2.123333333	15.36333333	-1.3915	0.025042	0.072693	2.7167	1.0014E-06	0.00001908	UP	1.3115	0.0041967	0.026784	UP
transcript_HQ_CoA_transcript8616/f2p0/3278	1.473333333	1.653333333	3.593333333	0.30252	0.47806	0.6486	0.98266	0.017471	0.061891		1.2735	0.0065538	0.037185	UP
transcript_HQ_CoA_transcript8638/f3p0/3285	13.89	5.386666667	0.063333333	-1.1726	0.1611	0.30369	-6.6152	1.0048E-07	2.482E-06	DOWN	-7.7633	3.9338E-29	8.0228E-26	DOWN

transcript_HQ_CoA_transcript8641/f2p0/3286	0.063333333	0.123333333	1.086666667	1.2294	0.67942	0.80599		2.9247	0.018138	0.063653		4.1329	0.00074754	0.0071052	UP
transcript_HQ_CoA_transcript8642/f4p0/3260	16.38666667	11.85333333	6.473333333	-0.31888	0.34422	0.51931		-1.0065	0.0026059	0.013777	DOWN	-1.3392	1.291E-07	5.3977E-06	DOWN
transcript_HQ_CoA_transcript8687/f2p0/3295	3.016666667	2.123333333	11.75333333	-0.31498	0.5469	0.7076		2.2897	1.189E-08	3.6783E-07	UP	1.9604	2.3751E-11	2.4491E-09	UP
transcript_HQ_CoA_transcript8705/f3p0/3270	0.753333333	0.313333333	3.403333333	-1.1283	0.092077	0.1999		3.3014	5.349E-13	4.3382E-11	UP	2.1605	2.4611E-07	9.5502E-06	UP
transcript_HQ_CoA_transcript871/f4p0/5805	0.986666667	1.103333333	6.156666667	0.40085	0.7794	0.8698		2.2607	0.22184	0.39973		2.6479	4.5433E-11	4.3661E-09	UP
transcript_HQ_CoA_transcript8750/f2p0/3278	3.2	3.84	1.116666667	0.42128	0.33188	0.50591		-1.916	3.2067E-07	7.0559E-06	DOWN	-1.5096	0.0006904	0.0066767	DOWN
transcript_HQ_CoA_transcript8770/f2p0/3274	7.44	5.09	3.656666667	-0.38479	0.31653	0.48913		-0.62214	0.029269	0.091593		-1.0216	0.0041104	0.026417	DOWN
transcript_HQ_CoA_transcript8805/f5p0/3223	0.806666667	1.903333333	2.033333333	1.3756	0.046025	0.11734		-0.036804	0.95127	0.9745		1.3272	0.0015338	0.012418	UP
transcript_HQ_CoA_transcript8808/f10p0/3246	1.15	2.073333333	3.916666667	0.99112	0.035536	0.095705		0.78672	0.017008	0.060745		1.7655	5.0331E-06	0.0001206	UP
transcript_HQ_CoA_transcript8810/f4p0/3219	190.7533333	78.16666667	59.87333333	-1.1715	0.083402	0.18564		-0.51692	0.31547	0.50642		-1.6944	0.0021297	0.016034	DOWN
transcript_HQ_CoA_transcript8853/f2p0/3250	13.89666667	7.323333333	6.206666667	-0.76892	0.0094708	0.033718		-0.38431	0.03475	0.10386		-1.1658	3.8844E-05	0.00065029	DOWN
transcript_HQ_CoA_transcript8910/f2p0/3321	12.56333333	8.486666667	2.933333333	-0.43918	0.4028	0.57716		-1.655	0.0017887	0.010188	DOWN	-2.1052	9.6769E-06	0.00020487	DOWN
transcript_HQ_CoA_transcript8942/f2p0/3290	6.74	4.53	20.13666667	-0.42111	0.41475	0.58948		2.0221	3.5986E-06	0.00005691	UP	1.5872	5.691E-07	1.9416E-05	UP
transcript_HQ_CoA_transcript8949/f2p0/3245	4.926666667	2.883333333	10.28	-0.62239	0.095753	0.20544		1.6902	1.0979E-06	2.0577E-05	UP	1.0552	0.00052043	0.0053276	UP
transcript_HQ_CoA_transcript8980/f4p0/3225	73.28333333	76.40666667	27.44333333	0.21688	0.67862	0.80552		-1.624	0.0018145	0.010298	DOWN	-1.4168	0.0085805	0.045127	DOWN
transcript_HQ_CoA_transcript900/f6p0/5813	1.163333333	0.453333333	0.316666667	-1.2062	0.016593	0.052402		-0.68854	0.16879	0.33084		-1.9054	6.9184E-05	0.0010512	DOWN
transcript_HQ_CoA_transcript9060/f3p0/3230	4.736666667	7.233333333	9.876666667	0.7902	0.19356	0.34611		0.27441	0.61106	0.76575		1.0544	0.0046482	0.029	UP
transcript_HQ_CoA_transcript907/f3p0/5843	5.686666667	1.65	1.143333333	-1.6283	0.025511	0.073748		-0.6436	0.29008	0.47969		-2.2873	0.00056095	0.0056479	DOWN
transcript_HQ_CoA_transcript9080/f2p0/3239	9.813333333	6.69	20.05	-0.34086	0.67382	0.80273		1.3796	0.089414	0.21049		1.0263	0.00048241	0.0050311	UP
transcript_HQ_CoA_transcript911/f6p0/5815	3.973333333	1.536666667	12.07333333	-1.1936	0.10153	0.21495		2.8155	3.837E-13	3.1823E-11	UP	1.6083	0.0064369	0.036784	UP
transcript_HQ_CoA_transcript9125/f2p0/3246	5.19	6.2	14.55	0.42066	0.21495	0.37315		1.0676	0.0010809	0.0068093	UP	1.4736	2.6161E-05	0.00047125	UP
transcript_HQ_CoA_transcript9140/f2p0/3221	22.13	19.12	9.796666667	-0.053829	0.73672	0.84232		-1.1067	6.5948E-08	1.7097E-06	DOWN	-1.1721	3.9706E-08	1.9332E-06	DOWN
transcript_HQ_CoA_transcript9144/f2p0/3267	10.11333333	6.513333333	4.14	-0.49658	0.15192	0.29046		-0.79964	0.0058064	0.026389		-1.3073	4.4852E-05	0.00073179	DOWN
transcript_HQ_CoA_transcript9164/f2p0/3207	3.923333333	3.236666667	8.44	-0.084584	0.86371	0.92367		1.2038	0.0054059	0.024902	UP	1.1041	9.4018E-05	0.0013514	UP
transcript_HQ_CoA_transcript9171/f2p0/3207	4.776666667	5.806666667	1.546666667	0.41255	0.35683	0.5321		-2.0416	3.9241E-06	6.1288E-05	DOWN	-1.6401	2.6545E-07	1.0151E-05	DOWN
transcript_HQ_CoA_transcript9191/f2p0/3229	12.96	15.35	4.556666667	0.40234	0.19272	0.34494		-1.8834	3.8473E-05	0.00043288	DOWN	-1.4952	0.00021763	0.0026666	DOWN
transcript_HQ_CoA_transcript9196/f5p0/3217	123.4133333	63.62333333	61.05333333	-0.81385	0.0106	0.036956		-0.19515	0.53365	0.70481		-1.0211	7.3124E-12	8.7155E-10	DOWN
transcript_HQ_CoA_transcript9252/f2p0/3188	16.30333333	6.436666667	0.603333333	-1.2259	0.037787	0.10039		-3.5598	5.1286E-09	1.7123E-07	DOWN	-4.7943	3.3621E-25	3.4284E-22	DOWN
transcript_HQ_CoA_transcript9277/f2p0/3203	3.253333333	4.886666667	17.02333333	0.7442	0.17523	0.3229		1.6846	0.00081989	0.0054468	UP	2.4163	6.5273E-06	0.00014902	UP
transcript_HQ_CoA_transcript9309/f2p0/3238	3.06	5.893333333	11.72666667	1.0792	0.016277	0.051601		0.85012	0.0010745	0.0067771		1.921	1.5507E-07	6.3536E-06	UP
transcript_HQ_CoA_transcript9371/f2p0/3138	10.77666667	17.97666667	24.90333333	0.89392	0.086457	0.19071		0.33058	0.4736	0.65718		1.21	0.0090142	0.046738	UP
transcript_HQ_CoA_transcript9398/f2p0/3202	1.913333333	2.156666667	0.03	0.32119	0.82461	0.89855		-6.341	2.4395E-06	0.00004106	DOWN	-6.0391	0.00044389	0.0047041	DOWN
transcript_HQ_CoA_transcript9402/f5p0/3216	10.53333333	5.61	2.4	-0.74005	0.020514	0.06195		-1.3998	0.00043164	0.0032279	DOWN	-2.1507	2.087E-11	2.189E-09	DOWN
transcript_HQ_CoA_transcript9409/f2p0/3188	0	0.736666667	1.763333333	7.1973	NA	NA	NA	1.072	0.56548	0.7314		8.2551	1.4185E-08	7.7491E-07	UP
transcript_HQ_CoA_transcript9432/f2p0/3177	0.446666667	34.77666667	10.36333333	6.5005	NA	NA	NA	-1.9585	NA	NA	NA	4.5232	2.2209E-09	1.4559E-07	UP
transcript_HQ_CoA_transcript9451/f2p0/3203	17.58333333	9.75	8.413333333	-0.69572	0.00042191	0.0024433		-0.36839	0.092729	0.2161		-1.0757	2.8176E-06	7.5498E-05	DOWN
transcript_HQ_CoA_transcript9496/f2p0/3203	1.936666667	1.403333333	5.336666667	-0.34371	0.57795	0.73264		1.7891	0.00010336	0.00099289	UP	1.4377	0.0061204	0.035515	UP
transcript_HQ_CoA_transcript9503/f3p0/3103	60.44333333	76.81	12.53333333	0.50383	0.27064	0.43879		-2.7449	1.1007E-10	5.3513E-09	DOWN	-2.2579	1.0371E-06	3.2597E-05	DOWN
transcript_HQ_CoA_transcript9508/f2p0/3178	7.073333333	4.823333333	16.55333333	-0.38313	0.19776	0.35139		1.6176	1.432E-10	6.7646E-09	UP	1.2211	4.1463E-06	0.00010284	UP
transcript_HQ_CoA_transcript9515/f2p0/3165	2.746666667	3.406666667	1.06	0.46741	0.26938	0.43734		-1.8271	3.108E-09	1.081E-07	DOWN	-1.3722	0.0036382	0.024039	DOWN
transcript_HQ_CoA_transcript9543/f2p0/3161	1.25	0.32	0.14	-1.804	0.031918	0.087934		-1.403	0.15663	0.31431		-3.218	8.0168E-05	0.0011848	DOWN
transcript_HQ_CoA_transcript9548/f2p0/3175	8.153333333	12.05666667	24.25666667	0.71353	0.079093	0.17863		0.86161	0.018696	0.065147		1.5617	2.2044E-06	6.1491E-05	UP
transcript_HQ_CoA_transcript9559/f3p0/3169	16.83666667	9.84	7.886666667	-0.64061	0.12354	0.24903		-0.46088	0.34686	0.5391		-1.1093	0.0055847	0.033099	DOWN
transcript_HQ_CoA_transcript9567/f2p0/3169	4.78	3.48	2.046666667	-0.29179	0.53935	0.70166		-0.91165	0.0033312	0.016899		-1.2189	0.0079577	0.042646	DOWN
transcript_HQ_CoA_transcript9598/f6p0/3139	16.44666667	7.486666667	5.313333333	-0.99934	0.0019316	0.0090084		-0.63404	0.040817	0.11691		-1.6432	5.4057E-09	3.2964E-07	DOWN
transcript_HQ_CoA_transcript9633/f2p0/3183	20.85	9.386666667	9.2	-0.99958	9.0739E-08	1.3042E-06		-0.17718	0.27265	0.46003		-1.1891	1.4337E-10	1.2472E-08	DOWN
transcript_HQ_CoA_transcript9659/f2p0/3156	22.07333333	14.12666667	6.253333333	-0.49474	0.051897	0.12903		-1.3192	2.079E-07	4.7812E-06	DOWN	-1.826	1.8432E-25	2.1145E-22	DOWN
transcript_HQ_CoA_transcript9694/f2p0/3148	3.786666667	3.96	1.17	0.2268	0.47144	0.64291		-1.9284	7.8186E-05	0.00078182	DOWN	-1.7175	0.00071001	0.0068267	DOWN
transcript_HQ_CoA_transcript9703/f3p0/3175	1.14	7.73	11.79666667	2.9783	NA	NA	NA	0.40279	0.81841	0.90075		3.3637	4.6398E-06	0.00011231	UP
transcript_HQ_CoA_transcript9720/f2p0/3145	5.76	3.79	1.683333333	-0.45387	0.38911	0.56427		-1.3025	0.01327	0.05015		-1.7706	2.6003E-06	7.0813E-05	DOWN
transcript_HQ_CoA_transcript9723/f2p0/3147	4.243333333	3.463333333	1.556666667	-0.11162	0.863	0.92336		-1.342	0.041354	0.11811		-1.4646	1.1446E-05	0.00023606	DOWN
transcript_HQ_CoA_transcript9724/f3p0/3170	15.81	5.32	4.163333333	-1.429	0.094901	0.20416		-0.47033	0.60351	0.76057		-1.9162	6.4305E-05	0.0009927	DOWN
transcript_HQ_CoA_transcript9728/f3p0/3152	4.24	5.49	13.59333333	0.49059	0.44759	0.62052		1.1804	0.0018886	0.010595	UP	1.6633	0.0015069	0.012277	UP
transcript_HQ_CoA_transcript9744/f2p0/3087	1.986666667	1.846666667	4.156666667	0.067437	0.8829	0.93491		0.9976	0.008239	0.034494		1.0534	0.00040048	0.0043113	UP
transcript_HQ_CoA_transcript9840/f2p0/3129	28.23333333	13.61	10.70333333	-0.90737	0.019099	0.058498		-0.49156	0.11802	0.25718		-1.4092	1.4401E-05	0.00028793	DOWN
transcript_HQ_CoA_transcript9871/f3p0/3133	27.21	15.30333333	9.88	-0.66318	0.016742	0.052765		-0.78362	0.0049943	0.023421		-1.4602	8.7097E-09	5.0213E-07	DOWN
transcript_HQ_CoA_transcript9996/f4p0/3095	14.24	23.51	35.86	0.88103	7.9165E-08	1.1541E-06		0.47012	0.093843	0.21796		1.3399	1.4748E-06	4.3873E-05	UP
transcript_LQ_CoA_transcript10526/f2p0/3069	2.393333333	2.383333333	0.52	0.14455	0.76599	0.86172		-2.3583	0.0020967	0.011537	DOWN	-2.2285	0.0039439	0.025607	DOWN
transcript_LQ_CoA_transcript13276/f2p0/2635	57.95	13.37333333	10.90333333	-1.9913	0.026878	0.076896		-0.4178	0.67211	0.80861		-2.4139	0.00023798	0.0028606	DOWN
transcript_LQ_CoA_transcript2268/f2p0/4829	8.693333333	5.9	4.32	-0.40104	0.037304	0.099314		-0.59981	0.0067587	0.029601		-1.0141	1.0719E-05	0.00022307	DOWN
transcript_LQ_CoA_transcript2910/f2p0/4556	32.56	15.71666667	13.27	-0.90851	0.10224	0.21594		-0.38019	0.50697	0.68311		-1.3006	0.00049139	0.0050983	DOWN
transcript_LQ_CoA_transcript29663/f2p0/1228	18.93666667	9.106666667	2.77	-0.87436	NA	NA		-1.8573	NA	NA	NA	-2.7497	0.00036464	0.0040271	DOWN
transcript_LQ_CoA_transcript7913/f2p0/3396	1.106666667	1.523333333	2.51	0.61146	0.1969	0.3503		0.55503	0.15662	0.31431		1.1546	0.0097219	0.049281	UP
transcript_HQ_CoA_transcript10005/f2p0/3111	24.49	37.39	14.76	0.76403	0.024778	0.072076		-1.4828	0.00014903	0.0013383	DOWN	-0.73148	0.013094	0.061109	
transcript_HQ_CoA_transcript10060/f4p0/3079	6.353333333	10.87333333	4.986666667	0.92363	0.012036	0.040903		-1.2668	0.00025266	0.002072	DOWN	-0.35614	0.21215	0.41638	
transcript_HQ_CoA_transcript10078/f2p0/3112	0.4	0.07	2.093333333	-2.4194	0.086821	0.19131		4.8225	0.0018028	0.010252	UP	2.3855	0.053864	0.16895	
transcript_HQ_CoA_transcript10198/f2p0/3111	1.3	0.486666667	2.016666667	-1.2696	0.04802	0.12131		1.9292	0.0011932	0.0073615	UP	0.64597	0.1796	0.37452	
transcript_HQ_CoA_transcript10233/f2p0/3091	8.033333333	12.58333333	4.633333333	0.81191	0.036876	0.098504		-1.59	7.2535E-11	3.6208E-09	DOWN	-0.79101	0.061329	0.18402	
transcript_HQ_CoA_transcript10272/f2p0/3082	11.54	5.526666667	12.85333333	-0.88544	0.048682	0.12272		1.0419	0.0090685	0.037185	UP	0.14386	0.59114	0.76697	
transcript_HQ_CoA_transcript10298/f4p0/3077	25.42333333	12.53666667	30.21	-0.86391	9.8549E-07	1.1601E-05		1.1182	6.8713E-09	2.2173E-07	UP	0.24227	0.18778	0.38489	
transcript_HQ_CoA_transcript10360/f2p0/3080	8.88	8.323333333	2.796666667	0.059013	0.94517	0.96975		-1.6933	0.005379	0.024815	DOWN	-1.651	0.022223	0.089359	
transcript_HQ_CoA_transcript10411/f2p0/3198	3.666666667	4.9	2.686666667	0.57558	0.10563	0.22144		-1.0204	0.0036802	0.01836	DOWN	-0.45805	0.20382	0.40603	
transcript_HQ_CoA_transcript10462/f3p0/3174	7.443333333	2.32	11.60666667	-1.5097	0.024416	0.071316		2.1699	3.2273E-19	6.7398E-17	UP	0.64819	0.26933	0.48179	
transcript_HQ_CoA_transcript10478/f2p0/3123	8.513333333	13.23	5.896666667	0.79299	0.033793	0.092016		-1.3284	0.00011894	0.001106	DOWN	-0.54659	0.06216	0.18558	
transcript_HQ_CoA_transcript10512/f2p0/3074	17.00666667	8.946666667	21.45333333	-0.77627	0.0094999	0.033779		1.1053	1.3891E-05	0.00017893	UP	0.31831	0.32402	0.54109	
transcript_HQ_CoA_transcript10578/f2p0/3050	12.98	14.15666667	38.77333333	0.30443	0.65109	0.78687		1.3082	1.885E-07	4.3734E-06	UP	1.5969	0.010366	0.051659	
transcript_HQ_CoA_transcript10589/f3p0/2978	13.50333333	8.063333333	19.68333333	-0.59018	0.0055624	0.021638		1.1474	2.7233E-08	7.7267E-07	UP	0.54499	0.0059343	0.034804	
transcript_HQ_CoA_transcript10603/f2p0/3039	5.79	8.973333333	3.366666667	0.79607	0.07081	0.16466		-1.5525	0.00026599	0.0021601	DOWN	-0.77243	0.14014	0.3191	
transcript_HQ_CoA_transcript10604/f3p0/3047	10.15333333	8.073333333	18.75333333	-0.14575	0.93878	0.96559		1.0518	0.00021751	0.0018375	UP	0.89065	0.63613	0.7978	

transcript_HQ_CoA_transcript10643/f2p0/3040	11.38666667	5.976666667	14.14333333	-0.78375	0.001819	0.0085663	1.0897	0.00049025	0.0035987	UP	0.29549	0.3211	0.53824	
transcript_HQ_CoA_transcript10687/f4p0/3052	5.07	1.753333333	9.086666667	-1.391	0.029595	0.082666	2.2456	2.4066E-05	0.00028662	UP	0.84134	0.076298	0.21237	
transcript_HQ_CoA_transcript10708/f2p0/3052	17.75666667	5.303333333	29.76333333	-1.6102	0.023258	0.068649	2.3299	2.2287E-15	2.6699E-13	UP	0.7113	0.2837	0.49773	
transcript_HQ_CoA_transcript10734/f3p0/3073	22.65	13.39333333	30.06	-0.61338	0.10315	0.21745	1.0228	0.0066082	0.029071	UP	0.39854	0.30984	0.52653	
transcript_HQ_CoA_transcript10928/f3p0/3006	7.173333333	4.096666667	9.056666667	-0.6788	0.16381	0.30745	1.0102	0.0083349	0.034823	UP	0.3236	0.44177	0.65272	
transcript_HQ_CoA_transcript10980/f2p0/3008	2.106666667	0.676666667	2.606666667	-1.511	0.041109	0.10747	1.8306	0.0027203	0.014246	UP	0.3061	0.51367	0.71111	
transcript_HQ_CoA_transcript11014/f2p0/3023	4.07	1.746666667	6.216666667	-1.1024	0.25479	0.42088	1.6822	0.0023741	0.012768	UP	0.5724	0.48769	0.69059	
transcript_HQ_CoA_transcript11043/f2p0/3051	6.313333333	10.07666667	4.123333333	0.81571	0.15959	0.30153	-1.4084	0.011583	0.045064	DOWN	-0.60775	0.1206	0.28977	
transcript_HQ_CoA_transcript11056/f2p0/3007	34.45333333	50.84333333	26.60333333	0.71495	0.02839	0.080172	-1.0759	0.00091094	0.005925	DOWN	-0.37263	0.22398	0.43085	
transcript_HQ_CoA_transcript11115/f2p0/2984	12.74666667	21.32333333	10.68	0.90931	0.00029371	0.0017887	-1.1451	1.7166E-12	1.2536E-10	DOWN	-0.24846	0.33657	0.55434	
transcript_HQ_CoA_transcript11226/f2p0/2985	14.34333333	25.31333333	13.62666667	0.98538	0.0069118	0.02592	-1.0386	0.011839	0.045847	DOWN	-0.06731	0.83086	0.92166	
transcript_HQ_CoA_transcript11305/f2p0/2997	1.476666667	1.886666667	0.826666667	0.53785	0.37986	0.55522	-1.3727	0.0047447	0.022472	DOWN	-0.85354	0.19101	0.38904	
transcript_HQ_CoA_transcript11326/f3p0/2977	0.973333333	3.77	0.43	2.0965	0.11646	0.23826	-3.2575	0.0036151	0.018079	DOWN	-1.1776	0.44358	0.65429	
transcript_HQ_CoA_transcript11354/f2p0/2977	0.433333333	0.723333333	0.063333333	0.88729	0.54073	0.70261	-3.6046	0.0080055	0.03377	DOWN	-2.7176	0.14796	0.3304	
transcript_HQ_CoA_transcript11358/f2p0/2969	5.62	7.663333333	4.213333333	0.58434	0.13936	0.27281	-1.0063	0.002561	0.013602	DOWN	-0.43322	0.17474	0.36753	
transcript_HQ_CoA_transcript1142/f3p0/5575	1.55	5.086666667	0.62	1.8487	0.037783	0.10039	-3.1401	0.00017474	0.0015354	DOWN	-1.3075	0.040622	0.13877	
transcript_HQ_CoA_transcript11434/f2p0/2941	4.453333333	5.836666667	2.816666667	0.55288	0.25515	0.42129	-1.1993	0.001378	0.0082572	DOWN	-0.66234	0.18063	0.37579	
transcript_HQ_CoA_transcript11461/f2p0/3007	7.293333333	3.94	8.92	-0.72464	0.0051955	0.020511	1.0396	0.0017429	0.0099738	UP	0.30144	0.35598	0.57315	
transcript_HQ_CoA_transcript11515/f4p0/2915	3.55	2.016666667	5.246666667	-0.66269	0.18078	0.33053	1.2349	0.002089	0.011502	UP	0.56011	0.11936	0.28754	
transcript_HQ_CoA_transcript11522/f2p0/2944	5.226666667	8.966666667	3.993333333	0.96208	0.24554	0.4101	-1.3137	1.6839E-08	4.9941E-07	DOWN	-0.36544	0.65723	0.81256	
transcript_HQ_CoA_transcript11535/f5p0/2944	8.17	4.34	14.92666667	-0.74791	0.0072716	0.027026	1.6541	0.003093	0.015854	UP	0.89216	0.094193	0.24653	
transcript_HQ_CoA_transcript1155/f2p0/5594	0.683333333	0.97	0.503333333	0.66541	0.1032	0.21747	-1.1046	0.008156	0.034247	DOWN	-0.44973	0.38435	0.59974	
transcript_HQ_CoA_transcript11556/f4p0/2931	11.18666667	7.4	18.59666667	-0.42885	0.22717	0.3879	1.1637	0.0026541	0.013954	UP	0.72274	0.011757	0.056508	
transcript_HQ_CoA_transcript11588/f2p0/2944	6.18	8.986666667	4.243333333	0.70752	0.046629	0.11859	-1.2411	0.0002899	0.0023173	DOWN	-0.54693	0.033477	0.1207	
transcript_HQ_CoA_transcript11602/f2p0/2961	2.13	5.906666667	1.113333333	1.6208	0.036934	0.098572	-2.5309	0.00041739	0.0031431	DOWN	-0.92391	0.12122	0.29067	
transcript_HQ_CoA_transcript11605/f2p0/2941	2.203333333	2.123333333	0.783333333	0.13851	0.83482	0.90521	-1.5942	0.0060227	0.027176	DOWN	-1.4693	0.033434	0.12059	
transcript_HQ_CoA_transcript11681/f2p0/2903	10.30333333	11.85	5.84	0.33962	0.44917	0.62173	-1.1556	0.0014546	0.0086284	DOWN	-0.82899	0.027414	0.10425	
transcript_HQ_CoA_transcript11715/f2p0/2928	13.5	7.84	19.52666667	-0.62743	0.0099183	0.035005	1.1558	0.0016244	0.009407	UP	0.5157	0.16107	0.34899	
transcript_HQ_CoA_transcript11716/f2p0/2923	38.64	22.10666667	55.97666667	-0.64372	0.0027626	0.012135	1.1899	4.0647E-11	2.1226E-09	UP	0.53353	0.0061344	0.035565	
transcript_HQ_CoA_transcript11737/f2p0/2915	19.36	8.703333333	27.04	-0.98977	0.00001323	0.00011974	1.4965	6.4725E-10	2.6305E-08	UP	0.49404	0.067174	0.19531	
transcript_HQ_CoA_transcript11751/f2p0/2908	4.22	6.44	3.16	0.7855	0.077934	0.17677	-1.1894	0.0033038	0.016783	DOWN	-0.41843	0.21623	0.42136	
transcript_HQ_CoA_transcript11767/f4p0/2889	9.35	5.84	14.07666667	-0.5344	0.071446	0.16576	1.1278	0.00096502	0.0062031	UP	0.58247	0.038382	0.13323	
transcript_HQ_CoA_transcript11783/f5p0/2868	10.39333333	4.056666667	12.54666667	-1.2229	0.016516	0.052212	1.492	0.005388	0.024838	UP	0.25994	0.40918	0.62317	
transcript_HQ_CoA_transcript11799/f2p0/2925	4.99	2.773333333	6.32	-0.69024	0.064446	0.15288	1.0247	0.00040086	0.0030386	UP	0.32293	0.29641	0.51186	
transcript_HQ_CoA_transcript11839/f13p0/2841	341.7266667	315.11	165.4766667	0.015393	0.97637	0.98824	-1.062	0.0014028	0.0083751	DOWN	-1.0544	0.049206	0.1587	
transcript_HQ_CoA_transcript11880/f2p0/2905	5.873333333	9.763333333	1.426666667	0.8943	0.29155	0.46176	-2.9134	3.3095E-12	2.1899E-10	DOWN	-2.0376	0.018117	0.077419	
transcript_HQ_CoA_transcript11919/f2p0/2906	3.876666667	3.026666667	7.843333333	-0.22705	0.59635	0.74726	1.2332	0.0066107	0.029071	UP	0.99638	0.0073979	0.040443	
transcript_HQ_CoA_transcript11959/f2p0/2889	0.476666667	0.14	1.49	-1.7752	0.50191	0.669	3.3538	0.0010811	0.0068093	UP	1.5713	0.35233	0.56993	
transcript_HQ_CoA_transcript11987/f3p0/2891	8.3	4.403333333	12.6	-0.77034	0.011757	0.040152	1.3621	0.0018614	0.010485	UP	0.58053	0.16484	0.35384	
transcript_HQ_CoA_transcript11990/f2p0/2914	3.21	8.196666667	2.696666667	1.5043	0.021422	0.064148	-1.7393	0.0044776	0.021524	DOWN	-0.24905	0.59791	0.77101	
transcript_HQ_CoA_transcript12121/f2p0/2883	30.19666667	50.49	24.13	0.88529	0.019553	0.059609	-1.1974	0.0024496	0.013083	DOWN	-0.3235	0.29153	0.50647	
transcript_HQ_CoA_transcript12176/f2p0/2878	1.523333333	1.653333333	4.83	0.25148	0.71807	0.83092	1.3869	0.0024079	0.012905	UP	1.6276	0.012992	0.060747	
transcript_HQ_CoA_transcript12187/f2p0/2883	3.316666667	1.05	3.56	-1.5459	0.031323	0.086606	1.6623	0.0077979	0.03307	UP	0.10744	0.87591	0.9445	
transcript_HQ_CoA_transcript12197/f3p0/2873	3.936666667	1.32	5.453333333	-1.4324	0.029375	0.082241	1.9161	0.0014641	0.0086763	UP	0.47224	0.36677	0.58383	
transcript_HQ_CoA_transcript12199/f2p0/2914	6.073333333	2.72	11.57333333	-1.0311	0.083078	0.18513	1.9774	0.00011321	0.0010658	UP	0.93477	0.0063364	0.036414	
transcript_HQ_CoA_transcript12200/f3p0/2868	10.95666667	12.61666667	5.046666667	0.34618	0.31892	0.49178	-1.4336	0.0044015	0.021209	DOWN	-1.1007	0.011251	0.055012	
transcript_HQ_CoA_transcript12247/f2p0/2868	4.836666667	3.043333333	7.023333333	-0.50838	0.13809	0.27111	1.0533	0.0015091	0.0088625	UP	0.53361	0.11661	0.28403	
transcript_HQ_CoA_transcript12255/f2p0/2863	12.66666667	16.59666667	8.78	0.55889	0.090494	0.19724	-1.0754	1.3561E-08	4.1288E-07	DOWN	-0.53112	0.10426	0.26433	
transcript_HQ_CoA_transcript12294/f2p0/2861	6.63	13.24	5.193333333	1.183	0.018153	0.056145	-1.5154	0.00019311	0.0016735	DOWN	-0.34594	0.3901	0.60583	
transcript_HQ_CoA_transcript12322/f2p0/2856	6.53	11.97	0.956666667	0.99967	NA	NA	-3.8127	0.00027437	0.0022171	DOWN	-2.8229	NA	NA	NA
transcript_HQ_CoA_transcript1233/f2p0/5523	2.046666667	4.043333333	0.593333333	1.1266	0.30617	0.47795	-2.8906	2.1007E-05	0.00025533	DOWN	-1.7772	0.083503	0.22634	
transcript_HQ_CoA_transcript12388/f2p0/2854	10.56666667	7.06	18.97	-0.45409	0.36343	0.53878	1.2902	8.2423E-05	0.00082105	UP	0.82642	0.039644	0.13637	
transcript_HQ_CoA_transcript12420/f2p0/2840	0.073333333	0.223333333	21.1	1.805	0.38279	0.55831	6.4233	0.0058161	0.026426	UP	8.2184	0.029294	0.10993	
transcript_HQ_CoA_transcript12492/f2p0/2864	8.9	11.82333333	5.36	0.54068	0.21834	0.377	-1.2646	0.0037468	0.01863	DOWN	-0.73456	0.002499	0.018066	
transcript_HQ_CoA_transcript12501/f2p0/2860	6.186666667	8.99	4.766666667	0.68909	0.040658	0.10643	-1.0618	6.6068E-07	1.3351E-05	DOWN	-0.38586	0.25345	0.4639	
transcript_HQ_CoA_transcript12622/f2p0/2831	10.70666667	21.23666667	3.29	1.1665	0.096457	0.20661	-2.8402	6.8982E-18	1.2396E-15	DOWN	-1.6922	0.021562	0.087714	
transcript_HQ_CoA_transcript1269/f4p0/5468	14.09666667	7.856666667	19.00666667	-0.705	0.050611	0.12651	1.1251	8.9956E-13	7.0462E-11	UP	0.40991	0.21633	0.42148	
transcript_HQ_CoA_transcript12692/f2p0/2818	24.27	11.74666667	29.35666667	-0.90238	0.0034085	0.014488	1.1736	0.0039703	0.019549	UP	0.25933	0.4862	0.68939	
transcript_HQ_CoA_transcript12880/f2p0/2806	9.383333333	10.31	5.04	0.27991	0.46586	0.63749	-1.1685	8.5595E-05	0.00084786	DOWN	-0.90075	0.013687	0.062934	
transcript_HQ_CoA_transcript13020/f2p0/2852	15.69666667	11.81333333	30.37333333	-0.25459	0.36522	0.54069	1.2134	1.1604E-05	0.00015412	UP	0.94678	0.00022155	0.0027092	
transcript_HQ_CoA_transcript13175/f2p0/2818	10.21333333	7.326666667	21.65666667	-0.32009	0.61052	0.75748	1.3862	0.0008995	0.0058735	UP	1.0579	0.036583	0.12856	
transcript_HQ_CoA_transcript13184/f6p0/2768	6.93	4.353333333	12.13333333	-0.50694	0.27267	0.44118	1.3095	0.0015202	0.0089095	UP	0.79299	0.013442	0.062195	
transcript_HQ_CoA_transcript13234/f5p0/2777	10.13	7.583333333	17.70333333	-0.26763	0.33284	0.5072	1.0741	5.2161E-09	1.7351E-07	UP	0.79584	0.0018241	0.014241	
transcript_HQ_CoA_transcript13253/f2p0/2781	0.353333333	0.363333333	1.643333333	0.23275	0.84972	0.91526	2.0008	0.003546	0.017821	UP	2.2267	0.019571	0.081905	
transcript_HQ_CoA_transcript13317/f3p0/2810	5.486666667	9.723333333	4.603333333	0.98045	1.9964E-05	0.00017143	-1.224	4.57E-09	1.5483E-07	DOWN	-0.2562	0.28051	0.49425	
transcript_HQ_CoA_transcript13337/f2p0/2764	14.09666667	8.573333333	21.40666667	-0.54317	0.15968	0.30165	1.147	0.001393	0.0083246	UP	0.59191	0.039497	0.13603	
transcript_HQ_CoA_transcript13338/f2p0/2755	14.45666667	22.98	10.94333333	0.83411	0.0068138	0.025605	-1.2242	0.00055974	0.0039874	DOWN	-0.40387	0.14218	0.32202	
transcript_HQ_CoA_transcript13374/f3p0/2799	14.39	19.85333333	10.20333333	0.63188	0.014502	0.047337	-1.1185	0.00018392	0.0016053	DOWN	-0.49828	0.12032	0.28918	
transcript_HQ_CoA_transcript13390/f2p0/2765	6.566666667	8.176666667	3.3	0.49403	0.23781	0.40085	-1.471	0.00028973	0.0023169	DOWN	-0.9931	0.0098151	0.049589	
transcript_HQ_CoA_transcript134/f2p0/7609	2.386666667	1.08	2.453333333	-0.97945	0.038749	0.10244	1.0238	0.0012244	0.0074894	UP	0.030295	0.94588	0.97572	
transcript_HQ_CoA_transcript13435/f5p0/2747	38.63333333	54.20333333	29.28666667	0.65175	0.035302	0.09525	-1.0387	0.0050938	0.023822	DOWN	-0.39991	0.20145	0.40292	
transcript_HQ_CoA_transcript13570/f3p0/2757	28.62	29.85666667	16.01666667	0.21258	0.36358	0.53883	-1.0319	1.6177E-05	0.00020393	DOWN	-0.83188	0.00067847	0.0065891	
transcript_HQ_CoA_transcript13594/f2p0/2751	16.67666667	16.97666667	8.966666667	0.1841	0.59296	0.74499	-1.0624	0.0021812	0.011888	DOWN	-0.89252	0.00015421	0.0020215	
transcript_HQ_CoA_transcript13608/f2p0/2728	6.846666667	7.45	3.64	0.28336	0.58749	0.74052	-1.1607	0.00061503	0.0043026	DOWN	-0.89088	0.079881	0.21933	
transcript_HQ_CoA_transcript13632/f2p0/2725	7.98	3.77	11.94	-0.9395	0.07887	0.17824	1.4991	4.8994E-07	1.0382E-05	UP	0.55044	0.26286	0.47461	
transcript_HQ_CoA_transcript1368/f2p0/5397	3.463333333	4.876666667	2.583333333	0.6466	0.029901	0.08329	-1.0572	5.6464E-05	0.00059754	DOWN	-0.42316	0.21096	0.41482	
transcript_HQ_CoA_transcript13686/f2p0/2744	7.6	11.65333333	5.666666667	0.76742	0.079766	0.17966	-1.1826	0.0087274	0.036089	DOWN	-0.4293	0.11134	0.27552	
transcript_HQ_CoA_transcript1369/f2p0/5434	3.403333333	8.963333333	1.916666667	1.4958	0.092994	0.2011	-2.3428	0.00022496	0.0018853	DOWN	-0.85387	0.31928	0.53627	
transcript_HQ_CoA_transcript13690/f10p0/2694	28.59	17.44666667	42.69666667	-0.5502	0.19251	0.34464	1.1357	0.00016999	0.0014987	UP	0.57286	0.19905	0.3997	

transcript_HQ_CoA_transcript13721/f16p0/2700	31.46333333	30.49	116.88	0.10738	0.59444	0.74574	1.8322	0.0098206	0.039452	UP	1.9268	0.034793	0.12399
transcript_HQ_CoA_transcript13728/f2p0/2711	3.003333333	2.286666667	5.09	-0.26416	0.57811	0.73272	1.0185	0.0096636	0.038963	UP	0.74501	0.024687	0.096473
transcript_HQ_CoA_transcript13833/f2p0/2769	0.306666667	0.746666667	0	1.468	0.2404	0.40402	-7.028	4.7633E-06	7.2035E-05	DOWN	-5.5721	0.049908	0.15989
transcript_HQ_CoA_transcript13854/f2p0/2728	3.416666667	1.39	3.64	-1.134	0.093751	0.20216	1.2305	0.0024095	0.012909	UP	0.082287	0.89223	0.9517
transcript_HQ_CoA_transcript1390/f2p0/5384	0.58	0.446666667	1.196666667	-0.23515	0.66233	0.79506	1.2761	0.0028589	0.014828	UP	1.0316	0.024092	0.094754
transcript_HQ_CoA_transcript13907/f2p0/2718	2.543333333	2.78	6.173333333	0.26097	0.76317	0.85987	1.0054	0.0073904	0.031746	UP	1.2597	0.11776	0.28558
transcript_HQ_CoA_transcript13920/f2p0/2725	3.623333333	1.266666667	7.243333333	-1.3885	0.095815	0.20555	2.3703	2.7375E-14	2.7722E-12	UP	0.97467	0.17564	0.36905
transcript_HQ_CoA_transcript14011/f3p0/2706	17.92666667	25.92333333	13.31666667	0.69503	0.005075	0.0201	-1.1142	1.3032E-05	0.00016976	DOWN	-0.43274	0.023146	0.091838
transcript_HQ_CoA_transcript14014/f2p0/2692	7.13	8.493333333	4.17	0.40629	0.53898	0.70132	-1.1578	0.0014928	0.008795	DOWN	-0.76687	0.21261	0.41697
transcript_HQ_CoA_transcript14015/f2p0/2733	0.893333333	0.333333333	1.436666667	-1.2855	0.06293	0.15006	1.9862	0.0017814	0.010159	UP	0.68916	0.19458	0.39359
transcript_HQ_CoA_transcript14110/f3p0/2712	8.343333333	4.113333333	16.66666667	-0.87619	0.0091593	0.032769	1.8772	4.4249E-10	1.8905E-08	UP	0.99063	0.00037388	0.0041118
transcript_HQ_CoA_transcript14131/f2p0/2599	3.273333333	2.873333333	0.606666667	-0.03874	0.97721	0.98845	-2.3519	0.0026708	0.014027	DOWN	-2.4042	0.069532	0.19995
transcript_HQ_CoA_transcript14192/f6p0/2621	7.24	3.616666667	10.73333333	-0.85198	0.022526	0.066822	1.4256	5.7808E-05	0.00060894	UP	0.56404	0.054367	0.1698
transcript_HQ_CoA_transcript14207/f2p0/2684	25.85	42.56333333	23.62333333	0.87608	0.0042248	0.017271	-1.0043	3.0606E-05	0.00035572	DOWN	-0.14061	0.56105	0.74548
transcript_HQ_CoA_transcript14273/f5p0/2651	0.743333333	1.48	0.54	1.1537	0.032085	0.088301	-1.6194	0.0046274	0.022059	DOWN	-0.48068	0.49786	0.69901
transcript_HQ_CoA_transcript14282/f2p0/2676	6.356666667	8.743333333	4.816666667	0.62424	0.048995	0.12329	-1.0234	0.0019191	0.010744	DOWN	-0.41195	0.13537	0.31199
transcript_HQ_CoA_transcript14304/f2p0/2668	11.37666667	22.66666667	10.10666667	1.1656	0.059087	0.1427	-1.2912	0.00038123	0.0029114	DOWN	-0.14116	0.8258	0.91897
transcript_HQ_CoA_transcript14389/f2p0/2657	1.803333333	1.483333333	3.663333333	-0.17269	0.80544	0.88705	1.1696	0.012127	0.046703	UP	0.99067	0.12402	0.29524
transcript_HQ_CoA_transcript14409/f2p0/2658	31.34666667	45.56666667	24.16	0.6797	0.058983	0.14258	-1.0494	0.0024319	0.013007	DOWN	-0.37958	0.38298	0.59878
transcript_HQ_CoA_transcript14414/f3p0/2693	10.93666667	17.78666667	7.99	0.858	0.05292	0.13097	-1.3066	1.9163E-06	3.3167E-05	DOWN	-0.462	0.25592	0.46643
transcript_HQ_CoA_transcript1451/f2p0/5324	3.54	1.073333333	5.883333333	-1.5702	0.087809	0.19311	2.2709	6.8155E-06	9.7824E-05	UP	0.69295	0.35972	0.57687
transcript_HQ_CoA_transcript14516/f2p0/2651	3.24	2.236666667	5.07	-0.38173	0.3585	0.53393	1.0377	0.0054653	0.025125	UP	0.64236	0.071005	0.20246
transcript_HQ_CoA_transcript14546/f3p0/2644	35.36666667	17.47666667	39.73666667	-0.86202	8.5838E-05	0.0006171	1.0478	0.00060614	0.0042518	UP	0.1747	0.57015	0.75197
transcript_HQ_CoA_transcript14564/f2p0/2645	1.45	1.706666667	0.523333333	0.3961	0.53152	0.69439	-1.8383	0.0055633	0.025486	DOWN	-1.4558	0.012273	0.058304
transcript_HQ_CoA_transcript14576/f2p0/2634	10.91333333	16.00333333	8.346666667	0.69669	0.044581	0.11458	-1.0732	0.0016864	0.0096988	DOWN	-0.38948	0.12938	0.30266
transcript_HQ_CoA_transcript14592/f2p0/2647	2.726666667	1.616666667	6.126666667	-0.64481	0.44798	0.62082	1.8365	0.0071226	0.030768	UP	1.1814	0.073057	0.20593
transcript_HQ_CoA_transcript14804/f3p0/2651	3.546666667	12.03333333	3.06	1.9401	0.070331	0.16385	-2.0783	0.0044933	0.021576	DOWN	-0.15682	0.88322	0.94788
transcript_HQ_CoA_transcript14842/f5p0/2621	39.71333333	18.28	44.87666667	-0.96124	0.00011805	0.0008138	1.1469	5.6396E-08	1.4938E-06	UP	0.17336	0.50292	0.70348
transcript_HQ_CoA_transcript14857/f4p0/2597	23.04666667	38.07	14.59	0.87375	0.0067904	0.025549	-1.5312	2.4596E-06	4.1359E-05	DOWN	-0.66887	0.00020384	0.002523
transcript_HQ_CoA_transcript14874/f2p0/2630	26.78333333	39.67	21	0.70261	0.1549	0.29514	-1.0568	3.8507E-05	0.000433	DOWN	-0.36237	0.45027	0.66039
transcript_HQ_CoA_transcript1493/f2p0/5500	1.416666667	0.69	2.89	-0.90337	0.090648	0.19748	1.9193	2.275E-11	1.2519E-09	UP	1.0064	0.026035	0.1005
transcript_HQ_CoA_transcript14948/f3p0/2602	1.846666667	1.186666667	2.646666667	-0.4804	0.40672	0.58114	1.0142	0.012915	0.049123	UP	0.51757	0.28708	0.50152
transcript_HQ_CoA_transcript14963/f2p0/2612	30.97666667	46.68	22.97	0.7738	0.022615	0.067051	-1.1812	3.086E-06	4.9968E-05	DOWN	-0.42299	0.17721	0.37097
transcript_HQ_CoA_transcript1499/f2p0/5261	0.706666667	1.563333333	0.453333333	1.3056	0.1362	0.26824	-1.9143	0.0011938	0.0073624	DOWN	-0.62454	0.4586	0.66743
transcript_HQ_CoA_transcript15021/f2p0/2608	7.056666667	3.596666667	8.92	-0.79838	0.06238	0.14898	1.1397	0.0057313	0.026108	UP	0.33047	0.18191	0.37757
transcript_HQ_CoA_transcript15023/f2p0/2619	1.616666667	3.803333333	1.53	1.3993	0.017421	0.054386	-1.4865	0.0076921	0.032753	DOWN	-0.096759	0.85374	0.93354
transcript_HQ_CoA_transcript1505/f2p0/5318	1.83	1.023333333	3.013333333	-0.69238	0.16862	0.3138	1.4024	1.0919E-05	0.00014661	UP	0.70135	0.11717	0.28484
transcript_HQ_CoA_transcript15059/f2p0/2598	9.58	4.523333333	12.68666667	-0.9303	0.015022	0.048607	1.3286	0.00078345	0.0052466	UP	0.38632	0.18141	0.37683
transcript_HQ_CoA_transcript1506/f3p0/5280	5.436666667	2.266666667	6.726666667	-1.1034	0.029001	0.08142	1.4037	0.00014877	0.0013369	UP	0.2903	0.43483	0.64635
transcript_HQ_CoA_transcript151/f2p0/7514	37.13	20.23	48.27666667	-0.73274	0.0024028	0.010807	1.113	6.8519E-10	2.7724E-08	UP	0.3701	0.10521	0.26575
transcript_HQ_CoA_transcript15135/f2p0/2591	4.303333333	8.286666667	2.63	1.0933	0.043176	0.11182	-1.782	0.0001853	0.0016158	DOWN	-0.70178	0.15411	0.33851
transcript_HQ_CoA_transcript15138/f6p0/2578	13.26666667	21.94333333	10.10666667	0.88438	0.010378	0.03632	-1.2725	8.9795E-05	0.00088297	DOWN	-0.40081	0.092612	0.24336
transcript_HQ_CoA_transcript1518/f2p0/5247	2.713333333	1.636666667	3.813333333	-0.58691	0.12889	0.25707	1.079	0.0059973	0.027082	UP	0.48131	0.098581	0.2545
transcript_HQ_CoA_transcript15205/f3p0/2593	5.743333333	3.016666667	8.186666667	-0.77453	0.14842	0.28582	1.2792	0.00022798	0.0019021	UP	0.49635	0.28197	0.49609
transcript_HQ_CoA_transcript15287/f2p0/2584	17.58	6.586666667	22.42666667	-1.2555	0.072662	0.16794	1.6327	5.8232E-05	0.00061095	UP	0.36305	0.57483	0.75488
transcript_HQ_CoA_transcript15291/f3p0/2577	5.453333333	3.366666667	8.093333333	-0.54481	0.20349	0.3586	1.1246	0.0055321	0.025368	UP	0.56793	0.21077	0.41451
transcript_HQ_CoA_transcript15305/f2p0/2591	1.57	0.403333333	3.19	-1.8325	0.016586	0.052389	2.85	3.123E-06	5.0457E-05	UP	1.0081	0.105	0.26536
transcript_HQ_CoA_transcript15317/f4p0/2564	2.02	0.466666667	7.553333333	-1.8594	0.26355	0.43101	3.7847	6.2887E-05	0.00065048	UP	1.9102	0.075652	0.2111
transcript_HQ_CoA_transcript15370/f3p0/2576	3.546666667	3.693333333	0.583333333	0.21439	0.86256	0.92304	-2.7556	0.00042052	0.0031636	DOWN	-2.5563	0.025935	0.10024
transcript_HQ_CoA_transcript15414/f3p0/2568	0.406666667	0.906666667	0.266666667	1.3053	0.09532	0.2047	-1.8999	0.0079056	0.033449	DOWN	-0.61371	0.51299	0.7106
transcript_HQ_CoA_transcript15427/f3p0/2571	28.50666667	11.91333333	30.56	-1.101	0.024605	0.071721	1.1991	0.0013807	0.0082703	UP	0.088493	0.82096	0.91691
transcript_HQ_CoA_transcript15519/f2p0/2632	6.756666667	9.996666667	4.406666667	0.72316	0.20953	0.36677	-1.3485	0.00025802	0.0021066	DOWN	-0.63466	0.23569	0.44453
transcript_HQ_CoA_transcript1558/f2p0/5240	7.473333333	9.97	5.32	0.59276	0.071094	0.16511	-1.0677	0.00016099	0.0014317	DOWN	-0.48953	0.063383	0.18774
transcript_HQ_CoA_transcript15676/f4p0/2554	22.36666667	36.51666667	19.93333333	0.86169	4.4229E-05	0.0003454	-1.015	6.7838E-08	1.7537E-06	DOWN	-0.16651	0.40156	0.61699
transcript_HQ_CoA_transcript15709/f2p0/2615	4.246666667	5.043333333	1.506666667	0.4035	0.32442	0.49756	-1.8725	0.00029968	0.0023799	DOWN	-1.4824	0.010209	0.051073
transcript_HQ_CoA_transcript15762/f3p0/2570	1.3	8.553333333	0.103333333	2.8097	0.24877	0.41388	-6.4687	0.0016747	0.0096436	DOWN	-3.6633	0.060978	0.18345
transcript_HQ_CoA_transcript15772/f2p0/2536	5.763333333	3.56	8.236666667	-0.54527	0.057204	0.13918	1.0585	0.0047934	0.022644	UP	0.50185	0.15919	0.34662
transcript_HQ_CoA_transcript15782/f2p0/2537	5.92	3.466666667	9.35	-0.63351	0.24876	0.41388	1.2821	5.7443E-09	1.8902E-07	UP	0.64167	0.18974	0.38727
transcript_HQ_CoA_transcript15820/f5p0/2535	18.46	31.03666667	14.75333333	0.91712	0.0044403	0.017964	-1.2254	8.9114E-10	3.5202E-08	DOWN	-0.32141	0.28878	0.50357
transcript_HQ_CoA_transcript15833/f2p0/2535	7.083333333	1.686666667	5.623333333	-1.9458	0.026292	0.07554	1.5908	0.00096521	0.0062031	UP	-0.3658	0.62112	0.7876
transcript_HQ_CoA_transcript15917/f2p0/2539	4.963333333	2.533333333	5.703333333	-0.83838	0.074464	0.17116	1.0424	0.0083514	0.034861	UP	0.19287	0.61726	0.78467
transcript_HQ_CoA_transcript15976/f2p0/2518	0.906666667	2.906666667	0.723333333	1.8477	0.089554	0.19582	-2.1458	0.013187	0.049886	DOWN	-0.32029	0.77036	0.88647
transcript_HQ_CoA_transcript15989/f6p0/2513	25.19	14.09333333	32.78333333	-0.67656	0.023457	0.06909	1.07	3.0072E-06	0.00004917	UP	0.38233	0.16938	0.36018
transcript_HQ_CoA_transcript16092/f5p0/2502	24.33333333	15.80666667	42.59333333	-0.47215	0.17295	0.3198	1.3039	0.00030097	0.002386	UP	0.82008	0.0086407	0.045355
transcript_HQ_CoA_transcript16093/f2p0/2489	3.33	2.113333333	5.276666667	-0.52325	0.28844	0.45883	1.1907	0.0059598	0.026932	UP	0.65688	0.037016	0.12949
transcript_HQ_CoA_transcript16147/f4p0/2436	13.72	7.163333333	18.76	-0.7755	0.016205	0.051416	1.2229	0.00039879	0.0030254	UP	0.4355	0.20726	0.40967
transcript_HQ_CoA_transcript16148/f2p0/2509	3.836666667	2.253333333	7.67	-0.62551	0.070584	0.16422	1.6241	4.4741E-09	1.5186E-07	UP	0.98685	0.00011827	0.0016359
transcript_HQ_CoA_transcript16223/f3p0/2530	17.56333333	21.62333333	11.51	0.44781	0.15074	0.28886	-1.0596	1.5478E-13	1.3705E-11	DOWN	-0.62281	0.042724	0.14343
transcript_HQ_CoA_transcript16244/f3p0/2496	18.82666667	9.12	27.74666667	-0.91864	0.037244	0.099213	1.4789	0.013009	0.049412	UP	0.54802	0.35742	0.57457
transcript_HQ_CoA_transcript16261/f2p0/2486	6.583333333	11.35666667	5.18	0.94818	0.00073386	0.0039206	-1.2907	7.4665E-06	0.0001056	DOWN	-0.35488	0.18633	0.38314
transcript_HQ_CoA_transcript16264/f3p0/2499	2.1	0.596666667	1.95	-1.6637	0.052303	0.12985	1.593	0.00087921	0.0057636	UP	-0.089711	0.90239	0.9567
transcript_HQ_CoA_transcript1627/f2p0/5185	0.32	4.853333333	0	4.1243	0.082912	0.18492	-10.712	4.8744E-17	7.3837E-15	DOWN	-6.6102	0.051886	0.16454
transcript_HQ_CoA_transcript16295/f4p0/2486	14.42333333	11.20333333	27.26333333	-0.18095	0.72701	0.83661	1.1097	0.0067939	0.029706	UP	0.91395	0.04753	0.15476
transcript_HQ_CoA_transcript16318/f2p0/2488	2.113333333	0.646666667	3.806666667	-1.5263	0.029678	0.08281	2.4167	0.0011095	0.0069526	UP	0.87543	0.14166	0.32116
transcript_HQ_CoA_transcript16333/f2p0/2484	3.99	2.396666667	7.043333333	-0.60266	0.31362	0.48598	1.4098	0.00003695	0.00041806	UP	0.7965	0.13886	0.31712
transcript_HQ_CoA_transcript16349/f4p0/2468	3.52	5.25	2.873333333	0.73683	0.035938	0.096508	-1.0257	0.00045311	0.0033637	DOWN	-0.303	0.40028	0.61589
transcript_HQ_CoA_transcript16477/f6p0/2460	16.63333333	9.733333333	26.25333333	-0.61225	0.065258	0.15438	1.2732	0.00019554	0.0016922	UP	0.64857	0.017344	0.074728
transcript_HQ_CoA_transcript16525/f2p0/2469	7.376666667	4.476666667	10.02333333	-0.58002	0.050463	0.12619	1.0246	0.00013939	0.0012629	UP	0.43389	0.07137	0.2032
transcript_HQ_CoA_transcript16536/f2p0/2466	0.55	0.123333333	0.726666667	-2.0939	0.050531	0.12632	2.4769	0.0048688	0.022959	UP	0.36962	0.64379	0.80264

transcript_HQ_CoA_transcript16662/f2p0/2458	9.293333333	15.24666667	7.636666667	0.87418	0.014213	0.046617		-1.1482	0.00055665	0.00397	DOWN	-0.28642	0.35422	0.57162
transcript_HQ_CoA_transcript16804/f2p0/2455	7.14	7.253333333	4.006666667	0.17105	0.62004	0.76468		-1.0002	0.00018193	0.0015901	DOWN	-0.83758	0.026616	0.10208
transcript_HQ_CoA_transcript16807/f2p0/2457	2.133333333	3.326666667	1.21	0.81374	0.20305	0.35819		-1.616	2.4733E-06	4.1552E-05	DOWN	-0.81503	0.2285	0.43574
transcript_HQ_CoA_transcript16811/f3p0/2449	11.13333333	13.45666667	6.906666667	0.4434	0.14213	0.27697		-1.1056	0.012564	0.048045	DOWN	-0.67704	0.11139	0.27552
transcript_HQ_CoA_transcript16838/f2p0/2424	11.43666667	6.456666667	18.59	-0.66673	0.38452	0.5601		1.3498	0.006451	0.028526	UP	0.67702	0.28664	0.50094
transcript_HQ_CoA_transcript16929/f2p0/2462	6.033333333	9.5	4.92	0.80061	0.059041	0.14264		-1.0979	0.0049949	0.023421	DOWN	-0.30973	0.37044	0.58763
transcript_HQ_CoA_transcript16939/f2p0/2441	312.0033333	322.1066667	153.8433333	0.18132	0.7469	0.84908		-1.1968	0.0044434	0.021382	DOWN	-1.0229	0.22561	0.43277
transcript_HQ_CoA_transcript16950/f2p0/2429	5.11	6.103333333	3.306666667	0.40532	0.12371	0.24933		-1.0268	0.0011575	0.0071796	DOWN	-0.63265	0.073727	0.20711
transcript_HQ_CoA_transcript17021/f2p0/2437	4.543333333	12.5	3.36	1.5878	0.035714	0.096045		-2.0221	0.0052626	0.024383	DOWN	-0.44525	0.41876	0.63159
transcript_HQ_CoA_transcript17031/f3p0/2437	9.91	12.50333333	5.866666667	0.48067	0.1442	0.27974		-1.2279	0.00011474	0.0010757	DOWN	-0.76008	0.00089718	0.0082052
transcript_HQ_CoA_transcript17046/f3p0/2442	8.376666667	4.686666667	15.74666667	-0.66137	0.13778	0.27074		1.5871	3.8491E-07	0.0000083	UP	0.91275	0.0087472	0.045716
transcript_HQ_CoA_transcript17096/f2p0/2429	8.043333333	3.703333333	10.18	-0.9843	0.027354	0.077858		1.311	4.1526E-06	6.4054E-05	UP	0.31703	0.41816	0.63099
transcript_HQ_CoA_transcript1714/f2p0/5164	0.983333333	2.116666667	0.746666667	1.2805	0.015677	0.050124		-1.6471	6.0325E-05	0.00062788	DOWN	-0.38567	0.52995	0.72324
transcript_HQ_CoA_transcript17171/f4p0/2404	9.366666667	4.233333333	10.94666667	-0.97193	0.0076985	0.028376		1.1941	0.0008945	0.0058534	UP	0.21152	0.49749	0.69891
transcript_HQ_CoA_transcript17172/f4p0/2411	16.36333333	28.49666667	10.73666667	0.94874	0.0040488	0.016713		-1.5527	7.9229E-09	2.5256E-07	DOWN	-0.61639	0.044692	0.14823
transcript_HQ_CoA_transcript17173/f2p0/2441	6.776666667	8.116666667	4.363333333	0.41678	0.072776	0.16812		-1.0403	8.7999E-06	0.00012127	DOWN	-0.63603	0.013482	0.062311
transcript_HQ_CoA_transcript1723/f2p0/5114	3.74	5.673333333	2.033333333	0.74537	0.0046669	0.018754		-1.6255	7.0597E-11	3.5451E-09	DOWN	-0.89087	0.0023817	0.017417
transcript_HQ_CoA_transcript17278/f2p0/2427	1.583333333	2.88	1.46	1.017	0.047023	0.11937		-1.122	0.0049711	0.023339	DOWN	-0.11869	0.81463	0.91369
transcript_HQ_CoA_transcript17297/f2p0/2426	0.16	0.07	0.57	-1.0942	0.62385	0.76746		2.9392	0.012405	0.047557	UP	1.8202	0.13708	0.31458
transcript_HQ_CoA_transcript17327/f3p0/2389	2.89	1.163333333	3.496666667	-1.1988	0.1157	0.23716		1.4526	0.00020585	0.0017672	UP	0.24328	0.71896	0.85431
transcript_HQ_CoA_transcript1736/f2p0/5117	2.853333333	1.363333333	3.963333333	-0.90499	0.084069	0.18666		1.3683	2.2105E-05	0.00026621	UP	0.45301	0.28552	0.49968
transcript_HQ_CoA_transcript17393/f4p0/2390	5.296666667	3.383333333	7.593333333	-0.50355	0.36425	0.53943		1.0102	0.00017177	0.0015122	UP	0.49684	0.31594	0.53271
transcript_HQ_CoA_transcript17453/f2p0/2435	5.666666667	0.68	4.586666667	-2.892	NA	NA	NA	2.5534	0.00007484	0.00075536	UP	-0.345	NA	NA
transcript_HQ_CoA_transcript17476/f8p0/2329	12.28333333	27.27333333	11.22666667	1.3143	0.060092	0.1447		-1.4245	2.3776E-06	4.0166E-05	DOWN	-0.12522	0.84987	0.93149
transcript_HQ_CoA_transcript17483/f2p0/2409	5.51	2.323333333	9.36	-1.0565	0.017765	0.055214		1.8335	6.9462E-05	0.00070811	UP	0.76546	0.028655	0.10802
transcript_HQ_CoA_transcript17512/f2p0/2398	5.756666667	10.11333333	5.376666667	0.95404	0.021251	0.063713		-1.0488	0.0034362	0.017341	DOWN	-0.10498	0.80747	0.90932
transcript_HQ_CoA_transcript1757/f2p0/5080	3.49	1.853333333	4.406666667	-0.75082	0.020265	0.061333		1.0961	0.00030251	0.0023951	UP	0.33177	0.33499	0.55268
transcript_HQ_CoA_transcript17575/f2p0/2364	13.96333333	8.47	28.07666667	-0.57604	0.013497	0.044767		1.5754	5.3064E-05	0.00056613	UP	0.98845	0.0066751	0.037711
transcript_HQ_CoA_transcript17577/f3p0/2458	5.963333333	10.17	3.153333333	0.95088	0.060763	0.14596		-1.8559	0.0021779	0.011873	DOWN	-0.91976	0.063235	0.18739
transcript_HQ_CoA_transcript17640/f2p0/2391	5.686666667	8.11	3.426666667	0.67242	0.15981	0.30179		-1.3909	0.00060958	0.0042726	DOWN	-0.73268	0.068998	0.19897
transcript_HQ_CoA_transcript17642/f4p0/2366	12.63	6	15.13333333	-0.93136	0.0022587	0.010264		1.2071	0.0010529	0.006664	UP	0.26293	0.38929	0.60502
transcript_HQ_CoA_transcript17651/f2p0/2375	5.79	2.75	15.94333333	-0.8946	0.20751	0.36384		2.4354	0.002642	0.013915	UP	1.5226	0.063546	0.18806
transcript_HQ_CoA_transcript17657/f3p0/2367	12.88333333	4.72	15.24333333	-1.2913	0.026644	0.076408		1.5292	0.0001083	0.0010296	UP	0.22912	0.63986	0.79975
transcript_HQ_CoA_transcript1770/f2p0/4890	2.8	4.426666667	2.343333333	0.79748	0.04754	0.12031		-1.0546	0.0049127	0.023136	DOWN	-0.26917	0.46427	0.6718
transcript_HQ_CoA_transcript17748/f2p0/2378	12.51333333	12.09	6.46	0.10134	0.68058	0.80663		-1.0572	5.662E-06	8.3223E-05	DOWN	-0.968	0.00017295	0.0022152
transcript_HQ_CoA_transcript17761/f7p0/2357	8.236666667	6.61	15.13333333	-0.16319	0.59679	0.7474		1.0475	0.000135	0.0012323	UP	0.87295	0.0036862	0.024321
transcript_HQ_CoA_transcript17809/f2p0/2369	9.743333333	16.19	8.55	0.8825	0.013225	0.044016		-1.0611	1.419E-06	2.5675E-05	DOWN	-0.19063	0.5863	0.76328
transcript_HQ_CoA_transcript1781/f2p0/5059	1.986666667	0.786666667	3.81	-1.1509	0.075397	0.17256		2.1156	7.0906E-08	1.8076E-06	UP	0.9493	0.052414	0.16565
transcript_HQ_CoA_transcript17849/f4p0/2369	13.51333333	13.55333333	4.793333333	0.17252	0.66109	0.79421		-1.6333	0.0051645	0.024039	DOWN	-1.4772	0.018675	0.079163
transcript_HQ_CoA_transcript17897/f3p0/2358	6.746666667	2.23	8.78	-1.4787	0.054678	0.13409		1.8654	0.0046851	0.022281	UP	0.37554	0.40625	0.62046
transcript_HQ_CoA_transcript17947/f2p0/2354	0.046666667	0.093333333	14.93666667	1.2593	0.68841	0.81154		7.2018	0.0087284	0.036089	UP	8.4362	0.016965	0.07346
transcript_HQ_CoA_transcript1803/f2p0/5060	13.08	20.30666667	9.56	0.79429	0.001085	0.0055085		-1.2311	0.00001494	0.00019003	DOWN	-0.4506	0.065132	0.19143
transcript_HQ_CoA_transcript18099/f2p0/2342	1.79	2.853333333	0.553333333	0.83919	0.37393	0.54927		-2.5478	8.7603E-05	0.00086373	DOWN	-1.7261	0.080605	0.22066
transcript_HQ_CoA_transcript1821/f2p0/5056	3.733333333	2.233333333	5.893333333	-0.58895	0.067817	0.15901		1.2474	1.8433E-05	0.00022736	UP	0.64815	0.055974	0.1732
transcript_HQ_CoA_transcript18224/f2p0/2340	16.17333333	7.863333333	17.59	-0.87728	1.2334E-05	0.00011264		1.0048	9.0996E-07	1.7631E-05	UP	0.11524	0.55318	0.73985
transcript_HQ_CoA_transcript18235/f3p0/2347	11.89333333	8.196666667	22.01333333	-0.36745	0.41851	0.59285		1.2738	9.3622E-09	2.9535E-07	UP	0.89079	0.03416	0.12244
transcript_HQ_CoA_transcript18270/f2p0/2342	5.306666667	2.433333333	7.286666667	-0.96408	0.01125	0.038745		1.4433	8.7158E-05	0.00085981	UP	0.4663	0.15246	0.33637
transcript_HQ_CoA_transcript18273/f2p0/2338	5.213333333	2.523333333	9.383333333	-0.89115	0.068006	0.15929		1.7467	0.00099781	0.0063747	UP	0.84391	0.12418	0.2954
transcript_HQ_CoA_transcript18301/f3p0/2342	3.543333333	2.87	6.48	-0.17931	0.74981	0.85069		1.0445	0.0026482	0.013938	UP	0.85621	0.06281	0.18668
transcript_HQ_CoA_transcript1835/f2p0/5026	3.453333333	1.42	3.593333333	-1.1421	0.12915	0.25742		1.1754	0.0012038	0.0074016	UP	0.026959	0.9684	0.98636
transcript_HQ_CoA_transcript18453/f3p0/2281	15.41	27.66333333	13.28333333	0.99705	0.0007777	0.0041307		-1.2019	0.00015958	0.0014199	DOWN	-0.21549	0.41548	0.62875
transcript_HQ_CoA_transcript18553/f2p0/2318	0.893333333	0.526666667	0.07	-0.56627	0.67907	0.80577		-3.0549	0.0042413	0.020664	DOWN	-3.6192	0.027077	0.10348
transcript_HQ_CoA_transcript18597/f6p0/2310	24.74333333	11.24666667	32.70666667	-0.98204	3.7097E-05	0.00029642		1.4035	2.0395E-08	5.9055E-07	UP	0.40983	0.10317	0.26239
transcript_HQ_CoA_transcript18599/f15p0/2237	33.40333333	19.14666667	49.73	-0.61508	0.14829	0.2857		1.2028	0.0031191	0.015978	UP	0.57355	0.0018393	0.014335
transcript_HQ_CoA_transcript18645/f5p0/2302	13.56	9.726666667	54.34666667	-0.31161	0.67371	0.80271		2.3952	0.0048067	0.022701	UP	2.066	0.098429	0.25425
transcript_HQ_CoA_transcript18647/f5p0/2312	7.57	11.74333333	4.52	0.79228	0.18721	0.33834		-1.5125	5.6157E-05	0.00059489	DOWN	-0.73383	0.23751	0.4459
transcript_HQ_CoA_transcript18697/f3p0/2328	17.03666667	29.61333333	9.8	0.96395	0.026818	0.076823		-1.7325	4.5473E-08	1.2257E-06	DOWN	-0.78353	0.10362	0.26302
transcript_HQ_CoA_transcript18729/f3p0/2285	61.97	30.7	70.16	-0.85264	0.00087663	0.0045855		1.0364	3.5598E-06	5.6442E-05	UP	0.1717	0.3597	0.57687
transcript_HQ_CoA_transcript18771/f15p0/2247	37.05333333	21.65666667	48.25333333	-0.61767	0.019574	0.059634		1.0029	1.0513E-05	0.00014189	UP	0.37406	0.070292	0.20131
transcript_HQ_CoA_transcript18895/f2p0/2271	1.576666667	0.743333333	1.94	-0.92427	0.10467	0.21989		1.2251	0.0059033	0.026716	UP	0.28807	0.55283	0.73975
transcript_HQ_CoA_transcript18954/f2p0/2279	5.8	1.573333333	6.926666667	-1.7857	0.077518	0.17603		2.0105	0.0018164	0.010298	UP	0.21888	0.79032	0.89895
transcript_HQ_CoA_transcript19070/f2p0/2271	24.29666667	11.47666667	29.07666667	-0.9285	4.6362E-05	0.00036006		1.195	4.0032E-11	2.0964E-09	UP	0.25315	0.18873	0.38604
transcript_HQ_CoA_transcript19156/f5p0/2260	24.24333333	36.32333333	18.51333333	0.74198	0.00032648	0.0019614		-1.1205	7.4065E-10	2.9771E-08	DOWN	-0.39119	0.10373	0.26323
transcript_HQ_CoA_transcript19211/f2p0/2265	9.813333333	5.003333333	15.32333333	-0.84566	0.099104	0.21103		1.4817	0.007198	0.031014	UP	0.62672	0.072335	0.20483
transcript_HQ_CoA_transcript19216/f3p0/2258	14.11666667	15.30666667	3.436666667	0.26966	0.76993	0.86408		-2.327	1.7338E-05	0.00021562	DOWN	-2.0623	0.015652	0.069512
transcript_HQ_CoA_transcript19254/f3p0/2263	3.97	2.23	5.003333333	-0.69701	0.15774	0.2993		1.0277	0.01123	0.043953	UP	0.31869	0.33914	0.55704
transcript_HQ_CoA_transcript19260/f2p0/2264	8.72	14.47333333	3.8	0.89509	0.14357	0.27901		-2.0629	0.00084015	0.0055433	DOWN	-1.1854	0.12927	0.30265
transcript_HQ_CoA_transcript19280/f2p0/2266	4.983333333	11.15666667	4.333333333	1.2956	0.019839	0.06029		-1.4894	0.0095023	0.038456	DOWN	-0.2032	0.59027	0.76655
transcript_HQ_CoA_transcript19283/f2p0/2262	6.32	4.52	10.9	-0.32063	0.45076	0.62318		1.1201	1.6814E-08	4.9941E-07	UP	0.78566	0.039367	0.13577
transcript_HQ_CoA_transcript19293/f2p0/2259	14.5	21.18666667	10.47333333	0.71284	0.020247	0.061305		-1.1699	0.00069467	0.0047652	DOWN	-0.47132	0.076839	0.2135
transcript_HQ_CoA_transcript19297/f2p0/2261	3.4	1.74	5.643333333	-0.78102	0.21738	0.3759		1.516	0.0080832	0.034012	UP	0.72293	0.022401	0.089854
transcript_HQ_CoA_transcript19301/f2p0/2254	7.416666667	5.41	14.82666667	-0.28931	0.4629	0.63472		1.284	0.00027618	0.0022256	UP	0.98366	0.0077148	0.041649
transcript_HQ_CoA_transcript19302/f4p0/2260	0.646666667	0.2	55.15	-1.5305	0.64463	0.78213		7.9499	0.00089705	0.0058617	UP	6.4126	0.016628	0.07241
transcript_HQ_CoA_transcript19360/f3p0/2255	8.693333333	14.80666667	3.72	0.90564	0.079549	0.17924		-2.1178	0.00025595	0.0020934	DOWN	-1.2235	0.011625	0.056183
transcript_HQ_CoA_transcript1937/f2p0/4949	4.32	7.223333333	3.35	0.93051	0.037323	0.099328		-1.2843	0.0043693	0.021103	DOWN	-0.36949	0.20471	0.40718
transcript_HQ_CoA_transcript19373/f3p0/2255	25.25666667	17.77666667	44.93666667	-0.36769	0.27853	0.44727		1.1876	4.0868E-05	0.00045508	UP	0.81033	0.017736	0.076078
transcript_HQ_CoA_transcript19385/f4p0/2221	7.57	4.513333333	11.13333333	-0.58255	0.087603	0.19273		1.131	0.0011218	0.0070106	UP	0.5366	0.087316	0.23331
transcript_HQ_CoA_transcript19514/f2p0/2254	5.296666667	8.463333333	4.376666667	0.8313	0.015352	0.049395		-1.1012	0.0018295	0.010353	DOWN	-0.28094	0.49456	0.69647
transcript_HQ_CoA_transcript19528/f2p0/2241	2.42	1.263333333	4.213333333	-0.75623	0.33294	0.50727		1.5774	0.00011745	0.0010938	UP	0.80454	0.23128	0.43935

transcript_HQ_CoA_transcript19561/f2p0/2228	32.43333333	16.37666667	37.43666667	-0.83709	0.036637	0.09801		1.0486	0.0016781	0.009657	UP	0.20026	0.62903	0.79335	
transcript_HQ_CoA_transcript1959/f2p0/4936	0.673333333	1.713333333	6.573333333	1.4923	0.21302	0.37079		1.7935	3.974E-09	1.3615E-07	UP	3.2732	0.029852	0.11139	
transcript_HQ_CoA_transcript19683/f3p0/2232	5.74	9.866666667	4.776666667	0.95281	0.00093742	0.0048642		-1.199	3.3535E-05	0.00038607	DOWN	-0.2591	0.40126	0.6168	
transcript_HQ_CoA_transcript19695/f2p0/2218	4.083333333	6.736666667	1.25	0.88014	0.27629	0.44488		-2.5473	0.0064205	0.02846	DOWN	-1.6854	0.032545	0.11818	
transcript_HQ_CoA_transcript19729/f2p0/2224	1.656666667	2.716666667	1.366666667	0.87707	0.025515	0.073748		-1.146	0.0018438	0.010411	DOWN	-0.28027	0.54103	0.73202	
transcript_HQ_CoA_transcript19755/f3p0/2223	47.14	80.45333333	36.77666667	0.91916	1.8855E-06	2.0917E-05		-1.2605	0.00003981	0.00044519	DOWN	-0.35352	0.21913	0.42511	
transcript_HQ_CoA_transcript19776/f2p0/2221	11.29666667	3.983333333	16.89666667	-1.3142	0.10795	0.22481		1.915	0.00005242	0.00056023	UP	0.58506	0.38884	0.60458	
transcript_HQ_CoA_transcript1979/f2p0/4955	2.716666667	1.336666667	6.26	-0.84177	0.15836	0.30007		2.0854	1.0532E-08	3.2997E-07	UP	1.226	0.025154	0.097837	
transcript_HQ_CoA_transcript1983/f3p0/4912	8.81	10.66333333	5.3	0.42815	0.011282	0.038833		-1.1483	8.6978E-07	0.00001696	DOWN	-0.73252	0.0029189	0.020325	
transcript_HQ_CoA_transcript20003/f6p0/2197	16.15	54.07	12.10333333	1.9543	NA	NA	NA	-2.3033	3.857E-21	1.0876E-18	DOWN	-0.37121	NA	NA	
transcript_HQ_CoA_transcript20004/f4p0/2198	15.24666667	22.80333333	8.316666667	0.73245	0.010884	0.037751		-1.5954	5.5836E-07	1.1603E-05	DOWN	-0.8772	0.0022841	0.016911	
transcript_HQ_CoA_transcript20012/f2p0/2215	6.643333333	3.226666667	7.536666667	-0.87544	0.032193	0.088544		1.0608	0.0042561	0.020717	UP	0.1724	0.58031	0.75899	
transcript_HQ_CoA_transcript20062/f3p0/2217	6.983333333	11.27666667	6.193333333	0.85926	0.0077202	0.028438		-1.0157	0.0044308	0.021327	DOWN	-0.16962	0.64219	0.80121	
transcript_HQ_CoA_transcript20069/f2p0/2196	1.203333333	1.943333333	0.593333333	0.83978	0.27937	0.44815		-1.864	0.0041583	0.020319	DOWN	-1.0396	0.23531	0.44407	
transcript_HQ_CoA_transcript20084/f2p0/2216	2.123333333	2.883333333	0.62	0.61514	0.43653	0.61023		-2.3446	0.0025755	0.013644	DOWN	-1.7398	0.090312	0.2392	
transcript_HQ_CoA_transcript20131/f2p0/2193	0.256666667	7.196666667	0	4.9303	0.18772	0.33892		-9.9437	0.010931	0.043032	DOWN	-5.0185	0.20083	0.40218	
transcript_HQ_CoA_transcript20143/f2p0/2198	6.11	3.313333333	9.606666667	-0.72237	0.033397	0.091196		1.3963	0.0032151	0.01641	UP	0.66083	0.12911	0.30242	
transcript_HQ_CoA_transcript20201/f7p0/2184	51.27333333	91.20666667	40.8	0.99671	1.0074E-07	0.00000143		-1.3152	5.8534E-19	1.1536E-16	DOWN	-0.33202	0.058316	0.17766	
transcript_HQ_CoA_transcript2021/f20p0/4824	15.76666667	8.533333333	22.89666667	-0.74126	0.046649	0.11862		1.2783	0.0040646	0.019952	UP	0.52489	0.16529	0.35464	
transcript_HQ_CoA_transcript2022/f2p0/4899	2.856666667	1.65	4.156666667	-0.64828	0.10109	0.2141		1.1841	0.0013815	0.0082703	UP	0.52394	0.14124	0.32061	
transcript_HQ_CoA_transcript20352/f2p0/2177	5.47	6.563333333	3.19	0.42637	0.15639	0.29735		-1.1865	0.00025425	0.0020823	DOWN	-0.77263	0.024768	0.096667	
transcript_HQ_CoA_transcript20382/f2p0/2214	11.46333333	18.04333333	9.193333333	0.81551	0.028555	0.08054		-1.1362	0.00053278	0.0038385	DOWN	-0.3315	0.22081	0.42703	
transcript_HQ_CoA_transcript20394/f2p0/2148	8.89	19.43333333	6.713333333	1.2549	0.022849	0.067649		-1.6471	0.0020916	0.011512	DOWN	-0.40437	0.24091	0.44988	
transcript_HQ_CoA_transcript20467/f2p0/2175	6.52	7.983333333	2.32	0.44725	0.52374	0.68811		-1.9172	2.3959E-05	0.00028553	DOWN	-1.4864	0.032904	0.11915	
transcript_HQ_CoA_transcript20471/f2p0/2177	17.04666667	3.36	24.80666667	-2.1177	0.019652	0.05982		2.685	0.0013157	0.0079565	UP	0.54996	0.21327	0.41799	
transcript_HQ_CoA_transcript20475/f2p0/2197	7.02	3.74	10.07	-0.74285	0.015761	0.05033		1.2826	1.1146E-06	2.0804E-05	UP	0.52738	0.071406	0.20325	
transcript_HQ_CoA_transcript20526/f2p0/2182	3.87	6.506666667	3.503333333	0.89601	0.0040687	0.016765		-1.0393	0.0003005	0.0023834	DOWN	-0.15617	0.62218	0.7882	
transcript_HQ_CoA_transcript2064/f3p0/4893	4.406666667	1.496666667	6.52	-1.4461	0.067744	0.1589		1.9814	0.00010272	0.00098899	UP	0.52828	0.42807	0.64031	
transcript_HQ_CoA_transcript20656/f3p0/2158	11.41333333	5.85	13.97333333	-0.80427	0.0062073	0.023711		1.0979	3.435E-06	5.4558E-05	UP	0.28215	0.19438	0.39341	
transcript_HQ_CoA_transcript20708/f2p0/2154	5.76	8.783333333	4.36	0.7541	0.033776	0.091997		-1.1528	0.0046662	0.022203	DOWN	-0.41047	0.39119	0.60707	
transcript_HQ_CoA_transcript20723/f3p0/2194	2.066666667	7.303333333	0.936666667	2.024	NA	NA	NA	-3.117	4.3391E-14	4.2304E-12	DOWN	-1.1132	NA	NA	NA
transcript_HQ_CoA_transcript20787/f2p0/2153	8.486666667	3.606666667	10.15666667	-1.073	0.016046	0.051028		1.3243	0.0015761	0.0091765	UP	0.24197	0.52074	0.7165	
transcript_HQ_CoA_transcript20891/f3p0/2134	3.663333333	2.323333333	5.883333333	-0.48993	0.2526	0.41837		1.1857	0.00052205	0.0037761	UP	0.68357	0.02915	0.10953	
transcript_HQ_CoA_transcript20979/f2p0/2141	19.48333333	10.55	23.31333333	-0.74999	0.088699	0.19439		1.0006	1.0035E-06	0.00001908	UP	0.24166	0.55357	0.74014	
transcript_HQ_CoA_transcript2099/f2p0/4868	2.153333333	3.123333333	1.4	0.69295	0.092134	0.19994		-1.2935	0.00079587	0.00532	DOWN	-0.61464	0.18688	0.38373	
transcript_HQ_CoA_transcript21171/f2p0/2160	11.48666667	13.45666667	5.156666667	0.35888	0.53526	0.69778		-1.5258	1.5574E-06	2.7822E-05	DOWN	-1.1747	0.04324	0.14467	
transcript_HQ_CoA_transcript21325/f3p0/2121	64.08333333	32.59666667	94.9	-0.82559	1.0692E-05	9.9223E-05		1.4027	3.2076E-16	4.2296E-14	UP	0.56555	0.0014114	0.011761	
transcript_HQ_CoA_transcript2133/f2p0/4870	1.01	1.57	0.686666667	0.78688	0.090998	0.19807		-1.3418	9.2501E-05	0.00090328	DOWN	-0.56951	0.26237	0.47409	
transcript_HQ_CoA_transcript2148/f2p0/4819	6.73	6.006666667	2.67	-0.0086813	0.98989	0.99532		-1.2915	0.012988	0.04935	DOWN	-1.3161	0.060595	0.18266	
transcript_HQ_CoA_transcript21502/f10p0/2070	156.65	210.45	104.52	0.5607	0.26257	0.42975		-1.1387	0.0037002	0.01845	DOWN	-0.58663	0.23336	0.44176	
transcript_HQ_CoA_transcript2152/f2p0/4830	15.41333333	8.066666667	19.98666667	-0.76751	0.019123	0.058556		1.1461	0.00022793	0.0019021	UP	0.36696	0.030153	0.11217	
transcript_HQ_CoA_transcript21543/f2p0/2120	27.07666667	45.10333333	23.60333333	0.8999	0.0070493	0.02635		-1.0951	0.0012789	0.0077668	DOWN	-0.20845	0.32107	0.53824	
transcript_HQ_CoA_transcript21590/f2p0/2092	20.15666667	32.72333333	13.55666667	0.86183	0.0010154	0.0052143		-1.4173	1.3708E-11	7.9076E-10	DOWN	-0.56878	0.027885	0.10575	
transcript_HQ_CoA_transcript21662/f2p0/2097	5.843333333	4.003333333	13.69	-0.42038	0.45216	0.62459		1.6336	6.8365E-07	0.00001377	UP	1.2055	0.021039	0.08618	
transcript_HQ_CoA_transcript21688/f2p0/2101	2.82	0.503333333	4.513333333	-2.3268	0.026	0.07495		2.9814	0.00033197	0.0025848	UP	0.64883	0.28121	0.49522	
transcript_HQ_CoA_transcript21697/f2p0/2093	2.62	9.826666667	1.17	2.1204	0.020756	0.062502		-3.2917	0.00046819	0.0034617	DOWN	-1.1801	0.011238	0.054963	
transcript_HQ_CoA_transcript21726/f2p0/2094	5.846666667	8.876666667	4.693333333	0.7599	0.0062024	0.023698		-1.0692	0.00011549	0.0010806	DOWN	-0.32071	0.31597	0.53271	
transcript_HQ_CoA_transcript21739/f2p0/2078	8.59	4.16	10.08666667	-0.90855	0.011976	0.040741		1.143	0.00027553	0.0022237	UP	0.22406	0.52936	0.72273	
transcript_HQ_CoA_transcript21748/f2p0/2065	9.036666667	14.26666667	6.836666667	0.84157	0.04013	0.10538		-1.2166	1.2917E-06	2.3605E-05	DOWN	-0.39003	0.33018	0.54717	
transcript_HQ_CoA_transcript21752/f2p0/2098	9.723333333	8.116666667	3.403333333	-0.15256	0.84658	0.91345		-1.3802	0.012603	0.048167	DOWN	-1.5403	0.021908	0.088653	
transcript_HQ_CoA_transcript21824/f2p0/2068	31.20333333	12.09333333	34.05333333	-1.2418	0.01754	0.054683		1.346	4.7892E-05	0.00052219	UP	0.096055	0.84916	0.93111	
transcript_HQ_CoA_transcript21932/f2p0/2065	16.96333333	8.16	23.32666667	-0.89734	0.0042804	0.01744		1.3623	6.8685E-09	2.2173E-07	UP	0.45405	0.092813	0.24379	
transcript_HQ_CoA_transcript21943/f3p0/2086	5.686666667	13.06	6.15	1.3332	0.046292	0.11787		-1.2343	6.8304E-08	1.7594E-06	DOWN	0.092093	0.89135	0.95154	
transcript_HQ_CoA_transcript21971/f2p0/2064	6.78	6.026666667	1.936666667	0.0025167	0.99737	0.9987		-1.7958	0.0011531	0.0071593	DOWN	-1.8087	0.033986	0.12208	
transcript_HQ_CoA_transcript22038/f5p0/2019	26.23666667	44.97666667	23.51333333	0.93802	8.8277E-05	0.00063264		-1.0895	5.2196E-07	1.0984E-05	DOWN	-0.16382	0.34771	0.56554	
transcript_HQ_CoA_transcript22065/f3p0/2067	20.19666667	14.28	31.84666667	-0.34416	0.32198	0.49528		1.008	2.3174E-05	0.00027672	UP	0.65145	0.019582	0.081929	
transcript_HQ_CoA_transcript22084/f2p0/2064	106.3233333	153.77	68.87333333	0.69478	6.1484E-06	6.0891E-05		-1.308	0.00055589	0.0039661	DOWN	-0.62777	0.076873	0.21356	
transcript_HQ_CoA_transcript22200/f2p0/2072	6.816666667	18.25	4.5	1.5334	0.047726	0.12065		-2.1404	0.00002438	0.00028999	DOWN	-0.61265	0.45331	0.66323	
transcript_HQ_CoA_transcript2230/f4p0/4787	2.316666667	1.08	3.533333333	-0.97562	0.17257	0.31938		1.5598	4.0771E-08	1.1147E-06	UP	0.57671	0.36941	0.58654	
transcript_HQ_CoA_transcript22384/f5p0/2014	33.16666667	56.00666667	25.93	0.91344	4.2404E-05	0.000333		-1.2609	2.6079E-07	5.8579E-06	DOWN	-0.35977	0.1625	0.35097	
transcript_HQ_CoA_transcript22425/f2p0/2034	15.57	7.673333333	17.29666667	-0.85277	0.014584	0.04751		1.0053	0.001941	0.01083	UP	0.14062	0.6291	0.79335	
transcript_HQ_CoA_transcript22490/f3p0/2038	13.10666667	24.55333333	10.76666667	1.0442	0.092668	0.20064		-1.3303	0.0016197	0.0093875	DOWN	-0.29339	0.63578	0.79766	
transcript_HQ_CoA_transcript22554/f2p0/2005	18.00333333	33.7	16.42666667	1.0644	0.017681	0.05502		-1.1955	0.0018491	0.010428	DOWN	-0.1422	0.71896	0.85431	
transcript_HQ_CoA_transcript22571/f3p0/2011	115.6433333	201.1733333	78.4	0.93805	0.035501	0.095657		-1.4938	7.6862E-06	0.00010779	DOWN	-0.56498	0.19524	0.39438	
transcript_HQ_CoA_transcript22593/f2p0/2079	8.026666667	6.763333333	25.54666667	-0.1194	0.87037	0.9276		1.7674	4.306E-06	6.6156E-05	UP	1.6409	0.011601	0.056097	
transcript_HQ_CoA_transcript22684/f3p0/2007	25.75	26.92	14.63333333	0.2218	0.39819	0.57276		-1.0301	0.00025704	0.0021004	DOWN	-0.82218	0.0075955	0.041244	
transcript_HQ_CoA_transcript22748/f4p0/2008	76.31333333	105.2133333	51.94333333	0.62564	0.0090116	0.032361		-1.1764	2.1867E-08	6.2919E-07	DOWN	-0.56357	0.0013698	0.011491	
transcript_HQ_CoA_transcript22826/f4p0/1997	2.526666667	4.853333333	1.966666667	1.1239	0.17996	0.3295		-1.4603	0.0044882	0.021563	DOWN	-0.35684	0.65546	0.81165	
transcript_HQ_CoA_transcript22845/f2p0/2018	12.42	21.73	9.713333333	0.94532	0.065284	0.15438		-1.2881	0.0023718	0.01276	DOWN	-0.35519	0.37946	0.59575	
transcript_HQ_CoA_transcript22896/f2p0/1990	46.44666667	22.01333333	52.27333333	-0.91547	0.034518	0.093582		1.0847	0.001454	0.0086273	UP	0.16014	0.66324	0.81627	
transcript_HQ_CoA_transcript22938/f2p0/2000	40.04666667	18.68333333	55.81666667	-0.92921	0.01176	0.040157		1.4035	0.00010872	0.001032	UP	0.46302	0.11386	0.27936	
transcript_HQ_CoA_transcript22953/f2p0/1496	18.35333333	23.36	11.11666667	0.51066	0.1136	0.23389		-1.2208	1.7297E-06	3.0425E-05	DOWN	-0.72609	0.042444	0.14297	
transcript_HQ_CoA_transcript23006/f6p0/1952	22.52333333	10.28333333	23.02	-0.99946	0.017568	0.054743		1.0131	0.0012973	0.0078603	UP	0.0044699	0.99088	0.99628	
transcript_HQ_CoA_transcript23061/f4p0/1965	1.673333333	0.816666667	10.14333333	-0.88255	0.37871	0.55407		3.5051	0.0011469	0.0071282	UP	2.6101	0.021319	0.087035	
transcript_HQ_CoA_transcript23158/f2p0/1984	7.47	4.003333333	10.37333333	-0.72341	0.14232	0.27721		1.2104	0.0049251	0.023165	UP	0.47509	0.13949	0.31793	
transcript_HQ_CoA_transcript23204/f4p0/1932	11.65	8.806666667	21.53	-0.26229	0.38672	0.56209		1.1437	0.00029678	0.002362	UP	0.87094	0.0039147	0.025453	
transcript_HQ_CoA_transcript23354/f10p0/1900	35.76	18	40.17333333	-0.8436	0.0041809	0.01713		1.0146	0.0024768	0.013209	UP	0.15993	0.57478	0.75487	
transcript_HQ_CoA_transcript2339/f2p0/4737	0.113333333	0.006666667	0.476666667	-3.9505	0.28889	0.45922		5.9976	0.0019604	0.010928	UP	2.035	0.29811	0.51384	
transcript_HQ_CoA_transcript23394/f11p0/1914	94.86666667	158.1766667	81.96	0.90811	0.0001017	0.00071291		-1.1004	4.1551E-08	1.1283E-06	DOWN	-0.20629	0.34037	0.55831	

transcript_HQ_CoA_transcript23455/f3p0/1937	33.25333333	19.56333333	43.58666667	-0.62849	0.027207	0.077584		1.0183	0.00028037	0.0022539	UP	0.37992	0.11799	0.28588	
transcript_HQ_CoA_transcript23474/f2p0/1959	17.23333333	28.79	13.77	0.90706	0.0022813	0.010346		-1.2176	5.7213E-05	0.00060371	DOWN	-0.32363	0.19292	0.39163	
transcript_HQ_CoA_transcript23539/f2p0/1953	7.256666667	3.673333333	12.33	-0.80361	0.10841	0.22544		1.6095	0.0038504	0.019064	UP	0.78948	0.16953	0.36031	
transcript_HQ_CoA_transcript23563/f6p0/1882	12.70333333	20.21	8.88	0.82313	0.045764	0.11685		-1.3338	0.00080647	0.0053674	DOWN	-0.52319	0.062117	0.18558	
transcript_HQ_CoA_transcript23596/f4p0/1919	15.44666667	28.33333333	13.95	1.0174	0.024841	0.072224		-1.166	0.00085754	0.0056377	DOWN	-0.15819	0.66861	0.81991	
transcript_HQ_CoA_transcript23673/f2p0/1969	5.773333333	4.3	9.786666667	-0.24747	0.51779	0.68315		1.0148	0.009559	0.038643	UP	0.75632	0.018758	0.079443	
transcript_HQ_CoA_transcript23725/f2p0/1943	21.28	9.816666667	25.52666667	-0.95713	0.006653	0.025156		1.2215	0.0016425	0.0094968	UP	0.25215	0.38264	0.5985	
transcript_HQ_CoA_transcript2382/f2p0/4712	4.226666667	6.653333333	3.56	0.8081	0.067377	0.15829		-1.054	0.0096019	0.038762	DOWN	-0.2575	0.38381	0.59941	
transcript_HQ_CoA_transcript23855/f2p0/1936	3.3	1.633333333	5.04	-0.86957	0.11165	0.23081		1.4953	0.002346	0.012636	UP	0.61477	0.0807	0.22078	
transcript_HQ_CoA_transcript23999/f2p0/1919	7.486666667	3.793333333	14.71333333	-0.79114	0.17943	0.32879		1.7678	0.00021903	0.001846	UP	0.9645	0.012646	0.059533	
transcript_HQ_CoA_transcript24058/f3p0/1889	87.23666667	41.60333333	116.3066667	-0.8887	0.0072537	0.026965		1.3165	0.00016905	0.0014911	UP	0.41539	0.094532	0.2471	
transcript_HQ_CoA_transcript24083/f2p0/1894	27.72333333	18.35	53.44	-0.45488	0.10974	0.22766		1.4076	9.4512E-06	0.00012937	UP	0.94212	0.00010832	0.0015236	
transcript_HQ_CoA_transcript24108/f2p0/1916	6.403333333	10.12666667	4.1	0.85589	0.10585	0.22173		-1.4813	0.0047614	0.022535	DOWN	-0.64058	0.028735	0.10828	
transcript_HQ_CoA_transcript24123/f3p0/1903	42.40666667	25.28333333	63.80333333	-0.58744	0.011065	0.038276		1.2023	0.0029793	0.015361	UP	0.60232	0.1215	0.29111	
transcript_HQ_CoA_transcript24271/f11p0/1880	2.196666667	1.13	3.923333333	-0.83982	0.31371	0.48603		1.669	0.001445	0.0085851	UP	0.82144	0.20514	0.40768	
transcript_HQ_CoA_transcript24311/f2p0/1888	4.53	2.59	9.756666667	-0.69593	0.51889	0.68419		1.7699	7.15E-07	1.4323E-05	UP	1.0666	0.27201	0.48469	
transcript_HQ_CoA_transcript24415/f2p0/1873	11.63666667	6.46	15.13	-0.68242	0.021076	0.063351		1.0749	5.0714E-05	0.00054775	UP	0.37906	0.12343	0.29449	
transcript_HQ_CoA_transcript24487/f3p0/1879	33.22333333	26.51666667	66.84	-0.15393	0.72485	0.83509		1.1786	1.2586E-05	0.0001649	UP	1.0095	0.02291	0.091278	
transcript_HQ_CoA_transcript24541/f2p0/1877	22.70333333	10.54	25.44666667	-0.95002	9.7649E-06	9.1597E-05		1.116	0.00016897	0.0014911	UP	0.15315	0.60562	0.77709	
transcript_HQ_CoA_transcript24662/f5p0/1855	24.7	38.25	19.95666667	0.78412	0.013101	0.043716		-1.0882	0.00008519	0.0008454	DOWN	-0.3155	0.24642	0.45577	
transcript_HQ_CoA_transcript24771/f2p0/1880	12.26	18.99666667	9.186666667	0.77911	0.001666	0.0079585		-1.1793	7.2081E-05	0.00073114	DOWN	-0.412	0.17278	0.365	
transcript_HQ_CoA_transcript24772/f2p0/1851	12.71	8.886666667	21.09666667	-0.35608	0.28345	0.45288		1.0992	5.8599E-05	0.0006141	UP	0.72811	0.021788	0.088313	
transcript_HQ_CoA_transcript2481/f2p0/4705	1.403333333	0	1.623333333	-8.5262	0.029132	0.081749		8.7039	6.4758E-12	4.01E-10	UP	0.17372	0.89217	0.9517	
transcript_HQ_CoA_transcript24811/f2p0/1849	95.50666667	46.10666667	103.3433333	-0.89825	0.011882	0.040491		1.0127	0.0030543	0.015687	UP	0.10191	0.77998	0.89267	
transcript_HQ_CoA_transcript24871/f2p0/1710	26.73666667	42.41	21.05	0.83275	0.021229	0.063676		-1.1651	7.7127E-05	0.00077461	DOWN	-0.34548	0.25635	0.46684	
transcript_HQ_CoA_transcript24874/f2p0/1839	1.273333333	3.73	0.766666667	1.6908	0.016723	0.05274		-2.4314	0.00074492	0.0050327	DOWN	-0.75255	0.25685	0.46734	
transcript_HQ_CoA_transcript24900/f2p0/1799	9.833333333	17.98666667	9.473333333	1.0185	0.05388	0.13275		-1.0518	0.00091209	0.0059283	DOWN	-0.046422	0.92521	0.96671	
transcript_HQ_CoA_transcript24981/f2p0/1843	4.323333333	6.496666667	1.666666667	0.74972	0.56691	0.7236		-2.0646	0.0076927	0.032753	DOWN	-1.3276	0.3168	0.53352	
transcript_HQ_CoA_transcript24986/f4p0/1817	10.13	4.813333333	13.25333333	-0.91075	0.031545	0.087102		1.311	0.00005138	0.00055224	UP	0.3857	0.31139	0.52815	
transcript_HQ_CoA_transcript25028/f5p0/1847	214.0533333	105.08	262.73	-0.88272	0.0046588	0.018731		1.1684	6.3984E-07	1.3002E-05	UP	0.27609	0.4008	0.61636	
transcript_HQ_CoA_transcript25060/f2p0/1834	2.343333333	4.206666667	1.216666667	0.98026	0.19296	0.34521		-1.9243	0.0067545	0.029601	DOWN	-0.95624	0.12679	0.29917	
transcript_HQ_CoA_transcript2509/f22p0/4564	9.673333333	8.216666667	20.83666667	-0.086994	0.88127	0.93399		1.1905	0.00045906	0.003401	UP	1.0944	0.03113	0.11474	
transcript_HQ_CoA_transcript25090/f2p0/1826	5.933333333	6.44	3.343333333	0.29081	0.51741	0.68288		-1.0921	0.00097309	0.0062465	DOWN	-0.81791	0.084071	0.22753	
transcript_HQ_CoA_transcript25125/f3p0/1853	11.75333333	18.74666667	7.943333333	0.82779	0.058422	0.14149		-1.3863	0.0068907	0.029993	DOWN	-0.56863	0.1475	0.32977	
transcript_HQ_CoA_transcript25168/f2p0/1841	9.586666667	3.236666667	13.86	-1.3776	0.16654	0.31097		1.9308	2.0341E-08	5.9055E-07	UP	0.53717	0.55115	0.73895	
transcript_HQ_CoA_transcript25214/f3p0/1818	10.01666667	5.54	13.71333333	-0.70708	0.068982	0.16115		1.1758	0.0019613	0.01093	UP	0.45687	0.13117	0.30535	
transcript_HQ_CoA_transcript25245/f2p0/1817	6.476666667	5.193333333	19.69666667	-0.13594	0.87008	0.9274		1.7813	0.012427	0.047623	UP	1.6321	0.060975	0.18345	
transcript_HQ_CoA_transcript25272/f3p0/1828	11.53666667	5.08	17.68666667	-1.0628	0.097924	0.20902		1.6853	0.0061292	0.027512	UP	0.6105	0.030298	0.11251	
transcript_HQ_CoA_transcript25379/f2p0/1800	45.84333333	79.69	35.83	0.9728	0.0070554	0.026356		-1.3227	0.00036262	0.0027961	DOWN	-0.36358	0.1222	0.29229	
transcript_HQ_CoA_transcript25383/f3p0/1797	10.72333333	5.133333333	23.45	-0.90601	0.16684	0.3113		2.0266	2.4567E-07	5.5728E-06	UP	1.1136	0.038497	0.13355	
transcript_HQ_CoA_transcript25410/f2p0/1827	10.73	3.73	15.15666667	-1.3779	0.047458	0.12018		1.896	7.3105E-08	1.8533E-06	UP	0.50734	0.39857	0.61436	
transcript_HQ_CoA_transcript25460/f3p0/1802	8.376666667	4.573333333	16.59333333	-0.71775	0.12677	0.25401		1.7165	0.00006698	0.00068547	UP	0.98544	0.023549	0.093175	
transcript_HQ_CoA_transcript25461/f2p0/1793	12.27	4.656666667	14.03666667	-1.1964	0.099075	0.21101		1.4375	5.1668E-05	0.00055447	UP	0.22484	0.73511	0.8651	
transcript_HQ_CoA_transcript255/f2p0/7033	3.556666667	1.743333333	5.27	-0.84961	0.07508	0.17214		1.4255	0.0025682	0.013624	UP	0.56135	0.016498	0.072033	
transcript_HQ_CoA_transcript25628/f2p0/1788	7.133333333	1.03	39.67333333	-2.6672	NA	NA	NA	5.1008	1.4963E-27	8.8468E-25	UP	2.4303	NA	NA	NA
transcript_HQ_CoA_transcript25668/f25p0/1684	44.73	35.05	77.80333333	-0.21184	0.46446	0.63614		1.0178	2.3335E-05	0.00027846	UP	0.79604	0.0030778	0.021206	
transcript_HQ_CoA_transcript25795/f11p0/1755	99.47	53.44	121.28	-0.75712	0.01787	0.055458		1.0353	5.2419E-05	0.00056023	UP	0.26831	0.40998	0.62399	
transcript_HQ_CoA_transcript25802/f5p0/1777	3.626666667	1.983333333	5.663333333	-0.69603	0.13386	0.26474		1.3535	0.0014469	0.0085935	UP	0.64386	0.17432	0.36711	
transcript_HQ_CoA_transcript25811/f2p0/1781	7.24	6.493333333	64.72666667	0.0058291	0.99234	0.99637		3.2098	0.01018	0.040626	UP	3.2034	0.01585	0.070138	
transcript_HQ_CoA_transcript25891/f2p0/1799	2.73	1.703333333	4.05	-0.51548	0.34255	0.51782		1.0941	0.0083375	0.034826	UP	0.56335	0.20353	0.40558	
transcript_HQ_CoA_transcript25944/f2p0/1782	8.63	12.42666667	5.843333333	0.68384	0.037594	0.09994		-1.2361	0.00022833	0.0019032	DOWN	-0.56505	0.080819	0.22085	
transcript_HQ_CoA_transcript25953/f2p0/1767	3.74	1.043333333	3.433333333	-1.6863	0.023883	0.070018		1.5577	0.0024917	0.013276	UP	-0.13878	0.83769	0.92508	
transcript_HQ_CoA_transcript25975/f2p0/1755	3.943333333	4.37	1.743333333	0.3167	0.50226	0.66927		-1.4866	6.4123E-05	0.00066066	DOWN	-1.1868	0.02913	0.1095	
transcript_HQ_CoA_transcript2603/f6p0/4598	5.553333333	9.93	5.063333333	1.0018	0.023841	0.069939		-1.1273	0.010344	0.041108	DOWN	-0.13974	0.58845	0.76516	
transcript_HQ_CoA_transcript26032/f3p0/1761	0.996666667	1.533333333	0.173333333	0.76302	0.2148	0.37296		-3.284	3.9328E-05	0.00044061	DOWN	-2.5219	0.011222	0.054898	
transcript_HQ_CoA_transcript26110/f3p0/1755	20.93666667	0.076666667	89.01333333	-7.8087	0.022427	0.066604		10.027	1.1047E-36	1.3499E-33	UP	2.1102	0.27128	0.48394	
transcript_HQ_CoA_transcript26125/f15p0/1708	44.46	65.83	31.72333333	0.71966	0.069802	0.16281		-1.2092	0.00056404	0.0040102	DOWN	-0.50092	0.049232	0.15873	
transcript_HQ_CoA_transcript26161/f4p0/1735	11.94333333	3.636666667	21.95666667	-1.4718	0.13812	0.27111		2.3885	0.0091734	0.037422	UP	0.90174	0.26335	0.47515	
transcript_HQ_CoA_transcript26271/f2p0/1742	5.063333333	2.153333333	5.536666667	-1.1076	0.065539	0.15478		1.2201	0.001816	0.010298	UP	0.1025	0.84685	0.92999	
transcript_HQ_CoA_transcript26281/f7p0/1748	29.77	63.16333333	23.76666667	1.2297	0.021222	0.063665		-1.5528	0.0033763	0.017085	DOWN	-0.33532	0.11437	0.28016	
transcript_HQ_CoA_transcript26324/f2p0/1730	1.176666667	0.826666667	0.15	-0.34306	0.72534	0.83542		-2.5829	0.0062822	0.028016	DOWN	-2.9456	0.013727	0.063054	
transcript_HQ_CoA_transcript2637/f4p0/4570	6.426666667	1.85	14.36666667	-1.5883	0.075494	0.17267		2.7613	0.00018427	0.0016076	UP	1.1559	0.041243	0.14019	
transcript_HQ_CoA_transcript2642/f10p0/4552	18.79	26.65333333	13.79666667	0.67555	0.018991	0.058235		-1.1159	0.00010422	0.00099667	DOWN	-0.45237	0.0071942	0.039607	
transcript_HQ_CoA_transcript26494/f2p0/1718	6.153333333	2.51	14.22333333	-1.13	0.061177	0.14675		2.3484	3.5607E-12	2.3144E-10	UP	1.2029	0.011125	0.054502	
transcript_HQ_CoA_transcript26590/f3p0/1706	4.253333333	2.676666667	7.923333333	-0.52997	0.23183	0.39364		1.4269	0.00073638	0.004986	UP	0.88346	0.021783	0.088313	
transcript_HQ_CoA_transcript26603/f2p0/1706	9.78	3.5	15.74666667	-1.3055	0.016902	0.053131		2.0112	2.7733E-08	7.8565E-07	UP	0.69105	0.15878	0.34589	
transcript_HQ_CoA_transcript26688/f3p0/1702	18.26333333	11.14333333	32.96	-0.51264	0.36945	0.54511		1.3777	0.010816	0.042681	UP	0.85087	0.0047756	0.029544	
transcript_HQ_CoA_transcript268/f2p0/6994	1.31	0.4	2.573333333	-1.5707	0.092447	0.20037		2.5139	9.8972E-10	3.8762E-08	UP	0.93351	0.23526	0.44404	
transcript_HQ_CoA_transcript26812/f3p0/1676	28.81666667	14.23	31.68666667	-0.86786	0.0090894	0.032557		1.0075	0.0011138	0.0069747	UP	0.12902	0.59493	0.76927	
transcript_HQ_CoA_transcript26864/f2p0/1696	143.9766667	50.6	137.8033333	-1.3469	0.036895	0.098528		1.2797	0.0027052	0.014183	UP	-0.07534	0.88807	0.94992	
transcript_HQ_CoA_transcript26878/f2p0/1691	46.36	69.99333333	38.3	0.76055	0.012788	0.042863		-1.0304	0.00027396	0.002215	DOWN	-0.28231	0.11516	0.28176	
transcript_HQ_CoA_transcript26918/f2p0/1710	2.216666667	5.23	1.333333333	1.4387	0.094673	0.20377		-2.1837	0.011669	0.045351	DOWN	-0.75393	0.23648	0.44511	
transcript_HQ_CoA_transcript26969/f2p0/1674	9.763333333	19.47666667	7.236666667	1.138	0.033688	0.091823		-1.5441	0.010571	0.041875	DOWN	-0.42221	0.41016	0.62415	
transcript_HQ_CoA_transcript26978/f3p0/1668	10.94333333	18.85666667	5.966666667	0.9449	0.0072983	0.027103		-1.8156	1.1084E-07	2.7125E-06	DOWN	-0.88065	0.0097541	0.049401	
transcript_HQ_CoA_transcript27/f2p0/8816	3.576666667	2.463333333	5.996666667	-0.39315	0.21595	0.37429		1.1341	0.0001408	0.0012744	UP	0.72998	0.027216	0.10375	
transcript_HQ_CoA_transcript27094/f2p0/1670	16.74666667	25.9	10.89333333	0.80154	0.040217	0.10558		-1.405	4.2756E-06	6.5744E-05	DOWN	-0.61659	0.091434	0.2411	
transcript_HQ_CoA_transcript2713/f2p0/4590	1.486666667	2.553333333	1.133333333	0.95285	0.010362	0.036269		-1.3358	0.00021356	0.0018164	DOWN	-0.39527	0.24204	0.45089	
transcript_HQ_CoA_transcript27218/f2p0/1660	56.14	21.17333333	98.01	-1.2074	0.037121	0.099001		2.013	0.00079517	0.0053173	UP	0.79203	0.022698	0.090727	
transcript_HQ_CoA_transcript27231/f2p0/1657	2.816666667	4.356666667	1.703333333	0.77015	0.054402	0.13364		-1.5058	0.0018796	0.010558	DOWN	-0.74387	0.20577	0.40826	

transcript_HQ_CoA_transcript27248/f2p0/1674	170.41	95.72	268.6833333	-0.68805	0.028707	0.080867		1.3465	0.0017257	0.0098939	UP	0.64685	0.090295	0.23919
transcript_HQ_CoA_transcript27304/f2p0/1658	4.636666667	6.946666667	1.73	0.71092	0.25888	0.4258		-2.14	0.0091166	0.037307	DOWN	-1.445	0.03409	0.12232
transcript_HQ_CoA_transcript27343/f5p0/1643	59.65	34.18333333	79.09	-0.66752	0.061445	0.14723		1.0793	0.00162	0.0093875	UP	0.40194	0.17008	0.36091
transcript_HQ_CoA_transcript27365/f2p0/1646	6.14	3.273333333	8.443333333	-0.75262	0.043952	0.11341		1.2099	9.6753E-05	0.00093879	UP	0.44467	0.1438	0.32402
transcript_HQ_CoA_transcript27378/f2p0/1645	30.44	14.42	39.25333333	-0.91577	0.057528	0.13976		1.2938	0.0072247	0.031107	UP	0.36583	0.28215	0.49625
transcript_HQ_CoA_transcript27379/f2p0/1649	18.33666667	10.53	27.21333333	-0.61989	0.19707	0.35047		1.1969	0.0090752	0.037188	UP	0.56334	0.0085389	0.044961
transcript_HQ_CoA_transcript27507/f2p0/1636	5.733333333	10.96	2.913333333	1.0798	0.027285	0.077734		-2.0563	1.4389E-11	8.165E-10	DOWN	-0.99106	0.072059	0.20436
transcript_HQ_CoA_transcript2751/f4p0/4543	11.60333333	19.59333333	6.32	0.92208	0.0000122	0.00011158		-1.7922	2.534E-18	4.7882E-16	DOWN	-0.88347	1.1136E-05	0.00023018
transcript_HQ_CoA_transcript27540/f2p0/1627	12.83333333	4.886666667	34.06	-1.2296	0.12316	0.24853		2.62	1.0179E-11	5.9419E-10	UP	1.3828	0.039576	0.13616
transcript_HQ_CoA_transcript27587/f4p0/1620	22.98666667	11.18666667	34.55333333	-0.86945	0.064154	0.15234		1.4694	2.031E-11	1.128E-09	UP	0.58759	0.16221	0.35062
transcript_HQ_CoA_transcript27642/f2p0/1619	18.24333333	24.66666667	10.40333333	0.62298	0.38671	0.56209		-1.3769	0.00035199	0.0027188	DOWN	-0.77005	0.30511	0.52178
transcript_HQ_CoA_transcript27651/f2p0/1641	24.42	10.63	45.43666667	-1.0766	0.16873	0.31393		1.9875	0.00019798	0.0017084	UP	0.89977	0.13117	0.30535
transcript_HQ_CoA_transcript27705/f3p0/1627	33.04	22.84333333	58.73666667	-0.38533	0.23592	0.39841		1.2203	0.0034555	0.017429	UP	0.82316	0.030142	0.11215
transcript_HQ_CoA_transcript27871/f2p0/1625	20.16	12.03333333	28.44	-0.58896	0.031916	0.087934		1.087	8.7947E-09	2.7841E-07	UP	0.48693	0.028774	0.10838
transcript_HQ_CoA_transcript27947/f3p0/1610	10.19666667	17	6.793333333	0.90173	0.0070568	0.026356		-1.4605	0.00003795	0.00042832	DOWN	-0.56963	0.2054	0.40795
transcript_HQ_CoA_transcript28029/f2p0/1472	21.84666667	18.71666667	130.4066667	-0.066336	0.90726	0.94883		2.7093	0.0013634	0.0081866	UP	2.6272	0.029667	0.11088
transcript_HQ_CoA_transcript28061/f5p0/1566	2.156666667	40.41333333	2.776666667	4.3477	0.017071	0.053576		-4.0033	0.0099964	0.040005	DOWN	0.33553	0.90368	0.95713
transcript_HQ_CoA_transcript28078/f2p0/1582	13.90333333	20.94333333	8.126666667	0.74202	0.11329	0.2335		-1.5072	5.464E-09	1.811E-07	DOWN	-0.77433	0.11913	0.28731
transcript_HQ_CoA_transcript28121/f2p0/1565	0.626666667	5.383333333	1.033333333	3.3027	0.034612	0.093772		-2.5133	0.0013482	0.0081126	DOWN	0.77816	0.68305	0.83061
transcript_HQ_CoA_transcript28181/f3p0/1554	77.39666667	55.85666667	128.72	-0.34018	0.36138	0.53683		1.0764	0.0042787	0.020791	UP	0.72611	0.0031298	0.021447
transcript_HQ_CoA_transcript28187/f2p0/1545	1.51	4.05	0.726666667	1.5721	0.095002	0.20428		-2.5925	0.0016336	0.0094512	DOWN	-1.0355	0.24381	0.453
transcript_HQ_CoA_transcript28219/f4p0/1526	7.363333333	5.096666667	12.87	-0.38351	0.30231	0.47351		1.1905	0.00045383	0.0033663	UP	0.79726	0.0094295	0.048198
transcript_HQ_CoA_transcript28237/f2p0/1547	6.833333333	41.02333333	0.406666667	2.776	0.099506	0.2116		-6.7675	0.00090588	0.0059063	DOWN	-4.0038	0.26642	0.47816
transcript_HQ_CoA_transcript2828/f8p0/4480	13.53666667	26.19333333	13.26333333	1.1079	0.016849	0.053046		-1.1325	0.013057	0.049547	DOWN	-0.037264	0.83721	0.92461
transcript_HQ_CoA_transcript28302/f3p0/1532	6.513333333	9.266666667	4.983333333	0.66716	0.10244	0.21625		-1.0489	0.0092839	0.037772	DOWN	-0.39371	0.2658	0.47781
transcript_HQ_CoA_transcript28364/f2p0/1540	16.55666667	7.2	21.27666667	-1.0711	0.016097	0.051136		1.4448	0.0013209	0.0079822	UP	0.36359	0.21529	0.42037
transcript_HQ_CoA_transcript28382/f3p0/1548	3.866666667	0.35	9.733333333	-3.3145	NA	NA	NA	4.6304	1.7643E-14	1.8911E-12	UP	1.3052	0.22583	0.43286
transcript_HQ_CoA_transcript28440/f2p0/1532	21.07666667	10.84	24.34	-0.80315	0.0034666	0.014701		1.0058	0.00011564	0.0010814	UP	0.19041	0.474	0.67945
transcript_HQ_CoA_transcript28610/f3p0/1518	44.00666667	19.08666667	56.75	-1.0531	0.086148	0.19019		1.436	0.00073162	0.0049666	UP	0.37012	0.43609	0.64755
transcript_HQ_CoA_transcript28680/f2p0/1492	16.48	29.22333333	7.776666667	0.98081	0.063111	0.15037		-2.0378	2.8234E-05	0.00032983	DOWN	-1.0703	0.010803	0.053205
transcript_HQ_CoA_transcript28691/f2p0/1493	24.77666667	41.49333333	19.57	0.90961	0.0023527	0.010625		-1.2407	3.6214E-05	0.00041074	DOWN	-0.34368	0.18982	0.38732
transcript_HQ_CoA_transcript28697/f13p0/1362	377.05	620.1133333	259.9966667	0.87366	2.7904E-09	5.3314E-08		-1.3963	8.7146E-17	1.2577E-14	DOWN	-0.53582	0.0032227	0.021933
transcript_HQ_CoA_transcript28707/f2p0/1485	13.85666667	24.5	11.70666667	0.97736	0.041418	0.10811		-1.2173	0.012189	0.046897	DOWN	-0.25206	0.38115	0.59709
transcript_HQ_CoA_transcript28732/f2p0/1481	188.4833333	141.2966667	55.64666667	-0.23853	0.72134	0.83301		-1.4736	0.0002011	0.0017313	DOWN	-1.727	0.062824	0.18668
transcript_HQ_CoA_transcript28773/f2p0/1493	38.03	44.5	23.48333333	0.38659	0.38039	0.55572		-1.0854	0.0066396	0.029163	DOWN	-0.71015	0.0086748	0.045428
transcript_HQ_CoA_transcript28803/f5p0/1461	55.37666667	31.34666667	90.40333333	-0.68035	0.060952	0.14635		1.3892	9.501E-07	1.8254E-05	UP	0.69837	0.022562	0.090291
transcript_HQ_CoA_transcript28814/f3p0/1480	4.89	2.813333333	7.26	-0.6334	0.24779	0.41289		1.2021	0.0031306	0.016028	UP	0.55953	0.16673	0.35668
transcript_HQ_CoA_transcript28854/f2p0/1447	19.88333333	11.22666667	27.42666667	-0.67431	0.01355	0.044887		1.1387	9.8955E-05	0.00095813	UP	0.45322	0.15127	0.33492
transcript_HQ_CoA_transcript28918/f2p0/1438	31.90333333	48.59666667	22.40333333	0.78253	0.024811	0.072161		-1.2836	0.00017452	0.0015342	DOWN	-0.5131	0.015507	0.069099
transcript_HQ_CoA_transcript28919/f2p0/1489	12.65333333	3.323333333	25.82	-1.7394	0.025165	0.072969		2.7751	1.7799E-06	3.1219E-05	UP	1.0236	0.080049	0.21963
transcript_HQ_CoA_transcript28940/f7p0/1431	33.40666667	58.1	25.57333333	0.95169	0.0040277	0.016644		-1.3168	0.00060349	0.0042381	DOWN	-0.37942	0.24304	0.45217
transcript_HQ_CoA_transcript29009/f2p0/1439	9.866666667	5.473333333	17.71666667	-0.68657	0.055997	0.13661		1.5302	8.6572E-07	1.6917E-05	UP	0.83238	0.0020205	0.015401
transcript_HQ_CoA_transcript29014/f4p0/1443	62.92	45.17666667	104.15	-0.30342	0.36304	0.53847		1.0383	0.0011955	0.0073672	UP	0.72217	0.0016521	0.013161
transcript_HQ_CoA_transcript2904/f2p0/4474	0.826666667	1.84	0.69	1.265	0.26671	0.43466		-1.544	0.01049	0.041609	DOWN	-0.28652	0.8028	0.90634
transcript_HQ_CoA_transcript29122/f2p0/1441	5.76	4.673333333	10.69666667	-0.12457	0.7495	0.85055		1.0472	3.8305E-05	0.00043152	UP	0.90965	0.0068067	0.038149
transcript_HQ_CoA_transcript29126/f2p0/1407	4.156666667	7.353333333	3.98	0.97581	0.0058703	0.022627		-1.0382	0.00074645	0.0050412	DOWN	-0.075322	0.8455	0.92935
transcript_HQ_CoA_transcript29153/f3p0/1427	65.28333333	47.29333333	126.37	-0.31837	0.25928	0.42619		1.2793	0.00060056	0.0042256	UP	0.94988	0.0051056	0.031007
transcript_HQ_CoA_transcript29173/f2p0/1401	39.03333333	24.48333333	64.26666667	-0.52546	0.023356	0.06889		1.2642	0.0011466	0.0071282	UP	0.72687	0.05492	0.17097
transcript_HQ_CoA_transcript29189/f2p0/1400	39.3	19.21666667	46.89	-0.87257	0.0083283	0.0303		1.1175	0.0042973	0.020859	UP	0.23513	0.54315	0.73322
transcript_HQ_CoA_transcript29200/f4p0/1318	37.95	66.69333333	35.78	0.97952	8.2242E-05	0.00059382		-1.0406	0.00008184	0.00081613	DOWN	-0.074823	0.80872	0.90979
transcript_HQ_CoA_transcript29219/f3p0/1392	48.77	22.25666667	68.29333333	-0.99767	0.0023194	0.010506		1.4794	0.00026276	0.0021395	UP	0.47147	0.13086	0.30481
transcript_HQ_CoA_transcript29231/f2p0/1381	50.54	27.95666667	63.89	-0.7178	0.081541	0.18275		1.0536	1.1084E-07	2.7125E-06	UP	0.32674	0.37931	0.59567
transcript_HQ_CoA_transcript29282/f7p0/1370	107.25	96.79333333	40.10666667	0.014498	0.98377	0.99225		-1.3935	0.0029632	0.015308	DOWN	-1.393	0.044453	0.14779
transcript_HQ_CoA_transcript29284/f2p0/1383	25.05	16.90666667	48.31	-0.41611	0.062942	0.15007		1.387	1.8349E-05	0.00022648	UP	0.95871	0.0025175	0.018157
transcript_HQ_CoA_transcript29369/f3p0/1315	19.54333333	23.17	11.00333333	0.41368	0.16714	0.31173		-1.234	2.1517E-05	0.00026083	DOWN	-0.83321	0.0011007	0.0096622
transcript_HQ_CoA_transcript29415/f2p0/1350	74.40333333	33.52666667	78.79	-0.99082	4.2154E-05	0.00033132		1.0811	2.8835E-08	8.0962E-07	UP	0.07757	0.75153	0.87478
transcript_HQ_CoA_transcript29436/f8p0/1326	384.01	185.5766667	413.9666667	-0.89293	0.0010095	0.005192		1.01	0.0043132	0.020903	UP	0.1085	0.75169	0.87491
transcript_HQ_CoA_transcript2946/f2p0/4458	3.723333333	6.573333333	1.71	0.9719	0.03175	0.087549		-2.0743	2.4767E-05	0.00029399	DOWN	-1.1173	0.053523	0.16813
transcript_HQ_CoA_transcript29501/f3p0/1320	71.63	59.53666667	137.0666667	-0.11707	0.65145	0.78717		1.0502	6.4749E-05	0.00066523	UP	0.92255	0.001405	0.011728
transcript_HQ_CoA_transcript29760/f2p0/1247	32.66	21.18333333	184.2066667	-0.48469	0.21326	0.37111		3.0152	0.00008527	0.00084573	UP	2.5195	0.01271	0.059745
transcript_HQ_CoA_transcript29785/f2p0/1227	62.52666667	41.19	112.4433333	-0.4571	0.099307	0.21131		1.3109	1.0658E-08	3.3335E-07	UP	0.84327	0.0016965	0.013463
transcript_HQ_CoA_transcript29827/f3p0/1250	367.56	179.9466667	553.9733333	-0.86877	0.00044278	0.0025415		1.4642	3.2641E-05	0.00037652	UP	0.58504	0.084734	0.22875
transcript_HQ_CoA_transcript29847/f2p0/1217	257.0733333	118.2	290.3033333	-0.9855	0.00025685	0.0015923		1.1618	0.00060543	0.0042485	UP	0.16659	0.55823	0.74374
transcript_HQ_CoA_transcript29933/f4p0/1159	137.22	112.94	53.46	-0.13214	0.81553	0.89231		-1.2237	0.0018072	0.010265	DOWN	-1.3638	0.012824	0.060141
transcript_HQ_CoA_transcript29944/f2p0/1152	106.3433333	189.4966667	99.02666667	0.99961	6.2599E-05	0.00046865		-1.0892	2.3321E-12	1.6377E-10	DOWN	-0.10285	0.65372	0.81026
transcript_HQ_CoA_transcript29991/f5p0/1122	421.1133333	195.1666667	553.1766667	-0.95844	0.00040869	0.0023803		1.3487	3.262E-08	9.0866E-07	UP	0.38056	0.18061	0.37579
transcript_HQ_CoA_transcript30010/f4p0/1080	269.6233333	466.5633333	256.8066667	0.94961	0.0013418	0.006623		-1.0116	0.00023324	0.001937	DOWN	-0.073729	0.70696	0.84618
transcript_HQ_CoA_transcript30118/f6p0/1003	418.3966667	216.9666667	716.0466667	-0.78368	0.053971	0.13293		1.5678	5.1362E-05	0.00055224	UP	0.76993	0.056888	0.1749
transcript_HQ_CoA_transcript30174/f2p0/906	1167.5	657.3466667	1871.17	-0.6771	0.012484	0.04211		1.3558	1.4604E-05	0.0001864	UP	0.6678	0.011376	0.055363
transcript_HQ_CoA_transcript30212/f2p0/818	58.44333333	41.69	105.31	-0.31613	0.21066	0.3683		1.1779	1.8809E-05	0.00023138	UP	0.85	0.00029605	0.0034112
transcript_HQ_CoA_transcript30217/f3p0/803	2171.256667	985.8966667	2462.906667	-0.99783	1.6153E-06	1.8206E-05		1.1818	0.00020119	0.0017313	UP	0.17389	0.55995	0.74476
transcript_HQ_CoA_transcript3023/f2p0/4466	11.56333333	11.98	6.223333333	0.19544	0.62483	0.76815		-1.0817	0.0065603	0.028898	DOWN	-0.89797	3.3955E-06	8.7412E-05
transcript_HQ_CoA_transcript30232/f2p0/777	1878.796667	993.6	2832.926667	-0.77395	7.5035E-06	7.2968E-05		1.3741	0.00001383	0.00017852	UP	0.58984	0.049978	0.16001
transcript_HQ_CoA_transcript3024/f3p0/4433	5.543333333	6.046666667	2.5	0.27931	0.55437	0.71401		-1.4251	0.00019274	0.0016711	DOWN	-1.162	0.015764	0.069888
transcript_HQ_CoA_transcript30242/f2p0/751	3020.55	1378.346667	3489.683333	-0.98365	3.0958E-07	3.9914E-06		1.1973	1.3891E-05	0.00017893	UP	0.20373	0.46053	0.66885
transcript_HQ_CoA_transcript30256/f2p0/702	2233.523333	1369.74	3347.506667	-0.54921	0.001152	0.005789		1.139	1.2544E-05	0.0001649	UP	0.57882	0.026799	0.10265
transcript_HQ_CoA_transcript30269/f3p0/622	3002.263333	1465.87	3510.226667	-0.89468	0.00064758	0.0035225		1.131	0.0010195	0.0064864	UP	0.22547	0.51563	0.71259
transcript_HQ_CoA_transcript3032/f2p0/4430	4.786666667	5.8	3.02	0.43477	0.3089	0.48087		-1.0895	0.00053615	0.0038583	DOWN	-0.66679	0.10441	0.26451
transcript_HQ_CoA_transcript3127/f2p0/4376	3.06	4.35	1.666666667	0.66498	0.022257	0.066175		-1.5436	6.2524E-07	1.2748E-05	DOWN	-0.89212	0.0030934	0.02125

transcript_HQ_CoA_transcript32/f3p0/8647	73.76666667	11.14666667	51.85666667	-2.6061	0.030879	0.085599		2.1083	0.00076914	0.0051677	UP	-0.50823	0.62513	0.7905	
transcript_HQ_CoA_transcript3225/f3p0/4390	13.94	9.56	22.12	-0.38684	0.032772	0.08992		1.0597	2.5918E-05	0.0003053	UP	0.66101	0.010448	0.051944	
transcript_HQ_CoA_transcript3239/f2p0/4344	8.44	5.016666667	16.81666667	-0.60003	0.024728	0.071989		1.5953	1.2528E-10	6.0111E-09	UP	0.98351	3.6807E-05	0.0006244	
transcript_HQ_CoA_transcript3262/f2p0/4419	2.06	5.4	0.843333333	1.5473	0.37378	0.54909		-2.8035	7.1364E-06	0.00010156	DOWN	-1.2688	0.32418	0.54128	
transcript_HQ_CoA_transcript3303/f2p0/4325	24.52666667	13.57	33.84333333	-0.70655	0.03461	0.093772		1.1673	0.0046802	0.022264	UP	0.44941	0.26623	0.47815	
transcript_HQ_CoA_transcript3448/f2p0/4286	2.123333333	1.13	2.683333333	-0.74685	0.10445	0.2195		1.0793	0.0015098	0.0088625	UP	0.32132	0.37136	0.58858	
transcript_HQ_CoA_transcript3476/f5p0/4259	7.216666667	9.106666667	4.053333333	0.48892	0.0092157	0.032939		-1.3199	8.1722E-14	7.6814E-12	DOWN	-0.84377	9.7865E-05	0.0013903	
transcript_HQ_CoA_transcript349/f2p0/6807	0.186666667	0.553333333	0.063333333	1.7424	0.039807	0.10465		-3.3433	5.8539E-08	1.5461E-06	DOWN	-1.618	0.1491	0.33209	
transcript_HQ_CoA_transcript3517/f3p0/4240	20.04	11.7	26.78333333	-0.60708	0.072245	0.16714		1.0395	0.00010861	0.0010315	UP	0.41884	0.20115	0.40256	
transcript_HQ_CoA_transcript3579/f2p0/4244	14.2	7.14	18.11333333	-0.83947	0.00064018	0.0034864		1.1961	5.1727E-05	0.00055454	UP	0.34449	0.20908	0.41244	
transcript_HQ_CoA_transcript3633/f3p0/4238	3	6.946666667	1.223333333	1.3854	0.04101	0.10725		-2.668	2.7902E-09	9.8159E-08	DOWN	-1.2995	0.098881	0.25502	
transcript_HQ_CoA_transcript3702/f2p0/4208	3.516666667	2.94	6.596666667	-0.098714	0.75611	0.85481		1.0099	0.00063416	0.0044212	UP	0.8989	0.00088483	0.0081205	
transcript_HQ_CoA_transcript3714/f3p0/4186	16.78666667	12.28	33.75	-0.30548	0.32113	0.49437		1.3097	0.0012757	0.0077504	UP	0.99317	0.0086045	0.045228	
transcript_HQ_CoA_transcript3718/f2p0/4196	12.59	9.726666667	25.00666667	-0.19369	0.5939	0.74531		1.1868	0.0091732	0.037422	UP	0.98017	0.011432	0.055529	
transcript_HQ_CoA_transcript3788/f2p0/4202	1.046666667	1.15	2.92	0.30461	0.65936	0.79319		1.2173	0.0059695	0.026969	UP	1.5051	0.017517	0.075332	
transcript_HQ_CoA_transcript3801/f3p0/4206	9.343333333	4.65	10.51	-0.8482	0.0033227	0.014173		1.0151	0.0001669	0.0014756	UP	0.15534	0.61492	0.78262	
transcript_HQ_CoA_transcript3918/f2p0/4143	10.00333333	5.02	14.17666667	-0.83583	0.020714	0.062424		1.3402	2.9396E-07	6.5309E-06	UP	0.49406	0.10531	0.26597	
transcript_HQ_CoA_transcript3942/f2p0/4130	6.913333333	3.72	11.36333333	-0.744	0.023481	0.069151		1.469	1.0588E-06	1.9925E-05	UP	0.71348	0.036293	0.12789	
transcript_HQ_CoA_transcript3971/f3p0/4115	1.01	1.753333333	0.553333333	0.93559	0.033465	0.091288		-1.7973	0.007789	0.033047	DOWN	-0.8722	0.24044	0.44919	
transcript_HQ_CoA_transcript4018/f2p0/4138	7.063333333	3.776666667	10.17	-0.74584	0.013151	0.043857		1.2734	0.00061192	0.0042825	UP	0.51481	0.14581	0.32726	
transcript_HQ_CoA_transcript4147/f3p0/4096	0.746666667	0.303333333	1.583333333	-1.0927	0.18735	0.33845		2.1793	0.0009263	0.0060036	UP	1.0733	0.015508	0.069099	
transcript_HQ_CoA_transcript4155/f6p0/4043	10.01333333	13.18333333	5.543333333	0.54197	0.14717	0.28397		-1.3963	0.00019734	0.0017054	DOWN	-0.86488	0.0010735	0.0094842	
transcript_HQ_CoA_transcript4244/f2p0/4068	3.113333333	4.26	2.13	0.59176	0.15605	0.2969		-1.126	0.012765	0.048629	DOWN	-0.54658	0.11228	0.27692	
transcript_HQ_CoA_transcript4248/f5p0/4048	6.7	4.263333333	11.3	-0.47283	0.31626	0.48902		1.2504	5.1555E-05	0.00055357	UP	0.76321	0.072574	0.2053	
transcript_HQ_CoA_transcript429/f2p0/6591	3.37	5.2	2.806666667	0.77589	0.00079421	0.0042099		-1.0271	1.0191E-05	0.00013825	DOWN	-0.26353	0.22752	0.43472	
transcript_HQ_CoA_transcript4298/f2p0/4029	0.51	1.17	0.14	1.3299	0.21385	0.37177		-3.2682	2.3938E-09	8.5695E-08	DOWN	-1.9444	0.1188	0.28691	
transcript_HQ_CoA_transcript4355/f2p0/4034	2.366666667	5.17	1.97	1.272	0.01834	0.056656		-1.5413	0.0069894	0.030322	DOWN	-0.28022	0.48109	0.68527	
transcript_HQ_CoA_transcript4377/f2p0/4020	12.77	4.883333333	15.6	-1.2516	0.018873	0.057941		1.5273	8.5334E-06	0.00011831	UP	0.26731	0.57036	0.75219	
transcript_HQ_CoA_transcript4400/f3p0/4050	3.273333333	1.26	7.263333333	-1.213	0.075621	0.1729		2.3489	1.5113E-06	2.7131E-05	UP	1.128	0.043138	0.14449	
transcript_HQ_CoA_transcript4418/f2p0/4015	4.183333333	4.44	2.196666667	0.24714	0.34731	0.52255		-1.1635	6.6291E-07	1.3367E-05	DOWN	-0.92862	0.0011716	0.010134	
transcript_HQ_CoA_transcript4467/f3p0/4146	0.626666667	0.066666667	1.973333333	-2.9066	0.054829	0.13435		4.592	5.1646E-07	1.0881E-05	UP	1.6638	0.074022	0.20768	
transcript_HQ_CoA_transcript4475/f2p0/4012	17.61666667	31.11333333	13.21	0.97871	1.6926E-06	1.8972E-05		-1.3716	7.4892E-05	0.00075548	DOWN	-0.40668	0.22518	0.43229	
transcript_HQ_CoA_transcript4537/f2p0/3988	0.563333333	1.063333333	0.276666667	1.0529	0.074252	0.17082		-2.0698	0.00097258	0.0062461	DOWN	-1.0313	0.118	0.28588	
transcript_HQ_CoA_transcript4547/f5p0/4000	5.663333333	6.993333333	3.543333333	0.46567	0.067952	0.15923		-1.1301	1.1411E-05	0.00015167	DOWN	-0.67715	0.02447	0.095849	
transcript_HQ_CoA_transcript4563/f4p0/3976	9.196666667	5.52	12.89	-0.57242	0.022448	0.066634		1.0664	6.5286E-07	1.3208E-05	UP	0.48002	0.054256	0.16974	
transcript_HQ_CoA_transcript4590/f4p0/3991	1.86	7.92	2.393333333	2.2723	0.16719	0.31176		-1.8843	4.0631E-09	1.3894E-07	DOWN	0.36912	0.75345	0.87596	
transcript_HQ_CoA_transcript4619/f3p0/3914	16.02333333	0.58	5.56	-4.5774	0.027273	0.077712		3.0726	1.5746E-07	3.7289E-06	UP	-1.5161	0.44913	0.6592	
transcript_HQ_CoA_transcript4669/f2p0/3968	4.12	2.03	5.193333333	-0.85144	0.026281	0.075533		1.1915	0.00021492	0.0018263	UP	0.32826	0.27319	0.48599	
transcript_HQ_CoA_transcript4680/f2p0/3970	9.493333333	14.67	7.39	0.77911	0.017178	0.053797		-1.1413	2.1011E-07	4.8259E-06	DOWN	-0.37373	0.25793	0.46859	
transcript_HQ_CoA_transcript4698/f3p0/3937	11.93333333	5.4	13.35666667	-0.97854	0.0010457	0.0053431		1.1347	0.00074873	0.0050547	UP	0.14434	0.58258	0.76091	
transcript_HQ_CoA_transcript4706/f2p0/3975	0.176666667	0.536666667	0	1.879	0.16275	0.30598		-7.1734	1.2633E-05	0.00016527	DOWN	-5.3141	0.076306	0.21237	
transcript_HQ_CoA_transcript4734/f2p0/3952	5.896666667	2.74	9.33	-0.95642	0.0004093	0.002383		1.6249	4.9267E-09	1.6539E-07	UP	0.65571	0.0081609	0.043487	
transcript_HQ_CoA_transcript4788/f2p0/3948	7.993333333	1.9	6.123333333	-1.856	0.025432	0.07358		1.5046	0.0074954	0.032084	UP	-0.36972	0.58623	0.76325	
transcript_HQ_CoA_transcript4796/f2p0/3924	2.443333333	1.153333333	2.84	-0.92796	0.27553	0.44408		1.1775	0.0054845	0.025182	UP	0.23437	0.75098	0.87449	
transcript_HQ_CoA_transcript4843/f2p0/3957	5.23	2.82	9.33	-0.72809	0.024053	0.070448		1.5549	1.1076E-05	0.00014829	UP	0.81535	0.016793	0.072954	
transcript_HQ_CoA_transcript4921/f2p0/3903	6.886666667	7.14	3.77	0.208	0.5491	0.70949		-1.078	2.016E-06	3.4663E-05	DOWN	-0.88053	0.0072141	0.039705	
transcript_HQ_CoA_transcript5052/f3p0/3891	14.38333333	23.28	10.77333333	0.87259	0.0080996	0.029598		-1.263	0.00061798	0.0043167	DOWN	-0.40528	0.25613	0.46668	
transcript_HQ_CoA_transcript5064/f2p0/3909	4.446666667	2.883333333	8.333333333	-0.48097	0.44322	0.61668		1.3736	1.8653E-06	3.2315E-05	UP	0.88499	0.10589	0.2667	
transcript_HQ_CoA_transcript5187/f2p0/3884	1.78	0.436666667	12.30666667	-1.9579	NA	NA	NA	4.6898	1.2294E-19	2.7442E-17	UP	2.7319	0.046247	0.15185	
transcript_HQ_CoA_transcript5214/f2p0/3851	3.283333333	4.923333333	2.02	0.77639	0.1103	0.2285		-1.4544	0.00015824	0.0014093	DOWN	-0.69295	0.11496	0.28138	
transcript_HQ_CoA_transcript5254/f2p0/3830	3.25	2.293333333	6.293333333	-0.37767	0.59304	0.74499		1.3121	4.2999E-10	1.8414E-08	UP	0.9257	0.14353	0.32377	
transcript_HQ_CoA_transcript5309/f2p0/3815	1.056666667	0.363333333	1.67	-1.3712	0.041431	0.10811		2.0284	0.00036471	0.0028098	UP	0.64639	0.12934	0.30266	
transcript_HQ_CoA_transcript5344/f2p0/3808	2.473333333	3.863333333	1.953333333	0.80072	0.022691	0.067268		-1.1347	0.0022414	0.012166	DOWN	-0.34805	0.39913	0.61481	
transcript_HQ_CoA_transcript5412/f2p0/3788	0.6	3.37	0.163333333	2.6704	0.04031	0.10578		-4.5685	5.0282E-05	0.00054437	DOWN	-1.9192	0.28696	0.50139	
transcript_HQ_CoA_transcript5420/f2p0/3797	2.606666667	1.68	3.783333333	-0.49445	0.19247	0.3446		1.0315	0.0059115	0.026747	UP	0.52686	0.11419	0.27996	
transcript_HQ_CoA_transcript5446/f3p0/3791	9.546666667	4.46	11.19333333	-0.94885	6.6074E-05	0.00049124		1.1745	6.2674E-06	9.0882E-05	UP	0.21415	0.40308	0.61799	
transcript_HQ_CoA_transcript5458/f2p0/3774	4.123333333	9.003333333	0.443333333	1.2024	0.52663	0.69051		-4.4618	2.2948E-05	0.00027461	DOWN	-3.2645	NA	NA	NA
transcript_HQ_CoA_transcript5463/f2p0/3788	5.353333333	6.47	2.926666667	0.43501	0.086261	0.19035		-1.2853	0.00064129	0.0044658	DOWN	-0.86407	0.034892	0.12421	
transcript_HQ_CoA_transcript5494/f2p0/3789	3.956666667	5.166666667	1.646666667	0.50461	0.4985	0.66605		-1.78	0.0057436	0.026149	DOWN	-1.2862	0.04951	0.15921	
transcript_HQ_CoA_transcript5576/f2p0/3787	2.956666667	1.616666667	5.416666667	-0.68215	0.14261	0.2776		1.5628	0.00011399	0.0010714	UP	0.86746	0.00161	0.012899	
transcript_HQ_CoA_transcript5643/f2p0/3758	26.64666667	40.13666667	21.67333333	0.75097	0.0082709	0.030151		-1.0483	6.2386E-06	9.0616E-05	DOWN	-0.30846	0.16256	0.35097	
transcript_HQ_CoA_transcript5647/f2p0/3774	8.903333333	8.826666667	4.81	0.14023	0.48544	0.65489		-1.0204	1.0512E-05	0.00014189	DOWN	-0.89187	0.00065512	0.0064029	
transcript_HQ_CoA_transcript5694/f2p0/3727	65.08333333	47.05	114.76	-0.33001	0.27696	0.44564		1.1426	2.9515E-05	0.00034348	UP	0.80294	0.0083635	0.044278	
transcript_HQ_CoA_transcript5701/f2p0/3740	0.733333333	0.32	0.983333333	-1.0311	0.14579	0.28204		1.4678	0.0091257	0.037319	UP	0.42634	0.44174	0.65272	
transcript_HQ_CoA_transcript5712/f2p0/3750	4.653333333	3.726666667	9.246666667	-0.18878	0.80603	0.88727		1.1616	5.8921E-05	0.00061607	UP	0.9678	0.16329	0.35195	
transcript_HQ_CoA_transcript5732/f2p0/3725	10.01333333	5.183333333	13.15	-0.80758	0.019068	0.058431		1.1991	0.00013265	0.0012156	UP	0.3819	0.22157	0.42787	
transcript_HQ_CoA_transcript5770/f2p0/3729	14.38666667	8.953333333	21.35666667	-0.54142	0.020917	0.062906		1.1147	1.9756E-05	0.00024124	UP	0.56353	0.029183	0.10963	
transcript_HQ_CoA_transcript5802/f2p0/3786	2.746666667	0.903333333	4.026666667	-1.4699	0.031759	0.08756		2.0207	0.0012068	0.0074125	UP	0.53994	0.23243	0.44082	
transcript_HQ_CoA_transcript5891/f2p0/3708	8.993333333	5.093333333	15.21333333	-0.66993	0.08015	0.18041		1.429	5.3104E-07	1.1137E-05	UP	0.74823	0.053068	0.16719	
transcript_HQ_CoA_transcript5900/f2p0/3722	1.23	2.053333333	0.763333333	0.87721	0.28236	0.45159		-1.5695	0.0035553	0.017853	DOWN	-0.70425	0.41978	0.63242	
transcript_HQ_CoA_transcript5965/f2p0/3715	3.826666667	2.276666667	6.533333333	-0.63863	0.25867	0.4255		1.401	0.0074563	0.031969	UP	0.75451	0.027117	0.10354	
transcript_HQ_CoA_transcript5980/f3p0/3708	3.926666667	6.75	2.456666667	0.92335	0.02791	0.079073		-1.5863	0.00013693	0.0012465	DOWN	-0.67551	0.029859	0.11139	
transcript_HQ_CoA_transcript5987/f3p0/3709	4.35	1.316666667	5.283333333	-1.557	0.052954	0.13102		1.8249	0.004359	0.021086	UP	0.25775	0.62678	0.79128	
transcript_HQ_CoA_transcript6062/f2p0/3762	0.933333333	0	1.28	-20.926	NA	NA	NA	7.991	0.00060467	0.0042447	UP	0.40498	0.91353	0.96179	
transcript_HQ_CoA_transcript6071/f3p0/3680	2.27	1.056666667	5.35	-0.98251	0.11523	0.23642		2.2023	3.8072E-05	0.00042943	UP	1.2111	0.01017	0.050913	
transcript_HQ_CoA_transcript6111/f6p0/3676	15.46666667	1.08	30.19	-3.6101	0.023876	0.070017		4.6006	5.0484E-09	1.6916E-07	UP	0.97463	0.46812	0.6752	
transcript_HQ_CoA_transcript6115/f3p0/3693	3.443333333	4.866666667	2.123333333	0.66144	0.055449	0.13554		-1.3603	2.9606E-06	4.8537E-05	DOWN	-0.71283	0.066785	0.1947	
transcript_HQ_CoA_transcript615/f10p0/6112	19.35666667	34.61	16.95	0.97237	0.0025454	0.01132		-1.1624	0.00047444	0.0035008	DOWN	-0.20215	0.29623	0.51178	
transcript_HQ_CoA_transcript6150/f4p0/3597	3.933333333	1.23	3.72	-1.5269	0.04344	0.11239		1.4142	0.0025982	0.013744	UP	-0.12251	0.85962	0.93686	

transcript_HQ_CoA_transcript6188/f2p0/3664	1.016666667	1.163333333	0.406666667	0.36845	0.54188	0.70369	-1.6711	0.00064892	0.0045087	DOWN	-1.3178	0.061356	0.18402	
transcript_HQ_CoA_transcript6233/f5p0/3674	31.06666667	18.36666667	45.35	-0.56752	0.27271	0.44122	1.1285	0.013186	0.049886	UP	0.54672	0.16421	0.35294	
transcript_HQ_CoA_transcript6253/f6p0/3659	3.873333333	2.03	7.75	-0.77309	0.070977	0.16498	1.7651	2.0189E-06	3.4681E-05	UP	0.98278	0.010543	0.052188	
transcript_HQ_CoA_transcript6282/f3p0/3679	33.68666667	38.84333333	17.33666667	0.36674	0.054415	0.13364	-1.3102	8.6276E-07	1.6877E-05	DOWN	-0.95704	0.00042686	0.0045552	
transcript_HQ_CoA_transcript6328/f2p0/3640	0.68	0.893333333	0.413333333	0.54648	0.3828	0.55831	-1.2638	0.0097784	0.039313	DOWN	-0.7265	0.29414	0.50984	
transcript_HQ_CoA_transcript643/f2p0/6148	1.016666667	2.77	0.69	1.5984	0.18801	0.33924	-2.1337	0.00014828	0.0013356	DOWN	-0.5483	0.66595	0.81807	
transcript_HQ_CoA_transcript646/f2p0/6159	9.67	12.84	6.506666667	0.56185	0.011728	0.040091	-1.1281	3.0819E-08	8.6241E-07	DOWN	-0.57803	0.0034897	0.023318	
transcript_HQ_CoA_transcript6460/f2p0/3614	8.096666667	10.93	5.13	0.56449	0.27053	0.43869	-1.2333	4.136E-06	6.3866E-05	DOWN	-0.67808	0.15364	0.33793	
transcript_HQ_CoA_transcript6488/f2p0/3632	17.98666667	9.376666667	21.67333333	-0.78977	0.046659	0.11862	1.0533	2.8844E-08	8.0962E-07	UP	0.25386	0.47588	0.68104	
transcript_HQ_CoA_transcript654/f2p0/6307	0.876666667	1.44	0.783333333	0.87901	0.071888	0.16657	-1.0266	0.010582	0.041903	DOWN	-0.15767	0.78045	0.89309	
transcript_HQ_CoA_transcript6579/f2p0/3588	7.976666667	5.25	11.79333333	-0.4674	0.18028	0.32987	1.03	0.0039656	0.019534	UP	0.5528	0.034261	0.1227	
transcript_HQ_CoA_transcript6599/f2p0/3589	15.59333333	19.91333333	5.583333333	0.50939	0.46323	0.63508	-1.9493	5.2824E-05	0.0005639	DOWN	-1.4539	0.031808	0.11632	
transcript_HQ_CoA_transcript6678/f2p0/3572	1.066666667	0.326666667	2.193333333	-1.56	0.050841	0.12698	2.6043	0.00053852	0.0038723	UP	1.0357	0.042595	0.14322	
transcript_HQ_CoA_transcript6749/f2p0/3566	1.706666667	0.92	2.483333333	-0.75584	0.10795	0.22481	1.3013	0.0019389	0.010825	UP	0.53456	0.17966	0.37458	
transcript_HQ_CoA_transcript6776/f2p0/3552	2.403333333	1.09	3.16	-0.96022	0.03123	0.086401	1.369	5.3751E-05	0.00057212	UP	0.39593	0.23739	0.44576	
transcript_HQ_CoA_transcript6796/f2p0/3551	3.056666667	4.54	1.593333333	0.69705	0.12832	0.25622	-1.6438	0.00094416	0.0060914	DOWN	-0.95989	0.013538	0.062496	
transcript_HQ_CoA_transcript6799/f2p0/3557	11.95666667	12.81666667	6.38	0.2721	0.31939	0.49233	-1.1652	1.6441E-06	2.9201E-05	DOWN	-0.90672	8.2449E-05	0.0012107	
transcript_HQ_CoA_transcript6848/f5p0/3537	12.62	5.26	11.85666667	-1.0946	0.072252	0.16714	1.0265	0.00013262	0.0012156	UP	-0.084498	0.87663	0.94489	
transcript_HQ_CoA_transcript6853/f2p0/3547	4.796666667	3.333333333	7.5	-0.35349	0.34035	0.51543	1.0072	0.003163	0.016171	UP	0.63898	0.10257	0.26137	
transcript_HQ_CoA_transcript6868/f2p0/3542	2.616666667	1.036666667	3.533333333	-1.1861	0.051529	0.12838	1.6287	2.0914E-06	3.5793E-05	UP	0.42995	0.39979	0.61526	
transcript_HQ_CoA_transcript687/f2p0/6097	0.383333333	0.916666667	0.326666667	1.3906	0.12416	0.2499	-1.602	0.011179	0.043792	DOWN	-0.22379	0.798	0.90355	
transcript_HQ_CoA_transcript6886/f2p0/3558	8.233333333	3.736666667	11.14	-0.97653	0.011156	0.038513	1.4258	6.2819E-06	9.0998E-05	UP	0.4369	0.24867	0.45868	
transcript_HQ_CoA_transcript6904/f3p0/3595	47.71	84.10666667	43.92333333	0.97231	0.00018966	0.0012278	-1.085	2.7947E-08	7.9049E-07	DOWN	-0.12425	0.63191	0.79519	
transcript_HQ_CoA_transcript6954/f2p0/3562	25.01333333	12.91	47.49333333	-0.7849	0.14533	0.28145	1.7006	0.00035771	0.0027595	UP	0.90576	0.027131	0.10355	
transcript_HQ_CoA_transcript6964/f2p0/3588	22.55	23.34	12.45666667	0.20248	0.40477	0.57899	-1.0482	1.6364E-06	2.9091E-05	DOWN	-0.85891	4.2714E-06	0.00010538	
transcript_HQ_CoA_transcript6974/f2p0/3543	5.713333333	3.636666667	9.87	-0.47627	0.24515	0.40971	1.2797	1.5857E-07	3.7453E-06	UP	0.78763	0.041932	0.14177	
transcript_HQ_CoA_transcript7006/f4p0/3514	5.943333333	10.02	4.173333333	0.91183	0.00016393	0.0010811	-1.4129	7.1219E-06	0.00010151	DOWN	-0.51352	0.12101	0.29041	
transcript_HQ_CoA_transcript7039/f2p0/3598	2.61	0.51	1.773333333	-2.2402	0.021249	0.063713	1.6613	0.01022	0.040756	UP	-0.58619	0.4722	0.67824	
transcript_HQ_CoA_transcript704/f2p0/6055	5.276666667	4.436666667	10.55	-0.093692	0.78215	0.87191	1.1006	0.00014331	0.0012933	UP	0.99296	0.0020416	0.015517	
transcript_HQ_CoA_transcript7046/f2p0/3510	5.726666667	4.94	2.473333333	-0.04048	0.96099	0.97914	-1.1444	3.2512E-05	0.0003755	DOWN	-1.1999	0.13729	0.31488	
transcript_HQ_CoA_transcript7050/f3p0/3549	30.57666667	19.27666667	46.20666667	-0.50526	0.014979	0.048504	1.0995	5.0449E-06	0.0000753	UP	0.58234	0.0039754	0.02574	
transcript_HQ_CoA_transcript7076/f2p0/3506	1.17	0.636666667	2.463333333	-0.7772	0.29205	0.46236	1.8339	0.0065591	0.028898	UP	1.0503	0.073097	0.20594	
transcript_HQ_CoA_transcript7095/f3p0/3516	13.66	22.27	11.15333333	0.85922	8.8354E-06	8.4349E-05	-1.1404	0.00010592	0.0010101	DOWN	-0.29359	0.33456	0.55219	
transcript_HQ_CoA_transcript7099/f4p0/3438	17.91666667	22.53	9.85	0.46929	0.22039	0.37954	-1.3258	0.00039965	0.0030307	DOWN	-0.86874	7.3676E-05	0.0011039	
transcript_HQ_CoA_transcript7102/f2p0/3511	6.8	2.856666667	10.82666667	-1.0608	0.10485	0.22022	1.7482	1.8439E-08	5.3989E-07	UP	0.67151	0.23948	0.44798	
transcript_HQ_CoA_transcript713/f2p0/6045	5.206666667	8.43	4.583333333	0.86067	0.0011082	0.0056066	-1.0319	9.9803E-10	3.9004E-08	DOWN	-0.18548	0.50899	0.7077	
transcript_HQ_CoA_transcript7194/f2p0/3454	15.15333333	17.71	6.42	0.38308	0.48416	0.65406	-1.5852	7.6642E-05	0.00077059	DOWN	-1.2145	0.032711	0.11866	
transcript_HQ_CoA_transcript7235/f4p0/3504	11.04	19.14	6.833333333	0.95308	0.0028004	0.01228	-1.6244	1.6983E-08	5.0288E-07	DOWN	-0.6855	0.059935	0.18118	
transcript_HQ_CoA_transcript7344/f2p0/3468	14.3	14.95333333	7.893333333	0.2212	0.74062	0.84461	-1.0462	0.0091005	0.037266	DOWN	-0.84006	0.16827	0.35871	
transcript_HQ_CoA_transcript7441/f2p0/3447	3.143333333	5.48	1.073333333	0.97048	0.16691	0.31136	-2.4822	4.3504E-08	1.1761E-06	DOWN	-1.5248	0.047504	0.15471	
transcript_HQ_CoA_transcript7467/f3p0/3449	1.92	0.396666667	3.646666667	-2.0315	0.057348	0.13946	2.9658	0.0029253	0.015134	UP	0.92122	0.016855	0.073102	
transcript_HQ_CoA_transcript7487/f2p0/3437	13.01666667	7.086666667	17.01333333	-0.70888	0.0077173	0.028434	1.1009	8.7082E-06	0.00012028	UP	0.37892	0.10014	0.25733	
transcript_HQ_CoA_transcript7530/f9p0/3424	9.51	7.823333333	18.55	-0.12269	0.6706	0.80088	1.0908	0.00024418	0.0020126	UP	0.95634	0.0042442	0.027031	
transcript_HQ_CoA_transcript7574/f2p0/3431	7.486666667	11.57	6.003333333	0.77072	0.0042128	0.017234	-1.0871	8.6216E-06	0.00011944	DOWN	-0.32798	0.16303	0.35156	
transcript_HQ_CoA_transcript7578/f3p0/3448	14.47666667	6.59	15.42666667	-0.99773	0.0039268	0.016312	1.0833	0.0010432	0.006607	UP	0.074506	0.7303	0.86209	
transcript_HQ_CoA_transcript7634/f2p0/3451	4.976666667	8.586666667	2.946666667	0.96105	0.015199	0.049024	-1.7084	1.6217E-06	2.8887E-05	DOWN	-0.7625	0.033148	0.11972	
transcript_HQ_CoA_transcript7675/f3p0/3429	15.71	15.3	8.383333333	0.10215	0.77288	0.86538	-1.0013	0.0035424	0.017808	DOWN	-0.91036	6.7677E-05	0.0010334	
transcript_HQ_CoA_transcript7738/f3p0/3418	12.93666667	21.96666667	8.736666667	0.9167	0.024001	0.070319	-1.4757	0.00036562	0.0028122	DOWN	-0.57236	0.016552	0.072148	
transcript_HQ_CoA_transcript7739/f2p0/3445	3.673333333	2.013333333	5.336666667	-0.68583	0.22899	0.38997	1.2367	0.0042969	0.020859	UP	0.53843	0.21954	0.42532	
transcript_HQ_CoA_transcript7778/f3p0/3383	1.153333333	0.42	1.213333333	-1.3061	0.016485	0.052141	1.401	0.0046883	0.022291	UP	0.083117	0.8599	0.93688	
transcript_HQ_CoA_transcript7781/f2p0/3433	2.103333333	1.913333333	0.556666667	0.020103	0.97952	0.9901	-1.9043	0.0052304	0.024282	DOWN	-1.8972	0.037935	0.1319	
transcript_HQ_CoA_transcript7830/f4p0/3391	7.45	3.523333333	8.44	-0.91163	0.0053312	0.02092	1.0967	0.00031144	0.002451	UP	0.17306	0.3537	0.57115	
transcript_HQ_CoA_transcript787/f2p0/5944	13.74666667	7.85	18.47333333	-0.64243	0.029478	0.08242	1.0704	0.00020627	0.001768	UP	0.41586	0.0093276	0.047831	
transcript_HQ_CoA_transcript7875/f2p0/3388	2.82	1.56	6.753333333	-0.71759	0.30168	0.47276	1.9634	3.4729E-08	9.6156E-07	UP	1.2399	0.042163	0.14237	
transcript_HQ_CoA_transcript7900/f2p0/3389	9.053333333	12.48666667	3.27	0.56409	0.48998	0.65857	-2.0589	0.0016285	0.0094248	DOWN	-1.5006	0.022642	0.090583	
transcript_HQ_CoA_transcript7925/f2p0/3401	17.31	9.736666667	28.18333333	-0.69672	0.0661	0.15583	1.3943	0.0020529	0.011337	UP	0.68762	0.067756	0.19647	
transcript_HQ_CoA_transcript7938/f3p0/3368	0.483333333	0.533333333	0.103333333	0.2766	0.7267	0.83636	-2.3884	0.012096	0.046662	DOWN	-2.1239	0.018321	0.07806	
transcript_HQ_CoA_transcript8008/f2p0/3364	1.356666667	0.646666667	1.536666667	-0.91889	0.15724	0.2986	1.0795	0.0098237	0.039452	UP	0.15239	0.78539	0.89579	
transcript_HQ_CoA_transcript8097/f3p0/3335	0.24	0.64	0.106666667	1.5572	0.052519	0.13023	-2.8086	0.00010399	0.00099667	DOWN	-1.255	0.26751	0.47956	
transcript_HQ_CoA_transcript8144/f2p0/3376	6.88	12.61	6.78	1.027	0.015856	0.050573	-1.0319	0.0022454	0.01218	DOWN	-0.01896	0.95637	0.98127	
transcript_HQ_CoA_transcript8201/f2p0/3344	1.646666667	0.113333333	3.21	-3.6268	0.016812	0.052957	4.6172	0.00057284	0.0040604	UP	0.97283	0.1003	0.25765	
transcript_HQ_CoA_transcript8212/f2p0/3335	3.37	1.44	5.486666667	-1.0476	0.017493	0.054584	1.76	7.7053E-06	0.00010797	UP	0.69936	0.015893	0.070261	
transcript_HQ_CoA_transcript8319/f2p0/3321	12.61333333	21.87333333	9.123333333	0.94887	0.0087125	0.031447	-1.4113	8.8064E-05	0.00086781	DOWN	-0.47533	0.033475	0.1207	
transcript_HQ_CoA_transcript8332/f2p0/3341	2.94	1.776666667	4.05	-0.5782	0.10189	0.21542	1.0352	0.0092593	0.037712	UP	0.44602	0.24912	0.45903	
transcript_HQ_CoA_transcript8340/f3p0/3318	9.43	3.2	12.3	-1.423	0.074503	0.17122	1.7715	7.3995E-06	0.00010489	UP	0.34006	0.64864	0.80633	
transcript_HQ_CoA_transcript8470/f4p0/3371	8.51	4.12	10.28	-0.87982	0.074345	0.17094	1.1425	0.010244	0.040826	UP	0.25282	0.48819	0.69065	
transcript_HQ_CoA_transcript8477/f2p0/3303	3.456666667	2.146666667	5.423333333	-0.50436	0.33763	0.51267	1.1579	0.011462	0.044689	UP	0.64298	0.093111	0.24443	
transcript_HQ_CoA_transcript85/f2p0/7988	4.42	2.863333333	7.79	-0.46824	0.44453	0.61783	1.3049	0.0003688	0.0028331	UP	0.82258	0.14968	0.33263	
transcript_HQ_CoA_transcript855/f4p0/5860	4.326666667	2.976666667	6.983333333	-0.3771	0.19076	0.34271	1.0786	6.3623E-07	1.2943E-05	UP	0.68811	0.011462	0.055658	
transcript_HQ_CoA_transcript8585/f2p0/3283	3.236666667	11.62666667	1.22	1.9998	0.016809	0.052957	-3.3725	9.9679E-07	1.9031E-05	DOWN	-1.388	0.048524	0.15694	
transcript_HQ_CoA_transcript8591/f5p0/3275	28.48	15.66666667	36.19666667	-0.70267	0.010914	0.037833	1.0582	0.00071388	0.004873	UP	0.34294	0.31077	0.52753	
transcript_HQ_CoA_transcript861/f3p0/5885	3.596666667	12.95666667	0.766666667	1.9531	0.13922	0.27267	-4.2176	1.1175E-21	3.5314E-19	DOWN	-2.2703	NA	NA	NA
transcript_HQ_CoA_transcript8610/f3p0/3248	2.79	1.75	4.333333333	-0.52445	0.11799	0.24058	1.1569	6.8259E-05	0.0006974	UP	0.62089	0.026054	0.10055	
transcript_HQ_CoA_transcript8715/f6p0/3256	3.546666667	1.866666667	7.173333333	-0.73692	0.33744	0.51247	1.7707	0.0035751	0.017923	UP	1.0169	0.13322	0.30878	
transcript_HQ_CoA_transcript8758/f2p0/3269	1.193333333	0.586666667	2.456666667	-0.88367	0.28106	0.45004	1.909	1.1832E-06	2.1884E-05	UP	1.0194	0.1339	0.30973	
transcript_HQ_CoA_transcript8762/f2p0/3271	6.01	3.46	8.076666667	-0.61677	0.1557	0.29635	1.0577	0.00011072	0.0010483	UP	0.42676	0.25117	0.46162	
transcript_HQ_CoA_transcript8788/f12p0/3226	11.25	16.28666667	7.126666667	0.67645	0.049275	0.12383	-1.3434	2.2223E-21	6.5699E-19	DOWN	-0.67743	0.047422	0.15455	
transcript_HQ_CoA_transcript8812/f2p0/3255	2.916666667	1.343333333	16.21666667	-0.96264	NA	NA	3.399	1.3629E-07	3.2782E-06	UP	2.4308	NA	NA	NA
transcript_HQ_CoA_transcript8831/f7p0/3218	42.55333333	28.59666667	63.66333333	-0.41126	0.25236	0.41821	1.0032	0.0012026	0.0073995	UP	0.57834	0.11782	0.28563	

transcript_HQ_CoA_transcript8846/f2p0/3292	1.836666667	4.076666667	1.12	1.2867	0.03512	0.094882		-1.9598	0.0078616	0.033286	DOWN	-0.68427	0.31727	0.53411
transcript_HQ_CoA_transcript8865/f2p0/3258	4.616666667	2.296666667	7.24	-0.8575	0.010234	0.035924		1.4994	9.5247E-07	0.00001828	UP	0.63022	0.018692	0.079217
transcript_HQ_CoA_transcript8871/f2p0/3235	10.74666667	6.513333333	16.85333333	-0.5489	0.46043	0.63262		1.2222	0.0013658	0.0081898	UP	0.65681	0.35564	0.57291
transcript_HQ_CoA_transcript8882/f4p0/3230	4.653333333	1.423333333	4.973333333	-1.5817	0.04578	0.11688		1.66	5.8866E-09	1.9336E-07	UP	0.071268	0.92081	0.96509
transcript_HQ_CoA_transcript8889/f2p0/3279	4.216666667	13.43	0	1.8576	0.29128	0.46151		-11.481	2.1523E-21	6.4671E-19	DOWN	-9.6414	0.013607	0.062707
transcript_HQ_CoA_transcript8965/f3p0/3180	5.59	6.9	3.283333333	0.44255	0.18689	0.33793		-1.2108	4.2017E-05	0.00046617	DOWN	-0.77884	0.011397	0.055413
transcript_HQ_CoA_transcript901/f2p0/5843	1.543333333	4.363333333	0.66	1.6553	0.39506	0.57		-2.8374	0.0014704	0.0086966	DOWN	-1.1993	0.34544	0.56321
transcript_HQ_CoA_transcript904/f2p0/5822	7.753333333	5.873333333	2.61	-0.24782	0.70184	0.82045		-1.2889	0.0035312	0.017762	DOWN	-1.5494	0.016029	0.070565
transcript_HQ_CoA_transcript9082/f2p0/3215	8.35	4.683333333	12.58	-0.67528	0.0724	0.16746		1.2617	7.5495E-05	0.0007603	UP	0.57661	0.12025	0.28918
transcript_HQ_CoA_transcript9099/f2p0/3239	8.346666667	13.87666667	7.37	0.88821	0.0082421	0.030052		-1.0617	0.0007034	0.0048143	DOWN	-0.18568	0.40478	0.61934
transcript_HQ_CoA_transcript9119/f2p0/3201	3.656666667	4.916666667	2.726666667	0.59345	0.077357	0.17573		-1.0104	0.0011993	0.0073841	DOWN	-0.43014	0.14563	0.32699
transcript_HQ_CoA_transcript9215/f2p0/3213	1.063333333	0.266666667	2.066666667	-1.9189	0.01722	0.053906		2.8788	0.0001411	0.0012759	UP	0.95291	0.10955	0.27268
transcript_HQ_CoA_transcript9245/f8p0/3165	9.166666667	4.343333333	16.35	-0.9596	0.15524	0.29561		1.7875	0.0044984	0.021584	UP	0.8173	0.016202	0.071037
transcript_HQ_CoA_transcript928/f3p0/5835	3.433333333	2.36	5.75	-0.37889	0.099108	0.21103		1.1313	7.6504E-05	0.00076962	UP	0.7408	0.011684	0.056289
transcript_HQ_CoA_transcript9307/f2p0/3204	27.7	12.58	30.88666667	-0.98211	0.00044669	0.0025616		1.1412	0.0002353	0.0019516	UP	0.14895	0.58576	0.76307
transcript_HQ_CoA_transcript933/f2p0/5800	3.926666667	1.973333333	5.403333333	-0.84195	0.0021069	0.0096845		1.3047	2.0497E-07	4.7196E-06	UP	0.45217	0.050657	0.16148
transcript_HQ_CoA_transcript9347/f3p0/3241	1.893333333	3.313333333	1.816666667	0.98402	0.32666	0.50011		-1.0139	0.0077138	0.03282	DOWN	-0.049094	0.96061	0.98285
transcript_HQ_CoA_transcript9442/f4p0/3184	14.57333333	15.43666667	8.346666667	0.24137	0.44641	0.61945		-1.0296	9.3736E-06	0.0001285	DOWN	-0.80229	0.0062972	0.036245
transcript_HQ_CoA_transcript9473/f2p0/3251	1.93	3.593333333	0.96	1.0167	0.16664	0.31113		-2.0602	2.365E-08	6.7626E-07	DOWN	-1.0537	0.18821	0.38545
transcript_HQ_CoA_transcript9533/f9p0/3113	6.033333333	2.766666667	7.84	-0.96846	0.063451	0.15102		1.366	0.004088	0.020045	UP	0.38548	0.38414	0.59957
transcript_HQ_CoA_transcript9542/f2p0/3169	9.71	8.013333333	4.11	-0.10929	0.82646	0.8999		-1.1003	2.9177E-05	0.00033977	DOWN	-1.2231	0.015382	0.068663
transcript_HQ_CoA_transcript9562/f2p0/3168	1.456666667	0.823333333	0.19	-0.70533	0.54602	0.70722		-2.2475	0.0062357	0.027877	DOWN	-2.9594	0.022116	0.089137
transcript_HQ_CoA_transcript9618/f2p0/3165	11.24	10.78666667	5.53	0.096502	0.83239	0.90367		-1.1013	0.0023388	0.012604	DOWN	-1.0202	0.011545	0.055941
transcript_HQ_CoA_transcript9655/f2p0/3183	4.256666667	7.546666667	2.883333333	0.99739	0.0021883	0.0099891		-1.5413	2.4857E-05	0.00029465	DOWN	-0.55846	0.15426	0.33877
transcript_HQ_CoA_transcript9692/f6p0/3040	46.04666667	80.87333333	43.31	0.97316	0.0000424	0.000333		-1.047	0.00088781	0.0058137	DOWN	-0.084352	0.76989	0.88614
transcript_HQ_CoA_transcript97/f2p0/7852	4.193333333	0.063333333	7.866666667	-5.8726	NA	NA	NA	6.7307	2.0662E-14	2.1766E-12	UP	0.85561	0.48148	0.68557
transcript_HQ_CoA_transcript9700/f9p0/3100	24.80333333	42.04666667	21.70666667	0.9237	0.0062779	0.023926		-1.1063	0.0014496	0.0086041	DOWN	-0.1963	0.30701	0.52385
transcript_HQ_CoA_transcript9795/f2p0/3136	4.75	3.093333333	7.376666667	-0.47236	0.13006	0.25879		1.1082	0.00013059	0.0011998	UP	0.62469	0.012467	0.058948
transcript_HQ_CoA_transcript98/f2p0/7836	13.24333333	14.32333333	7.35	0.2709	0.43437	0.60828		-1.1018	8.5607E-07	1.6764E-05	DOWN	-0.84491	0.0064721	0.036905
transcript_HQ_CoA_transcript9933/f2p0/3131	26.14666667	27.38	14.63	0.21061	0.45434	0.62662		-1.0436	0.00023129	0.0019243	DOWN	-0.84553	7.1246E-08	3.2531E-06
transcript_HQ_CoA_transcript9959/f2p0/3066	0.986666667	1.65	0.803333333	0.89625	0.046932	0.11921		-1.1978	0.008239	0.034494	DOWN	-0.31665	0.58182	0.76026
transcript_HQ_CoA_transcript9991/f2p0/3148	13.38666667	6.523333333	19.87	-0.87957	0.0192	0.058739		1.4506	7.1002E-05	0.00072168	UP	0.55826	0.17602	0.36934
transcript_HQ_CoA_transcript9999/f4p0/3075	7.643333333	4.806666667	12.91333333	-0.52885	0.18297	0.33308		1.2899	0.0045622	0.021816	UP	0.75	0.046182	0.15175

Supplementary Table S4. Significantly enriched GO categories of differentially accumulating mRNAs

			GO terms		p-value	Num.
biological_process	Up-regulated	CoA2 vs. CoA1	GO:0007018	microtubule-based movement	4.9927E-06	36
			GO:0005975	carbohydrate metabolic process	0.001049	114
			GO:0007017	microtubule-based process	0.0011279	41
			GO:0044723	single-organism carbohydrate metabolic process	0.008788	65
			GO:0006928	movement of cell or subcellular component	0.023529	41
			GO:0006749	glutathione metabolic process	0.030555	6
			GO:0055114	oxidation-reduction process	0.046251	148
		CoA3 vs. CoA2	GO:0006633	fatty acid biosynthetic process	0.0097762	15
			GO:0005984	disaccharide metabolic process	0.0097762	14
			GO:0008610	lipid biosynthetic process	0.0097762	31
			GO:0072330	monocarboxylic acid biosynthetic process	0.010131	15
			GO:0009311	oligosaccharide metabolic process	0.032696	15
		CoA3 vs. CoA1	GO:0007018	microtubule-based movement	1.5333E-11	33
			GO:0007017	microtubule-based process	1.6704E-08	36
			GO:0006928	movement of cell or subcellular component	5.8681E-07	36
			GO:0055114	oxidation-reduction process	1.309E-06	104
			GO:0044710	single-organism metabolic process	2.6059E-05	203
			GO:0044699	single-organism process	6.9978E-05	332
			GO:0006633	fatty acid biosynthetic process	0.0012779	15
			GO:0008610	lipid biosynthetic process	0.0018821	31

	GO:0072330	monocarboxylic acid biosynthetic process	0.0019568	15
Down-regulated CoA2 vs. CoA1	GO:0044703	multi-organism reproductive	0.022052	14
	GO:0000902	cell morphogenesis	0.041194	14
	GO:0032989	cellular component morphogenesis	0.041194	14
	GO:0009653	anatomical structure morphogenesis	0.041194	15
	GO:0006355	regulation of transcription, DNA-templated	0.041194	107
	GO:1903506	regulation of nucleic acid-templated transcription	0.041194	107
	GO:0051252	regulation of RNA metabolic process	0.041194	107
	GO:2001141	regulation of RNA biosynthetic process	0.041194	107
	GO:0019219	regulation of nucleobase-containing compound metabolic process	0.041194	107
	GO:0045132	meiotic chromosome segregation	0.041194	5
	GO:0007129	synapsis	0.041194	4
	GO:0007130	synaptonemal complex assembly	0.041194	4
	GO:0045143	homologous chromosome segregation	0.041194	4
	GO:0070192	chromosome organization involved in meiosis	0.041194	4
	GO:0070193	synaptonemal complex organization	0.041194	4
	GO:0031323	regulation of cellular metabolic process	0.04153	122
	GO:0015994	chlorophyll metabolic process	0.04153	7

		CoA3 vs. CoA2	None		None	None
		CoA3 vs. CoA1	GO:0009653	anatomical structure morphogenesis	1.5333E-11	33
			GO:0000902	cell morphogenesis	1.6704E-08	36
			GO:0032989	cellular component morphogenesis	5.8681E-07	36
			GO:0048869	cellular developmental process	1.309E-06	104
			GO:0048856	anatomical structure development	2.6059E-05	203
			GO:0051297	centrosome organization	6.9978E-05	332
			GO:0044767	single-organism developmental process	0.0012779	15
			GO:0032502	developmental process	0.0018821	31
			GO:0000226	microtubule cytoskeleton organization	0.0019568	15
molecular_function	Up-regulated	CoA2 vs. CoA1	GO:0003824	catalytic activity	2.0776E-06	862
			GO:0003777	microtubule motor activity	4.9927E-06	36
			GO:0016758	transferase activity, transferring hexosyl groups	0.0001217	55
			GO:0003774	motor activity	0.0001217	38
			GO:0008017	microtubule binding	0.0001442	38
			GO:0016757	transferase activity, transferring glycosyl groups	0.00020355	72
			GO:0015631	tubulin binding	0.0017997	40
			GO:0008378	galactosyltransferase activity	0.0032221	13
			GO:0016209	antioxidant activity	0.023717	21
			GO:0004594	pantothenate kinase activity	0.027408	5
			GO:0004017	adenylate kinase activity	0.031983	5
			GO:0071949	FAD binding	0.033679	17
			GO:0004536	deoxyribonuclease activity	0.034258	12

	GO:0051287	NAD binding	0.040395	30
CoA3 vs. CoA2	GO:0016702	oxidoreductase activity, acting on single donors with incorporation of molecular oxygen, incorporation of two atoms of oxygen	0.0097762	9
	GO:0016747	transferase activity, transferring acyl groups other than amino-acyl	0.0097762	25
	GO:0051213	dioxygenase activity	0.020177	9
	GO:0016798	hydrolase activity, acting on glycosyl bonds	0.032696	36
	GO:0016746	transferase activity, transferring acyl groups	0.032696	29
CoA3 vs. CoA1	GO:0003777	microtubule motor activity	1.5333E-11	33
	GO:0008017	microtubule binding	1.7382E-09	34
	GO:0003774	motor activity	4.5045E-09	33
	GO:0015631	tubulin binding	2.2367E-07	34
	GO:0003824	catalytic activity	4.168E-06	467
	GO:0016491	oxidoreductase activity	2.1973E-05	96
	GO:0008092	cytoskeletal protein binding	0.002106	39
	GO:0032403	protein complex binding	0.002231	35
	GO:0004312	fatty acid synthase activity	0.010038	9
	GO:0004315	3-oxoacyl-[acyl-carrier-protein] synthase activity	0.010038	9
	GO:0016787	hydrolase activity	0.0123	188
	GO:0050662	coenzyme binding	0.0123	44
	GO:0044877	macromolecular complex binding	0.021737	40

			GO:0016614	oxidoreductase activity, acting on CH-OH group of donors	0.021737	30
			GO:0016818	hydrolase activity, acting on acid anhydrides, in phosphorus-containing anhydrides	0.027522	80
			GO:0048037	cofactor binding	0.030177	49
	Down-regulated	CoA2 vs. CoA1	GO:0043531	ADP binding	9.6771E-08	60
			GO:0005199	structural constituent of cell wall	0.041194	6
			GO:0001071	nucleic acid binding transcription factor activity	0.041194	48
			GO:0003700	transcription factor activity, sequence-specific DNA binding	0.041194	48
		CoA3 vs. CoA2	None		None	None
		CoA3 vs. CoA1	GO:0043531	ADP binding	0.0015592	40
cellular_component	Up-regulated	CoA2 vs. CoA1	GO:0000120	RNA polymerase I transcription factor complex	0.031983	5
		CoA3 vs. CoA2	None		None	None
		CoA3 vs. CoA1	None		None	None
	Down-regulated	CoA2 vs. CoA1	None		None	None
		CoA3 vs. CoA2	None		None	None
		CoA3 vs. CoA1	GO:0005813	centrosome	0.0062322	5

Supplementary Table S5. List of differentially expressed genes crucial for the tropical *C. oleifera* anther

Transcript ID	Gene name	descriptiona	References
transcript_HQ_CoA_transcript522/f2p0/6395	*CALS5*	Required for exine formation and pollen viability	[38]
transcript_HQ_CoA_transcript18573/f2p0/2333	*ARF17*	The key of pollen wall pattern formation	[39]
transcript_HQ_CoA_transcript9289/f2p0/3200	*AMS*	A master regulator of pollen wall formation	[42]
transcript_HQ_CoA_transcript24309/f13p0/1833	*CYP704B1*	The key of sporopollenin synthesis in pollen	[5]
transcript_HQ_CoA_transcript25042/f2p0/1846	*CYP703A2*	Provided building blocks for sporopollenin synthesis in pollen	[44]
transcript_HQ_CoA_transcript17342/f7p0/2404	*ABCG26*	Required for male fertility and pollen exine formation	[45]
transcript_HQ_CoA_transcript29552/f4p0/1304	*MYB80*	Required for pollen development and the regulation of tapetal PCD	[46]
transcript_HQ_CoA_transcript1995/f2p0/4943	*RPK2*	A factor controlling anther development	[47]
transcript_HQ_CoA_transcript29844/f2p0/1184	*TKPR1*	Required for pollen wall formation	[6]
transcript_HQ_CoA_transcript26847/f2p0/1683	*TKPR2*	Required for pollen wall formation	[6]
transcript_HQ_CoA_transcript28366/f13p0/1438	*PKSA*	Required for pollen development and sporopollenin biosynthesis	[7]
transcript_HQ_CoA_transcript28893/f4p0/1455	*PKSB*	Required for pollen development and sporopollenin biosynthesis	[7]
transcript_HQ_CoA_transcript5309/f2p0/3815	*JMJ30*	Regulate flowering time at elevated temperature via regulation of *FLC*	[49]
transcript_HQ_CoA_transcript15577/f2p0/2556	*EBS*	Regulate flowering by repressing *FT*	[48]

Supplementary Table S6. Potential trans-regulated target genes of differentially accumulated lncRNAs

LncRNA ID	Target genes	Blast-Sore	Correlation-coefficient	P-value
CoA19759_2226	transcript_HQ_CoA_transcript12047/f2p0/2890	95.604	0.871395953	0.002209735
CoA19759_2226	transcript_HQ_CoA_transcript14906/f2p0/2609	93.407	0.930647956	0.000270142
CoA23805_1929	transcript_HQ_CoA_transcript557/f2p0/6308	95	-0.850307552	0.003679421
CoA18663_2328	transcript_HQ_CoA_transcript23484/f3p0/1962	88.93	0.993798205	6.14789E-08
CoA18663_2328	transcript_HQ_CoA_transcript3229/f6p0/4345	89.899	0.898120116	0.001004677
CoA18663_2328	transcript_HQ_CoA_transcript1802/f2p0/5043	92.476	0.914503413	0.000552961
CoA18663_2328	transcript_HQ_CoA_transcript9899/f2p0/3115	97.135	0.862841761	0.002744316
CoA7994_3361	transcript_HQ_CoA_transcript12045/f2p0/2878	92.181	0.83789505	0.004800844
CoA7994_3361	transcript_HQ_CoA_transcript9722/f2p0/3154	91.923	0.919984171	0.000440935
CoA7994_3361	transcript_HQ_CoA_transcript8818/f2p0/3263	91.358	0.906089636	0.000761555
CoA7994_3361	transcript_HQ_CoA_transcript1802/f2p0/5043	93.78	0.823620065	0.006356072
CoA7994_3361	transcript_HQ_CoA_transcript7912/f3p0/3386	91.221	0.828213215	0.005823021
CoA7994_3361	transcript_HQ_CoA_transcript4428/f3p0/4085	89.148	0.839647894	0.00462996
CoA8777_3260	transcript_HQ_CoA_transcript9713/f2p0/3147	98.18	0.803490863	0.009085589
CoA8777_3260	transcript_HQ_CoA_transcript14234/f4p0/2651	88.806	0.830802717	0.00553637
CoA8777_3260	transcript_HQ_CoA_transcript12103/f3p0/2847	89.773	-0.872875317	0.002125241
CoA8777_3260	transcript_HQ_CoA_transcript17096/f2p0/2429	87.5	-0.819783019	0.006826086
CoA8777_3260	transcript_HQ_CoA_transcript7574/f2p0/3431	90	0.801294607	0.009424316
CoA15206_2586	transcript_HQ_CoA_transcript12047/f2p0/2890	93.233	0.886401728	0.001453357
CoA15206_2586	transcript_HQ_CoA_transcript14906/f2p0/2609	94.977	0.964433101	2.69826E-05
CoA15206_2586	transcript_HQ_CoA_transcript5299/f2p0/3825	92.057	0.89047862	0.001284133
CoA15206_2586	transcript_HQ_CoA_transcript13012/f2p0/2744	75.889	-0.812462657	0.007787294
CoA21021_2108	transcript_HQ_CoA_transcript14349/f2p0/2676	96.939	0.892025962	0.001223662
CoA17976_2312	transcript_HQ_CoA_transcript11374/f2p0/2992	100	0.807531146	0.00848414
CoA14288_2671	transcript_HQ_CoA_transcript18621/f3p0/2319	98.93	0.855076483	0.003301346
CoA14288_2671	transcript_HQ_CoA_transcript20202/f5p0/2192	98.93	0.868039948	0.00240997
CoA14933_2611	transcript_HQ_CoA_transcript1591/f2p0/5211	80.58	0.87568357	0.001971037
CoA29697_1263	transcript_HQ_CoA_transcript11269/f2p0/2983	88.608	0.805727848	0.008749142
CoA3445_4292	transcript_HQ_CoA_transcript22060/f4p0/2012	88.934	0.91833073	0.000472874
CoA3445_4292	transcript_HQ_CoA_transcript23549/f2p0/1947	91.27	0.813635178	0.007627524
CoA29727_1231	transcript_HQ_CoA_transcript8868/f2p0/3243	97.041	0.827690281	0.005882111
CoA14354_2670	transcript_HQ_CoA_transcript12767/f2p0/2815	91.639	0.873551676	0.002087365
CoA22382_2058	transcript_HQ_CoA_transcript12047/f2p0/2890	90.968	0.937296091	0.000191113
CoA22382_2058	transcript_HQ_CoA_transcript14906/f2p0/2609	89.032	0.974387465	8.63489E-06
CoA22382_2058	transcript_HQ_CoA_transcript5299/f2p0/3825	87.097	0.817545336	0.007110811
CoA8255_3338	transcript_HQ_CoA_transcript12045/f2p0/2878	92.355	0.838726286	0.004719279
CoA8255_3338	transcript_HQ_CoA_transcript7163/f2p0/3482	95.602	0.859514064	0.002974364

CoA14161_2687	transcript_HQ_CoA_transcript8854/f2p0/3254	97.286	0.802598881	0.009222149
CoA17271_2457	transcript_HQ_CoA_transcript22060/f4p0/2012	88.934	0.94923397	9.23445E-05
CoA17271_2457	transcript_HQ_CoA_transcript22866/f2p0/1997	86.047	0.893715527	0.001159925
CoA17271_2457	transcript_HQ_CoA_transcript22069/f2p0/2067	84.8	0.889337719	0.001330029
CoA17271_2457	transcript_HQ_CoA_transcript20733/f2p0/2158	82.129	0.93458792	0.000221
CoA11906_2889	transcript_HQ_CoA_transcript12767/f2p0/2815	97.777	0.95197806	7.62295E-05
CoA11906_2889	transcript_HQ_CoA_transcript7163/f2p0/3482	94.605	0.862615055	0.002759585
CoA5701_3740	transcript_HQ_CoA_transcript4137/f2p0/4095	93.694	0.878449561	0.001826898
CoA5701_3740	transcript_HQ_CoA_transcript16123/f2p0/2507	85.776	0.868039324	0.002410008
CoA20069_2196	transcript_HQ_CoA_transcript12351/f2p0/2918	92.391	0.828173271	0.00582752
CoA26519_1736	transcript_HQ_CoA_transcript8982/f2p0/3111	95.763	0.80938637	0.008217234
CoA26519_1736	transcript_HQ_CoA_transcript18258/f2p0/2330	94.451	0.956819286	5.28052E-05
CoA26519_1736	transcript_HQ_CoA_transcript14205/f2p0/2701	98.202	0.870152168	0.002282548
CoA26519_1736	transcript_HQ_CoA_transcript28772/f2p0/1471	94.894	0.853429398	0.003428748
CoA27612_1606	transcript_HQ_CoA_transcript13822/f3p0/2723	87.732	-0.919860005	0.000443279
CoA27612_1606	transcript_HQ_CoA_transcript17886/f3p0/2375	98.291	0.937230914	0.000191797
CoA27612_1606	transcript_HQ_CoA_transcript26606/f2p0/1706	83.459	-0.802401113	0.009252613
CoA27612_1606	transcript_HQ_CoA_transcript13559/f3p0/2764	96	-0.867344101	0.002453
CoA11969_2826	transcript_HQ_CoA_transcript1341/f3p0/5394	92.632	0.921161445	0.000419137
CoA11478_2967	transcript_HQ_CoA_transcript10803/f3p0/3042	84.047	-0.835136698	0.005078412
CoA29174_1417	transcript_HQ_CoA_transcript14349/f2p0/2676	97.959	0.954310788	6.41851E-05
CoA11599_2875	transcript_HQ_CoA_transcript12767/f2p0/2815	92.587	0.849917285	0.003711617
CoA11599_2875	transcript_HQ_CoA_transcript7163/f2p0/3482	92.136	0.869552876	0.002318216
CoA10198_3111	transcript_HQ_CoA_transcript10078/f2p0/3112	91.026	0.819203274	0.006899095
CoA14994_2619	transcript_HQ_CoA_transcript1591/f2p0/5211	79.326	0.934206744	0.000225455
CoA14683_2634	transcript_HQ_CoA_transcript7912/f3p0/3386	91.518	0.92934302	0.00028798
CoA14683_2634	transcript_HQ_CoA_transcript12045/f2p0/2878	92.839	0.924515199	0.000361189
CoA14683_2634	transcript_HQ_CoA_transcript12767/f2p0/2815	92.377	0.862415088	0.002773102
CoA14683_2634	transcript_HQ_CoA_transcript1802/f2p0/5043	92.22	0.837021531	0.004887592
CoA14683_2634	transcript_HQ_CoA_transcript7163/f2p0/3482	92.286	0.8634583	0.002703085
CoA14683_2634	transcript_HQ_CoA_transcript9722/f2p0/3154	97.683	0.960610817	3.84244E-05
CoA14683_2634	transcript_HQ_CoA_transcript8818/f2p0/3263	96.518	0.859234598	0.002994268
CoA10727_3016	transcript_HQ_CoA_transcript7912/f3p0/3386	96.197	0.875497612	0.001981001
CoA10727_3016	transcript_HQ_CoA_transcript1802/f2p0/5043	98.19	0.897809444	0.001015123
CoA10727_3016	transcript_HQ_CoA_transcript12045/f2p0/2878	95.588	0.87073359	0.002248308
CoA10727_3016	transcript_HQ_CoA_transcript9722/f2p0/3154	97.67	0.963074024	3.07258E-05
CoA10727_3016	transcript_HQ_CoA_transcript8818/f2p0/3263	96.498	0.896586211	0.001056986
CoA27626_1626	transcript_HQ_CoA_transcript28311/f2p0/1558	95.455	0.857403574	0.00312695
CoA27626_1626	transcript_HQ_CoA_transcript20086/f2p0/2205	90.4	-0.821727574	0.00658504
CoA9012_3232	transcript_HQ_CoA_transcript7163/f2p0/3482	94.16	0.817368515	0.007133648

CoA9012_3232	transcript_HQ_CoA_transcript12767/f2p0/2815	90.774	0.940823487	0.000156605
CoA20948_2138	transcript_HQ_CoA_transcript22060/f4p0/2012	82.129	0.93458792	0.000221
CoA20948_2138	transcript_HQ_CoA_transcript14906/f2p0/2609	95.48	0.917878758	0.000481879
CoA20948_2138	transcript_HQ_CoA_transcript12047/f2p0/2890	94.906	0.927179066	0.000319359
CoA20948_2138	transcript_HQ_CoA_transcript5761/f2p0/3739	94.074	0.902854206	0.000854621
CoA20948_2138	transcript_HQ_CoA_transcript21282/f3p0/2135	91.011	-0.80540783	0.008796748
CoA20948_2138	transcript_HQ_CoA_transcript13012/f2p0/2744	76.744	-0.834512044	0.005142758
CoA29402_1347	transcript_HQ_CoA_transcript24425/f2p0/1891	94.531	0.939311979	0.000170801
CoA12817_2807	transcript_HQ_CoA_transcript9302/f5p0/3191	95.553	-0.806646014	0.008613525
CoA12817_2807	transcript_HQ_CoA_transcript13577/f2p0/2738	94.836	0.876373462	0.001934375
CoA12874_2812	transcript_HQ_CoA_transcript12047/f2p0/2890	98.297	0.946070127	0.000113749
CoA12874_2812	transcript_HQ_CoA_transcript5299/f2p0/3825	94.081	0.899477659	0.00095991
CoA12874_2812	transcript_HQ_CoA_transcript14906/f2p0/2609	94.238	0.961245929	3.6322E-05
CoA12874_2812	transcript_HQ_CoA_transcript5761/f2p0/3739	94.574	0.82481677	0.006214119
CoA7918_3371	transcript_HQ_CoA_transcript11684/f12p0/2902	92.493	0.870607217	0.00225572
CoA26792_1692	transcript_HQ_CoA_transcript7811/f8p0/3376	95.96	0.830204536	0.005601712
CoA11614_2938	transcript_HQ_CoA_transcript4488/f2p0/4020	90.566	0.951445972	7.91852E-05
CoA11614_2938	transcript_HQ_CoA_transcript6260/f2p0/3632	93.617	-0.802330826	0.009263456
CoA9691_3155	transcript_HQ_CoA_transcript12371/f3p0/2846	91.554	0.923574526	0.000376836
CoA9691_3155	transcript_HQ_CoA_transcript25127/f3p0/1802	91.386	0.851183743	0.003607837
CoA13009_2807	transcript_HQ_CoA_transcript12047/f2p0/2890	98.898	0.985298008	1.25063E-06
CoA13009_2807	transcript_HQ_CoA_transcript5299/f2p0/3825	94.704	0.810492128	0.00806089
CoA13009_2807	transcript_HQ_CoA_transcript14906/f2p0/2609	94.87	0.930855382	0.00026738
CoA13009_2807	transcript_HQ_CoA_transcript5761/f2p0/3739	96.124	0.905719382	0.000771828
CoA11954_2888	transcript_HQ_CoA_transcript9722/f2p0/3154	96.208	0.838815176	0.004710613
CoA11954_2888	transcript_HQ_CoA_transcript12767/f2p0/2815	95.164	0.959946077	4.07155E-05
CoA11954_2888	transcript_HQ_CoA_transcript7163/f2p0/3482	90.72	0.880598752	0.001720068
CoA11954_2888	transcript_HQ_CoA_transcript26464/f4p0/1727	93.496	0.844156492	0.004209628
CoA24164_1895	transcript_HQ_CoA_transcript26646/f4p0/1713	94.211	0.85347099	0.00342549
CoA24164_1895	transcript_HQ_CoA_transcript14427/f2p0/2684	95.745	0.818567749	0.006979737
CoA11759_2902	transcript_HQ_CoA_transcript12767/f2p0/2815	94.015	0.869944384	0.002294871
CoA11759_2902	transcript_HQ_CoA_transcript7163/f2p0/3482	93.462	0.886743776	0.001438596
CoA22190_2042	transcript_HQ_CoA_transcript12047/f2p0/2890	97.818	0.905774231	0.0007703
CoA22190_2042	transcript_HQ_CoA_transcript5761/f2p0/3739	93.548	0.862422786	0.002772581
CoA11324_2989	transcript_HQ_CoA_transcript30000/f2p0/1148	98.339	0.989715416	3.59617E-07
CoA11465_2974	transcript_HQ_CoA_transcript9722/f2p0/3154	92.782	0.829897668	0.005635436
CoA11465_2974	transcript_HQ_CoA_transcript8818/f2p0/3263	92.566	0.85597768	0.003233036
CoA11465_2974	transcript_HQ_CoA_transcript4428/f3p0/4085	96.705	0.949889257	8.82967E-05

Supplementary Table S7. The significant GO categories of significificantly expressed lncRNAs target genes

			GO terms		p-value	Num.
biological_process	Up-regulated	CoA2 vs. CoA1	GO:0006071	glycerol metabolic process	0.002307	1
			GO:0019400	alditol metabolic process	0.002307	1
			GO:0006094	gluconeogenesis	0.0050537	1
			GO:0019319	hexose biosynthetic process	0.0050537	1
			GO:0046364	monosaccharide biosynthetic process	0.0050537	1
			GO:0019751	polyol metabolic process	0.0054454	1
			GO:0006066	alcohol metabolic process	0.0058583	1
			GO:1901615	organic hydroxy compound metabolic process	0.006992	1
			GO:0015979	photosynthesis	0.0097506	1
			GO:0006006	glucose metabolic process	0.0097752	1
		CoA3 vs. CoA2	GO:0006071	glycerol metabolic process	0.0018327	1
			GO:0019400	alditol metabolic process	0.0018327	1
			GO:0006094	gluconeogenesis	0.003999	1
			GO:0019319	hexose biosynthetic process	0.003999	1
			GO:0046364	monosaccharide biosynthetic process	0.003999	1
			GO:0019751	polyol metabolic process	0.0042757	1
			GO:0006066	alcohol metabolic process	0.004583	1
			GO:1901615	organic hydroxy compound metabolic process	0.0054739	1
			GO:0006006	glucose metabolic process	0.0076825	1
			GO:0019318	hexose metabolic process	0.017055	1
			GO:0005996	monosaccharide metabolic process	0.017664	1
			GO:0008380	RNA splicing	0.019934	1
		CoA3 vs. CoA1	GO:0015979	photosynthesis	0.0021107	1
	Down-regulated	CoA2 vs. CoA1	GO:0007030	Golgi organization	0.00031829	2
			GO:0010256	endomembrane system organization	0.00072498	2
			GO:0010506	regulation of autophagy	0.0010536	2

	GO:0031329	regulation of cellular catabolic process	0.0016205	2
	GO:0009894	regulation of catabolic process	0.0018333	2
	GO:0048870	cell motility	0.002685	2
	GO:0051674	localization of cell	0.002685	2
	GO:0040011	locomotion	0.0042384	2
	GO:0044248	cellular catabolic process	0.0069675	3
	GO:0006751	glutathione catabolic process	0.0069757	1
	GO:0043171	peptide catabolic process	0.0069757	1
	GO:0006749	glutathione metabolic process	0.008947	1
CoA3 vs. CoA2	GO:0007030	Golgi organization	0.000060702	2
	GO:0010256	endomembrane system organization	0.00014328	2
	GO:0010506	regulation of autophagy	0.0002178	2
	GO:0031329	regulation of cellular catabolic process	0.0003324	2
	GO:0009894	regulation of catabolic process	0.0003771	2
	GO:0048870	cell motility	0.00054285	2
	GO:0051674	localization of cell	0.00054285	2
	GO:0040011	locomotion	0.00086816	2
	GO:0016032	viral process	0.0026282	2
	GO:0006914	autophagy	0.0028874	2
	GO:0044764	multi-organism cellular process	0.0030531	2
	GO:0044403	symbiosis, encompassing mutualism through parasitism	0.0032207	2
	GO:0044419	interspecies interaction between organisms	0.0032865	2
	GO:0006928	movement of cell or subcellular component	0.0033255	2
CoA3 vs. CoA1	GO:0006751	glutathione catabolic process	0.004273	1
	GO:0043171	peptide catabolic process	0.004273	1
	GO:0006749	glutathione metabolic process	0.0053595	1

			GO:0042219	cellular modified amino acid catabolic process	0.0069377	1
			GO:0044273	sulfur compound catabolic process	0.0069377	1
			GO:0006790	sulfur compound metabolic process	0.024257	1
			GO:1901565	organonitrogen compound catabolic process	0.030564	1
			GO:0006575	cellular modified amino acid metabolic process	0.030793	1
			GO:0006820	anion transport	0.039705	1
molecular_function	Up-regulated	CoA2 vs. CoA1	GO:0019203	carbohydrate phosphatase a	0.0012043	1
			GO:0042132	fructose 1,6-bisphosphate 1	0.0012043	1
			GO:0050308	sugar-phosphatase activity	0.0012043	1
			GO:0019843	rRNA binding	0.0045194	1
		CoA3 vs. CoA2	GO:0019203	carbohydrate phosphatase a	0.00092461	1
			GO:0042132	fructose 1,6-bisphosphate 1	0.00092461	1
			GO:0050308	sugar-phosphatase activity	0.00092461	1
			GO:0019843	rRNA binding	0.0033859	1
			GO:0045182	translation regulator activit	0.027169	1
			GO:0016705	oxidoreductase activity, act	0.028464	1
			GO:0020037	heme binding	0.030114	1
		CoA3 vs. CoA1	GO:0005215	transporter activity	0.049857	1
	Down-regulated	CoA2 vs. CoA1	GO:0004476	mannose-6-phosphate isomerase activity	0.002932	1
			GO:0036374	glutathione hydrolase activity	0.0069757	1
		CoA3 vs. CoA2	None		None	None
		CoA3 vs. CoA1	GO:0036374	glutathione hydrolase activity	0.004273	1
			GO:0008242	omega peptidase activity	0.0060311	1
			GO:0046983	protein dimerization activity	0.0096273	2
			GO:0070011	peptidase activity, acting on L-amino acid peptides	0.025015	2
			GO:0008233	peptidase activity	0.026611	2
			GO:0008238	exopeptidase activity	0.04127	1
			GO:0004252	serine-type endopeptidase activity	0.046177	1
			GO:0051082	unfolded protein binding	0.046518	1

cellular_component	Up-regulated	CoA2 vs. CoA1	GO:0009539	photosystem II reaction center	0.0020957	1
			GO:0009523	photosystem II	0.0046278	1
			GO:0009521	photosystem	0.0067412	1
			GO:0034357	photosynthetic membrane	0.0075084	1
			GO:0009579	thylakoid	0.0082761	1
			GO:0044436	thylakoid part	0.0082761	1
		CoA3 vs. CoA2	GO:0005681	spliceosomal complex	0.012119	1
		CoA3 vs. CoA1	GO:0009539	photosystem II reaction center	0.00045923	1
			GO:0009523	photosystem II	0.0010266	1
			GO:0009521	photosystem	0.0014878	1
			GO:0034357	photosynthetic membrane	0.0016639	1
			GO:0009579	thylakoid	0.0018346	1
			GO:0044436	thylakoid part	0.0018346	1
			GO:0098796	membrane protein	0.033396	1
	Down-regulated	CoA2 vs. CoA1	GO:0005929	cilium	0.0013297	2
			GO:0031514	motile cilium	0.0013297	2
			GO:0005615	extracellular space	0.0015992	2
			GO:0042995	cell projection	0.0024924	2
			GO:0044421	extracellular region part	0.0051113	2
			GO:0044599	AP-5 adaptor complex	0.0079669	1
		CoA3 vs. CoA2	GO:0005929	cilium	0.00026797	2
			GO:0031514	motile cilium	0.00026797	2
			GO:0005615	extracellular space	0.00031359	2
			GO:0042995	cell projection	0.00050662	2
			GO:0044421	extracellular region part	0.0010352	2
			GO:0005576	extracellular region	0.0039044	2
		CoA3 vs. CoA1	GO:0044599	AP-5 adaptor complex	0.0045175	1
			GO:0030119	AP-type membrane coat adaptor complex	0.023252	1

Supplementary Table S8. Significant KEGG categories of differentially accumulating lncRNA-target genes

		KEGG terms		p-value	Num.
Up-regulated	CoA2 vs. CoA1	ko03010	Ribosome	9.92266E-05	3
		ko02010	ABC transporters	0.031863386	1
		ko00053	Ascorbate and aldarate metabolism	0.031863386	1
	CoA3 vs. CoA2	ko03010	Ribosome	9.92266E-05	1
		ko02010	ABC transporters	0.031863386	1
		ko00053	Ascorbate and aldarate metabolism	0.031863386	1
	CoA3 vs. CoA1	None		None	None
Down-regulated	CoA2 vs. CoA1	ko00051	Fructose and mannose metabolism	0.034990908	2
		ko00520	Amino sugar and nucleotide sugar metabolism	0.085612956	2
		ko03020	RNA polymerase	0.154713062	1
		ko04145	Phagosome	0.308678745	1
		ko00240	Pyrimidine metabolism	0.308678745	1
		ko04120	Ubiquitin mediated	0.309890329	1
		ko00230	Purine metabolism	0.309890329	1
		ko00500	Starch and sucrose	0.312141962	1
		ko04141	Protein processing in endoplasmic reticulum	0.379572171	1
	CoA3 vs. CoA2	ko04120	Ubiquitin mediated	0.057413999	1
		ko00500	Starch and sucrose	0.057413999	1
	CoA3 vs. CoA1	ko03020	RNA polymerase	0.167652988	1
		ko04145	Phagosome	0.167652988	1
		ko00240	Pyrimidine metabolism	0.167652988	1
		ko04120	Ubiquitin mediated proteolysis	0.167652988	1
		ko00230	Purine metabolism	0.167652988	1
		ko04141	Protein processing in endoplas	0.229311004	1

Supplementary Table S9. Target genes of differentially expressed lncRNAs involved in floral bud development

LncRNA ID	Trans targets ID	Blast-Sore	Correlation-coefficient	p-value	Trans targets name	Functional annotation of target gene	References
CoA7994_3361	transcript_HQ_CoA_transcript12045/f2p0/2878	92.181	0.83789505	0.0048008	*MIEL1*	inflorescence stems development	
CoA8255_3338	transcript_HQ_CoA_transcript12045/f2p0/2878	92.355	0.838726286	0.0047193	*MIEL1*		
CoA14683_2634	transcript_HQ_CoA_transcript12045/f2p0/2878	92.839	0.924515199	0.0003612	*MIEL1*		
CoA10727_3016	transcript_HQ_CoA_transcript12045/f2p0/2878	95.588	0.87073359	0.0022483	*MIEL1*		
CoA8777_3260	transcript_HQ_CoA_transcript14234/f4p0/2651	88.806	0.830802717	0.0055364	*VTC2*	flowering time control	[55]
CoA11969_2826	transcript_HQ_CoA_transcript1341/f3p0/5394	92.632	0.921161445	0.0004191	*HAT*	flowering time control	
CoA7994_3361	transcript_HQ_CoA_transcript1802/f2p0/5043	93.78	0.823620065	0.0063561	*WAK2*	cell wall biogenesis	[58]
CoA14683_2634	transcript_HQ_CoA_transcript1802/f2p0/5043	92.22	0.837021531	0.0048876	*WAK2*		
CoA10727_3016	transcript_HQ_CoA_transcript1802/f2p0/5043	98.19	0.897809444	0.0010151	*WAK2*		
CoA18663_2328	transcript_HQ_CoA_transcript1802/f2p0/5043	92.476	0.914503413	*0.000553*	*WAK2*		
CoA18663_2328	transcript_HQ_CoA_transcript23484/f3p0/1962	88.93	0.993798205	*6.148E-08*	*AXR1*	DNA repair and meiosis	[74]
CoA18663_2328	transcript_HQ_CoA_transcript3229/f6p0/4345	89.899	0.898120116	*0.0010047*	*DCL3b*	small RNA biogenesis	[75]
CoA3445_4292	transcript_HQ_CoA_transcript22060/f4p0/2012	88.934	0.91833073	0.0004729	*PMI1*	cell wall biogenesis	[57]
CoA17271_2457	transcript_HQ_CoA_transcript22060/f4p0/2012	88.934	0.94923397	9.234E-05	*PMI1*		
CoA20948_2138	transcript_HQ_CoA_transcript22060/f4p0/2012	82.129	0.93458792	0.000221	*PMI1*		

Supplementary Table S10. Analysis of target genes for differentially accumulated miRNAs.

miRNA	Target gene	Expectation	UPE$	miRNA start	miRNA end	Target start	Target end	miRNA_aligned_fragment	Target_aligned_fragment	Inhibition	correlation_coefficient	p-value
ath-miR171a-3p	transcript_HQ_CoA_transcript13222/f2p0/2780	0	-1	1	21	1356	1376	UGAUUGAGCCGCGCCAAUAUC	GAUAUUGGCGCGGCUCAAUCA	Cleavage		
ath-miR171a-3p	transcript_HQ_CoA_transcript14170/f2p0/2693	0	-1	1	21	1423	1443	UGAUUGAGCCGCGCCAAUAUC	GAUAUUGGCGCGGCUCAAUCA	Cleavage	-0.904541037	0.000805166
ath-miR171a-3p	transcript_HQ_CoA_transcript13973/f3p0/2691	0	-1	1	21	1526	1546	UGAUUGAGCCGCGCCAAUAUC	GAUAUUGGCGCGGCUCAAUCA	Cleavage	-0.781192095	0.012929096
ath-miR171a-3p	transcript_HQ_CoA_transcript15446/f3p0/2572	0	-1	1	21	1297	1317	UGAUUGAGCCGCGCCAAUAUC	GAUAUUGGCGCGGCUCAAUCA	Cleavage	-0.768245864	0.015595598
osa-miR408-3p	transcript_HQ_CoA_transcript30249/f5p0/748	0	-1	1	21	117	137	CUGCACUGCCUCUUCCCUGGC	CUCAGGGAAGAGGCAGUGCAG	Cleavage		
zma-miR171a-3p	transcript_HQ_CoA_transcript13222/f2p0/2780	0	-1	1	20	1357	1376	UGAUUGAGCCGCGCCAAUAU	AUAUUGGCGCGGCUCAAUCA	Cleavage		
zma-miR171a-3p	transcript_HQ_CoA_transcript14170/f2p0/2693	0	-1	1	20	1424	1443	UGAUUGAGCCGCGCCAAUAU	AUAUUGGCGCGGCUCAAUCA	Cleavage	-0.904541037	0.000805166
zma-miR171a-3p	transcript_HQ_CoA_transcript13973/f3p0/2691	0	-1	1	20	1527	1546	UGAUUGAGCCGCGCCAAUAU	AUAUUGGCGCGGCUCAAUCA	Cleavage	-0.781192095	0.012929096
zma-miR171a-3p	transcript_HQ_CoA_transcript15446/f3p0/2572	0	-1	1	20	1298	1317	UGAUUGAGCCGCGCCAAUAU	AUAUUGGCGCGGCUCAAUCA	Cleavage	-0.768245864	0.015595598
aof-miR160b	transcript_HQ_CoA_transcript14581/f3p0/2607	0.5	-1	1	21	1689	1709	UGCCUGGUUCCCUGUAUGCCA	AGGCAUACAGGGAGCCAGGCA	Cleavage		
aof-miR160b	transcript_HQ_CoA_transcript12617/f2p0/2825	0.5	-1	1	21	1874	1894	UGCCUGGUUCCCUGUAUGCCA	AGGCAUACAGGGAGCCAGGCA	Cleavage		
aof-miR160b	transcript_HQ_CoA_transcript10120/f2p0/3101	0.5	-1	1	21	2061	2081	UGCCUGGUUCCCUGUAUGCCA	AGGCAUACAGGGAGCCAGGCA	Cleavage		
aof-miR160b	transcript_HQ_CoA_transcript10142/f2p0/3146	0.5	-1	1	21	2120	2140	UGCCUGGUUCCCUGUAUGCCA	AGGCAUACAGGGAGCCAGGCA	Cleavage		
gma-miR408d	transcript_HQ_CoA_transcript30249/f5p0/748	0.5	-1	1	20	117	136	UGCACUGCCUCUUCCCUGGC	CUCAGGGAAGAGGCAGUGCA	Cleavage		
ppt-miR408b	transcript_HQ_CoA_transcript30249/f5p0/748	0.5	-1	1	21	116	136	UGCACUGCCUCUUCCCUGGCU	UCUCAGGGAAGAGGCAGUGCA	Cleavage		
aof-miR160b	transcript_HQ_CoA_transcript18573/f2p0/2333	1	-1	1	21	1417	1437	UGCCUGGUUCCCUGUAUGCCA	UGGCAUGCAGGGAGCCAGGCA	Cleavage		
aof-miR160b	transcript_HQ_CoA_transcript8081/f2p0/3349	1	-1	1	21	2540	2560	UGCCUGGUUCCCUGUAUGCCA	UGGCAUGCAGGGAGCCAGGCA	Cleavage		
ata-miR408-3p	transcript_HQ_CoA_transcript30249/f5p0/748	1	-1	1	20	117	136	UGCACUGCCUCUUCCCUGCC	CUCAGGGAAGAGGCAGUGCA	Cleavage		
ath-miR171a-3p	transcript_HQ_CoA_transcript24326/f2p0/1895	1	-1	1	21	679	699	UGAUUGAGCCGCGCCAAUAUC	GAUUUUGGCGCGGCUCAAUCA	Cleavage		
ath-miR394a	transcript_HQ_CoA_transcript26135/f7p0/1748	1	-1	1	20	1290	1309	UUGGCAUUCUGUCCACCUCC	GGAGGUUGACAGAAUGCCAA	Cleavage		
ath-miR394a	transcript_HQ_CoA_transcript26424/f2p0/1735	1	-1	1	20	1217	1236	UUGGCAUUCUGUCCACCUCC	GGAGGUUGACAGAAUGCCAA	Cleavage	0.763298541	0.016704391
ath-miR408-3p	transcript_HQ_CoA_transcript30249/f5p0/748	1	-1	1	21	117	137	AUGCACUGCCUCUUCCCUGGC	CUCAGGGAAGAGGCAGUGCAG	Cleavage		
cme-miR166i	transcript_HQ_CoA_transcript7441/f2p0/3447	1	-1	1	20	1179	1198	UCGGACCAGGCUUCAUUCUC	UGGAAUGAAGCCUGGUCCGG	Cleavage		
cme-miR166i	transcript_HQ_CoA_transcript7456/f3p0/3462	1	-1	1	20	1183	1202	UCGGACCAGGCUUCAUUCUC	UGGAAUGAAGCCUGGUCCGG	Cleavage		
cme-miR166i	transcript_HQ_CoA_transcript5511/f3p0/3782	1	-1	1	20	1534	1553	UCGGACCAGGCUUCAUUCUC	UGGAAUGAAGCCUGGUCCGG	Cleavage	0.861062864	0.002865706
cme-miR166i	transcript_HQ_CoA_transcript5155/f2p0/3869	1	-1	1	20	1683	1702	UCGGACCAGGCUUCAUUCUC	CGGAAUGAAGCCUGGUCCGG	Cleavage		
cme-miR166i	transcript_HQ_CoA_transcript4140/f2p0/4159	1	-1	1	20	1505	1524	UCGGACCAGGCUUCAUUCUC	CGGAAUGAAGCCUGGUCCGG	Cleavage		
cme-miR166i	transcript_HQ_CoA_transcript3222/f2p0/4363	1	-1	1	20	1467	1486	UCGGACCAGGCUUCAUUCUC	UGGAAUGAAGCCUGGUCCGG	Cleavage		
cme-miR166i	transcript_HQ_CoA_transcript1941/f3p0/4960	1	-1	1	20	1275	1294	UCGGACCAGGCUUCAUUCUC	UGGAAUGAAGCCUGGUCCGG	Cleavage		
zma-miR171a-3p	transcript_HQ_CoA_transcript24326/f2p0/1895	1	-1	1	20	680	699	UGAUUGAGCCGCGCCAAUAU	AUUUUGGCGCGGCUCAAUCA	Cleavage		
ath-miR157a-5p	transcript_HQ_CoA_transcript21959/f2p0/2080	1.5	-1	1	21	1520	1540	UUGACAGAAGAUAGAGAGCAC	GUGCUCUCUCUCUUCUGUCAA	Cleavage	-0.857733243	0.003102769
ath-miR157a-5p	transcript_HQ_CoA_transcript27667/f3p0/1636	1.5	-1	1	21	832	852	UUGACAGAAGAUAGAGAGCAC	GUGCUCUCUCUCUUCUGUCAA	Cleavage	-0.79979813	0.009659939
ath-miR157a-5p	transcript_HQ_CoA_transcript6872/f2p0/3521	1.5	-1	1	21	3276	3296	UUGACAGAAGAUAGAGAGCAC	UAGCUUUCUGUUUUCUGUCAA	Cleavage		
ath-miR171a-3p	transcript_HQ_CoA_transcript23849/f2p0/1925	1.5	-1	1	21	758	778	UGAUUGAGCCGCGCCAAUAUC	GAUAUUGGCGCGGCUCAACCA	Cleavage		
cme-miR166i	transcript_HQ_CoA_transcript10643/f2p0/3040	1.5	-1	1	20	861	880	UCGGACCAGGCUUCAUUCUC	UGGGAUGAAGCCUGGUCCGG	Cleavage		
cme-miR166i	transcript_HQ_CoA_transcript10654/f2p0/3061	1.5	-1	1	20	849	868	UCGGACCAGGCUUCAUUCUC	UGGGAUGAAGCCUGGUCCGG	Cleavage		
cme-miR166i	transcript_HQ_CoA_transcript10287/f3p0/3067	1.5	-1	1	20	872	891	UCGGACCAGGCUUCAUUCUC	UGGGAUGAAGCCUGGUCCGG	Cleavage		
cme-miR166i	transcript_HQ_CoA_transcript10126/f4p0/3087	1.5	-1	1	20	814	833	UCGGACCAGGCUUCAUUCUC	UGGGAUGAAGCCUGGUCCGG	Cleavage		
cme-miR166i	transcript_HQ_CoA_transcript9864/f2p0/3129	1.5	-1	1	20	978	997	UCGGACCAGGCUUCAUUCUC	UGGGAUGAAGCCUGGUCCGG	Cleavage		
cme-miR166i	transcript_HQ_CoA_transcript9618/f2p0/3165	1.5	-1	1	20	842	861	UCGGACCAGGCUUCAUUCUC	UGGGAUGAAGCCUGGUCCGG	Cleavage		
cme-miR166i	transcript_HQ_CoA_transcript8673/f9p0/3245	1.5	-1	1	20	1100	1119	UCGGACCAGGCUUCAUUCUC	UGGGAUGAAGCCUGGUCCGG	Cleavage		
cme-miR166i	transcript_HQ_CoA_transcript8032/f3p0/3377	1.5	-1	1	20	1213	1232	UCGGACCAGGCUUCAUUCUC	UGGGAUGAAGCCUGGUCCGG	Cleavage	0.887414029	0.001409975
cme-miR166i	transcript_HQ_CoA_transcript6432/f3p0/3616	1.5	-1	1	20	1440	1459	UCGGACCAGGCUUCAUUCUC	UGGGAUGAAGCCUGGUCCGG	Cleavage		
gma-miR408d	transcript_HQ_CoA_transcript12663/f3p0/2798	1.5	-1	1	20	1030	1049	UGCACUGCCUCUUCCCUGGC	ACCAGGGAAGAUGCAGUGCA	Cleavage		
gma-miR408d	transcript_HQ_CoA_transcript13253/f2p0/2781	1.5	-1	1	20	1072	1091	UGCACUGCCUCUUCCCUGGC	ACCAGGGAAGAUGCAGUGCA	Cleavage	0.903924578	0.000823002
gma-miR408d	transcript_HQ_CoA_transcript14475/f2p0/2651	1.5	-1	1	20	956	975	UGCACUGCCUCUUCCCUGGC	ACCAGGGAAGAUGCAGUGCA	Cleavage		
hbr-miR156	transcript_HQ_CoA_transcript6872/f2p0/3521	1.5	-1	1	19	3278	3296	UUGACAGAAGAUAGAGAGC	GCUUUCUGUUUUCUGUCAA	Cleavage		
hbr-miR156	transcript_HQ_CoA_transcript21959/f2p0/2080	1.5	-1	1	19	1522	1540	UUGACAGAAGAUAGAGAGC	GCUCUCUCUCUUCUGUCAA	Cleavage	-0.857733243	0.003102769
hbr-miR156	transcript_HQ_CoA_transcript27667/f3p0/1636	1.5	-1	1	19	834	852	UUGACAGAAGAUAGAGAGC	GCUCUCUCUCUUCUGUCAA	Cleavage	-0.79979813	0.009659939
ppt-miR408b	transcript_HQ_CoA_transcript12663/f3p0/2798	1.5	-1	1	21	1029	1049	UGCACUGCCUCUUCCCUGGCU	CACCAGGGAAGAUGCAGUGCA	Cleavage		
ppt-miR408b	transcript_HQ_CoA_transcript13253/f2p0/2781	1.5	-1	1	21	1071	1091	UGCACUGCCUCUUCCCUGGCU	CACCAGGGAAGAUGCAGUGCA	Cleavage	0.904046981	0.000819439
ppt-miR408b	transcript_HQ_CoA_transcript14475/f2p0/2651	1.5	-1	1	21	955	975	UGCACUGCCUCUUCCCUGGCU	CACCAGGGAAGAUGCAGUGCA	Cleavage		
zma-miR171a-3p	transcript_HQ_CoA_transcript23849/f2p0/1925	1.5	-1	1	20	759	778	UGAUUGAGCCGCGCCAAUAU	AUAUUGGCGCGGCUCAACCA	Cleavage		
ath-miR159a	transcript_HQ_CoA_transcript10405/f2p0/3066	2	-1	1	21	1788	1808	UUUGGAUUGAAGGGAGCUCUA	CAGAGUUUCCUUUGAUCCAAA	Cleavage		
ath-miR159a	transcript_HQ_CoA_transcript17064/f2p0/2430	2	-1	1	21	84	104	UUUGGAUUGAAGGGAGCUCUA	UUGAGCUCCUUGCAAUCCAAA	Translation		
ath-miR159a	transcript_HQ_CoA_transcript17441/f2p0/2445	2	-1	1	21	27	47	UUUGGAUUGAAGGGAGCUCUA	UUGAGCUCCUUGCAAUCCAAA	Translation		
ath-miR159c	transcript_HQ_CoA_transcript17064/f2p0/2430	2	-1	1	21	84	104	UUUGGAUUGAAGGGAGCUCCU	UUGAGCUCCUUGCAAUCCAAA	Translation		
ath-miR159c	transcript_HQ_CoA_transcript17441/f2p0/2445	2	-1	1	21	27	47	UUUGGAUUGAAGGGAGCUCCU	UUGAGCUCCUUGCAAUCCAAA	Translation		
ath-miR159c	transcript_HQ_CoA_transcript10405/f2p0/3066	2	-1	1	21	1788	1808	UUUGGAUUGAAGGGAGCUCCU	CAGAGUUUCCUUUGAUCCAAA	Cleavage		
ath-miR396a-5p	transcript_HQ_CoA_transcript14572/f4p0/2629	2	-1	1	21	2337	2357	UUCCACAGCUUUCUUGAACUG	AAGUCUAAGGAAGCUGUGGAA	Cleavage	-0.758396231	0.017853706
ath-miR396b-5p	transcript_HQ_CoA_transcript14572/f4p0/2629	2	-1	1	21	2337	2357	UUCCACAGCUUUCUUGAACUU	AAGUCUAAGGAAGCUGUGGAA	Cleavage	-0.76198851	0.017006544
gma-miR396h	transcript_HQ_CoA_transcript14572/f4p0/2629	2	-1	1	20	2337	2356	UCCACAGCUUUCUUGAACUG	AAGUCUAAGGAAGCUGUGGA	Cleavage	-0.758396231	0.017853706
gma-miR396h	transcript_HQ_CoA_transcript976/f3p0/5771	2	-1	1	20	3270	3289	UCCACAGCUUUCUUGAACUG	GACUCCAAGAAAGCUGUGGA	Cleavage		
gma-miR396h	transcript_HQ_CoA_transcript1778/f2p0/5116	2	-1	1	20	275	294	UCCACAGCUUUCUUGAACUG	GACUCCAAGAAAGCUGUGGA	Cleavage		

osa-miR159f	transcript_HQ_CoA_transcript6229/f2p0/3655	2	-1	1	21	462	482	CUUGGAUUGAAGGGAGCUCUA	CGAAGCACCCUUCAAUCCAAG	Cleavage		
osa-miR159f	transcript_HQ_CoA_transcript5978/f3p0/3699	2	-1	1	21	462	482	CUUGGAUUGAAGGGAGCUCUA	CGAAGCACCCUUCAAUCCAAG	Cleavage		
tae-miR395b	transcript_HQ_CoA_transcript27318/f2p0/1637	2	-1	1	20	369	388	UGAAGUGUUUGGGGGAACUC	GAGUUCCUCCAAACUCUUCA	Cleavage		
tae-miR395b	transcript_HQ_CoA_transcript26410/f2p0/1754	2	-1	1	20	416	435	UGAAGUGUUUGGGGGAACUC	GAGUUCCUCCAAACUCUUCA	Cleavage		
vvi-miR396a	transcript_HQ_CoA_transcript14572/f4p0/2629	2	-1	1	21	2337	2357	UUCCACAGCUUUCUUGAACUA	AAGUCUAAGGAAGCUGUGGAA	Cleavage	-0.766463001	0.015989326
vvi-miR396b	transcript_HQ_CoA_transcript14572/f4p0/2629	2	-1	1	20	2338	2357	UUCCACAGCUUUCUUGAACU	AGUCUAAGGAAGCUGUGGAA	Cleavage	-0.761357036	0.017153482
ata-miR408-3p	transcript_HQ_CoA_transcript12663/f3p0/2798	2.5	-1	1	20	1030	1049	UGCACUGCCUCUUCCCUGCC	ACCAGGGAAGAUGCAGUGCA	Cleavage		
ata-miR408-3p	transcript_HQ_CoA_transcript13253/f2p0/2781	2.5	-1	1	20	1072	1091	UGCACUGCCUCUUCCCUGCC	ACCAGGGAAGAUGCAGUGCA	Cleavage	0.883194745	0.001596898
ata-miR408-3p	transcript_HQ_CoA_transcript14475/f2p0/2651	2.5	-1	1	20	956	975	UGCACUGCCUCUUCCCUGCC	ACCAGGGAAGAUGCAGUGCA	Cleavage		
ath-miR157a-5p	transcript_HQ_CoA_transcript11812/f2p0/2892	2.5	-1	1	21	2467	2487	UUGACAGAAGAUAGAGAGCAC	GUGCUCUCUCUCUUCUGUCAU	Cleavage	-0.772082878	0.014770298
ath-miR157a-5p	transcript_HQ_CoA_transcript13408/f2p0/2769	2.5	-1	1	21	1931	1951	UUGACAGAAGAUAGAGAGCAC	GUGCUCUCUCUCUUCUGUCAU	Cleavage	-0.88200172	0.001652715
ath-miR157a-5p	transcript_HQ_CoA_transcript14160/f2p0/2685	2.5	-1	1	21	1926	1946	UUGACAGAAGAUAGAGAGCAC	GUGCUCUCUCUCUUCUGUCAU	Cleavage	-0.970059945	1.48495E-05
ath-miR157a-5p	transcript_HQ_CoA_transcript27850/f2p0/1604	2.5	-1	1	21	1133	1153	UUGACAGAAGAUAGAGAGCAC	UUGCUCUCUCUCUUCUGUCAU	Cleavage	-0.872670387	0.00213681
ath-miR157a-5p	transcript_HQ_CoA_transcript15947/f3p0/2445	2.5	-1	1	21	2176	2196	UUGACAGAAGAUAGAGAGCAC	UUUCUUUCUGUUUUCUGUCAA	Cleavage	-0.89212371	0.001219909
ath-miR157a-5p	transcript_HQ_CoA_transcript15965/f4p0/2528	2.5	-1	1	21	34	54	UUGACAGAAGAUAGAGAGCAC	UCUCUCUCUCUCUUCUGUCAA	Cleavage		
ath-miR157a-5p	transcript_HQ_CoA_transcript16448/f2p0/2504	2.5	-1	1	21	41	61	UUGACAGAAGAUAGAGAGCAC	UCUCUCUCUCUCUUCUGUCAA	Cleavage		
ath-miR157a-5p	transcript_HQ_CoA_transcript16946/f3p0/2432	2.5	-1	1	21	3	23	UUGACAGAAGAUAGAGAGCAC	GGUCUCUCUCUCUUCUGUCAA	Cleavage		
ath-miR157a-5p	transcript_HQ_CoA_transcript18005/f9p0/2335	2.5	-1	1	21	49	69	UUGACAGAAGAUAGAGAGCAC	UCUCUCUCUCUCUUCUGUCAA	Cleavage		
ath-miR157a-5p	transcript_HQ_CoA_transcript19070/f2p0/2271	2.5	-1	1	21	9	29	UUGACAGAAGAUAGAGAGCAC	UCUCUCUCUCUCUUCUGUCAA	Cleavage	-0.716464597	0.029886005
ath-miR157a-5p	transcript_HQ_CoA_transcript1215/f2p0/5484	2.5	-1	1	21	4359	4379	UUGACAGAAGAUAGAGAGCAC	GCACUAUCUAUUUUCUGUCAA	Cleavage		
ath-miR157a-5p	transcript_HQ_CoA_transcript6684/f2p0/3554	2.5	-1	1	21	67	87	UUGACAGAAGAUAGAGAGCAC	AAGCUCACUCUCUUCUGUCAA	Cleavage		
ath-miR157a-5p	transcript_HQ_CoA_transcript7286/f2p0/3515	2.5	-1	1	21	65	85	UUGACAGAAGAUAGAGAGCAC	AAGCUCACUCUCUUCUGUCAA	Cleavage		
ath-miR159a	transcript_HQ_CoA_transcript14462/f14p0/2625	2.5	-1	1	21	134	154	UUUGGAUUGAAGGGAGCUCUA	UGGAGUUUCCUUCAAUUCAAC	Cleavage	-0.675003605	0.046057266
ath-miR159a	transcript_HQ_CoA_transcript20055/f2p0/2195	2.5	-1	1	21	1308	1328	UUUGGAUUGAAGGGAGCUCUA	UGGAGCCCCUUCAAACCAAA	Cleavage	-0.730646648	0.02535766
ath-miR159a	transcript_HQ_CoA_transcript6229/f2p0/3655	2.5	-1	1	21	462	482	UUUGGAUUGAAGGGAGCUCUA	CGAAGCACCCUUCAAUCCAAG	Cleavage		
ath-miR159a	transcript_HQ_CoA_transcript5978/f3p0/3699	2.5	-1	1	21	462	482	UUUGGAUUGAAGGGAGCUCUA	CGAAGCACCCUUCAAUCCAAG	Cleavage		
ath-miR159a	transcript_HQ_CoA_transcript4184/f2p0/4063	2.5	-1	1	21	1003	1023	UUUGGAUUGAAGGGAGCUCUA	AUGGGGUCCCUUCAAUUCAGA	Cleavage		
ath-miR159c	transcript_HQ_CoA_transcript14462/f14p0/2625	2.5	-1	1	21	134	154	UUUGGAUUGAAGGGAGCUCCU	UGGAGUUUCCUUCAAUUCAAC	Cleavage		
ath-miR159c	transcript_HQ_CoA_transcript20055/f2p0/2195	2.5	-1	1	21	1308	1328	UUUGGAUUGAAGGGAGCUCCU	UGGAGCCCCUUCAAACCAAA	Cleavage		
ath-miR159c	transcript_HQ_CoA_transcript6229/f2p0/3655	2.5	-1	1	21	462	482	UUUGGAUUGAAGGGAGCUCCU	CGAAGCACCCUUCAAUCCAAG	Cleavage		
ath-miR159c	transcript_HQ_CoA_transcript5978/f3p0/3699	2.5	-1	1	21	462	482	UUUGGAUUGAAGGGAGCUCCU	CGAAGCACCCUUCAAUCCAAG	Cleavage		
ath-miR159c	transcript_HQ_CoA_transcript4184/f2p0/4063	2.5	-1	1	21	1003	1023	UUUGGAUUGAAGGGAGCUCCU	AUGGGGUCCCUUCAAUUCAGA	Cleavage		
ath-miR162a-3p	transcript_HQ_CoA_transcript19045/f3p0/2263	2.5	-1	1	21	866	886	UCGAUAAACCUCUGCAUCCAG	UUGGAUGCAGAGGUUGAUUGG	Cleavage		
ath-miR162a-3p	transcript_HQ_CoA_transcript18770/f2p0/2301	2.5	-1	1	21	977	997	UCGAUAAACCUCUGCAUCCAG	UUGGAUGCAGAGGUUGAUUGG	Cleavage		
ath-miR162a-3p	transcript_HQ_CoA_transcript17171/f4p0/2404	2.5	-1	1	21	995	1015	UCGAUAAACCUCUGCAUCCAG	UUGGAUGCAGAGGUUGAUUGG	Cleavage	-0.889647209	0.001317468
ath-miR162a-3p	transcript_HQ_CoA_transcript11695/f2p0/2917	2.5	-1	1	21	1501	1521	UCGAUAAACCUCUGCAUCCAG	UUGGAUGCAGAGGUUGAUUGG	Cleavage		
ath-miR171a-3p	transcript_HQ_CoA_transcript10152/f2p0/3094	2.5	-1	1	21	2473	2493	UGAUUGAGCCGCGCCAAUAUC	UGAAUUGGCGUGGUUCGAUCA	Cleavage	-0.929117441	0.000291145
ath-miR319a	transcript_HQ_CoA_transcript5815/f6p0/3697	2.5	-1	1	21	646	666	UUGGACUGAAGGGAGCUCCCU	AUGGGCUUCCCUUCAGUUCAA	Cleavage		
ath-miR319a	transcript_HQ_CoA_transcript7017/f3p0/3537	2.5	-1	1	21	541	561	UUGGACUGAAGGGAGCUCCCU	CUGGGCCUCUCUUCAGUUCAA	Cleavage		
ath-miR319a	transcript_HQ_CoA_transcript24806/f3p0/1826	2.5	-1	1	21	1206	1226	UUGGACUGAAGGGAGCUCCCU	UAGGGGGACCCUUCAGUCCAA	Cleavage		
ath-miR319a	transcript_HQ_CoA_transcript23727/f3p0/1944	2.5	-1	1	21	1331	1351	UUGGACUGAAGGGAGCUCCCU	UAGGGGGACCCUUCAGUCCAA	Cleavage		
ath-miR319a	transcript_HQ_CoA_transcript16582/f5p0/2428	2.5	-1	1	21	1872	1892	UUGGACUGAAGGGAGCUCCCU	UAGGGGGACCCUUCAGUCCAA	Cleavage		
ath-miR394a	transcript_HQ_CoA_transcript3338/f2p0/4286	2.5	-1	1	20	295	314	UUGGCAUUCUGUCCACCUCC	AAAGAUGGACAGAAUGCUAA	Cleavage		
ath-miR395a	transcript_HQ_CoA_transcript7095/f3p0/3516	2.5	-1	1	21	2029	2049	CUGAAGUGUUUGGGGGAACUC	UACAUCCCUCAAACACUUCAG	Cleavage		
ath-miR395a	transcript_HQ_CoA_transcript5277/f2p0/3831	2.5	-1	1	21	2339	2359	CUGAAGUGUUUGGGGGAACUC	UACAUCCCUCAAACACUUCAG	Cleavage		
ath-miR396a-5p	transcript_HQ_CoA_transcript7092/f3p0/3560	2.5	-1	1	21	145	165	UUCCACAGCUUUCUUGAACUG	AUGUUCAAGAAGCCUGUGGAG	Cleavage		
ath-miR396a-5p	transcript_HQ_CoA_transcript9321/f2p0/3219	2.5	-1	1	21	30	50	UUCCACAGCUUUCUUGAACUG	AUGUUCAAGAAGCCUGUGGAG	Cleavage		
ath-miR396a-5p	transcript_HQ_CoA_transcript17211/f3p0/2438	2.5	-1	1	21	169	189	UUCCACAGCUUUCUUGAACUG	AUGUUCAAGAAGCCUGUGGAG	Cleavage		
ath-miR396a-5p	transcript_HQ_CoA_transcript20814/f2p0/2176	2.5	-1	1	21	182	202	UUCCACAGCUUUCUUGAACUG	AUGUUCAAGAAGCCUGUGGAG	Cleavage		
ath-miR396a-5p	transcript_HQ_CoA_transcript4547/f5p0/4000	2.5	-1	1	21	194	214	UUCCACAGCUUUCUUGAACUG	GUGUUGAAGGAAGCUGUGGAU	Cleavage		
ath-miR396a-5p	transcript_HQ_CoA_transcript6429/f2p0/3612	2.5	-1	1	21	196	216	UUCCACAGCUUUCUUGAACUG	GUGUUGAAGGAAGCUGUGGAU	Cleavage		
ath-miR396a-5p	transcript_HQ_CoA_transcript7619/f3p0/3429	2.5	-1	1	21	211	231	UUCCACAGCUUUCUUGAACUG	GUGUUGAAGGAAGCUGUGGAU	Cleavage		
ath-miR396a-5p	transcript_HQ_CoA_transcript8209/f2p0/3353	2.5	-1	1	21	290	310	UUCCACAGCUUUCUUGAACUG	GUGUUGAAGGAAGCUGUGGAU	Cleavage		
ath-miR396a-5p	transcript_HQ_CoA_transcript8127/f2p0/3346	2.5	-1	1	21	127	147	UUCCACAGCUUUCUUGAACUG	GUGUUGAAGGAAGCUGUGGAU	Cleavage	-0.736397361	0.023658459
ath-miR396a-5p	transcript_HQ_CoA_transcript8592/f5p0/3264	2.5	-1	1	21	171	191	UUCCACAGCUUUCUUGAACUG	GUGUUGAAGGAAGCUGUGGAU	Cleavage		
ath-miR396b-5p	transcript_HQ_CoA_transcript7092/f3p0/3560	2.5	-1	1	21	145	165	UUCCACAGCUUUCUUGAACUU	AUGUUCAAGAAGCCUGUGGAG	Cleavage		
ath-miR396b-5p	transcript_HQ_CoA_transcript9321/f2p0/3219	2.5	-1	1	21	30	50	UUCCACAGCUUUCUUGAACUU	AUGUUCAAGAAGCCUGUGGAG	Cleavage		
ath-miR396b-5p	transcript_HQ_CoA_transcript17211/f3p0/2438	2.5	-1	1	21	169	189	UUCCACAGCUUUCUUGAACUU	AUGUUCAAGAAGCCUGUGGAG	Cleavage		
ath-miR396b-5p	transcript_HQ_CoA_transcript20814/f2p0/2176	2.5	-1	1	21	182	202	UUCCACAGCUUUCUUGAACUU	AUGUUCAAGAAGCCUGUGGAG	Cleavage		
ath-miR396b-5p	transcript_HQ_CoA_transcript4547/f5p0/4000	2.5	-1	1	21	194	214	UUCCACAGCUUUCUUGAACUU	GUGUUGAAGGAAGCUGUGGAU	Cleavage		
ath-miR396b-5p	transcript_HQ_CoA_transcript6429/f2p0/3612	2.5	-1	1	21	196	216	UUCCACAGCUUUCUUGAACUU	GUGUUGAAGGAAGCUGUGGAU	Cleavage	-0.704924826	0.033937354
ath-miR396b-5p	transcript_HQ_CoA_transcript7619/f3p0/3429	2.5	-1	1	21	211	231	UUCCACAGCUUUCUUGAACUU	GUGUUGAAGGAAGCUGUGGAU	Cleavage		
ath-miR396b-5p	transcript_HQ_CoA_transcript8209/f2p0/3353	2.5	-1	1	21	290	310	UUCCACAGCUUUCUUGAACUU	GUGUUGAAGGAAGCUGUGGAU	Cleavage		
ath-miR396b-5p	transcript_HQ_CoA_transcript8127/f2p0/3346	2.5	-1	1	21	127	147	UUCCACAGCUUUCUUGAACUU	GUGUUGAAGGAAGCUGUGGAU	Cleavage	-0.677529167	0.04494113
ath-miR396b-5p	transcript_HQ_CoA_transcript8592/f5p0/3264	2.5	-1	1	21	171	191	UUCCACAGCUUUCUUGAACUU	GUGUUGAAGGAAGCUGUGGAU	Cleavage		
ath-miR408-3p	transcript_HQ_CoA_transcript12663/f3p0/2798	2.5	-1	1	21	1030	1050	AUGCACUGCCUCUUCCCUGGC	ACCAGGGAAGAUGCAGUGCAA	Translation		

ath-miR408-3p	transcript_HQ_CoA_transcript13253/f2p0/2781	2.5	-1	1	21	1072	1092	AUGCACUGCCUCUUCCCUGGC	ACCAGGGAAGAUGCAGUGCAA	Translation	0.903971246	0.000821642
ath-miR408-3p	transcript_HQ_CoA_transcript14475/f2p0/2651	2.5	-1	1	21	956	976	AUGCACUGCCUCUUCCCUGGC	ACCAGGGAAGAUGCAGUGCAA	Translation		
bra-miR408-5p	transcript_HQ_CoA_transcript24410/f3p0/1881	2.5	-1	1	21	812	832	GGGAGCCAGGGAAGAGGCAGU	AUUUUCUCUUUCCUGGCUCUC	Cleavage		
bra-miR408-5p	transcript_HQ_CoA_transcript270/f2p0/7011	2.5	-1	1	21	1088	1108	GGGAGCCAGGGAAGAGGCAGU	CAUGCCUCUUCUUUGGCUUCA	Cleavage		
bra-miR408-5p	transcript_HQ_CoA_transcript352/f2p0/6917	2.5	-1	1	21	1028	1048	GGGAGCCAGGGAAGAGGCAGU	CAUGCCUCUUCUUUGGCUUCA	Cleavage		
gma-miR167h	transcript_HQ_CoA_transcript16395/f2p0/2497	2.5	-1	1	24	1683	1706	AUCAUGCUGGCAGCUUCAACUGGU	AUUUGUUCAAUCUGCCAGUAUGAU	Cleavage		
gma-miR396h	transcript_HQ_CoA_transcript25598/f3p0/1743	2.5	-1	1	20	688	707	UCCACAGCUUUCUUGAACUG	CAGGUCAAGAAAGCUAUGGA	Cleavage	0.797851965	0.009972274
gma-miR396h	transcript_HQ_CoA_transcript26456/f33p0/1704	2.5	-1	1	20	688	707	UCCACAGCUUUCUUGAACUG	CAGGUCAAGAAAGCUAUGGA	Cleavage		
gma-miR396h	transcript_HQ_CoA_transcript4547/f5p0/4000	2.5	-1	1	20	194	213	UCCACAGCUUUCUUGAACUG	GUGUUGAAGGAAGCUGUGGA	Cleavage		
gma-miR396h	transcript_HQ_CoA_transcript6429/f2p0/3612	2.5	-1	1	20	196	215	UCCACAGCUUUCUUGAACUG	GUGUUGAAGGAAGCUGUGGA	Cleavage		
gma-miR396h	transcript_HQ_CoA_transcript7619/f3p0/3429	2.5	-1	1	20	211	230	UCCACAGCUUUCUUGAACUG	GUGUUGAAGGAAGCUGUGGA	Cleavage		
gma-miR396h	transcript_HQ_CoA_transcript8209/f2p0/3353	2.5	-1	1	20	290	309	UCCACAGCUUUCUUGAACUG	GUGUUGAAGGAAGCUGUGGA	Cleavage		
gma-miR396h	transcript_HQ_CoA_transcript8127/f2p0/3346	2.5	-1	1	20	127	146	UCCACAGCUUUCUUGAACUG	GUGUUGAAGGAAGCUGUGGA	Cleavage	-0.736397361	0.023658459
gma-miR396h	transcript_HQ_CoA_transcript8592/f5p0/3264	2.5	-1	1	20	171	190	UCCACAGCUUUCUUGAACUG	GUGUUGAAGGAAGCUGUGGA	Cleavage		
gma-miR396h	transcript_HQ_CoA_transcript29610/f4p0/1259	2.5	-1	1	20	632	651	UCCACAGCUUUCUUGAACUG	GAGGUCAAGGAGGCUGUGGG	Cleavage		
gma-miR396h	transcript_HQ_CoA_transcript15857/f2p0/2538	2.5	-1	1	20	2365	2384	UCCACAGCUUUCUUGAACUG	AAGCUGAAGAGAGCUGUGGA	Cleavage		
gma-miR396h	transcript_HQ_CoA_transcript17793/f2p0/2369	2.5	-1	1	20	2206	2225	UCCACAGCUUUCUUGAACUG	AAGCUGAAGAGAGCUGUGGA	Cleavage		
gma-miR396h	transcript_HQ_CoA_transcript23108/f2p0/1979	2.5	-1	1	20	1774	1793	UCCACAGCUUUCUUGAACUG	AAGCUGAAGAGAGCUGUGGA	Cleavage		
hbr-miR156	transcript_HQ_CoA_transcript11812/f2p0/2892	2.5	-1	1	19	2469	2487	UUGACAGAAGAUAGAGAGC	GCUCUCUCUCUUCUGUCAU	Cleavage	-0.772082878	0.014770298
hbr-miR156	transcript_HQ_CoA_transcript13408/f2p0/2769	2.5	-1	1	19	1933	1951	UUGACAGAAGAUAGAGAGC	GCUCUCUCUCUUCUGUCAU	Cleavage	-0.88200172	0.001652715
hbr-miR156	transcript_HQ_CoA_transcript14160/f2p0/2685	2.5	-1	1	19	1928	1946	UUGACAGAAGAUAGAGAGC	GCUCUCUCUCUUCUGUCAU	Cleavage	-0.970059945	1.48495E-05
hbr-miR156	transcript_HQ_CoA_transcript15965/f4p0/2528	2.5	-1	1	19	36	54	UUGACAGAAGAUAGAGAGC	UCUCUCUCUCUUCUGUCAA	Cleavage		
hbr-miR156	transcript_HQ_CoA_transcript16448/f2p0/2504	2.5	-1	1	19	43	61	UUGACAGAAGAUAGAGAGC	UCUCUCUCUCUUCUGUCAA	Cleavage		
hbr-miR156	transcript_HQ_CoA_transcript15947/f3p0/2445	2.5	-1	1	19	2178	2196	UUGACAGAAGAUAGAGAGC	UCUUUCUGUUUUCUGUCAA	Cleavage	-0.89212371	0.001219909
hbr-miR156	transcript_HQ_CoA_transcript16946/f3p0/2432	2.5	-1	1	19	5	23	UUGACAGAAGAUAGAGAGC	UCUCUCUCUCUUCUGUCAA	Cleavage		
hbr-miR156	transcript_HQ_CoA_transcript18005/f9p0/2335	2.5	-1	1	19	51	69	UUGACAGAAGAUAGAGAGC	UCUCUCUCUCUUCUGUCAA	Cleavage		
hbr-miR156	transcript_HQ_CoA_transcript19070/f2p0/2271	2.5	-1	1	19	11	29	UUGACAGAAGAUAGAGAGC	UCUCUCUCUCUUCUGUCAA	Cleavage	-0.716464597	0.029886005
hbr-miR156	transcript_HQ_CoA_transcript27850/f2p0/1604	2.5	-1	1	19	1135	1153	UUGACAGAAGAUAGAGAGC	GCUCUCUCUCUUCUGUCAU	Cleavage	-0.872670387	0.00213681
hbr-miR156	transcript_HQ_CoA_transcript1215/f2p0/5484	2.5	-1	1	19	4361	4379	UUGACAGAAGAUAGAGAGC	ACUAUCUAUUUUCUGUCAA	Cleavage		
hbr-miR156	transcript_HQ_CoA_transcript6684/f2p0/3554	2.5	-1	1	19	69	87	UUGACAGAAGAUAGAGAGC	GCUCACUCUCUUCUGUCAA	Cleavage		
hbr-miR156	transcript_HQ_CoA_transcript7286/f2p0/3515	2.5	-1	1	19	67	85	UUGACAGAAGAUAGAGAGC	GCUCACUCUCUUCUGUCAA	Cleavage		
mtr-miR319a-3p	transcript_HQ_CoA_transcript7017/f3p0/3537	2.5	-1	1	20	542	561	UUGGACUGAAGGGAGCUCCC	UGGGCCUCUCUUCAGUUCAA	Cleavage	0.712978725	0.031074514
mtr-miR319a-3p	transcript_HQ_CoA_transcript5815/f6p0/3697	2.5	-1	1	20	647	666	UUGGACUGAAGGGAGCUCCC	UGGGCUUCCCUUCAGUUCAA	Cleavage		
mtr-miR319a-3p	transcript_HQ_CoA_transcript24806/f3p0/1826	2.5	-1	1	20	1207	1226	UUGGACUGAAGGGAGCUCCC	AGGGGGACCCUUCAGUCCAA	Cleavage		
mtr-miR319a-3p	transcript_HQ_CoA_transcript23727/f3p0/1944	2.5	-1	1	20	1332	1351	UUGGACUGAAGGGAGCUCCC	AGGGGGACCCUUCAGUCCAA	Cleavage		
mtr-miR319a-3p	transcript_HQ_CoA_transcript16582/f5p0/2428	2.5	-1	1	20	1873	1892	UUGGACUGAAGGGAGCUCCC	AGGGGGACCCUUCAGUCCAA	Cleavage		
osa-miR159f	transcript_HQ_CoA_transcript14462/f14p0/2625	2.5	-1	1	21	134	154	CUUGGAUUGAAGGGAGCUCUA	UGGAGUUUCCUUCAAUUCAAC	Cleavage		
osa-miR395b	transcript_HQ_CoA_transcript27318/f2p0/1637	2.5	-1	1	21	369	389	GUGAAGUGUUUGGGGGAACUC	GAGUUCCUCCAAACUCUUCAU	Cleavage		
osa-miR395b	transcript_HQ_CoA_transcript26410/f2p0/1754	2.5	-1	1	21	416	436	GUGAAGUGUUUGGGGGAACUC	GAGUUCCUCCAAACUCUUCAU	Cleavage		
osa-miR395b	transcript_HQ_CoA_transcript20132/f3p0/2197	2.5	-1	1	21	214	234	GUGAAGUGUUUGGGGGAACUC	AAGUUCUCUUAAAUACUUCGC	Cleavage		
osa-miR395b	transcript_HQ_CoA_transcript11717/f10p0/2867	2.5	-1	1	21	151	171	GUGAAGUGUUUGGGGGAACUC	CUUUUCCCUCAAACAUUUCGC	Cleavage		
osa-miR395b	transcript_HQ_CoA_transcript9682/f2p0/3154	2.5	-1	1	21	154	174	GUGAAGUGUUUGGGGGAACUC	CUUUUCCCUCAAACAUUUCGC	Cleavage		
osa-miR395b	transcript_HQ_CoA_transcript12433/f2p0/2856	2.5	-1	1	21	641	661	GUGAAGUGUUUGGGGGAACUC	UUGUUCCCUCAAAUGCUUCGU	Cleavage		
osa-miR398b	transcript_HQ_CoA_transcript30164/f2p0/923	2.5	-1	1	21	112	132	UGUGUUCUCAGGUCGCCCCUG	AGGGGCCGACCUGAGAACAAA	Cleavage		
osa-miR408-3p	transcript_HQ_CoA_transcript12663/f3p0/2798	2.5	-1	1	21	1030	1050	CUGCACUGCCUCUUCCCUGGC	ACCAGGGAAGAUGCAGUGCAA	Translation		
osa-miR408-3p	transcript_HQ_CoA_transcript13253/f2p0/2781	2.5	-1	1	21	1072	1092	CUGCACUGCCUCUUCCCUGGC	ACCAGGGAAGAUGCAGUGCAA	Translation	0.90444662	0.00080788
osa-miR408-3p	transcript_HQ_CoA_transcript14475/f2p0/2651	2.5	-1	1	21	956	976	CUGCACUGCCUCUUCCCUGGC	ACCAGGGAAGAUGCAGUGCAA	Translation		
osa-miR408-3p	transcript_HQ_CoA_transcript13858/f10p0/2679	2.5	-1	1	21	1554	1574	CUGCACUGCCUCUUCCCUGGC	AACAGGGAGGAGGUAGUGAAG	Cleavage		
osa-miR408-3p	transcript_HQ_CoA_transcript15929/f5p0/2526	2.5	-1	1	21	1402	1422	CUGCACUGCCUCUUCCCUGGC	AACAGGGAGGAGGUAGUGAAG	Cleavage		
ppt-miR319a	transcript_HQ_CoA_transcript7017/f3p0/3537	2.5	-1	1	20	543	562	CUUGGACUGAAGGGAGCUCC	GGGCCUCUCUUCAGUUCAAG	Cleavage	0.767889515	0.015673771
ppt-miR319a	transcript_HQ_CoA_transcript5815/f6p0/3697	2.5	-1	1	20	648	667	CUUGGACUGAAGGGAGCUCC	GGGCUUCCCUUCAGUUCAAG	Cleavage		
pta-miR319	transcript_HQ_CoA_transcript7017/f3p0/3537	2.5	-1	1	19	543	561	UUGGACUGAAGGGAGCUCC	GGGCCUCUCUUCAGUUCAA	Cleavage	0.710777676	0.031840611
pta-miR319	transcript_HQ_CoA_transcript5815/f6p0/3697	2.5	-1	1	19	648	666	UUGGACUGAAGGGAGCUCC	GGGCUUCCCUUCAGUUCAA	Cleavage		
pta-miR319	transcript_HQ_CoA_transcript24806/f3p0/1826	2.5	-1	1	19	1208	1226	UUGGACUGAAGGGAGCUCC	GGGGGACCCUUCAGUCCAA	Cleavage		
pta-miR319	transcript_HQ_CoA_transcript23727/f3p0/1944	2.5	-1	1	19	1333	1351	UUGGACUGAAGGGAGCUCC	GGGGGACCCUUCAGUCCAA	Cleavage	-0.673525372	0.046718715
pta-miR319	transcript_HQ_CoA_transcript16582/f5p0/2428	2.5	-1	1	19	1874	1892	UUGGACUGAAGGGAGCUCC	GGGGGACCCUUCAGUCCAA	Cleavage		
tae-miR1122c-3p	transcript_HQ_CoA_transcript14251/f2p0/2673	2.5	-1	1	21	1862	1882	UCUAAUAUUAUGGGACGGAGG	CCUCCGUCCCAAAAUAUUAGU	Translation	0.806693893	0.008606492
tae-miR1122c-3p	transcript_HQ_CoA_transcript8904/f2p0/3235	2.5	-1	1	21	2798	2818	UCUAAUAUUAUGGGACGGAGG	CUUUUGUCCUAUAAUAUUAGU	Cleavage	0.692113673	0.038835113
tae-miR395b	transcript_HQ_CoA_transcript20132/f3p0/2197	2.5	-1	1	20	214	233	UGAAGUGUUUGGGGGAACUC	AAGUUCUCUUAAAUACUUCG	Cleavage		
vvi-miR396a	transcript_HQ_CoA_transcript7092/f3p0/3560	2.5	-1	1	21	145	165	UUCCACAGCUUUCUUGAACUA	AUGUUCAAGAAGCCUGUGGAG	Cleavage		
vvi-miR396a	transcript_HQ_CoA_transcript9321/f2p0/3219	2.5	-1	1	21	30	50	UUCCACAGCUUUCUUGAACUA	AUGUUCAAGAAGCCUGUGGAG	Cleavage		
vvi-miR396a	transcript_HQ_CoA_transcript17211/f3p0/2438	2.5	-1	1	21	169	189	UUCCACAGCUUUCUUGAACUA	AUGUUCAAGAAGCCUGUGGAG	Cleavage		
vvi-miR396a	transcript_HQ_CoA_transcript20814/f2p0/2176	2.5	-1	1	21	182	202	UUCCACAGCUUUCUUGAACUA	AUGUUCAAGAAGCCUGUGGAG	Cleavage		
vvi-miR396a	transcript_HQ_CoA_transcript4547/f5p0/4000	2.5	-1	1	21	194	214	UUCCACAGCUUUCUUGAACUA	GUGUUGAAGGAAGCUGUGGAU	Cleavage		
vvi-miR396a	transcript_HQ_CoA_transcript6429/f2p0/3612	2.5	-1	1	21	196	216	UUCCACAGCUUUCUUGAACUA	GUGUUGAAGGAAGCUGUGGAU	Cleavage		
vvi-miR396a	transcript_HQ_CoA_transcript7619/f3p0/3429	2.5	-1	1	21	211	231	UUCCACAGCUUUCUUGAACUA	GUGUUGAAGGAAGCUGUGGAU	Cleavage		

vvi-miR396a	transcript_HQ_CoA_transcript8209/f2p0/3353	2.5	-1	1	21	290	310	UUCCACAGCUUUCUUGAACUA	GUGUUGAAGGAAGCUGUGGAU	Cleavage		
vvi-miR396a	transcript_HQ_CoA_transcript8127/f2p0/3346	2.5	-1	1	21	127	147	UUCCACAGCUUUCUUGAACUA	GUGUUGAAGGAAGCUGUGGAU	Cleavage	-0.740815884	0.022405153
vvi-miR396a	transcript_HQ_CoA_transcript8592/f5p0/3264	2.5	-1	1	21	171	191	UUCCACAGCUUUCUUGAACUA	GUGUUGAAGGAAGCUGUGGAU	Cleavage		
vvi-miR396b	transcript_HQ_CoA_transcript7092/f3p0/3560	2.5	-1	1	20	146	165	UUCCACAGCUUUCUUGAACU	UGUUCAAGAAGCCUGUGGAG	Cleavage		
vvi-miR396b	transcript_HQ_CoA_transcript9321/f2p0/3219	2.5	-1	1	20	31	50	UUCCACAGCUUUCUUGAACU	UGUUCAAGAAGCCUGUGGAG	Cleavage		
vvi-miR396b	transcript_HQ_CoA_transcript17211/f3p0/2438	2.5	-1	1	20	170	189	UUCCACAGCUUUCUUGAACU	UGUUCAAGAAGCCUGUGGAG	Cleavage		
vvi-miR396b	transcript_HQ_CoA_transcript20814/f2p0/2176	2.5	-1	1	20	183	202	UUCCACAGCUUUCUUGAACU	UGUUCAAGAAGCCUGUGGAG	Cleavage		
vvi-miR396b	transcript_HQ_CoA_transcript4547/f5p0/4000	2.5	-1	1	20	195	214	UUCCACAGCUUUCUUGAACU	UGUUGAAGGAAGCUGUGGAU	Cleavage		
vvi-miR396b	transcript_HQ_CoA_transcript6429/f2p0/3612	2.5	-1	1	20	197	216	UUCCACAGCUUUCUUGAACU	UGUUGAAGGAAGCUGUGGAU	Cleavage	-0.705217542	0.033830418
vvi-miR396b	transcript_HQ_CoA_transcript7619/f3p0/3429	2.5	-1	1	20	212	231	UUCCACAGCUUUCUUGAACU	UGUUGAAGGAAGCUGUGGAU	Cleavage		
vvi-miR396b	transcript_HQ_CoA_transcript8209/f2p0/3353	2.5	-1	1	20	291	310	UUCCACAGCUUUCUUGAACU	UGUUGAAGGAAGCUGUGGAU	Cleavage		
vvi-miR396b	transcript_HQ_CoA_transcript8127/f2p0/3346	2.5	-1	1	20	128	147	UUCCACAGCUUUCUUGAACU	UGUUGAAGGAAGCUGUGGAU	Cleavage	-0.678077994	0.044700904
vvi-miR396b	transcript_HQ_CoA_transcript8592/f5p0/3264	2.5	-1	1	20	172	191	UUCCACAGCUUUCUUGAACU	UGUUGAAGGAAGCUGUGGAU	Cleavage		
zma-miR162-3p	transcript_HQ_CoA_transcript19045/f3p0/2263	2.5	-1	1	20	867	886	UCGAUAAACCUCUGCAUCCA	UGGAUGCAGAGGUUGAUUGG	Cleavage		
zma-miR162-3p	transcript_HQ_CoA_transcript18770/f2p0/2301	2.5	-1	1	20	978	997	UCGAUAAACCUCUGCAUCCA	UGGAUGCAGAGGUUGAUUGG	Cleavage		
zma-miR162-3p	transcript_HQ_CoA_transcript17171/f4p0/2404	2.5	-1	1	20	996	1015	UCGAUAAACCUCUGCAUCCA	UGGAUGCAGAGGUUGAUUGG	Cleavage	-0.884480908	0.001538203
zma-miR162-3p	transcript_HQ_CoA_transcript11695/f2p0/2917	2.5	-1	1	20	1502	1521	UCGAUAAACCUCUGCAUCCA	UGGAUGCAGAGGUUGAUUGG	Cleavage		
zma-miR171a-3p	transcript_HQ_CoA_transcript10152/f2p0/3094	2.5	-1	1	20	2474	2493	UGAUUGAGCCGCGCCAAUAU	GAAUUGGCGUGGUUCGAUCA	Cleavage	-0.929117441	0.000291145
zma-miR396g-3p	transcript_HQ_CoA_transcript4829/f3p0/4013	2.5	-1	1	21	3927	3947	GUUCAAGAAAGCUGUGGAAGA	CCUUACAUAGCUUUCUUGAAA	Cleavage		
zma-miR396g-3p	transcript_HQ_CoA_transcript25127/f3p0/1802	2.5	-1	1	21	930	950	GUUCAAGAAAGCUGUGGAAGA	GCUUCCUUAGUUUUCUUGAAU	Cleavage		
zma-miR396g-3p	transcript_HQ_CoA_transcript23209/f2p0/2005	2.5	-1	1	21	242	262	GUUCAAGAAAGCUGUGGAAGA	ACUAUCACAGCUUUUUUGAGC	Cleavage		
zma-miR396g-3p	transcript_HQ_CoA_transcript10327/f2p0/3066	2.5	-1	1	21	209	229	GUUCAAGAAAGCUGUGGAAGA	CAUUCCUUAGCUUUUUUGAGC	Cleavage		
zma-miR396g-3p	transcript_HQ_CoA_transcript9394/f2p0/3194	2.5	-1	1	21	293	313	GUUCAAGAAAGCUGUGGAAGA	CAUUCCUUAGCUUUUUUGAGC	Cleavage		
zma-miR396g-3p	transcript_HQ_CoA_transcript8721/f2p0/3275	2.5	-1	1	21	395	415	GUUCAAGAAAGCUGUGGAAGA	CAUUCCUUAGCUUUUUUGAGC	Cleavage		
zma-miR396g-3p	transcript_HQ_CoA_transcript3180/f2p0/4421	2.5	-1	1	21	1814	1834	GUUCAAGAAAGCUGUGGAAGA	GGUUUCUCAGUUUUCUUGAGC	Cleavage		
zma-miR398a-3p	transcript_HQ_CoA_transcript30164/f2p0/923	2.5	-1	1	21	112	132	UGUGUUCUCAGGUCGCCCCCG	AGGGGCCGACCUGAGAACAAA	Cleavage		
aof-miR160b	transcript_HQ_CoA_transcript25394/f2p0/1801	3	-1	1	21	190	210	UGCCUGGUUCCCUGUAUGCCA	UUGUAUAGAGGGAACCACGCA	Cleavage		
ata-miR408-3p	transcript_HQ_CoA_transcript10451/f4p0/3060	3	-1	1	20	407	426	UGCACUGCCUCUUCCCUGCC	UGAAGAGAAGAGGCAGUGCC	Cleavage		
ata-miR408-3p	transcript_HQ_CoA_transcript11719/f2p0/2901	3	-1	1	20	350	369	UGCACUGCCUCUUCCCUGCC	UGAAGAGAAGAGGCAGUGCC	Cleavage	0.684753121	0.041844953
ath-miR157a-5p	transcript_HQ_CoA_transcript25141/f2p0/1836	3	-1	1	21	661	681	UUGACAGAAGAUAGAGAGCAC	CUACUCUCUACUUUCUGUCAA	Translation	-0.696183884	0.037232581
ath-miR157a-5p	transcript_HQ_CoA_transcript14698/f2p0/2635	3	-1	1	21	178	198	UUGACAGAAGAUAGAGAGCAC	UCCCUCUCAAUCUUCUGUCGA	Cleavage		
ath-miR157a-5p	transcript_HQ_CoA_transcript20481/f2p0/2177	3	-1	1	21	1254	1274	UUGACAGAAGAUAGAGAGCAC	GGACUUUCUAUCUUCUUUCAA	Cleavage		
ath-miR157a-5p	transcript_HQ_CoA_transcript16040/f2p0/2524	3	-1	1	21	320	340	UUGACAGAAGAUAGAGAGCAC	UGGCUCUGUAUUUUUUGUUGA	Cleavage		
ath-miR159a	transcript_HQ_CoA_transcript12168/f3p0/2902	3	-1	1	21	1971	1991	UUUGGAUUGAAGGGAGCUCUA	AAGGGUUCUCUUCAAACCAAA	Cleavage		
ath-miR159a	transcript_HQ_CoA_transcript827/f2p0/5915	3	-1	1	21	5880	5900	UUUGGAUUGAAGGGAGCUCUA	UGGAGCCUUCUUUAAUCUAAA	Cleavage		
ath-miR159a	transcript_HQ_CoA_transcript18036/f2p0/2370	3	-1	1	21	362	382	UUUGGAUUGAAGGGAGCUCUA	AGGAGCUUCAUUCAAUCCAAU	Cleavage		
ath-miR159a	transcript_HQ_CoA_transcript7001/f2p0/3538	3	-1	1	21	320	340	UUUGGAUUGAAGGGAGCUCUA	AGGAGCUUCAUUCAAUCCAAU	Cleavage		
ath-miR159a	transcript_HQ_CoA_transcript21864/f2p0/2073	3	-1	1	21	1161	1181	UUUGGAUUGAAGGGAGCUCUA	UCGAGUUCUAUUCAAUUCAAA	Cleavage		
ath-miR159a	transcript_HQ_CoA_transcript20164/f2p0/2207	3	-1	1	21	330	350	UUUGGAUUGAAGGGAGCUCUA	AUGGGCUGUUUUCAGUCCAAA	Cleavage		
ath-miR159c	transcript_HQ_CoA_transcript18036/f2p0/2370	3	-1	1	21	362	382	UUUGGAUUGAAGGGAGCUCCU	AGGAGCUUCAUUCAAUCCAAU	Cleavage		
ath-miR159c	transcript_HQ_CoA_transcript7001/f2p0/3538	3	-1	1	21	320	340	UUUGGAUUGAAGGGAGCUCCU	AGGAGCUUCAUUCAAUCCAAU	Cleavage		
ath-miR159c	transcript_HQ_CoA_transcript827/f2p0/5915	3	-1	1	21	5880	5900	UUUGGAUUGAAGGGAGCUCCU	UGGAGCCUUCUUUAAUCUAAA	Cleavage		
ath-miR159c	transcript_HQ_CoA_transcript12168/f3p0/2902	3	-1	1	21	1971	1991	UUUGGAUUGAAGGGAGCUCCU	AAGGGUUCUCUUCAAACCAAA	Cleavage		
ath-miR159c	transcript_HQ_CoA_transcript21864/f2p0/2073	3	-1	1	21	1161	1181	UUUGGAUUGAAGGGAGCUCCU	UCGAGUUCUAUUCAAUUCAAA	Cleavage		
ath-miR159c	transcript_HQ_CoA_transcript20164/f2p0/2207	3	-1	1	21	330	350	UUUGGAUUGAAGGGAGCUCCU	AUGGGCUGUUUUCAGUCCAAA	Cleavage		
ath-miR162a-3p	transcript_HQ_CoA_transcript1140/f2p0/5609	3	-1	1	21	2524	2545	UCGAUAA-ACCUCUGCAUCCAG	CUGGAUGCAGAGGUGUUAUCGA	Cleavage	0.668948645	0.048805091
ath-miR162a-3p	transcript_HQ_CoA_transcript236/f2p0/7098	3	-1	1	21	3903	3924	UCGAUAA-ACCUCUGCAUCCAG	CUGGAUGCAGAGGUGUUUAUCGA	Cleavage		
ath-miR162a-3p	transcript_HQ_CoA_transcript23140/f4p0/1976	3	-1	1	21	506	526	UCGAUAAACCUCUGCAUCCAG	UUGGAGGUAGAGGUUUCUCGA	Cleavage	-0.727696927	0.026259533
ath-miR167a-5p	transcript_HQ_CoA_transcript24079/f2p0/1930	3	-1	1	21	1512	1532	UGAAGCUGCCAGCAUGAUCUA	UCGAUCAUGUUGGUAGAUUUA	Cleavage		
ath-miR167d	transcript_HQ_CoA_transcript24079/f2p0/1930	3	-1	1	22	1511	1532	UGAAGCUGCCAGCAUGAUCUGG	UUCGAUCAUGUUGGUAGAUUUA	Cleavage		
ath-miR171a-3p	transcript_HQ_CoA_transcript2950/f2p0/4495	3	-1	1	21	2792	2812	UGAUUGAGCCGCGCCAAUAUC	UGAAUUGGCGUGGUUUGAUCA	Cleavage	-0.887778985	0.001394559
ath-miR171a-3p	transcript_HQ_CoA_transcript13232/f2p0/2782	3	-1	1	21	1626	1646	UGAUUGAGCCGCGCCAAUAUC	UCUAUUGUUGCGGUUCAGUUA	Cleavage		
ath-miR319a	transcript_HQ_CoA_transcript14462/f14p0/2625	3	-1	1	21	133	153	UUGGACUGAAGGGAGCUCCCU	GUGGAGUUUCCUUCAAUUCAA	Cleavage		
ath-miR319a	transcript_HQ_CoA_transcript6680/f13p0/3559	3	-1	1	21	2300	2320	UUGGACUGAAGGGAGCUCCCU	AAGCAGCUUUCUUCAGUCCAU	Cleavage		
ath-miR319a	transcript_HQ_CoA_transcript20831/f3p0/2156	3	-1	1	21	1445	1465	UUGGACUGAAGGGAGCUCCCU	GAGGGGACCCCUUCAGUCCAG	Cleavage		
ath-miR319a	transcript_HQ_CoA_transcript17168/f2p0/2434	3	-1	1	21	1840	1860	UUGGACUGAAGGGAGCUCCCU	GAGGGGACCCCUUCAGUCCAG	Cleavage	-0.765679158	0.016164508
ath-miR394a	transcript_HQ_CoA_transcript12386/f4p0/2831	3	-1	1	20	2327	2346	UUGGCAUUCUGUCCACCUCC	GGAGUUGGACAGAAUGCUGC	Cleavage		
ath-miR394a	transcript_HQ_CoA_transcript565/f2p0/6250	3	-1	1	20	5982	6001	UUGGCAUUCUGUCCACCUCC	GGGGUUGGAUAGAAUGCUGA	Cleavage		
ath-miR394a	transcript_HQ_CoA_transcript28419/f2p0/1532	3	-1	1	20	621	640	UUGGCAUUCUGUCCACCUCC	UGAGGUGGAAGGAAUGCCAC	Translation		
ath-miR394a	transcript_HQ_CoA_transcript28377/f10p0/1492	3	-1	1	20	619	638	UUGGCAUUCUGUCCACCUCC	UGAGGUGGAAGGAAUGCCAC	Translation		
ath-miR394a	transcript_HQ_CoA_transcript4829/f3p0/4013	3	-1	1	20	1643	1662	UUGGCAUUCUGUCCACCUCC	UGAGGUUGAUAAAAUGCCAA	Cleavage		
ath-miR394a	transcript_HQ_CoA_transcript13365/f2p0/2755	3	-1	1	20	1674	1693	UUGGCAUUCUGUCCACCUCC	UGAGGUUGAUAAAAUGCCAA	Cleavage		
ath-miR394a	transcript_HQ_CoA_transcript14201/f3p0/2736	3	-1	1	20	450	469	UUGGCAUUCUGUCCACCUCC	UGGGUUGGACAGAAUUCCAA	Cleavage		
ath-miR394a	transcript_HQ_CoA_transcript14743/f2p0/2630	3	-1	1	20	623	642	UUGGCAUUCUGUCCACCUCC	UGGGUUGGACAGAAUUCCAA	Cleavage		
ath-miR394a	transcript_HQ_CoA_transcript17183/f4p0/2398	3	-1	1	20	396	415	UUGGCAUUCUGUCCACCUCC	UGGGUUGGACAGAAUUCCAA	Cleavage		
ath-miR394a	transcript_HQ_CoA_transcript16610/f8p0/2390	3	-1	1	20	443	462	UUGGCAUUCUGUCCACCUCC	UGGGUUGGACAGAAUUCCAA	Cleavage		

ath-miR394a	transcript_HQ_CoA_transcript18899/f2p0/2293	3	-1	1	20	469	488	UUGGCAUUCUGUCCACCUCC	UGGGUUGGACAGAAUUCCAA	Cleavage		
ath-miR395a	transcript_HQ_CoA_transcript27318/f2p0/1637	3	-1	1	21	369	389	CUGAAGUGUUUGGGGGAACUC	GAGUUCCUCCAAACUCUUCAU	Cleavage		
ath-miR395a	transcript_HQ_CoA_transcript26410/f2p0/1754	3	-1	1	21	416	436	CUGAAGUGUUUGGGGGAACUC	GAGUUCCUCCAAACUCUUCAU	Cleavage		
ath-miR395a	transcript_HQ_CoA_transcript21744/f2p0/2077	3	-1	1	21	146	166	CUGAAGUGUUUGGGGGAACUC	UUUAUCCCCCAAACACUUCAA	Cleavage	0.706729105	0.033281693
ath-miR395a	transcript_HQ_CoA_transcript12565/f4p0/2800	3	-1	1	21	93	113	CUGAAGUGUUUGGGGGAACUC	UUUAUCCCCCAAACACUUCAA	Cleavage	0.94213065	0.000145016
ath-miR395a	transcript_HQ_CoA_transcript12433/f2p0/2856	3	-1	1	21	641	661	CUGAAGUGUUUGGGGGAACUC	UUGUUCCCUCAAAUGCUUCGU	Cleavage		
ath-miR395a	transcript_HQ_CoA_transcript16055/f2p0/2593	3	-1	1	21	1327	1347	CUGAAGUGUUUGGGGGAACUC	UGGUUCAUCCAAAUGCUUCGG	Cleavage		
ath-miR396a-5p	transcript_HQ_CoA_transcript25598/f3p0/1743	3	-1	1	21	688	708	UUCCACAGCUUUCUUGAACUG	CAGGUCAAGAAAGCUAUGGAG	Cleavage	0.797851965	0.009972274
ath-miR396a-5p	transcript_HQ_CoA_transcript26456/f33p0/1704	3	-1	1	21	688	708	UUCCACAGCUUUCUUGAACUG	CAGGUCAAGAAAGCUAUGGAG	Cleavage		
ath-miR396a-5p	transcript_HQ_CoA_transcript19820/f2p0/2215	3	-1	1	21	512	533	UUCCACA-GCUUUCUUGAACUG	CCGUUCAAGAAAGCCUGUGGAA	Cleavage	-0.73487583	0.0241005
ath-miR396a-5p	transcript_HQ_CoA_transcript20271/f2p0/2193	3	-1	1	21	343	364	UUCCACA-GCUUUCUUGAACUG	CCGUUCAAGAAAGCCUGUGGAA	Cleavage		
ath-miR396a-5p	transcript_HQ_CoA_transcript21509/f4p0/2050	3	-1	1	21	373	394	UUCCACA-GCUUUCUUGAACUG	CCGUUCAAGAAAGCCUGUGGAA	Cleavage		
ath-miR396a-5p	transcript_HQ_CoA_transcript27471/f2p0/1641	3	-1	1	21	429	450	UUCCACA-GCUUUCUUGAACUG	CCGUUCAAGAAAGCCUGUGGAA	Cleavage	-0.707484057	0.033009814
ath-miR396a-5p	transcript_HQ_CoA_transcript27685/f2p0/1608	3	-1	1	21	509	530	UUCCACA-GCUUUCUUGAACUG	CCGUUCAAGAAAGCCUGUGGAA	Cleavage	-0.876766703	0.00191369
ath-miR396a-5p	transcript_HQ_CoA_transcript27879/f3p0/1596	3	-1	1	21	544	565	UUCCACA-GCUUUCUUGAACUG	CCGUUCAAGAAAGCCUGUGGAA	Cleavage		
ath-miR396a-5p	transcript_HQ_CoA_transcript27832/f2p0/1591	3	-1	1	21	492	513	UUCCACA-GCUUUCUUGAACUG	CCGUUCAAGAAAGCCUGUGGAA	Cleavage	-0.887755215	0.001395559
ath-miR396a-5p	transcript_HQ_CoA_transcript28053/f3p0/1571	3	-1	1	21	536	557	UUCCACA-GCUUUCUUGAACUG	CCGUUCAAGAAAGCCUGUGGAA	Cleavage	-0.838621758	0.004729483
ath-miR396a-5p	transcript_HQ_CoA_transcript29242/f2p0/1411	3	-1	1	21	470	491	UUCCACA-GCUUUCUUGAACUG	CCGUUCAAGAAAGCCUGUGGAA	Cleavage	-0.901260885	0.000903243
ath-miR396a-5p	transcript_HQ_CoA_transcript29824/f2p0/1232	3	-1	1	21	342	363	UUCCACA-GCUUUCUUGAACUG	CCGUUCAAGAAAGCCUGUGGAA	Cleavage		
ath-miR396a-5p	transcript_HQ_CoA_transcript19242/f4p0/2213	3	-1	1	21	997	1018	UUCCACA-GCUUUCUUGAACUG	UCGUUCAAGAAAGCCUGUGGAA	Cleavage	-0.677402939	0.044996498
ath-miR396a-5p	transcript_HQ_CoA_transcript19308/f8p0/2200	3	-1	1	21	890	911	UUCCACA-GCUUUCUUGAACUG	UCGUUCAAGAAAGCCUGUGGAA	Cleavage	-0.780367182	0.013089113
ath-miR396a-5p	transcript_HQ_CoA_transcript20070/f3p0/2193	3	-1	1	21	951	972	UUCCACA-GCUUUCUUGAACUG	UCGUUCAAGAAAGCCUGUGGAA	Cleavage	-0.702788147	0.034724598
ath-miR396a-5p	transcript_HQ_CoA_transcript20608/f3p0/2171	3	-1	1	21	1038	1059	UUCCACA-GCUUUCUUGAACUG	UCGUUCAAGAAAGCCUGUGGAA	Cleavage	-0.687087756	0.040874572
ath-miR396a-5p	transcript_HQ_CoA_transcript5396/f2p0/3810	3	-1	1	21	1039	1059	UUCCACAGCUUUCUUGAACUG	GGGUUCAGGAAAGUUGUUGGA	Cleavage		
ath-miR396a-5p	transcript_HQ_CoA_transcript8702/f2p0/3286	3	-1	1	21	2697	2717	UUCCACAGCUUUCUUGAACUG	GAGUUCAUGGAAGCUGUAGAA	Cleavage		
ath-miR396a-5p	transcript_HQ_CoA_transcript18586/f2p0/2310	3	-1	1	21	1606	1626	UUCCACAGCUUUCUUGAACUG	AAGAUCAAGAAAGUGGUGGAA	Cleavage		
ath-miR396a-5p	transcript_HQ_CoA_transcript4298/f2p0/4029	3	-1	1	21	2151	2171	UUCCACAGCUUUCUUGAACUG	AUUUUCAGGAAGGUUGUGGAG	Cleavage		
ath-miR396a-5p	transcript_HQ_CoA_transcript16798/f2p0/2437	3	-1	1	21	1376	1396	UUCCACAGCUUUCUUGAACUG	ACAUUCAGGAAAGCUCUGGAA	Cleavage		
ath-miR396a-5p	transcript_HQ_CoA_transcript976/f3p0/5771	3	-1	1	21	3270	3290	UUCCACAGCUUUCUUGAACUG	GACUCCAAGAAAGCUGUGGAU	Cleavage		
ath-miR396a-5p	transcript_HQ_CoA_transcript1778/f2p0/5116	3	-1	1	21	275	295	UUCCACAGCUUUCUUGAACUG	GACUCCAAGAAAGCUGUGGAU	Cleavage		
ath-miR396a-5p	transcript_HQ_CoA_transcript16187/f3p0/2582	3	-1	1	21	1820	1839	UUCCACAGCUUUCUUGAACUG	UAGUU-GAGGAAGCUGUGGAA	Cleavage	0.677173424	0.045097284
ath-miR396a-5p	transcript_HQ_CoA_transcript16411/f2p0/2480	3	-1	1	21	613	633	UUCCACAGCUUUCUUGAACUG	GCGUUCAAGAAGGUUGUGAGA	Cleavage	0.698741181	0.036247977
ath-miR396a-5p	transcript_HQ_CoA_transcript17868/f2p0/2352	3	-1	1	21	1899	1919	UUCCACAGCUUUCUUGAACUG	GAUUUGAGGAAGGCUGUGGAA	Cleavage	0.859509086	0.002974718
ath-miR396a-5p	transcript_HQ_CoA_transcript1328/f2p0/5403	3	-1	1	21	2238	2258	UUCCACAGCUUUCUUGAACUG	CUGUCUGGGAAAGCUGUGGGA	Cleavage		
ath-miR396a-5p	transcript_HQ_CoA_transcript1711/f8p0/5021	3	-1	1	21	2230	2250	UUCCACAGCUUUCUUGAACUG	CUGUCUGGGAAAGCUGUGGGA	Cleavage		
ath-miR396a-5p	transcript_HQ_CoA_transcript1959/f2p0/4936	3	-1	1	21	2324	2344	UUCCACAGCUUUCUUGAACUG	CUGUCUGGGAAAGCUGUGGGA	Cleavage		
ath-miR396a-5p	transcript_HQ_CoA_transcript1986/f2p0/4924	3	-1	1	21	2306	2326	UUCCACAGCUUUCUUGAACUG	CUGUCUGGGAAAGCUGUGGGA	Cleavage		
ath-miR396a-5p	transcript_HQ_CoA_transcript1991/f20p0/4837	3	-1	1	21	2230	2250	UUCCACAGCUUUCUUGAACUG	CUGUCUGGGAAAGCUGUGGGA	Cleavage		
ath-miR396a-5p	transcript_HQ_CoA_transcript2158/f3p0/4811	3	-1	1	21	2249	2269	UUCCACAGCUUUCUUGAACUG	CUGUCUGGGAAAGCUGUGGGA	Cleavage	-0.858839589	0.003022557
ath-miR396a-5p	transcript_HQ_CoA_transcript1910/f2p0/4979	3	-1	1	21	2263	2283	UUCCACAGCUUUCUUGAACUG	UUGUCUGGGAAAGCUGUGGGA	Cleavage		
ath-miR396a-5p	transcript_HQ_CoA_transcript8140/f2p0/3346	3	-1	1	21	446	466	UUCCACAGCUUUCUUGAACUG	UUGCUCAAGAACGCUGUGGAG	Translation	0.721679102	0.028164225
ath-miR396a-5p	transcript_HQ_CoA_transcript8912/f2p0/3245	3	-1	1	21	428	448	UUCCACAGCUUUCUUGAACUG	UUGCUCAAGAACGCUGUGGAG	Translation	0.820276316	0.006764379
ath-miR396a-5p	transcript_HQ_CoA_transcript9028/f2p0/3240	3	-1	1	21	355	375	UUCCACAGCUUUCUUGAACUG	UUGCUCAAGAACGCUGUGGAG	Translation		
ath-miR396a-5p	transcript_HQ_CoA_transcript10590/f3p0/3032	3	-1	1	21	349	369	UUCCACAGCUUUCUUGAACUG	UUGCUCAAGAACGCUGUGGAG	Translation	0.890569302	0.001280533
ath-miR396a-5p	transcript_HQ_CoA_transcript15769/f15p0/2406	3	-1	1	21	342	362	UUCCACAGCUUUCUUGAACUG	UCGUUGAAGGAAGCUCUGGAA	Cleavage	-0.820020618	0.006796317
ath-miR396b-5p	transcript_HQ_CoA_transcript18586/f2p0/2310	3	-1	1	21	1606	1626	UUCCACAGCUUUCUUGAACUU	AAGAUCAAGAAAGUGGUGGAA	Cleavage		
ath-miR396b-5p	transcript_HQ_CoA_transcript5396/f2p0/3810	3	-1	1	21	1039	1059	UUCCACAGCUUUCUUGAACUU	GGGUUCAGGAAAGUUGUUGGA	Cleavage		
ath-miR396b-5p	transcript_HQ_CoA_transcript8702/f2p0/3286	3	-1	1	21	2697	2717	UUCCACAGCUUUCUUGAACUU	GAGUUCAUGGAAGCUGUAGAA	Cleavage		
ath-miR396b-5p	transcript_HQ_CoA_transcript19820/f2p0/2215	3	-1	1	21	512	533	UUCCACA-GCUUUCUUGAACUU	CCGUUCAAGAAAGCCUGUGGAA	Cleavage	-0.723871285	0.027460244
ath-miR396b-5p	transcript_HQ_CoA_transcript19242/f4p0/2213	3	-1	1	21	997	1018	UUCCACA-GCUUUCUUGAACUU	UCGUUCAAGAAAGCCUGUGGAA	Cleavage	-0.711742808	0.031503189
ath-miR396b-5p	transcript_HQ_CoA_transcript19308/f8p0/2200	3	-1	1	21	890	911	UUCCACA-GCUUUCUUGAACUU	UCGUUCAAGAAAGCCUGUGGAA	Cleavage	-0.76751033	0.01575724
ath-miR396b-5p	transcript_HQ_CoA_transcript20271/f2p0/2193	3	-1	1	21	343	364	UUCCACA-GCUUUCUUGAACUU	CCGUUCAAGAAAGCCUGUGGAA	Cleavage		
ath-miR396b-5p	transcript_HQ_CoA_transcript20070/f3p0/2193	3	-1	1	21	951	972	UUCCACA-GCUUUCUUGAACUU	UCGUUCAAGAAAGCCUGUGGAA	Cleavage	-0.715859947	0.030089994
ath-miR396b-5p	transcript_HQ_CoA_transcript20608/f3p0/2171	3	-1	1	21	1038	1059	UUCCACA-GCUUUCUUGAACUU	UCGUUCAAGAAAGCCUGUGGAA	Cleavage	-0.686552252	0.041095855
ath-miR396b-5p	transcript_HQ_CoA_transcript21509/f4p0/2050	3	-1	1	21	373	394	UUCCACA-GCUUUCUUGAACUU	CCGUUCAAGAAAGCCUGUGGAA	Cleavage	-0.70809306	0.032791552
ath-miR396b-5p	transcript_HQ_CoA_transcript27471/f2p0/1641	3	-1	1	21	429	450	UUCCACA-GCUUUCUUGAACUU	CCGUUCAAGAAAGCCUGUGGAA	Cleavage	-0.748722509	0.020273416
ath-miR396b-5p	transcript_HQ_CoA_transcript27685/f2p0/1608	3	-1	1	21	509	530	UUCCACA-GCUUUCUUGAACUU	CCGUUCAAGAAAGCCUGUGGAA	Cleavage	-0.845455221	0.004093585
ath-miR396b-5p	transcript_HQ_CoA_transcript27879/f3p0/1596	3	-1	1	21	544	565	UUCCACA-GCUUUCUUGAACUU	CCGUUCAAGAAAGCCUGUGGAA	Cleavage	-0.677001705	0.045172784
ath-miR396b-5p	transcript_HQ_CoA_transcript27832/f2p0/1591	3	-1	1	21	492	513	UUCCACA-GCUUUCUUGAACUU	CCGUUCAAGAAAGCCUGUGGAA	Cleavage	-0.892381423	0.001210055
ath-miR396b-5p	transcript_HQ_CoA_transcript28053/f3p0/1571	3	-1	1	21	536	557	UUCCACA-GCUUUCUUGAACUU	CCGUUCAAGAAAGCCUGUGGAA	Cleavage	-0.840095407	0.004587008
ath-miR396b-5p	transcript_HQ_CoA_transcript29242/f2p0/1411	3	-1	1	21	470	491	UUCCACA-GCUUUCUUGAACUU	CCGUUCAAGAAAGCCUGUGGAA	Cleavage	-0.897576401	0.001023007
ath-miR396b-5p	transcript_HQ_CoA_transcript29824/f2p0/1232	3	-1	1	21	342	363	UUCCACA-GCUUUCUUGAACUU	CCGUUCAAGAAAGCCUGUGGAA	Cleavage		
ath-miR396b-5p	transcript_HQ_CoA_transcript976/f3p0/5771	3	-1	1	21	3270	3290	UUCCACAGCUUUCUUGAACUU	GACUCCAAGAAAGCUGUGGAU	Cleavage		
ath-miR396b-5p	transcript_HQ_CoA_transcript1778/f2p0/5116	3	-1	1	21	275	295	UUCCACAGCUUUCUUGAACUU	GACUCCAAGAAAGCUGUGGAU	Cleavage		
ath-miR396b-5p	transcript_HQ_CoA_transcript25598/f3p0/1743	3	-1	1	21	688	708	UUCCACAGCUUUCUUGAACUU	CAGGUCAAGAAAGCUAUGGAG	Cleavage	0.820669639	0.00671545
ath-miR396b-5p	transcript_HQ_CoA_transcript26456/f33p0/1704	3	-1	1	21	688	708	UUCCACAGCUUUCUUGAACUU	CAGGUCAAGAAAGCUAUGGAG	Cleavage		

ath-miR396b-5p	transcript_HQ_CoA_transcript4298/f2p0/4029	3	-1	1	21	2151	2171	UUCCACAGCUUUCUUGAACUU	AUUUUCAGGAAGGUUGUGGAG	Cleavage		
ath-miR396b-5p	transcript_HQ_CoA_transcript16798/f2p0/2437	3	-1	1	21	1376	1396	UUCCACAGCUUUCUUGAACUU	ACAUUCAGGAAAGCUCUGGAA	Cleavage		
ath-miR396b-5p	transcript_HQ_CoA_transcript17868/f2p0/2352	3	-1	1	21	1899	1919	UUCCACAGCUUUCUUGAACUU	GAUUUGAGGAAGGCUGUGGAA	Cleavage	0.889426878	0.001326402
ath-miR396b-5p	transcript_HQ_CoA_transcript16411/f2p0/2480	3	-1	1	21	613	633	UUCCACAGCUUUCUUGAACUU	GCGUUCAAGAAGGUUGUGAGA	Cleavage	0.772026167	0.014782278
ath-miR396b-5p	transcript_HQ_CoA_transcript1328/f2p0/5403	3	-1	1	21	2238	2258	UUCCACAGCUUUCUUGAACUU	CUGUCUGGGAAAGCUGUGGGA	Cleavage		
ath-miR396b-5p	transcript_HQ_CoA_transcript1711/f8p0/5021	3	-1	1	21	2230	2250	UUCCACAGCUUUCUUGAACUU	CUGUCUGGGAAAGCUGUGGGA	Cleavage		
ath-miR396b-5p	transcript_HQ_CoA_transcript1910/f2p0/4979	3	-1	1	21	2263	2283	UUCCACAGCUUUCUUGAACUU	UUGUCUGGGAAAGCUGUGGGA	Cleavage		
ath-miR396b-5p	transcript_HQ_CoA_transcript1959/f2p0/4936	3	-1	1	21	2324	2344	UUCCACAGCUUUCUUGAACUU	CUGUCUGGGAAAGCUGUGGGA	Cleavage		
ath-miR396b-5p	transcript_HQ_CoA_transcript1986/f2p0/4924	3	-1	1	21	2306	2326	UUCCACAGCUUUCUUGAACUU	CUGUCUGGGAAAGCUGUGGGA	Cleavage		
ath-miR396b-5p	transcript_HQ_CoA_transcript1991/f20p0/4837	3	-1	1	21	2230	2250	UUCCACAGCUUUCUUGAACUU	CUGUCUGGGAAAGCUGUGGGA	Cleavage		
ath-miR396b-5p	transcript_HQ_CoA_transcript2158/f3p0/4811	3	-1	1	21	2249	2269	UUCCACAGCUUUCUUGAACUU	CUGUCUGGGAAAGCUGUGGGA	Cleavage	-0.90047672	0.000927867
ath-miR396b-5p	transcript_HQ_CoA_transcript8140/f2p0/3346	3	-1	1	21	446	466	UUCCACAGCUUUCUUGAACUU	UUGCUCAAGAACGCUGUGGAG	Translation	0.684863588	0.041798707
ath-miR396b-5p	transcript_HQ_CoA_transcript8912/f2p0/3245	3	-1	1	21	428	448	UUCCACAGCUUUCUUGAACUU	UUGCUCAAGAACGCUGUGGAG	Translation	0.848425546	0.003836465
ath-miR396b-5p	transcript_HQ_CoA_transcript9028/f2p0/3240	3	-1	1	21	355	375	UUCCACAGCUUUCUUGAACUU	UUGCUCAAGAACGCUGUGGAG	Translation	0.704331307	0.034154856
ath-miR396b-5p	transcript_HQ_CoA_transcript10590/f3p0/3032	3	-1	1	21	349	369	UUCCACAGCUUUCUUGAACUU	UUGCUCAAGAACGCUGUGGAG	Translation	0.884123125	0.001554378
ath-miR396b-5p	transcript_HQ_CoA_transcript15769/f15p0/2406	3	-1	1	21	342	362	UUCCACAGCUUUCUUGAACUU	UCGUUGAAGGAAGCUCUGGAA	Cleavage	-0.806823274	0.008587507
ath-miR408-3p	transcript_HQ_CoA_transcript10228/f2p0/3091	3	-1	1	21	2688	2708	AUGCACUGCCUCUUCCCUGGC	GACAGUAAAGAGGUAGUGUAU	Cleavage		
bna-miR167d	transcript_HQ_CoA_transcript24079/f2p0/1930	3	-1	1	20	1513	1532	UGAAGCUGCCAGCAUGAUCU	CGAUCAUGUUGGUAGAUUUA	Cleavage		
bra-miR408-5p	transcript_HQ_CoA_transcript252/f2p0/7034	3	-1	1	21	5640	5660	GGGAGCCAGGGAAGAGGCAGU	UCUGCUUCUUCCUUGGCUUUA	Cleavage		
bra-miR408-5p	transcript_HQ_CoA_transcript14034/f2p0/2687	3	-1	1	21	1581	1601	GGGAGCCAGGGAAGAGGCAGU	GUUGUCUUUUUUUUGGUUCCC	Cleavage		
bra-miR408-5p	transcript_HQ_CoA_transcript24819/f4p0/1840	3	-1	1	21	353	373	GGGAGCCAGGGAAGAGGCAGU	UCCGCCUCUUCUCUGGCUGCC	Cleavage		
bra-miR408-5p	transcript_HQ_CoA_transcript25951/f3p0/1760	3	-1	1	21	379	399	GGGAGCCAGGGAAGAGGCAGU	UCCGCCUCUUCUCUGGCUGCC	Cleavage		
bra-miR408-5p	transcript_HQ_CoA_transcript1946/f3p0/4950	3	-1	1	21	4656	4676	GGGAGCCAGGGAAGAGGCAGU	UAUGCCUCUUUCGUGGCUUUC	Cleavage		
bra-miR408-5p	transcript_HQ_CoA_transcript2462/f8p0/4647	3	-1	1	21	4361	4381	GGGAGCCAGGGAAGAGGCAGU	UAUGCCUCUUUCGUGGCUUUC	Cleavage		
bra-miR408-5p	transcript_HQ_CoA_transcript2676/f2p0/4614	3	-1	1	21	4329	4349	GGGAGCCAGGGAAGAGGCAGU	UAUGCCUCUUUCGUGGCUUUC	Cleavage	0.669188564	0.048694269
bra-miR408-5p	transcript_HQ_CoA_transcript7/f2p0/10188	3	-1	1	21	1672	1692	GGGAGCCAGGGAAGAGGCAGU	UCUCCUACUUCCCUGGCUCCU	Cleavage		
bra-miR408-5p	transcript_HQ_CoA_transcript10/f2p0/9974	3	-1	1	21	1676	1696	GGGAGCCAGGGAAGAGGCAGU	UCUCCUACUUCCCUGGCUCCU	Cleavage		
bra-miR408-5p	transcript_HQ_CoA_transcript28114/f2p0/1613	3	-1	1	21	1174	1194	GGGAGCCAGGGAAGAGGCAGU	AGUACUUUUUCCCUGGCUCUU	Cleavage		
bra-miR408-5p	transcript_HQ_CoA_transcript1843/f2p0/5036	3	-1	1	21	4746	4766	GGGAGCCAGGGAAGAGGCAGU	UGUGGUUCUUUCUUGGUUCCC	Cleavage		
bra-miR408-5p	transcript_HQ_CoA_transcript2589/f2p0/4628	3	-1	1	21	4302	4322	GGGAGCCAGGGAAGAGGCAGU	UGUGGUUCUUUCUUGGUUCCC	Cleavage		
bra-miR408-5p	transcript_HQ_CoA_transcript2670/f2p0/4592	3	-1	1	21	4293	4313	GGGAGCCAGGGAAGAGGCAGU	UGUGGUUCUUUCUUGGUUCCC	Cleavage		
bra-miR408-5p	transcript_HQ_CoA_transcript4578/f2p0/3987	3	-1	1	21	3593	3613	GGGAGCCAGGGAAGAGGCAGU	UGUGGUUCUUUCUUGGUUCCC	Cleavage		
bra-miR408-5p	transcript_HQ_CoA_transcript7048/f2p0/3489	3	-1	1	21	3097	3117	GGGAGCCAGGGAAGAGGCAGU	UGUGGUUCUUUCUUGGUUCCC	Cleavage		
bra-miR408-5p	transcript_HQ_CoA_transcript7729/f2p0/3410	3	-1	1	21	2989	3009	GGGAGCCAGGGAAGAGGCAGU	UGUGGUUCUUUCUUGGUUCCC	Cleavage		
bra-miR408-5p	transcript_HQ_CoA_transcript9199/f2p0/3213	3	-1	1	21	2924	2944	GGGAGCCAGGGAAGAGGCAGU	UGUGGUUCUUUCUUGGUUCCC	Cleavage		
bra-miR408-5p	transcript_HQ_CoA_transcript9269/f4p0/3197	3	-1	1	21	2776	2796	GGGAGCCAGGGAAGAGGCAGU	UGUGGUUCUUUCUUGGUUCCC	Cleavage		
bra-miR408-5p	transcript_HQ_CoA_transcript10573/f6p0/3040	3	-1	1	21	2746	2766	GGGAGCCAGGGAAGAGGCAGU	UGUGGUUCUUUCUUGGUUCCC	Cleavage		
cme-miR166i	transcript_HQ_CoA_transcript20566/f5p0/2157	3	-1	1	20	1593	1613	UCGGACCAG-GCUUCAUUCUC	GAGAAUGAAGCACUGGUCCGA	Cleavage		
cme-miR166i	transcript_HQ_CoA_transcript2943/f3p0/4508	3	-1	1	20	2205	2224	UCGGACCAGGCUUCAUUCUC	AAGAAUUGAGUUUGGUUCGA	Cleavage		
csi-miR167d-3p	transcript_HQ_CoA_transcript13144/f4p0/2774	3	-1	1	24	1774	1797	AUCGGAUCAUGUGGUAGCUUCACC	AUUGCAGCUGACACGUGAUCCGAG	Cleavage	-0.79431582	0.010557089
csi-miR167d-3p	transcript_HQ_CoA_transcript11973/f3p0/2856	3	-1	1	24	1732	1755	AUCGGAUCAUGUGGUAGCUUCACC	AUUGCAGCUGACACGUGAUCCGAG	Cleavage	-0.825039901	0.006187892
csi-miR167d-3p	transcript_HQ_CoA_transcript10068/f3p0/3129	3	-1	1	24	1758	1781	AUCGGAUCAUGUGGUAGCUUCACC	CACCCAGCUACCAUACGGUCUGAU	Cleavage		
csi-miR167d-3p	transcript_HQ_CoA_transcript10209/f2p0/3158	3	-1	1	24	1760	1783	AUCGGAUCAUGUGGUAGCUUCACC	CACCCAGCUACCAUACGGUCUGAU	Cleavage		
gma-miR167h	transcript_HQ_CoA_transcript9667/f7p0/3091	3	-1	1	24	224	247	AUCAUGCUGGCAGCUUCAACUGGU	UUCAGUUGAAGCUUCCAGUAUGAG	Translation		
gma-miR167h	transcript_HQ_CoA_transcript9580/f3p0/3175	3	-1	1	24	205	228	AUCAUGCUGGCAGCUUCAACUGGU	UUCAGUUGAAGCUUCCAGUAUGAG	Translation		
gma-miR167h	transcript_HQ_CoA_transcript8957/f7p0/3188	3	-1	1	24	252	275	AUCAUGCUGGCAGCUUCAACUGGU	UUCAGUUGAAGCUUCCAGUAUGAG	Translation		
gma-miR167h	transcript_HQ_CoA_transcript9258/f3p0/3206	3	-1	1	24	305	328	AUCAUGCUGGCAGCUUCAACUGGU	UUCAGUUGAAGCUUCCAGUAUGAG	Translation		
gma-miR167h	transcript_HQ_CoA_transcript21336/f2p0/2120	3	-1	1	24	141	164	AUCAUGCUGGCAGCUUCAACUGGU	CAGAGUGGAAGUUGUCAGUAUGGU	Cleavage		
gma-miR167h	transcript_HQ_CoA_transcript16673/f2p0/2454	3	-1	1	24	1728	1751	AUCAUGCUGGCAGCUUCAACUGGU	AUUUGUUCAAUCUGCCGGUAUGAU	Cleavage		
gma-miR167h	transcript_HQ_CoA_transcript16941/f2p0/2502	3	-1	1	24	2140	2163	AUCAUGCUGGCAGCUUCAACUGGU	UGAACCUGAAGCUGUCGGUGUGAU	Cleavage		
gma-miR167h	transcript_HQ_CoA_transcript15749/f2p0/2543	3	-1	1	24	2453	2476	AUCAUGCUGGCAGCUUCAACUGGU	CAAUUUGAAGCUGCCAGUAUAAA	Cleavage		
gma-miR167h	transcript_HQ_CoA_transcript27856/f3p0/1603	3	-1	1	24	1376	1398	AUCAUGCUGGCAGCUUCAACUGGU	UGUGGUAGAA-CUGCCAGCAUGAU	Cleavage	0.70286291	0.034696854
gma-miR167h	transcript_HQ_CoA_transcript25675/f4p0/1782	3	-1	1	24	1071	1094	AUCAUGCUGGCAGCUUCAACUGGU	AGUGUUUGAGCCUGCAAGCAUGAU	Cleavage		
gma-miR396h	transcript_HQ_CoA_transcript3685/f2p0/4228	3	-1	1	20	665	684	UCCACAGCUUUCUUGAACUG	CAGAUCAAGGAAGCUGGGGA	Cleavage		
gma-miR396h	transcript_HQ_CoA_transcript17541/f4p0/2355	3	-1	1	20	711	730	UCCACAGCUUUCUUGAACUG	CAGAUCAAGGAAGCUGGGGA	Cleavage		
gma-miR396h	transcript_HQ_CoA_transcript7092/f3p0/3560	3	-1	1	20	145	164	UCCACAGCUUUCUUGAACUG	AUGUUCAAGAAGCCUGUGGA	Cleavage		
gma-miR396h	transcript_HQ_CoA_transcript9321/f2p0/3219	3	-1	1	20	30	49	UCCACAGCUUUCUUGAACUG	AUGUUCAAGAAGCCUGUGGA	Cleavage		
gma-miR396h	transcript_HQ_CoA_transcript17211/f3p0/2438	3	-1	1	20	169	188	UCCACAGCUUUCUUGAACUG	AUGUUCAAGAAGCCUGUGGA	Cleavage		
gma-miR396h	transcript_HQ_CoA_transcript20814/f2p0/2176	3	-1	1	20	182	201	UCCACAGCUUUCUUGAACUG	AUGUUCAAGAAGCCUGUGGA	Cleavage		
gma-miR396h	transcript_HQ_CoA_transcript5665/f2p0/3801	3	-1	1	20	3608	3627	UCCACAGCUUUCUUGAACUG	AAGUUGAAGAGAGCUUUGGA	Cleavage	-0.775418458	0.014077016
gma-miR396h	transcript_HQ_CoA_transcript6478/f3p0/3613	3	-1	1	20	2513	2532	UCCACAGCUUUCUUGAACUG	GAAUUCAAGAAAGUUGAGGA	Cleavage		
gma-miR396h	transcript_HQ_CoA_transcript7120/f2p0/3525	3	-1	1	20	2422	2441	UCCACAGCUUUCUUGAACUG	GAAUUCAAGAAAGUUGAGGA	Cleavage		
gma-miR396h	transcript_HQ_CoA_transcript7090/f8p0/3502	3	-1	1	20	2404	2423	UCCACAGCUUUCUUGAACUG	GAAUUCAAGAAAGUUGAGGA	Cleavage		
gma-miR396h	transcript_HQ_CoA_transcript13677/f2p0/2726	3	-1	1	20	1649	1668	UCCACAGCUUUCUUGAACUG	GAAUUCAAGAAAGUUGAGGA	Cleavage		
gma-miR396h	transcript_HQ_CoA_transcript14993/f3p0/2633	3	-1	1	20	1539	1558	UCCACAGCUUUCUUGAACUG	GAAUUCAAGAAAGUUGAGGA	Cleavage		
gma-miR396h	transcript_HQ_CoA_transcript18586/f2p0/2310	3	-1	1	20	1606	1625	UCCACAGCUUUCUUGAACUG	AAGAUCAAGAAAGUGGUGGA	Cleavage		

gma-miR396h	transcript_HQ_CoA_transcript12052/f2p0/2876	3	-1	1	20	856	875	UCCACAGCUUUCUUGAACUG	GAAUACAAGAAAGCUGUGGU	Cleavage	0.689311221	0.039964002
gma-miR396h	transcript_HQ_CoA_transcript12669/f6p0/2811	3	-1	1	20	753	772	UCCACAGCUUUCUUGAACUG	GAAUACAAGAAAGCUGUGGU	Cleavage	0.674274775	0.046382632
gma-miR396h	transcript_HQ_CoA_transcript16187/f3p0/2582	3	-1	1	20	1820	1838	UCCACAGCUUUCUUGAACUG	UAGUU-GAGGAAGCUGUGGA	Cleavage	0.677173424	0.045097284
gma-miR396h	transcript_HQ_CoA_transcript4026/f5p0/4108	3	-1	1	20	2837	2856	UCCACAGCUUUCUUGAACUG	AAGCUAAAGAAGGUUGUGGA	Cleavage		
gma-miR396h	transcript_HQ_CoA_transcript7563/f2p0/3463	3	-1	1	20	2184	2203	UCCACAGCUUUCUUGAACUG	AAGCUAAAGAAGGUUGUGGA	Cleavage		
gma-miR396h	transcript_HQ_CoA_transcript17868/f2p0/2352	3	-1	1	20	1899	1918	UCCACAGCUUUCUUGAACUG	GAUUUGAGGAAGGCUGUGGA	Cleavage	0.859509086	0.002974718
gma-miR408d	transcript_HQ_CoA_transcript15953/f3p0/2520	3	-1	1	20	1291	1310	UGCACUGCCUCUUCCCUGGC	CCUAGGGAAGAGGCAGUGAG	Cleavage		
gma-miR408d	transcript_HQ_CoA_transcript20676/f3p0/2160	3	-1	1	20	934	953	UGCACUGCCUCUUCCCUGGC	CCUAGGGAAGAGGCAGUGAG	Cleavage		
gma-miR408d	transcript_HQ_CoA_transcript24897/f6p0/1818	3	-1	1	20	1156	1175	UGCACUGCCUCUUCCCUGGC	GCAAGGGAAGAGGUAUUGCA	Cleavage		
gra-miR167c	transcript_HQ_CoA_transcript23455/f3p0/1937	3	-1	1	22	1056	1077	UCAGAUGAAGCUGCCAGCAUGA	CUAUGAUGGCGGCUUCAUUUGU	Cleavage		
gra-miR167c	transcript_HQ_CoA_transcript8568/f3p0/3311	3	-1	1	22	3073	3094	UCAGAUGAAGCUGCCAGCAUGA	CUCUGUUGGUGGUUUCAUCUGU	Cleavage		
gra-miR167c	transcript_HQ_CoA_transcript7868/f4p0/3396	3	-1	1	22	3151	3172	UCAGAUGAAGCUGCCAGCAUGA	CUCUGUUGGUGGUUUCAUCUGU	Cleavage		
gra-miR167c	transcript_HQ_CoA_transcript3413/f2p0/4329	3	-1	1	22	1977	1998	UCAGAUGAAGCUGCCAGCAUGA	CUCUGUUGGUGGUUUCAUCUGU	Cleavage		
gra-miR167c	transcript_HQ_CoA_transcript12790/f5p0/2781	3	-1	1	22	2445	2466	UCAGAUGAAGCUGCCAGCAUGA	UGUUGUUGGCAUCUUUAUUUGA	Translation		
gra-miR167c	transcript_HQ_CoA_transcript12713/f2p0/2834	3	-1	1	22	2468	2489	UCAGAUGAAGCUGCCAGCAUGA	UGUUGUUGGCAUCUUUAUUUGA	Translation	-0.775206599	0.014120388
gra-miR167c	transcript_HQ_CoA_transcript18990/f2p0/2285	3	-1	1	22	12	33	UCAGAUGAAGCUGCCAGCAUGA	GUUUUUUGGCGGUUUCAUCUGG	Cleavage		
gra-miR167c	transcript_HQ_CoA_transcript5883/f2p0/3706	3	-1	1	22	758	779	UCAGAUGAAGCUGCCAGCAUGA	CUUUUCUGGCAUUUUCAUCUGA	Translation		
gra-miR167c	transcript_HQ_CoA_transcript25232/f3p0/1872	3	-1	1	22	951	971	UCAGAUGAAGCUGCCAGCAUGA	GUUUGUUG-CAGCUUCAUUUGA	Cleavage		
hbr-miR156	transcript_HQ_CoA_transcript14698/f2p0/2635	3	-1	1	19	180	198	UUGACAGAAGAUAGAGAGC	CCUCUCAAUCUUCUGUCGA	Cleavage		
hbr-miR156	transcript_HQ_CoA_transcript20481/f2p0/2177	3	-1	1	19	1256	1274	UUGACAGAAGAUAGAGAGC	ACUUUCUAUCUUCUUUCAA	Cleavage		
hbr-miR156	transcript_HQ_CoA_transcript25141/f2p0/1836	3	-1	1	19	663	681	UUGACAGAAGAUAGAGAGC	ACUCUCUACUUUCUGUCAA	Translation	-0.696183884	0.037232581
hbr-miR156	transcript_HQ_CoA_transcript16040/f2p0/2524	3	-1	1	19	322	340	UUGACAGAAGAUAGAGAGC	GCUCUGUAUUUUUUGUUGA	Cleavage		
mtr-miR319a-3p	transcript_HQ_CoA_transcript14462/f14p0/2625	3	-1	1	20	134	153	UUGGACUGAAGGGAGCUCCC	UGGAGUUUCCUUCAAUUCAA	Cleavage	-0.896441087	0.001062031
mtr-miR319a-3p	transcript_HQ_CoA_transcript6680/f13p0/3559	3	-1	1	20	2301	2320	UUGGACUGAAGGGAGCUCCC	AGCAGCUUUCUUCAGUCCAU	Cleavage		
mtr-miR319a-3p	transcript_HQ_CoA_transcript20831/f3p0/2156	3	-1	1	20	1446	1465	UUGGACUGAAGGGAGCUCCC	AGGGGACCCCUUCAGUCCAG	Cleavage	-0.711412236	0.031618497
mtr-miR319a-3p	transcript_HQ_CoA_transcript17168/f2p0/2434	3	-1	1	20	1841	1860	UUGGACUGAAGGGAGCUCCC	AGGGGACCCCUUCAGUCCAG	Cleavage	-0.74228357	0.02199876
osa-miR159f	transcript_HQ_CoA_transcript12669/f6p0/2811	3	-1	1	21	113	133	CUUGGAUUGAAGGGAGCUCUA	UAGGGUUUCCUUCGAUCUAGG	Cleavage		
osa-miR159f	transcript_HQ_CoA_transcript12052/f2p0/2876	3	-1	1	21	238	258	CUUGGAUUGAAGGGAGCUCUA	UAGGGUUUCCUUCGAUCUAGG	Cleavage		
osa-miR159f	transcript_HQ_CoA_transcript10405/f2p0/3066	3	-1	1	21	1788	1808	CUUGGAUUGAAGGGAGCUCUA	CAGAGUUUCCUUUGAUCCAAA	Cleavage		
osa-miR159f	transcript_HQ_CoA_transcript7017/f3p0/3537	3	-1	1	21	542	562	CUUGGAUUGAAGGGAGCUCUA	UGGGCCUCUCUUCAGUUCAAG	Cleavage		
osa-miR159f	transcript_HQ_CoA_transcript5815/f6p0/3697	3	-1	1	21	647	667	CUUGGAUUGAAGGGAGCUCUA	UGGGCUUCCCUUCAGUUCAAG	Cleavage		
osa-miR159f	transcript_HQ_CoA_transcript18036/f2p0/2370	3	-1	1	21	362	382	CUUGGAUUGAAGGGAGCUCUA	AGGAGCUUCAUUCAAUCCAAU	Cleavage		
osa-miR159f	transcript_HQ_CoA_transcript7001/f2p0/3538	3	-1	1	21	320	340	CUUGGAUUGAAGGGAGCUCUA	AGGAGCUUCAUUCAAUCCAAU	Cleavage		
osa-miR159f	transcript_HQ_CoA_transcript17064/f2p0/2430	3	-1	1	21	84	104	CUUGGAUUGAAGGGAGCUCUA	UUGAGCUCCUUGCAAUCCAAA	Translation		
osa-miR159f	transcript_HQ_CoA_transcript17441/f2p0/2445	3	-1	1	21	27	47	CUUGGAUUGAAGGGAGCUCUA	UUGAGCUCCUUGCAAUCCAAA	Translation		
osa-miR159f	transcript_HQ_CoA_transcript96/f3p0/7811	3	-1	1	21	2036	2056	CUUGGAUUGAAGGGAGCUCUA	AGGAGCUCCCUUCAAUGGAAG	Cleavage		
osa-miR159f	transcript_HQ_CoA_transcript91/f2p0/7830	3	-1	1	21	1978	1998	CUUGGAUUGAAGGGAGCUCUA	AGGAGCUCCCUUCAAUGGAAG	Cleavage		
osa-miR159f	transcript_HQ_CoA_transcript25807/f3p0/1697	3	-1	1	21	751	771	CUUGGAUUGAAGGGAGCUCUA	UUGAGCUAUCUUCAUUCCAAG	Cleavage		
osa-miR159f	transcript_HQ_CoA_transcript12286/f4p0/2843	3	-1	1	21	1772	1792	CUUGGAUUGAAGGGAGCUCUA	ACCAGCACUCUUCAAUUCAAG	Cleavage		
osa-miR159f	transcript_HQ_CoA_transcript12207/f2p0/2868	3	-1	1	21	1783	1803	CUUGGAUUGAAGGGAGCUCUA	ACCAGCACUCUUCAAUUCAAG	Cleavage	0.832880636	0.005313433
osa-miR159f	transcript_HQ_CoA_transcript5639/f4p0/3770	3	-1	1	21	3179	3200	CUUGGAUUGAAGGGA-GCUCUA	CUGAGCAUUUCUUCAAUCCAAG	Cleavage		
osa-miR167d-5p	transcript_HQ_CoA_transcript24079/f2p0/1930	3	-1	1	21	1512	1532	UGAAGCUGCCAGCAUGAUCUG	UCGAUCAUGUUGGUAGAUUUA	Cleavage		
osa-miR395b	transcript_HQ_CoA_transcript12521/f2p0/2820	3	-1	1	21	2600	2620	GUGAAGUGUUUGGGGGAACUC	GGAUUCCUCUAAAUGCUUCAC	Cleavage		
osa-miR395b	transcript_HQ_CoA_transcript3453/f2p0/4272	3	-1	1	21	3551	3571	GUGAAGUGUUUGGGGGAACUC	GAGUUCAACCAGAUACUUCAC	Cleavage	0.754364138	0.018837488
osa-miR395b	transcript_HQ_CoA_transcript437/f2p0/6481	3	-1	1	21	3557	3577	GUGAAGUGUUUGGGGGAACUC	GAGUUCAACCAGAUACUUCAC	Cleavage		
osa-miR395b	transcript_HQ_CoA_transcript399/f2p0/6661	3	-1	1	21	3579	3599	GUGAAGUGUUUGGGGGAACUC	GAGUUCAACCAGAUACUUCAC	Cleavage	0.713694882	0.030827867
osa-miR395b	transcript_HQ_CoA_transcript367/f7p0/6733	3	-1	1	21	3651	3671	GUGAAGUGUUUGGGGGAACUC	GAGUUCAACCAGAUACUUCAC	Cleavage		
osa-miR395b	transcript_HQ_CoA_transcript21744/f2p0/2077	3	-1	1	21	146	166	GUGAAGUGUUUGGGGGAACUC	UUUAUCCCCCAAACACUUCAA	Cleavage		
osa-miR395b	transcript_HQ_CoA_transcript12565/f4p0/2800	3	-1	1	21	93	113	GUGAAGUGUUUGGGGGAACUC	UUUAUCCCCCAAACACUUCAA	Cleavage		
osa-miR408-3p	transcript_HQ_CoA_transcript3381/f3p0/4313	3	-1	1	21	2605	2625	CUGCACUGCCUCUUCCCUGGC	UAUAGAGAAGAGGCAGUGGAG	Cleavage		
ppt-miR319a	transcript_HQ_CoA_transcript8272/f4p0/3307	3	-1	1	20	3245	3264	CUUGGACUGAAGGGAGCUCC	UGAGCUCAUUUUAGUUCAAG	Cleavage		
ppt-miR319a	transcript_HQ_CoA_transcript25807/f3p0/1697	3	-1	1	20	752	771	CUUGGACUGAAGGGAGCUCC	UGAGCUAUCUUCAUUCCAAG	Cleavage		
ppt-miR408b	transcript_HQ_CoA_transcript15953/f3p0/2520	3	-1	1	21	1290	1310	UGCACUGCCUCUUCCCUGGCU	ACCUAGGGAAGAGGCAGUGAG	Cleavage		
ppt-miR408b	transcript_HQ_CoA_transcript20676/f3p0/2160	3	-1	1	21	933	953	UGCACUGCCUCUUCCCUGGCU	ACCUAGGGAAGAGGCAGUGAG	Cleavage		
ppt-miR408b	transcript_HQ_CoA_transcript24897/f6p0/1818	3	-1	1	21	1155	1175	UGCACUGCCUCUUCCCUGGCU	UGCAAGGGAAGAGGUAUUGCA	Cleavage		
pta-miR319	transcript_HQ_CoA_transcript14462/f14p0/2625	3	-1	1	19	135	153	UUGGACUGAAGGGAGCUCC	GGAGUUUCCUUCAAUUCAA	Cleavage	-0.887889668	0.001389906
pta-miR319	transcript_HQ_CoA_transcript6680/f13p0/3559	3	-1	1	19	2302	2320	UUGGACUGAAGGGAGCUCC	GCAGCUUUCUUCAGUCCAU	Cleavage		
pta-miR319	transcript_HQ_CoA_transcript20831/f3p0/2156	3	-1	1	19	1447	1465	UUGGACUGAAGGGAGCUCC	GGGGACCCCUUCAGUCCAG	Cleavage	-0.755187348	0.018633782
pta-miR319	transcript_HQ_CoA_transcript17168/f2p0/2434	3	-1	1	19	1842	1860	UUGGACUGAAGGGAGCUCC	GGGGACCCCUUCAGUCCAG	Cleavage	-0.760647534	0.017319581
ptc-miR167f-5p	transcript_HQ_CoA_transcript24079/f2p0/1930	3	-1	1	21	1512	1532	UGAAGCUGCCAGCAUGAUCUU	UCGAUCAUGUUGGUAGAUUUA	Cleavage		
stu-miR408b-5p	transcript_HQ_CoA_transcript3296/f3p0/4357	3	-1	1	21	965	985	ACGGGGACGAGACAGAGCAUG	CUUGCUCUGUUUUGUCUCUGA	Cleavage		
tae-miR1122c-3p	transcript_HQ_CoA_transcript3186/f2p0/4357	3	-1	1	21	3736	3756	UCUAAUAUUAUGGGACGGAGG	CCUCCGUCUCAAAAUAUUAGU	Translation		
tae-miR1122c-3p	transcript_HQ_CoA_transcript11227/f5p0/2963	3	-1	1	21	1430	1450	UCUAAUAUUAUGGGACGGAGG	CUUUCGUCCCAAAAUAUUAGU	Translation		
tae-miR1122c-3p	transcript_HQ_CoA_transcript5862/f2p0/3739	3	-1	1	21	931	951	UCUAAUAUUAUGGGACGGAGG	AAUCUGUUUCAUUAUAUUAGA	Cleavage		
tae-miR395b	transcript_HQ_CoA_transcript21744/f2p0/2077	3	-1	1	20	146	165	UGAAGUGUUUGGGGGAACUC	UUUAUCCCCCAAACACUUCA	Cleavage	0.71732101	0.02959863
tae-miR395b	transcript_HQ_CoA_transcript12565/f4p0/2800	3	-1	1	20	93	112	UGAAGUGUUUGGGGGAACUC	UUUAUCCCCCAAACACUUCA	Cleavage	0.946165957	0.000113054

tae-miR395b	transcript_HQ_CoA_transcript4794/f6p0/3911	3	-1	1	20	1207	1226	UGAAGUGUUUGGGGGAACUC	GAGUUUGCUCAAGCAUUUCA	Cleavage		
tae-miR395b	transcript_HQ_CoA_transcript12433/f2p0/2856	3	-1	1	20	641	660	UGAAGUGUUUGGGGGAACUC	UUGUUCCCUCAAAUGCUUCG	Cleavage		
tae-miR395b	transcript_HQ_CoA_transcript9792/f9p0/3096	3	-1	1	20	121	140	UGAAGUGUUUGGGGGAACUC	AAGUUUCCCUCAAUACUUCA	Translation		
tae-miR395b	transcript_HQ_CoA_transcript21096/f3p0/2132	3	-1	1	20	29	48	UGAAGUGUUUGGGGGAACUC	AAGGUCCCCCAAAUGUUUCG	Cleavage		
tae-miR395b	transcript_HQ_CoA_transcript8877/f19p0/3234	3	-1	1	20	2255	2274	UGAAGUGUUUGGGGGAACUC	CAGUACCCCCAAAUAUUUUG	Cleavage		
tae-miR395b	transcript_HQ_CoA_transcript7752/f34p0/3342	3	-1	1	20	2254	2273	UGAAGUGUUUGGGGGAACUC	CAGUACCCCCAAAUAUUUUG	Cleavage		
tae-miR395b	transcript_HQ_CoA_transcript1126/f2p0/5608	3	-1	1	20	2409	2428	UGAAGUGUUUGGGGGAACUC	CAGUACCCCCAAAUAUUUUG	Cleavage		
vvi-miR167c	transcript_HQ_CoA_transcript24079/f2p0/1930	3	-1	1	21	1512	1532	UGAAGCUGCCAGCAUGAUCUC	UCGAUCAUGUUGGUAGAUUUA	Cleavage		
vvi-miR396a	transcript_HQ_CoA_transcript19242/f4p0/2213	3	-1	1	21	997	1018	UUCCACA-GCUUUCUUGAACUA	UCGUUCAAGAAAGCCUGUGGAA	Cleavage	-0.712038553	0.031400262
vvi-miR396a	transcript_HQ_CoA_transcript19308/f8p0/2200	3	-1	1	21	890	911	UUCCACA-GCUUUCUUGAACUA	UCGUUCAAGAAAGCCUGUGGAA	Cleavage	-0.787056292	0.011829225
vvi-miR396a	transcript_HQ_CoA_transcript20070/f3p0/2193	3	-1	1	21	951	972	UUCCACA-GCUUUCUUGAACUA	UCGUUCAAGAAAGCCUGUGGAA	Cleavage	-0.704701827	0.034018969
vvi-miR396a	transcript_HQ_CoA_transcript20608/f3p0/2171	3	-1	1	21	1038	1059	UUCCACA-GCUUUCUUGAACUA	UCGUUCAAGAAAGCCUGUGGAA	Cleavage	-0.688509368	0.040290857
vvi-miR396a	transcript_HQ_CoA_transcript19820/f2p0/2215	3	-1	1	21	512	533	UUCCACA-GCUUUCUUGAACUA	CCGUUCAAGAAAGCCUGUGGAA	Cleavage	-0.723651616	0.027530261
vvi-miR396a	transcript_HQ_CoA_transcript20271/f2p0/2193	3	-1	1	21	343	364	UUCCACA-GCUUUCUUGAACUA	CCGUUCAAGAAAGCCUGUGGAA	Cleavage		
vvi-miR396a	transcript_HQ_CoA_transcript21509/f4p0/2050	3	-1	1	21	373	394	UUCCACA-GCUUUCUUGAACUA	CCGUUCAAGAAAGCCUGUGGAA	Cleavage	-0.683044267	0.042564554
vvi-miR396a	transcript_HQ_CoA_transcript27471/f2p0/1641	3	-1	1	21	429	450	UUCCACA-GCUUUCUUGAACUA	CCGUUCAAGAAAGCCUGUGGAA	Cleavage	-0.736352442	0.023671432
vvi-miR396a	transcript_HQ_CoA_transcript27685/f2p0/1608	3	-1	1	21	509	530	UUCCACA-GCUUUCUUGAACUA	CCGUUCAAGAAAGCCUGUGGAA	Cleavage	-0.888261193	0.001374369
vvi-miR396a	transcript_HQ_CoA_transcript27879/f3p0/1596	3	-1	1	21	544	565	UUCCACA-GCUUUCUUGAACUA	CCGUUCAAGAAAGCCUGUGGAA	Cleavage		
vvi-miR396a	transcript_HQ_CoA_transcript27832/f2p0/1591	3	-1	1	21	492	513	UUCCACA-GCUUUCUUGAACUA	CCGUUCAAGAAAGCCUGUGGAA	Cleavage	-0.920942909	0.000423125
vvi-miR396a	transcript_HQ_CoA_transcript28053/f3p0/1571	3	-1	1	21	536	557	UUCCACA-GCUUUCUUGAACUA	CCGUUCAAGAAAGCCUGUGGAA	Cleavage	-0.858552934	0.003043202
vvi-miR396a	transcript_HQ_CoA_transcript29242/f2p0/1411	3	-1	1	21	470	491	UUCCACA-GCUUUCUUGAACUA	CCGUUCAAGAAAGCCUGUGGAA	Cleavage	-0.918405712	0.000471391
vvi-miR396a	transcript_HQ_CoA_transcript29824/f2p0/1232	3	-1	1	21	342	363	UUCCACA-GCUUUCUUGAACUA	CCGUUCAAGAAAGCCUGUGGAA	Cleavage		
vvi-miR396a	transcript_HQ_CoA_transcript16187/f3p0/2582	3	-1	1	21	1820	1839	UUCCACAGCUUUCUUGAACUA	UAGUU-GAGGAAGCUGUGGAA	Cleavage	0.719652503	0.028825453
vvi-miR396a	transcript_HQ_CoA_transcript5396/f2p0/3810	3	-1	1	21	1039	1059	UUCCACAGCUUUCUUGAACUA	GGGUUCAGGAAAGUUGUUGGA	Cleavage		
vvi-miR396a	transcript_HQ_CoA_transcript8702/f2p0/3286	3	-1	1	21	2697	2717	UUCCACAGCUUUCUUGAACUA	GAGUUCAUGGAAGCUGUAGAA	Cleavage		
vvi-miR396a	transcript_HQ_CoA_transcript18586/f2p0/2310	3	-1	1	21	1606	1626	UUCCACAGCUUUCUUGAACUA	AAGAUCAAGAAAGUGGUGGAA	Cleavage		
vvi-miR396a	transcript_HQ_CoA_transcript25598/f3p0/1743	3	-1	1	21	688	708	UUCCACAGCUUUCUUGAACUA	CAGGUCAAGAAAGCUAUGGAG	Cleavage	0.808133538	0.008396842
vvi-miR396a	transcript_HQ_CoA_transcript26456/f33p0/1704	3	-1	1	21	688	708	UUCCACAGCUUUCUUGAACUA	CAGGUCAAGAAAGCUAUGGAG	Cleavage		
vvi-miR396a	transcript_HQ_CoA_transcript4298/f2p0/4029	3	-1	1	21	2151	2171	UUCCACAGCUUUCUUGAACUA	AUUUUCAGGAAGGUUGUGGAG	Cleavage		
vvi-miR396a	transcript_HQ_CoA_transcript16798/f2p0/2437	3	-1	1	21	1376	1396	UUCCACAGCUUUCUUGAACUA	ACAUUCAGGAAAGCUCUGGAA	Cleavage		
vvi-miR396a	transcript_HQ_CoA_transcript976/f3p0/5771	3	-1	1	21	3270	3290	UUCCACAGCUUUCUUGAACUA	GACUCCAAGAAAGCUGUGGAU	Cleavage		
vvi-miR396a	transcript_HQ_CoA_transcript1778/f2p0/5116	3	-1	1	21	275	295	UUCCACAGCUUUCUUGAACUA	GACUCCAAGAAAGCUGUGGAU	Cleavage		
vvi-miR396a	transcript_HQ_CoA_transcript16411/f2p0/2480	3	-1	1	21	613	633	UUCCACAGCUUUCUUGAACUA	GCGUUCAAGAAGGUUGUGAGA	Cleavage	0.715274331	0.030288425
vvi-miR396a	transcript_HQ_CoA_transcript17868/f2p0/2352	3	-1	1	21	1899	1919	UUCCACAGCUUUCUUGAACUA	GAUUUGAGGAAGGCUGUGGAA	Cleavage	0.881938069	0.001655731
vvi-miR396a	transcript_HQ_CoA_transcript1910/f2p0/4979	3	-1	1	21	2263	2283	UUCCACAGCUUUCUUGAACUA	UUGUCUGGGAAAGCUGUGGGA	Cleavage		
vvi-miR396a	transcript_HQ_CoA_transcript8140/f2p0/3346	3	-1	1	21	446	466	UUCCACAGCUUUCUUGAACUA	UUGCUCAAGAACGCUGUGGAG	Translation	0.71919003	0.028977756
vvi-miR396a	transcript_HQ_CoA_transcript8912/f2p0/3245	3	-1	1	21	428	448	UUCCACAGCUUUCUUGAACUA	UUGCUCAAGAACGCUGUGGAG	Translation	0.845988188	0.004046606
vvi-miR396a	transcript_HQ_CoA_transcript9028/f2p0/3240	3	-1	1	21	355	375	UUCCACAGCUUUCUUGAACUA	UUGCUCAAGAACGCUGUGGAG	Translation	0.685555545	0.041509773
vvi-miR396a	transcript_HQ_CoA_transcript10590/f3p0/3032	3	-1	1	21	349	369	UUCCACAGCUUUCUUGAACUA	UUGCUCAAGAACGCUGUGGAG	Translation	0.873853214	0.00207063
vvi-miR396a	transcript_HQ_CoA_transcript15769/f15p0/2406	3	-1	1	21	342	362	UUCCACAGCUUUCUUGAACUA	UCGUUGAAGGAAGCUCUGGAA	Cleavage	-0.826095527	0.006064836
vvi-miR396a	transcript_HQ_CoA_transcript1328/f2p0/5403	3	-1	1	21	2238	2258	UUCCACAGCUUUCUUGAACUA	CUGUCUGGGAAAGCUGUGGGA	Cleavage		
vvi-miR396a	transcript_HQ_CoA_transcript1711/f8p0/5021	3	-1	1	21	2230	2250	UUCCACAGCUUUCUUGAACUA	CUGUCUGGGAAAGCUGUGGGA	Cleavage		
vvi-miR396a	transcript_HQ_CoA_transcript1959/f2p0/4936	3	-1	1	21	2324	2344	UUCCACAGCUUUCUUGAACUA	CUGUCUGGGAAAGCUGUGGGA	Cleavage		
vvi-miR396a	transcript_HQ_CoA_transcript1986/f2p0/4924	3	-1	1	21	2306	2326	UUCCACAGCUUUCUUGAACUA	CUGUCUGGGAAAGCUGUGGGA	Cleavage		
vvi-miR396a	transcript_HQ_CoA_transcript1991/f20p0/4837	3	-1	1	21	2230	2250	UUCCACAGCUUUCUUGAACUA	CUGUCUGGGAAAGCUGUGGGA	Cleavage		
vvi-miR396a	transcript_HQ_CoA_transcript2158/f3p0/4811	3	-1	1	21	2249	2269	UUCCACAGCUUUCUUGAACUA	CUGUCUGGGAAAGCUGUGGGA	Cleavage	-0.90766539	0.000718899
vvi-miR396b	transcript_HQ_CoA_transcript19820/f2p0/2215	3	-1	1	20	513	533	UUCCACA-GCUUUCUUGAACU	CGUUCAAGAAAGCCUGUGGAA	Cleavage	-0.722785081	0.0278076
vvi-miR396b	transcript_HQ_CoA_transcript19242/f4p0/2213	3	-1	1	20	998	1018	UUCCACA-GCUUUCUUGAACU	CGUUCAAGAAAGCCUGUGGAA	Cleavage	-0.712386671	0.031279389
vvi-miR396b	transcript_HQ_CoA_transcript19308/f8p0/2200	3	-1	1	20	891	911	UUCCACA-GCUUUCUUGAACU	CGUUCAAGAAAGCCUGUGGAA	Cleavage	-0.768287685	0.015586441
vvi-miR396b	transcript_HQ_CoA_transcript20271/f2p0/2193	3	-1	1	20	344	364	UUCCACA-GCUUUCUUGAACU	CGUUCAAGAAAGCCUGUGGAA	Cleavage		
vvi-miR396b	transcript_HQ_CoA_transcript20070/f3p0/2193	3	-1	1	20	952	972	UUCCACA-GCUUUCUUGAACU	CGUUCAAGAAAGCCUGUGGAA	Cleavage	-0.716027636	0.03003333
vvi-miR396b	transcript_HQ_CoA_transcript20608/f3p0/2171	3	-1	1	20	1039	1059	UUCCACA-GCUUUCUUGAACU	CGUUCAAGAAAGCCUGUGGAA	Cleavage	-0.685959476	0.041341704
vvi-miR396b	transcript_HQ_CoA_transcript21509/f4p0/2050	3	-1	1	20	374	394	UUCCACA-GCUUUCUUGAACU	CGUUCAAGAAAGCCUGUGGAA	Cleavage	-0.708709429	0.03257161
vvi-miR396b	transcript_HQ_CoA_transcript27471/f2p0/1641	3	-1	1	20	430	450	UUCCACA-GCUUUCUUGAACU	CGUUCAAGAAAGCCUGUGGAA	Cleavage	-0.7495149	0.020067501
vvi-miR396b	transcript_HQ_CoA_transcript27685/f2p0/1608	3	-1	1	20	510	530	UUCCACA-GCUUUCUUGAACU	CGUUCAAGAAAGCCUGUGGAA	Cleavage	-0.847142308	0.003946143
vvi-miR396b	transcript_HQ_CoA_transcript27879/f3p0/1596	3	-1	1	20	545	565	UUCCACA-GCUUUCUUGAACU	CGUUCAAGAAAGCCUGUGGAA	Cleavage	-0.676765952	0.045276571
vvi-miR396b	transcript_HQ_CoA_transcript27832/f2p0/1591	3	-1	1	20	493	513	UUCCACA-GCUUUCUUGAACU	CGUUCAAGAAAGCCUGUGGAA	Cleavage	-0.893860029	0.001154583
vvi-miR396b	transcript_HQ_CoA_transcript28053/f3p0/1571	3	-1	1	20	537	557	UUCCACA-GCUUUCUUGAACU	CGUUCAAGAAAGCCUGUGGAA	Cleavage	-0.841195067	0.004482626
vvi-miR396b	transcript_HQ_CoA_transcript29242/f2p0/1411	3	-1	1	20	471	491	UUCCACA-GCUUUCUUGAACU	CGUUCAAGAAAGCCUGUGGAA	Cleavage	-0.898398639	0.000995376
vvi-miR396b	transcript_HQ_CoA_transcript29824/f2p0/1232	3	-1	1	20	343	363	UUCCACA-GCUUUCUUGAACU	CGUUCAAGAAAGCCUGUGGAA	Cleavage		
vvi-miR396b	transcript_HQ_CoA_transcript5396/f2p0/3810	3	-1	1	20	1040	1059	UUCCACAGCUUUCUUGAACU	GGUUCAGGAAAGUUGUUGGA	Cleavage		
vvi-miR396b	transcript_HQ_CoA_transcript8702/f2p0/3286	3	-1	1	20	2698	2717	UUCCACAGCUUUCUUGAACU	AGUUCAUGGAAGCUGUAGAA	Cleavage		
vvi-miR396b	transcript_HQ_CoA_transcript18586/f2p0/2310	3	-1	1	20	1607	1626	UUCCACAGCUUUCUUGAACU	AGAUCAAGAAAGUGGUGGAA	Cleavage		
vvi-miR396b	transcript_HQ_CoA_transcript25598/f3p0/1743	3	-1	1	20	689	708	UUCCACAGCUUUCUUGAACU	AGGUCAAGAAAGCUAUGGAG	Cleavage	0.819025755	0.006921556
vvi-miR396b	transcript_HQ_CoA_transcript26456/f33p0/1704	3	-1	1	20	689	708	UUCCACAGCUUUCUUGAACU	AGGUCAAGAAAGCUAUGGAG	Cleavage		
vvi-miR396b	transcript_HQ_CoA_transcript4298/f2p0/4029	3	-1	1	20	2152	2171	UUCCACAGCUUUCUUGAACU	UUUUCAGGAAGGUUGUGGAG	Cleavage		

vvi-miR396b	transcript_HQ_CoA_transcript16798/f2p0/2437	3	-1	1	20	1377	1396	UUCCACAGCUUUCUUGAACU	CAUUCAGGAAAGCUCUGGAA	Cleavage		
vvi-miR396b	transcript_HQ_CoA_transcript976/f3p0/5771	3	-1	1	20	3271	3290	UUCCACAGCUUUCUUGAACU	ACUCCAAGAAAGCUGUGGAU	Cleavage		
vvi-miR396b	transcript_HQ_CoA_transcript1778/f2p0/5116	3	-1	1	20	276	295	UUCCACAGCUUUCUUGAACU	ACUCCAAGAAAGCUGUGGAU	Cleavage		
vvi-miR396b	transcript_HQ_CoA_transcript16411/f2p0/2480	3	-1	1	20	614	633	UUCCACAGCUUUCUUGAACU	CGUUCAAGAAGGUUGUGAGA	Cleavage	0.77017364	0.015177208
vvi-miR396b	transcript_HQ_CoA_transcript17868/f2p0/2352	3	-1	1	20	1900	1919	UUCCACAGCUUUCUUGAACU	AUUUGAGGAAGGCUGUGGAA	Cleavage	0.888373036	0.001369715
vvi-miR396b	transcript_HQ_CoA_transcript1328/f2p0/5403	3	-1	1	20	2239	2258	UUCCACAGCUUUCUUGAACU	UGUCUGGGAAAGCUGUGGGA	Cleavage		
vvi-miR396b	transcript_HQ_CoA_transcript1711/f8p0/5021	3	-1	1	20	2231	2250	UUCCACAGCUUUCUUGAACU	UGUCUGGGAAAGCUGUGGGA	Cleavage		
vvi-miR396b	transcript_HQ_CoA_transcript1910/f2p0/4979	3	-1	1	20	2264	2283	UUCCACAGCUUUCUUGAACU	UGUCUGGGAAAGCUGUGGGA	Cleavage		
vvi-miR396b	transcript_HQ_CoA_transcript1959/f2p0/4936	3	-1	1	20	2325	2344	UUCCACAGCUUUCUUGAACU	UGUCUGGGAAAGCUGUGGGA	Cleavage		
vvi-miR396b	transcript_HQ_CoA_transcript1986/f2p0/4924	3	-1	1	20	2307	2326	UUCCACAGCUUUCUUGAACU	UGUCUGGGAAAGCUGUGGGA	Cleavage		
vvi-miR396b	transcript_HQ_CoA_transcript1991/f20p0/4837	3	-1	1	20	2231	2250	UUCCACAGCUUUCUUGAACU	UGUCUGGGAAAGCUGUGGGA	Cleavage		
vvi-miR396b	transcript_HQ_CoA_transcript2158/f3p0/4811	3	-1	1	20	2250	2269	UUCCACAGCUUUCUUGAACU	UGUCUGGGAAAGCUGUGGGA	Cleavage	-0.900854993	0.000915931
vvi-miR396b	transcript_HQ_CoA_transcript8140/f2p0/3346	3	-1	1	20	447	466	UUCCACAGCUUUCUUGAACU	UGCUCAAGAACGCUGUGGAG	Translation	0.682960662	0.042599963
vvi-miR396b	transcript_HQ_CoA_transcript8912/f2p0/3245	3	-1	1	20	429	448	UUCCACAGCUUUCUUGAACU	UGCUCAAGAACGCUGUGGAG	Translation	0.846778334	0.003977639
vvi-miR396b	transcript_HQ_CoA_transcript9028/f2p0/3240	3	-1	1	20	356	375	UUCCACAGCUUUCUUGAACU	UGCUCAAGAACGCUGUGGAG	Translation	0.703430423	0.034486722
vvi-miR396b	transcript_HQ_CoA_transcript10590/f3p0/3032	3	-1	1	20	350	369	UUCCACAGCUUUCUUGAACU	UGCUCAAGAACGCUGUGGAG	Translation	0.882970683	0.00160728
vvi-miR396b	transcript_HQ_CoA_transcript15769/f15p0/2406	3	-1	1	20	343	362	UUCCACAGCUUUCUUGAACU	CGUUGAAGGAAGCUCUGGAA	Cleavage	-0.80714336	0.008540662
zma-miR162-3p	transcript_HQ_CoA_transcript1140/f2p0/5609	3	-1	1	20	2525	2545	UCGAUAA-ACCUCUGCAUCCA	UGGAUGCAGAGGUGUUAUCGA	Cleavage		
zma-miR162-3p	transcript_HQ_CoA_transcript236/f2p0/7098	3	-1	1	20	3904	3924	UCGAUAA-ACCUCUGCAUCCA	UGGAUGCAGAGGUGUUAUCGA	Cleavage		
zma-miR162-3p	transcript_HQ_CoA_transcript23140/f4p0/1976	3	-1	1	20	507	526	UCGAUAAACCUCUGCAUCCA	UGGAGGUAGAGGUUUCUCGA	Cleavage	-0.727107593	0.026442209
zma-miR171a-3p	transcript_HQ_CoA_transcript2950/f2p0/4495	3	-1	1	20	2793	2812	UGAUUGAGCCGCGCCAAUAU	GAAUUGGCGUGGUUUGAUCA	Cleavage	-0.887778985	0.001394559
zma-miR171a-3p	transcript_HQ_CoA_transcript13232/f2p0/2782	3	-1	1	20	1627	1646	UGAUUGAGCCGCGCCAAUAU	CUAUUGUUGCGGUUCAGUUA	Cleavage		
zma-miR396g-3p	transcript_HQ_CoA_transcript274/f2p0/6978	3	-1	1	21	4218	4238	GUUCAAGAAAGCUGUGGAAGA	UCUUCCACAGCUUGCUUGGAA	Cleavage		
zma-miR396g-3p	transcript_HQ_CoA_transcript229/f17p0/7102	3	-1	1	21	4359	4379	GUUCAAGAAAGCUGUGGAAGA	UCUUCCACAGCUUGCUUGGAA	Cleavage		
zma-miR396g-3p	transcript_HQ_CoA_transcript218/f2p0/7216	3	-1	1	21	4421	4441	GUUCAAGAAAGCUGUGGAAGA	UCUUCCACAGCUUGCUUGGAA	Cleavage		
zma-miR396g-3p	transcript_HQ_CoA_transcript134/f2p0/7609	3	-1	1	21	5304	5324	GUUCAAGAAAGCUGUGGAAGA	UCUUCCACAGCUUGCUUGGAA	Cleavage		
zma-miR396g-3p	transcript_HQ_CoA_transcript4834/f2p0/3940	3	-1	1	21	3050	3070	GUUCAAGAAAGCUGUGGAAGA	UCUUUCACGGCUCUCUUGGAC	Cleavage		
zma-miR396g-3p	transcript_HQ_CoA_transcript4653/f2p0/3988	3	-1	1	21	3116	3136	GUUCAAGAAAGCUGUGGAAGA	UCUUUCACGGCUCUCUUGGAC	Cleavage		
zma-miR396g-3p	transcript_HQ_CoA_transcript306/f2p0/6891	3	-1	1	21	30	50	GUUCAAGAAAGCUGUGGAAGA	UCUUCUACAGUUUCCUUGAAU	Cleavage		
zma-miR396g-3p	transcript_HQ_CoA_transcript22587/f2p0/2013	3	-1	1	21	1069	1089	GUUCAAGAAAGCUGUGGAAGA	UCUUUCAAAGCUUUCAUGAAC	Cleavage		
zma-miR396g-3p	transcript_HQ_CoA_transcript21757/f2p0/2038	3	-1	1	21	723	743	GUUCAAGAAAGCUGUGGAAGA	CCUUCCAUUGCUUUCUUGGAU	Cleavage		
zma-miR396g-3p	transcript_HQ_CoA_transcript18752/f13p0/2197	3	-1	1	21	787	807	GUUCAAGAAAGCUGUGGAAGA	CCUUCCAUUGCUUUCUUGGAU	Cleavage		
zma-miR396g-3p	transcript_HQ_CoA_transcript20300/f3p0/2202	3	-1	1	21	724	744	GUUCAAGAAAGCUGUGGAAGA	CCUUCCAUUGCUUUCUUGGAU	Cleavage		
zma-miR396g-3p	transcript_HQ_CoA_transcript17110/f2p0/2441	3	-1	1	21	1017	1037	GUUCAAGAAAGCUGUGGAAGA	CCUUCCAUUGCUUUCUUGGAU	Cleavage		
zma-miR396g-3p	transcript_HQ_CoA_transcript15449/f51p0/2471	3	-1	1	21	722	742	GUUCAAGAAAGCUGUGGAAGA	CCUUCCAUUGCUUUCUUGGAU	Cleavage		
zma-miR396g-3p	transcript_HQ_CoA_transcript15761/f5p0/2477	3	-1	1	21	1066	1086	GUUCAAGAAAGCUGUGGAAGA	CCUUCCAUUGCUUUCUUGGAU	Cleavage	0.707633324	0.032956231
zma-miR396g-3p	transcript_HQ_CoA_transcript14234/f4p0/2651	3	-1	1	21	715	735	GUUCAAGAAAGCUGUGGAAGA	CCUUCCAUUGCUUUCUUGGAU	Cleavage		
zma-miR396g-3p	transcript_HQ_CoA_transcript12495/f10p0/2815	3	-1	1	21	1066	1086	GUUCAAGAAAGCUGUGGAAGA	CCUUCCAUUGCUUUCUUGGAU	Cleavage		
zma-miR396g-3p	transcript_HQ_CoA_transcript4581/f3p0/3951	3	-1	1	21	3621	3641	GUUCAAGAAAGCUGUGGAAGA	GCUUUCACAGCUUUCGUGGAU	Cleavage	0.800032194	0.009622826
zma-miR396g-3p	transcript_HQ_CoA_transcript25746/f2p0/1824	3	-1	1	21	124	144	GUUCAAGAAAGCUGUGGAAGA	ACUUCCAGAGCUUUCUCGAGC	Cleavage	-0.812968144	0.00771814
zma-miR396g-3p	transcript_HQ_CoA_transcript22887/f4p0/1953	3	-1	1	21	164	184	GUUCAAGAAAGCUGUGGAAGA	ACUUCCAGAGCUUUCUCGAGC	Cleavage		
zma-miR396g-3p	transcript_HQ_CoA_transcript20970/f2p0/2232	3	-1	1	21	1980	2000	GUUCAAGAAAGCUGUGGAAGA	GAUUCUGCAGUUUUUUUGAGU	Cleavage		
zma-miR396g-3p	transcript_HQ_CoA_transcript15992/f2p0/2516	3	-1	1	21	2311	2331	GUUCAAGAAAGCUGUGGAAGA	GAUUCUGCAGUUUUUUUGAGU	Cleavage		
zma-miR396g-3p	transcript_HQ_CoA_transcript6995/f3p0/3533	3	-1	1	21	1853	1873	GUUCAAGAAAGCUGUGGAAGA	GUUUCCUUGGUUUUCUUGAAU	Cleavage	0.873932893	0.002066224

Supplementary Table S11. The differently accumulated lncRNAs predicted as targets of differently accumulated miRNAs

miRNA	Target lncRNA	Expectation	UPE$	miRNA_start	miRNA_end	Target_start	Target_end	miRNA_aligned_fragment	Target_aligned_fragment	Inhibition	correlation_coefficient	p-value
ath-miR157a-5p	CoA12874_2812	4	-1	1	21	92	112	UUGACAGAAGAUAGAGAGCAC	GAUCUCUCUAUCUUCUAUCCA	Cleavage	-0.650340408	0.057897471
ath-miR157a-5p	CoA13009_2807	4	-1	1	21	204	224	UUGACAGAAGAUAGAGAGCAC	GAUCUCUCUAUCUUCUAUCCA	Cleavage	-0.653779725	0.056142257
ath-miR157a-5p	CoA22190_2042	4	-1	1	21	170	190	UUGACAGAAGAUAGAGAGCAC	GAUCUCUCUAUCUUCUAUCCA	Cleavage	-0.426775273	0.251964954
ath-miR157a-5p	CoA20948_2138	4	-1	1	21	214	234	UUGACAGAAGAUAGAGAGCAC	GAUCUCUCUAUCUUCUAUCCA	Cleavage	-0.81221107	0.007821869
bra-miR408-5p	CoA29019_1431	4	-1	1	21	88	108	GGGAGCCAGGGAAGAGGCAGU	ACUUCCUCUUCUUUCGCUUCC	Cleavage	-0.707409067	0.033036755
gra-miR167c	CoA24164_1895	4	-1	1	22	1176	1197	UCAGAUGAAGCUGCCAGCAUGA	CCUUUUUGGCGGUUUUGUUUGA	Cleavage	-0.443533802	0.231766069
hbr-miR156	CoA13009_2807	4	-1	1	19	206	224	UUGACAGAAGAUAGAGAGC	UCUCUCUAUCUUCUAUCCA	Cleavage	-0.653779725	0.056142257
hbr-miR156	CoA22190_2042	4	-1	1	19	172	190	UUGACAGAAGAUAGAGAGC	UCUCUCUAUCUUCUAUCCA	Cleavage	-0.426775273	0.251964954
hbr-miR156	CoA12874_2812	4	-1	1	19	94	112	UUGACAGAAGAUAGAGAGC	UCUCUCUAUCUUCUAUCCA	Cleavage	-0.650340408	0.057897471
hbr-miR156	CoA20948_2138	4	-1	1	19	216	234	UUGACAGAAGAUAGAGAGC	UCUCUCUAUCUUCUAUCCA	Cleavage	-0.81221107	0.007821869
stu-miR408b-5p	CoA28144_1561	4	-1	1	21	930	950	ACGGGGACGAGACAGAGCAUG	UUUGUUUUGUUUUGUUUUUGU	Cleavage	-0.462014163	0.210551035
ath-miR157a-5p	CoA3445_4292	4.5	-1	1	21	2626	2646	UUGACAGAAGAUAGAGAGCAC	UUGCCCUCUAUUUUCUGCUAU	Cleavage	-0.616908297	0.076780075
ath-miR157a-5p	CoA5789_3732	4.5	-1	1	21	1138	1158	UUGACAGAAGAUAGAGAGCAC	UUAUUUUUUAUUUUUUGUUGA	Cleavage	-0.347482617	0.359527955
ath-miR157a-5p	CoA11478_2967	4.5	-1	1	21	581	601	UUGACAGAAGAUAGAGAGCAC	UUAUUUUUUAUUUUUUGUUGA	Cleavage	-0.316752562	0.406262664
ath-miR157a-5p	CoA11879_2942	4.5	-1	1	21	2747	2767	UUGACAGAAGAUAGAGAGCAC	CAGCUCUUUAAGUUCUGUUAG	Translation	-0.11082866	0.77651998
ath-miR159a	CoA3445_4292	4.5	-1	1	21	1472	1492	UUUGGAUUGAAGGGAGCUCUA	GGCAGCUUCUUUGAAUUUAAA	Cleavage	0.968464265	1.7781E-05
ath-miR159a	CoA18346_2326	4.5	-1	1	21	1944	1964	UUUGGAUUGAAGGGAGCUCUA	AUCGGCUCCUUUUUAUUCAAA	Cleavage	0.638531769	0.064186607
ath-miR159a	CoA18663_2328	4.5	-1	1	21	931	951	UUUGGAUUGAAGGGAGCUCUA	CUGGCUUGUCUUUGAUCCAAA	Cleavage	0.562364789	0.114990906
ath-miR159c	CoA3445_4292	4.5	-1	1	21	1472	1492	UUUGGAUUGAAGGGAGCUCCU	GGCAGCUUCUUUGAAUUUAAA	Cleavage	0.600205671	0.087485601
ath-miR159c	CoA18346_2326	4.5	-1	1	21	1944	1964	UUUGGAUUGAAGGGAGCUCCU	AUCGGCUCCUUUUUAUUCAAA	Cleavage	0.967026727	2.07539E-05
ath-miR159c	CoA18663_2328	4.5	-1	1	21	931	951	UUUGGAUUGAAGGGAGCUCCU	CUGGCUUGUCUUUGAUCCAAA	Cleavage	0.657263343	0.054399226
ath-miR167a-5p	CoA18138_2358	4.5	-1	1	21	439	459	UGAAGCUGCCAGCAUGAUCUA	AUGGUUGUGUUGUUAGCUUCG	Cleavage	0.32076014	0.400017001
ath-miR167d	CoA18138_2358	4.5	-1	1	22	438	459	UGAAGCUGCCAGCAUGAUCUGG	AAUGGUUGUGUUGUUAGCUUCG	Cleavage	0.365553024	0.333322961
ath-miR396a-5p	CoA18663_2328	4.5	-1	1	21	1761	1781	UUCCACAGCUUUCUUGAACUG	UAAUGUAAUAGAGCUGUGGAA	Cleavage	-0.620490827	0.074595809
ath-miR396a-5p	CoA8777_3260	4.5	-1	1	21	2433	2453	UUCCACAGCUUUCUUGAACUG	UGUUUUGAGAGAGUUUUGGAA	Cleavage	0.898880746	0.000979419
ath-miR396b-5p	CoA8777_3260	4.5	-1	1	21	2433	2453	UUCCACAGCUUUCUUGAACUU	UGUUUUGAGAGAGUUUUGGAA	Cleavage	0.907574092	0.000721324
ath-miR396b-5p	CoA18663_2328	4.5	-1	1	21	1761	1781	UUCCACAGCUUUCUUGAACUU	UAAUGUAAUAGAGCUGUGGAA	Cleavage	-0.671729766	0.047530321
bna-miR167d	CoA18138_2358	4.5	-1	1	20	440	459	UGAAGCUGCCAGCAUGAUCU	UGGUUGUGUUGUUAGCUUCG	Cleavage	0.365553024	0.333322961
bra-miR408-5p	CoA21021_2108	4.5	-1	1	21	1714	1734	GGGAGCCAGGGAAGAGGCAGU	UUUUUUUUUUCUUUGGUUCCU	Cleavage	-0.476096536	0.195136837
gma-miR396h	CoA18663_2328	4.5	-1	1	20	1761	1780	UCCACAGCUUUCUUGAACUG	UAAUGUAAUAGAGCUGUGGA	Cleavage	-0.620490827	0.074595809
gra-miR167c	CoA14161_2687	4.5	-1	1	22	1813	1834	UCAGAUGAAGCUGCCAGCAUGA	CUAUGCUGCUAGCUUAAUUUGU	Cleavage	-0.666740896	0.049832451
hbr-miR156	CoA5789_3732	4.5	-1	1	19	1140	1158	UUGACAGAAGAUAGAGAGC	AUUUUUUAUUUUUUGUUGA	Cleavage	-0.347482617	0.359527955
hbr-miR156	CoA11478_2967	4.5	-1	1	19	583	601	UUGACAGAAGAUAGAGAGC	AUUUUUUAUUUUUUGUUGA	Cleavage	-0.316752562	0.406262664
hbr-miR156	CoA11879_2942	4.5	-1	1	19	2749	2767	UUGACAGAAGAUAGAGAGC	GCUCUUUAAGUUCUGUUAG	Translation	-0.11082866	0.77651998
hbr-miR156	CoA3445_4292	4.5	-1	1	19	2628	2646	UUGACAGAAGAUAGAGAGC	GCCCUCUAUUUUCUGCUAU	Cleavage	-0.616908297	0.076780075
osa-miR167d-5p	CoA18138_2358	4.5	-1	1	21	439	459	UGAAGCUGCCAGCAUGAUCUG	AUGGUUGUGUUGUUAGCUUCG	Cleavage	0.365718524	0.33308745
osa-miR395b	CoA28144_1561	4.5	-1	1	21	466	486	GUGAAGUGUUUGGGGGAACUC	GUGUAUCGUCAAACAUUUUAU	Cleavage	-0.679838125	0.043936048
osa-miR398b	CoA18138_2358	4.5	-1	1	21	2118	2138	UGUGUUCUCAGGUCGCCCCUG	UUGGGGUUACUUGGGGACAAA	Cleavage	0.407080502	0.276855843
ptc-miR167f-5p	CoA18138_2358	4.5	-1	1	21	439	459	UGAAGCUGCCAGCAUGAUCUU	AUGGUUGUGUUGUUAGCUUCG	Cleavage	0.292999214	0.444176249
tae-miR1122c-3p	CoA15702_2564	4.5	-1	1	21	1631	1651	UCUAAUAUUAUGGGACGGAGG	GAUUUGUUCUGUAAUGUUUGA	Cleavage	-0.213883121	0.580551155
tae-miR1122c-3p	CoA10727_3016	4.5	-1	1	21	1350	1370	UCUAAUAUUAUGGGACGGAGG	AUUCGGGUCUAUAAUAUUUGA	Cleavage	0.880188397	0.001740122
vvi-miR167c	CoA18138_2358	4.5	-1	1	21	439	459	UGAAGCUGCCAGCAUGAUCUC	AUGGUUGUGUUGUUAGCUUCG	Cleavage	0.310217158	0.416542535
vvi-miR396a	CoA8777_3260	4.5	-1	1	21	2433	2453	UUCCACAGCUUUCUUGAACUA	UGUUUUGAGAGAGUUUUGGAA	Cleavage	0.903747356	0.000828179
vvi-miR396a	CoA18663_2328	4.5	-1	1	21	1761	1781	UUCCACAGCUUUCUUGAACUA	UAAUGUAAUAGAGCUGUGGAA	Cleavage	-0.666143384	0.050112852
vvi-miR396b	CoA8777_3260	4.5	-1	1	20	2434	2453	UUCCACAGCUUUCUUGAACU	GUUUUGAGAGAGUUUUGGAA	Cleavage	0.906488398	0.000750598
vvi-miR396b	CoA18663_2328	4.5	-1	1	20	1762	1781	UUCCACAGCUUUCUUGAACU	AAUGUAAUAGAGCUGUGGAA	Cleavage	-0.673642471	0.046666097
zma-miR398a-3p	CoA18138_2358	4.5	-1	1	21	2118	2138	UGUGUUCUCAGGUCGCCCCCG	UUGGGGUUACUUGGGGACAAA	Cleavage	0.418324796	0.262493378
ata-miR408-3p	CoA19729_2224	5	-1	1	20	644	663	UGCACUGCCUCUUCCCUGCC	UGCCGGGGGGGGGGGGUGUA	Cleavage	-0.627728687	0.070302043
ath-miR157a-5p	CoA14161_2687	5	-1	1	21	1464	1484	UUGACAGAAGAUAGAGAGCAC	GUGUUCUUUUUUUUCUUUUAA	Cleavage	-0.524358504	0.147278328
ath-miR157a-5p	CoA26032_1761	5	-1	1	21	450	470	UUGACAGAAGAUAGAGAGCAC	GUGCUUGCUAUUUUUUAUUAG	Cleavage	0.52895755	0.143119315
ath-miR157a-5p	CoA20069_2196	5	-1	1	21	1525	1545	UUGACAGAAGAUAGAGAGCAC	UUUCUUUCUUUCUUUUGUCGU	Cleavage	0.52535659	0.146369815
ath-miR157a-5p	CoA7918_3371	5	-1	1	21	85	105	UUGACAGAAGAUAGAGAGCAC	GAACUCUCUCUCUUCUCUCAC	Cleavage	-0.781275533	0.012912984
ath-miR157a-5p	CoA25733_1794	5	-1	1	21	1384	1404	UUGACAGAAGAUAGAGAGCAC	AGGUUCUGUAAUUUUUGUUGA	Translation	0.094102791	0.809704411
ath-miR395a	CoA28144_1561	5	-1	1	21	466	486	CUGAAGUGUUUGGGGGAACUC	GUGUAUCGUCAAACAUUUUAU	Cleavage	-0.444902281	0.230156864
bra-miR408-5p	CoA8910_3321	5	-1	1	21	776	795	GGGAGCCAGGGAAGAGGCAGU	UUUGCCUCUUCU-UGGUUCUU	Cleavage	-0.622565437	0.073348821
bra-miR408-5p	CoA8777_3260	5	-1	1	21	768	787	GGGAGCCAGGGAAGAGGCAGU	UUUGCCUCUUCU-UGGUUCUU	Cleavage	-0.523663302	0.14791308
bra-miR408-5p	CoA12817_2807	5	-1	1	21	653	673	GGGAGCCAGGGAAGAGGCAGU	CUUGCGUCUUCUGUGUCUCUC	Cleavage	0.946059651	0.000113825
bra-miR408-5p	CoA19759_2226	5	-1	1	21	1909	1929	GGGAGCCAGGGAAGAGGCAGU	UUUUUCCUUUUCUUGGCUUCU	Cleavage	-0.22100726	0.567693291
bra-miR408-5p	CoA4364_4045	5	-1	1	21	2847	2867	GGGAGCCAGGGAAGAGGCAGU	GAUGCGUCUUCCUUUGUUUUU	Cleavage	0.647784383	0.059224144
gra-miR167c	CoA18138_2358	5	-1	1	22	443	464	UCAGAUGAAGCUGCCAGCAUGA	UUGUGUUGUUAGCUUCGUCGGU	Cleavage	0.292999214	0.444176249
gra-miR167c	CoA4364_4045	5	-1	1	22	2456	2477	UCAGAUGAAGCUGCCAGCAUGA	GCAGGGUGGCAGUUAUGUCUGA	Cleavage	0.217901612	0.573285599
gra-miR167c	CoA4789_3953	5	-1	1	22	2946	2967	UCAGAUGAAGCUGCCAGCAUGA	UAAUGCUAGCAACUUCAUAUGC	Translation	0.33902317	0.372124677
gra-miR167c	CoA18954_2279	5	-1	1	22	2179	2200	UCAGAUGAAGCUGCCAGCAUGA	AGUUCCUGUUGGCUUUAUUUGU	Cleavage	-0.342907354	0.366315113
hbr-miR156	CoA7918_3371	5	-1	1	19	87	105	UUGACAGAAGAUAGAGAGC	ACUCUCUCUCUUCUCUCAC	Cleavage	-0.781275533	0.012912984

hbr-miR156	CoA20069_2196	5	-1	1	19	1527	1545	UUGACAGAAGAUAGAGAGC	UCUUUCUUUCUUUUGUCGU	Cleavage	0.52535659	0.146369815
hbr-miR156	CoA14161_2687	5	-1	1	19	1466	1484	UUGACAGAAGAUAGAGAGC	GUUCUUUUUUUUCUUUUAA	Cleavage	-0.524358504	0.147278328
hbr-miR156	CoA25733_1794	5	-1	1	19	1386	1404	UUGACAGAAGAUAGAGAGC	GUUCUGUAAUUUUUGUUGA	Translation	0.094102791	0.809704411
hbr-miR156	CoA26032_1761	5	-1	1	19	452	470	UUGACAGAAGAUAGAGAGC	GCUUGCUAUUUUUUAUUAG	Cleavage	0.52895755	0.143119315
osa-miR395b	CoA10727_3016	5	-1	1	21	2354	2374	GUGAAGUGUUUGGGGGAACUC	GGGUACCCCCGGGCAUGUUAC	Cleavage	-0.534648162	0.138069577
osa-miR395b	CoA11954_2888	5	-1	1	21	2188	2208	GUGAAGUGUUUGGGGGAACUC	GGGUACCCCCGGGCAUGUUAC	Cleavage	-0.463696488	0.2086753
osa-miR395b	CoA4801_3956	5	-1	1	21	791	811	GUGAAGUGUUUGGGGGAACUC	UGGUUUCCCCCAAGAUUUUGC	Translation	-0.458648104	0.214331962
osa-miR395b	CoA9012_3232	5	-1	1	21	554	574	GUGAAGUGUUUGGGGGAACUC	CCUCUCUCUCUAACAUUUCAC	Translation	-0.591480296	0.093424034
osa-miR395b	CoA14354_2670	5	-1	1	21	554	574	GUGAAGUGUUUGGGGGAACUC	CCUCUCUCUCUAACAUUUCAC	Translation	-0.461322474	0.21132494
osa-miR395b	CoA23805_1929	5	-1	1	21	765	785	GUGAAGUGUUUGGGGGAACUC	CCUCUCUCUCUAACAUUUCAC	Translation	-0.436512077	0.240118665
ppt-miR319a	CoA21021_2108	5	-1	1	20	414	433	CUUGGACUGAAGGGAGCUCC	UGCGAUCGCUUCAGUCAAAG	Cleavage	0.341800627	0.367966005
ppt-miR319a	CoA29174_1417	5	-1	1	20	437	456	CUUGGACUGAAGGGAGCUCC	UGCGAUCGCUUCAGUCAAAG	Cleavage	0.570793873	0.108467574
stu-miR408b-5p	CoA26792_1692	5	-1	1	21	76	96	ACGGGGACGAGACAGAGCAUG	AGUUCUCUGUCUCGAUUUCGA	Cleavage	0.673216175	0.046857833
tae-miR1122c-3p	CoA27612_1606	5	-1	1	21	1113	1133	UCUAAUAUUAUGGGACGGAGG	UUUUUUUUUAAUAAUGUUAGA	Cleavage	-0.472672748	0.198824352
tae-miR1122c-3p	CoA11969_2826	5	-1	1	21	1752	1772	UCUAAUAUUAUGGGACGGAGG	GGUUGGGCCUAUGAAAUUAGA	Cleavage	0.824939705	0.00619966
tae-miR395b	CoA3445_4292	5	-1	1	20	2808	2827	UGAAGUGUUUGGGGGAACUC	CGGUUCCUUCAAGGGCUUCU	Cleavage	-0.450460719	0.223683373
tae-miR395b	CoA17976_2312	5	-1	1	20	1314	1333	UGAAGUGUUUGGGGGAACUC	AAUUUCCCCCGAAGAAUUUA	Cleavage	0.632541672	0.067534371
zma-miR396g-3p	CoA27626_1626	5	-1	1	21	937	957	GUUCAAGAAAGCUGUGGAAGA	UGUUUCAUAGUCUUUUUGGAA	Translation	-0.821717398	0.006586286
zma-miR396g-3p	CoA25733_1794	5	-1	1	21	1591	1611	GUUCAAGAAAGCUGUGGAAGA	AUUUAUUUGGUUUUCUUGAAG	Cleavage	0.088393309	0.821094996
zma-miR396g-3p	CoA5701_3740	5	-1	1	21	2792	2812	GUUCAAGAAAGCUGUGGAAGA	UGUAUUAUUGCUUUUUUGAAU	Cleavage	-0.583629207	0.098972877
zma-miR396g-3p	CoA3445_4292	5	-1	1	21	1467	1487	GUUCAAGAAAGCUGUGGAAGA	GGUUAGGCAGCUUCUUUGAAU	Cleavage	-0.617410018	0.076471813

Supplementary Table S12. Differentially expressed genes and lncRNAs in miRNA–lncRNA–mRNA network

Transcript ID	Protein ID	CoA1_FPKM	CoA2_FPKM	CoA3_FPKM	Fold_change-CoA2/CoA1	q-value	UP/DOWN regulate	Fold_change-CoA3/CoA2	q-value	UP/DOWN regulate	Fold_change-CoA3/CoA1	q-value	UP/DOWN regulate	Functional annotation of target gene	References
transcript_HQ_CoA_transcript27685/f2p0/1608	*GRF5*	12.28	2.586666667	8.59	-2.0821	9.4805E-08	DOWN	1.5655	0.0065679	UP	-0.52898	0.39216	\		
transcript_HQ_CoA_transcript27832/f2p0/1591	*GRF1*	19.34666667	1.996666667	9.976666667	-3.1313	6.0774E-20	DOWN	2.1753	0.000028887	UP	-0.96349	0.03185	\	Involved in development of the pistil and determination of floral organ specification	[20-22]
transcript_HQ_CoA_transcript29242/f2p0/1411	*GRF4*	40.64	7.043333333	31.39333333	-2.3777	1.3645E-25	DOWN	2.0011	3.8408E-07	UP	-0.3878	0.43644	\		
transcript_HQ_CoA_transcript21959/f2p0/2080	*SPL12*	64.81666667	22.45	58.47333333	-1.3813	6.9403E-12	DOWN	1.225	0.0035907	UP	-0.16816	0.7758	\		
transcript_HQ_CoA_transcript14160/f2p0/2685	*SPL6*	19.25666667	6.463333333	16.81666667	-1.4053	0.000019128	DOWN	1.217	0.002064	UP	-0.1997	0.63438	\	Involved in early anther development and regulating the flowering time	[17-19]
transcript_HQ_CoA_transcript27850/f2p0/1604	*SPL16*	51.68666667	12.71	28.65666667	-1.8679	1.2105E-09	DOWN	1.0145	0.000057441	UP	-0.86412	0.0036846	\		
transcript_HQ_CoA_transcript23484/f3p0/1962	*AXR1*	16.07666667	2.18	4.48	-2.6719	0.0003814	DOWN	0.8462	0.35287	\	-1.8392	0.0016414	DOWN	DNA repair and meiosis	[74]
transcript_HQ_CoA_transcript3229/f6p0/4345	*DCL3B*	17.46333333	3.76	3.503333333	-2.0092	0.011983	DOWN	-0.27478	0.84322	\	-2.3015	1.062E-05	DOWN	small RNA biogenesis	[75]
transcript_HQ_CoA_transcript1802/f2p0/5043	*WAK2*	5.62	1.97	0.866666667	-1.2994	0.24711	\	-1.3604	0.18024	\	-2.6734	0.0021278	DOWN	cell wall biogenesis	[58]
transcript_HQ_CoA_transcript22060/f4p0/2012	*PMI1*	117.72	46.63333333	56.44	-1.1809	1.4233E-07	DOWN	0.12133	0.67373	\	-1.0709	8.71E-07	DOWN	cell wall biogenesis	[57]

Supplementary Table S13. Oligonucleotide primers used in qRT-PCR assays in this study

Gene	Primer pairs
GRF1	F:CTCTTATTTCTCCCCGTTTCTTTC<br>R:GGACACTTGGTTCTTGCACTCA
GRF5	F:CCAAATCCACCTTCCCTACTG<br>R:AACATGCCTCCGAGCCAATA
SPL6	F:GATGGCACGGACTGTCTTTC<br>R:GCGACTCACCCTGATTCCTC
SPL16	F:TATTTCAAGTGGCACCTTACGA<br>R:TCTAAGAACCAGTGGAAGAACATC
WAK2	F:ATAGTCTGGCCCAATCCAACTC<br>R:GCAATATCACCATCCCTTACCC
DCL3b	F:CCTTCAACCACCTGTAACCACT<br>R:CAAAGATGCGTCAGCCAAAT
AXR1	F:TTCGCACATTTAGTCCCACA<br>R:TGACACCTCAACCGCTTCTT
CoA18663_2328	F:GGGGCTTGCTTTCTTTATTGT<br>R:GAATGCCTACTGTGATACCCAAA
CoA5789_3732	F:AAGTGTAATTGTTGCGTTTGGG<br>R:ATGCCGATTCATCCTCATCC
CoA30190_880	F:TTTCTTCCCACAGCCAACC<br>R:CGCAAAACAGACCCACAACT
CoA30219_795	F:CTTCACGGATCTGCCATCAC<br>R:CAGAGGGTTTCAAACCAGACAA

miR396b-5p	F:CGTTCCACAGCTTTCTTGAACTT
miR159a	F:CGTTTGGATTGAAGGGAGCTCTA
miR167d	F:GTGAAGCTGCCAGCATGATCT
miR396a	F:GTTCCACAGCTTTCTTGAACTAA
miR408d	F:TGCACTGCCTCTTCCCTGGC
miR171a-3p	F:TGATTGAGCCGCGCCAATAT
miR396b	F:CGTTCCACAGCTTTCTTGAACT
miR156	F:CGTTGACAGAAGATAGAGAGCA
TubA	F:TATCAACTACCAGCCACCCACT<br>R:TGCACAAAAGCCCTCTTCG
5S	F:TCAGGCACTCGCCATCTATT<br>R:GACCTCCCCTACAGTATCGTCA
